# Optimal Multi-Stage Arrhythmia Classification Approach

**DOI:** 10.1038/s41598-020-59821-7

**Published:** 2020-02-19

**Authors:** Jianwei Zheng, Huimin Chu, Daniele Struppa, Jianming Zhang, Sir Magdi Yacoub, Hesham El-Askary, Anthony Chang, Louis Ehwerhemuepha, Islam Abudayyeh, Alexander Barrett, Guohua Fu, Hai Yao, Dongbo Li, Hangyuan Guo, Cyril Rakovski

**Affiliations:** 1 Chapman University, Orange, USA; 2 Ningbo First Hospital of Zhejiang University, Hangzhou, China; 3 Shaoxing People’s Hospital (Shaoxing Hospital Zhejiang University School of Medicine), Shaoxing, China; 4 Imperial College London, London, USA; 5 CHOC Children’s Hospital, Orange, USA; 6 Loma Linda University Health, Loma Linda, USA; 7 Zhejiang Cachet Jetboom Medical Devices CO.LTD, Hangzhou, China

**Keywords:** Atrial fibrillation, Electrodiagnosis, Computational science, Software, Statistics

## Abstract

Arrhythmia constitutes a problem with the rate or rhythm of the heartbeat, and an early diagnosis is essential for the timely inception of successful treatment. We have jointly optimized the entire multi-stage arrhythmia classification scheme based on 12-lead surface ECGs that attains the accuracy performance level of professional cardiologists. The new approach is comprised of a three-step noise reduction stage, a novel feature extraction method and an optimal classification model with finely tuned hyperparameters. We carried out an exhaustive study comparing thousands of competing classification algorithms that were trained on our proprietary, large and expertly labeled dataset consisting of 12-lead ECGs from 40,258 patients with four arrhythmia classes: atrial fibrillation, general supraventricular tachycardia, sinus bradycardia and sinus rhythm including sinus irregularity rhythm. Our results show that the optimal approach consisted of Low Band Pass filter, Robust LOESS, Non Local Means smoothing, a proprietary feature extraction method based on percentiles of the empirical distribution of ratios of interval lengths and magnitudes of peaks and valleys, and Extreme Gradient Boosting Tree classifier, achieved an F_1_-Score of 0.988 on patients without additional cardiac conditions. The same noise reduction and feature extraction methods combined with Gradient Boosting Tree classifier achieved an F_1_-Score of 0.97 on patients with additional cardiac conditions. Our method achieved the highest classification accuracy (average 10-fold cross-validation F_1_-Score of 0.992) using an external validation data, MIT-BIH arrhythmia database. The proposed optimal multi-stage arrhythmia classification approach can dramatically benefit automatic ECG data analysis by providing cardiologist level accuracy and robust compatibility with various ECG data sources.

## Introduction

ECGs represent the filtered electrical activity generated by the heart. An ECG from lead II presents a normal heartbeat under sinus rhythm that has a characteristic shape with three features, a P-wave presenting the atrial depolarization process, a QRS complex denoting the ventricular depolarization process, and a T-wave representing the ventricular repolarization. The normal feature sequence of the cardiac cycle is P-wave, QRS complex, and T-wave with sections between them called segments. Three such major segments are the PR, ST, and TP segments. Important periods within and between ECG waves are the PR, QT, and RR intervals.

Damage to the heart muscle or nerves can change the electrical activity of the heart and induce a corresponding change in the shape of the ECGs. Thus, ECG is a major clinical diagnostic tool for various heart abnormalities. Arrhythmias are a family of conditions characterized by aberrations from the normal rate or rhythm of the heartbeats. There are several dozen classes of arrhythmia with various distinct manifestations, excessively slow or fast heartbeats such as sinus bradycardia and atrial tachycardia, irregular rhythm with missing or distorted wave segments and intervals, or both. Arrhythmias have a wide and significant impact on public health, quality of life, and medical expenditures. For example, the common type of arrhythmia, atrial fibrillation (AFIB), is associated with a significant increase in the risk of cardiac dysfunction and stroke. According to the American Heart Association^1^, in 2015 AFIB was the underlying cause of death in 23,862 people and was listed on 148,672 US death certificates. The estimates of the prevalence of AFIB in the United States ranged from 2.7 million to 6.1 million in 2010. Further, the AFIB prevalence is expected to rise to 12.1 million in 2030 as the average population age increases. In the European Union, the prevalence of AFIB in adults older than 55 years was estimated to be 8.8 million (95% CI, 6.5 –12.3 million) in 2010 and was projected to rise to 17.9 million in 2060 (95% CI, 13.6 –23.7 million). The weighted prevalence of AFIB in the Chinese population aged 35 years or older was 0.71%^2^.

According to the existing screening and diagnostic practice, cardiologists review ECG data, establish the diagnosis, and begin implementing subsequent treatment plans such as anticoagulation or radiofrequency catheter ablation. However, the demand for high-accuracy automatic heart condition diagnoses has recently increased sharply in parallel with the public health policy of implementing wider screening procedures, and the adoption of ECG enabled wearable devices. Such classification methods have to properly account for the inter-person and intra-personal variability of ECG signals, distortion from noise, missing feature waves and intervals in many arrhythmia cases. A variety of algorithms have been proposed for removing noise from raw ECG data, extracting salient features from the smoothed ECG signals, and feeding them into an optimal classification method.

Some previous studies^3–5^ have focused on the separation between AFIB and sinus rhythm (SR). These studies achieved a high accuracy of classification rate. Kennedy *et al*.^3^ proposed Random Forest (RF) and K Nearest Neighbors (KNN) to classify AFIB and SR by the coefficient of sample entropy (CoSEn), the coefficient of variance (CV), root mean square of the successive differences (RMSSD), and median absolute deviation (MAD). Zhu *et al*.^4^ suggested using maximum margin clustering with an immune evolutionary algorithm and features of wave and segment measurements for classifying ectopic heartbeats by the database from MIT laboratories at Boston’s Beth Israel Hospital (MIT-BIH). Asgari *et al*.^5^ proposed to use features of peak-to-average power ratio and log-energy entropy to detect AFIB by support vector machine (SVM) model. A high precision classification of a more extensive set of arrhythmia classes has been achieved with extensive neural network classification^6^. However, a complete comparison of the classification accuracy of multiple analytical algorithms and accompanying noise reduction and feature selection techniques for a large number of arrhythmia classes has not been performed yet.

**Figure 1:**
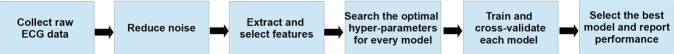
The pipeline of scheme.

In this work, we employed several signal noise reduction techniques, proposed a novel ECG feature extraction method, designed and implemented and a large computational comparison study across thousand of competing classification schemes based on new, proprietary, expertly labeled data. According to clinical relevance, 11 rhythms labeled by certified physicians were merged into 4 groups (SB, AFIB, GSVT, SR), SB only included sinus bradycardia, AFIB consisted of atrial fibrillation and atrial flutter (AFL), GSVT contained supraventricular tachycardia, atrial tachycardia, atrioventricular node reentrant tachycardia, atrioventricular reentrant tachycardia and wandering atrial pacemaker, and SR included sinus rhythm and sinus irregularity. These 4 group labels were used for training and testing of our models. The pipeline of the proposed multi-stage scheme is presented in Fig. 1. We utilized the Butterworth Low-pass filter to remove high-frequency noise, the Robust LOESS to eliminate baseline wandering and Non Local Means (NLM) to remove the remaining noise. The features extracted from ECGs included measurements of wave and segments provided by ECG machine and relation measurements among peaks and valleys, producing up to 39,830 features. In order to study the classification reliability of features, we defined 11 distinct feature combinations with respect to the type of features and the lead of the ECGs. This feature combination setting aimed to compare the performance of classification schemes using 12-lead and single-lead ECG data, and to evaluate the classification capacity of different feature combinations. Moreover, aiming to evaluate the additional cardiac conditions impact for the rhythm classification, we separated a small subset without such conditions from the entire dataset. Sequentially, these two datasets, with and without additional cardiac conditions, generated 22 datasets by 11 distinct feature combinations as mentioned above. As a common practice in machine learning, we rescaled the subject’s raw ECG signals to have maximum peak values of 1. Thus, we generated the new 22 datasets by rescaling the original 22 datasets. That allowed us to assess the effect of rescaling on classification accuracy as well. Using these 44 datasets, we carried out an exhaustive grid search spanning the ranges of all tuning hyperparameters for nineteen base classification algorithms and they combined with five optimal strategies such as bagging average, Adaboost, OneVsRest, OneVsOne, and Error-Correcting Output-Codes. The hyperparameters tuning, model fitting and optimal strategy evaluating were deployed on each dataset respectively. Thus, we compared thousands of competing strategies to discover the optimal multi-stage arrhythmia classification routine. The base classification algorithms that we studied were Decision Tree (DT), K Nearest Neighbors (KNN), Nearest Centroid (NC), Gaussian Naive Bayesian (GNB), Multinomial Naive Bayesian (MNB), Complement Naive Bayesian (CNB), Bernoulli Naive Bayesian (BNB), Linear Classifier (LC), Quadratic Discriminant Analysis (QDA), Multinomial Logistic Regression (MLR), Multi-layer Perceptron Neural Net (MPN), Ridge Regression Classifier (RRC), Linear Classifiers with Stochastic Gradient Descent (LCSGD), Passive Aggressive Classifier (PAC), Linear SVC (SVC), Random Forest (RF), Extremely Randomized Trees (ERT), Gradient Boosting Tree (GBT) and Extreme Gradient Boosting Tree (EGBT). Finally, EGBT and GBT models achieved the best classification performance and with details presented in the Results section. A presentation of complete results that include all competing schemes comparisons is shown in Supplementary sections C and D.

## Results

We used confusion matrices and normalized confusion matrices to evaluate the performance of classification models and weighted average F_1_-Score defined in 1 as criteria for selection of the best hyperparameters and models.

1 $$Weighted\ average\ {F}_{1}=\frac{\mathop{\sum }\limits_{j=1}^{n}{F}_{1j}\ast {N}_{j}}{\mathop{\sum }\limits_{j=1}^{n}{N}_{j}}$$

where *n* presents the number of different labels that will be classified, *N*_*j*_ is the total number of observations with label *j* and *F*_1*j*_ presents the F_1_-Score associated with label *j*.

2 $${F}_{1}-Score=\frac{2\ast (precision\ast recall)}{precision+recall}$$

The F_1_-Score, confusion matrix, and normalized confusion matrix presented below are the average results from 10-fold cross-validation with 20% testing data and 80% training data.

**Figure 2:**
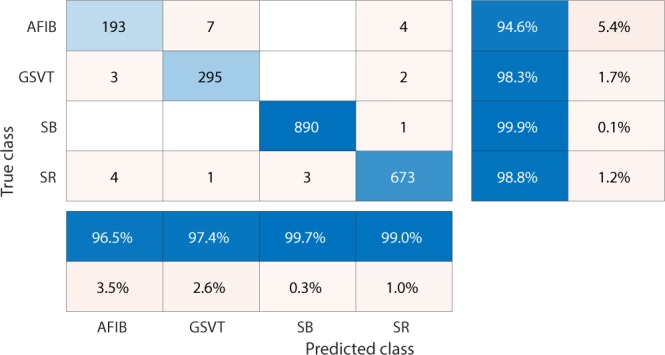
Confusion matrix of EGBT model fed by rescaled Feature Group 8 dataset of patients without additional cardiac conditions. The true class labels of AFIB, GSVT, SB and SR are provided by cardiologists who read the ECGs. The predicted class labels present the outcomes generated by classification model. Numbers in the diagonal line with blue color present the correct prediction. Percentage numbers with blue color present the accuracy of associated category.

**Figure 3:**
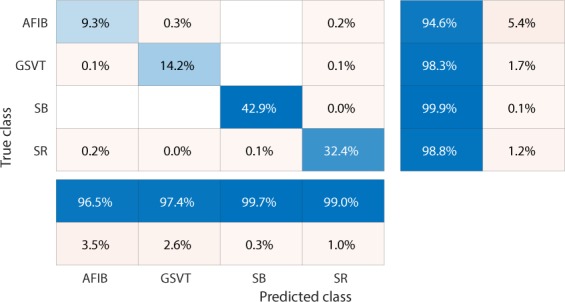
Normalized confusion matrix of EGBT model fed by rescaled Feature Group 8 dataset of patients without additional cardiac conditions. The true class labels of AFIB, GSVT, SB and SR are provided by cardiologists who read the ECGs. The predicted class labels present the outcomes generated by classification model. Numbers in the diagonal line with blue color present the normalized ratio of correct prediction, which is equal to the numbers in the diagonal line of Fig. 2 divided the total number of cases in validation cohort.

**Figure 4:**
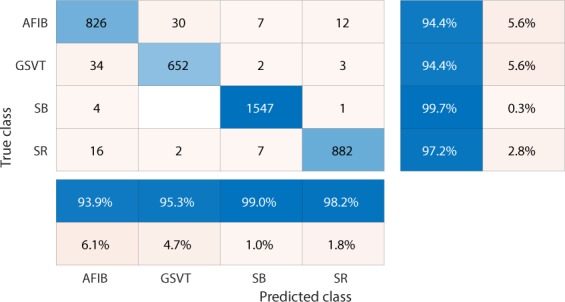
Confusion matrix of GBT model fed by rescaled Feature Group 5 dataset of patients with additional cardiac conditions.The true class labels of AFIB, GSVT, SB and SR are provided by cardiologists who read the ECGs. The predicted class labels present the outcomes generated by classification model. Numbers in the diagonal line with blue color present the correct prediction. Percentage numbers with blue color present the accuracy of associated category.

**Figure 5:**
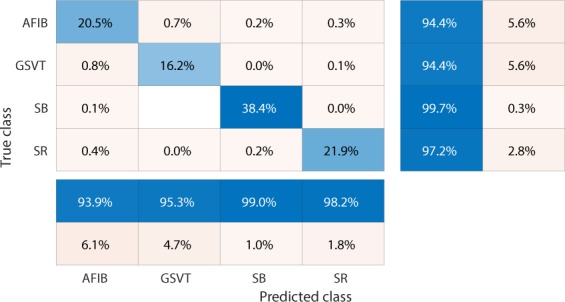
Normalized confusion matrix of GBT model fed by rescaled Feature Group 5 dataset of patients with additional cardiac conditions.The true class labels of AFIB, GSVT, SB and SR are provided by cardiologists who read the ECGs. The predicted class labels present the outcomes generated by classification model. Numbers in the diagonal line with blue color present the normalized ratio of correct prediction, which is equal to the numbers in the diagonal line of Fig. 4 divided the total number of cases in validation cohort.

Firstly, EGBT model using Feature Group 5 dataset of patients without additional cardiac conditions attained the highest weighted average F_1_-Score of 0.988 (shown in Table 1). GBT model using Feature Group 8 dataset of patients with additional cardiac conditions attained the highest weighted average F_1_-Score of 0.97 (shown in Table 2). The confusion matrix and normalized confusion matrix for each model were presented in Figs. 2, 3, 4, and 5 respectively. For the dataset of patients without additional cardiac conditions, the average F_1_-Score shown in Table 3 of the models using features in group 1 that were provided by the ECG machine is 0.021 lower than that of models using features in group 2 that includes engineered features on lead II. That is, the engineered features proposed by this work had higher classification capacity than the features measured by ECG machine. The full comparison results as mentioned in the introduction section is presented in Supplementary sections D.

**Table 1:** Report of EGBT with Feature Group 8 dataset of patients without additional cardiac conditions.

	F_1_-Score	Precision	Recall
AFIB	0.964	0.974	0.954
GSVT	0.979	0.977	0.980
SB	0.996	0.994	0.999
SR	0.989	0.990	0.989
macro avg	0.982	0.984	0.980
micro avg	0.988	0.988	0.988
weighted avg	0.988	0.988	0.988

**Table 2:** Report of GBT with Feature Group 5 dataset of patients with additional cardiac conditions.

	F_1_-Score	Precision	Recall
AFIB	0.941	0.938	0.944
GSVT	0.949	0.953	0.944
SB	0.993	0.990	0.996
SR	0.977	0.982	0.972
macro avg	0.965	0.966	0.964
micro avg	0.970	0.970	0.970
weighted avg	0.970	0.971	0.970

**Table 3:** F_1_-Score comparison for different feature groups.

F_1_-Score	Dataset of patients without additional cardiac conditions	Dataset of patients with additional cardiac conditions	Difference
Feature Group 1	0.962	0.937	0.025
Feature Group 2	0.983	0.949	0.034
Feature Group 3	0.987	0.961	0.026
Feature Group 4	0.986	0.963	0.023
Feature Group 5	0.987	0.970	0.017
Feature Group 6	0.887	0.868	0.019
Feature Group 7	0.984	0.956	0.028
Feature Group 8	0.988	0.965	0.023
Feature Group 9	0.972	0.954	0.018
Feature Group 10	0.983	0.965	0.018
Feature Group 11	0.987	0.968	0.019

Secondly, our results show that the presence of conduction findings such as premature ventricular contraction (PVC), right bundle branch block (RBBB), left bundle branch block (LBBB) and atrial premature contraction (APC) negatively impacted the accuracy of the arrhythmia classification algorithms. In particular, based on the same feature group, the average F_1_-Score of the ECG dataset with these conditions was lower than that of datasets without them by 0.017 to 0.034 respectively (shown in Table 3). Furthermore, the multi-classification strategy interacted with the feature groups to provide scenario specific optimal approaches. The best models associated with each feature group are presented in Table 4. Table 4 shows that EGBT and GBT models dominate the highest classifiers for most scenarios.

**Table 4:** The best classification model list for each feature group.

	Dataset of patients with additional cardiac conditions	Dataset of patients without additional cardiac conditions
Feature Group 1	ERT	ERT
Feature Group 2	OneVSOne ERT	GBT
Feature Group 3	OneVSRest ERT	EGBT
Feature Group 4	GBT	GBT
Feature Group 5	ERT	GBT
Feature Group 6	OneVSOne GBT	GBT
Feature Group 7	ERT	EGBT
Feature Group 8	EGBT	EGBT
Feature Group 9	GBT	GBT
Feature Group 10	EGBT	GBT
Feature Group 11	EGBT	EGBT

**Figure 6:**
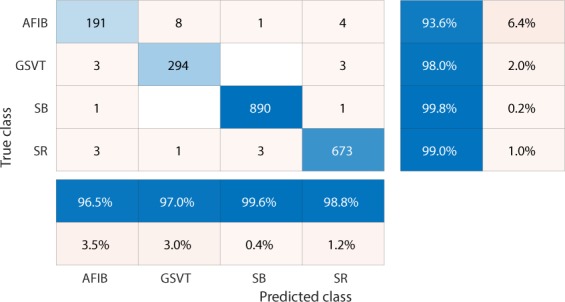
Confusion matrix of EGBT model fed by non-rescaled Feature Group 8 dataset of patients without additional cardiac conditions. The true class labels of AFIB, GSVT, SB and SR are provided by cardiologists who read the ECGs. The predicted class labels present the outcomes generated by classification model. Numbers in the diagonal line with blue color present the correct prediction. The blue color percentage show the general accuracy of associated category. Compared with confusion matrix shown in Fig. 2, the effect of rescaling the subject’s raw ECG signals to have maximum peak values of 1 has a very small positive effect on the classification accuracy of arrhythmia types.

**Figure 7:**
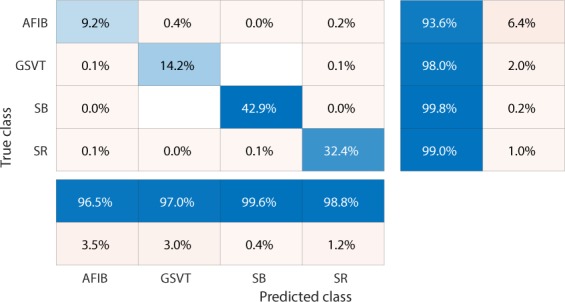
Normalized confusion matrix of EGBT model fed by non-rescaled Feature Group 8 dataset of patients without additional cardiac conditions.The true class labels of AFIB, GSVT, SB and SR are provided by cardiologists who read the ECGs. The predicted class labels present the outcomes generated by classification model. Numbers in the diagonal line with blue color present the normalized ratio of correct prediction, which is equal to the numbers in the diagonal line of Fig. 6 divided the total number of cases in validation cohort.

**Figure 8:**
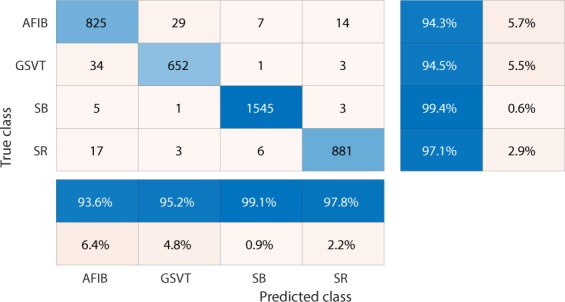
Confusion matrix of GBT model fed by non-rescaled Feature Group 5 dataset of patients with additional conditions. The true class labels of AFIB, GSVT, SB and SR are provided by cardiologists who read the ECGs. The predicted class labels present the outcomes generated by classification model. Numbers in the diagonal line with blue color present the correct prediction. The blue color percentage show the general accuracy of associated category. Compared with confusion matrix shown in Fig. 4, the effect of rescaling the subject’s raw ECG signals to have maximum peak values of 1 has a very small positive effect on the classification accuracy of arrhythmia types.

**Figure 9:**
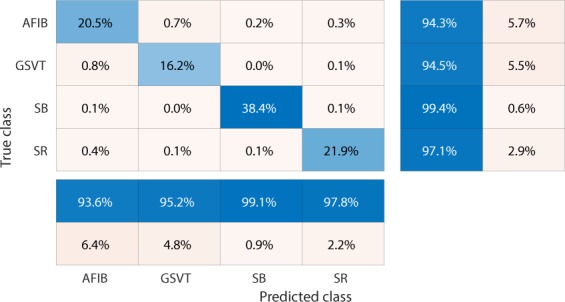
Normalized confusion matrix of GBT model fed by non-rescaled Feature Group 5 dataset of patients with additional cardiac conditions.The true class labels of AFIB, GSVT, SB and SR are provided by cardiologists who read the ECGs. The predicted class labels present the outcomes generated by classification model. Numbers in the diagonal line with blue color present the normalized ratio of correct prediction, which is equal to the numbers in the diagonal line of Fig. 8 divided the total number of cases in validation cohort.

Thirdly, we tested rescaling effects by the best performance classification models and feature groups reported in Tables 1 and 2. The results show that for the dataset of patients with additional cardiac conditions, weighted average F_1_-Score of the non-rescaling method is 0.001 lower than that of the rescaling method, while for the dataset of patients with additional cardiac conditions F_1_-Score of the non-rescaling method is 0.0016 lower than that of the rescaling method. For each model mentioned in Tables 1 and 2, the confusion matrix and normalized confusion matrix associated with the non-rescaling method are shown in Figs. 6, 7, 8, and 9 respectively. The effect of rescaling the subject’s raw ECG signals to have maximum peak values of 1 has a very small positive effect on the classification accuracy of arrhythmia types. The idea of this rescaling approach is similar to the inclusion of random effects in linear models. Morever, rescaling is a generally recommended preprocessing procedure in nonparametric classification methods such as neural networks and boosting trees.

Lastly, we ascertained the performance advantage of our method consisting of noise reduction methods, feature extraction scheme and Extreme Gradient Boosting Tree classification model to classify normal heart beat and four conduction conditions (shown in Table 5) in the MIT-BIH database^7^. Two RR intervals close to each heartbeat were used to extract features. The approach we proposed attains an F_1_-Score of 0.992 that is the weighted average score of 10-fold cross-validation with 10% testing data and 90% training data. Compared with available studies^8–17^, the approach we proposed achieved the highest accuracy score by using all the data files in MIT-BIH database.

**Table 5:** Report for the classification of normal heart beat and four conduction conditions. /: Paced beat; L: Left bundle branch block beat. N: Normal beat; R: Right bundle branch block beat. V: Premature ventricular contraction.

	F_1_-Score	Precision	Recall
/	0.996	0.998	0.995
L	0.994	0.998	0.992
N	0.991	0.991	0.992
R	0.997	0.998	0.997
V	0.986	0.980	0.991
macro avg	0.993	0.993	0.993
micro avg	0.992	0.992	0.992
weighted avg	0.992	0.992	0.992

## Discussion

We designed and implemented a large scale study aimed at finding the best multi-stage arrhythmia classification scheme. We carried out an extensive accuracy comparison among a range of 98 competing methods that are manifested in Supplementary section C. These multi-stage schemes consisted of a sequential application of denoising techniques, feature extraction methods and classification algorithms. We have provided methodological advancements to each of these steps. We propose a novel, three stage denoising method that includes Butterworth Low-pass filter to remove high-frequency noise (above 50 Hz), the Robust LOESS to eliminate baseline wandering and Non Local Means (NLM) to remove residual noise. We designed a novel, robust and optimal feature extraction strategy based on the magnitudes and lengths of peaks and valleys and distributional characteristics of their transformations. In particular, for each pair of peaks or valleys, we assessed the empirical frequency distribution of the ratio between the differences of heights and distances of the time, the ratio between the differences of widths and the distances of the times, as well as the ratio between the differences of the prominences and the distance of the time. The newly obtained features reveal the relationship between attributes of wave and time duration, which is a central key for recognizing possible rhythms. Thus, the feature extraction strategy in this project is more transparent and interpretable than the one that has been obtained via the use of deep neural networks^6^ and other automatic feature extraction methods^5^ where features are uninterpretable. We performed an extensive grid search of the classification method’s hyperparameters. We have shown that the optimal multi-stage classification approach as described above consisted of a three-stage noise reduction process, the new empirical frequency distribution feature extraction strategy, and extreme gradient boosting tree classification model combined with hyperparameters tuned via an exhaustive grid search attains arrhythmia classification accuracy that exceeds the level of professional cardiologists.

Our computational study compared 98 approaches that were trained on a new, expertly labeled high-quality data on 40,258 patients from the Shaoxing People’s Hospital (Shaoxing Hospital Zhejiang University School of Medicine) and Ningbo First Hospital of Zhejiang University. In total, 22 cardiologist and physician experts labeled and reviewed the rhythms and additional cardiac findings. This is a new, large size database of 12-lead ECGs and comprehensive rhythms and conditions labels. Previous related studies^3–6^ were limited in the degree of novelty of the methodological approaches, the size of the samples and the diversity of cardiac conditions considered. We have made our database accessible to the scientific community for further scientific endeavors.

We assessed, for the first time, the additional classification accuracy attributable to analyzing 12-lead ECGs vs single lead ECGs. We have found that the accuracy based on 12-lead data increases the F_1_-Score by 1.4%. We have also compared the algorithm’s ECG classification accuracy for patients with and without additional heart conditions. The accuracy decreases by 2% on average for patients with additional cardiac conditions such as PVC, APC, RBBB, and LBBB. The detrimental effect of these conditions on arrhythmia classification precision has not been previously studied^3–6^. For patients without additional cardiac condition, EGBT model that fed by rescaled features extracted from lead II ECGs produced the highest accuracy rate. Given these two results, the approach can have important arrhythmia classification applications to data collected from wearable devices such as Apple watch.

Lastly, we used our method to achieve the highest classification accuracy (average 10-fold cross-validation F_1_-Score of 0.992) using an external validation data, MIT-BIH. The proposed optimal multi-stage arrhythmia classification approach can dramatically benefit automatic ECG data analysis by providing cardiologist level accuracy and robust compatibility with various ECG data sources.

## Methods

### Study design and patients selection

Our novel data consisted of 40,258 12-lead ECGs, including 22,599 males and 17,659 females. The study participants were randomly chosen from over 120,000 subjects who visited the Shaoxing People’s Hospital (Shaoxing Hospital Zhejiang University School of Medicine) and the Ningbo First Hospital of Zhejiang University between 2013 and 2018. The institutional review board of Shaoxing People’s Hospital (Shaoxing Hospital Zhejiang University School of Medicine) and Ningbo First Hospital of Zhejiang University approved this study and granted the waiver of the requirement to obtain informed consent. The data contain 20% normal SR and 80% abnormal readings. The age groups with the highest prevalence were 51–60, 61–70, and 71–80 years representing 19.8%, 24%, and 17.3% respectively.

**Figure 10:**
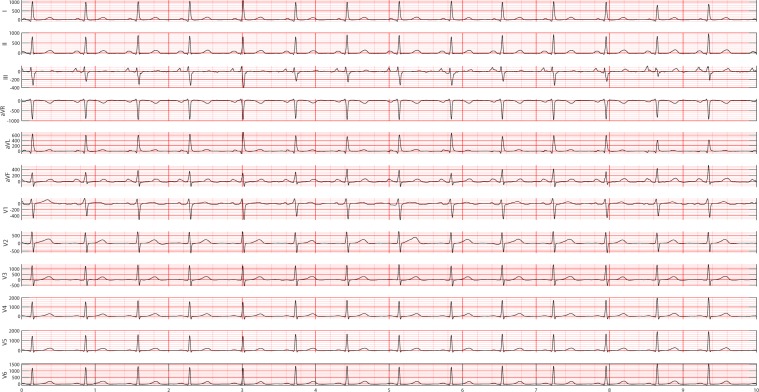
A 12-lead ECG presenting sinus normal rhythm. Normal sinus rhythm usually accompanies a heart rate of 60 to 100 beats per minute, with less than 0.16 second variation in the shortest and longest durations between successive P waves, and normal PR interval, QRS complex and QT interval.

**Figure 11:**
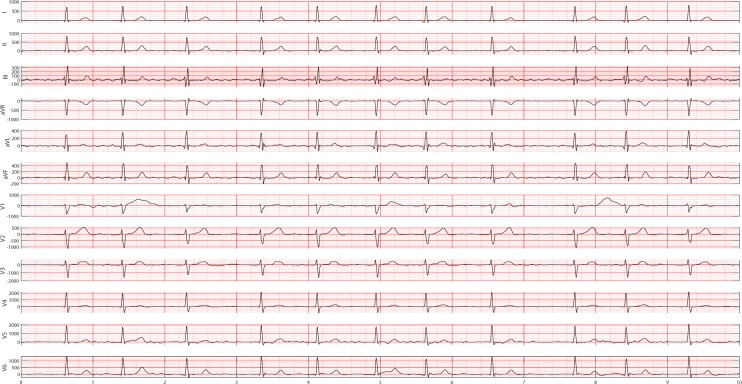
A 12-lead ECG showing atrial fibrillation rhythm that has no visible P waves that are replaced by coarse fibrillatory waves and an irregularly irregular QRS complex.

**Figure 12:**
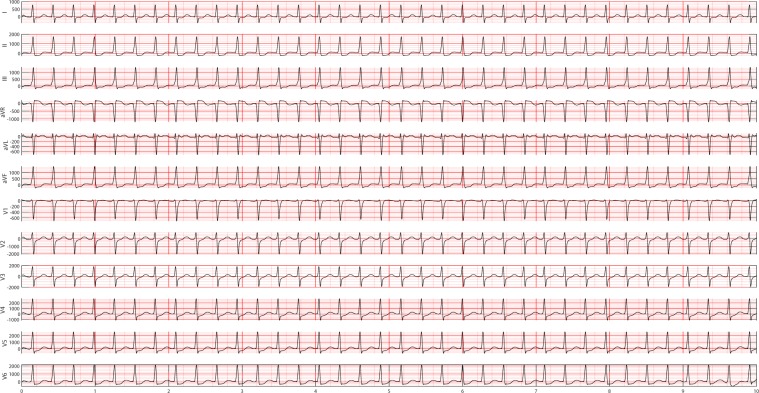
A 12-lead ECG example in GSVT group. In this study, GSVT refers to a group of rhythm that contained supraventricular tachycardia, atrial tachycardia, atrioventricular node reentrant tachycardia, atrioventricular reentrant tachycardia and wandering atrial pacemaker. The detailed definition of rhythm groups for classification and the definition of rhythms labeled by certified physicians are presented in Supplementary section A.

**Figure 13:**
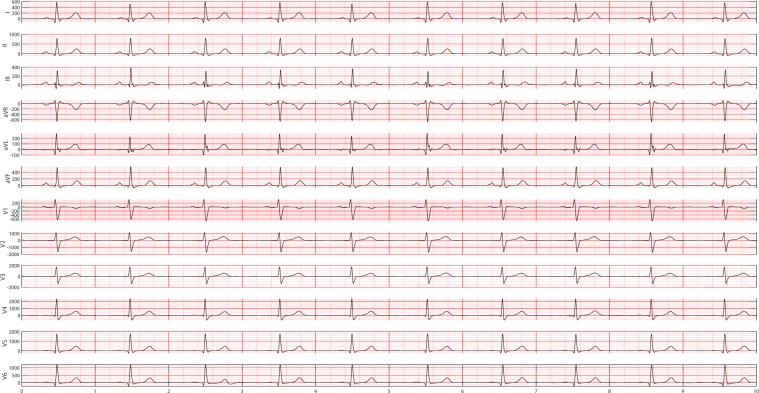
A 12-lead ECG depicted sinus bradycardia rhythm. Sinus bradycardia can be defined as a sinus rhythm with a resting heart rate of 60 beats per minute or less.

Each patient’s ECG data were collected over 10 seconds at a sampling rate of 500 Hz and labeled by cardiologist-supervised physicians. The data labels included 11 types of rhythm and 67 additional cardiac findings such as PVC, RBBB, LBBB and APC. A detailed description of the enrolled participants’ baseline characteristics and rhythm frequency distribution is presented in Table 6. Since some rare rhythms only have single unit readings, according to a suggestion from cardiologists, we have hierarchically merged several rare cases to upper-level arrhythmia types. After re-grouping labels of the dataset, this new setting of classes can significantly contribute to the training of the best approach. It also complies with and benefits the daily clinical practice. Thus, 11 rhythms were merged into 4 groups (SB, AFIB, GSVT, SR), SB only included sinus bradycardia, AFIB consisted of atrial fibrillation and atrial flutter (AFL), GSVT contained supraventricular tachycardia, atrial tachycardia, atrioventricular node reentrant tachycardia, atrioventricular reentrant tachycardia and wandering atrial pacemaker, and SR included sinus rhythm and sinus irregularity. Referring to guidelines^18–20^. that recommend AFIB and AFL often coexist, in the present study any ECG with a rhythm of AFIB or AFL was classified into AFIB group. Merging sinus rhythm and sinus irregularity to SR group helps with distinguishing such a combination from the GSVT group, and sinus irregularity can be easily separated from sinus rhythm later by one single criterion, RR interval variation. Supraventricular tachycardia actually is a general term used in the daily ECG screening. For example, if the cardiologists cannot confirm atrial tachycardia or atrioventricular node reentrant tachycardia purely by ECG, they will give the general name supraventricular tachycardia. Therefore, the practice of merging all tachycardia originating from supraventricular locations to GSVT group was adopted in this work. Figures 10, 11, 12, and 13 depict 12-lead ECGs of randomly selected patients from the SR, AFIB, GSVT and SB groups respectively. The detailed definition of rhythm groups subseuently used for classification and the definition of rhythms labeled by certified physicians are presented in Supplementary section A.

**Table 6:** Rhythm information and baseline characteristics of the enrolled participants.

Acronym Name	Full Name	Frequency, n(%)	Age, Mean ± SD	Male,n(%)
SB	Sinus Bradycardia	15,528 (38.6)	58.4 ± 14.02	9844 (63.4%)
SR	Sinus Rhythm	7,291 (18.1)	54.38 ± 16.17	4107 (56.33%)
AFIB	Atrial Fibrillation	7,028 (17.5)	73.07 ± 11.27	4051 (57.64%)
ST	Sinus Tachycardia	6,208 (15.4)	54.24 ± 21.41	3208 (51.68%)
AFL	Atrial Flutter	1,725 (4.3)	71.57 ± 13.23	1001 (58.03%)
SI	Sinus Irregularity	1,773 (4.4)	37.3 ± 22.98	979 (55.22%)
SVT	Supraventricular Tachycardia	542 (1.3)	55.44 ± 18.41	289 (53.32%)
AT	Atrial Tachycardia	133 (0.3)	65.92 ± 18.7	69 (51.88%)
AVNRT	Atrioventricular Node Reentrant Tachycardia	16 (0.03)	57.88 ± 17.34	12 (75%)
AVRT	Atrioventricular Reentrant Tachycardia	7 (0.01)	56.43 ± 17.89	5 (71.43%)
WAP	Wandering Atrial Pacemaker	7 (0.01)	51.14 ± 31.83	6(85.71%)

We also investigated a separate and important issue in ECG analysis, the evaluation of the impact of additional cardiac conditions such as PVC, LBBB, RBBB, or APC on the rhythm classification accuracy. We created a subset of the data containing ECGs of subjects without such conditions consisting of 20,766 samples. Details on the distribution of arrhythmia types in the two datasets and the p-values comparing their prevalence are shown in Table 7. The differences between the sample prevalence of SB, SR, AFIB, and GSVT are statistically significant with magnitudes of 4.4%, 10.3%, −7.3% and −7.4% respectively.

**Table 7:** Participants with and without additional cardiac conditions.

Rhythm	Participants With Additional Cardiac Conditions, n(%)	Participants Without Additional Cardiac Conditions, n(%)	P-value
SB	15,528 (38.57%)	8,914 (42.93 %)	0.001
SR	9,064 (22.52%)	6,812 (32.80 %)	0.001
AFIB	8,753 (21.74%)	3,003 (14.46 %)	0.001
GSVT	6,913 (17.17%)	2,037 (9.81 %)	0.001
Total	40,258 (100%)	20,766 (100 %)	

### Noise reduction

When the ECG data was collected, the major noise contamination sources were power line interference, electrode contact noise, motion artifacts, skeletal muscle contraction, baseline wandering, and random noise. The baseline wandering could be induced by respiration. The frequency of the power line interference is 50-60 Hz while the frequency of the baseline wander is less than 0.5 Hz. The currently available noise reduction methods have both pros and cons. Adaptive Filter^21^ possesses desirable performance, but the reference signal is hard to get. Wavelet and Band Pass Filter^22^ need predetermined thresholds, the Morphology Technique^23^ with dilation and erosion operation has similar issues. In the subsequent analyses, we implemented the Butterworth Low-pass filter to remove high-frequency noise (above 50 Hz), the Robust LOESS to eliminate baseline wandering and Non Local Means (NLM) to remove the remaining noise^24^.

#### Non local means denoising

The NLM algorithm was introduced to address the preservation of repeated structures in digital images^25^. Later, NLM was used to remove noise from ECG data^26^ and further combined with the Empirical Mode Decomposition (EMD)^27^.

NLM denoising reconstructs the true signal *S*(*i*) at all time points *i* through weighted averaging of all points *D*(*j*) within predefined range. The weights are determined by a similarity measure between *D*(*i* + *δ*) and *D*(*j* + *δ*), *δ* ∈ Δ.

3 $$S(i)=\frac{1}{Z(i)}\sum _{j\in N(i)}w(i,j)D(j)$$

where *Z*(*i*) = ∑_*j*_ *w*(*i*, *j*) and

4 $$w(i,j)=exp(-\frac{{\sum }_{\delta \in \Delta }{[D(i+\delta )-D(j+\delta )]}^{2}}{2{L}_{\Delta }{\lambda }^{2}})$$

wherein *λ* is a smoothness control parameter, and Δ represents a local patch of samples containing *L*_Δ_ samples. Thus, at each point, the NLM smoothing borrows information from all points that have similar patterns within the search range. The similarity measure determines how many periods will be included and averaged. We used the Gaussian kernel as a weight function in the smoothing step of our analysis. In this work, we included all data points in the patient 10-second ECG data, and set the patch window size to 10 and the smoothness control parameter *λ* to 1.5 times the estimated standard deviation of the noise *σ*. The median absolute deviation (MAD) method was used to estimate the variability of the noise.

5 $$\sigma =1.4826\ast MAD(R)=1.4826\ast median(| D-median(D)| )$$

where *median* denotes the median operator, and *D* = *D*(1), …, *D*(*l*), …, *D*(*L*) is the set of the local residuals of the selected homogeneous region of length L in the noisy signal *D*_*n*_ with *D*(*l*) = (2*D*_*n*_(*l*) − (*D*_*n*_(*l* − 1) + *D*_*n*_(*l* + 1)))/$\sqrt{6}$. After passing through the above three denoising filters, the high frequency noise and baseline wandering are removed from the raw ECG data.

### Features extraction

In previous studies, neural network models have been successfully employed in arrhythmia classification^6^. These models used sequential transformations of the raw data as features that were ultimately fed into a multinomial logistic regression classifier (softmax unit). The architecture of neural networks allows an infinite number of such models, and properly training even one of them requires large data and long computation time. Another common strategy is to extract features such as peak magnitudes, duration, distances between peaks, and their variability in the four major components of beats, P-wave, Q-wave, T-wave, and QRS complex. However, these features do not provide sufficient information for high accuracy classification of several arrhythmia types, especially the ones characterized by distortion or complete omission of some components. For instance, the P-waves of AFIB and AFL are commonly replaced by multiple flutter and fibrillation waves that are lower than a normal P-wave in amplitude and do not correspond to the QRS rhythm. Further, using Wavelet or Fast Fourier Transformation to extract frequency features will neglect the time domain information.

The major challenges of feature extraction are the variability in wave morphology among and within individuals and the distortion from various conditions. Moreover, individuals with different gender, age and race will have different ECGs in both amplitude and frequency. Thus, as a preliminary data manipulation step, we rescaled the ECG data using the maximum-minimum algorithm to unify the amplitude scale. We evaluated the rescaling influence for classification, and the performance of rescaling is discussed in the Results section.

**Figure 14:**
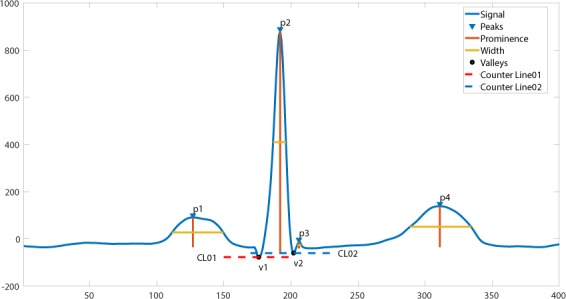
The definition of height, width, and prominence measurements in this study. The prominence of a peak or a valley measures how much the peak or valley stands out due to its intrinsic height and its location relative to neighbor peaks or valleys. Thus, the prominence is defined as the vertical distance between the peak point and its lowest contour line. For instance, the prominence of peak P2 is the vertical distance between point P2 and contour line CL02, rather than the distance between P2 and contour line CL01.

**Figure 15:**
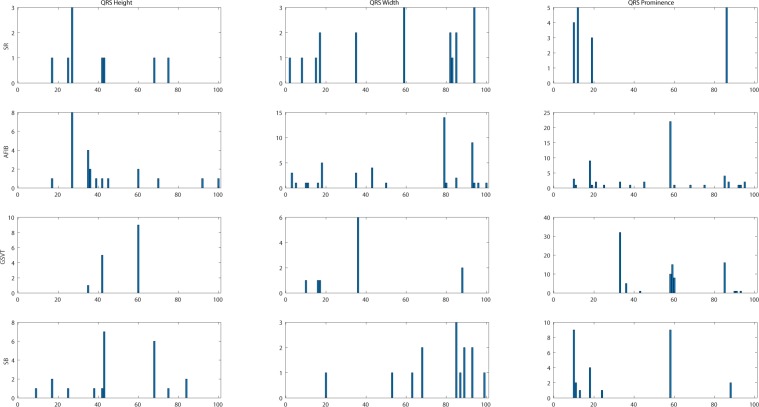
Empirical frequency distribution of QRS complex height, width, and prominence in lead II. The Y-axis presents the frequencies of height, width and prominence, and X-axis presents the scale that measures height, width and prominence of QRS complex. The unit step of X-axis is (the maximum of height, width or prominence - the minimum of height, width or prominence)/100. The frequencies shown in the rows named SR, AFIB, GSVT, and SB were computed from ECGs presented in Figs. 10, 11, 12, 13 respectively. The full demonstration for feature extraction from 12 leads ECG can be found in Supplementary section B.

**Figure 16:**
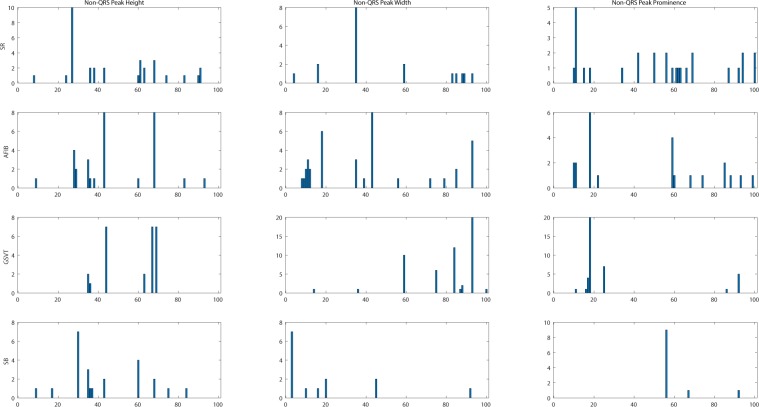
Empirical frequency distribution of non-QRS peaks height, width, and prominence in lead II. The Y-axis presents the frequencies of height, width and prominence, and X-axis presents the scale that measures height, width and prominence of non-QRS peaks. The unit step of X-axis is (the maximum of height, width or prominence - the minimum of height, width or prominence)/100.The frequencies shown in the rows named SR, AFIB, GSVT, and SB were computed from ECGs presented in Figs. 10,11,12,13 respectively. The full demonstration for feature extraction from 12 leads ECG can be found in Supplementary section B.

**Figure 17:**
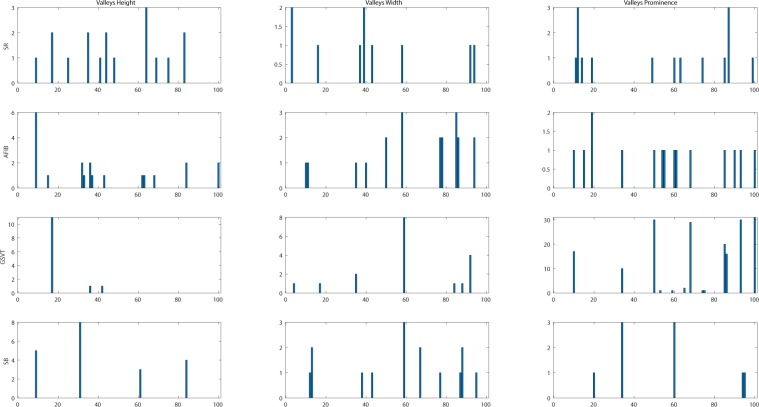
Empirical frequency distribution of valleys height, width, and prominence in lead II. The Y-axis presents the frequencies of height, width and prominence, and X-axis presents the scale that measures height, width and prominence of valleys. The unit step of X-axis is (the maximum of height, width or prominence - the minimum of height, width or prominence)/100.The frequencies shown in the rows named SR, AFIB, GSVT, and SB were computed from ECGs presented in Fig. 10,11,12,13 respectively. The full demonstration for feature extraction from 12 leads ECG can be found in Supplementary section B.

In this project, we designed a novel and interpretable feature extraction method. As a part of our comparison of competing multi-stage classification schemes, we carried out an analysis of feature selection approaches that included a total of 11 distinct scenarios. The first and simplest set of features only included 11 basic characteristics of the signal while the last and most exhaustive set included 39,830 features. We added age and gender as features due to their importance in almost all medical data analyses. Other meaningful features such as the mean and variance of the RR intervals as well as RR interval counts that are only computed in lead II ECG were also included. Table 8 shows other features, the Feature Group 1 includes ventricular rate in beats per minute (BPM), atrial rate in BPM, QRS duration in millisecond, QT interval in millisecond, R axis, T axis, QRS count, Q onset, Q offset, totally 11 variables. The Feature Group 2 in Table 8 includes mean and variance of RR intervals, RR interval count, mean and variance of height, width, and prominence of QRS complex, non-QRS peaks, and valleys in lead II ECG, totally 23 variables. As depicted in Fig. 14, peaks and valleys here represent the local maxima and minima. The prominence of a peak or a valley measures how much the peak or valley stands out due to its intrinsic height and its location relative to neighbor peaks or valleys. Thus, the prominence is defined as the vertical distance between the peak point and its lowest contour line. For instance, the prominence of peak P2 in Fig. 14 is the vertical distance between point P2 and contour line CL02, rather than the distance between P2 and contour line CL01. The peaks and valleys were assigned to 3 subsets, QRS complex, non-QRS peaks, and Valleys. So that the relationship among peaks and valleys were measured on 6 distinct pairwise combinations, which consist of QRS complex Vs QRS complex, non-QRS peaks Vs non-QRS peaks, valleys Vs valleys, QRS complex Vs non-QRS peaks, QRS complex Vs valleys, and non-QRS peaks Vs valleys. Sequentially, for the 6 distinct pairwise combinations mentioned above, we computed the ratio of width difference over time difference, the ratio of height difference over time difference, and the ratio of prominence difference over time difference. However, such ratios cannot be directly used as feature inputs to the classification model, since each patient will have a different number of such ratios. Therefore, we formed an empirical frequency distribution table spanning 100 groups between the maximum value and minimum value of ratios. The same empirical frequency distribution table was constructed for the attributes of peaks and valleys (height, width, and prominence), and the location difference between peaks and valleys in 6 distinct pairwise combinations. For instance, in Figs. 15, 16, 17 the frequencies of each variable (height, width and prominence) that extracted from Lead II can be used as features feeding into a classification model and each variable has uniform 100 length. The full demonstration for feature extraction from 12 leads ECG can be found in Supplementary section B. Thus, the Feature Group 6 is designed for lead II ECG and consists of a total of 900 frequencies of height, width, prominence for QRS complex, non-QRS peaks, and valleys; a total of 600 frequencies of location difference; and a total of 1800 frequencies of the ratio between width difference and time difference, the ratio between height difference and time difference, and the ratio between prominence difference and time difference. The remaining feature groups derive from the features in group 1, 2, and 6. From what has been discussed above, we proposed a feature extraction method that can fully reveal the empirical frequency distribution of P, Q, R, S, T and the segments among them, the key factors to identify rhythms, and the results discussed later testified such strategy is reliable and robust.

**Table 8:** Feature groups table.

Feature Group	Feature Description	Number of Features
1	Ventricular Rate, Atrial Rate, QRS Duration, QT Interval, R axis, T axis, QRS count, Q Onset, Q Offset	11
2	Mean of RR intervals, Variance of RR intervals, RR interval count, mean and variance of height, width, prominence for QRS complex, non-QRS peaks, and valleys in lead II	23
3	Features in Group 1, mean of RR, variance of RR interval, RR interval count, mean and variance of height, width, prominence for QRS complex, non-QRS peaks, and valleys in lead II	32
4	Mean of RR interval, Variance of RR interval, RR interval count, mean and variance of height, width, prominence for QRS complex, non-QRS peaks, and valleys in all leads	221
5	Features in Group 1, Mean of RR interval, Variance of RR interval, RR interval count, mean and variance of height, width, prominence for QRS complex, non-QRS complex, and valleys in all leads	230
6	For lead II ECG, a total of 900 frequencies of height, width, prominence for QRS complex, non-QRS peaks, and valleys; a total of 600 frequencies of location difference for QRS complex Vs QRS complex, non-QRS peaks Vs non-QRS peaks, valleys Vs valleys, QRS complex Vs non-QRS peaks, QRS complex Vs valleys, and non-QRS peaks Vs valleys; a total of 1800 frequencies including ratio between difference in heights and difference in locations, between difference in width and difference in locations, between difference in prominence and difference in locations, for QRS complex Vs QRS complex, non-QRS peaks Vs non-QRS peaks, valleys Vs valleys, QRS complex Vs non-QRS peaks, QRS complex Vs valleys, and non-QRS peaks Vs valleys.	3,302
7	Features in Group 2 and Group 6	3,323
8	Features in Group 1, Group 2, and group 6	3,332
9	Features in Group 6 in all leads	39,602
10	Features in Group 4 and Group 9	39,821
11	Features in Group 3 and Group 9	39,830

### Grid search for hyperparameters

We carried out the additional analysis that focused on the optimal selection of hyperparameters via a comprehensive grid search. We selected the optimal values of the hyperparameters based on the maximum average F_1_-Score over 10-fold validation datasets. The hyperparameters and the corresponding classification algorithms implemented in the scikit-learn package^28^ are presented in Table 9.

**Table 9:** Hyperparameters table.

Model Name	Hyperparameter Name	Hyperparameter Options
DT	criterion	‘gini’, ‘entropy’
	splitter	‘best’, ‘random’
	max_features	‘auto’, ‘sqrt’, ‘log2’, None
KNN	n_neighbors	15 31
	weights	‘uniform’, ‘distance’
	algorithm	‘ball_tree’, ‘kd_tree’
NC	shrink_threshold	0.01, 0.1, 0.2, 0.3
GNB	var_smoothing	10^−7~−12^
MNB	alpha	0, 0.1, 0.5, 0.8,1
CNB	alpha	0, 0.1, 0.5, 0.8,1
BNB	alpha	0, 0.1, 0.5, 0.8,1
MLR	solver	‘newton-cg’, ‘lbfgs’, ‘saga’, ‘sag’
RRC	alpha	1e-3, 1e-2, 1e-1, 1
	solver	‘svd’, ‘cholesky’, ‘lsqr’, ‘sparse_cg’, ‘sag’, ‘saga’
LCSGD	loss	‘hinge’, ‘log’, ‘modified_huber’, ‘squared_hinge’, ‘perceptron’
	alpha	1e-3, 1e-2, 1e-1, 1
	learning_rate	‘constant’, ‘optimal’, ‘invscaling’, ‘adaptive’
	eta0	0.01, 0.001, 0.0001
PAC	C	0.001, 0.01, 0.1,1
	loss	‘hinge’, ‘squared_hinge’
SVC	loss	‘hinge’, ‘squared_hinge’
	C	0.001, 0.01, 0.1, 1
RF	n_estimators	300, 500, 800
	criterion	‘gini’, ‘entropy’
	bootstrap	True, False
	max_features	‘auto’, ‘sqrt’, ‘log2’, None
ERT	n_estimators	300, 500, 800
	criterion	‘gini’, ‘entropy’
	bootstrap	True, False
	max_features	‘auto’, ‘sqrt’, ‘log2’, None
GBT	loss	deviance, exponential
	learning_rate	0.1, 0.01, 0.001, 0.1
	subsample	0.1, 0.5, 0.9
	n_estimators	300, 500, 800
	max_features	‘auto’, ‘sqrt’, ‘log2’, None
EGBT	tree_method	‘auto’, ‘exact’, ‘approx’, ‘hist’
	grow_policy	‘depthwise’, ‘lossguide’
	n_estimators	300, 500, 800
	learning_rate	0.001, 0.01
	max_depth	10, 15, 20, 50, 100

### Ensemble classification methods

After completing the identification of the optimal hyperparameters, ensemble machine learning methods based on multiple sampling can be used to improve classification results. We studied two families of ensemble methods: averaging, and boosting. The first method consists of building numerous classifiers that are independently trained on different observed samples, and the individual results are averaged. This approach has the computational advantage of carrying out the independent training steps in parallel. In contrast, the second method builds a set of classification models that will work sequentially. A boosting model *i* is trained to classify observations. The misclassified samples from model *i* are added to the training samples for model *i* + 1. This process continues until a quasi-optimal model with the lowest misclassification probability is obtained. In this work we compared Bagging Average^29^, Random Forest^30^, AdaBoost^31^, GBT, EGBT^32^, and ERT^33^.

Multi-classification problems can be decomposed into multiple binomial classification problems. In this study, we compared several strategies for the above decomposition such as, One Vs Rest, One Vs One, and Error-Correcting Output-Codes. After combining ensemble methods and the multi-classification strategies with meta classifiers including DT, KNN, NC, GNB, MNB, CNB, BNB, LC, QDA, MLR, MPN, RRC, LCSGD, PAC, SVC, RF, ERT, GBT and EGBT, 98 different combinations were compared in this study. Using 10-fold cross-validation, we found the best hyperparameters through an exhaustive grid search method. These optimal values attained the highest weighted average F_1_-Score for the validation datasets.

### Gradient boosting tree classifier

Gradient boosting tree classifier is an additive model that assembles a certain number of weak classifiers such as decision trees. The boosting procedure optimizes a cost function to find the best group of decision trees. Explicit regression gradient boosting algorithms^34,35^ were developed by Jerome H. Friedman. Unlike popular stochastic gradient decent optimization, gradient boosting tree classifier needs to learn both best-fit functions and hyperparameters. The boosting tree model is a sum of *M* decision trees, which can be formulated as following:

6 $${f}_{M}(x)=\mathop{\sum }\limits_{m=1}^{M}T(x;{\theta }_{m})$$

where

7 $$T(x;\theta )=\mathop{\sum }\limits_{j=1}^{J}{\gamma }_{j}I(x\in {R}_{j})$$

with parameters $\theta ={\{{R}_{j},{\gamma }_{j}\}}_{1}^{J}$. Decision tree partitions the space of all joint predictor values into disjoint regions *R*_*j*_, *j* = 1, 2, . . . , *J*. A constant *γ*_*j*_ is assigned to each such region according to the rule *x* ∈ *R*_*j*_ → *f*(*x*) = *γ*_*j*_. Therefore, after training data is given, the learning object is to minimize the cost or loss function to find *θ*_*m*_. Since directly minimizing the loss function *L*(*y*_*i*_ − *f*_*M*_(*x*)) is difficult, it can be approximated in a forward stagewise boosting fashion by minimizing loss function iteratively in (6) at a time.

8 $$L({y}_{i},{f}_{m-1}({x}_{i})+T({x}_{i};{\theta }_{m}))$$

where $${\theta }_{m}={\{{R}_{jm},{\gamma }_{jm}\}}_{1}^{{J}_{m}}$$.

If the region *R*_*jm*_ are given, finding the optimal constants *γ*_*jm*_ in each region is typically straightforward:

9 $${\widehat{\gamma }}_{jm}=argmi{n}_{{\gamma }_{jm}}\sum _{{x}_{i}\in {R}_{jm}}L({y}_{i},{f}_{m-1}({x}_{i})+{\gamma }_{jm})$$

Thus, the key question turns to finding proper regions *R*_*jm*_ and the one approximated solution is to fit the *m*^*th*^ iteration tree function *T*(*x*; *θ*_*m*_) as close as possible to the negative gradient *g*_*im*_ = *I*(*y*_*i*_ = *C*_*k*_) − *P*_*k*_(*x*) where *P*_*k*_(*x*) is the probability of the outcome variable that belongs to the ${K}_{}^{th}$ class *C*_*k*_.

10 $${P}_{k}(x)=\frac{{e}^{{f}_{k}(x)}}{{\sum }_{l=1}^{K}{f}_{l}(x)}$$

Finally, a pseudo gradient boosting algorithm is given as following^36^:

1. Start with a constant model *f*_*k*0_, *k* = 1, 2, …, *K*;
2. For *m* = 1 to *M*:
  2.1 For *k* = 1, 2, …, *K*:
    2.1.1 compute *r*_*ikm*_ = *y*_*ik*_ − *p*_*k*_(*x*_*i*_), *i* = 1, 2, …, *N*;
    2.1.2 Fit a regression tree to the targets *r*_*ikm*_, *i* = 1, 2, …, *N*, giving terminal regions *R*_*jkm*_, *j* = 1, 2, …, *J*_*m*_;
    2.1.3 For *j* = 1, 2, …, *J*_*m*_ compute $${\gamma }_{jkm}=\frac{K-1}{K}\ast \frac{\sum _{{x}_{i}\in {R}_{jkm}}{r}_{ikm}}{\sum _{{x}_{i}\in {R}_{jkm}}| {r}_{ikm}| (1-| {r}_{ikm}| )}$$;
    2.1.4 Update $${f}_{km}(x)={f}_{k,m-1}(x)+\mathop{\sum }\limits_{j=1}^{{J}_{m}}{\gamma }_{jkm}I(x\in {R}_{jkm})$$;
3. Output $${\widehat{f}}_{k}(x)={f}_{kM}(x),k=1,2,\ldots ,K.$$;

The GBT and EGBT models used in this work were both tree boosting models but with different numerical implementations. EGBT enhances the boosting optimization by the Newton-Raphson method and provides more hyperparameters for the penalization of trees and the shrinking of the leaf nodes.

## Supplementary information

Supplementary Information.

## Data Availability

The source code of converter tool that converts ECG data file from XML format to CSV format can be found https://github.com/zheng120/ECGConverter, which contains both binary executable files, source code, and the user manual. The MATALB program for ECG denoising was put under https://github.com/zheng120/ECGDenoisingTool.

## Data availability

Data records presented in this work consist of four parts: raw ECG data, denoised ECG data, diagnostic file, and attributes dictionary file. These files are available online from figshare (Data Citation 1: Figshare 10.6084/m9.figshare.c.4560497.v1).
