# Supplementary material for: Optimal Multi-Stage Arrhythmia Classification Approach
Source: Sci Rep. 2020 Feb 19;10:2898. doi: 10.1038/s41598-020-59821-7 (PMC7031229; doi:10.1038/s41598-020-59821-7)
Supplement: Supplementary file 1 — Supplementary Information. [file 41598_2020_59821_MOESM1_ESM.pdf]

Optimal Multi-Stage Arrhythmia Classification Approach

Jianwei Zheng [1], Huimin Chu[2], Daniele Struppa [1], Jianming Zhang[3], Sir Magdi Yacoub[4], Hesham El-Askary[1], Anthony Chang[5], Louis Ehwerhemuepha[1,5], Islam Abudayyeh[6], Alexander Barrett[1], Guohua Fu[2], Hai Yao[7], Dongbo Li[2], Guo Hangyuan[3] (corresponding author) and Cyril Rakovski[1]

[1]Chapman University, Orange, CA, USA
[2]Ningbo First Hospital of Zhejiang University, Ningbo, Zhejiang, China
[3]Shaoxing People's Hospital (Shaoxing Hospital Zhejiang University, Shaoxing, Zhejiang, China School of Medicine)
[4]Imperial College London, London, England
[5]CHOC Children's Hospital, Orange, CA, USA
[6]Loma Linda University International Heart Institute, Loma Linda, Riverside, USA
[7]Zhejiang Cachet Jetboom Medical Devices CO.LTD, Hangzhou, Zhejiang, China

Supplementary Materials

Section A.
The definition of rhythm groups and rhythms
In this study, a total 11 rhythms whose definitions were shown below were merged into 4 groups (SB, AFIB, GSVT, SR). In particular, SB only included sinus bradycardia, AFIB consisted of atrial fibrillation and atrial flutter (AFL), GSVT contained supraventricular tachycardia, atrial tachycardia, atrioventricular node reentrant tachycardia, atrioventricular reentrant tachycardia and sinus atrium to atrial wandering rhythm, and SR included sinus rhythm and sinus irregularity. Thus, our classification model was designed to classify these 4 groups of arrhythmias.

The definition table of 4 rhythm groups and the rhythms included.

Group Name	Arrhythmia Included in Group
SB	sinus bradycardia
AFIB	atrial fibrillation (AFIB), atrial flutter (AFL)
GSVT	supraventricular tachycardia (SVT), atrial tachycardia (AT), atrioventricular node reentrant tachycardia (AVNRT), atrioventricular reentrant tachycardia (AVRT) and sinus atrium to atrial wandering rhythm (SAAWR)
SR	sinus rhythm without other conditions and sinus irregularity

The definition table of 11 Rhythms labeled by cardiologists.

Acronym Name	Full Name	Definition
SB	Sinus Bradycardia	Sinus bradycardia can be defined as a sinus rhythm with a resting heart rate of 60 beats per minute or less.
SR	Sinus Rhythm	Normal sinus rhythm usually accompanies a heart rate of 60 to 100 beats per minute, with less than 0.16 second variation in the shortest and longest duration between successive P waves, and normal PR interval, QRS complex and QT interval.
AFIB	Atrial Fibrillation	ECG showing atrial fibrillation will have no visible P waves that are replaced by coarse fibrillatory waves and an irregularly irregular QRS complex.
ST	Sinus Tachycardia	Sinus tachycardia is recognized on an ECG with a normal upright P wave in lead II preceding every QRS complex, with an atrial rate of greater than 100 beats per minute.
AFL	Atrial Flutter	Atrial flutter is a health condition (arrhythmia) where the atria of the heart as an electrical problem (a re-entry loop) that causes the atria to beat at a rapid rate of about 242 - 360 beats per minute (bpm). Although usually flutter waves are regular and appear as "sawtooth" P waves in ECG's (typical atrial flutter); occasionally electrical conduction blocks can occur and produce 2:1, 3:1 or 4:1 wave or even appear as irregular bpm's resembling an irregular arrhythmia.
SI	Sinus Irregularity	A heart rate of 60 to 100 beats, P, QRS, T waves clear, normal PR interval, QRS complex, and AT interval, but the

		difference of two consecutive RR intervals greater than 120 milliseconds
SVT	Supraventricular Tachycardia	In this study, SVT refers to an abnormally fast (160~220 bpm) heart rhythm arising from improper electrical activity above the ventricle of the heart, but cannot confirm specified locations by ECG. The SVT might be atrial fibrillation, atrial flutter, or Wolff–Parkinson–White syndrome.
AT	Atrial Tachycardia	Atrial tachycardia is a fast heart rhythm in which the electrical impulse originates in atrial tissue different than the sinoatrial node. Atrial electrical activation during atrial tachycardias is mostly regular and by definition at a rate faster than 100 bpm.
AVNRT	Atrioventricular Node Reentrant Tachycardia	Rate: 118 to 264 bpm Rhythm: regular, narrow QRS complex (< 120 msec); regular, wide QRS complex (≥ 120 msec); may not see any P-wave activity in either type (atypical or typical)Atypical AVNRT: RP interval > PR interval; P waves negative in leads III and aVF Typical AVNRT: RP interval < PR interval; pseudo R wave in lead V1 with tachycardia, not with normal sinus rhythm; pseudo S wave in leads I, II, and aVF.
AVRT	Atrioventricular Reentrant Tachycardia	Rate: 124 to 256 bpm Rhythm: regular, narrow QRS complex common (orthodromic); regular, wide QRS complex uncommon (orthodromic or antidromic) if bundle branch block or aberrancy present. Orthodromic AVRT: RP interval < PR interval or RP interval > PR interval with a slowly conducting accessory pathway; retrograde P waves (leads I, II, III, aVF, V1); delta wave seen with normal sinus rhythm, not with tachycardia. Antidromic AVRT: short RP interval (< 100 msec); regular, wide QRS complex (≥ 120 msec); delta waves are seen with normal sinus rhythm and tachycardia; concealed accessory pathways do not show delta waves
SAAWR	Sinus Atrium to Atrial Wandering Rhythm	is an atrial rhythm with multiple P-wave morphologies (three or more), often occurring in a repetitive pattern at a rate of less than 100 bpm.

Section B.

Features extracted from 12-lead ECG demonstration.

For demonstration purposes, we used 36 graphics to show the features that are extracted from height, width, and prominence of QRS complex, non-QRS peaks, and valleys from 12 leads ECG. In addition, the four patient's ECG data that are assigned to SR, AFIB, GSVT, and SB were depicted in rows of each graphic below. The Y-axis presents the frequencies of height, width and prominence, and X-axis presents the scale that measures height, width and prominence of peaks and valleys. The unit step of the X-axis is: (the maximum of height, width or prominence - the minimum of height, width or prominence)/100.

Lead I

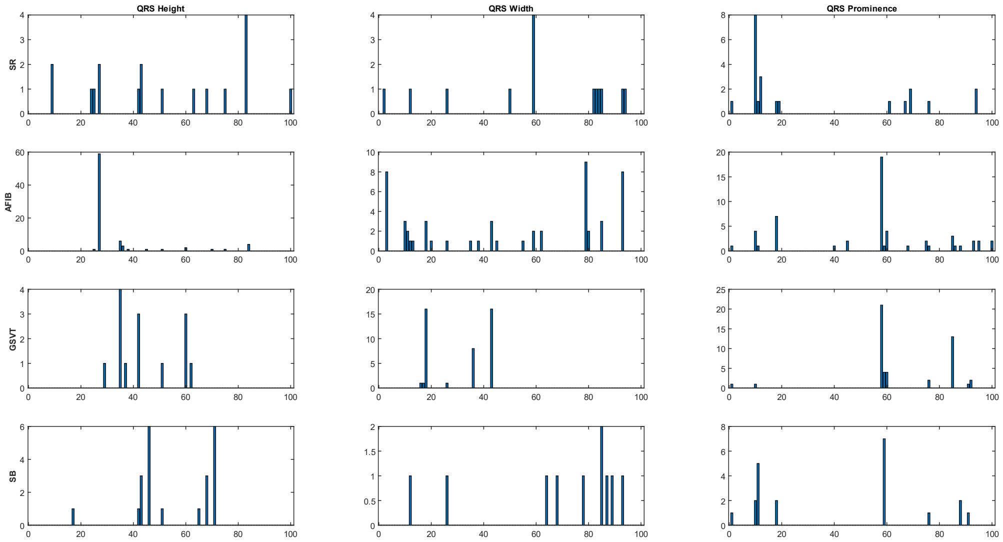

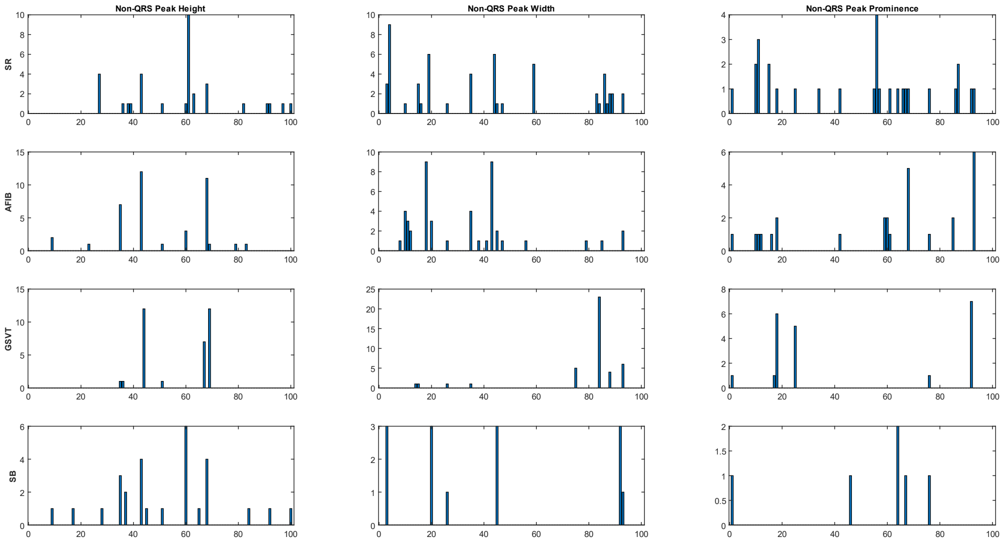
Non-QRS Peak Height
Non-QRS Peak Width
Non-QRS Peak Prominence
SR
AFIB
GSVT
SB

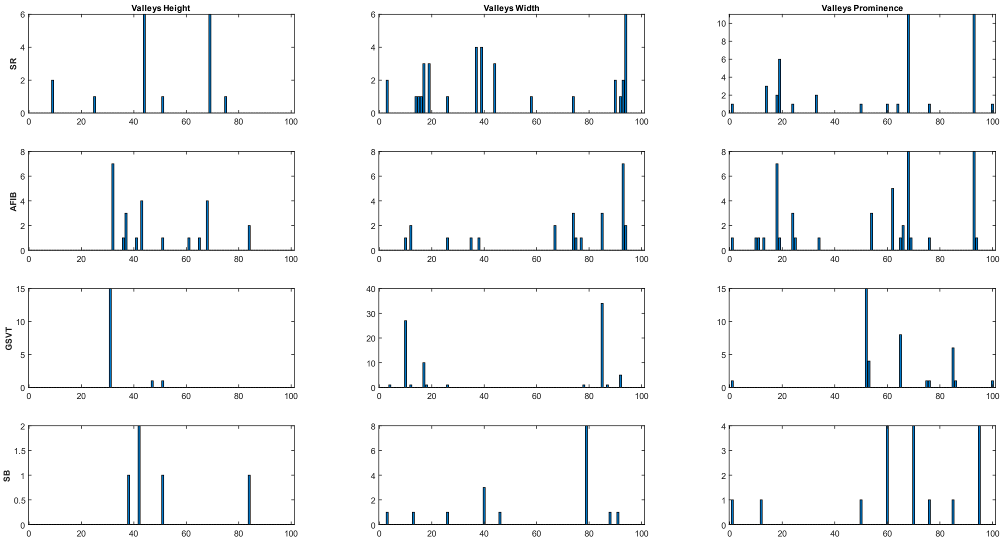
Valleys Height
Valleys Width
Valleys Prominence
SR
AFIB
GSVT
SB

Lead II

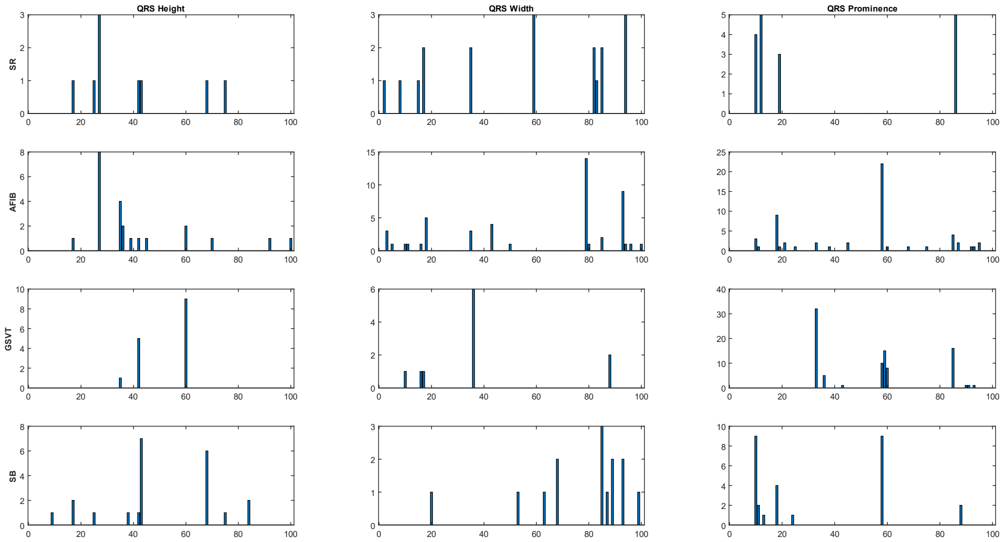
QRS Height
QRS Width
QRS Prominence
SR
AFIB
GSVT
SB

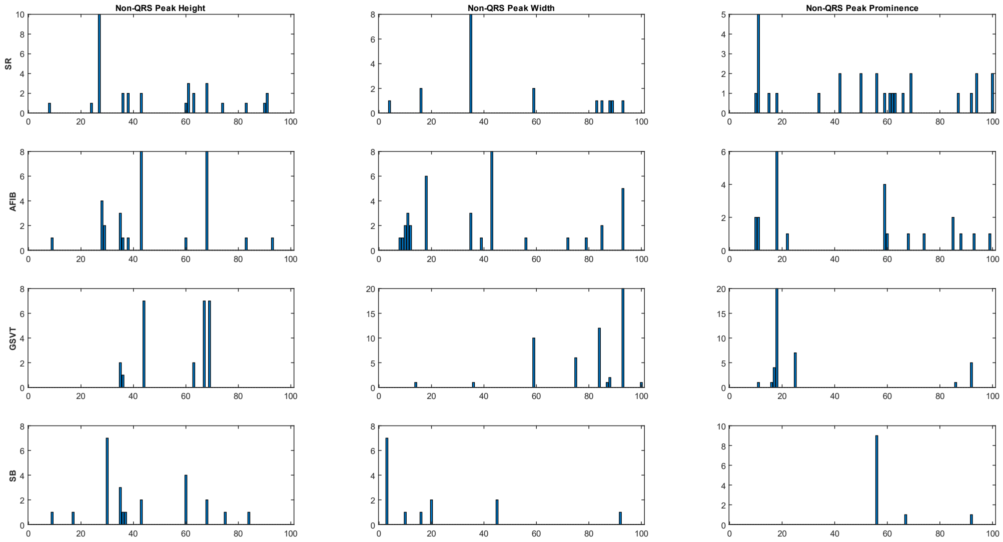
Non-QRS Peak Height
Non-QRS Peak Width
Non-QRS Peak Prominence
SR
AFIB
GSVT
SB

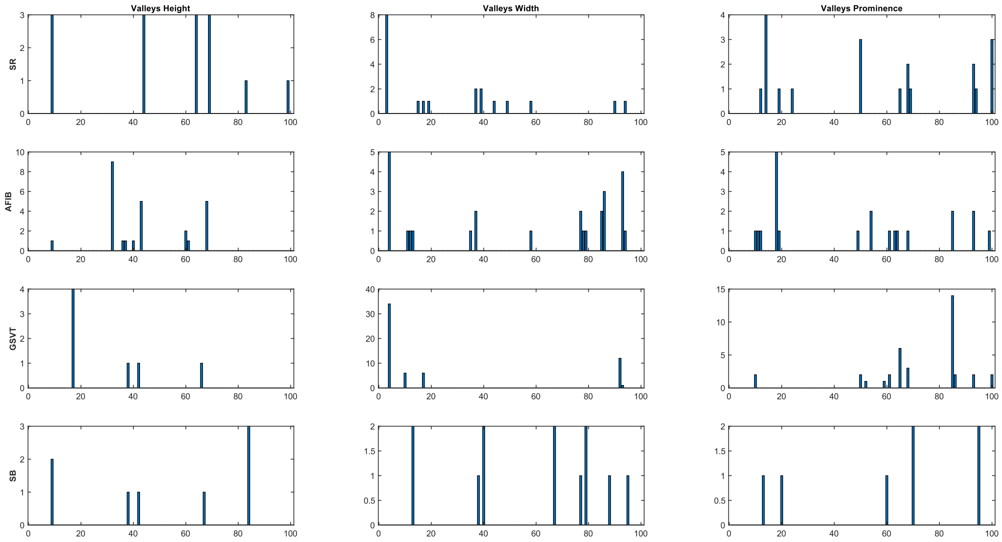
Valleys Height
Valleys Width
Valleys Prominence
SR
AFIB
GSVT
SB

Lead III

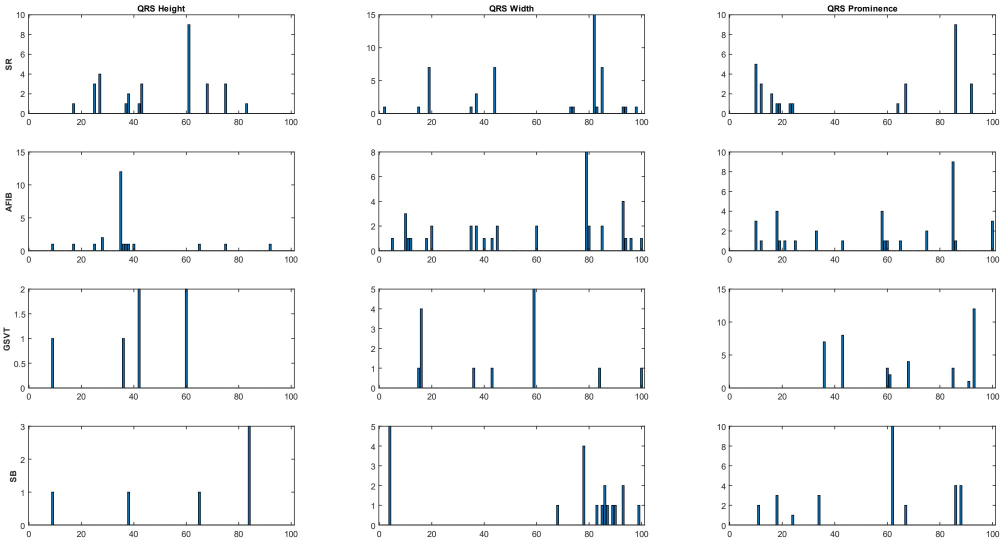
QRS Height
QRS Width
QRS Prominence
SR
AFIB
GSVT
SB

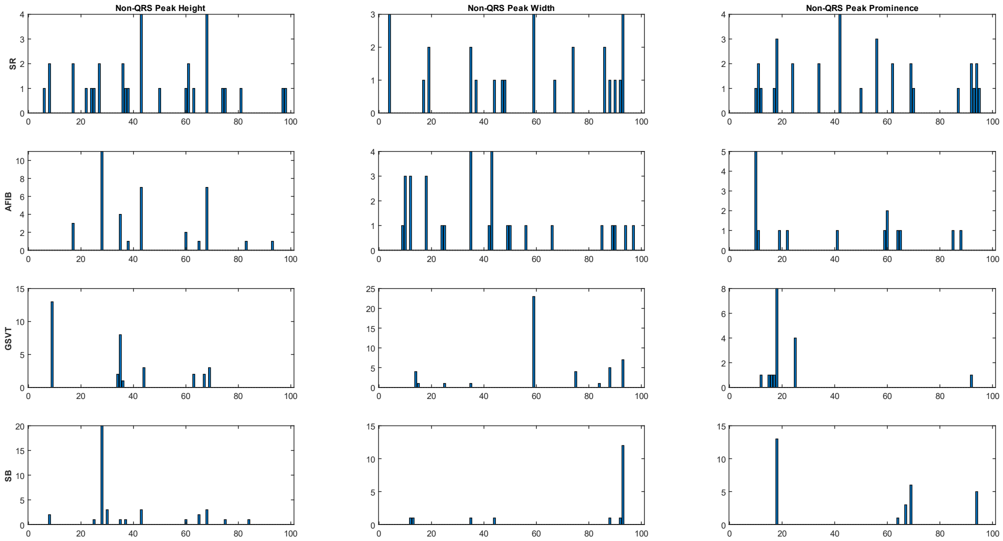
Non-QRS Peak Height
Non-QRS Peak Width
Non-QRS Peak Prominence
SR
AFIB
GSVT
SB

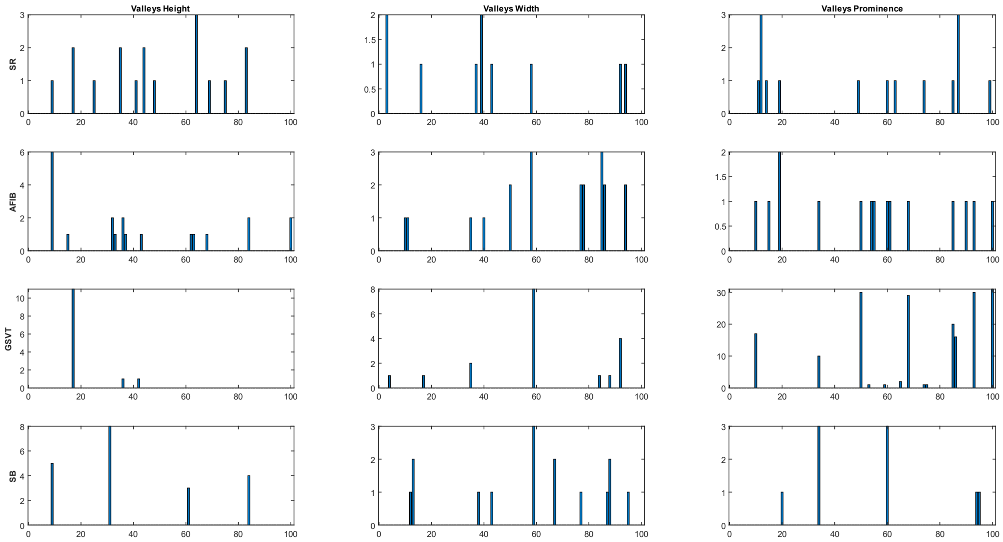
Valleys Height
Valleys Width
Valleys Prominence
SR
AFIB
GSVT
SB

Lead aVR

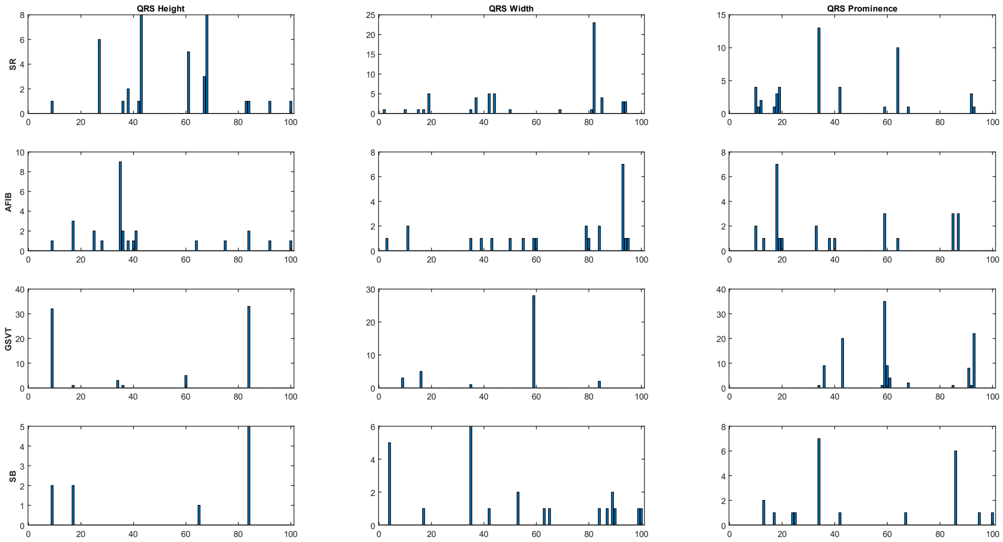
QRS Height
QRS Width
QRS Prominence
SR
AFIB
GSVT
SB

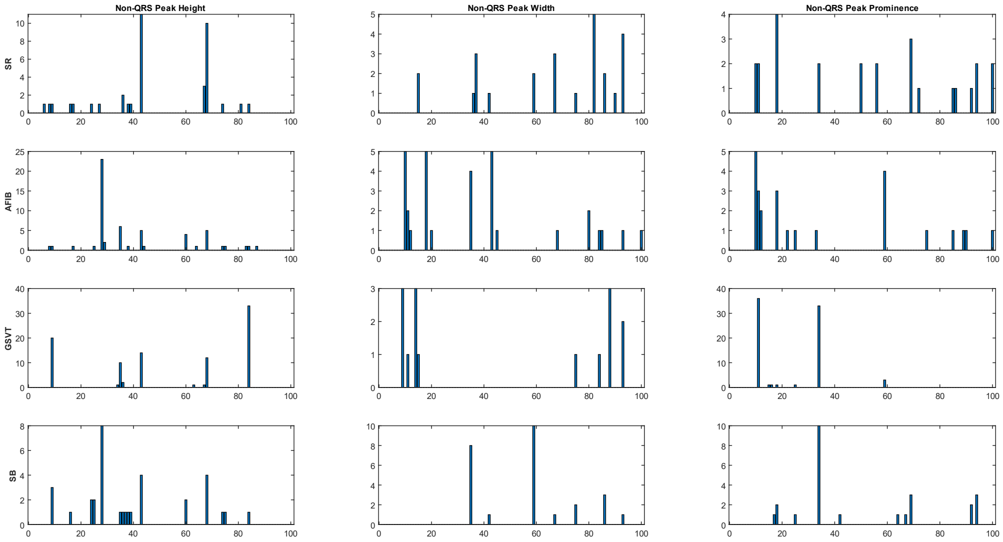
Non-QRS Peak Height
Non-QRS Peak Width
Non-QRS Peak Prominence
SR
AFIB
GSVT
SB

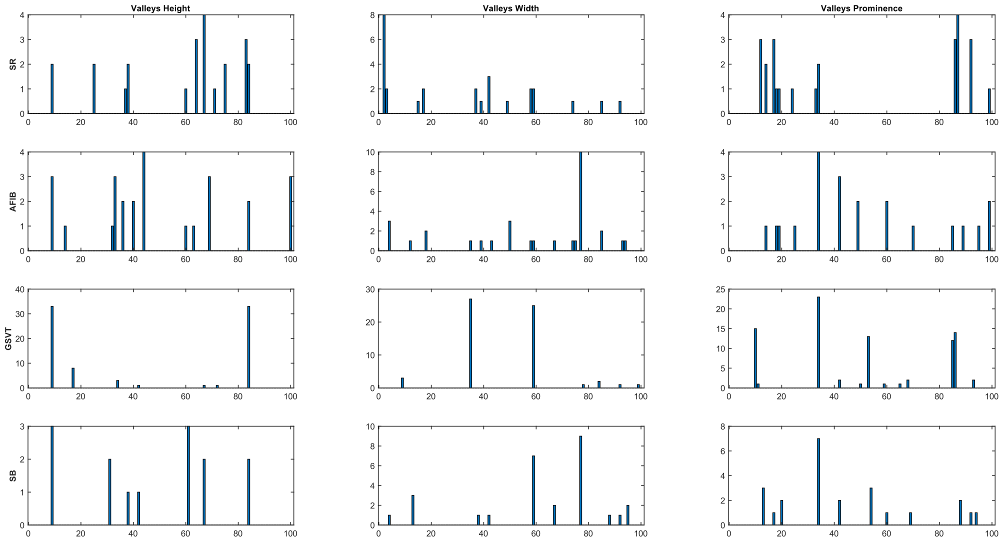
Valleys Height
Valleys Width
Valleys Prominence
SR
AFIB
GSVT
SB

Lead aVF

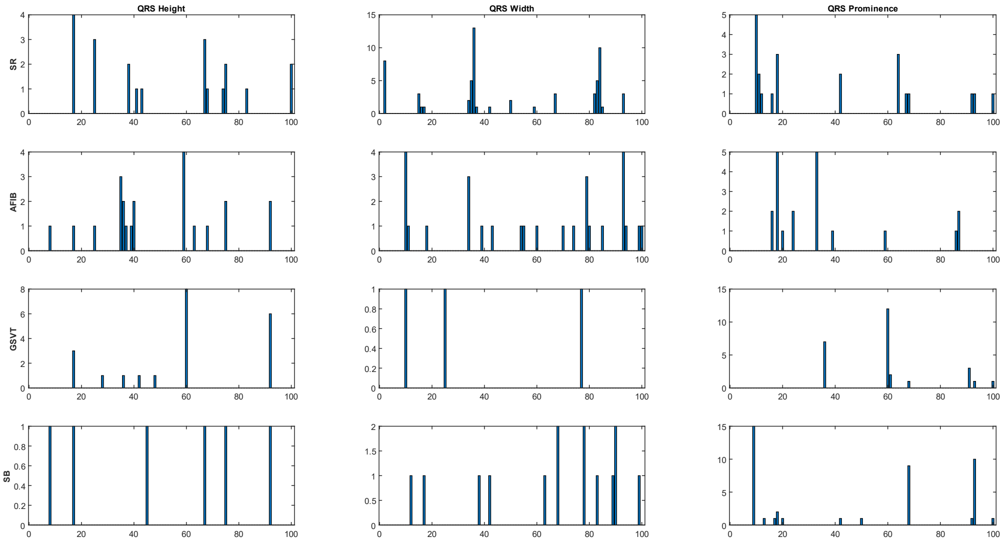
QRS Height
QRS Width
QRS Prominence
SR
AFIB
GSVT
SB

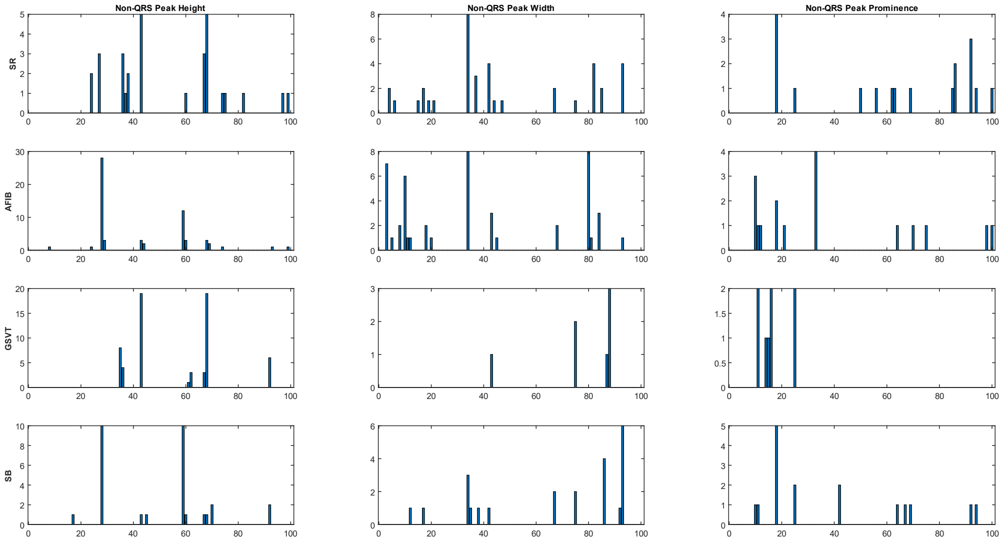
Non-QRS Peak Height
Non-QRS Peak Width
Non-QRS Peak Prominence
SR
AFIB
GSVT
SB

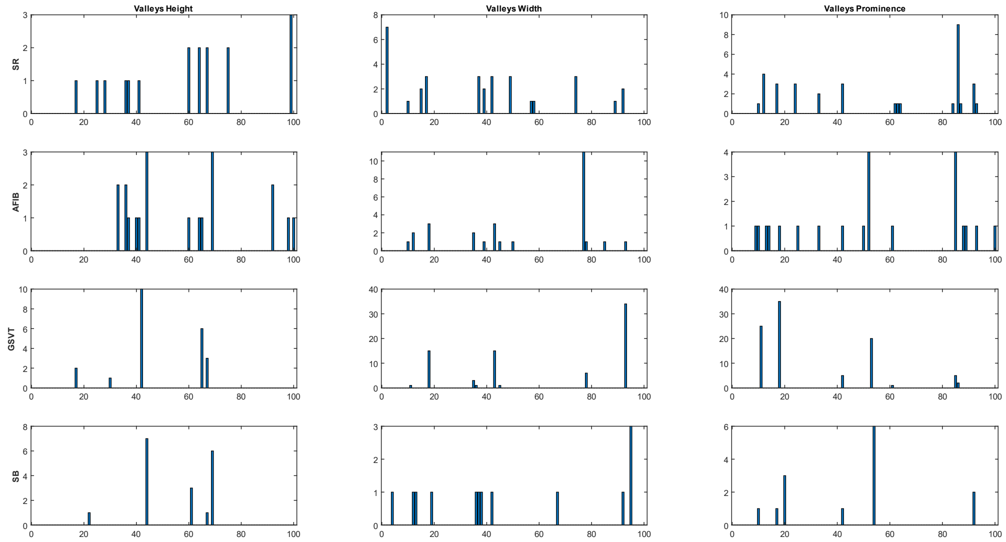
Valleys Height
Valleys Width
Valleys Prominence
SR
AFIB
GSVT
SB

Lead aVL

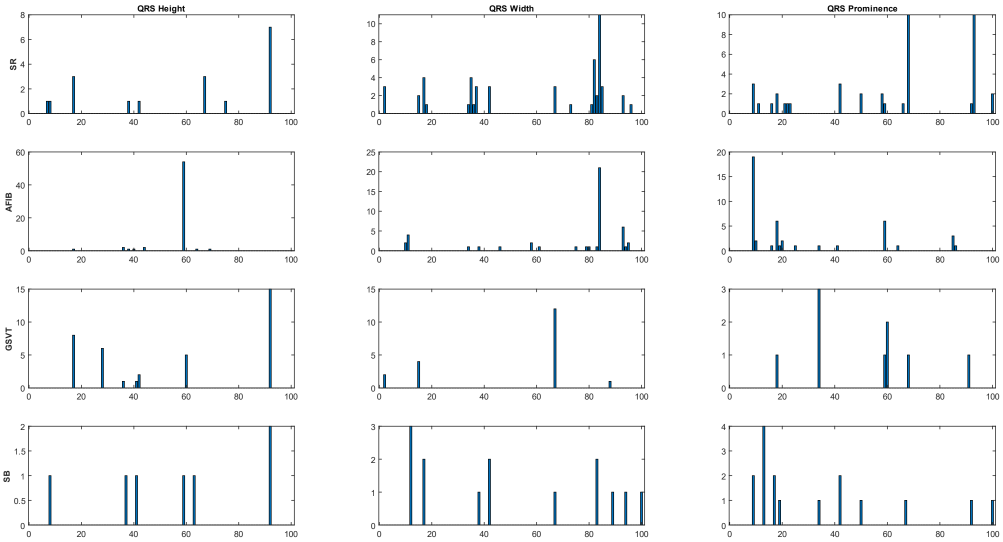
QRS Height
QRS Width
QRS Prominence
SR
AFIB
GSVT
SB

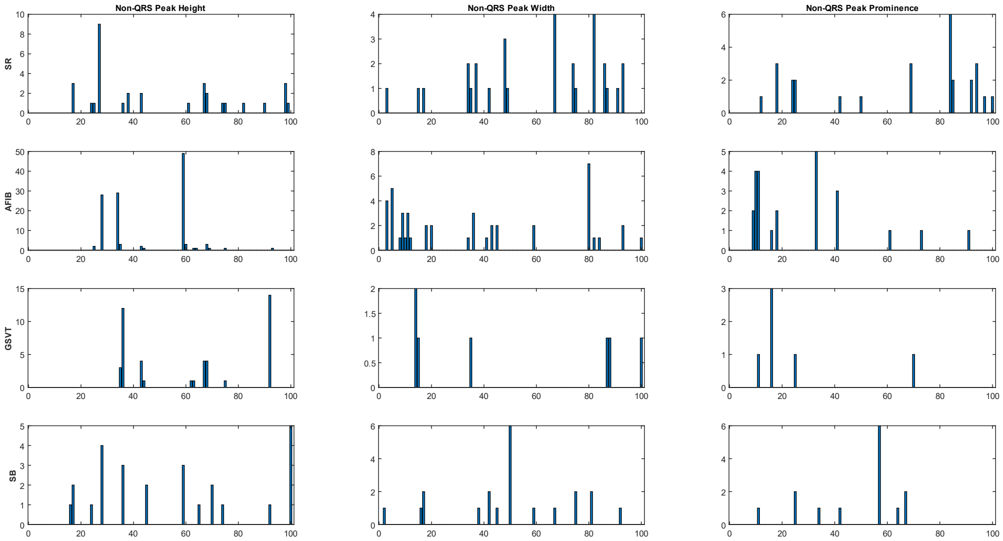
Non-QRS Peak Height
Non-QRS Peak Width
Non-QRS Peak Prominence
SR
AFIB
GSVT
SB

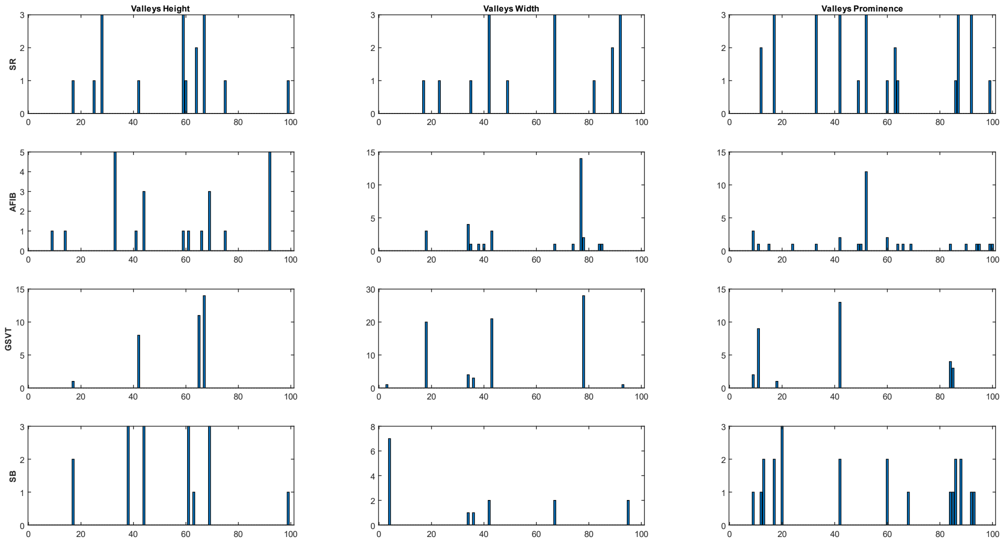
Valleys Height
Valleys Width
Valleys Prominence
SR
AFIB
GSVT
SB

Lead V1

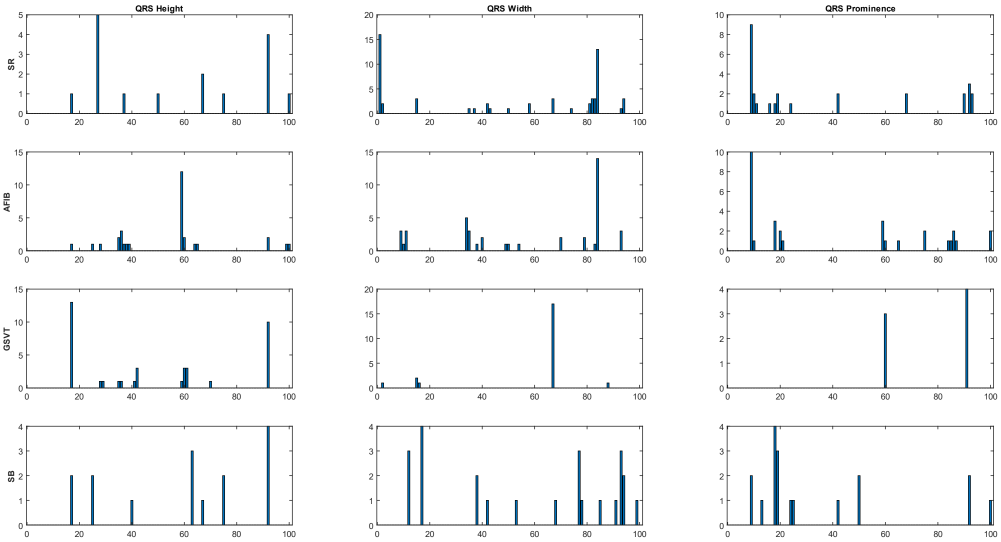
QRS Height
QRS Width
QRS Prominence
SR
AFIB
GSVT
SB

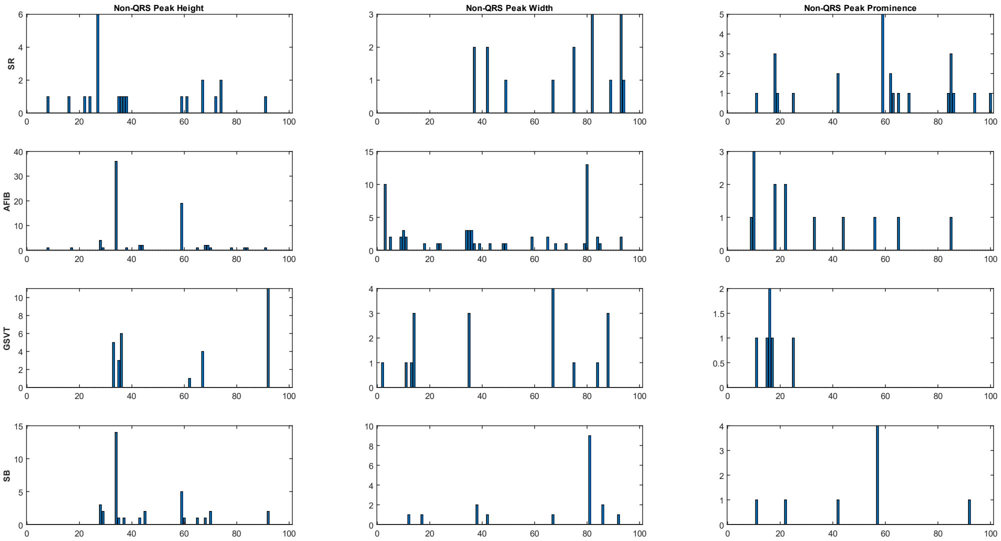
Non-QRS Peak Height
Non-QRS Peak Width
Non-QRS Peak Prominence
SR
AFIB
GSVT
SB

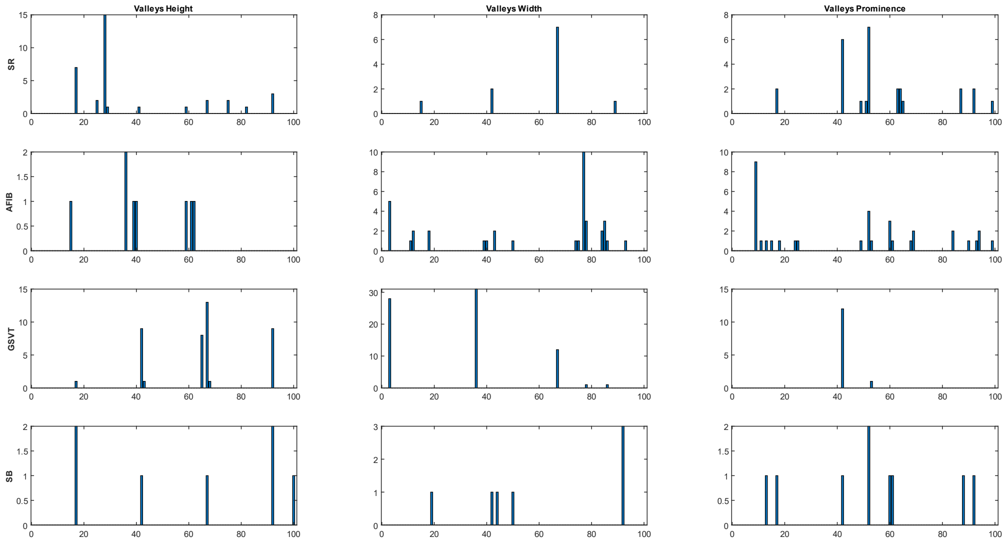
Valleys Height
Valleys Width
Valleys Prominence
SR
AFIB
GSVT
SB

Lead V2

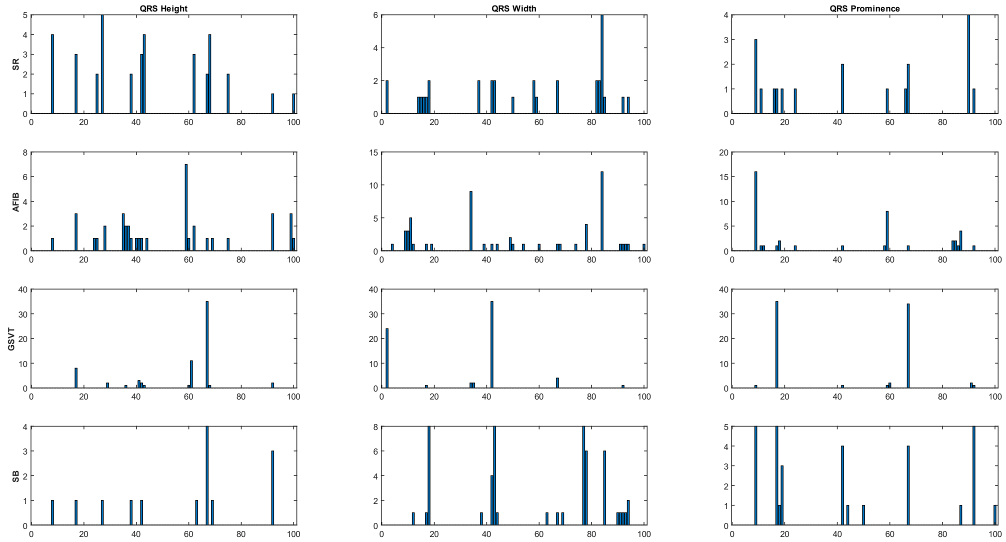
QRS Height
QRS Width
QRS Prominence
SR
AFIB
GSVT
SB

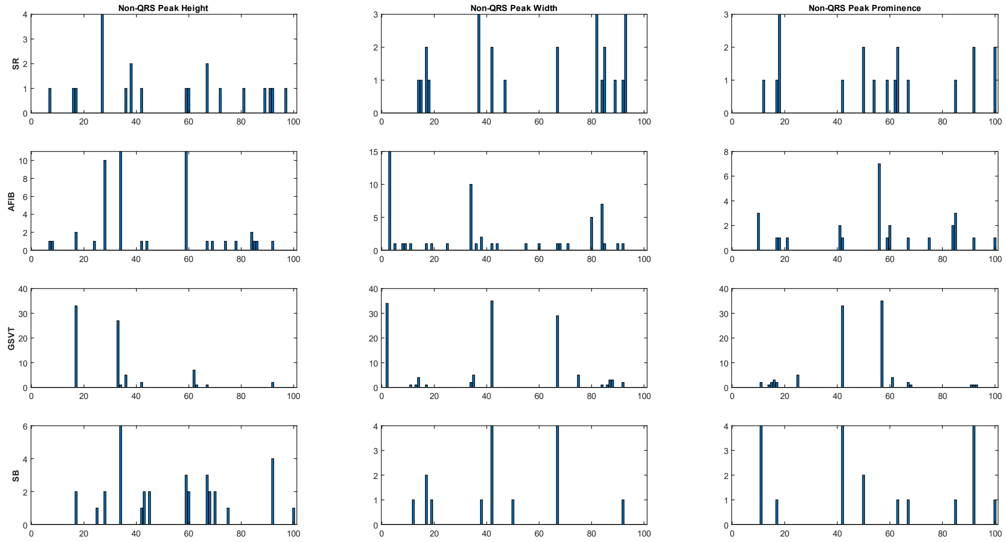
Non-QRS Peak Height
Non-QRS Peak Width
Non-QRS Peak Prominence
SR
AFIB
GSVT
SB

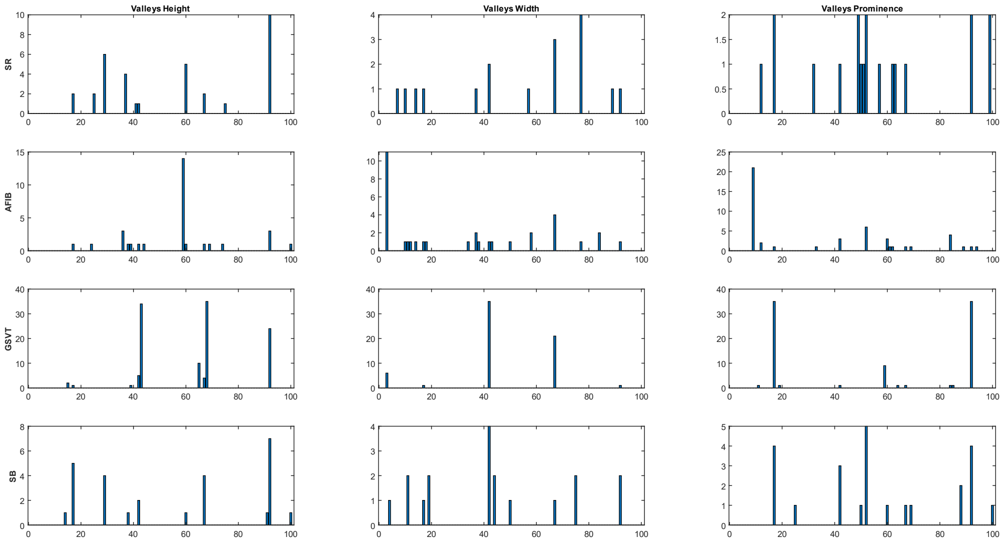
Valleys Height
Valleys Width
Valleys Prominence
SR
AFIB
GSVT
SB

Lead V3

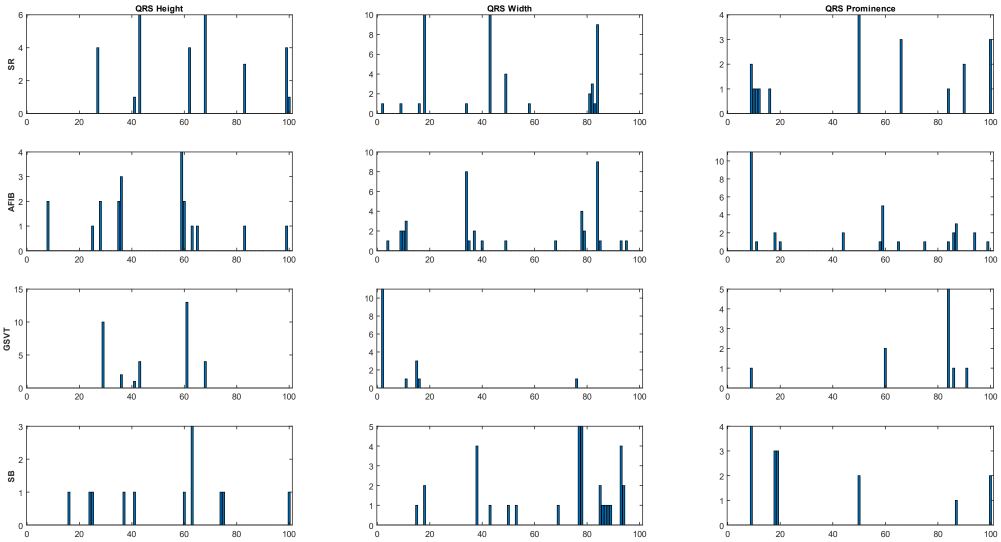
QRS Height
QRS Width
QRS Prominence
SR
AFIB
GSVT
SB

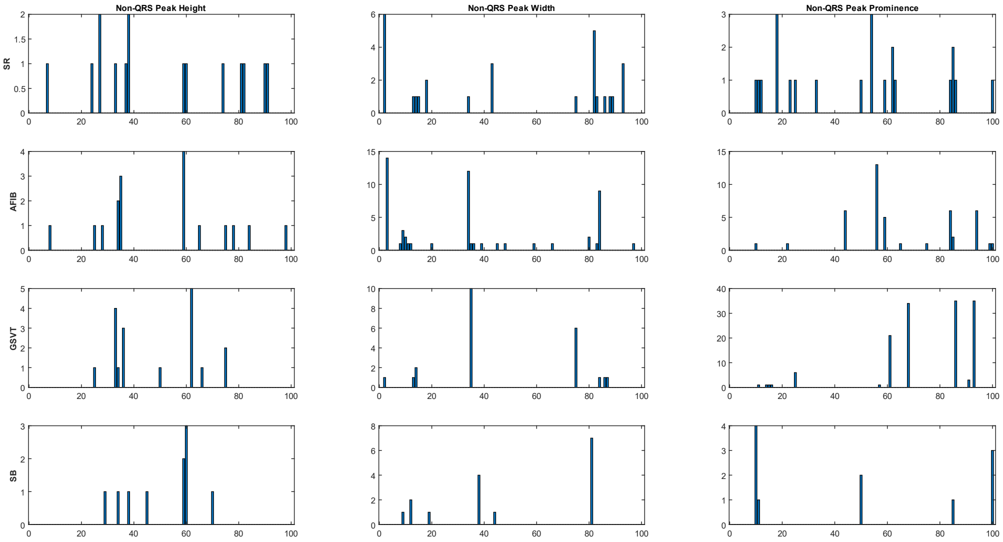
Non-QRS Peak Height
Non-QRS Peak Width
Non-QRS Peak Prominence
SR
AFIB
GSVT
SB

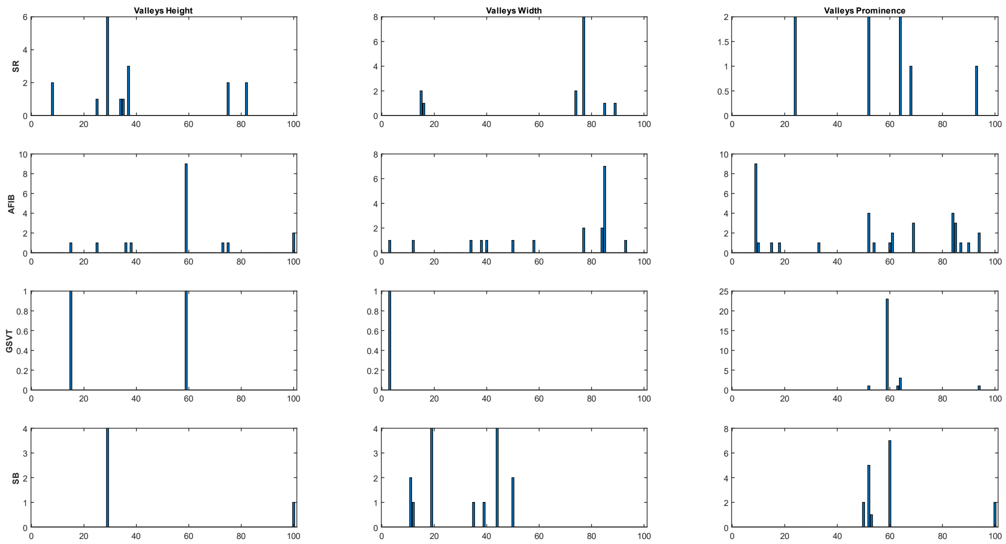

Valleys Height
Valleys Width
Valleys Prominence
SR
AFIB
GSVT
SB

Lead V4

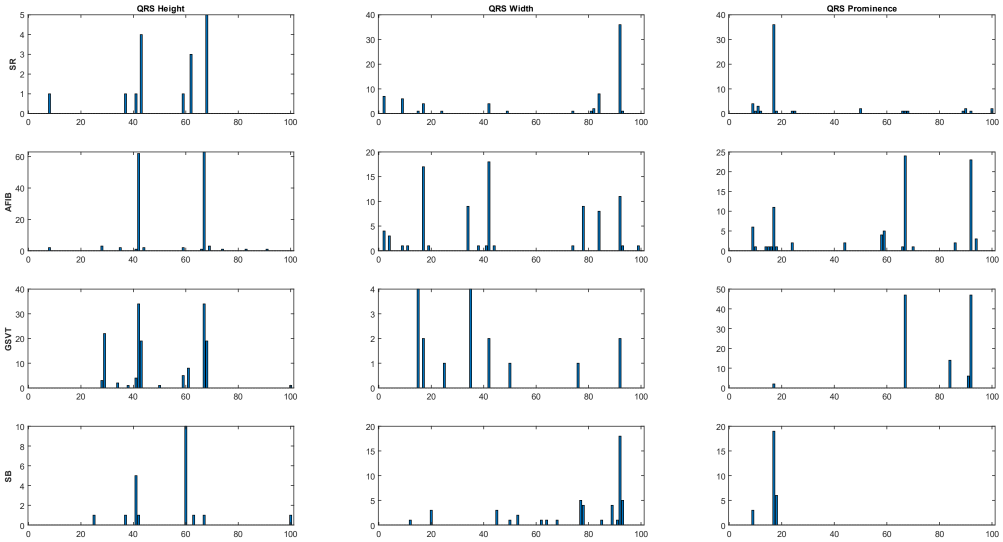
QRS Height
QRS Width
QRS Prominence
SR
AFIB
GSVT
SB

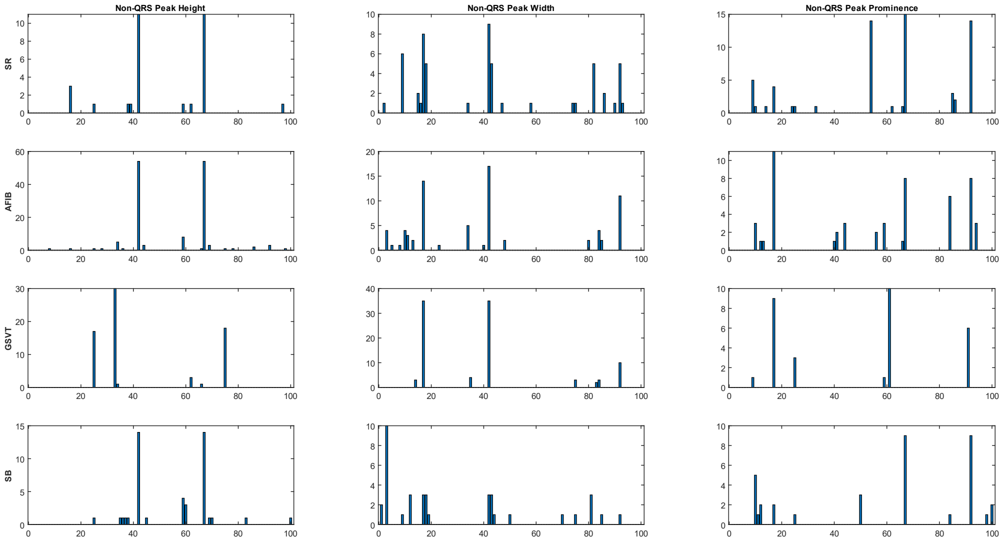
Non-QRS Peak Height
Non-QRS Peak Width
Non-QRS Peak Prominence
SR
AFIB
GSVT
SB

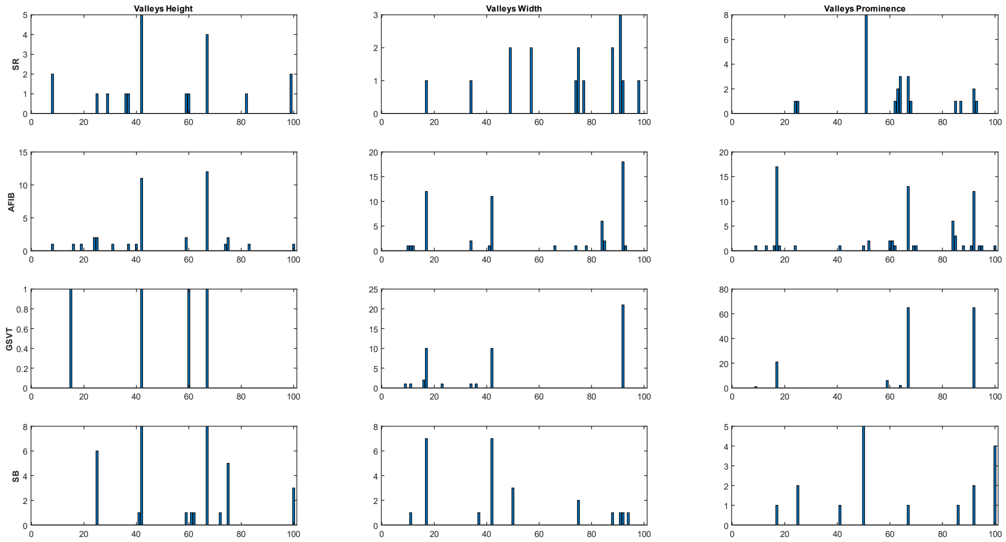
Valleys Height
Valleys Width
Valleys Prominence
SR
AFIB
GSVT
SB

Lead V5

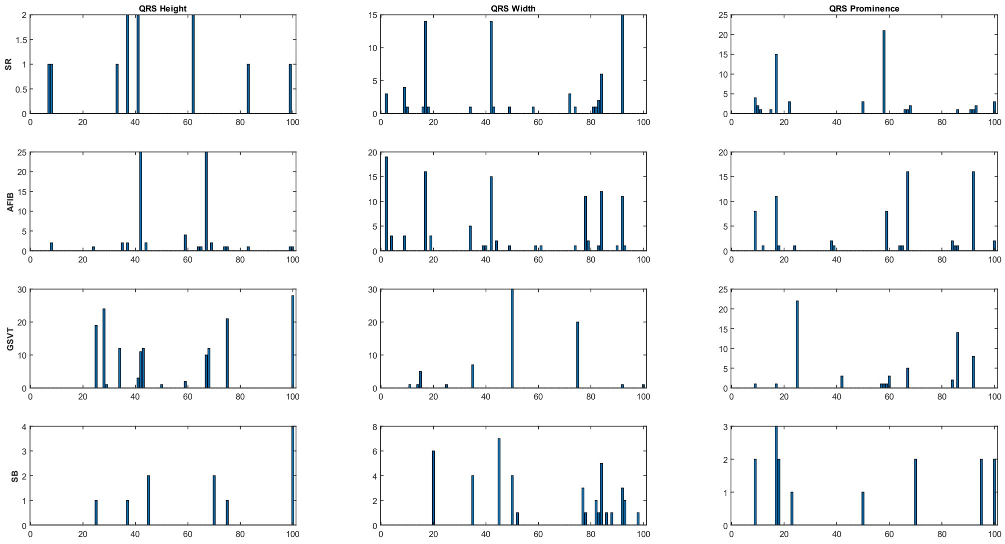
QRS Height
QRS Width
QRS Prominence
SR
AFIB
GSVT
SB

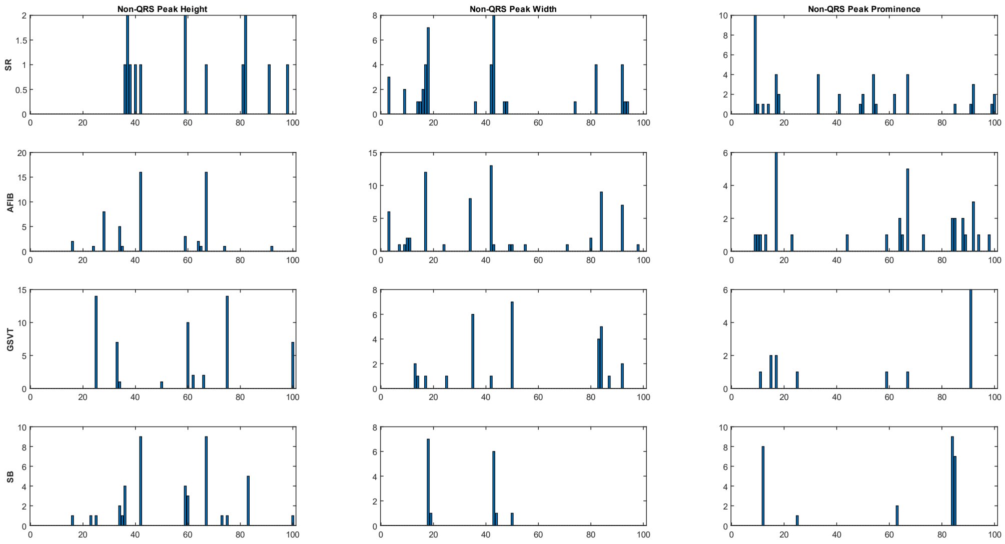
Non-QRS Peak Height
Non-QRS Peak Width
Non-QRS Peak Prominence
SR
AFIB
GSVT
SB

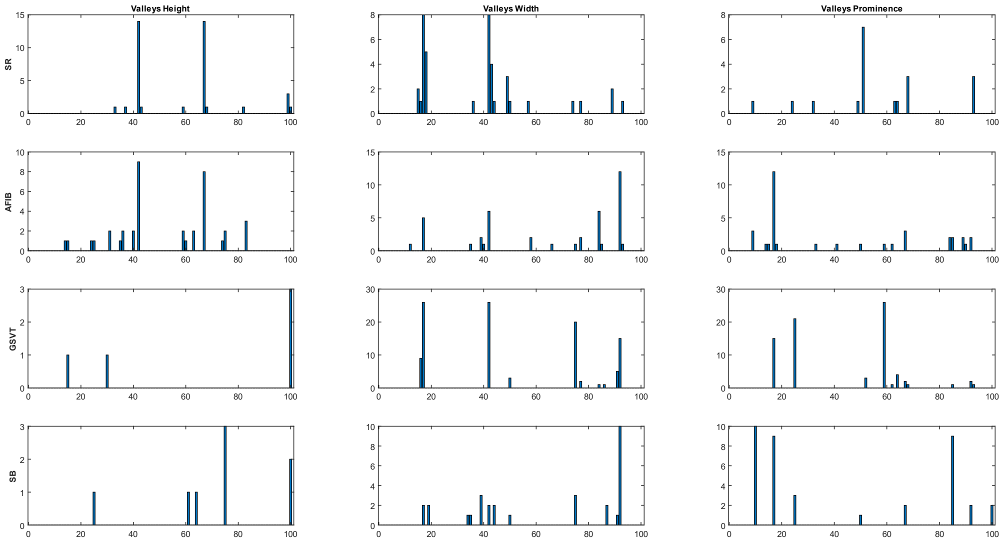
Valleys Height
Valleys Width
Valleys Prominence
SR
AFIB
GSVT
SB

Lead V6

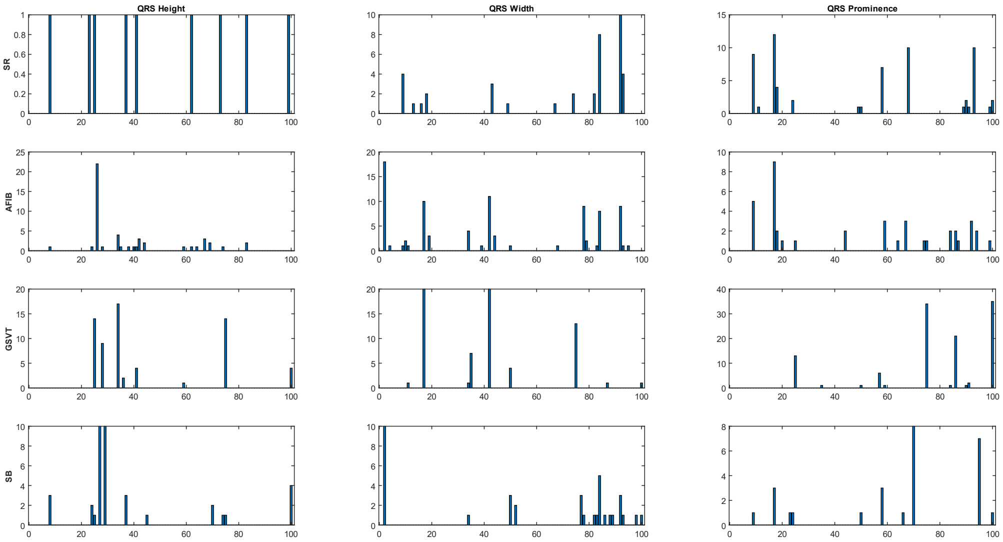
QRS Height
QRS Width
QRS Prominence
SR
AFIB
GSVT
SB

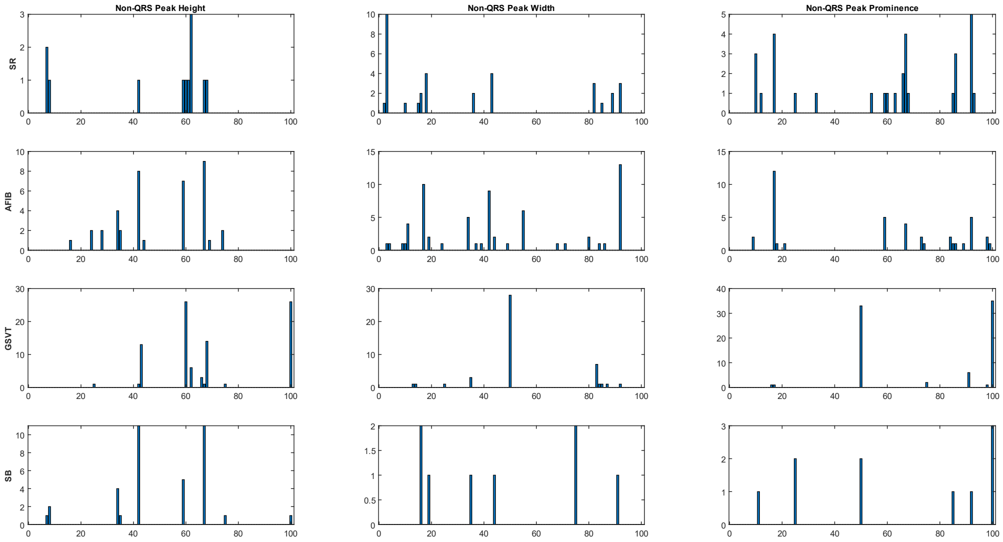
Non-QRS Peak Height
Non-QRS Peak Width
Non-QRS Peak Prominence
SR
AFIB
GSVT
SB

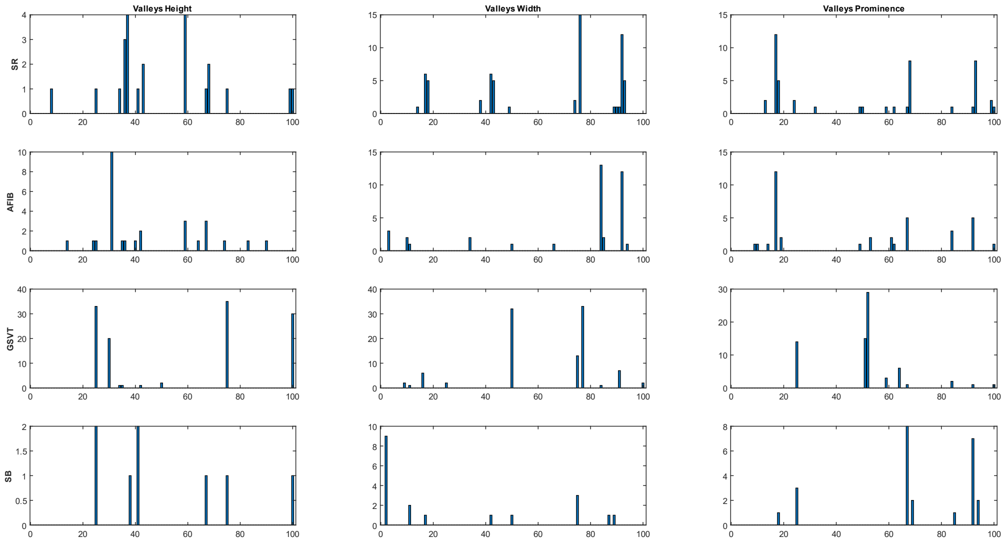

Section C.
Prsentation of 98 methods (models).
We compared a total of 98 methods (models) in the main paper list them below.

1) A total of 19 base classifiers below were studied:
Decision Tree (DT), K Nearest Neighbors (KNN), Nearest Centroid (NC), Gaussian Naive Bayesian (GNB), Multinomial Naive Bayesian (MNB), Complement Naive Bayesian (CNB), Bernoulli Naive Bayesian (BNB), Linear Classifier (LC), Quadratic Discriminant Analysis (QDA), Multinomial Logistic Regression (MLR), Multi-layer Perceptron Neural Net (MPN), Ridge Regression Classifier (RRC), Linear Classifiers with Stochastic Gradient Descent (LCSGD), Passive Aggressive Classifier (PAC), Linear SVC (SVC), Random Forest (RF), Extremely Randomized Trees (ERT), Gradient Boosting Tree (GBT) and Extreme Gradient Boosting Tree (EGBT)

2) Bagging classifier is a meta-estimator and uses above all 19 base classifiers as base classifiers.

3) This study, AdaBoost classifier is a meta-estimator and utilizes following base classifiers:
DT, GNB, MNB, CNB, BNB, MLR, RRC, LCSGD, and SVC.

4) Error-Correcting Output-Codes is another meta-estimator and use following base classifiers:
DT, KNN, GNB, MNG, CNB, BNB, MLR, LC, QDA, RRC, LCSGD, PAC, MPN, SVC, RF, ERT, and GBT.

5) One Vs Rest is a meta-estimator and use following base classifier:
DT, KNN, GNB, MNB, CNB, BNB, MLR, LC, QDA, RRC, LCSGD, PAC, MPN, SVC, RF, ERT, and GBT.

6) One Vs Rest is a meta-estimator and use following base classifier:
DT, KNN, GNB, MNB, CNB, BNB, MLR, LC, QDA, RRC, LCSGD, PAC, MPN, SVC, RF, ERT, and GBT.

Section D
Comparison result of 4, 312 classification schemes.

We trained 98 models as mentioned above by 22 datasets, and each dataset was derived by rescale and non-rescale transformation, respectively. Thus, a total of 4,312 classification schemes were compared, and the result was presented blow.

Model Number: matches the sequence of the model presented in section C.
Dataset Number: 0 presents the dataset without other conditions, 1 presents the dataset with other conditions.
Featuregroup Number: matches with the feature group presented in Feature Groups Table introduced in the main manuscript.
Rescale: 0 presents the dataset with rescaling transformation (normalization), 1 presents the dataset without rescaling transformation.

The result of Model Number:Dataset Number:Featuregroup Number:Rescale of 0 : 0 : 0 : 0

	f1-score	precision	recall	support
AFIB	0.798803	0.796217	0.801431	875.0
GSVT	0.808066	0.810576	0.805650	691.0
SB	0.852640	0.850785	0.854507	1553.0
SR	0.836917	0.840865	0.833037	907.0
macro avg	0.824106	0.824611	0.823656	4026.0
micro avg	0.829749	0.829749	0.829749	4026.0
weighted avg	0.829747	0.829789	0.829749	4026.0

The result of Model Number:Dataset Number:Featuregroup Number:Rescale of 0 : 0 : 0 : 1

	f1-score	precision	recall	support
AFIB	0.651113	0.646856	0.655484	875.0
GSVT	0.670466	0.676170	0.664946	691.0
SB	0.713801	0.712247	0.715364	1553.0
SR	0.696406	0.699202	0.693642	907.0
macro avg	0.682947	0.683619	0.682359	4026.0
micro avg	0.688803	0.688803	0.688803	4026.0
weighted avg	0.688820	0.688904	0.688803	4026.0

The result of Model Number:Dataset Number:Featuregroup Number:Rescale of 0 : 0 : 1 : 0

	f1-score	precision	recall	support
AFIB	0.786199	0.789069	0.783403	875.0
GSVT	0.799788	0.789373	0.810549	691.0
SB	0.843057	0.833772	0.852557	1553.0
SR	0.798976	0.821048	0.778104	907.0
macro avg	0.807005	0.808315	0.806154	4026.0
micro avg	0.813544	0.813544	0.813544	4026.0
weighted avg	0.813343	0.813569	0.813544	4026.0

The result of Model Number:Dataset Number:Featuregroup Number:Rescale of 0 : 0 : 1 : 1

	f1-score	precision	recall	support
AFIB	0.724635	0.727933	0.721412	875.0
GSVT	0.728157	0.720899	0.735624	691.0
SB	0.767479	0.759115	0.776028	1553.0

SR 0.734010 0.751387 0.717422 907.0
macro avg 0.738570 0.739834 0.737622 4026.0
micro avg 0.744020 0.744020 0.744020 4026.0
weighted avg 0.743878 0.744038 0.744020 4026.0
The result of Model Number:Dataset Number:Featuregroup Number:Rescale of 0 : 0 : 2 : 0
f1-score precision recall support
AFIB 0.822272 0.824459 0.820146 875.0
GSVT 0.838084 0.832180 0.844122 691.0
SB 0.885954 0.876920 0.895192 1553.0
SR 0.845455 0.863871 0.827839 907.0
macro avg 0.847941 0.849357 0.846825 4026.0
micro avg 0.854943 0.854943 0.854943 4026.0
weighted avg 0.854773 0.854900 0.854943 4026.0
The result of Model Number:Dataset Number:Featuregroup Number:Rescale of 0 : 0 : 2 : 1
f1-score precision recall support
AFIB 0.765499 0.762514 0.768563 875.0
GSVT 0.779826 0.781470 0.778234 691.0
SB 0.827812 0.825748 0.829890 1553.0
SR 0.808715 0.814147 0.803373 907.0
macro avg 0.795463 0.795970 0.795015 4026.0
micro avg 0.801721 0.801721 0.801721 4026.0
weighted avg 0.801731 0.801792 0.801721 4026.0
The result of Model Number:Dataset Number:Featuregroup Number:Rescale of 0 : 0 : 3 : 0
f1-score precision recall support
AFIB 0.915545 0.945286 0.887792 204.0
GSVT 0.932901 0.924550 0.941589 300.0
SB 0.955084 0.948538 0.961723 892.0
SR 0.939508 0.943472 0.935632 681.0
macro avg 0.935759 0.940461 0.931684 2077.0
micro avg 0.942999 0.942999 0.942999 2077.0
weighted avg 0.942889 0.943092 0.942999 2077.0
The result of Model Number:Dataset Number:Featuregroup Number:Rescale of 0 : 0 : 3 : 1
f1-score precision recall support
AFIB 0.736293 0.745750 0.727110 875.0
GSVT 0.744438 0.738419 0.750677 691.0
SB 0.796336 0.793942 0.798747 1553.0
SR 0.776617 0.776291 0.776954 907.0
macro avg 0.763421 0.763600 0.763372 4026.0
micro avg 0.770018 0.770018 0.770018 4026.0
weighted avg 0.769937 0.769962 0.770018 4026.0
The result of Model Number:Dataset Number:Featuregroup Number:Rescale of 0 : 0 : 4 : 0
f1-score precision recall support
AFIB 0.796390 0.830882 0.764874 204.0

GSVT 0.744487 0.751434 0.738040 300.0
SB 0.771714 0.733773 0.813848 892.0
SR 0.687807 0.731213 0.649487 681.0
macro avg 0.750099 0.761825 0.741562 2077.0
micro avg 0.744198 0.744198 0.744198 2077.0
weighted avg 0.742694 0.745022 0.744198 2077.0
The result of Model Number:Dataset Number:Featuregroup Number:Rescale of 0 : 0 : 4 : 1
f1-score precision recall support
AFIB 0.766199 0.770107 0.762355 875.0
GSVT 0.776773 0.772732 0.780924 691.0
SB 0.814179 0.813452 0.814914 1553.0
SR 0.799187 0.799839 0.798570 907.0
macro avg 0.789085 0.789032 0.789191 4026.0
micro avg 0.793975 0.793975 0.793975 4026.0
weighted avg 0.793954 0.793976 0.793975 4026.0
The result of Model Number:Dataset Number:Featuregroup Number:Rescale of 0 : 0 : 5 : 0
f1-score precision recall support
AFIB 0.757935 0.764213 0.853711 204.0
GSVT 0.821930 0.786750 0.927329 300.0
SB 0.750453 0.747845 0.753081 892.0
SR 0.742991 0.743131 0.742855 681.0
macro avg 0.768327 0.760485 0.819244 2077.0
micro avg 0.784780 0.784780 0.784780 2077.0
weighted avg 0.759065 0.753526 0.784780 2077.0
The result of Model Number:Dataset Number:Featuregroup Number:Rescale of 0 : 0 : 5 : 1
f1-score precision recall support
AFIB 0.699173 0.737496 0.665102 204.0
GSVT 0.665761 0.689103 0.644031 300.0
SB 0.684801 0.658389 0.713432 892.0
SR 0.609492 0.626027 0.593842 681.0
macro avg 0.664807 0.677754 0.654102 2077.0
micro avg 0.659450 0.659450 0.659450 2077.0
weighted avg 0.658770 0.659984 0.659450 2077.0
The result of Model Number:Dataset Number:Featuregroup Number:Rescale of 0 : 0 : 6 : 0
f1-score precision recall support
AFIB 0.736412 0.737889 0.734974 875.0
GSVT 0.738236 0.734251 0.742285 691.0
SB 0.771705 0.767582 0.775877 1553.0
SR 0.753243 0.762175 0.744538 907.0
macro avg 0.749899 0.750474 0.749419 4026.0
micro avg 0.754162 0.754162 0.754162 4026.0
weighted avg 0.754131 0.754190 0.754162 4026.0
The result of Model Number:Dataset Number:Featuregroup Number:Rescale of 0 : 0 : 6 : 1

	f1-score	precision	recall	support
AFIB	0.811973	0.807427	0.816609	875.0
GSVT	0.818585	0.823414	0.813862	691.0
SB	0.862312	0.859613	0.865030	1553.0
SR	0.845241	0.850832	0.839741	907.0
macro avg	0.834528	0.835322	0.833811	4026.0
micro avg	0.840027	0.840027	0.840027	4026.0
weighted avg	0.840020	0.840080	0.840027	4026.0

The result of Model Number:Dataset Number:Featuregroup Number:Rescale of 0 : 0 : 7 : 0

	f1-score	precision	recall	support
AFIB	0.653119	0.656215	0.650056	875.0
GSVT	0.661221	0.658102	0.664385	691.0
SB	0.697434	0.695962	0.698916	1553.0
SR	0.684218	0.686133	0.682328	907.0
macro avg	0.673998	0.674103	0.673922	4026.0
micro avg	0.678633	0.678633	0.678633	4026.0
weighted avg	0.678610	0.678611	0.678633	4026.0

The result of Model Number:Dataset Number:Featuregroup Number:Rescale of 0 : 0 : 7 : 1

	f1-score	precision	recall	support
AFIB	0.781213	0.788418	0.774327	204.0
GSVT	0.809773	0.809802	0.809766	300.0
SB	0.826732	0.825264	0.828213	892.0
SR	0.817958	0.817739	0.818197	681.0
macro avg	0.808919	0.810306	0.807626	2077.0
micro avg	0.816972	0.816972	0.816972	2077.0
weighted avg	0.816935	0.816945	0.816972	2077.0

The result of Model Number:Dataset Number:Featuregroup Number:Rescale of 0 : 0 : 8 : 0

	f1-score	precision	recall	support
AFIB	0.753517	0.789593	0.720771	204.0
GSVT	0.793656	0.785160	0.802368	300.0
SB	0.810514	0.806037	0.815052	892.0
SR	0.801358	0.800663	0.802068	681.0
macro avg	0.789761	0.795363	0.785065	2077.0
micro avg	0.799703	0.799703	0.799703	2077.0
weighted avg	0.799479	0.799644	0.799703	2077.0

The result of Model Number:Dataset Number:Featuregroup Number:Rescale of 0 : 0 : 8 : 1

	f1-score	precision	recall	support
AFIB	0.665442	0.660112	0.670937	875.0
GSVT	0.683820	0.684987	0.682737	691.0
SB	0.724957	0.723014	0.726917	1553.0
SR	0.705149	0.713358	0.697143	907.0
macro avg	0.694842	0.695368	0.694433	4026.0
micro avg	0.700460	0.700460	0.700460	4026.0

weighted avg 0.700499 0.700641 0.700460 4026.0
The result of Model Number:Dataset Number:Featuregroup Number:Rescale of 0 : 0 : 9 : 0
f1-score precision recall support
AFIB 0.865130 0.869879 0.860489 875.0
GSVT 0.866340 0.855387 0.877713 691.0
SB 0.911107 0.907451 0.914800 1553.0
SR 0.891548 0.902371 0.881037 907.0
macro avg 0.883531 0.883772 0.883510 4026.0
micro avg 0.889025 0.889025 0.889025 4026.0
weighted avg 0.889025 0.889205 0.889025 4026.0
The result of Model Number:Dataset Number:Featuregroup Number:Rescale of 0 : 0 : 9 : 1
f1-score precision recall support
AFIB 0.720217 0.723787 0.716760 875.0
GSVT 0.727389 0.728436 0.726454 691.0
SB 0.765674 0.762122 0.769269 1553.0
SR 0.751580 0.753462 0.749748 907.0
macro avg 0.741215 0.741952 0.740558 4026.0
micro avg 0.746110 0.746110 0.746110 4026.0
weighted avg 0.746048 0.746058 0.746110 4026.0
The result of Model Number:Dataset Number:Featuregroup Number:Rescale of 0 : 0 : 10 :
0
f1-score precision recall support
AFIB 0.724372 0.725670 0.723102 875.0
GSVT 0.693635 0.697278 0.690048 691.0
SB 0.733697 0.693337 0.779054 1553.0
SR 0.609339 0.685856 0.548235 907.0
macro avg 0.690261 0.700535 0.685110 4026.0
micro avg 0.699617 0.699617 0.699617 4026.0
weighted avg 0.696778 0.699355 0.699617 4026.0
The result of Model Number:Dataset Number:Featuregroup Number:Rescale of 0 : 0 : 10 :
1
f1-score precision recall support
AFIB 0.717829 0.717957 0.717732 875.0
GSVT 0.725423 0.727874 0.723035 691.0
SB 0.762020 0.759392 0.764669 1553.0
SR 0.747550 0.750071 0.745073 907.0
macro avg 0.738205 0.738824 0.737627 4026.0
micro avg 0.742907 0.742907 0.742907 4026.0
weighted avg 0.742874 0.742877 0.742907 4026.0
The result of Model Number:Dataset Number:Featuregroup Number:Rescale of 0 : 1 : 0 : 0
f1-score precision recall support
AFIB 0.904420 0.911289 0.897731 204.0
GSVT 0.922936 0.913922 0.932150 300.0

	f1-score	precision	recall	support
SB	0.944465	0.942050	0.946900	892.0
SR	0.934446	0.939725	0.929238	681.0
macro avg	0.926567	0.926747	0.926505	2077.0
micro avg	0.934149	0.934149	0.934149	2077.0
weighted avg	0.934137	0.934204	0.934149	2077.0

The result of Model Number:Dataset Number:Featuregroup Number:Rescale of 0 : 1 : 0 : 1

	f1-score	precision	recall	support
AFIB	0.886567	0.896419	0.877385	204.0
GSVT	0.914030	0.908826	0.919558	300.0
SB	0.932213	0.930964	0.933467	892.0
SR	0.924283	0.925521	0.923060	681.0
macro avg	0.914273	0.915432	0.913367	2077.0
micro avg	0.922537	0.922537	0.922537	2077.0
weighted avg	0.922503	0.922589	0.922537	2077.0

The result of Model Number:Dataset Number:Featuregroup Number:Rescale of 0 : 1 : 1 : 0

	f1-score	precision	recall	support
AFIB	0.860379	0.868702	0.852370	204.0
GSVT	0.890824	0.889068	0.892602	300.0
SB	0.909444	0.907833	0.911072	892.0
SR	0.901060	0.901496	0.900662	681.0
macro avg	0.890427	0.891775	0.889177	2077.0
micro avg	0.899226	0.899226	0.899226	2077.0
weighted avg	0.899186	0.899201	0.899226	2077.0

The result of Model Number:Dataset Number:Featuregroup Number:Rescale of 0 : 1 : 1 : 1

	f1-score	precision	recall	support
AFIB	0.796687	0.799667	0.794068	875.0
GSVT	0.814620	0.822479	0.807073	691.0
SB	0.866196	0.857110	0.875542	1553.0
SR	0.836200	0.843560	0.829256	907.0
macro avg	0.828426	0.830704	0.826485	4026.0
micro avg	0.835655	0.835655	0.835655	4026.0
weighted avg	0.835479	0.835629	0.835655	4026.0

The result of Model Number:Dataset Number:Featuregroup Number:Rescale of 0 : 1 : 2 : 0

	f1-score	precision	recall	support
AFIB	0.808786	0.863607	0.761047	204.0
GSVT	0.762068	0.784678	0.741010	300.0
SB	0.796551	0.758313	0.839304	892.0
SR	0.706792	0.738881	0.678145	681.0
macro avg	0.768549	0.786370	0.754877	2077.0
micro avg	0.764580	0.764580	0.764580	2077.0
weighted avg	0.763342	0.766092	0.764580	2077.0

The result of Model Number:Dataset Number:Featuregroup Number:Rescale of 0 : 1 : 2 : 1

	f1-score	precision	recall	support

AFIB	0.823767	0.813250	0.834608	875.0
GSVT	0.838226	0.843033	0.833496	691.0
SB	0.897269	0.893501	0.901078	1553.0
SR	0.870849	0.884945	0.857211	907.0
macro avg	0.857528	0.858682	0.856598	4026.0
micro avg	0.865150	0.865150	0.865150	4026.0
weighted avg	0.865208	0.865470	0.865150	4026.0

The result of Model Number:Dataset Number:Featuregroup Number:Rescale of 0 : 1 : 3 : 0

	f1-score	precision	recall	support
AFIB	0.677350	0.696022	0.659789	204.0
GSVT	0.695376	0.682504	0.708810	300.0
SB	0.706654	0.700048	0.713388	892.0
SR	0.688892	0.698251	0.679792	681.0
macro avg	0.692068	0.694206	0.690445	2077.0
micro avg	0.696447	0.696447	0.696447	2077.0
weighted avg	0.696323	0.696529	0.696447	2077.0

The result of Model Number:Dataset Number:Featuregroup Number:Rescale of 0 : 1 : 3 : 1

	f1-score	precision	recall	support
AFIB	0.876728	0.893618	0.860878	204.0
GSVT	0.893191	0.878678	0.908354	300.0
SB	0.908103	0.897273	0.919200	892.0
SR	0.891210	0.907919	0.875120	681.0
macro avg	0.892308	0.894372	0.890888	2077.0
micro avg	0.897452	0.897452	0.897452	2077.0
weighted avg	0.897329	0.897719	0.897452	2077.0

The result of Model Number:Dataset Number:Featuregroup Number:Rescale of 0 : 1 : 4 : 0

	f1-score	precision	recall	support
AFIB	0.694944	0.721949	0.669937	875.0
GSVT	0.747087	0.741570	0.752786	691.0
SB	0.820991	0.816762	0.825271	1553.0
SR	0.772628	0.757894	0.788028	907.0
macro avg	0.758913	0.759544	0.759006	4026.0
micro avg	0.770680	0.770680	0.770680	4026.0
weighted avg	0.770016	0.769988	0.770680	4026.0

The result of Model Number:Dataset Number:Featuregroup Number:Rescale of 0 : 1 : 4 : 1

	f1-score	precision	recall	support
AFIB	0.874717	0.864208	0.885517	875.0
GSVT	0.891541	0.898759	0.884443	691.0
SB	0.950313	0.946676	0.953983	1553.0
SR	0.928032	0.939898	0.916473	907.0
macro avg	0.911151	0.912385	0.910104	4026.0
micro avg	0.918717	0.918717	0.918717	4026.0
weighted avg	0.918776	0.919001	0.918717	4026.0

The result of Model Number:Dataset Number:Featuregroup Number:Rescale of 0 : 1 : 5 : 0

	f1-score	precision	recall	support
AFIB	0.762166	0.763639	0.760757	875.0
GSVT	0.738411	0.735167	0.741727	691.0
SB	0.771551	0.724656	0.824957	1553.0
SR	0.620991	0.719547	0.546328	907.0
macro avg	0.723280	0.735753	0.718442	4026.0
micro avg	0.733948	0.733948	0.733948	4026.0
weighted avg	0.729904	0.733782	0.733948	4026.0

The result of Model Number:Dataset Number:Featuregroup Number:Rescale of 0 : 1 : 5 : 1

	f1-score	precision	recall	support
AFIB	0.633601	0.629611	0.713708	204.0
GSVT	0.772370	0.768397	0.776514	300.0
SB	0.794362	0.790582	0.798187	892.0
SR	0.623087	0.625140	0.621424	681.0
macro avg	0.705855	0.703433	0.727458	2077.0
micro avg	0.728802	0.728802	0.728802	2077.0
weighted avg	0.719239	0.717323	0.728802	2077.0

The result of Model Number:Dataset Number:Featuregroup Number:Rescale of 0 : 1 : 6 : 0

	f1-score	precision	recall	support
AFIB	0.745517	0.766068	0.726188	204.0
GSVT	0.765357	0.751189	0.780143	300.0
SB	0.777770	0.770499	0.785181	892.0
SR	0.758220	0.768521	0.748205	681.0
macro avg	0.761716	0.764069	0.759929	2077.0
micro avg	0.766535	0.766535	0.766535	2077.0
weighted avg	0.766399	0.766626	0.766535	2077.0

The result of Model Number:Dataset Number:Featuregroup Number:Rescale of 0 : 1 : 6 : 1

	f1-score	precision	recall	support
AFIB	0.615463	0.609429	0.658053	204.0
GSVT	0.551857	0.545721	0.558270	300.0
SB	0.709171	0.704007	0.714416	892.0
SR	0.697277	0.702107	0.692571	681.0
macro avg	0.643442	0.640316	0.655827	2077.0
micro avg	0.679164	0.679164	0.679164	2077.0
weighted avg	0.673345	0.671232	0.679164	2077.0

The result of Model Number:Dataset Number:Featuregroup Number:Rescale of 0 : 1 : 7 : 0

	f1-score	precision	recall	support
AFIB	0.876948	0.864507	0.889774	875.0
GSVT	0.894990	0.903713	0.886440	691.0
SB	0.952444	0.949340	0.955570	1553.0
SR	0.930201	0.942090	0.918619	907.0
macro avg	0.913646	0.914912	0.912601	4026.0

micro avg	0.921080	0.921080	0.921080	4026.0
weighted avg	0.921164	0.921438	0.921080	4026.0

The result of Model Number:Dataset Number:Featuregroup Number:Rescale of 0 : 1 : 7 : 1

	f1-score	precision	recall	support
AFIB	0.768573	0.802732	0.737309	204.0
GSVT	0.800601	0.794316	0.807004	300.0
SB	0.812830	0.806560	0.819206	892.0
SR	0.803838	0.805165	0.802543	681.0
macro avg	0.796460	0.802193	0.791516	2077.0
micro avg	0.803937	0.803937	0.803937	2077.0
weighted avg	0.803769	0.803958	0.803937	2077.0

The result of Model Number:Dataset Number:Featuregroup Number:Rescale of 0 : 1 : 8 : 0

	f1-score	precision	recall	support
AFIB	0.717115	0.714199	0.720074	875.0
GSVT	0.727597	0.731996	0.723280	691.0
SB	0.767388	0.766454	0.768327	1553.0
SR	0.751379	0.752570	0.750215	907.0
macro avg	0.740870	0.741305	0.740474	4026.0
micro avg	0.746028	0.746028	0.746028	4026.0
weighted avg	0.746026	0.746055	0.746028	4026.0

The result of Model Number:Dataset Number:Featuregroup Number:Rescale of 0 : 1 : 8 : 1

	f1-score	precision	recall	support
AFIB	0.754461	0.831088	0.691496	204.0
GSVT	0.696741	0.744921	0.654740	300.0
SB	0.758544	0.708763	0.815857	892.0
SR	0.656532	0.691043	0.625484	681.0
macro avg	0.716570	0.743954	0.696894	2077.0
micro avg	0.717952	0.717952	0.717952	2077.0
weighted avg	0.715769	0.720190	0.717952	2077.0

The result of Model Number:Dataset Number:Featuregroup Number:Rescale of 0 : 1 : 9 : 0

	f1-score	precision	recall	support
AFIB	0.747587	0.761307	0.734712	204.0
GSVT	0.775966	0.769727	0.782449	300.0
SB	0.790361	0.788950	0.791778	892.0
SR	0.782535	0.783231	0.781844	681.0
macro avg	0.774112	0.775804	0.772696	2077.0
micro avg	0.781569	0.781569	0.781569	2077.0
weighted avg	0.781514	0.781583	0.781569	2077.0

The result of Model Number:Dataset Number:Featuregroup Number:Rescale of 0 : 1 : 9 : 1

	f1-score	precision	recall	support
AFIB	0.641515	0.633325	0.649958	875.0
GSVT	0.652776	0.656519	0.649092	691.0
SB	0.698756	0.695821	0.701722	1553.0

SR 0.678181 0.689158 0.667561 907.0
macro avg 0.667807 0.668706 0.667083 4026.0
micro avg 0.673743 0.673743 0.673743 4026.0
weighted avg 0.673788 0.673992 0.673743 4026.0
The result of Model Number:Dataset Number:Featuregroup Number:Rescale of 0 : 1 : 10 : 0

	f1-score	precision	recall	support
AFIB	0.730661	0.738131	0.723401	875.0
GSVT	0.730441	0.719156	0.742171	691.0
SB	0.770518	0.765917	0.775181	1553.0
SR	0.750123	0.760163	0.740367	907.0
macro avg	0.745436	0.745842	0.745280	4026.0
micro avg	0.750419	0.750419	0.750419	4026.0
weighted avg	0.750382	0.750556	0.750419	4026.0

The result of Model Number:Dataset Number:Featuregroup Number:Rescale of 0 : 1 : 10 : 1

	f1-score	precision	recall	support
AFIB	0.806582	0.816013	0.797522	204.0
GSVT	0.828771	0.824960	0.832640	300.0
SB	0.840505	0.837891	0.843141	892.0
SR	0.833266	0.835628	0.830954	681.0
macro avg	0.827281	0.828623	0.826064	2077.0
micro avg	0.833148	0.833148	0.833148	2077.0
weighted avg	0.833105	0.833133	0.833148	2077.0

The result of Model Number:Dataset Number:Featuregroup Number:Rescale of 1 : 0 : 0 : 0

	f1-score	precision	recall	support
AFIB	0.766628	0.766582	0.766723	875.0
GSVT	0.768288	0.764134	0.772500	691.0
SB	0.804992	0.799635	0.810431	1553.0
SR	0.783616	0.796473	0.771197	907.0
macro avg	0.780881	0.781706	0.780212	4026.0
micro avg	0.785582	0.785582	0.785582	4026.0
weighted avg	0.785539	0.785646	0.785582	4026.0

The result of Model Number:Dataset Number:Featuregroup Number:Rescale of 1 : 0 : 0 : 1

	f1-score	precision	recall	support
AFIB	0.740311	0.731417	0.749451	875.0
GSVT	0.754550	0.760659	0.748543	691.0
SB	0.804292	0.801213	0.807397	1553.0
SR	0.785434	0.795477	0.775651	907.0
macro avg	0.771147	0.772191	0.770261	4026.0
micro avg	0.777550	0.777550	0.777550	4026.0
weighted avg	0.777600	0.777791	0.777550	4026.0

The result of Model Number:Dataset Number:Featuregroup Number:Rescale of 1 : 0 : 1 : 0

	f1-score	precision	recall	support
AFIB	0.491597	0.535519	0.519137	204.0
GSVT	0.667725	0.656810	0.679240	300.0
SB	0.568441	0.564111	0.572880	892.0
SR	0.695110	0.687245	0.703178	681.0
macro avg	0.605718	0.610921	0.618609	2077.0
micro avg	0.625685	0.625685	0.625685	2077.0
weighted avg	0.616766	0.615065	0.625685	2077.0

The result of Model Number:Dataset Number:Featuregroup Number:Rescale of 1 : 0 : 1 : 1

	f1-score	precision	recall	support
AFIB	0.740280	0.760687	0.721087	204.0
GSVT	0.759981	0.745913	0.774663	300.0
SB	0.772306	0.765086	0.779666	892.0
SR	0.752894	0.763123	0.742949	681.0
macro avg	0.756365	0.758702	0.754591	2077.0
micro avg	0.761151	0.761151	0.761151	2077.0
weighted avg	0.761016	0.761241	0.761151	2077.0

The result of Model Number:Dataset Number:Featuregroup Number:Rescale of 1 : 0 : 2 : 0

	f1-score	precision	recall	support
AFIB	0.709568	0.693444	0.726510	875.0
GSVT	0.733886	0.745787	0.722419	691.0
SB	0.782976	0.782981	0.782976	1553.0
SR	0.762937	0.771628	0.754440	907.0
macro avg	0.747342	0.748460	0.746586	4026.0
micro avg	0.753882	0.753882	0.753882	4026.0
weighted avg	0.754082	0.754580	0.753882	4026.0

The result of Model Number:Dataset Number:Featuregroup Number:Rescale of 1 : 0 : 2 : 1

	f1-score	precision	recall	support
AFIB	0.855194	0.913585	0.804183	204.0
GSVT	0.904025	0.885603	0.923308	300.0
SB	0.926448	0.918325	0.934749	892.0
SR	0.903595	0.906604	0.900686	681.0
macro avg	0.897315	0.906029	0.890732	2077.0
micro avg	0.909104	0.909104	0.909104	2077.0
weighted avg	0.908718	0.909290	0.909104	2077.0

The result of Model Number:Dataset Number:Featuregroup Number:Rescale of 1 : 0 : 3 : 0

	f1-score	precision	recall	support
AFIB	0.851745	0.854489	0.849098	875.0
GSVT	0.863720	0.850007	0.877960	691.0
SB	0.910069	0.902470	0.917803	1553.0
SR	0.864323	0.886274	0.843464	907.0
macro avg	0.872464	0.873310	0.872081	4026.0
micro avg	0.879285	0.879285	0.879285	4026.0

weighted avg	0.879132	0.879389	0.879285	4026.0

The result of Model Number:Dataset Number:Featuregroup Number:Rescale of 1 : 0 : 3 : 1

	f1-score	precision	recall	support
AFIB	0.652554	0.651825	0.653304	875.0
GSVT	0.664490	0.656179	0.673034	691.0
SB	0.698043	0.692431	0.703752	1553.0
SR	0.666900	0.684398	0.650285	907.0
macro avg	0.670497	0.671208	0.670094	4026.0
micro avg	0.675470	0.675470	0.675470	4026.0
weighted avg	0.675382	0.675574	0.675470	4026.0

The result of Model Number:Dataset Number:Featuregroup Number:Rescale of 1 : 0 : 4 : 0

	f1-score	precision	recall	support
AFIB	0.762221	0.768263	0.756293	875.0
GSVT	0.771885	0.768482	0.775344	691.0
SB	0.820808	0.817564	0.824085	1553.0
SR	0.800638	0.802901	0.798428	907.0
macro avg	0.788888	0.789302	0.788537	4026.0
micro avg	0.795205	0.795205	0.795205	4026.0
weighted avg	0.795134	0.795121	0.795205	4026.0

The result of Model Number:Dataset Number:Featuregroup Number:Rescale of 1 : 0 : 4 : 1

	f1-score	precision	recall	support
AFIB	0.757406	0.748431	0.766663	875.0
GSVT	0.768187	0.775613	0.760976	691.0
SB	0.815302	0.812183	0.818452	1553.0
SR	0.796294	0.805481	0.787337	907.0
macro avg	0.784297	0.785427	0.783357	4026.0
micro avg	0.790322	0.790322	0.790322	4026.0
weighted avg	0.790350	0.790541	0.790322	4026.0

The result of Model Number:Dataset Number:Featuregroup Number:Rescale of 1 : 0 : 5 : 0

	f1-score	precision	recall	support
AFIB	0.890368	0.877736	0.903390	875.0
GSVT	0.908686	0.917542	0.900005	691.0
SB	0.967019	0.963867	0.970193	1553.0
SR	0.944436	0.956507	0.932676	907.0
macro avg	0.927627	0.928913	0.926566	4026.0
micro avg	0.935175	0.935175	0.935175	4026.0
weighted avg	0.935260	0.935539	0.935175	4026.0

The result of Model Number:Dataset Number:Featuregroup Number:Rescale of 1 : 0 : 5 : 1

	f1-score	precision	recall	support
AFIB	0.717773	0.734677	0.701691	875.0
GSVT	0.756639	0.758550	0.754788	691.0
SB	0.822859	0.819448	0.826302	1553.0
SR	0.782955	0.770625	0.795724	907.0

macro avg	0.770057	0.770825	0.769626	4026.0
micro avg	0.780056	0.780056	0.780056	4026.0
weighted avg	0.779665	0.779573	0.780056	4026.0

The result of Model Number:Dataset Number:Featuregroup Number:Rescale of 1 : 0 : 6 : 0

	f1-score	precision	recall	support
AFIB	0.740109	0.739873	0.740385	875.0
GSVT	0.751139	0.741484	0.761137	691.0
SB	0.794510	0.792782	0.796249	1553.0
SR	0.772527	0.783852	0.761538	907.0
macro avg	0.764571	0.764497	0.764827	4026.0
micro avg	0.770261	0.770261	0.770261	4026.0
weighted avg	0.770290	0.770466	0.770261	4026.0

The result of Model Number:Dataset Number:Featuregroup Number:Rescale of 1 : 0 : 6 : 1

	f1-score	precision	recall	support
AFIB	0.689385	0.687393	0.691453	875.0
GSVT	0.702163	0.701591	0.702783	691.0
SB	0.745799	0.742710	0.748919	1553.0
SR	0.723409	0.731356	0.715671	907.0
macro avg	0.715189	0.715762	0.714707	4026.0
micro avg	0.721021	0.721021	0.721021	4026.0
weighted avg	0.721004	0.721072	0.721021	4026.0

The result of Model Number:Dataset Number:Featuregroup Number:Rescale of 1 : 0 : 7 : 0

	f1-score	precision	recall	support
AFIB	0.557952	0.518853	0.655209	875.0
GSVT	0.478516	0.446761	0.541249	691.0
SB	0.571796	0.566971	0.576716	1553.0
SR	0.551128	0.564487	0.538579	907.0
macro avg	0.539848	0.524268	0.577938	4026.0
micro avg	0.579096	0.579096	0.579096	4026.0
weighted avg	0.548121	0.535322	0.579096	4026.0

The result of Model Number:Dataset Number:Featuregroup Number:Rescale of 1 : 0 : 7 : 1

	f1-score	precision	recall	support
AFIB	0.734596	0.755269	0.715175	204.0
GSVT	0.753588	0.748298	0.759095	300.0
SB	0.774311	0.771213	0.777436	892.0
SR	0.764019	0.764497	0.763565	681.0
macro avg	0.756628	0.759819	0.753818	2077.0
micro avg	0.764123	0.764123	0.764123	2077.0
weighted avg	0.764043	0.764135	0.764123	2077.0

The result of Model Number:Dataset Number:Featuregroup Number:Rescale of 1 : 0 : 8 : 0

	f1-score	precision	recall	support
AFIB	0.705692	0.727427	0.685360	204.0
GSVT	0.723696	0.714836	0.732856	300.0

SB	0.743352	0.738935	0.747847	892.0
SR	0.730822	0.734427	0.727282	681.0
macro avg	0.725891	0.728907	0.723336	2077.0
micro avg	0.732801	0.732801	0.732801	2077.0
weighted avg	0.732706	0.732846	0.732801	2077.0

The result of Model Number:Dataset Number:Featuregroup Number:Rescale of 1 : 0 : 8 : 1

	f1-score	precision	recall	support
AFIB	0.608351	0.599191	0.622861	875.0
GSVT	0.580865	0.579175	0.585820	691.0
SB	0.620289	0.578011	0.674399	1553.0
SR	0.459487	0.614198	0.380740	907.0
macro avg	0.567248	0.592643	0.565955	4026.0
micro avg	0.581838	0.581838	0.581838	4026.0
weighted avg	0.574702	0.590966	0.581838	4026.0

The result of Model Number:Dataset Number:Featuregroup Number:Rescale of 1 : 0 : 9 : 0

	f1-score	precision	recall	support
AFIB	0.788350	0.798880	0.778316	204.0
GSVT	0.802277	0.797389	0.807347	300.0
SB	0.821616	0.820887	0.822351	892.0
SR	0.815545	0.815668	0.815426	681.0
macro avg	0.806947	0.808206	0.805860	2077.0
micro avg	0.813588	0.813588	0.813588	2077.0
weighted avg	0.813564	0.813621	0.813588	2077.0

The result of Model Number:Dataset Number:Featuregroup Number:Rescale of 1 : 0 : 9 : 1

	f1-score	precision	recall	support
AFIB	0.685935	0.707531	0.665786	204.0
GSVT	0.651675	0.665594	0.638434	300.0
SB	0.672868	0.649323	0.698210	892.0
SR	0.614499	0.635267	0.595090	681.0
macro avg	0.656244	0.664429	0.649380	2077.0
micro avg	0.652581	0.652581	0.652581	2077.0
weighted avg	0.651953	0.652782	0.652581	2077.0

The result of Model Number:Dataset Number:Featuregroup Number:Rescale of 1 : 0 : 10 : 0

	f1-score	precision	recall	support
AFIB	0.725040	0.762497	0.691305	204.0
GSVT	0.760266	0.752834	0.767862	300.0
SB	0.773671	0.767447	0.780001	892.0
SR	0.764115	0.765045	0.763218	681.0
macro avg	0.755773	0.761956	0.750597	2077.0
micro avg	0.764033	0.764033	0.764033	2077.0
weighted avg	0.763825	0.764063	0.764033	2077.0

The result of Model Number:Dataset Number:Featuregroup Number:Rescale of 1 : 0 : 10 : 1

	f1-score	precision	recall	support
AFIB	0.702520	0.691749	0.713750	875.0
GSVT	0.726744	0.733868	0.719901	691.0
SB	0.773450	0.771643	0.775290	1553.0
SR	0.754129	0.763691	0.744820	907.0
macro avg	0.739211	0.740238	0.738440	4026.0
micro avg	0.745544	0.745544	0.745544	4026.0
weighted avg	0.745665	0.746004	0.745544	4026.0

The result of Model Number:Dataset Number:Featuregroup Number:Rescale of 1 : 1 : 0 : 0

	f1-score	precision	recall	support
AFIB	0.656168	0.650016	0.662520	875.0
GSVT	0.622110	0.607157	0.667842	691.0
SB	0.719354	0.716712	0.722035	1553.0
SR	0.559500	0.563424	0.555639	907.0
macro avg	0.639283	0.634327	0.652009	4026.0
micro avg	0.662312	0.662312	0.662312	4026.0
weighted avg	0.652918	0.648880	0.662312	4026.0

The result of Model Number:Dataset Number:Featuregroup Number:Rescale of 1 : 1 : 0 : 1

	f1-score	precision	recall	support
AFIB	0.863396	0.925802	0.809466	204.0
GSVT	0.817815	0.822239	0.813773	300.0
SB	0.854224	0.803002	0.912446	892.0
SR	0.750073	0.809497	0.698872	681.0
macro avg	0.821377	0.840135	0.808639	2077.0
micro avg	0.818053	0.818053	0.818053	2077.0
weighted avg	0.815717	0.819971	0.818053	2077.0

The result of Model Number:Dataset Number:Featuregroup Number:Rescale of 1 : 1 : 1 : 0

	f1-score	precision	recall	support
AFIB	0.680768	0.687728	0.674004	875.0
GSVT	0.680563	0.670049	0.691492	691.0
SB	0.717903	0.713616	0.722248	1553.0
SR	0.698901	0.708256	0.689812	907.0
macro avg	0.694534	0.694912	0.694389	4026.0
micro avg	0.699176	0.699176	0.699176	4026.0
weighted avg	0.699143	0.699305	0.699176	4026.0

The result of Model Number:Dataset Number:Featuregroup Number:Rescale of 1 : 1 : 1 : 1

	f1-score	precision	recall	support
AFIB	0.710041	0.699428	0.721007	875.0
GSVT	0.723654	0.729843	0.717581	691.0
SB	0.773208	0.770546	0.775894	1553.0
SR	0.750577	0.761771	0.739732	907.0

macro avg	0.739370	0.740397	0.738553	4026.0
micro avg	0.745810	0.745810	0.745810	4026.0
weighted avg	0.745876	0.746126	0.745810	4026.0

The result of Model Number:Dataset Number:Featuregroup Number:Rescale of 1 : 1 : 2 : 0

	f1-score	precision	recall	support
AFIB	0.798865	0.824816	0.774649	204.0
GSVT	0.814010	0.806723	0.821591	300.0
SB	0.833366	0.827654	0.839158	892.0
SR	0.819775	0.823233	0.816393	681.0
macro avg	0.816504	0.820607	0.812948	2077.0
micro avg	0.822821	0.822821	0.822821	2077.0
weighted avg	0.822725	0.822903	0.822821	2077.0

The result of Model Number:Dataset Number:Featuregroup Number:Rescale of 1 : 1 : 2 : 1

	f1-score	precision	recall	support
AFIB	0.820245	0.803905	0.837305	875.0
GSVT	0.667607	0.620839	0.836826	691.0
SB	0.541689	0.542622	0.540759	1553.0
SR	0.878147	0.885880	0.870585	907.0
macro avg	0.726922	0.713311	0.771369	4026.0
micro avg	0.730330	0.730330	0.730330	4026.0
weighted avg	0.699641	0.690164	0.730330	4026.0

The result of Model Number:Dataset Number:Featuregroup Number:Rescale of 1 : 1 : 3 : 0

	f1-score	precision	recall	support
AFIB	0.683574	0.708917	0.660077	204.0
GSVT	0.704053	0.695250	0.713171	300.0
SB	0.721906	0.715319	0.728622	892.0
SR	0.702927	0.708382	0.697562	681.0
macro avg	0.703115	0.706967	0.699858	2077.0
micro avg	0.709474	0.709474	0.709474	2077.0
weighted avg	0.709340	0.709517	0.709474	2077.0

The result of Model Number:Dataset Number:Featuregroup Number:Rescale of 1 : 1 : 3 : 1

	f1-score	precision	recall	support
AFIB	0.899933	0.908137	0.891994	204.0
GSVT	0.923419	0.920395	0.926491	300.0
SB	0.940299	0.939672	0.940930	892.0
SR	0.932030	0.931762	0.932311	681.0
macro avg	0.923920	0.924992	0.922932	2077.0
micro avg	0.931212	0.931212	0.931212	2077.0
weighted avg	0.931185	0.931197	0.931212	2077.0

The result of Model Number:Dataset Number:Featuregroup Number:Rescale of 1 : 1 : 4 : 0

	f1-score	precision	recall	support
AFIB	0.826507	0.835317	0.818016	204.0
GSVT	0.841707	0.837089	0.846469	300.0

SB 0.861136 0.859701 0.862582 892.0
SR 0.853958 0.855349 0.852582 681.0
macro avg 0.845827 0.846864 0.844912 2077.0
micro avg 0.852598 0.852598 0.852598 2077.0
weighted avg 0.852575 0.852613 0.852598 2077.0
The result of Model Number:Dataset Number:Featuregroup Number:Rescale of 1 : 1 : 4 : 1
f1-score precision recall support
AFIB 0.772367 0.790013 0.755689 204.0
GSVT 0.807455 0.802429 0.812565 300.0
SB 0.824894 0.822335 0.827480 892.0
SR 0.815447 0.815824 0.815092 681.0
macro avg 0.805041 0.807650 0.802706 2077.0
micro avg 0.814212 0.814212 0.814212 2077.0
weighted avg 0.814118 0.814150 0.814212 2077.0
The result of Model Number:Dataset Number:Featuregroup Number:Rescale of 1 : 1 : 5 : 0
f1-score precision recall support
AFIB 0.663139 0.644034 0.727592 875.0
GSVT 0.730869 0.728534 0.733312 691.0
SB 0.616961 0.607869 0.626379 1553.0
SR 0.738737 0.753156 0.724952 907.0
macro avg 0.687427 0.683398 0.703059 4026.0
micro avg 0.688937 0.688937 0.688937 4026.0
weighted avg 0.673982 0.669170 0.688937 4026.0
The result of Model Number:Dataset Number:Featuregroup Number:Rescale of 1 : 1 : 5 : 1
f1-score precision recall support
AFIB 0.659641 0.655183 0.664250 875.0
GSVT 0.675734 0.676003 0.675552 691.0
SB 0.717830 0.715998 0.719678 1553.0
SR 0.698284 0.706086 0.690662 907.0
macro avg 0.687872 0.688317 0.687536 4026.0
micro avg 0.693521 0.693521 0.693521 4026.0
weighted avg 0.693555 0.693683 0.693521 4026.0
The result of Model Number:Dataset Number:Featuregroup Number:Rescale of 1 : 1 : 6 : 0
f1-score precision recall support
AFIB 0.769671 0.768426 0.770989 875.0
GSVT 0.776723 0.779812 0.773698 691.0
SB 0.815695 0.812730 0.818688 1553.0
SR 0.802353 0.806412 0.798379 907.0
macro avg 0.791111 0.791845 0.790438 4026.0
micro avg 0.796024 0.796024 0.796024 4026.0
weighted avg 0.795998 0.796028 0.796024 4026.0
The result of Model Number:Dataset Number:Featuregroup Number:Rescale of 1 : 1 : 6 : 1
f1-score precision recall support

AFIB	0.760388	0.760342	0.760481	875.0
GSVT	0.762034	0.757914	0.766212	691.0
SB	0.798439	0.793126	0.803833	1553.0
SR	0.777237	0.789990	0.764919	907.0
macro avg	0.774524	0.775343	0.773861	4026.0
micro avg	0.779187	0.779187	0.779187	4026.0
weighted avg	0.779144	0.779251	0.779187	4026.0

The result of Model Number:Dataset Number:Featuregroup Number:Rescale of 1 : 1 : 7 : 0

	f1-score	precision	recall	support
AFIB	0.874779	0.914280	0.838835	204.0
GSVT	0.905204	0.895038	0.915769	300.0
SB	0.870773	0.832809	0.931740	892.0
SR	0.725473	0.731659	0.719515	681.0
macro avg	0.844057	0.843447	0.851464	2077.0
micro avg	0.850724	0.850724	0.850724	2077.0
weighted avg	0.828499	0.816635	0.850724	2077.0

The result of Model Number:Dataset Number:Featuregroup Number:Rescale of 1 : 1 : 7 : 1

	f1-score	precision	recall	support
AFIB	0.856632	0.855246	0.858099	875.0
GSVT	0.864481	0.867919	0.861114	691.0
SB	0.907856	0.904556	0.911187	1553.0
SR	0.893006	0.897524	0.888583	907.0
macro avg	0.880494	0.881311	0.879746	4026.0
micro avg	0.885962	0.885962	0.885962	4026.0
weighted avg	0.885933	0.885966	0.885962	4026.0

The result of Model Number:Dataset Number:Featuregroup Number:Rescale of 1 : 1 : 8 : 0

	f1-score	precision	recall	support
AFIB	0.852160	0.910345	0.801330	204.0
GSVT	0.900818	0.882462	0.920034	300.0
SB	0.923162	0.915068	0.931434	892.0
SR	0.900390	0.903389	0.897492	681.0
macro avg	0.894133	0.902816	0.887572	2077.0
micro avg	0.905880	0.905880	0.905880	2077.0
weighted avg	0.905495	0.906065	0.905880	2077.0

The result of Model Number:Dataset Number:Featuregroup Number:Rescale of 1 : 1 : 8 : 1

	f1-score	precision	recall	support
AFIB	0.915973	0.929860	0.902517	204.0
GSVT	0.930701	0.922855	0.938730	300.0
SB	0.951346	0.948277	0.954437	892.0
SR	0.941133	0.944629	0.937689	681.0
macro avg	0.934788	0.936405	0.933343	2077.0
micro avg	0.941578	0.941578	0.941578	2077.0
weighted avg	0.941541	0.941600	0.941578	2077.0

The result of Model Number:Dataset Number:Featuregroup Number:Rescale of 1 : 1 : 9 : 0

	f1-score	precision	recall	support
AFIB	0.812290	0.808222	0.816666	875.0
GSVT	0.830643	0.831766	0.829690	691.0
SB	0.884521	0.881608	0.887477	1553.0
SR	0.858545	0.867455	0.849917	907.0
macro avg	0.846500	0.847263	0.845937	4026.0
micro avg	0.853707	0.853707	0.853707	4026.0
weighted avg	0.853723	0.853916	0.853707	4026.0

The result of Model Number:Dataset Number:Featuregroup Number:Rescale of 1 : 1 : 9 : 1

	f1-score	precision	recall	support
AFIB	0.806559	0.812875	0.800382	875.0
GSVT	0.808686	0.798365	0.819327	691.0
SB	0.848220	0.844818	0.851659	1553.0
SR	0.830300	0.838450	0.822337	907.0
macro avg	0.823441	0.823627	0.823426	4026.0
micro avg	0.828359	0.828359	0.828359	4026.0
weighted avg	0.828343	0.828468	0.828359	4026.0

The result of Model Number:Dataset Number:Featuregroup Number:Rescale of 1 : 1 : 10 : 0

	f1-score	precision	recall	support
AFIB	0.848384	0.847011	0.849836	875.0
GSVT	0.856157	0.859562	0.852823	691.0
SB	0.899114	0.895846	0.902413	1553.0
SR	0.884407	0.888882	0.880027	907.0
macro avg	0.872016	0.872825	0.871275	4026.0
micro avg	0.877432	0.877432	0.877432	4026.0
weighted avg	0.877402	0.877436	0.877432	4026.0

The result of Model Number:Dataset Number:Featuregroup Number:Rescale of 1 : 1 : 10 : 1

	f1-score	precision	recall	support
AFIB	0.919340	0.927686	0.911614	204.0
GSVT	0.949725	0.943985	0.955783	300.0
SB	0.969594	0.968294	0.970898	892.0
SR	0.960622	0.962614	0.958645	681.0
macro avg	0.949820	0.950645	0.949235	2077.0
micro avg	0.958875	0.958875	0.958875	2077.0
weighted avg	0.958846	0.958932	0.958875	2077.0

The result of Model Number:Dataset Number:Featuregroup Number:Rescale of 2 : 0 : 0 : 0

	f1-score	precision	recall	support
AFIB	0.838020	0.834753	0.841374	875.0
GSVT	0.853704	0.855504	0.851962	691.0
SB	0.906236	0.903977	0.908511	1553.0

SR 0.885331 0.891277 0.879482 907.0
macro avg 0.870823 0.871378 0.870332 4026.0
micro avg 0.877674 0.877674 0.877674 4026.0
weighted avg 0.877684 0.877751 0.877674 4026.0
The result of Model Number:Dataset Number:Featuregroup Number:Rescale of 2 : 0 : 0 : 1
f1-score precision recall support
AFIB 0.802323 0.806532 0.798206 875.0
GSVT 0.819099 0.817628 0.820635 691.0
SB 0.889402 0.882581 0.896331 1553.0
SR 0.858023 0.866611 0.849608 907.0
macro avg 0.842212 0.843338 0.841195 4026.0
micro avg 0.851487 0.851487 0.851487 4026.0
weighted avg 0.851341 0.851307 0.851487 4026.0
The result of Model Number:Dataset Number:Featuregroup Number:Rescale of 2 : 0 : 1 : 0
f1-score precision recall support
AFIB 0.795311 0.787940 0.802866 875.0
GSVT 0.809594 0.813991 0.805258 691.0
SB 0.857811 0.855834 0.859799 1553.0
SR 0.837825 0.845557 0.830260 907.0
macro avg 0.825135 0.825830 0.824546 4026.0
micro avg 0.831409 0.831409 0.831409 4026.0
weighted avg 0.831449 0.831581 0.831409 4026.0
The result of Model Number:Dataset Number:Featuregroup Number:Rescale of 2 : 0 : 1 : 1
f1-score precision recall support
AFIB 0.791446 0.826621 0.759252 204.0
GSVT 0.824427 0.817955 0.831021 300.0
SB 0.837021 0.830564 0.843586 892.0
SR 0.827760 0.829127 0.826427 681.0
macro avg 0.820163 0.826067 0.815072 2077.0
micro avg 0.827862 0.827862 0.827862 2077.0
weighted avg 0.827689 0.827884 0.827862 2077.0
The result of Model Number:Dataset Number:Featuregroup Number:Rescale of 2 : 0 : 2 : 0
f1-score precision recall support
AFIB 0.899976 0.927695 0.874045 204.0
GSVT 0.922935 0.911637 0.934618 300.0
SB 0.948004 0.942371 0.953736 892.0
SR 0.932024 0.936621 0.927509 681.0
macro avg 0.925735 0.929581 0.922477 2077.0
micro avg 0.934548 0.934548 0.934548 2077.0
weighted avg 0.934426 0.934605 0.934548 2077.0
The result of Model Number:Dataset Number:Featuregroup Number:Rescale of 2 : 0 : 2 : 1
f1-score precision recall support
AFIB 0.716157 0.730459 0.702514 204.0

GSVT 0.724200 0.715151 0.733590 300.0
SB 0.738026 0.730969 0.745222 892.0
SR 0.724623 0.734129 0.715380 681.0
macro avg 0.725752 0.727677 0.724177 2077.0
micro avg 0.729563 0.729563 0.729563 2077.0
weighted avg 0.729487 0.729670 0.729563 2077.0

The result of Model Number:Dataset Number:Featuregroup Number:Rescale of 2 : 0 : 3 : 0

	f1-score	precision	recall	support
AFIB	0.798023	0.818575	0.778510	204.0
GSVT	0.732326	0.699727	0.826429	300.0
SB	0.840189	0.838223	0.842166	892.0
SR	0.666538	0.666663	0.666421	681.0
macro avg	0.759269	0.755797	0.778381	2077.0
micro avg	0.776018	0.776018	0.776018	2077.0
weighted avg	0.763531	0.760038	0.776018	2077.0

The result of Model Number:Dataset Number:Featuregroup Number:Rescale of 2 : 0 : 3 : 1

	f1-score	precision	recall	support
AFIB	0.733636	0.750397	0.717794	204.0
GSVT	0.766964	0.762190	0.771818	300.0
SB	0.783529	0.781098	0.785985	892.0
SR	0.774555	0.774914	0.774218	681.0
macro avg	0.764671	0.767150	0.762454	2077.0
micro avg	0.773383	0.773383	0.773383	2077.0
weighted avg	0.773294	0.773324	0.773383	2077.0

The result of Model Number:Dataset Number:Featuregroup Number:Rescale of 2 : 0 : 4 : 0

	f1-score	precision	recall	support
AFIB	0.742222	0.735071	0.749627	875.0
GSVT	0.761668	0.763847	0.759581	691.0
SB	0.809784	0.807922	0.811661	1553.0
SR	0.787005	0.796265	0.777974	907.0
macro avg	0.775170	0.775776	0.774711	4026.0
micro avg	0.781651	0.781651	0.781651	4026.0
weighted avg	0.781710	0.781898	0.781651	4026.0

The result of Model Number:Dataset Number:Featuregroup Number:Rescale of 2 : 0 : 4 : 1

	f1-score	precision	recall	support
AFIB	0.803652	0.821529	0.786709	204.0
GSVT	0.837892	0.832685	0.843197	300.0
SB	0.856081	0.854278	0.857902	892.0
SR	0.846963	0.846364	0.847589	681.0
macro avg	0.836147	0.838714	0.833849	2077.0
micro avg	0.845404	0.845404	0.845404	2077.0
weighted avg	0.845315	0.845348	0.845404	2077.0

The result of Model Number:Dataset Number:Featuregroup Number:Rescale of 2 : 0 : 5 : 0

	f1-score	precision	recall	support
AFIB	0.765185	0.772614	0.758372	204.0
GSVT	0.788122	0.784397	0.792092	300.0
SB	0.804082	0.803185	0.804983	892.0
SR	0.797007	0.797832	0.796188	681.0
macro avg	0.788599	0.789507	0.787909	2077.0
micro avg	0.795659	0.795659	0.795659	2077.0
weighted avg	0.795637	0.795714	0.795659	2077.0

The result of Model Number:Dataset Number:Featuregroup Number:Rescale of 2 : 0 : 5 : 1

	f1-score	precision	recall	support
AFIB	0.775442	0.774187	0.776770	875.0
GSVT	0.782547	0.785659	0.779500	691.0
SB	0.821811	0.818824	0.824826	1553.0
SR	0.808369	0.812458	0.804365	907.0
macro avg	0.797042	0.797782	0.796365	4026.0
micro avg	0.801993	0.801993	0.801993	4026.0
weighted avg	0.801966	0.801996	0.801993	4026.0

The result of Model Number:Dataset Number:Featuregroup Number:Rescale of 2 : 0 : 6 : 0

	f1-score	precision	recall	support
AFIB	0.863709	0.855388	0.872327	875.0
GSVT	0.886338	0.888874	0.883909	691.0
SB	0.942330	0.940163	0.944514	1553.0
SR	0.915822	0.926598	0.905313	907.0
macro avg	0.902050	0.902756	0.901516	4026.0
micro avg	0.909592	0.909592	0.909592	4026.0
weighted avg	0.909661	0.909879	0.909592	4026.0

The result of Model Number:Dataset Number:Featuregroup Number:Rescale of 2 : 0 : 6 : 1

	f1-score	precision	recall	support
AFIB	0.815471	0.814495	0.816587	875.0
GSVT	0.831367	0.834227	0.828613	691.0
SB	0.880542	0.875117	0.886041	1553.0
SR	0.862623	0.871048	0.854454	907.0
macro avg	0.847501	0.848722	0.846424	4026.0
micro avg	0.853973	0.853973	0.853973	4026.0
weighted avg	0.853922	0.854007	0.853973	4026.0

The result of Model Number:Dataset Number:Featuregroup Number:Rescale of 2 : 0 : 7 : 0

	f1-score	precision	recall	support
AFIB	0.916256	0.926970	0.905958	204.0
GSVT	0.826468	0.794914	0.942988	300.0
SB	0.766793	0.764986	0.768616	892.0
SR	0.952098	0.953928	0.950282	681.0
macro avg	0.865404	0.860200	0.891961	2077.0
micro avg	0.866856	0.866856	0.866856	2077.0

weighted avg 0.850850 0.847169 0.866856 2077.0
The result of Model Number:Dataset Number:Featuregroup Number:Rescale of 2 : 0 : 7 : 1
f1-score precision recall support
AFIB 0.800454 0.818695 0.783136 875.0
GSVT 0.851785 0.853469 0.850393 691.0
SB 0.931910 0.926809 0.937070 1553.0
SR 0.880795 0.870110 0.891867 907.0
macro avg 0.866236 0.867271 0.865616 4026.0
micro avg 0.878554 0.878554 0.878554 4026.0
weighted avg 0.878072 0.877951 0.878554 4026.0
The result of Model Number:Dataset Number:Featuregroup Number:Rescale of 2 : 0 : 8 : 0
f1-score precision recall support
AFIB 0.688320 0.755799 0.632575 204.0
GSVT 0.807581 0.796696 0.818897 300.0
SB 0.857398 0.854539 0.860281 892.0
SR 0.835116 0.823919 0.846642 681.0
macro avg 0.797104 0.807738 0.789599 2077.0
micro avg 0.827467 0.827467 0.827467 2077.0
weighted avg 0.826290 0.826446 0.827467 2077.0
The result of Model Number:Dataset Number:Featuregroup Number:Rescale of 2 : 0 : 8 : 1
f1-score precision recall support
AFIB 0.635829 0.678927 0.598299 204.0
GSVT 0.599101 0.616877 0.582547 300.0
SB 0.626210 0.596149 0.659821 892.0
SR 0.555646 0.580873 0.533125 681.0
macro avg 0.604197 0.618206 0.593448 2077.0
micro avg 0.601076 0.601076 0.601076 2077.0
weighted avg 0.600103 0.602265 0.601076 2077.0
The result of Model Number:Dataset Number:Featuregroup Number:Rescale of 2 : 0 : 9 : 0
f1-score precision recall support
AFIB 0.814953 0.817705 0.812248 875.0
GSVT 0.812801 0.807076 0.818681 691.0
SB 0.855216 0.847278 0.863309 1553.0
SR 0.822702 0.838557 0.807477 907.0
macro avg 0.826418 0.827654 0.825429 4026.0
micro avg 0.831974 0.831974 0.831974 4026.0
weighted avg 0.831860 0.831986 0.831974 4026.0
The result of Model Number:Dataset Number:Featuregroup Number:Rescale of 2 : 0 : 9 : 1
f1-score precision recall support
AFIB 0.827531 0.819777 0.835465 875.0
GSVT 0.841440 0.848188 0.834881 691.0
SB 0.891970 0.888555 0.895416 1553.0
SR 0.872013 0.880864 0.863361 907.0

macro avg 0.858239 0.859346 0.857281 4026.0
micro avg 0.864775 0.864775 0.864775 4026.0
weighted avg 0.864796 0.864946 0.864775 4026.0
The result of Model Number:Dataset Number:Featuregroup Number:Rescale of 2 : 0 : 10 : 0

	f1-score	precision	recall	support
AFIB	0.711417	0.737792	0.686963	204.0
GSVT	0.732730	0.723568	0.742220	300.0
SB	0.751310	0.744455	0.758299	892.0
SR	0.731558	0.737235	0.725974	681.0
macro avg	0.731753	0.735762	0.728364	2077.0
micro avg	0.738372	0.738372	0.738372	2077.0
weighted avg	0.738232	0.738416	0.738372	2077.0

The result of Model Number:Dataset Number:Featuregroup Number:Rescale of 2 : 0 : 10 : 1

	f1-score	precision	recall	support
AFIB	0.871841	0.881328	0.862810	204.0
GSVT	0.901345	0.897586	0.905282	300.0
SB	0.919815	0.918993	0.920640	892.0
SR	0.911123	0.911130	0.911123	681.0
macro avg	0.901031	0.902259	0.899964	2077.0
micro avg	0.909621	0.909621	0.909621	2077.0
weighted avg	0.909585	0.909623	0.909621	2077.0

The result of Model Number:Dataset Number:Featuregroup Number:Rescale of 2 : 1 : 0 : 0

	f1-score	precision	recall	support
AFIB	0.862507	0.870181	0.855013	875.0
GSVT	0.880611	0.870947	0.890548	691.0
SB	0.947092	0.939718	0.954591	1553.0
SR	0.915619	0.928597	0.903002	907.0
macro avg	0.901457	0.902361	0.900789	4026.0
micro avg	0.910335	0.910335	0.910335	4026.0
weighted avg	0.910208	0.910296	0.910335	4026.0

The result of Model Number:Dataset Number:Featuregroup Number:Rescale of 2 : 1 : 0 : 1

	f1-score	precision	recall	support
AFIB	0.846087	0.881870	0.813375	204.0
GSVT	0.779184	0.780549	0.789513	300.0
SB	0.838864	0.798901	0.883849	892.0
SR	0.741597	0.801327	0.691495	681.0
macro avg	0.801433	0.815662	0.794558	2077.0
micro avg	0.800233	0.800233	0.800233	2077.0
weighted avg	0.799062	0.805195	0.800233	2077.0

The result of Model Number:Dataset Number:Featuregroup Number:Rescale of 2 : 1 : 1 : 0

	f1-score	precision	recall	support

AFIB	0.832332	0.838851	0.825959	875.0
GSVT	0.834528	0.823877	0.845509	691.0
SB	0.875325	0.871815	0.878873	1553.0
SR	0.856832	0.865243	0.848615	907.0
macro avg	0.849754	0.849946	0.849739	4026.0
micro avg	0.854830	0.854830	0.854830	4026.0
weighted avg	0.854813	0.854942	0.854830	4026.0

The result of Model Number:Dataset Number:Featuregroup Number:Rescale of 2 : 1 : 1 : 1

	f1-score	precision	recall	support
AFIB	0.797156	0.802890	0.791505	875.0
GSVT	0.795886	0.791120	0.800721	691.0
SB	0.839543	0.830241	0.849057	1553.0
SR	0.805606	0.819979	0.791755	907.0
macro avg	0.809548	0.811058	0.808260	4026.0
micro avg	0.815344	0.815344	0.815344	4026.0
weighted avg	0.815192	0.815270	0.815344	4026.0

The result of Model Number:Dataset Number:Featuregroup Number:Rescale of 2 : 1 : 2 : 0

	f1-score	precision	recall	support
AFIB	0.723572	0.734542	0.712943	204.0
GSVT	0.735207	0.729009	0.741549	300.0
SB	0.751515	0.749090	0.753957	892.0
SR	0.743447	0.746209	0.740726	681.0
macro avg	0.738435	0.739713	0.737294	2077.0
micro avg	0.743798	0.743798	0.743798	2077.0
weighted avg	0.743770	0.743816	0.743798	2077.0

The result of Model Number:Dataset Number:Featuregroup Number:Rescale of 2 : 1 : 2 : 1

	f1-score	precision	recall	support
AFIB	0.860207	0.877385	0.843820	204.0
GSVT	0.869867	0.858998	0.881146	300.0
SB	0.886474	0.877999	0.895118	892.0
SR	0.870376	0.881794	0.859274	681.0
macro avg	0.871731	0.874044	0.869839	2077.0
micro avg	0.876309	0.876309	0.876309	2077.0
weighted avg	0.876218	0.876438	0.876309	2077.0

The result of Model Number:Dataset Number:Featuregroup Number:Rescale of 2 : 1 : 3 : 0

	f1-score	precision	recall	support
AFIB	0.688133	0.689325	0.686955	875.0
GSVT	0.696477	0.688474	0.704725	691.0
SB	0.733414	0.724571	0.742479	1553.0
SR	0.698553	0.719719	0.678612	907.0
macro avg	0.704144	0.705522	0.703193	4026.0
micro avg	0.709544	0.709544	0.709544	4026.0
weighted avg	0.709380	0.709622	0.709544	4026.0

The result of Model Number:Dataset Number:Featuregroup Number:Rescale of 2 : 1 : 3 : 1

	f1-score	precision	recall	support
AFIB	0.717192	0.716535	0.717936	875.0
GSVT	0.731892	0.730473	0.733395	691.0
SB	0.776633	0.774903	0.778383	1553.0
SR	0.755436	0.760364	0.750598	907.0
macro avg	0.745288	0.745569	0.745078	4026.0
micro avg	0.751264	0.751264	0.751264	4026.0
weighted avg	0.751260	0.751317	0.751264	4026.0

The result of Model Number:Dataset Number:Featuregroup Number:Rescale of 2 : 1 : 4 : 0

	f1-score	precision	recall	support
AFIB	0.716678	0.721364	0.712096	204.0
GSVT	0.734634	0.732942	0.736343	300.0
SB	0.747747	0.746668	0.748835	892.0
SR	0.741854	0.742629	0.741094	681.0
macro avg	0.735228	0.735901	0.734592	2077.0
micro avg	0.740884	0.740884	0.740884	2077.0
weighted avg	0.740869	0.740876	0.740884	2077.0

The result of Model Number:Dataset Number:Featuregroup Number:Rescale of 2 : 1 : 4 : 1

	f1-score	precision	recall	support
AFIB	0.734400	0.741095	0.727921	204.0
GSVT	0.753565	0.751098	0.756072	300.0
SB	0.767341	0.766830	0.767856	892.0
SR	0.760592	0.760374	0.760822	681.0
macro avg	0.753975	0.754849	0.753168	2077.0
micro avg	0.759925	0.759925	0.759925	2077.0
weighted avg	0.759903	0.759913	0.759925	2077.0

The result of Model Number:Dataset Number:Featuregroup Number:Rescale of 2 : 1 : 5 : 0

	f1-score	precision	recall	support
AFIB	0.693737	0.684446	0.703327	875.0
GSVT	0.709247	0.714783	0.703809	691.0
SB	0.753023	0.750667	0.755398	1553.0
SR	0.734813	0.744554	0.725336	907.0
macro avg	0.722705	0.723612	0.721967	4026.0
micro avg	0.728454	0.728454	0.728454	4026.0
weighted avg	0.728522	0.728739	0.728454	4026.0

The result of Model Number:Dataset Number:Featuregroup Number:Rescale of 2 : 1 : 5 : 1

	f1-score	precision	recall	support
AFIB	0.725931	0.730276	0.721675	204.0
GSVT	0.745269	0.743054	0.747503	300.0
SB	0.756681	0.755754	0.757614	892.0
SR	0.751165	0.752052	0.750287	681.0
macro avg	0.744762	0.745284	0.744270	2077.0

micro avg 0.750221 0.750221 0.750221 2077.0

weighted avg 0.750204 0.750203 0.750221 2077.0

The result of Model Number:Dataset Number:Featuregroup Number:Rescale of 2 : 1 : 6 : 0

f1-score precision recall support

AFIB 0.691610 0.681790 0.701794 875.0

GSVT 0.716692 0.725835 0.707885 691.0

SB 0.761646 0.760528 0.762774 1553.0

SR 0.742671 0.748242 0.737185 907.0

macro avg 0.728155 0.729099 0.727409 4026.0

micro avg 0.734335 0.734335 0.734335 4026.0

weighted avg 0.734434 0.734693 0.734335 4026.0

The result of Model Number:Dataset Number:Featuregroup Number:Rescale of 2 : 1 : 6 : 1

f1-score precision recall support

AFIB 0.799144 0.806956 0.791668 875.0

GSVT 0.793040 0.787023 0.799410 691.0

SB 0.839126 0.830493 0.848016 1553.0

SR 0.812491 0.825558 0.800061 907.0

macro avg 0.810950 0.812508 0.809789 4026.0

micro avg 0.816624 0.816624 0.816624 4026.0

weighted avg 0.816526 0.816805 0.816624 4026.0

The result of Model Number:Dataset Number:Featuregroup Number:Rescale of 2 : 1 : 7 : 0

f1-score precision recall support

AFIB 0.694596 0.696834 0.692438 875.0

GSVT 0.704362 0.693179 0.715975 691.0

SB 0.742160 0.735963 0.748467 1553.0

SR 0.704853 0.722755 0.687843 907.0

macro avg 0.711493 0.712183 0.711181 4026.0

micro avg 0.717055 0.717055 0.717055 4026.0

weighted avg 0.716930 0.717140 0.717055 4026.0

The result of Model Number:Dataset Number:Featuregroup Number:Rescale of 2 : 1 : 7 : 1

f1-score precision recall support

AFIB 0.770014 0.759318 0.781089 875.0

GSVT 0.797531 0.808170 0.787272 691.0

SB 0.847237 0.845501 0.848983 1553.0

SR 0.825186 0.831562 0.818908 907.0

macro avg 0.809992 0.811138 0.809063 4026.0

micro avg 0.816860 0.816860 0.816860 4026.0

weighted avg 0.816954 0.817223 0.816860 4026.0

The result of Model Number:Dataset Number:Featuregroup Number:Rescale of 2 : 1 : 8 : 0

f1-score precision recall support

AFIB 0.898697 0.903630 0.893876 875.0

GSVT 0.899954 0.888576 0.911768 691.0

SB 0.946457 0.942659 0.950294 1553.0

SR 0.926140 0.937383 0.915221 907.0
macro avg 0.917812 0.918062 0.917790 4026.0
micro avg 0.923519 0.923519 0.923519 4026.0
weighted avg 0.923518 0.923705 0.923519 4026.0
The result of Model Number:Dataset Number:Featuregroup Number:Rescale of 2 : 1 : 8 : 1

	f1-score	precision	recall	support
AFIB	0.738667	0.741362	0.736039	875.0
GSVT	0.751434	0.741649	0.761544	691.0
SB	0.792087	0.783363	0.801012	1553.0
SR	0.750671	0.771408	0.731061	907.0
macro avg	0.758215	0.759445	0.757414	4026.0
micro avg	0.764358	0.764358	0.764358	4026.0
weighted avg	0.764169	0.764382	0.764358	4026.0

The result of Model Number:Dataset Number:Featuregroup Number:Rescale of 2 : 1 : 9 : 0

	f1-score	precision	recall	support
AFIB	0.736895	0.752352	0.722080	204.0
GSVT	0.753989	0.749528	0.758512	300.0
SB	0.772371	0.770562	0.774189	892.0
SR	0.765847	0.765627	0.766074	681.0
macro avg	0.757276	0.759517	0.755214	2077.0
micro avg	0.764146	0.764146	0.764146	2077.0
weighted avg	0.764093	0.764117	0.764146	2077.0

The result of Model Number:Dataset Number:Featuregroup Number:Rescale of 2 : 1 : 9 : 1

	f1-score	precision	recall	support
AFIB	0.777748	0.794062	0.762112	204.0
GSVT	0.795790	0.791082	0.800563	300.0
SB	0.815191	0.813282	0.817110	892.0
SR	0.808305	0.808073	0.808545	681.0
macro avg	0.799259	0.801625	0.797083	2077.0
micro avg	0.806510	0.806510	0.806510	2077.0
weighted avg	0.806453	0.806480	0.806510	2077.0

The result of Model Number:Dataset Number:Featuregroup Number:Rescale of 2 : 1 : 10 : 0

	f1-score	precision	recall	support
AFIB	0.760245	0.779496	0.741993	204.0
GSVT	0.776756	0.768309	0.785519	300.0
SB	0.798634	0.794648	0.802663	892.0
SR	0.786256	0.789783	0.782791	681.0
macro avg	0.780473	0.783059	0.778242	2077.0
micro avg	0.787712	0.787712	0.787712	2077.0
weighted avg	0.787645	0.787761	0.787712	2077.0

The result of Model Number:Dataset Number:Featuregroup Number:Rescale of 2 : 1 : 10 : 1

	f1-score	precision	recall	support
AFIB	0.708175	0.717553	0.699145	204.0
GSVT	0.729546	0.726913	0.732215	300.0
SB	0.738633	0.736334	0.740949	892.0
SR	0.730584	0.731991	0.729203	681.0
macro avg	0.726734	0.728198	0.725378	2077.0
micro avg	0.731730	0.731730	0.731730	2077.0
weighted avg	0.731690	0.731705	0.731730	2077.0

The result of Model Number:Dataset Number:Featuregroup Number:Rescale of 3 : 0 : 0 : 0

	f1-score	precision	recall	support
AFIB	0.762086	0.777374	0.747540	204.0
GSVT	0.779748	0.771643	0.788066	300.0
SB	0.794169	0.791080	0.797285	892.0
SR	0.785824	0.789103	0.782590	681.0
macro avg	0.780457	0.782300	0.778870	2077.0
micro avg	0.786249	0.786249	0.786249	2077.0
weighted avg	0.786199	0.786278	0.786249	2077.0

The result of Model Number:Dataset Number:Featuregroup Number:Rescale of 3 : 0 : 0 : 1

	f1-score	precision	recall	support
AFIB	0.764919	0.824537	0.713695	204.0
GSVT	0.788245	0.801588	0.775372	300.0
SB	0.835391	0.827703	0.843250	892.0
SR	0.802142	0.790510	0.814158	681.0
macro avg	0.797674	0.811084	0.786619	2077.0
micro avg	0.811182	0.811182	0.811182	2077.0
weighted avg	0.810758	0.811425	0.811182	2077.0

The result of Model Number:Dataset Number:Featuregroup Number:Rescale of 3 : 0 : 1 : 0

	f1-score	precision	recall	support
AFIB	0.820069	0.830700	0.809925	204.0
GSVT	0.851801	0.851250	0.852378	300.0
SB	0.869749	0.867912	0.871599	892.0
SR	0.861001	0.860512	0.861510	681.0
macro avg	0.850655	0.852594	0.848853	2077.0
micro avg	0.859457	0.859457	0.859457	2077.0
weighted avg	0.859409	0.859424	0.859457	2077.0

The result of Model Number:Dataset Number:Featuregroup Number:Rescale of 3 : 0 : 1 : 1

	f1-score	precision	recall	support
AFIB	0.810902	0.820176	0.802148	204.0
GSVT	0.829882	0.824971	0.834866	300.0
SB	0.796672	0.767244	0.846345	892.0
SR	0.668282	0.671516	0.665096	681.0
macro avg	0.776434	0.770977	0.787114	2077.0
micro avg	0.780919	0.780919	0.780919	2077.0

weighted avg 0.760770 0.749394 0.780919 2077.0

The result of Model Number:Dataset Number:Featuregroup Number:Rescale of 3 : 0 : 2 : 0

	f1-score	precision	recall	support
AFIB	0.670146	0.656797	0.684085	875.0
GSVT	0.545440	0.507230	0.683693	691.0
SB	0.442564	0.443327	0.441805	1553.0
SR	0.717453	0.723771	0.711275	907.0
macro avg	0.593901	0.582781	0.630215	4026.0
micro avg	0.596685	0.596685	0.596685	4026.0
weighted avg	0.571612	0.563870	0.596685	4026.0

The result of Model Number:Dataset Number:Featuregroup Number:Rescale of 3 : 0 : 2 : 1

	f1-score	precision	recall	support
AFIB	0.730296	0.723260	0.737583	875.0
GSVT	0.749430	0.751574	0.747376	691.0
SB	0.796773	0.794941	0.798619	1553.0
SR	0.774359	0.783471	0.765474	907.0
macro avg	0.762715	0.763311	0.762263	4026.0
micro avg	0.769092	0.769092	0.769092	4026.0
weighted avg	0.769150	0.769335	0.769092	4026.0

The result of Model Number:Dataset Number:Featuregroup Number:Rescale of 3 : 0 : 3 : 0

	f1-score	precision	recall	support
AFIB	0.826842	0.840269	0.813882	875.0
GSVT	0.879371	0.882127	0.876686	691.0
SB	0.956712	0.953415	0.960036	1553.0
SR	0.905727	0.895639	0.916100	907.0
macro avg	0.892163	0.892862	0.891676	4026.0
micro avg	0.904068	0.904068	0.904068	4026.0
weighted avg	0.903726	0.903573	0.904068	4026.0

The result of Model Number:Dataset Number:Featuregroup Number:Rescale of 3 : 0 : 3 : 1

	f1-score	precision	recall	support
AFIB	0.649234	0.644399	0.654208	875.0
GSVT	0.661343	0.663704	0.659038	691.0
SB	0.701766	0.700103	0.703437	1553.0
SR	0.685751	0.691925	0.679711	907.0
macro avg	0.674523	0.675033	0.674098	4026.0
micro avg	0.679772	0.679772	0.679772	4026.0
weighted avg	0.679803	0.679907	0.679772	4026.0

The result of Model Number:Dataset Number:Featuregroup Number:Rescale of 3 : 0 : 4 : 0

	f1-score	precision	recall	support
AFIB	0.847811	0.893930	0.806434	204.0
GSVT	0.890045	0.876583	0.903962	300.0
SB	0.911196	0.905268	0.917215	892.0
SR	0.899954	0.900765	0.899165	681.0

macro avg 0.887251 0.894137 0.881694 2077.0
micro avg 0.898502 0.898502 0.898502 2077.0
weighted avg 0.898229 0.898535 0.898502 2077.0
The result of Model Number:Dataset Number:Featuregroup Number:Rescale of 3 : 0 : 4 : 1

	f1-score	precision	recall	support
AFIB	0.700956	0.723723	0.679715	204.0
GSVT	0.720308	0.711124	0.729878	300.0
SB	0.740630	0.737837	0.743452	892.0
SR	0.730271	0.731594	0.728982	681.0
macro avg	0.723041	0.726069	0.720507	2077.0
micro avg	0.730487	0.730487	0.730487	2077.0
weighted avg	0.730402	0.730545	0.730487	2077.0

The result of Model Number:Dataset Number:Featuregroup Number:Rescale of 3 : 0 : 5 : 0

	f1-score	precision	recall	support
AFIB	0.855490	0.848946	0.862248	875.0
GSVT	0.875174	0.879959	0.870491	691.0
SB	0.926738	0.926089	0.927395	1553.0
SR	0.902122	0.906498	0.897835	907.0
macro avg	0.889881	0.890373	0.889492	4026.0
micro avg	0.896810	0.896810	0.896810	4026.0
weighted avg	0.896857	0.896992	0.896810	4026.0

The result of Model Number:Dataset Number:Featuregroup Number:Rescale of 3 : 0 : 5 : 1

	f1-score	precision	recall	support
AFIB	0.677110	0.677871	0.676473	204.0
GSVT	0.692655	0.691109	0.694261	300.0
SB	0.707337	0.706392	0.708287	892.0
SR	0.702039	0.703807	0.700289	681.0
macro avg	0.694785	0.694795	0.694827	2077.0
micro avg	0.700514	0.700514	0.700514	2077.0
weighted avg	0.700510	0.700536	0.700514	2077.0

The result of Model Number:Dataset Number:Featuregroup Number:Rescale of 3 : 0 : 6 : 0

	f1-score	precision	recall	support
AFIB	0.910926	0.918523	0.903766	204.0
GSVT	0.934990	0.930684	0.939355	300.0
SB	0.949090	0.945719	0.952492	892.0
SR	0.940324	0.944536	0.936199	681.0
macro avg	0.933832	0.934865	0.932953	2077.0
micro avg	0.940466	0.940466	0.940466	2077.0
weighted avg	0.940431	0.940488	0.940466	2077.0

The result of Model Number:Dataset Number:Featuregroup Number:Rescale of 3 : 0 : 6 : 1

	f1-score	precision	recall	support
AFIB	0.687054	0.684829	0.689314	875.0
GSVT	0.695021	0.697180	0.692943	691.0

SB 0.733360 0.731763 0.734966 1553.0
SR 0.719836 0.723232 0.716499 907.0
macro avg 0.708818 0.709251 0.708430 4026.0
micro avg 0.713671 0.713671 0.713671 4026.0
weighted avg 0.713669 0.713705 0.713671 4026.0
The result of Model Number:Dataset Number:Featuregroup Number:Rescale of 3 : 0 : 7 : 0
f1-score precision recall support
AFIB 0.881992 0.873923 0.890250 875.0
GSVT 0.893293 0.901175 0.885682 691.0
SB 0.949542 0.944536 0.954615 1553.0
SR 0.928077 0.939261 0.917240 907.0
macro avg 0.913226 0.914724 0.911947 4026.0
micro avg 0.920375 0.920375 0.920375 4026.0
weighted avg 0.920371 0.920558 0.920375 4026.0
The result of Model Number:Dataset Number:Featuregroup Number:Rescale of 3 : 0 : 7 : 1
f1-score precision recall support
AFIB 0.850758 0.859017 0.843183 204.0
GSVT 0.876260 0.872118 0.880673 300.0
SB 0.894004 0.893007 0.895006 892.0
SR 0.886138 0.887056 0.885227 681.0
macro avg 0.876790 0.877799 0.876022 2077.0
micro avg 0.884640 0.884640 0.884640 2077.0
weighted avg 0.884615 0.884700 0.884640 2077.0
The result of Model Number:Dataset Number:Featuregroup Number:Rescale of 3 : 0 : 8 : 0
f1-score precision recall support
AFIB 0.649087 0.639135 0.659462 875.0
GSVT 0.671469 0.678050 0.665146 691.0
SB 0.714622 0.712952 0.716322 1553.0
SR 0.696770 0.705605 0.688169 907.0
macro avg 0.682987 0.683935 0.682275 4026.0
micro avg 0.688838 0.688838 0.688838 4026.0
weighted avg 0.688950 0.689263 0.688838 4026.0
The result of Model Number:Dataset Number:Featuregroup Number:Rescale of 3 : 0 : 8 : 1
f1-score precision recall support
AFIB 0.780269 0.767777 0.877961 204.0
GSVT 0.913489 0.902890 0.924674 300.0
SB 0.944076 0.940511 0.947674 892.0
SR 0.740739 0.744569 0.737074 681.0
macro avg 0.844644 0.838937 0.871846 2077.0
micro avg 0.868454 0.868454 0.868454 2077.0
weighted avg 0.856900 0.853867 0.868454 2077.0
The result of Model Number:Dataset Number:Featuregroup Number:Rescale of 3 : 0 : 9 : 0
f1-score precision recall support

AFIB	0.893640	0.904088	0.883601	204.0
GSVT	0.918223	0.914001	0.922509	300.0
SB	0.931224	0.928328	0.934144	892.0
SR	0.923204	0.925820	0.920641	681.0
macro avg	0.916572	0.918059	0.915224	2077.0
micro avg	0.923072	0.923072	0.923072	2077.0
weighted avg	0.923025	0.923056	0.923072	2077.0

The result of Model Number:Dataset Number:Featuregroup Number:Rescale of 3 : 0 : 9 : 1

	f1-score	precision	recall	support
AFIB	0.830689	0.839531	0.822492	204.0
GSVT	0.855102	0.849404	0.861127	300.0
SB	0.873172	0.872391	0.873955	892.0
SR	0.865809	0.866965	0.864663	681.0
macro avg	0.856193	0.857073	0.855559	2077.0
micro avg	0.864001	0.864001	0.864001	2077.0
weighted avg	0.863975	0.864064	0.864001	2077.0

The result of Model Number:Dataset Number:Featuregroup Number:Rescale of 3 : 0 : 10 : 0

	f1-score	precision	recall	support
AFIB	0.659950	0.702950	0.622200	204.0
GSVT	0.688862	0.671930	0.706763	300.0
SB	0.709064	0.699956	0.718438	892.0
SR	0.690647	0.698894	0.682639	681.0
macro avg	0.687131	0.693432	0.682510	2077.0
micro avg	0.695562	0.695562	0.695562	2077.0
weighted avg	0.695284	0.695854	0.695562	2077.0

The result of Model Number:Dataset Number:Featuregroup Number:Rescale of 3 : 0 : 10 : 1

	f1-score	precision	recall	support
AFIB	0.829488	0.833839	0.825232	875.0
GSVT	0.846833	0.845312	0.848420	691.0
SB	0.919515	0.912463	0.926679	1553.0
SR	0.887074	0.895953	0.878375	907.0
macro avg	0.870727	0.871892	0.869676	4026.0
micro avg	0.880316	0.880316	0.880316	4026.0
weighted avg	0.880166	0.880130	0.880316	4026.0

The result of Model Number:Dataset Number:Featuregroup Number:Rescale of 3 : 1 : 0 : 0

	f1-score	precision	recall	support
AFIB	0.759505	0.822908	0.706687	204.0
GSVT	0.814657	0.804414	0.825497	300.0
SB	0.835455	0.823750	0.847594	892.0
SR	0.812665	0.816472	0.809289	681.0
macro avg	0.805570	0.816886	0.797267	2077.0

micro avg 0.818003 0.818003 0.818003 2077.0
weighted avg 0.817519 0.818488 0.818003 2077.0
The result of Model Number:Dataset Number:Featuregroup Number:Rescale of 3 : 1 : 0 : 1
f1-score precision recall support
AFIB 0.767298 0.792220 0.744047 204.0
GSVT 0.788482 0.778428 0.798958 300.0
SB 0.810727 0.807670 0.813816 892.0
SR 0.799388 0.800836 0.797976 681.0
macro avg 0.791474 0.794789 0.788699 2077.0
micro avg 0.799624 0.799624 0.799624 2077.0
weighted avg 0.799531 0.799688 0.799624 2077.0
The result of Model Number:Dataset Number:Featuregroup Number:Rescale of 3 : 1 : 1 : 0
f1-score precision recall support
AFIB 0.698162 0.743652 0.658226 204.0
GSVT 0.728747 0.710835 0.747685 300.0
SB 0.750120 0.740484 0.760036 892.0
SR 0.730636 0.739361 0.722164 681.0
macro avg 0.726916 0.733583 0.722028 2077.0
micro avg 0.735835 0.735835 0.735835 2077.0
weighted avg 0.735541 0.736144 0.735835 2077.0
The result of Model Number:Dataset Number:Featuregroup Number:Rescale of 3 : 1 : 1 : 1
f1-score precision recall support
AFIB 0.879083 0.868714 0.889747 875.0
GSVT 0.893975 0.899631 0.888421 691.0
SB 0.953150 0.951868 0.954438 1553.0
SR 0.929734 0.938542 0.921121 907.0
macro avg 0.913985 0.914689 0.913432 4026.0
micro avg 0.921542 0.921542 0.921542 4026.0
weighted avg 0.921620 0.921828 0.921542 4026.0
The result of Model Number:Dataset Number:Featuregroup Number:Rescale of 3 : 1 : 2 : 0
f1-score precision recall support
AFIB 0.706548 0.723331 0.690590 204.0
GSVT 0.729756 0.722914 0.736806 300.0
SB 0.745428 0.744595 0.746264 892.0
SR 0.738233 0.737491 0.738989 681.0
macro avg 0.729991 0.732083 0.728162 2077.0
micro avg 0.737044 0.737044 0.737044 2077.0
weighted avg 0.736987 0.737046 0.737044 2077.0
The result of Model Number:Dataset Number:Featuregroup Number:Rescale of 3 : 1 : 2 : 1
f1-score precision recall support
AFIB 0.759329 0.769789 0.749484 875.0
GSVT 0.775906 0.772693 0.779356 691.0
SB 0.826461 0.815625 0.837623 1553.0

SR 0.800514 0.812468 0.789261 907.0
macro avg 0.790553 0.792644 0.788931 4026.0
micro avg 0.797571 0.797571 0.797571 4026.0
weighted avg 0.797348 0.797583 0.797571 4026.0
The result of Model Number:Dataset Number:Featuregroup Number:Rescale of 3 : 1 : 3 : 0
f1-score precision recall support
AFIB 0.723123 0.728530 0.717823 204.0
GSVT 0.741600 0.737686 0.745556 300.0
SB 0.754115 0.753107 0.755129 892.0
SR 0.747473 0.748906 0.746047 681.0
macro avg 0.741578 0.742057 0.741139 2077.0
micro avg 0.747104 0.747104 0.747104 2077.0
weighted avg 0.747086 0.747088 0.747104 2077.0
The result of Model Number:Dataset Number:Featuregroup Number:Rescale of 3 : 1 : 3 : 1
f1-score precision recall support
AFIB 0.795400 0.778403 0.813202 875.0
GSVT 0.823688 0.836283 0.811540 691.0
SB 0.878049 0.878165 0.877937 1553.0
SR 0.855429 0.864287 0.846753 907.0
macro avg 0.838142 0.839285 0.837358 4026.0
micro avg 0.845446 0.845446 0.845446 4026.0
weighted avg 0.845660 0.846168 0.845446 4026.0
The result of Model Number:Dataset Number:Featuregroup Number:Rescale of 3 : 1 : 4 : 0
f1-score precision recall support
AFIB 0.816919 0.843104 0.792556 204.0
GSVT 0.841359 0.826884 0.856474 300.0
SB 0.855913 0.847452 0.864547 892.0
SR 0.833028 0.843452 0.822876 681.0
macro avg 0.836805 0.840223 0.834113 2077.0
micro avg 0.842647 0.842647 0.842647 2077.0
weighted avg 0.842478 0.842743 0.842647 2077.0
The result of Model Number:Dataset Number:Featuregroup Number:Rescale of 3 : 1 : 4 : 1
f1-score precision recall support
AFIB 0.831703 0.825509 0.838074 875.0
GSVT 0.847214 0.850239 0.844261 691.0
SB 0.898998 0.896868 0.901139 1553.0
SR 0.878482 0.886391 0.870745 907.0
macro avg 0.864099 0.864752 0.863555 4026.0
micro avg 0.870823 0.870823 0.870823 4026.0
weighted avg 0.870862 0.870996 0.870823 4026.0
The result of Model Number:Dataset Number:Featuregroup Number:Rescale of 3 : 1 : 5 : 0
f1-score precision recall support
AFIB 0.615669 0.629591 0.602367 875.0

GSVT	0.652748	0.655527	0.650033	691.0
SB	0.708750	0.705409	0.712126	1553.0
SR	0.671531	0.661230	0.682185	907.0
macro avg	0.662174	0.662939	0.661678	4026.0
micro avg	0.670869	0.670869	0.670869	4026.0
weighted avg	0.670523	0.670417	0.670869	4026.0

The result of Model Number:Dataset Number:Featuregroup Number:Rescale of 3 : 1 : 5 : 1

	f1-score	precision	recall	support
AFIB	0.781017	0.775607	0.786661	875.0
GSVT	0.793066	0.796285	0.790002	691.0
SB	0.860479	0.851531	0.869621	1553.0
SR	0.817032	0.836044	0.798941	907.0
macro avg	0.812899	0.814867	0.811306	4026.0
micro avg	0.822002	0.822002	0.822002	4026.0
weighted avg	0.821851	0.822059	0.822002	4026.0

The result of Model Number:Dataset Number:Featuregroup Number:Rescale of 3 : 1 : 6 : 0

	f1-score	precision	recall	support
AFIB	0.842390	0.846238	0.838624	875.0
GSVT	0.856434	0.849191	0.863864	691.0
SB	0.905268	0.896024	0.914710	1553.0
SR	0.858547	0.876646	0.841226	907.0
macro avg	0.865660	0.867024	0.864606	4026.0
micro avg	0.872892	0.872892	0.872892	4026.0
weighted avg	0.872695	0.872800	0.872892	4026.0

The result of Model Number:Dataset Number:Featuregroup Number:Rescale of 3 : 1 : 6 : 1

	f1-score	precision	recall	support
AFIB	0.780851	0.776180	0.785685	875.0
GSVT	0.787246	0.791496	0.783154	691.0
SB	0.834362	0.831923	0.836828	1553.0
SR	0.815509	0.821367	0.809750	907.0
macro avg	0.804492	0.805241	0.803854	4026.0
micro avg	0.810400	0.810400	0.810400	4026.0
weighted avg	0.810398	0.810491	0.810400	4026.0

The result of Model Number:Dataset Number:Featuregroup Number:Rescale of 3 : 1 : 7 : 0

	f1-score	precision	recall	support
AFIB	0.765962	0.768000	0.763982	875.0
GSVT	0.780691	0.775192	0.786316	691.0
SB	0.825283	0.816868	0.833889	1553.0
SR	0.787558	0.804712	0.771148	907.0
macro avg	0.789874	0.791193	0.788834	4026.0
micro avg	0.796396	0.796396	0.796396	4026.0
weighted avg	0.796238	0.796356	0.796396	4026.0

The result of Model Number:Dataset Number:Featuregroup Number:Rescale of 3 : 1 : 7 : 1

	f1-score	precision	recall	support
AFIB	0.748553	0.825494	0.685191	204.0
GSVT	0.890756	0.878718	0.903216	300.0
SB	0.948591	0.944798	0.952419	892.0
SR	0.922845	0.910370	0.935707	681.0
macro avg	0.877686	0.889845	0.869133	2077.0
micro avg	0.913586	0.913586	0.913586	2077.0
weighted avg	0.912148	0.912247	0.913586	2077.0

The result of Model Number:Dataset Number:Featuregroup Number:Rescale of 3 : 1 : 8 : 0

	f1-score	precision	recall	support
AFIB	0.921615	0.931644	0.912069	204.0
GSVT	0.952804	0.948830	0.956965	300.0
SB	0.972329	0.971459	0.973200	892.0
SR	0.963140	0.963148	0.963140	681.0
macro avg	0.952472	0.953770	0.951344	2077.0
micro avg	0.961552	0.961552	0.961552	2077.0
weighted avg	0.961515	0.961555	0.961552	2077.0

The result of Model Number:Dataset Number:Featuregroup Number:Rescale of 3 : 1 : 8 : 1

	f1-score	precision	recall	support
AFIB	0.516723	0.562890	0.545671	204.0
GSVT	0.701854	0.690381	0.713957	300.0
SB	0.597494	0.592943	0.602160	892.0
SR	0.730638	0.722371	0.739118	681.0
macro avg	0.636678	0.642146	0.650227	2077.0
micro avg	0.657665	0.657665	0.657665	2077.0
weighted avg	0.648290	0.646502	0.657665	2077.0

The result of Model Number:Dataset Number:Featuregroup Number:Rescale of 3 : 1 : 9 : 0

	f1-score	precision	recall	support
AFIB	0.793201	0.818968	0.769157	204.0
GSVT	0.808238	0.801003	0.815765	300.0
SB	0.827457	0.821785	0.833209	892.0
SR	0.813962	0.817396	0.810605	681.0
macro avg	0.810715	0.814788	0.807184	2077.0
micro avg	0.816987	0.816987	0.816987	2077.0
weighted avg	0.816892	0.817068	0.816987	2077.0

The result of Model Number:Dataset Number:Featuregroup Number:Rescale of 3 : 1 : 9 : 1

	f1-score	precision	recall	support
AFIB	0.702159	0.723611	0.682020	875.0
GSVT	0.745141	0.742617	0.747747	691.0
SB	0.814249	0.810978	0.817550	1553.0
SR	0.771184	0.757808	0.785117	907.0
macro avg	0.758183	0.758754	0.758109	4026.0
micro avg	0.768807	0.768807	0.768807	4026.0

weighted avg 0.768324 0.768278 0.768807 4026.0

The result of Model Number:Dataset Number:Featuregroup Number:Rescale of 3 : 1 : 10 : 0

	f1-score	precision	recall	support
AFIB	0.732230	0.742011	0.722911	204.0
GSVT	0.745166	0.740626	0.749875	300.0
SB	0.763128	0.762452	0.763811	892.0
SR	0.757489	0.757604	0.757379	681.0
macro avg	0.749503	0.750673	0.748494	2077.0
micro avg	0.755672	0.755672	0.755672	2077.0
weighted avg	0.755650	0.755702	0.755672	2077.0

The result of Model Number:Dataset Number:Featuregroup Number:Rescale of 3 : 1 : 10 : 1

	f1-score	precision	recall	support
AFIB	0.674434	0.679715	0.669269	875.0
GSVT	0.676212	0.667582	0.685110	691.0
SB	0.709270	0.706426	0.712145	1553.0
SR	0.694286	0.701101	0.687627	907.0
macro avg	0.688550	0.688706	0.688538	4026.0
micro avg	0.692663	0.692663	0.692663	4026.0
weighted avg	0.692649	0.692754	0.692663	4026.0

The result of Model Number:Dataset Number:Featuregroup Number:Rescale of 4 : 0 : 0 : 0

	f1-score	precision	recall	support
AFIB	0.773062	0.779190	0.767050	875.0
GSVT	0.782863	0.779412	0.786372	691.0
SB	0.832483	0.829191	0.835806	1553.0
SR	0.812026	0.814320	0.809783	907.0
macro avg	0.800108	0.800528	0.799753	4026.0
micro avg	0.806515	0.806515	0.806515	4026.0
weighted avg	0.806443	0.806430	0.806515	4026.0

The result of Model Number:Dataset Number:Featuregroup Number:Rescale of 4 : 0 : 0 : 1

	f1-score	precision	recall	support
AFIB	0.878159	0.866947	0.889716	875.0
GSVT	0.893572	0.898697	0.888530	691.0
SB	0.956514	0.952497	0.960575	1553.0
SR	0.928350	0.943376	0.913811	907.0
macro avg	0.914149	0.915379	0.913158	4026.0
micro avg	0.922274	0.922274	0.922274	4026.0
weighted avg	0.922337	0.922615	0.922274	4026.0

The result of Model Number:Dataset Number:Featuregroup Number:Rescale of 4 : 0 : 1 : 0

	f1-score	precision	recall	support
AFIB	0.617145	0.593552	0.655966	875.0
GSVT	0.639467	0.617397	0.669402	691.0

SB	0.712485	0.699908	0.726304	1553.0
SR	0.603458	0.725624	0.521005	907.0
macro avg	0.643139	0.659120	0.643169	4026.0
micro avg	0.654999	0.654999	0.654999	4026.0
weighted avg	0.654669	0.668425	0.654999	4026.0

The result of Model Number:Dataset Number:Featuregroup Number:Rescale of 4 : 0 : 1 : 1

	f1-score	precision	recall	support
AFIB	0.664921	0.656874	0.673196	875.0
GSVT	0.673921	0.680831	0.667203	691.0
SB	0.715773	0.713443	0.718123	1553.0
SR	0.700117	0.707311	0.693092	907.0
macro avg	0.688683	0.689615	0.687904	4026.0
micro avg	0.693980	0.693980	0.693980	4026.0
weighted avg	0.694011	0.694170	0.693980	4026.0

The result of Model Number:Dataset Number:Featuregroup Number:Rescale of 4 : 0 : 2 : 0

	f1-score	precision	recall	support
AFIB	0.648596	0.640255	0.657204	875.0
GSVT	0.661799	0.667278	0.656437	691.0
SB	0.707597	0.704447	0.710782	1553.0
SR	0.687460	0.697628	0.677601	907.0
macro avg	0.676363	0.677402	0.675506	4026.0
micro avg	0.682335	0.682335	0.682335	4026.0
weighted avg	0.682377	0.682580	0.682335	4026.0

The result of Model Number:Dataset Number:Featuregroup Number:Rescale of 4 : 0 : 2 : 1

	f1-score	precision	recall	support
AFIB	0.642584	0.647906	0.723783	204.0
GSVT	0.696839	0.667013	0.786198	300.0
SB	0.636240	0.634029	0.638468	892.0
SR	0.629914	0.630033	0.629799	681.0
macro avg	0.651394	0.644745	0.694562	2077.0
micro avg	0.665343	0.665343	0.665343	2077.0
weighted avg	0.643542	0.638846	0.665343	2077.0

The result of Model Number:Dataset Number:Featuregroup Number:Rescale of 4 : 0 : 3 : 0

	f1-score	precision	recall	support
AFIB	0.666620	0.657876	0.675630	875.0
GSVT	0.676826	0.684349	0.669478	691.0
SB	0.717861	0.716167	0.719568	1553.0
SR	0.699888	0.706187	0.693710	907.0
macro avg	0.690299	0.691145	0.689597	4026.0
micro avg	0.695596	0.695596	0.695596	4026.0
weighted avg	0.695633	0.695789	0.695596	4026.0

The result of Model Number:Dataset Number:Featuregroup Number:Rescale of 4 : 0 : 3 : 1

	f1-score	precision	recall	support

AFIB	0.644225	0.643188	0.645528	875.0
GSVT	0.659245	0.655809	0.662756	691.0
SB	0.689275	0.683436	0.695221	1553.0
SR	0.662963	0.677702	0.649075	907.0
macro avg	0.663927	0.665034	0.663145	4026.0
micro avg	0.668453	0.668453	0.668453	4026.0
weighted avg	0.668402	0.668655	0.668453	4026.0

The result of Model Number:Dataset Number:Featuregroup Number:Rescale of 4 : 0 : 4 : 0

	f1-score	precision	recall	support
AFIB	0.673703	0.665101	0.682569	875.0
GSVT	0.685528	0.689459	0.681659	691.0
SB	0.733815	0.730733	0.736930	1553.0
SR	0.712208	0.723736	0.701054	907.0
macro avg	0.701313	0.702257	0.700553	4026.0
micro avg	0.707547	0.707547	0.707547	4026.0
weighted avg	0.707595	0.707808	0.707547	4026.0

The result of Model Number:Dataset Number:Featuregroup Number:Rescale of 4 : 0 : 4 : 1

	f1-score	precision	recall	support
AFIB	0.702998	0.778750	0.641240	204.0
GSVT	0.828650	0.817035	0.840814	300.0
SB	0.879826	0.876994	0.882686	892.0
SR	0.857300	0.844260	0.870772	681.0
macro avg	0.817194	0.829260	0.808878	2077.0
micro avg	0.849017	0.849017	0.849017	2077.0
weighted avg	0.847681	0.847951	0.849017	2077.0

The result of Model Number:Dataset Number:Featuregroup Number:Rescale of 4 : 0 : 5 : 0

	f1-score	precision	recall	support
AFIB	0.733480	0.818058	0.664849	204.0
GSVT	0.864848	0.849795	0.880628	300.0
SB	0.923390	0.919297	0.927530	892.0
SR	0.901962	0.889629	0.914669	681.0
macro avg	0.855920	0.869195	0.846919	2077.0
micro avg	0.890738	0.890738	0.890738	2077.0
weighted avg	0.889256	0.889587	0.890738	2077.0

The result of Model Number:Dataset Number:Featuregroup Number:Rescale of 4 : 0 : 5 : 1

	f1-score	precision	recall	support
AFIB	0.713436	0.713943	0.712943	875.0
GSVT	0.723049	0.724475	0.721691	691.0
SB	0.768000	0.765448	0.770572	1553.0
SR	0.749954	0.752742	0.747212	907.0
macro avg	0.738610	0.739152	0.738104	4026.0
micro avg	0.744395	0.744395	0.744395	4026.0
weighted avg	0.744361	0.744359	0.744395	4026.0

The result of Model Number:Dataset Number:Featuregroup Number:Rescale of 4 : 0 : 6 : 0

	f1-score	precision	recall	support
AFIB	0.840055	0.905529	0.783800	204.0
GSVT	0.865672	0.880326	0.851535	300.0
SB	0.917450	0.909006	0.926080	892.0
SR	0.880934	0.868160	0.894131	681.0
macro avg	0.876028	0.890755	0.863887	2077.0
micro avg	0.890863	0.890863	0.890863	2077.0
weighted avg	0.890397	0.891129	0.890863	2077.0

The result of Model Number:Dataset Number:Featuregroup Number:Rescale of 4 : 0 : 6 : 1

	f1-score	precision	recall	support
AFIB	0.669328	0.663053	0.675808	875.0
GSVT	0.634587	0.619334	0.681236	691.0
SB	0.733781	0.731087	0.736516	1553.0
SR	0.570722	0.574724	0.566783	907.0
macro avg	0.652105	0.647050	0.665086	4026.0
micro avg	0.675596	0.675596	0.675596	4026.0
weighted avg	0.666013	0.661894	0.675596	4026.0

The result of Model Number:Dataset Number:Featuregroup Number:Rescale of 4 : 0 : 7 : 0

	f1-score	precision	recall	support
AFIB	0.728959	0.724368	0.821122	204.0
GSVT	0.888613	0.884042	0.893381	300.0
SB	0.913914	0.909566	0.918315	892.0
SR	0.716863	0.719225	0.714949	681.0
macro avg	0.812087	0.809300	0.836941	2077.0
micro avg	0.838488	0.838488	0.838488	2077.0
weighted avg	0.827485	0.825281	0.838488	2077.0

The result of Model Number:Dataset Number:Featuregroup Number:Rescale of 4 : 0 : 7 : 1

	f1-score	precision	recall	support
AFIB	0.874411	0.885746	0.863595	204.0
GSVT	0.908245	0.907658	0.908860	300.0
SB	0.927383	0.925424	0.929356	892.0
SR	0.918055	0.917534	0.918597	681.0
macro avg	0.907024	0.909091	0.905102	2077.0
micro avg	0.916409	0.916409	0.916409	2077.0
weighted avg	0.916357	0.916374	0.916409	2077.0

The result of Model Number:Dataset Number:Featuregroup Number:Rescale of 4 : 0 : 8 : 0

	f1-score	precision	recall	support
AFIB	0.874674	0.897573	0.853314	204.0
GSVT	0.823008	0.846927	0.800851	300.0
SB	0.868449	0.837873	0.901358	892.0
SR	0.787143	0.812778	0.763235	681.0
macro avg	0.838319	0.848788	0.829689	2077.0

micro avg 0.836834 0.836834 0.836834 2077.0
weighted avg 0.835839 0.836816 0.836834 2077.0
The result of Model Number:Dataset Number:Featuregroup Number:Rescale of 4 : 0 : 8 : 1
f1-score precision recall support
AFIB 0.768705 0.751236 0.787059 875.0
GSVT 0.795049 0.807942 0.782626 691.0
SB 0.848231 0.848236 0.848231 1553.0
SR 0.826521 0.835937 0.817317 907.0
macro avg 0.809626 0.810838 0.808808 4026.0
micro avg 0.816711 0.816711 0.816711 4026.0
weighted avg 0.816928 0.817468 0.816711 4026.0
The result of Model Number:Dataset Number:Featuregroup Number:Rescale of 4 : 0 : 9 : 0
f1-score precision recall support
AFIB 0.670269 0.683257 0.658066 204.0
GSVT 0.696260 0.690438 0.702309 300.0
SB 0.709252 0.707828 0.710683 892.0
SR 0.702489 0.703217 0.701766 681.0
macro avg 0.694567 0.696185 0.693206 2077.0
micro avg 0.701382 0.701382 0.701382 2077.0
weighted avg 0.701329 0.701391 0.701382 2077.0
The result of Model Number:Dataset Number:Featuregroup Number:Rescale of 4 : 0 : 9 : 1
f1-score precision recall support
AFIB 0.841106 0.879087 0.806545 204.0
GSVT 0.870361 0.860586 0.880518 300.0
SB 0.837254 0.800752 0.895874 892.0
SR 0.697548 0.703495 0.691818 681.0
macro avg 0.811567 0.810980 0.818689 2077.0
micro avg 0.817977 0.817977 0.817977 2077.0
weighted avg 0.796608 0.785200 0.817977 2077.0
The result of Model Number:Dataset Number:Featuregroup Number:Rescale of 4 : 0 : 10 :
0
f1-score precision recall support
AFIB 0.666631 0.679474 0.654580 204.0
GSVT 0.679150 0.668115 0.690679 300.0
SB 0.690488 0.682253 0.698926 892.0
SR 0.677643 0.690348 0.665409 681.0
macro avg 0.678478 0.680047 0.677398 2077.0
micro avg 0.682390 0.682390 0.682390 2077.0
weighted avg 0.682296 0.682592 0.682390 2077.0
The result of Model Number:Dataset Number:Featuregroup Number:Rescale of 4 : 0 : 10 :
1
f1-score precision recall support
AFIB 0.766052 0.749682 0.783197 875.0

GSVT	0.793295	0.805426	0.781595	691.0
SB	0.845651	0.845763	0.845543	1553.0
SR	0.823865	0.832397	0.815509	907.0
macro avg	0.807216	0.808317	0.806461	4026.0
micro avg	0.814251	0.814251	0.814251	4026.0
weighted avg	0.814457	0.814946	0.814251	4026.0

The result of Model Number:Dataset Number:Featuregroup Number:Rescale of 4 : 1 : 0 : 0

	f1-score	precision	recall	support
AFIB	0.712194	0.785398	0.651910	204.0
GSVT	0.847490	0.836037	0.859345	300.0
SB	0.902516	0.898907	0.906158	892.0
SR	0.878021	0.866152	0.890257	681.0
macro avg	0.835055	0.846623	0.826918	2077.0
micro avg	0.869211	0.869211	0.869211	2077.0
weighted avg	0.867844	0.867938	0.869211	2077.0

The result of Model Number:Dataset Number:Featuregroup Number:Rescale of 4 : 1 : 0 : 1

	f1-score	precision	recall	support
AFIB	0.859406	0.867138	0.852079	875.0
GSVT	0.876912	0.859131	0.895651	691.0
SB	0.929971	0.919625	0.940664	1553.0
SR	0.893068	0.919615	0.868221	907.0
macro avg	0.889839	0.891377	0.889154	4026.0
micro avg	0.897365	0.897365	0.897365	4026.0
weighted avg	0.897214	0.897833	0.897365	4026.0

The result of Model Number:Dataset Number:Featuregroup Number:Rescale of 4 : 1 : 1 : 0

	f1-score	precision	recall	support
AFIB	0.801877	0.783655	0.821023	875.0
GSVT	0.829359	0.842808	0.816400	691.0
SB	0.884835	0.884841	0.884836	1553.0
SR	0.862189	0.872011	0.852587	907.0
macro avg	0.844565	0.845829	0.843712	4026.0
micro avg	0.851956	0.851956	0.851956	4026.0
weighted avg	0.852182	0.852745	0.851956	4026.0

The result of Model Number:Dataset Number:Featuregroup Number:Rescale of 4 : 1 : 1 : 1

	f1-score	precision	recall	support
AFIB	0.833009	0.851307	0.815793	204.0
GSVT	0.859855	0.852906	0.867037	300.0
SB	0.877876	0.876796	0.878959	892.0
SR	0.870465	0.869708	0.871229	681.0
macro avg	0.860301	0.862679	0.858255	2077.0
micro avg	0.868499	0.868499	0.868499	2077.0
weighted avg	0.868437	0.868518	0.868499	2077.0

The result of Model Number:Dataset Number:Featuregroup Number:Rescale of 4 : 1 : 2 : 0

	f1-score	precision	recall	support
AFIB	0.648997	0.639789	0.658489	875.0
GSVT	0.662349	0.668805	0.656022	691.0
SB	0.704868	0.702571	0.707182	1553.0
SR	0.688407	0.697206	0.679836	907.0
macro avg	0.676155	0.677093	0.675382	4026.0
micro avg	0.681658	0.681658	0.681658	4026.0
weighted avg	0.681719	0.681922	0.681658	4026.0

The result of Model Number:Dataset Number:Featuregroup Number:Rescale of 4 : 1 : 2 : 1

	f1-score	precision	recall	support
AFIB	0.889298	0.897372	0.881824	204.0
GSVT	0.918690	0.913138	0.924550	300.0
SB	0.937910	0.936653	0.939171	892.0
SR	0.929232	0.931158	0.927319	681.0
macro avg	0.918782	0.919580	0.918216	2077.0
micro avg	0.927541	0.927541	0.927541	2077.0
weighted avg	0.927514	0.927597	0.927541	2077.0

The result of Model Number:Dataset Number:Featuregroup Number:Rescale of 4 : 1 : 3 : 0

	f1-score	precision	recall	support
AFIB	0.795935	0.797188	0.794788	204.0
GSVT	0.815184	0.813912	0.816528	300.0
SB	0.832000	0.830985	0.833024	892.0
SR	0.826174	0.827759	0.824598	681.0
macro avg	0.817323	0.817461	0.817235	2077.0
micro avg	0.824123	0.824123	0.824123	2077.0
weighted avg	0.824119	0.824142	0.824123	2077.0

The result of Model Number:Dataset Number:Featuregroup Number:Rescale of 4 : 1 : 3 : 1

	f1-score	precision	recall	support
AFIB	0.614795	0.627715	0.602420	875.0
GSVT	0.654684	0.656933	0.652483	691.0
SB	0.713288	0.709926	0.716685	1553.0
SR	0.675207	0.666215	0.684501	907.0
macro avg	0.664493	0.665197	0.664022	4026.0
micro avg	0.673581	0.673581	0.673581	4026.0
weighted avg	0.673244	0.673116	0.673581	4026.0

The result of Model Number:Dataset Number:Featuregroup Number:Rescale of 4 : 1 : 4 : 0

	f1-score	precision	recall	support
AFIB	0.827472	0.864837	0.793472	204.0
GSVT	0.856252	0.846636	0.866245	300.0
SB	0.823683	0.787772	0.881352	892.0
SR	0.686241	0.692092	0.680604	681.0
macro avg	0.798412	0.797834	0.805418	2077.0
micro avg	0.804718	0.804718	0.804718	2077.0

weighted avg 0.783695 0.772472 0.804718 2077.0

The result of Model Number:Dataset Number:Featuregroup Number:Rescale of 4 : 1 : 4 : 1

	f1-score	precision	recall	support
AFIB	0.793206	0.800960	0.785786	875.0
GSVT	0.787147	0.781174	0.793470	691.0
SB	0.832891	0.824321	0.841714	1553.0
SR	0.806454	0.819423	0.794116	907.0
macro avg	0.804924	0.806470	0.803771	4026.0
micro avg	0.810555	0.810555	0.810555	4026.0
weighted avg	0.810459	0.810735	0.810555	4026.0

The result of Model Number:Dataset Number:Featuregroup Number:Rescale of 4 : 1 : 5 : 0

	f1-score	precision	recall	support
AFIB	0.839272	0.857968	0.821615	204.0
GSVT	0.849626	0.837183	0.862799	300.0
SB	0.868826	0.860191	0.877673	892.0
SR	0.852799	0.865048	0.840918	681.0
macro avg	0.852631	0.855098	0.850751	2077.0
micro avg	0.857968	0.857968	0.857968	2077.0
weighted avg	0.857895	0.858242	0.857968	2077.0

The result of Model Number:Dataset Number:Featuregroup Number:Rescale of 4 : 1 : 5 : 1

	f1-score	precision	recall	support
AFIB	0.665517	0.672912	0.658624	204.0
GSVT	0.686132	0.682226	0.690282	300.0
SB	0.699782	0.698844	0.700723	892.0
SR	0.693829	0.694758	0.692911	681.0
macro avg	0.686315	0.687185	0.685635	2077.0
micro avg	0.692519	0.692519	0.692519	2077.0
weighted avg	0.692493	0.692557	0.692519	2077.0

The result of Model Number:Dataset Number:Featuregroup Number:Rescale of 4 : 1 : 6 : 0

	f1-score	precision	recall	support
AFIB	0.846988	0.865829	0.829131	204.0
GSVT	0.883074	0.877587	0.888665	300.0
SB	0.902244	0.900344	0.904163	892.0
SR	0.892634	0.892003	0.893294	681.0
macro avg	0.881235	0.883940	0.878813	2077.0
micro avg	0.890991	0.890991	0.890991	2077.0
weighted avg	0.890897	0.890932	0.890991	2077.0

The result of Model Number:Dataset Number:Featuregroup Number:Rescale of 4 : 1 : 6 : 1

	f1-score	precision	recall	support
AFIB	0.819815	0.835701	0.804890	204.0
GSVT	0.851606	0.844485	0.859005	300.0
SB	0.867496	0.865755	0.869246	892.0
SR	0.859224	0.860114	0.858340	681.0

macro avg 0.849535 0.851514 0.847870 2077.0
micro avg 0.857870 0.857870 0.857870 2077.0
weighted avg 0.857806 0.857882 0.857870 2077.0
The result of Model Number:Dataset Number:Featuregroup Number:Rescale of 4 : 1 : 7 : 0

	f1-score	precision	recall	support
AFIB	0.758591	0.762350	0.754950	875.0
GSVT	0.766144	0.767248	0.765159	691.0
SB	0.806470	0.802728	0.810255	1553.0
SR	0.791624	0.793606	0.789695	907.0
macro avg	0.780707	0.781483	0.780015	4026.0
micro avg	0.785863	0.785863	0.785863	4026.0
weighted avg	0.785798	0.785808	0.785863	4026.0

The result of Model Number:Dataset Number:Featuregroup Number:Rescale of 4 : 1 : 7 : 1

	f1-score	precision	recall	support
AFIB	0.834841	0.880254	0.794097	204.0
GSVT	0.876429	0.863173	0.890133	300.0
SB	0.897256	0.891419	0.903183	892.0
SR	0.886186	0.886985	0.885410	681.0
macro avg	0.873678	0.880458	0.868206	2077.0
micro avg	0.884757	0.884757	0.884757	2077.0
weighted avg	0.884488	0.884789	0.884757	2077.0

The result of Model Number:Dataset Number:Featuregroup Number:Rescale of 4 : 1 : 8 : 0

	f1-score	precision	recall	support
AFIB	0.824715	0.829241	0.820290	875.0
GSVT	0.825868	0.815427	0.836710	691.0
SB	0.868543	0.865058	0.872064	1553.0
SR	0.849898	0.860216	0.839878	907.0
macro avg	0.842256	0.842485	0.842236	4026.0
micro avg	0.847493	0.847493	0.847493	4026.0
weighted avg	0.847493	0.847664	0.847493	4026.0

The result of Model Number:Dataset Number:Featuregroup Number:Rescale of 4 : 1 : 8 : 1

	f1-score	precision	recall	support
AFIB	0.817022	0.820895	0.813190	875.0
GSVT	0.827157	0.823254	0.831115	691.0
SB	0.872457	0.870616	0.874311	1553.0
SR	0.855924	0.858321	0.853561	907.0
macro avg	0.843140	0.843271	0.843044	4026.0
micro avg	0.848939	0.848939	0.848939	4026.0
weighted avg	0.848909	0.848911	0.848939	4026.0

The result of Model Number:Dataset Number:Featuregroup Number:Rescale of 4 : 1 : 9 : 0

	f1-score	precision	recall	support
AFIB	0.731597	0.736380	0.726920	204.0
GSVT	0.749926	0.748199	0.751671	300.0

SB 0.763313 0.762211 0.764423 892.0
SR 0.757297 0.758088 0.756521 681.0
macro avg 0.750533 0.751220 0.749884 2077.0
micro avg 0.756307 0.756307 0.756307 2077.0
weighted avg 0.756292 0.756299 0.756307 2077.0
The result of Model Number:Dataset Number:Featuregroup Number:Rescale of 4 : 1 : 9 : 1
f1-score precision recall support
AFIB 0.699335 0.716076 0.684384 204.0
GSVT 0.719929 0.709785 0.730668 300.0
SB 0.738635 0.731131 0.746329 892.0
SR 0.720981 0.731391 0.710920 681.0
macro avg 0.719720 0.722096 0.718075 2077.0
micro avg 0.726373 0.726373 0.726373 2077.0
weighted avg 0.726285 0.726654 0.726373 2077.0
The result of Model Number:Dataset Number:Featuregroup Number:Rescale of 4 : 1 : 10 :
0
f1-score precision recall support
AFIB 0.681167 0.754744 0.620786 204.0
GSVT 0.789618 0.776776 0.803016 300.0
SB 0.842079 0.838245 0.845949 892.0
SR 0.821732 0.811276 0.832474 681.0
macro avg 0.783649 0.795260 0.775556 2077.0
micro avg 0.813215 0.813215 0.813215 2077.0
weighted avg 0.812026 0.812323 0.813215 2077.0
The result of Model Number:Dataset Number:Featuregroup Number:Rescale of 4 : 1 : 10 :
1
f1-score precision recall support
AFIB 0.844401 0.849018 0.839861 875.0
GSVT 0.840377 0.833291 0.847668 691.0
SB 0.883866 0.875496 0.892402 1553.0
SR 0.850971 0.866845 0.835680 907.0
macro avg 0.854904 0.856163 0.853903 4026.0
micro avg 0.860526 0.860526 0.860526 4026.0
weighted avg 0.860414 0.860549 0.860526 4026.0
The result of Model Number:Dataset Number:Featuregroup Number:Rescale of 5 : 0 : 0 : 0
f1-score precision recall support
AFIB 0.748703 0.748028 0.749451 875.0
GSVT 0.761673 0.754699 0.768837 691.0
SB 0.799285 0.792258 0.806439 1553.0
SR 0.761656 0.780485 0.743780 907.0
macro avg 0.767829 0.768868 0.767127 4026.0
micro avg 0.773483 0.773483 0.773483 4026.0
weighted avg 0.773359 0.773547 0.773483 4026.0

The result of Model Number:Dataset Number:Featuregroup Number:Rescale of 5 : 0 : 0 : 1

	f1-score	precision	recall	support
AFIB	0.703666	0.694436	0.713177	875.0
GSVT	0.714439	0.722380	0.706683	691.0
SB	0.757755	0.755966	0.759556	1553.0
SR	0.738783	0.745432	0.732262	907.0
macro avg	0.728661	0.729554	0.727919	4026.0
micro avg	0.734252	0.734252	0.734252	4026.0
weighted avg	0.734291	0.734456	0.734252	4026.0

The result of Model Number:Dataset Number:Featuregroup Number:Rescale of 5 : 0 : 1 : 0

	f1-score	precision	recall	support
AFIB	0.653742	0.722867	0.597023	204.0
GSVT	0.752215	0.743510	0.761271	300.0
SB	0.795301	0.792031	0.798600	892.0
SR	0.778222	0.766695	0.790109	681.0
macro avg	0.744870	0.756276	0.736751	2077.0
micro avg	0.770626	0.770626	0.770626	2077.0
weighted avg	0.769574	0.769922	0.770626	2077.0

The result of Model Number:Dataset Number:Featuregroup Number:Rescale of 5 : 0 : 1 : 1

	f1-score	precision	recall	support
AFIB	0.837360	0.826943	0.848136	875.0
GSVT	0.863576	0.874390	0.853112	691.0
SB	0.921315	0.920317	0.922322	1553.0
SR	0.896466	0.901063	0.891920	907.0
macro avg	0.879679	0.880678	0.878872	4026.0
micro avg	0.887471	0.887471	0.887471	4026.0
weighted avg	0.887560	0.887803	0.887471	4026.0

The result of Model Number:Dataset Number:Featuregroup Number:Rescale of 5 : 0 : 2 : 0

	f1-score	precision	recall	support
AFIB	0.687223	0.688414	0.686047	875.0
GSVT	0.695556	0.687563	0.703793	691.0
SB	0.732444	0.723612	0.741497	1553.0
SR	0.697629	0.718767	0.677715	907.0
macro avg	0.703213	0.704589	0.702263	4026.0
micro avg	0.708605	0.708605	0.708605	4026.0
weighted avg	0.708441	0.708684	0.708605	4026.0

The result of Model Number:Dataset Number:Featuregroup Number:Rescale of 5 : 0 : 2 : 1

	f1-score	precision	recall	support
AFIB	0.640616	0.690546	0.597717	204.0
GSVT	0.660152	0.671327	0.649371	300.0
SB	0.699637	0.693197	0.706218	892.0
SR	0.671790	0.662048	0.681854	681.0
macro avg	0.668049	0.679280	0.658790	2077.0

	f1-score	precision	recall	support
micro avg	0.679362	0.679362	0.679362	2077.0
weighted avg	0.679006	0.679565	0.679362	2077.0

The result of Model Number:Dataset Number:Featuregroup Number:Rescale of 5 : 0 : 3 : 0

	f1-score	precision	recall	support
AFIB	0.903176	0.921373	0.886264	204.0
GSVT	0.932157	0.924670	0.939945	300.0
SB	0.952337	0.951378	0.953298	892.0
SR	0.943656	0.943114	0.944209	681.0
macro avg	0.932831	0.935134	0.930929	2077.0
micro avg	0.941805	0.941805	0.941805	2077.0
weighted avg	0.941747	0.941864	0.941805	2077.0

The result of Model Number:Dataset Number:Featuregroup Number:Rescale of 5 : 0 : 3 : 1

	f1-score	precision	recall	support
AFIB	0.786806	0.777526	0.796351	875.0
GSVT	0.800135	0.805198	0.795164	691.0
SB	0.853098	0.851951	0.854252	1553.0
SR	0.832140	0.840023	0.824431	907.0
macro avg	0.818045	0.818674	0.817549	4026.0
micro avg	0.824808	0.824808	0.824808	4026.0
weighted avg	0.824879	0.825064	0.824808	4026.0

The result of Model Number:Dataset Number:Featuregroup Number:Rescale of 5 : 0 : 4 : 0

	f1-score	precision	recall	support
AFIB	0.585311	0.581625	0.659312	204.0
GSVT	0.713504	0.709834	0.717332	300.0
SB	0.733819	0.730328	0.737353	892.0
SR	0.575599	0.577495	0.574062	681.0
macro avg	0.652058	0.649820	0.672015	2077.0
micro avg	0.673257	0.673257	0.673257	2077.0
weighted avg	0.664422	0.662652	0.673257	2077.0

The result of Model Number:Dataset Number:Featuregroup Number:Rescale of 5 : 0 : 4 : 1

	f1-score	precision	recall	support
AFIB	0.680706	0.675291	0.686273	875.0
GSVT	0.653716	0.641286	0.666691	691.0
SB	0.686098	0.633328	0.748473	1553.0
SR	0.525361	0.671751	0.431455	907.0
macro avg	0.636470	0.655414	0.633223	4026.0
micro avg	0.649498	0.649498	0.649498	4026.0
weighted avg	0.643157	0.652470	0.649498	4026.0

The result of Model Number:Dataset Number:Featuregroup Number:Rescale of 5 : 0 : 5 : 0

	f1-score	precision	recall	support
AFIB	0.833369	0.850678	0.816943	875.0
GSVT	0.812701	0.784474	0.879041	691.0
SB	0.957895	0.953576	0.962270	1553.0

SR 0.728237 0.716480 0.740453 907.0
macro avg 0.833051 0.826302 0.849677 4026.0
micro avg 0.866428 0.866428 0.866428 4026.0
weighted avg 0.854172 0.848774 0.866428 4026.0
The result of Model Number:Dataset Number:Featuregroup Number:Rescale of 5 : 0 : 5 : 1

	f1-score	precision	recall	support
AFIB	0.683796	0.709148	0.660292	204.0
GSVT	0.704282	0.695476	0.713403	300.0
SB	0.722141	0.715552	0.728859	892.0
SR	0.703156	0.708612	0.697789	681.0
macro avg	0.703344	0.707197	0.700086	2077.0
micro avg	0.709705	0.709705	0.709705	2077.0
weighted avg	0.709570	0.709748	0.709705	2077.0

The result of Model Number:Dataset Number:Featuregroup Number:Rescale of 5 : 0 : 6 : 0

	f1-score	precision	recall	support
AFIB	0.733804	0.724591	0.743467	875.0
GSVT	0.680824	0.701437	0.663665	691.0
SB	0.733016	0.678934	0.798397	1553.0
SR	0.569943	0.697567	0.486002	907.0
macro avg	0.679397	0.700632	0.672883	4026.0
micro avg	0.692956	0.692956	0.692956	4026.0
weighted avg	0.687491	0.696917	0.692956	4026.0

The result of Model Number:Dataset Number:Featuregroup Number:Rescale of 5 : 0 : 6 : 1

	f1-score	precision	recall	support
AFIB	0.614883	0.681141	0.560867	204.0
GSVT	0.724787	0.714628	0.735426	300.0
SB	0.769548	0.767071	0.772050	892.0
SR	0.749846	0.738440	0.761628	681.0
macro avg	0.714766	0.725320	0.707493	2077.0
micro avg	0.742601	0.742601	0.742601	2077.0
weighted avg	0.741432	0.741669	0.742601	2077.0

The result of Model Number:Dataset Number:Featuregroup Number:Rescale of 5 : 0 : 7 : 0

	f1-score	precision	recall	support
AFIB	0.721700	0.800939	0.656901	204.0
GSVT	0.842682	0.832147	0.853653	300.0
SB	0.896572	0.891997	0.901195	892.0
SR	0.875569	0.863328	0.888166	681.0
macro avg	0.834131	0.847103	0.824979	2077.0
micro avg	0.866062	0.866062	0.866062	2077.0
weighted avg	0.864726	0.865009	0.866062	2077.0

The result of Model Number:Dataset Number:Featuregroup Number:Rescale of 5 : 0 : 7 : 1

	f1-score	precision	recall	support
AFIB	0.780722	0.798558	0.763863	204.0

GSVT 0.816189 0.811108 0.821354 300.0
SB 0.833816 0.831230 0.836430 892.0
SR 0.824267 0.824648 0.823908 681.0
macro avg 0.813748 0.816386 0.811389 2077.0
micro avg 0.823020 0.823020 0.823020 2077.0
weighted avg 0.822924 0.822957 0.823020 2077.0
The result of Model Number:Dataset Number:Featuregroup Number:Rescale of 5 : 0 : 8 : 0
f1-score precision recall support
AFIB 0.896044 0.923642 0.870227 204.0
GSVT 0.918903 0.907654 0.930535 300.0
SB 0.943862 0.938254 0.949569 892.0
SR 0.927953 0.932529 0.923457 681.0
macro avg 0.921691 0.925520 0.918447 2077.0
micro avg 0.930466 0.930466 0.930466 2077.0
weighted avg 0.930344 0.930522 0.930466 2077.0
The result of Model Number:Dataset Number:Featuregroup Number:Rescale of 5 : 0 : 8 : 1
f1-score precision recall support
AFIB 0.682942 0.687819 0.678257 204.0
GSVT 0.706823 0.706367 0.707292 300.0
SB 0.720965 0.719365 0.722579 892.0
SR 0.714520 0.715384 0.713680 681.0
macro avg 0.706312 0.707234 0.705452 2077.0
micro avg 0.713100 0.713100 0.713100 2077.0
weighted avg 0.713074 0.713084 0.713100 2077.0
The result of Model Number:Dataset Number:Featuregroup Number:Rescale of 5 : 0 : 9 : 0
f1-score precision recall support
AFIB 0.683982 0.673759 0.694545 875.0
GSVT 0.697096 0.703058 0.691246 691.0
SB 0.744831 0.742266 0.747418 1553.0
SR 0.723030 0.733814 0.712583 907.0
macro avg 0.712235 0.713224 0.711448 4026.0
micro avg 0.718438 0.718438 0.718438 4026.0
weighted avg 0.718502 0.718743 0.718438 4026.0
The result of Model Number:Dataset Number:Featuregroup Number:Rescale of 5 : 0 : 9 : 1
f1-score precision recall support
AFIB 0.634668 0.625852 0.643797 875.0
GSVT 0.657348 0.666117 0.648892 691.0
SB 0.698317 0.696886 0.699756 1553.0
SR 0.680142 0.685397 0.674967 907.0
macro avg 0.667619 0.668563 0.666853 4026.0
micro avg 0.673279 0.673279 0.673279 4026.0
weighted avg 0.673357 0.673579 0.673279 4026.0

The result of Model Number:Dataset Number:Featuregroup Number:Rescale of 5 : 0 : 10 : 0

	f1-score	precision	recall	support
AFIB	0.845734	0.881981	0.812670	204.0
GSVT	0.874154	0.860946	0.887829	300.0
SB	0.891492	0.882675	0.900489	892.0
SR	0.867339	0.874759	0.860076	681.0
macro avg	0.869680	0.875090	0.865266	2077.0
micro avg	0.876784	0.876784	0.876784	2077.0
weighted avg	0.876574	0.876873	0.876784	2077.0

The result of Model Number:Dataset Number:Featuregroup Number:Rescale of 5 : 0 : 10 : 1

	f1-score	precision	recall	support
AFIB	0.571556	0.623321	0.528682	204.0
GSVT	0.663395	0.655815	0.671357	300.0
SB	0.703491	0.701690	0.705305	892.0
SR	0.686267	0.676974	0.695838	681.0
macro avg	0.656177	0.664450	0.650296	2077.0
micro avg	0.679950	0.679950	0.679950	2077.0
weighted avg	0.679094	0.679263	0.679950	2077.0

The result of Model Number:Dataset Number:Featuregroup Number:Rescale of 5 : 1 : 0 : 0

	f1-score	precision	recall	support
AFIB	0.748401	0.765341	0.732291	204.0
GSVT	0.768317	0.764556	0.772150	300.0
SB	0.784035	0.781771	0.786318	892.0
SR	0.778998	0.778661	0.779346	681.0
macro avg	0.769938	0.772582	0.767526	2077.0
micro avg	0.776679	0.776679	0.776679	2077.0
weighted avg	0.776613	0.776651	0.776679	2077.0

The result of Model Number:Dataset Number:Featuregroup Number:Rescale of 5 : 1 : 0 : 1

	f1-score	precision	recall	support
AFIB	0.770215	0.779220	0.761563	204.0
GSVT	0.791403	0.787764	0.795097	300.0
SB	0.802608	0.800112	0.805125	892.0
SR	0.795695	0.797951	0.793487	681.0
macro avg	0.789980	0.791262	0.788818	2077.0
micro avg	0.795582	0.795582	0.795582	2077.0
weighted avg	0.795541	0.795568	0.795582	2077.0

The result of Model Number:Dataset Number:Featuregroup Number:Rescale of 5 : 1 : 1 : 0

	f1-score	precision	recall	support
AFIB	0.717979	0.717621	0.718369	875.0
GSVT	0.726910	0.731773	0.722169	691.0
SB	0.770624	0.768853	0.772408	1553.0

SR 0.748304 0.748126 0.748611 907.0
macro avg 0.740954 0.741594 0.740389 4026.0
micro avg 0.746679 0.746679 0.746679 4026.0
weighted avg 0.746651 0.746685 0.746679 4026.0
The result of Model Number:Dataset Number:Featuregroup Number:Rescale of 5 : 1 : 1 : 1
f1-score precision recall support
AFIB 0.754658 0.744175 0.765512 875.0
GSVT 0.781625 0.792053 0.771571 691.0
SB 0.830340 0.828639 0.832051 1553.0
SR 0.808729 0.814978 0.802576 907.0
macro avg 0.793838 0.794961 0.792927 4026.0
micro avg 0.800569 0.800569 0.800569 4026.0
weighted avg 0.800661 0.800924 0.800569 4026.0
The result of Model Number:Dataset Number:Featuregroup Number:Rescale of 5 : 1 : 2 : 0
f1-score precision recall support
AFIB 0.695303 0.765921 0.637275 204.0
GSVT 0.642108 0.686510 0.603401 300.0
SB 0.699065 0.653188 0.751885 892.0
SR 0.605053 0.636857 0.576439 681.0
macro avg 0.660382 0.685619 0.642250 2077.0
micro avg 0.661656 0.661656 0.661656 2077.0
weighted avg 0.659644 0.663719 0.661656 2077.0
The result of Model Number:Dataset Number:Featuregroup Number:Rescale of 5 : 1 : 2 : 1
f1-score precision recall support
AFIB 0.838216 0.881520 0.799216 204.0
GSVT 0.878941 0.870349 0.887722 300.0
SB 0.894438 0.887243 0.901756 892.0
SR 0.883390 0.884465 0.882353 681.0
macro avg 0.873746 0.880894 0.867762 2077.0
micro avg 0.883296 0.883296 0.883296 2077.0
weighted avg 0.883055 0.883330 0.883296 2077.0
The result of Model Number:Dataset Number:Featuregroup Number:Rescale of 5 : 1 : 3 : 0
f1-score precision recall support
AFIB 0.796129 0.794118 0.798275 875.0
GSVT 0.811661 0.807199 0.816217 691.0
SB 0.849874 0.844750 0.855072 1553.0
SR 0.824816 0.839703 0.810463 907.0
macro avg 0.820620 0.821442 0.820007 4026.0
micro avg 0.826009 0.826009 0.826009 4026.0
weighted avg 0.825989 0.826164 0.826009 4026.0
The result of Model Number:Dataset Number:Featuregroup Number:Rescale of 5 : 1 : 3 : 1
f1-score precision recall support
AFIB 0.767891 0.776246 0.759937 204.0

GSVT	0.793877	0.790566	0.797344	300.0
SB	0.810145	0.809420	0.810871	892.0
SR	0.802489	0.802496	0.802489	681.0
macro avg	0.793600	0.794682	0.792660	2077.0
micro avg	0.801166	0.801166	0.801166	2077.0
weighted avg	0.801135	0.801168	0.801166	2077.0

The result of Model Number:Dataset Number:Featuregroup Number:Rescale of 5 : 1 : 4 : 0

	f1-score	precision	recall	support
AFIB	0.846165	0.857467	0.835395	204.0
GSVT	0.861113	0.855867	0.866555	300.0
SB	0.881870	0.881088	0.882659	892.0
SR	0.875354	0.875487	0.875227	681.0
macro avg	0.866125	0.867477	0.864959	2077.0
micro avg	0.873254	0.873254	0.873254	2077.0
weighted avg	0.873229	0.873289	0.873254	2077.0

The result of Model Number:Dataset Number:Featuregroup Number:Rescale of 5 : 1 : 4 : 1

	f1-score	precision	recall	support
AFIB	0.830667	0.849451	0.812720	875.0
GSVT	0.880695	0.884444	0.877031	691.0
SB	0.956253	0.951746	0.960808	1553.0
SR	0.906036	0.892139	0.920412	907.0
macro avg	0.893413	0.894445	0.892743	4026.0
micro avg	0.905143	0.905143	0.905143	4026.0
weighted avg	0.904677	0.904533	0.905143	4026.0

The result of Model Number:Dataset Number:Featuregroup Number:Rescale of 5 : 1 : 5 : 0

	f1-score	precision	recall	support
AFIB	0.808471	0.828141	0.789912	204.0
GSVT	0.818634	0.807364	0.830331	300.0
SB	0.835164	0.826823	0.843680	892.0
SR	0.820582	0.831350	0.810112	681.0
macro avg	0.820712	0.823420	0.818509	2077.0
micro avg	0.825465	0.825465	0.825465	2077.0
weighted avg	0.825373	0.825626	0.825465	2077.0

The result of Model Number:Dataset Number:Featuregroup Number:Rescale of 5 : 1 : 5 : 1

	f1-score	precision	recall	support
AFIB	0.787408	0.797615	0.777668	204.0
GSVT	0.817875	0.817347	0.818429	300.0
SB	0.835109	0.833345	0.836885	892.0
SR	0.826710	0.826240	0.827197	681.0
macro avg	0.816775	0.818637	0.815045	2077.0
micro avg	0.825227	0.825227	0.825227	2077.0
weighted avg	0.825181	0.825195	0.825227	2077.0

The result of Model Number:Dataset Number:Featuregroup Number:Rescale of 5 : 1 : 6 : 0

	f1-score	precision	recall	support
AFIB	0.709609	0.704968	0.714372	875.0
GSVT	0.730700	0.736917	0.724684	691.0
SB	0.777928	0.776234	0.779632	1553.0
SR	0.758970	0.762017	0.755958	907.0
macro avg	0.744302	0.745034	0.743661	4026.0
micro avg	0.750684	0.750684	0.750684	4026.0
weighted avg	0.750703	0.750794	0.750684	4026.0

The result of Model Number:Dataset Number:Featuregroup Number:Rescale of 5 : 1 : 6 : 1

	f1-score	precision	recall	support
AFIB	0.745530	0.761232	0.730580	204.0
GSVT	0.768710	0.761687	0.775881	300.0
SB	0.781264	0.779522	0.783016	892.0
SR	0.771979	0.772787	0.771183	681.0
macro avg	0.766871	0.768807	0.765165	2077.0
micro avg	0.772955	0.772955	0.772955	2077.0
weighted avg	0.772897	0.772941	0.772955	2077.0

The result of Model Number:Dataset Number:Featuregroup Number:Rescale of 5 : 1 : 7 : 0

	f1-score	precision	recall	support
AFIB	0.771370	0.798149	0.746434	204.0
GSVT	0.786160	0.777554	0.795056	300.0
SB	0.807089	0.801293	0.812970	892.0
SR	0.792674	0.796572	0.788829	681.0
macro avg	0.789323	0.793392	0.785822	2077.0
micro avg	0.795932	0.795932	0.795932	2077.0
weighted avg	0.795831	0.796008	0.795932	2077.0

The result of Model Number:Dataset Number:Featuregroup Number:Rescale of 5 : 1 : 7 : 1

	f1-score	precision	recall	support
AFIB	0.663398	0.650182	0.677196	875.0
GSVT	0.539947	0.502122	0.676808	691.0
SB	0.438107	0.438862	0.437356	1553.0
SR	0.710228	0.716482	0.704112	907.0
macro avg	0.587920	0.576912	0.623868	4026.0
micro avg	0.590677	0.590677	0.590677	4026.0
weighted avg	0.565856	0.558191	0.590677	4026.0

The result of Model Number:Dataset Number:Featuregroup Number:Rescale of 5 : 1 : 8 : 0

	f1-score	precision	recall	support
AFIB	0.840340	0.843231	0.837574	875.0
GSVT	0.856293	0.839209	0.874242	691.0
SB	0.906455	0.901553	0.911416	1553.0
SR	0.871395	0.891656	0.852086	907.0
macro avg	0.868621	0.868912	0.868830	4026.0
micro avg	0.875621	0.875621	0.875621	4026.0

weighted avg 0.875578 0.875948 0.875621 4026.0
The result of Model Number:Dataset Number:Featuregroup Number:Rescale of 5 : 1 : 8 : 1

	f1-score	precision	recall	support
AFIB	0.668221	0.665504	0.670978	875.0
GSVT	0.677989	0.682087	0.673965	691.0
SB	0.715067	0.714196	0.715942	1553.0
SR	0.700149	0.701258	0.699064	907.0
macro avg	0.690357	0.690761	0.689987	4026.0
micro avg	0.695163	0.695163	0.695163	4026.0
weighted avg	0.695161	0.695188	0.695163	4026.0

The result of Model Number:Dataset Number:Featuregroup Number:Rescale of 5 : 1 : 9 : 0

	f1-score	precision	recall	support
AFIB	0.912816	0.944739	0.883402	204.0
GSVT	0.951366	0.945567	0.957367	300.0
SB	0.970902	0.966913	0.974927	892.0
SR	0.961926	0.960523	0.963339	681.0
macro avg	0.949252	0.954435	0.944759	2077.0
micro avg	0.959602	0.959602	0.959602	2077.0
weighted avg	0.959432	0.959557	0.959602	2077.0

The result of Model Number:Dataset Number:Featuregroup Number:Rescale of 5 : 1 : 9 : 1

	f1-score	precision	recall	support
AFIB	0.842914	0.876936	0.811500	204.0
GSVT	0.874281	0.860079	0.889111	300.0
SB	0.896899	0.889689	0.904239	892.0
SR	0.872769	0.879054	0.866620	681.0
macro avg	0.871716	0.876439	0.867868	2077.0
micro avg	0.880611	0.880611	0.880611	2077.0
weighted avg	0.880418	0.880672	0.880611	2077.0

The result of Model Number:Dataset Number:Featuregroup Number:Rescale of 5 : 1 : 10 : 0

	f1-score	precision	recall	support
AFIB	0.732619	0.774295	0.695685	204.0
GSVT	0.774212	0.765687	0.782969	300.0
SB	0.785729	0.777911	0.793733	892.0
SR	0.776601	0.779256	0.774087	681.0
macro avg	0.767290	0.774287	0.761619	2077.0
micro avg	0.776107	0.776107	0.776107	2077.0
weighted avg	0.775857	0.776231	0.776107	2077.0

The result of Model Number:Dataset Number:Featuregroup Number:Rescale of 5 : 1 : 10 : 1

	f1-score	precision	recall	support
AFIB	0.815416	0.830375	0.801050	204.0
GSVT	0.826171	0.817833	0.834697	300.0

SB 0.846521 0.842115 0.850980 892.0
SR 0.834997 0.840229 0.829848 681.0
macro avg 0.830776 0.832638 0.829143 2077.0
micro avg 0.836795 0.836795 0.836795 2077.0
weighted avg 0.836748 0.836836 0.836795 2077.0
The result of Model Number:Dataset Number:Featuregroup Number:Rescale of 6 : 0 : 0 : 0
f1-score precision recall support
AFIB 0.790978 0.799397 0.783173 204.0
GSVT 0.814224 0.808798 0.819960 300.0
SB 0.831430 0.830687 0.832175 892.0
SR 0.824419 0.825519 0.823328 681.0
macro avg 0.815263 0.816100 0.814659 2077.0
micro avg 0.822697 0.822697 0.822697 2077.0
weighted avg 0.822673 0.822758 0.822697 2077.0
The result of Model Number:Dataset Number:Featuregroup Number:Rescale of 6 : 0 : 0 : 1
f1-score precision recall support
AFIB 0.751928 0.731073 0.776375 875.0
GSVT 0.761744 0.793830 0.736838 691.0
SB 0.836802 0.836380 0.837237 1553.0
SR 0.815809 0.820915 0.810773 907.0
macro avg 0.791571 0.795549 0.790306 4026.0
micro avg 0.800815 0.800815 0.800815 4026.0
weighted avg 0.800744 0.802706 0.800815 4026.0
The result of Model Number:Dataset Number:Featuregroup Number:Rescale of 6 : 0 : 1 : 0
f1-score precision recall support
AFIB 0.592969 0.570300 0.630269 875.0
GSVT 0.614416 0.593211 0.643178 691.0
SB 0.684574 0.672490 0.697851 1553.0
SR 0.579818 0.697198 0.500595 907.0
macro avg 0.617944 0.633300 0.617973 4026.0
micro avg 0.629340 0.629340 0.629340 4026.0
weighted avg 0.629023 0.642240 0.629340 4026.0
The result of Model Number:Dataset Number:Featuregroup Number:Rescale of 6 : 0 : 1 : 1
f1-score precision recall support
AFIB 0.795739 0.829843 0.764630 204.0
GSVT 0.822479 0.810052 0.835345 300.0
SB 0.838792 0.830497 0.847257 892.0
SR 0.816067 0.823049 0.809234 681.0
macro avg 0.818269 0.823360 0.814117 2077.0
micro avg 0.824954 0.824954 0.824954 2077.0
weighted avg 0.824756 0.825038 0.824954 2077.0
The result of Model Number:Dataset Number:Featuregroup Number:Rescale of 6 : 0 : 2 : 0
f1-score precision recall support

AFIB 0.642554 0.644765 0.640440 875.0
GSVT 0.654753 0.641690 0.668477 691.0
SB 0.693108 0.689360 0.696901 1553.0
SR 0.666300 0.681792 0.651536 907.0
macro avg 0.664179 0.664402 0.664339 4026.0
micro avg 0.669531 0.669531 0.669531 4026.0
weighted avg 0.669498 0.669781 0.669531 4026.0
The result of Model Number:Dataset Number:Featuregroup Number:Rescale of 6 : 0 : 2 : 1
f1-score precision recall support
AFIB 0.712561 0.707624 0.717710 875.0
GSVT 0.723554 0.726490 0.720758 691.0
SB 0.785057 0.776894 0.793398 1553.0
SR 0.745419 0.762764 0.728913 907.0
macro avg 0.741648 0.743443 0.740195 4026.0
micro avg 0.749953 0.749953 0.749953 4026.0
weighted avg 0.749815 0.750005 0.749953 4026.0
The result of Model Number:Dataset Number:Featuregroup Number:Rescale of 6 : 0 : 3 : 0
f1-score precision recall support
AFIB 0.798271 0.860488 0.744814 204.0
GSVT 0.822614 0.836539 0.809180 300.0
SB 0.871816 0.863792 0.880017 892.0
SR 0.837117 0.824977 0.849657 681.0
macro avg 0.832454 0.846449 0.820917 2077.0
micro avg 0.846552 0.846552 0.846552 2077.0
weighted avg 0.846109 0.846805 0.846552 2077.0
The result of Model Number:Dataset Number:Featuregroup Number:Rescale of 6 : 0 : 3 : 1
f1-score precision recall support
AFIB 0.676976 0.678112 0.693755 204.0
GSVT 0.670612 0.696635 0.647327 300.0
SB 0.711329 0.672319 0.755437 892.0
SR 0.616571 0.673472 0.570097 681.0
macro avg 0.668872 0.680135 0.666654 2077.0
micro avg 0.672994 0.672994 0.672994 2077.0
weighted avg 0.671005 0.676778 0.672994 2077.0
The result of Model Number:Dataset Number:Featuregroup Number:Rescale of 6 : 0 : 4 : 0
f1-score precision recall support
AFIB 0.794463 0.794841 0.794199 875.0
GSVT 0.815038 0.807983 0.822245 691.0
SB 0.870464 0.864120 0.876909 1553.0
SR 0.833276 0.849841 0.817388 907.0
macro avg 0.828310 0.829196 0.827685 4026.0
micro avg 0.836141 0.836141 0.836141 4026.0
weighted avg 0.836055 0.836211 0.836141 4026.0

The result of Model Number:Dataset Number:Featuregroup Number:Rescale of 6 : 0 : 4 : 1

	f1-score	precision	recall	support
AFIB	0.762566	0.753338	0.772057	875.0
GSVT	0.772889	0.780813	0.765183	691.0
SB	0.820886	0.818215	0.823581	1553.0
SR	0.802931	0.811181	0.794875	907.0
macro avg	0.789818	0.790887	0.788924	4026.0
micro avg	0.795893	0.795893	0.795893	4026.0
weighted avg	0.795928	0.796111	0.795893	4026.0

The result of Model Number:Dataset Number:Featuregroup Number:Rescale of 6 : 0 : 5 : 0

	f1-score	precision	recall	support
AFIB	0.781709	0.775690	0.787881	875.0
GSVT	0.791858	0.795534	0.788388	691.0
SB	0.848586	0.844072	0.853167	1553.0
SR	0.823584	0.835163	0.812412	907.0
macro avg	0.811434	0.812615	0.810462	4026.0
micro avg	0.818678	0.818678	0.818678	4026.0
weighted avg	0.818682	0.818872	0.818678	4026.0

The result of Model Number:Dataset Number:Featuregroup Number:Rescale of 6 : 0 : 5 : 1

	f1-score	precision	recall	support
AFIB	0.677643	0.682482	0.672994	204.0
GSVT	0.701338	0.700886	0.701804	300.0
SB	0.715370	0.713783	0.716972	892.0
SR	0.708976	0.709834	0.708142	681.0
macro avg	0.700832	0.701746	0.699978	2077.0
micro avg	0.707567	0.707567	0.707567	2077.0
weighted avg	0.707541	0.707551	0.707567	2077.0

The result of Model Number:Dataset Number:Featuregroup Number:Rescale of 6 : 0 : 6 : 0

	f1-score	precision	recall	support
AFIB	0.873658	0.869259	0.878140	875.0
GSVT	0.890675	0.894987	0.886418	691.0
SB	0.948623	0.946440	0.950820	1553.0
SR	0.924875	0.929823	0.919989	907.0
macro avg	0.909457	0.910127	0.908842	4026.0
micro avg	0.917024	0.917024	0.917024	4026.0
weighted avg	0.917034	0.917091	0.917024	4026.0

The result of Model Number:Dataset Number:Featuregroup Number:Rescale of 6 : 0 : 6 : 1

	f1-score	precision	recall	support
AFIB	0.791844	0.809319	0.775167	204.0
GSVT	0.809150	0.795732	0.823125	300.0
SB	0.824698	0.818247	0.831260	892.0
SR	0.804683	0.814369	0.795228	681.0
macro avg	0.807594	0.809417	0.806195	2077.0

micro avg 0.812761 0.812761 0.812761 2077.0
weighted avg 0.812663 0.812846 0.812761 2077.0
The result of Model Number:Dataset Number:Featuregroup Number:Rescale of 6 : 0 : 7 : 0
f1-score precision recall support
AFIB 0.769356 0.778803 0.760256 204.0
GSVT 0.784852 0.781500 0.788252 300.0
SB 0.794353 0.791969 0.796755 892.0
SR 0.787667 0.789542 0.785827 681.0
macro avg 0.784057 0.785453 0.782772 2077.0
micro avg 0.788359 0.788359 0.788359 2077.0
weighted avg 0.788333 0.788368 0.788359 2077.0
The result of Model Number:Dataset Number:Featuregroup Number:Rescale of 6 : 0 : 7 : 1
f1-score precision recall support
AFIB 0.780859 0.809687 0.754085 204.0
GSVT 0.797550 0.789364 0.806030 300.0
SB 0.819393 0.815572 0.823251 892.0
SR 0.806617 0.807122 0.806139 681.0
macro avg 0.801105 0.805436 0.797376 2077.0
micro avg 0.808360 0.808360 0.808360 2077.0
weighted avg 0.808264 0.808438 0.808360 2077.0
The result of Model Number:Dataset Number:Featuregroup Number:Rescale of 6 : 0 : 8 : 0
f1-score precision recall support
AFIB 0.666239 0.681421 0.651824 875.0
GSVT 0.708963 0.710364 0.707804 691.0
SB 0.775653 0.771408 0.779948 1553.0
SR 0.733108 0.724216 0.742324 907.0
macro avg 0.720991 0.721852 0.720475 4026.0
micro avg 0.731243 0.731243 0.731243 4026.0
weighted avg 0.730842 0.730741 0.731243 4026.0
The result of Model Number:Dataset Number:Featuregroup Number:Rescale of 6 : 0 : 8 : 1
f1-score precision recall support
AFIB 0.874898 0.877909 0.872019 875.0
GSVT 0.891508 0.873722 0.910195 691.0
SB 0.943733 0.938629 0.948897 1553.0
SR 0.907231 0.928325 0.887128 907.0
macro avg 0.904343 0.904646 0.904560 4026.0
micro avg 0.911631 0.911631 0.911631 4026.0
weighted avg 0.911586 0.911971 0.911631 4026.0
The result of Model Number:Dataset Number:Featuregroup Number:Rescale of 6 : 0 : 9 : 0
f1-score precision recall support
AFIB 0.625898 0.640161 0.612356 875.0
GSVT 0.666035 0.667351 0.664946 691.0
SB 0.728686 0.724698 0.732721 1553.0

SR 0.688718 0.680364 0.697376 907.0
macro avg 0.677334 0.678144 0.676850 4026.0
micro avg 0.686966 0.686966 0.686966 4026.0
weighted avg 0.686589 0.686495 0.686966 4026.0
The result of Model Number:Dataset Number:Featuregroup Number:Rescale of 6 : 0 : 9 : 1

	f1-score	precision	recall	support
AFIB	0.868790	0.873222	0.864431	875.0
GSVT	0.880780	0.876198	0.885487	691.0
SB	0.923195	0.922370	0.924028	1553.0
SR	0.906196	0.906935	0.905495	907.0
macro avg	0.894740	0.894681	0.894860	4026.0
micro avg	0.900285	0.900285	0.900285	4026.0
weighted avg	0.900261	0.900286	0.900285	4026.0

The result of Model Number:Dataset Number:Featuregroup Number:Rescale of 6 : 0 : 10 : 0

	f1-score	precision	recall	support
AFIB	0.771794	0.778025	0.765729	204.0
GSVT	0.789504	0.785632	0.793441	300.0
SB	0.804912	0.804019	0.805814	892.0
SR	0.798902	0.799964	0.797848	681.0
macro avg	0.791278	0.791910	0.790708	2077.0
micro avg	0.797478	0.797478	0.797478	2077.0
weighted avg	0.797463	0.797481	0.797478	2077.0

The result of Model Number:Dataset Number:Featuregroup Number:Rescale of 6 : 0 : 10 : 1

	f1-score	precision	recall	support
AFIB	0.659129	0.702075	0.621426	204.0
GSVT	0.688004	0.671093	0.705883	300.0
SB	0.708182	0.699085	0.717544	892.0
SR	0.689788	0.698024	0.681789	681.0
macro avg	0.686276	0.692569	0.681661	2077.0
micro avg	0.694696	0.694696	0.694696	2077.0
weighted avg	0.694419	0.694988	0.694696	2077.0

The result of Model Number:Dataset Number:Featuregroup Number:Rescale of 6 : 1 : 0 : 0

	f1-score	precision	recall	support
AFIB	0.665007	0.674421	0.656398	204.0
GSVT	0.678814	0.666316	0.691915	300.0
SB	0.693282	0.685617	0.701132	892.0
SR	0.678712	0.692535	0.665470	681.0
macro avg	0.678954	0.679722	0.678729	2077.0
micro avg	0.683714	0.683714	0.683714	2077.0
weighted avg	0.683638	0.683998	0.683714	2077.0

The result of Model Number:Dataset Number:Featuregroup Number:Rescale of 6 : 1 : 0 : 1

	f1-score	precision	recall	support
AFIB	0.920581	0.928013	0.913346	204.0
GSVT	0.941704	0.937087	0.946401	300.0
SB	0.960084	0.959019	0.961159	892.0
SR	0.952915	0.954182	0.951657	681.0
macro avg	0.943821	0.944575	0.943141	2077.0
micro avg	0.951216	0.951216	0.951216	2077.0
weighted avg	0.951198	0.951220	0.951216	2077.0

The result of Model Number:Dataset Number:Featuregroup Number:Rescale of 6 : 1 : 1 : 0

	f1-score	precision	recall	support
AFIB	0.869776	0.869724	0.869883	875.0
GSVT	0.871659	0.866946	0.876438	691.0
SB	0.913301	0.907224	0.919472	1553.0
SR	0.889050	0.903637	0.874960	907.0
macro avg	0.885947	0.886883	0.885188	4026.0
micro avg	0.891280	0.891280	0.891280	4026.0
weighted avg	0.891231	0.891353	0.891280	4026.0

The result of Model Number:Dataset Number:Featuregroup Number:Rescale of 6 : 1 : 1 : 1

	f1-score	precision	recall	support
AFIB	0.900914	0.929791	0.874045	204.0
GSVT	0.927866	0.911903	0.944535	300.0
SB	0.943917	0.934586	0.953439	892.0
SR	0.918679	0.930174	0.907483	681.0
macro avg	0.922844	0.926614	0.919876	2077.0
micro avg	0.929287	0.929287	0.929287	2077.0
weighted avg	0.929100	0.929392	0.929287	2077.0

The result of Model Number:Dataset Number:Featuregroup Number:Rescale of 6 : 1 : 2 : 0

	f1-score	precision	recall	support
AFIB	0.816554	0.861310	0.776762	204.0
GSVT	0.777532	0.804793	0.752154	300.0
SB	0.799768	0.768922	0.833206	892.0
SR	0.711816	0.731127	0.693538	681.0
macro avg	0.776418	0.791538	0.763915	2077.0
micro avg	0.770161	0.770161	0.770161	2077.0
weighted avg	0.769368	0.770785	0.770161	2077.0

The result of Model Number:Dataset Number:Featuregroup Number:Rescale of 6 : 1 : 2 : 1

	f1-score	precision	recall	support
AFIB	0.855631	0.854246	0.857096	875.0
GSVT	0.863471	0.866905	0.860108	691.0
SB	0.906795	0.903499	0.910122	1553.0
SR	0.891963	0.896475	0.887545	907.0
macro avg	0.879465	0.880281	0.878718	4026.0
micro avg	0.884927	0.884927	0.884927	4026.0

weighted avg	0.884898	0.884931	0.884927	4026.0

The result of Model Number:Dataset Number:Featuregroup Number:Rescale of 6 : 1 : 3 : 0

	f1-score	precision	recall	support
AFIB	0.703205	0.704386	0.720634	204.0
GSVT	0.696594	0.723627	0.672407	300.0
SB	0.738890	0.698368	0.784706	892.0
SR	0.640460	0.699565	0.592185	681.0
macro avg	0.694787	0.706486	0.692483	2077.0
micro avg	0.699070	0.699070	0.699070	2077.0
weighted avg	0.697003	0.703000	0.699070	2077.0

The result of Model Number:Dataset Number:Featuregroup Number:Rescale of 6 : 1 : 3 : 1

	f1-score	precision	recall	support
AFIB	0.593728	0.579763	0.625065	875.0
GSVT	0.570519	0.565259	0.583519	691.0
SB	0.618766	0.575198	0.670188	1553.0
SR	0.385808	0.483866	0.321294	907.0
macro avg	0.542205	0.551021	0.550016	4026.0
micro avg	0.566905	0.566905	0.566905	4026.0
weighted avg	0.552561	0.553908	0.566905	4026.0

The result of Model Number:Dataset Number:Featuregroup Number:Rescale of 6 : 1 : 4 : 0

	f1-score	precision	recall	support
AFIB	0.883944	0.891080	0.876997	204.0
GSVT	0.904226	0.899793	0.908736	300.0
SB	0.921874	0.920852	0.922907	892.0
SR	0.914990	0.916207	0.913783	681.0
macro avg	0.906259	0.906983	0.905606	2077.0
micro avg	0.913359	0.913359	0.913359	2077.0
weighted avg	0.913343	0.913363	0.913359	2077.0

The result of Model Number:Dataset Number:Featuregroup Number:Rescale of 6 : 1 : 4 : 1

	f1-score	precision	recall	support
AFIB	0.860816	0.843668	0.878720	875.0
GSVT	0.700628	0.651547	0.878217	691.0
SB	0.568482	0.569462	0.567507	1553.0
SR	0.921583	0.929698	0.913647	907.0
macro avg	0.762877	0.748594	0.809523	4026.0
micro avg	0.766454	0.766454	0.766454	4026.0
weighted avg	0.734247	0.724302	0.766454	4026.0

The result of Model Number:Dataset Number:Featuregroup Number:Rescale of 6 : 1 : 5 : 0

	f1-score	precision	recall	support
AFIB	0.762729	0.779993	0.746311	204.0
GSVT	0.783027	0.779193	0.786933	300.0
SB	0.799045	0.796738	0.801372	892.0
SR	0.793912	0.793568	0.794267	681.0

macro avg 0.784678 0.787373 0.782220 2077.0
micro avg 0.791549 0.791549 0.791549 2077.0
weighted avg 0.791481 0.791520 0.791549 2077.0
The result of Model Number:Dataset Number:Featuregroup Number:Rescale of 6 : 1 : 5 : 1

	f1-score	precision	recall	support
AFIB	0.782889	0.774238	0.791750	875.0
GSVT	0.796994	0.801902	0.792160	691.0
SB	0.848248	0.846836	0.849668	1553.0
SR	0.827593	0.835332	0.820018	907.0
macro avg	0.813931	0.814577	0.813399	4026.0
micro avg	0.820530	0.820530	0.820530	4026.0
weighted avg	0.820593	0.820754	0.820530	4026.0

The result of Model Number:Dataset Number:Featuregroup Number:Rescale of 6 : 1 : 6 : 0

	f1-score	precision	recall	support
AFIB	0.809233	0.826821	0.792599	204.0
GSVT	0.842638	0.837667	0.847691	300.0
SB	0.860741	0.858832	0.862668	892.0
SR	0.852112	0.851631	0.852615	681.0
macro avg	0.841181	0.843738	0.838893	2077.0
micro avg	0.850327	0.850327	0.850327	2077.0
weighted avg	0.850238	0.850270	0.850327	2077.0

The result of Model Number:Dataset Number:Featuregroup Number:Rescale of 6 : 1 : 6 : 1

	f1-score	precision	recall	support
AFIB	0.743474	0.760012	0.727799	204.0
GSVT	0.775150	0.770333	0.780057	300.0
SB	0.791976	0.790309	0.793661	892.0
SR	0.783541	0.782987	0.784121	681.0
macro avg	0.773535	0.775910	0.771410	2077.0
micro avg	0.782099	0.782099	0.782099	2077.0
weighted avg	0.782016	0.782047	0.782099	2077.0

The result of Model Number:Dataset Number:Featuregroup Number:Rescale of 6 : 1 : 7 : 0

	f1-score	precision	recall	support
AFIB	0.828156	0.844842	0.812649	204.0
GSVT	0.854729	0.847865	0.861871	300.0
SB	0.873233	0.872354	0.874115	892.0
SR	0.865273	0.864777	0.865781	681.0
macro avg	0.855348	0.857459	0.853604	2077.0
micro avg	0.863577	0.863577	0.863577	2077.0
weighted avg	0.863523	0.863630	0.863577	2077.0

The result of Model Number:Dataset Number:Featuregroup Number:Rescale of 6 : 1 : 7 : 1

	f1-score	precision	recall	support
AFIB	0.807856	0.825853	0.790860	204.0
GSVT	0.817823	0.805845	0.830503	300.0

SB	0.836304	0.827993	0.844820	892.0
SR	0.820877	0.832667	0.809441	681.0
macro avg	0.820715	0.823089	0.818906	2077.0
micro avg	0.825852	0.825852	0.825852	2077.0
weighted avg	0.825782	0.826116	0.825852	2077.0

The result of Model Number:Dataset Number:Featuregroup Number:Rescale of 6 : 1 : 8 : 0

	f1-score	precision	recall	support
AFIB	0.716833	0.720490	0.713237	875.0
GSVT	0.726726	0.722945	0.730610	691.0
SB	0.761722	0.761042	0.762410	1553.0
SR	0.747696	0.748306	0.747118	907.0
macro avg	0.738244	0.738196	0.738344	4026.0
micro avg	0.742820	0.742820	0.742820	4026.0
weighted avg	0.742800	0.742820	0.742820	4026.0

The result of Model Number:Dataset Number:Featuregroup Number:Rescale of 6 : 1 : 8 : 1

	f1-score	precision	recall	support
AFIB	0.704597	0.702316	0.706914	875.0
GSVT	0.712767	0.714982	0.710637	691.0
SB	0.752085	0.750448	0.753732	1553.0
SR	0.738216	0.741698	0.734793	907.0
macro avg	0.726916	0.727361	0.726519	4026.0
micro avg	0.731893	0.731893	0.731893	4026.0
weighted avg	0.731891	0.731929	0.731893	4026.0

The result of Model Number:Dataset Number:Featuregroup Number:Rescale of 6 : 1 : 9 : 0

	f1-score	precision	recall	support
AFIB	0.784163	0.783445	0.784976	875.0
GSVT	0.800236	0.798683	0.801878	691.0
SB	0.849154	0.847263	0.851067	1553.0
SR	0.825977	0.831366	0.820688	907.0
macro avg	0.814882	0.815189	0.814652	4026.0
micro avg	0.821417	0.821417	0.821417	4026.0
weighted avg	0.821412	0.821474	0.821417	4026.0

The result of Model Number:Dataset Number:Featuregroup Number:Rescale of 6 : 1 : 9 : 1

	f1-score	precision	recall	support
AFIB	0.910510	0.924938	0.896646	204.0
GSVT	0.933913	0.925966	0.942003	300.0
SB	0.950037	0.947920	0.952168	892.0
SR	0.941036	0.943128	0.938972	681.0
macro avg	0.933874	0.935488	0.932447	2077.0
micro avg	0.940919	0.940919	0.940919	2077.0
weighted avg	0.940875	0.940920	0.940919	2077.0

The result of Model Number:Dataset Number:Featuregroup Number:Rescale of 6 : 1 : 10 : 0

	f1-score	precision	recall	support
AFIB	0.749358	0.753843	0.744964	204.0
GSVT	0.769320	0.767033	0.771626	300.0
SB	0.781101	0.780143	0.782063	892.0
SR	0.775406	0.776321	0.774500	681.0
macro avg	0.768796	0.769335	0.768288	2077.0
micro avg	0.774432	0.774432	0.774432	2077.0
weighted avg	0.774414	0.774413	0.774432	2077.0

The result of Model Number:Dataset Number:Featuregroup Number:Rescale of 6 : 1 : 10 : 1

	f1-score	precision	recall	support
AFIB	0.664513	0.663224	0.665892	875.0
GSVT	0.676991	0.665146	0.689330	691.0
SB	0.725249	0.714958	0.735847	1553.0
SR	0.687002	0.717627	0.658912	907.0
macro avg	0.688438	0.690239	0.687495	4026.0
micro avg	0.695327	0.695327	0.695327	4026.0
weighted avg	0.695149	0.695766	0.695327	4026.0

The result of Model Number:Dataset Number:Featuregroup Number:Rescale of 7 : 0 : 0 : 0

	f1-score	precision	recall	support
AFIB	0.700450	0.691737	0.709464	875.0
GSVT	0.722380	0.731426	0.713627	691.0
SB	0.770679	0.769844	0.771521	1553.0
SR	0.749892	0.753738	0.746090	907.0
macro avg	0.735850	0.736686	0.735175	4026.0
micro avg	0.742368	0.742368	0.742368	4026.0
weighted avg	0.742443	0.742646	0.742368	4026.0

The result of Model Number:Dataset Number:Featuregroup Number:Rescale of 7 : 0 : 0 : 1

	f1-score	precision	recall	support
AFIB	0.687743	0.706331	0.670250	204.0
GSVT	0.693412	0.683204	0.704024	300.0
SB	0.706843	0.699324	0.714529	892.0
SR	0.693733	0.703265	0.684467	681.0
macro avg	0.695433	0.698031	0.693317	2077.0
micro avg	0.698806	0.698806	0.698806	2077.0
weighted avg	0.698729	0.698976	0.698806	2077.0

The result of Model Number:Dataset Number:Featuregroup Number:Rescale of 7 : 0 : 1 : 0

	f1-score	precision	recall	support
AFIB	0.712728	0.712831	0.712720	875.0
GSVT	0.725176	0.719165	0.731343	691.0
SB	0.759250	0.752682	0.765946	1553.0
SR	0.734231	0.750817	0.718387	907.0
macro avg	0.732846	0.733874	0.732099	4026.0

micro avg 0.737724 0.737724 0.737724 4026.0
weighted avg 0.737655 0.737848 0.737724 4026.0
The result of Model Number:Dataset Number:Featuregroup Number:Rescale of 7 : 0 : 1 : 1

	f1-score	precision	recall	support
AFIB	0.827086	0.825748	0.828502	875.0
GSVT	0.834664	0.837984	0.831414	691.0
SB	0.876543	0.873357	0.879759	1553.0
SR	0.862205	0.866567	0.857935	907.0
macro avg	0.850125	0.850914	0.849402	4026.0
micro avg	0.855405	0.855405	0.855405	4026.0
weighted avg	0.855376	0.855409	0.855405	4026.0

The result of Model Number:Dataset Number:Featuregroup Number:Rescale of 7 : 0 : 2 : 0

	f1-score	precision	recall	support
AFIB	0.844932	0.825239	0.865724	875.0
GSVT	0.811104	0.820020	0.802435	691.0
SB	0.853558	0.798440	0.916869	1553.0
SR	0.683140	0.820319	0.585633	907.0
macro avg	0.798184	0.816005	0.792665	4026.0
micro avg	0.811490	0.811490	0.811490	4026.0
weighted avg	0.806004	0.812898	0.811490	4026.0

The result of Model Number:Dataset Number:Featuregroup Number:Rescale of 7 : 0 : 2 : 1

	f1-score	precision	recall	support
AFIB	0.753228	0.751051	0.755488	875.0
GSVT	0.767189	0.766565	0.767867	691.0
SB	0.814866	0.811491	0.818276	1553.0
SR	0.790403	0.799086	0.781949	907.0
macro avg	0.781421	0.782048	0.780895	4026.0
micro avg	0.787794	0.787794	0.787794	4026.0
weighted avg	0.787776	0.787849	0.787794	4026.0

The result of Model Number:Dataset Number:Featuregroup Number:Rescale of 7 : 0 : 3 : 0

	f1-score	precision	recall	support
AFIB	0.852390	0.862356	0.842815	204.0
GSVT	0.875838	0.871811	0.879927	300.0
SB	0.888239	0.885477	0.891024	892.0
SR	0.880589	0.883085	0.878145	681.0
macro avg	0.874264	0.875682	0.872978	2077.0
micro avg	0.880464	0.880464	0.880464	2077.0
weighted avg	0.880419	0.880448	0.880464	2077.0

The result of Model Number:Dataset Number:Featuregroup Number:Rescale of 7 : 0 : 3 : 1

	f1-score	precision	recall	support
AFIB	0.836502	0.842137	0.831065	875.0
GSVT	0.851430	0.839604	0.863750	691.0
SB	0.907292	0.901079	0.913598	1553.0

SR	0.870524	0.885561	0.856037	907.0
macro avg	0.866437	0.867095	0.866113	4026.0
micro avg	0.874137	0.874137	0.874137	4026.0
weighted avg	0.874035	0.874222	0.874137	4026.0

The result of Model Number:Dataset Number:Featuregroup Number:Rescale of 7 : 0 : 4 : 0

	f1-score	precision	recall	support
AFIB	0.650921	0.667961	0.635024	204.0
GSVT	0.612471	0.630272	0.595982	300.0
SB	0.646288	0.623534	0.670778	892.0
SR	0.585781	0.604858	0.567989	681.0
macro avg	0.623865	0.631656	0.617443	2077.0
micro avg	0.622761	0.622761	0.622761	2077.0
weighted avg	0.622020	0.622747	0.622761	2077.0

The result of Model Number:Dataset Number:Featuregroup Number:Rescale of 7 : 0 : 4 : 1

	f1-score	precision	recall	support
AFIB	0.715559	0.696761	0.760355	875.0
GSVT	0.688361	0.651542	0.784717	691.0
SB	0.652257	0.648135	0.656452	1553.0
SR	0.633565	0.644481	0.623189	907.0
macro avg	0.672435	0.660230	0.706178	4026.0
micro avg	0.693555	0.693555	0.693555	4026.0
weighted avg	0.668001	0.658465	0.693555	4026.0

The result of Model Number:Dataset Number:Featuregroup Number:Rescale of 7 : 0 : 5 : 0

	f1-score	precision	recall	support
AFIB	0.848418	0.842541	0.854550	875.0
GSVT	0.861507	0.865004	0.858179	691.0
SB	0.934737	0.925018	0.944668	1553.0
SR	0.887541	0.908193	0.867889	907.0
macro avg	0.883051	0.885189	0.881321	4026.0
micro avg	0.892940	0.892940	0.892940	4026.0
weighted avg	0.892776	0.893002	0.892940	4026.0

The result of Model Number:Dataset Number:Featuregroup Number:Rescale of 7 : 0 : 5 : 1

	f1-score	precision	recall	support
AFIB	0.792983	0.812012	0.775186	204.0
GSVT	0.821179	0.816420	0.826094	300.0
SB	0.838717	0.835641	0.841819	892.0
SR	0.831429	0.832046	0.830823	681.0
macro avg	0.821077	0.824030	0.818480	2077.0
micro avg	0.829398	0.829398	0.829398	2077.0
weighted avg	0.829302	0.829365	0.829398	2077.0

The result of Model Number:Dataset Number:Featuregroup Number:Rescale of 7 : 0 : 6 : 0

	f1-score	precision	recall	support
AFIB	0.742650	0.741455	0.744152	875.0

GSVT	0.759965	0.756004	0.764012	691.0
SB	0.794583	0.787851	0.801437	1553.0
SR	0.764251	0.781241	0.748241	907.0
macro avg	0.765362	0.766638	0.764461	4026.0
micro avg	0.770579	0.770579	0.770579	4026.0
weighted avg	0.770521	0.770812	0.770579	4026.0

The result of Model Number:Dataset Number:Featuregroup Number:Rescale of 7 : 0 : 6 : 1

	f1-score	precision	recall	support
AFIB	0.711775	0.724984	0.699215	204.0
GSVT	0.727426	0.723612	0.731295	300.0
SB	0.739039	0.736412	0.741689	892.0
SR	0.731431	0.732749	0.730149	681.0
macro avg	0.727418	0.729439	0.725587	2077.0
micro avg	0.732232	0.732232	0.732232	2077.0
weighted avg	0.732189	0.732240	0.732232	2077.0

The result of Model Number:Dataset Number:Featuregroup Number:Rescale of 7 : 0 : 7 : 0

	f1-score	precision	recall	support
AFIB	0.669834	0.661282	0.678649	875.0
GSVT	0.681591	0.685500	0.677745	691.0
SB	0.729601	0.726537	0.732698	1553.0
SR	0.708119	0.719580	0.697029	907.0
macro avg	0.697286	0.698225	0.696530	4026.0
micro avg	0.703484	0.703484	0.703484	4026.0
weighted avg	0.703532	0.703744	0.703484	4026.0

The result of Model Number:Dataset Number:Featuregroup Number:Rescale of 7 : 0 : 7 : 1

	f1-score	precision	recall	support
AFIB	0.780420	0.768180	0.793141	875.0
GSVT	0.751986	0.773544	0.731684	691.0
SB	0.790212	0.750156	0.834833	1553.0
SR	0.642043	0.714463	0.583053	907.0
macro avg	0.741165	0.751586	0.735678	4026.0
micro avg	0.751345	0.751345	0.751345	4026.0
weighted avg	0.748142	0.750046	0.751345	4026.0

The result of Model Number:Dataset Number:Featuregroup Number:Rescale of 7 : 0 : 8 : 0

	f1-score	precision	recall	support
AFIB	0.888452	0.901367	0.876304	204.0
GSVT	0.908046	0.895761	0.920745	300.0
SB	0.921712	0.912107	0.931529	892.0
SR	0.903444	0.918394	0.888988	681.0
macro avg	0.905414	0.906907	0.904392	2077.0
micro avg	0.910599	0.910599	0.910599	2077.0
weighted avg	0.910482	0.910752	0.910599	2077.0

The result of Model Number:Dataset Number:Featuregroup Number:Rescale of 7 : 0 : 8 : 1

	f1-score	precision	recall	support
AFIB	0.753960	0.744263	0.763966	875.0
GSVT	0.769307	0.775676	0.763074	691.0
SB	0.822544	0.818883	0.826248	1553.0
SR	0.799137	0.810956	0.787676	907.0
macro avg	0.786237	0.787445	0.785241	4026.0
micro avg	0.793179	0.793179	0.793179	4026.0
weighted avg	0.793228	0.793464	0.793179	4026.0

The result of Model Number:Dataset Number:Featuregroup Number:Rescale of 7 : 0 : 9 : 0

	f1-score	precision	recall	support
AFIB	0.835100	0.823500	0.847111	875.0
GSVT	0.864942	0.876481	0.853816	691.0
SB	0.918849	0.916967	0.920743	1553.0
SR	0.894935	0.901850	0.888126	907.0
macro avg	0.878457	0.879699	0.877449	4026.0
micro avg	0.885905	0.885905	0.885905	4026.0
weighted avg	0.886008	0.886299	0.885905	4026.0

The result of Model Number:Dataset Number:Featuregroup Number:Rescale of 7 : 0 : 9 : 1

	f1-score	precision	recall	support
AFIB	0.812951	0.880815	0.756416	204.0
GSVT	0.871984	0.861019	0.883587	300.0
SB	0.894244	0.881716	0.907238	892.0
SR	0.869851	0.873926	0.866238	681.0
macro avg	0.862257	0.874369	0.853369	2077.0
micro avg	0.875565	0.875565	0.875565	2077.0
weighted avg	0.875046	0.876084	0.875565	2077.0

The result of Model Number:Dataset Number:Featuregroup Number:Rescale of 7 : 0 : 10 : 0

	f1-score	precision	recall	support
AFIB	0.734009	0.757118	0.712448	204.0
GSVT	0.697348	0.712243	0.683179	300.0
SB	0.720027	0.694831	0.747145	892.0
SR	0.657566	0.679790	0.636797	681.0
macro avg	0.702237	0.710996	0.694892	2077.0
micro avg	0.698317	0.698317	0.698317	2077.0
weighted avg	0.697645	0.698532	0.698317	2077.0

The result of Model Number:Dataset Number:Featuregroup Number:Rescale of 7 : 0 : 10 : 1

	f1-score	precision	recall	support
AFIB	0.858082	0.844925	0.871798	875.0
GSVT	0.887670	0.896371	0.879311	691.0
SB	0.944718	0.942511	0.946965	1553.0
SR	0.921118	0.932798	0.909748	907.0

macro avg	0.902897	0.904151	0.901956	4026.0
micro avg	0.910632	0.910632	0.910632	4026.0
weighted avg	0.910781	0.911194	0.910632	4026.0

The result of Model Number:Dataset Number:Featuregroup Number:Rescale of 7 : 1 : 0 : 0

	f1-score	precision	recall	support
AFIB	0.888416	0.911892	0.866205	204.0
GSVT	0.912780	0.905197	0.920658	300.0
SB	0.937932	0.934490	0.941402	892.0
SR	0.923410	0.924529	0.922318	681.0
macro avg	0.915634	0.919027	0.912646	2077.0
micro avg	0.924763	0.924763	0.924763	2077.0
weighted avg	0.924674	0.924773	0.924763	2077.0

The result of Model Number:Dataset Number:Featuregroup Number:Rescale of 7 : 1 : 0 : 1

	f1-score	precision	recall	support
AFIB	0.722268	0.700681	0.770126	875.0
GSVT	0.618516	0.604972	0.632910	691.0
SB	0.820388	0.813704	0.827401	1553.0
SR	0.769642	0.801955	0.741276	907.0
macro avg	0.732704	0.730328	0.742928	4026.0
micro avg	0.762169	0.762169	0.762169	4026.0
weighted avg	0.752983	0.750667	0.762169	4026.0

The result of Model Number:Dataset Number:Featuregroup Number:Rescale of 7 : 1 : 1 : 0

	f1-score	precision	recall	support
AFIB	0.750818	0.761447	0.741098	204.0
GSVT	0.766406	0.752295	0.781197	300.0
SB	0.782741	0.774087	0.791604	892.0
SR	0.766291	0.781898	0.751341	681.0
macro avg	0.766564	0.767432	0.766310	2077.0
micro avg	0.771939	0.771939	0.771939	2077.0
weighted avg	0.771853	0.772259	0.771939	2077.0

The result of Model Number:Dataset Number:Featuregroup Number:Rescale of 7 : 1 : 1 : 1

	f1-score	precision	recall	support
AFIB	0.797960	0.800655	0.795312	875.0
GSVT	0.795853	0.790247	0.801611	691.0
SB	0.837384	0.829612	0.845308	1553.0
SR	0.805548	0.821072	0.790640	907.0
macro avg	0.809186	0.810397	0.808218	4026.0
micro avg	0.814626	0.814626	0.814626	4026.0
weighted avg	0.814515	0.814638	0.814626	4026.0

The result of Model Number:Dataset Number:Featuregroup Number:Rescale of 7 : 1 : 2 : 0

	f1-score	precision	recall	support
AFIB	0.655293	0.654233	0.656415	875.0
GSVT	0.661298	0.663928	0.658722	691.0

SB 0.694478 0.691953 0.697026 1553.0
SR 0.683118 0.686574 0.679735 907.0
macro avg 0.673547 0.674172 0.672975 4026.0
micro avg 0.677730 0.677730 0.677730 4026.0
weighted avg 0.677708 0.677733 0.677730 4026.0
The result of Model Number:Dataset Number:Featuregroup Number:Rescale of 7 : 1 : 2 : 1
f1-score precision recall support
AFIB 0.786558 0.806174 0.767933 875.0
GSVT 0.836760 0.839968 0.833616 691.0
SB 0.906141 0.901754 0.910574 1553.0
SR 0.858619 0.844307 0.873479 907.0
macro avg 0.847019 0.848051 0.846400 4026.0
micro avg 0.858007 0.858007 0.858007 4026.0
weighted avg 0.857537 0.857434 0.858007 4026.0
The result of Model Number:Dataset Number:Featuregroup Number:Rescale of 7 : 1 : 3 : 0
f1-score precision recall support
AFIB 0.756439 0.759269 0.753952 875.0
GSVT 0.773466 0.780928 0.766301 691.0
SB 0.822436 0.813810 0.831311 1553.0
SR 0.793956 0.800944 0.787363 907.0
macro avg 0.786575 0.788738 0.784732 4026.0
micro avg 0.793439 0.793439 0.793439 4026.0
weighted avg 0.793272 0.793414 0.793439 4026.0
The result of Model Number:Dataset Number:Featuregroup Number:Rescale of 7 : 1 : 3 : 1
f1-score precision recall support
AFIB 0.737821 0.744630 0.731176 875.0
GSVT 0.755050 0.753011 0.757125 691.0
SB 0.804138 0.796580 0.811850 1553.0
SR 0.781921 0.789766 0.774244 907.0
macro avg 0.769732 0.770997 0.768599 4026.0
micro avg 0.776452 0.776452 0.776452 4026.0
weighted avg 0.776294 0.776276 0.776452 4026.0
The result of Model Number:Dataset Number:Featuregroup Number:Rescale of 7 : 1 : 4 : 0
f1-score precision recall support
AFIB 0.854431 0.855193 0.853758 875.0
GSVT 0.870082 0.872364 0.867957 691.0
SB 0.923603 0.917454 0.929845 1553.0
SR 0.905608 0.913876 0.897521 907.0
macro avg 0.888431 0.889722 0.887270 4026.0
micro avg 0.895404 0.895404 0.895404 4026.0
weighted avg 0.895329 0.895377 0.895404 4026.0
The result of Model Number:Dataset Number:Featuregroup Number:Rescale of 7 : 1 : 4 : 1
f1-score precision recall support

AFIB	0.814654	0.831068	0.799399	204.0
GSVT	0.840794	0.834041	0.847819	300.0
SB	0.858996	0.858132	0.859863	892.0
SR	0.851166	0.850678	0.851665	681.0
macro avg	0.841403	0.843480	0.839687	2077.0
micro avg	0.849497	0.849497	0.849497	2077.0
weighted avg	0.849445	0.849550	0.849497	2077.0

The result of Model Number:Dataset Number:Featuregroup Number:Rescale of 7 : 1 : 5 : 0

	f1-score	precision	recall	support
AFIB	0.824722	0.839878	0.810204	204.0
GSVT	0.838575	0.833084	0.844162	300.0
SB	0.852920	0.848755	0.857126	892.0
SR	0.844104	0.847613	0.840635	681.0
macro avg	0.840080	0.842333	0.838032	2077.0
micro avg	0.845238	0.845238	0.845238	2077.0
weighted avg	0.845188	0.845245	0.845238	2077.0

The result of Model Number:Dataset Number:Featuregroup Number:Rescale of 7 : 1 : 5 : 1

	f1-score	precision	recall	support
AFIB	0.702400	0.695335	0.709630	875.0
GSVT	0.708686	0.714995	0.702538	691.0
SB	0.749758	0.747457	0.752075	1553.0
SR	0.735239	0.741675	0.728917	907.0
macro avg	0.724021	0.724866	0.723290	4026.0
micro avg	0.729130	0.729130	0.729130	4026.0
weighted avg	0.729145	0.729255	0.729130	4026.0

The result of Model Number:Dataset Number:Featuregroup Number:Rescale of 7 : 1 : 6 : 0

	f1-score	precision	recall	support
AFIB	0.762135	0.756985	0.767460	875.0
GSVT	0.780729	0.781040	0.780519	691.0
SB	0.829365	0.827249	0.831502	1553.0
SR	0.806783	0.815797	0.797977	907.0
macro avg	0.794753	0.795268	0.794364	4026.0
micro avg	0.801280	0.801280	0.801280	4026.0
weighted avg	0.801319	0.801467	0.801280	4026.0

The result of Model Number:Dataset Number:Featuregroup Number:Rescale of 7 : 1 : 6 : 1

	f1-score	precision	recall	support
AFIB	0.648106	0.643220	0.735591	204.0
GSVT	0.785700	0.781048	0.790416	300.0
SB	0.642593	0.641252	0.643943	892.0
SR	0.794737	0.793465	0.796023	681.0
macro avg	0.717784	0.714746	0.741493	2077.0
micro avg	0.723965	0.723965	0.723965	2077.0
weighted avg	0.713689	0.711545	0.723965	2077.0

The result of Model Number:Dataset Number:Featuregroup Number:Rescale of 7 : 1 : 7 : 0

	f1-score	precision	recall	support
AFIB	0.672284	0.682477	0.662409	204.0
GSVT	0.683095	0.677336	0.688988	300.0
SB	0.698246	0.695994	0.700515	892.0
SR	0.690751	0.693317	0.688223	681.0
macro avg	0.686094	0.687281	0.685034	2077.0
micro avg	0.691077	0.691077	0.691077	2077.0
weighted avg	0.691050	0.691094	0.691077	2077.0

The result of Model Number:Dataset Number:Featuregroup Number:Rescale of 7 : 1 : 7 : 1

	f1-score	precision	recall	support
AFIB	0.779968	0.780339	0.779709	875.0
GSVT	0.800167	0.793241	0.807243	691.0
SB	0.854581	0.848354	0.860909	1553.0
SR	0.818072	0.834335	0.802474	907.0
macro avg	0.813197	0.814067	0.812584	4026.0
micro avg	0.820886	0.820886	0.820886	4026.0
weighted avg	0.820801	0.820954	0.820886	4026.0

The result of Model Number:Dataset Number:Featuregroup Number:Rescale of 7 : 1 : 8 : 0

	f1-score	precision	recall	support
AFIB	0.684282	0.763187	0.620254	204.0
GSVT	0.806838	0.792795	0.821560	300.0
SB	0.861454	0.857635	0.865315	892.0
SR	0.841463	0.829957	0.853317	681.0
macro avg	0.798509	0.810894	0.790112	2077.0
micro avg	0.830992	0.830992	0.830992	2077.0
weighted avg	0.829609	0.829918	0.830992	2077.0

The result of Model Number:Dataset Number:Featuregroup Number:Rescale of 7 : 1 : 8 : 1

	f1-score	precision	recall	support
AFIB	0.851197	0.887013	0.818237	204.0
GSVT	0.879755	0.866289	0.893780	300.0
SB	0.900975	0.894513	0.907541	892.0
SR	0.877591	0.882053	0.873206	681.0
macro avg	0.877379	0.882467	0.873191	2077.0
micro avg	0.885525	0.885525	0.885525	2077.0
weighted avg	0.885354	0.885614	0.885525	2077.0

The result of Model Number:Dataset Number:Featuregroup Number:Rescale of 7 : 1 : 9 : 0

	f1-score	precision	recall	support
AFIB	0.801842	0.811217	0.792835	204.0
GSVT	0.823900	0.820112	0.827746	300.0
SB	0.835565	0.832967	0.838186	892.0
SR	0.828369	0.830717	0.826070	681.0
macro avg	0.822419	0.823753	0.821209	2077.0

micro avg 0.828251 0.828251 0.828251 2077.0
weighted avg 0.828209 0.828236 0.828251 2077.0
The result of Model Number:Dataset Number:Featuregroup Number:Rescale of 7 : 1 : 9 : 1
f1-score precision recall support
AFIB 0.868898 0.876912 0.861239 204.0
GSVT 0.900664 0.900696 0.900656 300.0
SB 0.919527 0.917894 0.921174 892.0
SR 0.909768 0.909524 0.910034 681.0
macro avg 0.899714 0.901257 0.898276 2077.0
micro avg 0.908671 0.908671 0.908671 2077.0
weighted avg 0.908630 0.908640 0.908671 2077.0
The result of Model Number:Dataset Number:Featuregroup Number:Rescale of 7 : 1 : 10 : 0
f1-score precision recall support
AFIB 0.631525 0.700864 0.574823 204.0
GSVT 0.737392 0.728173 0.746991 300.0
SB 0.784548 0.780545 0.788594 892.0
SR 0.766169 0.755458 0.777192 681.0
macro avg 0.729909 0.741260 0.721900 2077.0
micro avg 0.757850 0.757850 0.757850 2077.0
weighted avg 0.756681 0.756929 0.757850 2077.0
The result of Model Number:Dataset Number:Featuregroup Number:Rescale of 7 : 1 : 10 : 1
f1-score precision recall support
AFIB 0.709188 0.719940 0.698770 204.0
GSVT 0.720591 0.714517 0.726808 300.0
SB 0.736575 0.734199 0.738968 892.0
SR 0.728668 0.731375 0.726001 681.0
macro avg 0.723755 0.725007 0.722637 2077.0
micro avg 0.729012 0.729012 0.729012 2077.0
weighted avg 0.728984 0.729029 0.729012 2077.0
The result of Model Number:Dataset Number:Featuregroup Number:Rescale of 8 : 0 : 0 : 0
f1-score precision recall support
AFIB 0.659666 0.652301 0.667207 875.0
GSVT 0.672790 0.678953 0.666749 691.0
SB 0.717562 0.715225 0.719917 1553.0
SR 0.698960 0.705962 0.692098 907.0
macro avg 0.687245 0.688110 0.686493 4026.0
micro avg 0.693068 0.693068 0.693068 4026.0
weighted avg 0.693104 0.693237 0.693068 4026.0
The result of Model Number:Dataset Number:Featuregroup Number:Rescale of 8 : 0 : 0 : 1
f1-score precision recall support
AFIB 0.691056 0.684877 0.697377 875.0

GSVT	0.702151	0.704929	0.699420	691.0
SB	0.748643	0.747254	0.750041	1553.0
SR	0.729673	0.736418	0.723077	907.0
macro avg	0.717881	0.718370	0.717479	4026.0
micro avg	0.723832	0.723832	0.723832	4026.0
weighted avg	0.723874	0.723992	0.723832	4026.0

The result of Model Number:Dataset Number:Featuregroup Number:Rescale of 8 : 0 : 1 : 0

	f1-score	precision	recall	support
AFIB	0.640008	0.631852	0.648399	875.0
GSVT	0.611091	0.615296	0.606959	691.0
SB	0.647036	0.609646	0.689326	1553.0
SR	0.527680	0.610511	0.464670	907.0
macro avg	0.606454	0.616826	0.602339	4026.0
micro avg	0.615682	0.615682	0.615682	4026.0
weighted avg	0.612450	0.615637	0.615682	4026.0

The result of Model Number:Dataset Number:Featuregroup Number:Rescale of 8 : 0 : 1 : 1

	f1-score	precision	recall	support
AFIB	0.766860	0.766615	0.767145	875.0
GSVT	0.778288	0.768284	0.788648	691.0
SB	0.823227	0.821436	0.825029	1553.0
SR	0.800449	0.812183	0.789063	907.0
macro avg	0.792206	0.792129	0.792471	4026.0
micro avg	0.798102	0.798102	0.798102	4026.0
weighted avg	0.798132	0.798314	0.798102	4026.0

The result of Model Number:Dataset Number:Featuregroup Number:Rescale of 8 : 0 : 2 : 0

	f1-score	precision	recall	support
AFIB	0.733387	0.727777	0.739181	875.0
GSVT	0.750261	0.754364	0.746247	691.0
SB	0.794466	0.793910	0.795029	1553.0
SR	0.773364	0.777115	0.769688	907.0
macro avg	0.762869	0.763291	0.762536	4026.0
micro avg	0.768810	0.768810	0.768810	4026.0
weighted avg	0.768850	0.768966	0.768810	4026.0

The result of Model Number:Dataset Number:Featuregroup Number:Rescale of 8 : 0 : 2 : 1

	f1-score	precision	recall	support
AFIB	0.694723	0.695926	0.693534	875.0
GSVT	0.703146	0.695067	0.711474	691.0
SB	0.740438	0.731509	0.749589	1553.0
SR	0.705242	0.726611	0.685111	907.0
macro avg	0.710887	0.712278	0.709927	4026.0
micro avg	0.716338	0.716338	0.716338	4026.0
weighted avg	0.716173	0.716417	0.716338	4026.0

The result of Model Number:Dataset Number:Featuregroup Number:Rescale of 8 : 0 : 3 : 0

	f1-score	precision	recall	support
AFIB	0.881480	0.869086	0.894274	875.0
GSVT	0.900458	0.907214	0.893825	691.0
SB	0.957534	0.954720	0.960368	1553.0
SR	0.933948	0.946868	0.921390	907.0
macro avg	0.918355	0.919472	0.917464	4026.0
micro avg	0.925801	0.925801	0.925801	4026.0
weighted avg	0.925895	0.926186	0.925801	4026.0

The result of Model Number:Dataset Number:Featuregroup Number:Rescale of 8 : 0 : 3 : 1

	f1-score	precision	recall	support
AFIB	0.716911	0.715390	0.718472	875.0
GSVT	0.728111	0.724621	0.731696	691.0
SB	0.766334	0.757186	0.775708	1553.0
SR	0.730721	0.751423	0.711144	907.0
macro avg	0.735519	0.737155	0.734255	4026.0
micro avg	0.741169	0.741169	0.741169	4026.0
weighted avg	0.741009	0.741214	0.741169	4026.0

The result of Model Number:Dataset Number:Featuregroup Number:Rescale of 8 : 0 : 4 : 0

	f1-score	precision	recall	support
AFIB	0.836198	0.828363	0.844215	875.0
GSVT	0.850253	0.857071	0.843625	691.0
SB	0.901312	0.897861	0.904794	1553.0
SR	0.881145	0.890089	0.872403	907.0
macro avg	0.867227	0.868346	0.866259	4026.0
micro avg	0.873832	0.873832	0.873832	4026.0
weighted avg	0.873853	0.874004	0.873832	4026.0

The result of Model Number:Dataset Number:Featuregroup Number:Rescale of 8 : 0 : 4 : 1

	f1-score	precision	recall	support
AFIB	0.806834	0.808463	0.805228	204.0
GSVT	0.829280	0.826815	0.831765	300.0
SB	0.843021	0.842270	0.843777	892.0
SR	0.835775	0.837380	0.834187	681.0
macro avg	0.828728	0.828732	0.828739	2077.0
micro avg	0.835111	0.835111	0.835111	2077.0
weighted avg	0.835106	0.835114	0.835111	2077.0

The result of Model Number:Dataset Number:Featuregroup Number:Rescale of 8 : 0 : 5 : 0

	f1-score	precision	recall	support
AFIB	0.825771	0.823232	0.828379	875.0
GSVT	0.804721	0.808668	0.800941	691.0
SB	0.845988	0.810881	0.884280	1553.0
SR	0.728043	0.794335	0.672020	907.0
macro avg	0.801131	0.809279	0.796405	4026.0
micro avg	0.810008	0.810008	0.810008	4026.0

weighted avg	0.807940	0.809458	0.810008	4026.0

The result of Model Number:Dataset Number:Featuregroup Number:Rescale of 8 : 0 : 5 : 1

	f1-score	precision	recall	support
AFIB	0.626282	0.641878	0.611457	875.0
GSVT	0.661712	0.664521	0.658954	691.0
SB	0.717747	0.715000	0.720518	1553.0
SR	0.682735	0.670012	0.695986	907.0
macro avg	0.672119	0.672853	0.671729	4026.0
micro avg	0.680722	0.680722	0.680722	4026.0
weighted avg	0.680363	0.680309	0.680722	4026.0

The result of Model Number:Dataset Number:Featuregroup Number:Rescale of 8 : 0 : 6 : 0

	f1-score	precision	recall	support
AFIB	0.915139	0.922527	0.907946	204.0
GSVT	0.936137	0.931547	0.940805	300.0
SB	0.954408	0.953349	0.955477	892.0
SR	0.947281	0.948541	0.946031	681.0
macro avg	0.938241	0.938991	0.937565	2077.0
micro avg	0.945592	0.945592	0.945592	2077.0
weighted avg	0.945575	0.945596	0.945592	2077.0

The result of Model Number:Dataset Number:Featuregroup Number:Rescale of 8 : 0 : 6 : 1

	f1-score	precision	recall	support
AFIB	0.738567	0.718082	0.762579	875.0
GSVT	0.748208	0.779724	0.723745	691.0
SB	0.821933	0.821518	0.822359	1553.0
SR	0.801313	0.806327	0.796366	907.0
macro avg	0.777505	0.781413	0.776262	4026.0
micro avg	0.786585	0.786585	0.786585	4026.0
weighted avg	0.786515	0.788442	0.786585	4026.0

The result of Model Number:Dataset Number:Featuregroup Number:Rescale of 8 : 0 : 7 : 0

	f1-score	precision	recall	support
AFIB	0.874392	0.882214	0.866816	875.0
GSVT	0.891091	0.874147	0.908876	691.0
SB	0.945819	0.937625	0.954161	1553.0
SR	0.906972	0.927826	0.887075	907.0
macro avg	0.904569	0.905453	0.904232	4026.0
micro avg	0.912292	0.912292	0.912292	4026.0
weighted avg	0.912150	0.912480	0.912292	4026.0

The result of Model Number:Dataset Number:Featuregroup Number:Rescale of 8 : 0 : 7 : 1

	f1-score	precision	recall	support
AFIB	0.729756	0.730981	0.747844	204.0
GSVT	0.722896	0.750949	0.697796	300.0
SB	0.766788	0.724737	0.814334	892.0
SR	0.664642	0.725979	0.614544	681.0

macro avg 0.721021 0.733161 0.718629 2077.0
micro avg 0.725464 0.725464 0.725464 2077.0
weighted avg 0.723320 0.729543 0.725464 2077.0
The result of Model Number:Dataset Number:Featuregroup Number:Rescale of 8 : 0 : 8 : 0

	f1-score	precision	recall	support
AFIB	0.920364	0.928391	0.912704	204.0
GSVT	0.952193	0.950638	0.953776	300.0
SB	0.972432	0.970061	0.974828	892.0
SR	0.963517	0.964969	0.962102	681.0
macro avg	0.952127	0.953515	0.950852	2077.0
micro avg	0.961513	0.961513	0.961513	2077.0
weighted avg	0.961472	0.961493	0.961513	2077.0

The result of Model Number:Dataset Number:Featuregroup Number:Rescale of 8 : 0 : 8 : 1

	f1-score	precision	recall	support
AFIB	0.795887	0.789759	0.802172	875.0
GSVT	0.806221	0.809964	0.802688	691.0
SB	0.863978	0.859382	0.868642	1553.0
SR	0.838522	0.850311	0.827148	907.0
macro avg	0.826152	0.827354	0.825162	4026.0
micro avg	0.833528	0.833528	0.833528	4026.0
weighted avg	0.833531	0.833725	0.833528	4026.0

The result of Model Number:Dataset Number:Featuregroup Number:Rescale of 8 : 0 : 9 : 0

	f1-score	precision	recall	support
AFIB	0.821533	0.841062	0.802982	204.0
GSVT	0.842861	0.839608	0.846227	300.0
SB	0.863747	0.861819	0.865683	892.0
SR	0.852880	0.851164	0.854633	681.0
macro avg	0.845255	0.848413	0.842381	2077.0
micro avg	0.853091	0.853091	0.853091	2077.0
weighted avg	0.853021	0.853079	0.853091	2077.0

The result of Model Number:Dataset Number:Featuregroup Number:Rescale of 8 : 0 : 9 : 1

	f1-score	precision	recall	support
AFIB	0.815102	0.802919	0.827690	875.0
GSVT	0.830730	0.837834	0.823758	691.0
SB	0.887616	0.884559	0.890699	1553.0
SR	0.861636	0.874487	0.849186	907.0
macro avg	0.848771	0.849950	0.847833	4026.0
micro avg	0.856163	0.856163	0.856163	4026.0
weighted avg	0.856239	0.856527	0.856163	4026.0

The result of Model Number:Dataset Number:Featuregroup Number:Rescale of 8 : 0 : 10 : 0

	f1-score	precision	recall	support
AFIB	0.664025	0.669965	0.658272	875.0

GSVT	0.676707	0.663839	0.690213	691.0
SB	0.718268	0.712045	0.724603	1553.0
SR	0.688767	0.704603	0.673657	907.0
macro avg	0.686942	0.687613	0.686686	4026.0
micro avg	0.692807	0.692807	0.692807	4026.0
weighted avg	0.692699	0.692949	0.692807	4026.0

The result of Model Number:Dataset Number:Featuregroup Number:Rescale of 8 : 0 : 10 : 1

	f1-score	precision	recall	support
AFIB	0.590067	0.649599	0.540876	204.0
GSVT	0.695861	0.686256	0.705850	300.0
SB	0.738870	0.736405	0.741355	892.0
SR	0.719948	0.709988	0.730202	681.0
macro avg	0.686187	0.695562	0.679571	2077.0
micro avg	0.712879	0.712879	0.712879	2077.0
weighted avg	0.711839	0.711974	0.712879	2077.0

The result of Model Number:Dataset Number:Featuregroup Number:Rescale of 8 : 1 : 0 : 0

	f1-score	precision	recall	support
AFIB	0.751279	0.755387	0.747240	875.0
GSVT	0.747698	0.741394	0.754186	691.0
SB	0.786392	0.778945	0.793986	1553.0
SR	0.757125	0.771248	0.743520	907.0
macro avg	0.760623	0.761743	0.759733	4026.0
micro avg	0.765626	0.765626	0.765626	4026.0
weighted avg	0.765526	0.765646	0.765626	4026.0

The result of Model Number:Dataset Number:Featuregroup Number:Rescale of 8 : 1 : 0 : 1

	f1-score	precision	recall	support
AFIB	0.679271	0.671932	0.773236	204.0
GSVT	0.812704	0.806371	0.819206	300.0
SB	0.663244	0.661857	0.664638	892.0
SR	0.822437	0.822206	0.822678	681.0
macro avg	0.744414	0.740592	0.769939	2077.0
micro avg	0.749448	0.749448	0.749448	2077.0
weighted avg	0.738601	0.736295	0.749448	2077.0

The result of Model Number:Dataset Number:Featuregroup Number:Rescale of 8 : 1 : 1 : 0

	f1-score	precision	recall	support
AFIB	0.781547	0.787169	0.776006	875.0
GSVT	0.780301	0.775629	0.785043	691.0
SB	0.823104	0.813984	0.832432	1553.0
SR	0.789832	0.803923	0.776252	907.0
macro avg	0.793696	0.795176	0.792433	4026.0
micro avg	0.799378	0.799378	0.799378	4026.0
weighted avg	0.799230	0.799307	0.799378	4026.0

The result of Model Number:Dataset Number:Featuregroup Number:Rescale of 8 : 1 : 1 : 1

	f1-score	precision	recall	support
AFIB	0.671546	0.685493	0.658309	875.0
GSVT	0.654891	0.632145	0.708349	691.0
SB	0.771891	0.768410	0.775416	1553.0
SR	0.586828	0.577354	0.596671	907.0
macro avg	0.671289	0.665851	0.684686	4026.0
micro avg	0.698185	0.698185	0.698185	4026.0
weighted avg	0.688309	0.683959	0.698185	4026.0

The result of Model Number:Dataset Number:Featuregroup Number:Rescale of 8 : 1 : 2 : 0

	f1-score	precision	recall	support
AFIB	0.711440	0.717011	0.705992	875.0
GSVT	0.713316	0.704212	0.722702	691.0
SB	0.748188	0.745187	0.751221	1553.0
SR	0.732381	0.739570	0.725357	907.0
macro avg	0.726331	0.726495	0.726318	4026.0
micro avg	0.730669	0.730669	0.730669	4026.0
weighted avg	0.730655	0.730765	0.730669	4026.0

The result of Model Number:Dataset Number:Featuregroup Number:Rescale of 8 : 1 : 2 : 1

	f1-score	precision	recall	support
AFIB	0.745692	0.743625	0.747826	875.0
GSVT	0.751593	0.755178	0.748117	691.0
SB	0.786786	0.784272	0.789319	1553.0
SR	0.774208	0.777947	0.770540	907.0
macro avg	0.764570	0.765256	0.763951	4026.0
micro avg	0.768999	0.768999	0.768999	4026.0
weighted avg	0.768981	0.769020	0.768999	4026.0

The result of Model Number:Dataset Number:Featuregroup Number:Rescale of 8 : 1 : 3 : 0

	f1-score	precision	recall	support
AFIB	0.750668	0.801549	0.706359	204.0
GSVT	0.707306	0.728292	0.687762	300.0
SB	0.739312	0.703821	0.778993	892.0
SR	0.656003	0.685786	0.629414	681.0
macro avg	0.713322	0.729862	0.700632	2077.0
micro avg	0.709638	0.709638	0.709638	2077.0
weighted avg	0.708489	0.711041	0.709638	2077.0

The result of Model Number:Dataset Number:Featuregroup Number:Rescale of 8 : 1 : 3 : 1

	f1-score	precision	recall	support
AFIB	0.691766	0.677795	0.706379	875.0
GSVT	0.714701	0.726065	0.703841	691.0
SB	0.760452	0.760410	0.760501	1553.0
SR	0.742015	0.748776	0.735387	907.0
macro avg	0.727234	0.728261	0.726527	4026.0

micro avg 0.733356 0.733356 0.733356 4026.0
weighted avg 0.733518 0.733939 0.733356 4026.0
The result of Model Number:Dataset Number:Featuregroup Number:Rescale of 8 : 1 : 4 : 0

	f1-score	precision	recall	support
AFIB	0.869560	0.916862	0.827122	204.0
GSVT	0.912878	0.899071	0.927152	300.0
SB	0.934571	0.928491	0.940745	892.0
SR	0.923041	0.923873	0.922232	681.0
macro avg	0.910012	0.917074	0.904313	2077.0
micro avg	0.921552	0.921552	0.921552	2077.0
weighted avg	0.921272	0.921585	0.921552	2077.0

The result of Model Number:Dataset Number:Featuregroup Number:Rescale of 8 : 1 : 4 : 1

	f1-score	precision	recall	support
AFIB	0.676369	0.683885	0.669364	204.0
GSVT	0.697320	0.693350	0.701538	300.0
SB	0.711192	0.710239	0.712149	892.0
SR	0.705142	0.706087	0.704209	681.0
macro avg	0.697506	0.698390	0.696815	2077.0
micro avg	0.703811	0.703811	0.703811	2077.0
weighted avg	0.703785	0.703850	0.703811	2077.0

The result of Model Number:Dataset Number:Featuregroup Number:Rescale of 8 : 1 : 5 : 0

	f1-score	precision	recall	support
AFIB	0.831917	0.816365	0.848161	875.0
GSVT	0.861057	0.874651	0.847988	691.0
SB	0.915698	0.914062	0.917348	1553.0
SR	0.892423	0.901877	0.883170	907.0
macro avg	0.875274	0.876739	0.874167	4026.0
micro avg	0.882707	0.882707	0.882707	4026.0
weighted avg	0.882867	0.883319	0.882707	4026.0

The result of Model Number:Dataset Number:Featuregroup Number:Rescale of 8 : 1 : 5 : 1

	f1-score	precision	recall	support
AFIB	0.856960	0.872709	0.841875	204.0
GSVT	0.871355	0.865648	0.877160	300.0
SB	0.886260	0.881932	0.890631	892.0
SR	0.877099	0.880746	0.873495	681.0
macro avg	0.872918	0.875259	0.870790	2077.0
micro avg	0.878278	0.878278	0.878278	2077.0
weighted avg	0.878225	0.878285	0.878278	2077.0

The result of Model Number:Dataset Number:Featuregroup Number:Rescale of 8 : 1 : 6 : 0

	f1-score	precision	recall	support
AFIB	0.709951	0.733527	0.688112	204.0
GSVT	0.736894	0.730982	0.743013	300.0
SB	0.753174	0.750080	0.756297	892.0

SR 0.746140 0.746031 0.746250 681.0
macro avg 0.736540 0.740155 0.733418 2077.0
micro avg 0.744387 0.744387 0.744387 2077.0
weighted avg 0.744271 0.744368 0.744387 2077.0
The result of Model Number:Dataset Number:Featuregroup Number:Rescale of 8 : 1 : 6 : 1
f1-score precision recall support
AFIB 0.747434 0.757542 0.737906 204.0
GSVT 0.775825 0.774565 0.777115 300.0
SB 0.792673 0.791617 0.793739 892.0
SR 0.784571 0.783565 0.785606 681.0
macro avg 0.775126 0.776822 0.773592 2077.0
micro avg 0.783187 0.783187 0.783187 2077.0
weighted avg 0.783139 0.783167 0.783187 2077.0
The result of Model Number:Dataset Number:Featuregroup Number:Rescale of 8 : 1 : 7 : 0
f1-score precision recall support
AFIB 0.589801 0.642496 0.622843 204.0
GSVT 0.801113 0.788017 0.814928 300.0
SB 0.681995 0.676800 0.687320 892.0
SR 0.833968 0.824532 0.843648 681.0
macro avg 0.726719 0.732961 0.742185 2077.0
micro avg 0.750675 0.750675 0.750675 2077.0
weighted avg 0.739973 0.737933 0.750675 2077.0
The result of Model Number:Dataset Number:Featuregroup Number:Rescale of 8 : 1 : 7 : 1
f1-score precision recall support
AFIB 0.879937 0.875506 0.884451 875.0
GSVT 0.897076 0.901419 0.892789 691.0
SB 0.955441 0.953242 0.957653 1553.0
SR 0.931522 0.936506 0.926601 907.0
macro avg 0.915994 0.916668 0.915374 4026.0
micro avg 0.923615 0.923615 0.923615 4026.0
weighted avg 0.923625 0.923682 0.923615 4026.0
The result of Model Number:Dataset Number:Featuregroup Number:Rescale of 8 : 1 : 8 : 0
f1-score precision recall support
AFIB 0.712621 0.726917 0.699019 204.0
GSVT 0.729136 0.721557 0.736915 300.0
SB 0.742622 0.739733 0.745535 892.0
SR 0.734818 0.737884 0.731794 681.0
macro avg 0.729799 0.731523 0.728316 2077.0
micro avg 0.735216 0.735216 0.735216 2077.0
weighted avg 0.735169 0.735243 0.735216 2077.0
The result of Model Number:Dataset Number:Featuregroup Number:Rescale of 8 : 1 : 8 : 1
f1-score precision recall support
AFIB 0.790106 0.792173 0.788206 204.0

GSVT 0.807933 0.804805 0.811153 300.0
SB 0.826053 0.824950 0.827162 892.0
SR 0.819437 0.821739 0.817151 681.0
macro avg 0.810882 0.810917 0.810918 2077.0
micro avg 0.817741 0.817741 0.817741 2077.0
weighted avg 0.817736 0.817768 0.817741 2077.0

The result of Model Number:Dataset Number:Featuregroup Number:Rescale of 8 : 1 : 9 : 0

	f1-score	precision	recall	support
AFIB	0.738476	0.748048	0.729341	204.0
GSVT	0.767050	0.766554	0.767569	300.0
SB	0.783212	0.781558	0.784878	892.0
SR	0.775335	0.774895	0.775793	681.0
macro avg	0.766018	0.767764	0.764395	2077.0
micro avg	0.773945	0.773945	0.773945	2077.0
weighted avg	0.773901	0.773915	0.773945	2077.0

The result of Model Number:Dataset Number:Featuregroup Number:Rescale of 8 : 1 : 9 : 1

	f1-score	precision	recall	support
AFIB	0.704790	0.706011	0.703583	875.0
GSVT	0.713335	0.705138	0.721783	691.0
SB	0.751167	0.742109	0.760451	1553.0
SR	0.715461	0.737140	0.695038	907.0
macro avg	0.721188	0.722599	0.720214	4026.0
micro avg	0.726718	0.726718	0.726718	4026.0
weighted avg	0.726550	0.726799	0.726718	4026.0

The result of Model Number:Dataset Number:Featuregroup Number:Rescale of 8 : 1 : 10 : 0

	f1-score	precision	recall	support
AFIB	0.739368	0.756259	0.723402	204.0
GSVT	0.772956	0.768145	0.777848	300.0
SB	0.789650	0.787201	0.792126	892.0
SR	0.780607	0.780968	0.780267	681.0
macro avg	0.770645	0.773143	0.768411	2077.0
micro avg	0.779425	0.779425	0.779425	2077.0
weighted avg	0.779335	0.779366	0.779425	2077.0

The result of Model Number:Dataset Number:Featuregroup Number:Rescale of 8 : 1 : 10 : 1

	f1-score	precision	recall	support
AFIB	0.145970	0.147503	0.144469	175.0
GSVT	0.146205	0.144652	0.147792	138.2
SB	0.153980	0.153389	0.154575	310.6
SR	0.150416	0.151170	0.149670	181.4
macro avg	0.149143	0.149179	0.149126	805.2
micro avg	0.150109	0.150109	0.150109	805.2

weighted avg 0.150102 0.150110 0.150109 805.2
The result of Model Number:Dataset Number:Featuregroup Number:Rescale of 9 : 0 : 0 : 0
f1-score precision recall support
AFIB 0.671617 0.664861 0.678530 875.0
GSVT 0.677627 0.683661 0.671749 691.0
SB 0.716900 0.714699 0.719115 1553.0
SR 0.703017 0.709171 0.696972 907.0
macro avg 0.692290 0.693098 0.691591 4026.0
micro avg 0.697176 0.697176 0.697176 4026.0
weighted avg 0.697190 0.697295 0.697176 4026.0
The result of Model Number:Dataset Number:Featuregroup Number:Rescale of 9 : 0 : 0 : 1
f1-score precision recall support
AFIB 0.669342 0.662111 0.761934 204.0
GSVT 0.800824 0.794585 0.807232 300.0
SB 0.653549 0.652182 0.654923 892.0
SR 0.810415 0.810188 0.810653 681.0
macro avg 0.733533 0.729766 0.758685 2077.0
micro avg 0.738493 0.738493 0.738493 2077.0
weighted avg 0.727805 0.725532 0.738493 2077.0
The result of Model Number:Dataset Number:Featuregroup Number:Rescale of 9 : 0 : 1 : 0
f1-score precision recall support
AFIB 0.686829 0.690099 0.683619 875.0
GSVT 0.695313 0.682677 0.708497 691.0
SB 0.735694 0.732877 0.738535 1553.0
SR 0.713949 0.726169 0.702144 907.0
macro avg 0.707946 0.707955 0.708199 4026.0
micro avg 0.713246 0.713246 0.713246 4026.0
weighted avg 0.713244 0.713452 0.713246 4026.0
The result of Model Number:Dataset Number:Featuregroup Number:Rescale of 9 : 0 : 1 : 1
f1-score precision recall support
AFIB 0.808092 0.830833 0.786728 204.0
GSVT 0.828984 0.823165 0.835042 300.0
SB 0.851781 0.848372 0.855218 892.0
SR 0.840459 0.840984 0.839959 681.0
macro avg 0.832329 0.835839 0.829237 2077.0
micro avg 0.840574 0.840574 0.840574 2077.0
weighted avg 0.840485 0.840586 0.840574 2077.0
The result of Model Number:Dataset Number:Featuregroup Number:Rescale of 9 : 0 : 2 : 0
f1-score precision recall support
AFIB 0.662944 0.708210 0.623401 204.0
GSVT 0.700798 0.686518 0.715747 300.0
SB 0.718180 0.711884 0.724616 892.0
SR 0.700465 0.702798 0.698210 681.0

	f1-score	precision	recall	support
macro avg	0.695597	0.702352	0.690493	2077.0
micro avg	0.704736	0.704736	0.704736	2077.0
weighted avg	0.704436	0.704880	0.704736	2077.0

The result of Model Number:Dataset Number:Featuregroup Number:Rescale of 9 : 0 : 2 : 1

	f1-score	precision	recall	support
AFIB	0.689017	0.703529	0.675200	204.0
GSVT	0.710439	0.703949	0.717067	300.0
SB	0.722042	0.720432	0.723661	892.0
SR	0.713461	0.714207	0.712725	681.0
macro avg	0.708740	0.710529	0.707163	2077.0
micro avg	0.714363	0.714363	0.714363	2077.0
weighted avg	0.714309	0.714350	0.714363	2077.0

The result of Model Number:Dataset Number:Featuregroup Number:Rescale of 9 : 0 : 3 : 0

	f1-score	precision	recall	support
AFIB	0.746882	0.724559	0.796371	875.0
GSVT	0.639594	0.625588	0.654479	691.0
SB	0.848345	0.841433	0.855597	1553.0
SR	0.795870	0.829284	0.766538	907.0
macro avg	0.757673	0.755216	0.768246	4026.0
micro avg	0.788142	0.788142	0.788142	4026.0
weighted avg	0.778643	0.776248	0.788142	4026.0

The result of Model Number:Dataset Number:Featuregroup Number:Rescale of 9 : 0 : 3 : 1

	f1-score	precision	recall	support
AFIB	0.875786	0.864057	0.887893	875.0
GSVT	0.895367	0.902355	0.888501	691.0
SB	0.950630	0.947656	0.953628	1553.0
SR	0.927641	0.939939	0.915678	907.0
macro avg	0.912356	0.913502	0.911425	4026.0
micro avg	0.919614	0.919614	0.919614	4026.0
weighted avg	0.919700	0.919973	0.919614	4026.0

The result of Model Number:Dataset Number:Featuregroup Number:Rescale of 9 : 0 : 4 : 0

	f1-score	precision	recall	support
AFIB	0.707023	0.710904	0.703230	875.0
GSVT	0.708012	0.699061	0.717306	691.0
SB	0.744597	0.741609	0.747616	1553.0
SR	0.728613	0.737458	0.720023	907.0
macro avg	0.722061	0.722258	0.722044	4026.0
micro avg	0.726551	0.726551	0.726551	4026.0
weighted avg	0.726551	0.726698	0.726551	4026.0

The result of Model Number:Dataset Number:Featuregroup Number:Rescale of 9 : 0 : 4 : 1

	f1-score	precision	recall	support
AFIB	0.899628	0.939611	0.863033	204.0
GSVT	0.937117	0.929761	0.944612	300.0

SB	0.951432	0.944093	0.958895	892.0
SR	0.940906	0.942460	0.939391	681.0
macro avg	0.932271	0.938981	0.926483	2077.0
micro avg	0.941022	0.941022	0.941022	2077.0
weighted avg	0.940825	0.941047	0.941022	2077.0

The result of Model Number:Dataset Number:Featuregroup Number:Rescale of 9 : 0 : 5 : 0

	f1-score	precision	recall	support
AFIB	0.844314	0.833456	0.855519	875.0
GSVT	0.861501	0.868633	0.854520	691.0
SB	0.921118	0.917018	0.925265	1553.0
SR	0.894905	0.908141	0.882071	907.0
macro avg	0.880460	0.881812	0.879344	4026.0
micro avg	0.888234	0.888234	0.888234	4026.0
weighted avg	0.888288	0.888552	0.888234	4026.0

The result of Model Number:Dataset Number:Featuregroup Number:Rescale of 9 : 0 : 5 : 1

	f1-score	precision	recall	support
AFIB	0.725943	0.750051	0.703613	204.0
GSVT	0.753493	0.747448	0.759750	300.0
SB	0.770141	0.766976	0.773334	892.0
SR	0.762948	0.762836	0.763060	681.0
macro avg	0.753131	0.756828	0.749939	2077.0
micro avg	0.761155	0.761155	0.761155	2077.0
weighted avg	0.761037	0.761136	0.761155	2077.0

The result of Model Number:Dataset Number:Featuregroup Number:Rescale of 9 : 0 : 6 : 0

	f1-score	precision	recall	support
AFIB	0.787993	0.830858	0.749535	204.0
GSVT	0.827247	0.814736	0.840182	300.0
SB	0.846906	0.841396	0.852500	892.0
SR	0.836457	0.837211	0.835724	681.0
macro avg	0.824651	0.831050	0.819486	2077.0
micro avg	0.835108	0.835108	0.835108	2077.0
weighted avg	0.834854	0.835138	0.835108	2077.0

The result of Model Number:Dataset Number:Featuregroup Number:Rescale of 9 : 0 : 6 : 1

	f1-score	precision	recall	support
AFIB	0.817841	0.816863	0.818960	875.0
GSVT	0.833784	0.836652	0.831021	691.0
SB	0.883101	0.877661	0.888616	1553.0
SR	0.865130	0.873579	0.856938	907.0
macro avg	0.849964	0.851189	0.848884	4026.0
micro avg	0.856455	0.856455	0.856455	4026.0
weighted avg	0.856404	0.856489	0.856455	4026.0

The result of Model Number:Dataset Number:Featuregroup Number:Rescale of 9 : 0 : 7 : 0

	f1-score	precision	recall	support

	f1-score	precision	recall	support
AFIB	0.651244	0.656406	0.646179	875.0
GSVT	0.659500	0.656593	0.662456	691.0
SB	0.701301	0.698528	0.704100	1553.0
SR	0.684067	0.686000	0.682179	907.0
macro avg	0.674028	0.674382	0.673728	4026.0
micro avg	0.679426	0.679426	0.679426	4026.0
weighted avg	0.679365	0.679354	0.679426	4026.0

The result of Model Number:Dataset Number:Featuregroup Number:Rescale of 9 : 0 : 7 : 1

	f1-score	precision	recall	support
AFIB	0.885645	0.909953	0.862667	204.0
GSVT	0.905675	0.891762	0.920116	300.0
SB	0.925574	0.916621	0.934705	892.0
SR	0.902181	0.913454	0.891189	681.0
macro avg	0.904769	0.907948	0.902169	2077.0
micro avg	0.911255	0.911255	0.911255	2077.0
weighted avg	0.911108	0.911337	0.911255	2077.0

The result of Model Number:Dataset Number:Featuregroup Number:Rescale of 9 : 0 : 8 : 0

	f1-score	precision	recall	support
AFIB	0.690220	0.720511	0.662546	204.0
GSVT	0.642550	0.647176	0.638253	300.0
SB	0.666375	0.632912	0.703577	892.0
SR	0.591179	0.630211	0.556824	681.0
macro avg	0.647581	0.657703	0.640300	2077.0
micro avg	0.641995	0.641995	0.641995	2077.0
weighted avg	0.640621	0.642691	0.641995	2077.0

The result of Model Number:Dataset Number:Featuregroup Number:Rescale of 9 : 0 : 8 : 1

	f1-score	precision	recall	support
AFIB	0.893685	0.901139	0.886661	204.0
GSVT	0.917293	0.913069	0.921576	300.0
SB	0.931127	0.927819	0.934464	892.0
SR	0.922527	0.926659	0.918480	681.0
macro avg	0.916158	0.917171	0.915295	2077.0
micro avg	0.922666	0.922666	0.922666	2077.0
weighted avg	0.922632	0.922688	0.922666	2077.0

The result of Model Number:Dataset Number:Featuregroup Number:Rescale of 9 : 0 : 9 : 0

	f1-score	precision	recall	support
AFIB	0.612235	0.676272	0.559455	204.0
GSVT	0.720252	0.710742	0.730096	300.0
SB	0.763824	0.760856	0.766821	892.0
SR	0.745470	0.734845	0.756414	681.0
macro avg	0.710445	0.720679	0.703197	2077.0
micro avg	0.737737	0.737737	0.737737	2077.0
weighted avg	0.736624	0.736782	0.737737	2077.0

The result of Model Number:Dataset Number:Featuregroup Number:Rescale of 9 : 0 : 9 : 1

	f1-score	precision	recall	support
AFIB	0.783278	0.760711	0.859407	875.0
GSVT	0.863277	0.860520	0.866164	691.0
SB	0.728733	0.717994	0.739858	1553.0
SR	0.872571	0.889602	0.856289	907.0
macro avg	0.811965	0.807207	0.830429	4026.0
micro avg	0.813749	0.813749	0.813749	4026.0
weighted avg	0.796085	0.790401	0.813749	4026.0

The result of Model Number:Dataset Number:Featuregroup Number:Rescale of 9 : 0 : 10 : 0

	f1-score	precision	recall	support
AFIB	0.648783	0.639797	0.658052	875.0
GSVT	0.663173	0.668540	0.657894	691.0
SB	0.703950	0.701927	0.705989	1553.0
SR	0.686825	0.695764	0.678119	907.0
macro avg	0.675683	0.676507	0.675013	4026.0
micro avg	0.681037	0.681037	0.681037	4026.0
weighted avg	0.681103	0.681305	0.681037	4026.0

The result of Model Number:Dataset Number:Featuregroup Number:Rescale of 9 : 0 : 10 : 1

	f1-score	precision	recall	support
AFIB	0.757965	0.782587	0.734989	204.0
GSVT	0.772334	0.765420	0.779527	300.0
SB	0.790699	0.785279	0.796195	892.0
SR	0.777803	0.781085	0.774595	681.0
macro avg	0.774700	0.778593	0.771326	2077.0
micro avg	0.780694	0.780694	0.780694	2077.0
weighted avg	0.780603	0.780771	0.780694	2077.0

The result of Model Number:Dataset Number:Featuregroup Number:Rescale of 9 : 1 : 0 : 0

	f1-score	precision	recall	support
AFIB	0.677390	0.677053	0.677759	875.0
GSVT	0.685817	0.690405	0.681343	691.0
SB	0.727060	0.725389	0.728742	1553.0
SR	0.706001	0.705834	0.706291	907.0
macro avg	0.699067	0.699670	0.698534	4026.0
micro avg	0.704468	0.704468	0.704468	4026.0
weighted avg	0.704442	0.704474	0.704468	4026.0

The result of Model Number:Dataset Number:Featuregroup Number:Rescale of 9 : 1 : 0 : 1

	f1-score	precision	recall	support
AFIB	0.802915	0.808325	0.797696	875.0
GSVT	0.817244	0.805893	0.829070	691.0
SB	0.870863	0.864900	0.876916	1553.0

SR 0.835572 0.850005 0.821666 907.0
macro avg 0.831648 0.832280 0.831337 4026.0
micro avg 0.839040 0.839040 0.839040 4026.0
weighted avg 0.838942 0.839121 0.839040 4026.0
The result of Model Number:Dataset Number:Featuregroup Number:Rescale of 9 : 1 : 1 : 0
f1-score precision recall support
AFIB 0.853725 0.855996 0.851518 875.0
GSVT 0.870142 0.864012 0.876411 691.0
SB 0.919843 0.910464 0.929435 1553.0
SR 0.877795 0.896915 0.859505 907.0
macro avg 0.880376 0.881847 0.879217 4026.0
micro avg 0.887646 0.887646 0.887646 4026.0
weighted avg 0.887470 0.887601 0.887646 4026.0
The result of Model Number:Dataset Number:Featuregroup Number:Rescale of 9 : 1 : 1 : 1
f1-score precision recall support
AFIB 0.739600 0.755846 0.724314 204.0
GSVT 0.763435 0.757265 0.769812 300.0
SB 0.779435 0.778477 0.780397 892.0
SR 0.772855 0.772183 0.773533 681.0
macro avg 0.763831 0.765942 0.762014 2077.0
micro avg 0.771109 0.771109 0.771109 2077.0
weighted avg 0.771054 0.771126 0.771109 2077.0
The result of Model Number:Dataset Number:Featuregroup Number:Rescale of 9 : 1 : 2 : 0
f1-score precision recall support
AFIB 0.707260 0.730235 0.685820 204.0
GSVT 0.720667 0.714216 0.727379 300.0
SB 0.737804 0.732747 0.742932 892.0
SR 0.725771 0.728833 0.722777 681.0
macro avg 0.722876 0.726508 0.719727 2077.0
micro avg 0.728468 0.728468 0.728468 2077.0
weighted avg 0.728383 0.728540 0.728468 2077.0
The result of Model Number:Dataset Number:Featuregroup Number:Rescale of 9 : 1 : 2 : 1
f1-score precision recall support
AFIB 0.844666 0.881248 0.811240 204.0
GSVT 0.789616 0.796985 0.782778 300.0
SB 0.818494 0.778253 0.863181 892.0
SR 0.729500 0.775537 0.688858 681.0
macro avg 0.795569 0.808006 0.786514 2077.0
micro avg 0.789310 0.789310 0.789310 2077.0
weighted avg 0.787714 0.790184 0.789310 2077.0
The result of Model Number:Dataset Number:Featuregroup Number:Rescale of 9 : 1 : 3 : 0
f1-score precision recall support
AFIB 0.693953 0.706832 0.681708 204.0

GSVT	0.709212	0.705494	0.712985	300.0
SB	0.720535	0.717974	0.723119	892.0
SR	0.713117	0.714402	0.711867	681.0
macro avg	0.709204	0.711175	0.707420	2077.0
micro avg	0.713899	0.713899	0.713899	2077.0
weighted avg	0.713857	0.713906	0.713899	2077.0

The result of Model Number:Dataset Number:Featuregroup Number:Rescale of 9 : 1 : 3 : 1

	f1-score	precision	recall	support
AFIB	0.690008	0.673926	0.706987	875.0
GSVT	0.662383	0.669664	0.655303	691.0
SB	0.697052	0.652041	0.748755	1553.0
SR	0.557881	0.669908	0.478253	907.0
macro avg	0.651831	0.666385	0.647324	4026.0
micro avg	0.662697	0.662697	0.662697	4026.0
weighted avg	0.658217	0.663847	0.662697	4026.0

The result of Model Number:Dataset Number:Featuregroup Number:Rescale of 9 : 1 : 4 : 0

	f1-score	precision	recall	support
AFIB	0.758219	0.740548	0.776877	875.0
GSVT	0.727864	0.735865	0.720084	691.0
SB	0.765960	0.716499	0.822774	1553.0
SR	0.613032	0.736133	0.525531	907.0
macro avg	0.716269	0.732261	0.711317	4026.0
micro avg	0.728209	0.728209	0.728209	4026.0
weighted avg	0.723287	0.729473	0.728209	4026.0

The result of Model Number:Dataset Number:Featuregroup Number:Rescale of 9 : 1 : 4 : 1

	f1-score	precision	recall	support
AFIB	0.849303	0.851562	0.847108	875.0
GSVT	0.865635	0.859537	0.871872	691.0
SB	0.915078	0.905748	0.924621	1553.0
SR	0.873249	0.892270	0.855053	907.0
macro avg	0.875816	0.877279	0.874663	4026.0
micro avg	0.883048	0.883048	0.883048	4026.0
weighted avg	0.882873	0.883004	0.883048	4026.0

The result of Model Number:Dataset Number:Featuregroup Number:Rescale of 9 : 1 : 5 : 0

	f1-score	precision	recall	support
AFIB	0.877248	0.889332	0.865874	875.0
GSVT	0.896399	0.892687	0.900385	691.0
SB	0.954805	0.942286	0.967701	1553.0
SR	0.924828	0.938639	0.911828	907.0
macro avg	0.913320	0.915736	0.911447	4026.0
micro avg	0.921429	0.921429	0.921429	4026.0
weighted avg	0.921171	0.921443	0.921429	4026.0

The result of Model Number:Dataset Number:Featuregroup Number:Rescale of 9 : 1 : 5 : 1

	f1-score	precision	recall	support
AFIB	0.830809	0.830759	0.830911	875.0
GSVT	0.832607	0.828106	0.837172	691.0
SB	0.872384	0.866579	0.878278	1553.0
SR	0.849219	0.863152	0.835760	907.0
macro avg	0.846255	0.847149	0.845530	4026.0
micro avg	0.851350	0.851350	0.851350	4026.0
weighted avg	0.851303	0.851419	0.851350	4026.0

The result of Model Number:Dataset Number:Featuregroup Number:Rescale of 9 : 1 : 6 : 0

	f1-score	precision	recall	support
AFIB	0.877319	0.893414	0.861861	204.0
GSVT	0.888890	0.879919	0.898063	300.0
SB	0.910785	0.906044	0.915582	892.0
SR	0.898386	0.904015	0.892846	681.0
macro avg	0.893845	0.895848	0.892088	2077.0
micro avg	0.900321	0.900321	0.900321	2077.0
weighted avg	0.900270	0.900365	0.900321	2077.0

The result of Model Number:Dataset Number:Featuregroup Number:Rescale of 9 : 1 : 6 : 1

	f1-score	precision	recall	support
AFIB	0.674302	0.696204	0.653870	204.0
GSVT	0.692919	0.684084	0.702125	300.0
SB	0.712468	0.709781	0.715183	892.0
SR	0.702503	0.703775	0.701263	681.0
macro avg	0.695548	0.698461	0.693110	2077.0
micro avg	0.702711	0.702711	0.702711	2077.0
weighted avg	0.702629	0.702767	0.702711	2077.0

The result of Model Number:Dataset Number:Featuregroup Number:Rescale of 9 : 1 : 7 : 0

	f1-score	precision	recall	support
AFIB	0.746743	0.718195	0.793716	875.0
GSVT	0.773752	0.747048	0.809973	691.0
SB	0.862104	0.846887	0.878825	1553.0
SR	0.730182	0.878002	0.630414	907.0
macro avg	0.778195	0.797533	0.778232	4026.0
micro avg	0.792547	0.792547	0.792547	4026.0
weighted avg	0.792148	0.808791	0.792547	4026.0

The result of Model Number:Dataset Number:Featuregroup Number:Rescale of 9 : 1 : 7 : 1

	f1-score	precision	recall	support
AFIB	0.676000	0.682235	0.670041	204.0
GSVT	0.700714	0.700739	0.700708	300.0
SB	0.715389	0.714119	0.716671	892.0
SR	0.707797	0.707607	0.708004	681.0
macro avg	0.699975	0.701175	0.698856	2077.0
micro avg	0.706944	0.706944	0.706944	2077.0

weighted avg	0.706912	0.706920	0.706944	2077.0

The result of Model Number:Dataset Number:Featuregroup Number:Rescale of 9 : 1 : 8 : 0

	f1-score	precision	recall	support
AFIB	0.757027	0.757387	0.756775	875.0
GSVT	0.776632	0.769910	0.783500	691.0
SB	0.829446	0.823401	0.835587	1553.0
SR	0.794011	0.809795	0.778871	907.0
macro avg	0.789279	0.790123	0.788683	4026.0
micro avg	0.796741	0.796741	0.796741	4026.0
weighted avg	0.796659	0.796808	0.796741	4026.0

The result of Model Number:Dataset Number:Featuregroup Number:Rescale of 9 : 1 : 8 : 1

	f1-score	precision	recall	support
AFIB	0.786749	0.801188	0.773200	204.0
GSVT	0.816615	0.810049	0.823438	300.0
SB	0.831763	0.830279	0.833255	892.0
SR	0.823528	0.824261	0.822800	681.0
macro avg	0.814664	0.816444	0.813173	2077.0
micro avg	0.822511	0.822511	0.822511	2077.0
weighted avg	0.822454	0.822526	0.822511	2077.0

The result of Model Number:Dataset Number:Featuregroup Number:Rescale of 9 : 1 : 9 : 0

	f1-score	precision	recall	support
AFIB	0.744780	0.749226	0.740407	204.0
GSVT	0.763080	0.759803	0.766386	300.0
SB	0.776149	0.775025	0.777279	892.0
SR	0.769052	0.770640	0.767474	681.0
macro avg	0.763265	0.763674	0.762886	2077.0
micro avg	0.768869	0.768869	0.768869	2077.0
weighted avg	0.768853	0.768855	0.768869	2077.0

The result of Model Number:Dataset Number:Featuregroup Number:Rescale of 9 : 1 : 9 : 1

	f1-score	precision	recall	support
AFIB	0.794868	0.798637	0.791141	875.0
GSVT	0.804729	0.800932	0.808580	691.0
SB	0.848801	0.847009	0.850605	1553.0
SR	0.832716	0.835048	0.830417	907.0
macro avg	0.820279	0.820406	0.820186	4026.0
micro avg	0.825920	0.825920	0.825920	4026.0
weighted avg	0.825891	0.825893	0.825920	4026.0

The result of Model Number:Dataset Number:Featuregroup Number:Rescale of 9 : 1 : 10 : 0

	f1-score	precision	recall	support
AFIB	0.751451	0.758746	0.744760	204.0
GSVT	0.773976	0.770318	0.777874	300.0
SB	0.789649	0.788768	0.790534	892.0

SR 0.782701 0.783512 0.781897 681.0
macro avg 0.774445 0.775336 0.773766 2077.0
micro avg 0.781378 0.781378 0.781378 2077.0
weighted avg 0.781356 0.781431 0.781378 2077.0
The result of Model Number:Dataset Number:Featuregroup Number:Rescale of 9 : 1 : 10 : 1

	f1-score	precision	recall	support
AFIB	0.827672	0.821299	0.834208	875.0
GSVT	0.838418	0.842311	0.834744	691.0
SB	0.898481	0.893702	0.903332	1553.0
SR	0.872009	0.884269	0.860181	907.0
macro avg	0.859145	0.860395	0.858116	4026.0
micro avg	0.866815	0.866815	0.866815	4026.0
weighted avg	0.866819	0.867020	0.866815	4026.0

The result of Model Number:Dataset Number:Featuregroup Number:Rescale of 10 : 0 : 0 : 0

	f1-score	precision	recall	support
AFIB	0.852900	0.846991	0.859064	875.0
GSVT	0.866058	0.869573	0.862712	691.0
SB	0.939675	0.929904	0.949658	1553.0
SR	0.892230	0.912991	0.872474	907.0
macro avg	0.887716	0.889865	0.885977	4026.0
micro avg	0.897657	0.897657	0.897657	4026.0
weighted avg	0.897492	0.897719	0.897657	4026.0

The result of Model Number:Dataset Number:Featuregroup Number:Rescale of 10 : 0 : 0 : 1

	f1-score	precision	recall	support
AFIB	0.862434	0.869956	0.855256	204.0
GSVT	0.892260	0.890803	0.893743	300.0
SB	0.911225	0.909003	0.913470	892.0
SR	0.902871	0.904232	0.901545	681.0
macro avg	0.892197	0.893498	0.891003	2077.0
micro avg	0.900993	0.900993	0.900993	2077.0
weighted avg	0.900955	0.900975	0.900993	2077.0

The result of Model Number:Dataset Number:Featuregroup Number:Rescale of 10 : 0 : 1 : 0

	f1-score	precision	recall	support
AFIB	0.649452	0.649128	0.649805	875.0
GSVT	0.657531	0.661930	0.653242	691.0
SB	0.697073	0.695471	0.698686	1553.0
SR	0.676883	0.676722	0.677161	907.0
macro avg	0.670235	0.670813	0.669724	4026.0
micro avg	0.675413	0.675413	0.675413	4026.0
weighted avg	0.675388	0.675419	0.675413	4026.0

The result of Model Number:Dataset Number:Featuregroup Number:Rescale of 10 : 0 : 1 : 1

	f1-score	precision	recall	support
AFIB	0.738567	0.765834	0.713243	204.0
GSVT	0.754353	0.746611	0.762374	300.0
SB	0.775014	0.771400	0.778663	892.0
SR	0.762930	0.763408	0.762478	681.0
macro avg	0.757716	0.761813	0.754190	2077.0
micro avg	0.764578	0.764578	0.764578	2077.0
weighted avg	0.764488	0.764652	0.764578	2077.0

The result of Model Number:Dataset Number:Featuregroup Number:Rescale of 10 : 0 : 2 : 0

	f1-score	precision	recall	support
AFIB	0.783119	0.804286	0.763201	204.0
GSVT	0.789574	0.777951	0.801658	300.0
SB	0.804869	0.796306	0.813620	892.0
SR	0.789940	0.800795	0.779389	681.0
macro avg	0.791876	0.794834	0.789467	2077.0
micro avg	0.795717	0.795717	0.795717	2077.0
weighted avg	0.795629	0.795910	0.795717	2077.0

The result of Model Number:Dataset Number:Featuregroup Number:Rescale of 10 : 0 : 2 : 1

	f1-score	precision	recall	support
AFIB	0.773266	0.774564	0.792432	204.0
GSVT	0.765996	0.795722	0.739400	300.0
SB	0.812506	0.767947	0.862887	892.0
SR	0.704269	0.769263	0.651185	681.0
macro avg	0.764009	0.776874	0.761476	2077.0
micro avg	0.768718	0.768718	0.768718	2077.0
weighted avg	0.766446	0.773040	0.768718	2077.0

The result of Model Number:Dataset Number:Featuregroup Number:Rescale of 10 : 0 : 3 : 0

	f1-score	precision	recall	support
AFIB	0.856860	0.867381	0.846724	204.0
GSVT	0.874119	0.870385	0.877905	300.0
SB	0.884700	0.882044	0.887375	892.0
SR	0.877253	0.879341	0.875203	681.0
macro avg	0.873233	0.874788	0.871802	2077.0
micro avg	0.878024	0.878024	0.878024	2077.0
weighted avg	0.877995	0.878034	0.878024	2077.0

The result of Model Number:Dataset Number:Featuregroup Number:Rescale of 10 : 0 : 3 : 1

	f1-score	precision	recall	support
AFIB	0.716645	0.743215	0.692012	204.0

GSVT	0.738115	0.728885	0.747674	300.0
SB	0.756832	0.749926	0.763872	892.0
SR	0.736934	0.742653	0.731310	681.0
macro avg	0.737131	0.741170	0.733717	2077.0
micro avg	0.743798	0.743798	0.743798	2077.0
weighted avg	0.743657	0.743843	0.743798	2077.0

The result of Model Number:Dataset Number:Featuregroup Number:Rescale of 10 : 0 : 4 : 0

	f1-score	precision	recall	support
AFIB	0.687307	0.687127	0.687553	875.0
GSVT	0.701665	0.699012	0.704413	691.0
SB	0.747028	0.743655	0.750443	1553.0
SR	0.721852	0.730099	0.713817	907.0
macro avg	0.714463	0.714973	0.714056	4026.0
micro avg	0.720623	0.720623	0.720623	4026.0
weighted avg	0.720591	0.720653	0.720623	4026.0

The result of Model Number:Dataset Number:Featuregroup Number:Rescale of 10 : 0 : 4 : 1

	f1-score	precision	recall	support
AFIB	0.762839	0.754322	0.771559	875.0
GSVT	0.778016	0.785142	0.771030	691.0
SB	0.829790	0.827088	0.832513	1553.0
SR	0.808279	0.816376	0.800343	907.0
macro avg	0.794731	0.795732	0.793862	4026.0
micro avg	0.801466	0.801466	0.801466	4026.0
weighted avg	0.801507	0.801661	0.801466	4026.0

The result of Model Number:Dataset Number:Featuregroup Number:Rescale of 10 : 0 : 5 : 0

	f1-score	precision	recall	support
AFIB	0.786598	0.789305	0.784009	875.0
GSVT	0.801531	0.785540	0.818332	691.0
SB	0.848485	0.843897	0.853129	1553.0
SR	0.815667	0.834632	0.797594	907.0
macro avg	0.813070	0.813343	0.813266	4026.0
micro avg	0.819623	0.819623	0.819623	4026.0
weighted avg	0.819583	0.819929	0.819623	4026.0

The result of Model Number:Dataset Number:Featuregroup Number:Rescale of 10 : 0 : 5 : 1

	f1-score	precision	recall	support
AFIB	0.646059	0.715844	0.588790	204.0
GSVT	0.748920	0.736740	0.761628	300.0
SB	0.798677	0.795041	0.802348	892.0
SR	0.779379	0.769462	0.789567	681.0
macro avg	0.743259	0.754272	0.735583	2077.0

micro avg 0.771301 0.771301 0.771301 2077.0
weighted avg 0.770173 0.770455 0.771301 2077.0
The result of Model Number:Dataset Number:Featuregroup Number:Rescale of 10 : 0 : 6 : 0

	f1-score	precision	recall	support
AFIB	0.737178	0.739868	0.734556	875.0
GSVT	0.749919	0.740154	0.760009	691.0
SB	0.790490	0.781784	0.799398	1553.0
SR	0.749158	0.769853	0.729587	907.0
macro avg	0.756686	0.757915	0.755888	4026.0
micro avg	0.762818	0.762818	0.762818	4026.0
weighted avg	0.762628	0.762841	0.762818	4026.0

The result of Model Number:Dataset Number:Featuregroup Number:Rescale of 10 : 0 : 6 : 1

	f1-score	precision	recall	support
AFIB	0.822452	0.836125	0.809407	204.0
GSVT	0.838222	0.832267	0.844326	300.0
SB	0.857664	0.852844	0.862559	892.0
SR	0.846738	0.851918	0.841647	681.0
macro avg	0.841269	0.843289	0.839485	2077.0
micro avg	0.847848	0.847848	0.847848	2077.0
weighted avg	0.847815	0.847926	0.847848	2077.0

The result of Model Number:Dataset Number:Featuregroup Number:Rescale of 10 : 0 : 7 : 0

	f1-score	precision	recall	support
AFIB	0.672146	0.700209	0.646368	875.0
GSVT	0.726152	0.722476	0.729954	691.0
SB	0.796248	0.791767	0.800802	1553.0
SR	0.745567	0.729011	0.762904	907.0
macro avg	0.735028	0.735866	0.735007	4026.0
micro avg	0.746540	0.746540	0.746540	4026.0
weighted avg	0.745828	0.745837	0.746540	4026.0

The result of Model Number:Dataset Number:Featuregroup Number:Rescale of 10 : 0 : 7 : 1

	f1-score	precision	recall	support
AFIB	0.928799	0.938700	0.919257	204.0
GSVT	0.945880	0.940692	0.951232	300.0
SB	0.967714	0.966101	0.969339	892.0
SR	0.959647	0.961211	0.958102	681.0
macro avg	0.950510	0.951676	0.949482	2077.0
micro avg	0.958120	0.958120	0.958120	2077.0
weighted avg	0.958093	0.958136	0.958120	2077.0

The result of Model Number:Dataset Number:Featuregroup Number:Rescale of 10 : 0 : 8 : 0

	f1-score	precision	recall	support
AFIB	0.891804	0.890987	0.892728	875.0
GSVT	0.910083	0.908318	0.911951	691.0
SB	0.965717	0.963566	0.967892	1553.0
SR	0.939358	0.945486	0.933343	907.0
macro avg	0.926740	0.927089	0.926479	4026.0
micro avg	0.934171	0.934171	0.934171	4026.0
weighted avg	0.934166	0.934236	0.934171	4026.0

The result of Model Number:Dataset Number:Featuregroup Number:Rescale of 10 : 0 : 8 : 1

	f1-score	precision	recall	support
AFIB	0.776085	0.764185	0.788490	875.0
GSVT	0.802845	0.810715	0.795285	691.0
SB	0.854442	0.852446	0.856474	1553.0
SR	0.833097	0.843661	0.822814	907.0
macro avg	0.816617	0.817752	0.815766	4026.0
micro avg	0.823613	0.823613	0.823613	4026.0
weighted avg	0.823748	0.824122	0.823613	4026.0

The result of Model Number:Dataset Number:Featuregroup Number:Rescale of 10 : 0 : 9 : 0

	f1-score	precision	recall	support
AFIB	0.746454	0.739624	0.753443	875.0
GSVT	0.756018	0.762689	0.749576	691.0
SB	0.803623	0.799386	0.807916	1553.0
SR	0.785457	0.794922	0.776285	907.0
macro avg	0.772888	0.774155	0.771805	4026.0
micro avg	0.778938	0.778938	0.778938	4026.0
weighted avg	0.778935	0.779094	0.778938	4026.0

The result of Model Number:Dataset Number:Featuregroup Number:Rescale of 10 : 0 : 9 : 1

	f1-score	precision	recall	support
AFIB	0.866355	0.882276	0.851104	204.0
GSVT	0.880907	0.875138	0.886776	300.0
SB	0.895975	0.891601	0.900395	892.0
SR	0.886714	0.890401	0.883071	681.0
macro avg	0.882488	0.884854	0.880336	2077.0
micro avg	0.887906	0.887906	0.887906	2077.0
weighted avg	0.887853	0.887914	0.887906	2077.0

The result of Model Number:Dataset Number:Featuregroup Number:Rescale of 10 : 0 : 10 : 0

	f1-score	precision	recall	support
AFIB	0.843985	0.878050	0.812531	204.0
GSVT	0.875392	0.861172	0.890241	300.0
SB	0.898039	0.890819	0.905388	892.0

SR 0.873878 0.880171 0.867721 681.0
macro avg 0.872823 0.877553 0.868970 2077.0
micro avg 0.881730 0.881730 0.881730 2077.0
weighted avg 0.881537 0.881791 0.881730 2077.0

The result of Model Number:Dataset Number:Featuregroup Number:Rescale of 10 : 0 : 10 : 1

	f1-score	precision	recall	support
AFIB	0.870632	0.862470	0.879061	875.0
GSVT	0.825443	0.805603	0.886122	691.0
SB	0.954471	0.950965	0.958028	1553.0
SR	0.742370	0.747576	0.737246	907.0
macro avg	0.848229	0.841654	0.865114	4026.0
micro avg	0.878785	0.878785	0.878785	4026.0
weighted avg	0.866321	0.860962	0.878785	4026.0

The result of Model Number:Dataset Number:Featuregroup Number:Rescale of 10 : 1 : 0 : 0

	f1-score	precision	recall	support
AFIB	0.819481	0.823780	0.815276	875.0
GSVT	0.836616	0.835114	0.838184	691.0
SB	0.908422	0.901455	0.915499	1553.0
SR	0.876372	0.885144	0.867778	907.0
macro avg	0.860223	0.861373	0.859184	4026.0
micro avg	0.869696	0.869696	0.869696	4026.0
weighted avg	0.869547	0.869512	0.869696	4026.0

The result of Model Number:Dataset Number:Featuregroup Number:Rescale of 10 : 1 : 0 : 1

	f1-score	precision	recall	support
AFIB	0.700016	0.699973	0.700102	875.0
GSVT	0.701531	0.697738	0.705377	691.0
SB	0.735045	0.730154	0.740012	1553.0
SR	0.715527	0.727267	0.704187	907.0
macro avg	0.713030	0.713783	0.712419	4026.0
micro avg	0.717322	0.717322	0.717322	4026.0
weighted avg	0.717283	0.717381	0.717322	4026.0

The result of Model Number:Dataset Number:Featuregroup Number:Rescale of 10 : 1 : 1 : 0

	f1-score	precision	recall	support
AFIB	0.869098	0.869511	0.868809	875.0
GSVT	0.891605	0.883888	0.899490	691.0
SB	0.952238	0.945299	0.959288	1553.0
SR	0.911557	0.929678	0.894176	907.0
macro avg	0.906124	0.907094	0.905441	4026.0
micro avg	0.914691	0.914691	0.914691	4026.0
weighted avg	0.914597	0.914768	0.914691	4026.0

The result of Model Number:Dataset Number:Featuregroup Number:Rescale of 10 : 1 : 1 : 1

	f1-score	precision	recall	support
AFIB	0.653933	0.639959	0.668569	875.0
GSVT	0.677190	0.687545	0.667202	691.0
SB	0.721883	0.721978	0.721790	1553.0
SR	0.703285	0.710568	0.696152	907.0
macro avg	0.689073	0.690013	0.688428	4026.0
micro avg	0.695078	0.695078	0.695078	4026.0
weighted avg	0.695254	0.695672	0.695078	4026.0

The result of Model Number:Dataset Number:Featuregroup Number:Rescale of 10 : 1 : 2 : 0

	f1-score	precision	recall	support
AFIB	0.699065	0.678925	0.767009	875.0
GSVT	0.770464	0.768003	0.773040	691.0
SB	0.650385	0.640800	0.660314	1553.0
SR	0.778758	0.793958	0.764227	907.0
macro avg	0.724668	0.720422	0.741148	4026.0
micro avg	0.726261	0.726261	0.726261	4026.0
weighted avg	0.710496	0.705423	0.726261	4026.0

The result of Model Number:Dataset Number:Featuregroup Number:Rescale of 10 : 1 : 2 : 1

	f1-score	precision	recall	support
AFIB	0.719223	0.722996	0.715533	875.0
GSVT	0.734262	0.732943	0.735638	691.0
SB	0.797283	0.791169	0.803494	1553.0
SR	0.769154	0.776853	0.761611	907.0
macro avg	0.754981	0.755990	0.754069	4026.0
micro avg	0.763295	0.763295	0.763295	4026.0
weighted avg	0.763164	0.763134	0.763295	4026.0

The result of Model Number:Dataset Number:Featuregroup Number:Rescale of 10 : 1 : 3 : 0

	f1-score	precision	recall	support
AFIB	0.851319	0.851441	0.851309	875.0
GSVT	0.866187	0.859007	0.873553	691.0
SB	0.906886	0.899041	0.914884	1553.0
SR	0.877002	0.896814	0.858077	907.0
macro avg	0.875349	0.876576	0.874456	4026.0
micro avg	0.881175	0.881175	0.881175	4026.0
weighted avg	0.881092	0.881323	0.881175	4026.0

The result of Model Number:Dataset Number:Featuregroup Number:Rescale of 10 : 1 : 3 : 1

	f1-score	precision	recall	support
AFIB	0.832509	0.832242	0.832818	875.0

	f1-score	precision	recall	support
GSVT	0.844916	0.834055	0.856162	691.0
SB	0.893701	0.891757	0.895657	1553.0
SR	0.868974	0.881712	0.856613	907.0
macro avg	0.860025	0.859942	0.860313	4026.0
micro avg	0.866425	0.866425	0.866425	4026.0
weighted avg	0.866458	0.866656	0.866425	4026.0

The result of Model Number:Dataset Number:Featuregroup Number:Rescale of 10 : 1 : 4 : 0

	f1-score	precision	recall	support
AFIB	0.787152	0.769264	0.805946	875.0
GSVT	0.814128	0.827331	0.801407	691.0
SB	0.868586	0.868591	0.868586	1553.0
SR	0.846355	0.855997	0.836930	907.0
macro avg	0.829055	0.830296	0.828217	4026.0
micro avg	0.836310	0.836310	0.836310	4026.0
weighted avg	0.836532	0.837085	0.836310	4026.0

The result of Model Number:Dataset Number:Featuregroup Number:Rescale of 10 : 1 : 4 : 1

	f1-score	precision	recall	support
AFIB	0.830545	0.809292	0.853022	875.0
GSVT	0.785122	0.829620	0.745336	691.0
SB	0.832504	0.766104	0.911527	1553.0
SR	0.665030	0.807766	0.565283	907.0
macro avg	0.778300	0.803195	0.768792	4026.0
micro avg	0.792284	0.792284	0.792284	4026.0
weighted avg	0.786216	0.795778	0.792284	4026.0

The result of Model Number:Dataset Number:Featuregroup Number:Rescale of 10 : 1 : 5 : 0

	f1-score	precision	recall	support
AFIB	0.641516	0.703262	0.589957	204.0
GSVT	0.746086	0.738419	0.754052	300.0
SB	0.790908	0.787832	0.794010	892.0
SR	0.772583	0.761900	0.783584	681.0
macro avg	0.737773	0.747853	0.730401	2077.0
micro avg	0.764778	0.764778	0.764778	2077.0
weighted avg	0.763752	0.763886	0.764778	2077.0

The result of Model Number:Dataset Number:Featuregroup Number:Rescale of 10 : 1 : 5 : 1

	f1-score	precision	recall	support
AFIB	0.858370	0.834562	0.886277	875.0
GSVT	0.869575	0.906203	0.841144	691.0
SB	0.955259	0.954776	0.955754	1553.0
SR	0.931294	0.937122	0.925544	907.0
macro avg	0.903624	0.908166	0.902180	4026.0

micro avg 0.914177 0.914177 0.914177 4026.0
weighted avg 0.914096 0.916335 0.914177 4026.0
The result of Model Number:Dataset Number:Featuregroup Number:Rescale of 10 : 1 : 6 : 0

	f1-score	precision	recall	support
AFIB	0.625014	0.613330	0.637218	875.0
GSVT	0.646907	0.657120	0.637088	691.0
SB	0.687958	0.686729	0.689198	1553.0
SR	0.670471	0.677575	0.663520	907.0
macro avg	0.657588	0.658688	0.656756	4026.0
micro avg	0.663172	0.663172	0.663172	4026.0
weighted avg	0.663293	0.663632	0.663172	4026.0

The result of Model Number:Dataset Number:Featuregroup Number:Rescale of 10 : 1 : 6 : 1

	f1-score	precision	recall	support
AFIB	0.811917	0.867354	0.763487	204.0
GSVT	0.858277	0.840787	0.876585	300.0
SB	0.879565	0.871854	0.887447	892.0
SR	0.857869	0.860726	0.855108	681.0
macro avg	0.851907	0.860180	0.845657	2077.0
micro avg	0.863100	0.863100	0.863100	2077.0
weighted avg	0.862733	0.863276	0.863100	2077.0

The result of Model Number:Dataset Number:Featuregroup Number:Rescale of 10 : 1 : 7 : 0

	f1-score	precision	recall	support
AFIB	0.883426	0.929066	0.842322	204.0
GSVT	0.926347	0.917293	0.935603	300.0
SB	0.942681	0.935097	0.950394	892.0
SR	0.931037	0.932170	0.929944	681.0
macro avg	0.920873	0.928407	0.914566	2077.0
micro avg	0.930938	0.930938	0.930938	2077.0
weighted avg	0.930684	0.930974	0.930938	2077.0

The result of Model Number:Dataset Number:Featuregroup Number:Rescale of 10 : 1 : 7 : 1

	f1-score	precision	recall	support
AFIB	0.766834	0.754557	0.862843	204.0
GSVT	0.897760	0.887343	0.908752	300.0
SB	0.927820	0.924316	0.931356	892.0
SR	0.727984	0.731748	0.724383	681.0
macro avg	0.830099	0.824491	0.856833	2077.0
micro avg	0.853500	0.853500	0.853500	2077.0
weighted avg	0.842145	0.839164	0.853500	2077.0

The result of Model Number:Dataset Number:Featuregroup Number:Rescale of 10 : 1 : 8 : 0

	f1-score	precision	recall	support
AFIB	0.791556	0.832450	0.754727	204.0
GSVT	0.830014	0.821901	0.838307	300.0
SB	0.844649	0.837854	0.851560	892.0
SR	0.834216	0.835232	0.833237	681.0
macro avg	0.825109	0.831859	0.819458	2077.0
micro avg	0.834127	0.834127	0.834127	2077.0
weighted avg	0.833900	0.834159	0.834127	2077.0

The result of Model Number:Dataset Number:Featuregroup Number:Rescale of 10 : 1 : 8 : 1

	f1-score	precision	recall	support
AFIB	0.811532	0.830809	0.793203	204.0
GSVT	0.838189	0.830330	0.846286	300.0
SB	0.856190	0.855232	0.857150	892.0
SR	0.847926	0.847074	0.848793	681.0
macro avg	0.838459	0.840861	0.836358	2077.0
micro avg	0.846560	0.846560	0.846560	2077.0
weighted avg	0.846494	0.846561	0.846560	2077.0

The result of Model Number:Dataset Number:Featuregroup Number:Rescale of 10 : 1 : 9 : 0

	f1-score	precision	recall	support
AFIB	0.856966	0.903937	0.815205	204.0
GSVT	0.816014	0.844623	0.789379	300.0
SB	0.839350	0.806977	0.874443	892.0
SR	0.747044	0.767312	0.727863	681.0
macro avg	0.814844	0.830712	0.801723	2077.0
micro avg	0.808278	0.808278	0.808278	2077.0
weighted avg	0.807445	0.808933	0.808278	2077.0

The result of Model Number:Dataset Number:Featuregroup Number:Rescale of 10 : 1 : 9 : 1

	f1-score	precision	recall	support
AFIB	0.822928	0.840189	0.806384	204.0
GSVT	0.842018	0.837036	0.847068	300.0
SB	0.862546	0.860526	0.864576	892.0
SR	0.855260	0.855014	0.855514	681.0
macro avg	0.845688	0.848191	0.843386	2077.0
micro avg	0.853360	0.853360	0.853360	2077.0
weighted avg	0.853301	0.853328	0.853360	2077.0

The result of Model Number:Dataset Number:Featuregroup Number:Rescale of 10 : 1 : 10 : 0

	f1-score	precision	recall	support
AFIB	0.850021	0.855095	0.845030	204.0
GSVT	0.870907	0.867168	0.874681	300.0
SB	0.885822	0.884540	0.887112	892.0

SR	0.877723	0.879536	0.875922	681.0
macro avg	0.871119	0.871585	0.870686	2077.0
micro avg	0.877514	0.877514	0.877514	2077.0
weighted avg	0.877496	0.877498	0.877514	2077.0

The result of Model Number:Dataset Number:Featuregroup Number:Rescale of 10 : 1 : 10 : 1

	f1-score	precision	recall	support
AFIB	0.732276	0.735642	0.728977	875.0
GSVT	0.701839	0.706067	0.697684	691.0
SB	0.752837	0.719279	0.789716	1553.0
SR	0.643840	0.700904	0.595498	907.0
macro avg	0.707698	0.715473	0.702969	4026.0
micro avg	0.716965	0.716965	0.716965	4026.0
weighted avg	0.715060	0.716428	0.716965	4026.0

The result of Model Number:Dataset Number:Featuregroup Number:Rescale of 11 : 0 : 0 : 0

	f1-score	precision	recall	support
AFIB	0.693109	0.693233	0.693015	875.0
GSVT	0.700442	0.702808	0.698136	691.0
SB	0.735778	0.733241	0.738336	1553.0
SR	0.721806	0.724241	0.719415	907.0
macro avg	0.712784	0.713381	0.712225	4026.0
micro avg	0.717324	0.717324	0.717324	4026.0
weighted avg	0.717292	0.717295	0.717324	4026.0

The result of Model Number:Dataset Number:Featuregroup Number:Rescale of 11 : 0 : 0 : 1

	f1-score	precision	recall	support
AFIB	0.634199	0.624650	0.649325	875.0
GSVT	0.605546	0.603783	0.610711	691.0
SB	0.646645	0.602570	0.703054	1553.0
SR	0.479010	0.640294	0.396917	907.0
macro avg	0.591350	0.617824	0.590002	4026.0
micro avg	0.606559	0.606559	0.606559	4026.0
weighted avg	0.599120	0.616076	0.606559	4026.0

The result of Model Number:Dataset Number:Featuregroup Number:Rescale of 11 : 0 : 1 : 0

	f1-score	precision	recall	support
AFIB	0.742214	0.747513	0.737122	204.0
GSVT	0.768167	0.767671	0.768677	300.0
SB	0.783536	0.781798	0.785290	892.0
SR	0.776532	0.777472	0.775619	681.0
macro avg	0.767612	0.768614	0.766677	2077.0
micro avg	0.774989	0.774989	0.774989	2077.0
weighted avg	0.774961	0.774972	0.774989	2077.0

The result of Model Number:Dataset Number:Featuregroup Number:Rescale of 11 : 0 : 1 : 1

	f1-score	precision	recall	support
AFIB	0.764517	0.754756	0.774578	875.0
GSVT	0.777936	0.782397	0.773546	691.0
SB	0.832732	0.829235	0.836267	1553.0
SR	0.808213	0.821295	0.795556	907.0
macro avg	0.795849	0.796921	0.794987	4026.0
micro avg	0.802923	0.802923	0.802923	4026.0
weighted avg	0.802978	0.803220	0.802923	4026.0

The result of Model Number:Dataset Number:Featuregroup Number:Rescale of 11 : 0 : 2 : 0

	f1-score	precision	recall	support
AFIB	0.777129	0.771745	0.782745	875.0
GSVT	0.789118	0.792321	0.786070	691.0
SB	0.856195	0.847292	0.865291	1553.0
SR	0.812965	0.831881	0.794964	907.0
macro avg	0.808852	0.810810	0.807267	4026.0
micro avg	0.817910	0.817910	0.817910	4026.0
weighted avg	0.817759	0.817966	0.817910	4026.0

The result of Model Number:Dataset Number:Featuregroup Number:Rescale of 11 : 0 : 2 : 1

	f1-score	precision	recall	support
AFIB	0.768360	0.810475	0.730917	204.0
GSVT	0.731642	0.757294	0.707762	300.0
SB	0.752566	0.723540	0.784030	892.0
SR	0.669804	0.687976	0.652606	681.0
macro avg	0.730593	0.744821	0.718829	2077.0
micro avg	0.724706	0.724706	0.724706	2077.0
weighted avg	0.723959	0.725293	0.724706	2077.0

The result of Model Number:Dataset Number:Featuregroup Number:Rescale of 11 : 0 : 3 : 0

	f1-score	precision	recall	support
AFIB	0.868831	0.895590	0.843798	204.0
GSVT	0.890996	0.880089	0.902274	300.0
SB	0.915197	0.909759	0.920731	892.0
SR	0.899770	0.904208	0.895411	681.0
macro avg	0.893698	0.897411	0.890553	2077.0
micro avg	0.902207	0.902207	0.902207	2077.0
weighted avg	0.902089	0.902262	0.902207	2077.0

The result of Model Number:Dataset Number:Featuregroup Number:Rescale of 11 : 0 : 3 : 1

	f1-score	precision	recall	support
AFIB	0.854662	0.843670	0.866004	875.0

GSVT 0.872059 0.879279 0.864993 691.0
SB 0.932407 0.928257 0.936605 1553.0
SR 0.905873 0.919271 0.892881 907.0
macro avg 0.891250 0.892619 0.890121 4026.0
micro avg 0.899119 0.899119 0.899119 4026.0
weighted avg 0.899174 0.899442 0.899119 4026.0
The result of Model Number:Dataset Number:Featuregroup Number:Rescale of 11 : 0 : 4 : 0
f1-score precision recall support
AFIB 0.747019 0.725497 0.819623 875.0
GSVT 0.823315 0.820685 0.826068 691.0
SB 0.694999 0.684757 0.705609 1553.0
SR 0.832178 0.848421 0.816650 907.0
macro avg 0.774378 0.769840 0.791987 4026.0
micro avg 0.776079 0.776079 0.776079 4026.0
weighted avg 0.759233 0.753812 0.776079 4026.0
The result of Model Number:Dataset Number:Featuregroup Number:Rescale of 11 : 0 : 4 : 1
f1-score precision recall support
AFIB 0.648304 0.642373 0.654374 875.0
GSVT 0.656611 0.662404 0.651016 691.0
SB 0.697956 0.694277 0.701685 1553.0
SR 0.682179 0.690400 0.674213 907.0
macro avg 0.671263 0.672363 0.670322 4026.0
micro avg 0.676517 0.676517 0.676517 4026.0
weighted avg 0.676514 0.676652 0.676517 4026.0
The result of Model Number:Dataset Number:Featuregroup Number:Rescale of 11 : 0 : 5 : 0
f1-score precision recall support
AFIB 0.654681 0.639423 0.670792 875.0
GSVT 0.628471 0.635379 0.621754 691.0
SB 0.661365 0.618658 0.710421 1553.0
SR 0.529320 0.635611 0.453768 907.0
macro avg 0.618459 0.632268 0.614184 4026.0
micro avg 0.628769 0.628769 0.628769 4026.0
weighted avg 0.624519 0.629860 0.628769 4026.0
The result of Model Number:Dataset Number:Featuregroup Number:Rescale of 11 : 0 : 5 : 1
f1-score precision recall support
AFIB 0.647901 0.653267 0.729772 204.0
GSVT 0.702605 0.672533 0.792703 300.0
SB 0.641504 0.639275 0.643751 892.0
SR 0.635126 0.635246 0.635010 681.0
macro avg 0.656784 0.650080 0.700309 2077.0

	f1-score	precision	recall	support
micro avg	0.670849	0.670849	0.670849	2077.0
weighted avg	0.648867	0.644132	0.670849	2077.0

The result of Model Number:Dataset Number:Featuregroup Number:Rescale of 11 : 0 : 6 : 0

	f1-score	precision	recall	support
AFIB	0.789091	0.791094	0.787127	875.0
GSVT	0.803016	0.794317	0.811941	691.0
SB	0.846335	0.836761	0.856143	1553.0
SR	0.807639	0.829750	0.786708	907.0
macro avg	0.811520	0.812980	0.810480	4026.0
micro avg	0.817914	0.817914	0.817914	4026.0
weighted avg	0.817741	0.817971	0.817914	4026.0

The result of Model Number:Dataset Number:Featuregroup Number:Rescale of 11 : 0 : 6 : 1

	f1-score	precision	recall	support
AFIB	0.740232	0.771539	0.711613	204.0
GSVT	0.681700	0.682894	0.690737	300.0
SB	0.733913	0.698950	0.773270	892.0
SR	0.648815	0.701072	0.604982	681.0
macro avg	0.701165	0.713614	0.695150	2077.0
micro avg	0.700115	0.700115	0.700115	2077.0
weighted avg	0.699090	0.704456	0.700115	2077.0

The result of Model Number:Dataset Number:Featuregroup Number:Rescale of 11 : 0 : 7 : 0

	f1-score	precision	recall	support
AFIB	0.692828	0.688684	0.697117	875.0
GSVT	0.698502	0.702273	0.694872	691.0
SB	0.740307	0.738143	0.742495	1553.0
SR	0.723579	0.728777	0.718469	907.0
macro avg	0.713804	0.714469	0.713238	4026.0
micro avg	0.719046	0.719046	0.719046	4026.0
weighted avg	0.719045	0.719127	0.719046	4026.0

The result of Model Number:Dataset Number:Featuregroup Number:Rescale of 11 : 0 : 7 : 1

	f1-score	precision	recall	support
AFIB	0.891026	0.914352	0.869266	204.0
GSVT	0.838394	0.862760	0.815822	300.0
SB	0.884684	0.853537	0.918208	892.0
SR	0.801859	0.827972	0.777503	681.0
macro avg	0.853991	0.864655	0.845200	2077.0
micro avg	0.852479	0.852479	0.852479	2077.0
weighted avg	0.851464	0.852460	0.852479	2077.0

The result of Model Number:Dataset Number:Featuregroup Number:Rescale of 11 : 0 : 8 : 0

	f1-score	precision	recall	support
AFIB	0.836237	0.842988	0.829665	204.0
GSVT	0.855425	0.851231	0.859691	300.0
SB	0.872120	0.871153	0.873097	892.0
SR	0.865608	0.866759	0.864466	681.0
macro avg	0.857348	0.858033	0.856730	2077.0
micro avg	0.864065	0.864065	0.864065	2077.0
weighted avg	0.864049	0.864069	0.864065	2077.0

The result of Model Number:Dataset Number:Featuregroup Number:Rescale of 11 : 0 : 8 : 1

	f1-score	precision	recall	support
AFIB	0.761729	0.776619	0.747533	204.0
GSVT	0.782660	0.777021	0.788408	300.0
SB	0.796667	0.793567	0.799793	892.0
SR	0.787959	0.790189	0.785759	681.0
macro avg	0.782254	0.784349	0.780373	2077.0
micro avg	0.788414	0.788414	0.788414	2077.0
weighted avg	0.788357	0.788405	0.788414	2077.0

The result of Model Number:Dataset Number:Featuregroup Number:Rescale of 11 : 0 : 9 : 0

	f1-score	precision	recall	support
AFIB	0.658271	0.656247	0.660350	875.0
GSVT	0.641491	0.644637	0.638478	691.0
SB	0.674387	0.646401	0.704912	1553.0
SR	0.580366	0.633212	0.535707	907.0
macro avg	0.638629	0.645124	0.634862	4026.0
micro avg	0.645705	0.645705	0.645705	4026.0
weighted avg	0.644057	0.645267	0.645705	4026.0

The result of Model Number:Dataset Number:Featuregroup Number:Rescale of 11 : 0 : 9 : 1

	f1-score	precision	recall	support
AFIB	0.850138	0.886958	0.816495	204.0
GSVT	0.794732	0.802148	0.787849	300.0
SB	0.823797	0.783295	0.868774	892.0
SR	0.734226	0.780562	0.693321	681.0
macro avg	0.800723	0.813241	0.791610	2077.0
micro avg	0.794423	0.794423	0.794423	2077.0
weighted avg	0.792818	0.795304	0.794423	2077.0

The result of Model Number:Dataset Number:Featuregroup Number:Rescale of 11 : 0 : 10 : 0

	f1-score	precision	recall	support
AFIB	0.709522	0.703030	0.716165	875.0
GSVT	0.718613	0.724953	0.712490	691.0
SB	0.763862	0.759835	0.767943	1553.0

SR	0.746595	0.755592	0.737877	907.0
macro avg	0.734648	0.735853	0.733619	4026.0
micro avg	0.740399	0.740399	0.740399	4026.0
weighted avg	0.740396	0.740547	0.740399	4026.0

The result of Model Number:Dataset Number:Featuregroup Number:Rescale of 11 : 0 : 10 : 1

	f1-score	precision	recall	support
AFIB	0.709307	0.755523	0.668734	204.0
GSVT	0.740381	0.722182	0.759621	300.0
SB	0.762094	0.752304	0.772169	892.0
SR	0.742300	0.751163	0.733692	681.0
macro avg	0.738521	0.745293	0.733554	2077.0
micro avg	0.747582	0.747582	0.747582	2077.0
weighted avg	0.747283	0.747896	0.747582	2077.0

The result of Model Number:Dataset Number:Featuregroup Number:Rescale of 11 : 1 : 0 : 0

	f1-score	precision	recall	support
AFIB	0.857339	0.893599	0.824192	204.0
GSVT	0.789547	0.790930	0.800014	300.0
SB	0.850021	0.809526	0.895604	892.0
SR	0.751460	0.811985	0.700692	681.0
macro avg	0.812092	0.826510	0.805126	2077.0
micro avg	0.810876	0.810876	0.810876	2077.0
weighted avg	0.809689	0.815904	0.810876	2077.0

The result of Model Number:Dataset Number:Featuregroup Number:Rescale of 11 : 1 : 0 : 1

	f1-score	precision	recall	support
AFIB	0.860118	0.887688	0.834466	204.0
GSVT	0.885850	0.870610	0.901765	300.0
SB	0.901174	0.892266	0.910265	892.0
SR	0.877079	0.888054	0.866390	681.0
macro avg	0.881055	0.884654	0.878221	2077.0
micro avg	0.887207	0.887207	0.887207	2077.0
weighted avg	0.887028	0.887307	0.887207	2077.0

The result of Model Number:Dataset Number:Featuregroup Number:Rescale of 11 : 1 : 1 : 0

	f1-score	precision	recall	support
AFIB	0.711132	0.742738	0.682205	204.0
GSVT	0.740766	0.734951	0.746691	300.0
SB	0.752082	0.746280	0.757982	892.0
SR	0.743761	0.744990	0.742564	681.0
macro avg	0.736936	0.742240	0.732360	2077.0
micro avg	0.743853	0.743853	0.743853	2077.0
weighted avg	0.743697	0.743873	0.743853	2077.0

The result of Model Number:Dataset Number:Featuregroup Number:Rescale of 11 : 1 : 1 : 1

	f1-score	precision	recall	support
AFIB	0.746171	0.766339	0.727193	204.0
GSVT	0.752321	0.741247	0.763835	300.0
SB	0.766894	0.758736	0.775233	892.0
SR	0.752670	0.763012	0.742617	681.0
macro avg	0.754514	0.757333	0.752219	2077.0
micro avg	0.758174	0.758174	0.758174	2077.0
weighted avg	0.758090	0.758359	0.758174	2077.0

The result of Model Number:Dataset Number:Featuregroup Number:Rescale of 11 : 1 : 2 : 0

	f1-score	precision	recall	support
AFIB	0.759798	0.749831	0.770067	875.0
GSVT	0.771430	0.780005	0.763055	691.0
SB	0.818201	0.816270	0.820146	1553.0
SR	0.797716	0.804895	0.790674	907.0
macro avg	0.786786	0.787750	0.785986	4026.0
micro avg	0.792824	0.792824	0.792824	4026.0
weighted avg	0.792865	0.793043	0.792824	4026.0

The result of Model Number:Dataset Number:Featuregroup Number:Rescale of 11 : 1 : 2 : 1

	f1-score	precision	recall	support
AFIB	0.784848	0.808650	0.762440	875.0
GSVT	0.837836	0.832026	0.843773	691.0
SB	0.916695	0.913188	0.920235	1553.0
SR	0.866973	0.853960	0.880438	907.0
macro avg	0.851588	0.851956	0.851722	4026.0
micro avg	0.863851	0.863851	0.863851	4026.0
weighted avg	0.863303	0.863195	0.863851	4026.0

The result of Model Number:Dataset Number:Featuregroup Number:Rescale of 11 : 1 : 3 : 0

	f1-score	precision	recall	support
AFIB	0.650083	0.664784	0.636038	875.0
GSVT	0.689236	0.692169	0.686368	691.0
SB	0.748367	0.744840	0.751932	1553.0
SR	0.709068	0.698191	0.720318	907.0
macro avg	0.699188	0.699996	0.698664	4026.0
micro avg	0.708369	0.708369	0.708369	4026.0
weighted avg	0.708004	0.707891	0.708369	4026.0

The result of Model Number:Dataset Number:Featuregroup Number:Rescale of 11 : 1 : 3 : 1

	f1-score	precision	recall	support
AFIB	0.687795	0.693079	0.682748	204.0

GSVT	0.712016	0.711334	0.712723	300.0
SB	0.726583	0.724891	0.728291	892.0
SR	0.719897	0.720867	0.718945	681.0
macro avg	0.711573	0.712543	0.710677	2077.0
micro avg	0.718505	0.718505	0.718505	2077.0
weighted avg	0.718477	0.718489	0.718505	2077.0

The result of Model Number:Dataset Number:Featuregroup Number:Rescale of 11 : 1 : 4 : 0

	f1-score	precision	recall	support
AFIB	0.688599	0.689833	0.687392	875.0
GSVT	0.659380	0.662844	0.655971	691.0
SB	0.697464	0.659097	0.740580	1553.0
SR	0.579247	0.651985	0.521161	907.0
macro avg	0.656173	0.665940	0.651276	4026.0
micro avg	0.665067	0.665067	0.665067	4026.0
weighted avg	0.662368	0.664818	0.665067	4026.0

The result of Model Number:Dataset Number:Featuregroup Number:Rescale of 11 : 1 : 4 : 1

	f1-score	precision	recall	support
AFIB	0.772848	0.775668	0.770099	875.0
GSVT	0.786205	0.775968	0.796784	691.0
SB	0.828740	0.819612	0.838079	1553.0
SR	0.785408	0.807104	0.764890	907.0
macro avg	0.793300	0.794588	0.792463	4026.0
micro avg	0.799728	0.799728	0.799728	4026.0
weighted avg	0.799530	0.799753	0.799728	4026.0

The result of Model Number:Dataset Number:Featuregroup Number:Rescale of 11 : 1 : 5 : 0

	f1-score	precision	recall	support
AFIB	0.905916	0.937597	0.876724	204.0
GSVT	0.944174	0.938419	0.950130	300.0
SB	0.963562	0.959603	0.967557	892.0
SR	0.954654	0.953262	0.956057	681.0
macro avg	0.942077	0.947220	0.937617	2077.0
micro avg	0.952348	0.952348	0.952348	2077.0
weighted avg	0.952179	0.952303	0.952348	2077.0

The result of Model Number:Dataset Number:Featuregroup Number:Rescale of 11 : 1 : 5 : 1

	f1-score	precision	recall	support
AFIB	0.796657	0.802345	0.791191	204.0
GSVT	0.824513	0.823982	0.825062	300.0
SB	0.841010	0.839144	0.842893	892.0
SR	0.833493	0.834501	0.832512	681.0
macro avg	0.823918	0.824993	0.822915	2077.0

micro avg 0.831836 0.831836 0.831836 2077.0
weighted avg 0.831806 0.831817 0.831836 2077.0
The result of Model Number:Dataset Number:Featuregroup Number:Rescale of 11 : 1 : 6 : 0

	f1-score	precision	recall	support
AFIB	0.675217	0.676599	0.673914	204.0
GSVT	0.690068	0.687386	0.692820	300.0
SB	0.705388	0.704446	0.706335	892.0
SR	0.700206	0.702276	0.698151	681.0
macro avg	0.692720	0.692677	0.692805	2077.0
micro avg	0.698515	0.698515	0.698515	2077.0
weighted avg	0.698513	0.698536	0.698515	2077.0

The result of Model Number:Dataset Number:Featuregroup Number:Rescale of 11 : 1 : 6 : 1

	f1-score	precision	recall	support
AFIB	0.599189	0.622473	0.577628	875.0
GSVT	0.644147	0.639391	0.649061	691.0
SB	0.707869	0.704222	0.711559	1553.0
SR	0.666169	0.653466	0.679448	907.0
macro avg	0.654344	0.654888	0.654424	4026.0
micro avg	0.664490	0.664490	0.664490	4026.0
weighted avg	0.663917	0.663893	0.664490	4026.0

The result of Model Number:Dataset Number:Featuregroup Number:Rescale of 11 : 1 : 7 : 0

	f1-score	precision	recall	support
AFIB	0.637271	0.637441	0.637212	875.0
GSVT	0.648368	0.645974	0.650861	691.0
SB	0.693627	0.691943	0.695322	1553.0
SR	0.677085	0.681944	0.672313	907.0
macro avg	0.664088	0.664326	0.663927	4026.0
micro avg	0.669878	0.669878	0.669878	4026.0
weighted avg	0.669884	0.669955	0.669878	4026.0

The result of Model Number:Dataset Number:Featuregroup Number:Rescale of 11 : 1 : 7 : 1

	f1-score	precision	recall	support
AFIB	0.697985	0.689600	0.706603	875.0
GSVT	0.711410	0.717170	0.705746	691.0
SB	0.758308	0.755405	0.761236	1553.0
SR	0.740528	0.749997	0.731305	907.0
macro avg	0.727058	0.728043	0.726222	4026.0
micro avg	0.733095	0.733095	0.733095	4026.0
weighted avg	0.733143	0.733322	0.733095	4026.0

The result of Model Number:Dataset Number:Featuregroup Number:Rescale of 11 : 1 : 8 : 0

	f1-score	precision	recall	support
AFIB	0.883779	0.889558	0.878129	204.0
GSVT	0.905922	0.903835	0.908029	300.0
SB	0.922093	0.920762	0.923434	892.0
SR	0.914825	0.915781	0.913888	681.0
macro avg	0.906655	0.907484	0.905870	2077.0
micro avg	0.913629	0.913629	0.913629	2077.0
weighted avg	0.913611	0.913619	0.913629	2077.0

The result of Model Number:Dataset Number:Featuregroup Number:Rescale of 11 : 1 : 8 : 1

	f1-score	precision	recall	support
AFIB	0.886002	0.878167	0.894016	875.0
GSVT	0.805530	0.780095	0.896168	691.0
SB	0.726229	0.697280	0.771500	1553.0
SR	0.748484	0.756229	0.740945	907.0
macro avg	0.791561	0.777943	0.825657	4026.0
micro avg	0.812641	0.812641	0.812641	4026.0
weighted avg	0.779578	0.764088	0.812641	4026.0

The result of Model Number:Dataset Number:Featuregroup Number:Rescale of 11 : 1 : 9 : 0

	f1-score	precision	recall	support
AFIB	0.927439	0.945960	0.909772	204.0
GSVT	0.937855	0.926136	0.950015	300.0
SB	0.955760	0.946621	0.965078	892.0
SR	0.938403	0.950713	0.926433	681.0
macro avg	0.939864	0.942358	0.937824	2077.0
micro avg	0.944800	0.944800	0.944800	2077.0
weighted avg	0.944701	0.944939	0.944800	2077.0

The result of Model Number:Dataset Number:Featuregroup Number:Rescale of 11 : 1 : 9 : 1

	f1-score	precision	recall	support
AFIB	0.652673	0.655338	0.650050	875.0
GSVT	0.607764	0.634587	0.583217	691.0
SB	0.652703	0.591264	0.728400	1553.0
SR	0.492356	0.612784	0.411654	907.0
macro avg	0.601374	0.623493	0.593330	4026.0
micro avg	0.615095	0.615095	0.615095	4026.0
weighted avg	0.608860	0.617474	0.615095	4026.0

The result of Model Number:Dataset Number:Featuregroup Number:Rescale of 11 : 1 : 10 : 0

	f1-score	precision	recall	support
AFIB	0.877445	0.878826	0.876181	204.0
GSVT	0.898665	0.897263	0.900147	300.0
SB	0.917204	0.916085	0.918333	892.0

SR	0.910781	0.912529	0.909043	681.0
macro avg	0.901024	0.901176	0.900926	2077.0
micro avg	0.908520	0.908520	0.908520	2077.0
weighted avg	0.908515	0.908541	0.908520	2077.0

The result of Model Number:Dataset Number:Featuregroup Number:Rescale of 11 : 1 : 10 : 1

	f1-score	precision	recall	support
AFIB	0.830207	0.813440	0.847745	875.0
GSVT	0.857732	0.871369	0.844699	691.0
SB	0.912639	0.912588	0.912697	1553.0
SR	0.890512	0.898626	0.882557	907.0
macro avg	0.872772	0.874006	0.871925	4026.0
micro avg	0.880120	0.880120	0.880120	4026.0
weighted avg	0.880314	0.880820	0.880120	4026.0

The result of Model Number:Dataset Number:Featuregroup Number:Rescale of 12 : 0 : 0 : 0

	f1-score	precision	recall	support
AFIB	0.846612	0.850808	0.842548	875.0
GSVT	0.855042	0.856274	0.853943	691.0
SB	0.900047	0.895871	0.904272	1553.0
SR	0.883478	0.885690	0.881325	907.0
macro avg	0.871295	0.872161	0.870522	4026.0
micro avg	0.877049	0.877049	0.877049	4026.0
weighted avg	0.876976	0.876987	0.877049	4026.0

The result of Model Number:Dataset Number:Featuregroup Number:Rescale of 12 : 0 : 0 : 1

	f1-score	precision	recall	support
AFIB	0.626847	0.620701	0.670224	204.0
GSVT	0.562064	0.555815	0.568596	300.0
SB	0.722288	0.717028	0.727630	892.0
SR	0.710174	0.715093	0.705381	681.0
macro avg	0.655343	0.652159	0.667958	2077.0
micro avg	0.691726	0.691726	0.691726	2077.0
weighted avg	0.685799	0.683647	0.691726	2077.0

The result of Model Number:Dataset Number:Featuregroup Number:Rescale of 12 : 0 : 1 : 0

	f1-score	precision	recall	support
AFIB	0.899943	0.899494	0.900432	875.0
GSVT	0.911138	0.917234	0.905195	691.0
SB	0.965931	0.963711	0.968166	1553.0
SR	0.937954	0.937731	0.938338	907.0
macro avg	0.928741	0.929542	0.928033	4026.0
micro avg	0.935917	0.935917	0.935917	4026.0
weighted avg	0.935882	0.935924	0.935917	4026.0

The result of Model Number:Dataset Number:Featuregroup Number:Rescale of 12 : 0 : 1 : 1

	f1-score	precision	recall	support
AFIB	0.820098	0.816763	0.823481	875.0
GSVT	0.832086	0.837116	0.827148	691.0
SB	0.877591	0.876522	0.878665	1553.0
SR	0.859283	0.860644	0.857951	907.0
macro avg	0.847264	0.847761	0.846811	4026.0
micro avg	0.853163	0.853163	0.853163	4026.0
weighted avg	0.853161	0.853194	0.853163	4026.0

The result of Model Number:Dataset Number:Featuregroup Number:Rescale of 12 : 0 : 2 : 0

	f1-score	precision	recall	support
AFIB	0.703105	0.709906	0.696560	204.0
GSVT	0.727984	0.726549	0.729437	300.0
SB	0.743201	0.741884	0.744531	892.0
SR	0.736349	0.736705	0.736024	681.0
macro avg	0.727660	0.728761	0.726638	2077.0
micro avg	0.734850	0.734850	0.734850	2077.0
weighted avg	0.734818	0.734830	0.734850	2077.0

The result of Model Number:Dataset Number:Featuregroup Number:Rescale of 12 : 0 : 2 : 1

	f1-score	precision	recall	support
AFIB	0.905007	0.912556	0.897894	204.0
GSVT	0.928915	0.924637	0.933252	300.0
SB	0.942923	0.939574	0.946303	892.0
SR	0.934215	0.938399	0.930116	681.0
macro avg	0.927765	0.928791	0.926891	2077.0
micro avg	0.934356	0.934356	0.934356	2077.0
weighted avg	0.934321	0.934377	0.934356	2077.0

The result of Model Number:Dataset Number:Featuregroup Number:Rescale of 12 : 0 : 3 : 0

	f1-score	precision	recall	support
AFIB	0.862381	0.863151	0.861702	875.0
GSVT	0.878178	0.880482	0.876034	691.0
SB	0.932198	0.925991	0.938497	1553.0
SR	0.914035	0.922380	0.905872	907.0
macro avg	0.896698	0.898001	0.895526	4026.0
micro avg	0.903736	0.903736	0.903736	4026.0
weighted avg	0.903660	0.903709	0.903736	4026.0

The result of Model Number:Dataset Number:Featuregroup Number:Rescale of 12 : 0 : 3 : 1

	f1-score	precision	recall	support
AFIB	0.794076	0.793868	0.794361	875.0

GSVT	0.810664	0.807599	0.813840	691.0
SB	0.863074	0.859178	0.867020	1553.0
SR	0.833987	0.843516	0.824704	907.0
macro avg	0.825451	0.826040	0.824981	4026.0
micro avg	0.832567	0.832567	0.832567	4026.0
weighted avg	0.832530	0.832602	0.832567	4026.0

The result of Model Number:Dataset Number:Featuregroup Number:Rescale of 12 : 0 : 4 : 0

	f1-score	precision	recall	support
AFIB	0.935091	0.947581	0.923189	204.0
GSVT	0.951610	0.945813	0.957624	300.0
SB	0.974549	0.973685	0.975421	892.0
SR	0.967348	0.967495	0.967207	681.0
macro avg	0.957149	0.958643	0.955860	2077.0
micro avg	0.965027	0.965027	0.965027	2077.0
weighted avg	0.964999	0.965066	0.965027	2077.0

The result of Model Number:Dataset Number:Featuregroup Number:Rescale of 12 : 0 : 4 : 1

	f1-score	precision	recall	support
AFIB	0.722243	0.720418	0.724189	875.0
GSVT	0.736333	0.732285	0.740466	691.0
SB	0.770999	0.766351	0.775715	1553.0
SR	0.748267	0.761772	0.735246	907.0
macro avg	0.744461	0.745207	0.743904	4026.0
micro avg	0.749350	0.749350	0.749350	4026.0
weighted avg	0.749332	0.749490	0.749350	4026.0

The result of Model Number:Dataset Number:Featuregroup Number:Rescale of 12 : 0 : 5 : 0

	f1-score	precision	recall	support
AFIB	0.718676	0.723705	0.713741	204.0
GSVT	0.735396	0.731771	0.739069	300.0
SB	0.750013	0.748764	0.751274	892.0
SR	0.742548	0.744311	0.740802	681.0
macro avg	0.736658	0.737138	0.736222	2077.0
micro avg	0.742391	0.742391	0.742391	2077.0
weighted avg	0.742376	0.742388	0.742391	2077.0

The result of Model Number:Dataset Number:Featuregroup Number:Rescale of 12 : 0 : 5 : 1

	f1-score	precision	recall	support
AFIB	0.666830	0.671591	0.662255	204.0
GSVT	0.690147	0.689702	0.690606	300.0
SB	0.703956	0.702394	0.705532	892.0
SR	0.697663	0.698507	0.696842	681.0
macro avg	0.689649	0.690549	0.688809	2077.0

micro avg 0.696276 0.696276 0.696276 2077.0
weighted avg 0.696251 0.696261 0.696276 2077.0
The result of Model Number:Dataset Number:Featuregroup Number:Rescale of 12 : 0 : 6 : 0

	f1-score	precision	recall	support
AFIB	0.660214	0.665286	0.655370	204.0
GSVT	0.683464	0.682809	0.684143	300.0
SB	0.697447	0.695822	0.699086	892.0
SR	0.691028	0.691960	0.690115	681.0
macro avg	0.683038	0.683969	0.682178	2077.0
micro avg	0.689692	0.689692	0.689692	2077.0
weighted avg	0.689666	0.689677	0.689692	2077.0

The result of Model Number:Dataset Number:Featuregroup Number:Rescale of 12 : 0 : 6 : 1

	f1-score	precision	recall	support
AFIB	0.663147	0.691194	0.637508	204.0
GSVT	0.610711	0.611780	0.618806	300.0
SB	0.657486	0.626164	0.692745	892.0
SR	0.581250	0.628065	0.541981	681.0
macro avg	0.628148	0.639301	0.622760	2077.0
micro avg	0.627208	0.627208	0.627208	2077.0
weighted avg	0.626290	0.631097	0.627208	2077.0

The result of Model Number:Dataset Number:Featuregroup Number:Rescale of 12 : 0 : 7 : 0

	f1-score	precision	recall	support
AFIB	0.667287	0.666954	0.667650	875.0
GSVT	0.675587	0.680107	0.671181	691.0
SB	0.716215	0.714569	0.717873	1553.0
SR	0.695471	0.695306	0.695756	907.0
macro avg	0.688640	0.689234	0.688115	4026.0
micro avg	0.693961	0.693961	0.693961	4026.0
weighted avg	0.693935	0.693966	0.693961	4026.0

The result of Model Number:Dataset Number:Featuregroup Number:Rescale of 12 : 0 : 7 : 1

	f1-score	precision	recall	support
AFIB	0.679491	0.704683	0.656135	204.0
GSVT	0.699847	0.691097	0.708912	300.0
SB	0.717594	0.711046	0.724270	892.0
SR	0.698728	0.704151	0.693395	681.0
macro avg	0.698915	0.702744	0.695678	2077.0
micro avg	0.705236	0.705236	0.705236	2077.0
weighted avg	0.705103	0.705279	0.705236	2077.0

The result of Model Number:Dataset Number:Featuregroup Number:Rescale of 12 : 0 : 8 : 0

	f1-score	precision	recall	support
AFIB	0.806487	0.812880	0.800215	875.0
GSVT	0.816712	0.813111	0.820372	691.0
SB	0.868477	0.865044	0.871944	1553.0
SR	0.847136	0.849529	0.844796	907.0
macro avg	0.834703	0.835141	0.834332	4026.0
micro avg	0.841387	0.841387	0.841387	4026.0
weighted avg	0.841312	0.841298	0.841387	4026.0

The result of Model Number:Dataset Number:Featuregroup Number:Rescale of 12 : 0 : 8 : 1

	f1-score	precision	recall	support
AFIB	0.661639	0.674337	0.649449	875.0
GSVT	0.705287	0.706363	0.704258	691.0
SB	0.767967	0.764592	0.771377	1553.0
SR	0.725705	0.717816	0.733843	907.0
macro avg	0.715149	0.715777	0.714732	4026.0
micro avg	0.724902	0.724902	0.724902	4026.0
weighted avg	0.724579	0.724444	0.724902	4026.0

The result of Model Number:Dataset Number:Featuregroup Number:Rescale of 12 : 0 : 9 : 0

	f1-score	precision	recall	support
AFIB	0.650583	0.656437	0.645037	875.0
GSVT	0.663835	0.650375	0.678021	691.0
SB	0.704002	0.696170	0.712096	1553.0
SR	0.676066	0.696162	0.657256	907.0
macro avg	0.673621	0.674786	0.673103	4026.0
micro avg	0.679319	0.679319	0.679319	4026.0
weighted avg	0.679204	0.679673	0.679319	4026.0

The result of Model Number:Dataset Number:Featuregroup Number:Rescale of 12 : 0 : 9 : 1

	f1-score	precision	recall	support
AFIB	0.837174	0.844663	0.829920	875.0
GSVT	0.853163	0.836940	0.870191	691.0
SB	0.905561	0.897716	0.913548	1553.0
SR	0.868367	0.888334	0.849318	907.0
macro avg	0.866066	0.866913	0.865744	4026.0
micro avg	0.873461	0.873461	0.873461	4026.0
weighted avg	0.873325	0.873641	0.873461	4026.0

The result of Model Number:Dataset Number:Featuregroup Number:Rescale of 12 : 0 : 10 : 0

	f1-score	precision	recall	support
AFIB	0.748964	0.751766	0.746502	875.0
GSVT	0.765823	0.773211	0.758728	691.0
SB	0.814309	0.805768	0.823096	1553.0

	f1-score	precision	recall	support
SR	0.786111	0.793029	0.779582	907.0
macro avg	0.778802	0.780943	0.776977	4026.0
micro avg	0.785598	0.785598	0.785598	4026.0
weighted avg	0.785433	0.785574	0.785598	4026.0

The result of Model Number:Dataset Number:Featuregroup Number:Rescale of 12 : 0 : 10 : 1

	f1-score	precision	recall	support
AFIB	0.744445	0.800407	0.696573	204.0
GSVT	0.709955	0.732490	0.688873	300.0
SB	0.739672	0.703070	0.780300	892.0
SR	0.654776	0.682920	0.628927	681.0
macro avg	0.712212	0.729722	0.698668	2077.0
micro avg	0.709239	0.709239	0.709239	2077.0
weighted avg	0.708013	0.710273	0.709239	2077.0

The result of Model Number:Dataset Number:Featuregroup Number:Rescale of 12 : 1 : 0 : 0

	f1-score	precision	recall	support
AFIB	0.839357	0.851738	0.827422	204.0
GSVT	0.859962	0.856587	0.863392	300.0
SB	0.879061	0.877298	0.880834	892.0
SR	0.872826	0.872958	0.872702	681.0
macro avg	0.862801	0.864645	0.861088	2077.0
micro avg	0.870403	0.870403	0.870403	2077.0
weighted avg	0.870358	0.870373	0.870403	2077.0

The result of Model Number:Dataset Number:Featuregroup Number:Rescale of 12 : 1 : 0 : 1

	f1-score	precision	recall	support
AFIB	0.769722	0.787263	0.753069	875.0
GSVT	0.819083	0.820702	0.817744	691.0
SB	0.896131	0.891226	0.901093	1553.0
SR	0.846978	0.836704	0.857626	907.0
macro avg	0.832979	0.833974	0.832383	4026.0
micro avg	0.844824	0.844824	0.844824	4026.0
weighted avg	0.844360	0.844244	0.844824	4026.0

The result of Model Number:Dataset Number:Featuregroup Number:Rescale of 12 : 1 : 1 : 0

	f1-score	precision	recall	support
AFIB	0.632763	0.680330	0.592072	204.0
GSVT	0.603447	0.622601	0.585528	300.0
SB	0.628706	0.597595	0.663239	892.0
SR	0.556546	0.580468	0.534575	681.0
macro avg	0.605365	0.620248	0.593853	2077.0
micro avg	0.602838	0.602838	0.602838	2077.0
weighted avg	0.601796	0.603717	0.602838	2077.0

The result of Model Number:Dataset Number:Featuregroup Number:Rescale of 12 : 1 : 1 : 1

	f1-score	precision	recall	support
AFIB	0.759593	0.766518	0.752892	204.0
GSVT	0.779416	0.776864	0.782009	300.0
SB	0.793664	0.793135	0.794197	892.0
SR	0.786684	0.786458	0.786921	681.0
macro avg	0.779839	0.780743	0.779005	2077.0
micro avg	0.785994	0.785994	0.785994	2077.0
weighted avg	0.785971	0.785981	0.785994	2077.0

The result of Model Number:Dataset Number:Featuregroup Number:Rescale of 12 : 1 : 2 : 0

	f1-score	precision	recall	support
AFIB	0.696309	0.696640	0.696078	875.0
GSVT	0.714342	0.708159	0.720659	691.0
SB	0.762920	0.757360	0.768569	1553.0
SR	0.730327	0.744845	0.716402	907.0
macro avg	0.725974	0.726751	0.725427	4026.0
micro avg	0.732838	0.732838	0.732838	4026.0
weighted avg	0.732763	0.732899	0.732838	4026.0

The result of Model Number:Dataset Number:Featuregroup Number:Rescale of 12 : 1 : 2 : 1

	f1-score	precision	recall	support
AFIB	0.678709	0.715910	0.645635	204.0
GSVT	0.646275	0.668934	0.625181	300.0
SB	0.664758	0.639118	0.692551	892.0
SR	0.591652	0.607704	0.576460	681.0
macro avg	0.645349	0.657916	0.634957	2077.0
micro avg	0.640149	0.640149	0.640149	2077.0
weighted avg	0.639489	0.640667	0.640149	2077.0

The result of Model Number:Dataset Number:Featuregroup Number:Rescale of 12 : 1 : 3 : 0

	f1-score	precision	recall	support
AFIB	0.700666	0.729919	0.673794	875.0
GSVT	0.756964	0.753132	0.760927	691.0
SB	0.830034	0.825363	0.834780	1553.0
SR	0.777202	0.759944	0.795275	907.0
macro avg	0.766216	0.767089	0.766194	4026.0
micro avg	0.778216	0.778216	0.778216	4026.0
weighted avg	0.777474	0.777484	0.778216	4026.0

The result of Model Number:Dataset Number:Featuregroup Number:Rescale of 12 : 1 : 3 : 1

	f1-score	precision	recall	support
AFIB	0.886874	0.906355	0.868545	204.0

GSVT 0.915456 0.908057 0.923102 300.0
SB 0.934642 0.933493 0.935795 892.0
SR 0.926752 0.925946 0.927565 681.0
macro avg 0.915931 0.918463 0.913752 2077.0
micro avg 0.924658 0.924658 0.924658 2077.0
weighted avg 0.924592 0.924679 0.924658 2077.0
The result of Model Number:Dataset Number:Featuregroup Number:Rescale of 12 : 1 : 4 : 0

	f1-score	precision	recall	support
AFIB	0.740013	0.754215	0.726379	875.0
GSVT	0.788831	0.790034	0.787680	691.0
SB	0.858936	0.855161	0.862750	1553.0
SR	0.811668	0.802844	0.820770	907.0
macro avg	0.799862	0.800564	0.799395	4026.0
micro avg	0.810770	0.810770	0.810770	4026.0
weighted avg	0.810408	0.810258	0.810770	4026.0

The result of Model Number:Dataset Number:Featuregroup Number:Rescale of 12 : 1 : 4 : 1

	f1-score	precision	recall	support
AFIB	0.783877	0.788179	0.779672	875.0
GSVT	0.784973	0.775049	0.795278	691.0
SB	0.825535	0.822223	0.828882	1553.0
SR	0.807814	0.817620	0.798290	907.0
macro avg	0.800550	0.800768	0.800530	4026.0
micro avg	0.805527	0.805527	0.805527	4026.0
weighted avg	0.805527	0.805690	0.805527	4026.0

The result of Model Number:Dataset Number:Featuregroup Number:Rescale of 12 : 1 : 5 : 0

	f1-score	precision	recall	support
AFIB	0.598005	0.594239	0.673611	204.0
GSVT	0.728978	0.725228	0.732889	300.0
SB	0.749734	0.746167	0.753344	892.0
SR	0.588082	0.590019	0.586512	681.0
macro avg	0.666200	0.663913	0.686589	2077.0
micro avg	0.687858	0.687858	0.687858	2077.0
weighted avg	0.678831	0.677023	0.687858	2077.0

The result of Model Number:Dataset Number:Featuregroup Number:Rescale of 12 : 1 : 5 : 1

	f1-score	precision	recall	support
AFIB	0.834965	0.854040	0.816934	204.0
GSVT	0.872896	0.867462	0.878420	300.0
SB	0.891748	0.888982	0.894544	892.0
SR	0.881535	0.881943	0.881152	681.0
macro avg	0.870286	0.873107	0.867762	2077.0

micro avg 0.880201 0.880201 0.880201 2077.0
weighted avg 0.880099 0.880134 0.880201 2077.0
The result of Model Number:Dataset Number:Featuregroup Number:Rescale of 12 : 1 : 6 : 0

	f1-score	precision	recall	support
AFIB	0.836570	0.876623	0.800215	204.0
GSVT	0.881134	0.871701	0.890805	300.0
SB	0.899850	0.894879	0.904888	892.0
SR	0.889685	0.888913	0.890472	681.0
macro avg	0.876809	0.883029	0.871595	2077.0
micro avg	0.887846	0.887846	0.887846	2077.0
weighted avg	0.887598	0.887782	0.887846	2077.0

The result of Model Number:Dataset Number:Featuregroup Number:Rescale of 12 : 1 : 6 : 1

	f1-score	precision	recall	support
AFIB	0.721979	0.735224	0.709259	204.0
GSVT	0.731501	0.724119	0.739051	300.0
SB	0.749520	0.745619	0.753467	892.0
SR	0.739316	0.743949	0.734757	681.0
macro avg	0.735579	0.737228	0.734133	2077.0
micro avg	0.740908	0.740908	0.740908	2077.0
weighted avg	0.740867	0.740945	0.740908	2077.0

The result of Model Number:Dataset Number:Featuregroup Number:Rescale of 12 : 1 : 7 : 0

	f1-score	precision	recall	support
AFIB	0.833891	0.879597	0.793254	204.0
GSVT	0.794041	0.821880	0.768124	300.0
SB	0.816749	0.785248	0.850897	892.0
SR	0.726929	0.746650	0.708264	681.0
macro avg	0.792902	0.808344	0.780135	2077.0
micro avg	0.786513	0.786513	0.786513	2077.0
weighted avg	0.785703	0.787151	0.786513	2077.0

The result of Model Number:Dataset Number:Featuregroup Number:Rescale of 12 : 1 : 7 : 1

	f1-score	precision	recall	support
AFIB	0.816743	0.829266	0.804726	204.0
GSVT	0.836109	0.829015	0.843360	300.0
SB	0.850653	0.847908	0.853419	892.0
SR	0.841912	0.845037	0.838820	681.0
macro avg	0.836354	0.837806	0.835081	2077.0
micro avg	0.842397	0.842397	0.842397	2077.0
weighted avg	0.842356	0.842407	0.842397	2077.0

The result of Model Number:Dataset Number:Featuregroup Number:Rescale of 12 : 1 : 8 : 0

	f1-score	precision	recall	support
AFIB	0.722530	0.723174	0.721961	875.0
GSVT	0.735765	0.737695	0.733968	691.0
SB	0.781024	0.775824	0.786302	1553.0
SR	0.765807	0.772798	0.758968	907.0
macro avg	0.751281	0.752373	0.750300	4026.0
micro avg	0.757178	0.757178	0.757178	4026.0
weighted avg	0.757115	0.757155	0.757178	4026.0

The result of Model Number:Dataset Number:Featuregroup Number:Rescale of 12 : 1 : 8 : 1

	f1-score	precision	recall	support
AFIB	0.640316	0.645378	0.635354	875.0
GSVT	0.656428	0.655040	0.657846	691.0
SB	0.699772	0.693586	0.706077	1553.0
SR	0.681143	0.687823	0.674607	907.0
macro avg	0.669415	0.670457	0.668471	4026.0
micro avg	0.675339	0.675339	0.675339	4026.0
weighted avg	0.675214	0.675195	0.675339	4026.0

The result of Model Number:Dataset Number:Featuregroup Number:Rescale of 12 : 1 : 9 : 0

	f1-score	precision	recall	support
AFIB	0.682256	0.698856	0.666594	204.0
GSVT	0.690832	0.681322	0.700704	300.0
SB	0.704782	0.697744	0.711968	892.0
SR	0.692476	0.701564	0.683641	681.0
macro avg	0.692587	0.694871	0.690727	2077.0
micro avg	0.696597	0.696597	0.696597	2077.0
weighted avg	0.696520	0.696733	0.696597	2077.0

The result of Model Number:Dataset Number:Featuregroup Number:Rescale of 12 : 1 : 9 : 1

	f1-score	precision	recall	support
AFIB	0.689908	0.681619	0.698425	875.0
GSVT	0.703177	0.708870	0.697579	691.0
SB	0.749532	0.746663	0.752426	1553.0
SR	0.731958	0.741317	0.722841	907.0
macro avg	0.718643	0.719617	0.717818	4026.0
micro avg	0.724611	0.724611	0.724611	4026.0
weighted avg	0.724658	0.724836	0.724611	4026.0

The result of Model Number:Dataset Number:Featuregroup Number:Rescale of 12 : 1 : 10 : 0

	f1-score	precision	recall	support
AFIB	0.663429	0.666138	0.660762	875.0
GSVT	0.617780	0.645045	0.592828	691.0
SB	0.663459	0.601008	0.740403	1553.0

SR	0.500470	0.622882	0.418438	907.0
macro avg	0.611284	0.633768	0.603108	4026.0
micro avg	0.625231	0.625231	0.625231	4026.0
weighted avg	0.618893	0.627649	0.625231	4026.0

The result of Model Number:Dataset Number:Featuregroup Number:Rescale of 12 : 1 : 10 : 1

	f1-score	precision	recall	support
AFIB	0.742112	0.735477	0.748900	875.0
GSVT	0.754027	0.757010	0.751094	691.0
SB	0.803954	0.802462	0.805455	1553.0
SR	0.783582	0.790825	0.776499	907.0
macro avg	0.770919	0.771444	0.770487	4026.0
micro avg	0.777310	0.777310	0.777310	4026.0
weighted avg	0.777355	0.777481	0.777310	4026.0

The result of Model Number:Dataset Number:Featuregroup Number:Rescale of 13 : 0 : 0 : 0

	f1-score	precision	recall	support
AFIB	0.898706	0.899921	0.897665	875.0
GSVT	0.912703	0.912234	0.913207	691.0
SB	0.953224	0.949813	0.956663	1553.0
SR	0.930397	0.935613	0.925300	907.0
macro avg	0.923758	0.924395	0.923209	4026.0
micro avg	0.929316	0.929316	0.929316	4026.0
weighted avg	0.929278	0.929321	0.929316	4026.0

The result of Model Number:Dataset Number:Featuregroup Number:Rescale of 13 : 0 : 0 : 1

	f1-score	precision	recall	support
AFIB	0.814251	0.833607	0.795864	204.0
GSVT	0.835390	0.832166	0.838726	300.0
SB	0.856090	0.854180	0.858010	892.0
SR	0.845320	0.843619	0.847058	681.0
macro avg	0.837763	0.840893	0.834914	2077.0
micro avg	0.845530	0.845530	0.845530	2077.0
weighted avg	0.845460	0.845517	0.845530	2077.0

The result of Model Number:Dataset Number:Featuregroup Number:Rescale of 13 : 0 : 1 : 0

	f1-score	precision	recall	support
AFIB	0.836418	0.876966	0.799546	204.0
GSVT	0.871252	0.859868	0.883119	300.0
SB	0.894718	0.887915	0.901640	892.0
SR	0.869806	0.872405	0.867261	681.0
macro avg	0.868049	0.874289	0.862891	2077.0
micro avg	0.877665	0.877665	0.877665	2077.0
weighted avg	0.877435	0.877703	0.877665	2077.0

The result of Model Number:Dataset Number:Featuregroup Number:Rescale of 13 : 0 : 1 : 1

	f1-score	precision	recall	support
AFIB	0.715031	0.730090	0.700692	204.0
GSVT	0.737262	0.730526	0.744140	300.0
SB	0.749303	0.747632	0.750983	892.0
SR	0.740398	0.741172	0.739634	681.0
macro avg	0.735498	0.737355	0.733862	2077.0
micro avg	0.741334	0.741334	0.741334	2077.0
weighted avg	0.741278	0.741320	0.741334	2077.0

The result of Model Number:Dataset Number:Featuregroup Number:Rescale of 13 : 0 : 2 : 0

	f1-score	precision	recall	support
AFIB	0.756920	0.764976	0.749450	204.0
GSVT	0.779164	0.773973	0.784654	300.0
SB	0.795630	0.794919	0.796343	892.0
SR	0.788921	0.789974	0.787876	681.0
macro avg	0.780159	0.780960	0.779581	2077.0
micro avg	0.787273	0.787273	0.787273	2077.0
weighted avg	0.787250	0.787331	0.787273	2077.0

The result of Model Number:Dataset Number:Featuregroup Number:Rescale of 13 : 0 : 2 : 1

	f1-score	precision	recall	support
AFIB	0.710074	0.715467	0.704822	204.0
GSVT	0.724611	0.717534	0.731845	300.0
SB	0.741513	0.739618	0.743425	892.0
SR	0.733648	0.737792	0.729559	681.0
macro avg	0.727462	0.727603	0.727413	2077.0
micro avg	0.733415	0.733415	0.733415	2077.0
weighted avg	0.733405	0.733458	0.733415	2077.0

The result of Model Number:Dataset Number:Featuregroup Number:Rescale of 13 : 0 : 3 : 0

	f1-score	precision	recall	support
AFIB	0.910916	0.933982	0.889046	204.0
GSVT	0.930699	0.920578	0.941199	300.0
SB	0.956913	0.952137	0.961740	892.0
SR	0.942081	0.946308	0.937930	681.0
macro avg	0.935152	0.938251	0.932479	2077.0
micro avg	0.943826	0.943826	0.943826	2077.0
weighted avg	0.943746	0.943884	0.943826	2077.0

The result of Model Number:Dataset Number:Featuregroup Number:Rescale of 13 : 0 : 3 : 1

	f1-score	precision	recall	support
AFIB	0.805702	0.807328	0.804098	204.0

GSVT	0.828117	0.825655	0.830598	300.0
SB	0.841839	0.841088	0.842593	892.0
SR	0.834602	0.836206	0.833017	681.0
macro avg	0.827565	0.827569	0.827577	2077.0
micro avg	0.833940	0.833940	0.833940	2077.0
weighted avg	0.833935	0.833942	0.833940	2077.0

The result of Model Number:Dataset Number:Featuregroup Number:Rescale of 13 : 0 : 4 : 0

	f1-score	precision	recall	support
AFIB	0.644218	0.660285	0.628964	875.0
GSVT	0.685336	0.687963	0.682760	691.0
SB	0.742161	0.738568	0.745792	1553.0
SR	0.703239	0.691517	0.715410	907.0
macro avg	0.693738	0.694583	0.693231	4026.0
micro avg	0.702738	0.702738	0.702738	4026.0
weighted avg	0.702353	0.702269	0.702738	4026.0

The result of Model Number:Dataset Number:Featuregroup Number:Rescale of 13 : 0 : 4 : 1

	f1-score	precision	recall	support
AFIB	0.802641	0.806851	0.798522	875.0
GSVT	0.819424	0.817952	0.820960	691.0
SB	0.889754	0.882931	0.896686	1553.0
SR	0.858363	0.866954	0.849945	907.0
macro avg	0.842545	0.843672	0.841528	4026.0
micro avg	0.851824	0.851824	0.851824	4026.0
weighted avg	0.851678	0.851644	0.851824	4026.0

The result of Model Number:Dataset Number:Featuregroup Number:Rescale of 13 : 0 : 5 : 0

	f1-score	precision	recall	support
AFIB	0.909839	0.931976	0.888953	204.0
GSVT	0.921276	0.908594	0.934441	300.0
SB	0.939879	0.930493	0.949462	892.0
SR	0.923469	0.935588	0.911686	681.0
macro avg	0.923616	0.926663	0.921136	2077.0
micro avg	0.928964	0.928964	0.928964	2077.0
weighted avg	0.928861	0.929146	0.928964	2077.0

The result of Model Number:Dataset Number:Featuregroup Number:Rescale of 13 : 0 : 5 : 1

	f1-score	precision	recall	support
AFIB	0.673045	0.671666	0.674917	875.0
GSVT	0.682226	0.675064	0.689663	691.0
SB	0.719519	0.709804	0.729644	1553.0
SR	0.684507	0.710611	0.660409	907.0
macro avg	0.689824	0.691786	0.688658	4026.0

micro avg 0.695290 0.695290 0.695290 4026.0
weighted avg 0.695130 0.695734 0.695290 4026.0
The result of Model Number:Dataset Number:Featuregroup Number:Rescale of 13 : 0 : 6 : 0

	f1-score	precision	recall	support
AFIB	0.645527	0.631660	0.660052	875.0
GSVT	0.668511	0.678206	0.659134	691.0
SB	0.711656	0.711521	0.711795	1553.0
SR	0.693820	0.701884	0.685943	907.0
macro avg	0.679879	0.680818	0.679231	4026.0
micro avg	0.685687	0.685687	0.685687	4026.0
weighted avg	0.685861	0.686275	0.685687	4026.0

The result of Model Number:Dataset Number:Featuregroup Number:Rescale of 13 : 0 : 6 : 1

	f1-score	precision	recall	support
AFIB	0.654286	0.646653	0.663808	875.0
GSVT	0.673221	0.665061	0.682126	691.0
SB	0.683368	0.656990	0.722060	1553.0
SR	0.542630	0.571253	0.516771	907.0
macro avg	0.638376	0.634989	0.646191	4026.0
micro avg	0.656297	0.656297	0.656297	4026.0
weighted avg	0.643600	0.636813	0.656297	4026.0

The result of Model Number:Dataset Number:Featuregroup Number:Rescale of 13 : 0 : 7 : 0

	f1-score	precision	recall	support
AFIB	0.772434	0.786860	0.855202	204.0
GSVT	0.927613	0.908821	0.947667	300.0
SB	0.763098	0.755969	0.770372	892.0
SR	0.928796	0.936555	0.921293	681.0
macro avg	0.847985	0.847051	0.873634	2077.0
micro avg	0.853796	0.853796	0.853796	2077.0
weighted avg	0.842106	0.840291	0.853796	2077.0

The result of Model Number:Dataset Number:Featuregroup Number:Rescale of 13 : 0 : 7 : 1

	f1-score	precision	recall	support
AFIB	0.737987	0.751567	0.725264	204.0
GSVT	0.765214	0.758805	0.771865	300.0
SB	0.779583	0.778277	0.780894	892.0
SR	0.772700	0.773386	0.772019	681.0
macro avg	0.763871	0.765509	0.762510	2077.0
micro avg	0.771216	0.771216	0.771216	2077.0
weighted avg	0.771165	0.771237	0.771216	2077.0

The result of Model Number:Dataset Number:Featuregroup Number:Rescale of 13 : 0 : 8 : 0

	f1-score	precision	recall	support
AFIB	0.735949	0.728547	0.743524	875.0
GSVT	0.742535	0.749147	0.736094	691.0
SB	0.785570	0.783159	0.787997	1553.0
SR	0.770357	0.777100	0.763733	907.0
macro avg	0.758603	0.759488	0.757837	4026.0
micro avg	0.763957	0.763957	0.763957	4026.0
weighted avg	0.763972	0.764087	0.763957	4026.0

The result of Model Number:Dataset Number:Featuregroup Number:Rescale of 13 : 0 : 8 : 1

	f1-score	precision	recall	support
AFIB	0.812733	0.846666	0.781563	875.0
GSVT	0.878036	0.873591	0.882633	691.0
SB	0.962793	0.957374	0.968299	1553.0
SR	0.901511	0.881492	0.922474	907.0
macro avg	0.888768	0.889781	0.888742	4026.0
micro avg	0.902687	0.902687	0.902687	4026.0
weighted avg	0.901826	0.901838	0.902687	4026.0

The result of Model Number:Dataset Number:Featuregroup Number:Rescale of 13 : 0 : 9 : 0

	f1-score	precision	recall	support
AFIB	0.756619	0.777476	0.737003	204.0
GSVT	0.776755	0.762376	0.791760	300.0
SB	0.789352	0.781973	0.796874	892.0
SR	0.769511	0.779966	0.759347	681.0
macro avg	0.773059	0.775448	0.771246	2077.0
micro avg	0.777951	0.777951	0.777951	2077.0
weighted avg	0.777812	0.778042	0.777951	2077.0

The result of Model Number:Dataset Number:Featuregroup Number:Rescale of 13 : 0 : 9 : 1

	f1-score	precision	recall	support
AFIB	0.741807	0.727030	0.757236	875.0
GSVT	0.603766	0.561470	0.756803	691.0
SB	0.489889	0.490733	0.489048	1553.0
SR	0.794173	0.801166	0.787334	907.0
macro avg	0.657409	0.645100	0.697605	4026.0
micro avg	0.660491	0.660491	0.660491	4026.0
weighted avg	0.632736	0.624166	0.660491	4026.0

The result of Model Number:Dataset Number:Featuregroup Number:Rescale of 13 : 0 : 10 : 0

	f1-score	precision	recall	support
AFIB	0.758801	0.778016	0.740584	204.0
GSVT	0.775281	0.766850	0.784028	300.0
SB	0.797118	0.793139	0.801139	892.0

SR 0.784763 0.788284 0.781304 681.0
macro avg 0.778991 0.781572 0.776764 2077.0
micro avg 0.786216 0.786216 0.786216 2077.0
weighted avg 0.786149 0.786265 0.786216 2077.0
The result of Model Number:Dataset Number:Featuregroup Number:Rescale of 13 : 0 : 10 : 1

	f1-score	precision	recall	support
AFIB	0.764769	0.764388	0.765185	875.0
GSVT	0.774282	0.779462	0.769232	691.0
SB	0.820845	0.818959	0.822745	1553.0
SR	0.797070	0.796881	0.797397	907.0
macro avg	0.789242	0.789923	0.788640	4026.0
micro avg	0.795340	0.795340	0.795340	4026.0
weighted avg	0.795310	0.795346	0.795340	4026.0

The result of Model Number:Dataset Number:Featuregroup Number:Rescale of 13 : 1 : 0 : 0

	f1-score	precision	recall	support
AFIB	0.744109	0.760662	0.728422	204.0
GSVT	0.775813	0.770992	0.780724	300.0
SB	0.792654	0.790984	0.794340	892.0
SR	0.784211	0.783657	0.784791	681.0
macro avg	0.774197	0.776574	0.772069	2077.0
micro avg	0.782768	0.782768	0.782768	2077.0
weighted avg	0.782685	0.782716	0.782768	2077.0

The result of Model Number:Dataset Number:Featuregroup Number:Rescale of 13 : 1 : 0 : 1

	f1-score	precision	recall	support
AFIB	0.704472	0.711312	0.698200	204.0
GSVT	0.725589	0.722160	0.729244	300.0
SB	0.740283	0.739457	0.741112	892.0
SR	0.733769	0.734529	0.733015	681.0
macro avg	0.726028	0.726864	0.725393	2077.0
micro avg	0.732528	0.732528	0.732528	2077.0
weighted avg	0.732508	0.732578	0.732528	2077.0

The result of Model Number:Dataset Number:Featuregroup Number:Rescale of 13 : 1 : 1 : 0

	f1-score	precision	recall	support
AFIB	0.882246	0.882481	0.882164	875.0
GSVT	0.897609	0.894295	0.901060	691.0
SB	0.960266	0.957935	0.962613	1553.0
SR	0.937365	0.944092	0.930759	907.0
macro avg	0.919371	0.919701	0.919149	4026.0
micro avg	0.927388	0.927388	0.927388	4026.0
weighted avg	0.927396	0.927495	0.927388	4026.0

The result of Model Number:Dataset Number:Featuregroup Number:Rescale of 13 : 1 : 1 : 1

	f1-score	precision	recall	support
AFIB	0.807821	0.797097	0.818901	875.0
GSVT	0.824218	0.832031	0.816585	691.0
SB	0.877086	0.875011	0.879174	1553.0
SR	0.853292	0.862264	0.844539	907.0
macro avg	0.840604	0.841601	0.839800	4026.0
micro avg	0.847529	0.847529	0.847529	4026.0
weighted avg	0.847597	0.847829	0.847529	4026.0

The result of Model Number:Dataset Number:Featuregroup Number:Rescale of 13 : 1 : 2 : 0

	f1-score	precision	recall	support
AFIB	0.721306	0.727759	0.715057	875.0
GSVT	0.735083	0.721105	0.749754	691.0
SB	0.780229	0.773470	0.787110	1553.0
SR	0.748183	0.765386	0.731770	907.0
macro avg	0.746200	0.746930	0.745923	4026.0
micro avg	0.752571	0.752571	0.752571	4026.0
weighted avg	0.752455	0.752726	0.752571	4026.0

The result of Model Number:Dataset Number:Featuregroup Number:Rescale of 13 : 1 : 2 : 1

	f1-score	precision	recall	support
AFIB	0.735173	0.735278	0.735164	875.0
GSVT	0.748013	0.741812	0.754374	691.0
SB	0.783159	0.776384	0.790066	1553.0
SR	0.757352	0.774461	0.741009	907.0
macro avg	0.755924	0.756984	0.755153	4026.0
micro avg	0.760956	0.760956	0.760956	4026.0
weighted avg	0.760884	0.761083	0.760956	4026.0

The result of Model Number:Dataset Number:Featuregroup Number:Rescale of 13 : 1 : 3 : 0

	f1-score	precision	recall	support
AFIB	0.799413	0.799794	0.799148	875.0
GSVT	0.820117	0.813018	0.827369	691.0
SB	0.875887	0.869505	0.882373	1553.0
SR	0.838468	0.855136	0.822481	907.0
macro avg	0.833471	0.834363	0.832843	4026.0
micro avg	0.841352	0.841352	0.841352	4026.0
weighted avg	0.841265	0.841422	0.841352	4026.0

The result of Model Number:Dataset Number:Featuregroup Number:Rescale of 13 : 1 : 3 : 1

	f1-score	precision	recall	support
AFIB	0.708753	0.721796	0.696534	204.0

GSVT 0.734902 0.728747 0.741290 300.0
SB 0.748702 0.747447 0.749961 892.0
SR 0.742091 0.742750 0.741437 681.0
macro avg 0.733612 0.735185 0.732306 2077.0
micro avg 0.740666 0.740666 0.740666 2077.0
weighted avg 0.740617 0.740687 0.740666 2077.0
The result of Model Number:Dataset Number:Featuregroup Number:Rescale of 13 : 1 : 4 :
0
f1-score precision recall support
AFIB 0.757370 0.758726 0.756042 875.0
GSVT 0.725233 0.729042 0.721483 691.0
SB 0.767120 0.724921 0.814542 1553.0
SR 0.637097 0.717099 0.573209 907.0
macro avg 0.721705 0.732447 0.716319 4026.0
micro avg 0.731487 0.731487 0.731487 4026.0
weighted avg 0.728519 0.731213 0.731487 4026.0
The result of Model Number:Dataset Number:Featuregroup Number:Rescale of 13 : 1 : 4 :
1
f1-score precision recall support
AFIB 0.676823 0.668403 0.685533 875.0
GSVT 0.698013 0.706754 0.689555 691.0
SB 0.744682 0.743875 0.745496 1553.0
SR 0.724597 0.728313 0.720923 907.0
macro avg 0.711029 0.711836 0.710377 4026.0
micro avg 0.717326 0.717326 0.717326 4026.0
weighted avg 0.717399 0.717595 0.717326 4026.0
The result of Model Number:Dataset Number:Featuregroup Number:Rescale of 13 : 1 : 5 :
0
f1-score precision recall support
AFIB 0.802274 0.790194 0.821409 875.0
GSVT 0.766027 0.763797 0.772562 691.0
SB 0.818018 0.762262 0.889377 1553.0
SR 0.605957 0.809985 0.502108 907.0
macro avg 0.748069 0.781560 0.746364 4026.0
micro avg 0.767309 0.767309 0.767309 4026.0
weighted avg 0.757899 0.779348 0.767309 4026.0
The result of Model Number:Dataset Number:Featuregroup Number:Rescale of 13 : 1 : 5 :
1
f1-score precision recall support
AFIB 0.719186 0.714483 0.724014 875.0
GSVT 0.740562 0.746863 0.734465 691.0
SB 0.788428 0.786711 0.790154 1553.0
SR 0.769213 0.772302 0.766161 907.0
macro avg 0.754347 0.755090 0.753698 4026.0

micro avg 0.760816 0.760816 0.760816 4026.0
weighted avg 0.760835 0.760928 0.760816 4026.0
The result of Model Number:Dataset Number:Featuregroup Number:Rescale of 13 : 1 : 6 :
0
f1-score precision recall support
AFIB 0.888444 0.876138 0.901136 875.0
GSVT 0.908149 0.915499 0.900921 691.0
SB 0.963989 0.961219 0.966782 1553.0
SR 0.940539 0.952780 0.928617 907.0
macro avg 0.925280 0.926409 0.924364 4026.0
micro avg 0.932613 0.932613 0.932613 4026.0
weighted avg 0.932703 0.932980 0.932613 4026.0
The result of Model Number:Dataset Number:Featuregroup Number:Rescale of 13 : 1 : 6 :
1
f1-score precision recall support
AFIB 0.833768 0.831457 0.836154 875.0
GSVT 0.840366 0.844375 0.836480 691.0
SB 0.879716 0.876905 0.882548 1553.0
SR 0.865652 0.869833 0.861551 907.0
macro avg 0.854876 0.855643 0.854183 4026.0
micro avg 0.859828 0.859828 0.859828 4026.0
weighted avg 0.859808 0.859851 0.859828 4026.0
The result of Model Number:Dataset Number:Featuregroup Number:Rescale of 13 : 1 : 7 :
0
f1-score precision recall support
AFIB 0.673414 0.698275 0.650324 204.0
GSVT 0.687807 0.680748 0.695121 300.0
SB 0.706645 0.703350 0.709973 892.0
SR 0.695627 0.696063 0.695215 681.0
macro avg 0.690873 0.694609 0.687658 2077.0
micro avg 0.697130 0.697130 0.697130 2077.0
weighted avg 0.697048 0.697198 0.697130 2077.0
The result of Model Number:Dataset Number:Featuregroup Number:Rescale of 13 : 1 : 7 :
1
f1-score precision recall support
AFIB 0.778525 0.788924 0.768616 204.0
GSVT 0.792278 0.787451 0.797285 300.0
SB 0.811376 0.810657 0.812102 892.0
SR 0.805380 0.805503 0.805263 681.0
macro avg 0.796890 0.798134 0.795817 2077.0
micro avg 0.803448 0.803448 0.803448 2077.0
weighted avg 0.803425 0.803480 0.803448 2077.0
The result of Model Number:Dataset Number:Featuregroup Number:Rescale of 13 : 1 : 8 :
0

	f1-score	precision	recall	support
AFIB	0.923464	0.940080	0.907698	204.0
GSVT	0.944298	0.931021	0.958116	300.0
SB	0.958827	0.947499	0.970435	892.0
SR	0.939544	0.956271	0.923402	681.0
macro avg	0.941533	0.943718	0.939913	2077.0
micro avg	0.947073	0.947073	0.947073	2077.0
weighted avg	0.946933	0.947266	0.947073	2077.0

The result of Model Number:Dataset Number:Featuregroup Number:Rescale of 13 : 1 : 8 : 1

	f1-score	precision	recall	support
AFIB	0.693819	0.687683	0.700095	875.0
GSVT	0.630802	0.610884	0.701780	691.0
SB	0.568702	0.546033	0.604154	1553.0
SR	0.586130	0.592195	0.580226	907.0
macro avg	0.619864	0.609199	0.646564	4026.0
micro avg	0.636371	0.636371	0.636371	4026.0
weighted avg	0.610480	0.598349	0.636371	4026.0

The result of Model Number:Dataset Number:Featuregroup Number:Rescale of 13 : 1 : 9 : 0

	f1-score	precision	recall	support
AFIB	0.674610	0.673525	0.675975	875.0
GSVT	0.690339	0.686741	0.694015	691.0
SB	0.721786	0.715670	0.728012	1553.0
SR	0.694232	0.709666	0.679689	907.0
macro avg	0.695242	0.696400	0.694423	4026.0
micro avg	0.699981	0.699981	0.699981	4026.0
weighted avg	0.699928	0.700193	0.699981	4026.0

The result of Model Number:Dataset Number:Featuregroup Number:Rescale of 13 : 1 : 9 : 1

	f1-score	precision	recall	support
AFIB	0.640915	0.622450	0.703207	875.0
GSVT	0.706375	0.704118	0.708736	691.0
SB	0.596284	0.587496	0.605387	1553.0
SR	0.713979	0.727915	0.700656	907.0
macro avg	0.664388	0.660495	0.679497	4026.0
micro avg	0.665848	0.665848	0.665848	4026.0
weighted avg	0.651394	0.646744	0.665848	4026.0

The result of Model Number:Dataset Number:Featuregroup Number:Rescale of 13 : 1 : 10 : 0

	f1-score	precision	recall	support
AFIB	0.735066	0.733507	0.736667	875.0
GSVT	0.746550	0.742972	0.750226	691.0
SB	0.785741	0.776361	0.795353	1553.0

SR 0.749226 0.770453 0.729154 907.0
macro avg 0.754146 0.755823 0.752850 4026.0
micro avg 0.759939 0.759939 0.759939 4026.0
weighted avg 0.759775 0.759985 0.759939 4026.0
The result of Model Number:Dataset Number:Featuregroup Number:Rescale of 13 : 1 : 10 : 1

	f1-score	precision	recall	support
AFIB	0.806853	0.812289	0.801608	875.0
GSVT	0.821252	0.809845	0.833136	691.0
SB	0.875134	0.869141	0.881217	1553.0
SR	0.839670	0.854174	0.825696	907.0
macro avg	0.835727	0.836362	0.835414	4026.0
micro avg	0.843155	0.843155	0.843155	4026.0
weighted avg	0.843056	0.843236	0.843155	4026.0

The result of Model Number:Dataset Number:Featuregroup Number:Rescale of 14 : 0 : 0 : 0

	f1-score	precision	recall	support
AFIB	0.745856	0.786737	0.709509	204.0
GSVT	0.710213	0.735113	0.687032	300.0
SB	0.730524	0.702348	0.761067	892.0
SR	0.650186	0.667826	0.633491	681.0
macro avg	0.709195	0.723006	0.697775	2077.0
micro avg	0.703480	0.703480	0.703480	2077.0
weighted avg	0.702755	0.704050	0.703480	2077.0

The result of Model Number:Dataset Number:Featuregroup Number:Rescale of 14 : 0 : 0 : 1

	f1-score	precision	recall	support
AFIB	0.806809	0.836824	0.779235	204.0
GSVT	0.748689	0.783431	0.716966	300.0
SB	0.784281	0.736977	0.838088	892.0
SR	0.688750	0.736604	0.646815	681.0
macro avg	0.757132	0.773459	0.745276	2077.0
micro avg	0.752099	0.752099	0.752099	2077.0
weighted avg	0.750031	0.753371	0.752099	2077.0

The result of Model Number:Dataset Number:Featuregroup Number:Rescale of 14 : 0 : 1 : 0

	f1-score	precision	recall	support
AFIB	0.880463	0.926822	0.838757	204.0
GSVT	0.924676	0.916577	0.932961	300.0
SB	0.948669	0.942813	0.954616	892.0
SR	0.935727	0.933829	0.937655	681.0
macro avg	0.922384	0.930010	0.915997	2077.0
micro avg	0.934548	0.934548	0.934548	2077.0
weighted avg	0.934261	0.934507	0.934548	2077.0

The result of Model Number:Dataset Number:Featuregroup Number:Rescale of 14 : 0 : 1 : 1

	f1-score	precision	recall	support
AFIB	0.633506	0.589113	0.743933	875.0
GSVT	0.543313	0.507259	0.614542	691.0
SB	0.649224	0.643747	0.654811	1553.0
SR	0.625758	0.640926	0.611510	907.0
macro avg	0.612950	0.595261	0.656199	4026.0
micro avg	0.657514	0.657514	0.657514	4026.0
weighted avg	0.622343	0.607811	0.657514	4026.0

The result of Model Number:Dataset Number:Featuregroup Number:Rescale of 14 : 0 : 2 : 0

	f1-score	precision	recall	support
AFIB	0.806872	0.839439	0.776802	204.0
GSVT	0.836898	0.823303	0.851094	300.0
SB	0.858549	0.851647	0.865575	892.0
SR	0.835451	0.841467	0.829564	681.0
macro avg	0.834443	0.838964	0.830759	2077.0
micro avg	0.842957	0.842957	0.842957	2077.0
weighted avg	0.842773	0.843016	0.842957	2077.0

The result of Model Number:Dataset Number:Featuregroup Number:Rescale of 14 : 0 : 2 : 1

	f1-score	precision	recall	support
AFIB	0.683449	0.688232	0.678757	204.0
GSVT	0.699350	0.695903	0.702843	300.0
SB	0.713250	0.712063	0.714450	892.0
SR	0.706152	0.707828	0.704491	681.0
macro avg	0.700550	0.701006	0.700135	2077.0
micro avg	0.706003	0.706003	0.706003	2077.0
weighted avg	0.705988	0.706000	0.706003	2077.0

The result of Model Number:Dataset Number:Featuregroup Number:Rescale of 14 : 0 : 3 : 0

	f1-score	precision	recall	support
AFIB	0.726984	0.733526	0.720787	875.0
GSVT	0.741793	0.726752	0.757645	691.0
SB	0.786677	0.777925	0.795722	1553.0
SR	0.755460	0.777917	0.734442	907.0
macro avg	0.752729	0.754030	0.752149	4026.0
micro avg	0.759095	0.759095	0.759095	4026.0
weighted avg	0.758967	0.759490	0.759095	4026.0

The result of Model Number:Dataset Number:Featuregroup Number:Rescale of 14 : 0 : 3 : 1

	f1-score	precision	recall	support
AFIB	0.864154	0.858314	0.870192	875.0

GSVT 0.885236 0.885589 0.884998 691.0
SB 0.940383 0.937984 0.942805 1553.0
SR 0.914778 0.924999 0.904793 907.0
macro avg 0.901138 0.901721 0.900697 4026.0
micro avg 0.908538 0.908538 0.908538 4026.0
weighted avg 0.908582 0.908750 0.908538 4026.0
The result of Model Number:Dataset Number:Featuregroup Number:Rescale of 14 : 0 : 4 : 0

	f1-score	precision	recall	support
AFIB	0.735523	0.744123	0.727261	204.0
GSVT	0.755757	0.752282	0.759285	300.0
SB	0.766457	0.764074	0.768861	892.0
SR	0.759856	0.762010	0.757747	681.0
macro avg	0.754399	0.755622	0.753289	2077.0
micro avg	0.759748	0.759748	0.759748	2077.0
weighted avg	0.759709	0.759735	0.759748	2077.0

The result of Model Number:Dataset Number:Featuregroup Number:Rescale of 14 : 0 : 4 : 1

	f1-score	precision	recall	support
AFIB	0.862852	0.870377	0.855671	204.0
GSVT	0.892692	0.891235	0.894176	300.0
SB	0.911667	0.909443	0.913912	892.0
SR	0.903309	0.904670	0.901982	681.0
macro avg	0.892630	0.893931	0.891435	2077.0
micro avg	0.901429	0.901429	0.901429	2077.0
weighted avg	0.901391	0.901411	0.901429	2077.0

The result of Model Number:Dataset Number:Featuregroup Number:Rescale of 14 : 0 : 5 : 0

	f1-score	precision	recall	support
AFIB	0.822802	0.821116	0.825091	875.0
GSVT	0.834026	0.825271	0.843119	691.0
SB	0.879617	0.867740	0.891996	1553.0
SR	0.836815	0.868727	0.807355	907.0
macro avg	0.843315	0.845714	0.841890	4026.0
micro avg	0.849997	0.849997	0.849997	4026.0
weighted avg	0.849801	0.850540	0.849997	4026.0

The result of Model Number:Dataset Number:Featuregroup Number:Rescale of 14 : 0 : 5 : 1

	f1-score	precision	recall	support
AFIB	0.686313	0.692544	0.680545	204.0
GSVT	0.708996	0.704711	0.713519	300.0
SB	0.723828	0.722859	0.724802	892.0
SR	0.717131	0.718618	0.715655	681.0
macro avg	0.709067	0.709683	0.708630	2077.0

micro avg 0.715826 0.715826 0.715826 2077.0
weighted avg 0.715805 0.715869 0.715826 2077.0
The result of Model Number:Dataset Number:Featuregroup Number:Rescale of 14 : 0 : 6 : 0

	f1-score	precision	recall	support
AFIB	0.701402	0.686414	0.717100	875.0
GSVT	0.726346	0.737453	0.715634	691.0
SB	0.774284	0.774386	0.774185	1553.0
SR	0.754336	0.762148	0.746686	907.0
macro avg	0.739092	0.740100	0.738401	4026.0
micro avg	0.745534	0.745534	0.745534	4026.0
weighted avg	0.745722	0.746170	0.745534	4026.0

The result of Model Number:Dataset Number:Featuregroup Number:Rescale of 14 : 0 : 6 : 1

	f1-score	precision	recall	support
AFIB	0.708321	0.716603	0.700359	204.0
GSVT	0.638909	0.614516	0.728986	300.0
SB	0.592777	0.591380	0.594186	892.0
SR	0.736029	0.737443	0.734625	681.0
macro avg	0.669009	0.664986	0.689539	2077.0
micro avg	0.670131	0.670131	0.670131	2077.0
weighted avg	0.657757	0.654912	0.670131	2077.0

The result of Model Number:Dataset Number:Featuregroup Number:Rescale of 14 : 0 : 7 : 0

	f1-score	precision	recall	support
AFIB	0.761735	0.773284	0.750545	204.0
GSVT	0.773983	0.767459	0.780661	300.0
SB	0.791151	0.788599	0.793722	892.0
SR	0.782659	0.785566	0.779794	681.0
macro avg	0.777382	0.778727	0.776181	2077.0
micro avg	0.783028	0.783028	0.783028	2077.0
weighted avg	0.782998	0.783047	0.783028	2077.0

The result of Model Number:Dataset Number:Featuregroup Number:Rescale of 14 : 0 : 7 : 1

	f1-score	precision	recall	support
AFIB	0.720070	0.726350	0.714077	204.0
GSVT	0.744972	0.743756	0.746211	300.0
SB	0.760807	0.758952	0.762681	892.0
SR	0.753832	0.754968	0.752725	681.0
macro avg	0.744920	0.746007	0.743923	2077.0
micro avg	0.752264	0.752264	0.752264	2077.0
weighted avg	0.752232	0.752249	0.752264	2077.0

The result of Model Number:Dataset Number:Featuregroup Number:Rescale of 14 : 0 : 8 : 0

	f1-score	precision	recall	support
AFIB	0.755989	0.760138	0.751933	875.0
GSVT	0.757046	0.747475	0.766984	691.0
SB	0.796165	0.792970	0.799393	1553.0
SR	0.779074	0.788532	0.769889	907.0
macro avg	0.772069	0.772279	0.772050	4026.0
micro avg	0.776869	0.776869	0.776869	4026.0
weighted avg	0.776869	0.777026	0.776869	4026.0

The result of Model Number:Dataset Number:Featuregroup Number:Rescale of 14 : 0 : 8 : 1

	f1-score	precision	recall	support
AFIB	0.698566	0.700302	0.696887	875.0
GSVT	0.710597	0.712732	0.708564	691.0
SB	0.753815	0.748698	0.759007	1553.0
SR	0.739471	0.744960	0.734090	907.0
macro avg	0.725612	0.726673	0.724637	4026.0
micro avg	0.731235	0.731235	0.731235	4026.0
weighted avg	0.731158	0.731165	0.731235	4026.0

The result of Model Number:Dataset Number:Featuregroup Number:Rescale of 14 : 0 : 9 : 0

	f1-score	precision	recall	support
AFIB	0.624397	0.688577	0.571544	204.0
GSVT	0.743014	0.732973	0.753408	300.0
SB	0.791257	0.788093	0.794450	892.0
SR	0.769781	0.759375	0.780509	681.0
macro avg	0.732112	0.742255	0.724978	2077.0
micro avg	0.762058	0.762058	0.762058	2077.0
weighted avg	0.760859	0.760941	0.762058	2077.0

The result of Model Number:Dataset Number:Featuregroup Number:Rescale of 14 : 0 : 9 : 1

	f1-score	precision	recall	support
AFIB	0.868224	0.863877	0.872902	875.0
GSVT	0.887841	0.889042	0.886822	691.0
SB	0.945429	0.942316	0.948589	1553.0
SR	0.917664	0.927189	0.908442	907.0
macro avg	0.904790	0.905606	0.904189	4026.0
micro avg	0.912494	0.912494	0.912494	4026.0
weighted avg	0.912511	0.912716	0.912494	4026.0

The result of Model Number:Dataset Number:Featuregroup Number:Rescale of 14 : 0 : 10 : 0

	f1-score	precision	recall	support
AFIB	0.691525	0.736582	0.651968	204.0
GSVT	0.721819	0.704077	0.740577	300.0
SB	0.742988	0.733444	0.752810	892.0

SR 0.723690 0.732331 0.715298 681.0
macro avg 0.720005 0.726608 0.715163 2077.0
micro avg 0.728839 0.728839 0.728839 2077.0
weighted avg 0.728548 0.729145 0.728839 2077.0
The result of Model Number:Dataset Number:Featuregroup Number:Rescale of 14 : 0 : 10 : 1

	f1-score	precision	recall	support
AFIB	0.779574	0.783837	0.775383	875.0
GSVT	0.775859	0.769317	0.782590	691.0
SB	0.816009	0.808281	0.823890	1553.0
SR	0.785640	0.800295	0.771523	907.0
macro avg	0.789270	0.790433	0.788346	4026.0
micro avg	0.794461	0.794461	0.794461	4026.0
weighted avg	0.794357	0.794482	0.794461	4026.0

The result of Model Number:Dataset Number:Featuregroup Number:Rescale of 14 : 1 : 0 : 0

	f1-score	precision	recall	support
AFIB	0.845629	0.854629	0.837284	204.0
GSVT	0.870480	0.864680	0.876614	300.0
SB	0.888875	0.888081	0.889672	892.0
SR	0.881381	0.882557	0.880213	681.0
macro avg	0.871591	0.872487	0.870946	2077.0
micro avg	0.879539	0.879539	0.879539	2077.0
weighted avg	0.879513	0.879604	0.879539	2077.0

The result of Model Number:Dataset Number:Featuregroup Number:Rescale of 14 : 1 : 0 : 1

	f1-score	precision	recall	support
AFIB	0.749176	0.771896	0.727787	875.0
GSVT	0.799756	0.794210	0.805423	691.0
SB	0.875031	0.871684	0.878410	1553.0
SR	0.827568	0.815147	0.840422	907.0
macro avg	0.812883	0.813234	0.813011	4026.0
micro avg	0.824589	0.824589	0.824589	4026.0
weighted avg	0.824066	0.823962	0.824589	4026.0

The result of Model Number:Dataset Number:Featuregroup Number:Rescale of 14 : 1 : 1 : 0

	f1-score	precision	recall	support
AFIB	0.909513	0.926205	0.893851	204.0
GSVT	0.944040	0.936449	0.951927	300.0
SB	0.961552	0.959835	0.963277	892.0
SR	0.952031	0.952879	0.951190	681.0
macro avg	0.941784	0.943842	0.940061	2077.0
micro avg	0.950856	0.950856	0.950856	2077.0
weighted avg	0.950790	0.950874	0.950856	2077.0

The result of Model Number:Dataset Number:Featuregroup Number:Rescale of 14 : 1 : 1 : 1

	f1-score	precision	recall	support
AFIB	0.789723	0.766970	0.866478	875.0
GSVT	0.870380	0.867600	0.873290	691.0
SB	0.734729	0.723901	0.745946	1553.0
SR	0.879750	0.896921	0.863335	907.0
macro avg	0.818646	0.813848	0.837262	4026.0
micro avg	0.820445	0.820445	0.820445	4026.0
weighted avg	0.802635	0.796904	0.820445	4026.0

The result of Model Number:Dataset Number:Featuregroup Number:Rescale of 14 : 1 : 2 : 0

	f1-score	precision	recall	support
AFIB	0.908207	0.921409	0.895789	204.0
GSVT	0.928237	0.915679	0.941219	300.0
SB	0.942207	0.932388	0.952242	892.0
SR	0.923533	0.938815	0.908756	681.0
macro avg	0.925546	0.927073	0.924502	2077.0
micro avg	0.930847	0.930847	0.930847	2077.0
weighted avg	0.930727	0.931003	0.930847	2077.0

The result of Model Number:Dataset Number:Featuregroup Number:Rescale of 14 : 1 : 2 : 1

	f1-score	precision	recall	support
AFIB	0.752594	0.764134	0.741522	204.0
GSVT	0.770439	0.763903	0.777121	300.0
SB	0.783841	0.781312	0.786390	892.0
SR	0.775787	0.778666	0.772938	681.0
macro avg	0.770665	0.772004	0.769493	2077.0
micro avg	0.776234	0.776234	0.776234	2077.0
weighted avg	0.776196	0.776243	0.776234	2077.0

The result of Model Number:Dataset Number:Featuregroup Number:Rescale of 14 : 1 : 3 : 0

	f1-score	precision	recall	support
AFIB	0.795560	0.793260	0.797946	875.0
GSVT	0.810305	0.809646	0.811022	691.0
SB	0.860662	0.857097	0.864263	1553.0
SR	0.834823	0.843995	0.825895	907.0
macro avg	0.825338	0.825999	0.824781	4026.0
micro avg	0.832068	0.832068	0.832068	4026.0
weighted avg	0.832049	0.832127	0.832068	4026.0

The result of Model Number:Dataset Number:Featuregroup Number:Rescale of 14 : 1 : 3 : 1

	f1-score	precision	recall	support
AFIB	0.720215	0.733665	0.797388	204.0

	f1-score	precision	recall	support
GSVT	0.864903	0.847382	0.883601	300.0
SB	0.711510	0.704862	0.718292	892.0
SR	0.866006	0.873240	0.859011	681.0
macro avg	0.790658	0.789787	0.814573	2077.0
micro avg	0.796076	0.796076	0.796076	2077.0
weighted avg	0.785176	0.783484	0.796076	2077.0

The result of Model Number:Dataset Number:Featuregroup Number:Rescale of 14 : 1 : 4 : 0

	f1-score	precision	recall	support
AFIB	0.754484	0.769019	0.740844	204.0
GSVT	0.768652	0.756162	0.781700	300.0
SB	0.781484	0.772164	0.791034	892.0
SR	0.766947	0.781326	0.753100	681.0
macro avg	0.767891	0.769668	0.766670	2077.0
micro avg	0.772319	0.772319	0.772319	2077.0
weighted avg	0.772212	0.772548	0.772319	2077.0

The result of Model Number:Dataset Number:Featuregroup Number:Rescale of 14 : 1 : 4 : 1

	f1-score	precision	recall	support
AFIB	0.765585	0.760281	0.771117	875.0
GSVT	0.777396	0.780551	0.774392	691.0
SB	0.843476	0.834705	0.852437	1553.0
SR	0.800888	0.819524	0.783154	907.0
macro avg	0.796836	0.798765	0.795275	4026.0
micro avg	0.805760	0.805760	0.805760	4026.0
weighted avg	0.805611	0.805815	0.805760	4026.0

The result of Model Number:Dataset Number:Featuregroup Number:Rescale of 14 : 1 : 5 : 0

	f1-score	precision	recall	support
AFIB	0.918337	0.925392	0.911599	204.0
GSVT	0.950677	0.949765	0.951621	300.0
SB	0.970126	0.967867	0.972406	892.0
SR	0.961198	0.962494	0.959928	681.0
macro avg	0.950085	0.951379	0.948888	2077.0
micro avg	0.959340	0.959340	0.959340	2077.0
weighted avg	0.959303	0.959319	0.959340	2077.0

The result of Model Number:Dataset Number:Featuregroup Number:Rescale of 14 : 1 : 5 : 1

	f1-score	precision	recall	support
AFIB	0.819238	0.852304	0.788707	204.0
GSVT	0.849724	0.835921	0.864138	300.0
SB	0.871707	0.864699	0.878841	892.0
SR	0.848255	0.854363	0.842278	681.0
macro avg	0.847231	0.851822	0.843491	2077.0

micro avg 0.855876 0.855876 0.855876 2077.0
weighted avg 0.855689 0.855936 0.855876 2077.0
The result of Model Number:Dataset Number:Featuregroup Number:Rescale of 14 : 1 : 6 : 0

	f1-score	precision	recall	support
AFIB	0.876884	0.881697	0.872180	875.0
GSVT	0.878111	0.867009	0.889638	691.0
SB	0.923485	0.919780	0.927229	1553.0
SR	0.903661	0.914631	0.893007	907.0
macro avg	0.895535	0.895779	0.895514	4026.0
micro avg	0.901103	0.901103	0.901103	4026.0
weighted avg	0.901103	0.901286	0.901103	4026.0

The result of Model Number:Dataset Number:Featuregroup Number:Rescale of 14 : 1 : 6 : 1

	f1-score	precision	recall	support
AFIB	0.779671	0.770236	0.789375	875.0
GSVT	0.790225	0.798327	0.782347	691.0
SB	0.839299	0.836568	0.842055	1553.0
SR	0.820941	0.829377	0.812704	907.0
macro avg	0.807534	0.808627	0.806620	4026.0
micro avg	0.813745	0.813745	0.813745	4026.0
weighted avg	0.813781	0.813968	0.813745	4026.0

The result of Model Number:Dataset Number:Featuregroup Number:Rescale of 14 : 1 : 7 : 0

	f1-score	precision	recall	support
AFIB	0.931179	0.949859	0.913404	204.0
GSVT	0.952759	0.942856	0.962924	300.0
SB	0.970380	0.966605	0.974187	892.0
SR	0.960183	0.964190	0.956232	681.0
macro avg	0.953625	0.955877	0.951687	2077.0
micro avg	0.960703	0.960703	0.960703	2077.0
weighted avg	0.960641	0.960738	0.960703	2077.0

The result of Model Number:Dataset Number:Featuregroup Number:Rescale of 14 : 1 : 7 : 1

	f1-score	precision	recall	support
AFIB	0.829891	0.839113	0.821296	204.0
GSVT	0.855598	0.850727	0.860773	300.0
SB	0.872619	0.871449	0.873792	892.0
SR	0.865195	0.866354	0.864051	681.0
macro avg	0.855826	0.856911	0.854978	2077.0
micro avg	0.863562	0.863562	0.863562	2077.0
weighted avg	0.863530	0.863610	0.863562	2077.0

The result of Model Number:Dataset Number:Featuregroup Number:Rescale of 14 : 1 : 8 : 0

	f1-score	precision	recall	support
AFIB	0.695162	0.698473	0.691914	875.0
GSVT	0.703750	0.690961	0.717094	691.0
SB	0.744621	0.741769	0.747496	1553.0
SR	0.722612	0.734980	0.710664	907.0
macro avg	0.716536	0.716546	0.716792	4026.0
micro avg	0.721900	0.721900	0.721900	4026.0
weighted avg	0.721898	0.722109	0.721900	4026.0

The result of Model Number:Dataset Number:Featuregroup Number:Rescale of 14 : 1 : 8 : 1

	f1-score	precision	recall	support
AFIB	0.820084	0.821006	0.819312	204.0
GSVT	0.838912	0.837039	0.840857	300.0
SB	0.856693	0.855549	0.857844	892.0
SR	0.850277	0.852418	0.848157	681.0
macro avg	0.841491	0.841503	0.841543	2077.0
micro avg	0.848430	0.848430	0.848430	2077.0
weighted avg	0.848425	0.848456	0.848430	2077.0

The result of Model Number:Dataset Number:Featuregroup Number:Rescale of 14 : 1 : 9 : 0

	f1-score	precision	recall	support
AFIB	0.870505	0.892548	0.849606	204.0
GSVT	0.889411	0.879739	0.899445	300.0
SB	0.914462	0.909898	0.919075	892.0
SR	0.900288	0.904327	0.896321	681.0
macro avg	0.893667	0.896628	0.891112	2077.0
micro avg	0.901956	0.901956	0.901956	2077.0
weighted avg	0.901879	0.902011	0.901956	2077.0

The result of Model Number:Dataset Number:Featuregroup Number:Rescale of 14 : 1 : 9 : 1

	f1-score	precision	recall	support
AFIB	0.669446	0.672180	0.666755	875.0
GSVT	0.623383	0.650896	0.598205	691.0
SB	0.669477	0.606459	0.747119	1553.0
SR	0.505010	0.628532	0.422233	907.0
macro avg	0.616829	0.639517	0.608578	4026.0
micro avg	0.630902	0.630902	0.630902	4026.0
weighted avg	0.624507	0.633342	0.630902	4026.0

The result of Model Number:Dataset Number:Featuregroup Number:Rescale of 14 : 1 : 10 : 0

	f1-score	precision	recall	support
AFIB	0.874336	0.890376	0.858931	204.0
GSVT	0.885867	0.876927	0.895009	300.0
SB	0.907688	0.902963	0.912469	892.0

SR 0.895331 0.900941 0.889810 681.0
macro avg 0.890805 0.892802 0.889055 2077.0
micro avg 0.897259 0.897259 0.897259 2077.0
weighted avg 0.897209 0.897303 0.897259 2077.0
The result of Model Number:Dataset Number:Featuregroup Number:Rescale of 14 : 1 : 10 : 1

	f1-score	precision	recall	support
AFIB	0.762407	0.779648	0.745935	875.0
GSVT	0.808325	0.811765	0.804962	691.0
SB	0.877673	0.873537	0.881854	1553.0
SR	0.831584	0.818828	0.844777	907.0
macro avg	0.819997	0.820944	0.819382	4026.0
micro avg	0.830764	0.830764	0.830764	4026.0
weighted avg	0.830336	0.830204	0.830764	4026.0

The result of Model Number:Dataset Number:Featuregroup Number:Rescale of 15 : 0 : 0 : 0

	f1-score	precision	recall	support
AFIB	0.817327	0.807065	0.828090	875.0
GSVT	0.758317	0.781277	0.739204	691.0
SB	0.816449	0.756212	0.889272	1553.0
SR	0.634815	0.776965	0.541320	907.0
macro avg	0.756727	0.780380	0.749472	4026.0
micro avg	0.771830	0.771830	0.771830	4026.0
weighted avg	0.765743	0.776242	0.771830	4026.0

The result of Model Number:Dataset Number:Featuregroup Number:Rescale of 15 : 0 : 0 : 1

	f1-score	precision	recall	support
AFIB	0.654048	0.650135	0.658096	875.0
GSVT	0.659404	0.662964	0.655977	691.0
SB	0.698869	0.696826	0.700935	1553.0
SR	0.683078	0.687984	0.678254	907.0
macro avg	0.673850	0.674477	0.673316	4026.0
micro avg	0.678799	0.678799	0.678799	4026.0
weighted avg	0.678797	0.678875	0.678799	4026.0

The result of Model Number:Dataset Number:Featuregroup Number:Rescale of 15 : 0 : 1 : 0

	f1-score	precision	recall	support
AFIB	0.748882	0.743116	0.754795	875.0
GSVT	0.758605	0.762127	0.755280	691.0
SB	0.812951	0.808626	0.817339	1553.0
SR	0.788999	0.800091	0.778296	907.0
macro avg	0.777359	0.778490	0.776428	4026.0
micro avg	0.784299	0.784299	0.784299	4026.0
weighted avg	0.784302	0.784484	0.784299	4026.0

The result of Model Number:Dataset Number:Featuregroup Number:Rescale of 15 : 0 : 1 : 1

	f1-score	precision	recall	support
AFIB	0.915877	0.924226	0.907797	204.0
GSVT	0.939778	0.936701	0.942905	300.0
SB	0.956958	0.956320	0.957600	892.0
SR	0.948542	0.948269	0.948828	681.0
macro avg	0.940289	0.941379	0.939282	2077.0
micro avg	0.947710	0.947710	0.947710	2077.0
weighted avg	0.947682	0.947694	0.947710	2077.0

The result of Model Number:Dataset Number:Featuregroup Number:Rescale of 15 : 0 : 2 : 0

	f1-score	precision	recall	support
AFIB	0.828953	0.846341	0.812288	204.0
GSVT	0.848183	0.843164	0.853270	300.0
SB	0.868861	0.866826	0.870906	892.0
SR	0.861522	0.861274	0.861778	681.0
macro avg	0.851880	0.854401	0.849560	2077.0
micro avg	0.859608	0.859608	0.859608	2077.0
weighted avg	0.859548	0.859576	0.859608	2077.0

The result of Model Number:Dataset Number:Featuregroup Number:Rescale of 15 : 0 : 2 : 1

	f1-score	precision	recall	support
AFIB	0.928966	0.939829	0.918525	204.0
GSVT	0.837932	0.805941	0.956069	300.0
SB	0.777430	0.775598	0.779278	892.0
SR	0.965305	0.967161	0.963464	681.0
macro avg	0.877408	0.872132	0.904334	2077.0
micro avg	0.878880	0.878880	0.878880	2077.0
weighted avg	0.862652	0.858920	0.878880	2077.0

The result of Model Number:Dataset Number:Featuregroup Number:Rescale of 15 : 0 : 3 : 0

	f1-score	precision	recall	support
AFIB	0.773137	0.779249	0.767146	875.0
GSVT	0.792592	0.790916	0.794304	691.0
SB	0.844926	0.837458	0.852539	1553.0
SR	0.822433	0.830499	0.814542	907.0
macro avg	0.808272	0.809531	0.807133	4026.0
micro avg	0.815425	0.815425	0.815425	4026.0
weighted avg	0.815274	0.815251	0.815425	4026.0

The result of Model Number:Dataset Number:Featuregroup Number:Rescale of 15 : 0 : 3 : 1

	f1-score	precision	recall	support
AFIB	0.676903	0.694624	0.660372	204.0

GSVT 0.636919 0.655430 0.619772 300.0
SB 0.672086 0.648423 0.697553 892.0
SR 0.609164 0.629002 0.590661 681.0
macro avg 0.648768 0.656870 0.642090 2077.0
micro avg 0.647619 0.647619 0.647619 2077.0
weighted avg 0.646849 0.647605 0.647619 2077.0

The result of Model Number:Dataset Number:Featuregroup Number:Rescale of 15 : 0 : 4 : 0

	f1-score	precision	recall	support
AFIB	0.827628	0.832348	0.823098	204.0
GSVT	0.844657	0.838565	0.850847	300.0
SB	0.861716	0.858568	0.864902	892.0
SR	0.852551	0.858149	0.847047	681.0
macro avg	0.846638	0.846907	0.846473	2077.0
micro avg	0.852912	0.852912	0.852912	2077.0
weighted avg	0.852899	0.852966	0.852912	2077.0

The result of Model Number:Dataset Number:Featuregroup Number:Rescale of 15 : 0 : 4 : 1

	f1-score	precision	recall	support
AFIB	0.621066	0.676555	0.655859	204.0
GSVT	0.843580	0.829790	0.858127	300.0
SB	0.718147	0.712677	0.723755	892.0
SR	0.878177	0.868240	0.888369	681.0
macro avg	0.765242	0.771815	0.781528	2077.0
micro avg	0.790468	0.790468	0.790468	2077.0
weighted avg	0.779199	0.777050	0.790468	2077.0

The result of Model Number:Dataset Number:Featuregroup Number:Rescale of 15 : 0 : 5 : 0

	f1-score	precision	recall	support
AFIB	0.857733	0.862239	0.853326	875.0
GSVT	0.872208	0.873827	0.870706	691.0
SB	0.923788	0.917228	0.930448	1553.0
SR	0.906076	0.911659	0.900583	907.0
macro avg	0.889951	0.891238	0.888765	4026.0
micro avg	0.896704	0.896704	0.896704	4026.0
weighted avg	0.896589	0.896573	0.896704	4026.0

The result of Model Number:Dataset Number:Featuregroup Number:Rescale of 15 : 0 : 5 : 1

	f1-score	precision	recall	support
AFIB	0.617066	0.635779	0.599448	875.0
GSVT	0.658726	0.654158	0.663394	691.0
SB	0.720727	0.717970	0.723510	1553.0
SR	0.681634	0.671403	0.692221	907.0
macro avg	0.669538	0.669827	0.669643	4026.0

micro avg 0.679180 0.679180 0.679180 4026.0
weighted avg 0.678749 0.678663 0.679180 4026.0
The result of Model Number:Dataset Number:Featuregroup Number:Rescale of 15 : 0 : 6 : 0

	f1-score	precision	recall	support
AFIB	0.765007	0.769574	0.760516	204.0
GSVT	0.783804	0.780439	0.787200	300.0
SB	0.797228	0.796073	0.798389	892.0
SR	0.789939	0.791570	0.788317	681.0
macro avg	0.783995	0.784414	0.783605	2077.0
micro avg	0.789751	0.789751	0.789751	2077.0
weighted avg	0.789734	0.789736	0.789751	2077.0

The result of Model Number:Dataset Number:Featuregroup Number:Rescale of 15 : 0 : 6 : 1

	f1-score	precision	recall	support
AFIB	0.590644	0.584263	0.672350	204.0
GSVT	0.706668	0.701162	0.712322	300.0
SB	0.576708	0.575502	0.577920	892.0
SR	0.715131	0.714931	0.715341	681.0
macro avg	0.647288	0.643965	0.669483	2077.0
micro avg	0.651665	0.651665	0.651665	2077.0
weighted avg	0.642234	0.640228	0.651665	2077.0

The result of Model Number:Dataset Number:Featuregroup Number:Rescale of 15 : 0 : 7 : 0

	f1-score	precision	recall	support
AFIB	0.847478	0.838114	0.857071	875.0
GSVT	0.862748	0.868060	0.857515	691.0
SB	0.918230	0.916701	0.919767	1553.0
SR	0.895871	0.904248	0.887671	907.0
macro avg	0.881082	0.881781	0.880506	4026.0
micro avg	0.888225	0.888225	0.888225	4026.0
weighted avg	0.888293	0.888467	0.888225	4026.0

The result of Model Number:Dataset Number:Featuregroup Number:Rescale of 15 : 0 : 7 : 1

	f1-score	precision	recall	support
AFIB	0.876604	0.883469	0.869891	875.0
GSVT	0.878916	0.867699	0.890481	691.0
SB	0.921884	0.918186	0.925621	1553.0
SR	0.902407	0.911265	0.893753	907.0
macro avg	0.894953	0.895155	0.894936	4026.0
micro avg	0.900298	0.900298	0.900298	4026.0
weighted avg	0.900280	0.900416	0.900298	4026.0

The result of Model Number:Dataset Number:Featuregroup Number:Rescale of 15 : 0 : 8 : 0

	f1-score	precision	recall	support
AFIB	0.784236	0.766414	0.802960	875.0
GSVT	0.811112	0.824266	0.798438	691.0
SB	0.865368	0.865373	0.865369	1553.0
SR	0.843220	0.852826	0.833830	907.0
macro avg	0.825984	0.827220	0.825149	4026.0
micro avg	0.833212	0.833212	0.833212	4026.0
weighted avg	0.833433	0.833984	0.833212	4026.0

The result of Model Number:Dataset Number:Featuregroup Number:Rescale of 15 : 0 : 8 : 1

	f1-score	precision	recall	support
AFIB	0.858167	0.866087	0.850438	875.0
GSVT	0.878206	0.875835	0.880620	691.0
SB	0.935302	0.926510	0.944271	1553.0
SR	0.909460	0.918585	0.900532	907.0
macro avg	0.895284	0.896754	0.893965	4026.0
micro avg	0.903099	0.903099	0.903099	4026.0
weighted avg	0.902916	0.902895	0.903099	4026.0

The result of Model Number:Dataset Number:Featuregroup Number:Rescale of 15 : 0 : 9 : 0

	f1-score	precision	recall	support
AFIB	0.906411	0.929754	0.884248	204.0
GSVT	0.831791	0.794764	0.938675	300.0
SB	0.954304	0.952071	0.956549	892.0
SR	0.757067	0.757209	0.756934	681.0
macro avg	0.862393	0.858450	0.884102	2077.0
micro avg	0.881417	0.881417	0.881417	2077.0
weighted avg	0.867235	0.863267	0.881417	2077.0

The result of Model Number:Dataset Number:Featuregroup Number:Rescale of 15 : 0 : 9 : 1

	f1-score	precision	recall	support
AFIB	0.889019	0.907743	0.871191	204.0
GSVT	0.916659	0.908285	0.925211	300.0
SB	0.931630	0.929553	0.933719	892.0
SR	0.920558	0.921521	0.919608	681.0
macro avg	0.914466	0.916775	0.912432	2077.0
micro avg	0.921722	0.921722	0.921722	2077.0
weighted avg	0.921652	0.921705	0.921722	2077.0

The result of Model Number:Dataset Number:Featuregroup Number:Rescale of 15 : 0 : 10 : 0

	f1-score	precision	recall	support
AFIB	0.853270	0.864570	0.842391	204.0
GSVT	0.879019	0.875847	0.882235	300.0
SB	0.889968	0.887198	0.892759	892.0

SR 0.880271 0.881966 0.878606 681.0
macro avg 0.875632 0.877395 0.873998 2077.0
micro avg 0.881652 0.881652 0.881652 2077.0
weighted avg 0.881603 0.881621 0.881652 2077.0
The result of Model Number:Dataset Number:Featuregroup Number:Rescale of 15 : 0 : 10 : 1

	f1-score	precision	recall	support
AFIB	0.606245	0.602428	0.682894	204.0
GSVT	0.739023	0.735222	0.742988	300.0
SB	0.760065	0.756449	0.763725	892.0
SR	0.596186	0.598150	0.594594	681.0
macro avg	0.675380	0.673062	0.696050	2077.0
micro avg	0.697336	0.697336	0.697336	2077.0
weighted avg	0.688186	0.686353	0.697336	2077.0

The result of Model Number:Dataset Number:Featuregroup Number:Rescale of 15 : 1 : 0 : 0

	f1-score	precision	recall	support
AFIB	0.861156	0.868975	0.853919	204.0
GSVT	0.889618	0.884242	0.895293	300.0
SB	0.908230	0.907013	0.909451	892.0
SR	0.899826	0.901692	0.897974	681.0
macro avg	0.889707	0.890480	0.889159	2077.0
micro avg	0.898189	0.898189	0.898189	2077.0
weighted avg	0.898163	0.898243	0.898189	2077.0

The result of Model Number:Dataset Number:Featuregroup Number:Rescale of 15 : 1 : 0 : 1

	f1-score	precision	recall	support
AFIB	0.835965	0.845061	0.827306	204.0
GSVT	0.864255	0.860651	0.868029	300.0
SB	0.881965	0.881176	0.882756	892.0
SR	0.873630	0.873638	0.873630	681.0
macro avg	0.863954	0.865131	0.862930	2077.0
micro avg	0.872191	0.872191	0.872191	2077.0
weighted avg	0.872156	0.872193	0.872191	2077.0

The result of Model Number:Dataset Number:Featuregroup Number:Rescale of 15 : 1 : 1 : 0

	f1-score	precision	recall	support
AFIB	0.889921	0.906275	0.874255	204.0
GSVT	0.904869	0.898943	0.910898	300.0
SB	0.920347	0.915854	0.924887	892.0
SR	0.910834	0.914621	0.907092	681.0
macro avg	0.906493	0.908923	0.904283	2077.0
micro avg	0.912059	0.912059	0.912059	2077.0
weighted avg	0.912004	0.912066	0.912059	2077.0

The result of Model Number:Dataset Number:Featuregroup Number:Rescale of 15 : 1 : 1 : 1

	f1-score	precision	recall	support
AFIB	0.808253	0.826548	0.790854	204.0
GSVT	0.829762	0.825699	0.833901	300.0
SB	0.846737	0.844292	0.849202	892.0
SR	0.841297	0.840933	0.841673	681.0
macro avg	0.831512	0.834368	0.828908	2077.0
micro avg	0.838793	0.838793	0.838793	2077.0
weighted avg	0.838721	0.838762	0.838793	2077.0

The result of Model Number:Dataset Number:Featuregroup Number:Rescale of 15 : 1 : 2 : 0

	f1-score	precision	recall	support
AFIB	0.790016	0.781481	0.899301	204.0
GSVT	0.945203	0.937838	0.952765	300.0
SB	0.771376	0.769762	0.772997	892.0
SR	0.956523	0.956255	0.956803	681.0
macro avg	0.865779	0.861334	0.895467	2077.0
micro avg	0.871634	0.871634	0.871634	2077.0
weighted avg	0.859019	0.856337	0.871634	2077.0

The result of Model Number:Dataset Number:Featuregroup Number:Rescale of 15 : 1 : 2 : 1

	f1-score	precision	recall	support
AFIB	0.721112	0.720307	0.721941	875.0
GSVT	0.734303	0.725119	0.743744	691.0
SB	0.771381	0.765180	0.777690	1553.0
SR	0.736966	0.756302	0.718606	907.0
macro avg	0.740941	0.741727	0.740495	4026.0
micro avg	0.746437	0.746437	0.746437	4026.0
weighted avg	0.746339	0.746551	0.746437	4026.0

The result of Model Number:Dataset Number:Featuregroup Number:Rescale of 15 : 1 : 3 : 0

	f1-score	precision	recall	support
AFIB	0.824749	0.814238	0.835563	875.0
GSVT	0.787485	0.792904	0.782161	691.0
SB	0.833805	0.785623	0.888303	1553.0
SR	0.679997	0.786738	0.598799	907.0
macro avg	0.781509	0.794876	0.776206	4026.0
micro avg	0.793402	0.793402	0.793402	4026.0
weighted avg	0.789236	0.793343	0.793402	4026.0

The result of Model Number:Dataset Number:Featuregroup Number:Rescale of 15 : 1 : 3 : 1

	f1-score	precision	recall	support
AFIB	0.740228	0.728377	0.832907	204.0

GSVT 0.866612 0.856556 0.877222 300.0
SB 0.895629 0.892247 0.899043 892.0
SR 0.702727 0.706360 0.699250 681.0
macro avg 0.801299 0.795885 0.827105 2077.0
micro avg 0.823888 0.823888 0.823888 2077.0
weighted avg 0.812926 0.810049 0.823888 2077.0
The result of Model Number:Dataset Number:Featuregroup Number:Rescale of 15 : 1 : 4 : 0

	f1-score	precision	recall	support
AFIB	0.676711	0.670520	0.683048	875.0
GSVT	0.685382	0.691430	0.679542	691.0
SB	0.728539	0.724699	0.732432	1553.0
SR	0.712070	0.720651	0.703756	907.0
macro avg	0.700676	0.701825	0.699694	4026.0
micro avg	0.706161	0.706161	0.706161	4026.0
weighted avg	0.706158	0.706302	0.706161	4026.0

The result of Model Number:Dataset Number:Featuregroup Number:Rescale of 15 : 1 : 4 : 1

	f1-score	precision	recall	support
AFIB	0.733628	0.764257	0.705492	875.0
GSVT	0.792574	0.788562	0.796724	691.0
SB	0.869082	0.864191	0.874052	1553.0
SR	0.813764	0.795694	0.832688	907.0
macro avg	0.802262	0.803176	0.802239	4026.0
micro avg	0.814826	0.814826	0.814826	4026.0
weighted avg	0.814049	0.814060	0.814826	4026.0

The result of Model Number:Dataset Number:Featuregroup Number:Rescale of 15 : 1 : 5 : 0

	f1-score	precision	recall	support
AFIB	0.695844	0.725274	0.668941	204.0
GSVT	0.640822	0.641944	0.649317	300.0
SB	0.689904	0.657038	0.726901	892.0
SR	0.609909	0.659033	0.568704	681.0
macro avg	0.659120	0.670822	0.653466	2077.0
micro avg	0.658133	0.658133	0.658133	2077.0
weighted avg	0.657170	0.662214	0.658133	2077.0

The result of Model Number:Dataset Number:Featuregroup Number:Rescale of 15 : 1 : 5 : 1

	f1-score	precision	recall	support
AFIB	0.868568	0.895416	0.844040	204.0
GSVT	0.810797	0.828423	0.794503	300.0
SB	0.855185	0.817231	0.896880	892.0
SR	0.770001	0.809568	0.734230	681.0
macro avg	0.826138	0.837659	0.817413	2077.0

micro avg 0.823574 0.823574 0.823574 2077.0
weighted avg 0.822158 0.824014 0.823574 2077.0
The result of Model Number:Dataset Number:Featuregroup Number:Rescale of 15 : 1 : 6 : 0

	f1-score	precision	recall	support
AFIB	0.777382	0.855813	0.712575	204.0
GSVT	0.916761	0.904106	0.929920	300.0
SB	0.973422	0.970174	0.976696	892.0
SR	0.948494	0.935371	0.962002	681.0
macro avg	0.904015	0.916366	0.895298	2077.0
micro avg	0.939180	0.939180	0.939180	2077.0
weighted avg	0.937810	0.937988	0.939180	2077.0

The result of Model Number:Dataset Number:Featuregroup Number:Rescale of 15 : 1 : 6 : 1

	f1-score	precision	recall	support
AFIB	0.812231	0.813801	0.810729	875.0
GSVT	0.786915	0.783458	0.790449	691.0
SB	0.822233	0.772257	0.879146	1553.0
SR	0.661782	0.766813	0.582215	907.0
macro avg	0.770790	0.784082	0.765635	4026.0
micro avg	0.782159	0.782159	0.782159	4026.0
weighted avg	0.777850	0.781982	0.782159	4026.0

The result of Model Number:Dataset Number:Featuregroup Number:Rescale of 15 : 1 : 7 : 0

	f1-score	precision	recall	support
AFIB	0.786192	0.776876	0.795800	875.0
GSVT	0.797382	0.805090	0.789898	691.0
SB	0.846288	0.843050	0.849557	1553.0
SR	0.826558	0.836094	0.817260	907.0
macro avg	0.814105	0.815277	0.813129	4026.0
micro avg	0.820358	0.820358	0.820358	4026.0
weighted avg	0.820388	0.820585	0.820358	4026.0

The result of Model Number:Dataset Number:Featuregroup Number:Rescale of 15 : 1 : 7 : 1

	f1-score	precision	recall	support
AFIB	0.917155	0.925614	0.909070	204.0
GSVT	0.950685	0.950719	0.950677	300.0
SB	0.970595	0.968871	0.972334	892.0
SR	0.960294	0.960037	0.960575	681.0
macro avg	0.949682	0.951310	0.948164	2077.0
micro avg	0.959137	0.959137	0.959137	2077.0
weighted avg	0.959093	0.959104	0.959137	2077.0

The result of Model Number:Dataset Number:Featuregroup Number:Rescale of 15 : 1 : 8 : 0

	f1-score	precision	recall	support
AFIB	0.722323	0.728623	0.716312	204.0
GSVT	0.747303	0.746083	0.748546	300.0
SB	0.763188	0.761326	0.765068	892.0
SR	0.756191	0.757331	0.755080	681.0
macro avg	0.747251	0.748341	0.746251	2077.0
micro avg	0.754618	0.754618	0.754618	2077.0
weighted avg	0.754586	0.754602	0.754618	2077.0

The result of Model Number:Dataset Number:Featuregroup Number:Rescale of 15 : 1 : 8 : 1

	f1-score	precision	recall	support
AFIB	0.827951	0.829551	0.826420	875.0
GSVT	0.802145	0.798621	0.805748	691.0
SB	0.838146	0.787203	0.896161	1553.0
SR	0.674590	0.781654	0.593483	907.0
macro avg	0.785708	0.799257	0.780453	4026.0
micro avg	0.797297	0.797297	0.797297	4026.0
weighted avg	0.792904	0.797116	0.797297	4026.0

The result of Model Number:Dataset Number:Featuregroup Number:Rescale of 15 : 1 : 9 : 0

	f1-score	precision	recall	support
AFIB	0.781891	0.810880	0.755015	204.0
GSVT	0.805315	0.795246	0.815746	300.0
SB	0.825737	0.818202	0.833418	892.0
SR	0.804027	0.810267	0.797891	681.0
macro avg	0.804243	0.808649	0.800517	2077.0
micro avg	0.811516	0.811516	0.811516	2077.0
weighted avg	0.811363	0.811565	0.811516	2077.0

The result of Model Number:Dataset Number:Featuregroup Number:Rescale of 15 : 1 : 9 : 1

	f1-score	precision	recall	support
AFIB	0.678183	0.669287	0.687350	875.0
GSVT	0.688566	0.696220	0.681091	691.0
SB	0.730313	0.728589	0.732049	1553.0
SR	0.712028	0.718437	0.705743	907.0
macro avg	0.702273	0.703133	0.701558	4026.0
micro avg	0.707662	0.707662	0.707662	4026.0
weighted avg	0.707699	0.707858	0.707662	4026.0

The result of Model Number:Dataset Number:Featuregroup Number:Rescale of 15 : 1 : 10 : 0

	f1-score	precision	recall	support
AFIB	0.147974	0.149528	0.146452	175.0
GSVT	0.148212	0.146637	0.149821	138.2
SB	0.156093	0.155495	0.156696	310.6

SR 0.152481 0.153245 0.151724 181.4
macro avg 0.151190 0.151226 0.151173 805.2
micro avg 0.152170 0.152170 0.152170 805.2
weighted avg 0.152162 0.152171 0.152170 805.2
The result of Model Number:Dataset Number:Featuregroup Number:Rescale of 15 : 1 : 10 : 1

	f1-score	precision	recall	support
AFIB	0.848539	0.854598	0.842717	204.0
GSVT	0.878210	0.877643	0.878793	300.0
SB	0.895781	0.893793	0.897786	892.0
SR	0.887774	0.888848	0.886729	681.0
macro avg	0.877576	0.878720	0.876507	2077.0
micro avg	0.886009	0.886009	0.886009	2077.0
weighted avg	0.885977	0.885989	0.886009	2077.0

The result of Model Number:Dataset Number:Featuregroup Number:Rescale of 16 : 0 : 0 : 0

	f1-score	precision	recall	support
AFIB	0.661756	0.663082	0.660463	875.0
GSVT	0.663395	0.659813	0.667033	691.0
SB	0.693470	0.689765	0.697220	1553.0
SR	0.676880	0.684907	0.669058	907.0
macro avg	0.673875	0.674392	0.673444	4026.0
micro avg	0.677706	0.677706	0.677706	4026.0
weighted avg	0.677678	0.677731	0.677706	4026.0

The result of Model Number:Dataset Number:Featuregroup Number:Rescale of 16 : 0 : 0 : 1

	f1-score	precision	recall	support
AFIB	0.817660	0.817778	0.817651	875.0
GSVT	0.831941	0.825044	0.839015	691.0
SB	0.871031	0.863496	0.878712	1553.0
SR	0.842328	0.861357	0.824152	907.0
macro avg	0.840740	0.841919	0.839882	4026.0
micro avg	0.846336	0.846336	0.846336	4026.0
weighted avg	0.846256	0.846478	0.846336	4026.0

The result of Model Number:Dataset Number:Featuregroup Number:Rescale of 16 : 0 : 1 : 0

	f1-score	precision	recall	support
AFIB	0.735402	0.757704	0.714406	875.0
GSVT	0.785052	0.779607	0.790614	691.0
SB	0.858942	0.855656	0.862259	1553.0
SR	0.812352	0.800160	0.824970	907.0
macro avg	0.797937	0.798282	0.798062	4026.0
micro avg	0.809427	0.809427	0.809427	4026.0
weighted avg	0.808914	0.808812	0.809427	4026.0

The result of Model Number:Dataset Number:Featuregroup Number:Rescale of 16 : 0 : 1 : 1

	f1-score	precision	recall	support
AFIB	0.855763	0.849174	0.862521	875.0
GSVT	0.866874	0.870898	0.863075	691.0
SB	0.928976	0.924034	0.933991	1553.0
SR	0.901605	0.914281	0.889375	907.0
macro avg	0.888304	0.889597	0.887240	4026.0
micro avg	0.896235	0.896235	0.896235	4026.0
weighted avg	0.896239	0.896447	0.896235	4026.0

The result of Model Number:Dataset Number:Featuregroup Number:Rescale of 16 : 0 : 2 : 0

	f1-score	precision	recall	support
AFIB	0.830076	0.865652	0.797624	204.0
GSVT	0.857969	0.845006	0.871391	300.0
SB	0.874987	0.866333	0.883817	892.0
SR	0.851281	0.858564	0.844152	681.0
macro avg	0.853578	0.858889	0.849246	2077.0
micro avg	0.860551	0.860551	0.860551	2077.0
weighted avg	0.860345	0.860638	0.860551	2077.0

The result of Model Number:Dataset Number:Featuregroup Number:Rescale of 16 : 0 : 2 : 1

	f1-score	precision	recall	support
AFIB	0.838200	0.854260	0.822945	204.0
GSVT	0.864854	0.860399	0.869436	300.0
SB	0.886243	0.884862	0.887634	892.0
SR	0.877510	0.876638	0.878411	681.0
macro avg	0.866701	0.869040	0.864606	2077.0
micro avg	0.875628	0.875628	0.875628	2077.0
weighted avg	0.875571	0.875627	0.875628	2077.0

The result of Model Number:Dataset Number:Featuregroup Number:Rescale of 16 : 0 : 3 : 0

	f1-score	precision	recall	support
AFIB	0.772907	0.784758	0.761536	204.0
GSVT	0.791234	0.784521	0.798096	300.0
SB	0.804997	0.802400	0.807615	892.0
SR	0.796726	0.799683	0.793800	681.0
macro avg	0.791466	0.792840	0.790261	2077.0
micro avg	0.797184	0.797184	0.797184	2077.0
weighted avg	0.797145	0.797194	0.797184	2077.0

The result of Model Number:Dataset Number:Featuregroup Number:Rescale of 16 : 0 : 3 : 1

	f1-score	precision	recall	support
AFIB	0.835896	0.851231	0.821168	204.0

GSVT 0.846921 0.838374 0.855661 300.0
SB 0.867782 0.863265 0.872353 892.0
SR 0.855968 0.861332 0.850690 681.0
macro avg 0.851642 0.853550 0.849968 2077.0
micro avg 0.857812 0.857812 0.857812 2077.0
weighted avg 0.857764 0.857854 0.857812 2077.0
The result of Model Number:Dataset Number:Featuregroup Number:Rescale of 16 : 0 : 4 : 0

	f1-score	precision	recall	support
AFIB	0.828775	0.845694	0.812887	204.0
GSVT	0.858869	0.851408	0.866620	300.0
SB	0.875382	0.873820	0.876953	892.0
SR	0.867234	0.867752	0.866723	681.0
macro avg	0.857565	0.859668	0.855796	2077.0
micro avg	0.865814	0.865814	0.865814	2077.0
weighted avg	0.865748	0.865830	0.865814	2077.0

The result of Model Number:Dataset Number:Featuregroup Number:Rescale of 16 : 0 : 4 : 1

	f1-score	precision	recall	support
AFIB	0.650960	0.652770	0.649193	875.0
GSVT	0.665871	0.665686	0.666062	691.0
SB	0.709174	0.702508	0.715975	1553.0
SR	0.690383	0.700339	0.680714	907.0
macro avg	0.679097	0.680326	0.677986	4026.0
micro avg	0.684950	0.684950	0.684950	4026.0
weighted avg	0.684856	0.684890	0.684950	4026.0

The result of Model Number:Dataset Number:Featuregroup Number:Rescale of 16 : 0 : 5 : 0

	f1-score	precision	recall	support
AFIB	0.685307	0.695416	0.675562	204.0
GSVT	0.702130	0.699375	0.704931	300.0
SB	0.717724	0.716285	0.719172	892.0
SR	0.712633	0.712741	0.712533	681.0
macro avg	0.704449	0.705954	0.703050	2077.0
micro avg	0.710655	0.710655	0.710655	2077.0
weighted avg	0.710619	0.710631	0.710655	2077.0

The result of Model Number:Dataset Number:Featuregroup Number:Rescale of 16 : 0 : 5 : 1

	f1-score	precision	recall	support
AFIB	0.722624	0.705782	0.740406	875.0
GSVT	0.693693	0.701318	0.686279	691.0
SB	0.730001	0.682862	0.784148	1553.0
SR	0.584252	0.701574	0.500859	907.0
macro avg	0.682643	0.697884	0.677923	4026.0

micro avg 0.694023 0.694023 0.694023 4026.0
weighted avg 0.689331 0.695227 0.694023 4026.0
The result of Model Number:Dataset Number:Featuregroup Number:Rescale of 16 : 0 : 6 : 0

	f1-score	precision	recall	support
AFIB	0.807885	0.810833	0.805012	875.0
GSVT	0.821849	0.811147	0.832907	691.0
SB	0.866311	0.856770	0.876073	1553.0
SR	0.821015	0.843695	0.799567	907.0
macro avg	0.829265	0.830611	0.828390	4026.0
micro avg	0.835984	0.835984	0.835984	4026.0
weighted avg	0.835777	0.836010	0.835984	4026.0

The result of Model Number:Dataset Number:Featuregroup Number:Rescale of 16 : 0 : 6 : 1

	f1-score	precision	recall	support
AFIB	0.849293	0.850441	0.848309	875.0
GSVT	0.862520	0.862077	0.862996	691.0
SB	0.900813	0.897590	0.904063	1553.0
SR	0.879242	0.884170	0.874425	907.0
macro avg	0.872967	0.873570	0.872448	4026.0
micro avg	0.878220	0.878220	0.878220	4026.0
weighted avg	0.878184	0.878224	0.878220	4026.0

The result of Model Number:Dataset Number:Featuregroup Number:Rescale of 16 : 0 : 7 : 0

	f1-score	precision	recall	support
AFIB	0.890629	0.889196	0.892430	875.0
GSVT	0.911394	0.906643	0.916248	691.0
SB	0.952910	0.944837	0.961130	1553.0
SR	0.916534	0.936910	0.897334	907.0
macro avg	0.917867	0.919396	0.916785	4026.0
micro avg	0.924123	0.924123	0.924123	4026.0
weighted avg	0.924053	0.924403	0.924123	4026.0

The result of Model Number:Dataset Number:Featuregroup Number:Rescale of 16 : 0 : 7 : 1

	f1-score	precision	recall	support
AFIB	0.605632	0.590134	0.622022	875.0
GSVT	0.572509	0.604957	0.543498	691.0
SB	0.607060	0.558641	0.664684	1553.0
SR	0.484939	0.589021	0.412203	907.0
macro avg	0.567535	0.585688	0.560602	4026.0
micro avg	0.577732	0.577732	0.577732	4026.0
weighted avg	0.573307	0.580279	0.577732	4026.0

The result of Model Number:Dataset Number:Featuregroup Number:Rescale of 16 : 0 : 8 : 0

	f1-score	precision	recall	support
AFIB	0.776688	0.779859	0.773566	875.0
GSVT	0.723246	0.755166	0.694034	691.0
SB	0.776723	0.703611	0.866803	1553.0
SR	0.585909	0.729219	0.489873	907.0
macro avg	0.715642	0.741964	0.706069	4026.0
micro avg	0.731969	0.731969	0.731969	4026.0
weighted avg	0.724549	0.734800	0.731969	4026.0

The result of Model Number:Dataset Number:Featuregroup Number:Rescale of 16 : 0 : 8 : 1

	f1-score	precision	recall	support
AFIB	0.699980	0.703449	0.696620	875.0
GSVT	0.706950	0.707969	0.706041	691.0
SB	0.744160	0.740708	0.747653	1553.0
SR	0.730461	0.732291	0.728681	907.0
macro avg	0.720388	0.721104	0.719749	4026.0
micro avg	0.725146	0.725146	0.725146	4026.0
weighted avg	0.725086	0.725095	0.725146	4026.0

The result of Model Number:Dataset Number:Featuregroup Number:Rescale of 16 : 0 : 9 : 0

	f1-score	precision	recall	support
AFIB	0.700247	0.722692	0.679363	204.0
GSVT	0.721196	0.708788	0.734152	300.0
SB	0.733672	0.726419	0.741073	892.0
SR	0.714055	0.722990	0.705353	681.0
macro avg	0.717292	0.720222	0.714985	2077.0
micro avg	0.722300	0.722300	0.722300	2077.0
weighted avg	0.722155	0.722382	0.722300	2077.0

The result of Model Number:Dataset Number:Featuregroup Number:Rescale of 16 : 0 : 9 : 1

	f1-score	precision	recall	support
AFIB	0.867674	0.874264	0.861256	204.0
GSVT	0.885437	0.876789	0.894277	300.0
SB	0.906091	0.903775	0.908427	892.0
SR	0.896480	0.901544	0.891483	681.0
macro avg	0.888920	0.889093	0.888861	2077.0
micro avg	0.896195	0.896195	0.896195	2077.0
weighted avg	0.896183	0.896247	0.896195	2077.0

The result of Model Number:Dataset Number:Featuregroup Number:Rescale of 16 : 0 : 10 : 0

	f1-score	precision	recall	support
AFIB	0.764693	0.755674	0.773961	875.0
GSVT	0.779719	0.786501	0.773061	691.0
SB	0.830988	0.827808	0.834197	1553.0

	f1-score	precision	recall	support
SR	0.811381	0.821191	0.801812	907.0
macro avg	0.796695	0.797793	0.795758	4026.0
micro avg	0.803316	0.803316	0.803316	4026.0
weighted avg	0.803363	0.803550	0.803316	4026.0

The result of Model Number:Dataset Number:Featuregroup Number:Rescale of 16 : 0 : 10 : 1

	f1-score	precision	recall	support
AFIB	0.828772	0.830876	0.826709	875.0
GSVT	0.843397	0.834261	0.852770	691.0
SB	0.888894	0.878839	0.899196	1553.0
SR	0.848252	0.871475	0.826269	907.0
macro avg	0.852329	0.853863	0.851236	4026.0
micro avg	0.859044	0.859044	0.859044	4026.0
weighted avg	0.858862	0.859105	0.859044	4026.0

The result of Model Number:Dataset Number:Featuregroup Number:Rescale of 16 : 1 : 0 : 0

	f1-score	precision	recall	support
AFIB	0.765216	0.760398	0.861963	204.0
GSVT	0.932811	0.928013	0.937816	300.0
SB	0.959371	0.954807	0.963990	892.0
SR	0.752519	0.754998	0.750509	681.0
macro avg	0.852479	0.849554	0.878570	2077.0
micro avg	0.880193	0.880193	0.880193	2077.0
weighted avg	0.868643	0.866330	0.880193	2077.0

The result of Model Number:Dataset Number:Featuregroup Number:Rescale of 16 : 1 : 0 : 1

	f1-score	precision	recall	support
AFIB	0.747896	0.750470	0.745435	875.0
GSVT	0.762095	0.746890	0.778069	691.0
SB	0.806738	0.802376	0.811153	1553.0
SR	0.775535	0.793567	0.758351	907.0
macro avg	0.773066	0.773326	0.773252	4026.0
micro avg	0.779296	0.779296	0.779296	4026.0
weighted avg	0.779258	0.779587	0.779296	4026.0

The result of Model Number:Dataset Number:Featuregroup Number:Rescale of 16 : 1 : 1 : 0

	f1-score	precision	recall	support
AFIB	0.775519	0.763103	0.872616	204.0
GSVT	0.907928	0.897393	0.919045	300.0
SB	0.938329	0.934786	0.941905	892.0
SR	0.736230	0.740037	0.732587	681.0
macro avg	0.839502	0.833830	0.866538	2077.0
micro avg	0.863167	0.863167	0.863167	2077.0
weighted avg	0.851683	0.848669	0.863167	2077.0

The result of Model Number:Dataset Number:Featuregroup Number:Rescale of 16 : 1 : 1 : 1

	f1-score	precision	recall	support
AFIB	0.698534	0.710147	0.687455	204.0
GSVT	0.711928	0.706870	0.717112	300.0
SB	0.728441	0.724347	0.732598	892.0
SR	0.719161	0.723561	0.714837	681.0
macro avg	0.714516	0.716231	0.713001	2077.0
micro avg	0.720104	0.720104	0.720104	2077.0
weighted avg	0.720076	0.720170	0.720104	2077.0

The result of Model Number:Dataset Number:Featuregroup Number:Rescale of 16 : 1 : 2 : 0

	f1-score	precision	recall	support
AFIB	0.790629	0.768700	0.816334	875.0
GSVT	0.800950	0.834687	0.774763	691.0
SB	0.879872	0.879427	0.880328	1553.0
SR	0.857798	0.863166	0.852502	907.0
macro avg	0.832312	0.836495	0.830982	4026.0
micro avg	0.842032	0.842032	0.842032	4026.0
weighted avg	0.841957	0.844020	0.842032	4026.0

The result of Model Number:Dataset Number:Featuregroup Number:Rescale of 16 : 1 : 2 : 1

	f1-score	precision	recall	support
AFIB	0.870306	0.861552	0.879264	875.0
GSVT	0.878094	0.885912	0.870477	691.0
SB	0.928985	0.926134	0.931855	1553.0
SR	0.910995	0.918969	0.903162	907.0
macro avg	0.897095	0.898142	0.896189	4026.0
micro avg	0.903426	0.903426	0.903426	4026.0
weighted avg	0.903444	0.903580	0.903426	4026.0

The result of Model Number:Dataset Number:Featuregroup Number:Rescale of 16 : 1 : 3 : 0

	f1-score	precision	recall	support
AFIB	0.914115	0.923019	0.905604	204.0
GSVT	0.935254	0.930960	0.939620	300.0
SB	0.948178	0.944916	0.951470	892.0
SR	0.940171	0.943819	0.936596	681.0
macro avg	0.934430	0.935678	0.933323	2077.0
micro avg	0.940377	0.940377	0.940377	2077.0
weighted avg	0.940341	0.940390	0.940377	2077.0

The result of Model Number:Dataset Number:Featuregroup Number:Rescale of 16 : 1 : 3 : 1

	f1-score	precision	recall	support
AFIB	0.739758	0.819664	0.674184	204.0

GSVT 0.857538 0.843591 0.872089 300.0
SB 0.914511 0.910348 0.918714 892.0
SR 0.892415 0.881058 0.904080 681.0
macro avg 0.851056 0.863665 0.842267 2077.0
micro avg 0.883164 0.883164 0.883164 2077.0
weighted avg 0.881873 0.882195 0.883164 2077.0
The result of Model Number:Dataset Number:Featuregroup Number:Rescale of 16 : 1 : 4 : 0

	f1-score	precision	recall	support
AFIB	0.888444	0.903870	0.873649	204.0
GSVT	0.905952	0.903271	0.908667	300.0
SB	0.916635	0.913373	0.919923	892.0
SR	0.909182	0.910131	0.908255	681.0
macro avg	0.905053	0.907661	0.902623	2077.0
micro avg	0.909926	0.909926	0.909926	2077.0
weighted avg	0.909879	0.909918	0.909926	2077.0

The result of Model Number:Dataset Number:Featuregroup Number:Rescale of 16 : 1 : 4 : 1

	f1-score	precision	recall	support
AFIB	0.603027	0.585653	0.661637	875.0
GSVT	0.664617	0.662494	0.666839	691.0
SB	0.561034	0.552766	0.569599	1553.0
SR	0.671771	0.684883	0.659236	907.0
macro avg	0.625112	0.621449	0.639328	4026.0
micro avg	0.626486	0.626486	0.626486	4026.0
weighted avg	0.612887	0.608511	0.626486	4026.0

The result of Model Number:Dataset Number:Featuregroup Number:Rescale of 16 : 1 : 5 : 0

	f1-score	precision	recall	support
AFIB	0.742258	0.760741	0.724687	875.0
GSVT	0.784249	0.787578	0.780980	691.0
SB	0.850660	0.847405	0.853945	1553.0
SR	0.809165	0.794086	0.824870	907.0
macro avg	0.796583	0.797453	0.796120	4026.0
micro avg	0.806779	0.806779	0.806779	4026.0
weighted avg	0.806353	0.806289	0.806779	4026.0

The result of Model Number:Dataset Number:Featuregroup Number:Rescale of 16 : 1 : 5 : 1

	f1-score	precision	recall	support
AFIB	0.631390	0.616675	0.646927	875.0
GSVT	0.606112	0.612775	0.599634	691.0
SB	0.637836	0.596648	0.685146	1553.0
SR	0.510488	0.612998	0.437624	907.0
macro avg	0.596457	0.609774	0.592333	4026.0

micro avg	0.606400	0.606400	0.606400	4026.0
weighted avg	0.602301	0.607452	0.606400	4026.0

The result of Model Number:Dataset Number:Featuregroup Number:Rescale of 16 : 1 : 6 : 0

	f1-score	precision	recall	support
AFIB	0.822047	0.814128	0.830249	875.0
GSVT	0.843585	0.845998	0.841273	691.0
SB	0.896876	0.894813	0.898954	1553.0
SR	0.871647	0.881903	0.861645	907.0
macro avg	0.858539	0.859211	0.858030	4026.0
micro avg	0.865717	0.865717	0.865717	4026.0
weighted avg	0.865782	0.865990	0.865717	4026.0

The result of Model Number:Dataset Number:Featuregroup Number:Rescale of 16 : 1 : 6 : 1

	f1-score	precision	recall	support
AFIB	0.880184	0.885015	0.875462	875.0
GSVT	0.881415	0.870271	0.892986	691.0
SB	0.926960	0.923241	0.930718	1553.0
SR	0.907061	0.918073	0.896368	907.0
macro avg	0.898905	0.899150	0.898883	4026.0
micro avg	0.904494	0.904494	0.904494	4026.0
weighted avg	0.904494	0.904677	0.904494	4026.0

The result of Model Number:Dataset Number:Featuregroup Number:Rescale of 16 : 1 : 7 : 0

	f1-score	precision	recall	support
AFIB	0.859586	0.860197	0.858993	875.0
GSVT	0.871169	0.872887	0.869533	691.0
SB	0.925328	0.922253	0.928426	1553.0
SR	0.903585	0.906944	0.900281	907.0
macro avg	0.889917	0.890570	0.889308	4026.0
micro avg	0.896887	0.896887	0.896887	4026.0
weighted avg	0.896846	0.896844	0.896887	4026.0

The result of Model Number:Dataset Number:Featuregroup Number:Rescale of 16 : 1 : 7 : 1

	f1-score	precision	recall	support
AFIB	0.901755	0.948342	0.859799	204.0
GSVT	0.945567	0.936325	0.955015	300.0
SB	0.962240	0.954499	0.970113	892.0
SR	0.950354	0.951511	0.949239	681.0
macro avg	0.939979	0.947669	0.933541	2077.0
micro avg	0.950253	0.950253	0.950253	2077.0
weighted avg	0.949994	0.950289	0.950253	2077.0

The result of Model Number:Dataset Number:Featuregroup Number:Rescale of 16 : 1 : 8 : 0

	f1-score	precision	recall	support
AFIB	0.827200	0.839741	0.815048	204.0
GSVT	0.840501	0.833415	0.847752	300.0
SB	0.859144	0.856373	0.861936	892.0
SR	0.849922	0.853079	0.846811	681.0
macro avg	0.844191	0.845652	0.842887	2077.0
micro avg	0.850323	0.850323	0.850323	2077.0
weighted avg	0.850290	0.850343	0.850323	2077.0

The result of Model Number:Dataset Number:Featuregroup Number:Rescale of 16 : 1 : 8 : 1

	f1-score	precision	recall	support
AFIB	0.869911	0.902026	0.840084	204.0
GSVT	0.888504	0.879385	0.897952	300.0
SB	0.912839	0.908582	0.917137	892.0
SR	0.898606	0.899169	0.898073	681.0
macro avg	0.892465	0.897291	0.888311	2077.0
micro avg	0.900547	0.900547	0.900547	2077.0
weighted avg	0.900441	0.900635	0.900547	2077.0

The result of Model Number:Dataset Number:Featuregroup Number:Rescale of 16 : 1 : 9 : 0

	f1-score	precision	recall	support
AFIB	0.817032	0.807395	0.826943	875.0
GSVT	0.830872	0.836130	0.825711	691.0
SB	0.885870	0.884679	0.887068	1553.0
SR	0.864107	0.872293	0.856102	907.0
macro avg	0.849470	0.850124	0.848956	4026.0
micro avg	0.856493	0.856493	0.856493	4026.0
weighted avg	0.856567	0.856759	0.856493	4026.0

The result of Model Number:Dataset Number:Featuregroup Number:Rescale of 16 : 1 : 9 : 1

	f1-score	precision	recall	support
AFIB	0.801020	0.807701	0.794725	204.0
GSVT	0.822181	0.818395	0.826019	300.0
SB	0.834580	0.831615	0.837571	892.0
SR	0.826872	0.830575	0.823244	681.0
macro avg	0.821163	0.822072	0.820390	2077.0
micro avg	0.826997	0.826997	0.826997	2077.0
weighted avg	0.826966	0.827016	0.826997	2077.0

The result of Model Number:Dataset Number:Featuregroup Number:Rescale of 16 : 1 : 10 : 0

	f1-score	precision	recall	support
AFIB	0.926387	0.943412	0.910080	204.0
GSVT	0.941948	0.935779	0.948224	300.0
SB	0.958060	0.953383	0.962786	892.0

SR 0.948158 0.952100 0.944262 681.0
macro avg 0.943638 0.946168 0.941338 2077.0
micro avg 0.949432 0.949432 0.949432 2077.0
weighted avg 0.949375 0.949440 0.949432 2077.0
The result of Model Number:Dataset Number:Featuregroup Number:Rescale of 16 : 1 : 10 : 1

	f1-score	precision	recall	support
AFIB	0.669701	0.659433	0.680406	875.0
GSVT	0.692793	0.699584	0.686270	691.0
SB	0.737317	0.735595	0.739071	1553.0
SR	0.718899	0.728014	0.710024	907.0
macro avg	0.704678	0.705656	0.703943	4026.0
micro avg	0.710715	0.710715	0.710715	4026.0
weighted avg	0.710830	0.711153	0.710715	4026.0

The result of Model Number:Dataset Number:Featuregroup Number:Rescale of 17 : 0 : 0 : 0

	f1-score	precision	recall	support
AFIB	0.847261	0.857168	0.837738	204.0
GSVT	0.764233	0.735056	0.871979	300.0
SB	0.709052	0.707381	0.710738	892.0
SR	0.880404	0.882096	0.878725	681.0
macro avg	0.800238	0.795425	0.824795	2077.0
micro avg	0.801580	0.801580	0.801580	2077.0
weighted avg	0.786779	0.783375	0.801580	2077.0

The result of Model Number:Dataset Number:Featuregroup Number:Rescale of 17 : 0 : 0 : 1

	f1-score	precision	recall	support
AFIB	0.672709	0.661939	0.756934	204.0
GSVT	0.787565	0.778426	0.797208	300.0
SB	0.813935	0.810862	0.817038	892.0
SR	0.638628	0.641930	0.635469	681.0
macro avg	0.728210	0.723290	0.751662	2077.0
micro avg	0.748738	0.748738	0.748738	2077.0
weighted avg	0.738776	0.736161	0.748738	2077.0

The result of Model Number:Dataset Number:Featuregroup Number:Rescale of 17 : 0 : 1 : 0

	f1-score	precision	recall	support
AFIB	0.694686	0.709258	0.680720	204.0
GSVT	0.710802	0.706596	0.715065	300.0
SB	0.728130	0.726425	0.729844	892.0
SR	0.721980	0.721772	0.722194	681.0
macro avg	0.713899	0.716013	0.711956	2077.0
micro avg	0.720376	0.720376	0.720376	2077.0
weighted avg	0.720326	0.720349	0.720376	2077.0

The result of Model Number:Dataset Number:Featuregroup Number:Rescale of 17 : 0 : 1 : 1

	f1-score	precision	recall	support
AFIB	0.897802	0.946641	0.853985	204.0
GSVT	0.942527	0.928272	0.957264	300.0
SB	0.964925	0.958648	0.971299	892.0
SR	0.953020	0.953879	0.952185	681.0
macro avg	0.939568	0.946860	0.933683	2077.0
micro avg	0.951482	0.951482	0.951482	2077.0
weighted avg	0.951194	0.951517	0.951482	2077.0

The result of Model Number:Dataset Number:Featuregroup Number:Rescale of 17 : 0 : 2 : 0

	f1-score	precision	recall	support
AFIB	0.673067	0.687696	0.659232	204.0
GSVT	0.700851	0.696717	0.705054	300.0
SB	0.715908	0.714320	0.717511	892.0
SR	0.708731	0.708331	0.709149	681.0
macro avg	0.699639	0.701766	0.697737	2077.0
micro avg	0.707246	0.707246	0.707246	2077.0
weighted avg	0.707173	0.707199	0.707246	2077.0

The result of Model Number:Dataset Number:Featuregroup Number:Rescale of 17 : 0 : 2 : 1

	f1-score	precision	recall	support
AFIB	0.808673	0.803071	0.814517	875.0
GSVT	0.821149	0.824481	0.817976	691.0
SB	0.890948	0.881684	0.900413	1553.0
SR	0.845963	0.865648	0.827231	907.0
macro avg	0.841683	0.843721	0.840034	4026.0
micro avg	0.851109	0.851109	0.851109	4026.0
weighted avg	0.850952	0.851167	0.851109	4026.0

The result of Model Number:Dataset Number:Featuregroup Number:Rescale of 17 : 0 : 3 : 0

	f1-score	precision	recall	support
AFIB	0.771467	0.787649	0.755957	204.0
GSVT	0.789363	0.784693	0.794098	300.0
SB	0.808607	0.806713	0.810511	892.0
SR	0.801777	0.801547	0.802015	681.0
macro avg	0.792804	0.795150	0.790645	2077.0
micro avg	0.799996	0.799996	0.799996	2077.0
weighted avg	0.799940	0.799966	0.799996	2077.0

The result of Model Number:Dataset Number:Featuregroup Number:Rescale of 17 : 0 : 3 : 1

	f1-score	precision	recall	support
AFIB	0.857298	0.889191	0.827999	204.0

GSVT	0.795541	0.832457	0.761833	300.0
SB	0.833360	0.783096	0.890535	892.0
SR	0.731851	0.782700	0.687292	681.0
macro avg	0.804513	0.821861	0.791915	2077.0
micro avg	0.799164	0.799164	0.799164	2077.0
weighted avg	0.796966	0.800516	0.799164	2077.0

The result of Model Number:Dataset Number:Featuregroup Number:Rescale of 17 : 0 : 4 : 0

	f1-score	precision	recall	support
AFIB	0.799550	0.815043	0.784994	204.0
GSVT	0.830555	0.823610	0.837770	300.0
SB	0.846052	0.844354	0.847759	892.0
SR	0.837985	0.838853	0.837122	681.0
macro avg	0.828535	0.830465	0.826911	2077.0
micro avg	0.836664	0.836664	0.836664	2077.0
weighted avg	0.836601	0.836675	0.836664	2077.0

The result of Model Number:Dataset Number:Featuregroup Number:Rescale of 17 : 0 : 4 : 1

	f1-score	precision	recall	support
AFIB	0.569506	0.626964	0.522029	204.0
GSVT	0.671614	0.662344	0.681255	300.0
SB	0.713124	0.710745	0.715523	892.0
SR	0.694862	0.685249	0.704758	681.0
macro avg	0.662277	0.671326	0.655891	2077.0
micro avg	0.688039	0.688039	0.688039	2077.0
weighted avg	0.687035	0.687166	0.688039	2077.0

The result of Model Number:Dataset Number:Featuregroup Number:Rescale of 17 : 0 : 5 : 0

	f1-score	precision	recall	support
AFIB	0.870248	0.858160	0.882765	875.0
GSVT	0.901346	0.913371	0.889752	691.0
SB	0.957522	0.955561	0.959496	1553.0
SR	0.932601	0.939807	0.925506	907.0
macro avg	0.915429	0.916725	0.914380	4026.0
micro avg	0.923191	0.923191	0.923191	4026.0
weighted avg	0.923298	0.923602	0.923191	4026.0

The result of Model Number:Dataset Number:Featuregroup Number:Rescale of 17 : 0 : 5 : 1

	f1-score	precision	recall	support
AFIB	0.677827	0.675632	0.680057	875.0
GSVT	0.685687	0.687817	0.683637	691.0
SB	0.723511	0.721936	0.725095	1553.0
SR	0.710169	0.713519	0.706876	907.0
macro avg	0.699298	0.699726	0.698916	4026.0

micro avg 0.704087 0.704087 0.704087 4026.0
weighted avg 0.704084 0.704120 0.704087 4026.0
The result of Model Number:Dataset Number:Featuregroup Number:Rescale of 17 : 0 : 6 : 0

	f1-score	precision	recall	support
AFIB	0.796361	0.789318	0.803564	875.0
GSVT	0.724030	0.701168	0.805498	691.0
SB	0.652752	0.626732	0.693443	1553.0
SR	0.672756	0.679717	0.665980	907.0
macro avg	0.711475	0.699234	0.742121	4026.0
micro avg	0.730422	0.730422	0.730422	4026.0
weighted avg	0.700704	0.686781	0.730422	4026.0

The result of Model Number:Dataset Number:Featuregroup Number:Rescale of 17 : 0 : 6 : 1

	f1-score	precision	recall	support
AFIB	0.913761	0.926118	0.902113	204.0
GSVT	0.948469	0.946930	0.950047	300.0
SB	0.969066	0.967776	0.970370	892.0
SR	0.959161	0.957933	0.960427	681.0
macro avg	0.947615	0.949689	0.945739	2077.0
micro avg	0.957470	0.957470	0.957470	2077.0
weighted avg	0.957412	0.957446	0.957470	2077.0

The result of Model Number:Dataset Number:Featuregroup Number:Rescale of 17 : 0 : 7 : 0

	f1-score	precision	recall	support
AFIB	0.878119	0.884035	0.872411	875.0
GSVT	0.893790	0.881376	0.906723	691.0
SB	0.952431	0.945909	0.959051	1553.0
SR	0.913834	0.929619	0.898626	907.0
macro avg	0.909543	0.910235	0.909203	4026.0
micro avg	0.917627	0.917627	0.917627	4026.0
weighted avg	0.917520	0.917715	0.917627	4026.0

The result of Model Number:Dataset Number:Featuregroup Number:Rescale of 17 : 0 : 7 : 1

	f1-score	precision	recall	support
AFIB	0.626473	0.611872	0.641889	875.0
GSVT	0.601392	0.608002	0.594964	691.0
SB	0.632869	0.592002	0.679811	1553.0
SR	0.506513	0.608224	0.434216	907.0
macro avg	0.591811	0.605025	0.587720	4026.0
micro avg	0.601677	0.601677	0.601677	4026.0
weighted avg	0.597610	0.602721	0.601677	4026.0

The result of Model Number:Dataset Number:Featuregroup Number:Rescale of 17 : 0 : 8 : 0

	f1-score	precision	recall	support
AFIB	0.756039	0.762802	0.749488	875.0
GSVT	0.770478	0.755828	0.785856	691.0
SB	0.817798	0.810714	0.825011	1553.0
SR	0.784209	0.802240	0.767006	907.0
macro avg	0.782131	0.782896	0.781840	4026.0
micro avg	0.788809	0.788809	0.788809	4026.0
weighted avg	0.788687	0.788971	0.788809	4026.0

The result of Model Number:Dataset Number:Featuregroup Number:Rescale of 17 : 0 : 8 : 1

	f1-score	precision	recall	support
AFIB	0.871973	0.880498	0.863817	875.0
GSVT	0.865313	0.858748	0.872264	691.0
SB	0.915600	0.906179	0.925299	1553.0
SR	0.886537	0.900795	0.872974	907.0
macro avg	0.884856	0.886555	0.883588	4026.0
micro avg	0.891046	0.891046	0.891046	4026.0
weighted avg	0.890940	0.891244	0.891046	4026.0

The result of Model Number:Dataset Number:Featuregroup Number:Rescale of 17 : 0 : 9 : 0

	f1-score	precision	recall	support
AFIB	0.679370	0.675567	0.683249	875.0
GSVT	0.684902	0.688943	0.680951	691.0
SB	0.721488	0.719230	0.723763	1553.0
SR	0.707205	0.711883	0.702604	907.0
macro avg	0.698241	0.698906	0.697642	4026.0
micro avg	0.702843	0.702843	0.702843	4026.0
weighted avg	0.702837	0.702887	0.702843	4026.0

The result of Model Number:Dataset Number:Featuregroup Number:Rescale of 17 : 0 : 9 : 1

	f1-score	precision	recall	support
AFIB	0.705074	0.695631	0.714821	875.0
GSVT	0.720838	0.726464	0.715311	691.0
SB	0.765329	0.762935	0.767743	1553.0
SR	0.746821	0.756722	0.737190	907.0
macro avg	0.734516	0.735438	0.733766	4026.0
micro avg	0.740359	0.740359	0.740359	4026.0
weighted avg	0.740428	0.740648	0.740359	4026.0

The result of Model Number:Dataset Number:Featuregroup Number:Rescale of 17 : 0 : 10 : 0

	f1-score	precision	recall	support
AFIB	0.643052	0.658196	0.628645	875.0
GSVT	0.677872	0.679584	0.676214	691.0
SB	0.737199	0.734142	0.740283	1553.0

SR 0.701449 0.690402 0.712889 907.0
macro avg 0.689893 0.690581 0.689508 4026.0
micro avg 0.698852 0.698852 0.698852 4026.0
weighted avg 0.698501 0.698418 0.698852 4026.0
The result of Model Number:Dataset Number:Featuregroup Number:Rescale of 17 : 0 : 10 : 1
f1-score precision recall support
AFIB 0.706606 0.719575 0.694438 204.0
GSVT 0.733430 0.727533 0.739558 300.0
SB 0.747036 0.745702 0.748376 892.0
SR 0.739639 0.740297 0.738986 681.0
macro avg 0.731678 0.733277 0.730339 2077.0
micro avg 0.738726 0.738726 0.738726 2077.0
weighted avg 0.738675 0.738740 0.738726 2077.0
The result of Model Number:Dataset Number:Featuregroup Number:Rescale of 17 : 1 : 0 : 0
f1-score precision recall support
AFIB 0.596221 0.591727 0.676703 204.0
GSVT 0.722801 0.718521 0.727139 300.0
SB 0.591150 0.589917 0.592393 892.0
SR 0.731115 0.729944 0.732297 681.0
macro avg 0.660322 0.657527 0.682133 2077.0
micro avg 0.666008 0.666008 0.666008 2077.0
weighted avg 0.656555 0.654582 0.666008 2077.0
The result of Model Number:Dataset Number:Featuregroup Number:Rescale of 17 : 1 : 0 : 1
f1-score precision recall support
AFIB 0.857781 0.846749 0.869165 875.0
GSVT 0.875242 0.882488 0.868150 691.0
SB 0.935810 0.931645 0.940023 1553.0
SR 0.909179 0.922626 0.896140 907.0
macro avg 0.894503 0.895877 0.893370 4026.0
micro avg 0.902401 0.902401 0.902401 4026.0
weighted avg 0.902456 0.902725 0.902401 4026.0
The result of Model Number:Dataset Number:Featuregroup Number:Rescale of 17 : 1 : 1 : 0
f1-score precision recall support
AFIB 0.710557 0.699858 0.727504 875.0
GSVT 0.678453 0.676479 0.684241 691.0
SB 0.724501 0.675119 0.787702 1553.0
SR 0.536683 0.717386 0.444706 907.0
macro avg 0.662548 0.692210 0.661038 4026.0
micro avg 0.679589 0.679589 0.679589 4026.0
weighted avg 0.671254 0.690251 0.679589 4026.0

The result of Model Number:Dataset Number:Featuregroup Number:Rescale of 17 : 1 : 1 : 1

	f1-score	precision	recall	support
AFIB	0.670451	0.674275	0.666782	204.0
GSVT	0.684246	0.679311	0.689261	300.0
SB	0.698065	0.695516	0.700646	892.0
SR	0.690641	0.695176	0.686183	681.0
macro avg	0.685851	0.686069	0.685718	2077.0
micro avg	0.690933	0.690933	0.690933	2077.0
weighted avg	0.690923	0.690977	0.690933	2077.0

The result of Model Number:Dataset Number:Featuregroup Number:Rescale of 17 : 1 : 2 : 0

	f1-score	precision	recall	support
AFIB	0.905455	0.919338	0.892134	204.0
GSVT	0.926925	0.919060	0.934964	300.0
SB	0.943049	0.940005	0.946115	892.0
SR	0.933358	0.936823	0.929931	681.0
macro avg	0.927197	0.928807	0.925786	2077.0
micro avg	0.933896	0.933896	0.933896	2077.0
weighted avg	0.933850	0.933907	0.933896	2077.0

The result of Model Number:Dataset Number:Featuregroup Number:Rescale of 17 : 1 : 2 : 1

	f1-score	precision	recall	support
AFIB	0.882112	0.891515	0.873050	204.0
GSVT	0.898335	0.893407	0.903417	300.0
SB	0.919071	0.917539	0.920614	892.0
SR	0.911410	0.912895	0.909942	681.0
macro avg	0.902732	0.903839	0.901756	2077.0
micro avg	0.909959	0.909959	0.909959	2077.0
weighted avg	0.909934	0.909975	0.909959	2077.0

The result of Model Number:Dataset Number:Featuregroup Number:Rescale of 17 : 1 : 3 : 0

	f1-score	precision	recall	support
AFIB	0.747296	0.750710	0.743955	875.0
GSVT	0.759755	0.753329	0.766346	691.0
SB	0.803076	0.794875	0.811452	1553.0
SR	0.761629	0.777685	0.746263	907.0
macro avg	0.767939	0.769150	0.767004	4026.0
micro avg	0.774355	0.774355	0.774355	4026.0
weighted avg	0.774180	0.774273	0.774355	4026.0

The result of Model Number:Dataset Number:Featuregroup Number:Rescale of 17 : 1 : 3 : 1

	f1-score	precision	recall	support
AFIB	0.735688	0.735453	0.735962	875.0

GSVT	0.746652	0.737055	0.756591	691.0
SB	0.789764	0.788046	0.791493	1553.0
SR	0.767913	0.779169	0.756989	907.0
macro avg	0.760004	0.759931	0.760259	4026.0
micro avg	0.765660	0.765660	0.765660	4026.0
weighted avg	0.765689	0.765864	0.765660	4026.0

The result of Model Number:Dataset Number:Featuregroup Number:Rescale of 17 : 1 : 4 : 0

	f1-score	precision	recall	support
AFIB	0.941078	0.938470	0.943771	875.0
GSVT	0.948526	0.953050	0.944139	691.0
SB	0.992940	0.989767	0.996137	1553.0
SR	0.977065	0.981785	0.972437	907.0
macro avg	0.964902	0.965768	0.964121	4026.0
micro avg	0.970492	0.970492	0.970492	4026.0
weighted avg	0.970469	0.970518	0.970492	4026.0

The result of Model Number:Dataset Number:Featuregroup Number:Rescale of 17 : 1 : 4 : 1

	f1-score	precision	recall	support
AFIB	0.816958	0.903341	0.746078	204.0
GSVT	0.940017	0.929138	0.951333	300.0
SB	0.993860	0.989773	0.997982	892.0
SR	0.972516	0.958111	0.987372	681.0
macro avg	0.930838	0.945091	0.920691	2077.0
micro avg	0.963024	0.963024	0.963024	2077.0
weighted avg	0.961710	0.962145	0.963024	2077.0

The result of Model Number:Dataset Number:Featuregroup Number:Rescale of 17 : 1 : 5 : 0

	f1-score	precision	recall	support
AFIB	0.703924	0.697630	0.710363	875.0
GSVT	0.715226	0.718056	0.712444	691.0
SB	0.762584	0.761169	0.764007	1553.0
SR	0.743260	0.750131	0.736541	907.0
macro avg	0.731248	0.731746	0.730839	4026.0
micro avg	0.737311	0.737311	0.737311	4026.0
weighted avg	0.737353	0.737473	0.737311	4026.0

The result of Model Number:Dataset Number:Featuregroup Number:Rescale of 17 : 1 : 5 : 1

	f1-score	precision	recall	support
AFIB	0.697635	0.704073	0.691351	875.0
GSVT	0.713925	0.711998	0.715888	691.0
SB	0.760340	0.753193	0.767632	1553.0
SR	0.739333	0.746751	0.732075	907.0
macro avg	0.727808	0.729004	0.726736	4026.0

micro avg 0.734162 0.734162 0.734162 4026.0
weighted avg 0.734013 0.733996 0.734162 4026.0
The result of Model Number:Dataset Number:Featuregroup Number:Rescale of 17 : 1 : 6 : 0

	f1-score	precision	recall	support
AFIB	0.820548	0.832476	0.809329	204.0
GSVT	0.838645	0.827299	0.850374	300.0
SB	0.851267	0.842395	0.860333	892.0
SR	0.834395	0.848202	0.821044	681.0
macro avg	0.836214	0.837593	0.835270	2077.0
micro avg	0.841003	0.841003	0.841003	2077.0
weighted avg	0.840895	0.841144	0.841003	2077.0

The result of Model Number:Dataset Number:Featuregroup Number:Rescale of 17 : 1 : 6 : 1

	f1-score	precision	recall	support
AFIB	0.905981	0.937664	0.876787	204.0
GSVT	0.944242	0.938486	0.950198	300.0
SB	0.963631	0.959672	0.967627	892.0
SR	0.954723	0.953331	0.956125	681.0
macro avg	0.942144	0.947288	0.937684	2077.0
micro avg	0.952416	0.952416	0.952416	2077.0
weighted avg	0.952247	0.952371	0.952416	2077.0

The result of Model Number:Dataset Number:Featuregroup Number:Rescale of 17 : 1 : 7 : 0

	f1-score	precision	recall	support
AFIB	0.693349	0.699670	0.687232	204.0
GSVT	0.711443	0.709114	0.713810	300.0
SB	0.724449	0.723966	0.724935	892.0
SR	0.718078	0.717871	0.718294	681.0
macro avg	0.711830	0.712655	0.711068	2077.0
micro avg	0.717448	0.717448	0.717448	2077.0
weighted avg	0.717427	0.717436	0.717448	2077.0

The result of Model Number:Dataset Number:Featuregroup Number:Rescale of 17 : 1 : 7 : 1

	f1-score	precision	recall	support
AFIB	0.663974	0.679143	0.649637	204.0
GSVT	0.694138	0.689817	0.698531	300.0
SB	0.709129	0.706930	0.711352	892.0
SR	0.701008	0.701332	0.700703	681.0
macro avg	0.692062	0.694306	0.690056	2077.0
micro avg	0.699947	0.699947	0.699947	2077.0
weighted avg	0.699866	0.699894	0.699947	2077.0

The result of Model Number:Dataset Number:Featuregroup Number:Rescale of 17 : 1 : 8 : 0

	f1-score	precision	recall	support
AFIB	0.766741	0.803912	0.732941	204.0
GSVT	0.798674	0.788238	0.809552	300.0
SB	0.820185	0.813948	0.826530	892.0
SR	0.797348	0.799731	0.795015	681.0
macro avg	0.795737	0.801457	0.791010	2077.0
micro avg	0.804553	0.804553	0.804553	2077.0
weighted avg	0.804341	0.804587	0.804553	2077.0

The result of Model Number:Dataset Number:Featuregroup Number:Rescale of 17 : 1 : 8 : 1

	f1-score	precision	recall	support
AFIB	0.868830	0.936547	0.810648	204.0
GSVT	0.895325	0.910481	0.880703	300.0
SB	0.948876	0.940143	0.957802	892.0
SR	0.911109	0.897897	0.924758	681.0
macro avg	0.906035	0.921267	0.893478	2077.0
micro avg	0.921378	0.921378	0.921378	2077.0
weighted avg	0.920896	0.921654	0.921378	2077.0

The result of Model Number:Dataset Number:Featuregroup Number:Rescale of 17 : 1 : 9 : 0

	f1-score	precision	recall	support
AFIB	0.810155	0.865069	0.762336	204.0
GSVT	0.763358	0.786007	0.742265	300.0
SB	0.797900	0.759597	0.840725	892.0
SR	0.707989	0.740132	0.679293	681.0
macro avg	0.769850	0.787701	0.756155	2077.0
micro avg	0.765875	0.765875	0.765875	2077.0
weighted avg	0.764635	0.767389	0.765875	2077.0

The result of Model Number:Dataset Number:Featuregroup Number:Rescale of 17 : 1 : 9 : 1

	f1-score	precision	recall	support
AFIB	0.787134	0.793113	0.781313	204.0
GSVT	0.803249	0.795404	0.811268	300.0
SB	0.821986	0.819885	0.824105	892.0
SR	0.813267	0.817861	0.808734	681.0
macro avg	0.806409	0.806566	0.806355	2077.0
micro avg	0.813008	0.813008	0.813008	2077.0
weighted avg	0.812998	0.813056	0.813008	2077.0

The result of Model Number:Dataset Number:Featuregroup Number:Rescale of 17 : 1 : 10 : 0

	f1-score	precision	recall	support
AFIB	0.793031	0.784269	0.802007	875.0
GSVT	0.807319	0.812290	0.802423	691.0
SB	0.859237	0.857807	0.860676	1553.0

SR 0.838315 0.846154 0.830641 907.0
macro avg 0.824476 0.825130 0.823937 4026.0
micro avg 0.831160 0.831160 0.831160 4026.0
weighted avg 0.831224 0.831387 0.831160 4026.0
The result of Model Number:Dataset Number:Featuregroup Number:Rescale of 17 : 1 : 10 : 1

	f1-score	precision	recall	support
AFIB	0.872138	0.890431	0.854604	204.0
GSVT	0.892370	0.887090	0.897722	300.0
SB	0.914125	0.911984	0.916277	892.0
SR	0.906404	0.906143	0.906673	681.0
macro avg	0.896259	0.898912	0.893819	2077.0
micro avg	0.904390	0.904390	0.904390	2077.0
weighted avg	0.904327	0.904356	0.904390	2077.0

The result of Model Number:Dataset Number:Featuregroup Number:Rescale of 18 : 0 : 0 : 0

	f1-score	precision	recall	support
AFIB	0.663369	0.662628	0.664132	875.0
GSVT	0.675504	0.667055	0.684189	691.0
SB	0.709613	0.703908	0.715416	1553.0
SR	0.677953	0.695741	0.661063	907.0
macro avg	0.681610	0.682333	0.681200	4026.0
micro avg	0.686666	0.686666	0.686666	4026.0
weighted avg	0.686576	0.686771	0.686666	4026.0

The result of Model Number:Dataset Number:Featuregroup Number:Rescale of 18 : 0 : 0 : 1

	f1-score	precision	recall	support
AFIB	0.702692	0.722467	0.684114	204.0
GSVT	0.720858	0.715799	0.726126	300.0
SB	0.740682	0.737718	0.743671	892.0
SR	0.730837	0.731294	0.730402	681.0
macro avg	0.723767	0.726819	0.721078	2077.0
micro avg	0.730937	0.730937	0.730937	2077.0
weighted avg	0.730859	0.730948	0.730937	2077.0

The result of Model Number:Dataset Number:Featuregroup Number:Rescale of 18 : 0 : 1 : 0

	f1-score	precision	recall	support
AFIB	0.897082	0.913590	0.881617	204.0
GSVT	0.930180	0.922389	0.938264	300.0
SB	0.947646	0.946058	0.949239	892.0
SR	0.939279	0.940113	0.938451	681.0
macro avg	0.928547	0.930538	0.926893	2077.0
micro avg	0.937475	0.937475	0.937475	2077.0
weighted avg	0.937414	0.937501	0.937475	2077.0

The result of Model Number:Dataset Number:Featuregroup Number:Rescale of 18 : 0 : 1 : 1

	f1-score	precision	recall	support
AFIB	0.796312	0.797942	0.794775	204.0
GSVT	0.813827	0.810663	0.817072	300.0
SB	0.831893	0.830783	0.833011	892.0
SR	0.825783	0.828224	0.823359	681.0
macro avg	0.816954	0.816903	0.817054	2077.0
micro avg	0.823788	0.823788	0.823788	2077.0
weighted avg	0.823785	0.823812	0.823788	2077.0

The result of Model Number:Dataset Number:Featuregroup Number:Rescale of 18 : 0 : 2 : 0

	f1-score	precision	recall	support
AFIB	0.694773	0.711276	0.679081	204.0
GSVT	0.717594	0.710866	0.724526	300.0
SB	0.733005	0.732185	0.733827	892.0
SR	0.725930	0.725200	0.726673	681.0
macro avg	0.717825	0.719882	0.716027	2077.0
micro avg	0.724761	0.724761	0.724761	2077.0
weighted avg	0.724704	0.724762	0.724761	2077.0

The result of Model Number:Dataset Number:Featuregroup Number:Rescale of 18 : 0 : 2 : 1

	f1-score	precision	recall	support
AFIB	0.748354	0.757543	0.739502	204.0
GSVT	0.763427	0.760167	0.766734	300.0
SB	0.772669	0.770349	0.775006	892.0
SR	0.766165	0.767989	0.764375	681.0
macro avg	0.762654	0.764012	0.761404	2077.0
micro avg	0.766838	0.766838	0.766838	2077.0
weighted avg	0.766813	0.766847	0.766838	2077.0

The result of Model Number:Dataset Number:Featuregroup Number:Rescale of 18 : 0 : 3 : 0

	f1-score	precision	recall	support
AFIB	0.852780	0.852556	0.853085	875.0
GSVT	0.870594	0.867302	0.874004	691.0
SB	0.926879	0.922694	0.931115	1553.0
SR	0.895641	0.905874	0.885671	907.0
macro avg	0.886473	0.887107	0.885969	4026.0
micro avg	0.894116	0.894116	0.894116	4026.0
weighted avg	0.894076	0.894154	0.894116	4026.0

The result of Model Number:Dataset Number:Featuregroup Number:Rescale of 18 : 0 : 3 : 1

	f1-score	precision	recall	support
AFIB	0.879192	0.939223	0.826750	204.0

GSVT	0.929394	0.910455	0.949219	300.0
SB	0.952446	0.944096	0.960981	892.0
SR	0.928952	0.932046	0.925962	681.0
macro avg	0.922496	0.931455	0.915728	2077.0
micro avg	0.934616	0.934616	0.934616	2077.0
weighted avg	0.934218	0.934807	0.934616	2077.0

The result of Model Number:Dataset Number:Featuregroup Number:Rescale of 18 : 0 : 4 : 0

	f1-score	precision	recall	support
AFIB	0.686108	0.677284	0.695214	875.0
GSVT	0.700075	0.705870	0.694402	691.0
SB	0.748521	0.745189	0.751891	1553.0
SR	0.727219	0.737975	0.716790	907.0
macro avg	0.715481	0.716580	0.714574	4026.0
micro avg	0.721798	0.721798	0.721798	4026.0
weighted avg	0.721842	0.722057	0.721798	4026.0

The result of Model Number:Dataset Number:Featuregroup Number:Rescale of 18 : 0 : 4 : 1

	f1-score	precision	recall	support
AFIB	0.698270	0.717519	0.680166	204.0
GSVT	0.716853	0.703583	0.730701	300.0
SB	0.728479	0.721668	0.735420	892.0
SR	0.710168	0.719816	0.700787	681.0
macro avg	0.713442	0.715646	0.711769	2077.0
micro avg	0.717956	0.717956	0.717956	2077.0
weighted avg	0.717829	0.718041	0.717956	2077.0

The result of Model Number:Dataset Number:Featuregroup Number:Rescale of 18 : 0 : 5 : 0

	f1-score	precision	recall	support
AFIB	0.691133	0.682068	0.700475	875.0
GSVT	0.701714	0.709514	0.694097	691.0
SB	0.744259	0.742502	0.746028	1553.0
SR	0.725625	0.732156	0.719220	907.0
macro avg	0.715683	0.716560	0.714955	4026.0
micro avg	0.721175	0.721175	0.721175	4026.0
weighted avg	0.721212	0.721375	0.721175	4026.0

The result of Model Number:Dataset Number:Featuregroup Number:Rescale of 18 : 0 : 5 : 1

	f1-score	precision	recall	support
AFIB	0.805002	0.859566	0.757486	204.0
GSVT	0.758502	0.781007	0.737543	300.0
SB	0.792824	0.754765	0.835377	892.0
SR	0.703485	0.735424	0.674972	681.0
macro avg	0.764953	0.782690	0.751345	2077.0

micro avg 0.761003 0.761003 0.761003 2077.0
weighted avg 0.759770 0.762507 0.761003 2077.0
The result of Model Number:Dataset Number:Featuregroup Number:Rescale of 18 : 0 : 6 : 0

	f1-score	precision	recall	support
AFIB	0.725900	0.740462	0.712044	204.0
GSVT	0.742723	0.735003	0.750647	300.0
SB	0.756459	0.753517	0.759427	892.0
SR	0.748510	0.751633	0.745430	681.0
macro avg	0.743398	0.745154	0.741887	2077.0
micro avg	0.748916	0.748916	0.748916	2077.0
weighted avg	0.748867	0.748943	0.748916	2077.0

The result of Model Number:Dataset Number:Featuregroup Number:Rescale of 18 : 0 : 6 : 1

	f1-score	precision	recall	support
AFIB	0.669355	0.677182	0.661832	204.0
GSVT	0.603762	0.580711	0.688884	300.0
SB	0.560168	0.558848	0.561499	892.0
SR	0.695539	0.696876	0.694213	681.0
macro avg	0.632206	0.628404	0.651607	2077.0
micro avg	0.633267	0.633267	0.633267	2077.0
weighted avg	0.621574	0.618885	0.633267	2077.0

The result of Model Number:Dataset Number:Featuregroup Number:Rescale of 18 : 0 : 7 : 0

	f1-score	precision	recall	support
AFIB	0.964130	0.974231	0.954444	204.0
GSVT	0.9791332	0.9771854	0.9801256	300.0
SB	0.9963326	0.9941962	0.9992955	892.0
SR	0.9891665	0.9904365	0.9891845	681.0
macro avg	0.9822256	0.9843124	0.9804356	2077.0
micro avg	0.9883564	0.9884429	0.9883211	2077.0
weighted avg	0.9880449	0.9882568	0.9883568	2077.0

The result of Model Number:Dataset Number:Featuregroup Number:Rescale of 18 : 0 : 7 : 1

	f1-score	precision	recall	support
AFIB	0.9632589	0.9742568	0.9535896	204.0
GSVT	0.9783333	0.9765455	0.98125488	300.0
SB	0.99655245	0.99468745	0.9993566	892.0
SR	0.9882335	0.99125466	0.989854333	681.0
macro avg	0.98256333	0.98395688	0.98045211	2077.0
micro avg	0.9889565	0.9885657	0.9884911	2077.0
weighted avg	0.9884256	0.9885877	0.988324561	2077.0

The result of Model Number:Dataset Number:Featuregroup Number:Rescale of 18 : 0 : 8 : 0

	f1-score	precision	recall	support
AFIB	0.179182	0.181064	0.177339	175.0
GSVT	0.179471	0.177564	0.181419	138.2
SB	0.189014	0.188290	0.189745	310.6
SR	0.184640	0.185565	0.183724	181.4
macro avg	0.183077	0.183121	0.183057	805.2
micro avg	0.184263	0.184263	0.184263	805.2
weighted avg	0.184254	0.184265	0.184263	805.2

The result of Model Number:Dataset Number:Featuregroup Number:Rescale of 18 : 0 : 8 : 1

	f1-score	precision	recall	support
AFIB	0.869467	0.907625	0.834607	204.0
GSVT	0.809417	0.815245	0.804005	300.0
SB	0.839429	0.797276	0.886293	892.0
SR	0.744705	0.793874	0.701429	681.0
macro avg	0.815755	0.828505	0.806583	2077.0
micro avg	0.808718	0.808718	0.808718	2077.0
weighted avg	0.806987	0.809594	0.808718	2077.0

The result of Model Number:Dataset Number:Featuregroup Number:Rescale of 18 : 0 : 9 : 0

	f1-score	precision	recall	support
AFIB	0.683142	0.673450	0.693133	875.0
GSVT	0.697197	0.703992	0.690537	691.0
SB	0.741953	0.739535	0.744389	1553.0
SR	0.724626	0.733888	0.715604	907.0
macro avg	0.711730	0.712716	0.710916	4026.0
micro avg	0.717521	0.717521	0.717521	4026.0
weighted avg	0.717586	0.717800	0.717521	4026.0

The result of Model Number:Dataset Number:Featuregroup Number:Rescale of 18 : 0 : 9 : 1

	f1-score	precision	recall	support
AFIB	0.757860	0.761725	0.754057	875.0
GSVT	0.768318	0.764321	0.772424	691.0
SB	0.805317	0.804598	0.806044	1553.0
SR	0.790489	0.791133	0.789878	907.0
macro avg	0.780496	0.780444	0.780601	4026.0
micro avg	0.785333	0.785333	0.785333	4026.0
weighted avg	0.785312	0.785334	0.785333	4026.0

The result of Model Number:Dataset Number:Featuregroup Number:Rescale of 18 : 0 : 10 : 0

	f1-score	precision	recall	support
AFIB	0.649464	0.649140	0.649817	875.0
GSVT	0.657543	0.661942	0.653254	691.0
SB	0.697085	0.695484	0.698699	1553.0

SR 0.676895 0.676734 0.677173 907.0
macro avg 0.670247 0.670825 0.669736 4026.0
micro avg 0.675426 0.675426 0.675426 4026.0
weighted avg 0.675400 0.675431 0.675426 4026.0
The result of Model Number:Dataset Number:Featuregroup Number:Rescale of 18 : 0 : 10 : 1

	f1-score	precision	recall	support
AFIB	0.870227	0.870382	0.870110	875.0
GSVT	0.879434	0.882405	0.876538	691.0
SB	0.923800	0.920615	0.927012	1553.0
SR	0.906258	0.909314	0.903255	907.0
macro avg	0.894930	0.895679	0.894229	4026.0
micro avg	0.900630	0.900630	0.900630	4026.0
weighted avg	0.900590	0.900593	0.900630	4026.0

The result of Model Number:Dataset Number:Featuregroup Number:Rescale of 18 : 1 : 0 : 0

	f1-score	precision	recall	support
AFIB	0.652314	0.677663	0.628841	875.0
GSVT	0.701259	0.696080	0.706608	691.0
SB	0.770630	0.766660	0.774647	1553.0
SR	0.725233	0.711403	0.739689	907.0
macro avg	0.712359	0.712951	0.712446	4026.0
micro avg	0.723405	0.723405	0.723405	4026.0
weighted avg	0.722782	0.722755	0.723405	4026.0

The result of Model Number:Dataset Number:Featuregroup Number:Rescale of 18 : 1 : 0 : 1

	f1-score	precision	recall	support
AFIB	0.754940	0.748934	0.761114	875.0
GSVT	0.725006	0.711221	0.739396	691.0
SB	0.760920	0.702395	0.830097	1553.0
SR	0.582653	0.745008	0.478507	907.0
macro avg	0.705880	0.726889	0.702278	4026.0
micro avg	0.720329	0.720329	0.720329	4026.0
weighted avg	0.713295	0.723625	0.720329	4026.0

The result of Model Number:Dataset Number:Featuregroup Number:Rescale of 18 : 1 : 1 : 0

	f1-score	precision	recall	support
AFIB	0.798478	0.800461	0.796558	875.0
GSVT	0.812228	0.814669	0.809906	691.0
SB	0.861628	0.855780	0.867563	1553.0
SR	0.845232	0.851507	0.839082	907.0
macro avg	0.829392	0.830604	0.828277	4026.0
micro avg	0.835819	0.835819	0.835819	4026.0
weighted avg	0.835731	0.835738	0.835819	4026.0

The result of Model Number:Dataset Number:Featuregroup Number:Rescale of 18 : 1 : 1 : 1

	f1-score	precision	recall	support
AFIB	0.785267	0.809541	0.763092	204.0
GSVT	0.733037	0.748973	0.718306	300.0
SB	0.773169	0.738854	0.810864	892.0
SR	0.696154	0.731926	0.663813	681.0
macro avg	0.746907	0.757324	0.739019	2077.0
micro avg	0.744589	0.744589	0.744589	2077.0
weighted avg	0.743309	0.744987	0.744589	2077.0

The result of Model Number:Dataset Number:Featuregroup Number:Rescale of 18 : 1 : 2 : 0

	f1-score	precision	recall	support
AFIB	0.878616	0.897712	0.860556	204.0
GSVT	0.914885	0.909488	0.920371	300.0
SB	0.934541	0.932468	0.936633	892.0
SR	0.925172	0.924650	0.925718	681.0
macro avg	0.913303	0.916080	0.910820	2077.0
micro avg	0.923233	0.923233	0.923233	2077.0
weighted avg	0.923137	0.923172	0.923233	2077.0

The result of Model Number:Dataset Number:Featuregroup Number:Rescale of 18 : 1 : 2 : 1

	f1-score	precision	recall	support
AFIB	0.732700	0.738508	0.727001	875.0
GSVT	0.741989	0.738718	0.745314	691.0
SB	0.789018	0.785898	0.792167	1553.0
SR	0.769629	0.771803	0.767504	907.0
macro avg	0.758334	0.758732	0.757997	4026.0
micro avg	0.764406	0.764406	0.764406	4026.0
weighted avg	0.764338	0.764325	0.764406	4026.0

The result of Model Number:Dataset Number:Featuregroup Number:Rescale of 18 : 1 : 3 : 0

	f1-score	precision	recall	support
AFIB	0.718535	0.765352	0.677433	204.0
GSVT	0.750012	0.731577	0.769503	300.0
SB	0.772008	0.762091	0.782214	892.0
SR	0.751956	0.760935	0.743237	681.0
macro avg	0.748128	0.754989	0.743097	2077.0
micro avg	0.757307	0.757307	0.757307	2077.0
weighted avg	0.757005	0.757625	0.757307	2077.0

The result of Model Number:Dataset Number:Featuregroup Number:Rescale of 18 : 1 : 3 : 1

	f1-score	precision	recall	support
AFIB	0.897665	0.892295	0.903222	875.0

GSVT 0.905016 0.909902 0.900313 691.0
SB 0.959182 0.956378 0.962017 1553.0
SR 0.937508 0.944242 0.930887 907.0
macro avg 0.924843 0.925704 0.924110 4026.0
micro avg 0.931635 0.931635 0.931635 4026.0
weighted avg 0.931632 0.931739 0.931635 4026.0
The result of Model Number:Dataset Number:Featuregroup Number:Rescale of 18 : 1 : 4 : 0

	f1-score	precision	recall	support
AFIB	0.699644	0.766985	0.643413	204.0
GSVT	0.813689	0.805328	0.822377	300.0
SB	0.862572	0.859217	0.865956	892.0
SR	0.842587	0.830935	0.854584	681.0
macro avg	0.804623	0.815616	0.796582	2077.0
micro avg	0.834075	0.834075	0.834075	2077.0
weighted avg	0.832956	0.833102	0.834075	2077.0

The result of Model Number:Dataset Number:Featuregroup Number:Rescale of 18 : 1 : 4 : 1

	f1-score	precision	recall	support
AFIB	0.781827	0.780891	0.782897	875.0
GSVT	0.797067	0.799809	0.794426	691.0
SB	0.844213	0.839012	0.849485	1553.0
SR	0.827033	0.835110	0.819202	907.0
macro avg	0.812535	0.813706	0.811502	4026.0
micro avg	0.818740	0.818740	0.818740	4026.0
weighted avg	0.818692	0.818773	0.818740	4026.0

The result of Model Number:Dataset Number:Featuregroup Number:Rescale of 18 : 1 : 5 : 0

	f1-score	precision	recall	support
AFIB	0.811582	0.809532	0.813769	875.0
GSVT	0.827416	0.822867	0.832060	691.0
SB	0.866370	0.861146	0.871668	1553.0
SR	0.840825	0.856001	0.826194	907.0
macro avg	0.836548	0.837387	0.835923	4026.0
micro avg	0.842042	0.842042	0.842042	4026.0
weighted avg	0.842022	0.842199	0.842042	4026.0

The result of Model Number:Dataset Number:Featuregroup Number:Rescale of 18 : 1 : 5 : 1

	f1-score	precision	recall	support
AFIB	0.853762	0.896420	0.815410	204.0
GSVT	0.879501	0.863202	0.896734	300.0
SB	0.899629	0.890560	0.908902	892.0
SR	0.874678	0.882491	0.867082	681.0
macro avg	0.876893	0.883168	0.872032	2077.0

micro avg 0.884250 0.884250 0.884250 2077.0
weighted avg 0.884036 0.884539 0.884250 2077.0
The result of Model Number:Dataset Number:Featuregroup Number:Rescale of 18 : 1 : 6 : 0

	f1-score	precision	recall	support
AFIB	0.869403	0.888742	0.851073	204.0
GSVT	0.906444	0.900811	0.912183	300.0
SB	0.926121	0.924170	0.928091	892.0
SR	0.916256	0.915609	0.916934	681.0
macro avg	0.904556	0.907333	0.902070	2077.0
micro avg	0.914571	0.914571	0.914571	2077.0
weighted avg	0.914474	0.914509	0.914571	2077.0

The result of Model Number:Dataset Number:Featuregroup Number:Rescale of 18 : 1 : 6 : 1

	f1-score	precision	recall	support
AFIB	0.622168	0.598383	0.661305	875.0
GSVT	0.644672	0.622422	0.674850	691.0
SB	0.718284	0.705605	0.732216	1553.0
SR	0.608370	0.731530	0.525245	907.0
macro avg	0.648373	0.664485	0.648404	4026.0
micro avg	0.660331	0.660331	0.660331	4026.0
weighted avg	0.659998	0.673865	0.660331	4026.0

The result of Model Number:Dataset Number:Featuregroup Number:Rescale of 18 : 1 : 7 : 0

	f1-score	precision	recall	support
AFIB	0.717448	0.709521	0.725569	875.0
GSVT	0.730375	0.734872	0.725945	691.0
SB	0.777344	0.776050	0.778646	1553.0
SR	0.758416	0.765508	0.751474	907.0
macro avg	0.745896	0.746488	0.745408	4026.0
micro avg	0.751943	0.751943	0.751943	4026.0
weighted avg	0.752001	0.752148	0.751943	4026.0

The result of Model Number:Dataset Number:Featuregroup Number:Rescale of 18 : 1 : 7 : 1

	f1-score	precision	recall	support
AFIB	0.830710	0.826060	0.835454	875.0
GSVT	0.837475	0.842416	0.832643	691.0
SB	0.882211	0.879450	0.884993	1553.0
SR	0.864746	0.870467	0.859120	907.0
macro avg	0.853786	0.854598	0.853052	4026.0
micro avg	0.859412	0.859412	0.859412	4026.0
weighted avg	0.859405	0.859466	0.859412	4026.0

The result of Model Number:Dataset Number:Featuregroup Number:Rescale of 18 : 1 : 8 : 0

	f1-score	precision	recall	support
AFIB	0.707948	0.711202	0.704758	875.0
GSVT	0.678521	0.682609	0.674505	691.0
SB	0.727825	0.695383	0.763479	1553.0
SR	0.622450	0.677618	0.575713	907.0
macro avg	0.684186	0.691703	0.679614	4026.0
micro avg	0.693145	0.693145	0.693145	4026.0
weighted avg	0.691303	0.692626	0.693145	4026.0

The result of Model Number:Dataset Number:Featuregroup Number:Rescale of 18 : 1 : 8 :
1

	f1-score	precision	recall	support
AFIB	0.742555	0.766357	0.720410	204.0
GSVT	0.764770	0.751613	0.778510	300.0
SB	0.778000	0.770309	0.785848	892.0
SR	0.757198	0.766673	0.747970	681.0
macro avg	0.760631	0.763738	0.758184	2077.0
micro avg	0.765942	0.765942	0.765942	2077.0
weighted avg	0.765787	0.766028	0.765942	2077.0

The result of Model Number:Dataset Number:Featuregroup Number:Rescale of 18 : 1 : 9 :
0

	f1-score	precision	recall	support
AFIB	0.761576	0.767360	0.755943	204.0
GSVT	0.777168	0.769577	0.784926	300.0
SB	0.795296	0.793263	0.797346	892.0
SR	0.786860	0.791305	0.782474	681.0
macro avg	0.780225	0.780376	0.780172	2077.0
micro avg	0.786609	0.786609	0.786609	2077.0
weighted avg	0.786599	0.786655	0.786609	2077.0

The result of Model Number:Dataset Number:Featuregroup Number:Rescale of 18 : 1 : 9 :
1

	f1-score	precision	recall	support
AFIB	0.905047	0.911381	0.898833	204.0
GSVT	0.926104	0.921538	0.930730	300.0
SB	0.944511	0.942939	0.946099	892.0
SR	0.935110	0.937330	0.932912	681.0
macro avg	0.927693	0.928297	0.927143	2077.0
micro avg	0.934913	0.934913	0.934913	2077.0
weighted avg	0.934894	0.934909	0.934913	2077.0

The result of Model Number:Dataset Number:Featuregroup Number:Rescale of 18 : 1 :
10 : 0

	f1-score	precision	recall	support
AFIB	0.885177	0.877263	0.893275	875.0
GSVT	0.899390	0.902948	0.895891	691.0
SB	0.958942	0.957162	0.960732	1553.0

SR 0.934642 0.943282 0.926193 907.0
macro avg 0.919538 0.920164 0.919023 4026.0
micro avg 0.927161 0.927161 0.927161 4026.0
weighted avg 0.927214 0.927365 0.927161 4026.0
The result of Model Number:Dataset Number:Featuregroup Number:Rescale of 18 : 1 : 10 : 1

	f1-score	precision	recall	support
AFIB	0.659260	0.690911	0.630513	204.0
GSVT	0.687935	0.682093	0.693896	300.0
SB	0.698746	0.693657	0.703914	892.0
SR	0.691396	0.691716	0.691096	681.0
macro avg	0.684334	0.689594	0.679855	2077.0
micro avg	0.691055	0.691055	0.691055	2077.0
weighted avg	0.690896	0.691081	0.691055	2077.0

The result of Model Number:Dataset Number:Featuregroup Number:Rescale of 19 : 0 : 0 : 0

	f1-score	precision	recall	support
AFIB	0.726600	0.736886	0.717194	204.0
GSVT	0.741686	0.728030	0.756000	300.0
SB	0.757494	0.749118	0.766071	892.0
SR	0.741574	0.756678	0.727106	681.0
macro avg	0.741839	0.742678	0.741593	2077.0
micro avg	0.747040	0.747040	0.747040	2077.0
weighted avg	0.746957	0.747350	0.747040	2077.0

The result of Model Number:Dataset Number:Featuregroup Number:Rescale of 19 : 0 : 0 : 1

	f1-score	precision	recall	support
AFIB	0.795715	0.837611	0.758024	204.0
GSVT	0.835672	0.828352	0.843159	300.0
SB	0.857356	0.852063	0.862731	892.0
SR	0.845659	0.843944	0.847401	681.0
macro avg	0.833600	0.840493	0.827829	2077.0
micro avg	0.844593	0.844593	0.844593	2077.0
weighted avg	0.844335	0.844557	0.844593	2077.0

The result of Model Number:Dataset Number:Featuregroup Number:Rescale of 19 : 0 : 1 : 0

	f1-score	precision	recall	support
AFIB	0.864178	0.859008	0.869527	875.0
GSVT	0.871254	0.875958	0.866727	691.0
SB	0.923399	0.920700	0.926128	1553.0
SR	0.902534	0.909017	0.896160	907.0
macro avg	0.890341	0.891171	0.889636	4026.0
micro avg	0.896880	0.896880	0.896880	4026.0
weighted avg	0.896878	0.896981	0.896880	4026.0

The result of Model Number:Dataset Number:Featuregroup Number:Rescale of 19 : 0 : 1 : 1

	f1-score	precision	recall	support
AFIB	0.887041	0.891246	0.882882	875.0
GSVT	0.898045	0.893808	0.902343	691.0
SB	0.947228	0.945228	0.949241	1553.0
SR	0.929278	0.931880	0.926712	907.0
macro avg	0.915398	0.915541	0.915294	4026.0
micro avg	0.921694	0.921694	0.921694	4026.0
weighted avg	0.921662	0.921663	0.921694	4026.0

The result of Model Number:Dataset Number:Featuregroup Number:Rescale of 19 : 0 : 2 : 0

	f1-score	precision	recall	support
AFIB	0.717243	0.731884	0.703492	204.0
GSVT	0.743287	0.736829	0.749994	300.0
SB	0.757578	0.756225	0.758937	892.0
SR	0.750526	0.750974	0.750084	681.0
macro avg	0.742158	0.743978	0.740627	2077.0
micro avg	0.749297	0.749297	0.749297	2077.0
weighted avg	0.749240	0.749311	0.749297	2077.0

The result of Model Number:Dataset Number:Featuregroup Number:Rescale of 19 : 0 : 2 : 1

	f1-score	precision	recall	support
AFIB	0.745423	0.728483	0.763221	875.0
GSVT	0.770969	0.783472	0.758923	691.0
SB	0.822540	0.822545	0.822540	1553.0
SR	0.801488	0.810619	0.792562	907.0
macro avg	0.785105	0.786280	0.784312	4026.0
micro avg	0.791975	0.791975	0.791975	4026.0
weighted avg	0.792185	0.792709	0.791975	4026.0

The result of Model Number:Dataset Number:Featuregroup Number:Rescale of 19 : 0 : 3 : 0

	f1-score	precision	recall	support
AFIB	0.791218	0.826947	0.758708	204.0
GSVT	0.818738	0.809543	0.828293	300.0
SB	0.787595	0.753258	0.842738	892.0
SR	0.656175	0.661770	0.650785	681.0
macro avg	0.763432	0.762879	0.770131	2077.0
micro avg	0.769462	0.769462	0.769462	2077.0
weighted avg	0.749360	0.738628	0.769462	2077.0

The result of Model Number:Dataset Number:Featuregroup Number:Rescale of 19 : 0 : 3 : 1

	f1-score	precision	recall	support
AFIB	0.784399	0.821954	0.750311	204.0

GSVT 0.826184 0.817339 0.835252 300.0
SB 0.843732 0.839071 0.848456 892.0
SR 0.834201 0.833478 0.834940 681.0
macro avg 0.822129 0.827961 0.817240 2077.0
micro avg 0.832478 0.832478 0.832478 2077.0
weighted avg 0.832245 0.832417 0.832478 2077.0
The result of Model Number:Dataset Number:Featuregroup Number:Rescale of 19 : 0 : 4 : 0

	f1-score	precision	recall	support
AFIB	0.704128	0.699523	0.708854	875.0
GSVT	0.725056	0.731225	0.719086	691.0
SB	0.771919	0.770238	0.773610	1553.0
SR	0.753107	0.756131	0.750119	907.0
macro avg	0.738553	0.739279	0.737917	4026.0
micro avg	0.744886	0.744886	0.744886	4026.0
weighted avg	0.744904	0.744995	0.744886	4026.0

The result of Model Number:Dataset Number:Featuregroup Number:Rescale of 19 : 0 : 4 : 1

	f1-score	precision	recall	support
AFIB	0.661450	0.666679	0.656324	875.0
GSVT	0.678094	0.676660	0.679559	691.0
SB	0.722868	0.716479	0.729381	1553.0
SR	0.703624	0.710525	0.696873	907.0
macro avg	0.691509	0.692586	0.690534	4026.0
micro avg	0.697629	0.697629	0.697629	4026.0
weighted avg	0.697499	0.697480	0.697629	4026.0

The result of Model Number:Dataset Number:Featuregroup Number:Rescale of 19 : 0 : 5 : 0

	f1-score	precision	recall	support
AFIB	0.922831	0.941669	0.905139	204.0
GSVT	0.956340	0.948032	0.964971	300.0
SB	0.974727	0.972987	0.976476	892.0
SR	0.965655	0.966230	0.965085	681.0
macro avg	0.954888	0.957230	0.952918	2077.0
micro avg	0.964073	0.964073	0.964073	2077.0
weighted avg	0.964000	0.964091	0.964073	2077.0

The result of Model Number:Dataset Number:Featuregroup Number:Rescale of 19 : 0 : 5 : 1

	f1-score	precision	recall	support
AFIB	0.879611	0.891360	0.868415	204.0
GSVT	0.895150	0.889697	0.900807	300.0
SB	0.916728	0.915915	0.917548	892.0
SR	0.909954	0.910092	0.909822	681.0
macro avg	0.900360	0.901766	0.899148	2077.0

micro avg 0.907771 0.907771 0.907771 2077.0
weighted avg 0.907744 0.907807 0.907771 2077.0
The result of Model Number:Dataset Number:Featuregroup Number:Rescale of 19 : 0 : 6 : 0
f1-score precision recall support
AFIB 0.765407 0.784467 0.747288 875.0
GSVT 0.808707 0.812141 0.805337 691.0
SB 0.877190 0.873834 0.880577 1553.0
SR 0.834401 0.818852 0.850596 907.0
macro avg 0.821426 0.822323 0.820949 4026.0
micro avg 0.831940 0.831940 0.831940 4026.0
weighted avg 0.831502 0.831436 0.831940 4026.0
The result of Model Number:Dataset Number:Featuregroup Number:Rescale of 19 : 0 : 6 : 1
f1-score precision recall support
AFIB 0.888847 0.906678 0.871881 204.0
GSVT 0.909447 0.899993 0.919149 300.0
SB 0.926266 0.922663 0.929901 892.0
SR 0.916533 0.920357 0.912762 681.0
macro avg 0.910273 0.912423 0.908423 2077.0
micro avg 0.917030 0.917030 0.917030 2077.0
weighted avg 0.916970 0.917063 0.917030 2077.0
The result of Model Number:Dataset Number:Featuregroup Number:Rescale of 19 : 0 : 7 : 0
f1-score precision recall support
AFIB 0.850000 0.858252 0.842432 204.0
GSVT 0.875480 0.871342 0.879889 300.0
SB 0.893209 0.892212 0.894209 892.0
SR 0.885349 0.886266 0.884439 681.0
macro avg 0.876010 0.877018 0.875242 2077.0
micro avg 0.883852 0.883852 0.883852 2077.0
weighted avg 0.883827 0.883913 0.883852 2077.0
The result of Model Number:Dataset Number:Featuregroup Number:Rescale of 19 : 0 : 7 : 1
f1-score precision recall support
AFIB 0.736668 0.731851 0.741614 875.0
GSVT 0.758564 0.765018 0.752318 691.0
SB 0.807593 0.805834 0.809361 1553.0
SR 0.787912 0.791075 0.784785 907.0
macro avg 0.772684 0.773445 0.772020 4026.0
micro avg 0.779310 0.779310 0.779310 4026.0
weighted avg 0.779329 0.779424 0.779310 4026.0
The result of Model Number:Dataset Number:Featuregroup Number:Rescale of 19 : 0 : 8 : 0

	f1-score	precision	recall	support
AFIB	0.641109	0.634183	0.729795	204.0
GSVT	0.767045	0.761069	0.773183	300.0
SB	0.625982	0.624673	0.627298	892.0
SR	0.776232	0.776014	0.776460	681.0
macro avg	0.702592	0.698985	0.726684	2077.0
micro avg	0.707343	0.707343	0.707343	2077.0
weighted avg	0.697106	0.694929	0.707343	2077.0

The result of Model Number:Dataset Number:Featuregroup Number:Rescale of 19 : 0 : 8 : 1

	f1-score	precision	recall	support
AFIB	0.621806	0.699165	0.560408	204.0
GSVT	0.737727	0.721974	0.754216	300.0
SB	0.787537	0.783523	0.791597	892.0
SR	0.769869	0.760120	0.779922	681.0
macro avg	0.729235	0.741195	0.721535	2077.0
micro avg	0.759662	0.759662	0.759662	2077.0
weighted avg	0.758272	0.758674	0.759662	2077.0

The result of Model Number:Dataset Number:Featuregroup Number:Rescale of 19 : 0 : 9 : 0

	f1-score	precision	recall	support
AFIB	0.803569	0.779552	0.856814	875.0
GSVT	0.688138	0.673069	0.704153	691.0
SB	0.912733	0.905297	0.920536	1553.0
SR	0.856275	0.892226	0.824717	907.0
macro avg	0.815179	0.812536	0.826555	4026.0
micro avg	0.847961	0.847961	0.847961	4026.0
weighted avg	0.837741	0.835165	0.847961	4026.0

The result of Model Number:Dataset Number:Featuregroup Number:Rescale of 19 : 0 : 9 : 1

	f1-score	precision	recall	support
AFIB	0.823795	0.829774	0.817961	875.0
GSVT	0.841866	0.834041	0.849902	691.0
SB	0.904759	0.899297	0.910293	1553.0
SR	0.875106	0.884878	0.865552	907.0
macro avg	0.861382	0.861997	0.860927	4026.0
micro avg	0.869781	0.869781	0.869781	4026.0
weighted avg	0.869688	0.869738	0.869781	4026.0

The result of Model Number:Dataset Number:Featuregroup Number:Rescale of 19 : 0 : 10 : 0

	f1-score	precision	recall	support
AFIB	0.801922	0.835838	0.770918	204.0
GSVT	0.738512	0.739805	0.748302	300.0
SB	0.795077	0.757200	0.837714	892.0

SR 0.702887 0.759499 0.655400 681.0
macro avg 0.759599 0.773086 0.753083 2077.0
micro avg 0.758462 0.758462 0.758462 2077.0
weighted avg 0.757352 0.763165 0.758462 2077.0
The result of Model Number:Dataset Number:Featuregroup Number:Rescale of 19 : 0 : 10 : 1

	f1-score	precision	recall	support
AFIB	0.668408	0.664198	0.752915	204.0
GSVT	0.814800	0.810609	0.819171	300.0
SB	0.838000	0.834013	0.842034	892.0
SR	0.657316	0.659482	0.655561	681.0
macro avg	0.744631	0.742075	0.767421	2077.0
micro avg	0.768839	0.768839	0.768839	2077.0
weighted avg	0.758750	0.756729	0.768839	2077.0

The result of Model Number:Dataset Number:Featuregroup Number:Rescale of 19 : 1 : 0 : 0

	f1-score	precision	recall	support
AFIB	0.671119	0.663203	0.679260	875.0
GSVT	0.682488	0.686806	0.678248	691.0
SB	0.727664	0.726685	0.728648	1553.0
SR	0.709787	0.716511	0.703212	907.0
macro avg	0.697764	0.698301	0.697342	4026.0
micro avg	0.703533	0.703533	0.703533	4026.0
weighted avg	0.703593	0.703752	0.703533	4026.0

The result of Model Number:Dataset Number:Featuregroup Number:Rescale of 19 : 1 : 0 : 1

	f1-score	precision	recall	support
AFIB	0.678699	0.670657	0.686994	875.0
GSVT	0.688359	0.695014	0.681898	691.0
SB	0.730579	0.727783	0.733401	1553.0
SR	0.713546	0.721778	0.705520	907.0
macro avg	0.702796	0.703808	0.701953	4026.0
micro avg	0.708194	0.708194	0.708194	4026.0
weighted avg	0.708220	0.708390	0.708194	4026.0

The result of Model Number:Dataset Number:Featuregroup Number:Rescale of 19 : 1 : 1 : 0

	f1-score	precision	recall	support
AFIB	0.776769	0.795409	0.759336	204.0
GSVT	0.804388	0.799726	0.809203	300.0
SB	0.821568	0.818554	0.824606	892.0
SR	0.814428	0.815032	0.813834	681.0
macro avg	0.804288	0.807180	0.801745	2077.0
micro avg	0.812439	0.812439	0.812439	2077.0
weighted avg	0.812345	0.812407	0.812439	2077.0

The result of Model Number:Dataset Number:Featuregroup Number:Rescale of 19 : 1 : 1 : 1

	f1-score	precision	recall	support
AFIB	0.780296	0.784389	0.776292	875.0
GSVT	0.796612	0.795181	0.798105	691.0
SB	0.864984	0.858351	0.871723	1553.0
SR	0.834467	0.842819	0.826283	907.0
macro avg	0.819090	0.820185	0.818101	4026.0
micro avg	0.828110	0.828110	0.828110	4026.0
weighted avg	0.827968	0.827935	0.828110	4026.0

The result of Model Number:Dataset Number:Featuregroup Number:Rescale of 19 : 1 : 2 : 0

	f1-score	precision	recall	support
AFIB	0.780747	0.779491	0.782326	875.0
GSVT	0.798951	0.794786	0.803205	691.0
SB	0.835344	0.828267	0.842550	1553.0
SR	0.803456	0.821318	0.786625	907.0
macro avg	0.804624	0.805965	0.803677	4026.0
micro avg	0.810109	0.810109	0.810109	4026.0
weighted avg	0.810048	0.810354	0.810109	4026.0

The result of Model Number:Dataset Number:Featuregroup Number:Rescale of 19 : 1 : 2 : 1

	f1-score	precision	recall	support
AFIB	0.883544	0.926377	0.844594	204.0
GSVT	0.920341	0.908315	0.932876	300.0
SB	0.945129	0.937942	0.952440	892.0
SR	0.918813	0.921559	0.916125	681.0
macro avg	0.916957	0.923548	0.911509	2077.0
micro avg	0.927115	0.927115	0.927115	2077.0
weighted avg	0.926871	0.927155	0.927115	2077.0

The result of Model Number:Dataset Number:Featuregroup Number:Rescale of 19 : 1 : 3 : 0

	f1-score	precision	recall	support
AFIB	0.797715	0.813787	0.782777	204.0
GSVT	0.823311	0.816699	0.830190	300.0
SB	0.841135	0.840288	0.841984	892.0
SR	0.833467	0.832989	0.833956	681.0
macro avg	0.823907	0.825941	0.822227	2077.0
micro avg	0.831833	0.831833	0.831833	2077.0
weighted avg	0.831782	0.831885	0.831833	2077.0

The result of Model Number:Dataset Number:Featuregroup Number:Rescale of 19 : 1 : 3 : 1

	f1-score	precision	recall	support
AFIB	0.685627	0.675379	0.696215	875.0

GSVT 0.698772 0.704748 0.692908 691.0
SB 0.746622 0.744051 0.749216 1553.0
SR 0.724769 0.735579 0.714297 907.0
macro avg 0.713947 0.714939 0.713159 4026.0
micro avg 0.720166 0.720166 0.720166 4026.0
weighted avg 0.720230 0.720472 0.720166 4026.0
The result of Model Number:Dataset Number:Featuregroup Number:Rescale of 19 : 1 : 4 : 0

	f1-score	precision	recall	support
AFIB	0.816791	0.814147	0.819478	875.0
GSVT	0.826263	0.828830	0.823793	691.0
SB	0.871841	0.869944	0.873750	1553.0
SR	0.855764	0.859801	0.851796	907.0
macro avg	0.842665	0.843180	0.842204	4026.0
micro avg	0.848435	0.848435	0.848435	4026.0
weighted avg	0.848432	0.848475	0.848435	4026.0

The result of Model Number:Dataset Number:Featuregroup Number:Rescale of 19 : 1 : 4 : 1

	f1-score	precision	recall	support
AFIB	0.698719	0.711577	0.686673	204.0
GSVT	0.724498	0.718430	0.730795	300.0
SB	0.738102	0.736865	0.739343	892.0
SR	0.731585	0.732235	0.730941	681.0
macro avg	0.723226	0.724777	0.721938	2077.0
micro avg	0.730180	0.730180	0.730180	2077.0
weighted avg	0.730132	0.730201	0.730180	2077.0

The result of Model Number:Dataset Number:Featuregroup Number:Rescale of 19 : 1 : 5 : 0

	f1-score	precision	recall	support
AFIB	0.846915	0.858131	0.836117	204.0
GSVT	0.872473	0.869324	0.875664	300.0
SB	0.883340	0.880591	0.886110	892.0
SR	0.873715	0.875398	0.872063	681.0
macro avg	0.869111	0.870861	0.867488	2077.0
micro avg	0.875085	0.875085	0.875085	2077.0
weighted avg	0.875037	0.875055	0.875085	2077.0

The result of Model Number:Dataset Number:Featuregroup Number:Rescale of 19 : 1 : 5 : 1

	f1-score	precision	recall	support
AFIB	0.754318	0.753639	0.755071	875.0
GSVT	0.767386	0.760360	0.774603	691.0
SB	0.805280	0.798200	0.812487	1553.0
SR	0.767368	0.786339	0.749358	907.0
macro avg	0.773588	0.774634	0.772880	4026.0

micro avg 0.779284 0.779284 0.779284 4026.0
weighted avg 0.779159 0.779348 0.779284 4026.0
The result of Model Number:Dataset Number:Featuregroup Number:Rescale of 19 : 1 : 6 : 0

	f1-score	precision	recall	support
AFIB	0.683172	0.750146	0.627844	204.0
GSVT	0.801541	0.790737	0.812772	300.0
SB	0.850986	0.848147	0.853847	892.0
SR	0.828870	0.817757	0.840310	681.0
macro avg	0.791142	0.801697	0.783693	2077.0
micro avg	0.821278	0.821278	0.821278	2077.0
weighted avg	0.820110	0.820265	0.821278	2077.0

The result of Model Number:Dataset Number:Featuregroup Number:Rescale of 19 : 1 : 6 : 1

	f1-score	precision	recall	support
AFIB	0.870292	0.894284	0.847729	204.0
GSVT	0.893454	0.876914	0.910714	300.0
SB	0.907944	0.899456	0.916596	892.0
SR	0.885122	0.897147	0.873430	681.0
macro avg	0.889203	0.891950	0.887117	2077.0
micro avg	0.894829	0.894829	0.894829	2077.0
weighted avg	0.894670	0.894935	0.894829	2077.0

The result of Model Number:Dataset Number:Featuregroup Number:Rescale of 19 : 1 : 7 : 0

	f1-score	precision	recall	support
AFIB	0.812777	0.813502	0.812136	875.0
GSVT	0.827665	0.829836	0.825644	691.0
SB	0.878577	0.872727	0.884515	1553.0
SR	0.861459	0.869324	0.853766	907.0
macro avg	0.845119	0.846347	0.844015	4026.0
micro avg	0.851753	0.851753	0.851753	4026.0
weighted avg	0.851681	0.851727	0.851753	4026.0

The result of Model Number:Dataset Number:Featuregroup Number:Rescale of 19 : 1 : 7 : 1

	f1-score	precision	recall	support
AFIB	0.713658	0.718995	0.708427	204.0
GSVT	0.731893	0.728031	0.735798	300.0
SB	0.744245	0.743249	0.745245	892.0
SR	0.737690	0.739104	0.736283	681.0
macro avg	0.731871	0.732345	0.731438	2077.0
micro avg	0.737326	0.737326	0.737326	2077.0
weighted avg	0.737307	0.737310	0.737326	2077.0

The result of Model Number:Dataset Number:Featuregroup Number:Rescale of 19 : 1 : 8 : 0

	f1-score	precision	recall	support
AFIB	0.690010	0.747612	0.642025	204.0
GSVT	0.740116	0.730810	0.749964	300.0
SB	0.759010	0.748377	0.770039	892.0
SR	0.738306	0.741765	0.735239	681.0
macro avg	0.731861	0.742141	0.724317	2077.0
micro avg	0.743156	0.743156	0.743156	2077.0
weighted avg	0.742716	0.743596	0.743156	2077.0

The result of Model Number:Dataset Number:Featuregroup Number:Rescale of 19 : 1 : 8 : 1

	f1-score	precision	recall	support
AFIB	0.777658	0.793809	0.762329	875.0
GSVT	0.758371	0.732031	0.820276	691.0
SB	0.893859	0.889828	0.897941	1553.0
SR	0.679554	0.668582	0.690952	907.0
macro avg	0.777360	0.771063	0.792875	4026.0
micro avg	0.808506	0.808506	0.808506	4026.0
weighted avg	0.797070	0.792033	0.808506	4026.0

The result of Model Number:Dataset Number:Featuregroup Number:Rescale of 19 : 1 : 9 : 0

	f1-score	precision	recall	support
AFIB	0.848708	0.849311	0.848122	875.0
GSVT	0.860144	0.861840	0.858529	691.0
SB	0.913618	0.910582	0.916677	1553.0
SR	0.892150	0.895467	0.888888	907.0
macro avg	0.878655	0.879300	0.878054	4026.0
micro avg	0.885537	0.885537	0.885537	4026.0
weighted avg	0.885496	0.885494	0.885537	4026.0

The result of Model Number:Dataset Number:Featuregroup Number:Rescale of 19 : 1 : 9 : 1

	f1-score	precision	recall	support
AFIB	0.810692	0.825279	0.796851	204.0
GSVT	0.828981	0.817325	0.841111	300.0
SB	0.841736	0.831791	0.851926	892.0
SR	0.824807	0.839492	0.810637	681.0
macro avg	0.826554	0.828472	0.825132	2077.0
micro avg	0.831417	0.831417	0.831417	2077.0
weighted avg	0.831294	0.831587	0.831417	2077.0

The result of Model Number:Dataset Number:Featuregroup Number:Rescale of 19 : 1 : 10 : 0

	f1-score	precision	recall	support
AFIB	0.839135	0.829054	0.849495	875.0
GSVT	0.855275	0.862200	0.848466	691.0
SB	0.911656	0.908167	0.915177	1553.0

SR 0.890281 0.901665 0.879193 907.0
macro avg 0.874087 0.875271 0.873083 4026.0
micro avg 0.881345 0.881345 0.881345 4026.0
weighted avg 0.881402 0.881618 0.881345 4026.0
The result of Model Number:Dataset Number:Featuregroup Number:Rescale of 19 : 1 : 10 : 1

	f1-score	precision	recall	support
AFIB	0.675533	0.671751	0.679390	875.0
GSVT	0.681034	0.685052	0.677105	691.0
SB	0.717413	0.715168	0.719675	1553.0
SR	0.703210	0.707863	0.698635	907.0
macro avg	0.694297	0.694958	0.693701	4026.0
micro avg	0.698873	0.698873	0.698873	4026.0
weighted avg	0.698867	0.698917	0.698873	4026.0

The result of Model Number:Dataset Number:Featuregroup Number:Rescale of 20 : 0 : 0 : 0

	f1-score	precision	recall	support
AFIB	0.703792	0.722389	0.686197	204.0
GSVT	0.723092	0.717086	0.729333	300.0
SB	0.743018	0.740291	0.745766	892.0
SR	0.731513	0.732400	0.730649	681.0
macro avg	0.725354	0.728041	0.722986	2077.0
micro avg	0.732585	0.732585	0.732585	2077.0
weighted avg	0.732515	0.732594	0.732585	2077.0

The result of Model Number:Dataset Number:Featuregroup Number:Rescale of 20 : 0 : 0 : 1

	f1-score	precision	recall	support
AFIB	0.771219	0.789381	0.753940	875.0
GSVT	0.812979	0.815032	0.810989	691.0
SB	0.884129	0.880464	0.887828	1553.0
SR	0.841255	0.828006	0.854974	907.0
macro avg	0.827395	0.828221	0.826933	4026.0
micro avg	0.838140	0.838140	0.838140	4026.0
weighted avg	0.837719	0.837620	0.838140	4026.0

The result of Model Number:Dataset Number:Featuregroup Number:Rescale of 20 : 0 : 1 : 0

	f1-score	precision	recall	support
AFIB	0.686877	0.696601	0.677985	204.0
GSVT	0.701138	0.688229	0.714669	300.0
SB	0.716082	0.708164	0.724190	892.0
SR	0.701033	0.715311	0.687355	681.0
macro avg	0.701282	0.702076	0.701050	2077.0
micro avg	0.706199	0.706199	0.706199	2077.0
weighted avg	0.706120	0.706492	0.706199	2077.0

The result of Model Number:Dataset Number:Featuregroup Number:Rescale of 20 : 0 : 1 : 1

	f1-score	precision	recall	support
AFIB	0.852786	0.840796	0.865164	875.0
GSVT	0.871146	0.877683	0.864729	691.0
SB	0.926364	0.923642	0.929107	1553.0
SR	0.903546	0.916046	0.891397	907.0
macro avg	0.888461	0.889542	0.887599	4026.0
micro avg	0.895665	0.895665	0.895665	4026.0
weighted avg	0.895755	0.896037	0.895665	4026.0

The result of Model Number:Dataset Number:Featuregroup Number:Rescale of 20 : 0 : 2 : 0

	f1-score	precision	recall	support
AFIB	0.770226	0.780641	0.760407	204.0
GSVT	0.799482	0.798184	0.800812	300.0
SB	0.816844	0.815756	0.817942	892.0
SR	0.808494	0.807459	0.809562	681.0
macro avg	0.798761	0.800510	0.797181	2077.0
micro avg	0.807069	0.807069	0.807069	2077.0
weighted avg	0.807020	0.807048	0.807069	2077.0

The result of Model Number:Dataset Number:Featuregroup Number:Rescale of 20 : 0 : 2 : 1

	f1-score	precision	recall	support
AFIB	0.761790	0.761591	0.762063	875.0
GSVT	0.777704	0.774763	0.780750	691.0
SB	0.827983	0.824245	0.831768	1553.0
SR	0.800078	0.809219	0.791172	907.0
macro avg	0.791889	0.792455	0.791438	4026.0
micro avg	0.798716	0.798716	0.798716	4026.0
weighted avg	0.798681	0.798750	0.798716	4026.0

The result of Model Number:Dataset Number:Featuregroup Number:Rescale of 20 : 0 : 3 : 0

	f1-score	precision	recall	support
AFIB	0.757947	0.781291	0.736109	204.0
GSVT	0.777283	0.767768	0.787122	300.0
SB	0.798396	0.793652	0.803223	892.0
SR	0.784938	0.788809	0.781135	681.0
macro avg	0.779641	0.782880	0.776897	2077.0
micro avg	0.787063	0.787063	0.787063	2077.0
weighted avg	0.786961	0.787111	0.787063	2077.0

The result of Model Number:Dataset Number:Featuregroup Number:Rescale of 20 : 0 : 3 : 1

	f1-score	precision	recall	support
AFIB	0.748878	0.738505	0.759576	875.0

GSVT 0.765487 0.771682 0.759395 691.0
SB 0.812555 0.810221 0.814909 1553.0
SR 0.792789 0.803107 0.782740 907.0
macro avg 0.779927 0.780879 0.779155 4026.0
micro avg 0.786108 0.786108 0.786108 4026.0
weighted avg 0.786184 0.786417 0.786108 4026.0
The result of Model Number:Dataset Number:Featuregroup Number:Rescale of 20 : 0 : 4 : 0
f1-score precision recall support
AFIB 0.815438 0.819898 0.811054 875.0
GSVT 0.811552 0.804709 0.818594 691.0
SB 0.853550 0.845467 0.861793 1553.0
SR 0.821783 0.837113 0.807017 907.0
macro avg 0.825581 0.826797 0.824614 4026.0
micro avg 0.831011 0.831011 0.831011 4026.0
weighted avg 0.830902 0.831032 0.831011 4026.0
The result of Model Number:Dataset Number:Featuregroup Number:Rescale of 20 : 0 : 4 : 1
f1-score precision recall support
AFIB 0.812504 0.791160 0.863369 875.0
GSVT 0.781621 0.739814 0.891032 691.0
SB 0.740627 0.735946 0.745389 1553.0
SR 0.719402 0.731797 0.707620 907.0
macro avg 0.763539 0.749679 0.801853 4026.0
micro avg 0.787519 0.787519 0.787519 4026.0
weighted avg 0.758503 0.747675 0.787519 4026.0
The result of Model Number:Dataset Number:Featuregroup Number:Rescale of 20 : 0 : 5 : 0
f1-score precision recall support
AFIB 0.732917 0.746405 0.720282 204.0
GSVT 0.759958 0.753593 0.766563 300.0
SB 0.774228 0.772931 0.775530 892.0
SR 0.767392 0.768074 0.766716 681.0
macro avg 0.758624 0.760251 0.757273 2077.0
micro avg 0.765919 0.765919 0.765919 2077.0
weighted avg 0.765868 0.765940 0.765919 2077.0
The result of Model Number:Dataset Number:Featuregroup Number:Rescale of 20 : 0 : 5 : 1
f1-score precision recall support
AFIB 0.831774 0.824418 0.839297 875.0
GSVT 0.756227 0.732348 0.841317 691.0
SB 0.681779 0.654602 0.724280 1553.0
SR 0.702673 0.709943 0.695595 907.0
macro avg 0.743113 0.730328 0.775122 4026.0

micro avg 0.762903 0.762903 0.762903 4026.0
weighted avg 0.731863 0.717321 0.762903 4026.0
The result of Model Number:Dataset Number:Featuregroup Number:Rescale of 20 : 0 : 6 : 0

	f1-score	precision	recall	support
AFIB	0.822431	0.891087	0.765237	204.0
GSVT	0.882152	0.871060	0.893891	300.0
SB	0.904673	0.891999	0.917817	892.0
SR	0.879995	0.884117	0.876339	681.0
macro avg	0.872313	0.884566	0.863321	2077.0
micro avg	0.885776	0.885776	0.885776	2077.0
weighted avg	0.885251	0.886301	0.885776	2077.0

The result of Model Number:Dataset Number:Featuregroup Number:Rescale of 20 : 0 : 6 : 1

	f1-score	precision	recall	support
AFIB	0.799555	0.801192	0.798013	204.0
GSVT	0.817141	0.813965	0.820400	300.0
SB	0.835281	0.834167	0.836403	892.0
SR	0.829146	0.831597	0.826712	681.0
macro avg	0.820281	0.820230	0.820382	2077.0
micro avg	0.827144	0.827144	0.827144	2077.0
weighted avg	0.827141	0.827168	0.827144	2077.0

The result of Model Number:Dataset Number:Featuregroup Number:Rescale of 20 : 0 : 7 : 0

	f1-score	precision	recall	support
AFIB	0.772582	0.778633	0.766666	875.0
GSVT	0.774620	0.764734	0.784813	691.0
SB	0.812488	0.809230	0.815782	1553.0
SR	0.795323	0.803130	0.787696	907.0
macro avg	0.788753	0.788931	0.788739	4026.0
micro avg	0.793464	0.793464	0.793464	4026.0
weighted avg	0.793449	0.793569	0.793464	4026.0

The result of Model Number:Dataset Number:Featuregroup Number:Rescale of 20 : 0 : 7 : 1

	f1-score	precision	recall	support
AFIB	0.724947	0.697232	0.770548	875.0
GSVT	0.751167	0.725243	0.786331	691.0
SB	0.836940	0.822167	0.853173	1553.0
SR	0.708869	0.852374	0.612013	907.0
macro avg	0.755481	0.774254	0.755516	4026.0
micro avg	0.769413	0.769413	0.769413	4026.0
weighted avg	0.769026	0.785184	0.769413	4026.0

The result of Model Number:Dataset Number:Featuregroup Number:Rescale of 20 : 0 : 8 : 0

	f1-score	precision	recall	support
AFIB	0.797388	0.776440	0.847306	875.0
GSVT	0.767079	0.726050	0.874455	691.0
SB	0.726847	0.722254	0.731521	1553.0
SR	0.706017	0.718182	0.694455	907.0
macro avg	0.749333	0.735731	0.786934	4026.0
micro avg	0.772867	0.772867	0.772867	4026.0
weighted avg	0.744391	0.733765	0.772867	4026.0

The result of Model Number:Dataset Number:Featuregroup Number:Rescale of 20 : 0 : 8 : 1

	f1-score	precision	recall	support
AFIB	0.703754	0.716815	0.691336	204.0
GSVT	0.719229	0.715458	0.723055	300.0
SB	0.730711	0.728114	0.733332	892.0
SR	0.723189	0.724492	0.721921	681.0
macro avg	0.719221	0.721219	0.717411	2077.0
micro avg	0.723981	0.723981	0.723981	2077.0
weighted avg	0.723939	0.723988	0.723981	2077.0

The result of Model Number:Dataset Number:Featuregroup Number:Rescale of 20 : 0 : 9 : 0

	f1-score	precision	recall	support
AFIB	0.663122	0.656908	0.669480	875.0
GSVT	0.674268	0.679675	0.669012	691.0
SB	0.714759	0.712022	0.717520	1553.0
SR	0.698766	0.705859	0.691833	907.0
macro avg	0.687729	0.688616	0.686961	4026.0
micro avg	0.692966	0.692966	0.692966	4026.0
weighted avg	0.692984	0.693103	0.692966	4026.0

The result of Model Number:Dataset Number:Featuregroup Number:Rescale of 20 : 0 : 9 : 1

	f1-score	precision	recall	support
AFIB	0.737400	0.746871	0.728203	875.0
GSVT	0.745558	0.739529	0.751806	691.0
SB	0.797534	0.795136	0.799948	1553.0
SR	0.777785	0.777458	0.778123	907.0
macro avg	0.764569	0.764749	0.764520	4026.0
micro avg	0.771176	0.771176	0.771176	4026.0
weighted avg	0.771095	0.771120	0.771176	4026.0

The result of Model Number:Dataset Number:Featuregroup Number:Rescale of 20 : 0 : 10 : 0

	f1-score	precision	recall	support
AFIB	0.842057	0.855400	0.829235	204.0
GSVT	0.863701	0.856351	0.871182	300.0
SB	0.878613	0.876655	0.880583	892.0

SR 0.870288 0.872222 0.868379 681.0
macro avg 0.863664 0.865157 0.862345 2077.0
micro avg 0.870180 0.870180 0.870180 2077.0
weighted avg 0.870139 0.870181 0.870180 2077.0
The result of Model Number:Dataset Number:Featuregroup Number:Rescale of 20 : 0 : 10 : 1

	f1-score	precision	recall	support
AFIB	0.826863	0.826812	0.826964	875.0
GSVT	0.828652	0.824172	0.833195	691.0
SB	0.868240	0.862463	0.874106	1553.0
SR	0.845185	0.859052	0.831790	907.0
macro avg	0.842235	0.843125	0.841514	4026.0
micro avg	0.847306	0.847306	0.847306	4026.0
weighted avg	0.847259	0.847374	0.847306	4026.0

The result of Model Number:Dataset Number:Featuregroup Number:Rescale of 20 : 1 : 0 : 0

	f1-score	precision	recall	support
AFIB	0.818687	0.818425	0.818992	875.0
GSVT	0.830888	0.820207	0.841947	691.0
SB	0.878863	0.876952	0.880787	1553.0
SR	0.854547	0.867073	0.842391	907.0
macro avg	0.845746	0.845664	0.846029	4026.0
micro avg	0.852040	0.852040	0.852040	4026.0
weighted avg	0.852072	0.852267	0.852040	4026.0

The result of Model Number:Dataset Number:Featuregroup Number:Rescale of 20 : 1 : 0 : 1

	f1-score	precision	recall	support
AFIB	0.808047	0.820298	0.796177	204.0
GSVT	0.821040	0.814119	0.828123	300.0
SB	0.839252	0.836545	0.841979	892.0
SR	0.830243	0.833327	0.827204	681.0
macro avg	0.824646	0.826072	0.823371	2077.0
micro avg	0.830635	0.830635	0.830635	2077.0
weighted avg	0.830603	0.830655	0.830635	2077.0

The result of Model Number:Dataset Number:Featuregroup Number:Rescale of 20 : 1 : 1 : 0

	f1-score	precision	recall	support
AFIB	0.637102	0.707053	0.579899	204.0
GSVT	0.743903	0.734603	0.753588	300.0
SB	0.791477	0.787438	0.795558	892.0
SR	0.772935	0.762129	0.784056	681.0
macro avg	0.736354	0.747806	0.728275	2077.0
micro avg	0.764543	0.764543	0.764543	2077.0
weighted avg	0.763363	0.763613	0.764543	2077.0

The result of Model Number:Dataset Number:Featuregroup Number:Rescale of 20 : 1 : 1 : 1

	f1-score	precision	recall	support
AFIB	0.661750	0.672496	0.651378	875.0
GSVT	0.703791	0.705996	0.701641	691.0
SB	0.765689	0.763050	0.768350	1553.0
SR	0.724884	0.716810	0.733186	907.0
macro avg	0.714028	0.714588	0.713639	4026.0
micro avg	0.723556	0.723556	0.723556	4026.0
weighted avg	0.723283	0.723160	0.723556	4026.0

The result of Model Number:Dataset Number:Featuregroup Number:Rescale of 20 : 1 : 2 : 0

	f1-score	precision	recall	support
AFIB	0.678332	0.684918	0.672293	204.0
GSVT	0.698666	0.695363	0.702185	300.0
SB	0.712814	0.712019	0.713613	892.0
SR	0.706542	0.707274	0.705816	681.0
macro avg	0.699089	0.699893	0.698476	2077.0
micro avg	0.705347	0.705347	0.705347	2077.0
weighted avg	0.705327	0.705395	0.705347	2077.0

The result of Model Number:Dataset Number:Featuregroup Number:Rescale of 20 : 1 : 2 : 1

	f1-score	precision	recall	support
AFIB	0.848771	0.856364	0.841417	875.0
GSVT	0.864982	0.848534	0.882245	691.0
SB	0.918106	0.910152	0.926203	1553.0
SR	0.880397	0.900639	0.861083	907.0
macro avg	0.878064	0.878922	0.877737	4026.0
micro avg	0.885561	0.885561	0.885561	4026.0
weighted avg	0.885423	0.885743	0.885561	4026.0

The result of Model Number:Dataset Number:Featuregroup Number:Rescale of 20 : 1 : 3 : 0

	f1-score	precision	recall	support
AFIB	0.810925	0.801965	0.820104	875.0
GSVT	0.825536	0.830619	0.820529	691.0
SB	0.878625	0.877162	0.880096	1553.0
SR	0.857231	0.865246	0.849384	907.0
macro avg	0.843079	0.843748	0.842528	4026.0
micro avg	0.849915	0.849915	0.849915	4026.0
weighted avg	0.849979	0.850146	0.849915	4026.0

The result of Model Number:Dataset Number:Featuregroup Number:Rescale of 20 : 1 : 3 : 1

	f1-score	precision	recall	support
AFIB	0.738318	0.744433	0.831614	204.0

GSVT	0.800656	0.766387	0.903327	300.0
SB	0.731028	0.728488	0.733589	892.0
SR	0.723760	0.723897	0.723628	681.0
macro avg	0.748440	0.740801	0.798039	2077.0
micro avg	0.764468	0.764468	0.764468	2077.0
weighted avg	0.739418	0.734023	0.764468	2077.0

The result of Model Number:Dataset Number:Featuregroup Number:Rescale of 20 : 1 : 4 : 0

	f1-score	precision	recall	support
AFIB	0.708055	0.779968	0.648962	204.0
GSVT	0.653884	0.699101	0.614467	300.0
SB	0.711886	0.665168	0.765674	892.0
SR	0.616149	0.648537	0.587011	681.0
macro avg	0.672493	0.698193	0.654029	2077.0
micro avg	0.673791	0.673791	0.673791	2077.0
weighted avg	0.671742	0.675892	0.673791	2077.0

The result of Model Number:Dataset Number:Featuregroup Number:Rescale of 20 : 1 : 4 : 1

	f1-score	precision	recall	support
AFIB	0.862056	0.881568	0.843499	204.0
GSVT	0.884996	0.880663	0.889411	300.0
SB	0.903101	0.900493	0.905731	892.0
SR	0.897299	0.896911	0.897700	681.0
macro avg	0.886863	0.889909	0.884085	2077.0
micro avg	0.894628	0.894628	0.894628	2077.0
weighted avg	0.894552	0.894596	0.894628	2077.0

The result of Model Number:Dataset Number:Featuregroup Number:Rescale of 20 : 1 : 5 : 0

	f1-score	precision	recall	support
AFIB	0.645631	0.640823	0.650577	875.0
GSVT	0.657672	0.660021	0.655380	691.0
SB	0.697871	0.696218	0.699533	1553.0
SR	0.681945	0.688085	0.675939	907.0
macro avg	0.670780	0.671287	0.670357	4026.0
micro avg	0.676000	0.676000	0.676000	4026.0
weighted avg	0.676030	0.676134	0.676000	4026.0

The result of Model Number:Dataset Number:Featuregroup Number:Rescale of 20 : 1 : 5 : 1

	f1-score	precision	recall	support
AFIB	0.700812	0.686659	0.715617	875.0
GSVT	0.724047	0.735560	0.713046	691.0
SB	0.770397	0.770354	0.770446	1553.0
SR	0.751719	0.758568	0.745004	907.0
macro avg	0.736744	0.737785	0.736028	4026.0

micro avg	0.742946	0.742946	0.742946	4026.0
weighted avg	0.743111	0.743537	0.742946	4026.0

The result of Model Number:Dataset Number:Featuregroup Number:Rescale of 20 : 1 : 6 : 0

	f1-score	precision	recall	support
AFIB	0.666860	0.656819	0.682765	875.0
GSVT	0.636731	0.634878	0.642163	691.0
SB	0.679947	0.633602	0.739261	1553.0
SR	0.503679	0.673269	0.417359	907.0
macro avg	0.621804	0.649642	0.620387	4026.0
micro avg	0.637797	0.637797	0.637797	4026.0
weighted avg	0.629975	0.647803	0.637797	4026.0

The result of Model Number:Dataset Number:Featuregroup Number:Rescale of 20 : 1 : 6 : 1

	f1-score	precision	recall	support
AFIB	0.836383	0.836164	0.836683	875.0
GSVT	0.853855	0.850627	0.857200	691.0
SB	0.909058	0.904953	0.913213	1553.0
SR	0.878421	0.888457	0.868643	907.0
macro avg	0.869429	0.870050	0.868935	4026.0
micro avg	0.876925	0.876925	0.876925	4026.0
weighted avg	0.876886	0.876962	0.876925	4026.0

The result of Model Number:Dataset Number:Featuregroup Number:Rescale of 20 : 1 : 7 : 0

	f1-score	precision	recall	support
AFIB	0.779693	0.817023	0.745810	204.0
GSVT	0.821227	0.812435	0.830241	300.0
SB	0.838671	0.834037	0.843366	892.0
SR	0.829197	0.828478	0.829931	681.0
macro avg	0.817197	0.822993	0.812337	2077.0
micro avg	0.827483	0.827483	0.827483	2077.0
weighted avg	0.827252	0.827423	0.827483	2077.0

The result of Model Number:Dataset Number:Featuregroup Number:Rescale of 20 : 1 : 7 : 1

	f1-score	precision	recall	support
AFIB	0.787647	0.867644	0.721912	204.0
GSVT	0.727388	0.777687	0.683540	300.0
SB	0.791910	0.739939	0.851744	892.0
SR	0.685411	0.721440	0.652997	681.0
macro avg	0.748089	0.776678	0.727548	2077.0
micro avg	0.749532	0.749532	0.749532	2077.0
weighted avg	0.747253	0.751869	0.749532	2077.0

The result of Model Number:Dataset Number:Featuregroup Number:Rescale of 20 : 1 : 8 : 0

	f1-score	precision	recall	support
AFIB	0.860547	0.878597	0.843246	204.0
GSVT	0.880510	0.875300	0.885791	300.0
SB	0.901976	0.899863	0.904099	892.0
SR	0.894357	0.894100	0.894622	681.0
macro avg	0.884347	0.886965	0.881940	2077.0
micro avg	0.892371	0.892371	0.892371	2077.0
weighted avg	0.892308	0.892337	0.892371	2077.0

The result of Model Number:Dataset Number:Featuregroup Number:Rescale of 20 : 1 : 8 : 1

	f1-score	precision	recall	support
AFIB	0.770417	0.797163	0.745512	204.0
GSVT	0.785189	0.776594	0.794074	300.0
SB	0.806092	0.800304	0.811966	892.0
SR	0.791696	0.795588	0.787855	681.0
macro avg	0.788349	0.792412	0.784852	2077.0
micro avg	0.794949	0.794949	0.794949	2077.0
weighted avg	0.794849	0.795025	0.794949	2077.0

The result of Model Number:Dataset Number:Featuregroup Number:Rescale of 20 : 1 : 9 : 0

	f1-score	precision	recall	support
AFIB	0.637802	0.707830	0.580536	204.0
GSVT	0.744721	0.735410	0.754416	300.0
SB	0.792346	0.788303	0.796432	892.0
SR	0.773784	0.762966	0.784917	681.0
macro avg	0.737163	0.748627	0.729075	2077.0
micro avg	0.765383	0.765383	0.765383	2077.0
weighted avg	0.764202	0.764452	0.765383	2077.0

The result of Model Number:Dataset Number:Featuregroup Number:Rescale of 20 : 1 : 9 : 1

	f1-score	precision	recall	support
AFIB	0.157858	0.159516	0.156234	175.0
GSVT	0.158113	0.156433	0.159829	138.2
SB	0.166520	0.165882	0.167164	310.6
SR	0.162667	0.163482	0.161859	181.4
macro avg	0.161289	0.161328	0.161272	805.2
micro avg	0.162334	0.162334	0.162334	805.2
weighted avg	0.162326	0.162336	0.162334	805.2

The result of Model Number:Dataset Number:Featuregroup Number:Rescale of 20 : 1 : 10 : 0

	f1-score	precision	recall	support
AFIB	0.628724	0.629779	0.644307	204.0
GSVT	0.622813	0.646982	0.601188	300.0
SB	0.660628	0.624399	0.701592	892.0

SR 0.572624 0.625469 0.529462 681.0
macro avg 0.621197 0.631657 0.619137 2077.0
micro avg 0.625026 0.625026 0.625026 2077.0
weighted avg 0.623178 0.628540 0.625026 2077.0
The result of Model Number:Dataset Number:Featuregroup Number:Rescale of 20 : 1 : 10 : 1

	f1-score	precision	recall	support
AFIB	0.667433	0.731673	0.613790	204.0
GSVT	0.776226	0.768250	0.784514	300.0
SB	0.822859	0.819659	0.826087	892.0
SR	0.803794	0.792679	0.815239	681.0
macro avg	0.767578	0.778065	0.759907	2077.0
micro avg	0.795674	0.795674	0.795674	2077.0
weighted avg	0.794607	0.794745	0.795674	2077.0

The result of Model Number:Dataset Number:Featuregroup Number:Rescale of 21 : 0 : 0 : 0

	f1-score	precision	recall	support
AFIB	0.648374	0.639258	0.657785	875.0
GSVT	0.662333	0.667303	0.657454	691.0
SB	0.704316	0.702246	0.706401	1553.0
SR	0.686967	0.696471	0.677730	907.0
macro avg	0.675497	0.676319	0.674842	4026.0
micro avg	0.680975	0.680975	0.680975	4026.0
weighted avg	0.681043	0.681258	0.680975	4026.0

The result of Model Number:Dataset Number:Featuregroup Number:Rescale of 21 : 0 : 0 : 1

	f1-score	precision	recall	support
AFIB	0.879993	0.919104	0.844197	204.0
GSVT	0.916664	0.909468	0.923995	300.0
SB	0.930667	0.923487	0.937967	892.0
SR	0.920370	0.921890	0.918888	681.0
macro avg	0.911923	0.918487	0.906262	2077.0
micro avg	0.920483	0.920483	0.920483	2077.0
weighted avg	0.920291	0.920508	0.920483	2077.0

The result of Model Number:Dataset Number:Featuregroup Number:Rescale of 21 : 0 : 1 : 0

	f1-score	precision	recall	support
AFIB	0.664577	0.666228	0.662979	875.0
GSVT	0.676022	0.678053	0.674089	691.0
SB	0.717138	0.712270	0.722077	1553.0
SR	0.703491	0.708713	0.698372	907.0
macro avg	0.690307	0.691316	0.689379	4026.0
micro avg	0.695656	0.695656	0.695656	4026.0
weighted avg	0.695583	0.695589	0.695656	4026.0

The result of Model Number:Dataset Number:Featuregroup Number:Rescale of 21 : 0 : 1 : 1

	f1-score	precision	recall	support
AFIB	0.748827	0.785129	0.715816	204.0
GSVT	0.780014	0.769821	0.790638	300.0
SB	0.801022	0.794931	0.807219	892.0
SR	0.778719	0.781046	0.776440	681.0
macro avg	0.777145	0.782732	0.772528	2077.0
micro avg	0.785755	0.785755	0.785755	2077.0
weighted avg	0.785548	0.785789	0.785755	2077.0

The result of Model Number:Dataset Number:Featuregroup Number:Rescale of 21 : 0 : 2 : 0

	f1-score	precision	recall	support
AFIB	0.760609	0.760971	0.760357	875.0
GSVT	0.780307	0.773553	0.787208	691.0
SB	0.833371	0.827298	0.839541	1553.0
SR	0.797768	0.813627	0.782557	907.0
macro avg	0.793014	0.793862	0.792416	4026.0
micro avg	0.800512	0.800512	0.800512	4026.0
weighted avg	0.800429	0.800578	0.800512	4026.0

The result of Model Number:Dataset Number:Featuregroup Number:Rescale of 21 : 0 : 2 : 1

	f1-score	precision	recall	support
AFIB	0.876719	0.895109	0.859093	204.0
GSVT	0.897057	0.891749	0.902437	300.0
SB	0.918927	0.916774	0.921090	892.0
SR	0.911165	0.910903	0.911435	681.0
macro avg	0.900967	0.903634	0.898514	2077.0
micro avg	0.909141	0.909141	0.909141	2077.0
weighted avg	0.909077	0.909107	0.909141	2077.0

The result of Model Number:Dataset Number:Featuregroup Number:Rescale of 21 : 0 : 3 : 0

	f1-score	precision	recall	support
AFIB	0.778386	0.791901	0.765424	204.0
GSVT	0.793725	0.791376	0.796104	300.0
SB	0.803084	0.800227	0.805965	892.0
SR	0.796555	0.797387	0.795743	681.0
macro avg	0.792937	0.795223	0.790809	2077.0
micro avg	0.797207	0.797207	0.797207	2077.0
weighted avg	0.797166	0.797200	0.797207	2077.0

The result of Model Number:Dataset Number:Featuregroup Number:Rescale of 21 : 0 : 3 : 1

	f1-score	precision	recall	support
AFIB	0.858428	0.905125	0.816533	204.0

GSVT 0.901191 0.887561 0.915283 300.0
SB 0.922607 0.916605 0.928702 892.0
SR 0.911224 0.912046 0.910426 681.0
macro avg 0.898363 0.905334 0.892736 2077.0
micro avg 0.909754 0.909754 0.909754 2077.0
weighted avg 0.909478 0.909788 0.909754 2077.0
The result of Model Number:Dataset Number:Featuregroup Number:Rescale of 21 : 0 : 4 : 0

	f1-score	precision	recall	support
AFIB	0.680515	0.676229	0.766553	204.0
GSVT	0.829558	0.825292	0.834009	300.0
SB	0.853178	0.849119	0.857286	892.0
SR	0.669222	0.671427	0.667436	681.0
macro avg	0.758118	0.755517	0.781321	2077.0
micro avg	0.782765	0.782765	0.782765	2077.0
weighted avg	0.772493	0.770435	0.782765	2077.0

The result of Model Number:Dataset Number:Featuregroup Number:Rescale of 21 : 0 : 4 : 1

	f1-score	precision	recall	support
AFIB	0.709925	0.728511	0.692588	204.0
GSVT	0.667990	0.687405	0.650007	300.0
SB	0.704873	0.680056	0.731583	892.0
SR	0.638881	0.659687	0.619476	681.0
macro avg	0.680417	0.688915	0.673413	2077.0
micro avg	0.679213	0.679213	0.679213	2077.0
weighted avg	0.678405	0.679198	0.679213	2077.0

The result of Model Number:Dataset Number:Featuregroup Number:Rescale of 21 : 0 : 5 : 0

	f1-score	precision	recall	support
AFIB	0.770856	0.772872	0.769001	204.0
GSVT	0.788248	0.785196	0.791389	300.0
SB	0.805926	0.804851	0.807008	892.0
SR	0.799471	0.801718	0.797241	681.0
macro avg	0.791125	0.791159	0.791160	2077.0
micro avg	0.797817	0.797817	0.797817	2077.0
weighted avg	0.797812	0.797844	0.797817	2077.0

The result of Model Number:Dataset Number:Featuregroup Number:Rescale of 21 : 0 : 5 : 1

	f1-score	precision	recall	support
AFIB	0.839364	0.817885	0.862079	875.0
GSVT	0.793458	0.838429	0.753250	691.0
SB	0.841343	0.774238	0.921206	1553.0
SR	0.672091	0.816343	0.571285	907.0
macro avg	0.786564	0.811724	0.776955	4026.0

micro avg	0.800696	0.800696	0.800696	4026.0
weighted avg	0.794564	0.804227	0.800696	4026.0

The result of Model Number:Dataset Number:Featuregroup Number:Rescale of 21 : 0 : 6 : 0

	f1-score	precision	recall	support
AFIB	0.693948	0.707490	0.681036	204.0
GSVT	0.711244	0.702066	0.720712	300.0
SB	0.723350	0.716198	0.730648	892.0
SR	0.708230	0.718070	0.698661	681.0
macro avg	0.709193	0.710956	0.707764	2077.0
micro avg	0.713852	0.713852	0.713852	2077.0
weighted avg	0.713756	0.713916	0.713852	2077.0

The result of Model Number:Dataset Number:Featuregroup Number:Rescale of 21 : 0 : 6 : 1

	f1-score	precision	recall	support
AFIB	0.801052	0.801953	0.800299	204.0
GSVT	0.819443	0.817614	0.821343	300.0
SB	0.836812	0.835695	0.837936	892.0
SR	0.830545	0.832636	0.828474	681.0
macro avg	0.821963	0.821974	0.822013	2077.0
micro avg	0.828740	0.828740	0.828740	2077.0
weighted avg	0.828736	0.828766	0.828740	2077.0

The result of Model Number:Dataset Number:Featuregroup Number:Rescale of 21 : 0 : 7 : 0

	f1-score	precision	recall	support
AFIB	0.673087	0.665572	0.680781	875.0
GSVT	0.686478	0.692766	0.680314	691.0
SB	0.732160	0.729776	0.734563	1553.0
SR	0.713180	0.720325	0.706178	907.0
macro avg	0.701226	0.702110	0.700459	4026.0
micro avg	0.707169	0.707169	0.707169	4026.0
weighted avg	0.707205	0.707341	0.707169	4026.0

The result of Model Number:Dataset Number:Featuregroup Number:Rescale of 21 : 0 : 7 : 1

	f1-score	precision	recall	support
AFIB	0.908405	0.915383	0.901739	204.0
GSVT	0.940394	0.939493	0.941328	300.0
SB	0.959634	0.957398	0.961889	892.0
SR	0.950802	0.952084	0.949545	681.0
macro avg	0.939809	0.941089	0.938625	2077.0
micro avg	0.948964	0.948964	0.948964	2077.0
weighted avg	0.948928	0.948943	0.948964	2077.0

The result of Model Number:Dataset Number:Featuregroup Number:Rescale of 21 : 0 : 8 : 0

	f1-score	precision	recall	support
AFIB	0.798300	0.804432	0.792442	204.0
GSVT	0.826412	0.825620	0.827233	300.0
SB	0.843320	0.841355	0.845302	892.0
SR	0.835559	0.836685	0.834454	681.0
macro avg	0.825898	0.827023	0.824858	2077.0
micro avg	0.833943	0.833943	0.833943	2077.0
weighted avg	0.833911	0.833925	0.833943	2077.0

The result of Model Number:Dataset Number:Featuregroup Number:Rescale of 21 : 0 : 8 : 1

	f1-score	precision	recall	support
AFIB	0.715958	0.729831	0.702923	204.0
GSVT	0.743721	0.737502	0.750182	300.0
SB	0.757598	0.756078	0.759127	892.0
SR	0.750374	0.751152	0.749602	681.0
macro avg	0.741913	0.743641	0.740458	2077.0
micro avg	0.749191	0.749191	0.749191	2077.0
weighted avg	0.749135	0.749202	0.749191	2077.0

The result of Model Number:Dataset Number:Featuregroup Number:Rescale of 21 : 0 : 9 : 0

	f1-score	precision	recall	support
AFIB	0.716531	0.739498	0.695161	204.0
GSVT	0.737967	0.725271	0.751225	300.0
SB	0.750733	0.743312	0.758306	892.0
SR	0.730660	0.739803	0.721756	681.0
macro avg	0.733973	0.736971	0.731612	2077.0
micro avg	0.739097	0.739097	0.739097	2077.0
weighted avg	0.738948	0.739181	0.739097	2077.0

The result of Model Number:Dataset Number:Featuregroup Number:Rescale of 21 : 0 : 9 : 1

	f1-score	precision	recall	support
AFIB	0.658303	0.683884	0.634615	875.0
GSVT	0.707697	0.702471	0.713096	691.0
SB	0.777705	0.773698	0.781759	1553.0
SR	0.731891	0.717935	0.746480	907.0
macro avg	0.718899	0.719497	0.718987	4026.0
micro avg	0.730046	0.730046	0.730046	4026.0
weighted avg	0.729418	0.729391	0.730046	4026.0

The result of Model Number:Dataset Number:Featuregroup Number:Rescale of 21 : 0 : 10 : 0

	f1-score	precision	recall	support
AFIB	0.757461	0.758653	0.756370	204.0
GSVT	0.775779	0.774569	0.777059	300.0
SB	0.791783	0.790817	0.792757	892.0

SR 0.786238 0.787747 0.784738 681.0
macro avg 0.777815 0.777947 0.777731 2077.0
micro avg 0.784287 0.784287 0.784287 2077.0
weighted avg 0.784282 0.784305 0.784287 2077.0
The result of Model Number:Dataset Number:Featuregroup Number:Rescale of 21 : 0 : 10 : 1

	f1-score	precision	recall	support
AFIB	0.815084	0.822375	0.808022	875.0
GSVT	0.830651	0.814856	0.847229	691.0
SB	0.881666	0.874028	0.889442	1553.0
SR	0.845454	0.864893	0.826907	907.0
macro avg	0.843214	0.844038	0.842900	4026.0
micro avg	0.850413	0.850413	0.850413	4026.0
weighted avg	0.850281	0.850588	0.850413	4026.0

The result of Model Number:Dataset Number:Featuregroup Number:Rescale of 21 : 1 : 0 : 0

	f1-score	precision	recall	support
AFIB	0.750489	0.721798	0.797697	875.0
GSVT	0.777634	0.750795	0.814036	691.0
SB	0.866428	0.851135	0.883233	1553.0
SR	0.733845	0.882406	0.633576	907.0
macro avg	0.782099	0.801534	0.782136	4026.0
micro avg	0.796522	0.796522	0.796522	4026.0
weighted avg	0.796121	0.812848	0.796522	4026.0

The result of Model Number:Dataset Number:Featuregroup Number:Rescale of 21 : 1 : 0 : 1

	f1-score	precision	recall	support
AFIB	0.737622	0.772938	0.705567	204.0
GSVT	0.776915	0.768598	0.785443	300.0
SB	0.793418	0.789035	0.797860	892.0
SR	0.784455	0.783775	0.785150	681.0
macro avg	0.773103	0.778586	0.768505	2077.0
micro avg	0.782834	0.782834	0.782834	2077.0
weighted avg	0.782615	0.782777	0.782834	2077.0

The result of Model Number:Dataset Number:Featuregroup Number:Rescale of 21 : 1 : 1 : 0

	f1-score	precision	recall	support
AFIB	0.714118	0.782852	0.656723	204.0
GSVT	0.830521	0.821988	0.839389	300.0
SB	0.880416	0.876992	0.883870	892.0
SR	0.860018	0.848125	0.872263	681.0
macro avg	0.821268	0.832489	0.813061	2077.0
micro avg	0.851329	0.851329	0.851329	2077.0
weighted avg	0.850188	0.850336	0.851329	2077.0

The result of Model Number:Dataset Number:Featuregroup Number:Rescale of 21 : 1 : 1 : 1

	f1-score	precision	recall	support
AFIB	0.679714	0.685762	0.673808	875.0
GSVT	0.693981	0.686365	0.701812	691.0
SB	0.746373	0.740562	0.752282	1553.0
SR	0.721570	0.731797	0.711627	907.0
macro avg	0.710409	0.711122	0.709882	4026.0
micro avg	0.717405	0.717405	0.717405	4026.0
weighted avg	0.717305	0.717375	0.717405	4026.0

The result of Model Number:Dataset Number:Featuregroup Number:Rescale of 21 : 1 : 2 : 0

	f1-score	precision	recall	support
AFIB	0.645601	0.630417	0.679677	875.0
GSVT	0.620364	0.614645	0.634501	691.0
SB	0.672827	0.625452	0.728742	1553.0
SR	0.419516	0.526141	0.349365	907.0
macro avg	0.589577	0.599164	0.598071	4026.0
micro avg	0.616435	0.616435	0.616435	4026.0
weighted avg	0.600838	0.602303	0.616435	4026.0

The result of Model Number:Dataset Number:Featuregroup Number:Rescale of 21 : 1 : 2 : 1

	f1-score	precision	recall	support
AFIB	0.821614	0.816699	0.826700	875.0
GSVT	0.828342	0.832815	0.824038	691.0
SB	0.877919	0.875352	0.880514	1553.0
SR	0.858081	0.864245	0.852022	907.0
macro avg	0.846489	0.847278	0.845818	4026.0
micro avg	0.852706	0.852706	0.852706	4026.0
weighted avg	0.852704	0.852802	0.852706	4026.0

The result of Model Number:Dataset Number:Featuregroup Number:Rescale of 21 : 1 : 3 : 0

	f1-score	precision	recall	support
AFIB	0.651999	0.671919	0.633300	875.0
GSVT	0.691912	0.689568	0.694332	691.0
SB	0.756082	0.753045	0.759148	1553.0
SR	0.716094	0.703674	0.729032	907.0
macro avg	0.704022	0.704551	0.703953	4026.0
micro avg	0.713887	0.713887	0.713887	4026.0
weighted avg	0.713439	0.713396	0.713887	4026.0

The result of Model Number:Dataset Number:Featuregroup Number:Rescale of 21 : 1 : 3 : 1

	f1-score	precision	recall	support
AFIB	0.728180	0.717215	0.745547	875.0

GSVT 0.695280 0.693256 0.701211 691.0
SB 0.742470 0.691863 0.807238 1553.0
SR 0.549993 0.735178 0.455736 907.0
macro avg 0.678981 0.709378 0.677433 4026.0
micro avg 0.696444 0.696444 0.696444 4026.0
weighted avg 0.687902 0.707370 0.696444 4026.0
The result of Model Number:Dataset Number:Featuregroup Number:Rescale of 21 : 1 : 4 : 0

	f1-score	precision	recall	support
AFIB	0.789087	0.798313	0.780223	204.0
GSVT	0.810794	0.807066	0.814579	300.0
SB	0.822274	0.819717	0.824852	892.0
SR	0.815192	0.817503	0.812929	681.0
macro avg	0.809337	0.810650	0.808146	2077.0
micro avg	0.815076	0.815076	0.815076	2077.0
weighted avg	0.815034	0.815061	0.815076	2077.0

The result of Model Number:Dataset Number:Featuregroup Number:Rescale of 21 : 1 : 4 : 1

	f1-score	precision	recall	support
AFIB	0.703834	0.711325	0.696888	204.0
GSVT	0.724518	0.719690	0.729623	300.0
SB	0.739829	0.739167	0.740492	892.0
SR	0.733590	0.734569	0.732619	681.0
macro avg	0.725443	0.726188	0.724905	2077.0
micro avg	0.732058	0.732058	0.732058	2077.0
weighted avg	0.732036	0.732112	0.732058	2077.0

The result of Model Number:Dataset Number:Featuregroup Number:Rescale of 21 : 1 : 5 : 0

	f1-score	precision	recall	support
AFIB	0.692671	0.710805	0.675755	204.0
GSVT	0.651755	0.670698	0.634209	300.0
SB	0.687741	0.663527	0.713802	892.0
SR	0.623353	0.643654	0.604420	681.0
macro avg	0.663880	0.672171	0.657046	2077.0
micro avg	0.662705	0.662705	0.662705	2077.0
weighted avg	0.661916	0.662690	0.662705	2077.0

The result of Model Number:Dataset Number:Featuregroup Number:Rescale of 21 : 1 : 5 : 1

	f1-score	precision	recall	support
AFIB	0.911785	0.941404	0.884146	204.0
GSVT	0.929070	0.920753	0.937723	300.0
SB	0.951162	0.944643	0.957773	892.0
SR	0.935650	0.939597	0.931790	681.0
macro avg	0.931917	0.936599	0.927858	2077.0

micro avg 0.939126 0.939126 0.939126 2077.0
weighted avg 0.939017 0.939220 0.939126 2077.0
The result of Model Number:Dataset Number:Featuregroup Number:Rescale of 21 : 1 : 6 : 0

	f1-score	precision	recall	support
AFIB	0.702680	0.724803	0.682039	204.0
GSVT	0.667583	0.681843	0.654019	300.0
SB	0.689294	0.665174	0.715255	892.0
SR	0.629500	0.650775	0.609617	681.0
macro avg	0.672264	0.680649	0.665232	2077.0
micro avg	0.668511	0.668511	0.668511	2077.0
weighted avg	0.667868	0.668717	0.668511	2077.0

The result of Model Number:Dataset Number:Featuregroup Number:Rescale of 21 : 1 : 6 : 1

	f1-score	precision	recall	support
AFIB	0.807461	0.816248	0.799098	204.0
GSVT	0.834787	0.831306	0.838433	300.0
SB	0.851893	0.851131	0.852657	892.0
SR	0.843842	0.843850	0.843843	681.0
macro avg	0.834496	0.835634	0.833507	2077.0
micro avg	0.842452	0.842452	0.842452	2077.0
weighted avg	0.842419	0.842454	0.842452	2077.0

The result of Model Number:Dataset Number:Featuregroup Number:Rescale of 21 : 1 : 7 : 0

	f1-score	precision	recall	support
AFIB	0.568116	0.564538	0.639943	204.0
GSVT	0.692543	0.688981	0.696259	300.0
SB	0.712262	0.708873	0.715691	892.0
SR	0.558689	0.560530	0.557197	681.0
macro avg	0.632902	0.630730	0.652273	2077.0
micro avg	0.653478	0.653478	0.653478	2077.0
weighted avg	0.644903	0.643185	0.653478	2077.0

The result of Model Number:Dataset Number:Featuregroup Number:Rescale of 21 : 1 : 7 : 1

	f1-score	precision	recall	support
AFIB	0.719516	0.698787	0.789448	875.0
GSVT	0.793004	0.790471	0.795655	691.0
SB	0.669412	0.659546	0.679631	1553.0
SR	0.801540	0.817185	0.786584	907.0
macro avg	0.745868	0.741497	0.762830	4026.0
micro avg	0.747507	0.747507	0.747507	4026.0
weighted avg	0.731281	0.726060	0.747507	4026.0

The result of Model Number:Dataset Number:Featuregroup Number:Rescale of 21 : 1 : 8 : 0

	f1-score	precision	recall	support
AFIB	0.883159	0.900173	0.867193	204.0
GSVT	0.899743	0.885124	0.915017	300.0
SB	0.914764	0.903854	0.925943	892.0
SR	0.897747	0.914579	0.881539	681.0
macro avg	0.898853	0.900932	0.897423	2077.0
micro avg	0.904035	0.904035	0.904035	2077.0
weighted avg	0.903911	0.904304	0.904035	2077.0

The result of Model Number:Dataset Number:Featuregroup Number:Rescale of 21 : 1 : 8 : 1

	f1-score	precision	recall	support
AFIB	0.780087	0.795335	0.765549	204.0
GSVT	0.801522	0.795747	0.807408	300.0
SB	0.815867	0.812692	0.819068	892.0
SR	0.806949	0.809232	0.804696	681.0
macro avg	0.801106	0.803251	0.799180	2077.0
micro avg	0.807415	0.807415	0.807415	2077.0
weighted avg	0.807356	0.807405	0.807415	2077.0

The result of Model Number:Dataset Number:Featuregroup Number:Rescale of 21 : 1 : 9 : 0

	f1-score	precision	recall	support
AFIB	0.759733	0.763203	0.756337	875.0
GSVT	0.772399	0.765866	0.779100	691.0
SB	0.816441	0.808104	0.824957	1553.0
SR	0.774304	0.790628	0.758683	907.0
macro avg	0.780719	0.781950	0.779769	4026.0
micro avg	0.787242	0.787242	0.787242	4026.0
weighted avg	0.787064	0.787159	0.787242	4026.0

The result of Model Number:Dataset Number:Featuregroup Number:Rescale of 21 : 1 : 9 : 1

	f1-score	precision	recall	support
AFIB	0.741126	0.744906	0.737407	875.0
GSVT	0.751354	0.747445	0.755369	691.0
SB	0.787536	0.786832	0.788247	1553.0
SR	0.773035	0.773665	0.772437	907.0
macro avg	0.763262	0.763212	0.763365	4026.0
micro avg	0.767993	0.767993	0.767993	4026.0
weighted avg	0.767972	0.767993	0.767993	4026.0

The result of Model Number:Dataset Number:Featuregroup Number:Rescale of 21 : 1 : 10 : 0

	f1-score	precision	recall	support
AFIB	0.697607	0.713397	0.682590	204.0
GSVT	0.716172	0.712665	0.719744	300.0
SB	0.730823	0.728712	0.732951	892.0

SR 0.726127 0.725813 0.726452 681.0
macro avg 0.717682 0.720147 0.715434 2077.0
micro avg 0.723966 0.723966 0.723966 2077.0
weighted avg 0.723905 0.723940 0.723966 2077.0
The result of Model Number:Dataset Number:Featuregroup Number:Rescale of 21 : 1 : 10 : 1
f1-score precision recall support
AFIB 0.711372 0.701928 0.721129 875.0
GSVT 0.725811 0.732692 0.719090 691.0
SB 0.772367 0.770540 0.774206 1553.0
SR 0.751414 0.759315 0.743706 907.0
macro avg 0.740241 0.741119 0.739533 4026.0
micro avg 0.746339 0.746339 0.746339 4026.0
weighted avg 0.746399 0.746603 0.746339 4026.0
The result of Model Number:Dataset Number:Featuregroup Number:Rescale of 22 : 0 : 0 : 0
f1-score precision recall support
AFIB 0.903121 0.911857 0.894714 204.0
GSVT 0.935078 0.933235 0.936945 300.0
SB 0.954623 0.952932 0.956333 892.0
SR 0.945823 0.946280 0.945406 681.0
macro avg 0.934661 0.936076 0.933349 2077.0
micro avg 0.943897 0.943897 0.943897 2077.0
weighted avg 0.943856 0.943872 0.943897 2077.0
The result of Model Number:Dataset Number:Featuregroup Number:Rescale of 22 : 0 : 0 : 1
f1-score precision recall support
AFIB 0.856048 0.861645 0.850575 204.0
GSVT 0.877496 0.875475 0.879537 300.0
SB 0.893159 0.891870 0.894459 892.0
SR 0.886120 0.887046 0.885212 681.0
macro avg 0.878206 0.879009 0.877446 2077.0
micro avg 0.884962 0.884962 0.884962 2077.0
weighted avg 0.884944 0.884952 0.884962 2077.0
The result of Model Number:Dataset Number:Featuregroup Number:Rescale of 22 : 0 : 1 : 0
f1-score precision recall support
AFIB 0.694954 0.696717 0.693224 875.0
GSVT 0.707217 0.699556 0.715077 691.0
SB 0.745368 0.736936 0.754006 1553.0
SR 0.711288 0.730762 0.692855 907.0
macro avg 0.714707 0.715993 0.713790 4026.0
micro avg 0.720338 0.720338 0.720338 4026.0
weighted avg 0.720185 0.720388 0.720338 4026.0

The result of Model Number:Dataset Number:Featuregroup Number:Rescale of 22 : 0 : 1 : 1

	f1-score	precision	recall	support
AFIB	0.787477	0.779556	0.795582	875.0
GSVT	0.794524	0.801598	0.787632	691.0
SB	0.840571	0.837991	0.843168	1553.0
SR	0.824294	0.831509	0.817206	907.0
macro avg	0.811716	0.812664	0.810897	4026.0
micro avg	0.817445	0.817445	0.817445	4026.0
weighted avg	0.817461	0.817584	0.817445	4026.0

The result of Model Number:Dataset Number:Featuregroup Number:Rescale of 22 : 0 : 2 : 0

	f1-score	precision	recall	support
AFIB	0.747030	0.727913	0.767246	875.0
GSVT	0.706174	0.746198	0.670389	691.0
SB	0.748791	0.689068	0.819869	1553.0
SR	0.598158	0.726541	0.508441	907.0
macro avg	0.700038	0.722430	0.691486	4026.0
micro avg	0.712616	0.712616	0.712616	4026.0
weighted avg	0.707158	0.715758	0.712616	4026.0

The result of Model Number:Dataset Number:Featuregroup Number:Rescale of 22 : 0 : 2 : 1

	f1-score	precision	recall	support
AFIB	0.725034	0.717926	0.775206	204.0
GSVT	0.650105	0.642876	0.657660	300.0
SB	0.835425	0.829341	0.841604	892.0
SR	0.821414	0.827103	0.815869	681.0
macro avg	0.757994	0.754312	0.772585	2077.0
micro avg	0.800076	0.800076	0.800076	2077.0
weighted avg	0.793221	0.790731	0.800076	2077.0

The result of Model Number:Dataset Number:Featuregroup Number:Rescale of 22 : 0 : 3 : 0

	f1-score	precision	recall	support
AFIB	0.842594	0.868545	0.818317	204.0
GSVT	0.864089	0.853511	0.875027	300.0
SB	0.887560	0.882286	0.892926	892.0
SR	0.872599	0.876903	0.868371	681.0
macro avg	0.866710	0.870311	0.863660	2077.0
micro avg	0.874962	0.874962	0.874962	2077.0
weighted avg	0.874848	0.875015	0.874962	2077.0

The result of Model Number:Dataset Number:Featuregroup Number:Rescale of 22 : 0 : 3 : 1

	f1-score	precision	recall	support
AFIB	0.720045	0.792691	0.660018	204.0

GSVT 0.849144 0.837423 0.861332 300.0
SB 0.901626 0.898618 0.904658 892.0
SR 0.878536 0.866382 0.891048 681.0
macro avg 0.837338 0.848778 0.829264 2077.0
micro avg 0.869910 0.869910 0.869910 2077.0
weighted avg 0.868640 0.868805 0.869910 2077.0
The result of Model Number:Dataset Number:Featuregroup Number:Rescale of 22 : 0 : 4 : 0

	f1-score	precision	recall	support
AFIB	0.785552	0.774398	0.797120	875.0
GSVT	0.814040	0.824425	0.804037	691.0
SB	0.865101	0.863831	0.866382	1553.0
SR	0.843549	0.849876	0.837317	907.0
macro avg	0.827060	0.828133	0.826214	4026.0
micro avg	0.834080	0.834080	0.834080	4026.0
weighted avg	0.834193	0.834487	0.834080	4026.0

The result of Model Number:Dataset Number:Featuregroup Number:Rescale of 22 : 0 : 4 : 1

	f1-score	precision	recall	support
AFIB	0.785233	0.765139	0.806484	875.0
GSVT	0.742288	0.784359	0.704673	691.0
SB	0.787085	0.724308	0.861797	1553.0
SR	0.628748	0.763697	0.534443	907.0
macro avg	0.735839	0.759376	0.726850	4026.0
micro avg	0.749060	0.749060	0.749060	4026.0
weighted avg	0.743323	0.752363	0.749060	4026.0

The result of Model Number:Dataset Number:Featuregroup Number:Rescale of 22 : 0 : 5 : 0

	f1-score	precision	recall	support
AFIB	0.691247	0.721584	0.663532	204.0
GSVT	0.643506	0.648139	0.639203	300.0
SB	0.667366	0.633853	0.704624	892.0
SR	0.592059	0.631149	0.557653	681.0
macro avg	0.648545	0.658681	0.641253	2077.0
micro avg	0.642950	0.642950	0.642950	2077.0
weighted avg	0.641574	0.643647	0.642950	2077.0

The result of Model Number:Dataset Number:Featuregroup Number:Rescale of 22 : 0 : 5 : 1

	f1-score	precision	recall	support
AFIB	0.725964	0.767262	0.689366	204.0
GSVT	0.767180	0.758732	0.775857	300.0
SB	0.778592	0.770845	0.786523	892.0
SR	0.769547	0.772178	0.767056	681.0
macro avg	0.760321	0.767254	0.754701	2077.0

micro avg 0.769057 0.769057 0.769057 2077.0
weighted avg 0.768809 0.769181 0.769057 2077.0
The result of Model Number:Dataset Number:Featuregroup Number:Rescale of 22 : 0 : 6 : 0

	f1-score	precision	recall	support
AFIB	0.654011	0.646297	0.661944	875.0
GSVT	0.665090	0.669298	0.660958	691.0
SB	0.709114	0.708161	0.710073	1553.0
SR	0.691693	0.698246	0.685286	907.0
macro avg	0.679977	0.680501	0.679565	4026.0
micro avg	0.685599	0.685599	0.685599	4026.0
weighted avg	0.685658	0.685812	0.685599	4026.0

The result of Model Number:Dataset Number:Featuregroup Number:Rescale of 22 : 0 : 6 : 1

	f1-score	precision	recall	support
AFIB	0.691245	0.704757	0.678363	204.0
GSVT	0.710239	0.705122	0.715455	300.0
SB	0.722950	0.720137	0.725787	892.0
SR	0.715048	0.717072	0.713051	681.0
macro avg	0.709871	0.711772	0.708164	2077.0
micro avg	0.715461	0.715461	0.715461	2077.0
weighted avg	0.715409	0.715453	0.715461	2077.0

The result of Model Number:Dataset Number:Featuregroup Number:Rescale of 22 : 0 : 7 : 0

	f1-score	precision	recall	support
AFIB	0.866709	0.886326	0.848052	204.0
GSVT	0.889773	0.885417	0.894212	300.0
SB	0.907976	0.905354	0.910620	892.0
SR	0.902142	0.901752	0.902546	681.0
macro avg	0.891650	0.894712	0.888857	2077.0
micro avg	0.899457	0.899457	0.899457	2077.0
weighted avg	0.899381	0.899424	0.899457	2077.0

The result of Model Number:Dataset Number:Featuregroup Number:Rescale of 22 : 0 : 7 : 1

	f1-score	precision	recall	support
AFIB	0.758557	0.748487	0.768961	875.0
GSVT	0.773953	0.781290	0.766786	691.0
SB	0.823597	0.821649	0.825558	1553.0
SR	0.801254	0.809679	0.793036	907.0
macro avg	0.789340	0.790276	0.788585	4026.0
micro avg	0.795843	0.795843	0.795843	4026.0
weighted avg	0.795907	0.796125	0.795843	4026.0

The result of Model Number:Dataset Number:Featuregroup Number:Rescale of 22 : 0 : 8 : 0

	f1-score	precision	recall	support
AFIB	0.777402	0.855834	0.712593	204.0
GSVT	0.916784	0.904129	0.929944	300.0
SB	0.973446	0.970199	0.976720	892.0
SR	0.948518	0.935395	0.962027	681.0
macro avg	0.904037	0.916389	0.895321	2077.0
micro avg	0.939204	0.939204	0.939204	2077.0
weighted avg	0.937833	0.938012	0.939204	2077.0

The result of Model Number:Dataset Number:Featuregroup Number:Rescale of 22 : 0 : 8 : 1

	f1-score	precision	recall	support
AFIB	0.899853	0.890802	0.909115	875.0
GSVT	0.907906	0.915989	0.900030	691.0
SB	0.960524	0.957576	0.963492	1553.0
SR	0.941924	0.950169	0.933824	907.0
macro avg	0.927552	0.928634	0.926615	4026.0
micro avg	0.934098	0.934098	0.934098	4026.0
weighted avg	0.934117	0.934257	0.934098	4026.0

The result of Model Number:Dataset Number:Featuregroup Number:Rescale of 22 : 0 : 9 : 0

	f1-score	precision	recall	support
AFIB	0.888886	0.911777	0.867151	204.0
GSVT	0.815708	0.779398	0.920526	300.0
SB	0.935852	0.933663	0.938055	892.0
SR	0.742429	0.742569	0.742299	681.0
macro avg	0.845719	0.841852	0.867008	2077.0
micro avg	0.864375	0.864375	0.864375	2077.0
weighted avg	0.850467	0.846576	0.864375	2077.0

The result of Model Number:Dataset Number:Featuregroup Number:Rescale of 22 : 0 : 9 : 1

	f1-score	precision	recall	support
AFIB	0.828029	0.841795	0.814896	204.0
GSVT	0.843907	0.837911	0.850051	300.0
SB	0.863481	0.858628	0.868408	892.0
SR	0.852480	0.857695	0.847355	681.0
macro avg	0.846974	0.849007	0.845178	2077.0
micro avg	0.853598	0.853598	0.853598	2077.0
weighted avg	0.853564	0.853677	0.853598	2077.0

The result of Model Number:Dataset Number:Featuregroup Number:Rescale of 22 : 0 : 10 : 0

	f1-score	precision	recall	support
AFIB	0.844601	0.836773	0.852624	875.0
GSVT	0.859769	0.864438	0.855165	691.0
SB	0.910974	0.908874	0.913085	1553.0

SR 0.889750 0.897960 0.881716 907.0
macro avg 0.876274 0.877012 0.875647 4026.0
micro avg 0.882936 0.882936 0.882936 4026.0
weighted avg 0.882979 0.883119 0.882936 4026.0
The result of Model Number:Dataset Number:Featuregroup Number:Rescale of 22 : 0 : 10 : 1
f1-score precision recall support
AFIB 0.670012 0.695526 0.646450 204.0
GSVT 0.691359 0.684391 0.698501 300.0
SB 0.705197 0.701686 0.708753 892.0
SR 0.696679 0.697117 0.696264 681.0
macro avg 0.690812 0.694680 0.687492 2077.0
micro avg 0.697058 0.697058 0.697058 2077.0
weighted avg 0.696949 0.697085 0.697058 2077.0
The result of Model Number:Dataset Number:Featuregroup Number:Rescale of 22 : 1 : 0 : 0
f1-score precision recall support
AFIB 0.760006 0.759097 0.761046 875.0
GSVT 0.774821 0.777487 0.772254 691.0
SB 0.820651 0.815596 0.825776 1553.0
SR 0.803951 0.811803 0.796338 907.0
macro avg 0.789857 0.790996 0.788854 4026.0
micro avg 0.795890 0.795890 0.795890 4026.0
weighted avg 0.795842 0.795921 0.795890 4026.0
The result of Model Number:Dataset Number:Featuregroup Number:Rescale of 22 : 1 : 0 : 1
f1-score precision recall support
AFIB 0.673817 0.648057 0.716202 875.0
GSVT 0.698188 0.674092 0.730872 691.0
SB 0.777911 0.764180 0.792999 1553.0
SR 0.658873 0.792257 0.568848 907.0
macro avg 0.702197 0.719646 0.702230 4026.0
micro avg 0.715147 0.715147 0.715147 4026.0
weighted avg 0.714787 0.729805 0.715147 4026.0
The result of Model Number:Dataset Number:Featuregroup Number:Rescale of 22 : 1 : 1 : 0
f1-score precision recall support
AFIB 0.851547 0.833254 0.870708 875.0
GSVT 0.881867 0.894656 0.869497 691.0
SB 0.938782 0.938603 0.938964 1553.0
SR 0.915253 0.925891 0.904862 907.0
macro avg 0.896862 0.898101 0.896008 4026.0
micro avg 0.904524 0.904524 0.904524 4026.0
weighted avg 0.904753 0.905300 0.904524 4026.0

The result of Model Number:Dataset Number:Featuregroup Number:Rescale of 22 : 1 : 1 : 1

	f1-score	precision	recall	support
AFIB	0.837411	0.839491	0.835398	875.0
GSVT	0.851833	0.854392	0.849396	691.0
SB	0.903641	0.897507	0.909865	1553.0
SR	0.886445	0.893026	0.879995	907.0
macro avg	0.869833	0.871104	0.868664	4026.0
micro avg	0.876573	0.876573	0.876573	4026.0
weighted avg	0.876481	0.876489	0.876573	4026.0

The result of Model Number:Dataset Number:Featuregroup Number:Rescale of 22 : 1 : 2 : 0

	f1-score	precision	recall	support
AFIB	0.846679	0.845904	0.847557	875.0
GSVT	0.864034	0.862358	0.865807	691.0
SB	0.916852	0.914810	0.918918	1553.0
SR	0.891827	0.897646	0.886117	907.0
macro avg	0.879848	0.880179	0.879600	4026.0
micro avg	0.886903	0.886903	0.886903	4026.0
weighted avg	0.886898	0.886965	0.886903	4026.0

The result of Model Number:Dataset Number:Featuregroup Number:Rescale of 22 : 1 : 2 : 1

	f1-score	precision	recall	support
AFIB	0.798628	0.809295	0.788463	204.0
GSVT	0.812736	0.807785	0.817872	300.0
SB	0.832327	0.831589	0.833072	892.0
SR	0.826177	0.826302	0.826057	681.0
macro avg	0.817467	0.818743	0.816366	2077.0
micro avg	0.824195	0.824195	0.824195	2077.0
weighted avg	0.824171	0.824228	0.824195	2077.0

The result of Model Number:Dataset Number:Featuregroup Number:Rescale of 22 : 1 : 3 : 0

	f1-score	precision	recall	support
AFIB	0.739760	0.793230	0.693553	204.0
GSVT	0.700706	0.704496	0.697243	300.0
SB	0.731901	0.688014	0.781786	892.0
SR	0.642664	0.693579	0.598796	681.0
macro avg	0.703758	0.719830	0.692844	2077.0
micro avg	0.700910	0.700910	0.700910	2077.0
weighted avg	0.698909	0.702554	0.700910	2077.0

The result of Model Number:Dataset Number:Featuregroup Number:Rescale of 22 : 1 : 3 : 1

	f1-score	precision	recall	support
AFIB	0.739637	0.740962	0.738341	875.0

GSVT	0.708252	0.711973	0.704590	691.0
SB	0.749159	0.707948	0.795471	1553.0
SR	0.622180	0.700309	0.559789	907.0
macro avg	0.704807	0.715298	0.699548	4026.0
micro avg	0.714360	0.714360	0.714360	4026.0
weighted avg	0.711462	0.714093	0.714360	4026.0

The result of Model Number:Dataset Number:Featuregroup Number:Rescale of 22 : 1 : 4 : 0

	f1-score	precision	recall	support
AFIB	0.867532	0.855516	0.879926	875.0
GSVT	0.886774	0.893951	0.879716	691.0
SB	0.941300	0.938595	0.944026	1553.0
SR	0.918401	0.930354	0.906760	907.0
macro avg	0.903502	0.904604	0.902607	4026.0
micro avg	0.910661	0.910661	0.910661	4026.0
weighted avg	0.910750	0.911020	0.910661	4026.0

The result of Model Number:Dataset Number:Featuregroup Number:Rescale of 22 : 1 : 4 : 1

	f1-score	precision	recall	support
AFIB	0.848856	0.858565	0.839693	204.0
GSVT	0.868725	0.863585	0.873942	300.0
SB	0.833960	0.803155	0.885959	892.0
SR	0.699561	0.702947	0.696226	681.0
macro avg	0.812776	0.807063	0.823955	2077.0
micro avg	0.817470	0.817470	0.817470	2077.0
weighted avg	0.796379	0.784470	0.817470	2077.0

The result of Model Number:Dataset Number:Featuregroup Number:Rescale of 22 : 1 : 5 : 0

	f1-score	precision	recall	support
AFIB	0.815688	0.804358	0.827420	875.0
GSVT	0.844837	0.856107	0.833969	691.0
SB	0.897491	0.895652	0.899340	1553.0
SR	0.874132	0.880886	0.867481	907.0
macro avg	0.858037	0.859251	0.857053	4026.0
micro avg	0.865312	0.865312	0.865312	4026.0
weighted avg	0.865412	0.865697	0.865312	4026.0

The result of Model Number:Dataset Number:Featuregroup Number:Rescale of 22 : 1 : 5 : 1

	f1-score	precision	recall	support
AFIB	0.823006	0.840269	0.806460	204.0
GSVT	0.842098	0.837116	0.847149	300.0
SB	0.862628	0.860607	0.864658	892.0
SR	0.855341	0.855095	0.855595	681.0
macro avg	0.845768	0.848272	0.843465	2077.0

micro avg 0.853441 0.853441 0.853441 2077.0
weighted avg 0.853382 0.853409 0.853441 2077.0
The result of Model Number:Dataset Number:Featuregroup Number:Rescale of 22 : 1 : 6 : 0

	f1-score	precision	recall	support
AFIB	0.779538	0.832765	0.733040	204.0
GSVT	0.824050	0.807257	0.841627	300.0
SB	0.844489	0.837085	0.852056	892.0
SR	0.823658	0.826401	0.821007	681.0
macro avg	0.817934	0.825877	0.811933	2077.0
micro avg	0.828680	0.828680	0.828680	2077.0
weighted avg	0.828327	0.828850	0.828680	2077.0

The result of Model Number:Dataset Number:Featuregroup Number:Rescale of 22 : 1 : 6 : 1

	f1-score	precision	recall	support
AFIB	0.683901	0.714296	0.656082	204.0
GSVT	0.712400	0.706808	0.718098	300.0
SB	0.723283	0.717703	0.728956	892.0
SR	0.715281	0.716462	0.714129	681.0
macro avg	0.708716	0.713817	0.704316	2077.0
micro avg	0.715369	0.715369	0.715369	2077.0
weighted avg	0.715219	0.715388	0.715369	2077.0

The result of Model Number:Dataset Number:Featuregroup Number:Rescale of 22 : 1 : 7 : 0

	f1-score	precision	recall	support
AFIB	0.832324	0.897196	0.776587	204.0
GSVT	0.857706	0.872225	0.843699	300.0
SB	0.909007	0.900641	0.917558	892.0
SR	0.872828	0.860171	0.885903	681.0
macro avg	0.867966	0.882558	0.855937	2077.0
micro avg	0.882665	0.882665	0.882665	2077.0
weighted avg	0.882203	0.882929	0.882665	2077.0

The result of Model Number:Dataset Number:Featuregroup Number:Rescale of 22 : 1 : 7 : 1

	f1-score	precision	recall	support
AFIB	0.571943	0.634740	0.520590	204.0
GSVT	0.667821	0.659472	0.676515	300.0
SB	0.710529	0.706903	0.714192	892.0
SR	0.693884	0.684183	0.703867	681.0
macro avg	0.661044	0.671324	0.653791	2077.0
micro avg	0.686349	0.686349	0.686349	2077.0
weighted avg	0.685291	0.685515	0.686349	2077.0

The result of Model Number:Dataset Number:Featuregroup Number:Rescale of 22 : 1 : 8 : 0

	f1-score	precision	recall	support
AFIB	0.863408	0.867813	0.859076	875.0
GSVT	0.875324	0.870770	0.880002	691.0
SB	0.917476	0.916656	0.918304	1553.0
SR	0.900582	0.901316	0.899886	907.0
macro avg	0.889198	0.889139	0.889317	4026.0
micro avg	0.894708	0.894708	0.894708	4026.0
weighted avg	0.894684	0.894709	0.894708	4026.0

The result of Model Number:Dataset Number:Featuregroup Number:Rescale of 22 : 1 : 8 : 1

	f1-score	precision	recall	support
AFIB	0.920468	0.939775	0.901962	204.0
GSVT	0.941821	0.936248	0.947469	300.0
SB	0.964781	0.962522	0.967052	892.0
SR	0.956632	0.956357	0.956916	681.0
macro avg	0.945925	0.948726	0.943350	2077.0
micro avg	0.954507	0.954507	0.954507	2077.0
weighted avg	0.954441	0.954472	0.954507	2077.0

The result of Model Number:Dataset Number:Featuregroup Number:Rescale of 22 : 1 : 9 : 0

	f1-score	precision	recall	support
AFIB	0.637302	0.632197	0.642565	875.0
GSVT	0.654902	0.656020	0.653865	691.0
SB	0.694300	0.692439	0.696177	1553.0
SR	0.675330	0.683191	0.667662	907.0
macro avg	0.665459	0.665962	0.665067	4026.0
micro avg	0.670839	0.670839	0.670839	4026.0
weighted avg	0.670877	0.671012	0.670839	4026.0

The result of Model Number:Dataset Number:Featuregroup Number:Rescale of 22 : 1 : 9 : 1

	f1-score	precision	recall	support
AFIB	0.792623	0.795299	0.789992	875.0
GSVT	0.790529	0.784961	0.796249	691.0
SB	0.831782	0.824062	0.839654	1553.0
SR	0.800160	0.815580	0.785351	907.0
macro avg	0.803773	0.804976	0.802811	4026.0
micro avg	0.809177	0.809177	0.809177	4026.0
weighted avg	0.809067	0.809189	0.809177	4026.0

The result of Model Number:Dataset Number:Featuregroup Number:Rescale of 22 : 1 : 10 : 0

	f1-score	precision	recall	support
AFIB	0.725650	0.731979	0.719611	204.0
GSVT	0.750745	0.749520	0.751993	300.0
SB	0.766703	0.764833	0.768591	892.0

SR 0.759674 0.760819 0.758558 681.0
macro avg 0.750693 0.751787 0.749688 2077.0
micro avg 0.758093 0.758093 0.758093 2077.0
weighted avg 0.758061 0.758078 0.758093 2077.0
The result of Model Number:Dataset Number:Featuregroup Number:Rescale of 22 : 1 : 10 : 1

	f1-score	precision	recall	support
AFIB	0.592576	0.654557	0.541491	204.0
GSVT	0.697125	0.687920	0.706653	300.0
SB	0.739298	0.736425	0.742198	892.0
SR	0.721533	0.711250	0.732126	681.0
macro avg	0.687633	0.697538	0.680617	2077.0
micro avg	0.714048	0.714048	0.714048	2077.0
weighted avg	0.712971	0.713124	0.714048	2077.0

The result of Model Number:Dataset Number:Featuregroup Number:Rescale of 23 : 0 : 0 : 0

	f1-score	precision	recall	support
AFIB	0.836724	0.891242	0.788862	204.0
GSVT	0.873379	0.851912	0.896076	300.0
SB	0.898993	0.887445	0.910878	892.0
SR	0.875643	0.886099	0.865489	681.0
macro avg	0.871185	0.879174	0.865326	2077.0
micro avg	0.881874	0.881874	0.881874	2077.0
weighted avg	0.881522	0.882244	0.881874	2077.0

The result of Model Number:Dataset Number:Featuregroup Number:Rescale of 23 : 0 : 0 : 1

	f1-score	precision	recall	support
AFIB	0.669673	0.675778	0.663765	204.0
GSVT	0.687149	0.684900	0.689435	300.0
SB	0.699711	0.699245	0.700181	892.0
SR	0.693557	0.693358	0.693767	681.0
macro avg	0.687523	0.688320	0.686787	2077.0
micro avg	0.692949	0.692949	0.692949	2077.0
weighted avg	0.692929	0.692938	0.692949	2077.0

The result of Model Number:Dataset Number:Featuregroup Number:Rescale of 23 : 0 : 1 : 0

	f1-score	precision	recall	support
AFIB	0.827328	0.816688	0.838308	875.0
GSVT	0.844170	0.851159	0.837329	691.0
SB	0.902587	0.898570	0.906651	1553.0
SR	0.876902	0.889871	0.864326	907.0
macro avg	0.862747	0.864072	0.861654	4026.0
micro avg	0.870364	0.870364	0.870364	4026.0
weighted avg	0.870418	0.870677	0.870364	4026.0

The result of Model Number:Dataset Number:Featuregroup Number:Rescale of 23 : 0 : 1 : 1

	f1-score	precision	recall	support
AFIB	0.845408	0.863782	0.828031	204.0
GSVT	0.880306	0.875113	0.885585	300.0
SB	0.899219	0.897224	0.901232	892.0
SR	0.890204	0.889702	0.890729	681.0
macro avg	0.878784	0.881455	0.876394	2077.0
micro avg	0.888339	0.888339	0.888339	2077.0
weighted avg	0.888246	0.888280	0.888339	2077.0

The result of Model Number:Dataset Number:Featuregroup Number:Rescale of 23 : 0 : 2 : 0

	f1-score	precision	recall	support
AFIB	0.865217	0.901810	0.831765	204.0
GSVT	0.796802	0.798197	0.807364	300.0
SB	0.857831	0.816964	0.903833	892.0
SR	0.758364	0.819445	0.707130	681.0
macro avg	0.819553	0.834104	0.812523	2077.0
micro avg	0.818326	0.818326	0.818326	2077.0
weighted avg	0.817128	0.823400	0.818326	2077.0

The result of Model Number:Dataset Number:Featuregroup Number:Rescale of 23 : 0 : 2 : 1

	f1-score	precision	recall	support
AFIB	0.831264	0.851010	0.812490	204.0
GSVT	0.858569	0.850519	0.866863	300.0
SB	0.877007	0.876027	0.877991	892.0
SR	0.868543	0.867670	0.869431	681.0
macro avg	0.858846	0.861306	0.856694	2077.0
micro avg	0.867144	0.867144	0.867144	2077.0
weighted avg	0.867076	0.867145	0.867144	2077.0

The result of Model Number:Dataset Number:Featuregroup Number:Rescale of 23 : 0 : 3 : 0

	f1-score	precision	recall	support
AFIB	0.718734	0.725163	0.712507	875.0
GSVT	0.732461	0.718533	0.747079	691.0
SB	0.777446	0.770711	0.784303	1553.0
SR	0.745514	0.762656	0.729160	907.0
macro avg	0.743539	0.744266	0.743262	4026.0
micro avg	0.749887	0.749887	0.749887	4026.0
weighted avg	0.749771	0.750041	0.749887	4026.0

The result of Model Number:Dataset Number:Featuregroup Number:Rescale of 23 : 0 : 3 : 1

	f1-score	precision	recall	support
AFIB	0.641421	0.633232	0.649862	875.0

GSVT 0.652679 0.656422 0.648997 691.0
SB 0.698653 0.695719 0.701619 1553.0
SR 0.678081 0.689057 0.667462 907.0
macro avg 0.667709 0.668607 0.666985 4026.0
micro avg 0.673643 0.673643 0.673643 4026.0
weighted avg 0.673689 0.673893 0.673643 4026.0

The result of Model Number:Dataset Number:Featuregroup Number:Rescale of 23 : 0 : 4 : 0

	f1-score	precision	recall	support
AFIB	0.804819	0.809624	0.800094	204.0
GSVT	0.824595	0.821054	0.828167	300.0
SB	0.838717	0.837502	0.839938	892.0
SR	0.831048	0.832765	0.829342	681.0
macro avg	0.824795	0.825236	0.824385	2077.0
micro avg	0.830850	0.830850	0.830850	2077.0
weighted avg	0.830833	0.830835	0.830850	2077.0

The result of Model Number:Dataset Number:Featuregroup Number:Rescale of 23 : 0 : 4 : 1

	f1-score	precision	recall	support
AFIB	0.767351	0.765224	0.769547	875.0
GSVT	0.773424	0.777112	0.769847	691.0
SB	0.809639	0.807052	0.812245	1553.0
SR	0.796695	0.800543	0.792921	907.0
macro avg	0.786777	0.787483	0.786140	4026.0
micro avg	0.791335	0.791335	0.791335	4026.0
weighted avg	0.791316	0.791356	0.791335	4026.0

The result of Model Number:Dataset Number:Featuregroup Number:Rescale of 23 : 0 : 5 : 0

	f1-score	precision	recall	support
AFIB	0.853699	0.871679	0.836580	204.0
GSVT	0.880241	0.872200	0.888453	300.0
SB	0.894617	0.892623	0.896623	892.0
SR	0.883986	0.884910	0.883073	681.0
macro avg	0.878136	0.880353	0.876182	2077.0
micro avg	0.885103	0.885103	0.885103	2077.0
weighted avg	0.885036	0.885087	0.885103	2077.0

The result of Model Number:Dataset Number:Featuregroup Number:Rescale of 23 : 0 : 5 : 1

	f1-score	precision	recall	support
AFIB	0.706421	0.727844	0.686252	875.0
GSVT	0.754114	0.748885	0.759458	691.0
SB	0.825093	0.821937	0.828279	1553.0
SR	0.780339	0.768627	0.792459	907.0
macro avg	0.766492	0.766823	0.766612	4026.0

micro avg 0.777530 0.777530 0.777530 4026.0
weighted avg 0.777036 0.776939 0.777530 4026.0
The result of Model Number:Dataset Number:Featuregroup Number:Rescale of 23 : 0 : 6 : 0

	f1-score	precision	recall	support
AFIB	0.664532	0.661830	0.667273	875.0
GSVT	0.674245	0.678321	0.670244	691.0
SB	0.711119	0.710253	0.711989	1553.0
SR	0.696283	0.697386	0.695204	907.0
macro avg	0.686545	0.686948	0.686178	4026.0
micro avg	0.691324	0.691324	0.691324	4026.0
weighted avg	0.691323	0.691349	0.691324	4026.0

The result of Model Number:Dataset Number:Featuregroup Number:Rescale of 23 : 0 : 6 : 1

	f1-score	precision	recall	support
AFIB	0.874316	0.880206	0.868633	875.0
GSVT	0.889919	0.877559	0.902797	691.0
SB	0.948306	0.941812	0.954898	1553.0
SR	0.909876	0.925593	0.894734	907.0
macro avg	0.905604	0.906293	0.905265	4026.0
micro avg	0.913653	0.913653	0.913653	4026.0
weighted avg	0.913546	0.913741	0.913653	4026.0

The result of Model Number:Dataset Number:Featuregroup Number:Rescale of 23 : 0 : 7 : 0

	f1-score	precision	recall	support
AFIB	0.621223	0.635380	0.607783	875.0
GSVT	0.661061	0.662368	0.659980	691.0
SB	0.723245	0.719286	0.727249	1553.0
SR	0.683575	0.675283	0.692168	907.0
macro avg	0.672276	0.673079	0.671795	4026.0
micro avg	0.681836	0.681836	0.681836	4026.0
weighted avg	0.681462	0.681368	0.681836	4026.0

The result of Model Number:Dataset Number:Featuregroup Number:Rescale of 23 : 0 : 7 : 1

	f1-score	precision	recall	support
AFIB	0.799693	0.820629	0.780164	204.0
GSVT	0.752456	0.774325	0.732198	300.0
SB	0.794002	0.766047	0.824089	892.0
SR	0.719666	0.743103	0.697807	681.0
macro avg	0.766454	0.776026	0.758565	2077.0
micro avg	0.765097	0.765097	0.765097	2077.0
weighted avg	0.764187	0.765081	0.765097	2077.0

The result of Model Number:Dataset Number:Featuregroup Number:Rescale of 23 : 0 : 8 : 0

	f1-score	precision	recall	support
AFIB	0.845162	0.868946	0.822818	204.0
GSVT	0.867012	0.860926	0.873348	300.0
SB	0.890854	0.887290	0.894449	892.0
SR	0.879013	0.879563	0.878491	681.0
macro avg	0.870510	0.874181	0.867276	2077.0
micro avg	0.879133	0.879133	0.879133	2077.0
weighted avg	0.879040	0.879147	0.879133	2077.0

The result of Model Number:Dataset Number:Featuregroup Number:Rescale of 23 : 0 : 8 : 1

	f1-score	precision	recall	support
AFIB	0.735072	0.730265	0.740007	875.0
GSVT	0.756920	0.763360	0.750688	691.0
SB	0.805843	0.804088	0.807608	1553.0
SR	0.786204	0.789361	0.783084	907.0
macro avg	0.771010	0.771769	0.770347	4026.0
micro avg	0.777621	0.777621	0.777621	4026.0
weighted avg	0.777641	0.777736	0.777621	4026.0

The result of Model Number:Dataset Number:Featuregroup Number:Rescale of 23 : 0 : 9 : 0

	f1-score	precision	recall	support
AFIB	0.680265	0.698651	0.662963	204.0
GSVT	0.685872	0.675776	0.696369	300.0
SB	0.699158	0.691720	0.706760	892.0
SR	0.686190	0.695619	0.677025	681.0
macro avg	0.687871	0.690442	0.685779	2077.0
micro avg	0.691208	0.691208	0.691208	2077.0
weighted avg	0.691132	0.691376	0.691208	2077.0

The result of Model Number:Dataset Number:Featuregroup Number:Rescale of 23 : 0 : 9 : 1

	f1-score	precision	recall	support
AFIB	0.833374	0.842442	0.824742	204.0
GSVT	0.861576	0.857983	0.865339	300.0
SB	0.879232	0.878445	0.880020	892.0
SR	0.870922	0.870930	0.870922	681.0
macro avg	0.861276	0.862450	0.860256	2077.0
micro avg	0.869487	0.869487	0.869487	2077.0
weighted avg	0.869453	0.869489	0.869487	2077.0

The result of Model Number:Dataset Number:Featuregroup Number:Rescale of 23 : 0 : 10 : 0

	f1-score	precision	recall	support
AFIB	0.838628	0.835217	0.842087	875.0
GSVT	0.850886	0.856030	0.845837	691.0
SB	0.897419	0.896327	0.898518	1553.0

SR 0.878698 0.880090 0.877336 907.0
macro avg 0.866408 0.866916 0.865944 4026.0
micro avg 0.872439 0.872439 0.872439 4026.0
weighted avg 0.872437 0.872471 0.872439 4026.0
The result of Model Number:Dataset Number:Featuregroup Number:Rescale of 23 : 0 : 10 : 1

	f1-score	precision	recall	support
AFIB	0.821403	0.829801	0.813242	875.0
GSVT	0.821156	0.808470	0.834342	691.0
SB	0.866209	0.861037	0.871452	1553.0
SR	0.843282	0.854569	0.832315	907.0
macro avg	0.838013	0.838469	0.837838	4026.0
micro avg	0.843614	0.843614	0.843614	4026.0
weighted avg	0.843573	0.843769	0.843614	4026.0

The result of Model Number:Dataset Number:Featuregroup Number:Rescale of 23 : 1 : 0 : 0

	f1-score	precision	recall	support
AFIB	0.705024	0.706466	0.703663	204.0
GSVT	0.720530	0.717730	0.723403	300.0
SB	0.736526	0.735543	0.737515	892.0
SR	0.731116	0.733277	0.728970	681.0
macro avg	0.723299	0.723254	0.723388	2077.0
micro avg	0.729350	0.729350	0.729350	2077.0
weighted avg	0.729348	0.729371	0.729350	2077.0

The result of Model Number:Dataset Number:Featuregroup Number:Rescale of 23 : 1 : 0 : 1

	f1-score	precision	recall	support
AFIB	0.830792	0.873713	0.792137	204.0
GSVT	0.871156	0.862641	0.879860	300.0
SB	0.886517	0.879385	0.893770	892.0
SR	0.875567	0.876632	0.874539	681.0
macro avg	0.866008	0.873093	0.860077	2077.0
micro avg	0.875473	0.875473	0.875473	2077.0
weighted avg	0.875235	0.875507	0.875473	2077.0

The result of Model Number:Dataset Number:Featuregroup Number:Rescale of 23 : 1 : 1 : 0

	f1-score	precision	recall	support
AFIB	0.749935	0.741090	0.759025	875.0
GSVT	0.764672	0.771322	0.758141	691.0
SB	0.814951	0.811832	0.818097	1553.0
SR	0.795723	0.805343	0.786338	907.0
macro avg	0.781320	0.782397	0.780400	4026.0
micro avg	0.787813	0.787813	0.787813	4026.0
weighted avg	0.787859	0.788042	0.787813	4026.0

The result of Model Number:Dataset Number:Featuregroup Number:Rescale of 23 : 1 : 1 : 1

	f1-score	precision	recall	support
AFIB	0.653247	0.644907	0.661844	875.0
GSVT	0.664713	0.668525	0.660962	691.0
SB	0.711534	0.708546	0.714555	1553.0
SR	0.690583	0.701761	0.679768	907.0
macro avg	0.680019	0.680935	0.679282	4026.0
micro avg	0.686063	0.686063	0.686063	4026.0
weighted avg	0.686110	0.686317	0.686063	4026.0

The result of Model Number:Dataset Number:Featuregroup Number:Rescale of 23 : 1 : 2 : 0

	f1-score	precision	recall	support
AFIB	0.675010	0.673701	0.676411	875.0
GSVT	0.687685	0.675653	0.700219	691.0
SB	0.736705	0.726252	0.747471	1553.0
SR	0.697854	0.728963	0.669321	907.0
macro avg	0.699313	0.701142	0.698356	4026.0
micro avg	0.706311	0.706311	0.706311	4026.0
weighted avg	0.706130	0.706757	0.706311	4026.0

The result of Model Number:Dataset Number:Featuregroup Number:Rescale of 23 : 1 : 2 : 1

	f1-score	precision	recall	support
AFIB	0.688292	0.721659	0.657949	204.0
GSVT	0.716957	0.707589	0.726722	300.0
SB	0.736267	0.730669	0.741963	892.0
SR	0.715767	0.717906	0.713673	681.0
macro avg	0.714321	0.719456	0.710077	2077.0
micro avg	0.722234	0.722234	0.722234	2077.0
weighted avg	0.722045	0.722266	0.722234	2077.0

The result of Model Number:Dataset Number:Featuregroup Number:Rescale of 23 : 1 : 3 : 0

	f1-score	precision	recall	support
AFIB	0.710468	0.711922	0.709097	204.0
GSVT	0.726094	0.723272	0.728990	300.0
SB	0.742213	0.741223	0.743210	892.0
SR	0.736761	0.738939	0.734599	681.0
macro avg	0.728884	0.728839	0.728974	2077.0
micro avg	0.734982	0.734982	0.734982	2077.0
weighted avg	0.734979	0.735003	0.734982	2077.0

The result of Model Number:Dataset Number:Featuregroup Number:Rescale of 23 : 1 : 3 : 1

	f1-score	precision	recall	support
AFIB	0.632621	0.613713	0.674539	875.0

GSVT 0.541746 0.529883 0.554354 691.0
SB 0.718562 0.712707 0.724704 1553.0
SR 0.674114 0.702417 0.649270 907.0
macro avg 0.641761 0.639680 0.650717 4026.0
micro avg 0.667569 0.667569 0.667569 4026.0
weighted avg 0.659523 0.657495 0.667569 4026.0
The result of Model Number:Dataset Number:Featuregroup Number:Rescale of 23 : 1 : 4 : 0

	f1-score	precision	recall	support
AFIB	0.738863	0.754922	0.723676	204.0
GSVT	0.769363	0.764825	0.773977	300.0
SB	0.785892	0.784149	0.787652	892.0
SR	0.778014	0.777575	0.778473	681.0
macro avg	0.768033	0.770368	0.765944	2077.0
micro avg	0.776383	0.776383	0.776383	2077.0
weighted avg	0.776302	0.776332	0.776383	2077.0

The result of Model Number:Dataset Number:Featuregroup Number:Rescale of 23 : 1 : 4 : 1

	f1-score	precision	recall	support
AFIB	0.616729	0.573512	0.724231	875.0
GSVT	0.528924	0.493825	0.598267	691.0
SB	0.632031	0.626698	0.637469	1553.0
SR	0.609186	0.623952	0.595315	907.0
macro avg	0.596718	0.579497	0.638821	4026.0
micro avg	0.640101	0.640101	0.640101	4026.0
weighted avg	0.605862	0.591714	0.640101	4026.0

The result of Model Number:Dataset Number:Featuregroup Number:Rescale of 23 : 1 : 5 : 0

	f1-score	precision	recall	support
AFIB	0.839763	0.848349	0.831419	875.0
GSVT	0.839510	0.826541	0.852992	691.0
SB	0.885571	0.880283	0.890931	1553.0
SR	0.862132	0.873671	0.850919	907.0
macro avg	0.856744	0.857211	0.856565	4026.0
micro avg	0.862471	0.862471	0.862471	4026.0
weighted avg	0.862429	0.862629	0.862471	4026.0

The result of Model Number:Dataset Number:Featuregroup Number:Rescale of 23 : 1 : 5 : 1

	f1-score	precision	recall	support
AFIB	0.800410	0.821364	0.780863	204.0
GSVT	0.753131	0.775019	0.732855	300.0
SB	0.794714	0.766734	0.824828	892.0
SR	0.720311	0.743769	0.698432	681.0
macro avg	0.767141	0.776722	0.759245	2077.0

micro avg 0.765783 0.765783 0.765783 2077.0
weighted avg 0.764872 0.765767 0.765783 2077.0
The result of Model Number:Dataset Number:Featuregroup Number:Rescale of 23 : 1 : 6 : 0

	f1-score	precision	recall	support
AFIB	0.704869	0.695106	0.714938	875.0
GSVT	0.720502	0.726333	0.714768	691.0
SB	0.764804	0.762607	0.767020	1553.0
SR	0.746199	0.755911	0.736741	907.0
macro avg	0.734094	0.734989	0.733367	4026.0
micro avg	0.739911	0.739911	0.739911	4026.0
weighted avg	0.739983	0.740202	0.739911	4026.0

The result of Model Number:Dataset Number:Featuregroup Number:Rescale of 23 : 1 : 6 : 1

	f1-score	precision	recall	support
AFIB	0.709788	0.708995	0.710604	875.0
GSVT	0.722771	0.713731	0.732064	691.0
SB	0.759267	0.753163	0.765477	1553.0
SR	0.725392	0.744425	0.707320	907.0
macro avg	0.729305	0.730079	0.728866	4026.0
micro avg	0.734714	0.734714	0.734714	4026.0
weighted avg	0.734618	0.734827	0.734714	4026.0

The result of Model Number:Dataset Number:Featuregroup Number:Rescale of 23 : 1 : 7 : 0

	f1-score	precision	recall	support
AFIB	0.741712	0.747855	0.835437	204.0
GSVT	0.804337	0.769910	0.907480	300.0
SB	0.734389	0.731837	0.736962	892.0
SR	0.727087	0.727225	0.726955	681.0
macro avg	0.751881	0.744207	0.801708	2077.0
micro avg	0.767982	0.767982	0.767982	2077.0
weighted avg	0.742818	0.737397	0.767982	2077.0

The result of Model Number:Dataset Number:Featuregroup Number:Rescale of 23 : 1 : 7 : 1

	f1-score	precision	recall	support
AFIB	0.892825	0.916418	0.870505	204.0
GSVT	0.917310	0.909690	0.925227	300.0
SB	0.942588	0.939128	0.946074	892.0
SR	0.927993	0.929117	0.926896	681.0
macro avg	0.920179	0.923588	0.917176	2077.0
micro avg	0.929353	0.929353	0.929353	2077.0
weighted avg	0.929264	0.929363	0.929353	2077.0

The result of Model Number:Dataset Number:Featuregroup Number:Rescale of 23 : 1 : 8 : 0

	f1-score	precision	recall	support
AFIB	0.770157	0.749925	0.818371	875.0
GSVT	0.740884	0.701256	0.844593	691.0
SB	0.702026	0.697589	0.706540	1553.0
SR	0.681907	0.693656	0.670740	907.0
macro avg	0.723744	0.710607	0.760061	4026.0
micro avg	0.746475	0.746475	0.746475	4026.0
weighted avg	0.718970	0.708707	0.746475	4026.0

The result of Model Number:Dataset Number:Featuregroup Number:Rescale of 23 : 1 : 8 : 1

	f1-score	precision	recall	support
AFIB	0.818638	0.822695	0.814709	875.0
GSVT	0.826789	0.827980	0.825727	691.0
SB	0.870307	0.866269	0.874393	1553.0
SR	0.854286	0.856425	0.852204	907.0
macro avg	0.842505	0.843343	0.841758	4026.0
micro avg	0.848070	0.848070	0.848070	4026.0
weighted avg	0.847999	0.848010	0.848070	4026.0

The result of Model Number:Dataset Number:Featuregroup Number:Rescale of 23 : 1 : 9 : 0

	f1-score	precision	recall	support
AFIB	0.630241	0.702915	0.571270	204.0
GSVT	0.743119	0.730185	0.756678	300.0
SB	0.793422	0.789905	0.796978	892.0
SR	0.775009	0.764412	0.785927	681.0
macro avg	0.735448	0.746854	0.727713	2077.0
micro avg	0.765365	0.765365	0.765365	2077.0
weighted avg	0.764092	0.764376	0.765365	2077.0

The result of Model Number:Dataset Number:Featuregroup Number:Rescale of 23 : 1 : 9 : 1

	f1-score	precision	recall	support
AFIB	0.769731	0.763804	0.775809	875.0
GSVT	0.779725	0.783345	0.776308	691.0
SB	0.835584	0.831139	0.840094	1553.0
SR	0.810965	0.822366	0.799964	907.0
macro avg	0.799001	0.800164	0.798044	4026.0
micro avg	0.806134	0.806134	0.806134	4026.0
weighted avg	0.806138	0.806325	0.806134	4026.0

The result of Model Number:Dataset Number:Featuregroup Number:Rescale of 23 : 1 : 10 : 0

	f1-score	precision	recall	support
AFIB	0.662044	0.653177	0.671196	875.0
GSVT	0.676846	0.682129	0.671656	691.0
SB	0.718621	0.716373	0.720888	1553.0

SR	0.701243	0.710540	0.692200	907.0
macro avg	0.689689	0.690555	0.688985	4026.0
micro avg	0.695175	0.695175	0.695175	4026.0
weighted avg	0.695240	0.695447	0.695175	4026.0

The result of Model Number:Dataset Number:Featuregroup Number:Rescale of 23 : 1 : 10 : 1

	f1-score	precision	recall	support
AFIB	0.679614	0.670938	0.688558	875.0
GSVT	0.691543	0.695509	0.687641	691.0
SB	0.740254	0.737145	0.743397	1553.0
SR	0.718458	0.730087	0.707206	907.0
macro avg	0.707467	0.708420	0.706701	4026.0
micro avg	0.713755	0.713755	0.713755	4026.0
weighted avg	0.713804	0.714019	0.713755	4026.0

The result of Model Number:Dataset Number:Featuregroup Number:Rescale of 24 : 0 : 0 : 0

	f1-score	precision	recall	support
AFIB	0.691413	0.725959	0.660354	204.0
GSVT	0.712258	0.699058	0.726214	300.0
SB	0.728558	0.721214	0.736068	892.0
SR	0.708352	0.714680	0.702200	681.0
macro avg	0.710145	0.715228	0.706209	2077.0
micro avg	0.716104	0.716104	0.716104	2077.0
weighted avg	0.715930	0.716337	0.716104	2077.0

The result of Model Number:Dataset Number:Featuregroup Number:Rescale of 24 : 0 : 0 : 1

	f1-score	precision	recall	support
AFIB	0.820864	0.825546	0.816372	204.0
GSVT	0.837754	0.831712	0.843894	300.0
SB	0.854674	0.851552	0.857834	892.0
SR	0.845584	0.851136	0.840126	681.0
macro avg	0.839719	0.839987	0.839556	2077.0
micro avg	0.845942	0.845942	0.845942	2077.0
weighted avg	0.845929	0.845996	0.845942	2077.0

The result of Model Number:Dataset Number:Featuregroup Number:Rescale of 24 : 0 : 1 : 0

	f1-score	precision	recall	support
AFIB	0.673380	0.673164	0.673630	875.0
GSVT	0.683415	0.674630	0.692511	691.0
SB	0.722875	0.721303	0.724458	1553.0
SR	0.702875	0.713178	0.692877	907.0
macro avg	0.695636	0.695569	0.695869	4026.0
micro avg	0.700813	0.700813	0.700813	4026.0
weighted avg	0.700839	0.701000	0.700813	4026.0

The result of Model Number:Dataset Number:Featuregroup Number:Rescale of 24 : 0 : 1 : 1

	f1-score	precision	recall	support
AFIB	0.690991	0.690368	0.691681	875.0
GSVT	0.702961	0.696525	0.709573	691.0
SB	0.737674	0.731188	0.744276	1553.0
SR	0.702945	0.720323	0.686447	907.0
macro avg	0.708643	0.709601	0.707994	4026.0
micro avg	0.713861	0.713861	0.713861	4026.0
weighted avg	0.713746	0.713919	0.713861	4026.0

The result of Model Number:Dataset Number:Featuregroup Number:Rescale of 24 : 0 : 2 : 0

	f1-score	precision	recall	support
AFIB	0.738290	0.769932	0.709427	204.0
GSVT	0.763099	0.751570	0.775037	300.0
SB	0.778235	0.770538	0.786089	892.0
SR	0.757150	0.763628	0.750810	681.0
macro avg	0.759194	0.763917	0.755341	2077.0
micro avg	0.765396	0.765396	0.765396	2077.0
weighted avg	0.765212	0.765473	0.765396	2077.0

The result of Model Number:Dataset Number:Featuregroup Number:Rescale of 24 : 0 : 2 : 1

	f1-score	precision	recall	support
AFIB	0.689459	0.691377	0.687587	875.0
GSVT	0.705252	0.705057	0.705455	691.0
SB	0.751116	0.744056	0.758319	1553.0
SR	0.731213	0.741758	0.720973	907.0
macro avg	0.719260	0.720562	0.718084	4026.0
micro avg	0.725460	0.725460	0.725460	4026.0
weighted avg	0.725360	0.725396	0.725460	4026.0

The result of Model Number:Dataset Number:Featuregroup Number:Rescale of 24 : 0 : 3 : 0

	f1-score	precision	recall	support
AFIB	0.623842	0.599993	0.663084	875.0
GSVT	0.646406	0.624097	0.676666	691.0
SB	0.720216	0.707503	0.734185	1553.0
SR	0.610007	0.733498	0.526658	907.0
macro avg	0.650118	0.666273	0.650148	4026.0
micro avg	0.662107	0.662107	0.662107	4026.0
weighted avg	0.661774	0.675678	0.662107	4026.0

The result of Model Number:Dataset Number:Featuregroup Number:Rescale of 24 : 0 : 3 : 1

	f1-score	precision	recall	support
AFIB	0.747000	0.724673	0.796496	875.0

GSVT 0.639695 0.625687 0.654582 691.0
SB 0.848479 0.841566 0.855732 1553.0
SR 0.795996 0.829415 0.766659 907.0
macro avg 0.757792 0.755335 0.768367 4026.0
micro avg 0.788267 0.788267 0.788267 4026.0
weighted avg 0.778766 0.776371 0.788267 4026.0
The result of Model Number:Dataset Number:Featuregroup Number:Rescale of 24 : 0 : 4 : 0

	f1-score	precision	recall	support
AFIB	0.857227	0.850627	0.863996	875.0
GSVT	0.868357	0.872389	0.864552	691.0
SB	0.930565	0.925615	0.935589	1553.0
SR	0.903148	0.915846	0.890897	907.0
macro avg	0.889825	0.891119	0.888759	4026.0
micro avg	0.897768	0.897768	0.897768	4026.0
weighted avg	0.897773	0.897981	0.897768	4026.0

The result of Model Number:Dataset Number:Featuregroup Number:Rescale of 24 : 0 : 4 : 1

	f1-score	precision	recall	support
AFIB	0.808305	0.809214	0.807545	204.0
GSVT	0.826863	0.825017	0.828781	300.0
SB	0.844389	0.843262	0.845524	892.0
SR	0.838065	0.840176	0.835976	681.0
macro avg	0.829406	0.829417	0.829456	2077.0
micro avg	0.836245	0.836245	0.836245	2077.0
weighted avg	0.836240	0.836271	0.836245	2077.0

The result of Model Number:Dataset Number:Featuregroup Number:Rescale of 24 : 0 : 5 : 0

	f1-score	precision	recall	support
AFIB	0.663464	0.676197	0.651240	875.0
GSVT	0.707232	0.708311	0.706200	691.0
SB	0.770085	0.766701	0.773505	1553.0
SR	0.727706	0.719795	0.735868	907.0
macro avg	0.717122	0.717751	0.716703	4026.0
micro avg	0.726901	0.726901	0.726901	4026.0
weighted avg	0.726577	0.726442	0.726901	4026.0

The result of Model Number:Dataset Number:Featuregroup Number:Rescale of 24 : 0 : 5 : 1

	f1-score	precision	recall	support
AFIB	0.784548	0.819416	0.752634	204.0
GSVT	0.817241	0.810826	0.823777	300.0
SB	0.829725	0.823324	0.836233	892.0
SR	0.820545	0.821900	0.819224	681.0
macro avg	0.813015	0.818867	0.807967	2077.0

micro avg 0.820646 0.820646 0.820646 2077.0
weighted avg 0.820475 0.820668 0.820646 2077.0
The result of Model Number:Dataset Number:Featuregroup Number:Rescale of 24 : 0 : 6 : 0

	f1-score	precision	recall	support
AFIB	0.617520	0.672692	0.652115	204.0
GSVT	0.838763	0.825052	0.853227	300.0
SB	0.714047	0.708608	0.719623	892.0
SR	0.873163	0.863283	0.883297	681.0
macro avg	0.760873	0.767409	0.777065	2077.0
micro avg	0.785955	0.785955	0.785955	2077.0
weighted avg	0.774750	0.772614	0.785955	2077.0

The result of Model Number:Dataset Number:Featuregroup Number:Rescale of 24 : 0 : 6 : 1

	f1-score	precision	recall	support
AFIB	0.720609	0.799728	0.655908	204.0
GSVT	0.841408	0.830889	0.852362	300.0
SB	0.895217	0.890649	0.899833	892.0
SR	0.874246	0.862023	0.886824	681.0
macro avg	0.832870	0.845822	0.823732	2077.0
micro avg	0.864753	0.864753	0.864753	2077.0
weighted avg	0.863419	0.863701	0.864753	2077.0

The result of Model Number:Dataset Number:Featuregroup Number:Rescale of 24 : 0 : 7 : 0

	f1-score	precision	recall	support
AFIB	0.760314	0.750607	0.770320	875.0
GSVT	0.773660	0.778096	0.769294	691.0
SB	0.828155	0.824677	0.831670	1553.0
SR	0.803770	0.816780	0.791183	907.0
macro avg	0.791475	0.792540	0.790617	4026.0
micro avg	0.798509	0.798509	0.798509	4026.0
weighted avg	0.798564	0.798805	0.798509	4026.0

The result of Model Number:Dataset Number:Featuregroup Number:Rescale of 24 : 0 : 7 : 1

	f1-score	precision	recall	support
AFIB	0.917812	0.946080	0.891368	204.0
GSVT	0.941226	0.929704	0.953141	300.0
SB	0.966792	0.961047	0.972637	892.0
SR	0.950496	0.955184	0.945890	681.0
macro avg	0.944081	0.948004	0.940759	2077.0
micro avg	0.953069	0.953069	0.953069	2077.0
weighted avg	0.952945	0.953127	0.953069	2077.0

The result of Model Number:Dataset Number:Featuregroup Number:Rescale of 24 : 0 : 8 : 0

	f1-score	precision	recall	support
AFIB	0.843205	0.865486	0.822125	204.0
GSVT	0.866329	0.859132	0.873806	300.0
SB	0.890201	0.886935	0.893495	892.0
SR	0.876418	0.877480	0.875382	681.0
macro avg	0.869038	0.872258	0.866202	2077.0
micro avg	0.877702	0.877702	0.877702	2077.0
weighted avg	0.877618	0.877712	0.877702	2077.0

The result of Model Number:Dataset Number:Featuregroup Number:Rescale of 24 : 0 : 8 : 1

	f1-score	precision	recall	support
AFIB	0.743598	0.757246	0.730793	204.0
GSVT	0.771827	0.765621	0.778275	300.0
SB	0.786144	0.784741	0.787554	892.0
SR	0.778360	0.779053	0.777673	681.0
macro avg	0.769982	0.771665	0.768574	2077.0
micro avg	0.777399	0.777399	0.777399	2077.0
weighted avg	0.777345	0.777414	0.777399	2077.0

The result of Model Number:Dataset Number:Featuregroup Number:Rescale of 24 : 0 : 9 : 0

	f1-score	precision	recall	support
AFIB	0.679161	0.687588	0.671166	204.0
GSVT	0.691339	0.686616	0.696201	300.0
SB	0.707711	0.706605	0.708820	892.0
SR	0.702627	0.703766	0.701497	681.0
macro avg	0.695210	0.696144	0.694421	2077.0
micro avg	0.700898	0.700898	0.700898	2077.0
weighted avg	0.700875	0.700919	0.700898	2077.0

The result of Model Number:Dataset Number:Featuregroup Number:Rescale of 24 : 0 : 9 : 1

	f1-score	precision	recall	support
AFIB	0.693863	0.709297	0.679234	204.0
GSVT	0.723425	0.718929	0.728005	300.0
SB	0.739129	0.737572	0.740701	892.0
SR	0.731256	0.730739	0.731797	681.0
macro avg	0.721918	0.724134	0.719934	2077.0
micro avg	0.729911	0.729911	0.729911	2077.0
weighted avg	0.729833	0.729862	0.729911	2077.0

The result of Model Number:Dataset Number:Featuregroup Number:Rescale of 24 : 0 : 10 : 0

	f1-score	precision	recall	support
AFIB	0.142771	0.144270	0.141302	175.0
GSVT	0.143001	0.141482	0.144553	138.2
SB	0.150605	0.150027	0.151187	310.6

SR 0.147119 0.147857 0.146390 181.4
macro avg 0.145874 0.145909 0.145858 805.2
micro avg 0.146819 0.146819 0.146819 805.2
weighted avg 0.146812 0.146820 0.146819 805.2
The result of Model Number:Dataset Number:Featuregroup Number:Rescale of 24 : 0 : 10 : 1

	f1-score	precision	recall	support
AFIB	0.675495	0.670464	0.680669	875.0
GSVT	0.688093	0.690550	0.685695	691.0
SB	0.730151	0.728421	0.731890	1553.0
SR	0.713488	0.719912	0.707204	907.0
macro avg	0.701807	0.702337	0.701364	4026.0
micro avg	0.707268	0.707268	0.707268	4026.0
weighted avg	0.707300	0.707408	0.707268	4026.0

The result of Model Number:Dataset Number:Featuregroup Number:Rescale of 24 : 1 : 0 : 0

	f1-score	precision	recall	support
AFIB	0.852847	0.869489	0.836977	204.0
GSVT	0.874103	0.862824	0.885739	300.0
SB	0.888980	0.880192	0.897950	892.0
SR	0.870399	0.882492	0.858638	681.0
macro avg	0.871582	0.873749	0.869826	2077.0
micro avg	0.877308	0.877308	0.877308	2077.0
weighted avg	0.877190	0.877386	0.877308	2077.0

The result of Model Number:Dataset Number:Featuregroup Number:Rescale of 24 : 1 : 0 : 1

	f1-score	precision	recall	support
AFIB	0.883211	0.900929	0.866353	204.0
GSVT	0.903680	0.894287	0.913321	300.0
SB	0.920393	0.916813	0.924004	892.0
SR	0.910722	0.914522	0.906974	681.0
macro avg	0.904502	0.906638	0.902663	2077.0
micro avg	0.911215	0.911215	0.911215	2077.0
weighted avg	0.911156	0.911248	0.911215	2077.0

The result of Model Number:Dataset Number:Featuregroup Number:Rescale of 24 : 1 : 1 : 0

	f1-score	precision	recall	support
AFIB	0.834094	0.879812	0.793448	204.0
GSVT	0.794235	0.822081	0.768311	300.0
SB	0.816949	0.785440	0.851105	892.0
SR	0.727106	0.746833	0.708437	681.0
macro avg	0.793096	0.808541	0.780325	2077.0
micro avg	0.786706	0.786706	0.786706	2077.0
weighted avg	0.785895	0.787343	0.786706	2077.0

The result of Model Number:Dataset Number:Featuregroup Number:Rescale of 24 : 1 : 1 : 1

	f1-score	precision	recall	support
AFIB	0.853775	0.853552	0.854081	875.0
GSVT	0.871611	0.868315	0.875025	691.0
SB	0.927961	0.923771	0.932203	1553.0
SR	0.896687	0.906932	0.886706	907.0
macro avg	0.887509	0.888143	0.887004	4026.0
micro avg	0.895161	0.895161	0.895161	4026.0
weighted avg	0.895121	0.895198	0.895161	4026.0

The result of Model Number:Dataset Number:Featuregroup Number:Rescale of 24 : 1 : 2 : 0

	f1-score	precision	recall	support
AFIB	0.720094	0.725802	0.714494	875.0
GSVT	0.729224	0.726009	0.732492	691.0
SB	0.775443	0.772378	0.778539	1553.0
SR	0.756388	0.758525	0.754299	907.0
macro avg	0.745287	0.745678	0.744956	4026.0
micro avg	0.751255	0.751255	0.751255	4026.0
weighted avg	0.751188	0.751176	0.751255	4026.0

The result of Model Number:Dataset Number:Featuregroup Number:Rescale of 24 : 1 : 2 : 1

	f1-score	precision	recall	support
AFIB	0.853222	0.857905	0.848645	875.0
GSVT	0.854415	0.843613	0.865632	691.0
SB	0.898565	0.894960	0.902208	1553.0
SR	0.879276	0.889950	0.868910	907.0
macro avg	0.871370	0.871607	0.871349	4026.0
micro avg	0.876787	0.876787	0.876787	4026.0
weighted avg	0.876787	0.876965	0.876787	4026.0

The result of Model Number:Dataset Number:Featuregroup Number:Rescale of 24 : 1 : 3 : 0

	f1-score	precision	recall	support
AFIB	0.924354	0.942162	0.907644	204.0
GSVT	0.941712	0.926411	0.957698	300.0
SB	0.957434	0.946015	0.969134	892.0
SR	0.939623	0.957240	0.922659	681.0
macro avg	0.940781	0.942957	0.939284	2077.0
micro avg	0.946205	0.946205	0.946205	2077.0
weighted avg	0.946074	0.946485	0.946205	2077.0

The result of Model Number:Dataset Number:Featuregroup Number:Rescale of 24 : 1 : 3 : 1

	f1-score	precision	recall	support
AFIB	0.745398	0.759078	0.732561	204.0

GSVT	0.773694	0.767473	0.780158	300.0
SB	0.788046	0.786640	0.789460	892.0
SR	0.780244	0.780938	0.779554	681.0
macro avg	0.771845	0.773532	0.770433	2077.0
micro avg	0.779280	0.779280	0.779280	2077.0
weighted avg	0.779226	0.779295	0.779280	2077.0

The result of Model Number:Dataset Number:Featuregroup Number:Rescale of 24 : 1 : 4 : 0

	f1-score	precision	recall	support
AFIB	0.832942	0.828771	0.837430	875.0
GSVT	0.851762	0.852914	0.850785	691.0
SB	0.907010	0.904023	0.910041	1553.0
SR	0.880373	0.889510	0.871526	907.0
macro avg	0.868022	0.868805	0.867445	4026.0
micro avg	0.875413	0.875413	0.875413	4026.0
weighted avg	0.875429	0.875627	0.875413	4026.0

The result of Model Number:Dataset Number:Featuregroup Number:Rescale of 24 : 1 : 4 : 1

	f1-score	precision	recall	support
AFIB	0.820674	0.855847	0.788590	204.0
GSVT	0.848252	0.835435	0.861521	300.0
SB	0.865076	0.856521	0.873806	892.0
SR	0.841639	0.848839	0.834591	681.0
macro avg	0.843910	0.849161	0.839627	2077.0
micro avg	0.850804	0.850804	0.850804	2077.0
weighted avg	0.850600	0.850891	0.850804	2077.0

The result of Model Number:Dataset Number:Featuregroup Number:Rescale of 24 : 1 : 5 : 0

	f1-score	precision	recall	support
AFIB	0.898842	0.935121	0.865344	204.0
GSVT	0.932291	0.917146	0.948104	300.0
SB	0.956409	0.948720	0.964236	892.0
SR	0.930678	0.937380	0.924121	681.0
macro avg	0.929555	0.934592	0.925451	2077.0
micro avg	0.939040	0.939040	0.939040	2077.0
weighted avg	0.938835	0.939106	0.939040	2077.0

The result of Model Number:Dataset Number:Featuregroup Number:Rescale of 24 : 1 : 5 : 1

	f1-score	precision	recall	support
AFIB	0.596709	0.601651	0.672111	204.0
GSVT	0.647091	0.619394	0.730070	300.0
SB	0.590818	0.588765	0.592887	892.0
SR	0.584943	0.585054	0.584837	681.0
macro avg	0.604890	0.598716	0.644976	2077.0

micro avg 0.617843 0.617843 0.617843 2077.0
weighted avg 0.597598 0.593238 0.617843 2077.0
The result of Model Number:Dataset Number:Featuregroup Number:Rescale of 24 : 1 : 6 : 0

	f1-score	precision	recall	support
AFIB	0.862701	0.875638	0.850386	204.0
GSVT	0.893854	0.891481	0.896246	300.0
SB	0.915028	0.913808	0.916258	892.0
SR	0.906078	0.904910	0.907279	681.0
macro avg	0.894415	0.896459	0.892542	2077.0
micro avg	0.903954	0.903954	0.903954	2077.0
weighted avg	0.903896	0.903917	0.903954	2077.0

The result of Model Number:Dataset Number:Featuregroup Number:Rescale of 24 : 1 : 6 : 1

	f1-score	precision	recall	support
AFIB	0.743259	0.805306	0.691570	204.0
GSVT	0.797231	0.787206	0.807839	300.0
SB	0.817583	0.806129	0.829463	892.0
SR	0.795281	0.799007	0.791978	681.0
macro avg	0.788338	0.799412	0.780212	2077.0
micro avg	0.800505	0.800505	0.800505	2077.0
weighted avg	0.800031	0.800980	0.800505	2077.0

The result of Model Number:Dataset Number:Featuregroup Number:Rescale of 24 : 1 : 7 : 0

	f1-score	precision	recall	support
AFIB	0.704312	0.777981	0.643595	204.0
GSVT	0.828575	0.817635	0.839900	300.0
SB	0.878700	0.875286	0.882148	892.0
SR	0.857586	0.845363	0.870176	681.0
macro avg	0.817293	0.829066	0.808955	2077.0
micro avg	0.848690	0.848690	0.848690	2077.0
weighted avg	0.847409	0.847591	0.848690	2077.0

The result of Model Number:Dataset Number:Featuregroup Number:Rescale of 24 : 1 : 7 : 1

	f1-score	precision	recall	support
AFIB	0.887557	0.903526	0.872404	204.0
GSVT	0.907580	0.894819	0.920861	300.0
SB	0.921545	0.910656	0.932701	892.0
SR	0.903011	0.919088	0.887497	681.0
macro avg	0.904923	0.907022	0.903366	2077.0
micro avg	0.910247	0.910247	0.910247	2077.0
weighted avg	0.910113	0.910433	0.910247	2077.0

The result of Model Number:Dataset Number:Featuregroup Number:Rescale of 24 : 1 : 8 : 0

	f1-score	precision	recall	support
AFIB	0.886419	0.878493	0.894528	875.0
GSVT	0.900651	0.904214	0.897148	691.0
SB	0.960287	0.958504	0.962079	1553.0
SR	0.935953	0.944605	0.927493	907.0
macro avg	0.920827	0.921454	0.920312	4026.0
micro avg	0.928461	0.928461	0.928461	4026.0
weighted avg	0.928515	0.928666	0.928461	4026.0

The result of Model Number:Dataset Number:Featuregroup Number:Rescale of 24 : 1 : 8 : 1

	f1-score	precision	recall	support
AFIB	0.884001	0.934290	0.839437	204.0
GSVT	0.934190	0.923903	0.944756	300.0
SB	0.948087	0.938653	0.957744	892.0
SR	0.937073	0.940276	0.934039	681.0
macro avg	0.925838	0.934280	0.918994	2077.0
micro avg	0.936476	0.936476	0.936476	2077.0
weighted avg	0.936174	0.936626	0.936476	2077.0

The result of Model Number:Dataset Number:Featuregroup Number:Rescale of 24 : 1 : 9 : 0

	f1-score	precision	recall	support
AFIB	0.691532	0.707185	0.676646	204.0
GSVT	0.709935	0.706459	0.713476	300.0
SB	0.724458	0.722366	0.726568	892.0
SR	0.719804	0.719493	0.720126	681.0
macro avg	0.711432	0.713876	0.709204	2077.0
micro avg	0.717662	0.717662	0.717662	2077.0
weighted avg	0.717601	0.717635	0.717662	2077.0

The result of Model Number:Dataset Number:Featuregroup Number:Rescale of 24 : 1 : 9 : 1

	f1-score	precision	recall	support
AFIB	0.645397	0.630217	0.679461	875.0
GSVT	0.620168	0.614450	0.634299	691.0
SB	0.672614	0.625254	0.728511	1553.0
SR	0.419383	0.525974	0.349254	907.0
macro avg	0.589390	0.598974	0.597881	4026.0
micro avg	0.616240	0.616240	0.616240	4026.0
weighted avg	0.600648	0.602112	0.616240	4026.0

The result of Model Number:Dataset Number:Featuregroup Number:Rescale of 24 : 1 : 10 : 0

	f1-score	precision	recall	support
AFIB	0.628365	0.621576	0.715288	204.0
GSVT	0.751798	0.745940	0.757813	300.0
SB	0.613539	0.612256	0.614828	892.0

SR	0.760802	0.760588	0.761025	681.0
macro avg	0.688626	0.685090	0.712238	2077.0
micro avg	0.693282	0.693282	0.693282	2077.0
weighted avg	0.683249	0.681115	0.693282	2077.0

The result of Model Number:Dataset Number:Featuregroup Number:Rescale of 24 : 1 : 10 : 1

	f1-score	precision	recall	support
AFIB	0.811773	0.817242	0.806497	875.0
GSVT	0.826260	0.814784	0.838216	691.0
SB	0.880471	0.874441	0.886591	1553.0
SR	0.844790	0.859382	0.830731	907.0
macro avg	0.840824	0.841463	0.840509	4026.0
micro avg	0.848296	0.848296	0.848296	4026.0
weighted avg	0.848197	0.848378	0.848296	4026.0

The result of Model Number:Dataset Number:Featuregroup Number:Rescale of 25 : 0 : 0 : 0

	f1-score	precision	recall	support
AFIB	0.874143	0.880857	0.867728	204.0
GSVT	0.904926	0.904058	0.905824	300.0
SB	0.923439	0.921288	0.925610	892.0
SR	0.914941	0.916174	0.913731	681.0
macro avg	0.904362	0.905594	0.903223	2077.0
micro avg	0.913172	0.913172	0.913172	2077.0
weighted avg	0.913137	0.913152	0.913172	2077.0

The result of Model Number:Dataset Number:Featuregroup Number:Rescale of 25 : 0 : 0 : 1

	f1-score	precision	recall	support
AFIB	0.693572	0.699621	0.687800	204.0
GSVT	0.717558	0.716386	0.718751	300.0
SB	0.732810	0.731023	0.734615	892.0
SR	0.726092	0.727186	0.725025	681.0
macro avg	0.717508	0.718554	0.716548	2077.0
micro avg	0.724581	0.724581	0.724581	2077.0
weighted avg	0.724550	0.724567	0.724581	2077.0

The result of Model Number:Dataset Number:Featuregroup Number:Rescale of 25 : 0 : 1 : 0

	f1-score	precision	recall	support
AFIB	0.833554	0.848893	0.819184	204.0
GSVT	0.864308	0.857069	0.871820	300.0
SB	0.880537	0.879062	0.882018	892.0
SR	0.872762	0.873538	0.871994	681.0
macro avg	0.862790	0.864640	0.861254	2077.0
micro avg	0.871087	0.871087	0.871087	2077.0
weighted avg	0.871029	0.871111	0.871087	2077.0

The result of Model Number:Dataset Number:Featuregroup Number:Rescale of 25 : 0 : 1 : 1

	f1-score	precision	recall	support
AFIB	0.656874	0.637949	0.720717	875.0
GSVT	0.723963	0.721651	0.726384	691.0
SB	0.611132	0.602125	0.620461	1553.0
SR	0.731757	0.746040	0.718103	907.0
macro avg	0.680932	0.676941	0.696416	4026.0
micro avg	0.682428	0.682428	0.682428	4026.0
weighted avg	0.667614	0.662848	0.682428	4026.0

The result of Model Number:Dataset Number:Featuregroup Number:Rescale of 25 : 0 : 2 : 0

	f1-score	precision	recall	support
AFIB	0.622881	0.635818	0.610604	875.0
GSVT	0.607433	0.586335	0.657017	691.0
SB	0.715955	0.712726	0.719224	1553.0
SR	0.544303	0.535515	0.553433	907.0
macro avg	0.622643	0.617599	0.635069	4026.0
micro avg	0.647590	0.647590	0.647590	4026.0
weighted avg	0.638429	0.634395	0.647590	4026.0

The result of Model Number:Dataset Number:Featuregroup Number:Rescale of 25 : 0 : 2 : 1

	f1-score	precision	recall	support
AFIB	0.775940	0.785013	0.767218	204.0
GSVT	0.699901	0.673180	0.798577	300.0
SB	0.649365	0.647835	0.650909	892.0
SR	0.806293	0.807843	0.804755	681.0
macro avg	0.732875	0.728468	0.755365	2077.0
micro avg	0.734104	0.734104	0.734104	2077.0
weighted avg	0.720550	0.717432	0.734104	2077.0

The result of Model Number:Dataset Number:Featuregroup Number:Rescale of 25 : 0 : 3 : 0

	f1-score	precision	recall	support
AFIB	0.764657	0.764793	0.764554	875.0
GSVT	0.772747	0.775358	0.770203	691.0
SB	0.811731	0.808932	0.814553	1553.0
SR	0.796317	0.799003	0.793678	907.0
macro avg	0.786363	0.787022	0.785747	4026.0
micro avg	0.791372	0.791372	0.791372	4026.0
weighted avg	0.791336	0.791340	0.791372	4026.0

The result of Model Number:Dataset Number:Featuregroup Number:Rescale of 25 : 0 : 3 : 1

	f1-score	precision	recall	support
AFIB	0.833869	0.833118	0.834702	875.0

GSVT 0.848315 0.840548 0.856294 691.0
SB 0.890206 0.882379 0.898173 1553.0
SR 0.848296 0.869267 0.828386 907.0
macro avg 0.855171 0.856328 0.854389 4026.0
micro avg 0.861469 0.861469 0.861469 4026.0
weighted avg 0.861330 0.861539 0.861469 4026.0
The result of Model Number:Dataset Number:Featuregroup Number:Rescale of 25 : 0 : 4 : 0

	f1-score	precision	recall	support
AFIB	0.682512	0.689939	0.675443	204.0
GSVT	0.705609	0.702667	0.708691	300.0
SB	0.720069	0.719424	0.720714	892.0
SR	0.713264	0.713270	0.713264	681.0
macro avg	0.705363	0.706325	0.704528	2077.0
micro avg	0.712088	0.712088	0.712088	2077.0
weighted avg	0.712060	0.712090	0.712088	2077.0

The result of Model Number:Dataset Number:Featuregroup Number:Rescale of 25 : 0 : 4 : 1

	f1-score	precision	recall	support
AFIB	0.640629	0.641704	0.656507	204.0
GSVT	0.634606	0.659233	0.612571	300.0
SB	0.673138	0.636222	0.714877	892.0
SR	0.583467	0.637313	0.539488	681.0
macro avg	0.632960	0.643618	0.630861	2077.0
micro avg	0.636861	0.636861	0.636861	2077.0
weighted avg	0.634978	0.640442	0.636861	2077.0

The result of Model Number:Dataset Number:Featuregroup Number:Rescale of 25 : 0 : 5 : 0

	f1-score	precision	recall	support
AFIB	0.680209	0.685146	0.675392	875.0
GSVT	0.695130	0.688669	0.701766	691.0
SB	0.747061	0.742551	0.751631	1553.0
SR	0.722577	0.730645	0.714688	907.0
macro avg	0.711244	0.711753	0.710869	4026.0
micro avg	0.718180	0.718180	0.718180	4026.0
weighted avg	0.718103	0.718145	0.718180	4026.0

The result of Model Number:Dataset Number:Featuregroup Number:Rescale of 25 : 0 : 5 : 1

	f1-score	precision	recall	support
AFIB	0.687577	0.693128	0.682173	204.0
GSVT	0.703354	0.699905	0.706861	300.0
SB	0.717081	0.716286	0.717884	892.0
SR	0.711727	0.712673	0.710787	681.0
macro avg	0.704935	0.705498	0.704427	2077.0

micro avg 0.710458 0.710458 0.710458 2077.0
weighted avg 0.710445 0.710461 0.710458 2077.0
The result of Model Number:Dataset Number:Featuregroup Number:Rescale of 25 : 0 : 6 : 0

	f1-score	precision	recall	support
AFIB	0.660219	0.662154	0.658345	875.0
GSVT	0.671143	0.662523	0.680077	691.0
SB	0.710293	0.708567	0.712031	1553.0
SR	0.692307	0.700523	0.684289	907.0
macro avg	0.683491	0.683442	0.683686	4026.0
micro avg	0.688629	0.688629	0.688629	4026.0
weighted avg	0.688639	0.688765	0.688629	4026.0

The result of Model Number:Dataset Number:Featuregroup Number:Rescale of 25 : 0 : 6 : 1

	f1-score	precision	recall	support
AFIB	0.736531	0.740927	0.732206	204.0
GSVT	0.754628	0.751388	0.757898	300.0
SB	0.767552	0.766440	0.768670	892.0
SR	0.760534	0.762105	0.758973	681.0
macro avg	0.754811	0.755215	0.754437	2077.0
micro avg	0.760353	0.760353	0.760353	2077.0
weighted avg	0.760337	0.760339	0.760353	2077.0

The result of Model Number:Dataset Number:Featuregroup Number:Rescale of 25 : 0 : 7 : 0

	f1-score	precision	recall	support
AFIB	0.727244	0.725407	0.729204	875.0
GSVT	0.741432	0.737356	0.745594	691.0
SB	0.776338	0.771658	0.781086	1553.0
SR	0.753448	0.767047	0.740337	907.0
macro avg	0.749616	0.750367	0.749055	4026.0
micro avg	0.754538	0.754538	0.754538	4026.0
weighted avg	0.754520	0.754679	0.754538	4026.0

The result of Model Number:Dataset Number:Featuregroup Number:Rescale of 25 : 0 : 7 : 1

	f1-score	precision	recall	support
AFIB	0.749263	0.750443	0.748185	204.0
GSVT	0.767384	0.766187	0.768650	300.0
SB	0.783214	0.782259	0.784178	892.0
SR	0.777730	0.779222	0.776246	681.0
macro avg	0.769398	0.769528	0.769315	2077.0
micro avg	0.775799	0.775799	0.775799	2077.0
weighted avg	0.775795	0.775817	0.775799	2077.0

The result of Model Number:Dataset Number:Featuregroup Number:Rescale of 25 : 0 : 8 : 0

	f1-score	precision	recall	support
AFIB	0.772451	0.801187	0.746052	204.0
GSVT	0.716806	0.750069	0.686434	300.0
SB	0.750883	0.705593	0.802398	892.0
SR	0.659420	0.705236	0.619271	681.0
macro avg	0.724890	0.740521	0.713539	2077.0
micro avg	0.720071	0.720071	0.720071	2077.0
weighted avg	0.718091	0.721289	0.720071	2077.0

The result of Model Number:Dataset Number:Featuregroup Number:Rescale of 25 : 0 : 8 : 1

	f1-score	precision	recall	support
AFIB	0.861889	0.865825	0.858035	875.0
GSVT	0.876257	0.868846	0.883859	691.0
SB	0.926221	0.916763	0.935882	1553.0
SR	0.878419	0.896937	0.860697	907.0
macro avg	0.885697	0.887093	0.884618	4026.0
micro avg	0.893096	0.893096	0.893096	4026.0
weighted avg	0.892895	0.893002	0.893096	4026.0

The result of Model Number:Dataset Number:Featuregroup Number:Rescale of 25 : 0 : 9 : 0

	f1-score	precision	recall	support
AFIB	0.765346	0.749889	0.781513	875.0
GSVT	0.790720	0.803292	0.778706	691.0
SB	0.841338	0.841291	0.841392	1553.0
SR	0.820939	0.828420	0.813606	907.0
macro avg	0.804586	0.805723	0.803804	4026.0
micro avg	0.811359	0.811359	0.811359	4026.0
weighted avg	0.811539	0.812004	0.811359	4026.0

The result of Model Number:Dataset Number:Featuregroup Number:Rescale of 25 : 0 : 9 : 1

	f1-score	precision	recall	support
AFIB	0.749693	0.743921	0.755613	875.0
GSVT	0.759427	0.762953	0.756099	691.0
SB	0.813831	0.809502	0.818225	1553.0
SR	0.789853	0.800958	0.779139	907.0
macro avg	0.778201	0.779333	0.777269	4026.0
micro avg	0.785149	0.785149	0.785149	4026.0
weighted avg	0.785152	0.785335	0.785149	4026.0

The result of Model Number:Dataset Number:Featuregroup Number:Rescale of 25 : 0 : 10 : 0

	f1-score	precision	recall	support
AFIB	0.830975	0.833652	0.828393	875.0
GSVT	0.842658	0.829280	0.856551	691.0
SB	0.887877	0.880464	0.895423	1553.0

SR	0.843246	0.864663	0.822896	907.0
macro avg	0.851189	0.852015	0.850816	4026.0
micro avg	0.857844	0.857844	0.857844	4026.0
weighted avg	0.857694	0.857945	0.857844	4026.0

The result of Model Number:Dataset Number:Featuregroup Number:Rescale of 25 : 0 : 10 : 1

	f1-score	precision	recall	support
AFIB	0.862478	0.899832	0.828347	204.0
GSVT	0.806268	0.813792	0.799286	300.0
SB	0.835755	0.794665	0.881384	892.0
SR	0.744884	0.791892	0.703385	681.0
macro avg	0.812346	0.825045	0.803100	2077.0
micro avg	0.805955	0.805955	0.805955	2077.0
weighted avg	0.804326	0.806848	0.805955	2077.0

The result of Model Number:Dataset Number:Featuregroup Number:Rescale of 25 : 1 : 0 : 0

	f1-score	precision	recall	support
AFIB	0.730735	0.757827	0.705617	204.0
GSVT	0.752627	0.743216	0.762374	300.0
SB	0.771712	0.764670	0.778891	892.0
SR	0.751423	0.757254	0.745688	681.0
macro avg	0.751624	0.755742	0.748143	2077.0
micro avg	0.758422	0.758422	0.758422	2077.0
weighted avg	0.758278	0.758468	0.758422	2077.0

The result of Model Number:Dataset Number:Featuregroup Number:Rescale of 25 : 1 : 0 : 1

	f1-score	precision	recall	support
AFIB	0.722991	0.729459	0.716727	875.0
GSVT	0.736800	0.722790	0.751505	691.0
SB	0.782052	0.775276	0.788949	1553.0
SR	0.749931	0.767174	0.733479	907.0
macro avg	0.747943	0.748675	0.747665	4026.0
micro avg	0.754329	0.754329	0.754329	4026.0
weighted avg	0.754212	0.754485	0.754329	4026.0

The result of Model Number:Dataset Number:Featuregroup Number:Rescale of 25 : 1 : 1 : 0

	f1-score	precision	recall	support
AFIB	0.849597	0.853808	0.845519	875.0
GSVT	0.858057	0.859293	0.856954	691.0
SB	0.903220	0.899030	0.907460	1553.0
SR	0.886593	0.888814	0.884433	907.0
macro avg	0.874367	0.875236	0.873592	4026.0
micro avg	0.880142	0.880142	0.880142	4026.0
weighted avg	0.880069	0.880080	0.880142	4026.0

The result of Model Number:Dataset Number:Featuregroup Number:Rescale of 25 : 1 : 1 : 1

	f1-score	precision	recall	support
AFIB	0.671493	0.673034	0.670032	875.0
GSVT	0.673834	0.671934	0.675767	691.0
SB	0.707178	0.701322	0.713143	1553.0
SR	0.685963	0.696258	0.675997	907.0
macro avg	0.684617	0.685637	0.683735	4026.0
micro avg	0.688990	0.688990	0.688990	4026.0
weighted avg	0.688920	0.688989	0.688990	4026.0

The result of Model Number:Dataset Number:Featuregroup Number:Rescale of 25 : 1 : 2 : 0

	f1-score	precision	recall	support
AFIB	0.676914	0.723133	0.636537	204.0
GSVT	0.715565	0.700984	0.730829	300.0
SB	0.733314	0.726885	0.739885	892.0
SR	0.715225	0.717607	0.712923	681.0
macro avg	0.710254	0.717152	0.705043	2077.0
micro avg	0.719586	0.719586	0.719586	2077.0
weighted avg	0.719280	0.719733	0.719586	2077.0

The result of Model Number:Dataset Number:Featuregroup Number:Rescale of 25 : 1 : 2 : 1

	f1-score	precision	recall	support
AFIB	0.757333	0.750233	0.764665	875.0
GSVT	0.718024	0.700766	0.770807	691.0
SB	0.830261	0.827212	0.833355	1553.0
SR	0.645761	0.650290	0.641304	907.0
macro avg	0.737845	0.732125	0.752533	4026.0
micro avg	0.764424	0.764424	0.764424	4026.0
weighted avg	0.753582	0.748921	0.764424	4026.0

The result of Model Number:Dataset Number:Featuregroup Number:Rescale of 25 : 1 : 3 : 0

	f1-score	precision	recall	support
AFIB	0.739485	0.763504	0.717077	204.0
GSVT	0.759902	0.750212	0.769997	300.0
SB	0.781340	0.778394	0.784317	892.0
SR	0.770412	0.771807	0.769052	681.0
macro avg	0.762785	0.765979	0.760111	2077.0
micro avg	0.770640	0.770640	0.770640	2077.0
weighted avg	0.770550	0.770701	0.770640	2077.0

The result of Model Number:Dataset Number:Featuregroup Number:Rescale of 25 : 1 : 3 : 1

	f1-score	precision	recall	support
AFIB	0.750843	0.774910	0.728450	204.0

GSVT 0.773306 0.760002 0.787198 300.0
SB 0.786683 0.778906 0.794619 892.0
SR 0.765649 0.775230 0.756318 681.0
macro avg 0.769120 0.772262 0.766646 2077.0
micro avg 0.774490 0.774490 0.774490 2077.0
weighted avg 0.774334 0.774578 0.774490 2077.0
The result of Model Number:Dataset Number:Featuregroup Number:Rescale of 25 : 1 : 4 : 0

	f1-score	precision	recall	support
AFIB	0.828139	0.848761	0.808535	875.0
GSVT	0.874988	0.878703	0.871341	691.0
SB	0.949084	0.945452	0.952749	1553.0
SR	0.902787	0.885964	0.920309	907.0
macro avg	0.888749	0.889720	0.888234	4026.0
micro avg	0.900125	0.900125	0.900125	4026.0
weighted avg	0.899651	0.899579	0.900125	4026.0

The result of Model Number:Dataset Number:Featuregroup Number:Rescale of 25 : 1 : 4 : 1

	f1-score	precision	recall	support
AFIB	0.863685	0.880324	0.848071	204.0
GSVT	0.879904	0.865607	0.894841	300.0
SB	0.894594	0.883925	0.905526	892.0
SR	0.877952	0.894412	0.862101	681.0
macro avg	0.879034	0.881067	0.877635	2077.0
micro avg	0.884101	0.884101	0.884101	2077.0
weighted avg	0.883980	0.884364	0.884101	2077.0

The result of Model Number:Dataset Number:Featuregroup Number:Rescale of 25 : 1 : 5 : 0

	f1-score	precision	recall	support
AFIB	0.772792	0.809894	0.739094	204.0
GSVT	0.806405	0.799557	0.813393	300.0
SB	0.819078	0.813113	0.825136	892.0
SR	0.810462	0.810838	0.810111	681.0
macro avg	0.802184	0.808350	0.796933	2077.0
micro avg	0.810062	0.810062	0.810062	2077.0
weighted avg	0.809876	0.810093	0.810062	2077.0

The result of Model Number:Dataset Number:Featuregroup Number:Rescale of 25 : 1 : 5 : 1

	f1-score	precision	recall	support
AFIB	0.774782	0.781753	0.768177	875.0
GSVT	0.790564	0.774535	0.807458	691.0
SB	0.838399	0.829072	0.848039	1553.0
SR	0.805130	0.829063	0.782729	907.0
macro avg	0.802219	0.803606	0.801601	4026.0

micro avg 0.809004 0.809004 0.809004 4026.0
weighted avg 0.808867 0.809425 0.809004 4026.0
The result of Model Number:Dataset Number:Featuregroup Number:Rescale of 25 : 1 : 6 : 0

	f1-score	precision	recall	support
AFIB	0.782162	0.796235	0.768808	204.0
GSVT	0.799807	0.788562	0.811511	300.0
SB	0.812114	0.802519	0.821945	892.0
SR	0.795781	0.809949	0.782109	681.0
macro avg	0.797466	0.799316	0.796093	2077.0
micro avg	0.802158	0.802158	0.802158	2077.0
weighted avg	0.802039	0.802322	0.802158	2077.0

The result of Model Number:Dataset Number:Featuregroup Number:Rescale of 25 : 1 : 6 : 1

	f1-score	precision	recall	support
AFIB	0.665833	0.669229	0.662492	875.0
GSVT	0.675022	0.671510	0.678629	691.0
SB	0.707528	0.706896	0.708166	1553.0
SR	0.694500	0.695066	0.693963	907.0
macro avg	0.685721	0.685675	0.685813	4026.0
micro avg	0.689970	0.689970	0.689970	4026.0
weighted avg	0.689952	0.689971	0.689970	4026.0

The result of Model Number:Dataset Number:Featuregroup Number:Rescale of 25 : 1 : 7 : 0

	f1-score	precision	recall	support
AFIB	0.814958	0.831378	0.799698	204.0
GSVT	0.841108	0.834353	0.848136	300.0
SB	0.859317	0.858452	0.860184	892.0
SR	0.851484	0.850995	0.851983	681.0
macro avg	0.841717	0.843794	0.840000	2077.0
micro avg	0.849814	0.849814	0.849814	2077.0
weighted avg	0.849762	0.849867	0.849814	2077.0

The result of Model Number:Dataset Number:Featuregroup Number:Rescale of 25 : 1 : 7 : 1

	f1-score	precision	recall	support
AFIB	0.861617	0.885266	0.839263	204.0
GSVT	0.881105	0.867568	0.895153	300.0
SB	0.900463	0.891754	0.909347	892.0
SR	0.877705	0.888673	0.867011	681.0
macro avg	0.880223	0.883315	0.877694	2077.0
micro avg	0.886532	0.886532	0.886532	2077.0
weighted avg	0.886390	0.886613	0.886532	2077.0

The result of Model Number:Dataset Number:Featuregroup Number:Rescale of 25 : 1 : 8 : 0

f1-score precision recall support
AFIB 0.771791 0.767174 0.776568 875.0
GSVT 0.778111 0.782312 0.774068 691.0
SB 0.824681 0.822271 0.827119 1553.0
SR 0.806047 0.811837 0.800355 907.0
macro avg 0.795158 0.795898 0.794527 4026.0
micro avg 0.800997 0.800997 0.800997 4026.0
weighted avg 0.800995 0.801087 0.800997 4026.0
The result of Model Number:Dataset Number:Featuregroup Number:Rescale of 25 : 1 : 8 : 1
f1-score precision recall support
AFIB 0.847660 0.855478 0.840188 204.0
GSVT 0.878650 0.878681 0.878642 300.0
SB 0.897051 0.895458 0.898658 892.0
SR 0.887531 0.887293 0.887790 681.0
macro avg 0.877723 0.879228 0.876320 2077.0
micro avg 0.886461 0.886461 0.886461 2077.0
weighted avg 0.886421 0.886431 0.886461 2077.0
The result of Model Number:Dataset Number:Featuregroup Number:Rescale of 25 : 1 : 9 : 0
f1-score precision recall support
AFIB 0.701432 0.708242 0.695187 204.0
GSVT 0.722458 0.719043 0.726097 300.0
SB 0.737088 0.736266 0.737914 892.0
SR 0.730603 0.731359 0.729851 681.0
macro avg 0.722895 0.723727 0.722262 2077.0
micro avg 0.729367 0.729367 0.729367 2077.0
weighted avg 0.729346 0.729417 0.729367 2077.0
The result of Model Number:Dataset Number:Featuregroup Number:Rescale of 25 : 1 : 9 : 1
f1-score precision recall support
AFIB 0.849318 0.830017 0.869596 875.0
GSVT 0.878425 0.892670 0.864699 691.0
SB 0.937184 0.937189 0.937184 1553.0
SR 0.913197 0.923601 0.903028 907.0
macro avg 0.894531 0.895869 0.893627 4026.0
micro avg 0.902359 0.902359 0.902359 4026.0
weighted avg 0.902598 0.903194 0.902359 4026.0
The result of Model Number:Dataset Number:Featuregroup Number:Rescale of 25 : 1 : 10 : 0
f1-score precision recall support
AFIB 0.716592 0.716405 0.716849 875.0
GSVT 0.731562 0.728796 0.734428 691.0
SB 0.778858 0.775342 0.782418 1553.0

SR 0.752609 0.761208 0.744231 907.0
macro avg 0.744905 0.745438 0.744482 4026.0
micro avg 0.751328 0.751328 0.751328 4026.0
weighted avg 0.751294 0.751359 0.751328 4026.0
The result of Model Number:Dataset Number:Featuregroup Number:Rescale of 25 : 1 : 10 : 1

	f1-score	precision	recall	support
AFIB	0.899791	0.905177	0.894516	204.0
GSVT	0.923761	0.921015	0.926530	300.0
SB	0.937907	0.936757	0.939062	892.0
SR	0.931069	0.932168	0.929981	681.0
macro avg	0.923132	0.923779	0.922522	2077.0
micro avg	0.929899	0.929899	0.929899	2077.0
weighted avg	0.929878	0.929877	0.929899	2077.0

The result of Model Number:Dataset Number:Featuregroup Number:Rescale of 26 : 0 : 0 : 0

	f1-score	precision	recall	support
AFIB	0.787507	0.785230	0.789869	875.0
GSVT	0.802102	0.801450	0.802811	691.0
SB	0.851949	0.848420	0.855514	1553.0
SR	0.826372	0.835451	0.817534	907.0
macro avg	0.816983	0.817638	0.816432	4026.0
micro avg	0.823645	0.823645	0.823645	4026.0
weighted avg	0.823626	0.823703	0.823645	4026.0

The result of Model Number:Dataset Number:Featuregroup Number:Rescale of 26 : 0 : 0 : 1

	f1-score	precision	recall	support
AFIB	0.857149	0.868599	0.846239	204.0
GSVT	0.872291	0.866978	0.877804	300.0
SB	0.893318	0.892526	0.894118	892.0
SR	0.886717	0.886852	0.886589	681.0
macro avg	0.877369	0.878739	0.876188	2077.0
micro avg	0.884590	0.884590	0.884590	2077.0
weighted avg	0.884564	0.884625	0.884590	2077.0

The result of Model Number:Dataset Number:Featuregroup Number:Rescale of 26 : 0 : 1 : 0

	f1-score	precision	recall	support
AFIB	0.759611	0.758702	0.760650	875.0
GSVT	0.774418	0.777082	0.771852	691.0
SB	0.820224	0.815171	0.825346	1553.0
SR	0.803532	0.811380	0.795924	907.0
macro avg	0.789446	0.790584	0.788443	4026.0
micro avg	0.795475	0.795475	0.795475	4026.0
weighted avg	0.795428	0.795507	0.795475	4026.0

The result of Model Number:Dataset Number:Featuregroup Number:Rescale of 26 : 0 : 1 : 1

	f1-score	precision	recall	support
AFIB	0.758577	0.760905	0.756383	204.0
GSVT	0.778504	0.776526	0.780563	300.0
SB	0.795896	0.794923	0.796875	892.0
SR	0.789381	0.790899	0.787876	681.0
macro avg	0.780590	0.780813	0.780424	2077.0
micro avg	0.787591	0.787591	0.787591	2077.0
weighted avg	0.787582	0.787605	0.787591	2077.0

The result of Model Number:Dataset Number:Featuregroup Number:Rescale of 26 : 0 : 2 : 0

	f1-score	precision	recall	support
AFIB	0.824268	0.828033	0.820583	875.0
GSVT	0.838010	0.830922	0.845280	691.0
SB	0.885793	0.876748	0.895032	1553.0
SR	0.840077	0.857787	0.823129	907.0
macro avg	0.847037	0.848373	0.846006	4026.0
micro avg	0.854114	0.854114	0.854114	4026.0
weighted avg	0.853921	0.854024	0.854114	4026.0

The result of Model Number:Dataset Number:Featuregroup Number:Rescale of 26 : 0 : 2 : 1

	f1-score	precision	recall	support
AFIB	0.752448	0.743531	0.761644	875.0
GSVT	0.763157	0.770535	0.755994	691.0
SB	0.809964	0.806866	0.813093	1553.0
SR	0.791081	0.800208	0.782183	907.0
macro avg	0.779163	0.780285	0.778229	4026.0
micro avg	0.785148	0.785148	0.785148	4026.0
weighted avg	0.785176	0.785365	0.785148	4026.0

The result of Model Number:Dataset Number:Featuregroup Number:Rescale of 26 : 0 : 3 : 0

	f1-score	precision	recall	support
AFIB	0.813517	0.813662	0.813407	875.0
GSVT	0.822124	0.824901	0.819417	691.0
SB	0.863598	0.860621	0.866601	1553.0
SR	0.847199	0.850057	0.844392	907.0
macro avg	0.836609	0.837310	0.835954	4026.0
micro avg	0.841938	0.841938	0.841938	4026.0
weighted avg	0.841901	0.841904	0.841938	4026.0

The result of Model Number:Dataset Number:Featuregroup Number:Rescale of 26 : 0 : 3 : 1

	f1-score	precision	recall	support
AFIB	0.875819	0.864090	0.887927	875.0

GSVT 0.895401 0.902390 0.888535 691.0
SB 0.950666 0.947692 0.953664 1553.0
SR 0.927677 0.939975 0.915712 907.0
macro avg 0.912391 0.913537 0.911460 4026.0
micro avg 0.919649 0.919649 0.919649 4026.0
weighted avg 0.919734 0.920008 0.919649 4026.0
The result of Model Number:Dataset Number:Featuregroup Number:Rescale of 26 : 0 : 4 : 0

	f1-score	precision	recall	support
AFIB	0.730611	0.721522	0.740013	875.0
GSVT	0.753485	0.762920	0.744355	691.0
SB	0.803863	0.802992	0.804742	1553.0
SR	0.782182	0.786193	0.778215	907.0
macro avg	0.767535	0.768407	0.766831	4026.0
micro avg	0.774333	0.774333	0.774333	4026.0
weighted avg	0.774411	0.774623	0.774333	4026.0

The result of Model Number:Dataset Number:Featuregroup Number:Rescale of 26 : 0 : 4 : 1

	f1-score	precision	recall	support
AFIB	0.879337	0.900732	0.859151	204.0
GSVT	0.890391	0.878133	0.903114	300.0
SB	0.908370	0.899298	0.917632	892.0
SR	0.892510	0.904222	0.881123	681.0
macro avg	0.892652	0.895597	0.890255	2077.0
micro avg	0.897821	0.897821	0.897821	2077.0
weighted avg	0.897721	0.897997	0.897821	2077.0

The result of Model Number:Dataset Number:Featuregroup Number:Rescale of 26 : 0 : 5 : 0

	f1-score	precision	recall	support
AFIB	0.840223	0.834544	0.846093	875.0
GSVT	0.860721	0.861064	0.860490	691.0
SB	0.914341	0.912008	0.916696	1553.0
SR	0.889445	0.899382	0.879737	907.0
macro avg	0.876183	0.876750	0.875754	4026.0
micro avg	0.883378	0.883378	0.883378	4026.0
weighted avg	0.883421	0.883584	0.883378	4026.0

The result of Model Number:Dataset Number:Featuregroup Number:Rescale of 26 : 0 : 5 : 1

	f1-score	precision	recall	support
AFIB	0.742994	0.733248	0.753036	875.0
GSVT	0.754369	0.762754	0.746179	691.0
SB	0.800105	0.798217	0.802008	1553.0
SR	0.780073	0.787094	0.773187	907.0
macro avg	0.769385	0.770328	0.768603	4026.0

micro avg 0.775289 0.775289 0.775289 4026.0
weighted avg 0.775330 0.775504 0.775289 4026.0
The result of Model Number:Dataset Number:Featuregroup Number:Rescale of 26 : 0 : 6 : 0

	f1-score	precision	recall	support
AFIB	0.839240	0.848372	0.830547	204.0
GSVT	0.867640	0.864022	0.871430	300.0
SB	0.885420	0.884628	0.886214	892.0
SR	0.877053	0.877060	0.877053	681.0
macro avg	0.867338	0.868520	0.866311	2077.0
micro avg	0.875607	0.875607	0.875607	2077.0
weighted avg	0.875573	0.875609	0.875607	2077.0

The result of Model Number:Dataset Number:Featuregroup Number:Rescale of 26 : 0 : 6 : 1

	f1-score	precision	recall	support
AFIB	0.687635	0.693186	0.682230	204.0
GSVT	0.703413	0.699964	0.706921	300.0
SB	0.717141	0.716346	0.717945	892.0
SR	0.711786	0.712733	0.710847	681.0
macro avg	0.704994	0.705557	0.704486	2077.0
micro avg	0.710517	0.710517	0.710517	2077.0
weighted avg	0.710504	0.710520	0.710517	2077.0

The result of Model Number:Dataset Number:Featuregroup Number:Rescale of 26 : 0 : 7 : 0

	f1-score	precision	recall	support
AFIB	0.737354	0.732370	0.742506	875.0
GSVT	0.755343	0.755644	0.755140	691.0
SB	0.802398	0.800350	0.804465	1553.0
SR	0.780550	0.789270	0.772030	907.0
macro avg	0.768911	0.769409	0.768535	4026.0
micro avg	0.775226	0.775226	0.775226	4026.0
weighted avg	0.775263	0.775406	0.775226	4026.0

The result of Model Number:Dataset Number:Featuregroup Number:Rescale of 26 : 0 : 7 : 1

	f1-score	precision	recall	support
AFIB	0.892615	0.904538	0.881254	204.0
GSVT	0.908384	0.902850	0.914125	300.0
SB	0.930281	0.929456	0.931113	892.0
SR	0.923407	0.923547	0.923273	681.0
macro avg	0.913672	0.915098	0.912441	2077.0
micro avg	0.921192	0.921192	0.921192	2077.0
weighted avg	0.921165	0.921228	0.921192	2077.0

The result of Model Number:Dataset Number:Featuregroup Number:Rescale of 26 : 0 : 8 : 0

	f1-score	precision	recall	support
AFIB	0.750858	0.761487	0.741138	204.0
GSVT	0.766447	0.752336	0.781239	300.0
SB	0.782783	0.774128	0.791647	892.0
SR	0.766332	0.781940	0.751381	681.0
macro avg	0.766605	0.767473	0.766351	2077.0
micro avg	0.771980	0.771980	0.771980	2077.0
weighted avg	0.771894	0.772300	0.771980	2077.0

The result of Model Number:Dataset Number:Featuregroup Number:Rescale of 26 : 0 : 8 : 1

	f1-score	precision	recall	support
AFIB	0.853322	0.917469	0.798448	204.0
GSVT	0.813787	0.839618	0.789622	300.0
SB	0.847851	0.805896	0.894421	892.0
SR	0.750538	0.782798	0.720909	681.0
macro avg	0.816374	0.836445	0.800850	2077.0
micro avg	0.812967	0.812967	0.812967	2077.0
weighted avg	0.811562	0.814152	0.812967	2077.0

The result of Model Number:Dataset Number:Featuregroup Number:Rescale of 26 : 0 : 9 : 0

	f1-score	precision	recall	support
AFIB	0.667557	0.653216	0.682577	875.0
GSVT	0.691325	0.701351	0.681628	691.0
SB	0.735943	0.735803	0.736086	1553.0
SR	0.717498	0.725837	0.709352	907.0
macro avg	0.703081	0.704052	0.702411	4026.0
micro avg	0.709087	0.709087	0.709087	4026.0
weighted avg	0.709267	0.709696	0.709087	4026.0

The result of Model Number:Dataset Number:Featuregroup Number:Rescale of 26 : 0 : 9 : 1

	f1-score	precision	recall	support
AFIB	0.662452	0.663732	0.661227	875.0
GSVT	0.641804	0.638985	0.644687	691.0
SB	0.670609	0.629849	0.717028	1553.0
SR	0.539746	0.625409	0.474851	907.0
macro avg	0.628653	0.639494	0.624448	4026.0
micro avg	0.637925	0.637925	0.637925	4026.0
weighted avg	0.634411	0.637781	0.637925	4026.0

The result of Model Number:Dataset Number:Featuregroup Number:Rescale of 26 : 0 : 10 : 0

	f1-score	precision	recall	support
AFIB	0.782249	0.782947	0.781633	875.0
GSVT	0.796578	0.798668	0.794633	691.0
SB	0.845578	0.839948	0.851293	1553.0

SR 0.829103 0.836672 0.821699 907.0
macro avg 0.813377 0.814559 0.812314 4026.0
micro avg 0.819761 0.819761 0.819761 4026.0
weighted avg 0.819693 0.819737 0.819761 4026.0
The result of Model Number:Dataset Number:Featuregroup Number:Rescale of 26 : 0 : 10 : 1

	f1-score	precision	recall	support
AFIB	0.867171	0.861501	0.872993	875.0
GSVT	0.892946	0.900543	0.885594	691.0
SB	0.950661	0.948591	0.952742	1553.0
SR	0.927493	0.931217	0.923812	907.0
macro avg	0.909568	0.910463	0.908785	4026.0
micro avg	0.917367	0.917367	0.917367	4026.0
weighted avg	0.917390	0.917502	0.917367	4026.0

The result of Model Number:Dataset Number:Featuregroup Number:Rescale of 26 : 1 : 0 : 0

	f1-score	precision	recall	support
AFIB	0.754314	0.754850	0.753794	875.0
GSVT	0.764479	0.765986	0.763043	691.0
SB	0.812005	0.809307	0.814724	1553.0
SR	0.792925	0.795873	0.790026	907.0
macro avg	0.780931	0.781504	0.780397	4026.0
micro avg	0.787047	0.787047	0.787047	4026.0
weighted avg	0.787011	0.787009	0.787047	4026.0

The result of Model Number:Dataset Number:Featuregroup Number:Rescale of 26 : 1 : 0 : 1

	f1-score	precision	recall	support
AFIB	0.778450	0.769759	0.787349	875.0
GSVT	0.793938	0.801210	0.786809	691.0
SB	0.846771	0.844014	0.849550	1553.0
SR	0.824820	0.833083	0.816722	907.0
macro avg	0.810995	0.812016	0.810108	4026.0
micro avg	0.817867	0.817867	0.817867	4026.0
weighted avg	0.817909	0.818066	0.817867	4026.0

The result of Model Number:Dataset Number:Featuregroup Number:Rescale of 26 : 1 : 1 : 0

	f1-score	precision	recall	support
AFIB	0.724084	0.740458	0.708440	875.0
GSVT	0.767693	0.770961	0.764499	691.0
SB	0.833556	0.829628	0.837527	1553.0
SR	0.789783	0.777668	0.802314	907.0
macro avg	0.778779	0.779679	0.778195	4026.0
micro avg	0.789004	0.789004	0.789004	4026.0
weighted avg	0.788598	0.788473	0.789004	4026.0

The result of Model Number:Dataset Number:Featuregroup Number:Rescale of 26 : 1 : 1 : 1

	f1-score	precision	recall	support
AFIB	0.674163	0.680229	0.668416	875.0
GSVT	0.687896	0.673948	0.702596	691.0
SB	0.729518	0.721403	0.737907	1553.0
SR	0.700570	0.721395	0.681079	907.0
macro avg	0.698037	0.699244	0.697499	4026.0
micro avg	0.703941	0.703941	0.703941	4026.0
weighted avg	0.703822	0.704308	0.703941	4026.0

The result of Model Number:Dataset Number:Featuregroup Number:Rescale of 26 : 1 : 2 : 0

	f1-score	precision	recall	support
AFIB	0.766491	0.781344	0.752536	204.0
GSVT	0.796214	0.789556	0.803131	300.0
SB	0.811070	0.809443	0.812706	892.0
SR	0.803336	0.804169	0.802509	681.0
macro avg	0.794278	0.796128	0.792721	2077.0
micro avg	0.802070	0.802070	0.802070	2077.0
weighted avg	0.802010	0.802081	0.802070	2077.0

The result of Model Number:Dataset Number:Featuregroup Number:Rescale of 26 : 1 : 2 : 1

	f1-score	precision	recall	support
AFIB	0.854947	0.878516	0.832782	204.0
GSVT	0.877700	0.861452	0.894656	300.0
SB	0.891934	0.883596	0.900434	892.0
SR	0.869515	0.881328	0.858030	681.0
macro avg	0.873524	0.876223	0.871475	2077.0
micro avg	0.879051	0.879051	0.879051	2077.0
weighted avg	0.878895	0.879155	0.879051	2077.0

The result of Model Number:Dataset Number:Featuregroup Number:Rescale of 26 : 1 : 3 : 0

	f1-score	precision	recall	support
AFIB	0.900314	0.909083	0.891930	204.0
GSVT	0.921133	0.916904	0.925433	300.0
SB	0.933862	0.930649	0.937105	892.0
SR	0.925976	0.929568	0.922455	681.0
macro avg	0.920321	0.921551	0.919231	2077.0
micro avg	0.926178	0.926178	0.926178	2077.0
weighted avg	0.926143	0.926191	0.926178	2077.0

The result of Model Number:Dataset Number:Featuregroup Number:Rescale of 26 : 1 : 3 : 1

	f1-score	precision	recall	support
AFIB	0.745438	0.736645	0.754480	875.0

GSVT 0.758065 0.762862 0.753356 691.0
SB 0.808244 0.807157 0.809337 1553.0
SR 0.788388 0.795857 0.781084 907.0
macro avg 0.775034 0.775630 0.774564 4026.0
micro avg 0.781441 0.781441 0.781441 4026.0
weighted avg 0.781508 0.781684 0.781441 4026.0
The result of Model Number:Dataset Number:Featuregroup Number:Rescale of 26 : 1 : 4 : 0

	f1-score	precision	recall	support
AFIB	0.825251	0.826551	0.824063	204.0
GSVT	0.845210	0.843891	0.846604	300.0
SB	0.862646	0.861593	0.863707	892.0
SR	0.856605	0.858249	0.854971	681.0
macro avg	0.847428	0.847571	0.847336	2077.0
micro avg	0.854479	0.854479	0.854479	2077.0
weighted avg	0.854474	0.854498	0.854479	2077.0

The result of Model Number:Dataset Number:Featuregroup Number:Rescale of 26 : 1 : 4 : 1

	f1-score	precision	recall	support
AFIB	0.667397	0.670445	0.664413	875.0
GSVT	0.678523	0.672785	0.684410	691.0
SB	0.717213	0.709889	0.724693	1553.0
SR	0.680197	0.694536	0.666474	907.0
macro avg	0.685833	0.686914	0.684998	4026.0
micro avg	0.691562	0.691562	0.691562	4026.0
weighted avg	0.691406	0.691489	0.691562	4026.0

The result of Model Number:Dataset Number:Featuregroup Number:Rescale of 26 : 1 : 5 : 0

	f1-score	precision	recall	support
AFIB	0.734755	0.752208	0.718160	204.0
GSVT	0.758889	0.751774	0.766220	300.0
SB	0.775187	0.774320	0.776056	892.0
SR	0.767705	0.766934	0.768490	681.0
macro avg	0.759134	0.761309	0.757232	2077.0
micro avg	0.766468	0.766468	0.766468	2077.0
weighted avg	0.766409	0.766470	0.766468	2077.0

The result of Model Number:Dataset Number:Featuregroup Number:Rescale of 26 : 1 : 5 : 1

	f1-score	precision	recall	support
AFIB	0.757101	0.772289	0.742649	204.0
GSVT	0.774647	0.766595	0.782911	300.0
SB	0.788974	0.785905	0.792069	892.0
SR	0.780683	0.783941	0.777471	681.0
macro avg	0.775351	0.777182	0.773775	2077.0

micro avg 0.781106 0.781106 0.781106 2077.0
weighted avg 0.781056 0.781134 0.781106 2077.0
The result of Model Number:Dataset Number:Featuregroup Number:Rescale of 26 : 1 : 6 : 0

	f1-score	precision	recall	support
AFIB	0.689263	0.693046	0.685565	875.0
GSVT	0.690227	0.681500	0.699288	691.0
SB	0.725893	0.722980	0.728835	1553.0
SR	0.710310	0.718933	0.701936	907.0
macro avg	0.703923	0.704115	0.703906	4026.0
micro avg	0.708300	0.708300	0.708300	4026.0
weighted avg	0.708300	0.708443	0.708300	4026.0

The result of Model Number:Dataset Number:Featuregroup Number:Rescale of 26 : 1 : 6 : 1

	f1-score	precision	recall	support
AFIB	0.841590	0.860077	0.824197	204.0
GSVT	0.868713	0.861691	0.875968	300.0
SB	0.886919	0.885828	0.888013	892.0
SR	0.879432	0.878667	0.880204	681.0
macro avg	0.869164	0.871566	0.867096	2077.0
micro avg	0.877445	0.877445	0.877445	2077.0
weighted avg	0.877382	0.877465	0.877445	2077.0

The result of Model Number:Dataset Number:Featuregroup Number:Rescale of 26 : 1 : 7 : 0

	f1-score	precision	recall	support
AFIB	0.681100	0.687382	0.675096	204.0
GSVT	0.706000	0.706025	0.705994	300.0
SB	0.720786	0.719506	0.722077	892.0
SR	0.713136	0.712945	0.713344	681.0
macro avg	0.705255	0.706464	0.704128	2077.0
micro avg	0.712276	0.712276	0.712276	2077.0
weighted avg	0.712244	0.712252	0.712276	2077.0

The result of Model Number:Dataset Number:Featuregroup Number:Rescale of 26 : 1 : 7 : 1

	f1-score	precision	recall	support
AFIB	0.843942	0.850013	0.837959	875.0
GSVT	0.842597	0.837552	0.847717	691.0
SB	0.888817	0.878969	0.898890	1553.0
SR	0.852889	0.868105	0.838224	907.0
macro avg	0.857061	0.858660	0.855698	4026.0
micro avg	0.863197	0.863197	0.863197	4026.0
weighted avg	0.863037	0.863120	0.863197	4026.0

The result of Model Number:Dataset Number:Featuregroup Number:Rescale of 26 : 1 : 8 : 0

	f1-score	precision	recall	support
AFIB	0.855976	0.862088	0.850104	204.0
GSVT	0.885907	0.885336	0.886496	300.0
SB	0.903632	0.901627	0.905655	892.0
SR	0.895555	0.896638	0.894501	681.0
macro avg	0.885267	0.886422	0.884189	2077.0
micro avg	0.893774	0.893774	0.893774	2077.0
weighted avg	0.893743	0.893755	0.893774	2077.0

The result of Model Number:Dataset Number:Featuregroup Number:Rescale of 26 : 1 : 8 : 1

	f1-score	precision	recall	support
AFIB	0.713958	0.718231	0.709772	204.0
GSVT	0.732977	0.730798	0.735174	300.0
SB	0.744201	0.743289	0.745118	892.0
SR	0.738776	0.739648	0.737912	681.0
macro avg	0.732478	0.732991	0.731994	2077.0
micro avg	0.737847	0.737847	0.737847	2077.0
weighted avg	0.737831	0.737830	0.737847	2077.0

The result of Model Number:Dataset Number:Featuregroup Number:Rescale of 26 : 1 : 9 : 0

	f1-score	precision	recall	support
AFIB	0.661025	0.660700	0.661402	875.0
GSVT	0.675375	0.672052	0.678773	691.0
SB	0.706454	0.701836	0.711139	1553.0
SR	0.686343	0.697523	0.675535	907.0
macro avg	0.682299	0.683028	0.681712	4026.0
micro avg	0.686753	0.686753	0.686753	4026.0
weighted avg	0.686716	0.686812	0.686753	4026.0

The result of Model Number:Dataset Number:Featuregroup Number:Rescale of 26 : 1 : 9 : 1

	f1-score	precision	recall	support
AFIB	0.705332	0.707123	0.703577	875.0
GSVT	0.717779	0.710004	0.725756	691.0
SB	0.756500	0.747942	0.765267	1553.0
SR	0.721911	0.741675	0.703202	907.0
macro avg	0.725381	0.726686	0.724451	4026.0
micro avg	0.731096	0.731096	0.731096	4026.0
weighted avg	0.730941	0.731147	0.731096	4026.0

The result of Model Number:Dataset Number:Featuregroup Number:Rescale of 26 : 1 : 10 : 0

	f1-score	precision	recall	support
AFIB	0.730012	0.743646	0.808235	204.0
GSVT	0.876668	0.858909	0.895621	300.0
SB	0.721189	0.714451	0.728063	892.0

SR	0.877787	0.885120	0.870696	681.0
macro avg	0.801414	0.800531	0.825654	2077.0
micro avg	0.806906	0.806906	0.806906	2077.0
weighted avg	0.795858	0.794142	0.806906	2077.0

The result of Model Number:Dataset Number:Featuregroup Number:Rescale of 26 : 1 : 10 : 1

	f1-score	precision	recall	support
AFIB	0.907531	0.920918	0.894626	204.0
GSVT	0.929810	0.926160	0.933518	300.0
SB	0.950460	0.948554	0.952377	892.0
SR	0.943718	0.943861	0.943585	681.0
macro avg	0.932880	0.934873	0.931027	2077.0
micro avg	0.941098	0.941098	0.941098	2077.0
weighted avg	0.941051	0.941066	0.941098	2077.0

The result of Model Number:Dataset Number:Featuregroup Number:Rescale of 27 : 0 : 0 : 0

	f1-score	precision	recall	support
AFIB	0.754799	0.771884	0.738551	204.0
GSVT	0.774885	0.771091	0.778751	300.0
SB	0.790738	0.788454	0.793040	892.0
SR	0.785657	0.785318	0.786009	681.0
macro avg	0.776520	0.779187	0.774088	2077.0
micro avg	0.783319	0.783319	0.783319	2077.0
weighted avg	0.783252	0.783290	0.783319	2077.0

The result of Model Number:Dataset Number:Featuregroup Number:Rescale of 27 : 0 : 0 : 1

	f1-score	precision	recall	support
AFIB	0.806237	0.814831	0.797955	204.0
GSVT	0.821064	0.816560	0.825710	300.0
SB	0.840017	0.838617	0.841427	892.0
SR	0.833015	0.834372	0.831673	681.0
macro avg	0.825083	0.826095	0.824191	2077.0
micro avg	0.831689	0.831689	0.831689	2077.0
weighted avg	0.831666	0.831703	0.831689	2077.0

The result of Model Number:Dataset Number:Featuregroup Number:Rescale of 27 : 0 : 1 : 0

	f1-score	precision	recall	support
AFIB	0.910176	0.922229	0.898571	204.0
GSVT	0.937642	0.934258	0.941072	300.0
SB	0.949321	0.946367	0.952298	892.0
SR	0.938977	0.940786	0.937202	681.0
macro avg	0.934029	0.935910	0.932286	2077.0
micro avg	0.940450	0.940450	0.940450	2077.0
weighted avg	0.940398	0.940417	0.940450	2077.0

The result of Model Number:Dataset Number:Featuregroup Number:Rescale of 27 : 0 : 1 : 1

	f1-score	precision	recall	support
AFIB	0.752136	0.758209	0.746225	204.0
GSVT	0.769395	0.765622	0.773231	300.0
SB	0.784411	0.783541	0.785290	892.0
SR	0.778554	0.779589	0.777526	681.0
macro avg	0.771124	0.771740	0.770568	2077.0
micro avg	0.777166	0.777166	0.777166	2077.0
weighted avg	0.777152	0.777169	0.777166	2077.0

The result of Model Number:Dataset Number:Featuregroup Number:Rescale of 27 : 0 : 2 : 0

	f1-score	precision	recall	support
AFIB	0.820881	0.843983	0.799180	204.0
GSVT	0.842104	0.836193	0.848257	300.0
SB	0.865261	0.861799	0.868753	892.0
SR	0.853761	0.854294	0.853253	681.0
macro avg	0.845502	0.849067	0.842361	2077.0
micro avg	0.853877	0.853877	0.853877	2077.0
weighted avg	0.853787	0.853890	0.853877	2077.0

The result of Model Number:Dataset Number:Featuregroup Number:Rescale of 27 : 0 : 2 : 1

	f1-score	precision	recall	support
AFIB	0.792907	0.826271	0.762204	204.0
GSVT	0.819510	0.806966	0.832574	300.0
SB	0.839276	0.833257	0.845393	892.0
SR	0.817494	0.821651	0.813410	681.0
macro avg	0.817297	0.822036	0.813395	2077.0
micro avg	0.824884	0.824884	0.824884	2077.0
weighted avg	0.824725	0.824968	0.824884	2077.0

The result of Model Number:Dataset Number:Featuregroup Number:Rescale of 27 : 0 : 3 : 0

	f1-score	precision	recall	support
AFIB	0.765393	0.802499	0.731652	204.0
GSVT	0.797270	0.786853	0.808129	300.0
SB	0.818743	0.812517	0.825077	892.0
SR	0.795947	0.798325	0.793618	681.0
macro avg	0.794338	0.800048	0.789619	2077.0
micro avg	0.803138	0.803138	0.803138	2077.0
weighted avg	0.802927	0.803173	0.803138	2077.0

The result of Model Number:Dataset Number:Featuregroup Number:Rescale of 27 : 0 : 3 : 1

	f1-score	precision	recall	support
AFIB	0.669453	0.670747	0.668215	875.0

GSVT 0.648588 0.645738 0.651501 691.0
SB 0.677697 0.636506 0.724606 1553.0
SR 0.545451 0.632019 0.479870 907.0
macro avg 0.635297 0.646253 0.631048 4026.0
micro avg 0.644667 0.644667 0.644667 4026.0
weighted avg 0.641116 0.644522 0.644667 4026.0
The result of Model Number:Dataset Number:Featuregroup Number:Rescale of 27 : 0 : 4 : 0

	f1-score	precision	recall	support
AFIB	0.794170	0.796852	0.791534	875.0
GSVT	0.792073	0.786494	0.797803	691.0
SB	0.833406	0.825671	0.841293	1553.0
SR	0.801722	0.817172	0.786884	907.0
macro avg	0.805343	0.806547	0.804379	4026.0
micro avg	0.810757	0.810757	0.810757	4026.0
weighted avg	0.810646	0.810769	0.810757	4026.0

The result of Model Number:Dataset Number:Featuregroup Number:Rescale of 27 : 0 : 4 : 1

	f1-score	precision	recall	support
AFIB	0.824740	0.853372	0.798079	204.0
GSVT	0.840554	0.831352	0.850065	300.0
SB	0.862931	0.856735	0.869219	892.0
SR	0.847519	0.851686	0.843407	681.0
macro avg	0.843936	0.848286	0.840193	2077.0
micro avg	0.851002	0.851002	0.851002	2077.0
weighted avg	0.850895	0.851083	0.851002	2077.0

The result of Model Number:Dataset Number:Featuregroup Number:Rescale of 27 : 0 : 5 : 0

	f1-score	precision	recall	support
AFIB	0.767123	0.756338	0.778258	875.0
GSVT	0.783639	0.789519	0.777867	691.0
SB	0.833311	0.830862	0.835778	1553.0
SR	0.812784	0.824029	0.801856	907.0
macro avg	0.799214	0.800187	0.798440	4026.0
micro avg	0.805695	0.805695	0.805695	4026.0
weighted avg	0.805776	0.806030	0.805695	4026.0

The result of Model Number:Dataset Number:Featuregroup Number:Rescale of 27 : 0 : 5 : 1

	f1-score	precision	recall	support
AFIB	0.822751	0.845905	0.801000	204.0
GSVT	0.844021	0.838097	0.850189	300.0
SB	0.867232	0.863762	0.870731	892.0
SR	0.855705	0.856240	0.855196	681.0
macro avg	0.847427	0.851001	0.844279	2077.0

micro avg 0.855821 0.855821 0.855821 2077.0
weighted avg 0.855731 0.855834 0.855821 2077.0
The result of Model Number:Dataset Number:Featuregroup Number:Rescale of 27 : 0 : 6 : 0

	f1-score	precision	recall	support
AFIB	0.680195	0.673642	0.686982	875.0
GSVT	0.698016	0.700013	0.696103	691.0
SB	0.742111	0.740404	0.743831	1553.0
SR	0.721235	0.729722	0.712959	907.0
macro avg	0.710389	0.710945	0.709969	4026.0
micro avg	0.716329	0.716329	0.716329	4026.0
weighted avg	0.716383	0.716555	0.716329	4026.0

The result of Model Number:Dataset Number:Featuregroup Number:Rescale of 27 : 0 : 6 : 1

	f1-score	precision	recall	support
AFIB	0.920299	0.942176	0.899518	204.0
GSVT	0.944191	0.940548	0.947962	300.0
SB	0.967588	0.965429	0.969757	892.0
SR	0.955415	0.953493	0.957379	681.0
macro avg	0.946873	0.950411	0.943654	2077.0
micro avg	0.955652	0.955652	0.955652	2077.0
weighted avg	0.955573	0.955638	0.955652	2077.0

The result of Model Number:Dataset Number:Featuregroup Number:Rescale of 27 : 0 : 7 : 0

	f1-score	precision	recall	support
AFIB	0.714419	0.718167	0.710753	875.0
GSVT	0.729357	0.728047	0.730724	691.0
SB	0.791957	0.785884	0.798127	1553.0
SR	0.764016	0.771663	0.756524	907.0
macro avg	0.749937	0.750940	0.749032	4026.0
micro avg	0.758196	0.758196	0.758196	4026.0
weighted avg	0.758066	0.758036	0.758196	4026.0

The result of Model Number:Dataset Number:Featuregroup Number:Rescale of 27 : 0 : 7 : 1

	f1-score	precision	recall	support
AFIB	0.826886	0.842730	0.811837	204.0
GSVT	0.853181	0.848786	0.857700	300.0
SB	0.874281	0.872919	0.875653	892.0
SR	0.865666	0.864806	0.866555	681.0
macro avg	0.855003	0.857310	0.852936	2077.0
micro avg	0.863809	0.863809	0.863809	2077.0
weighted avg	0.863753	0.863808	0.863809	2077.0

The result of Model Number:Dataset Number:Featuregroup Number:Rescale of 27 : 0 : 8 : 0

	f1-score	precision	recall	support
AFIB	0.853721	0.894708	0.816495	204.0
GSVT	0.890854	0.883290	0.898574	300.0
SB	0.904854	0.898264	0.911547	892.0
SR	0.895336	0.895751	0.894948	681.0
macro avg	0.886191	0.893003	0.880391	2077.0
micro avg	0.894895	0.894895	0.894895	2077.0
weighted avg	0.894689	0.894928	0.894895	2077.0

The result of Model Number:Dataset Number:Featuregroup Number:Rescale of 27 : 0 : 8 : 1

	f1-score	precision	recall	support
AFIB	0.741807	0.808991	0.686162	204.0
GSVT	0.861002	0.851164	0.871336	300.0
SB	0.913042	0.910705	0.915396	892.0
SR	0.890687	0.878626	0.903110	681.0
macro avg	0.851635	0.862371	0.844001	2077.0
micro avg	0.882489	0.882489	0.882489	2077.0
weighted avg	0.881377	0.881597	0.882489	2077.0

The result of Model Number:Dataset Number:Featuregroup Number:Rescale of 27 : 0 : 9 : 0

	f1-score	precision	recall	support
AFIB	0.660377	0.662313	0.658503	875.0
GSVT	0.671304	0.662682	0.680240	691.0
SB	0.710464	0.708737	0.712202	1553.0
SR	0.692474	0.700692	0.684454	907.0
macro avg	0.683655	0.683606	0.683850	4026.0
micro avg	0.688794	0.688794	0.688794	4026.0
weighted avg	0.688804	0.688930	0.688794	4026.0

The result of Model Number:Dataset Number:Featuregroup Number:Rescale of 27 : 0 : 9 : 1

	f1-score	precision	recall	support
AFIB	0.661883	0.663823	0.660005	875.0
GSVT	0.672835	0.664193	0.681792	691.0
SB	0.712084	0.710353	0.713826	1553.0
SR	0.694053	0.702290	0.686014	907.0
macro avg	0.685214	0.685165	0.685409	4026.0
micro avg	0.690365	0.690365	0.690365	4026.0
weighted avg	0.690375	0.690501	0.690365	4026.0

The result of Model Number:Dataset Number:Featuregroup Number:Rescale of 27 : 0 : 10 : 0

	f1-score	precision	recall	support
AFIB	0.667105	0.679957	0.655045	204.0
GSVT	0.679632	0.668590	0.691170	300.0
SB	0.690979	0.682738	0.699423	892.0

SR 0.678125 0.690839 0.665882 681.0
macro avg 0.678960 0.680531 0.677880 2077.0
micro avg 0.682875 0.682875 0.682875 2077.0
weighted avg 0.682781 0.683077 0.682875 2077.0
The result of Model Number:Dataset Number:Featuregroup Number:Rescale of 27 : 0 : 10 : 1

	f1-score	precision	recall	support
AFIB	0.750213	0.735268	0.765817	875.0
GSVT	0.610607	0.567832	0.765379	691.0
SB	0.495440	0.496294	0.494590	1553.0
SR	0.803172	0.810245	0.796256	907.0
macro avg	0.664858	0.652410	0.705510	4026.0
micro avg	0.667975	0.667975	0.667975	4026.0
weighted avg	0.639906	0.631239	0.667975	4026.0

The result of Model Number:Dataset Number:Featuregroup Number:Rescale of 27 : 1 : 0 : 0

	f1-score	precision	recall	support
AFIB	0.918563	0.935415	0.902379	204.0
GSVT	0.930678	0.921286	0.940283	300.0
SB	0.953603	0.948639	0.958625	892.0
SR	0.940621	0.946515	0.934820	681.0
macro avg	0.935866	0.937964	0.934027	2077.0
micro avg	0.942646	0.942646	0.942646	2077.0
weighted avg	0.942594	0.942693	0.942646	2077.0

The result of Model Number:Dataset Number:Featuregroup Number:Rescale of 27 : 1 : 0 : 1

	f1-score	precision	recall	support
AFIB	0.874708	0.895646	0.856006	204.0
GSVT	0.900466	0.887778	0.913897	300.0
SB	0.923862	0.914476	0.933485	892.0
SR	0.901781	0.914802	0.889197	681.0
macro avg	0.900204	0.903175	0.898146	2077.0
micro avg	0.908525	0.908525	0.908525	2077.0
weighted avg	0.908415	0.908877	0.908525	2077.0

The result of Model Number:Dataset Number:Featuregroup Number:Rescale of 27 : 1 : 1 : 0

	f1-score	precision	recall	support
AFIB	0.897348	0.905529	0.889432	204.0
GSVT	0.920766	0.917751	0.923829	300.0
SB	0.937598	0.936973	0.938228	892.0
SR	0.929352	0.929085	0.929633	681.0
macro avg	0.921266	0.922335	0.920280	2077.0
micro avg	0.928537	0.928537	0.928537	2077.0
weighted avg	0.928510	0.928522	0.928537	2077.0

The result of Model Number:Dataset Number:Featuregroup Number:Rescale of 27 : 1 : 1 : 1

	f1-score	precision	recall	support
AFIB	0.809179	0.807877	0.810816	875.0
GSVT	0.828046	0.823729	0.832455	691.0
SB	0.865765	0.858430	0.873233	1553.0
SR	0.832715	0.851228	0.815271	907.0
macro avg	0.833926	0.835316	0.832944	4026.0
micro avg	0.839611	0.839611	0.839611	4026.0
weighted avg	0.839547	0.839865	0.839611	4026.0

The result of Model Number:Dataset Number:Featuregroup Number:Rescale of 27 : 1 : 2 : 0

	f1-score	precision	recall	support
AFIB	0.835730	0.863484	0.810022	204.0
GSVT	0.867447	0.860487	0.874649	300.0
SB	0.886612	0.882969	0.890288	892.0
SR	0.878331	0.878202	0.878461	681.0
macro avg	0.867030	0.871285	0.863355	2077.0
micro avg	0.876268	0.876268	0.876268	2077.0
weighted avg	0.876131	0.876245	0.876268	2077.0

The result of Model Number:Dataset Number:Featuregroup Number:Rescale of 27 : 1 : 2 : 1

	f1-score	precision	recall	support
AFIB	0.694771	0.712664	0.677783	204.0
GSVT	0.637574	0.609193	0.719502	300.0
SB	0.731481	0.729770	0.733203	892.0
SR	0.580298	0.580407	0.580196	681.0
macro avg	0.661031	0.658008	0.677671	2077.0
micro avg	0.675613	0.675613	0.675613	2077.0
weighted avg	0.664742	0.661701	0.675613	2077.0

The result of Model Number:Dataset Number:Featuregroup Number:Rescale of 27 : 1 : 3 : 0

	f1-score	precision	recall	support
AFIB	0.182964	0.184885	0.181082	175.0
GSVT	0.183259	0.181312	0.185248	138.2
SB	0.193003	0.192263	0.193749	310.6
SR	0.188537	0.189482	0.187601	181.4
macro avg	0.186941	0.186985	0.186920	805.2
micro avg	0.188152	0.188152	0.188152	805.2
weighted avg	0.188143	0.188153	0.188152	805.2

The result of Model Number:Dataset Number:Featuregroup Number:Rescale of 27 : 1 : 3 : 1

	f1-score	precision	recall	support
AFIB	0.698634	0.711205	0.686707	204.0

GSVT 0.714395 0.704351 0.724849 300.0
SB 0.725388 0.716817 0.734169 892.0
SR 0.710799 0.723454 0.698587 681.0
macro avg 0.712304 0.713957 0.711078 2077.0
micro avg 0.716495 0.716495 0.716495 2077.0
weighted avg 0.716389 0.716641 0.716495 2077.0
The result of Model Number:Dataset Number:Featuregroup Number:Rescale of 27 : 1 : 4 : 0

	f1-score	precision	recall	support
AFIB	0.764816	0.759999	0.861512	204.0
GSVT	0.932323	0.927527	0.937325	300.0
SB	0.958869	0.954307	0.963486	892.0
SR	0.752125	0.754603	0.750116	681.0
macro avg	0.852033	0.849109	0.878110	2077.0
micro avg	0.879732	0.879732	0.879732	2077.0
weighted avg	0.868188	0.865876	0.879732	2077.0

The result of Model Number:Dataset Number:Featuregroup Number:Rescale of 27 : 1 : 4 : 1

	f1-score	precision	recall	support
AFIB	0.893408	0.892410	0.894435	875.0
GSVT	0.909750	0.898371	0.921447	691.0
SB	0.955687	0.948004	0.963503	1553.0
SR	0.913049	0.937005	0.890302	907.0
macro avg	0.917973	0.918948	0.917422	4026.0
micro avg	0.924783	0.924783	0.924783	4026.0
weighted avg	0.924661	0.924925	0.924783	4026.0

The result of Model Number:Dataset Number:Featuregroup Number:Rescale of 27 : 1 : 5 : 0

	f1-score	precision	recall	support
AFIB	0.869142	0.857069	0.881643	875.0
GSVT	0.900201	0.912210	0.888621	691.0
SB	0.956306	0.954347	0.958277	1553.0
SR	0.931416	0.938613	0.924330	907.0
macro avg	0.914266	0.915560	0.913218	4026.0
micro avg	0.922018	0.922018	0.922018	4026.0
weighted avg	0.922125	0.922428	0.922018	4026.0

The result of Model Number:Dataset Number:Featuregroup Number:Rescale of 27 : 1 : 5 : 1

	f1-score	precision	recall	support
AFIB	0.745610	0.794191	0.702960	204.0
GSVT	0.778274	0.759144	0.798499	300.0
SB	0.801099	0.790808	0.811689	892.0
SR	0.780291	0.789608	0.771243	681.0
macro avg	0.776319	0.783438	0.771098	2077.0

micro avg 0.785843 0.785843 0.785843 2077.0
weighted avg 0.785530 0.786173 0.785843 2077.0
The result of Model Number:Dataset Number:Featuregroup Number:Rescale of 27 : 1 : 6 : 0

	f1-score	precision	recall	support
AFIB	0.801306	0.803766	0.798989	204.0
GSVT	0.822356	0.820266	0.824530	300.0
SB	0.840727	0.839700	0.841761	892.0
SR	0.833846	0.835449	0.832255	681.0
macro avg	0.824559	0.824795	0.824384	2077.0
micro avg	0.831955	0.831955	0.831955	2077.0
weighted avg	0.831945	0.831969	0.831955	2077.0

The result of Model Number:Dataset Number:Featuregroup Number:Rescale of 27 : 1 : 6 : 1

	f1-score	precision	recall	support
AFIB	0.667763	0.659175	0.676625	875.0
GSVT	0.681356	0.686997	0.675835	691.0
SB	0.728506	0.725264	0.731786	1553.0
SR	0.707775	0.718243	0.697624	907.0
macro avg	0.696350	0.697419	0.695468	4026.0
micro avg	0.702498	0.702498	0.702498	4026.0
weighted avg	0.702541	0.702750	0.702498	4026.0

The result of Model Number:Dataset Number:Featuregroup Number:Rescale of 27 : 1 : 7 : 0

	f1-score	precision	recall	support
AFIB	0.707978	0.702641	0.803545	204.0
GSVT	0.858284	0.853202	0.863435	300.0
SB	0.701956	0.700492	0.703431	892.0
SR	0.868156	0.866766	0.869560	681.0
macro avg	0.784094	0.780775	0.809993	2077.0
micro avg	0.790845	0.790845	0.790845	2077.0
weighted avg	0.779621	0.777278	0.790845	2077.0

The result of Model Number:Dataset Number:Featuregroup Number:Rescale of 27 : 1 : 7 : 1

	f1-score	precision	recall	support
AFIB	0.867319	0.877461	0.857570	204.0
GSVT	0.782326	0.752458	0.892622	300.0
SB	0.725838	0.724128	0.727564	892.0
SR	0.901246	0.902979	0.899527	681.0
macro avg	0.819182	0.814256	0.844321	2077.0
micro avg	0.820557	0.820557	0.820557	2077.0
weighted avg	0.805405	0.801921	0.820557	2077.0

The result of Model Number:Dataset Number:Featuregroup Number:Rescale of 27 : 1 : 8 : 0

	f1-score	precision	recall	support
AFIB	0.723427	0.729093	0.717887	875.0
GSVT	0.725335	0.716078	0.734879	691.0
SB	0.760794	0.757743	0.763878	1553.0
SR	0.744721	0.752031	0.737579	907.0
macro avg	0.738569	0.738736	0.738556	4026.0
micro avg	0.742981	0.742981	0.742981	4026.0
weighted avg	0.742966	0.743078	0.742981	4026.0

The result of Model Number:Dataset Number:Featuregroup Number:Rescale of 27 : 1 : 8 : 1

	f1-score	precision	recall	support
AFIB	0.713528	0.723357	0.704277	875.0
GSVT	0.729105	0.726086	0.732347	691.0
SB	0.776611	0.766428	0.787100	1553.0
SR	0.752228	0.763462	0.741655	907.0
macro avg	0.742868	0.744833	0.741345	4026.0
micro avg	0.749464	0.749464	0.749464	4026.0
weighted avg	0.749254	0.749475	0.749464	4026.0

The result of Model Number:Dataset Number:Featuregroup Number:Rescale of 27 : 1 : 9 : 0

	f1-score	precision	recall	support
AFIB	0.753139	0.755010	0.751328	875.0
GSVT	0.766109	0.768411	0.763918	691.0
SB	0.812704	0.807187	0.818301	1553.0
SR	0.797239	0.803157	0.791437	907.0
macro avg	0.782297	0.783441	0.781246	4026.0
micro avg	0.788359	0.788359	0.788359	4026.0
weighted avg	0.788277	0.788284	0.788359	4026.0

The result of Model Number:Dataset Number:Featuregroup Number:Rescale of 27 : 1 : 9 : 1

	f1-score	precision	recall	support
AFIB	0.857700	0.900554	0.819171	204.0
GSVT	0.883558	0.867184	0.900870	300.0
SB	0.903778	0.894668	0.913095	892.0
SR	0.878713	0.886562	0.871082	681.0
macro avg	0.880937	0.887242	0.876054	2077.0
micro avg	0.888329	0.888329	0.888329	2077.0
weighted avg	0.888114	0.888619	0.888329	2077.0

The result of Model Number:Dataset Number:Featuregroup Number:Rescale of 27 : 1 : 10 : 0

	f1-score	precision	recall	support
AFIB	0.809448	0.817278	0.801913	204.0
GSVT	0.838090	0.836438	0.839763	300.0
SB	0.855608	0.854092	0.857140	892.0

SR 0.847720 0.848130 0.847346 681.0
macro avg 0.837717 0.838985 0.836541 2077.0
micro avg 0.845995 0.845995 0.845995 2077.0
weighted avg 0.845958 0.845972 0.845995 2077.0
The result of Model Number:Dataset Number:Featuregroup Number:Rescale of 27 : 1 : 10 : 1

	f1-score	precision	recall	support
AFIB	0.824625	0.824213	0.825073	875.0
GSVT	0.834882	0.840468	0.829437	691.0
SB	0.885090	0.883056	0.887138	1553.0
SR	0.859454	0.859250	0.859807	907.0
macro avg	0.851013	0.851747	0.850364	4026.0
micro avg	0.857588	0.857588	0.857588	4026.0
weighted avg	0.857556	0.857595	0.857588	4026.0

The result of Model Number:Dataset Number:Featuregroup Number:Rescale of 28 : 0 : 0 : 0

	f1-score	precision	recall	support
AFIB	0.725200	0.754470	0.698173	204.0
GSVT	0.752187	0.739968	0.764946	300.0
SB	0.771646	0.765442	0.777961	892.0
SR	0.750886	0.756293	0.745595	681.0
macro avg	0.749980	0.754043	0.746669	2077.0
micro avg	0.757632	0.757632	0.757632	2077.0
weighted avg	0.757467	0.757685	0.757632	2077.0

The result of Model Number:Dataset Number:Featuregroup Number:Rescale of 28 : 0 : 0 : 1

	f1-score	precision	recall	support
AFIB	0.687046	0.709861	0.665911	204.0
GSVT	0.713119	0.707398	0.719041	300.0
SB	0.728875	0.725880	0.731897	892.0
SR	0.722067	0.721962	0.722174	681.0
macro avg	0.712777	0.716275	0.709756	2077.0
micro avg	0.720371	0.720371	0.720371	2077.0
weighted avg	0.720259	0.720352	0.720371	2077.0

The result of Model Number:Dataset Number:Featuregroup Number:Rescale of 28 : 0 : 1 : 0

	f1-score	precision	recall	support
AFIB	0.817720	0.831918	0.804103	204.0
GSVT	0.833834	0.831366	0.836334	300.0
SB	0.843667	0.840665	0.846693	892.0
SR	0.836807	0.837681	0.835954	681.0
macro avg	0.833007	0.835408	0.830771	2077.0
micro avg	0.837492	0.837492	0.837492	2077.0
weighted avg	0.837449	0.837484	0.837492	2077.0

The result of Model Number:Dataset Number:Featuregroup Number:Rescale of 28 : 0 : 1 : 1

	f1-score	precision	recall	support
AFIB	0.774056	0.807231	0.743794	204.0
GSVT	0.800067	0.787979	0.812583	300.0
SB	0.815936	0.807866	0.824170	892.0
SR	0.793830	0.800621	0.787183	681.0
macro avg	0.795972	0.800924	0.791932	2077.0
micro avg	0.802475	0.802475	0.802475	2077.0
weighted avg	0.802282	0.802556	0.802475	2077.0

The result of Model Number:Dataset Number:Featuregroup Number:Rescale of 28 : 0 : 2 : 0

	f1-score	precision	recall	support
AFIB	0.871649	0.863674	0.879810	875.0
GSVT	0.882817	0.890607	0.875295	691.0
SB	0.938407	0.933459	0.943420	1553.0
SR	0.917193	0.928246	0.906484	907.0
macro avg	0.902516	0.903997	0.901252	4026.0
micro avg	0.909582	0.909582	0.909582	4026.0
weighted avg	0.909578	0.909763	0.909582	4026.0

The result of Model Number:Dataset Number:Featuregroup Number:Rescale of 28 : 0 : 2 : 1

	f1-score	precision	recall	support
AFIB	0.757155	0.762256	0.752233	875.0
GSVT	0.770667	0.759963	0.781819	691.0
SB	0.821230	0.815606	0.826938	1553.0
SR	0.787950	0.801561	0.774837	907.0
macro avg	0.784250	0.784846	0.783957	4026.0
micro avg	0.791220	0.791220	0.791220	4026.0
weighted avg	0.791128	0.791297	0.791220	4026.0

The result of Model Number:Dataset Number:Featuregroup Number:Rescale of 28 : 0 : 3 : 0

	f1-score	precision	recall	support
AFIB	0.696867	0.690334	0.703614	875.0
GSVT	0.660697	0.644817	0.709266	691.0
SB	0.763973	0.761167	0.766820	1553.0
SR	0.594204	0.598371	0.590103	907.0
macro avg	0.678935	0.673672	0.692451	4026.0
micro avg	0.703393	0.703393	0.703393	4026.0
weighted avg	0.693416	0.689127	0.703393	4026.0

The result of Model Number:Dataset Number:Featuregroup Number:Rescale of 28 : 0 : 3 : 1

	f1-score	precision	recall	support
AFIB	0.737593	0.756903	0.719581	204.0

GSVT 0.694025 0.714195 0.675340 300.0
SB 0.732344 0.706560 0.760095 892.0
SR 0.663781 0.685398 0.643619 681.0
macro avg 0.706936 0.715764 0.699659 2077.0
micro avg 0.705684 0.705684 0.705684 2077.0
weighted avg 0.704845 0.705669 0.705684 2077.0
The result of Model Number:Dataset Number:Featuregroup Number:Rescale of 28 : 0 : 4 : 0

	f1-score	precision	recall	support
AFIB	0.859071	0.885530	0.834319	204.0
GSVT	0.880987	0.870202	0.892139	300.0
SB	0.904916	0.899539	0.910388	892.0
SR	0.889663	0.894051	0.885353	681.0
macro avg	0.883659	0.887331	0.880550	2077.0
micro avg	0.892072	0.892072	0.892072	2077.0
weighted avg	0.891956	0.892127	0.892072	2077.0

The result of Model Number:Dataset Number:Featuregroup Number:Rescale of 28 : 0 : 4 : 1

	f1-score	precision	recall	support
AFIB	0.775322	0.764583	0.786399	875.0
GSVT	0.792518	0.798932	0.786211	691.0
SB	0.841249	0.838831	0.843685	1553.0
SR	0.820784	0.831466	0.810380	907.0
macro avg	0.807468	0.808453	0.806669	4026.0
micro avg	0.813867	0.813867	0.813867	4026.0
weighted avg	0.813946	0.814187	0.813867	4026.0

The result of Model Number:Dataset Number:Featuregroup Number:Rescale of 28 : 0 : 5 : 0

	f1-score	precision	recall	support
AFIB	0.762105	0.778091	0.746784	204.0
GSVT	0.779785	0.775171	0.784462	300.0
SB	0.798795	0.796924	0.800675	892.0
SR	0.792048	0.791820	0.792283	681.0
macro avg	0.783183	0.785502	0.781051	2077.0
micro avg	0.790289	0.790289	0.790289	2077.0
weighted avg	0.790233	0.790259	0.790289	2077.0

The result of Model Number:Dataset Number:Featuregroup Number:Rescale of 28 : 0 : 5 : 1

	f1-score	precision	recall	support
AFIB	0.767451	0.758497	0.778620	875.0
GSVT	0.789661	0.780089	0.800106	691.0
SB	0.801563	0.770622	0.846947	1553.0
SR	0.636483	0.670056	0.606151	907.0
macro avg	0.748789	0.744816	0.757956	4026.0

micro avg 0.769810 0.769810 0.769810 4026.0
weighted avg 0.754916 0.746956 0.769810 4026.0
The result of Model Number:Dataset Number:Featuregroup Number:Rescale of 28 : 0 : 6 : 0

	f1-score	precision	recall	support
AFIB	0.806954	0.826588	0.788430	204.0
GSVT	0.817098	0.805850	0.828774	300.0
SB	0.833597	0.825273	0.842097	892.0
SR	0.819043	0.829791	0.808593	681.0
macro avg	0.819173	0.821875	0.816974	2077.0
micro avg	0.823916	0.823916	0.823916	2077.0
weighted avg	0.823825	0.824078	0.823916	2077.0

The result of Model Number:Dataset Number:Featuregroup Number:Rescale of 28 : 0 : 6 : 1

	f1-score	precision	recall	support
AFIB	0.622956	0.624160	0.621804	875.0
GSVT	0.603539	0.600888	0.606250	691.0
SB	0.630627	0.592297	0.674278	1553.0
SR	0.507566	0.588122	0.446541	907.0
macro avg	0.591172	0.601367	0.587218	4026.0
micro avg	0.599892	0.599892	0.599892	4026.0
weighted avg	0.596587	0.599756	0.599892	4026.0

The result of Model Number:Dataset Number:Featuregroup Number:Rescale of 28 : 0 : 7 : 0

	f1-score	precision	recall	support
AFIB	0.657390	0.657565	0.657329	875.0
GSVT	0.668837	0.666368	0.671409	691.0
SB	0.715525	0.713788	0.717274	1553.0
SR	0.698461	0.703474	0.693538	907.0
macro avg	0.685053	0.685299	0.684888	4026.0
micro avg	0.691026	0.691026	0.691026	4026.0
weighted avg	0.691033	0.691106	0.691026	4026.0

The result of Model Number:Dataset Number:Featuregroup Number:Rescale of 28 : 0 : 7 : 1

	f1-score	precision	recall	support
AFIB	0.791743	0.827495	0.759210	204.0
GSVT	0.819280	0.810079	0.828842	300.0
SB	0.788117	0.753757	0.843297	892.0
SR	0.656610	0.662208	0.651217	681.0
macro avg	0.763937	0.763385	0.770641	2077.0
micro avg	0.769971	0.769971	0.769971	2077.0
weighted avg	0.749856	0.739118	0.769971	2077.0

The result of Model Number:Dataset Number:Featuregroup Number:Rescale of 28 : 0 : 8 : 0

	f1-score	precision	recall	support
AFIB	0.682242	0.739195	0.634797	204.0
GSVT	0.731784	0.722582	0.741521	300.0
SB	0.750465	0.739952	0.761369	892.0
SR	0.729994	0.733414	0.726962	681.0
macro avg	0.723621	0.733786	0.716162	2077.0
micro avg	0.734789	0.734789	0.734789	2077.0
weighted avg	0.734354	0.735225	0.734789	2077.0

The result of Model Number:Dataset Number:Featuregroup Number:Rescale of 28 : 0 : 8 : 1

	f1-score	precision	recall	support
AFIB	0.758214	0.756987	0.759512	875.0
GSVT	0.765161	0.768204	0.762181	691.0
SB	0.803552	0.800631	0.806501	1553.0
SR	0.790409	0.794407	0.786494	907.0
macro avg	0.779334	0.780057	0.778672	4026.0
micro avg	0.784174	0.784174	0.784174	4026.0
weighted avg	0.784148	0.784178	0.784174	4026.0

The result of Model Number:Dataset Number:Featuregroup Number:Rescale of 28 : 0 : 9 : 0

	f1-score	precision	recall	support
AFIB	0.833570	0.851088	0.816791	875.0
GSVT	0.887655	0.890704	0.884670	691.0
SB	0.967112	0.962554	0.971718	1553.0
SR	0.915480	0.903288	0.928082	907.0
macro avg	0.900954	0.901909	0.900315	4026.0
micro avg	0.913276	0.913276	0.913276	4026.0
weighted avg	0.912819	0.912645	0.913276	4026.0

The result of Model Number:Dataset Number:Featuregroup Number:Rescale of 28 : 0 : 9 : 1

	f1-score	precision	recall	support
AFIB	0.826153	0.839888	0.813050	204.0
GSVT	0.841995	0.836013	0.848126	300.0
SB	0.861525	0.856683	0.866441	892.0
SR	0.850549	0.855753	0.845435	681.0
macro avg	0.845055	0.847084	0.843263	2077.0
micro avg	0.851664	0.851664	0.851664	2077.0
weighted avg	0.851631	0.851743	0.851664	2077.0

The result of Model Number:Dataset Number:Featuregroup Number:Rescale of 28 : 0 : 10 : 0

	f1-score	precision	recall	support
AFIB	0.825146	0.821791	0.828550	875.0
GSVT	0.837208	0.842269	0.832239	691.0
SB	0.882993	0.881918	0.884073	1553.0

SR 0.864572 0.865942 0.863232 907.0
macro avg 0.852480 0.852980 0.852024 4026.0
micro avg 0.858414 0.858414 0.858414 4026.0
weighted avg 0.858412 0.858446 0.858414 4026.0
The result of Model Number:Dataset Number:Featuregroup Number:Rescale of 28 : 0 : 10 : 1

	f1-score	precision	recall	support
AFIB	0.729948	0.731097	0.728897	204.0
GSVT	0.747601	0.746435	0.748835	300.0
SB	0.763024	0.762093	0.763963	892.0
SR	0.757680	0.759134	0.756235	681.0
macro avg	0.749563	0.749690	0.749482	2077.0
micro avg	0.755800	0.755800	0.755800	2077.0
weighted avg	0.755796	0.755817	0.755800	2077.0

The result of Model Number:Dataset Number:Featuregroup Number:Rescale of 28 : 1 : 0 : 0

	f1-score	precision	recall	support
AFIB	0.757347	0.765588	0.749503	204.0
GSVT	0.782977	0.779712	0.786396	300.0
SB	0.799022	0.798307	0.799738	892.0
SR	0.791470	0.791477	0.791470	681.0
macro avg	0.782704	0.783771	0.781777	2077.0
micro avg	0.790166	0.790166	0.790166	2077.0
weighted avg	0.790135	0.790168	0.790166	2077.0

The result of Model Number:Dataset Number:Featuregroup Number:Rescale of 28 : 1 : 0 : 1

	f1-score	precision	recall	support
AFIB	0.688316	0.707290	0.670470	204.0
GSVT	0.706634	0.693553	0.720285	300.0
SB	0.718094	0.711381	0.724937	892.0
SR	0.700044	0.709555	0.690797	681.0
macro avg	0.703272	0.705445	0.701622	2077.0
micro avg	0.707722	0.707722	0.707722	2077.0
weighted avg	0.707596	0.707805	0.707722	2077.0

The result of Model Number:Dataset Number:Featuregroup Number:Rescale of 28 : 1 : 1 : 0

	f1-score	precision	recall	support
AFIB	0.774905	0.779543	0.770361	204.0
GSVT	0.795547	0.793183	0.797932	300.0
SB	0.807730	0.806740	0.808725	892.0
SR	0.801842	0.802788	0.800904	681.0
macro avg	0.795006	0.795563	0.794481	2077.0
micro avg	0.800834	0.800834	0.800834	2077.0
weighted avg	0.800816	0.800815	0.800834	2077.0

The result of Model Number:Dataset Number:Featuregroup Number:Rescale of 28 : 1 : 1 : 1

	f1-score	precision	recall	support
AFIB	0.870270	0.859957	0.880906	875.0
GSVT	0.882657	0.891189	0.874372	691.0
SB	0.936793	0.933209	0.940412	1553.0
SR	0.914953	0.925509	0.904661	907.0
macro avg	0.901168	0.902466	0.900088	4026.0
micro avg	0.908090	0.908090	0.908090	4026.0
weighted avg	0.908123	0.908342	0.908090	4026.0

The result of Model Number:Dataset Number:Featuregroup Number:Rescale of 28 : 1 : 2 : 0

	f1-score	precision	recall	support
AFIB	0.855935	0.874737	0.838245	204.0
GSVT	0.883520	0.876379	0.890899	300.0
SB	0.902037	0.900927	0.903150	892.0
SR	0.894422	0.893644	0.895207	681.0
macro avg	0.883978	0.886422	0.881875	2077.0
micro avg	0.892401	0.892401	0.892401	2077.0
weighted avg	0.892337	0.892421	0.892401	2077.0

The result of Model Number:Dataset Number:Featuregroup Number:Rescale of 28 : 1 : 2 : 1

	f1-score	precision	recall	support
AFIB	0.676445	0.666848	0.686338	875.0
GSVT	0.690361	0.697090	0.683767	691.0
SB	0.734679	0.732285	0.737091	1553.0
SR	0.717522	0.726693	0.708588	907.0
macro avg	0.704752	0.705729	0.703946	4026.0
micro avg	0.710487	0.710487	0.710487	4026.0
weighted avg	0.710551	0.710763	0.710487	4026.0

The result of Model Number:Dataset Number:Featuregroup Number:Rescale of 28 : 1 : 3 : 0

	f1-score	precision	recall	support
AFIB	0.718948	0.710311	0.727825	875.0
GSVT	0.732776	0.738709	0.726942	691.0
SB	0.781082	0.778093	0.784099	1553.0
SR	0.762769	0.772522	0.753268	907.0
macro avg	0.748894	0.749909	0.748034	4026.0
micro avg	0.755113	0.755113	0.755113	4026.0
weighted avg	0.755162	0.755347	0.755113	4026.0

The result of Model Number:Dataset Number:Featuregroup Number:Rescale of 28 : 1 : 3 : 1

	f1-score	precision	recall	support
AFIB	0.710406	0.710744	0.710170	875.0

GSVT 0.728804 0.722495 0.735249 691.0
SB 0.778365 0.772693 0.784128 1553.0
SR 0.745112 0.759924 0.730905 907.0
macro avg 0.740672 0.741464 0.740113 4026.0
micro avg 0.747674 0.747674 0.747674 4026.0
weighted avg 0.747597 0.747737 0.747674 4026.0
The result of Model Number:Dataset Number:Featuregroup Number:Rescale of 28 : 1 : 4 : 0
f1-score precision recall support
AFIB 0.680333 0.678241 0.682482 875.0
GSVT 0.662991 0.666242 0.659876 691.0
SB 0.696989 0.668065 0.728537 1553.0
SR 0.599817 0.654434 0.553661 907.0
macro avg 0.660033 0.666746 0.656139 4026.0
micro avg 0.667346 0.667346 0.667346 4026.0
weighted avg 0.665642 0.666893 0.667346 4026.0
The result of Model Number:Dataset Number:Featuregroup Number:Rescale of 28 : 1 : 4 : 1
f1-score precision recall support
AFIB 0.752446 0.762810 0.742690 875.0
GSVT 0.768872 0.765688 0.772291 691.0
SB 0.818969 0.808231 0.830030 1553.0
SR 0.793257 0.805103 0.782106 907.0
macro avg 0.783386 0.785458 0.781779 4026.0
micro avg 0.790341 0.790341 0.790341 4026.0
weighted avg 0.790120 0.790353 0.790341 4026.0
The result of Model Number:Dataset Number:Featuregroup Number:Rescale of 28 : 1 : 5 : 0
f1-score precision recall support
AFIB 0.627406 0.643053 0.612550 875.0
GSVT 0.667451 0.670009 0.664942 691.0
SB 0.722793 0.719293 0.726329 1553.0
SR 0.684886 0.673471 0.696740 907.0
macro avg 0.675634 0.676457 0.675140 4026.0
micro avg 0.684398 0.684398 0.684398 4026.0
weighted avg 0.684023 0.683941 0.684398 4026.0
The result of Model Number:Dataset Number:Featuregroup Number:Rescale of 28 : 1 : 5 : 1
f1-score precision recall support
AFIB 0.845294 0.849728 0.840957 875.0
GSVT 0.862969 0.861419 0.864586 691.0
SB 0.937037 0.929851 0.944337 1553.0
SR 0.903977 0.913025 0.895112 907.0
macro avg 0.887319 0.888506 0.886248 4026.0

micro avg 0.897091 0.897091 0.897091 4026.0
weighted avg 0.896937 0.896901 0.897091 4026.0
The result of Model Number:Dataset Number:Featuregroup Number:Rescale of 28 : 1 : 6 : 0

	f1-score	precision	recall	support
AFIB	0.750399	0.736371	0.765051	875.0
GSVT	0.776684	0.788946	0.764895	691.0
SB	0.825971	0.824495	0.827459	1553.0
SR	0.804976	0.813504	0.796630	907.0
macro avg	0.789508	0.790829	0.788509	4026.0
micro avg	0.796212	0.796212	0.796212	4026.0
weighted avg	0.796357	0.796765	0.796212	4026.0

The result of Model Number:Dataset Number:Featuregroup Number:Rescale of 28 : 1 : 6 : 1

	f1-score	precision	recall	support
AFIB	0.830228	0.822533	0.838115	875.0
GSVT	0.845138	0.849728	0.840612	691.0
SB	0.895472	0.893408	0.897547	1553.0
SR	0.874608	0.882679	0.866711	907.0
macro avg	0.861362	0.862087	0.860746	4026.0
micro avg	0.867911	0.867911	0.867911	4026.0
weighted avg	0.867953	0.868090	0.867911	4026.0

The result of Model Number:Dataset Number:Featuregroup Number:Rescale of 28 : 1 : 7 : 0

	f1-score	precision	recall	support
AFIB	0.867194	0.878920	0.856139	204.0
GSVT	0.900133	0.898672	0.901630	300.0
SB	0.919680	0.918455	0.920917	892.0
SR	0.910280	0.909114	0.911481	681.0
macro avg	0.899322	0.901290	0.897542	2077.0
micro avg	0.908675	0.908675	0.908675	2077.0
weighted avg	0.908619	0.908652	0.908675	2077.0

The result of Model Number:Dataset Number:Featuregroup Number:Rescale of 28 : 1 : 7 : 1

	f1-score	precision	recall	support
AFIB	0.750099	0.748507	0.751732	875.0
GSVT	0.761818	0.758166	0.765569	691.0
SB	0.801810	0.792238	0.811618	1553.0
SR	0.764548	0.786209	0.744066	907.0
macro avg	0.769569	0.771280	0.768246	4026.0
micro avg	0.775480	0.775480	0.775480	4026.0
weighted avg	0.775313	0.775528	0.775480	4026.0

The result of Model Number:Dataset Number:Featuregroup Number:Rescale of 28 : 1 : 8 : 0

	f1-score	precision	recall	support
AFIB	0.581928	0.645821	0.529679	204.0
GSVT	0.679480	0.670985	0.688326	300.0
SB	0.722933	0.719244	0.726661	892.0
SR	0.705998	0.696127	0.716155	681.0
macro avg	0.672585	0.683044	0.665205	2077.0
micro avg	0.698332	0.698332	0.698332	2077.0
weighted avg	0.697255	0.697483	0.698332	2077.0

The result of Model Number:Dataset Number:Featuregroup Number:Rescale of 28 : 1 : 8 : 1

	f1-score	precision	recall	support
AFIB	0.771627	0.764803	0.778607	875.0
GSVT	0.701543	0.679391	0.780481	691.0
SB	0.632479	0.607267	0.671906	1553.0
SR	0.651862	0.658607	0.645296	907.0
macro avg	0.689378	0.677517	0.719072	4026.0
micro avg	0.707736	0.707736	0.707736	4026.0
weighted avg	0.678942	0.665451	0.707736	4026.0

The result of Model Number:Dataset Number:Featuregroup Number:Rescale of 28 : 1 : 9 : 0

	f1-score	precision	recall	support
AFIB	0.846161	0.867952	0.825471	204.0
GSVT	0.776501	0.741936	0.876280	300.0
SB	0.890870	0.888786	0.892967	892.0
SR	0.706744	0.706877	0.706620	681.0
macro avg	0.805069	0.801388	0.825335	2077.0
micro avg	0.822829	0.822829	0.822829	2077.0
weighted avg	0.809589	0.805885	0.822829	2077.0

The result of Model Number:Dataset Number:Featuregroup Number:Rescale of 28 : 1 : 9 : 1

	f1-score	precision	recall	support
AFIB	0.819780	0.807528	0.832441	875.0
GSVT	0.835498	0.842644	0.828486	691.0
SB	0.892711	0.889637	0.895812	1553.0
SR	0.866581	0.879506	0.854060	907.0
macro avg	0.853643	0.854829	0.852700	4026.0
micro avg	0.861078	0.861078	0.861078	4026.0
weighted avg	0.861154	0.861444	0.861078	4026.0

The result of Model Number:Dataset Number:Featuregroup Number:Rescale of 28 : 1 : 10 : 0

	f1-score	precision	recall	support
AFIB	0.838828	0.837110	0.841161	875.0
GSVT	0.850270	0.841345	0.859540	691.0
SB	0.896750	0.884641	0.909369	1553.0

	f1-score	precision	recall	support
SR	0.853114	0.885648	0.823080	907.0
macro avg	0.859740	0.862186	0.858288	4026.0
micro avg	0.866553	0.866553	0.866553	4026.0
weighted avg	0.866353	0.867106	0.866553	4026.0

The result of Model Number:Dataset Number:Featuregroup Number:Rescale of 28 : 1 : 10 : 1

	f1-score	precision	recall	support
AFIB	0.830918	0.806083	0.885975	875.0
GSVT	0.711558	0.695976	0.728118	691.0
SB	0.943798	0.936108	0.951865	1553.0
SR	0.885418	0.922592	0.852785	907.0
macro avg	0.842923	0.840190	0.854686	4026.0
micro avg	0.876821	0.876821	0.876821	4026.0
weighted avg	0.866252	0.863589	0.876821	4026.0

The result of Model Number:Dataset Number:Featuregroup Number:Rescale of 29 : 0 : 0 : 0

	f1-score	precision	recall	support
AFIB	0.812544	0.813121	0.811983	875.0
GSVT	0.823493	0.825117	0.821946	691.0
SB	0.874688	0.871781	0.877617	1553.0
SR	0.854135	0.857310	0.851012	907.0
macro avg	0.841215	0.841832	0.840639	4026.0
micro avg	0.847803	0.847803	0.847803	4026.0
weighted avg	0.847765	0.847763	0.847803	4026.0

The result of Model Number:Dataset Number:Featuregroup Number:Rescale of 29 : 0 : 0 : 1

	f1-score	precision	recall	support
AFIB	0.786983	0.770077	0.804690	875.0
GSVT	0.815003	0.826823	0.803571	691.0
SB	0.867603	0.867438	0.867772	1553.0
SR	0.845858	0.855690	0.836255	907.0
macro avg	0.828862	0.830007	0.828072	4026.0
micro avg	0.835943	0.835943	0.835943	4026.0
weighted avg	0.836155	0.836660	0.835943	4026.0

The result of Model Number:Dataset Number:Featuregroup Number:Rescale of 29 : 0 : 1 : 0

	f1-score	precision	recall	support
AFIB	0.821600	0.848286	0.796704	204.0
GSVT	0.844283	0.833518	0.855500	300.0
SB	0.868103	0.864829	0.871410	892.0
SR	0.855961	0.857511	0.854449	681.0
macro avg	0.847487	0.851036	0.844516	2077.0
micro avg	0.856214	0.856214	0.856214	2077.0
weighted avg	0.856114	0.856282	0.856214	2077.0

The result of Model Number:Dataset Number:Featuregroup Number:Rescale of 29 : 0 : 1 : 1

	f1-score	precision	recall	support
AFIB	0.858411	0.871284	0.846158	204.0
GSVT	0.889410	0.887048	0.891789	300.0
SB	0.910478	0.909265	0.911702	892.0
SR	0.901573	0.900411	0.902768	681.0
macro avg	0.889968	0.892002	0.888104	2077.0
micro avg	0.899459	0.899459	0.899459	2077.0
weighted avg	0.899401	0.899422	0.899459	2077.0

The result of Model Number:Dataset Number:Featuregroup Number:Rescale of 29 : 0 : 2 : 0

	f1-score	precision	recall	support
AFIB	0.680677	0.672648	0.688934	875.0
GSVT	0.692208	0.696588	0.687907	691.0
SB	0.738027	0.737035	0.739025	1553.0
SR	0.719896	0.726716	0.713227	907.0
macro avg	0.707702	0.708247	0.707273	4026.0
micro avg	0.713553	0.713553	0.713553	4026.0
weighted avg	0.713614	0.713774	0.713553	4026.0

The result of Model Number:Dataset Number:Featuregroup Number:Rescale of 29 : 0 : 2 : 1

	f1-score	precision	recall	support
AFIB	0.660383	0.646271	0.675163	875.0
GSVT	0.683868	0.694326	0.673782	691.0
SB	0.729002	0.729098	0.728909	1553.0
SR	0.710221	0.717576	0.703018	907.0
macro avg	0.695869	0.696818	0.695218	4026.0
micro avg	0.701933	0.701933	0.701933	4026.0
weighted avg	0.702111	0.702533	0.701933	4026.0

The result of Model Number:Dataset Number:Featuregroup Number:Rescale of 29 : 0 : 3 : 0

	f1-score	precision	recall	support
AFIB	0.790112	0.777462	0.889036	204.0
GSVT	0.925012	0.914279	0.936338	300.0
SB	0.955985	0.952375	0.959628	892.0
SR	0.750083	0.753961	0.746372	681.0
macro avg	0.855298	0.849519	0.882843	2077.0
micro avg	0.879409	0.879409	0.879409	2077.0
weighted avg	0.867709	0.864637	0.879409	2077.0

The result of Model Number:Dataset Number:Featuregroup Number:Rescale of 29 : 0 : 3 : 1

	f1-score	precision	recall	support
AFIB	0.790640	0.844624	0.743480	204.0

GSVT 0.835785 0.818754 0.853614 300.0
SB 0.856516 0.849007 0.864191 892.0
SR 0.835388 0.838170 0.832699 681.0
macro avg 0.829582 0.837639 0.823496 2077.0
micro avg 0.840482 0.840482 0.840482 2077.0
weighted avg 0.840124 0.840654 0.840482 2077.0
The result of Model Number:Dataset Number:Featuregroup Number:Rescale of 29 : 0 : 4 : 0
f1-score precision recall support
AFIB 0.816657 0.814984 0.818929 875.0
GSVT 0.827797 0.819107 0.836822 691.0
SB 0.873048 0.861259 0.885334 1553.0
SR 0.830565 0.862239 0.801325 907.0
macro avg 0.837017 0.839397 0.835602 4026.0
micro avg 0.843649 0.843649 0.843649 4026.0
weighted avg 0.843454 0.844188 0.843649 4026.0
The result of Model Number:Dataset Number:Featuregroup Number:Rescale of 29 : 0 : 4 : 1
f1-score precision recall support
AFIB 0.568653 0.620155 0.525997 204.0
GSVT 0.660026 0.652484 0.667948 300.0
SB 0.699918 0.698127 0.701723 892.0
SR 0.682782 0.673536 0.692304 681.0
macro avg 0.652845 0.661075 0.646993 2077.0
micro avg 0.676497 0.676497 0.676497 2077.0
weighted avg 0.675645 0.675813 0.676497 2077.0
The result of Model Number:Dataset Number:Featuregroup Number:Rescale of 29 : 0 : 5 : 0
f1-score precision recall support
AFIB 0.675010 0.691208 0.659861 204.0
GSVT 0.699011 0.694960 0.703195 300.0
SB 0.713940 0.711321 0.716580 892.0
SR 0.707736 0.708261 0.707220 681.0
macro avg 0.698924 0.701437 0.696714 2077.0
micro avg 0.706007 0.706007 0.706007 2077.0
weighted avg 0.705926 0.705979 0.706007 2077.0
The result of Model Number:Dataset Number:Featuregroup Number:Rescale of 29 : 0 : 5 : 1
f1-score precision recall support
AFIB 0.858831 0.863545 0.854224 875.0
GSVT 0.860032 0.849159 0.871322 691.0
SB 0.904473 0.900843 0.908139 1553.0
SR 0.885057 0.895801 0.874622 907.0
macro avg 0.877098 0.877337 0.877077 4026.0

micro avg 0.882552 0.882552 0.882552 4026.0
weighted avg 0.882551 0.882730 0.882552 4026.0
The result of Model Number:Dataset Number:Featuregroup Number:Rescale of 29 : 0 : 6 : 0

	f1-score	precision	recall	support
AFIB	0.791590	0.851097	0.740686	204.0
GSVT	0.754915	0.778878	0.732499	300.0
SB	0.786515	0.747595	0.829716	892.0
SR	0.696242	0.726169	0.668756	681.0
macro avg	0.757316	0.775935	0.742914	2077.0
micro avg	0.754155	0.754155	0.754155	2077.0
weighted avg	0.752851	0.755254	0.754155	2077.0

The result of Model Number:Dataset Number:Featuregroup Number:Rescale of 29 : 0 : 6 : 1

	f1-score	precision	recall	support
AFIB	0.913499	0.933820	0.894241	204.0
GSVT	0.952420	0.946501	0.958449	300.0
SB	0.973094	0.971045	0.975164	892.0
SR	0.962730	0.962049	0.963442	681.0
macro avg	0.950436	0.953354	0.947824	2077.0
micro avg	0.960958	0.960958	0.960958	2077.0
weighted avg	0.960856	0.960894	0.960958	2077.0

The result of Model Number:Dataset Number:Featuregroup Number:Rescale of 29 : 0 : 7 : 0

	f1-score	precision	recall	support
AFIB	0.815491	0.824365	0.807044	204.0
GSVT	0.843088	0.839573	0.846770	300.0
SB	0.860365	0.859595	0.861136	892.0
SR	0.852234	0.852241	0.852234	681.0
macro avg	0.842795	0.843943	0.841796	2077.0
micro avg	0.850830	0.850830	0.850830	2077.0
weighted avg	0.850796	0.850832	0.850830	2077.0

The result of Model Number:Dataset Number:Featuregroup Number:Rescale of 29 : 0 : 7 : 1

	f1-score	precision	recall	support
AFIB	0.860867	0.872266	0.849890	204.0
GSVT	0.886845	0.883644	0.890089	300.0
SB	0.897891	0.895097	0.900707	892.0
SR	0.888107	0.889818	0.886428	681.0
macro avg	0.883427	0.885206	0.881778	2077.0
micro avg	0.889500	0.889500	0.889500	2077.0
weighted avg	0.889451	0.889469	0.889500	2077.0

The result of Model Number:Dataset Number:Featuregroup Number:Rescale of 29 : 0 : 8 : 0

	f1-score	precision	recall	support
AFIB	0.866547	0.871489	0.861804	204.0
GSVT	0.884377	0.877998	0.890858	300.0
SB	0.902238	0.898942	0.905574	892.0
SR	0.892642	0.898503	0.886880	681.0
macro avg	0.886451	0.886733	0.886279	2077.0
micro avg	0.893020	0.893020	0.893020	2077.0
weighted avg	0.893006	0.893077	0.893020	2077.0

The result of Model Number:Dataset Number:Featuregroup Number:Rescale of 29 : 0 : 8 : 1

	f1-score	precision	recall	support
AFIB	0.809605	0.821746	0.798048	204.0
GSVT	0.838841	0.836614	0.841085	300.0
SB	0.858712	0.857567	0.859866	892.0
SR	0.850313	0.849217	0.851440	681.0
macro avg	0.839368	0.841286	0.837610	2077.0
micro avg	0.848319	0.848319	0.848319	2077.0
weighted avg	0.848265	0.848284	0.848319	2077.0

The result of Model Number:Dataset Number:Featuregroup Number:Rescale of 29 : 0 : 9 : 0

	f1-score	precision	recall	support
AFIB	0.760529	0.752038	0.769223	875.0
GSVT	0.775660	0.782765	0.768696	691.0
SB	0.827277	0.824583	0.829992	1553.0
SR	0.805831	0.813904	0.797920	907.0
macro avg	0.792324	0.793322	0.791458	4026.0
micro avg	0.799039	0.799039	0.799039	4026.0
weighted avg	0.799080	0.799233	0.799039	4026.0

The result of Model Number:Dataset Number:Featuregroup Number:Rescale of 29 : 0 : 9 : 1

	f1-score	precision	recall	support
AFIB	0.864879	0.892109	0.839473	204.0
GSVT	0.821681	0.839232	0.804986	300.0
SB	0.848404	0.818716	0.880357	892.0
SR	0.774807	0.800993	0.750334	681.0
macro avg	0.827443	0.837763	0.818788	2077.0
micro avg	0.822823	0.822823	0.822823	2077.0
weighted avg	0.822031	0.823077	0.822823	2077.0

The result of Model Number:Dataset Number:Featuregroup Number:Rescale of 29 : 0 : 10 : 0

	f1-score	precision	recall	support
AFIB	0.745258	0.747545	0.743103	204.0
GSVT	0.764835	0.762892	0.766857	300.0
SB	0.781921	0.780966	0.782883	892.0

	f1-score	precision	recall	support
SR	0.775521	0.777012	0.774042	681.0
macro avg	0.766884	0.767104	0.766721	2077.0
micro avg	0.773762	0.773762	0.773762	2077.0
weighted avg	0.773754	0.773776	0.773762	2077.0

The result of Model Number:Dataset Number:Featuregroup Number:Rescale of 29 : 0 : 10 : 1

	f1-score	precision	recall	support
AFIB	0.703782	0.697002	0.710804	875.0
GSVT	0.722221	0.724287	0.720242	691.0
SB	0.767846	0.766080	0.769625	1553.0
SR	0.746246	0.755026	0.737683	907.0
macro avg	0.735024	0.735599	0.734589	4026.0
micro avg	0.741169	0.741169	0.741169	4026.0
weighted avg	0.741225	0.741403	0.741169	4026.0

The result of Model Number:Dataset Number:Featuregroup Number:Rescale of 29 : 1 : 0 : 0

	f1-score	precision	recall	support
AFIB	0.779124	0.799712	0.759646	204.0
GSVT	0.800491	0.793841	0.807400	300.0
SB	0.822549	0.819531	0.825592	892.0
SR	0.809813	0.810795	0.808856	681.0
macro avg	0.802995	0.805970	0.800374	2077.0
micro avg	0.811000	0.811000	0.811000	2077.0
weighted avg	0.810922	0.811009	0.811000	2077.0

The result of Model Number:Dataset Number:Featuregroup Number:Rescale of 29 : 1 : 0 : 1

	f1-score	precision	recall	support
AFIB	0.730103	0.741052	0.719681	204.0
GSVT	0.756468	0.754459	0.758492	300.0
SB	0.774387	0.773355	0.775428	892.0
SR	0.766813	0.765824	0.767829	681.0
macro avg	0.756943	0.758673	0.755357	2077.0
micro avg	0.765015	0.765015	0.765015	2077.0
weighted avg	0.764966	0.764984	0.765015	2077.0

The result of Model Number:Dataset Number:Featuregroup Number:Rescale of 29 : 1 : 1 : 0

	f1-score	precision	recall	support
AFIB	0.807672	0.821695	0.794222	204.0
GSVT	0.823588	0.821151	0.826057	300.0
SB	0.833300	0.830335	0.836289	892.0
SR	0.826525	0.827388	0.825682	681.0
macro avg	0.822771	0.825142	0.820562	2077.0
micro avg	0.827201	0.827201	0.827201	2077.0
weighted avg	0.827158	0.827193	0.827201	2077.0

The result of Model Number:Dataset Number:Featuregroup Number:Rescale of 29 : 1 : 1 : 1

	f1-score	precision	recall	support
AFIB	0.664068	0.715826	0.619599	204.0
GSVT	0.684319	0.695903	0.673143	300.0
SB	0.725249	0.718575	0.732072	892.0
SR	0.696384	0.686285	0.706816	681.0
macro avg	0.692505	0.704147	0.682907	2077.0
micro avg	0.704232	0.704232	0.704232	2077.0
weighted avg	0.703864	0.704443	0.704232	2077.0

The result of Model Number:Dataset Number:Featuregroup Number:Rescale of 29 : 1 : 2 : 0

	f1-score	precision	recall	support
AFIB	0.733483	0.739880	0.727379	204.0
GSVT	0.758849	0.757610	0.760111	300.0
SB	0.774979	0.773089	0.776888	892.0
SR	0.767874	0.769031	0.766746	681.0
macro avg	0.758796	0.759903	0.757781	2077.0
micro avg	0.766277	0.766277	0.766277	2077.0
weighted avg	0.766244	0.766261	0.766277	2077.0

The result of Model Number:Dataset Number:Featuregroup Number:Rescale of 29 : 1 : 2 : 1

	f1-score	precision	recall	support
AFIB	0.735181	0.741855	0.729002	204.0
GSVT	0.759479	0.754889	0.764324	300.0
SB	0.775368	0.774329	0.776411	892.0
SR	0.768194	0.769786	0.766613	681.0
macro avg	0.759555	0.760215	0.759087	2077.0
micro avg	0.766796	0.766796	0.766796	2077.0
weighted avg	0.766773	0.766842	0.766796	2077.0

The result of Model Number:Dataset Number:Featuregroup Number:Rescale of 29 : 1 : 3 : 0

	f1-score	precision	recall	support
AFIB	0.737257	0.734989	0.739585	875.0
GSVT	0.718463	0.721986	0.715088	691.0
SB	0.755306	0.723962	0.789494	1553.0
SR	0.650003	0.709190	0.599986	907.0
macro avg	0.715257	0.722532	0.711038	4026.0
micro avg	0.723183	0.723183	0.723183	4026.0
weighted avg	0.721336	0.722692	0.723183	4026.0

The result of Model Number:Dataset Number:Featuregroup Number:Rescale of 29 : 1 : 3 : 1

	f1-score	precision	recall	support
AFIB	0.771466	0.823757	0.725930	204.0

GSVT 0.726903 0.748471 0.706818 300.0
SB 0.759795 0.723322 0.800576 892.0
SR 0.674179 0.704787 0.646853 681.0
macro avg 0.733086 0.750084 0.720044 2077.0
micro avg 0.729300 0.729300 0.729300 2077.0
weighted avg 0.728119 0.730742 0.729300 2077.0
The result of Model Number:Dataset Number:Featuregroup Number:Rescale of 29 : 1 : 4 : 0

	f1-score	precision	recall	support
AFIB	0.820834	0.822873	0.818861	875.0
GSVT	0.834970	0.837479	0.832582	691.0
SB	0.885753	0.879741	0.891854	1553.0
SR	0.868898	0.875348	0.862575	907.0
macro avg	0.852614	0.853860	0.851468	4026.0
micro avg	0.859221	0.859221	0.859221	4026.0
weighted avg	0.859130	0.859138	0.859221	4026.0

The result of Model Number:Dataset Number:Featuregroup Number:Rescale of 29 : 1 : 4 : 1

	f1-score	precision	recall	support
AFIB	0.785822	0.839477	0.738949	204.0
GSVT	0.830692	0.813765	0.848412	300.0
SB	0.851296	0.843833	0.858925	892.0
SR	0.830298	0.833063	0.827625	681.0
macro avg	0.824527	0.832534	0.818478	2077.0
micro avg	0.835360	0.835360	0.835360	2077.0
weighted avg	0.835005	0.835531	0.835360	2077.0

The result of Model Number:Dataset Number:Featuregroup Number:Rescale of 29 : 1 : 5 : 0

	f1-score	precision	recall	support
AFIB	0.798068	0.809793	0.786751	204.0
GSVT	0.818289	0.811332	0.825383	300.0
SB	0.832431	0.829101	0.835790	892.0
SR	0.824198	0.828244	0.820202	681.0
macro avg	0.818247	0.819617	0.817032	2077.0
micro avg	0.824359	0.824359	0.824359	2077.0
weighted avg	0.824314	0.824357	0.824359	2077.0

The result of Model Number:Dataset Number:Featuregroup Number:Rescale of 29 : 1 : 5 : 1

	f1-score	precision	recall	support
AFIB	0.800707	0.806467	0.795030	875.0
GSVT	0.799431	0.794644	0.804288	691.0
SB	0.843283	0.833940	0.852840	1553.0
SR	0.809195	0.823632	0.795282	907.0
macro avg	0.813154	0.814671	0.811860	4026.0

micro avg   0.818976   0.818976 0.818976   4026.0
weighted avg 0.818824   0.818902 0.818976   4026.0
The result of Model Number:Dataset Number:Featuregroup Number:Rescale of 29 : 1 : 6 : 0

	f1-score	precision	recall	support
AFIB	0.739343	0.738147	0.740609	875.0
GSVT	0.746118	0.749085	0.743212	691.0
SB	0.783554	0.780705	0.786429	1553.0
SR	0.770737	0.774636	0.766920	907.0
macro avg	0.759938	0.760643	0.759292	4026.0
micro avg	0.764658	0.764658	0.764658	4026.0
weighted avg	0.764633	0.764661	0.764658	4026.0

The result of Model Number:Dataset Number:Featuregroup Number:Rescale of 29 : 1 : 6 : 1

	f1-score	precision	recall	support
AFIB	0.884089	0.896604	0.872643	204.0
GSVT	0.902444	0.885828	0.919860	300.0
SB	0.921678	0.911487	0.932114	892.0
SR	0.902308	0.920686	0.884704	681.0
macro avg	0.902630	0.903651	0.902330	2077.0
micro avg	0.908958	0.908958	0.908958	2077.0
weighted avg	0.908857	0.909335	0.908958	2077.0

The result of Model Number:Dataset Number:Featuregroup Number:Rescale of 29 : 1 : 7 : 0

	f1-score	precision	recall	support
AFIB	0.723403	0.729437	0.717718	204.0
GSVT	0.742514	0.739094	0.745980	300.0
SB	0.753711	0.751034	0.756413	892.0
SR	0.746750	0.750095	0.743474	681.0
macro avg	0.741595	0.742415	0.740896	2077.0
micro avg	0.746863	0.746863	0.746863	2077.0
weighted avg	0.746835	0.746880	0.746863	2077.0

The result of Model Number:Dataset Number:Featuregroup Number:Rescale of 29 : 1 : 7 : 1

	f1-score	precision	recall	support
AFIB	0.674321	0.690339	0.659091	204.0
GSVT	0.696470	0.689940	0.703198	300.0
SB	0.711428	0.710632	0.712225	892.0
SR	0.704561	0.703853	0.705282	681.0
macro avg	0.696695	0.698691	0.694949	2077.0
micro avg	0.703426	0.703426	0.703426	2077.0
weighted avg	0.703371	0.703427	0.703426	2077.0

The result of Model Number:Dataset Number:Featuregroup Number:Rescale of 29 : 1 : 8 : 0

	f1-score	precision	recall	support
AFIB	0.727517	0.742368	0.713570	204.0
GSVT	0.753934	0.747384	0.760738	300.0
SB	0.768429	0.767058	0.769808	892.0
SR	0.761277	0.761731	0.760828	681.0
macro avg	0.752789	0.754635	0.751236	2077.0
micro avg	0.760030	0.760030	0.760030	2077.0
weighted avg	0.759972	0.760044	0.760030	2077.0

The result of Model Number:Dataset Number:Featuregroup Number:Rescale of 29 : 1 : 8 : 1

	f1-score	precision	recall	support
AFIB	0.881519	0.877934	0.885156	875.0
GSVT	0.894404	0.899811	0.889097	691.0
SB	0.943318	0.942169	0.944472	1553.0
SR	0.923639	0.925102	0.922207	907.0
macro avg	0.910720	0.911254	0.910233	4026.0
micro avg	0.917060	0.917060	0.917060	4026.0
weighted avg	0.917058	0.917093	0.917060	4026.0

The result of Model Number:Dataset Number:Featuregroup Number:Rescale of 29 : 1 : 9 : 0

	f1-score	precision	recall	support
AFIB	0.745978	0.764867	0.728068	204.0
GSVT	0.762179	0.753890	0.770777	300.0
SB	0.783646	0.779735	0.787599	892.0
SR	0.771500	0.774962	0.768100	681.0
macro avg	0.765826	0.768364	0.763636	2077.0
micro avg	0.772929	0.772929	0.772929	2077.0
weighted avg	0.772863	0.772977	0.772929	2077.0

The result of Model Number:Dataset Number:Featuregroup Number:Rescale of 29 : 1 : 9 : 1

	f1-score	precision	recall	support
AFIB	0.911087	0.919994	0.902565	875.0
GSVT	0.904128	0.897268	0.911391	691.0
SB	0.956670	0.946827	0.966805	1553.0
SR	0.926304	0.941201	0.912133	907.0
macro avg	0.924548	0.926323	0.923223	4026.0
micro avg	0.931015	0.931015	0.931015	4026.0
weighted avg	0.930904	0.931222	0.931015	4026.0

The result of Model Number:Dataset Number:Featuregroup Number:Rescale of 29 : 1 : 10 : 0

	f1-score	precision	recall	support
AFIB	0.740108	0.747293	0.733518	204.0
GSVT	0.762293	0.758690	0.766132	300.0
SB	0.777729	0.776862	0.778601	892.0

SR 0.770886 0.771685 0.770094 681.0
macro avg 0.762754 0.763632 0.762086 2077.0
micro avg 0.769583 0.769583 0.769583 2077.0
weighted avg 0.769561 0.769635 0.769583 2077.0
The result of Model Number:Dataset Number:Featuregroup Number:Rescale of 29 : 1 : 10 : 1
f1-score precision recall support
AFIB 0.695149 0.694024 0.696339 875.0
GSVT 0.701519 0.704308 0.698787 691.0
SB 0.736717 0.734039 0.739420 1553.0
SR 0.724666 0.728332 0.721078 907.0
macro avg 0.714513 0.715176 0.713906 4026.0
micro avg 0.718950 0.718950 0.718950 4026.0
weighted avg 0.718927 0.718954 0.718950 4026.0
The result of Model Number:Dataset Number:Featuregroup Number:Rescale of 30 : 0 : 0 : 0
f1-score precision recall support
AFIB 0.901852 0.940504 0.866594 204.0
GSVT 0.932157 0.918073 0.946740 300.0
SB 0.950646 0.941244 0.960240 892.0
SR 0.924890 0.932803 0.917146 681.0
macro avg 0.927387 0.933156 0.922680 2077.0
micro avg 0.934962 0.934962 0.934962 2077.0
weighted avg 0.934738 0.935057 0.934962 2077.0
The result of Model Number:Dataset Number:Featuregroup Number:Rescale of 30 : 0 : 0 : 1
f1-score precision recall support
AFIB 0.889621 0.900545 0.879098 204.0
GSVT 0.907540 0.903664 0.911471 300.0
SB 0.918526 0.915769 0.921304 892.0
SR 0.910795 0.912962 0.908666 681.0
macro avg 0.906620 0.908235 0.905135 2077.0
micro avg 0.911595 0.911595 0.911595 2077.0
weighted avg 0.911565 0.911605 0.911595 2077.0
The result of Model Number:Dataset Number:Featuregroup Number:Rescale of 30 : 0 : 1 : 0
f1-score precision recall support
AFIB 0.743325 0.793709 0.699450 204.0
GSVT 0.700388 0.721168 0.681035 300.0
SB 0.732080 0.696937 0.771373 892.0
SR 0.649586 0.679078 0.623258 681.0
macro avg 0.706345 0.722723 0.693779 2077.0
micro avg 0.702697 0.702697 0.702697 2077.0
weighted avg 0.701559 0.704086 0.702697 2077.0

The result of Model Number:Dataset Number:Featuregroup Number:Rescale of 30 : 0 : 1 : 1

	f1-score	precision	recall	support
AFIB	0.762129	0.785687	0.740607	204.0
GSVT	0.711438	0.726904	0.697141	300.0
SB	0.750387	0.717083	0.786972	892.0
SR	0.675641	0.710360	0.644254	681.0
macro avg	0.724899	0.735009	0.717243	2077.0
micro avg	0.722649	0.722649	0.722649	2077.0
weighted avg	0.721407	0.723036	0.722649	2077.0

The result of Model Number:Dataset Number:Featuregroup Number:Rescale of 30 : 0 : 2 : 0

	f1-score	precision	recall	support
AFIB	0.700962	0.696224	0.705859	875.0
GSVT	0.718063	0.718349	0.717870	691.0
SB	0.762796	0.760849	0.764760	1553.0
SR	0.742026	0.750316	0.733926	907.0
macro avg	0.730961	0.731435	0.730604	4026.0
micro avg	0.736964	0.736964	0.736964	4026.0
weighted avg	0.737000	0.737136	0.736964	4026.0

The result of Model Number:Dataset Number:Featuregroup Number:Rescale of 30 : 0 : 2 : 1

	f1-score	precision	recall	support
AFIB	0.666711	0.710151	0.628574	204.0
GSVT	0.695918	0.678813	0.714003	300.0
SB	0.716328	0.707126	0.725797	892.0
SR	0.697722	0.706053	0.689632	681.0
macro avg	0.694170	0.700536	0.689502	2077.0
micro avg	0.702687	0.702687	0.702687	2077.0
weighted avg	0.702406	0.702982	0.702687	2077.0

The result of Model Number:Dataset Number:Featuregroup Number:Rescale of 30 : 0 : 3 : 0

	f1-score	precision	recall	support
AFIB	0.797999	0.802363	0.793709	875.0
GSVT	0.794196	0.787500	0.801087	691.0
SB	0.835296	0.827386	0.843363	1553.0
SR	0.804209	0.819210	0.789758	907.0
macro avg	0.807925	0.809115	0.806979	4026.0
micro avg	0.813239	0.813239	0.813239	4026.0
weighted avg	0.813132	0.813260	0.813239	4026.0

The result of Model Number:Dataset Number:Featuregroup Number:Rescale of 30 : 0 : 3 : 1

	f1-score	precision	recall	support
AFIB	0.801937	0.802653	0.801306	875.0

GSVT	0.816627	0.818769	0.814633	691.0
SB	0.866860	0.861089	0.872719	1553.0
SR	0.849970	0.857730	0.842380	907.0
macro avg	0.833849	0.835060	0.832759	4026.0
micro avg	0.840394	0.840394	0.840394	4026.0
weighted avg	0.840323	0.840368	0.840394	4026.0

The result of Model Number:Dataset Number:Featuregroup Number:Rescale of 30 : 0 : 4 : 0

	f1-score	precision	recall	support
AFIB	0.788050	0.802552	0.774464	204.0
GSVT	0.817125	0.810281	0.824227	300.0
SB	0.832468	0.831073	0.833868	892.0
SR	0.825118	0.825851	0.824391	681.0
macro avg	0.815690	0.817439	0.814237	2077.0
micro avg	0.823534	0.823534	0.823534	2077.0
weighted avg	0.823479	0.823557	0.823534	2077.0

The result of Model Number:Dataset Number:Featuregroup Number:Rescale of 30 : 0 : 4 : 1

	f1-score	precision	recall	support
AFIB	0.613587	0.673740	0.563895	204.0
GSVT	0.719900	0.710197	0.729988	300.0
SB	0.764309	0.761759	0.766879	892.0
SR	0.744446	0.734465	0.754720	681.0
macro avg	0.710560	0.720040	0.703870	2077.0
micro avg	0.737627	0.737627	0.737627	2077.0
weighted avg	0.736578	0.736717	0.737627	2077.0

The result of Model Number:Dataset Number:Featuregroup Number:Rescale of 30 : 0 : 5 : 0

	f1-score	precision	recall	support
AFIB	0.662003	0.657834	0.745701	204.0
GSVT	0.806993	0.802842	0.811322	300.0
SB	0.829970	0.826021	0.833966	892.0
SR	0.651018	0.653163	0.649280	681.0
macro avg	0.737496	0.734965	0.760067	2077.0
micro avg	0.761472	0.761472	0.761472	2077.0
weighted avg	0.751480	0.749478	0.761472	2077.0

The result of Model Number:Dataset Number:Featuregroup Number:Rescale of 30 : 0 : 5 : 1

	f1-score	precision	recall	support
AFIB	0.805763	0.784596	0.856206	875.0
GSVT	0.775136	0.733676	0.883639	691.0
SB	0.734482	0.729840	0.739205	1553.0
SR	0.713433	0.725725	0.701749	907.0
macro avg	0.757203	0.743459	0.795200	4026.0

micro avg 0.780985 0.780985 0.780985 4026.0
weighted avg 0.752210 0.741472 0.780985 4026.0
The result of Model Number:Dataset Number:Featuregroup Number:Rescale of 30 : 0 : 6 : 0

	f1-score	precision	recall	support
AFIB	0.763679	0.775388	0.752444	204.0
GSVT	0.781787	0.775154	0.788567	300.0
SB	0.795386	0.792819	0.797972	892.0
SR	0.787213	0.790135	0.784322	681.0
macro avg	0.782016	0.783374	0.780826	2077.0
micro avg	0.787666	0.787666	0.787666	2077.0
weighted avg	0.787628	0.787676	0.787666	2077.0

The result of Model Number:Dataset Number:Featuregroup Number:Rescale of 30 : 0 : 6 : 1

	f1-score	precision	recall	support
AFIB	0.827317	0.858094	0.799042	204.0
GSVT	0.767719	0.803345	0.735190	300.0
SB	0.804216	0.755710	0.859391	892.0
SR	0.706257	0.755327	0.663256	681.0
macro avg	0.776377	0.793119	0.764220	2077.0
micro avg	0.771216	0.771216	0.771216	2077.0
weighted avg	0.769095	0.772521	0.771216	2077.0

The result of Model Number:Dataset Number:Featuregroup Number:Rescale of 30 : 0 : 7 : 0

	f1-score	precision	recall	support
AFIB	0.774211	0.769335	0.872095	204.0
GSVT	0.943776	0.938921	0.948839	300.0
SB	0.970648	0.966030	0.975321	892.0
SR	0.761364	0.763872	0.759331	681.0
macro avg	0.862500	0.859540	0.888897	2077.0
micro avg	0.890539	0.890539	0.890539	2077.0
weighted avg	0.878853	0.876512	0.890539	2077.0

The result of Model Number:Dataset Number:Featuregroup Number:Rescale of 30 : 0 : 7 : 1

	f1-score	precision	recall	support
AFIB	0.893030	0.898375	0.887794	204.0
GSVT	0.916820	0.914095	0.919568	300.0
SB	0.930859	0.929719	0.932006	892.0
SR	0.924074	0.925164	0.922993	681.0
macro avg	0.916196	0.916838	0.915590	2077.0
micro avg	0.922912	0.922912	0.922912	2077.0
weighted avg	0.922891	0.922890	0.922912	2077.0

The result of Model Number:Dataset Number:Featuregroup Number:Rescale of 30 : 0 : 8 : 0

	f1-score	precision	recall	support
AFIB	0.834464	0.870228	0.801841	204.0
GSVT	0.862505	0.849473	0.875998	300.0
SB	0.879612	0.870913	0.888489	892.0
SR	0.855781	0.863103	0.848615	681.0
macro avg	0.858091	0.863429	0.853736	2077.0
micro avg	0.865101	0.865101	0.865101	2077.0
weighted avg	0.864893	0.865188	0.865101	2077.0

The result of Model Number:Dataset Number:Featuregroup Number:Rescale of 30 : 0 : 8 : 1

	f1-score	precision	recall	support
AFIB	0.620087	0.688170	0.564412	204.0
GSVT	0.724036	0.714984	0.733462	300.0
SB	0.770339	0.766408	0.774311	892.0
SR	0.752293	0.741775	0.763116	681.0
macro avg	0.716689	0.727834	0.708825	2077.0
micro avg	0.744124	0.744124	0.744124	2077.0
weighted avg	0.742976	0.743219	0.744124	2077.0

The result of Model Number:Dataset Number:Featuregroup Number:Rescale of 30 : 0 : 9 : 0

	f1-score	precision	recall	support
AFIB	0.711316	0.703457	0.719367	875.0
GSVT	0.724132	0.728591	0.719740	691.0
SB	0.770700	0.769417	0.771990	1553.0
SR	0.751934	0.758965	0.745051	907.0
macro avg	0.739521	0.740107	0.739037	4026.0
micro avg	0.745516	0.745516	0.745516	4026.0
weighted avg	0.745573	0.745720	0.745516	4026.0

The result of Model Number:Dataset Number:Featuregroup Number:Rescale of 30 : 0 : 9 : 1

	f1-score	precision	recall	support
AFIB	0.644691	0.702291	0.680808	204.0
GSVT	0.875670	0.861355	0.890770	300.0
SB	0.745465	0.739787	0.751287	892.0
SR	0.911583	0.901268	0.922163	681.0
macro avg	0.794352	0.801175	0.811257	2077.0
micro avg	0.820538	0.820538	0.820538	2077.0
weighted avg	0.808840	0.806609	0.820538	2077.0

The result of Model Number:Dataset Number:Featuregroup Number:Rescale of 30 : 0 : 10 : 0

	f1-score	precision	recall	support
AFIB	0.644770	0.650110	0.726245	204.0
GSVT	0.699209	0.669282	0.788872	300.0
SB	0.638404	0.636186	0.640640	892.0

SR	0.632056	0.632176	0.631941	681.0
macro avg	0.653610	0.646938	0.696924	2077.0
micro avg	0.667606	0.667606	0.667606	2077.0
weighted avg	0.645731	0.641019	0.667606	2077.0

The result of Model Number:Dataset Number:Featuregroup Number:Rescale of 30 : 0 : 10 : 1

	f1-score	precision	recall	support
AFIB	0.851655	0.841060	0.862615	875.0
GSVT	0.878318	0.889317	0.867676	691.0
SB	0.937043	0.936028	0.938067	1553.0
SR	0.911770	0.916445	0.907146	907.0
macro avg	0.894696	0.895713	0.893876	4026.0
micro avg	0.902621	0.902621	0.902621	4026.0
weighted avg	0.902712	0.902959	0.902621	4026.0

The result of Model Number:Dataset Number:Featuregroup Number:Rescale of 30 : 1 : 0 : 0

	f1-score	precision	recall	support
AFIB	0.643722	0.702023	0.595435	204.0
GSVT	0.747157	0.738620	0.756125	300.0
SB	0.792316	0.790288	0.794359	892.0
SR	0.772917	0.762451	0.783697	681.0
macro avg	0.739028	0.748345	0.732404	2077.0
micro avg	0.765803	0.765803	0.765803	2077.0
weighted avg	0.764838	0.765029	0.765803	2077.0

The result of Model Number:Dataset Number:Featuregroup Number:Rescale of 30 : 1 : 0 : 1

	f1-score	precision	recall	support
AFIB	0.801144	0.808947	0.793684	204.0
GSVT	0.819670	0.815906	0.823496	300.0
SB	0.830997	0.828138	0.833882	892.0
SR	0.823979	0.827176	0.820846	681.0
macro avg	0.818947	0.820042	0.817977	2077.0
micro avg	0.824159	0.824159	0.824159	2077.0
weighted avg	0.824128	0.824171	0.824159	2077.0

The result of Model Number:Dataset Number:Featuregroup Number:Rescale of 30 : 1 : 1 : 0

	f1-score	precision	recall	support
AFIB	0.885376	0.873622	0.897519	875.0
GSVT	0.903346	0.911910	0.894981	691.0
SB	0.961290	0.959016	0.963579	1553.0
SR	0.935212	0.945045	0.925619	907.0
macro avg	0.921306	0.922398	0.920425	4026.0
micro avg	0.928896	0.928896	0.928896	4026.0
weighted avg	0.928971	0.929224	0.928896	4026.0

The result of Model Number:Dataset Number:Featuregroup Number:Rescale of 30 : 1 : 1 : 1

	f1-score	precision	recall	support
AFIB	0.833200	0.853145	0.815386	204.0
GSVT	0.857736	0.845650	0.870530	300.0
SB	0.880022	0.871082	0.889189	892.0
SR	0.858989	0.871392	0.847002	681.0
macro avg	0.857487	0.860317	0.855527	2077.0
micro avg	0.865413	0.865413	0.865413	2077.0
weighted avg	0.865308	0.865748	0.865413	2077.0

The result of Model Number:Dataset Number:Featuregroup Number:Rescale of 30 : 1 : 2 : 0

	f1-score	precision	recall	support
AFIB	0.848519	0.876079	0.822807	204.0
GSVT	0.871946	0.860828	0.883530	300.0
SB	0.896545	0.893165	0.899961	892.0
SR	0.884006	0.885607	0.882445	681.0
macro avg	0.875254	0.878920	0.872186	2077.0
micro avg	0.884267	0.884267	0.884267	2077.0
weighted avg	0.884164	0.884338	0.884267	2077.0

The result of Model Number:Dataset Number:Featuregroup Number:Rescale of 30 : 1 : 2 : 1

	f1-score	precision	recall	support
AFIB	0.699644	0.713926	0.686231	204.0
GSVT	0.725049	0.718750	0.731592	300.0
SB	0.738989	0.737670	0.740315	892.0
SR	0.732111	0.732547	0.731679	681.0
macro avg	0.723948	0.725723	0.722454	2077.0
micro avg	0.730912	0.730912	0.730912	2077.0
weighted avg	0.730856	0.730926	0.730912	2077.0

The result of Model Number:Dataset Number:Featuregroup Number:Rescale of 30 : 1 : 3 : 0

	f1-score	precision	recall	support
AFIB	0.666859	0.687233	0.647733	875.0
GSVT	0.707681	0.705284	0.710156	691.0
SB	0.773314	0.770207	0.776449	1553.0
SR	0.732415	0.719711	0.745647	907.0
macro avg	0.720067	0.720609	0.719996	4026.0
micro avg	0.730157	0.730157	0.730157	4026.0
weighted avg	0.729698	0.729655	0.730157	4026.0

The result of Model Number:Dataset Number:Featuregroup Number:Rescale of 30 : 1 : 3 : 1

	f1-score	precision	recall	support
AFIB	0.828735	0.882732	0.781330	204.0

GSVT 0.865041 0.843778 0.887520 300.0
SB 0.890410 0.878972 0.902181 892.0
SR 0.867283 0.877639 0.857226 681.0
macro avg 0.862867 0.870780 0.857064 2077.0
micro avg 0.873454 0.873454 0.873454 2077.0
weighted avg 0.873105 0.873821 0.873454 2077.0
The result of Model Number:Dataset Number:Featuregroup Number:Rescale of 30 : 1 : 4 : 0

	f1-score	precision	recall	support
AFIB	0.633103	0.628066	0.638281	875.0
GSVT	0.608000	0.596440	0.620068	691.0
SB	0.638118	0.589039	0.696131	1553.0
SR	0.488621	0.624774	0.401283	907.0
macro avg	0.591961	0.609580	0.588941	4026.0
micro avg	0.604078	0.604078	0.604078	4026.0
weighted avg	0.598179	0.606842	0.604078	4026.0

The result of Model Number:Dataset Number:Featuregroup Number:Rescale of 30 : 1 : 4 : 1

	f1-score	precision	recall	support
AFIB	0.790724	0.805256	0.776805	204.0
GSVT	0.804006	0.798741	0.809363	300.0
SB	0.817759	0.813767	0.821793	892.0
SR	0.809307	0.812672	0.805981	681.0
macro avg	0.805449	0.807609	0.803486	2077.0
micro avg	0.810395	0.810395	0.810395	2077.0
weighted avg	0.810346	0.810401	0.810395	2077.0

The result of Model Number:Dataset Number:Featuregroup Number:Rescale of 30 : 1 : 5 : 0

	f1-score	precision	recall	support
AFIB	0.846279	0.850719	0.841937	875.0
GSVT	0.863975	0.862423	0.865594	691.0
SB	0.938129	0.930934	0.945437	1553.0
SR	0.905031	0.914089	0.896155	907.0
macro avg	0.888353	0.889541	0.887281	4026.0
micro avg	0.898136	0.898136	0.898136	4026.0
weighted avg	0.897983	0.897947	0.898136	4026.0

The result of Model Number:Dataset Number:Featuregroup Number:Rescale of 30 : 1 : 5 : 1

	f1-score	precision	recall	support
AFIB	0.900420	0.922328	0.879751	204.0
GSVT	0.911739	0.899187	0.924767	300.0
SB	0.930149	0.920860	0.939633	892.0
SR	0.913909	0.925902	0.902248	681.0
macro avg	0.914054	0.917069	0.911600	2077.0

micro avg 0.919347 0.919347 0.919347 2077.0
weighted avg 0.919245 0.919527 0.919347 2077.0
The result of Model Number:Dataset Number:Featuregroup Number:Rescale of 30 : 1 : 6 : 0

	f1-score	precision	recall	support
AFIB	0.693455	0.702832	0.684615	204.0
GSVT	0.719795	0.718626	0.720992	300.0
SB	0.735426	0.734446	0.736415	892.0
SR	0.727909	0.726976	0.728870	681.0
macro avg	0.719146	0.720720	0.717723	2077.0
micro avg	0.726626	0.726626	0.726626	2077.0
weighted avg	0.726581	0.726607	0.726626	2077.0

The result of Model Number:Dataset Number:Featuregroup Number:Rescale of 30 : 1 : 6 : 1

	f1-score	precision	recall	support
AFIB	0.779431	0.756135	0.831076	875.0
GSVT	0.667467	0.652851	0.683000	691.0
SB	0.885316	0.878102	0.892884	1553.0
SR	0.830554	0.865424	0.799943	907.0
macro avg	0.790692	0.788128	0.801726	4026.0
micro avg	0.822489	0.822489	0.822489	4026.0
weighted avg	0.812576	0.810077	0.822489	4026.0

The result of Model Number:Dataset Number:Featuregroup Number:Rescale of 30 : 1 : 7 : 0

	f1-score	precision	recall	support
AFIB	0.778996	0.792521	0.766024	204.0
GSVT	0.794347	0.791996	0.796728	300.0
SB	0.803714	0.800854	0.806597	892.0
SR	0.797179	0.798012	0.796367	681.0
macro avg	0.793559	0.795846	0.791429	2077.0
micro avg	0.797832	0.797832	0.797832	2077.0
weighted avg	0.797790	0.797824	0.797832	2077.0

The result of Model Number:Dataset Number:Featuregroup Number:Rescale of 30 : 1 : 7 : 1

	f1-score	precision	recall	support
AFIB	0.745699	0.774676	0.718866	875.0
GSVT	0.801651	0.795731	0.807766	691.0
SB	0.880953	0.876414	0.885545	1553.0
SR	0.829057	0.813247	0.845582	907.0
macro avg	0.814340	0.815017	0.814440	4026.0
micro avg	0.826967	0.826967	0.826967	4026.0
weighted avg	0.826255	0.826224	0.826967	4026.0

The result of Model Number:Dataset Number:Featuregroup Number:Rescale of 30 : 1 : 8 : 0

	f1-score	precision	recall	support
AFIB	0.688103	0.739830	0.643854	204.0
GSVT	0.656223	0.677053	0.636737	300.0
SB	0.683691	0.649860	0.721245	892.0
SR	0.605220	0.631234	0.581328	681.0
macro avg	0.658309	0.674494	0.645791	2077.0
micro avg	0.655562	0.655562	0.655562	2077.0
weighted avg	0.654428	0.656517	0.655562	2077.0

The result of Model Number:Dataset Number:Featuregroup Number:Rescale of 30 : 1 : 8 : 1

	f1-score	precision	recall	support
AFIB	0.763994	0.770050	0.758052	875.0
GSVT	0.773680	0.770269	0.777147	691.0
SB	0.822717	0.819465	0.826001	1553.0
SR	0.802500	0.804768	0.800284	907.0
macro avg	0.790723	0.791138	0.790371	4026.0
micro avg	0.797055	0.797055	0.797055	4026.0
weighted avg	0.796983	0.796970	0.797055	4026.0

The result of Model Number:Dataset Number:Featuregroup Number:Rescale of 30 : 1 : 9 : 0

	f1-score	precision	recall	support
AFIB	0.713434	0.709166	0.717850	875.0
GSVT	0.719276	0.723160	0.715539	691.0
SB	0.762325	0.760097	0.764578	1553.0
SR	0.745100	0.750452	0.739838	907.0
macro avg	0.735034	0.735719	0.734451	4026.0
micro avg	0.740432	0.740432	0.740432	4026.0
weighted avg	0.740430	0.740515	0.740432	4026.0

The result of Model Number:Dataset Number:Featuregroup Number:Rescale of 30 : 1 : 9 : 1

	f1-score	precision	recall	support
AFIB	0.803092	0.791689	0.814917	875.0
GSVT	0.832216	0.842833	0.821989	691.0
SB	0.884416	0.883118	0.885726	1553.0
SR	0.862383	0.868852	0.856012	907.0
macro avg	0.845527	0.846623	0.844661	4026.0
micro avg	0.852703	0.852703	0.852703	4026.0
weighted avg	0.852818	0.853119	0.852703	4026.0

The result of Model Number:Dataset Number:Featuregroup Number:Rescale of 30 : 1 : 10 : 0

	f1-score	precision	recall	support
AFIB	0.816805	0.814443	0.819255	875.0
GSVT	0.831943	0.831267	0.832679	691.0
SB	0.883645	0.879985	0.887342	1553.0

SR 0.857117 0.866533 0.847949 907.0
macro avg 0.847377 0.848057 0.846806 4026.0
micro avg 0.854287 0.854287 0.854287 4026.0
weighted avg 0.854268 0.854348 0.854287 4026.0

The result of Model Number:Dataset Number:Featuregroup Number:Rescale of 30 : 1 : 10 : 1

	f1-score	precision	recall	support
AFIB	0.678459	0.673269	0.683819	875.0
GSVT	0.694069	0.697865	0.690356	691.0
SB	0.734963	0.734449	0.735484	1553.0
SR	0.715442	0.718912	0.712042	907.0
macro avg	0.705733	0.706124	0.705425	4026.0
micro avg	0.711228	0.711228	0.711228	4026.0
weighted avg	0.711266	0.711373	0.711228	4026.0

The result of Model Number:Dataset Number:Featuregroup Number:Rescale of 31 : 0 : 0 : 0

	f1-score	precision	recall	support
AFIB	0.735006	0.749750	0.720976	204.0
GSVT	0.752040	0.744223	0.760063	300.0
SB	0.765949	0.762969	0.768954	892.0
SR	0.757900	0.761062	0.754781	681.0
macro avg	0.752724	0.754501	0.751194	2077.0
micro avg	0.758310	0.758310	0.758310	2077.0
weighted avg	0.758262	0.758338	0.758310	2077.0

The result of Model Number:Dataset Number:Featuregroup Number:Rescale of 31 : 0 : 0 : 1

	f1-score	precision	recall	support
AFIB	0.732229	0.737705	0.726862	204.0
GSVT	0.750939	0.746976	0.754945	300.0
SB	0.763612	0.762590	0.764638	892.0
SR	0.756886	0.758337	0.755442	681.0
macro avg	0.750916	0.751402	0.750472	2077.0
micro avg	0.756513	0.756513	0.756513	2077.0
weighted avg	0.756494	0.756496	0.756513	2077.0

The result of Model Number:Dataset Number:Featuregroup Number:Rescale of 31 : 0 : 1 : 0

	f1-score	precision	recall	support
AFIB	0.827843	0.838008	0.818051	204.0
GSVT	0.844518	0.840911	0.848176	300.0
SB	0.854740	0.852175	0.857325	892.0
SR	0.847546	0.849563	0.845566	681.0
macro avg	0.843662	0.845164	0.842279	2077.0
micro avg	0.848291	0.848291	0.848291	2077.0
weighted avg	0.848263	0.848300	0.848291	2077.0

The result of Model Number:Dataset Number:Featuregroup Number:Rescale of 31 : 0 : 1 : 1

	f1-score	precision	recall	support
AFIB	0.681454	0.686400	0.676628	875.0
GSVT	0.696402	0.689930	0.703050	691.0
SB	0.748428	0.743910	0.753007	1553.0
SR	0.723900	0.731983	0.715996	907.0
macro avg	0.712546	0.713056	0.712170	4026.0
micro avg	0.719494	0.719494	0.719494	4026.0
weighted avg	0.719417	0.719459	0.719494	4026.0

The result of Model Number:Dataset Number:Featuregroup Number:Rescale of 31 : 0 : 2 : 0

	f1-score	precision	recall	support
AFIB	0.893040	0.909423	0.877305	204.0
GSVT	0.904818	0.895687	0.914156	300.0
SB	0.927106	0.922280	0.931989	892.0
SR	0.914484	0.920215	0.908845	681.0
macro avg	0.909862	0.911901	0.908074	2077.0
micro avg	0.916454	0.916454	0.916454	2077.0
weighted avg	0.916402	0.916499	0.916454	2077.0

The result of Model Number:Dataset Number:Featuregroup Number:Rescale of 31 : 0 : 2 : 1

	f1-score	precision	recall	support
AFIB	0.916811	0.918649	0.915020	875.0
GSVT	0.919082	0.914120	0.924122	691.0
SB	0.960749	0.955616	0.965944	1553.0
SR	0.937765	0.948885	0.926928	907.0
macro avg	0.933602	0.934318	0.933004	4026.0
micro avg	0.938908	0.938908	0.938908	4026.0
weighted avg	0.938870	0.938943	0.938908	4026.0

The result of Model Number:Dataset Number:Featuregroup Number:Rescale of 31 : 0 : 3 : 0

	f1-score	precision	recall	support
AFIB	0.763166	0.772537	0.754139	204.0
GSVT	0.778538	0.775212	0.781910	300.0
SB	0.787962	0.785596	0.790345	892.0
SR	0.781330	0.783189	0.779504	681.0
macro avg	0.777749	0.779134	0.776474	2077.0
micro avg	0.782016	0.782016	0.782016	2077.0
weighted avg	0.781991	0.782024	0.782016	2077.0

The result of Model Number:Dataset Number:Featuregroup Number:Rescale of 31 : 0 : 3 : 1

	f1-score	precision	recall	support
AFIB	0.803818	0.810172	0.797589	875.0

GSVT 0.824044 0.822302 0.825824 691.0
SB 0.878455 0.870691 0.886370 1553.0
SR 0.855070 0.863456 0.846866 907.0
macro avg 0.840347 0.841655 0.839162 4026.0
micro avg 0.847783 0.847783 0.847783 4026.0
weighted avg 0.847626 0.847603 0.847783 4026.0
The result of Model Number:Dataset Number:Featuregroup Number:Rescale of 31 : 0 : 4 : 0

	f1-score	precision	recall	support
AFIB	0.862933	0.865902	0.860093	875.0
GSVT	0.879316	0.861772	0.897747	691.0
SB	0.930826	0.925792	0.935920	1553.0
SR	0.894824	0.915629	0.874996	907.0
macro avg	0.891975	0.892274	0.892189	4026.0
micro avg	0.899163	0.899163	0.899163	4026.0
weighted avg	0.899119	0.899498	0.899163	4026.0

The result of Model Number:Dataset Number:Featuregroup Number:Rescale of 31 : 0 : 4 : 1

	f1-score	precision	recall	support
AFIB	0.734253	0.762785	0.707832	875.0
GSVT	0.789346	0.783517	0.795367	691.0
SB	0.867431	0.862962	0.871953	1553.0
SR	0.816331	0.800765	0.832603	907.0
macro avg	0.801840	0.802507	0.801939	4026.0
micro avg	0.814273	0.814273	0.814273	4026.0
weighted avg	0.813572	0.813542	0.814273	4026.0

The result of Model Number:Dataset Number:Featuregroup Number:Rescale of 31 : 0 : 5 : 0

	f1-score	precision	recall	support
AFIB	0.729308	0.714579	0.744714	875.0
GSVT	0.753487	0.765468	0.742039	691.0
SB	0.801722	0.801677	0.801773	1553.0
SR	0.782284	0.789412	0.775296	907.0
macro avg	0.766700	0.767784	0.765955	4026.0
micro avg	0.773155	0.773155	0.773155	4026.0
weighted avg	0.773326	0.773769	0.773155	4026.0

The result of Model Number:Dataset Number:Featuregroup Number:Rescale of 31 : 0 : 5 : 1

	f1-score	precision	recall	support
AFIB	0.761209	0.794616	0.730689	204.0
GSVT	0.708636	0.713738	0.703897	300.0
SB	0.734911	0.698006	0.775939	892.0
SR	0.651981	0.695028	0.614093	681.0
macro avg	0.714184	0.725347	0.706155	2077.0

micro avg 0.708024 0.708024 0.708024 2077.0
weighted avg 0.706508 0.708791 0.708024 2077.0
The result of Model Number:Dataset Number:Featuregroup Number:Rescale of 31 : 0 : 6 : 0

	f1-score	precision	recall	support
AFIB	0.825738	0.824750	0.826868	875.0
GSVT	0.841835	0.844730	0.839045	691.0
SB	0.891628	0.886135	0.897197	1553.0
SR	0.873484	0.882015	0.865213	907.0
macro avg	0.858171	0.859408	0.857081	4026.0
micro avg	0.864725	0.864725	0.864725	4026.0
weighted avg	0.864674	0.864759	0.864725	4026.0

The result of Model Number:Dataset Number:Featuregroup Number:Rescale of 31 : 0 : 6 : 1

	f1-score	precision	recall	support
AFIB	0.787347	0.817942	0.759015	875.0
GSVT	0.846423	0.840173	0.852880	691.0
SB	0.930154	0.925362	0.935003	1553.0
SR	0.875360	0.858667	0.892808	907.0
macro avg	0.859821	0.860536	0.859926	4026.0
micro avg	0.873153	0.873153	0.873153	4026.0
weighted avg	0.872401	0.872369	0.873153	4026.0

The result of Model Number:Dataset Number:Featuregroup Number:Rescale of 31 : 0 : 7 : 0

	f1-score	precision	recall	support
AFIB	0.889920	0.888097	0.892395	875.0
GSVT	0.902059	0.892590	0.911894	691.0
SB	0.951369	0.938523	0.964758	1553.0
SR	0.905076	0.939591	0.873212	907.0
macro avg	0.912106	0.914700	0.910565	4026.0
micro avg	0.919333	0.919333	0.919333	4026.0
weighted avg	0.919121	0.919921	0.919333	4026.0

The result of Model Number:Dataset Number:Featuregroup Number:Rescale of 31 : 0 : 7 : 1

	f1-score	precision	recall	support
AFIB	0.845298	0.871427	0.821427	204.0
GSVT	0.789075	0.806229	0.773218	300.0
SB	0.832275	0.795337	0.872852	892.0
SR	0.749372	0.787879	0.714559	681.0
macro avg	0.804005	0.815218	0.795514	2077.0
micro avg	0.801510	0.801510	0.801510	2077.0
weighted avg	0.800132	0.801938	0.801510	2077.0

The result of Model Number:Dataset Number:Featuregroup Number:Rescale of 31 : 0 : 8 : 0

```
            f1-score  precision   recall  support
AFIB         0.793110   0.781858  0.804710    875.0
GSVT         0.809428   0.817317  0.801695    691.0
SB           0.861389   0.858582  0.864216   1553.0
SR           0.841273   0.852025  0.830798    907.0
macro avg    0.826300   0.827445  0.825355   4026.0
micro avg    0.833024   0.833024  0.833024   4026.0
weighted avg  0.833099   0.833347  0.833024   4026.0
```
The result of Model Number:Dataset Number:Featuregroup Number:Rescale of 31 : 0 : 8 : 1
```
            f1-score  precision   recall  support
AFIB         0.912306   0.921193  0.903812    204.0
GSVT         0.933403   0.929117  0.937761    300.0
SB           0.946302   0.943046  0.949587    892.0
SR           0.938310   0.941951  0.934743    681.0
macro avg    0.932581   0.933827  0.931476   2077.0
micro avg    0.938516   0.938516  0.938516   2077.0
weighted avg  0.938480   0.938529  0.938516   2077.0
```
The result of Model Number:Dataset Number:Featuregroup Number:Rescale of 31 : 0 : 9 : 0
```
            f1-score  precision   recall  support
AFIB         0.863709   0.850800  0.877048    875.0
GSVT         0.880269   0.887797  0.872881    691.0
SB           0.940547   0.937309  0.943815   1553.0
SR           0.913018   0.926636  0.899826    907.0
macro avg    0.899386   0.900635  0.898393   4026.0
micro avg    0.907219   0.907219  0.907219   4026.0
weighted avg  0.907300   0.907605  0.907219   4026.0
```
The result of Model Number:Dataset Number:Featuregroup Number:Rescale of 31 : 0 : 9 : 1
```
            f1-score  precision   recall  support
AFIB         0.675658   0.691719  0.660401    204.0
GSVT         0.693199   0.690524  0.695967    300.0
SB           0.710376   0.708791  0.711969    892.0
SR           0.701439   0.700028  0.702881    681.0
macro avg    0.695168   0.697765  0.692804   2077.0
micro avg    0.701613   0.701613  0.701613   2077.0
weighted avg  0.701555   0.701602  0.701613   2077.0
```
The result of Model Number:Dataset Number:Featuregroup Number:Rescale of 31 : 0 : 10 : 0
```
            f1-score  precision   recall  support
AFIB         0.604777   0.607247  0.602346    875.0
GSVT         0.563164   0.588019  0.540418    691.0
SB           0.604805   0.547875  0.674947   1553.0
```

SR 0.456225 0.567815 0.381445 907.0
macro avg 0.557243 0.577739 0.549789 4026.0
micro avg 0.569956 0.569956 0.569956 4026.0
weighted avg 0.564179 0.572161 0.569956 4026.0
The result of Model Number:Dataset Number:Featuregroup Number:Rescale of 31 : 0 : 10 : 1

	f1-score	precision	recall	support
AFIB	0.717635	0.711887	0.723561	875.0
GSVT	0.737454	0.738712	0.736286	691.0
SB	0.781818	0.779722	0.783931	1553.0
SR	0.760456	0.769309	0.751822	907.0
macro avg	0.749341	0.749908	0.748900	4026.0
micro avg	0.755399	0.755399	0.755399	4026.0
weighted avg	0.755442	0.755594	0.755399	4026.0

The result of Model Number:Dataset Number:Featuregroup Number:Rescale of 31 : 1 : 0 : 0

	f1-score	precision	recall	support
AFIB	0.783570	0.771556	0.796095	875.0
GSVT	0.810589	0.818534	0.802956	691.0
SB	0.862683	0.860668	0.864735	1553.0
SR	0.841133	0.851798	0.830750	907.0
macro avg	0.824494	0.825639	0.823634	4026.0
micro avg	0.831557	0.831557	0.831557	4026.0
weighted avg	0.831693	0.832070	0.831557	4026.0

The result of Model Number:Dataset Number:Featuregroup Number:Rescale of 31 : 1 : 0 : 1

	f1-score	precision	recall	support
AFIB	0.680089	0.679911	0.680333	875.0
GSVT	0.694296	0.691671	0.697016	691.0
SB	0.739183	0.735846	0.742562	1553.0
SR	0.714271	0.722432	0.706320	907.0
macro avg	0.706960	0.707465	0.706558	4026.0
micro avg	0.713055	0.713055	0.713055	4026.0
weighted avg	0.713023	0.713085	0.713055	4026.0

The result of Model Number:Dataset Number:Featuregroup Number:Rescale of 31 : 1 : 1 : 0

	f1-score	precision	recall	support
AFIB	0.795600	0.774700	0.845407	875.0
GSVT	0.765360	0.724423	0.872494	691.0
SB	0.725218	0.720635	0.729882	1553.0
SR	0.704435	0.716572	0.692898	907.0
macro avg	0.747653	0.734082	0.785170	4026.0
micro avg	0.771135	0.771135	0.771135	4026.0
weighted avg	0.742722	0.732120	0.771135	4026.0

The result of Model Number:Dataset Number:Featuregroup Number:Rescale of 31 : 1 : 1 : 1

	f1-score	precision	recall	support
AFIB	0.665971	0.657052	0.675178	875.0
GSVT	0.680861	0.686175	0.675640	691.0
SB	0.722885	0.720623	0.725164	1553.0
SR	0.705403	0.714755	0.696306	907.0
macro avg	0.693780	0.694651	0.693072	4026.0
micro avg	0.699299	0.699299	0.699299	4026.0
weighted avg	0.699364	0.699572	0.699299	4026.0

The result of Model Number:Dataset Number:Featuregroup Number:Rescale of 31 : 1 : 2 : 0

	f1-score	precision	recall	support
AFIB	0.773335	0.777398	0.769361	875.0
GSVT	0.786386	0.787845	0.785031	691.0
SB	0.832890	0.826975	0.838895	1553.0
SR	0.816921	0.821954	0.811968	907.0
macro avg	0.802383	0.803543	0.801314	4026.0
micro avg	0.808471	0.808471	0.808471	4026.0
weighted avg	0.808367	0.808353	0.808471	4026.0

The result of Model Number:Dataset Number:Featuregroup Number:Rescale of 31 : 1 : 2 : 1

	f1-score	precision	recall	support
AFIB	0.887331	0.888531	0.886303	875.0
GSVT	0.901152	0.900688	0.901649	691.0
SB	0.941160	0.937792	0.944554	1553.0
SR	0.918622	0.923771	0.913589	907.0
macro avg	0.912066	0.912696	0.911524	4026.0
micro avg	0.917554	0.917554	0.917554	4026.0
weighted avg	0.917516	0.917559	0.917554	4026.0

The result of Model Number:Dataset Number:Featuregroup Number:Rescale of 31 : 1 : 3 : 0

	f1-score	precision	recall	support
AFIB	0.731160	0.725649	0.829857	204.0
GSVT	0.886388	0.881140	0.891708	300.0
SB	0.724942	0.723429	0.726465	892.0
SR	0.896583	0.895148	0.898034	681.0
macro avg	0.809768	0.806341	0.836516	2077.0
micro avg	0.816741	0.816741	0.816741	2077.0
weighted avg	0.805149	0.802729	0.816741	2077.0

The result of Model Number:Dataset Number:Featuregroup Number:Rescale of 31 : 1 : 3 : 1

	f1-score	precision	recall	support
AFIB	0.708221	0.714651	0.702269	204.0

GSVT	0.731628	0.727207	0.736296	300.0
SB	0.746935	0.745934	0.747939	892.0
SR	0.740024	0.741558	0.738501	681.0
macro avg	0.731702	0.732337	0.731251	2077.0
micro avg	0.738677	0.738677	0.738677	2077.0
weighted avg	0.738656	0.738722	0.738677	2077.0

The result of Model Number:Dataset Number:Featuregroup Number:Rescale of 31 : 1 : 4 : 0

	f1-score	precision	recall	support
AFIB	0.695214	0.700710	0.689827	875.0
GSVT	0.712707	0.711201	0.714247	691.0
SB	0.759767	0.753052	0.766613	1553.0
SR	0.739541	0.746794	0.732445	907.0
macro avg	0.726807	0.727939	0.725783	4026.0
micro avg	0.733239	0.733239	0.733239	4026.0
weighted avg	0.733104	0.733083	0.733239	4026.0

The result of Model Number:Dataset Number:Featuregroup Number:Rescale of 31 : 1 : 4 : 1

	f1-score	precision	recall	support
AFIB	0.748061	0.733159	0.763620	875.0
GSVT	0.608855	0.566203	0.763183	691.0
SB	0.494019	0.494870	0.493171	1553.0
SR	0.800868	0.807920	0.793971	907.0
macro avg	0.662951	0.650538	0.703486	4026.0
micro avg	0.666059	0.666059	0.666059	4026.0
weighted avg	0.638070	0.629428	0.666059	4026.0

The result of Model Number:Dataset Number:Featuregroup Number:Rescale of 31 : 1 : 5 : 0

	f1-score	precision	recall	support
AFIB	0.703498	0.689484	0.718130	875.0
GSVT	0.572585	0.532474	0.717719	691.0
SB	0.464590	0.465390	0.463792	1553.0
SR	0.753159	0.759791	0.746674	907.0
macro avg	0.623458	0.611785	0.661579	4026.0
micro avg	0.626381	0.626381	0.626381	4026.0
weighted avg	0.600060	0.591932	0.626381	4026.0

The result of Model Number:Dataset Number:Featuregroup Number:Rescale of 31 : 1 : 5 : 1

	f1-score	precision	recall	support
AFIB	0.627408	0.693032	0.573320	204.0
GSVT	0.738102	0.728356	0.748191	300.0
SB	0.782754	0.779712	0.785825	892.0
SR	0.763945	0.753057	0.775160	681.0
macro avg	0.728052	0.738540	0.720624	2077.0

micro avg 0.756020 0.756020 0.756020 2077.0
weighted avg 0.754880 0.755041 0.756020 2077.0
The result of Model Number:Dataset Number:Featuregroup Number:Rescale of 31 : 1 : 6 : 0

	f1-score	precision	recall	support
AFIB	0.895287	0.914067	0.877289	204.0
GSVT	0.916056	0.910636	0.921551	300.0
SB	0.938389	0.936191	0.940598	892.0
SR	0.930463	0.930195	0.930739	681.0
macro avg	0.920049	0.922772	0.917544	2077.0
micro avg	0.928396	0.928396	0.928396	2077.0
weighted avg	0.928331	0.928361	0.928396	2077.0

The result of Model Number:Dataset Number:Featuregroup Number:Rescale of 31 : 1 : 6 : 1

	f1-score	precision	recall	support
AFIB	0.787563	0.788974	0.786183	875.0
GSVT	0.754145	0.758106	0.750245	691.0
SB	0.797702	0.753821	0.847015	1553.0
SR	0.662495	0.745687	0.596061	907.0
macro avg	0.750476	0.761647	0.744876	4026.0
micro avg	0.760649	0.760649	0.760649	4026.0
weighted avg	0.757562	0.760364	0.760649	4026.0

The result of Model Number:Dataset Number:Featuregroup Number:Rescale of 31 : 1 : 7 : 0

	f1-score	precision	recall	support
AFIB	0.907076	0.908932	0.905326	204.0
GSVT	0.927027	0.923423	0.930723	300.0
SB	0.947606	0.946342	0.948879	892.0
SR	0.940646	0.943427	0.937884	681.0
macro avg	0.930589	0.930531	0.930703	2077.0
micro avg	0.938374	0.938374	0.938374	2077.0
weighted avg	0.938371	0.938401	0.938374	2077.0

The result of Model Number:Dataset Number:Featuregroup Number:Rescale of 31 : 1 : 7 : 1

	f1-score	precision	recall	support
AFIB	0.723322	0.738345	0.709065	875.0
GSVT	0.705383	0.680884	0.762963	691.0
SB	0.831404	0.827655	0.835201	1553.0
SR	0.632073	0.621868	0.642675	907.0
macro avg	0.723046	0.717188	0.737476	4026.0
micro avg	0.752015	0.752015	0.752015	4026.0
weighted avg	0.741378	0.736693	0.752015	4026.0

The result of Model Number:Dataset Number:Featuregroup Number:Rescale of 31 : 1 : 8 : 0

	f1-score	precision	recall	support
AFIB	0.781239	0.808360	0.755984	204.0
GSVT	0.796219	0.787502	0.805228	300.0
SB	0.817415	0.811545	0.823371	892.0
SR	0.802816	0.806764	0.798921	681.0
macro avg	0.799422	0.803543	0.795876	2077.0
micro avg	0.806115	0.806115	0.806115	2077.0
weighted avg	0.806014	0.806192	0.806115	2077.0

The result of Model Number:Dataset Number:Featuregroup Number:Rescale of 31 : 1 : 8 : 1

	f1-score	precision	recall	support
AFIB	0.671150	0.680395	0.662448	875.0
GSVT	0.685802	0.682962	0.688851	691.0
SB	0.730486	0.720908	0.740352	1553.0
SR	0.707552	0.718118	0.697606	907.0
macro avg	0.698747	0.700596	0.697314	4026.0
micro avg	0.704951	0.704951	0.704951	4026.0
weighted avg	0.704754	0.704962	0.704951	4026.0

The result of Model Number:Dataset Number:Featuregroup Number:Rescale of 31 : 1 : 9 : 0

	f1-score	precision	recall	support
AFIB	0.888239	0.895648	0.881258	204.0
GSVT	0.911704	0.907506	0.915961	300.0
SB	0.925453	0.922166	0.928770	892.0
SR	0.916906	0.921012	0.912883	681.0
macro avg	0.910576	0.911583	0.909718	2077.0
micro avg	0.917044	0.917044	0.917044	2077.0
weighted avg	0.917010	0.917065	0.917044	2077.0

The result of Model Number:Dataset Number:Featuregroup Number:Rescale of 31 : 1 : 9 : 1

	f1-score	precision	recall	support
AFIB	0.773125	0.772417	0.773927	875.0
GSVT	0.788972	0.787441	0.790591	691.0
SB	0.837202	0.835337	0.839088	1553.0
SR	0.814351	0.819664	0.809137	907.0
macro avg	0.803412	0.803715	0.803186	4026.0
micro avg	0.809855	0.809855	0.809855	4026.0
weighted avg	0.809850	0.809911	0.809855	4026.0

The result of Model Number:Dataset Number:Featuregroup Number:Rescale of 31 : 1 : 10 : 0

	f1-score	precision	recall	support
AFIB	0.816759	0.812646	0.820949	875.0
GSVT	0.832668	0.836699	0.828689	691.0
SB	0.886842	0.884801	0.888895	1553.0

SR 0.864641 0.869267 0.860073 907.0
macro avg 0.850227 0.850853 0.849651 4026.0
micro avg 0.857301 0.857301 0.857301 4026.0
weighted avg 0.857310 0.857363 0.857301 4026.0
The result of Model Number:Dataset Number:Featuregroup Number:Rescale of 31 : 1 : 10 : 1

	f1-score	precision	recall	support
AFIB	0.741308	0.752675	0.730402	204.0
GSVT	0.758886	0.752447	0.765467	300.0
SB	0.772087	0.769595	0.774597	892.0
SR	0.764153	0.766989	0.761347	681.0
macro avg	0.759108	0.760426	0.757953	2077.0
micro avg	0.764593	0.764593	0.764593	2077.0
weighted avg	0.764556	0.764602	0.764593	2077.0

The result of Model Number:Dataset Number:Featuregroup Number:Rescale of 32 : 0 : 0 : 0

	f1-score	precision	recall	support
AFIB	0.781745	0.785467	0.778092	875.0
GSVT	0.791402	0.777020	0.806408	691.0
SB	0.837363	0.834157	0.840597	1553.0
SR	0.812613	0.826522	0.799177	907.0
macro avg	0.805781	0.805791	0.806068	4026.0
micro avg	0.811813	0.811813	0.811813	4026.0
weighted avg	0.811811	0.812048	0.811813	4026.0

The result of Model Number:Dataset Number:Featuregroup Number:Rescale of 32 : 0 : 0 : 1

	f1-score	precision	recall	support
AFIB	0.911720	0.918101	0.905460	204.0
GSVT	0.932932	0.928333	0.937592	300.0
SB	0.951475	0.949891	0.953075	892.0
SR	0.942005	0.944241	0.939790	681.0
macro avg	0.934533	0.935141	0.933979	2077.0
micro avg	0.941806	0.941806	0.941806	2077.0
weighted avg	0.941787	0.941802	0.941806	2077.0

The result of Model Number:Dataset Number:Featuregroup Number:Rescale of 32 : 0 : 1 : 0

	f1-score	precision	recall	support
AFIB	0.794165	0.783952	0.804705	875.0
GSVT	0.810332	0.817040	0.803765	691.0
SB	0.866407	0.862551	0.870308	1553.0
SR	0.841751	0.854201	0.829680	907.0
macro avg	0.828164	0.829436	0.827115	4026.0
micro avg	0.835476	0.835476	0.835476	4026.0
weighted avg	0.835527	0.835776	0.835476	4026.0

The result of Model Number:Dataset Number:Featuregroup Number:Rescale of 32 : 0 : 1 : 1

	f1-score	precision	recall	support
AFIB	0.633325	0.680935	0.592599	204.0
GSVT	0.603983	0.623155	0.586048	300.0
SB	0.629265	0.598127	0.663829	892.0
SR	0.557041	0.580984	0.535050	681.0
macro avg	0.605904	0.620800	0.594381	2077.0
micro avg	0.603375	0.603375	0.603375	2077.0
weighted avg	0.602331	0.604254	0.603375	2077.0

The result of Model Number:Dataset Number:Featuregroup Number:Rescale of 32 : 0 : 2 : 0

	f1-score	precision	recall	support
AFIB	0.727472	0.801357	0.666759	204.0
GSVT	0.671816	0.718273	0.631318	300.0
SB	0.731409	0.683409	0.786671	892.0
SR	0.633046	0.666322	0.603109	681.0
macro avg	0.690936	0.717340	0.671964	2077.0
micro avg	0.692269	0.692269	0.692269	2077.0
weighted avg	0.690164	0.694427	0.692269	2077.0

The result of Model Number:Dataset Number:Featuregroup Number:Rescale of 32 : 0 : 2 : 1

	f1-score	precision	recall	support
AFIB	0.829786	0.829934	0.829674	875.0
GSVT	0.838565	0.841398	0.835804	691.0
SB	0.880870	0.877832	0.883932	1553.0
SR	0.864143	0.867057	0.861279	907.0
macro avg	0.853341	0.854056	0.852673	4026.0
micro avg	0.858776	0.858776	0.858776	4026.0
weighted avg	0.858738	0.858742	0.858776	4026.0

The result of Model Number:Dataset Number:Featuregroup Number:Rescale of 32 : 0 : 3 : 0

	f1-score	precision	recall	support
AFIB	0.890681	0.906707	0.875475	204.0
GSVT	0.910774	0.897969	0.924102	300.0
SB	0.924788	0.913862	0.935984	892.0
SR	0.906189	0.922323	0.890620	681.0
macro avg	0.908108	0.910215	0.906545	2077.0
micro avg	0.913451	0.913451	0.913451	2077.0
weighted avg	0.913316	0.913638	0.913451	2077.0

The result of Model Number:Dataset Number:Featuregroup Number:Rescale of 32 : 0 : 3 : 1

	f1-score	precision	recall	support
AFIB	0.806227	0.800223	0.812404	875.0

GSVT 0.821264 0.824196 0.818401 691.0
SB 0.871462 0.869397 0.873537 1553.0
SR 0.851574 0.859241 0.844074 907.0
macro avg 0.837632 0.838264 0.837104 4026.0
micro avg 0.844150 0.844150 0.844150 4026.0
weighted avg 0.844188 0.844317 0.844150 4026.0
The result of Model Number:Dataset Number:Featuregroup Number:Rescale of 32 : 0 : 4 : 0

	f1-score	precision	recall	support
AFIB	0.883634	0.919299	0.850703	204.0
GSVT	0.916517	0.901629	0.932063	300.0
SB	0.940228	0.932668	0.947922	892.0
SR	0.914932	0.921520	0.908485	681.0
macro avg	0.913828	0.918779	0.909793	2077.0
micro avg	0.923152	0.923152	0.923152	2077.0
weighted avg	0.922950	0.923217	0.923152	2077.0

The result of Model Number:Dataset Number:Featuregroup Number:Rescale of 32 : 0 : 4 : 1

	f1-score	precision	recall	support
AFIB	0.663218	0.678215	0.648889	875.0
GSVT	0.703162	0.706154	0.700236	691.0
SB	0.763488	0.759890	0.767125	1553.0
SR	0.723394	0.712298	0.734872	907.0
macro avg	0.713315	0.714139	0.712780	4026.0
micro avg	0.722681	0.722681	0.722681	4026.0
weighted avg	0.722309	0.722194	0.722681	4026.0

The result of Model Number:Dataset Number:Featuregroup Number:Rescale of 32 : 0 : 5 : 0

	f1-score	precision	recall	support
AFIB	0.893479	0.906659	0.880774	204.0
GSVT	0.915413	0.911820	0.919064	300.0
SB	0.935744	0.933867	0.937631	892.0
SR	0.929106	0.929246	0.928975	681.0
macro avg	0.918435	0.920398	0.916611	2077.0
micro avg	0.926527	0.926527	0.926527	2077.0
weighted avg	0.926480	0.926495	0.926527	2077.0

The result of Model Number:Dataset Number:Featuregroup Number:Rescale of 32 : 0 : 5 : 1

	f1-score	precision	recall	support
AFIB	0.902806	0.919561	0.886875	204.0
GSVT	0.922658	0.917820	0.927566	300.0
SB	0.937388	0.934056	0.940749	892.0
SR	0.927738	0.929410	0.926112	681.0
macro avg	0.922647	0.925212	0.920326	2077.0

micro avg 0.928754 0.928754 0.928754 2077.0
weighted avg 0.928700 0.928764 0.928754 2077.0
The result of Model Number:Dataset Number:Featuregroup Number:Rescale of 32 : 0 : 6 : 0

	f1-score	precision	recall	support
AFIB	0.764886	0.754261	0.775887	875.0
GSVT	0.792219	0.802788	0.782028	691.0
SB	0.841594	0.839870	0.843328	1553.0
SR	0.819690	0.826023	0.813453	907.0
macro avg	0.804597	0.805735	0.803674	4026.0
micro avg	0.811419	0.811419	0.811419	4026.0
weighted avg	0.811513	0.811780	0.811419	4026.0

The result of Model Number:Dataset Number:Featuregroup Number:Rescale of 32 : 0 : 6 : 1

	f1-score	precision	recall	support
AFIB	0.761823	0.755012	0.768792	875.0
GSVT	0.774055	0.777117	0.771044	691.0
SB	0.825308	0.823776	0.826849	1553.0
SR	0.804395	0.811831	0.797123	907.0
macro avg	0.791395	0.791934	0.790952	4026.0
micro avg	0.797956	0.797956	0.797956	4026.0
weighted avg	0.798002	0.798132	0.797956	4026.0

The result of Model Number:Dataset Number:Featuregroup Number:Rescale of 32 : 0 : 7 : 0

	f1-score	precision	recall	support
AFIB	0.789612	0.808560	0.771891	204.0
GSVT	0.817688	0.812949	0.822582	300.0
SB	0.835151	0.832088	0.838240	892.0
SR	0.827894	0.828508	0.827290	681.0
macro avg	0.817586	0.820526	0.815001	2077.0
micro avg	0.825871	0.825871	0.825871	2077.0
weighted avg	0.825777	0.825839	0.825871	2077.0

The result of Model Number:Dataset Number:Featuregroup Number:Rescale of 32 : 0 : 7 : 1

	f1-score	precision	recall	support
AFIB	0.724574	0.705540	0.769934	875.0
GSVT	0.697034	0.659751	0.794604	691.0
SB	0.660475	0.656301	0.664723	1553.0
SR	0.641547	0.652601	0.631041	907.0
macro avg	0.680908	0.668548	0.715075	4026.0
micro avg	0.702293	0.702293	0.702293	4026.0
weighted avg	0.676417	0.666761	0.702293	4026.0

The result of Model Number:Dataset Number:Featuregroup Number:Rescale of 32 : 0 : 8 : 0

	f1-score	precision	recall	support
AFIB	0.575249	0.566039	0.647272	204.0
GSVT	0.673465	0.665650	0.681710	300.0
SB	0.696015	0.693386	0.698667	892.0
SR	0.546106	0.548929	0.543404	681.0
macro avg	0.622709	0.618501	0.642763	2077.0
micro avg	0.640263	0.640263	0.640263	2077.0
weighted avg	0.631745	0.629508	0.640263	2077.0

The result of Model Number:Dataset Number:Featuregroup Number:Rescale of 32 : 0 : 8 : 1

	f1-score	precision	recall	support
AFIB	0.892129	0.888650	0.895699	875.0
GSVT	0.908826	0.910742	0.906971	691.0
SB	0.964749	0.962344	0.967171	1553.0
SR	0.942494	0.948824	0.936267	907.0
macro avg	0.927050	0.927640	0.926527	4026.0
micro avg	0.934343	0.934343	0.934343	4026.0
weighted avg	0.934354	0.934425	0.934343	4026.0

The result of Model Number:Dataset Number:Featuregroup Number:Rescale of 32 : 0 : 9 : 0

	f1-score	precision	recall	support
AFIB	0.656295	0.672662	0.640754	875.0
GSVT	0.698183	0.700859	0.695559	691.0
SB	0.756073	0.752413	0.759772	1553.0
SR	0.716421	0.704480	0.728821	907.0
macro avg	0.706743	0.707604	0.706227	4026.0
micro avg	0.715911	0.715911	0.715911	4026.0
weighted avg	0.715519	0.715433	0.715911	4026.0

The result of Model Number:Dataset Number:Featuregroup Number:Rescale of 32 : 0 : 9 : 1

	f1-score	precision	recall	support
AFIB	0.927988	0.934928	0.921187	204.0
GSVT	0.951700	0.946677	0.956777	300.0
SB	0.967761	0.966467	0.969062	892.0
SR	0.959237	0.961077	0.957408	681.0
macro avg	0.951672	0.952287	0.951108	2077.0
micro avg	0.958764	0.958764	0.958764	2077.0
weighted avg	0.958740	0.958743	0.958764	2077.0

The result of Model Number:Dataset Number:Featuregroup Number:Rescale of 32 : 0 : 10 : 0

	f1-score	precision	recall	support
AFIB	0.765148	0.755379	0.775218	875.0
GSVT	0.778578	0.783043	0.774185	691.0
SB	0.833420	0.829920	0.836958	1553.0

SR 0.808881 0.821973 0.796213 907.0
macro avg 0.796507 0.797579 0.795643 4026.0
micro avg 0.803586 0.803586 0.803586 4026.0
weighted avg 0.803641 0.803884 0.803586 4026.0
The result of Model Number:Dataset Number:Featuregroup Number:Rescale of 32 : 0 : 10 : 1

	f1-score	precision	recall	support
AFIB	0.840559	0.847345	0.833953	204.0
GSVT	0.859846	0.855630	0.864134	300.0
SB	0.876627	0.875655	0.877610	892.0
SR	0.870082	0.871239	0.868934	681.0
macro avg	0.861778	0.862467	0.861157	2077.0
micro avg	0.868531	0.868531	0.868531	2077.0
weighted avg	0.868515	0.868534	0.868531	2077.0

The result of Model Number:Dataset Number:Featuregroup Number:Rescale of 32 : 1 : 0 : 0

	f1-score	precision	recall	support
AFIB	0.709420	0.725478	0.694149	204.0
GSVT	0.728299	0.724733	0.731932	300.0
SB	0.743198	0.741052	0.745362	892.0
SR	0.738423	0.738104	0.738754	681.0
macro avg	0.729835	0.732342	0.727549	2077.0
micro avg	0.736226	0.736226	0.736226	2077.0
weighted avg	0.736163	0.736199	0.736226	2077.0

The result of Model Number:Dataset Number:Featuregroup Number:Rescale of 32 : 1 : 0 : 1

	f1-score	precision	recall	support
AFIB	0.608156	0.609177	0.623229	204.0
GSVT	0.602439	0.625817	0.581521	300.0
SB	0.639017	0.603973	0.678641	892.0
SR	0.553892	0.605008	0.512142	681.0
macro avg	0.600876	0.610994	0.598883	2077.0
micro avg	0.604579	0.604579	0.604579	2077.0
weighted avg	0.602792	0.607979	0.604579	2077.0

The result of Model Number:Dataset Number:Featuregroup Number:Rescale of 32 : 1 : 1 : 0

	f1-score	precision	recall	support
AFIB	0.723058	0.713758	0.732654	875.0
GSVT	0.737776	0.743884	0.731798	691.0
SB	0.788831	0.785320	0.792383	1553.0
SR	0.766383	0.777718	0.755392	907.0
macro avg	0.754012	0.755170	0.753057	4026.0
micro avg	0.760670	0.760670	0.760670	4026.0
weighted avg	0.760716	0.760943	0.760670	4026.0

The result of Model Number:Dataset Number:Featuregroup Number:Rescale of 32 : 1 : 1 : 1

	f1-score	precision	recall	support
AFIB	0.710170	0.721702	0.699038	875.0
GSVT	0.755286	0.757653	0.752980	691.0
SB	0.821713	0.818882	0.824569	1553.0
SR	0.777923	0.769258	0.786832	907.0
macro avg	0.766273	0.766874	0.765855	4026.0
micro avg	0.776498	0.776498	0.776498	4026.0
weighted avg	0.776204	0.776073	0.776498	4026.0

The result of Model Number:Dataset Number:Featuregroup Number:Rescale of 32 : 1 : 2 : 0

	f1-score	precision	recall	support
AFIB	0.842268	0.848162	0.836485	204.0
GSVT	0.861864	0.857615	0.866168	300.0
SB	0.878994	0.877531	0.880472	892.0
SR	0.870245	0.872311	0.868200	681.0
macro avg	0.863343	0.863905	0.862831	2077.0
micro avg	0.870062	0.870062	0.870062	2077.0
weighted avg	0.870044	0.870058	0.870062	2077.0

The result of Model Number:Dataset Number:Featuregroup Number:Rescale of 32 : 1 : 2 : 1

	f1-score	precision	recall	support
AFIB	0.792601	0.824592	0.763063	204.0
GSVT	0.822097	0.808742	0.836041	300.0
SB	0.843365	0.836584	0.850266	892.0
SR	0.820675	0.826585	0.814892	681.0
macro avg	0.819684	0.824126	0.816066	2077.0
micro avg	0.828048	0.828048	0.828048	2077.0
weighted avg	0.827867	0.828106	0.828048	2077.0

The result of Model Number:Dataset Number:Featuregroup Number:Rescale of 32 : 1 : 3 : 0

	f1-score	precision	recall	support
AFIB	0.870519	0.877419	0.863748	875.0
GSVT	0.881555	0.877669	0.885506	691.0
SB	0.937430	0.933724	0.941172	1553.0
SR	0.914394	0.916978	0.911869	907.0
macro avg	0.900974	0.901447	0.900574	4026.0
micro avg	0.908189	0.908189	0.908189	4026.0
weighted avg	0.908108	0.908093	0.908189	4026.0

The result of Model Number:Dataset Number:Featuregroup Number:Rescale of 32 : 1 : 3 : 1

	f1-score	precision	recall	support
AFIB	0.731472	0.737530	0.823903	204.0

GSVT 0.793232 0.759281 0.894951 300.0
SB 0.724250 0.721734 0.726787 892.0
SR 0.717049 0.717185 0.716918 681.0
macro avg 0.741501 0.733932 0.790640 2077.0
micro avg 0.757379 0.757379 0.757379 2077.0
weighted avg 0.732562 0.727217 0.757379 2077.0
The result of Model Number:Dataset Number:Featuregroup Number:Rescale of 32 : 1 : 4 : 0

	f1-score	precision	recall	support
AFIB	0.837984	0.907939	0.779709	204.0
GSVT	0.898835	0.887533	0.910795	300.0
SB	0.921781	0.908868	0.935175	892.0
SR	0.896637	0.900837	0.892912	681.0
macro avg	0.888810	0.901294	0.879648	2077.0
micro avg	0.902527	0.902527	0.902527	2077.0
weighted avg	0.901992	0.903062	0.902527	2077.0

The result of Model Number:Dataset Number:Featuregroup Number:Rescale of 32 : 1 : 4 : 1

	f1-score	precision	recall	support
AFIB	0.894399	0.898469	0.890420	875.0
GSVT	0.898745	0.889787	0.907962	691.0
SB	0.947279	0.936957	0.957832	1553.0
SR	0.905970	0.927418	0.885495	907.0
macro avg	0.911598	0.913158	0.910427	4026.0
micro avg	0.918325	0.918325	0.918325	4026.0
weighted avg	0.918150	0.918347	0.918325	4026.0

The result of Model Number:Dataset Number:Featuregroup Number:Rescale of 32 : 1 : 5 : 0

	f1-score	precision	recall	support
AFIB	0.830443	0.829107	0.832122	875.0
GSVT	0.849805	0.845375	0.854331	691.0
SB	0.888515	0.880988	0.896180	1553.0
SR	0.854597	0.873596	0.836695	907.0
macro avg	0.855840	0.857266	0.854832	4026.0
micro avg	0.861674	0.861674	0.861674	4026.0
weighted avg	0.861609	0.861935	0.861674	4026.0

The result of Model Number:Dataset Number:Featuregroup Number:Rescale of 32 : 1 : 5 : 1

	f1-score	precision	recall	support
AFIB	0.719371	0.735764	0.703807	875.0
GSVT	0.765503	0.767016	0.764252	691.0
SB	0.837511	0.832927	0.842149	1553.0
SR	0.791574	0.781972	0.801525	907.0
macro avg	0.778490	0.779420	0.777933	4026.0

micro avg 0.789560 0.789560 0.789560 4026.0
weighted avg 0.789127 0.789018 0.789560 4026.0
The result of Model Number:Dataset Number:Featuregroup Number:Rescale of 32 : 1 : 6 : 0

	f1-score	precision	recall	support
AFIB	0.641212	0.643277	0.639219	875.0
GSVT	0.650226	0.639903	0.660947	691.0
SB	0.685119	0.679399	0.690942	1553.0
SR	0.650680	0.667206	0.634977	907.0
macro avg	0.656809	0.657446	0.656521	4026.0
micro avg	0.661944	0.661944	0.661944	4026.0
weighted avg	0.661829	0.662022	0.661944	4026.0

The result of Model Number:Dataset Number:Featuregroup Number:Rescale of 32 : 1 : 6 : 1

	f1-score	precision	recall	support
AFIB	0.872855	0.877441	0.868370	875.0
GSVT	0.887585	0.889233	0.886056	691.0
SB	0.940074	0.933398	0.946852	1553.0
SR	0.922050	0.927731	0.916460	907.0
macro avg	0.905641	0.906951	0.904434	4026.0
micro avg	0.912513	0.912513	0.912513	4026.0
weighted avg	0.912395	0.912380	0.912513	4026.0

The result of Model Number:Dataset Number:Featuregroup Number:Rescale of 32 : 1 : 7 : 0

	f1-score	precision	recall	support
AFIB	0.873716	0.876666	0.870817	875.0
GSVT	0.871409	0.865271	0.877713	691.0
SB	0.916882	0.908372	0.925559	1553.0
SR	0.882024	0.899022	0.865701	907.0
macro avg	0.886008	0.887333	0.884947	4026.0
micro avg	0.891964	0.891964	0.891964	4026.0
weighted avg	0.891843	0.891977	0.891964	4026.0

The result of Model Number:Dataset Number:Featuregroup Number:Rescale of 32 : 1 : 7 : 1

	f1-score	precision	recall	support
AFIB	0.591679	0.648628	0.544124	204.0
GSVT	0.688124	0.681054	0.695472	300.0
SB	0.729464	0.726627	0.732326	892.0
SR	0.712563	0.702710	0.722709	681.0
macro avg	0.680458	0.689754	0.673658	2077.0
micro avg	0.705365	0.705365	0.705365	2077.0
weighted avg	0.704418	0.704541	0.705365	2077.0

The result of Model Number:Dataset Number:Featuregroup Number:Rescale of 32 : 1 : 8 : 0

	f1-score	precision	recall	support
AFIB	0.831939	0.820136	0.844107	875.0
GSVT	0.849055	0.857330	0.840944	691.0
SB	0.903560	0.900616	0.906526	1553.0
SR	0.882459	0.893738	0.871471	907.0
macro avg	0.866753	0.867955	0.865762	4026.0
micro avg	0.873807	0.873807	0.873807	4026.0
weighted avg	0.873885	0.874146	0.873807	4026.0

The result of Model Number:Dataset Number:Featuregroup Number:Rescale of 32 : 1 : 8 : 1

	f1-score	precision	recall	support
AFIB	0.867795	0.878563	0.857580	204.0
GSVT	0.883356	0.877320	0.889567	300.0
SB	0.904274	0.902862	0.905692	892.0
SR	0.897778	0.899235	0.896335	681.0
macro avg	0.888301	0.889495	0.887294	2077.0
micro avg	0.895570	0.895570	0.895570	2077.0
weighted avg	0.895540	0.895597	0.895570	2077.0

The result of Model Number:Dataset Number:Featuregroup Number:Rescale of 32 : 1 : 9 : 0

	f1-score	precision	recall	support
AFIB	0.809537	0.798790	0.820640	875.0
GSVT	0.825968	0.833798	0.818319	691.0
SB	0.878948	0.876869	0.881041	1553.0
SR	0.855104	0.864095	0.846333	907.0
macro avg	0.842389	0.843388	0.841583	4026.0
micro avg	0.849329	0.849329	0.849329	4026.0
weighted avg	0.849397	0.849629	0.849329	4026.0

The result of Model Number:Dataset Number:Featuregroup Number:Rescale of 32 : 1 : 9 : 1

	f1-score	precision	recall	support
AFIB	0.773922	0.785172	0.763341	204.0
GSVT	0.790990	0.780289	0.802053	300.0
SB	0.802895	0.794528	0.811447	892.0
SR	0.786982	0.800004	0.774390	681.0
macro avg	0.788697	0.789998	0.787807	2077.0
micro avg	0.793215	0.793215	0.793215	2077.0
weighted avg	0.793112	0.793348	0.793215	2077.0

The result of Model Number:Dataset Number:Featuregroup Number:Rescale of 32 : 1 : 10 : 0

	f1-score	precision	recall	support
AFIB	0.618692	0.695664	0.557601	204.0
GSVT	0.734033	0.718358	0.750439	300.0
SB	0.783593	0.779599	0.787633	892.0

	f1-score	precision	recall	support
SR	0.766014	0.756314	0.776016	681.0
macro avg	0.725583	0.737484	0.717922	2077.0
micro avg	0.755858	0.755858	0.755858	2077.0
weighted avg	0.754475	0.754875	0.755858	2077.0

The result of Model Number:Dataset Number:Featuregroup Number:Rescale of 32 : 1 : 10 : 1

	f1-score	precision	recall	support
AFIB	0.662914	0.662174	0.663676	875.0
GSVT	0.675040	0.666597	0.683719	691.0
SB	0.709126	0.703425	0.714926	1553.0
SR	0.677488	0.695264	0.660610	907.0
macro avg	0.681142	0.681865	0.680733	4026.0
micro avg	0.686195	0.686195	0.686195	4026.0
weighted avg	0.686105	0.686300	0.686195	4026.0

The result of Model Number:Dataset Number:Featuregroup Number:Rescale of 33 : 0 : 0 : 0

	f1-score	precision	recall	support
AFIB	0.777782	0.783225	0.772442	204.0
GSVT	0.795878	0.791954	0.799853	300.0
SB	0.811696	0.810345	0.813061	892.0
SR	0.803618	0.805526	0.801729	681.0
macro avg	0.797243	0.797763	0.796771	2077.0
micro avg	0.803448	0.803448	0.803448	2077.0
weighted avg	0.803432	0.803445	0.803448	2077.0

The result of Model Number:Dataset Number:Featuregroup Number:Rescale of 33 : 0 : 0 : 1

	f1-score	precision	recall	support
AFIB	0.818172	0.837757	0.800679	204.0
GSVT	0.842265	0.830397	0.854829	300.0
SB	0.864149	0.855370	0.873151	892.0
SR	0.843495	0.855675	0.831725	681.0
macro avg	0.842020	0.844800	0.840096	2077.0
micro avg	0.849804	0.849804	0.849804	2077.0
weighted avg	0.849701	0.850133	0.849804	2077.0

The result of Model Number:Dataset Number:Featuregroup Number:Rescale of 33 : 0 : 1 : 0

	f1-score	precision	recall	support
AFIB	0.661965	0.673876	0.650664	204.0
GSVT	0.676899	0.667382	0.686804	300.0
SB	0.687315	0.679194	0.695635	892.0
SR	0.673492	0.685482	0.661921	681.0
macro avg	0.674918	0.676484	0.673756	2077.0
micro avg	0.678889	0.678889	0.678889	2077.0
weighted avg	0.678788	0.679027	0.678889	2077.0

The result of Model Number:Dataset Number:Featuregroup Number:Rescale of 33 : 0 : 1 : 1

	f1-score	precision	recall	support
AFIB	0.888200	0.926668	0.853051	204.0
GSVT	0.830313	0.838061	0.823123	300.0
SB	0.860679	0.818364	0.907670	892.0
SR	0.767099	0.815509	0.724362	681.0
macro avg	0.836573	0.849651	0.827051	2077.0
micro avg	0.829991	0.829991	0.829991	2077.0
weighted avg	0.828313	0.830910	0.829991	2077.0

The result of Model Number:Dataset Number:Featuregroup Number:Rescale of 33 : 0 : 2 : 0

	f1-score	precision	recall	support
AFIB	0.571804	0.633568	0.521118	204.0
GSVT	0.662843	0.652063	0.674090	300.0
SB	0.706882	0.703663	0.710130	892.0
SR	0.689802	0.681024	0.698819	681.0
macro avg	0.657833	0.667580	0.651039	2077.0
micro avg	0.682651	0.682651	0.682651	2077.0
weighted avg	0.681654	0.681903	0.682651	2077.0

The result of Model Number:Dataset Number:Featuregroup Number:Rescale of 33 : 0 : 2 : 1

	f1-score	precision	recall	support
AFIB	0.682359	0.684054	0.680719	875.0
GSVT	0.694111	0.696196	0.692126	691.0
SB	0.736326	0.731328	0.741398	1553.0
SR	0.722315	0.727677	0.717059	907.0
macro avg	0.708778	0.709814	0.707825	4026.0
micro avg	0.714270	0.714270	0.714270	4026.0
weighted avg	0.714195	0.714201	0.714270	4026.0

The result of Model Number:Dataset Number:Featuregroup Number:Rescale of 33 : 0 : 3 : 0

	f1-score	precision	recall	support
AFIB	0.913049	0.923724	0.902793	204.0
GSVT	0.938166	0.933853	0.942546	300.0
SB	0.951449	0.948491	0.954433	892.0
SR	0.943255	0.945929	0.940637	681.0
macro avg	0.936480	0.937999	0.935102	2077.0
micro avg	0.943121	0.943121	0.943121	2077.0
weighted avg	0.943072	0.943104	0.943121	2077.0

The result of Model Number:Dataset Number:Featuregroup Number:Rescale of 33 : 0 : 3 : 1

	f1-score	precision	recall	support
AFIB	0.893379	0.903306	0.884126	204.0

GSVT 0.921052 0.915809 0.926623 300.0
SB 0.939375 0.938116 0.940638 892.0
SR 0.931384 0.932632 0.930152 681.0
macro avg 0.921297 0.922466 0.920385 2077.0
micro avg 0.929625 0.929625 0.929625 2077.0
weighted avg 0.929591 0.929677 0.929625 2077.0
The result of Model Number:Dataset Number:Featuregroup Number:Rescale of 33 : 0 : 4 : 0

	f1-score	precision	recall	support
AFIB	0.801608	0.804366	0.798970	875.0
GSVT	0.816826	0.800530	0.833947	691.0
SB	0.864676	0.860000	0.869408	1553.0
SR	0.831232	0.850559	0.812813	907.0
macro avg	0.828585	0.828864	0.828785	4026.0
micro avg	0.835263	0.835263	0.835263	4026.0
weighted avg	0.835222	0.835574	0.835263	4026.0

The result of Model Number:Dataset Number:Featuregroup Number:Rescale of 33 : 0 : 4 : 1

	f1-score	precision	recall	support
AFIB	0.843354	0.845044	0.841706	875.0
GSVT	0.845442	0.840878	0.850079	691.0
SB	0.883771	0.879050	0.888550	1553.0
SR	0.862628	0.872858	0.852660	907.0
macro avg	0.858799	0.859458	0.858249	4026.0
micro avg	0.863681	0.863681	0.863681	4026.0
weighted avg	0.863645	0.863713	0.863681	4026.0

The result of Model Number:Dataset Number:Featuregroup Number:Rescale of 33 : 0 : 5 : 0

	f1-score	precision	recall	support
AFIB	0.697184	0.713112	0.682129	204.0
GSVT	0.728856	0.724319	0.733469	300.0
SB	0.744597	0.742288	0.746932	892.0
SR	0.736070	0.736410	0.735750	681.0
macro avg	0.726677	0.729032	0.724570	2077.0
micro avg	0.734956	0.734956	0.734956	2077.0
weighted avg	0.734871	0.734900	0.734956	2077.0

The result of Model Number:Dataset Number:Featuregroup Number:Rescale of 33 : 0 : 5 : 1

	f1-score	precision	recall	support
AFIB	0.769878	0.802273	0.740067	204.0
GSVT	0.795708	0.783529	0.808393	300.0
SB	0.814900	0.809056	0.820840	892.0
SR	0.793750	0.797787	0.789785	681.0
macro avg	0.793559	0.798161	0.789771	2077.0

micro avg 0.800926 0.800926 0.800926 2077.0
weighted avg 0.800772 0.801008 0.800926 2077.0
The result of Model Number:Dataset Number:Featuregroup Number:Rescale of 33 : 0 : 6 : 0

	f1-score	precision	recall	support
AFIB	0.690082	0.695046	0.685190	875.0
GSVT	0.688982	0.684857	0.693169	691.0
SB	0.726776	0.718723	0.735012	1553.0
SR	0.697397	0.709840	0.685407	907.0
macro avg	0.700809	0.702117	0.699694	4026.0
micro avg	0.705827	0.705827	0.705827	4026.0
weighted avg	0.705696	0.705763	0.705827	4026.0

The result of Model Number:Dataset Number:Featuregroup Number:Rescale of 33 : 0 : 6 : 1

	f1-score	precision	recall	support
AFIB	0.755169	0.762134	0.748512	204.0
GSVT	0.782777	0.782805	0.782770	300.0
SB	0.799171	0.797751	0.800602	892.0
SR	0.790689	0.790478	0.790920	681.0
macro avg	0.781952	0.783292	0.780701	2077.0
micro avg	0.789736	0.789736	0.789736	2077.0
weighted avg	0.789700	0.789709	0.789736	2077.0

The result of Model Number:Dataset Number:Featuregroup Number:Rescale of 33 : 0 : 7 : 0

	f1-score	precision	recall	support
AFIB	0.687949	0.681204	0.735555	204.0
GSVT	0.616852	0.609994	0.624021	300.0
SB	0.792693	0.786921	0.798556	892.0
SR	0.779399	0.784797	0.774138	681.0
macro avg	0.719223	0.715729	0.733067	2077.0
micro avg	0.759152	0.759152	0.759152	2077.0
weighted avg	0.752648	0.750286	0.759152	2077.0

The result of Model Number:Dataset Number:Featuregroup Number:Rescale of 33 : 0 : 7 : 1

	f1-score	precision	recall	support
AFIB	0.652193	0.636854	0.686616	875.0
GSVT	0.626698	0.620921	0.640979	691.0
SB	0.679697	0.631838	0.736183	1553.0
SR	0.423799	0.531513	0.352932	907.0
macro avg	0.595597	0.605281	0.604177	4026.0
micro avg	0.622729	0.622729	0.622729	4026.0
weighted avg	0.606973	0.608453	0.622729	4026.0

The result of Model Number:Dataset Number:Featuregroup Number:Rescale of 33 : 0 : 8 : 0

f1-score precision recall support
AFIB 0.807204 0.785999 0.857738 875.0
GSVT 0.776523 0.734989 0.885220 691.0
SB 0.735796 0.731146 0.740527 1553.0
SR 0.714709 0.727023 0.703005 907.0
macro avg 0.758558 0.744789 0.796622 4026.0
micro avg 0.782383 0.782383 0.782383 4026.0
weighted avg 0.753555 0.742798 0.782383 4026.0
The result of Model Number:Dataset Number:Featuregroup Number:Rescale of 33 : 0 : 8 : 1

	f1-score	precision	recall	support
AFIB	0.693474	0.706912	0.680849	204.0
GSVT	0.720365	0.714342	0.726624	300.0
SB	0.733807	0.732334	0.735287	892.0
SR	0.726810	0.727563	0.726061	681.0
macro avg	0.718614	0.720288	0.717205	2077.0
micro avg	0.725664	0.725664	0.725664	2077.0
weighted avg	0.725610	0.725674	0.725664	2077.0

The result of Model Number:Dataset Number:Featuregroup Number:Rescale of 33 : 0 : 9 : 0

	f1-score	precision	recall	support
AFIB	0.878684	0.879468	0.877991	875.0
GSVT	0.894779	0.897126	0.892594	691.0
SB	0.949820	0.943496	0.956239	1553.0
SR	0.931313	0.939816	0.922997	907.0
macro avg	0.913649	0.914976	0.912455	4026.0
micro avg	0.920820	0.920820	0.920820	4026.0
weighted avg	0.920743	0.920792	0.920820	4026.0

The result of Model Number:Dataset Number:Featuregroup Number:Rescale of 33 : 0 : 9 : 1

	f1-score	precision	recall	support
AFIB	0.670929	0.671406	0.670466	875.0
GSVT	0.679970	0.681311	0.678693	691.0
SB	0.722242	0.719842	0.724661	1553.0
SR	0.705272	0.707893	0.702693	907.0
macro avg	0.694603	0.695113	0.694128	4026.0
micro avg	0.700043	0.700043	0.700043	4026.0
weighted avg	0.700011	0.700010	0.700043	4026.0

The result of Model Number:Dataset Number:Featuregroup Number:Rescale of 33 : 0 : 10 : 0

	f1-score	precision	recall	support
AFIB	0.803687	0.794714	0.812874	875.0
GSVT	0.819677	0.827185	0.812317	691.0
SB	0.874223	0.871376	0.877092	1553.0

SR 0.851560 0.860091 0.843199 907.0
macro avg 0.837287 0.838341 0.836371 4026.0
micro avg 0.844382 0.844382 0.844382 4026.0
weighted avg 0.844425 0.844587 0.844382 4026.0
The result of Model Number:Dataset Number:Featuregroup Number:Rescale of 33 : 0 : 10 : 1

	f1-score	precision	recall	support
AFIB	0.741086	0.736239	0.746060	875.0
GSVT	0.763112	0.769605	0.756829	691.0
SB	0.812436	0.810666	0.814215	1553.0
SR	0.792636	0.795819	0.789491	907.0
macro avg	0.777317	0.778082	0.776649	4026.0
micro avg	0.783983	0.783983	0.783983	4026.0
weighted avg	0.784002	0.784098	0.783983	4026.0

The result of Model Number:Dataset Number:Featuregroup Number:Rescale of 33 : 1 : 0 : 0

	f1-score	precision	recall	support
AFIB	0.875417	0.864240	0.886938	875.0
GSVT	0.890783	0.895891	0.885756	691.0
SB	0.953528	0.949523	0.957575	1553.0
SR	0.925452	0.940431	0.910958	907.0
macro avg	0.911295	0.912521	0.910307	4026.0
micro avg	0.919394	0.919394	0.919394	4026.0
weighted avg	0.919457	0.919734	0.919394	4026.0

The result of Model Number:Dataset Number:Featuregroup Number:Rescale of 33 : 1 : 0 : 1

	f1-score	precision	recall	support
AFIB	0.652813	0.685430	0.623487	204.0
GSVT	0.672494	0.660031	0.685671	300.0
SB	0.687884	0.680950	0.694975	892.0
SR	0.668806	0.674780	0.662998	681.0
macro avg	0.670499	0.675298	0.666783	2077.0
micro avg	0.676125	0.676125	0.676125	2077.0
weighted avg	0.675961	0.676346	0.676125	2077.0

The result of Model Number:Dataset Number:Featuregroup Number:Rescale of 33 : 1 : 1 : 0

	f1-score	precision	recall	support
AFIB	0.853756	0.881486	0.827885	204.0
GSVT	0.877327	0.866140	0.888983	300.0
SB	0.902078	0.898677	0.905516	892.0
SR	0.889462	0.891072	0.887891	681.0
macro avg	0.880656	0.884344	0.877568	2077.0
micro avg	0.889724	0.889724	0.889724	2077.0
weighted avg	0.889620	0.889795	0.889724	2077.0

The result of Model Number:Dataset Number:Featuregroup Number:Rescale of 33 : 1 : 1 : 1

	f1-score	precision	recall	support
AFIB	0.630284	0.620793	0.645316	875.0
GSVT	0.601807	0.600055	0.606941	691.0
SB	0.642652	0.598850	0.698713	1553.0
SR	0.476053	0.636341	0.394467	907.0
macro avg	0.587699	0.614010	0.586359	4026.0
micro avg	0.602814	0.602814	0.602814	4026.0
weighted avg	0.595421	0.612272	0.602814	4026.0

The result of Model Number:Dataset Number:Featuregroup Number:Rescale of 33 : 1 : 2 : 0

	f1-score	precision	recall	support
AFIB	0.173163	0.174981	0.171382	175.0
GSVT	0.173442	0.171599	0.175324	138.2
SB	0.182665	0.181964	0.183370	310.6
SR	0.178437	0.179331	0.177552	181.4
macro avg	0.176927	0.176969	0.176907	805.2
micro avg	0.178073	0.178073	0.178073	805.2
weighted avg	0.178064	0.178074	0.178073	805.2

The result of Model Number:Dataset Number:Featuregroup Number:Rescale of 33 : 1 : 2 : 1

	f1-score	precision	recall	support
AFIB	0.701975	0.694138	0.710000	875.0
GSVT	0.715942	0.722499	0.709513	691.0
SB	0.763585	0.761098	0.766091	1553.0
SR	0.743789	0.751241	0.736487	907.0
macro avg	0.731323	0.732244	0.730523	4026.0
micro avg	0.737520	0.737520	0.737520	4026.0
weighted avg	0.737558	0.737699	0.737520	4026.0

The result of Model Number:Dataset Number:Featuregroup Number:Rescale of 33 : 1 : 3 : 0

	f1-score	precision	recall	support
AFIB	0.689137	0.710036	0.669462	875.0
GSVT	0.735664	0.730562	0.740877	691.0
SB	0.804906	0.801827	0.808014	1553.0
SR	0.761247	0.749821	0.773071	907.0
macro avg	0.747738	0.748062	0.747856	4026.0
micro avg	0.758506	0.758506	0.758506	4026.0
weighted avg	0.758025	0.757930	0.758506	4026.0

The result of Model Number:Dataset Number:Featuregroup Number:Rescale of 33 : 1 : 3 : 1

	f1-score	precision	recall	support
AFIB	0.800354	0.789269	0.811788	875.0

GSVT 0.818106 0.824727 0.811594 691.0
SB 0.868409 0.865914 0.870925 1553.0
SR 0.847284 0.858311 0.836544 907.0
macro avg 0.833538 0.834555 0.832713 4026.0
micro avg 0.840143 0.840143 0.840143 4026.0
weighted avg 0.840225 0.840474 0.840143 4026.0
The result of Model Number:Dataset Number:Featuregroup Number:Rescale of 33 : 1 : 4 : 0

	f1-score	precision	recall	support
AFIB	0.838594	0.828657	0.848843	875.0
GSVT	0.850530	0.858752	0.842547	691.0
SB	0.902696	0.899242	0.906183	1553.0
SR	0.881650	0.891822	0.871733	907.0
macro avg	0.868367	0.869618	0.867326	4026.0
micro avg	0.875038	0.875038	0.875038	4026.0
weighted avg	0.875069	0.875280	0.875038	4026.0

The result of Model Number:Dataset Number:Featuregroup Number:Rescale of 33 : 1 : 4 : 1

	f1-score	precision	recall	support
AFIB	0.773587	0.774277	0.772977	875.0
GSVT	0.787757	0.789823	0.785833	691.0
SB	0.836214	0.830647	0.841866	1553.0
SR	0.819921	0.827407	0.812599	907.0
macro avg	0.804370	0.805539	0.803319	4026.0
micro avg	0.810683	0.810683	0.810683	4026.0
weighted avg	0.810616	0.810659	0.810683	4026.0

The result of Model Number:Dataset Number:Featuregroup Number:Rescale of 33 : 1 : 5 : 0

	f1-score	precision	recall	support
AFIB	0.588646	0.641238	0.621623	204.0
GSVT	0.799544	0.786474	0.813332	300.0
SB	0.680659	0.675475	0.685975	892.0
SR	0.832335	0.822918	0.841996	681.0
macro avg	0.725296	0.731526	0.740731	2077.0
micro avg	0.749205	0.749205	0.749205	2077.0
weighted avg	0.738525	0.736488	0.749205	2077.0

The result of Model Number:Dataset Number:Featuregroup Number:Rescale of 33 : 1 : 5 : 1

	f1-score	precision	recall	support
AFIB	0.901373	0.902792	0.900075	204.0
GSVT	0.923172	0.921732	0.924695	300.0
SB	0.942217	0.941067	0.943376	892.0
SR	0.935618	0.937414	0.933834	681.0
macro avg	0.925595	0.925751	0.925495	2077.0

micro avg 0.933296 0.933296 0.933296 2077.0
weighted avg 0.933291 0.933317 0.933296 2077.0
The result of Model Number:Dataset Number:Featuregroup Number:Rescale of 33 : 1 : 6 : 0

	f1-score	precision	recall	support
AFIB	0.760346	0.755078	0.765840	875.0
GSVT	0.772076	0.775209	0.769093	691.0
SB	0.837704	0.828993	0.846604	1553.0
SR	0.795407	0.813915	0.777795	907.0
macro avg	0.791383	0.793299	0.789833	4026.0
micro avg	0.800246	0.800246	0.800246	4026.0
weighted avg	0.800098	0.800301	0.800246	4026.0

The result of Model Number:Dataset Number:Featuregroup Number:Rescale of 33 : 1 : 6 : 1

	f1-score	precision	recall	support
AFIB	0.782376	0.773248	0.793762	875.0
GSVT	0.805018	0.795260	0.815666	691.0
SB	0.817152	0.785609	0.863418	1553.0
SR	0.648861	0.683088	0.617939	907.0
macro avg	0.763352	0.759301	0.772697	4026.0
micro avg	0.784781	0.784781	0.784781	4026.0
weighted avg	0.769598	0.761482	0.784781	4026.0

The result of Model Number:Dataset Number:Featuregroup Number:Rescale of 33 : 1 : 7 : 0

	f1-score	precision	recall	support
AFIB	0.788567	0.795144	0.782369	204.0
GSVT	0.809398	0.805671	0.813177	300.0
SB	0.821605	0.818686	0.824549	892.0
SR	0.814016	0.817662	0.810445	681.0
macro avg	0.808396	0.809291	0.807635	2077.0
micro avg	0.814139	0.814139	0.814139	2077.0
weighted avg	0.814109	0.814158	0.814139	2077.0

The result of Model Number:Dataset Number:Featuregroup Number:Rescale of 33 : 1 : 7 : 1

	f1-score	precision	recall	support
AFIB	0.756845	0.793081	0.723955	204.0
GSVT	0.797162	0.788628	0.805912	300.0
SB	0.814095	0.809597	0.818653	892.0
SR	0.804898	0.804200	0.805611	681.0
macro avg	0.793250	0.798877	0.788533	2077.0
micro avg	0.803235	0.803235	0.803235	2077.0
weighted avg	0.803011	0.803177	0.803235	2077.0

The result of Model Number:Dataset Number:Featuregroup Number:Rescale of 33 : 1 : 8 : 0

	f1-score	precision	recall	support
AFIB	0.641128	0.635458	0.646927	875.0
GSVT	0.582897	0.564491	0.648484	691.0
SB	0.525513	0.504565	0.558272	1553.0
SR	0.541617	0.547222	0.536162	907.0
macro avg	0.572789	0.562934	0.597461	4026.0
micro avg	0.588042	0.588042	0.588042	4026.0
weighted avg	0.564118	0.552908	0.588042	4026.0

The result of Model Number:Dataset Number:Featuregroup Number:Rescale of 33 : 1 : 8 : 1

	f1-score	precision	recall	support
AFIB	0.744839	0.748752	0.741012	875.0
GSVT	0.757409	0.758815	0.756104	691.0
SB	0.802200	0.796503	0.807983	1553.0
SR	0.786819	0.791667	0.782049	907.0
macro avg	0.772817	0.773934	0.771787	4026.0
micro avg	0.778681	0.778681	0.778681	4026.0
weighted avg	0.778581	0.778567	0.778681	4026.0

The result of Model Number:Dataset Number:Featuregroup Number:Rescale of 33 : 1 : 9 : 0

	f1-score	precision	recall	support
AFIB	0.707588	0.704710	0.710506	875.0
GSVT	0.717930	0.722270	0.713670	691.0
SB	0.757193	0.756271	0.758119	1553.0
SR	0.741396	0.742571	0.740247	907.0
macro avg	0.731027	0.731455	0.730636	4026.0
micro avg	0.736116	0.736116	0.736116	4026.0
weighted avg	0.736114	0.736143	0.736116	4026.0

The result of Model Number:Dataset Number:Featuregroup Number:Rescale of 33 : 1 : 9 : 1

	f1-score	precision	recall	support
AFIB	0.594718	0.580730	0.626108	875.0
GSVT	0.571470	0.566201	0.584492	691.0
SB	0.619798	0.576157	0.671306	1553.0
SR	0.386452	0.484673	0.321829	907.0
macro avg	0.543109	0.551940	0.550934	4026.0
micro avg	0.567850	0.567850	0.567850	4026.0
weighted avg	0.553483	0.554832	0.567850	4026.0

The result of Model Number:Dataset Number:Featuregroup Number:Rescale of 33 : 1 : 10 : 0

	f1-score	precision	recall	support
AFIB	0.815575	0.861970	0.774459	204.0
GSVT	0.861878	0.852387	0.871627	300.0
SB	0.874699	0.865996	0.883609	892.0

SR 0.864538 0.867493 0.861739 681.0
macro avg 0.854172 0.861962 0.847858 2077.0
micro avg 0.863987 0.863987 0.863987 2077.0
weighted avg 0.863708 0.864126 0.863987 2077.0
The result of Model Number:Dataset Number:Featuregroup Number:Rescale of 33 : 1 : 10 : 1

	f1-score	precision	recall	support
AFIB	0.638443	0.630773	0.646326	875.0
GSVT	0.650723	0.655991	0.645542	691.0
SB	0.693620	0.690965	0.696298	1553.0
SR	0.677357	0.686018	0.668920	907.0
macro avg	0.665036	0.665937	0.664272	4026.0
micro avg	0.670558	0.670558	0.670558	4026.0
weighted avg	0.670601	0.670766	0.670558	4026.0

The result of Model Number:Dataset Number:Featuregroup Number:Rescale of 34 : 0 : 0 : 0

	f1-score	precision	recall	support
AFIB	0.674685	0.668432	0.681094	875.0
GSVT	0.686801	0.690531	0.683123	691.0
SB	0.727705	0.726027	0.729391	1553.0
SR	0.710750	0.717309	0.704333	907.0
macro avg	0.699985	0.700575	0.699485	4026.0
micro avg	0.705308	0.705308	0.705308	4026.0
weighted avg	0.705342	0.705453	0.705308	4026.0

The result of Model Number:Dataset Number:Featuregroup Number:Rescale of 34 : 0 : 0 : 1

	f1-score	precision	recall	support
AFIB	0.759811	0.752859	0.766925	875.0
GSVT	0.769546	0.776336	0.762989	691.0
SB	0.818003	0.813690	0.822373	1553.0
SR	0.799511	0.809146	0.790176	907.0
macro avg	0.786718	0.788008	0.785616	4026.0
micro avg	0.792876	0.792876	0.792876	4026.0
weighted avg	0.792873	0.793034	0.792876	4026.0

The result of Model Number:Dataset Number:Featuregroup Number:Rescale of 34 : 0 : 1 : 0

	f1-score	precision	recall	support
AFIB	0.777913	0.795217	0.761512	204.0
GSVT	0.811056	0.806016	0.816191	300.0
SB	0.828662	0.826917	0.830425	892.0
SR	0.819836	0.819256	0.820442	681.0
macro avg	0.809367	0.811851	0.807143	2077.0
micro avg	0.818327	0.818327	0.818327	2077.0
weighted avg	0.818241	0.818273	0.818327	2077.0

The result of Model Number:Dataset Number:Featuregroup Number:Rescale of 34 : 0 : 1 : 1

	f1-score	precision	recall	support
AFIB	0.677282	0.702286	0.654059	204.0
GSVT	0.691758	0.684658	0.699114	300.0
SB	0.710704	0.707390	0.714051	892.0
SR	0.699623	0.700061	0.699208	681.0
macro avg	0.694842	0.698599	0.691608	2077.0
micro avg	0.701135	0.701135	0.701135	2077.0
weighted avg	0.701052	0.701203	0.701135	2077.0

The result of Model Number:Dataset Number:Featuregroup Number:Rescale of 34 : 0 : 2 : 0

	f1-score	precision	recall	support
AFIB	0.631592	0.587333	0.741685	875.0
GSVT	0.541671	0.505726	0.612685	691.0
SB	0.647262	0.641801	0.652832	1553.0
SR	0.623867	0.638989	0.609662	907.0
macro avg	0.611098	0.593462	0.654216	4026.0
micro avg	0.655527	0.655527	0.655527	4026.0
weighted avg	0.620463	0.605974	0.655527	4026.0

The result of Model Number:Dataset Number:Featuregroup Number:Rescale of 34 : 0 : 2 : 1

	f1-score	precision	recall	support
AFIB	0.732348	0.720862	0.744285	875.0
GSVT	0.705665	0.725895	0.686614	691.0
SB	0.741537	0.703948	0.783409	1553.0
SR	0.602495	0.670454	0.547138	907.0
macro avg	0.695511	0.705290	0.690362	4026.0
micro avg	0.705064	0.705064	0.705064	4026.0
weighted avg	0.702059	0.703845	0.705064	4026.0

The result of Model Number:Dataset Number:Featuregroup Number:Rescale of 34 : 0 : 3 : 0

	f1-score	precision	recall	support
AFIB	0.877931	0.915954	0.843188	204.0
GSVT	0.820713	0.828372	0.813606	300.0
SB	0.850729	0.808903	0.897176	892.0
SR	0.758230	0.806080	0.715987	681.0
macro avg	0.826901	0.839827	0.817489	2077.0
micro avg	0.820395	0.820395	0.820395	2077.0
weighted avg	0.818737	0.821304	0.820395	2077.0

The result of Model Number:Dataset Number:Featuregroup Number:Rescale of 34 : 0 : 3 : 1

	f1-score	precision	recall	support
AFIB	0.792636	0.833585	0.755756	204.0

GSVT 0.831146 0.823022 0.839450 300.0
SB 0.845801 0.838997 0.852721 892.0
SR 0.835354 0.836370 0.834373 681.0
macro avg 0.826234 0.832994 0.820575 2077.0
micro avg 0.835265 0.835265 0.835265 2077.0
weighted avg 0.835037 0.835297 0.835265 2077.0
The result of Model Number:Dataset Number:Featuregroup Number:Rescale of 34 : 0 : 4 : 0

	f1-score	precision	recall	support
AFIB	0.811831	0.803665	0.820187	875.0
GSVT	0.819096	0.826389	0.811991	691.0
SB	0.866568	0.863908	0.869245	1553.0
SR	0.849787	0.857225	0.842479	907.0
macro avg	0.836820	0.837797	0.835976	4026.0
micro avg	0.842726	0.842726	0.842726	4026.0
weighted avg	0.842743	0.842870	0.842726	4026.0

The result of Model Number:Dataset Number:Featuregroup Number:Rescale of 34 : 0 : 4 : 1

	f1-score	precision	recall	support
AFIB	0.756114	0.750057	0.762357	875.0
GSVT	0.776995	0.778321	0.775765	691.0
SB	0.823738	0.821530	0.825965	1553.0
SR	0.801231	0.810558	0.792133	907.0
macro avg	0.789519	0.790117	0.789055	4026.0
micro avg	0.795903	0.795903	0.795903	4026.0
weighted avg	0.795947	0.796108	0.795903	4026.0

The result of Model Number:Dataset Number:Featuregroup Number:Rescale of 34 : 0 : 5 : 0

	f1-score	precision	recall	support
AFIB	0.811036	0.813827	0.808367	875.0
GSVT	0.826434	0.809945	0.843756	691.0
SB	0.874846	0.870115	0.879634	1553.0
SR	0.841009	0.860563	0.822373	907.0
macro avg	0.838331	0.838613	0.838533	4026.0
micro avg	0.845087	0.845087	0.845087	4026.0
weighted avg	0.845046	0.845402	0.845087	4026.0

The result of Model Number:Dataset Number:Featuregroup Number:Rescale of 34 : 0 : 5 : 1

	f1-score	precision	recall	support
AFIB	0.810241	0.833043	0.788821	204.0
GSVT	0.831189	0.825354	0.837263	300.0
SB	0.854046	0.850629	0.857492	892.0
SR	0.842694	0.843221	0.842193	681.0
macro avg	0.834543	0.838062	0.831442	2077.0

micro avg 0.842809 0.842809 0.842809 2077.0
weighted avg 0.842720 0.842822 0.842809 2077.0
The result of Model Number:Dataset Number:Featuregroup Number:Rescale of 34 : 0 : 6 : 0

	f1-score	precision	recall	support
AFIB	0.844415	0.875721	0.815389	204.0
GSVT	0.869712	0.858837	0.880976	300.0
SB	0.891766	0.883629	0.900062	892.0
SR	0.868321	0.875060	0.861694	681.0
macro avg	0.868553	0.873312	0.864530	2077.0
micro avg	0.876409	0.876409	0.876409	2077.0
weighted avg	0.876243	0.876462	0.876409	2077.0

The result of Model Number:Dataset Number:Featuregroup Number:Rescale of 34 : 0 : 6 : 1

	f1-score	precision	recall	support
AFIB	0.787037	0.802894	0.772299	204.0
GSVT	0.812291	0.805767	0.819078	300.0
SB	0.829876	0.829040	0.830713	892.0
SR	0.822311	0.821839	0.822793	681.0
macro avg	0.812879	0.814885	0.811221	2077.0
micro avg	0.820699	0.820699	0.820699	2077.0
weighted avg	0.820648	0.820750	0.820699	2077.0

The result of Model Number:Dataset Number:Featuregroup Number:Rescale of 34 : 0 : 7 : 0

	f1-score	precision	recall	support
AFIB	0.811104	0.810361	0.811945	875.0
GSVT	0.827729	0.826123	0.829428	691.0
SB	0.878328	0.876372	0.880307	1553.0
SR	0.854355	0.859929	0.848884	907.0
macro avg	0.842879	0.843196	0.842641	4026.0
micro avg	0.849637	0.849637	0.849637	4026.0
weighted avg	0.849632	0.849696	0.849637	4026.0

The result of Model Number:Dataset Number:Featuregroup Number:Rescale of 34 : 0 : 7 : 1

	f1-score	precision	recall	support
AFIB	0.777461	0.820074	0.739574	204.0
GSVT	0.740308	0.766264	0.716145	300.0
SB	0.761480	0.732110	0.793317	892.0
SR	0.677737	0.696124	0.660335	681.0
macro avg	0.739247	0.753643	0.727343	2077.0
micro avg	0.733290	0.733290	0.733290	2077.0
weighted avg	0.732534	0.733884	0.733290	2077.0

The result of Model Number:Dataset Number:Featuregroup Number:Rescale of 34 : 0 : 8 : 0

	f1-score	precision	recall	support
AFIB	0.859041	0.876195	0.842676	204.0
GSVT	0.868688	0.857834	0.879952	300.0
SB	0.885273	0.876808	0.893904	892.0
SR	0.869196	0.880598	0.858109	681.0
macro avg	0.870549	0.872859	0.868660	2077.0
micro avg	0.875121	0.875121	0.875121	2077.0
weighted avg	0.875030	0.875250	0.875121	2077.0

The result of Model Number:Dataset Number:Featuregroup Number:Rescale of 34 : 0 : 8 : 1

	f1-score	precision	recall	support
AFIB	0.631732	0.641990	0.621830	875.0
GSVT	0.671865	0.673971	0.669813	691.0
SB	0.730955	0.728437	0.733496	1553.0
SR	0.692002	0.684294	0.699927	907.0
macro avg	0.681638	0.682173	0.681266	4026.0
micro avg	0.690734	0.690734	0.690734	4026.0
weighted avg	0.690473	0.690356	0.690734	4026.0

The result of Model Number:Dataset Number:Featuregroup Number:Rescale of 34 : 0 : 9 : 0

	f1-score	precision	recall	support
AFIB	0.680783	0.698022	0.664439	204.0
GSVT	0.695568	0.688004	0.703416	300.0
SB	0.715160	0.711591	0.718767	892.0
SR	0.704075	0.707234	0.700972	681.0
macro avg	0.698897	0.701213	0.696899	2077.0
micro avg	0.705379	0.705379	0.705379	2077.0
weighted avg	0.705319	0.705423	0.705379	2077.0

The result of Model Number:Dataset Number:Featuregroup Number:Rescale of 34 : 0 : 9 : 1

	f1-score	precision	recall	support
AFIB	0.650514	0.649133	0.651930	875.0
GSVT	0.660677	0.657510	0.663930	691.0
SB	0.695360	0.687059	0.703866	1553.0
SR	0.663045	0.681830	0.645281	907.0
macro avg	0.667399	0.668883	0.666252	4026.0
micro avg	0.672525	0.672525	0.672525	4026.0
weighted avg	0.672380	0.672566	0.672525	4026.0

The result of Model Number:Dataset Number:Featuregroup Number:Rescale of 34 : 0 : 10 : 0

	f1-score	precision	recall	support
AFIB	0.790448	0.797625	0.783806	204.0
GSVT	0.816573	0.811638	0.821782	300.0
SB	0.833657	0.832540	0.834778	892.0

SR	0.825943	0.827656	0.824243	681.0
macro avg	0.816655	0.817365	0.816152	2077.0
micro avg	0.824440	0.824440	0.824440	2077.0
weighted avg	0.824416	0.824490	0.824440	2077.0

The result of Model Number:Dataset Number:Featuregroup Number:Rescale of 34 : 0 : 10 : 1

	f1-score	precision	recall	support
AFIB	0.755843	0.758671	0.753358	875.0
GSVT	0.772857	0.780313	0.765697	691.0
SB	0.821789	0.813169	0.830656	1553.0
SR	0.793331	0.800313	0.786743	907.0
macro avg	0.785955	0.788117	0.784114	4026.0
micro avg	0.792814	0.792814	0.792814	4026.0
weighted avg	0.792647	0.792789	0.792814	4026.0

The result of Model Number:Dataset Number:Featuregroup Number:Rescale of 34 : 1 : 0 : 0

	f1-score	precision	recall	support
AFIB	0.672668	0.652562	0.717239	875.0
GSVT	0.576040	0.563426	0.589446	691.0
SB	0.764049	0.757824	0.770580	1553.0
SR	0.716788	0.746882	0.690370	907.0
macro avg	0.682386	0.680173	0.691909	4026.0
micro avg	0.709828	0.709828	0.709828	4026.0
weighted avg	0.701272	0.699116	0.709828	4026.0

The result of Model Number:Dataset Number:Featuregroup Number:Rescale of 34 : 1 : 0 : 1

	f1-score	precision	recall	support
AFIB	0.911085	0.923150	0.899468	204.0
GSVT	0.938579	0.935192	0.942012	300.0
SB	0.950269	0.947312	0.953250	892.0
SR	0.939915	0.941725	0.938138	681.0
macro avg	0.934962	0.936845	0.933217	2077.0
micro avg	0.941389	0.941389	0.941389	2077.0
weighted avg	0.941337	0.941357	0.941389	2077.0

The result of Model Number:Dataset Number:Featuregroup Number:Rescale of 34 : 1 : 1 : 0

	f1-score	precision	recall	support
AFIB	0.686509	0.703631	0.670254	875.0
GSVT	0.730326	0.733126	0.727581	691.0
SB	0.790882	0.787052	0.794751	1553.0
SR	0.749404	0.736913	0.762374	907.0
macro avg	0.739280	0.740180	0.738740	4026.0
micro avg	0.748870	0.748870	0.748870	4026.0
weighted avg	0.748460	0.748370	0.748870	4026.0

The result of Model Number:Dataset Number:Featuregroup Number:Rescale of 34 : 1 : 1 : 1

	f1-score	precision	recall	support
AFIB	0.732206	0.721181	0.749670	875.0
GSVT	0.699125	0.697090	0.705088	691.0
SB	0.746575	0.695689	0.811701	1553.0
SR	0.553035	0.739243	0.458256	907.0
macro avg	0.682735	0.713301	0.681179	4026.0
micro avg	0.700295	0.700295	0.700295	4026.0
weighted avg	0.691706	0.711282	0.700295	4026.0

The result of Model Number:Dataset Number:Featuregroup Number:Rescale of 34 : 1 : 2 : 0

	f1-score	precision	recall	support
AFIB	0.711661	0.702726	0.721033	875.0
GSVT	0.660281	0.680272	0.643639	691.0
SB	0.710897	0.658448	0.774305	1553.0
SR	0.552745	0.676518	0.471338	907.0
macro avg	0.658896	0.679491	0.652579	4026.0
micro avg	0.672046	0.672046	0.672046	4026.0
weighted avg	0.666746	0.675888	0.672046	4026.0

The result of Model Number:Dataset Number:Featuregroup Number:Rescale of 34 : 1 : 2 : 1

	f1-score	precision	recall	support
AFIB	0.692322	0.677528	0.707817	875.0
GSVT	0.716944	0.727907	0.706370	691.0
SB	0.764260	0.764361	0.764163	1553.0
SR	0.744571	0.752282	0.737020	907.0
macro avg	0.729524	0.730520	0.728842	4026.0
micro avg	0.735883	0.735883	0.735883	4026.0
weighted avg	0.736069	0.736511	0.735883	4026.0

The result of Model Number:Dataset Number:Featuregroup Number:Rescale of 34 : 1 : 3 : 0

	f1-score	precision	recall	support
AFIB	0.880385	0.901459	0.861562	204.0
GSVT	0.906310	0.893540	0.919829	300.0
SB	0.929858	0.920411	0.939544	892.0
SR	0.907634	0.920739	0.894968	681.0
macro avg	0.906047	0.909037	0.903976	2077.0
micro avg	0.914422	0.914422	0.914422	2077.0
weighted avg	0.914311	0.914776	0.914422	2077.0

The result of Model Number:Dataset Number:Featuregroup Number:Rescale of 34 : 1 : 3 : 1

	f1-score	precision	recall	support
AFIB	0.800835	0.805214	0.796529	875.0

GSVT	0.797018	0.790298	0.803934	691.0
SB	0.838264	0.830325	0.846359	1553.0
SR	0.807066	0.822121	0.792564	907.0
macro avg	0.810796	0.811990	0.809847	4026.0
micro avg	0.816128	0.816128	0.816128	4026.0
weighted avg	0.816022	0.816149	0.816128	4026.0

The result of Model Number:Dataset Number:Featuregroup Number:Rescale of 34 : 1 : 4 : 0

	f1-score	precision	recall	support
AFIB	0.758298	0.776748	0.740891	204.0
GSVT	0.767830	0.757260	0.778802	300.0
SB	0.783335	0.775512	0.791322	892.0
SR	0.769658	0.779758	0.759838	681.0
macro avg	0.769780	0.772320	0.767713	2077.0
micro avg	0.774238	0.774238	0.774238	2077.0
weighted avg	0.774152	0.774389	0.774238	2077.0

The result of Model Number:Dataset Number:Featuregroup Number:Rescale of 34 : 1 : 4 : 1

	f1-score	precision	recall	support
AFIB	0.572500	0.623649	0.604572	204.0
GSVT	0.777613	0.764902	0.791023	300.0
SB	0.661989	0.656947	0.667159	892.0
SR	0.809505	0.800346	0.818900	681.0
macro avg	0.705402	0.711461	0.720414	2077.0
micro avg	0.728655	0.728655	0.728655	2077.0
weighted avg	0.718267	0.716286	0.728655	2077.0

The result of Model Number:Dataset Number:Featuregroup Number:Rescale of 34 : 1 : 5 : 0

	f1-score	precision	recall	support
AFIB	0.598305	0.661568	0.546395	204.0
GSVT	0.688427	0.680460	0.696715	300.0
SB	0.727860	0.724867	0.730879	892.0
SR	0.712229	0.701679	0.723108	681.0
macro avg	0.681705	0.692143	0.674274	2077.0
micro avg	0.705277	0.705277	0.705277	2077.0
weighted avg	0.704314	0.704633	0.705277	2077.0

The result of Model Number:Dataset Number:Featuregroup Number:Rescale of 34 : 1 : 5 : 1

	f1-score	precision	recall	support
AFIB	0.766921	0.770573	0.763337	875.0
GSVT	0.776395	0.762285	0.791116	691.0
SB	0.821484	0.818339	0.824657	1553.0
SR	0.797203	0.810849	0.784022	907.0
macro avg	0.790501	0.790511	0.790783	4026.0

micro avg	0.796419	0.796419	0.796419	4026.0
weighted avg	0.796417	0.796649	0.796419	4026.0

The result of Model Number:Dataset Number:Featuregroup Number:Rescale of 34 : 1 : 6 : 0

	f1-score	precision	recall	support
AFIB	0.699868	0.703200	0.696598	875.0
GSVT	0.708513	0.695638	0.721948	691.0
SB	0.749661	0.746790	0.752556	1553.0
SR	0.727503	0.739955	0.715474	907.0
macro avg	0.721386	0.721396	0.721644	4026.0
micro avg	0.726787	0.726787	0.726787	4026.0
weighted avg	0.726785	0.726997	0.726787	4026.0

The result of Model Number:Dataset Number:Featuregroup Number:Rescale of 34 : 1 : 6 : 1

	f1-score	precision	recall	support
AFIB	0.696693	0.691867	0.701728	875.0
GSVT	0.707441	0.710312	0.704708	691.0
SB	0.767575	0.759594	0.775730	1553.0
SR	0.728820	0.745778	0.712682	907.0
macro avg	0.725132	0.726888	0.723712	4026.0
micro avg	0.733253	0.733253	0.733253	4026.0
weighted avg	0.733118	0.733303	0.733253	4026.0

The result of Model Number:Dataset Number:Featuregroup Number:Rescale of 34 : 1 : 7 : 0

	f1-score	precision	recall	support
AFIB	0.861195	0.861605	0.860909	875.0
GSVT	0.883498	0.875851	0.891311	691.0
SB	0.943579	0.936703	0.950566	1553.0
SR	0.903268	0.921225	0.886046	907.0
macro avg	0.897885	0.898846	0.897208	4026.0
micro avg	0.906374	0.906374	0.906374	4026.0
weighted avg	0.906281	0.906450	0.906374	4026.0

The result of Model Number:Dataset Number:Featuregroup Number:Rescale of 34 : 1 : 7 : 1

	f1-score	precision	recall	support
AFIB	0.683543	0.753803	0.625684	204.0
GSVT	0.813396	0.802404	0.824775	300.0
SB	0.866209	0.862745	0.869705	892.0
SR	0.842699	0.831308	0.854443	681.0
macro avg	0.801462	0.812565	0.793652	2077.0
micro avg	0.834244	0.834244	0.834244	2077.0
weighted avg	0.832931	0.833022	0.834244	2077.0

The result of Model Number:Dataset Number:Featuregroup Number:Rescale of 34 : 1 : 8 : 0

f1-score precision recall support
AFIB 0.871056 0.895569 0.848028 204.0
GSVT 0.893575 0.887303 0.900105 300.0
SB 0.918149 0.914475 0.921853 892.0
SR 0.905945 0.906511 0.905406 681.0
macro avg 0.897181 0.900965 0.893848 2077.0
micro avg 0.906068 0.906068 0.906068 2077.0
weighted avg 0.905972 0.906082 0.906068 2077.0
The result of Model Number:Dataset Number:Featuregroup Number:Rescale of 34 : 1 : 8 : 1
f1-score precision recall support
AFIB 0.720858 0.722864 0.718902 875.0
GSVT 0.737371 0.737166 0.737583 691.0
SB 0.785323 0.777942 0.792855 1553.0
SR 0.764514 0.775539 0.753807 907.0
macro avg 0.752017 0.753378 0.750787 4026.0
micro avg 0.758499 0.758499 0.758499 4026.0
weighted avg 0.758394 0.758432 0.758499 4026.0
The result of Model Number:Dataset Number:Featuregroup Number:Rescale of 34 : 1 : 9 : 0
f1-score precision recall support
AFIB 0.659434 0.727215 0.603615 204.0
GSVT 0.784707 0.774102 0.795684 300.0
SB 0.835657 0.832315 0.839029 892.0
SR 0.812976 0.801986 0.824306 681.0
macro avg 0.773193 0.783905 0.765659 2077.0
micro avg 0.804819 0.804819 0.804819 2077.0
weighted avg 0.803553 0.803640 0.804819 2077.0
The result of Model Number:Dataset Number:Featuregroup Number:Rescale of 34 : 1 : 9 : 1
f1-score precision recall support
AFIB 0.857484 0.865867 0.849463 875.0
GSVT 0.850934 0.844478 0.857770 691.0
SB 0.900385 0.891121 0.909923 1553.0
SR 0.871806 0.885826 0.858468 907.0
macro avg 0.870152 0.871823 0.868906 4026.0
micro avg 0.876240 0.876240 0.876240 4026.0
weighted avg 0.876135 0.876434 0.876240 4026.0
The result of Model Number:Dataset Number:Featuregroup Number:Rescale of 34 : 1 : 10 : 0
f1-score precision recall support
AFIB 0.796543 0.814555 0.779334 875.0
GSVT 0.844517 0.848111 0.841003 691.0
SB 0.916970 0.912648 0.921338 1553.0

SR 0.868816 0.855490 0.882601 907.0
macro avg 0.856711 0.857701 0.856069 4026.0
micro avg 0.867960 0.867960 0.867960 4026.0
weighted avg 0.867513 0.867375 0.867960 4026.0
The result of Model Number:Dataset Number:Featuregroup Number:Rescale of 34 : 1 : 10 : 1

	f1-score	precision	recall	support
AFIB	0.726179	0.732675	0.719887	875.0
GSVT	0.740048	0.725976	0.754818	691.0
SB	0.785499	0.778694	0.792427	1553.0
SR	0.753237	0.770556	0.736713	907.0
macro avg	0.751241	0.751975	0.750961	4026.0
micro avg	0.757655	0.757655	0.757655	4026.0
weighted avg	0.757537	0.757811	0.757655	4026.0

The result of Model Number:Dataset Number:Featuregroup Number:Rescale of 35 : 0 : 0 : 0

	f1-score	precision	recall	support
AFIB	0.708946	0.697596	0.797708	204.0
GSVT	0.829989	0.820358	0.840151	300.0
SB	0.857780	0.854541	0.861049	892.0
SR	0.673030	0.676510	0.669700	681.0
macro avg	0.767436	0.762251	0.792152	2077.0
micro avg	0.789071	0.789071	0.789071	2077.0
weighted avg	0.778572	0.775816	0.789071	2077.0

The result of Model Number:Dataset Number:Featuregroup Number:Rescale of 35 : 0 : 0 : 1

	f1-score	precision	recall	support
AFIB	0.836070	0.855170	0.818016	204.0
GSVT	0.874051	0.868610	0.879583	300.0
SB	0.892928	0.890159	0.895728	892.0
SR	0.882702	0.883111	0.882318	681.0
macro avg	0.871438	0.874262	0.868911	2077.0
micro avg	0.881366	0.881366	0.881366	2077.0
weighted avg	0.881264	0.881299	0.881366	2077.0

The result of Model Number:Dataset Number:Featuregroup Number:Rescale of 35 : 0 : 1 : 0

	f1-score	precision	recall	support
AFIB	0.736554	0.728330	0.744973	875.0
GSVT	0.751208	0.758089	0.744463	691.0
SB	0.801198	0.798589	0.803827	1553.0
SR	0.780428	0.788246	0.772766	907.0
macro avg	0.767347	0.768313	0.766507	4026.0
micro avg	0.773850	0.773850	0.773850	4026.0
weighted avg	0.773889	0.774038	0.773850	4026.0

The result of Model Number:Dataset Number:Featuregroup Number:Rescale of 35 : 0 : 1 : 1

	f1-score	precision	recall	support
AFIB	0.801405	0.801786	0.801139	875.0
GSVT	0.822159	0.815043	0.829430	691.0
SB	0.878069	0.871671	0.884571	1553.0
SR	0.840557	0.857267	0.824530	907.0
macro avg	0.835548	0.836442	0.834917	4026.0
micro avg	0.843447	0.843447	0.843447	4026.0
weighted avg	0.843360	0.843518	0.843447	4026.0

The result of Model Number:Dataset Number:Featuregroup Number:Rescale of 35 : 0 : 2 : 0

	f1-score	precision	recall	support
AFIB	0.667882	0.642349	0.709894	875.0
GSVT	0.692039	0.668155	0.724435	691.0
SB	0.771060	0.757450	0.786015	1553.0
SR	0.653070	0.785279	0.563838	907.0
macro avg	0.696013	0.713308	0.696046	4026.0
micro avg	0.708849	0.708849	0.708849	4026.0
weighted avg	0.708492	0.723378	0.708849	4026.0

The result of Model Number:Dataset Number:Featuregroup Number:Rescale of 35 : 0 : 2 : 1

	f1-score	precision	recall	support
AFIB	0.762457	0.759988	0.764965	875.0
GSVT	0.771298	0.773695	0.768993	691.0
SB	0.813845	0.812073	0.815627	1553.0
SR	0.798837	0.802605	0.795133	907.0
macro avg	0.786609	0.787090	0.786179	4026.0
micro avg	0.791995	0.791995	0.791995	4026.0
weighted avg	0.791993	0.792033	0.791995	4026.0

The result of Model Number:Dataset Number:Featuregroup Number:Rescale of 35 : 0 : 3 : 0

	f1-score	precision	recall	support
AFIB	0.728642	0.719340	0.738231	875.0
GSVT	0.741432	0.745684	0.737248	691.0
SB	0.793657	0.790324	0.797026	1553.0
SR	0.770288	0.782756	0.758225	907.0
macro avg	0.758505	0.759526	0.757682	4026.0
micro avg	0.765246	0.765246	0.765246	4026.0
weighted avg	0.765298	0.765529	0.765246	4026.0

The result of Model Number:Dataset Number:Featuregroup Number:Rescale of 35 : 0 : 3 : 1

	f1-score	precision	recall	support
AFIB	0.807278	0.800856	0.813880	875.0

GSVT 0.775269 0.760529 0.790657 691.0
SB 0.813673 0.751091 0.887646 1553.0
SR 0.623047 0.796658 0.511681 907.0
macro avg 0.754817 0.777283 0.750966 4026.0
micro avg 0.770268 0.770268 0.770268 4026.0
weighted avg 0.762747 0.773792 0.770268 4026.0
The result of Model Number:Dataset Number:Featuregroup Number:Rescale of 35 : 0 : 4 : 0

	f1-score	precision	recall	support
AFIB	0.834236	0.878160	0.794720	204.0
GSVT	0.876127	0.868453	0.883977	300.0
SB	0.898861	0.893312	0.904496	892.0
SR	0.886598	0.884799	0.888424	681.0
macro avg	0.873955	0.881181	0.867904	2077.0
micro avg	0.885480	0.885480	0.885480	2077.0
weighted avg	0.885209	0.885442	0.885480	2077.0

The result of Model Number:Dataset Number:Featuregroup Number:Rescale of 35 : 0 : 4 : 1

	f1-score	precision	recall	support
AFIB	0.785531	0.778167	0.793136	875.0
GSVT	0.744759	0.726858	0.799507	691.0
SB	0.861175	0.858012	0.864384	1553.0
SR	0.669806	0.674503	0.665183	907.0
macro avg	0.765318	0.759385	0.780553	4026.0
micro avg	0.792887	0.792887	0.792887	4026.0
weighted avg	0.781641	0.776806	0.792887	4026.0

The result of Model Number:Dataset Number:Featuregroup Number:Rescale of 35 : 0 : 5 : 0

	f1-score	precision	recall	support
AFIB	0.688820	0.723236	0.657877	204.0
GSVT	0.709587	0.696436	0.723490	300.0
SB	0.725825	0.718509	0.733307	892.0
SR	0.705695	0.711999	0.699567	681.0
macro avg	0.707482	0.712545	0.703560	2077.0
micro avg	0.713418	0.713418	0.713418	2077.0
weighted avg	0.713245	0.713651	0.713418	2077.0

The result of Model Number:Dataset Number:Featuregroup Number:Rescale of 35 : 0 : 5 : 1

	f1-score	precision	recall	support
AFIB	0.720425	0.718210	0.722700	875.0
GSVT	0.702060	0.705503	0.698763	691.0
SB	0.738063	0.707434	0.771470	1553.0
SR	0.635164	0.693000	0.586288	907.0
macro avg	0.698928	0.706037	0.694805	4026.0

micro avg 0.706673 0.706673 0.706673 4026.0
weighted avg 0.704869 0.706193 0.706673 4026.0
The result of Model Number:Dataset Number:Featuregroup Number:Rescale of 35 : 0 : 6 : 0

	f1-score	precision	recall	support
AFIB	0.811746	0.825242	0.798871	204.0
GSVT	0.827312	0.821434	0.833336	300.0
SB	0.846501	0.841743	0.851331	892.0
SR	0.835716	0.840829	0.830692	681.0
macro avg	0.830319	0.832312	0.828557	2077.0
micro avg	0.836812	0.836812	0.836812	2077.0
weighted avg	0.836780	0.836889	0.836812	2077.0

The result of Model Number:Dataset Number:Featuregroup Number:Rescale of 35 : 0 : 6 : 1

	f1-score	precision	recall	support
AFIB	0.906920	0.947227	0.870029	204.0
GSVT	0.944713	0.937297	0.952269	300.0
SB	0.959144	0.951745	0.966668	892.0
SR	0.948533	0.950099	0.947005	681.0
macro avg	0.939828	0.946592	0.933993	2077.0
micro avg	0.948649	0.948649	0.948649	2077.0
weighted avg	0.948451	0.948675	0.948649	2077.0

The result of Model Number:Dataset Number:Featuregroup Number:Rescale of 35 : 0 : 7 : 0

	f1-score	precision	recall	support
AFIB	0.725906	0.805608	0.660730	204.0
GSVT	0.847594	0.836998	0.858629	300.0
SB	0.901798	0.897197	0.906448	892.0
SR	0.880673	0.868360	0.893343	681.0
macro avg	0.838993	0.852041	0.829788	2077.0
micro avg	0.871110	0.871110	0.871110	2077.0
weighted avg	0.869767	0.870051	0.871110	2077.0

The result of Model Number:Dataset Number:Featuregroup Number:Rescale of 35 : 0 : 7 : 1

	f1-score	precision	recall	support
AFIB	0.687132	0.766366	0.622837	204.0
GSVT	0.810199	0.796097	0.824982	300.0
SB	0.865042	0.861208	0.868920	892.0
SR	0.844968	0.833414	0.856871	681.0
macro avg	0.801835	0.814271	0.793403	2077.0
micro avg	0.834453	0.834453	0.834453	2077.0
weighted avg	0.833065	0.833375	0.834453	2077.0

The result of Model Number:Dataset Number:Featuregroup Number:Rescale of 35 : 0 : 8 : 0

	f1-score	precision	recall	support
AFIB	0.775294	0.763407	0.787687	875.0
GSVT	0.802028	0.809889	0.794476	691.0
SB	0.853572	0.851578	0.855602	1553.0
SR	0.832249	0.842801	0.821976	907.0
macro avg	0.815786	0.816919	0.814935	4026.0
micro avg	0.822775	0.822775	0.822775	4026.0
weighted avg	0.822909	0.823283	0.822775	4026.0

The result of Model Number:Dataset Number:Featuregroup Number:Rescale of 35 : 0 : 8 : 1

	f1-score	precision	recall	support
AFIB	0.653700	0.656940	0.650562	875.0
GSVT	0.660209	0.661160	0.659360	691.0
SB	0.694959	0.691734	0.698221	1553.0
SR	0.682166	0.683874	0.680503	907.0
macro avg	0.672758	0.673427	0.672162	4026.0
micro avg	0.677202	0.677202	0.677202	4026.0
weighted avg	0.677145	0.677154	0.677202	4026.0

The result of Model Number:Dataset Number:Featuregroup Number:Rescale of 35 : 0 : 9 : 0

	f1-score	precision	recall	support
AFIB	0.719757	0.746533	0.695158	204.0
GSVT	0.667908	0.698901	0.639608	300.0
SB	0.699660	0.657459	0.747661	892.0
SR	0.614436	0.657127	0.577026	681.0
macro avg	0.675440	0.690005	0.664863	2077.0
micro avg	0.670950	0.670950	0.670950	2077.0
weighted avg	0.669104	0.672085	0.670950	2077.0

The result of Model Number:Dataset Number:Featuregroup Number:Rescale of 35 : 0 : 9 : 1

	f1-score	precision	recall	support
AFIB	0.880825	0.913592	0.850721	204.0
GSVT	0.817372	0.855302	0.782739	300.0
SB	0.856230	0.804586	0.914973	892.0
SR	0.751935	0.804179	0.706153	681.0
macro avg	0.826590	0.844415	0.813647	2077.0
micro avg	0.821096	0.821096	0.821096	2077.0
weighted avg	0.818837	0.822484	0.821096	2077.0

The result of Model Number:Dataset Number:Featuregroup Number:Rescale of 35 : 0 : 10 : 0

	f1-score	precision	recall	support
AFIB	0.777735	0.754489	0.829268	875.0
GSVT	0.666015	0.651430	0.681514	691.0
SB	0.883389	0.876192	0.890941	1553.0

SR 0.828746 0.863541 0.798202 907.0
macro avg 0.788971 0.786413 0.799981 4026.0
micro avg 0.820699 0.820699 0.820699 4026.0
weighted avg 0.810807 0.808314 0.820699 4026.0
The result of Model Number:Dataset Number:Featuregroup Number:Rescale of 35 : 0 : 10 : 1

	f1-score	precision	recall	support
AFIB	0.613021	0.676032	0.561131	204.0
GSVT	0.729477	0.719619	0.739681	300.0
SB	0.776841	0.773734	0.779976	892.0
SR	0.755756	0.745540	0.766289	681.0
macro avg	0.718774	0.728731	0.711769	2077.0
micro avg	0.748174	0.748174	0.748174	2077.0
weighted avg	0.746996	0.747078	0.748174	2077.0

The result of Model Number:Dataset Number:Featuregroup Number:Rescale of 35 : 1 : 0 : 0

	f1-score	precision	recall	support
AFIB	0.812711	0.846909	0.781242	204.0
GSVT	0.839979	0.827122	0.853369	300.0
SB	0.860239	0.854069	0.866508	892.0
SR	0.837912	0.842173	0.833726	681.0
macro avg	0.837710	0.842568	0.833711	2077.0
micro avg	0.845487	0.845487	0.845487	2077.0
weighted avg	0.845324	0.845573	0.845487	2077.0

The result of Model Number:Dataset Number:Featuregroup Number:Rescale of 35 : 1 : 0 : 1

	f1-score	precision	recall	support
AFIB	0.879647	0.879882	0.879566	875.0
GSVT	0.894965	0.891661	0.898406	691.0
SB	0.957438	0.955114	0.959778	1553.0
SR	0.934604	0.941312	0.928017	907.0
macro avg	0.916664	0.916992	0.916442	4026.0
micro avg	0.924656	0.924656	0.924656	4026.0
weighted avg	0.924664	0.924763	0.924656	4026.0

The result of Model Number:Dataset Number:Featuregroup Number:Rescale of 35 : 1 : 1 : 0

	f1-score	precision	recall	support
AFIB	0.862636	0.876978	0.848954	204.0
GSVT	0.879177	0.872931	0.885579	300.0
SB	0.899570	0.894514	0.904703	892.0
SR	0.888109	0.893543	0.882770	681.0
macro avg	0.882373	0.884491	0.880501	2077.0
micro avg	0.889274	0.889274	0.889274	2077.0
weighted avg	0.889239	0.889356	0.889274	2077.0

The result of Model Number:Dataset Number:Featuregroup Number:Rescale of 35 : 1 : 1 : 1

	f1-score	precision	recall	support
AFIB	0.762951	0.741790	0.787757	875.0
GSVT	0.772911	0.805467	0.747640	691.0
SB	0.849070	0.848641	0.849510	1553.0
SR	0.827769	0.832949	0.822659	907.0
macro avg	0.803175	0.807212	0.801892	4026.0
micro avg	0.812555	0.812555	0.812555	4026.0
weighted avg	0.812483	0.814473	0.812555	4026.0

The result of Model Number:Dataset Number:Featuregroup Number:Rescale of 35 : 1 : 2 : 0

	f1-score	precision	recall	support
AFIB	0.678947	0.668799	0.689432	875.0
GSVT	0.691964	0.697882	0.686157	691.0
SB	0.739348	0.736802	0.741916	1553.0
SR	0.717707	0.728412	0.707337	907.0
macro avg	0.706992	0.707974	0.706211	4026.0
micro avg	0.713149	0.713149	0.713149	4026.0
weighted avg	0.713212	0.713452	0.713149	4026.0

The result of Model Number:Dataset Number:Featuregroup Number:Rescale of 35 : 1 : 2 : 1

	f1-score	precision	recall	support
AFIB	0.793531	0.795607	0.791622	204.0
GSVT	0.811435	0.808294	0.814669	300.0
SB	0.829633	0.828526	0.830748	892.0
SR	0.822989	0.825301	0.820693	681.0
macro avg	0.814397	0.814432	0.814433	2077.0
micro avg	0.821286	0.821286	0.821286	2077.0
weighted avg	0.821280	0.821313	0.821286	2077.0

The result of Model Number:Dataset Number:Featuregroup Number:Rescale of 35 : 1 : 3 : 0

	f1-score	precision	recall	support
AFIB	0.763787	0.779379	0.749144	204.0
GSVT	0.791521	0.784644	0.798664	300.0
SB	0.806739	0.805299	0.808186	892.0
SR	0.799230	0.799707	0.798759	681.0
macro avg	0.790319	0.792257	0.788689	2077.0
micro avg	0.797921	0.797921	0.797921	2077.0
weighted avg	0.797860	0.797936	0.797921	2077.0

The result of Model Number:Dataset Number:Featuregroup Number:Rescale of 35 : 1 : 3 : 1

	f1-score	precision	recall	support
AFIB	0.653083	0.645237	0.661147	875.0

GSVT 0.665644 0.671034 0.660345 691.0
SB 0.709525 0.706809 0.712265 1553.0
SR 0.692889 0.701749 0.684259 907.0
macro avg 0.680285 0.681207 0.679504 4026.0
micro avg 0.685934 0.685934 0.685934 4026.0
weighted avg 0.685979 0.686147 0.685934 4026.0
The result of Model Number:Dataset Number:Featuregroup Number:Rescale of 35 : 1 : 4 : 0

	f1-score	precision	recall	support
AFIB	0.687701	0.671527	0.723999	875.0
GSVT	0.660819	0.654726	0.675877	691.0
SB	0.716703	0.666238	0.776264	1553.0
SR	0.446873	0.560451	0.372147	907.0
macro avg	0.628024	0.638236	0.637072	4026.0
micro avg	0.656633	0.656633	0.656633	4026.0
weighted avg	0.640019	0.641580	0.656633	4026.0

The result of Model Number:Dataset Number:Featuregroup Number:Rescale of 35 : 1 : 4 : 1

	f1-score	precision	recall	support
AFIB	0.855162	0.845184	0.867607	875.0
GSVT	0.879910	0.869244	0.891549	691.0
SB	0.893172	0.858696	0.943743	1553.0
SR	0.709225	0.746636	0.675427	907.0
macro avg	0.834367	0.829940	0.844582	4026.0
micro avg	0.857790	0.857790	0.857790	4026.0
weighted avg	0.841194	0.832324	0.857790	4026.0

The result of Model Number:Dataset Number:Featuregroup Number:Rescale of 35 : 1 : 5 : 0

	f1-score	precision	recall	support
AFIB	0.886039	0.895680	0.876861	204.0
GSVT	0.916023	0.912203	0.920024	300.0
SB	0.934794	0.933958	0.935632	892.0
SR	0.925960	0.925968	0.925960	681.0
macro avg	0.915704	0.916952	0.914619	2077.0
micro avg	0.924434	0.924434	0.924434	2077.0
weighted avg	0.924398	0.924436	0.924434	2077.0

The result of Model Number:Dataset Number:Featuregroup Number:Rescale of 35 : 1 : 5 : 1

	f1-score	precision	recall	support
AFIB	0.667370	0.719386	0.622679	204.0
GSVT	0.687722	0.699363	0.676491	300.0
SB	0.728856	0.722148	0.735712	892.0
SR	0.699846	0.689698	0.710330	681.0
macro avg	0.695948	0.707649	0.686303	2077.0

micro avg 0.707734 0.707734 0.707734 2077.0
weighted avg 0.707364 0.707946 0.707734 2077.0
The result of Model Number:Dataset Number:Featuregroup Number:Rescale of 35 : 1 : 6 : 0

	f1-score	precision	recall	support
AFIB	0.667362	0.659491	0.675457	875.0
GSVT	0.678667	0.682961	0.674451	691.0
SB	0.723590	0.722617	0.724568	1553.0
SR	0.705814	0.712500	0.699275	907.0
macro avg	0.693858	0.694392	0.693438	4026.0
micro avg	0.699595	0.699595	0.699595	4026.0
weighted avg	0.699655	0.699812	0.699595	4026.0

The result of Model Number:Dataset Number:Featuregroup Number:Rescale of 35 : 1 : 6 : 1

	f1-score	precision	recall	support
AFIB	0.878260	0.910684	0.848147	204.0
GSVT	0.897032	0.887825	0.906570	300.0
SB	0.921601	0.917303	0.925940	892.0
SR	0.907231	0.907799	0.906693	681.0
macro avg	0.901031	0.905903	0.896837	2077.0
micro avg	0.909191	0.909191	0.909191	2077.0
weighted avg	0.909083	0.909279	0.909191	2077.0

The result of Model Number:Dataset Number:Featuregroup Number:Rescale of 35 : 1 : 7 : 0

	f1-score	precision	recall	support
AFIB	0.809073	0.823916	0.794818	204.0
GSVT	0.819744	0.811471	0.828204	300.0
SB	0.839936	0.835564	0.844360	892.0
SR	0.828501	0.833693	0.823392	681.0
macro avg	0.824314	0.826161	0.822693	2077.0
micro avg	0.830285	0.830285	0.830285	2077.0
weighted avg	0.830239	0.830326	0.830285	2077.0

The result of Model Number:Dataset Number:Featuregroup Number:Rescale of 35 : 1 : 7 : 1

	f1-score	precision	recall	support
AFIB	0.764792	0.765652	0.764073	204.0
GSVT	0.782351	0.780604	0.784165	300.0
SB	0.798934	0.797867	0.800007	892.0
SR	0.792950	0.794947	0.790973	681.0
macro avg	0.784757	0.784768	0.784805	2077.0
micro avg	0.791227	0.791227	0.791227	2077.0
weighted avg	0.791223	0.791252	0.791227	2077.0

The result of Model Number:Dataset Number:Featuregroup Number:Rescale of 35 : 1 : 8 : 0

	f1-score	precision	recall	support
AFIB	0.824026	0.817681	0.830533	875.0
GSVT	0.834725	0.838600	0.831067	691.0
SB	0.894524	0.889765	0.899353	1553.0
SR	0.868168	0.880374	0.856391	907.0
macro avg	0.855361	0.856605	0.854336	4026.0
micro avg	0.862997	0.862997	0.862997	4026.0
weighted avg	0.863001	0.863201	0.862997	4026.0

The result of Model Number:Dataset Number:Featuregroup Number:Rescale of 35 : 1 : 8 : 1

	f1-score	precision	recall	support
AFIB	0.659647	0.657819	0.661535	875.0
GSVT	0.664867	0.668038	0.661792	691.0
SB	0.695999	0.693775	0.698240	1553.0
SR	0.684872	0.688180	0.681627	907.0
macro avg	0.676346	0.676953	0.675799	4026.0
micro avg	0.680264	0.680264	0.680264	4026.0
weighted avg	0.680248	0.680283	0.680264	4026.0

The result of Model Number:Dataset Number:Featuregroup Number:Rescale of 35 : 1 : 9 : 0

	f1-score	precision	recall	support
AFIB	0.872385	0.879086	0.865983	204.0
GSVT	0.903106	0.902241	0.904003	300.0
SB	0.921583	0.919436	0.923749	892.0
SR	0.913101	0.914332	0.911894	681.0
macro avg	0.902544	0.903774	0.901407	2077.0
micro avg	0.911336	0.911336	0.911336	2077.0
weighted avg	0.911301	0.911316	0.911336	2077.0

The result of Model Number:Dataset Number:Featuregroup Number:Rescale of 35 : 1 : 9 : 1

	f1-score	precision	recall	support
AFIB	0.891545	0.881694	0.901636	875.0
GSVT	0.907608	0.913196	0.902103	691.0
SB	0.965975	0.964367	0.967592	1553.0
SR	0.942454	0.951266	0.933827	907.0
macro avg	0.926895	0.927631	0.926290	4026.0
micro avg	0.934410	0.934410	0.934410	4026.0
weighted avg	0.934482	0.934665	0.934410	4026.0

The result of Model Number:Dataset Number:Featuregroup Number:Rescale of 35 : 1 : 10 : 0

	f1-score	precision	recall	support
AFIB	0.721560	0.727579	0.715889	204.0
GSVT	0.740622	0.737211	0.744080	300.0
SB	0.751791	0.749120	0.754485	892.0

	f1-score	precision	recall	support
SR	0.744847	0.748183	0.741579	681.0
macro avg	0.739705	0.740523	0.739008	2077.0
micro avg	0.744960	0.744960	0.744960	2077.0
weighted avg	0.744932	0.744977	0.744960	2077.0

The result of Model Number:Dataset Number:Featuregroup Number:Rescale of 35 : 1 : 10 : 1

	f1-score	precision	recall	support
AFIB	0.632150	0.689402	0.584731	204.0
GSVT	0.733725	0.725341	0.742531	300.0
SB	0.778072	0.776080	0.780078	892.0
SR	0.759022	0.748743	0.769608	681.0
macro avg	0.725742	0.734892	0.719237	2077.0
micro avg	0.752035	0.752035	0.752035	2077.0
weighted avg	0.751088	0.751275	0.752035	2077.0

The result of Model Number:Dataset Number:Featuregroup Number:Rescale of 36 : 0 : 0 : 0

	f1-score	precision	recall	support
AFIB	0.889477	0.920356	0.860723	204.0
GSVT	0.906532	0.896608	0.916790	300.0
SB	0.930665	0.923982	0.937447	892.0
SR	0.914044	0.918538	0.909609	681.0
macro avg	0.910179	0.914871	0.906142	2077.0
micro avg	0.917800	0.917800	0.917800	2077.0
weighted avg	0.917684	0.917887	0.917800	2077.0

The result of Model Number:Dataset Number:Featuregroup Number:Rescale of 36 : 0 : 0 : 1

	f1-score	precision	recall	support
AFIB	0.874279	0.880522	0.868282	204.0
GSVT	0.904850	0.904267	0.905452	300.0
SB	0.922955	0.920907	0.925021	892.0
SR	0.914705	0.915811	0.913629	681.0
macro avg	0.904197	0.905377	0.903096	2077.0
micro avg	0.912886	0.912886	0.912886	2077.0
weighted avg	0.912854	0.912866	0.912886	2077.0

The result of Model Number:Dataset Number:Featuregroup Number:Rescale of 36 : 0 : 1 : 0

	f1-score	precision	recall	support
AFIB	0.685898	0.676398	0.695697	875.0
GSVT	0.701111	0.706785	0.695531	691.0
SB	0.744221	0.742083	0.746377	1553.0
SR	0.726117	0.735567	0.716913	907.0
macro avg	0.714337	0.715208	0.713629	4026.0
micro avg	0.719997	0.719997	0.719997	4026.0
weighted avg	0.720067	0.720281	0.719997	4026.0

The result of Model Number:Dataset Number:Featuregroup Number:Rescale of 36 : 0 : 1 : 1

	f1-score	precision	recall	support
AFIB	0.658275	0.657488	0.659176	875.0
GSVT	0.671107	0.673416	0.668884	691.0
SB	0.710802	0.706424	0.715241	1553.0
SR	0.696338	0.703139	0.689744	907.0
macro avg	0.684131	0.685116	0.683261	4026.0
micro avg	0.689355	0.689355	0.689355	4026.0
weighted avg	0.689314	0.689383	0.689355	4026.0

The result of Model Number:Dataset Number:Featuregroup Number:Rescale of 36 : 0 : 2 : 0

	f1-score	precision	recall	support
AFIB	0.773862	0.757325	0.791182	875.0
GSVT	0.801383	0.813638	0.789564	691.0
SB	0.854273	0.854386	0.854163	1553.0
SR	0.832265	0.840883	0.823824	907.0
macro avg	0.815446	0.816558	0.814683	4026.0
micro avg	0.822553	0.822553	0.822553	4026.0
weighted avg	0.822761	0.823255	0.822553	4026.0

The result of Model Number:Dataset Number:Featuregroup Number:Rescale of 36 : 0 : 2 : 1

	f1-score	precision	recall	support
AFIB	0.865326	0.873112	0.857949	875.0
GSVT	0.882953	0.865050	0.901821	691.0
SB	0.936377	0.925960	0.947144	1553.0
SR	0.899220	0.925950	0.874202	907.0
macro avg	0.895969	0.897518	0.895279	4026.0
micro avg	0.903547	0.903547	0.903547	4026.0
weighted avg	0.903395	0.904018	0.903547	4026.0

The result of Model Number:Dataset Number:Featuregroup Number:Rescale of 36 : 0 : 3 : 0

	f1-score	precision	recall	support
AFIB	0.857867	0.876978	0.839818	204.0
GSVT	0.868451	0.855731	0.881916	300.0
SB	0.888076	0.879250	0.897119	892.0
SR	0.871694	0.884214	0.859550	681.0
macro avg	0.871522	0.874043	0.869601	2077.0
micro avg	0.876977	0.876977	0.876977	2077.0
weighted avg	0.876903	0.877257	0.876977	2077.0

The result of Model Number:Dataset Number:Featuregroup Number:Rescale of 36 : 0 : 3 : 1

	f1-score	precision	recall	support
AFIB	0.874814	0.881061	0.868813	204.0

GSVT 0.905404 0.904820 0.906006 300.0
SB 0.923519 0.921470 0.925587 892.0
SR 0.915264 0.916371 0.914187 681.0
macro avg 0.904750 0.905931 0.903648 2077.0
micro avg 0.913445 0.913445 0.913445 2077.0
weighted avg 0.913412 0.913424 0.913445 2077.0
The result of Model Number:Dataset Number:Featuregroup Number:Rescale of 36 : 0 : 4 : 0

	f1-score	precision	recall	support
AFIB	0.694505	0.705510	0.683930	204.0
GSVT	0.712356	0.706294	0.718527	300.0
SB	0.724655	0.723040	0.726280	892.0
SR	0.717789	0.719385	0.716214	681.0
macro avg	0.712326	0.713557	0.711238	2077.0
micro avg	0.717700	0.717700	0.717700	2077.0
weighted avg	0.717666	0.717701	0.717700	2077.0

The result of Model Number:Dataset Number:Featuregroup Number:Rescale of 36 : 0 : 4 : 1

	f1-score	precision	recall	support
AFIB	0.908460	0.926601	0.891154	204.0
GSVT	0.918662	0.907183	0.930574	300.0
SB	0.936201	0.927249	0.945329	892.0
SR	0.919199	0.931257	0.907474	681.0
macro avg	0.920630	0.923073	0.918632	2077.0
micro avg	0.925465	0.925465	0.925465	2077.0
weighted avg	0.925368	0.925602	0.925465	2077.0

The result of Model Number:Dataset Number:Featuregroup Number:Rescale of 36 : 0 : 5 : 0

	f1-score	precision	recall	support
AFIB	0.644317	0.646393	0.642315	875.0
GSVT	0.653376	0.643002	0.664148	691.0
SB	0.688437	0.682689	0.694288	1553.0
SR	0.653832	0.670437	0.638053	907.0
macro avg	0.659990	0.660630	0.659701	4026.0
micro avg	0.665150	0.665150	0.665150	4026.0
weighted avg	0.665034	0.665229	0.665150	4026.0

The result of Model Number:Dataset Number:Featuregroup Number:Rescale of 36 : 0 : 5 : 1

	f1-score	precision	recall	support
AFIB	0.697547	0.703985	0.691264	875.0
GSVT	0.713835	0.711908	0.715798	691.0
SB	0.760245	0.753099	0.767535	1553.0
SR	0.739240	0.746657	0.731983	907.0
macro avg	0.727717	0.728912	0.726645	4026.0

micro avg 0.734069 0.734069 0.734069 4026.0
weighted avg 0.733921 0.733903 0.734069 4026.0
The result of Model Number:Dataset Number:Featuregroup Number:Rescale of 36 : 0 : 6 : 0

	f1-score	precision	recall	support
AFIB	0.796841	0.812417	0.781991	204.0
GSVT	0.818737	0.812838	0.824750	300.0
SB	0.833390	0.830147	0.836660	892.0
SR	0.824280	0.826613	0.821979	681.0
macro avg	0.818312	0.820504	0.816345	2077.0
micro avg	0.824756	0.824756	0.824756	2077.0
weighted avg	0.824697	0.824747	0.824756	2077.0

The result of Model Number:Dataset Number:Featuregroup Number:Rescale of 36 : 0 : 6 : 1

	f1-score	precision	recall	support
AFIB	0.756304	0.779682	0.734946	204.0
GSVT	0.706000	0.721348	0.691812	300.0
SB	0.744651	0.711602	0.780957	892.0
SR	0.670477	0.704930	0.639329	681.0
macro avg	0.719358	0.729391	0.711761	2077.0
micro avg	0.717125	0.717125	0.717125	2077.0
weighted avg	0.715893	0.717509	0.717125	2077.0

The result of Model Number:Dataset Number:Featuregroup Number:Rescale of 36 : 0 : 7 : 0

	f1-score	precision	recall	support
AFIB	0.791877	0.811144	0.773699	204.0
GSVT	0.801831	0.790793	0.813289	300.0
SB	0.818022	0.809853	0.826363	892.0
SR	0.803740	0.814287	0.793485	681.0
macro avg	0.803868	0.806519	0.801709	2077.0
micro avg	0.808522	0.808522	0.808522	2077.0
weighted avg	0.808433	0.808681	0.808522	2077.0

The result of Model Number:Dataset Number:Featuregroup Number:Rescale of 36 : 0 : 7 : 1

	f1-score	precision	recall	support
AFIB	0.679832	0.692879	0.667306	875.0
GSVT	0.724680	0.725785	0.723623	691.0
SB	0.789083	0.785616	0.792587	1553.0
SR	0.745659	0.737553	0.754022	907.0
macro avg	0.734814	0.735458	0.734385	4026.0
micro avg	0.744834	0.744834	0.744834	4026.0
weighted avg	0.744502	0.744364	0.744834	4026.0

The result of Model Number:Dataset Number:Featuregroup Number:Rescale of 36 : 0 : 8 : 0

	f1-score	precision	recall	support
AFIB	0.791375	0.810173	0.773501	204.0
GSVT	0.817369	0.809705	0.825265	300.0
SB	0.834923	0.833989	0.835859	892.0
SR	0.826864	0.826033	0.827710	681.0
macro avg	0.817633	0.819975	0.815584	2077.0
micro avg	0.825533	0.825533	0.825533	2077.0
weighted avg	0.825468	0.825534	0.825533	2077.0

The result of Model Number:Dataset Number:Featuregroup Number:Rescale of 36 : 0 : 8 : 1

	f1-score	precision	recall	support
AFIB	0.717490	0.716689	0.718315	875.0
GSVT	0.730614	0.721476	0.740008	691.0
SB	0.767506	0.761336	0.773783	1553.0
SR	0.733264	0.752503	0.714996	907.0
macro avg	0.737219	0.738001	0.736776	4026.0
micro avg	0.742687	0.742687	0.742687	4026.0
weighted avg	0.742590	0.742801	0.742687	4026.0

The result of Model Number:Dataset Number:Featuregroup Number:Rescale of 36 : 0 : 9 : 0

	f1-score	precision	recall	support
AFIB	0.856950	0.903566	0.815127	204.0
GSVT	0.899639	0.886033	0.913706	300.0
SB	0.921018	0.915027	0.927102	892.0
SR	0.909655	0.910475	0.908858	681.0
macro avg	0.896816	0.903775	0.891198	2077.0
micro avg	0.908187	0.908187	0.908187	2077.0
weighted avg	0.907912	0.908221	0.908187	2077.0

The result of Model Number:Dataset Number:Featuregroup Number:Rescale of 36 : 0 : 9 : 1

	f1-score	precision	recall	support
AFIB	0.647899	0.674950	0.623051	875.0
GSVT	0.699957	0.696414	0.703622	691.0
SB	0.767525	0.763205	0.771914	1553.0
SR	0.718671	0.702713	0.735383	907.0
macro avg	0.708513	0.709320	0.708493	4026.0
micro avg	0.719609	0.719609	0.719609	4026.0
weighted avg	0.718923	0.718932	0.719609	4026.0

The result of Model Number:Dataset Number:Featuregroup Number:Rescale of 36 : 0 : 10 : 0

	f1-score	precision	recall	support
AFIB	0.664713	0.668204	0.661297	875.0
GSVT	0.675930	0.677185	0.674765	691.0
SB	0.715902	0.710819	0.721064	1553.0

SR 0.702176 0.706503 0.697919 907.0
macro avg 0.689680 0.690678 0.688761 4026.0
micro avg 0.694914 0.694914 0.694914 4026.0
weighted avg 0.694824 0.694812 0.694914 4026.0
The result of Model Number:Dataset Number:Featuregroup Number:Rescale of 36 : 0 : 10 : 1

	f1-score	precision	recall	support
AFIB	0.787638	0.830484	0.749198	204.0
GSVT	0.826875	0.814369	0.839804	300.0
SB	0.846524	0.841018	0.852117	892.0
SR	0.836080	0.836834	0.835348	681.0
macro avg	0.824279	0.830676	0.819117	2077.0
micro avg	0.834732	0.834732	0.834732	2077.0
weighted avg	0.834478	0.834762	0.834732	2077.0

The result of Model Number:Dataset Number:Featuregroup Number:Rescale of 36 : 1 : 0 : 0

	f1-score	precision	recall	support
AFIB	0.773965	0.768006	0.780077	875.0
GSVT	0.784014	0.787654	0.780578	691.0
SB	0.840180	0.835710	0.844715	1553.0
SR	0.815425	0.826890	0.804364	907.0
macro avg	0.803396	0.804565	0.802434	4026.0
micro avg	0.810568	0.810568	0.810568	4026.0
weighted avg	0.810572	0.810760	0.810568	4026.0

The result of Model Number:Dataset Number:Featuregroup Number:Rescale of 36 : 1 : 0 : 1

	f1-score	precision	recall	support
AFIB	0.860664	0.853770	0.867771	875.0
GSVT	0.884433	0.885942	0.883032	691.0
SB	0.937639	0.935126	0.940173	1553.0
SR	0.912020	0.922637	0.901664	907.0
macro avg	0.898689	0.899369	0.898160	4026.0
micro avg	0.905955	0.905955	0.905955	4026.0
weighted avg	0.906006	0.906189	0.905955	4026.0

The result of Model Number:Dataset Number:Featuregroup Number:Rescale of 36 : 1 : 1 : 0

	f1-score	precision	recall	support
AFIB	0.852347	0.853689	0.851120	204.0
GSVT	0.872961	0.871598	0.874400	300.0
SB	0.890969	0.889882	0.892065	892.0
SR	0.884730	0.886427	0.883042	681.0
macro avg	0.875252	0.875399	0.875157	2077.0
micro avg	0.882534	0.882534	0.882534	2077.0
weighted avg	0.882529	0.882554	0.882534	2077.0

The result of Model Number:Dataset Number:Featuregroup Number:Rescale of 36 : 1 : 1 : 1

	f1-score	precision	recall	support
AFIB	0.925576	0.942556	0.909268	204.0
GSVT	0.937783	0.928319	0.947461	300.0
SB	0.960883	0.955882	0.965944	892.0
SR	0.947802	0.953741	0.941957	681.0
macro avg	0.943011	0.945124	0.941158	2077.0
micro avg	0.949843	0.949843	0.949843	2077.0
weighted avg	0.949790	0.949890	0.949843	2077.0

The result of Model Number:Dataset Number:Featuregroup Number:Rescale of 36 : 1 : 2 : 0

	f1-score	precision	recall	support
AFIB	0.819326	0.825940	0.812887	204.0
GSVT	0.838126	0.834016	0.842305	300.0
SB	0.854483	0.853536	0.855441	892.0
SR	0.848103	0.849231	0.846984	681.0
macro avg	0.840009	0.840681	0.839404	2077.0
micro avg	0.846591	0.846591	0.846591	2077.0
weighted avg	0.846576	0.846594	0.846591	2077.0

The result of Model Number:Dataset Number:Featuregroup Number:Rescale of 36 : 1 : 2 : 1

	f1-score	precision	recall	support
AFIB	0.773644	0.777478	0.769930	875.0
GSVT	0.781347	0.782473	0.780343	691.0
SB	0.822473	0.818657	0.826334	1553.0
SR	0.807333	0.809354	0.805365	907.0
macro avg	0.796199	0.796990	0.795493	4026.0
micro avg	0.801458	0.801458	0.801458	4026.0
weighted avg	0.801391	0.801401	0.801458	4026.0

The result of Model Number:Dataset Number:Featuregroup Number:Rescale of 36 : 1 : 3 : 0

	f1-score	precision	recall	support
AFIB	0.733324	0.803907	0.674386	204.0
GSVT	0.852858	0.844095	0.861965	300.0
SB	0.904095	0.900579	0.907641	892.0
SR	0.883148	0.870935	0.895723	681.0
macro avg	0.843356	0.854879	0.834929	2077.0
micro avg	0.874226	0.874226	0.874226	2077.0
weighted avg	0.873053	0.873206	0.874226	2077.0

The result of Model Number:Dataset Number:Featuregroup Number:Rescale of 36 : 1 : 3 : 1

	f1-score	precision	recall	support
AFIB	0.664996	0.667483	0.662810	875.0

GSVT 0.679964 0.686524 0.673665 691.0
SB 0.723015 0.715431 0.730816 1553.0
SR 0.697978 0.704121 0.692181 907.0
macro avg 0.691488 0.693390 0.689868 4026.0
micro avg 0.697523 0.697523 0.697523 4026.0
weighted avg 0.697376 0.697501 0.697523 4026.0
The result of Model Number:Dataset Number:Featuregroup Number:Rescale of 36 : 1 : 4 : 0

	f1-score	precision	recall	support
AFIB	0.602722	0.656571	0.636487	204.0
GSVT	0.818663	0.805280	0.832781	300.0
SB	0.696935	0.691627	0.702378	892.0
SR	0.852238	0.842595	0.862130	681.0
macro avg	0.742639	0.749018	0.758444	2077.0
micro avg	0.767120	0.767120	0.767120	2077.0
weighted avg	0.756184	0.754099	0.767120	2077.0

The result of Model Number:Dataset Number:Featuregroup Number:Rescale of 36 : 1 : 4 : 1

	f1-score	precision	recall	support
AFIB	0.690155	0.705155	0.675969	204.0
GSVT	0.718644	0.714405	0.722954	300.0
SB	0.734084	0.732455	0.735727	892.0
SR	0.726724	0.726314	0.727153	681.0
macro avg	0.717402	0.719582	0.715451	2077.0
micro avg	0.725202	0.725202	0.725202	2077.0
weighted avg	0.725126	0.725153	0.725202	2077.0

The result of Model Number:Dataset Number:Featuregroup Number:Rescale of 36 : 1 : 5 : 0

	f1-score	precision	recall	support
AFIB	0.814512	0.867582	0.767920	204.0
GSVT	0.850194	0.829296	0.872288	300.0
SB	0.875128	0.863886	0.886697	892.0
SR	0.852398	0.862576	0.842513	681.0
macro avg	0.848058	0.855835	0.842355	2077.0
micro avg	0.858463	0.858463	0.858463	2077.0
weighted avg	0.858120	0.858823	0.858463	2077.0

The result of Model Number:Dataset Number:Featuregroup Number:Rescale of 36 : 1 : 5 : 1

	f1-score	precision	recall	support
AFIB	0.718568	0.715766	0.721443	875.0
GSVT	0.732016	0.733559	0.730522	691.0
SB	0.777060	0.775122	0.779010	1553.0
SR	0.759134	0.764233	0.754119	907.0
macro avg	0.746694	0.747170	0.746274	4026.0

micro avg 0.752569 0.752569 0.752569 4026.0
weighted avg 0.752578 0.752635 0.752569 4026.0
The result of Model Number:Dataset Number:Featuregroup Number:Rescale of 36 : 1 : 6 : 0

	f1-score	precision	recall	support
AFIB	0.142614	0.144112	0.141147	175.0
GSVT	0.142844	0.141327	0.144394	138.2
SB	0.150440	0.149863	0.151021	310.6
SR	0.146958	0.147695	0.146229	181.4
macro avg	0.145714	0.145749	0.145698	805.2
micro avg	0.146658	0.146658	0.146658	805.2
weighted avg	0.146651	0.146659	0.146658	805.2

The result of Model Number:Dataset Number:Featuregroup Number:Rescale of 36 : 1 : 6 : 1

	f1-score	precision	recall	support
AFIB	0.696703	0.688486	0.705148	875.0
GSVT	0.710394	0.716572	0.704327	691.0
SB	0.757104	0.754207	0.760027	1553.0
SR	0.739241	0.748178	0.730522	907.0
macro avg	0.725860	0.726861	0.725006	4026.0
micro avg	0.731893	0.731893	0.731893	4026.0
weighted avg	0.731935	0.732106	0.731893	4026.0

The result of Model Number:Dataset Number:Featuregroup Number:Rescale of 36 : 1 : 7 : 0

	f1-score	precision	recall	support
AFIB	0.770791	0.752827	0.789759	875.0
GSVT	0.739932	0.748066	0.732024	691.0
SB	0.778661	0.728379	0.836416	1553.0
SR	0.623196	0.748338	0.534245	907.0
macro avg	0.728145	0.744403	0.723111	4026.0
micro avg	0.740284	0.740284	0.740284	4026.0
weighted avg	0.735279	0.741568	0.740284	4026.0

The result of Model Number:Dataset Number:Featuregroup Number:Rescale of 36 : 1 : 7 : 1

	f1-score	precision	recall	support
AFIB	0.785864	0.786748	0.785125	204.0
GSVT	0.803907	0.802112	0.805771	300.0
SB	0.820946	0.819850	0.822050	892.0
SR	0.814798	0.816850	0.812766	681.0
macro avg	0.806379	0.806390	0.806428	2077.0
micro avg	0.813028	0.813028	0.813028	2077.0
weighted avg	0.813024	0.813053	0.813028	2077.0

The result of Model Number:Dataset Number:Featuregroup Number:Rescale of 36 : 1 : 8 : 0

f1-score precision recall support
AFIB 0.862609 0.852387 0.873151 875.0
GSVT 0.874887 0.883344 0.866675 691.0
SB 0.928546 0.924994 0.932133 1553.0
SR 0.906898 0.917361 0.896697 907.0
macro avg 0.893235 0.894522 0.892164 4026.0
micro avg 0.900096 0.900096 0.900096 4026.0
weighted avg 0.900129 0.900346 0.900096 4026.0
The result of Model Number:Dataset Number:Featuregroup Number:Rescale of 36 : 1 : 8 : 1
f1-score precision recall support
AFIB 0.907057 0.910120 0.904047 875.0
GSVT 0.904661 0.898289 0.911206 691.0
SB 0.951870 0.943036 0.960878 1553.0
SR 0.915682 0.933329 0.898736 907.0
macro avg 0.919818 0.921193 0.918717 4026.0
micro avg 0.926001 0.926001 0.926001 4026.0
weighted avg 0.925875 0.926015 0.926001 4026.0
The result of Model Number:Dataset Number:Featuregroup Number:Rescale of 36 : 1 : 9 : 0
f1-score precision recall support
AFIB 0.632467 0.622341 0.711653 204.0
GSVT 0.740451 0.731860 0.749517 300.0
SB 0.765244 0.762355 0.768161 892.0
SR 0.600425 0.603529 0.597454 681.0
macro avg 0.684647 0.680021 0.706696 2077.0
micro avg 0.703947 0.703947 0.703947 2077.0
weighted avg 0.694581 0.692123 0.703947 2077.0
The result of Model Number:Dataset Number:Featuregroup Number:Rescale of 36 : 1 : 9 : 1
f1-score precision recall support
AFIB 0.706162 0.730678 0.683334 204.0
GSVT 0.719703 0.711824 0.727846 300.0
SB 0.738862 0.733557 0.744246 892.0
SR 0.725666 0.729234 0.722146 681.0
macro avg 0.722598 0.726323 0.719393 2077.0
micro avg 0.728648 0.728648 0.728648 2077.0
weighted avg 0.728556 0.728718 0.728648 2077.0
The result of Model Number:Dataset Number:Featuregroup Number:Rescale of 36 : 1 : 10 : 0
f1-score precision recall support
AFIB 0.659739 0.718683 0.696699 204.0
GSVT 0.896109 0.881460 0.911562 300.0
SB 0.762865 0.757055 0.768822 892.0

SR 0.932860 0.922305 0.943687 681.0
macro avg 0.812893 0.819876 0.830193 2077.0
micro avg 0.839690 0.839690 0.839690 2077.0
weighted avg 0.827719 0.825437 0.839690 2077.0
The result of Model Number:Dataset Number:Featuregroup Number:Rescale of 36 : 1 : 10 : 1

	f1-score	precision	recall	support
AFIB	0.778470	0.784624	0.772438	875.0
GSVT	0.798059	0.796371	0.799783	691.0
SB	0.850754	0.843234	0.858420	1553.0
SR	0.828106	0.836228	0.820161	907.0
macro avg	0.813847	0.815114	0.812700	4026.0
micro avg	0.821049	0.821049	0.821049	4026.0
weighted avg	0.820897	0.820874	0.821049	4026.0

The result of Model Number:Dataset Number:Featuregroup Number:Rescale of 37 : 0 : 0 : 0

	f1-score	precision	recall	support
AFIB	0.832200	0.841071	0.823651	204.0
GSVT	0.847505	0.842856	0.852299	300.0
SB	0.867068	0.865623	0.868523	892.0
SR	0.859840	0.861241	0.858455	681.0
macro avg	0.851653	0.852697	0.850732	2077.0
micro avg	0.858471	0.858471	0.858471	2077.0
weighted avg	0.858448	0.858486	0.858471	2077.0

The result of Model Number:Dataset Number:Featuregroup Number:Rescale of 37 : 0 : 0 : 1

	f1-score	precision	recall	support
AFIB	0.181360	0.183265	0.179495	175.0
GSVT	0.181652	0.179723	0.183624	138.2
SB	0.191312	0.190578	0.192051	310.6
SR	0.186884	0.187821	0.185957	181.4
macro avg	0.185302	0.185347	0.185282	805.2
micro avg	0.186503	0.186503	0.186503	805.2
weighted avg	0.186494	0.186504	0.186503	805.2

The result of Model Number:Dataset Number:Featuregroup Number:Rescale of 37 : 0 : 1 : 0

	f1-score	precision	recall	support
AFIB	0.717931	0.713236	0.722750	875.0
GSVT	0.739269	0.745559	0.733183	691.0
SB	0.787052	0.785338	0.788775	1553.0
SR	0.767871	0.770954	0.764823	907.0
macro avg	0.753031	0.753772	0.752383	4026.0
micro avg	0.759488	0.759488	0.759488	4026.0
weighted avg	0.759507	0.759599	0.759488	4026.0

The result of Model Number:Dataset Number:Featuregroup Number:Rescale of 37 : 0 : 1 : 1

	f1-score	precision	recall	support
AFIB	0.812305	0.781250	0.863401	875.0
GSVT	0.841685	0.812636	0.881086	691.0
SB	0.937793	0.921240	0.955983	1553.0
SR	0.794290	0.955088	0.685762	907.0
macro avg	0.846518	0.867554	0.846558	4026.0
micro avg	0.862130	0.862130	0.862130	4026.0
weighted avg	0.861695	0.879800	0.862130	4026.0

The result of Model Number:Dataset Number:Featuregroup Number:Rescale of 37 : 0 : 2 : 0

	f1-score	precision	recall	support
AFIB	0.757893	0.782187	0.735290	204.0
GSVT	0.780567	0.767138	0.794590	300.0
SB	0.794070	0.786220	0.802080	892.0
SR	0.772838	0.782509	0.763420	681.0
macro avg	0.776342	0.779514	0.773845	2077.0
micro avg	0.781763	0.781763	0.781763	2077.0
weighted avg	0.781605	0.781851	0.781763	2077.0

The result of Model Number:Dataset Number:Featuregroup Number:Rescale of 37 : 0 : 2 : 1

	f1-score	precision	recall	support
AFIB	0.743564	0.743762	0.743495	875.0
GSVT	0.756512	0.753719	0.759421	691.0
SB	0.809320	0.807355	0.811298	1553.0
SR	0.790019	0.795689	0.784451	907.0
macro avg	0.774854	0.775131	0.774666	4026.0
micro avg	0.781610	0.781610	0.781610	4026.0
weighted avg	0.781617	0.781700	0.781610	4026.0

The result of Model Number:Dataset Number:Featuregroup Number:Rescale of 37 : 0 : 3 : 0

	f1-score	precision	recall	support
AFIB	0.670610	0.652010	0.692413	875.0
GSVT	0.679365	0.707980	0.657152	691.0
SB	0.746306	0.745929	0.746693	1553.0
SR	0.727583	0.732136	0.723091	907.0
macro avg	0.705966	0.709514	0.704837	4026.0
micro avg	0.714211	0.714211	0.714211	4026.0
weighted avg	0.714147	0.715896	0.714211	4026.0

The result of Model Number:Dataset Number:Featuregroup Number:Rescale of 37 : 0 : 3 : 1

	f1-score	precision	recall	support
AFIB	0.763980	0.765878	0.762143	875.0

GSVT 0.777137 0.779471 0.774914 691.0
SB 0.824402 0.818806 0.830080 1553.0
SR 0.808714 0.814718 0.802830 907.0
macro avg 0.793558 0.794718 0.792492 4026.0
micro avg 0.799708 0.799708 0.799708 4026.0
weighted avg 0.799623 0.799631 0.799708 4026.0
The result of Model Number:Dataset Number:Featuregroup Number:Rescale of 37 : 0 : 4 : 0

	f1-score	precision	recall	support
AFIB	0.910834	0.934902	0.888063	204.0
GSVT	0.935813	0.928039	0.943889	300.0
SB	0.961600	0.958071	0.965157	892.0
SR	0.946711	0.947858	0.945592	681.0
macro avg	0.938739	0.942218	0.935675	2077.0
micro avg	0.948098	0.948098	0.948098	2077.0
weighted avg	0.948007	0.948109	0.948098	2077.0

The result of Model Number:Dataset Number:Featuregroup Number:Rescale of 37 : 0 : 4 : 1

	f1-score	precision	recall	support
AFIB	0.842209	0.873541	0.813426	204.0
GSVT	0.781539	0.817806	0.748424	300.0
SB	0.818693	0.769313	0.874861	892.0
SR	0.718970	0.768924	0.675195	681.0
macro avg	0.790353	0.807396	0.777977	2077.0
micro avg	0.785099	0.785099	0.785099	2077.0
weighted avg	0.782939	0.786427	0.785099	2077.0

The result of Model Number:Dataset Number:Featuregroup Number:Rescale of 37 : 0 : 5 : 0

	f1-score	precision	recall	support
AFIB	0.797221	0.784997	0.809963	875.0
GSVT	0.824710	0.832794	0.816944	691.0
SB	0.877712	0.875661	0.879799	1553.0
SR	0.855786	0.866637	0.845222	907.0
macro avg	0.838857	0.840022	0.837982	4026.0
micro avg	0.846044	0.846044	0.846044	4026.0
weighted avg	0.846181	0.846566	0.846044	4026.0

The result of Model Number:Dataset Number:Featuregroup Number:Rescale of 37 : 0 : 5 : 1

	f1-score	precision	recall	support
AFIB	0.757894	0.762430	0.753450	204.0
GSVT	0.778084	0.775771	0.780416	300.0
SB	0.789999	0.789030	0.790972	892.0
SR	0.784240	0.785165	0.783323	681.0
macro avg	0.777554	0.778099	0.777040	2077.0

micro avg 0.783254 0.783254 0.783254 2077.0
weighted avg 0.783236 0.783235 0.783254 2077.0
The result of Model Number:Dataset Number:Featuregroup Number:Rescale of 37 : 0 : 6 : 0

	f1-score	precision	recall	support
AFIB	0.760720	0.766152	0.755501	204.0
GSVT	0.787320	0.786812	0.787844	300.0
SB	0.803073	0.801291	0.804871	892.0
SR	0.795894	0.796857	0.794958	681.0
macro avg	0.786752	0.787778	0.785793	2077.0
micro avg	0.794312	0.794312	0.794312	2077.0
weighted avg	0.794284	0.794295	0.794312	2077.0

The result of Model Number:Dataset Number:Featuregroup Number:Rescale of 37 : 0 : 6 : 1

	f1-score	precision	recall	support
AFIB	0.725101	0.732141	0.718645	204.0
GSVT	0.746837	0.743306	0.750598	300.0
SB	0.761960	0.761110	0.762814	892.0
SR	0.755256	0.756038	0.754479	681.0
macro avg	0.747289	0.748149	0.746634	2077.0
micro avg	0.753979	0.753979	0.753979	2077.0
weighted avg	0.753957	0.754030	0.753979	2077.0

The result of Model Number:Dataset Number:Featuregroup Number:Rescale of 37 : 0 : 7 : 0

	f1-score	precision	recall	support
AFIB	0.915606	0.945349	0.887851	204.0
GSVT	0.932963	0.924612	0.941652	300.0
SB	0.955148	0.948601	0.961787	892.0
SR	0.939570	0.943535	0.935695	681.0
macro avg	0.935822	0.940524	0.931746	2077.0
micro avg	0.943062	0.943062	0.943062	2077.0
weighted avg	0.942952	0.943155	0.943062	2077.0

The result of Model Number:Dataset Number:Featuregroup Number:Rescale of 37 : 0 : 7 : 1

	f1-score	precision	recall	support
AFIB	0.709979	0.715859	0.799694	204.0
GSVT	0.769924	0.736970	0.868655	300.0
SB	0.702969	0.700527	0.705432	892.0
SR	0.695980	0.696111	0.695853	681.0
macro avg	0.719713	0.712367	0.767408	2077.0
micro avg	0.735125	0.735125	0.735125	2077.0
weighted avg	0.711037	0.705849	0.735125	2077.0

The result of Model Number:Dataset Number:Featuregroup Number:Rescale of 37 : 0 : 8 : 0

	f1-score	precision	recall	support
AFIB	0.777870	0.777487	0.778313	875.0
GSVT	0.794756	0.790845	0.798755	691.0
SB	0.831329	0.825894	0.836841	1553.0
SR	0.807663	0.820819	0.794944	907.0
macro avg	0.802905	0.803762	0.802213	4026.0
micro avg	0.808145	0.808145	0.808145	4026.0
weighted avg	0.808102	0.808215	0.808145	4026.0

The result of Model Number:Dataset Number:Featuregroup Number:Rescale of 37 : 0 : 8 : 1

	f1-score	precision	recall	support
AFIB	0.908122	0.920148	0.896543	204.0
GSVT	0.935527	0.932150	0.938949	300.0
SB	0.947179	0.944231	0.950149	892.0
SR	0.936858	0.938663	0.935087	681.0
macro avg	0.931922	0.933798	0.930182	2077.0
micro avg	0.938328	0.938328	0.938328	2077.0
weighted avg	0.938276	0.938295	0.938328	2077.0

The result of Model Number:Dataset Number:Featuregroup Number:Rescale of 37 : 0 : 9 : 0

	f1-score	precision	recall	support
AFIB	0.781907	0.800670	0.764359	204.0
GSVT	0.809710	0.805017	0.814556	300.0
SB	0.827003	0.823969	0.830061	892.0
SR	0.819816	0.820424	0.819218	681.0
macro avg	0.809609	0.812520	0.807048	2077.0
micro avg	0.817813	0.817813	0.817813	2077.0
weighted avg	0.817719	0.817781	0.817813	2077.0

The result of Model Number:Dataset Number:Featuregroup Number:Rescale of 37 : 0 : 9 : 1

	f1-score	precision	recall	support
AFIB	0.780891	0.795877	0.766503	875.0
GSVT	0.832405	0.833675	0.831191	691.0
SB	0.906383	0.902399	0.910407	1553.0
SR	0.856503	0.847192	0.866109	907.0
macro avg	0.844045	0.844786	0.843553	4026.0
micro avg	0.855556	0.855556	0.855556	4026.0
weighted avg	0.855174	0.855015	0.855556	4026.0

The result of Model Number:Dataset Number:Featuregroup Number:Rescale of 37 : 0 : 10 : 0

	f1-score	precision	recall	support
AFIB	0.740009	0.749191	0.731298	204.0
GSVT	0.753278	0.748131	0.758575	300.0
SB	0.771117	0.769912	0.772326	892.0

SR	0.765577	0.766819	0.764347	681.0
macro avg	0.757495	0.758513	0.756636	2077.0
micro avg	0.763694	0.763694	0.763694	2077.0
weighted avg	0.763669	0.763717	0.763694	2077.0

The result of Model Number:Dataset Number:Featuregroup Number:Rescale of 37 : 0 : 10 : 1

	f1-score	precision	recall	support
AFIB	0.647372	0.691252	0.609161	204.0
GSVT	0.609978	0.628076	0.593123	300.0
SB	0.637579	0.606972	0.671800	892.0
SR	0.565734	0.591419	0.542804	681.0
macro avg	0.615166	0.629430	0.604222	2077.0
micro avg	0.611989	0.611989	0.611989	2077.0
weighted avg	0.610998	0.613199	0.611989	2077.0

The result of Model Number:Dataset Number:Featuregroup Number:Rescale of 37 : 1 : 0 : 0

	f1-score	precision	recall	support
AFIB	0.767076	0.779533	0.755053	875.0
GSVT	0.815808	0.818365	0.813317	691.0
SB	0.887558	0.884500	0.890643	1553.0
SR	0.840259	0.830900	0.849882	907.0
macro avg	0.827675	0.828324	0.827224	4026.0
micro avg	0.838719	0.838719	0.838719	4026.0
weighted avg	0.838402	0.838260	0.838719	4026.0

The result of Model Number:Dataset Number:Featuregroup Number:Rescale of 37 : 1 : 0 : 1

	f1-score	precision	recall	support
AFIB	0.673336	0.757107	0.606850	204.0
GSVT	0.798864	0.781805	0.816719	300.0
SB	0.852802	0.848454	0.857198	892.0
SR	0.833670	0.823113	0.844555	681.0
macro avg	0.789668	0.802620	0.781330	2077.0
micro avg	0.822617	0.822617	0.822617	2077.0
weighted avg	0.821111	0.821547	0.822617	2077.0

The result of Model Number:Dataset Number:Featuregroup Number:Rescale of 37 : 1 : 1 : 0

	f1-score	precision	recall	support
AFIB	0.809502	0.788236	0.860179	875.0
GSVT	0.778733	0.737081	0.887740	691.0
SB	0.737890	0.733227	0.742635	1553.0
SR	0.716743	0.729093	0.705005	907.0
macro avg	0.760717	0.746909	0.798890	4026.0
micro avg	0.784609	0.784609	0.784609	4026.0
weighted avg	0.755700	0.744913	0.784609	4026.0

The result of Model Number:Dataset Number:Featuregroup Number:Rescale of 37 : 1 : 1 : 1

	f1-score	precision	recall	support
AFIB	0.642825	0.644896	0.640828	875.0
GSVT	0.651863	0.641514	0.662610	691.0
SB	0.686843	0.681108	0.692680	1553.0
SR	0.652318	0.668885	0.636575	907.0
macro avg	0.658462	0.659101	0.658173	4026.0
micro avg	0.663610	0.663610	0.663610	4026.0
weighted avg	0.663495	0.663689	0.663610	4026.0

The result of Model Number:Dataset Number:Featuregroup Number:Rescale of 37 : 1 : 2 : 0

	f1-score	precision	recall	support
AFIB	0.693202	0.706752	0.680283	204.0
GSVT	0.712249	0.707118	0.717481	300.0
SB	0.724997	0.722175	0.727842	892.0
SR	0.717072	0.719101	0.715070	681.0
macro avg	0.711880	0.713787	0.710169	2077.0
micro avg	0.717486	0.717486	0.717486	2077.0
weighted avg	0.717434	0.717478	0.717486	2077.0

The result of Model Number:Dataset Number:Featuregroup Number:Rescale of 37 : 1 : 2 : 1

	f1-score	precision	recall	support
AFIB	0.791026	0.816718	0.767056	204.0
GSVT	0.812865	0.802500	0.823664	300.0
SB	0.835798	0.832646	0.838982	892.0
SR	0.824108	0.825600	0.822653	681.0
macro avg	0.815949	0.819366	0.813089	2077.0
micro avg	0.824351	0.824351	0.824351	2077.0
weighted avg	0.824255	0.824417	0.824351	2077.0

The result of Model Number:Dataset Number:Featuregroup Number:Rescale of 37 : 1 : 3 : 0

	f1-score	precision	recall	support
AFIB	0.785231	0.794413	0.776405	204.0
GSVT	0.708282	0.681241	0.808140	300.0
SB	0.657141	0.655593	0.658703	892.0
SR	0.815948	0.817516	0.814391	681.0
macro avg	0.741651	0.737191	0.764410	2077.0
micro avg	0.742895	0.742895	0.742895	2077.0
weighted avg	0.729178	0.726023	0.742895	2077.0

The result of Model Number:Dataset Number:Featuregroup Number:Rescale of 37 : 1 : 3 : 1

	f1-score	precision	recall	support
AFIB	0.934181	0.944139	0.924584	204.0

GSVT 0.951361 0.946142 0.956743 300.0
SB 0.973321 0.971699 0.974955 892.0
SR 0.965208 0.966780 0.963653 681.0
macro avg 0.956018 0.957190 0.954984 2077.0
micro avg 0.963671 0.963671 0.963671 2077.0
weighted avg 0.963645 0.963688 0.963671 2077.0
The result of Model Number:Dataset Number:Featuregroup Number:Rescale of 37 : 1 : 4 : 0

	f1-score	precision	recall	support
AFIB	0.739579	0.749458	0.730166	204.0
GSVT	0.752645	0.748060	0.757401	300.0
SB	0.770787	0.770104	0.771477	892.0
SR	0.765092	0.765208	0.764981	681.0
macro avg	0.757026	0.758207	0.756006	2077.0
micro avg	0.763256	0.763256	0.763256	2077.0
weighted avg	0.763234	0.763287	0.763256	2077.0

The result of Model Number:Dataset Number:Featuregroup Number:Rescale of 37 : 1 : 4 : 1

	f1-score	precision	recall	support
AFIB	0.691561	0.701086	0.682594	875.0
GSVT	0.706658	0.703731	0.709800	691.0
SB	0.752701	0.742832	0.762867	1553.0
SR	0.729069	0.739957	0.718821	907.0
macro avg	0.719997	0.721902	0.718521	4026.0
micro avg	0.726390	0.726390	0.726390	4026.0
weighted avg	0.726186	0.726400	0.726390	4026.0

The result of Model Number:Dataset Number:Featuregroup Number:Rescale of 37 : 1 : 5 : 0

	f1-score	precision	recall	support
AFIB	0.830418	0.886706	0.781403	204.0
GSVT	0.782451	0.805666	0.760830	300.0
SB	0.817856	0.778595	0.861753	892.0
SR	0.725697	0.758644	0.696283	681.0
macro avg	0.789105	0.807403	0.775067	2077.0
micro avg	0.785030	0.785030	0.785030	2077.0
weighted avg	0.783759	0.786582	0.785030	2077.0

The result of Model Number:Dataset Number:Featuregroup Number:Rescale of 37 : 1 : 5 : 1

	f1-score	precision	recall	support
AFIB	0.818114	0.823840	0.812497	204.0
GSVT	0.837149	0.833021	0.841330	300.0
SB	0.853787	0.852366	0.855223	892.0
SR	0.845290	0.847296	0.843303	681.0
macro avg	0.838585	0.839131	0.838088	2077.0

micro avg 0.845111 0.845111 0.845111 2077.0
weighted avg 0.845094 0.845108 0.845111 2077.0
The result of Model Number:Dataset Number:Featuregroup Number:Rescale of 37 : 1 : 6 : 0
f1-score precision recall support
AFIB 0.649951 0.658903 0.641523 875.0
GSVT 0.664140 0.661389 0.667093 691.0
SB 0.707412 0.698137 0.716967 1553.0
SR 0.685203 0.695435 0.675571 907.0
macro avg 0.676676 0.678466 0.675289 4026.0
micro avg 0.682684 0.682684 0.682684 4026.0
weighted avg 0.682493 0.682694 0.682684 4026.0
The result of Model Number:Dataset Number:Featuregroup Number:Rescale of 37 : 1 : 6 : 1
f1-score precision recall support
AFIB 0.857712 0.874993 0.841651 204.0
GSVT 0.885233 0.878124 0.892630 300.0
SB 0.904397 0.903487 0.905310 892.0
SR 0.896154 0.895639 0.896679 681.0
macro avg 0.885874 0.888061 0.884067 2077.0
micro avg 0.894396 0.894396 0.894396 2077.0
weighted avg 0.894341 0.894452 0.894396 2077.0
The result of Model Number:Dataset Number:Featuregroup Number:Rescale of 37 : 1 : 7 : 0
f1-score precision recall support
AFIB 0.786721 0.805863 0.768662 204.0
GSVT 0.796611 0.785644 0.807994 300.0
SB 0.812696 0.804580 0.820983 892.0
SR 0.798507 0.808985 0.788319 681.0
macro avg 0.798634 0.801268 0.796489 2077.0
micro avg 0.803258 0.803258 0.803258 2077.0
weighted avg 0.803169 0.803415 0.803258 2077.0
The result of Model Number:Dataset Number:Featuregroup Number:Rescale of 37 : 1 : 7 : 1
f1-score precision recall support
AFIB 0.885145 0.897675 0.873686 204.0
GSVT 0.903522 0.886887 0.920959 300.0
SB 0.922779 0.912577 0.933228 892.0
SR 0.903386 0.921786 0.885761 681.0
macro avg 0.903708 0.904731 0.903409 2077.0
micro avg 0.910044 0.910044 0.910044 2077.0
weighted avg 0.909943 0.910422 0.910044 2077.0
The result of Model Number:Dataset Number:Featuregroup Number:Rescale of 37 : 1 : 8 : 0

	f1-score	precision	recall	support
AFIB	0.742056	0.752958	0.731534	204.0
GSVT	0.760858	0.754389	0.767454	300.0
SB	0.774007	0.770911	0.777131	892.0
SR	0.766352	0.770114	0.762636	681.0
macro avg	0.760818	0.762093	0.759689	2077.0
micro avg	0.766502	0.766502	0.766502	2077.0
weighted avg	0.766460	0.766500	0.766502	2077.0

The result of Model Number:Dataset Number:Featuregroup Number:Rescale of 37 : 1 : 8 : 1

	f1-score	precision	recall	support
AFIB	0.786775	0.776331	0.797566	875.0
GSVT	0.802745	0.810355	0.795311	691.0
SB	0.854235	0.852215	0.856270	1553.0
SR	0.831061	0.839800	0.822537	907.0
macro avg	0.818704	0.819675	0.817921	4026.0
micro avg	0.825449	0.825449	0.825449	4026.0
weighted avg	0.825515	0.825741	0.825449	4026.0

The result of Model Number:Dataset Number:Featuregroup Number:Rescale of 37 : 1 : 9 : 0

	f1-score	precision	recall	support
AFIB	0.851934	0.862876	0.841308	875.0
GSVT	0.861359	0.854393	0.868577	691.0
SB	0.921407	0.918637	0.924197	1553.0
SR	0.898592	0.898214	0.898981	907.0
macro avg	0.883323	0.883530	0.883266	4026.0
micro avg	0.890955	0.890955	0.890955	4026.0
weighted avg	0.890862	0.890891	0.890955	4026.0

The result of Model Number:Dataset Number:Featuregroup Number:Rescale of 37 : 1 : 9 : 1

	f1-score	precision	recall	support
AFIB	0.859672	0.904085	0.819674	204.0
GSVT	0.901440	0.892628	0.910446	300.0
SB	0.917334	0.909954	0.924839	892.0
SR	0.906003	0.907106	0.904940	681.0
macro avg	0.896112	0.903443	0.889975	2077.0
micro avg	0.905907	0.905907	0.905907	2077.0
weighted avg	0.905660	0.905941	0.905907	2077.0

The result of Model Number:Dataset Number:Featuregroup Number:Rescale of 37 : 1 : 10 : 0

	f1-score	precision	recall	support
AFIB	0.883710	0.889489	0.878061	204.0
GSVT	0.905851	0.903765	0.907958	300.0
SB	0.922021	0.920690	0.923362	892.0

SR 0.914754 0.915710 0.913817 681.0
macro avg 0.906584 0.907413 0.905799 2077.0
micro avg 0.913558 0.913558 0.913558 2077.0
weighted avg 0.913540 0.913548 0.913558 2077.0
The result of Model Number:Dataset Number:Featuregroup Number:Rescale of 37 : 1 : 10 : 1

	f1-score	precision	recall	support
AFIB	0.741763	0.759811	0.724736	204.0
GSVT	0.751088	0.740748	0.761820	300.0
SB	0.766254	0.758602	0.774067	892.0
SR	0.752875	0.762755	0.743269	681.0
macro avg	0.752995	0.755479	0.750973	2077.0
micro avg	0.757355	0.757355	0.757355	2077.0
weighted avg	0.757271	0.757503	0.757355	2077.0

The result of Model Number:Dataset Number:Featuregroup Number:Rescale of 38 : 0 : 0 : 0

	f1-score	precision	recall	support
AFIB	0.687693	0.699126	0.676786	204.0
GSVT	0.700880	0.695900	0.705983	300.0
SB	0.717136	0.713106	0.721229	892.0
SR	0.708000	0.712332	0.703744	681.0
macro avg	0.703427	0.705116	0.701935	2077.0
micro avg	0.708929	0.708929	0.708929	2077.0
weighted avg	0.708901	0.708994	0.708929	2077.0

The result of Model Number:Dataset Number:Featuregroup Number:Rescale of 38 : 0 : 0 : 1

	f1-score	precision	recall	support
AFIB	0.586294	0.650666	0.533653	204.0
GSVT	0.684578	0.676019	0.693490	300.0
SB	0.728357	0.724640	0.732112	892.0
SR	0.711294	0.701350	0.721528	681.0
macro avg	0.677631	0.688169	0.670196	2077.0
micro avg	0.703571	0.703571	0.703571	2077.0
weighted avg	0.702486	0.702715	0.703571	2077.0

The result of Model Number:Dataset Number:Featuregroup Number:Rescale of 38 : 0 : 1 : 0

	f1-score	precision	recall	support
AFIB	0.774440	0.775993	0.772927	875.0
GSVT	0.776358	0.772167	0.780616	691.0
SB	0.811555	0.807219	0.815943	1553.0
SR	0.792140	0.801534	0.782986	907.0
macro avg	0.788623	0.789228	0.788118	4026.0
micro avg	0.793106	0.793106	0.793106	4026.0
weighted avg	0.793074	0.793135	0.793106	4026.0

The result of Model Number:Dataset Number:Featuregroup Number:Rescale of 38 : 0 : 1 : 1

	f1-score	precision	recall	support
AFIB	0.855720	0.858229	0.853292	875.0
GSVT	0.869880	0.858707	0.881459	691.0
SB	0.920623	0.918385	0.922876	1553.0
SR	0.897311	0.907960	0.886919	907.0
macro avg	0.885884	0.885820	0.886136	4026.0
micro avg	0.892543	0.892543	0.892543	4026.0
weighted avg	0.892556	0.892720	0.892543	4026.0

The result of Model Number:Dataset Number:Featuregroup Number:Rescale of 38 : 0 : 2 : 0

	f1-score	precision	recall	support
AFIB	0.782931	0.867500	0.713529	204.0
GSVT	0.907584	0.892823	0.922984	300.0
SB	0.967882	0.963476	0.972331	892.0
SR	0.944496	0.932477	0.956842	681.0
macro avg	0.900723	0.914069	0.891421	2077.0
micro avg	0.934706	0.934706	0.934706	2077.0
weighted avg	0.933339	0.933680	0.934706	2077.0

The result of Model Number:Dataset Number:Featuregroup Number:Rescale of 38 : 0 : 2 : 1

	f1-score	precision	recall	support
AFIB	0.848307	0.850460	0.846195	875.0
GSVT	0.863276	0.853925	0.872871	691.0
SB	0.909846	0.899554	0.920390	1553.0
SR	0.868246	0.892016	0.845745	907.0
macro avg	0.872419	0.873989	0.871300	4026.0
micro avg	0.879293	0.879293	0.879293	4026.0
weighted avg	0.879107	0.879354	0.879293	4026.0

The result of Model Number:Dataset Number:Featuregroup Number:Rescale of 38 : 0 : 3 : 0

	f1-score	precision	recall	support
AFIB	0.855206	0.843073	0.867714	875.0
GSVT	0.872801	0.881308	0.864463	691.0
SB	0.928830	0.925803	0.931879	1553.0
SR	0.907139	0.918733	0.895844	907.0
macro avg	0.890994	0.892229	0.889975	4026.0
micro avg	0.898245	0.898245	0.898245	4026.0
weighted avg	0.898326	0.898593	0.898245	4026.0

The result of Model Number:Dataset Number:Featuregroup Number:Rescale of 38 : 0 : 3 : 1

	f1-score	precision	recall	support
AFIB	0.729889	0.734998	0.724878	204.0

GSVT 0.746871 0.743189 0.750601 300.0
SB 0.761715 0.760448 0.762996 892.0
SR 0.754134 0.755925 0.752361 681.0
macro avg 0.748153 0.748640 0.747709 2077.0
micro avg 0.753975 0.753975 0.753975 2077.0
weighted avg 0.753960 0.753972 0.753975 2077.0
The result of Model Number:Dataset Number:Featuregroup Number:Rescale of 38 : 0 : 4 : 0
f1-score precision recall support
AFIB 0.883236 0.881005 0.885617 875.0
GSVT 0.900468 0.895517 0.905522 691.0
SB 0.942861 0.937177 0.948628 1553.0
SR 0.915061 0.931577 0.899138 907.0
macro avg 0.910407 0.911319 0.909726 4026.0
micro avg 0.916385 0.916385 0.916385 4026.0
weighted avg 0.916363 0.916557 0.916385 4026.0
The result of Model Number:Dataset Number:Featuregroup Number:Rescale of 38 : 0 : 4 : 1
f1-score precision recall support
AFIB 0.861323 0.880483 0.843164 204.0
GSVT 0.898020 0.892439 0.903705 300.0
SB 0.917514 0.915581 0.919466 892.0
SR 0.907741 0.907099 0.908413 681.0
macro avg 0.896149 0.898901 0.893687 2077.0
micro avg 0.906071 0.906071 0.906071 2077.0
weighted avg 0.905975 0.906010 0.906071 2077.0
The result of Model Number:Dataset Number:Featuregroup Number:Rescale of 38 : 0 : 5 : 0
f1-score precision recall support
AFIB 0.597867 0.611491 0.584932 875.0
GSVT 0.636207 0.637464 0.635167 691.0
SB 0.696053 0.692243 0.699907 1553.0
SR 0.657874 0.649894 0.666145 907.0
macro avg 0.647000 0.647773 0.646537 4026.0
micro avg 0.656201 0.656201 0.656201 4026.0
weighted avg 0.655841 0.655750 0.656201 4026.0
The result of Model Number:Dataset Number:Featuregroup Number:Rescale of 38 : 0 : 5 : 1
f1-score precision recall support
AFIB 0.638252 0.665245 0.613575 204.0
GSVT 0.587783 0.588812 0.595575 300.0
SB 0.632803 0.602657 0.666738 892.0
SR 0.559429 0.604487 0.521634 681.0
macro avg 0.604567 0.615300 0.599381 2077.0

micro avg 0.603662 0.603662 0.603662 2077.0
weighted avg 0.602778 0.607405 0.603662 2077.0
The result of Model Number:Dataset Number:Featuregroup Number:Rescale of 38 : 0 : 6 : 0
f1-score precision recall support
AFIB 0.688152 0.722535 0.657239 204.0
GSVT 0.708898 0.695761 0.722788 300.0
SB 0.725121 0.717812 0.732596 892.0
SR 0.705011 0.711308 0.698888 681.0
macro avg 0.706795 0.711854 0.702878 2077.0
micro avg 0.712726 0.712726 0.712726 2077.0
weighted avg 0.712553 0.712958 0.712726 2077.0
The result of Model Number:Dataset Number:Featuregroup Number:Rescale of 38 : 0 : 6 : 1
f1-score precision recall support
AFIB 0.646698 0.652452 0.641079 875.0
GSVT 0.660272 0.653026 0.667723 691.0
SB 0.710119 0.704590 0.715741 1553.0
SR 0.686521 0.696252 0.677061 907.0
macro avg 0.675902 0.676580 0.675401 4026.0
micro avg 0.682559 0.682559 0.682559 4026.0
weighted avg 0.682463 0.682530 0.682559 4026.0
The result of Model Number:Dataset Number:Featuregroup Number:Rescale of 38 : 0 : 7 : 0
f1-score precision recall support
AFIB 0.690536 0.703351 0.678351 204.0
GSVT 0.705720 0.702020 0.709474 300.0
SB 0.716987 0.714438 0.719558 892.0
SR 0.709605 0.710884 0.708362 681.0
macro avg 0.705712 0.707673 0.703936 2077.0
micro avg 0.710383 0.710383 0.710383 2077.0
weighted avg 0.710341 0.710390 0.710383 2077.0
The result of Model Number:Dataset Number:Featuregroup Number:Rescale of 38 : 0 : 7 : 1
f1-score precision recall support
AFIB 0.710937 0.724002 0.698422 204.0
GSVT 0.722878 0.718144 0.727695 300.0
SB 0.735244 0.731654 0.738870 892.0
SR 0.727644 0.730669 0.724654 681.0
macro avg 0.724176 0.726117 0.722410 2077.0
micro avg 0.728622 0.728622 0.728622 2077.0
weighted avg 0.728578 0.728628 0.728622 2077.0
The result of Model Number:Dataset Number:Featuregroup Number:Rescale of 38 : 0 : 8 : 0

	f1-score	precision	recall	support
AFIB	0.901903	0.919502	0.885121	204.0
GSVT	0.924382	0.912454	0.936687	300.0
SB	0.940115	0.930821	0.949601	892.0
SR	0.920465	0.933254	0.908028	681.0
macro avg	0.921716	0.924008	0.919859	2077.0
micro avg	0.927772	0.927772	0.927772	2077.0
weighted avg	0.927647	0.927854	0.927772	2077.0

The result of Model Number:Dataset Number:Featuregroup Number:Rescale of 38 : 0 : 8 : 1

	f1-score	precision	recall	support
AFIB	0.742147	0.721563	0.766276	875.0
GSVT	0.751835	0.783504	0.727254	691.0
SB	0.825917	0.825500	0.826346	1553.0
SR	0.805197	0.810236	0.800226	907.0
macro avg	0.781274	0.785201	0.780025	4026.0
micro avg	0.790398	0.790398	0.790398	4026.0
weighted avg	0.790328	0.792264	0.790398	4026.0

The result of Model Number:Dataset Number:Featuregroup Number:Rescale of 38 : 0 : 9 : 0

	f1-score	precision	recall	support
AFIB	0.691727	0.671798	0.758957	875.0
GSVT	0.762376	0.759941	0.764925	691.0
SB	0.643557	0.634073	0.653382	1553.0
SR	0.770583	0.785623	0.756204	907.0
macro avg	0.717061	0.712859	0.733367	4026.0
micro avg	0.718636	0.718636	0.718636	4026.0
weighted avg	0.703037	0.698017	0.718636	4026.0

The result of Model Number:Dataset Number:Featuregroup Number:Rescale of 38 : 0 : 9 : 1

	f1-score	precision	recall	support
AFIB	0.781667	0.785261	0.778146	875.0
GSVT	0.749177	0.753690	0.744742	691.0
SB	0.803615	0.767794	0.842981	1553.0
SR	0.687266	0.748179	0.635663	907.0
macro avg	0.755431	0.763731	0.750383	4026.0
micro avg	0.765323	0.765323	0.765323	4026.0
weighted avg	0.763290	0.764750	0.765323	4026.0

The result of Model Number:Dataset Number:Featuregroup Number:Rescale of 38 : 0 : 10 : 0

	f1-score	precision	recall	support
AFIB	0.682110	0.681294	0.683044	875.0
GSVT	0.695407	0.697799	0.693103	691.0
SB	0.736539	0.732002	0.741139	1553.0

SR 0.721551 0.728598 0.714718 907.0
macro avg 0.708902 0.709923 0.708001 4026.0
micro avg 0.714316 0.714316 0.714316 4026.0
weighted avg 0.714273 0.714344 0.714316 4026.0
The result of Model Number:Dataset Number:Featuregroup Number:Rescale of 38 : 0 : 10 : 1

	f1-score	precision	recall	support
AFIB	0.691159	0.698226	0.684292	875.0
GSVT	0.690951	0.680277	0.702047	691.0
SB	0.728861	0.724509	0.733272	1553.0
SR	0.709569	0.719067	0.700341	907.0
macro avg	0.705135	0.705519	0.704988	4026.0
micro avg	0.709849	0.709849	0.709849	4026.0
weighted avg	0.709814	0.709979	0.709849	4026.0

The result of Model Number:Dataset Number:Featuregroup Number:Rescale of 38 : 1 : 0 : 0

	f1-score	precision	recall	support
AFIB	0.877633	0.865294	0.890372	875.0
GSVT	0.896528	0.903255	0.889924	691.0
SB	0.953355	0.950553	0.956177	1553.0
SR	0.929872	0.942736	0.917369	907.0
macro avg	0.914347	0.915460	0.913461	4026.0
micro avg	0.921761	0.921761	0.921761	4026.0
weighted avg	0.921854	0.922144	0.921761	4026.0

The result of Model Number:Dataset Number:Featuregroup Number:Rescale of 38 : 1 : 0 : 1

	f1-score	precision	recall	support
AFIB	0.820478	0.822883	0.818149	875.0
GSVT	0.834054	0.823341	0.845157	691.0
SB	0.882707	0.880562	0.884867	1553.0
SR	0.860356	0.870566	0.850391	907.0
macro avg	0.849399	0.849338	0.849641	4026.0
micro avg	0.855784	0.855784	0.855784	4026.0
weighted avg	0.855796	0.855953	0.855784	4026.0

The result of Model Number:Dataset Number:Featuregroup Number:Rescale of 38 : 1 : 1 : 0

	f1-score	precision	recall	support
AFIB	0.783060	0.793649	0.773078	204.0
GSVT	0.812803	0.811484	0.814156	300.0
SB	0.830454	0.829348	0.831571	892.0
SR	0.821966	0.820913	0.823051	681.0
macro avg	0.812071	0.813848	0.810464	2077.0
micro avg	0.820517	0.820517	0.820517	2077.0
weighted avg	0.820467	0.820496	0.820517	2077.0

The result of Model Number:Dataset Number:Featuregroup Number:Rescale of 38 : 1 : 1 : 1

	f1-score	precision	recall	support
AFIB	0.713505	0.720455	0.706862	204.0
GSVT	0.730005	0.726653	0.733413	300.0
SB	0.740093	0.737547	0.742662	892.0
SR	0.733843	0.736690	0.731052	681.0
macro avg	0.729361	0.730336	0.728497	2077.0
micro avg	0.734003	0.734003	0.734003	2077.0
weighted avg	0.733975	0.734014	0.734003	2077.0

The result of Model Number:Dataset Number:Featuregroup Number:Rescale of 38 : 1 : 2 : 0

	f1-score	precision	recall	support
AFIB	0.747451	0.783687	0.714501	204.0
GSVT	0.778581	0.768407	0.789185	300.0
SB	0.799551	0.793471	0.805736	892.0
SR	0.777289	0.779611	0.775014	681.0
macro avg	0.775718	0.781294	0.771109	2077.0
micro avg	0.784312	0.784312	0.784312	2077.0
weighted avg	0.784106	0.784345	0.784312	2077.0

The result of Model Number:Dataset Number:Featuregroup Number:Rescale of 38 : 1 : 2 : 1

	f1-score	precision	recall	support
AFIB	0.694525	0.718814	0.672145	204.0
GSVT	0.723856	0.719444	0.728422	300.0
SB	0.738720	0.735685	0.741783	892.0
SR	0.731891	0.730823	0.732966	681.0
macro avg	0.722248	0.726191	0.718829	2077.0
micro avg	0.730122	0.730122	0.730122	2077.0
weighted avg	0.729993	0.730088	0.730122	2077.0

The result of Model Number:Dataset Number:Featuregroup Number:Rescale of 38 : 1 : 3 : 0

	f1-score	precision	recall	support
AFIB	0.737016	0.785037	0.694857	204.0
GSVT	0.769303	0.750394	0.789295	300.0
SB	0.791865	0.781693	0.802333	892.0
SR	0.771297	0.780507	0.762354	681.0
macro avg	0.767370	0.774408	0.762210	2077.0
micro avg	0.776785	0.776785	0.776785	2077.0
weighted avg	0.776475	0.777112	0.776785	2077.0

The result of Model Number:Dataset Number:Featuregroup Number:Rescale of 38 : 1 : 3 : 1

	f1-score	precision	recall	support
AFIB	0.721762	0.735157	0.709026	204.0

GSVT	0.737633	0.733765	0.741557	300.0
SB	0.749409	0.746745	0.752097	892.0
SR	0.741694	0.743031	0.740394	681.0
macro avg	0.737625	0.739675	0.735768	2077.0
micro avg	0.742507	0.742507	0.742507	2077.0
weighted avg	0.742463	0.742514	0.742507	2077.0

The result of Model Number:Dataset Number:Featuregroup Number:Rescale of 38 : 1 : 4 : 0

	f1-score	precision	recall	support
AFIB	0.770838	0.787007	0.755341	204.0
GSVT	0.788720	0.784053	0.793450	300.0
SB	0.807948	0.806056	0.809850	892.0
SR	0.801124	0.800894	0.801362	681.0
macro avg	0.792158	0.794502	0.790001	2077.0
micro avg	0.799344	0.799344	0.799344	2077.0
weighted avg	0.799288	0.799314	0.799344	2077.0

The result of Model Number:Dataset Number:Featuregroup Number:Rescale of 38 : 1 : 4 : 1

	f1-score	precision	recall	support
AFIB	0.840577	0.859603	0.822483	204.0
GSVT	0.862946	0.858721	0.867251	300.0
SB	0.880600	0.878057	0.883164	892.0
SR	0.874942	0.874564	0.875334	681.0
macro avg	0.864767	0.867736	0.862058	2077.0
micro avg	0.872338	0.872338	0.872338	2077.0
weighted avg	0.872264	0.872306	0.872338	2077.0

The result of Model Number:Dataset Number:Featuregroup Number:Rescale of 38 : 1 : 5 : 0

	f1-score	precision	recall	support
AFIB	0.857709	0.851322	0.864280	875.0
GSVT	0.873706	0.876825	0.870660	691.0
SB	0.927109	0.924912	0.929317	1553.0
SR	0.905951	0.914108	0.897972	907.0
macro avg	0.891119	0.891792	0.890557	4026.0
micro avg	0.898053	0.898053	0.898053	4026.0
weighted avg	0.898093	0.898231	0.898053	4026.0

The result of Model Number:Dataset Number:Featuregroup Number:Rescale of 38 : 1 : 5 : 1

	f1-score	precision	recall	support
AFIB	0.827579	0.836400	0.819077	204.0
GSVT	0.842798	0.838175	0.847566	300.0
SB	0.862253	0.860815	0.863700	892.0
SR	0.855065	0.856458	0.853688	681.0
macro avg	0.846924	0.847962	0.846008	2077.0

micro avg 0.853704 0.853704 0.853704 2077.0
weighted avg 0.853680 0.853718 0.853704 2077.0
The result of Model Number:Dataset Number:Featuregroup Number:Rescale of 38 : 1 : 6 : 0

	f1-score	precision	recall	support
AFIB	0.822335	0.815748	0.829126	875.0
GSVT	0.845045	0.846487	0.843707	691.0
SB	0.895882	0.893480	0.898304	1553.0
SR	0.871403	0.881548	0.861509	907.0
macro avg	0.858666	0.859316	0.858161	4026.0
micro avg	0.865609	0.865609	0.865609	4026.0
weighted avg	0.865657	0.865832	0.865609	4026.0

The result of Model Number:Dataset Number:Featuregroup Number:Rescale of 38 : 1 : 6 : 1

	f1-score	precision	recall	support
AFIB	0.691705	0.709220	0.675099	204.0
GSVT	0.706727	0.699042	0.714701	300.0
SB	0.726633	0.723007	0.730299	892.0
SR	0.715371	0.718580	0.712218	681.0
macro avg	0.710109	0.712462	0.708079	2077.0
micro avg	0.716696	0.716696	0.716696	2077.0
weighted avg	0.716635	0.716740	0.716696	2077.0

The result of Model Number:Dataset Number:Featuregroup Number:Rescale of 38 : 1 : 7 : 0

	f1-score	precision	recall	support
AFIB	0.768383	0.751876	0.785672	875.0
GSVT	0.795741	0.807281	0.784579	691.0
SB	0.847098	0.846937	0.847262	1553.0
SR	0.825867	0.835466	0.816490	907.0
macro avg	0.809272	0.810390	0.808501	4026.0
micro avg	0.816185	0.816185	0.816185	4026.0
weighted avg	0.816392	0.816886	0.816185	4026.0

The result of Model Number:Dataset Number:Featuregroup Number:Rescale of 38 : 1 : 7 : 1

	f1-score	precision	recall	support
AFIB	0.820341	0.829267	0.811844	204.0
GSVT	0.848102	0.844565	0.851806	300.0
SB	0.865481	0.864707	0.866257	892.0
SR	0.857302	0.857309	0.857302	681.0
macro avg	0.847806	0.848962	0.846802	2077.0
micro avg	0.855889	0.855889	0.855889	2077.0
weighted avg	0.855856	0.855891	0.855889	2077.0

The result of Model Number:Dataset Number:Featuregroup Number:Rescale of 38 : 1 : 8 : 0

	f1-score	precision	recall	support
AFIB	0.742265	0.757899	0.727381	204.0
GSVT	0.765343	0.758351	0.772483	300.0
SB	0.777843	0.776109	0.779587	892.0
SR	0.768599	0.769402	0.767805	681.0
macro avg	0.763512	0.765440	0.761814	2077.0
micro avg	0.769570	0.769570	0.769570	2077.0
weighted avg	0.769512	0.769556	0.769570	2077.0

The result of Model Number:Dataset Number:Featuregroup Number:Rescale of 38 : 1 : 8 : 1

	f1-score	precision	recall	support
AFIB	0.800979	0.806968	0.795108	204.0
GSVT	0.821445	0.817110	0.825827	300.0
SB	0.835308	0.834191	0.836430	892.0
SR	0.827951	0.829538	0.826371	681.0
macro avg	0.821420	0.821952	0.820934	2077.0
micro avg	0.827542	0.827542	0.827542	2077.0
weighted avg	0.827521	0.827524	0.827542	2077.0

The result of Model Number:Dataset Number:Featuregroup Number:Rescale of 38 : 1 : 9 : 0

	f1-score	precision	recall	support
AFIB	0.889542	0.897747	0.881701	204.0
GSVT	0.922063	0.922096	0.922055	300.0
SB	0.941374	0.939702	0.943060	892.0
SR	0.931383	0.931134	0.931655	681.0
macro avg	0.921090	0.922669	0.919618	2077.0
micro avg	0.930260	0.930260	0.930260	2077.0
weighted avg	0.930218	0.930229	0.930260	2077.0

The result of Model Number:Dataset Number:Featuregroup Number:Rescale of 38 : 1 : 9 : 1

	f1-score	precision	recall	support
AFIB	0.904014	0.912820	0.895597	204.0
GSVT	0.924919	0.920672	0.929237	300.0
SB	0.937701	0.934475	0.940956	892.0
SR	0.929782	0.933389	0.926247	681.0
macro avg	0.924104	0.925339	0.923009	2077.0
micro avg	0.929986	0.929986	0.929986	2077.0
weighted avg	0.929950	0.929998	0.929986	2077.0

The result of Model Number:Dataset Number:Featuregroup Number:Rescale of 38 : 1 : 10 : 0

	f1-score	precision	recall	support
AFIB	0.883652	0.888941	0.878471	204.0
GSVT	0.907192	0.904495	0.909911	300.0
SB	0.921084	0.919955	0.922218	892.0

SR	0.914369	0.915448	0.913300	681.0
macro avg	0.906574	0.907210	0.905975	2077.0
micro avg	0.913220	0.913220	0.913220	2077.0
weighted avg	0.913199	0.913198	0.913220	2077.0

The result of Model Number:Dataset Number:Featuregroup Number:Rescale of 38 : 1 : 10 : 1

	f1-score	precision	recall	support
AFIB	0.768606	0.784759	0.753135	875.0
GSVT	0.818476	0.821287	0.815723	691.0
SB	0.891741	0.887538	0.895988	1553.0
SR	0.844133	0.832891	0.855753	907.0
macro avg	0.830739	0.831619	0.830150	4026.0
micro avg	0.842100	0.842100	0.842100	4026.0
weighted avg	0.841679	0.841518	0.842100	4026.0

The result of Model Number:Dataset Number:Featuregroup Number:Rescale of 39 : 0 : 0 : 0

	f1-score	precision	recall	support
AFIB	0.647153	0.637409	0.662588	875.0
GSVT	0.617914	0.616116	0.623185	691.0
SB	0.659853	0.614878	0.717414	1553.0
SR	0.488794	0.653373	0.405025	907.0
macro avg	0.603429	0.630444	0.602053	4026.0
micro avg	0.618949	0.618949	0.618949	4026.0
weighted avg	0.611358	0.628659	0.618949	4026.0

The result of Model Number:Dataset Number:Featuregroup Number:Rescale of 39 : 0 : 0 : 1

	f1-score	precision	recall	support
AFIB	0.880479	0.890773	0.870589	204.0
GSVT	0.904700	0.900541	0.908924	300.0
SB	0.917509	0.914656	0.920387	892.0
SR	0.909608	0.912186	0.907083	681.0
macro avg	0.903074	0.904539	0.901746	2077.0
micro avg	0.909478	0.909478	0.909478	2077.0
weighted avg	0.909431	0.909462	0.909478	2077.0

The result of Model Number:Dataset Number:Featuregroup Number:Rescale of 39 : 0 : 1 : 0

	f1-score	precision	recall	support
AFIB	0.758937	0.793207	0.727752	204.0
GSVT	0.785333	0.776513	0.794499	300.0
SB	0.755461	0.722525	0.808355	892.0
SR	0.629403	0.634770	0.624233	681.0
macro avg	0.732284	0.731754	0.738710	2077.0
micro avg	0.738068	0.738068	0.738068	2077.0
weighted avg	0.718786	0.708492	0.738068	2077.0

The result of Model Number:Dataset Number:Featuregroup Number:Rescale of 39 : 0 : 1 : 1

	f1-score	precision	recall	support
AFIB	0.790129	0.803265	0.777597	204.0
GSVT	0.805280	0.799559	0.811144	300.0
SB	0.823958	0.819328	0.828660	892.0
SR	0.813461	0.818438	0.808570	681.0
macro avg	0.808207	0.810148	0.806493	2077.0
micro avg	0.814528	0.814528	0.814528	2077.0
weighted avg	0.814496	0.814603	0.814528	2077.0

The result of Model Number:Dataset Number:Featuregroup Number:Rescale of 39 : 0 : 2 : 0

	f1-score	precision	recall	support
AFIB	0.765780	0.763657	0.767971	875.0
GSVT	0.771840	0.775521	0.768270	691.0
SB	0.807981	0.805399	0.810582	1553.0
SR	0.795063	0.798903	0.791297	907.0
macro avg	0.785166	0.785870	0.784530	4026.0
micro avg	0.789714	0.789714	0.789714	4026.0
weighted avg	0.789696	0.789735	0.789714	4026.0

The result of Model Number:Dataset Number:Featuregroup Number:Rescale of 39 : 0 : 2 : 1

	f1-score	precision	recall	support
AFIB	0.733732	0.783465	0.690423	204.0
GSVT	0.691349	0.711861	0.672245	300.0
SB	0.722632	0.687942	0.761418	892.0
SR	0.641203	0.670314	0.615214	681.0
macro avg	0.697229	0.713396	0.684825	2077.0
micro avg	0.693628	0.693628	0.693628	2077.0
weighted avg	0.692505	0.694999	0.693628	2077.0

The result of Model Number:Dataset Number:Featuregroup Number:Rescale of 39 : 0 : 3 : 0

	f1-score	precision	recall	support
AFIB	0.827069	0.825738	0.828741	875.0
GSVT	0.846352	0.841941	0.850859	691.0
SB	0.884905	0.877408	0.892539	1553.0
SR	0.851125	0.870047	0.833295	907.0
macro avg	0.852363	0.853783	0.851359	4026.0
micro avg	0.858173	0.858173	0.858173	4026.0
weighted avg	0.858108	0.858432	0.858173	4026.0

The result of Model Number:Dataset Number:Featuregroup Number:Rescale of 39 : 0 : 3 : 1

	f1-score	precision	recall	support
AFIB	0.753403	0.742811	0.764339	875.0

GSVT 0.769624 0.775399 0.763955 691.0
SB 0.818407 0.816002 0.820830 1553.0
SR 0.798248 0.809291 0.787515 907.0
macro avg 0.784920 0.785876 0.784159 4026.0
micro avg 0.791285 0.791285 0.791285 4026.0
weighted avg 0.791365 0.791614 0.791285 4026.0
The result of Model Number:Dataset Number:Featuregroup Number:Rescale of 39 : 0 : 4 : 0

	f1-score	precision	recall	support
AFIB	0.841579	0.864678	0.819744	204.0
GSVT	0.860613	0.847392	0.874335	300.0
SB	0.879522	0.871014	0.888198	892.0
SR	0.857293	0.868005	0.846847	681.0
macro avg	0.859751	0.862772	0.857281	2077.0
micro avg	0.865914	0.865914	0.865914	2077.0
weighted avg	0.865775	0.865993	0.865914	2077.0

The result of Model Number:Dataset Number:Featuregroup Number:Rescale of 39 : 0 : 4 : 1

	f1-score	precision	recall	support
AFIB	0.688688	0.683210	0.694321	875.0
GSVT	0.661382	0.648806	0.674509	691.0
SB	0.694144	0.640755	0.757250	1553.0
SR	0.531521	0.679628	0.436515	907.0
macro avg	0.643934	0.663100	0.640649	4026.0
micro avg	0.657115	0.657115	0.657115	4026.0
weighted avg	0.650699	0.660121	0.657115	4026.0

The result of Model Number:Dataset Number:Featuregroup Number:Rescale of 39 : 0 : 5 : 0

	f1-score	precision	recall	support
AFIB	0.801057	0.791092	0.811366	875.0
GSVT	0.826137	0.836482	0.816127	691.0
SB	0.881373	0.880418	0.882336	1553.0
SR	0.857601	0.861999	0.853252	907.0
macro avg	0.841542	0.842498	0.840770	4026.0
micro avg	0.848996	0.848996	0.848996	4026.0
weighted avg	0.849081	0.849314	0.848996	4026.0

The result of Model Number:Dataset Number:Featuregroup Number:Rescale of 39 : 0 : 5 : 1

	f1-score	precision	recall	support
AFIB	0.572892	0.568574	0.650225	204.0
GSVT	0.694519	0.690407	0.698687	300.0
SB	0.568020	0.566834	0.569213	892.0
SR	0.702507	0.701383	0.703644	681.0
macro avg	0.634484	0.631799	0.655442	2077.0

micro avg	0.639948	0.639948	0.639948	2077.0
weighted avg	0.630865	0.628969	0.639948	2077.0

The result of Model Number:Dataset Number:Featuregroup Number:Rescale of 39 : 0 : 6 : 0

	f1-score	precision	recall	support
AFIB	0.735413	0.742030	0.729143	875.0
GSVT	0.750393	0.735178	0.766429	691.0
SB	0.795797	0.786944	0.804947	1553.0
SR	0.764219	0.786935	0.742956	907.0
macro avg	0.761455	0.762772	0.760869	4026.0
micro avg	0.767896	0.767896	0.767896	4026.0
weighted avg	0.767766	0.768296	0.767896	4026.0

The result of Model Number:Dataset Number:Featuregroup Number:Rescale of 39 : 0 : 6 : 1

	f1-score	precision	recall	support
AFIB	0.853092	0.847326	0.859052	875.0
GSVT	0.873904	0.874252	0.873669	691.0
SB	0.928345	0.925976	0.930736	1553.0
SR	0.903068	0.913157	0.893211	907.0
macro avg	0.889602	0.890178	0.889167	4026.0
micro avg	0.896908	0.896908	0.896908	4026.0
weighted avg	0.896951	0.897117	0.896908	4026.0

The result of Model Number:Dataset Number:Featuregroup Number:Rescale of 39 : 0 : 7 : 0

	f1-score	precision	recall	support
AFIB	0.846381	0.882178	0.813658	204.0
GSVT	0.779456	0.780820	0.789788	300.0
SB	0.839156	0.799179	0.884157	892.0
SR	0.741855	0.801606	0.691736	681.0
macro avg	0.801712	0.815946	0.794835	2077.0
micro avg	0.800512	0.800512	0.800512	2077.0
weighted avg	0.799340	0.805475	0.800512	2077.0

The result of Model Number:Dataset Number:Featuregroup Number:Rescale of 39 : 0 : 7 : 1

	f1-score	precision	recall	support
AFIB	0.683947	0.686256	0.681677	875.0
GSVT	0.682141	0.677336	0.687076	691.0
SB	0.717737	0.711076	0.724530	1553.0
SR	0.690451	0.703757	0.677673	907.0
macro avg	0.693569	0.694606	0.692739	4026.0
micro avg	0.698232	0.698232	0.698232	4026.0
weighted avg	0.698136	0.698242	0.698232	4026.0

The result of Model Number:Dataset Number:Featuregroup Number:Rescale of 39 : 0 : 8 : 0

	f1-score	precision	recall	support
AFIB	0.780714	0.771462	0.790255	875.0
GSVT	0.791826	0.799480	0.784394	691.0
SB	0.840391	0.837176	0.843637	1553.0
SR	0.820798	0.830268	0.811566	907.0
macro avg	0.808432	0.809597	0.807463	4026.0
micro avg	0.814642	0.814642	0.814642	4026.0
weighted avg	0.814671	0.814868	0.814642	4026.0

The result of Model Number:Dataset Number:Featuregroup Number:Rescale of 39 : 0 : 8 : 1

	f1-score	precision	recall	support
AFIB	0.730745	0.719823	0.742031	875.0
GSVT	0.744756	0.751125	0.738505	691.0
SB	0.795754	0.793014	0.798519	1553.0
SR	0.772463	0.783984	0.761302	907.0
macro avg	0.760930	0.761987	0.760089	4026.0
micro avg	0.767557	0.767557	0.767557	4026.0
weighted avg	0.767625	0.767883	0.767557	4026.0

The result of Model Number:Dataset Number:Featuregroup Number:Rescale of 39 : 0 : 9 : 0

	f1-score	precision	recall	support
AFIB	0.848171	0.857872	0.839015	204.0
GSVT	0.868024	0.862888	0.873237	300.0
SB	0.833287	0.802507	0.885244	892.0
SR	0.698997	0.702380	0.695664	681.0
macro avg	0.812120	0.806412	0.823290	2077.0
micro avg	0.816810	0.816810	0.816810	2077.0
weighted avg	0.795736	0.783837	0.816810	2077.0

The result of Model Number:Dataset Number:Featuregroup Number:Rescale of 39 : 0 : 9 : 1

	f1-score	precision	recall	support
AFIB	0.767543	0.784900	0.750960	875.0
GSVT	0.813770	0.817233	0.810384	691.0
SB	0.883585	0.879421	0.887794	1553.0
SR	0.837185	0.824343	0.850468	907.0
macro avg	0.825521	0.826474	0.824902	4026.0
micro avg	0.836360	0.836360	0.836360	4026.0
weighted avg	0.835929	0.835796	0.836360	4026.0

The result of Model Number:Dataset Number:Featuregroup Number:Rescale of 39 : 0 : 10 : 0

	f1-score	precision	recall	support
AFIB	0.841541	0.845409	0.837749	875.0
GSVT	0.806561	0.811421	0.801787	691.0
SB	0.865169	0.826604	0.907551	1553.0

SR 0.739909 0.805487 0.684353 907.0

macro avg 0.813295 0.822231 0.807860 4026.0

micro avg 0.823944 0.823944 0.823944 4026.0

weighted avg 0.821755 0.823328 0.823944 4026.0

The result of Model Number:Dataset Number:Featuregroup Number:Rescale of 39 : 0 : 10 : 1

	f1-score	precision	recall	support
AFIB	0.678098	0.714985	0.645004	204.0
GSVT	0.711878	0.701111	0.723009	300.0
SB	0.728795	0.724054	0.733609	892.0
SR	0.719803	0.720452	0.719173	681.0
macro avg	0.709644	0.715151	0.705199	2077.0
micro avg	0.718642	0.718642	0.718642	2077.0
weighted avg	0.718424	0.718669	0.718642	2077.0

The result of Model Number:Dataset Number:Featuregroup Number:Rescale of 39 : 1 : 0 : 0

	f1-score	precision	recall	support
AFIB	0.710983	0.712215	0.709766	875.0
GSVT	0.719604	0.711335	0.728126	691.0
SB	0.757768	0.748631	0.767134	1553.0
SR	0.721749	0.743618	0.701146	907.0
macro avg	0.727526	0.728950	0.726543	4026.0
micro avg	0.733105	0.733105	0.733105	4026.0
weighted avg	0.732935	0.733186	0.733105	4026.0

The result of Model Number:Dataset Number:Featuregroup Number:Rescale of 39 : 1 : 0 : 1

	f1-score	precision	recall	support
AFIB	0.796163	0.799068	0.793331	875.0
GSVT	0.809924	0.799377	0.820821	691.0
SB	0.853741	0.844338	0.863361	1553.0
SR	0.809102	0.831453	0.787965	907.0
macro avg	0.817232	0.818559	0.816370	4026.0
micro avg	0.823854	0.823854	0.823854	4026.0
weighted avg	0.823650	0.823879	0.823854	4026.0

The result of Model Number:Dataset Number:Featuregroup Number:Rescale of 39 : 1 : 1 : 0

	f1-score	precision	recall	support
AFIB	0.852108	0.845590	0.858839	875.0
GSVT	0.871714	0.876480	0.867050	691.0
SB	0.923074	0.922428	0.923728	1553.0
SR	0.898556	0.902914	0.894286	907.0
macro avg	0.886363	0.886853	0.885976	4026.0
micro avg	0.893265	0.893265	0.893265	4026.0
weighted avg	0.893312	0.893446	0.893265	4026.0

The result of Model Number:Dataset Number:Featuregroup Number:Rescale of 39 : 1 : 1 : 1

	f1-score	precision	recall	support
AFIB	0.754682	0.761433	0.748143	875.0
GSVT	0.769095	0.754471	0.784445	691.0
SB	0.816331	0.809259	0.823530	1553.0
SR	0.782802	0.800800	0.765629	907.0
macro avg	0.780727	0.781491	0.780437	4026.0
micro avg	0.787393	0.787393	0.787393	4026.0
weighted avg	0.787271	0.787555	0.787393	4026.0

The result of Model Number:Dataset Number:Featuregroup Number:Rescale of 39 : 1 : 2 : 0

	f1-score	precision	recall	support
AFIB	0.848507	0.850147	0.846938	875.0
GSVT	0.822060	0.818449	0.825752	691.0
SB	0.858955	0.806747	0.918411	1553.0
SR	0.691338	0.801060	0.608217	907.0
macro avg	0.805215	0.819101	0.799829	4026.0
micro avg	0.817091	0.817091	0.817091	4026.0
weighted avg	0.812590	0.816907	0.817091	4026.0

The result of Model Number:Dataset Number:Featuregroup Number:Rescale of 39 : 1 : 2 : 1

	f1-score	precision	recall	support
AFIB	0.780680	0.799238	0.763051	204.0
GSVT	0.800947	0.797856	0.804146	300.0
SB	0.820794	0.818963	0.822634	892.0
SR	0.810468	0.808837	0.812134	681.0
macro avg	0.803222	0.806223	0.800491	2077.0
micro avg	0.810669	0.810669	0.810669	2077.0
weighted avg	0.810602	0.810657	0.810669	2077.0

The result of Model Number:Dataset Number:Featuregroup Number:Rescale of 39 : 1 : 3 : 0

	f1-score	precision	recall	support
AFIB	0.769884	0.784032	0.756331	204.0
GSVT	0.782815	0.777689	0.788031	300.0
SB	0.796206	0.792318	0.800133	892.0
SR	0.787976	0.791252	0.784738	681.0
macro avg	0.784220	0.786323	0.782308	2077.0
micro avg	0.789035	0.789035	0.789035	2077.0
weighted avg	0.788988	0.789042	0.789035	2077.0

The result of Model Number:Dataset Number:Featuregroup Number:Rescale of 39 : 1 : 3 : 1

	f1-score	precision	recall	support
AFIB	0.686426	0.689561	0.683357	875.0

GSVT	0.697869	0.691967	0.703923	691.0
SB	0.737662	0.730129	0.745355	1553.0
SR	0.699591	0.714339	0.685477	907.0
macro avg	0.705387	0.706499	0.704528	4026.0
micro avg	0.711280	0.711280	0.711280	4026.0
weighted avg	0.711120	0.711205	0.711280	4026.0

The result of Model Number:Dataset Number:Featuregroup Number:Rescale of 39 : 1 : 4 : 0

	f1-score	precision	recall	support
AFIB	0.742018	0.761444	0.723897	204.0
GSVT	0.698188	0.718480	0.679391	300.0
SB	0.736738	0.710799	0.764655	892.0
SR	0.667763	0.689509	0.647480	681.0
macro avg	0.711177	0.720058	0.703856	2077.0
micro avg	0.709917	0.709917	0.709917	2077.0
weighted avg	0.709073	0.709902	0.709917	2077.0

The result of Model Number:Dataset Number:Featuregroup Number:Rescale of 39 : 1 : 4 : 1

	f1-score	precision	recall	support
AFIB	0.790193	0.806323	0.775044	204.0
GSVT	0.818886	0.811771	0.826276	300.0
SB	0.834630	0.833140	0.836127	892.0
SR	0.826861	0.827354	0.826374	681.0
macro avg	0.817643	0.819647	0.815955	2077.0
micro avg	0.825507	0.825507	0.825507	2077.0
weighted avg	0.825444	0.825523	0.825507	2077.0

The result of Model Number:Dataset Number:Featuregroup Number:Rescale of 39 : 1 : 5 : 0

	f1-score	precision	recall	support
AFIB	0.871742	0.874550	0.869033	875.0
GSVT	0.883998	0.869963	0.898573	691.0
SB	0.931435	0.923658	0.939351	1553.0
SR	0.884615	0.907082	0.863266	907.0
macro avg	0.892947	0.893813	0.892556	4026.0
micro avg	0.899929	0.899929	0.899929	4026.0
weighted avg	0.899772	0.900035	0.899929	4026.0

The result of Model Number:Dataset Number:Featuregroup Number:Rescale of 39 : 1 : 5 : 1

	f1-score	precision	recall	support
AFIB	0.730556	0.747246	0.714780	204.0
GSVT	0.763744	0.758990	0.768577	300.0
SB	0.780239	0.777819	0.782685	892.0
SR	0.771303	0.771660	0.770968	681.0
macro avg	0.761461	0.763929	0.759253	2077.0

micro avg	0.770136	0.770136	0.770136	2077.0
weighted avg	0.770047	0.770077	0.770136	2077.0

The result of Model Number:Dataset Number:Featuregroup Number:Rescale of 39 : 1 : 6 : 0

	f1-score	precision	recall	support
AFIB	0.812768	0.833038	0.793523	875.0
GSVT	0.864643	0.867958	0.861394	691.0
SB	0.936336	0.931802	0.940917	1553.0
SR	0.887230	0.872442	0.902586	907.0
macro avg	0.875244	0.876310	0.874605	4026.0
micro avg	0.886598	0.886598	0.886598	4026.0
weighted avg	0.886112	0.886006	0.886598	4026.0

The result of Model Number:Dataset Number:Featuregroup Number:Rescale of 39 : 1 : 6 : 1

	f1-score	precision	recall	support
AFIB	0.654756	0.659946	0.649664	875.0
GSVT	0.663057	0.660134	0.666029	691.0
SB	0.705083	0.702296	0.707898	1553.0
SR	0.687757	0.689700	0.685858	907.0
macro avg	0.677663	0.678019	0.677362	4026.0
micro avg	0.683090	0.683090	0.683090	4026.0
weighted avg	0.683029	0.683018	0.683090	4026.0

The result of Model Number:Dataset Number:Featuregroup Number:Rescale of 39 : 1 : 7 : 0

	f1-score	precision	recall	support
AFIB	0.752470	0.736390	0.769311	875.0
GSVT	0.779230	0.791146	0.767738	691.0
SB	0.830658	0.830767	0.830551	1553.0
SR	0.809258	0.817638	0.801050	907.0
macro avg	0.792904	0.793985	0.792163	4026.0
micro avg	0.799814	0.799814	0.799814	4026.0
weighted avg	0.800017	0.800498	0.799814	4026.0

The result of Model Number:Dataset Number:Featuregroup Number:Rescale of 39 : 1 : 7 : 1

	f1-score	precision	recall	support
AFIB	0.807978	0.802517	0.813623	875.0
GSVT	0.827690	0.828019	0.827467	691.0
SB	0.879252	0.877008	0.881516	1553.0
SR	0.855311	0.864867	0.845975	907.0
macro avg	0.842558	0.843103	0.842145	4026.0
micro avg	0.849477	0.849477	0.849477	4026.0
weighted avg	0.849518	0.849675	0.849477	4026.0

The result of Model Number:Dataset Number:Featuregroup Number:Rescale of 39 : 1 : 8 : 0

	f1-score	precision	recall	support
AFIB	0.904302	0.912643	0.896331	204.0
GSVT	0.937362	0.937396	0.937354	300.0
SB	0.956993	0.955294	0.958708	892.0
SR	0.946837	0.946584	0.947114	681.0
macro avg	0.936374	0.937979	0.934877	2077.0
micro avg	0.945695	0.945695	0.945695	2077.0
weighted avg	0.945653	0.945664	0.945695	2077.0

The result of Model Number:Dataset Number:Featuregroup Number:Rescale of 39 : 1 : 8 : 1

	f1-score	precision	recall	support
AFIB	0.886017	0.891811	0.880353	204.0
GSVT	0.908216	0.906124	0.910328	300.0
SB	0.924428	0.923094	0.925773	892.0
SR	0.917142	0.918100	0.916202	681.0
macro avg	0.908951	0.909782	0.908164	2077.0
micro avg	0.915943	0.915943	0.915943	2077.0
weighted avg	0.915925	0.915933	0.915943	2077.0

The result of Model Number:Dataset Number:Featuregroup Number:Rescale of 39 : 1 : 9 : 0

	f1-score	precision	recall	support
AFIB	0.728307	0.789106	0.677659	204.0
GSVT	0.781194	0.771371	0.791589	300.0
SB	0.801137	0.789913	0.812777	892.0
SR	0.779283	0.782934	0.776046	681.0
macro avg	0.772480	0.783331	0.764518	2077.0
micro avg	0.784402	0.784402	0.784402	2077.0
weighted avg	0.783938	0.784867	0.784402	2077.0

The result of Model Number:Dataset Number:Featuregroup Number:Rescale of 39 : 1 : 9 : 1

	f1-score	precision	recall	support
AFIB	0.713769	0.705132	0.722653	875.0
GSVT	0.723431	0.730848	0.716219	691.0
SB	0.768357	0.765857	0.770880	1553.0
SR	0.751551	0.759274	0.744010	907.0
macro avg	0.739277	0.740278	0.738441	4026.0
micro avg	0.744964	0.744964	0.744964	4026.0
weighted avg	0.744996	0.745167	0.744964	4026.0

The result of Model Number:Dataset Number:Featuregroup Number:Rescale of 39 : 1 : 10 : 0

	f1-score	precision	recall	support
AFIB	0.714961	0.716424	0.713581	204.0
GSVT	0.730686	0.727846	0.733600	300.0
SB	0.746907	0.745910	0.747910	892.0

SR	0.741421	0.743612	0.739244	681.0
macro avg	0.733493	0.733448	0.733584	2077.0
micro avg	0.739630	0.739630	0.739630	2077.0
weighted avg	0.739627	0.739651	0.739630	2077.0

The result of Model Number:Dataset Number:Featuregroup Number:Rescale of 39 : 1 : 10 : 1

	f1-score	precision	recall	support
AFIB	0.883705	0.923610	0.847394	204.0
GSVT	0.914441	0.904171	0.925113	300.0
SB	0.879658	0.841307	0.941247	892.0
SR	0.732876	0.739125	0.726856	681.0
macro avg	0.852670	0.852053	0.860153	2077.0
micro avg	0.859405	0.859405	0.859405	2077.0
weighted avg	0.836953	0.824967	0.859405	2077.0

The result of Model Number:Dataset Number:Featuregroup Number:Rescale of 40 : 0 : 0 : 0

	f1-score	precision	recall	support
AFIB	0.839842	0.835613	0.844151	875.0
GSVT	0.856201	0.860345	0.852109	691.0
SB	0.911906	0.909807	0.914017	1553.0
SR	0.889077	0.893834	0.884380	907.0
macro avg	0.874256	0.874900	0.873664	4026.0
micro avg	0.881530	0.881530	0.881530	4026.0
weighted avg	0.881540	0.881594	0.881530	4026.0

The result of Model Number:Dataset Number:Featuregroup Number:Rescale of 40 : 0 : 0 : 1

	f1-score	precision	recall	support
AFIB	0.773815	0.775873	0.771815	875.0
GSVT	0.788695	0.783139	0.794378	691.0
SB	0.833744	0.825243	0.842439	1553.0
SR	0.795632	0.812962	0.779054	907.0
macro avg	0.797972	0.799305	0.796921	4026.0
micro avg	0.804561	0.804561	0.804561	4026.0
weighted avg	0.804401	0.804520	0.804561	4026.0

The result of Model Number:Dataset Number:Featuregroup Number:Rescale of 40 : 0 : 1 : 0

	f1-score	precision	recall	support
AFIB	0.740431	0.764165	0.718349	204.0
GSVT	0.762583	0.749463	0.776283	300.0
SB	0.775774	0.768106	0.783600	892.0
SR	0.755032	0.764480	0.745831	681.0
macro avg	0.758455	0.761553	0.756016	2077.0
micro avg	0.763751	0.763751	0.763751	2077.0
weighted avg	0.763597	0.763837	0.763751	2077.0

The result of Model Number:Dataset Number:Featuregroup Number:Rescale of 40 : 0 : 1 : 1

	f1-score	precision	recall	support
AFIB	0.658142	0.656239	0.660116	875.0
GSVT	0.670340	0.669794	0.670932	691.0
SB	0.711998	0.709049	0.714977	1553.0
SR	0.690623	0.698210	0.683236	907.0
macro avg	0.682776	0.683323	0.682315	4026.0
micro avg	0.688343	0.688343	0.688343	4026.0
weighted avg	0.688327	0.688392	0.688343	4026.0

The result of Model Number:Dataset Number:Featuregroup Number:Rescale of 40 : 0 : 2 : 0

	f1-score	precision	recall	support
AFIB	0.828722	0.821313	0.836303	875.0
GSVT	0.842028	0.845360	0.838753	691.0
SB	0.897782	0.896116	0.899458	1553.0
SR	0.875032	0.883121	0.867122	907.0
macro avg	0.860891	0.861477	0.860409	4026.0
micro avg	0.868028	0.868028	0.868028	4026.0
weighted avg	0.868078	0.868219	0.868028	4026.0

The result of Model Number:Dataset Number:Featuregroup Number:Rescale of 40 : 0 : 2 : 1

	f1-score	precision	recall	support
AFIB	0.705620	0.708259	0.703300	875.0
GSVT	0.721503	0.728464	0.714819	691.0
SB	0.767183	0.759137	0.775462	1553.0
SR	0.740617	0.747135	0.734466	907.0
macro avg	0.733731	0.735749	0.732012	4026.0
micro avg	0.740134	0.740134	0.740134	4026.0
weighted avg	0.739978	0.740111	0.740134	4026.0

The result of Model Number:Dataset Number:Featuregroup Number:Rescale of 40 : 0 : 3 : 0

	f1-score	precision	recall	support
AFIB	0.913522	0.926997	0.900532	204.0
GSVT	0.935947	0.932274	0.939681	300.0
SB	0.956734	0.954816	0.958664	892.0
SR	0.949948	0.950091	0.949814	681.0
macro avg	0.939038	0.941045	0.937173	2077.0
micro avg	0.947311	0.947311	0.947311	2077.0
weighted avg	0.947263	0.947279	0.947311	2077.0

The result of Model Number:Dataset Number:Featuregroup Number:Rescale of 40 : 0 : 3 : 1

	f1-score	precision	recall	support
AFIB	0.721172	0.714411	0.728154	875.0

GSVT	0.683740	0.667305	0.734002	691.0
SB	0.790617	0.787714	0.793564	1553.0
SR	0.614928	0.619240	0.610683	907.0
macro avg	0.702614	0.697168	0.716601	4026.0
micro avg	0.727925	0.727925	0.727925	4026.0
weighted avg	0.717600	0.713161	0.727925	4026.0

The result of Model Number:Dataset Number:Featuregroup Number:Rescale of 40 : 0 : 4 : 0

	f1-score	precision	recall	support
AFIB	0.688422	0.717164	0.662020	875.0
GSVT	0.743736	0.739971	0.747630	691.0
SB	0.815529	0.810940	0.820193	1553.0
SR	0.763621	0.746664	0.781378	907.0
macro avg	0.752827	0.753685	0.752805	4026.0
micro avg	0.764617	0.764617	0.764617	4026.0
weighted avg	0.763888	0.763898	0.764617	4026.0

The result of Model Number:Dataset Number:Featuregroup Number:Rescale of 40 : 0 : 4 : 1

	f1-score	precision	recall	support
AFIB	0.658097	0.729182	0.599761	204.0
GSVT	0.762875	0.750468	0.775819	300.0
SB	0.813559	0.809855	0.817298	892.0
SR	0.793901	0.783799	0.804279	681.0
macro avg	0.757108	0.768326	0.749289	2077.0
micro avg	0.785672	0.785672	0.785672	2077.0
weighted avg	0.784524	0.784810	0.785672	2077.0

The result of Model Number:Dataset Number:Featuregroup Number:Rescale of 40 : 0 : 5 : 0

	f1-score	precision	recall	support
AFIB	0.800579	0.798116	0.803107	875.0
GSVT	0.780171	0.783997	0.776506	691.0
SB	0.820178	0.786142	0.857303	1553.0
SR	0.705832	0.770102	0.651518	907.0
macro avg	0.776690	0.784589	0.772108	4026.0
micro avg	0.785296	0.785296	0.785296	4026.0
weighted avg	0.783291	0.784763	0.785296	4026.0

The result of Model Number:Dataset Number:Featuregroup Number:Rescale of 40 : 0 : 5 : 1

	f1-score	precision	recall	support
AFIB	0.849167	0.852343	0.846375	875.0
GSVT	0.868281	0.876658	0.860237	691.0
SB	0.923254	0.913571	0.933217	1553.0
SR	0.891283	0.899127	0.883881	907.0
macro avg	0.882996	0.885425	0.880927	4026.0

micro avg   0.890702   0.890702 0.890702   4026.0
weighted avg 0.890514   0.890674 0.890702   4026.0
The result of Model Number:Dataset Number:Featuregroup Number:Rescale of 40 : 0 : 6 : 0

	f1-score	precision	recall	support
AFIB	0.727369	0.734454	0.720597	204.0
GSVT	0.744190	0.740772	0.747664	300.0
SB	0.754474	0.751878	0.757093	892.0
SR	0.748102	0.751004	0.745257	681.0
macro avg	0.743534	0.744527	0.742653	2077.0
micro avg	0.748266	0.748266	0.748266	2077.0
weighted avg	0.748237	0.748276	0.748266	2077.0

The result of Model Number:Dataset Number:Featuregroup Number:Rescale of 40 : 0 : 6 : 1

	f1-score	precision	recall	support
AFIB	0.690238	0.752751	0.638462	204.0
GSVT	0.801147	0.791993	0.810763	300.0
SB	0.849569	0.847394	0.851759	892.0
SR	0.828768	0.817545	0.840327	681.0
macro avg	0.792431	0.802421	0.785328	2077.0
micro avg	0.821140	0.821140	0.821140	2077.0
weighted avg	0.820106	0.820310	0.821140	2077.0

The result of Model Number:Dataset Number:Featuregroup Number:Rescale of 40 : 0 : 7 : 0

	f1-score	precision	recall	support
AFIB	0.743471	0.737867	0.843830	204.0
GSVT	0.901312	0.895976	0.906722	300.0
SB	0.737148	0.735609	0.738697	892.0
SR	0.911679	0.910220	0.913154	681.0
macro avg	0.823403	0.819918	0.850601	2077.0
micro avg	0.830493	0.830493	0.830493	2077.0
weighted avg	0.818705	0.816245	0.830493	2077.0

The result of Model Number:Dataset Number:Featuregroup Number:Rescale of 40 : 0 : 7 : 1

	f1-score	precision	recall	support
AFIB	0.870812	0.865937	0.875785	875.0
GSVT	0.877904	0.883083	0.872839	691.0
SB	0.924799	0.921905	0.927715	1553.0
SR	0.906491	0.912488	0.900593	907.0
macro avg	0.895002	0.895853	0.894233	4026.0
micro avg	0.900900	0.900900	0.900900	4026.0
weighted avg	0.900893	0.900956	0.900900	4026.0

The result of Model Number:Dataset Number:Featuregroup Number:Rescale of 40 : 0 : 8 : 0

	f1-score	precision	recall	support
AFIB	0.853553	0.866353	0.841369	204.0
GSVT	0.884376	0.882028	0.886742	300.0
SB	0.905325	0.904119	0.906543	892.0
SR	0.896470	0.895315	0.897659	681.0
macro avg	0.884931	0.886954	0.883078	2077.0
micro avg	0.894368	0.894368	0.894368	2077.0
weighted avg	0.894311	0.894332	0.894368	2077.0

The result of Model Number:Dataset Number:Featuregroup Number:Rescale of 40 : 0 : 8 : 1

	f1-score	precision	recall	support
AFIB	0.660846	0.656524	0.665282	875.0
GSVT	0.680487	0.686277	0.674885	691.0
SB	0.724470	0.722893	0.726057	1553.0
SR	0.706815	0.709653	0.704010	907.0
macro avg	0.693155	0.693837	0.692558	4026.0
micro avg	0.699098	0.699098	0.699098	4026.0
weighted avg	0.699116	0.699201	0.699098	4026.0

The result of Model Number:Dataset Number:Featuregroup Number:Rescale of 40 : 0 : 9 : 0

	f1-score	precision	recall	support
AFIB	0.730626	0.758449	0.704933	204.0
GSVT	0.753904	0.746306	0.761692	300.0
SB	0.768994	0.765166	0.772872	892.0
SR	0.759705	0.760183	0.759253	681.0
macro avg	0.753307	0.757526	0.749687	2077.0
micro avg	0.760119	0.760119	0.760119	2077.0
weighted avg	0.760000	0.760148	0.760119	2077.0

The result of Model Number:Dataset Number:Featuregroup Number:Rescale of 40 : 0 : 9 : 1

	f1-score	precision	recall	support
AFIB	0.646971	0.682164	0.615395	204.0
GSVT	0.679200	0.668927	0.689820	300.0
SB	0.695340	0.690817	0.699933	892.0
SR	0.686761	0.687380	0.686160	681.0
macro avg	0.677068	0.682322	0.672827	2077.0
micro avg	0.685653	0.685653	0.685653	2077.0
weighted avg	0.685445	0.685678	0.685653	2077.0

The result of Model Number:Dataset Number:Featuregroup Number:Rescale of 40 : 0 : 10 : 0

	f1-score	precision	recall	support
AFIB	0.763519	0.756443	0.770772	875.0
GSVT	0.777231	0.781452	0.773069	691.0
SB	0.823521	0.821622	0.825429	1553.0

SR 0.804334 0.811756 0.797071 907.0
macro avg 0.792151 0.792818 0.791585 4026.0
micro avg 0.798174 0.798174 0.798174 4026.0
weighted avg 0.798213 0.798339 0.798174 4026.0
The result of Model Number:Dataset Number:Featuregroup Number:Rescale of 40 : 0 : 10 : 1

	f1-score	precision	recall	support
AFIB	0.698011	0.712014	0.684688	204.0
GSVT	0.714188	0.706764	0.721807	300.0
SB	0.727397	0.724567	0.730251	892.0
SR	0.719753	0.722756	0.716791	681.0
macro avg	0.714837	0.716525	0.713384	2077.0
micro avg	0.720143	0.720143	0.720143	2077.0
weighted avg	0.720097	0.720169	0.720143	2077.0

The result of Model Number:Dataset Number:Featuregroup Number:Rescale of 40 : 1 : 0 : 0

	f1-score	precision	recall	support
AFIB	0.771059	0.782882	0.759715	204.0
GSVT	0.789342	0.782645	0.796188	300.0
SB	0.803073	0.800481	0.805684	892.0
SR	0.794821	0.797771	0.791902	681.0
macro avg	0.789574	0.790945	0.788372	2077.0
micro avg	0.795279	0.795279	0.795279	2077.0
weighted avg	0.795240	0.795288	0.795279	2077.0

The result of Model Number:Dataset Number:Featuregroup Number:Rescale of 40 : 1 : 0 : 1

	f1-score	precision	recall	support
AFIB	0.634492	0.625483	0.643835	875.0
GSVT	0.657502	0.665890	0.649422	691.0
SB	0.698744	0.697718	0.699779	1553.0
SR	0.681336	0.686447	0.676303	907.0
macro avg	0.668019	0.668885	0.667335	4026.0
micro avg	0.673688	0.673688	0.673688	4026.0
weighted avg	0.673779	0.674017	0.673688	4026.0

The result of Model Number:Dataset Number:Featuregroup Number:Rescale of 40 : 1 : 1 : 0

	f1-score	precision	recall	support
AFIB	0.683057	0.677832	0.688453	875.0
GSVT	0.698773	0.702594	0.695034	691.0
SB	0.739944	0.739426	0.740469	1553.0
SR	0.720290	0.723784	0.716867	907.0
macro avg	0.710516	0.710909	0.710206	4026.0
micro avg	0.716049	0.716049	0.716049	4026.0
weighted avg	0.716086	0.716194	0.716049	4026.0

The result of Model Number:Dataset Number:Featuregroup Number:Rescale of 40 : 1 : 1 : 1

	f1-score	precision	recall	support
AFIB	0.847379	0.887949	0.810554	204.0
GSVT	0.892519	0.882964	0.902315	300.0
SB	0.911476	0.906441	0.916580	892.0
SR	0.901180	0.900398	0.901978	681.0
macro avg	0.888138	0.894438	0.882857	2077.0
micro avg	0.899318	0.899318	0.899318	2077.0
weighted avg	0.899067	0.899253	0.899318	2077.0

The result of Model Number:Dataset Number:Featuregroup Number:Rescale of 40 : 1 : 2 : 0

	f1-score	precision	recall	support
AFIB	0.678263	0.662455	0.694953	875.0
GSVT	0.651108	0.658265	0.644149	691.0
SB	0.685187	0.640942	0.736010	1553.0
SR	0.548385	0.658505	0.470112	907.0
macro avg	0.640736	0.655042	0.636306	4026.0
micro avg	0.651417	0.651417	0.651417	4026.0
weighted avg	0.647014	0.652547	0.651417	4026.0

The result of Model Number:Dataset Number:Featuregroup Number:Rescale of 40 : 1 : 2 : 1

	f1-score	precision	recall	support
AFIB	0.850910	0.850143	0.851760	875.0
GSVT	0.865651	0.857725	0.873793	691.0
SB	0.908397	0.900411	0.916528	1553.0
SR	0.865631	0.887031	0.845315	907.0
macro avg	0.872647	0.873827	0.871849	4026.0
micro avg	0.879073	0.879073	0.879073	4026.0
weighted avg	0.878932	0.879145	0.879073	4026.0

The result of Model Number:Dataset Number:Featuregroup Number:Rescale of 40 : 1 : 3 : 0

	f1-score	precision	recall	support
AFIB	0.814813	0.818693	0.811006	875.0
GSVT	0.824879	0.809888	0.840519	691.0
SB	0.872784	0.869442	0.876155	1553.0
SR	0.846987	0.861484	0.832982	907.0
macro avg	0.839866	0.839877	0.840166	4026.0
micro avg	0.846153	0.846153	0.846153	4026.0
weighted avg	0.846151	0.846398	0.846153	4026.0

The result of Model Number:Dataset Number:Featuregroup Number:Rescale of 40 : 1 : 3 : 1

	f1-score	precision	recall	support
AFIB	0.815021	0.816596	0.813513	875.0

GSVT 0.789618 0.786149 0.793164 691.0
SB 0.825057 0.774909 0.882166 1553.0
SR 0.664055 0.769446 0.584214 907.0
macro avg 0.773437 0.786775 0.768264 4026.0
micro avg 0.784845 0.784845 0.784845 4026.0
weighted avg 0.780521 0.784668 0.784845 4026.0
The result of Model Number:Dataset Number:Featuregroup Number:Rescale of 40 : 1 : 4 : 0

	f1-score	precision	recall	support
AFIB	0.801370	0.819196	0.784475	204.0
GSVT	0.835513	0.830321	0.840803	300.0
SB	0.853650	0.851852	0.855466	892.0
SR	0.844558	0.843961	0.845183	681.0
macro avg	0.833773	0.836333	0.831482	2077.0
micro avg	0.843004	0.843004	0.843004	2077.0
weighted avg	0.842914	0.842947	0.843004	2077.0

The result of Model Number:Dataset Number:Featuregroup Number:Rescale of 40 : 1 : 4 : 1

	f1-score	precision	recall	support
AFIB	0.747457	0.767025	0.729204	204.0
GSVT	0.703306	0.723746	0.684371	300.0
SB	0.742138	0.716009	0.770260	892.0
SR	0.672657	0.694564	0.652226	681.0
macro avg	0.716390	0.725336	0.709015	2077.0
micro avg	0.715121	0.715121	0.715121	2077.0
weighted avg	0.714270	0.715106	0.715121	2077.0

The result of Model Number:Dataset Number:Featuregroup Number:Rescale of 40 : 1 : 5 : 0

	f1-score	precision	recall	support
AFIB	0.762114	0.767249	0.757160	875.0
GSVT	0.775715	0.764941	0.786940	691.0
SB	0.826609	0.820949	0.832355	1553.0
SR	0.793111	0.806811	0.779912	907.0
macro avg	0.789387	0.789987	0.789092	4026.0
micro avg	0.796403	0.796403	0.796403	4026.0
weighted avg	0.796310	0.796480	0.796403	4026.0

The result of Model Number:Dataset Number:Featuregroup Number:Rescale of 40 : 1 : 5 : 1

	f1-score	precision	recall	support
AFIB	0.769136	0.788612	0.750671	204.0
GSVT	0.785840	0.777294	0.794706	300.0
SB	0.807974	0.803942	0.812050	892.0
SR	0.795451	0.799020	0.791946	681.0
macro avg	0.789600	0.792217	0.787343	2077.0

micro avg 0.796924 0.796924 0.796924 2077.0
weighted avg 0.796856 0.796973 0.796924 2077.0
The result of Model Number:Dataset Number:Featuregroup Number:Rescale of 40 : 1 : 6 : 0

	f1-score	precision	recall	support
AFIB	0.669756	0.660971	0.678809	875.0
GSVT	0.680010	0.687569	0.672628	691.0
SB	0.721238	0.719536	0.722953	1553.0
SR	0.703181	0.709510	0.696974	907.0
macro avg	0.693546	0.694396	0.692841	4026.0
micro avg	0.698869	0.698869	0.698869	4026.0
weighted avg	0.698905	0.699062	0.698869	4026.0

The result of Model Number:Dataset Number:Featuregroup Number:Rescale of 40 : 1 : 6 : 1

	f1-score	precision	recall	support
AFIB	0.860015	0.924666	0.804711	204.0
GSVT	0.820170	0.846204	0.795816	300.0
SB	0.854502	0.812218	0.901437	892.0
SR	0.756426	0.788939	0.726564	681.0
macro avg	0.822778	0.843007	0.807132	2077.0
micro avg	0.819344	0.819344	0.819344	2077.0
weighted avg	0.817928	0.820539	0.819344	2077.0

The result of Model Number:Dataset Number:Featuregroup Number:Rescale of 40 : 1 : 7 : 0

	f1-score	precision	recall	support
AFIB	0.751747	0.757008	0.746585	204.0
GSVT	0.769237	0.765445	0.773079	300.0
SB	0.784526	0.783220	0.785845	892.0
SR	0.776718	0.778562	0.774892	681.0
macro avg	0.770557	0.771058	0.770100	2077.0
micro avg	0.776554	0.776554	0.776554	2077.0
weighted avg	0.776538	0.776551	0.776554	2077.0

The result of Model Number:Dataset Number:Featuregroup Number:Rescale of 40 : 1 : 7 : 1

	f1-score	precision	recall	support
AFIB	0.825322	0.881264	0.776607	204.0
GSVT	0.777649	0.800722	0.756161	300.0
SB	0.812837	0.773817	0.856465	892.0
SR	0.721243	0.753988	0.692010	681.0
macro avg	0.784263	0.802448	0.770311	2077.0
micro avg	0.780213	0.780213	0.780213	2077.0
weighted avg	0.778949	0.781755	0.780213	2077.0

The result of Model Number:Dataset Number:Featuregroup Number:Rescale of 40 : 1 : 8 : 0

	f1-score	precision	recall	support
AFIB	0.855761	0.858651	0.852922	875.0
GSVT	0.853501	0.847490	0.859676	691.0
SB	0.898040	0.889706	0.906539	1553.0
SR	0.863899	0.880548	0.847911	907.0
macro avg	0.867801	0.869098	0.866762	4026.0
micro avg	0.873635	0.873635	0.873635	4026.0
weighted avg	0.873516	0.873647	0.873635	4026.0

The result of Model Number:Dataset Number:Featuregroup Number:Rescale of 40 : 1 : 8 : 1

	f1-score	precision	recall	support
AFIB	0.815393	0.822914	0.808206	204.0
GSVT	0.845204	0.845234	0.845196	300.0
SB	0.862905	0.861372	0.864450	892.0
SR	0.853747	0.853518	0.853996	681.0
macro avg	0.844312	0.845760	0.842962	2077.0
micro avg	0.852717	0.852717	0.852717	2077.0
weighted avg	0.852679	0.852689	0.852717	2077.0

The result of Model Number:Dataset Number:Featuregroup Number:Rescale of 40 : 1 : 9 : 0

	f1-score	precision	recall	support
AFIB	0.811388	0.800745	0.822355	875.0
GSVT	0.823811	0.832968	0.814867	691.0
SB	0.873757	0.871695	0.875835	1553.0
SR	0.851881	0.859548	0.844362	907.0
macro avg	0.840209	0.841239	0.839355	4026.0
micro avg	0.846657	0.846657	0.846657	4026.0
weighted avg	0.846701	0.846892	0.846657	4026.0

The result of Model Number:Dataset Number:Featuregroup Number:Rescale of 40 : 1 : 9 : 1

	f1-score	precision	recall	support
AFIB	0.825514	0.851975	0.800894	204.0
GSVT	0.850211	0.835584	0.865485	300.0
SB	0.864919	0.856369	0.873644	892.0
SR	0.841793	0.852326	0.831534	681.0
macro avg	0.845609	0.849064	0.842889	2077.0
micro avg	0.851513	0.851513	0.851513	2077.0
weighted avg	0.851342	0.851610	0.851513	2077.0

The result of Model Number:Dataset Number:Featuregroup Number:Rescale of 40 : 1 : 10 : 0

	f1-score	precision	recall	support
AFIB	0.865075	0.912132	0.822855	204.0
GSVT	0.908168	0.894433	0.922369	300.0
SB	0.929750	0.923702	0.935892	892.0

SR 0.918279 0.919107 0.917475 681.0
macro avg 0.905318 0.912344 0.899648 2077.0
micro avg 0.916798 0.916798 0.916798 2077.0
weighted avg 0.916519 0.916831 0.916798 2077.0

The result of Model Number:Dataset Number:Featuregroup Number:Rescale of 40 : 1 : 10 : 1

	f1-score	precision	recall	support
AFIB	0.753633	0.774841	0.733709	204.0
GSVT	0.773116	0.767690	0.778766	300.0
SB	0.794377	0.791198	0.797582	892.0
SR	0.783818	0.784308	0.783352	681.0
macro avg	0.776236	0.779509	0.773352	2077.0
micro avg	0.783925	0.783925	0.783925	2077.0
weighted avg	0.783842	0.783937	0.783925	2077.0

The result of Model Number:Dataset Number:Featuregroup Number:Rescale of 41 : 0 : 0 : 0

	f1-score	precision	recall	support
AFIB	0.682816	0.751706	0.625893	204.0
GSVT	0.805240	0.794125	0.816799	300.0
SB	0.855008	0.852156	0.857884	892.0
SR	0.833113	0.821587	0.844978	681.0
macro avg	0.794044	0.804893	0.786388	2077.0
micro avg	0.824932	0.824932	0.824932	2077.0
weighted avg	0.823728	0.823885	0.824932	2077.0

The result of Model Number:Dataset Number:Featuregroup Number:Rescale of 41 : 0 : 0 : 1

	f1-score	precision	recall	support
AFIB	0.752081	0.738022	0.766767	875.0
GSVT	0.778426	0.790715	0.766610	691.0
SB	0.827823	0.826343	0.829314	1553.0
SR	0.806781	0.815328	0.798416	907.0
macro avg	0.791278	0.792602	0.790277	4026.0
micro avg	0.797997	0.797997	0.797997	4026.0
weighted avg	0.798143	0.798551	0.797997	4026.0

The result of Model Number:Dataset Number:Featuregroup Number:Rescale of 41 : 0 : 1 : 0

	f1-score	precision	recall	support
AFIB	0.706115	0.716379	0.696460	204.0
GSVT	0.721687	0.711924	0.731781	300.0
SB	0.732549	0.724915	0.740352	892.0
SR	0.718030	0.729912	0.706541	681.0
macro avg	0.719595	0.720782	0.718783	2077.0
micro avg	0.723717	0.723717	0.723717	2077.0
weighted avg	0.723624	0.723838	0.723717	2077.0

The result of Model Number:Dataset Number:Featuregroup Number:Rescale of 41 : 0 : 1 : 1

	f1-score	precision	recall	support
AFIB	0.609746	0.622560	0.597473	875.0
GSVT	0.649308	0.651539	0.647125	691.0
SB	0.707431	0.704097	0.710800	1553.0
SR	0.669663	0.660744	0.678881	907.0
macro avg	0.659037	0.659735	0.658570	4026.0
micro avg	0.668050	0.668050	0.668050	4026.0
weighted avg	0.667716	0.667588	0.668050	4026.0

The result of Model Number:Dataset Number:Featuregroup Number:Rescale of 41 : 0 : 2 : 0

	f1-score	precision	recall	support
AFIB	0.805223	0.822993	0.788263	204.0
GSVT	0.822820	0.809175	0.837031	300.0
SB	0.838631	0.832072	0.845304	892.0
SR	0.818278	0.828127	0.808663	681.0
macro avg	0.821238	0.823092	0.819815	2077.0
micro avg	0.826493	0.826493	0.826493	2077.0
weighted avg	0.826393	0.826579	0.826493	2077.0

The result of Model Number:Dataset Number:Featuregroup Number:Rescale of 41 : 0 : 2 : 1

	f1-score	precision	recall	support
AFIB	0.818100	0.808271	0.828201	875.0
GSVT	0.833835	0.840586	0.827197	691.0
SB	0.888803	0.885402	0.892236	1553.0
SR	0.867964	0.879062	0.857153	907.0
macro avg	0.852176	0.853330	0.851197	4026.0
micro avg	0.859252	0.859252	0.859252	4026.0
weighted avg	0.859308	0.859518	0.859252	4026.0

The result of Model Number:Dataset Number:Featuregroup Number:Rescale of 41 : 0 : 3 : 0

	f1-score	precision	recall	support
AFIB	0.731828	0.768393	0.698953	204.0
GSVT	0.753891	0.739920	0.768663	300.0
SB	0.771144	0.763370	0.779093	892.0
SR	0.749757	0.756454	0.743245	681.0
macro avg	0.751655	0.757034	0.747488	2077.0
micro avg	0.757962	0.757962	0.757962	2077.0
weighted avg	0.757778	0.758209	0.757962	2077.0

The result of Model Number:Dataset Number:Featuregroup Number:Rescale of 41 : 0 : 3 : 1

	f1-score	precision	recall	support
AFIB	0.853838	0.872859	0.835874	204.0

GSVT	0.864372	0.851713	0.877774	300.0
SB	0.883905	0.875121	0.892906	892.0
SR	0.867600	0.880061	0.855513	681.0
macro avg	0.867429	0.869938	0.865517	2077.0
micro avg	0.872858	0.872858	0.872858	2077.0
weighted avg	0.872785	0.873137	0.872858	2077.0

The result of Model Number:Dataset Number:Featuregroup Number:Rescale of 41 : 0 : 4 : 0

	f1-score	precision	recall	support
AFIB	0.755500	0.756768	0.774225	204.0
GSVT	0.748397	0.777440	0.722411	300.0
SB	0.793838	0.750303	0.843061	892.0
SR	0.688088	0.751589	0.636224	681.0
macro avg	0.746456	0.759025	0.743980	2077.0
micro avg	0.751056	0.751056	0.751056	2077.0
weighted avg	0.748836	0.755279	0.751056	2077.0

The result of Model Number:Dataset Number:Featuregroup Number:Rescale of 41 : 0 : 4 : 1

	f1-score	precision	recall	support
AFIB	0.734846	0.726178	0.743760	875.0
GSVT	0.747294	0.752023	0.742652	691.0
SB	0.796760	0.795688	0.797837	1553.0
SR	0.777186	0.784549	0.769986	907.0
macro avg	0.764021	0.764610	0.763559	4026.0
micro avg	0.770338	0.770338	0.770338	4026.0
weighted avg	0.770404	0.770577	0.770338	4026.0

The result of Model Number:Dataset Number:Featuregroup Number:Rescale of 41 : 0 : 5 : 0

	f1-score	precision	recall	support
AFIB	0.658011	0.644723	0.671912	875.0
GSVT	0.679828	0.690637	0.669498	691.0
SB	0.723346	0.723306	0.723393	1553.0
SR	0.705809	0.712240	0.699504	907.0
macro avg	0.691749	0.692726	0.691077	4026.0
micro avg	0.697572	0.697572	0.697572	4026.0
weighted avg	0.697726	0.698127	0.697572	4026.0

The result of Model Number:Dataset Number:Featuregroup Number:Rescale of 41 : 0 : 5 : 1

	f1-score	precision	recall	support
AFIB	0.710870	0.723911	0.698345	204.0
GSVT	0.720245	0.712977	0.727678	300.0
SB	0.737987	0.734145	0.741873	892.0
SR	0.727940	0.732501	0.723451	681.0
macro avg	0.724260	0.725883	0.722837	2077.0

micro avg 0.729507 0.729507 0.729507 2077.0
weighted avg 0.729467 0.729543 0.729507 2077.0
The result of Model Number:Dataset Number:Featuregroup Number:Rescale of 41 : 0 : 6 : 0

	f1-score	precision	recall	support
AFIB	0.826362	0.842283	0.811423	204.0
GSVT	0.841880	0.828201	0.856171	300.0
SB	0.855935	0.845727	0.866395	892.0
SR	0.840012	0.855762	0.824847	681.0
macro avg	0.841047	0.842993	0.839709	2077.0
micro avg	0.845896	0.845896	0.845896	2077.0
weighted avg	0.845780	0.846147	0.845896	2077.0

The result of Model Number:Dataset Number:Featuregroup Number:Rescale of 41 : 0 : 6 : 1

	f1-score	precision	recall	support
AFIB	0.813736	0.815193	0.812309	875.0
GSVT	0.779207	0.783300	0.775178	691.0
SB	0.824211	0.778872	0.875163	1553.0
SR	0.684511	0.770468	0.615870	907.0
macro avg	0.775416	0.786958	0.769630	4026.0
micro avg	0.785927	0.785927	0.785927	4026.0
weighted avg	0.782738	0.785633	0.785927	4026.0

The result of Model Number:Dataset Number:Featuregroup Number:Rescale of 41 : 0 : 7 : 0

	f1-score	precision	recall	support
AFIB	0.722149	0.729847	0.714730	204.0
GSVT	0.735430	0.731396	0.739591	300.0
SB	0.752406	0.751152	0.753669	892.0
SR	0.746134	0.747349	0.744932	681.0
macro avg	0.739030	0.739936	0.738231	2077.0
micro avg	0.744946	0.744946	0.744946	2077.0
weighted avg	0.744926	0.744959	0.744946	2077.0

The result of Model Number:Dataset Number:Featuregroup Number:Rescale of 41 : 0 : 7 : 1

	f1-score	precision	recall	support
AFIB	0.700963	0.694550	0.707526	875.0
GSVT	0.709944	0.716208	0.703895	691.0
SB	0.754648	0.750669	0.758680	1553.0
SR	0.737589	0.746477	0.728976	907.0
macro avg	0.725786	0.726976	0.724769	4026.0
micro avg	0.731467	0.731467	0.731467	4026.0
weighted avg	0.731464	0.731613	0.731467	4026.0

The result of Model Number:Dataset Number:Featuregroup Number:Rescale of 41 : 0 : 8 : 0

	f1-score	precision	recall	support
AFIB	0.589928	0.642634	0.622977	204.0
GSVT	0.801285	0.788187	0.815103	300.0
SB	0.682141	0.676946	0.687468	892.0
SR	0.834148	0.824710	0.843829	681.0
macro avg	0.726876	0.733119	0.742344	2077.0
micro avg	0.750837	0.750837	0.750837	2077.0
weighted avg	0.740133	0.738092	0.750837	2077.0

The result of Model Number:Dataset Number:Featuregroup Number:Rescale of 41 : 0 : 8 : 1

	f1-score	precision	recall	support
AFIB	0.794858	0.812134	0.778520	204.0
GSVT	0.827669	0.822787	0.832633	300.0
SB	0.845451	0.843576	0.847344	892.0
SR	0.836976	0.836503	0.837469	681.0
macro avg	0.826239	0.828750	0.823991	2077.0
micro avg	0.835222	0.835222	0.835222	2077.0
weighted avg	0.835135	0.835166	0.835222	2077.0

The result of Model Number:Dataset Number:Featuregroup Number:Rescale of 41 : 0 : 9 : 0

	f1-score	precision	recall	support
AFIB	0.737924	0.765283	0.712559	204.0
GSVT	0.760031	0.750528	0.769875	300.0
SB	0.779304	0.772193	0.786554	892.0
SR	0.758816	0.764705	0.753024	681.0
macro avg	0.759019	0.763177	0.755503	2077.0
micro avg	0.765883	0.765883	0.765883	2077.0
weighted avg	0.765738	0.765930	0.765883	2077.0

The result of Model Number:Dataset Number:Featuregroup Number:Rescale of 41 : 0 : 9 : 1

	f1-score	precision	recall	support
AFIB	0.879202	0.883217	0.875271	875.0
GSVT	0.893859	0.886299	0.901613	691.0
SB	0.944827	0.935179	0.954681	1553.0
SR	0.896064	0.914954	0.877986	907.0
macro avg	0.903488	0.904912	0.902388	4026.0
micro avg	0.911036	0.911036	0.911036	4026.0
weighted avg	0.910831	0.910940	0.911036	4026.0

The result of Model Number:Dataset Number:Featuregroup Number:Rescale of 41 : 0 : 10 : 0

	f1-score	precision	recall	support
AFIB	0.792116	0.794438	0.789868	875.0
GSVT	0.805223	0.794880	0.815942	691.0
SB	0.852194	0.850123	0.854279	1553.0

SR 0.830615 0.840473 0.820995 907.0
macro avg 0.820037 0.819979 0.820271 4026.0
micro avg 0.826202 0.826202 0.826202 4026.0
weighted avg 0.826214 0.826365 0.826202 4026.0
The result of Model Number:Dataset Number:Featuregroup Number:Rescale of 41 : 0 : 10 : 1

	f1-score	precision	recall	support
AFIB	0.717376	0.699019	0.736790	875.0
GSVT	0.678142	0.716577	0.643778	691.0
SB	0.719068	0.661716	0.787324	1553.0
SR	0.574414	0.697701	0.488259	907.0
macro avg	0.672250	0.693753	0.664038	4026.0
micro avg	0.684329	0.684329	0.684329	4026.0
weighted avg	0.679088	0.687346	0.684329	4026.0

The result of Model Number:Dataset Number:Featuregroup Number:Rescale of 41 : 1 : 0 : 0

	f1-score	precision	recall	support
AFIB	0.865877	0.897979	0.836113	204.0
GSVT	0.891817	0.880666	0.903367	300.0
SB	0.914431	0.906088	0.922938	892.0
SR	0.890390	0.897300	0.883595	681.0
macro avg	0.890629	0.895508	0.886503	2077.0
micro avg	0.898684	0.898684	0.898684	2077.0
weighted avg	0.898514	0.898738	0.898684	2077.0

The result of Model Number:Dataset Number:Featuregroup Number:Rescale of 41 : 1 : 0 : 1

	f1-score	precision	recall	support
AFIB	0.733455	0.714187	0.779371	875.0
GSVT	0.705577	0.667837	0.804343	691.0
SB	0.668571	0.664345	0.672870	1553.0
SR	0.649410	0.660600	0.638775	907.0
macro avg	0.689253	0.676742	0.723840	4026.0
micro avg	0.710901	0.710901	0.710901	4026.0
weighted avg	0.684707	0.674933	0.710901	4026.0

The result of Model Number:Dataset Number:Featuregroup Number:Rescale of 41 : 1 : 1 : 0

	f1-score	precision	recall	support
AFIB	0.702903	0.790353	0.633498	204.0
GSVT	0.833943	0.816136	0.852583	300.0
SB	0.890250	0.885712	0.894839	892.0
SR	0.870278	0.859257	0.881641	681.0
macro avg	0.824344	0.837864	0.815640	2077.0
micro avg	0.858740	0.858740	0.858740	2077.0
weighted avg	0.857168	0.857622	0.858740	2077.0

The result of Model Number:Dataset Number:Featuregroup Number:Rescale of 41 : 1 : 1 : 1

	f1-score	precision	recall	support
AFIB	0.768363	0.779652	0.757468	204.0
GSVT	0.787831	0.781133	0.794661	300.0
SB	0.801447	0.798241	0.804681	892.0
SR	0.793521	0.797416	0.789673	681.0
macro avg	0.787791	0.789110	0.786621	2077.0
micro avg	0.793676	0.793676	0.793676	2077.0
weighted avg	0.793632	0.793673	0.793676	2077.0

The result of Model Number:Dataset Number:Featuregroup Number:Rescale of 41 : 1 : 2 : 0

	f1-score	precision	recall	support
AFIB	0.848195	0.880494	0.818367	204.0
GSVT	0.875219	0.866397	0.884260	300.0
SB	0.892737	0.888293	0.897238	892.0
SR	0.881953	0.882508	0.881428	681.0
macro avg	0.874526	0.879423	0.870323	2077.0
micro avg	0.882433	0.882433	0.882433	2077.0
weighted avg	0.882296	0.882468	0.882433	2077.0

The result of Model Number:Dataset Number:Featuregroup Number:Rescale of 41 : 1 : 2 : 1

	f1-score	precision	recall	support
AFIB	0.849060	0.875211	0.824597	204.0
GSVT	0.870721	0.860062	0.881743	300.0
SB	0.894371	0.889057	0.899779	892.0
SR	0.879296	0.883632	0.875035	681.0
macro avg	0.873362	0.876990	0.870288	2077.0
micro avg	0.881677	0.881677	0.881677	2077.0
weighted avg	0.881562	0.881730	0.881677	2077.0

The result of Model Number:Dataset Number:Featuregroup Number:Rescale of 41 : 1 : 3 : 0

	f1-score	precision	recall	support
AFIB	0.800963	0.802046	0.800035	875.0
GSVT	0.813438	0.813020	0.813887	691.0
SB	0.849552	0.846512	0.852616	1553.0
SR	0.829208	0.833856	0.824665	907.0
macro avg	0.823290	0.823858	0.822801	4026.0
micro avg	0.828244	0.828244	0.828244	4026.0
weighted avg	0.828210	0.828248	0.828244	4026.0

The result of Model Number:Dataset Number:Featuregroup Number:Rescale of 41 : 1 : 3 : 1

	f1-score	precision	recall	support
AFIB	0.741310	0.757594	0.725989	204.0

GSVT	0.765201	0.759016	0.771592	300.0
SB	0.781238	0.780277	0.782202	892.0
SR	0.774642	0.773969	0.775322	681.0
macro avg	0.765598	0.767714	0.763776	2077.0
micro avg	0.772892	0.772892	0.772892	2077.0
weighted avg	0.772837	0.772910	0.772892	2077.0

The result of Model Number:Dataset Number:Featuregroup Number:Rescale of 41 : 1 : 4 : 0

	f1-score	precision	recall	support
AFIB	0.825103	0.818958	0.831423	875.0
GSVT	0.840491	0.843492	0.837562	691.0
SB	0.891864	0.889751	0.893988	1553.0
SR	0.871511	0.879357	0.863835	907.0
macro avg	0.857242	0.857890	0.856702	4026.0
micro avg	0.863913	0.863913	0.863913	4026.0
weighted avg	0.863952	0.864084	0.863913	4026.0

The result of Model Number:Dataset Number:Featuregroup Number:Rescale of 41 : 1 : 4 : 1

	f1-score	precision	recall	support
AFIB	0.856885	0.853401	0.860420	875.0
GSVT	0.869411	0.874666	0.864251	691.0
SB	0.916957	0.915841	0.918079	1553.0
SR	0.897828	0.899250	0.896436	907.0
macro avg	0.885270	0.885789	0.884797	4026.0
micro avg	0.891433	0.891433	0.891433	4026.0
weighted avg	0.891431	0.891466	0.891433	4026.0

The result of Model Number:Dataset Number:Featuregroup Number:Rescale of 41 : 1 : 5 : 0

	f1-score	precision	recall	support
AFIB	0.867001	0.912651	0.825933	204.0
GSVT	0.910538	0.902563	0.918696	300.0
SB	0.934165	0.928398	0.940021	892.0
SR	0.921420	0.919551	0.923318	681.0
macro avg	0.908281	0.915791	0.901992	2077.0
micro avg	0.920259	0.920259	0.920259	2077.0
weighted avg	0.919977	0.920219	0.920259	2077.0

The result of Model Number:Dataset Number:Featuregroup Number:Rescale of 41 : 1 : 5 : 1

	f1-score	precision	recall	support
AFIB	0.735748	0.733485	0.738071	875.0
GSVT	0.716992	0.720509	0.713625	691.0
SB	0.753760	0.722481	0.787878	1553.0
SR	0.648673	0.707739	0.598758	907.0
macro avg	0.713794	0.721053	0.709583	4026.0

micro avg 0.721703 0.721703 0.721703 4026.0
weighted avg 0.719860 0.721213 0.721703 4026.0
The result of Model Number:Dataset Number:Featuregroup Number:Rescale of 41 : 1 : 6 : 0

	f1-score	precision	recall	support
AFIB	0.167908	0.169671	0.166181	175.0
GSVT	0.168178	0.166392	0.170004	138.2
SB	0.177121	0.176442	0.177806	310.6
SR	0.173022	0.173889	0.172164	181.4
macro avg	0.171557	0.171599	0.171538	805.2
micro avg	0.172669	0.172669	0.172669	805.2
weighted avg	0.172660	0.172670	0.172669	805.2

The result of Model Number:Dataset Number:Featuregroup Number:Rescale of 41 : 1 : 6 : 1

	f1-score	precision	recall	support
AFIB	0.854374	0.916128	0.801008	204.0
GSVT	0.809270	0.813647	0.805270	300.0
SB	0.845298	0.794611	0.902911	892.0
SR	0.742235	0.801038	0.691570	681.0
macro avg	0.812794	0.831356	0.800190	2077.0
micro avg	0.809505	0.809505	0.809505	2077.0
weighted avg	0.807193	0.811403	0.809505	2077.0

The result of Model Number:Dataset Number:Featuregroup Number:Rescale of 41 : 1 : 7 : 0

	f1-score	precision	recall	support
AFIB	0.666965	0.658465	0.675710	875.0
GSVT	0.636830	0.641212	0.632524	691.0
SB	0.674289	0.635324	0.718360	1553.0
SR	0.549905	0.636226	0.484241	907.0
macro avg	0.631997	0.642807	0.627709	4026.0
micro avg	0.641614	0.641614	0.641614	4026.0
weighted avg	0.638246	0.641567	0.641614	4026.0

The result of Model Number:Dataset Number:Featuregroup Number:Rescale of 41 : 1 : 7 : 1

	f1-score	precision	recall	support
AFIB	0.640197	0.641434	0.639013	875.0
GSVT	0.620243	0.617518	0.623029	691.0
SB	0.648080	0.608689	0.692939	1553.0
SR	0.521613	0.604398	0.458899	907.0
macro avg	0.607533	0.618010	0.603470	4026.0
micro avg	0.616494	0.616494	0.616494	4026.0
weighted avg	0.613098	0.616355	0.616494	4026.0

The result of Model Number:Dataset Number:Featuregroup Number:Rescale of 41 : 1 : 8 : 0

	f1-score	precision	recall	support
AFIB	0.890312	0.907565	0.874104	204.0
GSVT	0.924837	0.917104	0.932872	300.0
SB	0.942093	0.940203	0.943994	892.0
SR	0.933110	0.934077	0.932150	681.0
macro avg	0.922588	0.924737	0.920780	2077.0
micro avg	0.931639	0.931639	0.931639	2077.0
weighted avg	0.931570	0.931652	0.931639	2077.0

The result of Model Number:Dataset Number:Featuregroup Number:Rescale of 41 : 1 : 8 : 1

	f1-score	precision	recall	support
AFIB	0.696131	0.710733	0.682136	204.0
GSVT	0.712280	0.708065	0.716552	300.0
SB	0.729644	0.727936	0.731362	892.0
SR	0.723481	0.723273	0.723696	681.0
macro avg	0.715384	0.717502	0.713436	2077.0
micro avg	0.721874	0.721874	0.721874	2077.0
weighted avg	0.721824	0.721847	0.721874	2077.0

The result of Model Number:Dataset Number:Featuregroup Number:Rescale of 41 : 1 : 9 : 0

	f1-score	precision	recall	support
AFIB	0.829443	0.817781	0.841482	875.0
GSVT	0.847301	0.853658	0.841060	691.0
SB	0.901008	0.898360	0.903675	1553.0
SR	0.878814	0.890972	0.866997	907.0
macro avg	0.864141	0.865193	0.863303	4026.0
micro avg	0.871148	0.871148	0.871148	4026.0
weighted avg	0.871236	0.871510	0.871148	4026.0

The result of Model Number:Dataset Number:Featuregroup Number:Rescale of 41 : 1 : 9 : 1

	f1-score	precision	recall	support
AFIB	0.741707	0.792350	0.697465	204.0
GSVT	0.784058	0.768080	0.800783	300.0
SB	0.803505	0.796461	0.810705	892.0
SR	0.783685	0.786295	0.781163	681.0
macro avg	0.778239	0.785797	0.772529	2077.0
micro avg	0.788463	0.788463	0.788463	2077.0
weighted avg	0.788128	0.788625	0.788463	2077.0

The result of Model Number:Dataset Number:Featuregroup Number:Rescale of 41 : 1 : 10 : 0

	f1-score	precision	recall	support
AFIB	0.805672	0.784508	0.856110	875.0
GSVT	0.775049	0.733594	0.883540	691.0
SB	0.734399	0.729758	0.739122	1553.0

SR 0.713353 0.725644 0.701670 907.0
macro avg 0.757119 0.743376 0.795111 4026.0
micro avg 0.780898 0.780898 0.780898 4026.0
weighted avg 0.752125 0.741389 0.780898 4026.0
The result of Model Number:Dataset Number:Featuregroup Number:Rescale of 41 : 1 : 10 : 1

	f1-score	precision	recall	support
AFIB	0.884371	0.921774	0.850179	204.0
GSVT	0.814442	0.815867	0.825238	300.0
SB	0.876821	0.835050	0.923842	892.0
SR	0.775153	0.837586	0.722785	681.0
macro avg	0.837697	0.852570	0.830511	2077.0
micro avg	0.836443	0.836443	0.836443	2077.0
weighted avg	0.835218	0.841629	0.836443	2077.0

The result of Model Number:Dataset Number:Featuregroup Number:Rescale of 42 : 0 : 0 : 0

	f1-score	precision	recall	support
AFIB	0.686005	0.691216	0.680931	204.0
GSVT	0.700050	0.693212	0.707039	300.0
SB	0.716379	0.714548	0.718226	892.0
SR	0.708780	0.712784	0.704829	681.0
macro avg	0.702804	0.702940	0.702756	2077.0
micro avg	0.708555	0.708555	0.708555	2077.0
weighted avg	0.708546	0.708596	0.708555	2077.0

The result of Model Number:Dataset Number:Featuregroup Number:Rescale of 42 : 0 : 0 : 1

	f1-score	precision	recall	support
AFIB	0.671402	0.681306	0.661855	204.0
GSVT	0.687884	0.685185	0.690628	300.0
SB	0.703162	0.701752	0.704580	892.0
SR	0.698174	0.698280	0.698075	681.0
macro avg	0.690156	0.691631	0.688785	2077.0
micro avg	0.696236	0.696236	0.696236	2077.0
weighted avg	0.696201	0.696212	0.696236	2077.0

The result of Model Number:Dataset Number:Featuregroup Number:Rescale of 42 : 0 : 1 : 0

	f1-score	precision	recall	support
AFIB	0.695865	0.703271	0.688997	204.0
GSVT	0.716315	0.711542	0.721362	300.0
SB	0.731452	0.730798	0.732108	892.0
SR	0.725284	0.726252	0.724324	681.0
macro avg	0.717229	0.717966	0.716698	2077.0
micro avg	0.723769	0.723769	0.723769	2077.0
weighted avg	0.723748	0.723823	0.723769	2077.0

The result of Model Number:Dataset Number:Featuregroup Number:Rescale of 42 : 0 : 1 : 1

	f1-score	precision	recall	support
AFIB	0.798263	0.788673	0.808119	875.0
GSVT	0.813617	0.820204	0.807139	691.0
SB	0.867252	0.863933	0.870601	1553.0
SR	0.846918	0.857747	0.836369	907.0
macro avg	0.831512	0.832639	0.830557	4026.0
micro avg	0.838417	0.838417	0.838417	4026.0
weighted avg	0.838471	0.838677	0.838417	4026.0

The result of Model Number:Dataset Number:Featuregroup Number:Rescale of 42 : 0 : 2 : 0

	f1-score	precision	recall	support
AFIB	0.835980	0.841994	0.830054	875.0
GSVT	0.834648	0.829651	0.839720	691.0
SB	0.880432	0.870677	0.890410	1553.0
SR	0.844842	0.859915	0.830317	907.0
macro avg	0.848976	0.850559	0.847625	4026.0
micro avg	0.855054	0.855054	0.855054	4026.0
weighted avg	0.854895	0.854977	0.855054	4026.0

The result of Model Number:Dataset Number:Featuregroup Number:Rescale of 42 : 0 : 2 : 1

	f1-score	precision	recall	support
AFIB	0.765921	0.846907	0.699469	204.0
GSVT	0.881292	0.871093	0.891901	300.0
SB	0.931771	0.927939	0.935636	892.0
SR	0.911761	0.898256	0.925688	681.0
macro avg	0.872686	0.886049	0.863174	2077.0
micro avg	0.902861	0.902861	0.902861	2077.0
weighted avg	0.901630	0.902037	0.902861	2077.0

The result of Model Number:Dataset Number:Featuregroup Number:Rescale of 42 : 0 : 3 : 0

	f1-score	precision	recall	support
AFIB	0.772192	0.778057	0.766481	204.0
GSVT	0.788001	0.780305	0.795868	300.0
SB	0.806382	0.804321	0.808461	892.0
SR	0.797829	0.802335	0.793382	681.0
macro avg	0.791101	0.791255	0.791048	2077.0
micro avg	0.797575	0.797575	0.797575	2077.0
weighted avg	0.797565	0.797622	0.797575	2077.0

The result of Model Number:Dataset Number:Featuregroup Number:Rescale of 42 : 0 : 3 : 1

	f1-score	precision	recall	support
AFIB	0.739849	0.754691	0.725727	204.0

GSVT	0.756995	0.749127	0.765071	300.0
SB	0.770996	0.767996	0.774020	892.0
SR	0.762894	0.766077	0.759755	681.0
macro avg	0.757683	0.759473	0.756143	2077.0
micro avg	0.763307	0.763307	0.763307	2077.0
weighted avg	0.763258	0.763335	0.763307	2077.0

The result of Model Number:Dataset Number:Featuregroup Number:Rescale of 42 : 0 : 4 : 0

	f1-score	precision	recall	support
AFIB	0.805339	0.799896	0.810966	875.0
GSVT	0.824987	0.825315	0.824765	691.0
SB	0.876380	0.874144	0.878637	1553.0
SR	0.852517	0.862042	0.843212	907.0
macro avg	0.839806	0.840349	0.839395	4026.0
micro avg	0.846703	0.846703	0.846703	4026.0
weighted avg	0.846743	0.846900	0.846703	4026.0

The result of Model Number:Dataset Number:Featuregroup Number:Rescale of 42 : 0 : 4 : 1

	f1-score	precision	recall	support
AFIB	0.846952	0.875078	0.820899	204.0
GSVT	0.879094	0.872041	0.886394	300.0
SB	0.898517	0.894825	0.902242	892.0
SR	0.890125	0.889994	0.890256	681.0
macro avg	0.878672	0.882985	0.874948	2077.0
micro avg	0.888034	0.888034	0.888034	2077.0
weighted avg	0.887895	0.888011	0.888034	2077.0

The result of Model Number:Dataset Number:Featuregroup Number:Rescale of 42 : 0 : 5 : 0

	f1-score	precision	recall	support
AFIB	0.863967	0.875167	0.853280	204.0
GSVT	0.897397	0.896817	0.898005	300.0
SB	0.916306	0.914371	0.918255	892.0
SR	0.907090	0.906575	0.907625	681.0
macro avg	0.896190	0.898232	0.894291	2077.0
micro avg	0.905463	0.905463	0.905463	2077.0
weighted avg	0.905412	0.905429	0.905463	2077.0

The result of Model Number:Dataset Number:Featuregroup Number:Rescale of 42 : 0 : 5 : 1

	f1-score	precision	recall	support
AFIB	0.669640	0.738470	0.612957	204.0
GSVT	0.796852	0.786083	0.807998	300.0
SB	0.848590	0.845196	0.852014	892.0
SR	0.825558	0.814398	0.837064	681.0
macro avg	0.785160	0.796037	0.777508	2077.0

micro avg 0.817275 0.817275 0.817275 2077.0
weighted avg 0.815989 0.816078 0.817275 2077.0
The result of Model Number:Dataset Number:Featuregroup Number:Rescale of 42 : 0 : 6 : 0

	f1-score	precision	recall	support
AFIB	0.824013	0.850777	0.799043	204.0
GSVT	0.846763	0.835966	0.858012	300.0
SB	0.870652	0.867369	0.873969	892.0
SR	0.858475	0.860029	0.856959	681.0
macro avg	0.849975	0.853535	0.846996	2077.0
micro avg	0.858728	0.858728	0.858728	2077.0
weighted avg	0.858628	0.858797	0.858728	2077.0

The result of Model Number:Dataset Number:Featuregroup Number:Rescale of 42 : 0 : 6 : 1

	f1-score	precision	recall	support
AFIB	0.797299	0.795285	0.799448	875.0
GSVT	0.812854	0.808385	0.817417	691.0
SB	0.851123	0.845991	0.856328	1553.0
SR	0.826028	0.840937	0.811654	907.0
macro avg	0.821826	0.822650	0.821212	4026.0
micro avg	0.827223	0.827223	0.827223	4026.0
weighted avg	0.827203	0.827378	0.827223	4026.0

The result of Model Number:Dataset Number:Featuregroup Number:Rescale of 42 : 0 : 7 : 0

	f1-score	precision	recall	support
AFIB	0.883232	0.901262	0.866299	204.0
GSVT	0.915303	0.907351	0.923563	300.0
SB	0.932901	0.931236	0.934574	892.0
SR	0.924218	0.924769	0.923673	681.0
macro avg	0.913913	0.916154	0.912027	2077.0
micro avg	0.922704	0.922704	0.922704	2077.0
weighted avg	0.922634	0.922721	0.922704	2077.0

The result of Model Number:Dataset Number:Featuregroup Number:Rescale of 42 : 0 : 7 : 1

	f1-score	precision	recall	support
AFIB	0.792388	0.777576	0.807861	875.0
GSVT	0.820145	0.833093	0.807696	691.0
SB	0.872189	0.870630	0.873761	1553.0
SR	0.850020	0.859025	0.841206	907.0
macro avg	0.833685	0.835081	0.832631	4026.0
micro avg	0.840765	0.840765	0.840765	4026.0
weighted avg	0.840918	0.841349	0.840765	4026.0

The result of Model Number:Dataset Number:Featuregroup Number:Rescale of 42 : 0 : 8 : 0

	f1-score	precision	recall	support
AFIB	0.707579	0.708946	0.706270	875.0
GSVT	0.685524	0.682513	0.688604	691.0
SB	0.716292	0.672755	0.765872	1553.0
SR	0.576514	0.668012	0.507199	907.0
macro avg	0.671477	0.683057	0.666986	4026.0
micro avg	0.681381	0.681381	0.681381	4026.0
weighted avg	0.677627	0.681227	0.681381	4026.0

The result of Model Number:Dataset Number:Featuregroup Number:Rescale of 42 : 0 : 8 : 1

	f1-score	precision	recall	support
AFIB	0.628942	0.676222	0.588498	204.0
GSVT	0.599803	0.618842	0.581992	300.0
SB	0.624910	0.593987	0.659235	892.0
SR	0.553186	0.576963	0.531347	681.0
macro avg	0.601710	0.616504	0.590268	2077.0
micro avg	0.599199	0.599199	0.599199	2077.0
weighted avg	0.598163	0.600072	0.599199	2077.0

The result of Model Number:Dataset Number:Featuregroup Number:Rescale of 42 : 0 : 9 : 0

	f1-score	precision	recall	support
AFIB	0.813845	0.816467	0.811316	875.0
GSVT	0.825287	0.812185	0.838894	691.0
SB	0.869574	0.862314	0.876964	1553.0
SR	0.825863	0.846838	0.805933	907.0
macro avg	0.833643	0.834451	0.833277	4026.0
micro avg	0.840160	0.840160	0.840160	4026.0
weighted avg	0.840014	0.840259	0.840160	4026.0

The result of Model Number:Dataset Number:Featuregroup Number:Rescale of 42 : 0 : 9 : 1

	f1-score	precision	recall	support
AFIB	0.823580	0.859724	0.790560	204.0
GSVT	0.766699	0.772220	0.761572	300.0
SB	0.795128	0.755199	0.839518	892.0
SR	0.705403	0.751977	0.664411	681.0
macro avg	0.772703	0.784780	0.764015	2077.0
micro avg	0.766037	0.766037	0.766037	2077.0
weighted avg	0.764397	0.766867	0.766037	2077.0

The result of Model Number:Dataset Number:Featuregroup Number:Rescale of 42 : 0 : 10 : 0

	f1-score	precision	recall	support
AFIB	0.791432	0.784433	0.798591	875.0
GSVT	0.719549	0.696829	0.800513	691.0
SB	0.648713	0.622854	0.689151	1553.0

SR 0.668592 0.675511 0.661858 907.0
macro avg 0.707072 0.694906 0.737528 4026.0
micro avg 0.725901 0.725901 0.725901 4026.0
weighted avg 0.696368 0.682530 0.725901 4026.0
The result of Model Number:Dataset Number:Featuregroup Number:Rescale of 42 : 0 : 10 : 1

	f1-score	precision	recall	support
AFIB	0.631220	0.625031	0.674900	204.0
GSVT	0.565985	0.559693	0.572563	300.0
SB	0.727326	0.722030	0.732706	892.0
SR	0.715128	0.720081	0.710301	681.0
macro avg	0.659915	0.656709	0.672618	2077.0
micro avg	0.696551	0.696551	0.696551	2077.0
weighted avg	0.690584	0.688416	0.696551	2077.0

The result of Model Number:Dataset Number:Featuregroup Number:Rescale of 42 : 1 : 0 : 0

	f1-score	precision	recall	support
AFIB	0.849816	0.885758	0.816960	204.0
GSVT	0.782619	0.783989	0.792994	300.0
SB	0.842562	0.802423	0.887745	892.0
SR	0.744866	0.804860	0.694544	681.0
macro avg	0.804966	0.819257	0.798061	2077.0
micro avg	0.803761	0.803761	0.803761	2077.0
weighted avg	0.802584	0.808744	0.803761	2077.0

The result of Model Number:Dataset Number:Featuregroup Number:Rescale of 42 : 1 : 0 : 1

	f1-score	precision	recall	support
AFIB	0.735388	0.735343	0.735478	875.0
GSVT	0.736979	0.732995	0.741020	691.0
SB	0.772188	0.767050	0.777405	1553.0
SR	0.751684	0.764016	0.739770	907.0
macro avg	0.749060	0.749851	0.748418	4026.0
micro avg	0.753569	0.753569	0.753569	4026.0
weighted avg	0.753528	0.753630	0.753569	4026.0

The result of Model Number:Dataset Number:Featuregroup Number:Rescale of 42 : 1 : 1 : 0

	f1-score	precision	recall	support
AFIB	0.747711	0.762060	0.733934	875.0
GSVT	0.797036	0.798252	0.795873	691.0
SB	0.867870	0.864056	0.871724	1553.0
SR	0.820110	0.811195	0.829308	907.0
macro avg	0.808182	0.808891	0.807710	4026.0
micro avg	0.819203	0.819203	0.819203	4026.0
weighted avg	0.818838	0.818685	0.819203	4026.0

The result of Model Number:Dataset Number:Featuregroup Number:Rescale of 42 : 1 : 1 : 1

	f1-score	precision	recall	support
AFIB	0.676308	0.685453	0.667686	204.0
GSVT	0.701996	0.700857	0.703164	300.0
SB	0.717241	0.716286	0.718205	892.0
SR	0.709910	0.709000	0.710847	681.0
macro avg	0.701364	0.702899	0.699976	2077.0
micro avg	0.708658	0.708658	0.708658	2077.0
weighted avg	0.708615	0.708640	0.708658	2077.0

The result of Model Number:Dataset Number:Featuregroup Number:Rescale of 42 : 1 : 2 : 0

	f1-score	precision	recall	support
AFIB	0.727664	0.732899	0.722505	875.0
GSVT	0.726505	0.722155	0.730919	691.0
SB	0.766356	0.757865	0.775041	1553.0
SR	0.735378	0.748498	0.722734	907.0
macro avg	0.738976	0.740354	0.737800	4026.0
micro avg	0.744266	0.744266	0.744266	4026.0
weighted avg	0.744128	0.744199	0.744266	4026.0

The result of Model Number:Dataset Number:Featuregroup Number:Rescale of 42 : 1 : 2 : 1

	f1-score	precision	recall	support
AFIB	0.803955	0.818710	0.790110	204.0
GSVT	0.834474	0.827765	0.841446	300.0
SB	0.849954	0.848437	0.851479	892.0
SR	0.841538	0.842287	0.840795	681.0
macro avg	0.832480	0.834300	0.830958	2077.0
micro avg	0.840499	0.840499	0.840499	2077.0
weighted avg	0.840441	0.840515	0.840499	2077.0

The result of Model Number:Dataset Number:Featuregroup Number:Rescale of 42 : 1 : 3 : 0

	f1-score	precision	recall	support
AFIB	0.766910	0.747284	0.787664	875.0
GSVT	0.724966	0.766055	0.688229	691.0
SB	0.768718	0.707406	0.841687	1553.0
SR	0.614076	0.745876	0.521972	907.0
macro avg	0.718668	0.741655	0.709888	4026.0
micro avg	0.731580	0.731580	0.731580	4026.0
weighted avg	0.725977	0.734806	0.731580	4026.0

The result of Model Number:Dataset Number:Featuregroup Number:Rescale of 42 : 1 : 3 : 1

	f1-score	precision	recall	support
AFIB	0.750275	0.765258	0.735982	204.0

GSVT	0.758701	0.749221	0.768538	300.0
SB	0.773186	0.765793	0.780724	892.0
SR	0.759144	0.769103	0.749461	681.0
macro avg	0.760326	0.762344	0.758676	2077.0
micro avg	0.764319	0.764319	0.764319	2077.0
weighted avg	0.764239	0.764432	0.764319	2077.0

The result of Model Number:Dataset Number:Featuregroup Number:Rescale of 42 : 1 : 4 : 0

	f1-score	precision	recall	support
AFIB	0.666369	0.670014	0.662787	875.0
GSVT	0.663194	0.657602	0.668948	691.0
SB	0.697514	0.690908	0.704250	1553.0
SR	0.671555	0.684082	0.659487	907.0
macro avg	0.674658	0.675651	0.673868	4026.0
micro avg	0.679095	0.679095	0.679095	4026.0
weighted avg	0.679006	0.679113	0.679095	4026.0

The result of Model Number:Dataset Number:Featuregroup Number:Rescale of 42 : 1 : 4 : 1

	f1-score	precision	recall	support
AFIB	0.758618	0.781624	0.736959	875.0
GSVT	0.809836	0.804219	0.815574	691.0
SB	0.886059	0.882669	0.889480	1553.0
SR	0.837998	0.825421	0.851014	907.0
macro avg	0.823128	0.823483	0.823257	4026.0
micro avg	0.834981	0.834981	0.834981	4026.0
weighted avg	0.834451	0.834346	0.834981	4026.0

The result of Model Number:Dataset Number:Featuregroup Number:Rescale of 42 : 1 : 5 : 0

	f1-score	precision	recall	support
AFIB	0.755096	0.776346	0.735133	204.0
GSVT	0.774617	0.769180	0.780278	300.0
SB	0.795919	0.792735	0.799131	892.0
SR	0.785340	0.785831	0.784873	681.0
macro avg	0.777743	0.781023	0.774854	2077.0
micro avg	0.785447	0.785447	0.785447	2077.0
weighted avg	0.785364	0.785459	0.785447	2077.0

The result of Model Number:Dataset Number:Featuregroup Number:Rescale of 42 : 1 : 5 : 1

	f1-score	precision	recall	support
AFIB	0.869501	0.872170	0.866986	204.0
GSVT	0.892342	0.890075	0.894701	300.0
SB	0.912276	0.911162	0.913399	892.0
SR	0.904809	0.906549	0.903084	681.0
macro avg	0.894732	0.894989	0.894543	2077.0

micro avg 0.902758 0.902758 0.902758 2077.0
weighted avg 0.902747 0.902774 0.902758 2077.0
The result of Model Number:Dataset Number:Featuregroup Number:Rescale of 42 : 1 : 6 : 0

	f1-score	precision	recall	support
AFIB	0.716650	0.724614	0.709228	204.0
GSVT	0.738849	0.734643	0.743318	300.0
SB	0.753547	0.752538	0.754561	892.0
SR	0.747137	0.748138	0.746149	681.0
macro avg	0.739046	0.739983	0.738314	2077.0
micro avg	0.745726	0.745726	0.745726	2077.0
weighted avg	0.745699	0.745768	0.745726	2077.0

The result of Model Number:Dataset Number:Featuregroup Number:Rescale of 42 : 1 : 6 : 1

	f1-score	precision	recall	support
AFIB	0.750803	0.743251	0.758531	875.0
GSVT	0.757522	0.764266	0.750951	691.0
SB	0.801425	0.798965	0.803901	1553.0
SR	0.785905	0.792784	0.779147	907.0
macro avg	0.773914	0.774817	0.773132	4026.0
micro avg	0.779375	0.779375	0.779375	4026.0
weighted avg	0.779391	0.779508	0.779375	4026.0

The result of Model Number:Dataset Number:Featuregroup Number:Rescale of 42 : 1 : 7 : 0

	f1-score	precision	recall	support
AFIB	0.688817	0.702165	0.676276	204.0
GSVT	0.715528	0.709545	0.721744	300.0
SB	0.728879	0.727416	0.730349	892.0
SR	0.721929	0.722677	0.721186	681.0
macro avg	0.713788	0.715451	0.712389	2077.0
micro avg	0.720791	0.720791	0.720791	2077.0
weighted avg	0.720737	0.720801	0.720791	2077.0

The result of Model Number:Dataset Number:Featuregroup Number:Rescale of 42 : 1 : 7 : 1

	f1-score	precision	recall	support
AFIB	0.838240	0.845739	0.830977	875.0
GSVT	0.854249	0.838006	0.871299	691.0
SB	0.906714	0.898859	0.914711	1553.0
SR	0.869473	0.889465	0.850399	907.0
macro avg	0.867169	0.868017	0.866847	4026.0
micro avg	0.874573	0.874573	0.874573	4026.0
weighted avg	0.874438	0.874753	0.874573	4026.0

The result of Model Number:Dataset Number:Featuregroup Number:Rescale of 42 : 1 : 8 : 0

	f1-score	precision	recall	support
AFIB	0.711187	0.789273	0.647333	204.0
GSVT	0.830408	0.820026	0.841218	300.0
SB	0.883513	0.879004	0.888068	892.0
SR	0.862815	0.850753	0.875229	681.0
macro avg	0.821981	0.834764	0.812962	2077.0
micro avg	0.853447	0.853447	0.853447	2077.0
weighted avg	0.852131	0.852409	0.853447	2077.0

The result of Model Number:Dataset Number:Featuregroup Number:Rescale of 42 : 1 : 8 : 1

	f1-score	precision	recall	support
AFIB	0.664493	0.678488	0.651168	204.0
GSVT	0.685152	0.678893	0.691544	300.0
SB	0.696342	0.694790	0.697904	892.0
SR	0.688067	0.688786	0.687357	681.0
macro avg	0.683514	0.685239	0.681993	2077.0
micro avg	0.688937	0.688937	0.688937	2077.0
weighted avg	0.688885	0.688924	0.688937	2077.0

The result of Model Number:Dataset Number:Featuregroup Number:Rescale of 42 : 1 : 9 : 0

	f1-score	precision	recall	support
AFIB	0.817731	0.831326	0.804761	204.0
GSVT	0.833411	0.827490	0.839479	300.0
SB	0.852742	0.847949	0.857608	892.0
SR	0.841878	0.847028	0.836816	681.0
macro avg	0.836440	0.838448	0.834666	2077.0
micro avg	0.842982	0.842982	0.842982	2077.0
weighted avg	0.842949	0.843059	0.842982	2077.0

The result of Model Number:Dataset Number:Featuregroup Number:Rescale of 42 : 1 : 9 : 1

	f1-score	precision	recall	support
AFIB	0.901706	0.918984	0.885296	204.0
GSVT	0.930380	0.925588	0.935309	300.0
SB	0.953389	0.951905	0.954886	892.0
SR	0.943995	0.943057	0.944964	681.0
macro avg	0.932368	0.934883	0.930114	2077.0
micro avg	0.941970	0.941970	0.941970	2077.0
weighted avg	0.941910	0.941969	0.941970	2077.0

The result of Model Number:Dataset Number:Featuregroup Number:Rescale of 42 : 1 : 10 : 0

	f1-score	precision	recall	support
AFIB	0.648555	0.650456	0.646715	875.0
GSVT	0.659286	0.650819	0.668063	691.0
SB	0.697745	0.696049	0.699452	1553.0

SR 0.680077 0.688148 0.672200 907.0
macro avg 0.671416 0.671368 0.671608 4026.0
micro avg 0.676463 0.676463 0.676463 4026.0
weighted avg 0.676473 0.676597 0.676463 4026.0
The result of Model Number:Dataset Number:Featuregroup Number:Rescale of 42 : 1 : 10 : 1

	f1-score	precision	recall	support
AFIB	0.845912	0.869718	0.823548	204.0
GSVT	0.867781	0.861690	0.874123	300.0
SB	0.891645	0.888077	0.895243	892.0
SR	0.879794	0.880343	0.879270	681.0
macro avg	0.871283	0.874957	0.868046	2077.0
micro avg	0.879914	0.879914	0.879914	2077.0
weighted avg	0.879821	0.879927	0.879914	2077.0

The result of Model Number:Dataset Number:Featuregroup Number:Rescale of 43 : 0 : 0 : 0

	f1-score	precision	recall	support
AFIB	0.807574	0.822435	0.793651	204.0
GSVT	0.837369	0.830356	0.844647	300.0
SB	0.853092	0.851663	0.854527	892.0
SR	0.845560	0.846311	0.844815	681.0
macro avg	0.835899	0.837691	0.834410	2077.0
micro avg	0.843936	0.843936	0.843936	2077.0
weighted avg	0.843881	0.843960	0.843936	2077.0

The result of Model Number:Dataset Number:Featuregroup Number:Rescale of 43 : 0 : 0 : 1

	f1-score	precision	recall	support
AFIB	0.659201	0.695060	0.627029	204.0
GSVT	0.692039	0.681572	0.702860	300.0
SB	0.708485	0.703876	0.713165	892.0
SR	0.699744	0.700374	0.699131	681.0
macro avg	0.689867	0.695221	0.685546	2077.0
micro avg	0.698615	0.698615	0.698615	2077.0
weighted avg	0.698403	0.698640	0.698615	2077.0

The result of Model Number:Dataset Number:Featuregroup Number:Rescale of 43 : 0 : 1 : 0

	f1-score	precision	recall	support
AFIB	0.773282	0.791792	0.756749	204.0
GSVT	0.796053	0.784837	0.807927	300.0
SB	0.816737	0.808439	0.825244	892.0
SR	0.797216	0.808727	0.786091	681.0
macro avg	0.795822	0.798449	0.794003	2077.0
micro avg	0.803178	0.803178	0.803178	2077.0
weighted avg	0.803081	0.803489	0.803178	2077.0

The result of Model Number:Dataset Number:Featuregroup Number:Rescale of 43 : 0 : 1 : 1

	f1-score	precision	recall	support
AFIB	0.679079	0.684448	0.673817	875.0
GSVT	0.696167	0.694695	0.697671	691.0
SB	0.742134	0.735575	0.748821	1553.0
SR	0.722378	0.729463	0.715447	907.0
macro avg	0.709940	0.711045	0.708939	4026.0
micro avg	0.716222	0.716222	0.716222	4026.0
weighted avg	0.716090	0.716070	0.716222	4026.0

The result of Model Number:Dataset Number:Featuregroup Number:Rescale of 43 : 0 : 2 : 0

	f1-score	precision	recall	support
AFIB	0.751255	0.766326	0.736915	204.0
GSVT	0.768666	0.760676	0.776866	300.0
SB	0.782882	0.779836	0.785953	892.0
SR	0.774655	0.777887	0.771468	681.0
macro avg	0.769364	0.771181	0.767801	2077.0
micro avg	0.775075	0.775075	0.775075	2077.0
weighted avg	0.775025	0.775103	0.775075	2077.0

The result of Model Number:Dataset Number:Featuregroup Number:Rescale of 43 : 0 : 2 : 1

	f1-score	precision	recall	support
AFIB	0.912590	0.938272	0.888464	204.0
GSVT	0.936183	0.929612	0.943025	300.0
SB	0.961928	0.958079	0.965810	892.0
SR	0.949142	0.949736	0.948578	681.0
macro avg	0.939961	0.943925	0.936469	2077.0
micro avg	0.949272	0.949272	0.949272	2077.0
weighted avg	0.949171	0.949286	0.949272	2077.0

The result of Model Number:Dataset Number:Featuregroup Number:Rescale of 43 : 0 : 3 : 0

	f1-score	precision	recall	support
AFIB	0.654935	0.648944	0.661068	875.0
GSVT	0.663327	0.669180	0.657675	691.0
SB	0.705095	0.701378	0.708863	1553.0
SR	0.689156	0.697461	0.681109	907.0
macro avg	0.678129	0.679241	0.677179	4026.0
micro avg	0.683437	0.683437	0.683437	4026.0
weighted avg	0.683434	0.683573	0.683437	4026.0

The result of Model Number:Dataset Number:Featuregroup Number:Rescale of 43 : 0 : 3 : 1

	f1-score	precision	recall	support
AFIB	0.755117	0.759261	0.751066	875.0

GSVT 0.756173 0.746613 0.766100 691.0
SB 0.795247 0.792056 0.798471 1553.0
SR 0.778175 0.787622 0.769001 907.0
macro avg 0.771178 0.771388 0.771159 4026.0
micro avg 0.775973 0.775973 0.775973 4026.0
weighted avg 0.775973 0.776130 0.775973 4026.0
The result of Model Number:Dataset Number:Featuregroup Number:Rescale of 43 : 0 : 4 : 0

	f1-score	precision	recall	support
AFIB	0.857215	0.858946	0.855510	204.0
GSVT	0.881064	0.878444	0.883704	300.0
SB	0.895663	0.894865	0.896465	892.0
SR	0.887964	0.889670	0.886277	681.0
macro avg	0.880477	0.880481	0.880489	2077.0
micro avg	0.887259	0.887259	0.887259	2077.0
weighted avg	0.887254	0.887262	0.887259	2077.0

The result of Model Number:Dataset Number:Featuregroup Number:Rescale of 43 : 0 : 4 : 1

	f1-score	precision	recall	support
AFIB	0.675431	0.680721	0.670259	875.0
GSVT	0.677212	0.668569	0.686124	691.0
SB	0.710319	0.707471	0.713199	1553.0
SR	0.695312	0.702137	0.688644	907.0
macro avg	0.689569	0.689725	0.689556	4026.0
micro avg	0.693687	0.693687	0.693687	4026.0
weighted avg	0.693674	0.693779	0.693687	4026.0

The result of Model Number:Dataset Number:Featuregroup Number:Rescale of 43 : 0 : 5 : 0

	f1-score	precision	recall	support
AFIB	0.861557	0.860769	0.862451	875.0
GSVT	0.879217	0.877511	0.881021	691.0
SB	0.932964	0.930886	0.935065	1553.0
SR	0.907499	0.913420	0.901688	907.0
macro avg	0.895309	0.895646	0.895056	4026.0
micro avg	0.902488	0.902488	0.902488	4026.0
weighted avg	0.902483	0.902551	0.902488	4026.0

The result of Model Number:Dataset Number:Featuregroup Number:Rescale of 43 : 0 : 5 : 1

	f1-score	precision	recall	support
AFIB	0.739589	0.797233	0.690062	204.0
GSVT	0.762143	0.775044	0.749696	300.0
SB	0.807728	0.800294	0.815326	892.0
SR	0.775579	0.764333	0.787198	681.0
macro avg	0.771260	0.784226	0.760571	2077.0

micro avg 0.784321 0.784321 0.784321 2077.0
weighted avg 0.783910 0.784555 0.784321 2077.0
The result of Model Number:Dataset Number:Featuregroup Number:Rescale of 43 : 0 : 6 : 0
f1-score precision recall support
AFIB 0.832557 0.821849 0.843606 875.0
GSVT 0.849504 0.856537 0.842621 691.0
SB 0.908291 0.904248 0.912381 1553.0
SR 0.882443 0.895495 0.869788 907.0
macro avg 0.868199 0.869532 0.867099 4026.0
micro avg 0.875865 0.875865 0.875865 4026.0
weighted avg 0.875918 0.876179 0.875865 4026.0
The result of Model Number:Dataset Number:Featuregroup Number:Rescale of 43 : 0 : 6 : 1
f1-score precision recall support
AFIB 0.751535 0.757492 0.745690 875.0
GSVT 0.761063 0.757708 0.764474 691.0
SB 0.809301 0.806101 0.812531 1553.0
SR 0.789413 0.791644 0.787234 907.0
macro avg 0.777828 0.778236 0.777482 4026.0
micro avg 0.784057 0.784057 0.784057 4026.0
weighted avg 0.783987 0.783974 0.784057 4026.0
The result of Model Number:Dataset Number:Featuregroup Number:Rescale of 43 : 0 : 7 : 0
f1-score precision recall support
AFIB 0.687215 0.688405 0.686039 875.0
GSVT 0.695547 0.687555 0.703785 691.0
SB 0.732435 0.723604 0.741488 1553.0
SR 0.697620 0.718758 0.677706 907.0
macro avg 0.703205 0.704581 0.702255 4026.0
micro avg 0.708597 0.708597 0.708597 4026.0
weighted avg 0.708433 0.708675 0.708597 4026.0
The result of Model Number:Dataset Number:Featuregroup Number:Rescale of 43 : 0 : 7 : 1
f1-score precision recall support
AFIB 0.841443 0.848929 0.834132 875.0
GSVT 0.859104 0.849677 0.868799 691.0
SB 0.923962 0.916768 0.931277 1553.0
SR 0.893257 0.905919 0.880949 907.0
macro avg 0.879442 0.880323 0.878789 4026.0
micro avg 0.888102 0.888102 0.888102 4026.0
weighted avg 0.887978 0.888065 0.888102 4026.0
The result of Model Number:Dataset Number:Featuregroup Number:Rescale of 43 : 0 : 8 : 0

	f1-score	precision	recall	support
AFIB	0.673701	0.672949	0.674475	875.0
GSVT	0.686024	0.677444	0.694845	691.0
SB	0.720665	0.714871	0.726559	1553.0
SR	0.688512	0.706577	0.671359	907.0
macro avg	0.692226	0.692960	0.691809	4026.0
micro avg	0.697360	0.697360	0.697360	4026.0
weighted avg	0.697269	0.697467	0.697360	4026.0

The result of Model Number:Dataset Number:Featuregroup Number:Rescale of 43 : 0 : 8 : 1

	f1-score	precision	recall	support
AFIB	0.863861	0.845304	0.883299	875.0
GSVT	0.894619	0.907593	0.882070	691.0
SB	0.952357	0.952176	0.952542	1553.0
SR	0.928488	0.939280	0.917947	907.0
macro avg	0.909832	0.911088	0.908965	4026.0
micro avg	0.917604	0.917604	0.917604	4026.0
weighted avg	0.917837	0.918391	0.917604	4026.0

The result of Model Number:Dataset Number:Featuregroup Number:Rescale of 43 : 0 : 9 : 0

	f1-score	precision	recall	support
AFIB	0.698453	0.778992	0.633099	204.0
GSVT	0.823548	0.809213	0.838574	300.0
SB	0.879294	0.875397	0.883236	892.0
SR	0.858889	0.847145	0.870989	681.0
macro avg	0.815046	0.827687	0.806475	2077.0
micro avg	0.848201	0.848201	0.848201	2077.0
weighted avg	0.846790	0.847106	0.848201	2077.0

The result of Model Number:Dataset Number:Featuregroup Number:Rescale of 43 : 0 : 9 : 1

	f1-score	precision	recall	support
AFIB	0.782419	0.761865	0.831401	875.0
GSVT	0.752680	0.712421	0.858040	691.0
SB	0.713203	0.708696	0.717789	1553.0
SR	0.692764	0.704700	0.681419	907.0
macro avg	0.735267	0.721921	0.772162	4026.0
micro avg	0.758359	0.758359	0.758359	4026.0
weighted avg	0.730417	0.719991	0.758359	4026.0

The result of Model Number:Dataset Number:Featuregroup Number:Rescale of 43 : 0 : 10 : 0

	f1-score	precision	recall	support
AFIB	0.726265	0.786893	0.675758	204.0
GSVT	0.779003	0.769207	0.789368	300.0
SB	0.798890	0.787698	0.810498	892.0

SR 0.777097 0.780738 0.773870 681.0
macro avg 0.770314 0.781134 0.762373 2077.0
micro avg 0.782202 0.782202 0.782202 2077.0
weighted avg 0.781739 0.782666 0.782202 2077.0
The result of Model Number:Dataset Number:Featuregroup Number:Rescale of 43 : 0 : 10 : 1

	f1-score	precision	recall	support
AFIB	0.833841	0.854636	0.814096	875.0
GSVT	0.887061	0.890461	0.883727	691.0
SB	0.960612	0.955961	0.965312	1553.0
SR	0.910233	0.895061	0.925987	907.0
macro avg	0.897937	0.899030	0.897280	4026.0
micro avg	0.909585	0.909585	0.909585	4026.0
weighted avg	0.909086	0.908978	0.909585	4026.0

The result of Model Number:Dataset Number:Featuregroup Number:Rescale of 43 : 1 : 0 : 0

	f1-score	precision	recall	support
AFIB	0.661856	0.697859	0.629554	204.0
GSVT	0.694826	0.684317	0.705691	300.0
SB	0.711338	0.706710	0.716037	892.0
SR	0.702561	0.703195	0.701946	681.0
macro avg	0.692645	0.698020	0.688307	2077.0
micro avg	0.701428	0.701428	0.701428	2077.0
weighted avg	0.701215	0.701454	0.701428	2077.0

The result of Model Number:Dataset Number:Featuregroup Number:Rescale of 43 : 1 : 0 : 1

	f1-score	precision	recall	support
AFIB	0.631952	0.637638	0.626565	875.0
GSVT	0.644825	0.631750	0.658605	691.0
SB	0.683841	0.676234	0.691704	1553.0
SR	0.656705	0.676226	0.638435	907.0
macro avg	0.654331	0.655462	0.653827	4026.0
micro avg	0.659865	0.659865	0.659865	4026.0
weighted avg	0.659754	0.660209	0.659865	4026.0

The result of Model Number:Dataset Number:Featuregroup Number:Rescale of 43 : 1 : 1 : 0

	f1-score	precision	recall	support
AFIB	0.657267	0.655873	0.658698	875.0
GSVT	0.667536	0.664336	0.670823	691.0
SB	0.702579	0.694192	0.711173	1553.0
SR	0.669929	0.688908	0.651981	907.0
macro avg	0.674328	0.675827	0.673169	4026.0
micro avg	0.679508	0.679508	0.679508	4026.0
weighted avg	0.679361	0.679549	0.679508	4026.0

The result of Model Number:Dataset Number:Featuregroup Number:Rescale of 43 : 1 : 1 : 1

	f1-score	precision	recall	support
AFIB	0.769641	0.754309	0.785649	875.0
GSVT	0.626420	0.582537	0.785199	691.0
SB	0.508270	0.509146	0.507398	1553.0
SR	0.823971	0.831227	0.816876	907.0
macro avg	0.682076	0.669305	0.723780	4026.0
micro avg	0.685273	0.685273	0.685273	4026.0
weighted avg	0.656477	0.647586	0.685273	4026.0

The result of Model Number:Dataset Number:Featuregroup Number:Rescale of 43 : 1 : 2 : 0

	f1-score	precision	recall	support
AFIB	0.578834	0.639378	0.528934	204.0
GSVT	0.680958	0.671967	0.690266	300.0
SB	0.722153	0.719347	0.724986	892.0
SR	0.704801	0.694756	0.715148	681.0
macro avg	0.671687	0.681362	0.664833	2077.0
micro avg	0.697489	0.697489	0.697489	2077.0
weighted avg	0.696437	0.696586	0.697489	2077.0

The result of Model Number:Dataset Number:Featuregroup Number:Rescale of 43 : 1 : 2 : 1

	f1-score	precision	recall	support
AFIB	0.902359	0.920460	0.885135	204.0
GSVT	0.923271	0.913674	0.933121	300.0
SB	0.940347	0.936689	0.944036	892.0
SR	0.930466	0.934348	0.926637	681.0
macro avg	0.924111	0.926293	0.922232	2077.0
micro avg	0.930970	0.930970	0.930970	2077.0
weighted avg	0.930909	0.931003	0.930970	2077.0

The result of Model Number:Dataset Number:Featuregroup Number:Rescale of 43 : 1 : 3 : 0

	f1-score	precision	recall	support
AFIB	0.715451	0.732300	0.699422	875.0
GSVT	0.754192	0.756096	0.752346	691.0
SB	0.820197	0.816797	0.823629	1553.0
SR	0.780423	0.768132	0.793150	907.0
macro avg	0.767566	0.768331	0.767137	4026.0
micro avg	0.777533	0.777533	0.777533	4026.0
weighted avg	0.777142	0.777051	0.777533	4026.0

The result of Model Number:Dataset Number:Featuregroup Number:Rescale of 43 : 1 : 3 : 1

	f1-score	precision	recall	support
AFIB	0.677034	0.680488	0.673637	875.0

	f1-score	precision	recall	support
GSVT	0.686378	0.682807	0.690046	691.0
SB	0.719431	0.718788	0.720080	1553.0
SR	0.706184	0.706760	0.705638	907.0
macro avg	0.697257	0.697211	0.697350	4026.0
micro avg	0.701578	0.701578	0.701578	4026.0
weighted avg	0.701559	0.701579	0.701578	4026.0

The result of Model Number:Dataset Number:Featuregroup Number:Rescale of 43 : 1 : 4 : 0

	f1-score	precision	recall	support
AFIB	0.922957	0.937108	0.909378	204.0
GSVT	0.944841	0.936825	0.953035	300.0
SB	0.961277	0.958175	0.964402	892.0
SR	0.951399	0.954931	0.947905	681.0
macro avg	0.945118	0.946760	0.943680	2077.0
micro avg	0.951947	0.951947	0.951947	2077.0
weighted avg	0.951900	0.951958	0.951947	2077.0

The result of Model Number:Dataset Number:Featuregroup Number:Rescale of 43 : 1 : 4 : 1

	f1-score	precision	recall	support
AFIB	0.862383	0.869345	0.855605	204.0
GSVT	0.882171	0.877845	0.886570	300.0
SB	0.899388	0.898390	0.900396	892.0
SR	0.892672	0.893859	0.891494	681.0
macro avg	0.884153	0.884860	0.883516	2077.0
micro avg	0.891081	0.891081	0.891081	2077.0
weighted avg	0.891065	0.891084	0.891081	2077.0

The result of Model Number:Dataset Number:Featuregroup Number:Rescale of 43 : 1 : 5 : 0

	f1-score	precision	recall	support
AFIB	0.683332	0.682514	0.684267	875.0
GSVT	0.696652	0.699049	0.694344	691.0
SB	0.737859	0.733313	0.742466	1553.0
SR	0.722843	0.729903	0.715999	907.0
macro avg	0.710171	0.711195	0.709269	4026.0
micro avg	0.715595	0.715595	0.715595	4026.0
weighted avg	0.715553	0.715623	0.715595	4026.0

The result of Model Number:Dataset Number:Featuregroup Number:Rescale of 43 : 1 : 5 : 1

	f1-score	precision	recall	support
AFIB	0.858275	0.878820	0.839925	204.0
GSVT	0.883549	0.871100	0.896728	300.0
SB	0.906506	0.897296	0.915948	892.0
SR	0.884840	0.897616	0.872492	681.0
macro avg	0.883292	0.886208	0.881273	2077.0

micro avg 0.891457 0.891457 0.891457 2077.0
weighted avg 0.891349 0.891803 0.891457 2077.0
The result of Model Number:Dataset Number:Featuregroup Number:Rescale of 43 : 1 : 6 : 0

	f1-score	precision	recall	support
AFIB	0.780737	0.809561	0.753968	204.0
GSVT	0.797425	0.789241	0.805904	300.0
SB	0.819266	0.815445	0.823123	892.0
SR	0.806491	0.806997	0.806013	681.0
macro avg	0.800980	0.805311	0.797252	2077.0
micro avg	0.808234	0.808234	0.808234	2077.0
weighted avg	0.808138	0.808312	0.808234	2077.0

The result of Model Number:Dataset Number:Featuregroup Number:Rescale of 43 : 1 : 6 : 1

	f1-score	precision	recall	support
AFIB	0.700014	0.697748	0.702317	875.0
GSVT	0.708131	0.710331	0.706015	691.0
SB	0.747193	0.745567	0.748829	1553.0
SR	0.733414	0.736874	0.730014	907.0
macro avg	0.722188	0.722630	0.721794	4026.0
micro avg	0.727133	0.727133	0.727133	4026.0
weighted avg	0.727131	0.727168	0.727133	4026.0

The result of Model Number:Dataset Number:Featuregroup Number:Rescale of 43 : 1 : 7 : 0

	f1-score	precision	recall	support
AFIB	0.784940	0.787241	0.782713	875.0
GSVT	0.797928	0.787680	0.808550	691.0
SB	0.844474	0.842422	0.846541	1553.0
SR	0.823091	0.832859	0.813558	907.0
macro avg	0.812608	0.812550	0.812840	4026.0
micro avg	0.818717	0.818717	0.818717	4026.0
weighted avg	0.818729	0.818879	0.818717	4026.0

The result of Model Number:Dataset Number:Featuregroup Number:Rescale of 43 : 1 : 7 : 1

	f1-score	precision	recall	support
AFIB	0.735337	0.738021	0.732722	875.0
GSVT	0.748047	0.738306	0.758112	691.0
SB	0.788517	0.779832	0.797402	1553.0
SR	0.747288	0.767931	0.727766	907.0
macro avg	0.754797	0.756023	0.754001	4026.0
micro avg	0.760913	0.760913	0.760913	4026.0
weighted avg	0.760725	0.760937	0.760913	4026.0

The result of Model Number:Dataset Number:Featuregroup Number:Rescale of 43 : 1 : 8 : 0

	f1-score	precision	recall	support
AFIB	0.650268	0.670369	0.631905	204.0
GSVT	0.607017	0.620214	0.594819	300.0
SB	0.640250	0.611834	0.671465	892.0
SR	0.576475	0.606097	0.549694	681.0
macro avg	0.618503	0.627129	0.611971	2077.0
micro avg	0.616583	0.616583	0.616583	2077.0
weighted avg	0.615523	0.616913	0.616583	2077.0

The result of Model Number:Dataset Number:Featuregroup Number:Rescale of 43 : 1 : 8 : 1

	f1-score	precision	recall	support
AFIB	0.563948	0.620845	0.516934	204.0
GSVT	0.665059	0.655879	0.674605	300.0
SB	0.706164	0.703808	0.708538	892.0
SR	0.688079	0.678560	0.697879	681.0
macro avg	0.655812	0.664773	0.649489	2077.0
micro avg	0.681323	0.681323	0.681323	2077.0
weighted avg	0.680329	0.680458	0.681323	2077.0

The result of Model Number:Dataset Number:Featuregroup Number:Rescale of 43 : 1 : 9 : 0

	f1-score	precision	recall	support
AFIB	0.616030	0.627535	0.682039	204.0
GSVT	0.739787	0.724801	0.755781	300.0
SB	0.608584	0.602898	0.614385	892.0
SR	0.740731	0.746919	0.734748	681.0
macro avg	0.676283	0.675538	0.696738	2077.0
micro avg	0.680917	0.680917	0.680917	2077.0
weighted avg	0.671594	0.670147	0.680917	2077.0

The result of Model Number:Dataset Number:Featuregroup Number:Rescale of 43 : 1 : 9 : 1

	f1-score	precision	recall	support
AFIB	0.672905	0.672154	0.673679	875.0
GSVT	0.685214	0.676644	0.694024	691.0
SB	0.719813	0.714026	0.725700	1553.0
SR	0.687699	0.705742	0.670566	907.0
macro avg	0.691408	0.692141	0.690992	4026.0
micro avg	0.696536	0.696536	0.696536	4026.0
weighted avg	0.696445	0.696643	0.696536	4026.0

The result of Model Number:Dataset Number:Featuregroup Number:Rescale of 43 : 1 : 10 : 0

	f1-score	precision	recall	support
AFIB	0.808131	0.800738	0.815698	875.0
GSVT	0.818486	0.825708	0.811512	691.0
SB	0.870024	0.865438	0.874672	1553.0

SR 0.850357 0.860604 0.840427 907.0
macro avg 0.836749 0.838122 0.835577 4026.0
micro avg 0.843300 0.843300 0.843300 4026.0
weighted avg 0.843296 0.843468 0.843300 4026.0
The result of Model Number:Dataset Number:Featuregroup Number:Rescale of 43 : 1 : 10 : 1

	f1-score	precision	recall	support
AFIB	0.793433	0.804162	0.783318	204.0
GSVT	0.823570	0.822233	0.824940	300.0
SB	0.841455	0.840334	0.842586	892.0
SR	0.832854	0.831787	0.833953	681.0
macro avg	0.822828	0.824629	0.821199	2077.0
micro avg	0.831386	0.831386	0.831386	2077.0
weighted avg	0.831335	0.831364	0.831386	2077.0

The result of Model Number:Dataset Number:Featuregroup Number:Rescale of 44 : 0 : 0 : 0

	f1-score	precision	recall	support
AFIB	0.889017	0.889810	0.888316	875.0
GSVT	0.905301	0.907676	0.903091	691.0
SB	0.960989	0.954591	0.967484	1553.0
SR	0.942265	0.950868	0.933851	907.0
macro avg	0.924393	0.925736	0.923185	4026.0
micro avg	0.931649	0.931649	0.931649	4026.0
weighted avg	0.931571	0.931621	0.931649	4026.0

The result of Model Number:Dataset Number:Featuregroup Number:Rescale of 44 : 0 : 0 : 1

	f1-score	precision	recall	support
AFIB	0.814202	0.858492	0.774465	204.0
GSVT	0.854761	0.841834	0.868127	300.0
SB	0.875074	0.869381	0.880854	892.0
SR	0.864277	0.865056	0.863520	681.0
macro avg	0.852078	0.858691	0.846742	2077.0
micro avg	0.862883	0.862883	0.862883	2077.0
weighted avg	0.862621	0.862915	0.862883	2077.0

The result of Model Number:Dataset Number:Featuregroup Number:Rescale of 44 : 0 : 1 : 0

	f1-score	precision	recall	support
AFIB	0.831758	0.835544	0.828058	875.0
GSVT	0.835800	0.827469	0.844372	691.0
SB	0.880935	0.871336	0.890748	1553.0
SR	0.842519	0.862465	0.823478	907.0
macro avg	0.847753	0.849203	0.846664	4026.0
micro avg	0.854009	0.854009	0.854009	4026.0
weighted avg	0.853846	0.854029	0.854009	4026.0

The result of Model Number:Dataset Number:Featuregroup Number:Rescale of 44 : 0 : 1 : 1

	f1-score	precision	recall	support
AFIB	0.710725	0.726409	0.695755	204.0
GSVT	0.726257	0.714214	0.738801	300.0
SB	0.740212	0.734423	0.746102	892.0
SR	0.722248	0.730941	0.713761	681.0
macro avg	0.724861	0.726497	0.723605	2077.0
micro avg	0.729499	0.729499	0.729499	2077.0
weighted avg	0.729410	0.729575	0.729499	2077.0

The result of Model Number:Dataset Number:Featuregroup Number:Rescale of 44 : 0 : 2 : 0

	f1-score	precision	recall	support
AFIB	0.709549	0.755781	0.668962	204.0
GSVT	0.740634	0.722429	0.759880	300.0
SB	0.762355	0.752561	0.772433	892.0
SR	0.742553	0.751420	0.733943	681.0
macro avg	0.738773	0.745548	0.733805	2077.0
micro avg	0.747837	0.747837	0.747837	2077.0
weighted avg	0.747538	0.748151	0.747837	2077.0

The result of Model Number:Dataset Number:Featuregroup Number:Rescale of 44 : 0 : 2 : 1

	f1-score	precision	recall	support
AFIB	0.897617	0.911226	0.884431	204.0
GSVT	0.912051	0.904362	0.919919	300.0
SB	0.932281	0.929274	0.935311	892.0
SR	0.922274	0.925699	0.918898	681.0
macro avg	0.916056	0.917640	0.914640	2077.0
micro avg	0.922709	0.922709	0.922709	2077.0
weighted avg	0.922673	0.922731	0.922709	2077.0

The result of Model Number:Dataset Number:Featuregroup Number:Rescale of 44 : 0 : 3 : 0

	f1-score	precision	recall	support
AFIB	0.779469	0.763728	0.795936	875.0
GSVT	0.805312	0.818117	0.793076	691.0
SB	0.856864	0.856816	0.856919	1553.0
SR	0.836089	0.843708	0.828621	907.0
macro avg	0.819434	0.820592	0.818638	4026.0
micro avg	0.826332	0.826332	0.826332	4026.0
weighted avg	0.826515	0.826989	0.826332	4026.0

The result of Model Number:Dataset Number:Featuregroup Number:Rescale of 44 : 0 : 3 : 1

	f1-score	precision	recall	support
AFIB	0.735495	0.720641	0.751032	875.0

GSVT 0.759880 0.771961 0.748334 691.0
SB 0.808523 0.808478 0.808575 1553.0
SR 0.788920 0.796109 0.781873 907.0
macro avg 0.773204 0.774297 0.772453 4026.0
micro avg 0.779714 0.779714 0.779714 4026.0
weighted avg 0.779886 0.780334 0.779714 4026.0
The result of Model Number:Dataset Number:Featuregroup Number:Rescale of 44 : 0 : 4 : 0

	f1-score	precision	recall	support
AFIB	0.747081	0.756974	0.737556	204.0
GSVT	0.769626	0.766848	0.772441	300.0
SB	0.779212	0.776787	0.781656	892.0
SR	0.770722	0.772206	0.769264	681.0
macro avg	0.766660	0.768204	0.765229	2077.0
micro avg	0.771930	0.771930	0.771930	2077.0
weighted avg	0.771888	0.771904	0.771930	2077.0

The result of Model Number:Dataset Number:Featuregroup Number:Rescale of 44 : 0 : 4 : 1

	f1-score	precision	recall	support
AFIB	0.677738	0.674982	0.680534	875.0
GSVT	0.687645	0.691802	0.683564	691.0
SB	0.725251	0.724368	0.726138	1553.0
SR	0.710121	0.711246	0.709020	907.0
macro avg	0.700189	0.700599	0.699814	4026.0
micro avg	0.705063	0.705063	0.705063	4026.0
weighted avg	0.705061	0.705089	0.705063	4026.0

The result of Model Number:Dataset Number:Featuregroup Number:Rescale of 44 : 0 : 5 : 0

	f1-score	precision	recall	support
AFIB	0.815700	0.827247	0.805141	204.0
GSVT	0.832636	0.817305	0.848705	300.0
SB	0.850382	0.840980	0.860011	892.0
SR	0.832511	0.849467	0.816268	681.0
macro avg	0.832807	0.833750	0.832531	2077.0
micro avg	0.838646	0.838646	0.838646	2077.0
weighted avg	0.838553	0.838994	0.838646	2077.0

The result of Model Number:Dataset Number:Featuregroup Number:Rescale of 44 : 0 : 5 : 1

	f1-score	precision	recall	support
AFIB	0.727307	0.734391	0.720535	204.0
GSVT	0.744126	0.740709	0.747599	300.0
SB	0.754409	0.751813	0.757028	892.0
SR	0.748038	0.750940	0.745193	681.0
macro avg	0.743470	0.744463	0.742589	2077.0

micro avg 0.748201 0.748201 0.748201 2077.0
weighted avg 0.748173 0.748212 0.748201 2077.0
The result of Model Number:Dataset Number:Featuregroup Number:Rescale of 44 : 0 : 6 : 0

	f1-score	precision	recall	support
AFIB	0.868205	0.891670	0.846122	204.0
GSVT	0.875361	0.862475	0.888757	300.0
SB	0.892317	0.882824	0.902019	892.0
SR	0.875767	0.887800	0.864069	681.0
macro avg	0.877912	0.881192	0.875242	2077.0
micro avg	0.882170	0.882170	0.882170	2077.0
weighted avg	0.882073	0.882385	0.882170	2077.0

The result of Model Number:Dataset Number:Featuregroup Number:Rescale of 44 : 0 : 6 : 1

	f1-score	precision	recall	support
AFIB	0.795331	0.794615	0.796125	875.0
GSVT	0.809109	0.801701	0.816719	691.0
SB	0.849064	0.841599	0.856663	1553.0
SR	0.809091	0.829093	0.790102	907.0
macro avg	0.815649	0.816752	0.814902	4026.0
micro avg	0.821655	0.821655	0.821655	4026.0
weighted avg	0.821523	0.821722	0.821655	4026.0

The result of Model Number:Dataset Number:Featuregroup Number:Rescale of 44 : 0 : 7 : 0

	f1-score	precision	recall	support
AFIB	0.779218	0.779357	0.779113	875.0
GSVT	0.787462	0.790122	0.784869	691.0
SB	0.827188	0.824336	0.830064	1553.0
SR	0.811481	0.814218	0.808792	907.0
macro avg	0.801337	0.802008	0.800710	4026.0
micro avg	0.806441	0.806441	0.806441	4026.0
weighted avg	0.806405	0.806409	0.806441	4026.0

The result of Model Number:Dataset Number:Featuregroup Number:Rescale of 44 : 0 : 7 : 1

	f1-score	precision	recall	support
AFIB	0.639793	0.710039	0.582348	204.0
GSVT	0.747044	0.737705	0.756770	300.0
SB	0.794819	0.790763	0.798917	892.0
SR	0.776199	0.765347	0.787366	681.0
macro avg	0.739464	0.750963	0.731350	2077.0
micro avg	0.767771	0.767771	0.767771	2077.0
weighted avg	0.766587	0.766837	0.767771	2077.0

The result of Model Number:Dataset Number:Featuregroup Number:Rescale of 44 : 0 : 8 : 0

	f1-score	precision	recall	support
AFIB	0.813947	0.813897	0.814046	875.0
GSVT	0.815708	0.811298	0.820180	691.0
SB	0.854678	0.848991	0.860452	1553.0
SR	0.831983	0.845633	0.818797	907.0
macro avg	0.829079	0.829955	0.828369	4026.0
micro avg	0.834070	0.834070	0.834070	4026.0
weighted avg	0.834024	0.834138	0.834070	4026.0

The result of Model Number:Dataset Number:Featuregroup Number:Rescale of 44 : 0 : 8 : 1

	f1-score	precision	recall	support
AFIB	0.711816	0.738296	0.687488	204.0
GSVT	0.660538	0.691190	0.632551	300.0
SB	0.691940	0.650205	0.739412	892.0
SR	0.607657	0.649876	0.570659	681.0
macro avg	0.667988	0.682392	0.657527	2077.0
micro avg	0.663547	0.663547	0.663547	2077.0
weighted avg	0.661722	0.664669	0.663547	2077.0

The result of Model Number:Dataset Number:Featuregroup Number:Rescale of 44 : 0 : 9 : 0

	f1-score	precision	recall	support
AFIB	0.644192	0.629178	0.660044	875.0
GSVT	0.618401	0.625199	0.611791	691.0
SB	0.650768	0.608746	0.699038	1553.0
SR	0.520838	0.625426	0.446497	907.0
macro avg	0.608550	0.622137	0.604342	4026.0
micro avg	0.618695	0.618695	0.618695	4026.0
weighted avg	0.614512	0.619768	0.618695	4026.0

The result of Model Number:Dataset Number:Featuregroup Number:Rescale of 44 : 0 : 9 : 1

	f1-score	precision	recall	support
AFIB	0.754875	0.841920	0.684242	204.0
GSVT	0.890075	0.874582	0.906315	300.0
SB	0.950325	0.946113	0.954585	892.0
SR	0.928271	0.915579	0.941349	681.0
macro avg	0.880887	0.894549	0.871623	2077.0
micro avg	0.916720	0.916720	0.916720	2077.0
weighted avg	0.915195	0.915536	0.916720	2077.0

The result of Model Number:Dataset Number:Featuregroup Number:Rescale of 44 : 0 : 10 : 0

	f1-score	precision	recall	support
AFIB	0.856231	0.914694	0.805158	204.0
GSVT	0.905122	0.886677	0.924429	300.0
SB	0.927572	0.919440	0.935883	892.0

SR 0.904692 0.907704 0.901779 681.0
macro avg 0.898404 0.907129 0.891812 2077.0
micro avg 0.910207 0.910207 0.910207 2077.0
weighted avg 0.909820 0.910394 0.910207 2077.0
The result of Model Number:Dataset Number:Featuregroup Number:Rescale of 44 : 0 : 10 : 1

	f1-score	precision	recall	support
AFIB	0.647698	0.628339	0.690614	875.0
GSVT	0.554657	0.542511	0.567565	691.0
SB	0.735687	0.729693	0.741976	1553.0
SR	0.690180	0.719157	0.664743	907.0
macro avg	0.657056	0.654925	0.666225	4026.0
micro avg	0.683479	0.683479	0.683479	4026.0
weighted avg	0.675241	0.673165	0.683479	4026.0

The result of Model Number:Dataset Number:Featuregroup Number:Rescale of 44 : 1 : 0 : 0

	f1-score	precision	recall	support
AFIB	0.773871	0.786438	0.761741	875.0
GSVT	0.823035	0.825614	0.820521	691.0
SB	0.895420	0.892335	0.898532	1553.0
SR	0.847702	0.838260	0.857410	907.0
macro avg	0.835007	0.835662	0.834551	4026.0
micro avg	0.846149	0.846149	0.846149	4026.0
weighted avg	0.845829	0.845686	0.846149	4026.0

The result of Model Number:Dataset Number:Featuregroup Number:Rescale of 44 : 1 : 0 : 1

	f1-score	precision	recall	support
AFIB	0.844775	0.844724	0.844879	875.0
GSVT	0.846603	0.842026	0.851245	691.0
SB	0.887049	0.881146	0.893042	1553.0
SR	0.863494	0.877662	0.849809	907.0
macro avg	0.860480	0.861389	0.859744	4026.0
micro avg	0.865661	0.865661	0.865661	4026.0
weighted avg	0.865613	0.865731	0.865661	4026.0

The result of Model Number:Dataset Number:Featuregroup Number:Rescale of 44 : 1 : 1 : 0

	f1-score	precision	recall	support
AFIB	0.670674	0.656266	0.685764	875.0
GSVT	0.694553	0.704626	0.684810	691.0
SB	0.739379	0.739239	0.739523	1553.0
SR	0.720848	0.729226	0.712664	907.0
macro avg	0.706364	0.707339	0.705690	4026.0
micro avg	0.712398	0.712398	0.712398	4026.0
weighted avg	0.712578	0.713009	0.712398	4026.0

The result of Model Number:Dataset Number:Featuregroup Number:Rescale of 44 : 1 : 1 : 1

	f1-score	precision	recall	support
AFIB	0.662579	0.657511	0.667813	875.0
GSVT	0.677824	0.681531	0.674197	691.0
SB	0.717761	0.717258	0.718270	1553.0
SR	0.698696	0.702085	0.695376	907.0
macro avg	0.689215	0.689596	0.688914	4026.0
micro avg	0.694582	0.694582	0.694582	4026.0
weighted avg	0.694618	0.694723	0.694582	4026.0

The result of Model Number:Dataset Number:Featuregroup Number:Rescale of 44 : 1 : 2 : 0

	f1-score	precision	recall	support
AFIB	0.865011	0.884590	0.846391	204.0
GSVT	0.888030	0.883682	0.892460	300.0
SB	0.906197	0.903580	0.908836	892.0
SR	0.900375	0.899986	0.900778	681.0
macro avg	0.889903	0.892960	0.887116	2077.0
micro avg	0.897695	0.897695	0.897695	2077.0
weighted avg	0.897619	0.897663	0.897695	2077.0

The result of Model Number:Dataset Number:Featuregroup Number:Rescale of 44 : 1 : 2 : 1

	f1-score	precision	recall	support
AFIB	0.769179	0.739774	0.817563	875.0
GSVT	0.797000	0.769494	0.834309	691.0
SB	0.888006	0.872332	0.905230	1553.0
SR	0.752121	0.904382	0.649355	907.0
macro avg	0.801577	0.821495	0.801614	4026.0
micro avg	0.816359	0.816359	0.816359	4026.0
weighted avg	0.815948	0.833092	0.816359	4026.0

The result of Model Number:Dataset Number:Featuregroup Number:Rescale of 44 : 1 : 3 : 0

	f1-score	precision	recall	support
AFIB	0.836905	0.828700	0.894819	204.0
GSVT	0.750414	0.742071	0.759135	300.0
SB	0.964329	0.957306	0.971461	892.0
SR	0.948156	0.954723	0.941756	681.0
macro avg	0.874951	0.870700	0.891793	2077.0
micro avg	0.923526	0.923526	0.923526	2077.0
weighted avg	0.915613	0.912739	0.923526	2077.0

The result of Model Number:Dataset Number:Featuregroup Number:Rescale of 44 : 1 : 3 : 1

	f1-score	precision	recall	support
AFIB	0.837689	0.841678	0.833775	875.0

GSVT 0.848038 0.832626 0.864117 691.0
SB 0.897288 0.893852 0.900753 1553.0
SR 0.870766 0.885671 0.856369 907.0
macro avg 0.863445 0.863457 0.863754 4026.0
micro avg 0.869909 0.869909 0.869909 4026.0
weighted avg 0.869907 0.870161 0.869909 4026.0
The result of Model Number:Dataset Number:Featuregroup Number:Rescale of 44 : 1 : 4 : 0

	f1-score	precision	recall	support
AFIB	0.560119	0.570580	0.620137	204.0
GSVT	0.672644	0.659018	0.687186	300.0
SB	0.553349	0.548179	0.558623	892.0
SR	0.673502	0.679129	0.668062	681.0
macro avg	0.614904	0.614226	0.633502	2077.0
micro avg	0.619117	0.619117	0.619117	2077.0
weighted avg	0.610640	0.609324	0.619117	2077.0

The result of Model Number:Dataset Number:Featuregroup Number:Rescale of 44 : 1 : 4 : 1

	f1-score	precision	recall	support
AFIB	0.668678	0.667596	0.669823	875.0
GSVT	0.674805	0.677488	0.672177	691.0
SB	0.708663	0.706087	0.711263	1553.0
SR	0.697071	0.700598	0.693619	907.0
macro avg	0.687304	0.687942	0.686720	4026.0
micro avg	0.691573	0.691573	0.691573	4026.0
weighted avg	0.691550	0.691576	0.691573	4026.0

The result of Model Number:Dataset Number:Featuregroup Number:Rescale of 44 : 1 : 5 : 0

	f1-score	precision	recall	support
AFIB	0.847513	0.855741	0.839967	204.0
GSVT	0.872918	0.868791	0.877314	300.0
SB	0.890594	0.889601	0.891592	892.0
SR	0.882758	0.883673	0.881851	681.0
macro avg	0.873446	0.874451	0.872681	2077.0
micro avg	0.881265	0.881265	0.881265	2077.0
weighted avg	0.881240	0.881326	0.881265	2077.0

The result of Model Number:Dataset Number:Featuregroup Number:Rescale of 44 : 1 : 5 : 1

	f1-score	precision	recall	support
AFIB	0.823289	0.823436	0.823178	875.0
GSVT	0.831999	0.834810	0.829260	691.0
SB	0.873972	0.870959	0.877011	1553.0
SR	0.857376	0.860268	0.854535	907.0
macro avg	0.846659	0.847368	0.845996	4026.0

micro avg 0.852052 0.852052 0.852052 4026.0
weighted avg 0.852014 0.852017 0.852052 4026.0
The result of Model Number:Dataset Number:Featuregroup Number:Rescale of 44 : 1 : 6 : 0

	f1-score	precision	recall	support
AFIB	0.793090	0.762770	0.842978	875.0
GSVT	0.821775	0.793414	0.860244	691.0
SB	0.915610	0.899449	0.933369	1553.0
SR	0.775501	0.932495	0.669541	907.0
macro avg	0.826494	0.847032	0.826533	4026.0
micro avg	0.841736	0.841736	0.841736	4026.0
weighted avg	0.841312	0.858989	0.841736	4026.0

The result of Model Number:Dataset Number:Featuregroup Number:Rescale of 44 : 1 : 6 : 1

	f1-score	precision	recall	support
AFIB	0.877986	0.884946	0.871158	875.0
GSVT	0.889117	0.885197	0.893102	691.0
SB	0.945472	0.941734	0.949246	1553.0
SR	0.922238	0.924844	0.919691	907.0
macro avg	0.908703	0.909180	0.908299	4026.0
micro avg	0.915980	0.915980	0.915980	4026.0
weighted avg	0.915898	0.915883	0.915980	4026.0

The result of Model Number:Dataset Number:Featuregroup Number:Rescale of 44 : 1 : 7 : 0

	f1-score	precision	recall	support
AFIB	0.687613	0.678980	0.696668	875.0
GSVT	0.637969	0.657285	0.621889	691.0
SB	0.686875	0.636198	0.748141	1553.0
SR	0.534067	0.653658	0.455410	907.0
macro avg	0.636631	0.656530	0.630527	4026.0
micro avg	0.649337	0.649337	0.649337	4026.0
weighted avg	0.644216	0.653049	0.649337	4026.0

The result of Model Number:Dataset Number:Featuregroup Number:Rescale of 44 : 1 : 7 : 1

	f1-score	precision	recall	support
AFIB	0.738905	0.750903	0.727323	875.0
GSVT	0.785847	0.788309	0.783447	691.0
SB	0.854962	0.852015	0.857933	1553.0
SR	0.809399	0.800384	0.818669	907.0
macro avg	0.797278	0.797903	0.796843	4026.0
micro avg	0.807916	0.807916	0.807916	4026.0
weighted avg	0.807611	0.807474	0.807916	4026.0

The result of Model Number:Dataset Number:Featuregroup Number:Rescale of 44 : 1 : 8 : 0

	f1-score	precision	recall	support
AFIB	0.645759	0.645168	0.646429	875.0
GSVT	0.658996	0.657717	0.660348	691.0
SB	0.699280	0.697723	0.700856	1553.0
SR	0.680194	0.684631	0.675838	907.0
macro avg	0.671057	0.671310	0.670868	4026.0
micro avg	0.676438	0.676438	0.676438	4026.0
weighted avg	0.676434	0.676485	0.676438	4026.0

The result of Model Number:Dataset Number:Featuregroup Number:Rescale of 44 : 1 : 8 : 1

	f1-score	precision	recall	support
AFIB	0.759875	0.778826	0.741882	875.0
GSVT	0.808374	0.811473	0.805337	691.0
SB	0.875401	0.871163	0.879684	1553.0
SR	0.829491	0.815665	0.843848	907.0
macro avg	0.818286	0.819282	0.817688	4026.0
micro avg	0.828901	0.828901	0.828901	4026.0
weighted avg	0.828446	0.828347	0.828901	4026.0

The result of Model Number:Dataset Number:Featuregroup Number:Rescale of 44 : 1 : 9 : 0

	f1-score	precision	recall	support
AFIB	0.696058	0.721952	0.672269	204.0
GSVT	0.645915	0.675889	0.618547	300.0
SB	0.676622	0.635811	0.723043	892.0
SR	0.594205	0.635490	0.558026	681.0
macro avg	0.653200	0.667285	0.642971	2077.0
micro avg	0.648858	0.648858	0.648858	2077.0
weighted avg	0.647073	0.649955	0.648858	2077.0

The result of Model Number:Dataset Number:Featuregroup Number:Rescale of 44 : 1 : 9 : 1

	f1-score	precision	recall	support
AFIB	0.723583	0.714582	0.732895	875.0
GSVT	0.746237	0.755582	0.737195	691.0
SB	0.796131	0.795268	0.797001	1553.0
SR	0.774658	0.778630	0.770730	907.0
macro avg	0.760152	0.761015	0.759455	4026.0
micro avg	0.766885	0.766885	0.766885	4026.0
weighted avg	0.766962	0.767172	0.766885	4026.0

The result of Model Number:Dataset Number:Featuregroup Number:Rescale of 44 : 1 : 10 : 0

	f1-score	precision	recall	support
AFIB	0.773197	0.762842	0.783886	875.0
GSVT	0.790484	0.796654	0.784423	691.0
SB	0.839274	0.836648	0.841921	1553.0

SR 0.818978 0.829835 0.808416 907.0
macro avg 0.805483 0.806495 0.804661 4026.0
micro avg 0.811891 0.811891 0.811891 4026.0
weighted avg 0.811967 0.812208 0.811891 4026.0
The result of Model Number:Dataset Number:Featuregroup Number:Rescale of 44 : 1 : 10 : 1

	f1-score	precision	recall	support
AFIB	0.796117	0.812848	0.780092	875.0
GSVT	0.847772	0.850684	0.844921	691.0
SB	0.923659	0.919306	0.928058	1553.0
SR	0.874347	0.862703	0.886383	907.0
macro avg	0.860474	0.861385	0.859863	4026.0
micro avg	0.872241	0.872241	0.872241	4026.0
weighted avg	0.871805	0.871639	0.872241	4026.0

The result of Model Number:Dataset Number:Featuregroup Number:Rescale of 45 : 0 : 0 : 0

	f1-score	precision	recall	support
AFIB	0.609440	0.595106	0.641607	875.0
GSVT	0.585616	0.580217	0.598961	691.0
SB	0.635141	0.590419	0.687924	1553.0
SR	0.396018	0.496671	0.329796	907.0
macro avg	0.556554	0.565603	0.564572	4026.0
micro avg	0.581907	0.581907	0.581907	4026.0
weighted avg	0.567184	0.568567	0.581907	4026.0

The result of Model Number:Dataset Number:Featuregroup Number:Rescale of 45 : 0 : 0 : 1

	f1-score	precision	recall	support
AFIB	0.642314	0.633417	0.651490	875.0
GSVT	0.656560	0.661873	0.651334	691.0
SB	0.696930	0.694928	0.698949	1553.0
SR	0.679976	0.688826	0.671357	907.0
macro avg	0.668945	0.669761	0.668283	4026.0
micro avg	0.674246	0.674246	0.674246	4026.0
weighted avg	0.674312	0.674511	0.674246	4026.0

The result of Model Number:Dataset Number:Featuregroup Number:Rescale of 45 : 0 : 1 : 0

	f1-score	precision	recall	support
AFIB	0.841564	0.836530	0.846773	875.0
GSVT	0.848456	0.853037	0.844047	691.0
SB	0.899236	0.896607	0.901894	1553.0
SR	0.878917	0.885230	0.872710	907.0
macro avg	0.867043	0.867851	0.866356	4026.0
micro avg	0.873411	0.873411	0.873411	4026.0
weighted avg	0.873409	0.873509	0.873411	4026.0

The result of Model Number:Dataset Number:Featuregroup Number:Rescale of 45 : 0 : 1 : 1

	f1-score	precision	recall	support
AFIB	0.912665	0.932501	0.893905	204.0
GSVT	0.950339	0.944733	0.956038	300.0
SB	0.970756	0.968603	0.972930	892.0
SR	0.961024	0.960482	0.961591	681.0
macro avg	0.948696	0.951580	0.946116	2077.0
micro avg	0.959011	0.959011	0.959011	2077.0
weighted avg	0.958911	0.958947	0.959011	2077.0

The result of Model Number:Dataset Number:Featuregroup Number:Rescale of 45 : 0 : 2 : 0

	f1-score	precision	recall	support
AFIB	0.759652	0.767027	0.752889	204.0
GSVT	0.782423	0.778725	0.786364	300.0
SB	0.798268	0.797377	0.799162	892.0
SR	0.791244	0.792063	0.790430	681.0
macro avg	0.782897	0.783798	0.782211	2077.0
micro avg	0.789906	0.789906	0.789906	2077.0
weighted avg	0.789884	0.789960	0.789906	2077.0

The result of Model Number:Dataset Number:Featuregroup Number:Rescale of 45 : 0 : 2 : 1

	f1-score	precision	recall	support
AFIB	0.809444	0.810538	0.808506	875.0
GSVT	0.822051	0.821628	0.822504	691.0
SB	0.858547	0.855475	0.861644	1553.0
SR	0.837987	0.842685	0.833396	907.0
macro avg	0.832007	0.832582	0.831513	4026.0
micro avg	0.837014	0.837014	0.837014	4026.0
weighted avg	0.836979	0.837018	0.837014	4026.0

The result of Model Number:Dataset Number:Featuregroup Number:Rescale of 45 : 0 : 3 : 0

	f1-score	precision	recall	support
AFIB	0.668669	0.705319	0.636084	204.0
GSVT	0.636715	0.659038	0.615933	300.0
SB	0.654924	0.629664	0.682306	892.0
SR	0.582900	0.598714	0.567933	681.0
macro avg	0.635802	0.648184	0.625564	2077.0
micro avg	0.630679	0.630679	0.630679	2077.0
weighted avg	0.630029	0.631190	0.630679	2077.0

The result of Model Number:Dataset Number:Featuregroup Number:Rescale of 45 : 0 : 3 : 1

	f1-score	precision	recall	support
AFIB	0.689164	0.708162	0.671297	204.0

GSVT 0.707505 0.694408 0.721173 300.0
SB 0.718979 0.712258 0.725831 892.0
SR 0.700907 0.710430 0.691649 681.0
macro avg 0.704139 0.706314 0.702487 2077.0
micro avg 0.708594 0.708594 0.708594 2077.0
weighted avg 0.708468 0.708678 0.708594 2077.0
The result of Model Number:Dataset Number:Featuregroup Number:Rescale of 45 : 0 : 4 : 0
f1-score precision recall support
AFIB 0.683044 0.684268 0.681847 875.0
GSVT 0.654061 0.657496 0.650679 691.0
SB 0.691837 0.653780 0.734606 1553.0
SR 0.574574 0.646725 0.516957 907.0
macro avg 0.650879 0.660567 0.646022 4026.0
micro avg 0.659701 0.659701 0.659701 4026.0
weighted avg 0.657025 0.659455 0.659701 4026.0
The result of Model Number:Dataset Number:Featuregroup Number:Rescale of 45 : 0 : 4 : 1
f1-score precision recall support
AFIB 0.664606 0.690432 0.640691 875.0
GSVT 0.714473 0.709197 0.719923 691.0
SB 0.785151 0.781106 0.789244 1553.0
SR 0.738899 0.724809 0.753627 907.0
macro avg 0.725782 0.726386 0.725871 4026.0
micro avg 0.737036 0.737036 0.737036 4026.0
weighted avg 0.736401 0.736374 0.737036 4026.0
The result of Model Number:Dataset Number:Featuregroup Number:Rescale of 45 : 0 : 5 : 0
f1-score precision recall support
AFIB 0.738925 0.780961 0.701674 204.0
GSVT 0.780878 0.772279 0.789710 300.0
SB 0.792493 0.784608 0.800566 892.0
SR 0.783287 0.785965 0.780751 681.0
macro avg 0.773896 0.780953 0.768175 2077.0
micro avg 0.782788 0.782788 0.782788 2077.0
weighted avg 0.782536 0.782914 0.782788 2077.0
The result of Model Number:Dataset Number:Featuregroup Number:Rescale of 45 : 0 : 5 : 1
f1-score precision recall support
AFIB 0.678848 0.672192 0.725824 204.0
GSVT 0.608692 0.601924 0.615765 300.0
SB 0.782207 0.776510 0.787992 892.0
SR 0.769088 0.774415 0.763897 681.0
macro avg 0.709709 0.706260 0.723370 2077.0

micro avg 0.749109 0.749109 0.749109 2077.0
weighted avg 0.742691 0.740360 0.749109 2077.0
The result of Model Number:Dataset Number:Featuregroup Number:Rescale of 45 : 0 : 6 : 0

	f1-score	precision	recall	support
AFIB	0.792030	0.825527	0.761408	204.0
GSVT	0.729402	0.730679	0.739071	300.0
SB	0.785268	0.747859	0.827379	892.0
SR	0.694216	0.750130	0.647315	681.0
macro avg	0.750229	0.763549	0.743793	2077.0
micro avg	0.749106	0.749106	0.749106	2077.0
weighted avg	0.748009	0.753750	0.749106	2077.0

The result of Model Number:Dataset Number:Featuregroup Number:Rescale of 45 : 0 : 6 : 1

	f1-score	precision	recall	support
AFIB	0.748687	0.780351	0.719740	204.0
GSVT	0.689486	0.690693	0.698626	300.0
SB	0.742295	0.706933	0.782102	892.0
SR	0.656226	0.709080	0.611891	681.0
macro avg	0.709173	0.721764	0.703090	2077.0
micro avg	0.708112	0.708112	0.708112	2077.0
weighted avg	0.707075	0.712502	0.708112	2077.0

The result of Model Number:Dataset Number:Featuregroup Number:Rescale of 45 : 0 : 7 : 0

	f1-score	precision	recall	support
AFIB	0.925743	0.928164	0.923516	204.0
GSVT	0.946630	0.942965	0.950402	300.0
SB	0.967860	0.966568	0.969160	892.0
SR	0.960108	0.962806	0.957430	681.0
macro avg	0.950085	0.950126	0.950127	2077.0
micro avg	0.958122	0.958122	0.958122	2077.0
weighted avg	0.958115	0.958154	0.958122	2077.0

The result of Model Number:Dataset Number:Featuregroup Number:Rescale of 45 : 0 : 7 : 1

	f1-score	precision	recall	support
AFIB	0.785464	0.802962	0.768939	204.0
GSVT	0.795155	0.783509	0.807483	300.0
SB	0.813123	0.805042	0.821403	892.0
SR	0.798124	0.809588	0.787005	681.0
macro avg	0.797966	0.800275	0.796208	2077.0
micro avg	0.802961	0.802961	0.802961	2077.0
weighted avg	0.802893	0.803218	0.802961	2077.0

The result of Model Number:Dataset Number:Featuregroup Number:Rescale of 45 : 0 : 8 : 0

	f1-score	precision	recall	support
AFIB	0.663450	0.666469	0.660499	875.0
GSVT	0.666674	0.660029	0.673511	691.0
SB	0.702675	0.695018	0.710503	1553.0
SR	0.672033	0.687943	0.656845	907.0
macro avg	0.676208	0.677365	0.675339	4026.0
micro avg	0.681198	0.681198	0.681198	4026.0
weighted avg	0.681068	0.681214	0.681198	4026.0

The result of Model Number:Dataset Number:Featuregroup Number:Rescale of 45 : 0 : 8 : 1

	f1-score	precision	recall	support
AFIB	0.662082	0.664559	0.659906	875.0
GSVT	0.676986	0.683517	0.670714	691.0
SB	0.719847	0.712297	0.727615	1553.0
SR	0.694920	0.701036	0.689149	907.0
macro avg	0.688459	0.690352	0.686846	4026.0
micro avg	0.694467	0.694467	0.694467	4026.0
weighted avg	0.694321	0.694445	0.694467	4026.0

The result of Model Number:Dataset Number:Featuregroup Number:Rescale of 45 : 0 : 9 : 0

	f1-score	precision	recall	support
AFIB	0.722881	0.724892	0.720919	875.0
GSVT	0.739440	0.739235	0.739652	691.0
SB	0.787527	0.780125	0.795079	1553.0
SR	0.766660	0.777715	0.755923	907.0
macro avg	0.754127	0.755492	0.752893	4026.0
micro avg	0.760627	0.760627	0.760627	4026.0
weighted avg	0.760522	0.760560	0.760627	4026.0

The result of Model Number:Dataset Number:Featuregroup Number:Rescale of 45 : 0 : 9 : 1

	f1-score	precision	recall	support
AFIB	0.680936	0.681701	0.680295	204.0
GSVT	0.696569	0.695014	0.698184	300.0
SB	0.711333	0.710384	0.712289	892.0
SR	0.706006	0.707784	0.704245	681.0
macro avg	0.698711	0.698721	0.698753	2077.0
micro avg	0.704472	0.704472	0.704472	2077.0
weighted avg	0.704468	0.704494	0.704472	2077.0

The result of Model Number:Dataset Number:Featuregroup Number:Rescale of 45 : 0 : 10 : 0

	f1-score	precision	recall	support
AFIB	0.689028	0.703339	0.675447	875.0
GSVT	0.671940	0.648602	0.726789	691.0
SB	0.791986	0.788415	0.795603	1553.0

SR	0.602105	0.592384	0.612205	907.0
macro avg	0.688765	0.683185	0.702511	4026.0
micro avg	0.716361	0.716361	0.716361	4026.0
weighted avg	0.706228	0.701765	0.716361	4026.0

The result of Model Number:Dataset Number:Featuregroup Number:Rescale of 45 : 0 : 10 : 1

	f1-score	precision	recall	support
AFIB	0.717099	0.728744	0.705859	875.0
GSVT	0.762656	0.765046	0.760327	691.0
SB	0.829731	0.826872	0.832614	1553.0
SR	0.785513	0.776764	0.794509	907.0
macro avg	0.773750	0.774356	0.773327	4026.0
micro avg	0.784074	0.784074	0.784074	4026.0
weighted avg	0.783778	0.783645	0.784074	4026.0

The result of Model Number:Dataset Number:Featuregroup Number:Rescale of 45 : 1 : 0 : 0

	f1-score	precision	recall	support
AFIB	0.816334	0.805912	0.827077	875.0
GSVT	0.830662	0.835426	0.825975	691.0
SB	0.889173	0.885438	0.892947	1553.0
SR	0.862992	0.876960	0.849476	907.0
macro avg	0.849790	0.850934	0.848869	4026.0
micro avg	0.857343	0.857343	0.857343	4026.0
weighted avg	0.857402	0.857660	0.857343	4026.0

The result of Model Number:Dataset Number:Featuregroup Number:Rescale of 45 : 1 : 0 : 1

	f1-score	precision	recall	support
AFIB	0.669080	0.670431	0.667749	204.0
GSVT	0.687694	0.685650	0.689755	300.0
SB	0.699089	0.698466	0.699715	892.0
SR	0.693080	0.694411	0.691763	681.0
macro avg	0.687236	0.687239	0.687245	2077.0
micro avg	0.692530	0.692530	0.692530	2077.0
weighted avg	0.692526	0.692532	0.692530	2077.0

The result of Model Number:Dataset Number:Featuregroup Number:Rescale of 45 : 1 : 1 : 0

	f1-score	precision	recall	support
AFIB	0.673460	0.680638	0.666541	204.0
GSVT	0.685845	0.682083	0.689725	300.0
SB	0.701677	0.700507	0.702854	892.0
SR	0.695827	0.696961	0.694707	681.0
macro avg	0.689202	0.690047	0.688457	2077.0
micro avg	0.694720	0.694720	0.694720	2077.0
weighted avg	0.694701	0.694732	0.694720	2077.0

The result of Model Number:Dataset Number:Featuregroup Number:Rescale of 45 : 1 : 1 : 1

	f1-score	precision	recall	support
AFIB	0.762009	0.772796	0.752144	204.0
GSVT	0.777829	0.763508	0.792841	300.0
SB	0.794408	0.785624	0.803403	892.0
SR	0.777712	0.793552	0.762539	681.0
macro avg	0.777989	0.778870	0.777732	2077.0
micro avg	0.783444	0.783444	0.783444	2077.0
weighted avg	0.783357	0.783769	0.783444	2077.0

The result of Model Number:Dataset Number:Featuregroup Number:Rescale of 45 : 1 : 2 : 0

	f1-score	precision	recall	support
AFIB	0.826286	0.829377	0.823569	875.0
GSVT	0.844885	0.853036	0.837058	691.0
SB	0.898377	0.888954	0.908071	1553.0
SR	0.867267	0.874900	0.860065	907.0
macro avg	0.859204	0.861567	0.857191	4026.0
micro avg	0.866702	0.866702	0.866702	4026.0
weighted avg	0.866519	0.866675	0.866702	4026.0

The result of Model Number:Dataset Number:Featuregroup Number:Rescale of 45 : 1 : 2 : 1

	f1-score	precision	recall	support
AFIB	0.784475	0.774982	0.794239	875.0
GSVT	0.795094	0.803246	0.787168	691.0
SB	0.844470	0.841722	0.847243	1553.0
SR	0.826000	0.834487	0.817712	907.0
macro avg	0.812510	0.813609	0.811590	4026.0
micro avg	0.818759	0.818759	0.818759	4026.0
weighted avg	0.818795	0.818983	0.818759	4026.0

The result of Model Number:Dataset Number:Featuregroup Number:Rescale of 45 : 1 : 3 : 0

	f1-score	precision	recall	support
AFIB	0.822286	0.822236	0.822387	875.0
GSVT	0.824065	0.819610	0.828583	691.0
SB	0.863434	0.857689	0.869268	1553.0
SR	0.840507	0.854297	0.827186	907.0
macro avg	0.837573	0.838458	0.836856	4026.0
micro avg	0.842616	0.842616	0.842616	4026.0
weighted avg	0.842569	0.842684	0.842616	4026.0

The result of Model Number:Dataset Number:Featuregroup Number:Rescale of 45 : 1 : 3 : 1

	f1-score	precision	recall	support
AFIB	0.887721	0.887879	0.887601	875.0

GSVT 0.897113 0.900144 0.894159 691.0
SB 0.942371 0.939122 0.945647 1553.0
SR 0.924476 0.927594 0.921413 907.0
macro avg 0.912920 0.913685 0.912205 4026.0
micro avg 0.918735 0.918735 0.918735 4026.0
weighted avg 0.918694 0.918698 0.918735 4026.0
The result of Model Number:Dataset Number:Featuregroup Number:Rescale of 45 : 1 : 4 : 0
f1-score precision recall support
AFIB 0.813637 0.819722 0.807674 204.0
GSVT 0.834427 0.830023 0.838878 300.0
SB 0.848509 0.847374 0.849649 892.0
SR 0.841036 0.842648 0.839431 681.0
macro avg 0.834402 0.834942 0.833908 2077.0
micro avg 0.840621 0.840621 0.840621 2077.0
weighted avg 0.840600 0.840602 0.840621 2077.0
The result of Model Number:Dataset Number:Featuregroup Number:Rescale of 45 : 1 : 4 : 1
f1-score precision recall support
AFIB 0.757610 0.749151 0.766271 875.0
GSVT 0.772683 0.779761 0.765745 691.0
SB 0.824102 0.821419 0.826807 1553.0
SR 0.802738 0.810780 0.794857 907.0
macro avg 0.789284 0.790278 0.788420 4026.0
micro avg 0.795972 0.795972 0.795972 4026.0
weighted avg 0.796013 0.796166 0.795972 4026.0
The result of Model Number:Dataset Number:Featuregroup Number:Rescale of 45 : 1 : 5 : 0
f1-score precision recall support
AFIB 0.896603 0.912170 0.881672 204.0
GSVT 0.914271 0.911566 0.917012 300.0
SB 0.925053 0.921761 0.928371 892.0
SR 0.917531 0.918490 0.916596 681.0
macro avg 0.913365 0.915997 0.910913 2077.0
micro avg 0.918283 0.918283 0.918283 2077.0
weighted avg 0.918235 0.918274 0.918283 2077.0
The result of Model Number:Dataset Number:Featuregroup Number:Rescale of 45 : 1 : 5 : 1
f1-score precision recall support
AFIB 0.816215 0.836883 0.796619 204.0
GSVT 0.833942 0.824873 0.843350 300.0
SB 0.857430 0.853151 0.861756 892.0
SR 0.844141 0.847928 0.840421 681.0
macro avg 0.837932 0.840709 0.835536 2077.0

micro avg	0.845704	0.845704	0.845704	2077.0
weighted avg	0.845632	0.845756	0.845704	2077.0

The result of Model Number:Dataset Number:Featuregroup Number:Rescale of 45 : 1 : 6 : 0

	f1-score	precision	recall	support
AFIB	0.801637	0.792006	0.811534	875.0
GSVT	0.817055	0.823670	0.810550	691.0
SB	0.870917	0.867584	0.874280	1553.0
SR	0.850497	0.861372	0.839904	907.0
macro avg	0.835026	0.836158	0.834067	4026.0
micro avg	0.841960	0.841960	0.841960	4026.0
weighted avg	0.842015	0.842221	0.841960	4026.0

The result of Model Number:Dataset Number:Featuregroup Number:Rescale of 45 : 1 : 6 : 1

	f1-score	precision	recall	support
AFIB	0.930051	0.931097	0.929176	204.0
GSVT	0.951404	0.949280	0.953610	300.0
SB	0.971570	0.970273	0.972875	892.0
SR	0.964293	0.966722	0.961889	681.0
macro avg	0.954330	0.954343	0.954388	2077.0
micro avg	0.962199	0.962199	0.962199	2077.0
weighted avg	0.962193	0.962229	0.962199	2077.0

The result of Model Number:Dataset Number:Featuregroup Number:Rescale of 45 : 1 : 7 : 0

	f1-score	precision	recall	support
AFIB	0.692427	0.665955	0.735982	875.0
GSVT	0.717471	0.692709	0.751057	691.0
SB	0.799396	0.785285	0.814901	1553.0
SR	0.677070	0.814138	0.584559	907.0
macro avg	0.721591	0.739522	0.721625	4026.0
micro avg	0.734898	0.734898	0.734898	4026.0
weighted avg	0.734528	0.749961	0.734898	4026.0

The result of Model Number:Dataset Number:Featuregroup Number:Rescale of 45 : 1 : 7 : 1

	f1-score	precision	recall	support
AFIB	0.695710	0.713627	0.678699	204.0
GSVT	0.638436	0.610016	0.720474	300.0
SB	0.732470	0.730756	0.734193	892.0
SR	0.581082	0.581191	0.580980	681.0
macro avg	0.661924	0.658898	0.678587	2077.0
micro avg	0.676526	0.676526	0.676526	2077.0
weighted avg	0.665641	0.662595	0.676526	2077.0

The result of Model Number:Dataset Number:Featuregroup Number:Rescale of 45 : 1 : 8 : 0

	f1-score	precision	recall	support
AFIB	0.792728	0.772442	0.814181	875.0
GSVT	0.749372	0.791845	0.711399	691.0
SB	0.794597	0.731220	0.870022	1553.0
SR	0.634749	0.770986	0.539544	907.0
macro avg	0.742862	0.766623	0.733786	4026.0
micro avg	0.756209	0.756209	0.756209	4026.0
weighted avg	0.750417	0.759543	0.756209	4026.0

The result of Model Number:Dataset Number:Featuregroup Number:Rescale of 45 : 1 : 8 : 1

	f1-score	precision	recall	support
AFIB	0.769029	0.759374	0.779156	875.0
GSVT	0.713507	0.735109	0.695523	691.0
SB	0.768203	0.711526	0.836723	1553.0
SR	0.597303	0.731053	0.509333	907.0
macro avg	0.712010	0.734265	0.705184	4026.0
micro avg	0.726220	0.726220	0.726220	4026.0
weighted avg	0.720493	0.730372	0.726220	4026.0

The result of Model Number:Dataset Number:Featuregroup Number:Rescale of 45 : 1 : 9 : 0

	f1-score	precision	recall	support
AFIB	0.892032	0.900692	0.884090	204.0
GSVT	0.918771	0.914428	0.923399	300.0
SB	0.937377	0.936331	0.938427	892.0
SR	0.929129	0.930091	0.928174	681.0
macro avg	0.919327	0.920386	0.918522	2077.0
micro avg	0.927558	0.927558	0.927558	2077.0
weighted avg	0.927531	0.927621	0.927558	2077.0

The result of Model Number:Dataset Number:Featuregroup Number:Rescale of 45 : 1 : 9 : 1

	f1-score	precision	recall	support
AFIB	0.738470	0.757488	0.720414	204.0
GSVT	0.677676	0.647510	0.764756	300.0
SB	0.777490	0.775671	0.779319	892.0
SR	0.616797	0.616913	0.616689	681.0
macro avg	0.702608	0.699395	0.720295	2077.0
micro avg	0.718107	0.718107	0.718107	2077.0
weighted avg	0.706553	0.703320	0.718107	2077.0

The result of Model Number:Dataset Number:Featuregroup Number:Rescale of 45 : 1 : 10 : 0

	f1-score	precision	recall	support
AFIB	0.849890	0.859334	0.841087	204.0
GSVT	0.876216	0.871228	0.881516	300.0
SB	0.893647	0.892449	0.894849	892.0

SR 0.886045 0.887232 0.884873 681.0
macro avg 0.876449 0.877561 0.875581 2077.0
micro avg 0.884372 0.884372 0.884372 2077.0
weighted avg 0.884339 0.884421 0.884372 2077.0
The result of Model Number:Dataset Number:Featuregroup Number:Rescale of 45 : 1 : 10 : 1

	f1-score	precision	recall	support
AFIB	0.693913	0.715363	0.674318	204.0
GSVT	0.647759	0.661841	0.634742	300.0
SB	0.683222	0.652900	0.716533	892.0
SR	0.615167	0.646778	0.586589	681.0
macro avg	0.660015	0.669220	0.653045	2077.0
micro avg	0.657967	0.657967	0.657967	2077.0
weighted avg	0.656836	0.658319	0.657967	2077.0

The result of Model Number:Dataset Number:Featuregroup Number:Rescale of 46 : 0 : 0 : 0

	f1-score	precision	recall	support
AFIB	0.686555	0.701849	0.672110	204.0
GSVT	0.695025	0.684846	0.705801	300.0
SB	0.710731	0.703667	0.717968	892.0
SR	0.697620	0.707640	0.687901	681.0
macro avg	0.697483	0.699500	0.695945	2077.0
micro avg	0.701848	0.701848	0.701848	2077.0
weighted avg	0.701789	0.702073	0.701848	2077.0

The result of Model Number:Dataset Number:Featuregroup Number:Rescale of 46 : 0 : 0 : 1

	f1-score	precision	recall	support
AFIB	0.716497	0.705788	0.727562	875.0
GSVT	0.730234	0.736479	0.724106	691.0
SB	0.780238	0.777552	0.782949	1553.0
SR	0.757401	0.768698	0.746458	907.0
macro avg	0.746093	0.747129	0.745269	4026.0
micro avg	0.752591	0.752591	0.752591	4026.0
weighted avg	0.752658	0.752911	0.752591	4026.0

The result of Model Number:Dataset Number:Featuregroup Number:Rescale of 46 : 0 : 1 : 0

	f1-score	precision	recall	support
AFIB	0.881637	0.870073	0.893554	875.0
GSVT	0.895135	0.905085	0.885417	691.0
SB	0.949406	0.947165	0.951663	1553.0
SR	0.925636	0.933967	0.917465	907.0
macro avg	0.912954	0.914072	0.912025	4026.0
micro avg	0.919960	0.919960	0.919960	4026.0
weighted avg	0.920008	0.920214	0.919960	4026.0

The result of Model Number:Dataset Number:Featuregroup Number:Rescale of 46 : 0 : 1 : 1

	f1-score	precision	recall	support
AFIB	0.729896	0.820703	0.657825	204.0
GSVT	0.865968	0.847476	0.885323	300.0
SB	0.924437	0.919724	0.929202	892.0
SR	0.903698	0.892254	0.915497	681.0
macro avg	0.856000	0.870039	0.846962	2077.0
micro avg	0.891717	0.891717	0.891717	2077.0
weighted avg	0.890084	0.890556	0.891717	2077.0

The result of Model Number:Dataset Number:Featuregroup Number:Rescale of 46 : 0 : 2 : 0

	f1-score	precision	recall	support
AFIB	0.796621	0.812602	0.781415	204.0
GSVT	0.815083	0.806611	0.823779	300.0
SB	0.830158	0.826929	0.833415	892.0
SR	0.821435	0.824862	0.818055	681.0
macro avg	0.815824	0.817751	0.814166	2077.0
micro avg	0.821880	0.821880	0.821880	2077.0
weighted avg	0.821826	0.821909	0.821880	2077.0

The result of Model Number:Dataset Number:Featuregroup Number:Rescale of 46 : 0 : 2 : 1

	f1-score	precision	recall	support
AFIB	0.771557	0.776602	0.766624	204.0
GSVT	0.790888	0.789066	0.792727	300.0
SB	0.805005	0.803844	0.806176	892.0
SR	0.798661	0.799495	0.797842	681.0
macro avg	0.791528	0.792252	0.790843	2077.0
micro avg	0.797617	0.797617	0.797617	2077.0
weighted avg	0.797601	0.797608	0.797617	2077.0

The result of Model Number:Dataset Number:Featuregroup Number:Rescale of 46 : 0 : 3 : 0

	f1-score	precision	recall	support
AFIB	0.706107	0.701215	0.711210	875.0
GSVT	0.717001	0.719910	0.714230	691.0
SB	0.777947	0.769858	0.786212	1553.0
SR	0.738668	0.755856	0.722312	907.0
macro avg	0.734931	0.736710	0.733491	4026.0
micro avg	0.743161	0.743161	0.743161	4026.0
weighted avg	0.743024	0.743212	0.743161	4026.0

The result of Model Number:Dataset Number:Featuregroup Number:Rescale of 46 : 0 : 3 : 1

	f1-score	precision	recall	support
AFIB	0.782095	0.789091	0.775319	875.0

GSVT 0.797032 0.781876 0.812939 691.0
SB 0.845983 0.838654 0.853444 1553.0
SR 0.811236 0.829889 0.793440 907.0
macro avg 0.809086 0.809877 0.808785 4026.0
micro avg 0.815994 0.815994 0.815994 4026.0
weighted avg 0.815868 0.816162 0.815994 4026.0
The result of Model Number:Dataset Number:Featuregroup Number:Rescale of 46 : 0 : 4 : 0

	f1-score	precision	recall	support
AFIB	0.709378	0.718089	0.700987	204.0
GSVT	0.723666	0.720576	0.726801	300.0
SB	0.732426	0.730228	0.734641	892.0
SR	0.726262	0.727990	0.724565	681.0
macro avg	0.722933	0.724221	0.721749	2077.0
micro avg	0.726900	0.726900	0.726900	2077.0
weighted avg	0.726876	0.726908	0.726900	2077.0

The result of Model Number:Dataset Number:Featuregroup Number:Rescale of 46 : 0 : 4 : 1

	f1-score	precision	recall	support
AFIB	0.731467	0.722679	0.740498	875.0
GSVT	0.745536	0.751572	0.739600	691.0
SB	0.794683	0.791641	0.797752	1553.0
SR	0.776050	0.785973	0.766385	907.0
macro avg	0.761934	0.762966	0.761059	4026.0
micro avg	0.768261	0.768261	0.768261	4026.0
weighted avg	0.768311	0.768499	0.768261	4026.0

The result of Model Number:Dataset Number:Featuregroup Number:Rescale of 46 : 0 : 5 : 0

	f1-score	precision	recall	support
AFIB	0.857612	0.878141	0.839276	204.0
GSVT	0.882866	0.870427	0.896035	300.0
SB	0.905805	0.896603	0.915241	892.0
SR	0.884156	0.896922	0.871818	681.0
macro avg	0.882610	0.885523	0.880592	2077.0
micro avg	0.890768	0.890768	0.890768	2077.0
weighted avg	0.890660	0.891113	0.890768	2077.0

The result of Model Number:Dataset Number:Featuregroup Number:Rescale of 46 : 0 : 5 : 1

	f1-score	precision	recall	support
AFIB	0.778487	0.773396	0.783713	875.0
GSVT	0.801625	0.808446	0.795025	691.0
SB	0.853438	0.851579	0.855307	1553.0
SR	0.832639	0.835982	0.829335	907.0
macro avg	0.816547	0.817351	0.815845	4026.0

micro avg 0.823549 0.823549 0.823549 4026.0
weighted avg 0.823570 0.823670 0.823549 4026.0
The result of Model Number:Dataset Number:Featuregroup Number:Rescale of 46 : 0 : 6 : 0

	f1-score	precision	recall	support
AFIB	0.847916	0.850171	0.845724	875.0
GSVT	0.864221	0.858133	0.870447	691.0
SB	0.913583	0.904268	0.923110	1553.0
SR	0.871822	0.890812	0.853656	907.0
macro avg	0.874385	0.875846	0.873234	4026.0
micro avg	0.881605	0.881605	0.881605	4026.0
weighted avg	0.881431	0.881561	0.881605	4026.0

The result of Model Number:Dataset Number:Featuregroup Number:Rescale of 46 : 0 : 6 : 1

	f1-score	precision	recall	support
AFIB	0.881426	0.876988	0.885948	875.0
GSVT	0.898594	0.902945	0.894300	691.0
SB	0.957058	0.954855	0.959274	1553.0
SR	0.933099	0.938091	0.928169	907.0
macro avg	0.917544	0.918220	0.916923	4026.0
micro avg	0.925178	0.925178	0.925178	4026.0
weighted avg	0.925188	0.925245	0.925178	4026.0

The result of Model Number:Dataset Number:Featuregroup Number:Rescale of 46 : 0 : 7 : 0

	f1-score	precision	recall	support
AFIB	0.800547	0.816168	0.785651	204.0
GSVT	0.820500	0.809912	0.831422	300.0
SB	0.834465	0.826215	0.842884	892.0
SR	0.817023	0.828374	0.805983	681.0
macro avg	0.818134	0.820167	0.816485	2077.0
micro avg	0.823508	0.823508	0.823508	2077.0
weighted avg	0.823397	0.823581	0.823508	2077.0

The result of Model Number:Dataset Number:Featuregroup Number:Rescale of 46 : 0 : 7 : 1

	f1-score	precision	recall	support
AFIB	0.884687	0.900923	0.869452	204.0
GSVT	0.918271	0.910888	0.925943	300.0
SB	0.935305	0.933635	0.936983	892.0
SR	0.926044	0.926869	0.925226	681.0
macro avg	0.916077	0.918079	0.914401	2077.0
micro avg	0.924901	0.924901	0.924901	2077.0
weighted avg	0.924837	0.924918	0.924901	2077.0

The result of Model Number:Dataset Number:Featuregroup Number:Rescale of 46 : 0 : 8 : 0

	f1-score	precision	recall	support
AFIB	0.760629	0.760386	0.760912	875.0
GSVT	0.771965	0.762042	0.782240	691.0
SB	0.816538	0.814762	0.818325	1553.0
SR	0.793946	0.805584	0.782652	907.0
macro avg	0.785770	0.785693	0.786032	4026.0
micro avg	0.791617	0.791617	0.791617	4026.0
weighted avg	0.791647	0.791828	0.791617	4026.0

The result of Model Number:Dataset Number:Featuregroup Number:Rescale of 46 : 0 : 8 : 1

	f1-score	precision	recall	support
AFIB	0.701614	0.765158	0.648984	204.0
GSVT	0.814351	0.805046	0.824125	300.0
SB	0.863571	0.861361	0.865798	892.0
SR	0.842428	0.831020	0.854177	681.0
macro avg	0.805491	0.815646	0.798271	2077.0
micro avg	0.834673	0.834673	0.834673	2077.0
weighted avg	0.833622	0.833830	0.834673	2077.0

The result of Model Number:Dataset Number:Featuregroup Number:Rescale of 46 : 0 : 9 : 0

	f1-score	precision	recall	support
AFIB	0.874039	0.942162	0.815508	204.0
GSVT	0.900692	0.915939	0.885983	300.0
SB	0.954564	0.945779	0.963544	892.0
SR	0.916572	0.903280	0.930303	681.0
macro avg	0.911467	0.926790	0.898834	2077.0
micro avg	0.926902	0.926902	0.926902	2077.0
weighted avg	0.926417	0.927179	0.926902	2077.0

The result of Model Number:Dataset Number:Featuregroup Number:Rescale of 46 : 0 : 9 : 1

	f1-score	precision	recall	support
AFIB	0.823675	0.817333	0.830179	875.0
GSVT	0.834369	0.838243	0.830712	691.0
SB	0.894142	0.889386	0.898969	1553.0
SR	0.867798	0.879999	0.856026	907.0
macro avg	0.854996	0.856240	0.853972	4026.0
micro avg	0.862629	0.862629	0.862629	4026.0
weighted avg	0.862633	0.862833	0.862629	4026.0

The result of Model Number:Dataset Number:Featuregroup Number:Rescale of 46 : 0 : 10 : 0

	f1-score	precision	recall	support
AFIB	0.749857	0.751719	0.748054	875.0
GSVT	0.762770	0.765062	0.760589	691.0
SB	0.809162	0.803669	0.814735	1553.0

SR 0.793764 0.799657 0.787989 907.0
macro avg 0.778888 0.780027 0.777842 4026.0
micro avg 0.784924 0.784924 0.784924 4026.0
weighted avg 0.784841 0.784848 0.784924 4026.0
The result of Model Number:Dataset Number:Featuregroup Number:Rescale of 46 : 0 : 10 : 1

	f1-score	precision	recall	support
AFIB	0.765361	0.776195	0.755453	204.0
GSVT	0.781251	0.766867	0.796329	300.0
SB	0.797902	0.789080	0.806937	892.0
SR	0.781134	0.797043	0.765894	681.0
macro avg	0.781412	0.782296	0.781153	2077.0
micro avg	0.786891	0.786891	0.786891	2077.0
weighted avg	0.786803	0.787217	0.786891	2077.0

The result of Model Number:Dataset Number:Featuregroup Number:Rescale of 46 : 1 : 0 : 0

	f1-score	precision	recall	support
AFIB	0.760886	0.751911	0.770116	875.0
GSVT	0.773775	0.778671	0.768968	691.0
SB	0.824994	0.823884	0.826109	1553.0
SR	0.804726	0.812350	0.797271	907.0
macro avg	0.791095	0.791704	0.790616	4026.0
micro avg	0.797636	0.797636	0.797636	4026.0
weighted avg	0.797704	0.797883	0.797636	4026.0

The result of Model Number:Dataset Number:Featuregroup Number:Rescale of 46 : 1 : 0 : 1

	f1-score	precision	recall	support
AFIB	0.789535	0.811206	0.769051	204.0
GSVT	0.807392	0.794989	0.820266	300.0
SB	0.825132	0.817151	0.833272	892.0
SR	0.804277	0.814327	0.794478	681.0
macro avg	0.806584	0.809418	0.804267	2077.0
micro avg	0.812366	0.812366	0.812366	2077.0
weighted avg	0.812236	0.812440	0.812366	2077.0

The result of Model Number:Dataset Number:Featuregroup Number:Rescale of 46 : 1 : 1 : 0

	f1-score	precision	recall	support
AFIB	0.712496	0.746608	0.681532	204.0
GSVT	0.750450	0.742416	0.758687	300.0
SB	0.766390	0.762156	0.770681	892.0
SR	0.757733	0.757076	0.758404	681.0
macro avg	0.746767	0.752064	0.742326	2077.0
micro avg	0.756167	0.756167	0.756167	2077.0
weighted avg	0.755956	0.756112	0.756167	2077.0

The result of Model Number:Dataset Number:Featuregroup Number:Rescale of 46 : 1 : 1 : 1

	f1-score	precision	recall	support
AFIB	0.747517	0.815218	0.691444	204.0
GSVT	0.867630	0.857716	0.878043	300.0
SB	0.920070	0.917715	0.922442	892.0
SR	0.897543	0.885389	0.910061	681.0
macro avg	0.858190	0.869010	0.850498	2077.0
micro avg	0.889282	0.889282	0.889282	2077.0
weighted avg	0.888162	0.888383	0.889282	2077.0

The result of Model Number:Dataset Number:Featuregroup Number:Rescale of 46 : 1 : 2 : 0

	f1-score	precision	recall	support
AFIB	0.751434	0.743131	0.759939	875.0
GSVT	0.764973	0.769683	0.760333	691.0
SB	0.814167	0.812812	0.815530	1553.0
SR	0.794342	0.801770	0.787071	907.0
macro avg	0.781229	0.781849	0.780718	4026.0
micro avg	0.787563	0.787563	0.787563	4026.0
weighted avg	0.787623	0.787778	0.787563	4026.0

The result of Model Number:Dataset Number:Featuregroup Number:Rescale of 46 : 1 : 2 : 1

	f1-score	precision	recall	support
AFIB	0.669343	0.736874	0.613543	204.0
GSVT	0.789351	0.778456	0.800682	300.0
SB	0.838138	0.835342	0.840957	892.0
SR	0.816674	0.805376	0.828305	681.0
macro avg	0.778377	0.789012	0.770872	2077.0
micro avg	0.808655	0.808655	0.808655	2077.0
weighted avg	0.807475	0.807629	0.808655	2077.0

The result of Model Number:Dataset Number:Featuregroup Number:Rescale of 46 : 1 : 3 : 0

	f1-score	precision	recall	support
AFIB	0.821793	0.836869	0.807314	204.0
GSVT	0.832631	0.824228	0.841224	300.0
SB	0.853141	0.848700	0.857634	892.0
SR	0.841526	0.846799	0.836337	681.0
macro avg	0.837273	0.839149	0.835627	2077.0
micro avg	0.843339	0.843339	0.843339	2077.0
weighted avg	0.843291	0.843380	0.843339	2077.0

The result of Model Number:Dataset Number:Featuregroup Number:Rescale of 46 : 1 : 3 : 1

	f1-score	precision	recall	support
AFIB	0.783859	0.790072	0.777763	875.0

GSVT 0.793797 0.790297 0.797354 691.0
SB 0.844109 0.840772 0.847479 1553.0
SR 0.823367 0.825693 0.821093 907.0
macro avg 0.811283 0.811709 0.810922 4026.0
micro avg 0.817780 0.817780 0.817780 4026.0
weighted avg 0.817706 0.817693 0.817780 4026.0
The result of Model Number:Dataset Number:Featuregroup Number:Rescale of 46 : 1 : 4 : 0

	f1-score	precision	recall	support
AFIB	0.689375	0.687255	0.691552	875.0
GSVT	0.671802	0.675096	0.668646	691.0
SB	0.706252	0.676944	0.738220	1553.0
SR	0.607788	0.663131	0.561019	907.0
macro avg	0.668804	0.675607	0.664859	4026.0
micro avg	0.676215	0.676215	0.676215	4026.0
weighted avg	0.674489	0.675756	0.676215	4026.0

The result of Model Number:Dataset Number:Featuregroup Number:Rescale of 46 : 1 : 4 : 1

	f1-score	precision	recall	support
AFIB	0.852175	0.868833	0.836294	204.0
GSVT	0.875591	0.869283	0.882022	300.0
SB	0.891262	0.887794	0.894759	892.0
SR	0.881520	0.884014	0.879058	681.0
macro avg	0.875137	0.877481	0.873033	2077.0
micro avg	0.882029	0.882029	0.882029	2077.0
weighted avg	0.881965	0.882018	0.882029	2077.0

The result of Model Number:Dataset Number:Featuregroup Number:Rescale of 46 : 1 : 5 : 0

	f1-score	precision	recall	support
AFIB	0.678214	0.747096	0.621612	204.0
GSVT	0.626327	0.669637	0.588571	300.0
SB	0.681884	0.637134	0.733405	892.0
SR	0.590182	0.621205	0.562271	681.0
macro avg	0.644152	0.668768	0.626465	2077.0
micro avg	0.645394	0.645394	0.645394	2077.0
weighted avg	0.643432	0.647406	0.645394	2077.0

The result of Model Number:Dataset Number:Featuregroup Number:Rescale of 46 : 1 : 5 : 1

	f1-score	precision	recall	support
AFIB	0.614454	0.616963	0.611984	875.0
GSVT	0.572175	0.597427	0.549065	691.0
SB	0.614482	0.556641	0.685746	1553.0
SR	0.463525	0.576901	0.387549	907.0
macro avg	0.566159	0.586983	0.558586	4026.0

micro avg 0.579076 0.579076 0.579076 4026.0
weighted avg 0.573206 0.581316 0.579076 4026.0
The result of Model Number:Dataset Number:Featuregroup Number:Rescale of 46 : 1 : 6 : 0

	f1-score	precision	recall	support
AFIB	0.693948	0.700707	0.687486	204.0
GSVT	0.709995	0.706735	0.713310	300.0
SB	0.719807	0.717330	0.722306	892.0
SR	0.713728	0.716497	0.711014	681.0
macro avg	0.709369	0.710317	0.708529	2077.0
micro avg	0.713884	0.713884	0.713884	2077.0
weighted avg	0.713857	0.713894	0.713884	2077.0

The result of Model Number:Dataset Number:Featuregroup Number:Rescale of 46 : 1 : 6 : 1

	f1-score	precision	recall	support
AFIB	0.769123	0.783233	0.755572	204.0
GSVT	0.779267	0.771403	0.787309	300.0
SB	0.798462	0.794306	0.802667	892.0
SR	0.787592	0.792527	0.782735	681.0
macro avg	0.783611	0.785367	0.782071	2077.0
micro avg	0.789288	0.789288	0.789288	2077.0
weighted avg	0.789244	0.789327	0.789288	2077.0

The result of Model Number:Dataset Number:Featuregroup Number:Rescale of 46 : 1 : 7 : 0

	f1-score	precision	recall	support
AFIB	0.825132	0.832743	0.817859	204.0
GSVT	0.855299	0.855329	0.855291	300.0
SB	0.873211	0.871660	0.874775	892.0
SR	0.863944	0.863712	0.864196	681.0
macro avg	0.854396	0.855861	0.853030	2077.0
micro avg	0.862902	0.862902	0.862902	2077.0
weighted avg	0.862863	0.862873	0.862902	2077.0

The result of Model Number:Dataset Number:Featuregroup Number:Rescale of 46 : 1 : 7 : 1

	f1-score	precision	recall	support
AFIB	0.713960	0.789452	0.652016	204.0
GSVT	0.821504	0.811996	0.831393	300.0
SB	0.868558	0.864987	0.872161	892.0
SR	0.849905	0.837317	0.862888	681.0
macro avg	0.813482	0.825938	0.804615	2077.0
micro avg	0.841610	0.841610	0.841610	2077.0
weighted avg	0.840462	0.840842	0.841610	2077.0

The result of Model Number:Dataset Number:Featuregroup Number:Rescale of 46 : 1 : 8 : 0

	f1-score	precision	recall	support
AFIB	0.698759	0.702222	0.695405	875.0
GSVT	0.705717	0.706733	0.704810	691.0
SB	0.742862	0.739415	0.746349	1553.0
SR	0.729187	0.731013	0.727410	907.0
macro avg	0.719131	0.719846	0.718493	4026.0
micro avg	0.723881	0.723881	0.723881	4026.0
weighted avg	0.723821	0.723830	0.723881	4026.0

The result of Model Number:Dataset Number:Featuregroup Number:Rescale of 46 : 1 : 8 : 1

	f1-score	precision	recall	support
AFIB	0.840818	0.854797	0.827482	204.0
GSVT	0.856941	0.850853	0.863181	300.0
SB	0.876817	0.871890	0.881821	892.0
SR	0.865647	0.870943	0.860442	681.0
macro avg	0.860056	0.862121	0.858232	2077.0
micro avg	0.866782	0.866782	0.866782	2077.0
weighted avg	0.866748	0.866862	0.866782	2077.0

The result of Model Number:Dataset Number:Featuregroup Number:Rescale of 46 : 1 : 9 : 0

	f1-score	precision	recall	support
AFIB	0.608191	0.590668	0.667302	875.0
GSVT	0.670308	0.668167	0.672549	691.0
SB	0.565838	0.557499	0.574477	1553.0
SR	0.677524	0.690748	0.664882	907.0
macro avg	0.630465	0.626771	0.644802	4026.0
micro avg	0.631851	0.631851	0.631851	4026.0
weighted avg	0.618135	0.613722	0.631851	4026.0

The result of Model Number:Dataset Number:Featuregroup Number:Rescale of 46 : 1 : 9 : 1

	f1-score	precision	recall	support
AFIB	0.710137	0.699523	0.721104	875.0
GSVT	0.723752	0.729942	0.717678	691.0
SB	0.773313	0.770650	0.775999	1553.0
SR	0.750678	0.761875	0.739832	907.0
macro avg	0.739470	0.740498	0.738654	4026.0
micro avg	0.745911	0.745911	0.745911	4026.0
weighted avg	0.745977	0.746228	0.745911	4026.0

The result of Model Number:Dataset Number:Featuregroup Number:Rescale of 46 : 1 : 10 : 0

	f1-score	precision	recall	support
AFIB	0.766976	0.756095	0.778194	875.0
GSVT	0.782756	0.790385	0.775278	691.0
SB	0.833005	0.830290	0.835739	1553.0

SR 0.813552 0.823950 0.803422 907.0
macro avg 0.799072 0.800180 0.798158 4026.0
micro avg 0.805575 0.805575 0.805575 4026.0
weighted avg 0.805647 0.805887 0.805575 4026.0
The result of Model Number:Dataset Number:Featuregroup Number:Rescale of 46 : 1 : 10 : 1

	f1-score	precision	recall	support
AFIB	0.870828	0.857476	0.884748	875.0
GSVT	0.900856	0.909686	0.892373	691.0
SB	0.958751	0.956511	0.961031	1553.0
SR	0.934801	0.946653	0.923261	907.0
macro avg	0.916309	0.917581	0.915353	4026.0
micro avg	0.924159	0.924159	0.924159	4026.0
weighted avg	0.924309	0.924729	0.924159	4026.0

The result of Model Number:Dataset Number:Featuregroup Number:Rescale of 47 : 0 : 0 : 0

	f1-score	precision	recall	support
AFIB	0.839721	0.847466	0.832319	204.0
GSVT	0.870421	0.870452	0.870413	300.0
SB	0.888650	0.887071	0.890242	892.0
SR	0.879219	0.878983	0.879476	681.0
macro avg	0.869503	0.870993	0.868112	2077.0
micro avg	0.878159	0.878159	0.878159	2077.0
weighted avg	0.878119	0.878129	0.878159	2077.0

The result of Model Number:Dataset Number:Featuregroup Number:Rescale of 47 : 0 : 0 : 1

	f1-score	precision	recall	support
AFIB	0.686970	0.700993	0.673800	204.0
GSVT	0.711915	0.705729	0.718339	300.0
SB	0.725602	0.724307	0.726904	892.0
SR	0.718848	0.719277	0.718425	681.0
macro avg	0.710834	0.712577	0.709367	2077.0
micro avg	0.717671	0.717671	0.717671	2077.0
weighted avg	0.717616	0.717684	0.717671	2077.0

The result of Model Number:Dataset Number:Featuregroup Number:Rescale of 47 : 0 : 1 : 0

	f1-score	precision	recall	support
AFIB	0.786840	0.799617	0.774507	875.0
GSVT	0.836828	0.839450	0.834272	691.0
SB	0.910426	0.907289	0.913590	1553.0
SR	0.861908	0.852308	0.871779	907.0
macro avg	0.849001	0.849666	0.848537	4026.0
micro avg	0.860329	0.860329	0.860329	4026.0
weighted avg	0.860004	0.859858	0.860329	4026.0

The result of Model Number:Dataset Number:Featuregroup Number:Rescale of 47 : 0 : 1 : 1

	f1-score	precision	recall	support
AFIB	0.597718	0.659156	0.547123	204.0
GSVT	0.711267	0.701654	0.721216	300.0
SB	0.757448	0.754419	0.760505	892.0
SR	0.736890	0.726929	0.747160	681.0
macro avg	0.700831	0.710540	0.694001	2077.0
micro avg	0.729496	0.729496	0.729496	2077.0
weighted avg	0.728349	0.728428	0.729496	2077.0

The result of Model Number:Dataset Number:Featuregroup Number:Rescale of 47 : 0 : 2 : 0

	f1-score	precision	recall	support
AFIB	0.779016	0.781930	0.776455	875.0
GSVT	0.796552	0.804236	0.789172	691.0
SB	0.846984	0.838100	0.856123	1553.0
SR	0.817654	0.824850	0.810863	907.0
macro avg	0.810051	0.812279	0.808153	4026.0
micro avg	0.817121	0.817121	0.817121	4026.0
weighted avg	0.816948	0.817095	0.817121	4026.0

The result of Model Number:Dataset Number:Featuregroup Number:Rescale of 47 : 0 : 2 : 1

	f1-score	precision	recall	support
AFIB	0.718700	0.720349	0.717136	875.0
GSVT	0.721205	0.719172	0.723274	691.0
SB	0.756894	0.750626	0.763277	1553.0
SR	0.734187	0.745205	0.723521	907.0
macro avg	0.732746	0.733838	0.731802	4026.0
micro avg	0.737427	0.737427	0.737427	4026.0
weighted avg	0.737352	0.737426	0.737427	4026.0

The result of Model Number:Dataset Number:Featuregroup Number:Rescale of 47 : 0 : 3 : 0

	f1-score	precision	recall	support
AFIB	0.667105	0.656425	0.750628	204.0
GSVT	0.781004	0.771941	0.790566	300.0
SB	0.807155	0.804107	0.810231	892.0
SR	0.633308	0.636583	0.630175	681.0
macro avg	0.722143	0.717264	0.745400	2077.0
micro avg	0.742500	0.742500	0.742500	2077.0
weighted avg	0.732622	0.730028	0.742500	2077.0

The result of Model Number:Dataset Number:Featuregroup Number:Rescale of 47 : 0 : 3 : 1

	f1-score	precision	recall	support
AFIB	0.768669	0.766446	0.770974	875.0

GSVT 0.782915 0.782278 0.783607 691.0
SB 0.831569 0.828125 0.835049 1553.0
SR 0.806605 0.815466 0.797978 907.0
macro avg 0.797439 0.798079 0.796902 4026.0
micro avg 0.803942 0.803942 0.803942 4026.0
weighted avg 0.803924 0.803999 0.803942 4026.0
The result of Model Number:Dataset Number:Featuregroup Number:Rescale of 47 : 0 : 4 : 0

	f1-score	precision	recall	support
AFIB	0.612276	0.675211	0.560450	204.0
GSVT	0.728591	0.718745	0.738783	300.0
SB	0.775897	0.772794	0.779028	892.0
SR	0.754838	0.744635	0.765358	681.0
macro avg	0.717901	0.727846	0.710905	2077.0
micro avg	0.747265	0.747265	0.747265	2077.0
weighted avg	0.746089	0.746170	0.747265	2077.0

The result of Model Number:Dataset Number:Featuregroup Number:Rescale of 47 : 0 : 4 : 1

	f1-score	precision	recall	support
AFIB	0.683554	0.685907	0.681305	875.0
GSVT	0.696532	0.682635	0.711132	691.0
SB	0.737335	0.733347	0.741370	1553.0
SR	0.708816	0.725297	0.693110	907.0
macro avg	0.706559	0.706796	0.706729	4026.0
micro avg	0.712253	0.712253	0.712253	4026.0
weighted avg	0.712218	0.712519	0.712253	4026.0

The result of Model Number:Dataset Number:Featuregroup Number:Rescale of 47 : 0 : 5 : 0

	f1-score	precision	recall	support
AFIB	0.719490	0.727904	0.711404	204.0
GSVT	0.648984	0.624207	0.740481	300.0
SB	0.602124	0.600706	0.603556	892.0
SR	0.747635	0.749073	0.746209	681.0
macro avg	0.679559	0.675472	0.700413	2077.0
micro avg	0.680699	0.680699	0.680699	2077.0
weighted avg	0.668130	0.665239	0.680699	2077.0

The result of Model Number:Dataset Number:Featuregroup Number:Rescale of 47 : 0 : 5 : 1

	f1-score	precision	recall	support
AFIB	0.739117	0.741660	0.736684	875.0
GSVT	0.753149	0.738123	0.768935	691.0
SB	0.797268	0.792957	0.801631	1553.0
SR	0.766432	0.784252	0.749449	907.0
macro avg	0.763991	0.764248	0.764175	4026.0

micro avg 0.770148 0.770148 0.770148 4026.0
weighted avg 0.770110 0.770436 0.770148 4026.0
The result of Model Number:Dataset Number:Featuregroup Number:Rescale of 47 : 0 : 6 : 0

	f1-score	precision	recall	support
AFIB	0.679087	0.716308	0.645994	204.0
GSVT	0.646635	0.669306	0.625529	300.0
SB	0.665128	0.639474	0.692936	892.0
SR	0.591982	0.608042	0.576781	681.0
macro avg	0.645708	0.658283	0.635310	2077.0
micro avg	0.640505	0.640505	0.640505	2077.0
weighted avg	0.639845	0.641024	0.640505	2077.0

The result of Model Number:Dataset Number:Featuregroup Number:Rescale of 47 : 0 : 6 : 1

	f1-score	precision	recall	support
AFIB	0.652727	0.654543	0.650955	875.0
GSVT	0.667679	0.667494	0.667871	691.0
SB	0.711099	0.704415	0.717919	1553.0
SR	0.692257	0.702240	0.682562	907.0
macro avg	0.680940	0.682173	0.679827	4026.0
micro avg	0.686810	0.686810	0.686810	4026.0
weighted avg	0.686715	0.686749	0.686810	4026.0

The result of Model Number:Dataset Number:Featuregroup Number:Rescale of 47 : 0 : 7 : 0

	f1-score	precision	recall	support
AFIB	0.764524	0.781812	0.748006	875.0
GSVT	0.810569	0.814018	0.807196	691.0
SB	0.880110	0.875962	0.884302	1553.0
SR	0.833892	0.821101	0.847122	907.0
macro avg	0.822273	0.823223	0.821657	4026.0
micro avg	0.833070	0.833070	0.833070	4026.0
weighted avg	0.832641	0.832509	0.833070	4026.0

The result of Model Number:Dataset Number:Featuregroup Number:Rescale of 47 : 0 : 7 : 1

	f1-score	precision	recall	support
AFIB	0.742156	0.743325	0.741087	204.0
GSVT	0.760105	0.758919	0.761358	300.0
SB	0.775785	0.774839	0.776740	892.0
SR	0.770352	0.771830	0.768883	681.0
macro avg	0.762099	0.762228	0.762017	2077.0
micro avg	0.768440	0.768440	0.768440	2077.0
weighted avg	0.768436	0.768458	0.768440	2077.0

The result of Model Number:Dataset Number:Featuregroup Number:Rescale of 47 : 0 : 8 : 0

f1-score precision recall support
AFIB 0.681112 0.671449 0.691074 875.0
GSVT 0.695125 0.701900 0.688485 691.0
SB 0.739749 0.737338 0.742177 1553.0
SR 0.722473 0.731707 0.713477 907.0
macro avg 0.709615 0.710599 0.708803 4026.0
micro avg 0.715389 0.715389 0.715389 4026.0
weighted avg 0.715454 0.715667 0.715389 4026.0
The result of Model Number:Dataset Number:Featuregroup Number:Rescale of 47 : 0 : 8 : 1
f1-score precision recall support
AFIB 0.890986 0.890932 0.891095 875.0
GSVT 0.892914 0.888087 0.897810 691.0
SB 0.935572 0.929347 0.941893 1553.0
SR 0.910729 0.925672 0.896295 907.0
macro avg 0.907550 0.908509 0.906773 4026.0
micro avg 0.913014 0.913014 0.913014 4026.0
weighted avg 0.912963 0.913088 0.913014 4026.0
The result of Model Number:Dataset Number:Featuregroup Number:Rescale of 47 : 0 : 9 : 0
f1-score precision recall support
AFIB 0.704411 0.706161 0.702718 875.0
GSVT 0.716542 0.718695 0.714493 691.0
SB 0.760122 0.754963 0.765358 1553.0
SR 0.745658 0.751193 0.740232 907.0
macro avg 0.731684 0.732753 0.730700 4026.0
micro avg 0.737353 0.737353 0.737353 4026.0
weighted avg 0.737276 0.737282 0.737353 4026.0
The result of Model Number:Dataset Number:Featuregroup Number:Rescale of 47 : 0 : 9 : 1
f1-score precision recall support
AFIB 0.863565 0.876806 0.850860 204.0
GSVT 0.884041 0.876540 0.891708 300.0
SB 0.899419 0.896517 0.902343 892.0
SR 0.890177 0.893481 0.886908 681.0
macro avg 0.884300 0.885836 0.882955 2077.0
micro avg 0.890690 0.890690 0.890690 2077.0
weighted avg 0.890646 0.890700 0.890690 2077.0
The result of Model Number:Dataset Number:Featuregroup Number:Rescale of 47 : 0 : 10 : 0
f1-score precision recall support
AFIB 0.881034 0.897204 0.865862 204.0
GSVT 0.914480 0.907127 0.922120 300.0
SB 0.931444 0.929781 0.933115 892.0

SR 0.922221 0.923042 0.921407 681.0
macro avg 0.912295 0.914289 0.910626 2077.0
micro avg 0.921082 0.921082 0.921082 2077.0
weighted avg 0.921019 0.921100 0.921082 2077.0
The result of Model Number:Dataset Number:Featuregroup Number:Rescale of 47 : 0 : 10 : 1
f1-score precision recall support
AFIB 0.827600 0.802864 0.882437 875.0
GSVT 0.708717 0.693197 0.725210 691.0
SB 0.940028 0.932369 0.948064 1553.0
SR 0.881882 0.918907 0.849380 907.0
macro avg 0.839557 0.836834 0.851272 4026.0
micro avg 0.873319 0.873319 0.873319 4026.0
weighted avg 0.862793 0.860140 0.873319 4026.0
The result of Model Number:Dataset Number:Featuregroup Number:Rescale of 47 : 1 : 0 : 0
f1-score precision recall support
AFIB 0.760497 0.777280 0.744479 204.0
GSVT 0.777117 0.764230 0.790539 300.0
SB 0.792050 0.785855 0.798352 892.0
SR 0.772828 0.782130 0.763746 681.0
macro avg 0.775623 0.777374 0.774279 2077.0
micro avg 0.780586 0.780586 0.780586 2077.0
weighted avg 0.780491 0.780668 0.780586 2077.0
The result of Model Number:Dataset Number:Featuregroup Number:Rescale of 47 : 1 : 0 : 1
f1-score precision recall support
AFIB 0.745366 0.787768 0.707790 204.0
GSVT 0.787684 0.779010 0.796593 300.0
SB 0.799401 0.791447 0.807544 892.0
SR 0.790114 0.792815 0.787556 681.0
macro avg 0.780641 0.787760 0.774871 2077.0
micro avg 0.789611 0.789611 0.789611 2077.0
weighted avg 0.789356 0.789738 0.789611 2077.0
The result of Model Number:Dataset Number:Featuregroup Number:Rescale of 47 : 1 : 1 : 0
f1-score precision recall support
AFIB 0.879001 0.889054 0.869512 204.0
GSVT 0.899575 0.894252 0.904977 300.0
SB 0.863576 0.831677 0.917421 892.0
SR 0.724404 0.727910 0.720950 681.0
macro avg 0.841639 0.835723 0.853215 2077.0
micro avg 0.846500 0.846500 0.846500 2077.0
weighted avg 0.824659 0.812328 0.846500 2077.0

The result of Model Number:Dataset Number:Featuregroup Number:Rescale of 47 : 1 : 1 : 1

	f1-score	precision	recall	support
AFIB	0.699723	0.689788	0.710027	875.0
GSVT	0.725099	0.734349	0.716188	691.0
SB	0.770580	0.769449	0.771722	1553.0
SR	0.751383	0.757019	0.745832	907.0
macro avg	0.736696	0.737651	0.735942	4026.0
micro avg	0.742949	0.742949	0.742949	4026.0
weighted avg	0.743049	0.743311	0.742949	4026.0

The result of Model Number:Dataset Number:Featuregroup Number:Rescale of 47 : 1 : 2 : 0

	f1-score	precision	recall	support
AFIB	0.768547	0.767311	0.770102	875.0
GSVT	0.786467	0.782367	0.790655	691.0
SB	0.822292	0.815325	0.829385	1553.0
SR	0.790902	0.808485	0.774334	907.0
macro avg	0.792052	0.793372	0.791119	4026.0
micro avg	0.797451	0.797451	0.797451	4026.0
weighted avg	0.797390	0.797692	0.797451	4026.0

The result of Model Number:Dataset Number:Featuregroup Number:Rescale of 47 : 1 : 2 : 1

	f1-score	precision	recall	support
AFIB	0.779697	0.803343	0.757437	875.0
GSVT	0.832338	0.826566	0.838236	691.0
SB	0.910679	0.907196	0.914196	1553.0
SR	0.861283	0.848356	0.874660	907.0
macro avg	0.845999	0.846365	0.846132	4026.0
micro avg	0.858182	0.858182	0.858182	4026.0
weighted avg	0.857638	0.857530	0.858182	4026.0

The result of Model Number:Dataset Number:Featuregroup Number:Rescale of 47 : 1 : 3 : 0

	f1-score	precision	recall	support
AFIB	0.797450	0.790059	0.805026	875.0
GSVT	0.811772	0.816180	0.807424	691.0
SB	0.860118	0.858135	0.862111	1553.0
SR	0.840078	0.847831	0.832493	907.0
macro avg	0.827355	0.828051	0.826763	4026.0
micro avg	0.833646	0.833646	0.833646	4026.0
weighted avg	0.833685	0.833817	0.833646	4026.0

The result of Model Number:Dataset Number:Featuregroup Number:Rescale of 47 : 1 : 3 : 1

	f1-score	precision	recall	support
AFIB	0.879835	0.883839	0.875921	875.0

GSVT 0.884110 0.875298 0.893177 691.0
SB 0.931854 0.921699 0.942234 1553.0
SR 0.891217 0.912316 0.871076 907.0
macro avg 0.896754 0.898288 0.895602 4026.0
micro avg 0.903371 0.903371 0.903371 4026.0
weighted avg 0.903199 0.903393 0.903371 4026.0
The result of Model Number:Dataset Number:Featuregroup Number:Rescale of 47 : 1 : 4 : 0

	f1-score	precision	recall	support
AFIB	0.828094	0.875202	0.786348	204.0
GSVT	0.875109	0.865472	0.885007	300.0
SB	0.888126	0.879289	0.897173	892.0
SR	0.877809	0.880810	0.874967	681.0
macro avg	0.867285	0.875193	0.860874	2077.0
micro avg	0.877250	0.877250	0.877250	2077.0
weighted avg	0.876967	0.877391	0.877250	2077.0

The result of Model Number:Dataset Number:Featuregroup Number:Rescale of 47 : 1 : 4 : 1

	f1-score	precision	recall	support
AFIB	0.784321	0.801630	0.767801	204.0
GSVT	0.801461	0.788171	0.815304	300.0
SB	0.816862	0.810473	0.823362	892.0
SR	0.797038	0.806631	0.787672	681.0
macro avg	0.799920	0.801726	0.798535	2077.0
micro avg	0.805039	0.805039	0.805039	2077.0
weighted avg	0.804941	0.805123	0.805039	2077.0

The result of Model Number:Dataset Number:Featuregroup Number:Rescale of 47 : 1 : 5 : 0

	f1-score	precision	recall	support
AFIB	0.845291	0.857967	0.833224	204.0
GSVT	0.875815	0.873490	0.878158	300.0
SB	0.896562	0.895367	0.897767	892.0
SR	0.887793	0.886648	0.888969	681.0
macro avg	0.876365	0.878368	0.874530	2077.0
micro avg	0.885711	0.885711	0.885711	2077.0
weighted avg	0.885654	0.885675	0.885711	2077.0

The result of Model Number:Dataset Number:Featuregroup Number:Rescale of 47 : 1 : 5 : 1

	f1-score	precision	recall	support
AFIB	0.833667	0.853623	0.815843	204.0
GSVT	0.858217	0.846124	0.871018	300.0
SB	0.880516	0.871570	0.889687	892.0
SR	0.859470	0.871880	0.847477	681.0
macro avg	0.857968	0.860800	0.856007	2077.0

micro avg 0.865898 0.865898 0.865898 2077.0
weighted avg 0.865793 0.866234 0.865898 2077.0
The result of Model Number:Dataset Number:Featuregroup Number:Rescale of 47 : 1 : 6 : 0

	f1-score	precision	recall	support
AFIB	0.768642	0.757996	0.779623	875.0
GSVT	0.785690	0.792049	0.779437	691.0
SB	0.834001	0.831604	0.836417	1553.0
SR	0.813712	0.824303	0.803398	907.0
macro avg	0.800512	0.801488	0.799719	4026.0
micro avg	0.806855	0.806855	0.806855	4026.0
weighted avg	0.806934	0.807173	0.806855	4026.0

The result of Model Number:Dataset Number:Featuregroup Number:Rescale of 47 : 1 : 6 : 1

	f1-score	precision	recall	support
AFIB	0.771313	0.770607	0.772113	875.0
GSVT	0.787123	0.785596	0.788738	691.0
SB	0.835240	0.833380	0.837121	1553.0
SR	0.812443	0.817743	0.807240	907.0
macro avg	0.801530	0.801831	0.801303	4026.0
micro avg	0.807957	0.807957	0.807957	4026.0
weighted avg	0.807952	0.808013	0.807957	4026.0

The result of Model Number:Dataset Number:Featuregroup Number:Rescale of 47 : 1 : 7 : 0

	f1-score	precision	recall	support
AFIB	0.813363	0.812047	0.814755	875.0
GSVT	0.820815	0.824079	0.817618	691.0
SB	0.861999	0.858866	0.865162	1553.0
SR	0.847899	0.852189	0.843700	907.0
macro avg	0.836019	0.836795	0.835309	4026.0
micro avg	0.841211	0.841211	0.841211	4026.0
weighted avg	0.841184	0.841215	0.841211	4026.0

The result of Model Number:Dataset Number:Featuregroup Number:Rescale of 47 : 1 : 7 : 1

	f1-score	precision	recall	support
AFIB	0.840064	0.855482	0.825597	204.0
GSVT	0.871954	0.864943	0.879239	300.0
SB	0.888129	0.886544	0.889722	892.0
SR	0.879336	0.880118	0.878559	681.0
macro avg	0.869871	0.871772	0.868280	2077.0
micro avg	0.878250	0.878250	0.878250	2077.0
weighted avg	0.878189	0.878266	0.878250	2077.0

The result of Model Number:Dataset Number:Featuregroup Number:Rescale of 47 : 1 : 8 : 0

	f1-score	precision	recall	support
AFIB	0.832452	0.836698	0.828275	875.0
GSVT	0.843940	0.839549	0.848450	691.0
SB	0.884581	0.883790	0.885379	1553.0
SR	0.868292	0.869000	0.867621	907.0
macro avg	0.857316	0.857259	0.857431	4026.0
micro avg	0.862629	0.862629	0.862629	4026.0
weighted avg	0.862606	0.862630	0.862629	4026.0

The result of Model Number:Dataset Number:Featuregroup Number:Rescale of 47 : 1 : 8 : 1

	f1-score	precision	recall	support
AFIB	0.627797	0.609710	0.688814	875.0
GSVT	0.691917	0.689707	0.694230	691.0
SB	0.584080	0.575472	0.592996	1553.0
SR	0.699365	0.713016	0.686316	907.0
macro avg	0.650790	0.646976	0.665589	4026.0
micro avg	0.652220	0.652220	0.652220	4026.0
weighted avg	0.638062	0.633506	0.652220	4026.0

The result of Model Number:Dataset Number:Featuregroup Number:Rescale of 47 : 1 : 9 : 0

	f1-score	precision	recall	support
AFIB	0.802127	0.817670	0.787524	204.0
GSVT	0.833232	0.826265	0.840471	300.0
SB	0.848779	0.847076	0.850491	892.0
SR	0.840686	0.841557	0.839820	681.0
macro avg	0.831206	0.833142	0.829577	2077.0
micro avg	0.839361	0.839361	0.839361	2077.0
weighted avg	0.839298	0.839372	0.839361	2077.0

The result of Model Number:Dataset Number:Featuregroup Number:Rescale of 47 : 1 : 9 : 1

	f1-score	precision	recall	support
AFIB	0.705839	0.701616	0.710208	875.0
GSVT	0.711619	0.715461	0.707921	691.0
SB	0.754210	0.752005	0.756439	1553.0
SR	0.737167	0.742462	0.731961	907.0
macro avg	0.727209	0.727886	0.726632	4026.0
micro avg	0.732549	0.732549	0.732549	4026.0
weighted avg	0.732547	0.732631	0.732549	4026.0

The result of Model Number:Dataset Number:Featuregroup Number:Rescale of 47 : 1 : 10 : 0

	f1-score	precision	recall	support
AFIB	0.668816	0.742249	0.608766	204.0
GSVT	0.780933	0.771170	0.791100	300.0
SB	0.830875	0.826635	0.835159	892.0

SR 0.811410 0.800066 0.823084 681.0
macro avg 0.773009 0.785030 0.764527 2077.0
micro avg 0.802600 0.802600 0.802600 2077.0
weighted avg 0.801362 0.801624 0.802600 2077.0
The result of Model Number:Dataset Number:Featuregroup Number:Rescale of 47 : 1 : 10 : 1

	f1-score	precision	recall	support
AFIB	0.870691	0.864207	0.877361	875.0
GSVT	0.886930	0.890097	0.883839	691.0
SB	0.941142	0.938912	0.943383	1553.0
SR	0.919664	0.927944	0.911564	907.0
macro avg	0.904607	0.905290	0.904037	4026.0
micro avg	0.911646	0.911646	0.911646	4026.0
weighted avg	0.911687	0.911826	0.911646	4026.0

The result of Model Number:Dataset Number:Featuregroup Number:Rescale of 48 : 0 : 0 : 0

	f1-score	precision	recall	support
AFIB	0.665335	0.681004	0.650428	875.0
GSVT	0.701362	0.703133	0.699646	691.0
SB	0.762744	0.759582	0.765935	1553.0
SR	0.725756	0.714326	0.737592	907.0
macro avg	0.713799	0.714511	0.713400	4026.0
micro avg	0.723068	0.723068	0.723068	4026.0
weighted avg	0.722705	0.722620	0.723068	4026.0

The result of Model Number:Dataset Number:Featuregroup Number:Rescale of 48 : 0 : 0 : 1

	f1-score	precision	recall	support
AFIB	0.707479	0.712568	0.702464	875.0
GSVT	0.706352	0.702123	0.710644	691.0
SB	0.745098	0.736843	0.753542	1553.0
SR	0.714979	0.727735	0.702686	907.0
macro avg	0.718477	0.719817	0.717334	4026.0
micro avg	0.723621	0.723621	0.723621	4026.0
weighted avg	0.723486	0.723556	0.723621	4026.0

The result of Model Number:Dataset Number:Featuregroup Number:Rescale of 48 : 0 : 1 : 0

	f1-score	precision	recall	support
AFIB	0.674127	0.757996	0.607563	204.0
GSVT	0.799803	0.782724	0.817679	300.0
SB	0.853804	0.849452	0.858206	892.0
SR	0.834650	0.824080	0.845548	681.0
macro avg	0.790596	0.803563	0.782249	2077.0
micro avg	0.823584	0.823584	0.823584	2077.0
weighted avg	0.822076	0.822512	0.823584	2077.0

The result of Model Number:Dataset Number:Featuregroup Number:Rescale of 48 : 0 : 1 : 1

	f1-score	precision	recall	support
AFIB	0.822710	0.826787	0.818761	875.0
GSVT	0.830901	0.832098	0.829833	691.0
SB	0.874636	0.870578	0.878741	1553.0
SR	0.858535	0.860685	0.856443	907.0
macro avg	0.846695	0.847537	0.845945	4026.0
micro avg	0.852287	0.852287	0.852287	4026.0
weighted avg	0.852217	0.852227	0.852287	4026.0

The result of Model Number:Dataset Number:Featuregroup Number:Rescale of 48 : 0 : 2 : 0

	f1-score	precision	recall	support
AFIB	0.747202	0.747558	0.746954	875.0
GSVT	0.766553	0.759918	0.773332	691.0
SB	0.818682	0.812716	0.824743	1553.0
SR	0.783706	0.799286	0.768763	907.0
macro avg	0.779036	0.779869	0.778448	4026.0
micro avg	0.786401	0.786401	0.786401	4026.0
weighted avg	0.786320	0.786467	0.786401	4026.0

The result of Model Number:Dataset Number:Featuregroup Number:Rescale of 48 : 0 : 2 : 1

	f1-score	precision	recall	support
AFIB	0.780446	0.792813	0.768563	204.0
GSVT	0.800507	0.793694	0.807441	300.0
SB	0.814327	0.812513	0.816153	892.0
SR	0.806612	0.808405	0.804842	681.0
macro avg	0.800473	0.801856	0.799250	2077.0
micro avg	0.806512	0.806512	0.806512	2077.0
weighted avg	0.806474	0.806513	0.806512	2077.0

The result of Model Number:Dataset Number:Featuregroup Number:Rescale of 48 : 0 : 3 : 0

	f1-score	precision	recall	support
AFIB	0.781640	0.763256	0.822896	875.0
GSVT	0.751086	0.744161	0.768201	691.0
SB	0.814603	0.757246	0.882300	1553.0
SR	0.507915	0.637007	0.422982	907.0
macro avg	0.713811	0.725418	0.724095	4026.0
micro avg	0.746328	0.746328	0.746328	4026.0
weighted avg	0.727445	0.729218	0.746328	4026.0

The result of Model Number:Dataset Number:Featuregroup Number:Rescale of 48 : 0 : 3 : 1

	f1-score	precision	recall	support
AFIB	0.724730	0.726955	0.722634	204.0

GSVT 0.743768 0.741878 0.745735 300.0
SB 0.760384 0.759455 0.761319 892.0
SR 0.754160 0.755610 0.752722 681.0
macro avg 0.745761 0.745974 0.745603 2077.0
micro avg 0.752450 0.752450 0.752450 2077.0
weighted avg 0.752441 0.752463 0.752450 2077.0
The result of Model Number:Dataset Number:Featuregroup Number:Rescale of 48 : 0 : 4 : 0
f1-score precision recall support
AFIB 0.632111 0.627732 0.636679 875.0
GSVT 0.641863 0.644468 0.639383 691.0
SB 0.696422 0.689181 0.703821 1553.0
SR 0.661259 0.676646 0.646617 907.0
macro avg 0.657914 0.659507 0.656625 4026.0
micro avg 0.665282 0.665282 0.665282 4026.0
weighted avg 0.665159 0.665327 0.665282 4026.0
The result of Model Number:Dataset Number:Featuregroup Number:Rescale of 48 : 0 : 4 : 1
f1-score precision recall support
AFIB 0.878546 0.887896 0.869876 204.0
GSVT 0.904364 0.898338 0.910736 300.0
SB 0.923475 0.922650 0.924304 892.0
SR 0.915689 0.916911 0.914476 681.0
macro avg 0.905519 0.906449 0.904848 2077.0
micro avg 0.913776 0.913776 0.913776 2077.0
weighted avg 0.913749 0.913843 0.913776 2077.0
The result of Model Number:Dataset Number:Featuregroup Number:Rescale of 48 : 0 : 5 : 0
f1-score precision recall support
AFIB 0.888014 0.899773 0.876691 204.0
GSVT 0.914812 0.911510 0.918158 300.0
SB 0.926206 0.923324 0.929111 892.0
SR 0.916114 0.917878 0.914382 681.0
macro avg 0.911286 0.913121 0.909585 2077.0
micro avg 0.917551 0.917551 0.917551 2077.0
weighted avg 0.917500 0.917519 0.917551 2077.0
The result of Model Number:Dataset Number:Featuregroup Number:Rescale of 48 : 0 : 5 : 1
f1-score precision recall support
AFIB 0.608463 0.621249 0.596215 875.0
GSVT 0.647941 0.650167 0.645763 691.0
SB 0.705941 0.702614 0.709303 1553.0
SR 0.668253 0.659353 0.677451 907.0
macro avg 0.657650 0.658346 0.657183 4026.0

micro avg 0.666643 0.666643 0.666643 4026.0
weighted avg 0.666310 0.666183 0.666643 4026.0
The result of Model Number:Dataset Number:Featuregroup Number:Rescale of 48 : 0 : 6 : 0

	f1-score	precision	recall	support
AFIB	0.872092	0.907292	0.839591	204.0
GSVT	0.904545	0.889851	0.919888	300.0
SB	0.927946	0.920486	0.935540	892.0
SR	0.902981	0.909483	0.896619	681.0
macro avg	0.901891	0.906778	0.897910	2077.0
micro avg	0.911094	0.911094	0.911094	2077.0
weighted avg	0.910895	0.911158	0.911094	2077.0

The result of Model Number:Dataset Number:Featuregroup Number:Rescale of 48 : 0 : 6 : 1

	f1-score	precision	recall	support
AFIB	0.621320	0.602749	0.662489	875.0
GSVT	0.532069	0.520417	0.544451	691.0
SB	0.705726	0.699976	0.711758	1553.0
SR	0.662072	0.689869	0.637671	907.0
macro avg	0.630297	0.628253	0.639092	4026.0
micro avg	0.655644	0.655644	0.655644	4026.0
weighted avg	0.647741	0.645749	0.655644	4026.0

The result of Model Number:Dataset Number:Featuregroup Number:Rescale of 48 : 0 : 7 : 0

	f1-score	precision	recall	support
AFIB	0.781778	0.775759	0.787951	875.0
GSVT	0.791928	0.795605	0.788458	691.0
SB	0.848661	0.844147	0.853243	1553.0
SR	0.823657	0.835237	0.812484	907.0
macro avg	0.811506	0.812687	0.810534	4026.0
micro avg	0.818751	0.818751	0.818751	4026.0
weighted avg	0.818755	0.818945	0.818751	4026.0

The result of Model Number:Dataset Number:Featuregroup Number:Rescale of 48 : 0 : 7 : 1

	f1-score	precision	recall	support
AFIB	0.685024	0.686930	0.683165	875.0
GSVT	0.700716	0.700522	0.700918	691.0
SB	0.746285	0.739270	0.753442	1553.0
SR	0.726510	0.736987	0.716336	907.0
macro avg	0.714634	0.715927	0.713465	4026.0
micro avg	0.720794	0.720794	0.720794	4026.0
weighted avg	0.720695	0.720730	0.720794	4026.0

The result of Model Number:Dataset Number:Featuregroup Number:Rescale of 48 : 0 : 8 : 0

f1-score precision recall support
AFIB 0.718991 0.700592 0.738449 875.0
GSVT 0.679669 0.718191 0.645227 691.0
SB 0.720687 0.663205 0.789096 1553.0
SR 0.575707 0.699272 0.489358 907.0
macro avg 0.673764 0.695315 0.665533 4026.0
micro avg 0.685869 0.685869 0.685869 4026.0
weighted avg 0.680616 0.688894 0.685869 4026.0
The result of Model Number:Dataset Number:Featuregroup Number:Rescale of 48 : 0 : 8 : 1
f1-score precision recall support
AFIB 0.854285 0.846730 0.862012 875.0
GSVT 0.776693 0.752168 0.864087 691.0
SB 0.700231 0.672318 0.743881 1553.0
SR 0.721690 0.729157 0.714420 907.0
macro avg 0.763225 0.750093 0.796100 4026.0
micro avg 0.783550 0.783550 0.783550 4026.0
weighted avg 0.751671 0.736735 0.783550 4026.0
The result of Model Number:Dataset Number:Featuregroup Number:Rescale of 48 : 0 : 9 : 0
f1-score precision recall support
AFIB 0.845650 0.862037 0.830255 204.0
GSVT 0.878443 0.871098 0.886074 300.0
SB 0.894834 0.893038 0.896639 892.0
SR 0.886301 0.887219 0.885389 681.0
macro avg 0.876307 0.878348 0.874589 2077.0
micro avg 0.884904 0.884904 0.884904 2077.0
weighted avg 0.884838 0.884916 0.884904 2077.0
The result of Model Number:Dataset Number:Featuregroup Number:Rescale of 48 : 0 : 9 : 1
f1-score precision recall support
AFIB 0.688580 0.705103 0.673126 204.0
GSVT 0.713064 0.708931 0.717331 300.0
SB 0.728293 0.725621 0.730986 892.0
SR 0.721964 0.722499 0.721437 681.0
macro avg 0.712975 0.715539 0.710720 2077.0
micro avg 0.720200 0.720200 0.720200 2077.0
weighted avg 0.720117 0.720172 0.720200 2077.0
The result of Model Number:Dataset Number:Featuregroup Number:Rescale of 48 : 0 : 10 : 0
f1-score precision recall support
AFIB 0.727326 0.727094 0.727597 875.0
GSVT 0.738166 0.728677 0.747991 691.0
SB 0.780787 0.779089 0.782496 1553.0

SR 0.759184 0.770313 0.748385 907.0
macro avg 0.751366 0.751293 0.751617 4026.0
micro avg 0.756958 0.756958 0.756958 4026.0
weighted avg 0.756986 0.757159 0.756958 4026.0
The result of Model Number:Dataset Number:Featuregroup Number:Rescale of 48 : 0 : 10 : 1

	f1-score	precision	recall	support
AFIB	0.717987	0.744697	0.693449	204.0
GSVT	0.666266	0.697183	0.638035	300.0
SB	0.697939	0.655843	0.745823	892.0
SR	0.612925	0.655511	0.575607	681.0
macro avg	0.673779	0.688309	0.663229	2077.0
micro avg	0.669300	0.669300	0.669300	2077.0
weighted avg	0.667459	0.670433	0.669300	2077.0

The result of Model Number:Dataset Number:Featuregroup Number:Rescale of 48 : 1 : 0 : 0

	f1-score	precision	recall	support
AFIB	0.778541	0.802327	0.756212	875.0
GSVT	0.826199	0.823400	0.829089	691.0
SB	0.902824	0.899197	0.906485	1553.0
SR	0.855075	0.840244	0.870523	907.0
macro avg	0.840660	0.841292	0.840577	4026.0
micro avg	0.852439	0.852439	0.852439	4026.0
weighted avg	0.851904	0.851853	0.852439	4026.0

The result of Model Number:Dataset Number:Featuregroup Number:Rescale of 48 : 1 : 0 : 1

	f1-score	precision	recall	support
AFIB	0.829800	0.816786	0.843326	875.0
GSVT	0.799567	0.822489	0.777980	691.0
SB	0.840212	0.797621	0.887656	1553.0
SR	0.682667	0.759670	0.619945	907.0
macro avg	0.788061	0.799141	0.782227	4026.0
micro avg	0.798886	0.798886	0.798886	4026.0
weighted avg	0.795480	0.797504	0.798886	4026.0

The result of Model Number:Dataset Number:Featuregroup Number:Rescale of 48 : 1 : 1 : 0

	f1-score	precision	recall	support
AFIB	0.910791	0.917709	0.904055	204.0
GSVT	0.929438	0.920360	0.938717	300.0
SB	0.951118	0.948687	0.953570	892.0
SR	0.941029	0.946345	0.935784	681.0
macro avg	0.933094	0.933275	0.933031	2077.0
micro avg	0.940730	0.940730	0.940730	2077.0
weighted avg	0.940718	0.940785	0.940730	2077.0

The result of Model Number:Dataset Number:Featuregroup Number:Rescale of 48 : 1 : 1 : 1

	f1-score	precision	recall	support
AFIB	0.620941	0.692542	0.562840	204.0
GSVT	0.732153	0.719409	0.745512	300.0
SB	0.781713	0.778248	0.785217	892.0
SR	0.763573	0.753132	0.774330	681.0
macro avg	0.724595	0.735833	0.716975	2077.0
micro avg	0.754071	0.754071	0.754071	2077.0
weighted avg	0.752816	0.753097	0.754071	2077.0

The result of Model Number:Dataset Number:Featuregroup Number:Rescale of 48 : 1 : 2 : 0

	f1-score	precision	recall	support
AFIB	0.808125	0.809525	0.806741	875.0
GSVT	0.817923	0.808525	0.827610	691.0
SB	0.861301	0.850916	0.871947	1553.0
SR	0.820361	0.845218	0.796943	907.0
macro avg	0.826927	0.828546	0.825810	4026.0
micro avg	0.833268	0.833268	0.833268	4026.0
weighted avg	0.833076	0.833360	0.833268	4026.0

The result of Model Number:Dataset Number:Featuregroup Number:Rescale of 48 : 1 : 2 : 1

	f1-score	precision	recall	support
AFIB	0.760661	0.771719	0.750261	204.0
GSVT	0.777437	0.766919	0.788310	300.0
SB	0.789138	0.780914	0.797543	892.0
SR	0.773497	0.786297	0.761121	681.0
macro avg	0.775183	0.776462	0.774309	2077.0
micro avg	0.779623	0.779623	0.779623	2077.0
weighted avg	0.779523	0.779754	0.779623	2077.0

The result of Model Number:Dataset Number:Featuregroup Number:Rescale of 48 : 1 : 3 : 0

	f1-score	precision	recall	support
AFIB	0.801729	0.809484	0.794266	204.0
GSVT	0.830099	0.828462	0.831755	300.0
SB	0.847449	0.845948	0.848967	892.0
SR	0.839637	0.840043	0.839266	681.0
macro avg	0.829728	0.830984	0.828563	2077.0
micro avg	0.837927	0.837927	0.837927	2077.0
weighted avg	0.837891	0.837905	0.837927	2077.0

The result of Model Number:Dataset Number:Featuregroup Number:Rescale of 48 : 1 : 3 : 1

	f1-score	precision	recall	support
AFIB	0.831305	0.830544	0.832168	875.0

GSVT 0.848345 0.846699 0.850086 691.0
SB 0.900204 0.898199 0.902232 1553.0
SR 0.875634 0.881346 0.870027 907.0
macro avg 0.863872 0.864197 0.863628 4026.0
micro avg 0.870799 0.870799 0.870799 4026.0
weighted avg 0.870794 0.870859 0.870799 4026.0
The result of Model Number:Dataset Number:Featuregroup Number:Rescale of 48 : 1 : 4 : 0

	f1-score	precision	recall	support
AFIB	0.699451	0.737500	0.665315	204.0
GSVT	0.734295	0.723189	0.745776	300.0
SB	0.751744	0.746854	0.756710	892.0
SR	0.742469	0.743139	0.741819	681.0
macro avg	0.731990	0.737670	0.727405	2077.0
micro avg	0.741272	0.741272	0.741272	2077.0
weighted avg	0.741047	0.741299	0.741272	2077.0

The result of Model Number:Dataset Number:Featuregroup Number:Rescale of 48 : 1 : 4 : 1

	f1-score	precision	recall	support
AFIB	0.758682	0.768729	0.749008	204.0
GSVT	0.781577	0.778756	0.784436	300.0
SB	0.791312	0.788849	0.793793	892.0
SR	0.782689	0.784197	0.781209	681.0
macro avg	0.778565	0.780133	0.777112	2077.0
micro avg	0.783917	0.783917	0.783917	2077.0
weighted avg	0.783874	0.783890	0.783917	2077.0

The result of Model Number:Dataset Number:Featuregroup Number:Rescale of 48 : 1 : 5 : 0

	f1-score	precision	recall	support
AFIB	0.855161	0.863433	0.847201	204.0
GSVT	0.885422	0.883676	0.887189	300.0
SB	0.903928	0.902327	0.905547	892.0
SR	0.895595	0.896028	0.895200	681.0
macro avg	0.885027	0.886366	0.883784	2077.0
micro avg	0.893772	0.893772	0.893772	2077.0
weighted avg	0.893733	0.893748	0.893772	2077.0

The result of Model Number:Dataset Number:Featuregroup Number:Rescale of 48 : 1 : 5 : 1

	f1-score	precision	recall	support
AFIB	0.722974	0.717444	0.728685	875.0
GSVT	0.739608	0.743653	0.735651	691.0
SB	0.783186	0.782637	0.783741	1553.0
SR	0.762383	0.766081	0.758760	907.0
macro avg	0.752038	0.752454	0.751709	4026.0

micro avg 0.757893 0.757893 0.757893 4026.0
weighted avg 0.757933 0.758047 0.757893 4026.0
The result of Model Number:Dataset Number:Featuregroup Number:Rescale of 48 : 1 : 6 : 0

	f1-score	precision	recall	support
AFIB	0.697291	0.698639	0.696001	875.0
GSVT	0.675557	0.672590	0.678592	691.0
SB	0.705877	0.662974	0.754737	1553.0
SR	0.568132	0.658300	0.499824	907.0
macro avg	0.661714	0.673125	0.657289	4026.0
micro avg	0.671474	0.671474	0.671474	4026.0
weighted avg	0.667775	0.671322	0.671474	4026.0

The result of Model Number:Dataset Number:Featuregroup Number:Rescale of 48 : 1 : 6 : 1

	f1-score	precision	recall	support
AFIB	0.857973	0.867506	0.849086	204.0
GSVT	0.884549	0.879514	0.889899	300.0
SB	0.902146	0.900937	0.903359	892.0
SR	0.894471	0.895670	0.893288	681.0
macro avg	0.884785	0.885907	0.883908	2077.0
micro avg	0.892782	0.892782	0.892782	2077.0
weighted avg	0.892749	0.892832	0.892782	2077.0

The result of Model Number:Dataset Number:Featuregroup Number:Rescale of 48 : 1 : 7 : 0

	f1-score	precision	recall	support
AFIB	0.661287	0.672026	0.650922	875.0
GSVT	0.703298	0.705503	0.701151	691.0
SB	0.765153	0.762517	0.767812	1553.0
SR	0.724377	0.716309	0.732673	907.0
macro avg	0.713529	0.714089	0.713140	4026.0
micro avg	0.723050	0.723050	0.723050	4026.0
weighted avg	0.722777	0.722654	0.723050	4026.0

The result of Model Number:Dataset Number:Featuregroup Number:Rescale of 48 : 1 : 7 : 1

	f1-score	precision	recall	support
AFIB	0.915894	0.924753	0.907368	204.0
GSVT	0.948303	0.946433	0.950196	300.0
SB	0.968124	0.966409	0.969858	892.0
SR	0.959199	0.959663	0.958776	681.0
macro avg	0.947880	0.949315	0.946549	2077.0
micro avg	0.957247	0.957247	0.957247	2077.0
weighted avg	0.957205	0.957221	0.957247	2077.0

The result of Model Number:Dataset Number:Featuregroup Number:Rescale of 48 : 1 : 8 : 0

	f1-score	precision	recall	support
AFIB	0.881383	0.905574	0.858516	204.0
GSVT	0.901317	0.887471	0.915689	300.0
SB	0.921120	0.912211	0.930208	892.0
SR	0.897840	0.909059	0.886901	681.0
macro avg	0.900415	0.903579	0.897828	2077.0
micro avg	0.906870	0.906870	0.906870	2077.0
weighted avg	0.906724	0.906952	0.906870	2077.0

The result of Model Number:Dataset Number:Featuregroup Number:Rescale of 48 : 1 : 8 : 1

	f1-score	precision	recall	support
AFIB	0.723953	0.739201	0.709436	204.0
GSVT	0.746462	0.739642	0.753426	300.0
SB	0.758653	0.756962	0.760354	892.0
SR	0.749637	0.750421	0.748863	681.0
macro avg	0.744676	0.746556	0.743020	2077.0
micro avg	0.750585	0.750585	0.750585	2077.0
weighted avg	0.750528	0.750571	0.750585	2077.0

The result of Model Number:Dataset Number:Featuregroup Number:Rescale of 48 : 1 : 9 : 0

	f1-score	precision	recall	support
AFIB	0.640170	0.631671	0.648950	875.0
GSVT	0.653163	0.659355	0.647115	691.0
SB	0.695059	0.693415	0.696714	1553.0
SR	0.676203	0.683314	0.669268	907.0
macro avg	0.666149	0.666939	0.665512	4026.0
micro avg	0.671637	0.671637	0.671637	4026.0
weighted avg	0.671691	0.671874	0.671637	4026.0

The result of Model Number:Dataset Number:Featuregroup Number:Rescale of 48 : 1 : 9 : 1

	f1-score	precision	recall	support
AFIB	0.730686	0.804898	0.669705	204.0
GSVT	0.674784	0.721446	0.634107	300.0
SB	0.734640	0.686428	0.790147	892.0
SR	0.635843	0.669266	0.605773	681.0
macro avg	0.693988	0.720510	0.674933	2077.0
micro avg	0.695327	0.695327	0.695327	2077.0
weighted avg	0.693213	0.697495	0.695327	2077.0

The result of Model Number:Dataset Number:Featuregroup Number:Rescale of 48 : 1 : 10 : 0

	f1-score	precision	recall	support
AFIB	0.650484	0.665307	0.636410	875.0
GSVT	0.692198	0.693566	0.691067	691.0
SB	0.757311	0.753166	0.761504	1553.0

SR 0.715772 0.707090 0.724771 907.0
macro avg 0.703941 0.704782 0.703438 4026.0
micro avg 0.713952 0.713952 0.713952 4026.0
weighted avg 0.713560 0.713461 0.713952 4026.0
The result of Model Number:Dataset Number:Featuregroup Number:Rescale of 48 : 1 : 10 : 1

	f1-score	precision	recall	support
AFIB	0.932958	0.944535	0.921975	204.0
GSVT	0.949687	0.943198	0.956365	300.0
SB	0.972177	0.970658	0.973701	892.0
SR	0.965193	0.966758	0.963641	681.0
macro avg	0.955004	0.956287	0.953921	2077.0
micro avg	0.962818	0.962818	0.962818	2077.0
weighted avg	0.962786	0.962847	0.962818	2077.0

The result of Model Number:Dataset Number:Featuregroup Number:Rescale of 49 : 0 : 0 : 0

	f1-score	precision	recall	support
AFIB	0.906773	0.914682	0.899227	204.0
GSVT	0.938132	0.936601	0.939692	300.0
SB	0.958073	0.955736	0.960433	892.0
SR	0.949289	0.950720	0.947895	681.0
macro avg	0.938067	0.939435	0.936812	2077.0
micro avg	0.947314	0.947314	0.947314	2077.0
weighted avg	0.947274	0.947295	0.947314	2077.0

The result of Model Number:Dataset Number:Featuregroup Number:Rescale of 49 : 0 : 0 : 1

	f1-score	precision	recall	support
AFIB	0.774818	0.796177	0.754730	204.0
GSVT	0.795438	0.780713	0.810805	300.0
SB	0.808338	0.800781	0.816041	892.0
SR	0.788020	0.798726	0.777611	681.0
macro avg	0.791654	0.794099	0.789797	2077.0
micro avg	0.796663	0.796663	0.796663	2077.0
weighted avg	0.796521	0.796757	0.796663	2077.0

The result of Model Number:Dataset Number:Featuregroup Number:Rescale of 49 : 0 : 1 : 0

	f1-score	precision	recall	support
AFIB	0.862570	0.892515	0.834686	204.0
GSVT	0.879109	0.869485	0.889056	300.0
SB	0.902512	0.896032	0.909088	892.0
SR	0.886394	0.890752	0.882093	681.0
macro avg	0.882646	0.887196	0.878731	2077.0
micro avg	0.890036	0.890036	0.890036	2077.0
weighted avg	0.889924	0.890121	0.890036	2077.0

The result of Model Number:Dataset Number:Featuregroup Number:Rescale of 49 : 0 : 1 : 1

	f1-score	precision	recall	support
AFIB	0.853023	0.859910	0.846319	204.0
GSVT	0.872596	0.868318	0.876948	300.0
SB	0.889627	0.888640	0.890623	892.0
SR	0.882984	0.884158	0.881819	681.0
macro avg	0.874557	0.875256	0.873927	2077.0
micro avg	0.881410	0.881410	0.881410	2077.0
weighted avg	0.881394	0.881413	0.881410	2077.0

The result of Model Number:Dataset Number:Featuregroup Number:Rescale of 49 : 0 : 2 : 0

	f1-score	precision	recall	support
AFIB	0.691142	0.704652	0.678262	204.0
GSVT	0.710133	0.705017	0.715349	300.0
SB	0.722843	0.720030	0.725679	892.0
SR	0.714942	0.716965	0.712945	681.0
macro avg	0.709765	0.711666	0.708059	2077.0
micro avg	0.715355	0.715355	0.715355	2077.0
weighted avg	0.715303	0.715346	0.715355	2077.0

The result of Model Number:Dataset Number:Featuregroup Number:Rescale of 49 : 0 : 2 : 1

	f1-score	precision	recall	support
AFIB	0.879905	0.903687	0.857525	204.0
GSVT	0.887157	0.874098	0.900735	300.0
SB	0.904342	0.894721	0.914175	892.0
SR	0.887569	0.899765	0.875714	681.0
macro avg	0.889743	0.893068	0.887037	2077.0
micro avg	0.894059	0.894059	0.894059	2077.0
weighted avg	0.893960	0.894277	0.894059	2077.0

The result of Model Number:Dataset Number:Featuregroup Number:Rescale of 49 : 0 : 3 : 0

	f1-score	precision	recall	support
AFIB	0.788616	0.790209	0.787047	204.0
GSVT	0.810556	0.808146	0.812985	300.0
SB	0.823987	0.823253	0.824725	892.0
SR	0.816904	0.818473	0.815352	681.0
macro avg	0.810016	0.810020	0.810027	2077.0
micro avg	0.816256	0.816256	0.816256	2077.0
weighted avg	0.816251	0.816258	0.816256	2077.0

The result of Model Number:Dataset Number:Featuregroup Number:Rescale of 49 : 0 : 3 : 1

	f1-score	precision	recall	support
AFIB	0.929597	0.946681	0.913233	204.0

GSVT 0.945212 0.939022 0.951509 300.0
SB 0.961380 0.956686 0.966122 892.0
SR 0.951443 0.955399 0.947534 681.0
macro avg 0.946908 0.949447 0.944600 2077.0
micro avg 0.952722 0.952722 0.952722 2077.0
weighted avg 0.952665 0.952730 0.952722 2077.0
The result of Model Number:Dataset Number:Featuregroup Number:Rescale of 49 : 0 : 4 : 0

	f1-score	precision	recall	support
AFIB	0.822194	0.834273	0.810535	204.0
GSVT	0.843026	0.835859	0.850334	300.0
SB	0.857596	0.854165	0.861057	892.0
SR	0.849114	0.853282	0.844997	681.0
macro avg	0.842982	0.844395	0.841731	2077.0
micro avg	0.849280	0.849280	0.849280	2077.0
weighted avg	0.849233	0.849277	0.849280	2077.0

The result of Model Number:Dataset Number:Featuregroup Number:Rescale of 49 : 0 : 4 : 1

	f1-score	precision	recall	support
AFIB	0.782902	0.784846	0.781019	875.0
GSVT	0.796384	0.798777	0.794107	691.0
SB	0.844820	0.839086	0.850639	1553.0
SR	0.828744	0.834896	0.822714	907.0
macro avg	0.813212	0.814401	0.812120	4026.0
micro avg	0.819514	0.819514	0.819514	4026.0
weighted avg	0.819428	0.819435	0.819514	4026.0

The result of Model Number:Dataset Number:Featuregroup Number:Rescale of 49 : 0 : 5 : 0

	f1-score	precision	recall	support
AFIB	0.738535	0.729106	0.748254	875.0
GSVT	0.751498	0.755808	0.747258	691.0
SB	0.804432	0.801054	0.807847	1553.0
SR	0.780746	0.793383	0.768519	907.0
macro avg	0.768803	0.769838	0.767969	4026.0
micro avg	0.775636	0.775636	0.775636	4026.0
weighted avg	0.775689	0.775923	0.775636	4026.0

The result of Model Number:Dataset Number:Featuregroup Number:Rescale of 49 : 0 : 5 : 1

	f1-score	precision	recall	support
AFIB	0.790094	0.790305	0.790021	875.0
GSVT	0.803852	0.800884	0.806943	691.0
SB	0.859965	0.857877	0.862067	1553.0
SR	0.839456	0.845480	0.833539	907.0
macro avg	0.823341	0.823637	0.823142	4026.0

micro avg 0.830520 0.830520 0.830520 4026.0
weighted avg 0.830528 0.830616 0.830520 4026.0
The result of Model Number:Dataset Number:Featuregroup Number:Rescale of 49 : 0 : 6 :
0
f1-score precision recall support
AFIB 0.676321 0.744555 0.619939 204.0
GSVT 0.797580 0.786571 0.809028 300.0
SB 0.846875 0.844049 0.849723 892.0
SR 0.825187 0.813771 0.836940 681.0
macro avg 0.786491 0.797237 0.778908 2077.0
micro avg 0.817085 0.817085 0.817085 2077.0
weighted avg 0.815892 0.816048 0.817085 2077.0
The result of Model Number:Dataset Number:Featuregroup Number:Rescale of 49 : 0 : 6 :
1
f1-score precision recall support
AFIB 0.746498 0.755036 0.738439 204.0
GSVT 0.763971 0.759450 0.768558 300.0
SB 0.733398 0.706307 0.779126 892.0
SR 0.615205 0.618182 0.612272 681.0
macro avg 0.714768 0.709744 0.724599 2077.0
micro avg 0.718896 0.718896 0.718896 2077.0
weighted avg 0.700348 0.689875 0.718896 2077.0
The result of Model Number:Dataset Number:Featuregroup Number:Rescale of 49 : 0 : 7 :
0
f1-score precision recall support
AFIB 0.871446 0.875412 0.867570 875.0
GSVT 0.875681 0.866953 0.884661 691.0
SB 0.922969 0.912912 0.933251 1553.0
SR 0.882720 0.903618 0.862771 907.0
macro avg 0.888204 0.889724 0.887063 4026.0
micro avg 0.894758 0.894758 0.894758 4026.0
weighted avg 0.894587 0.894780 0.894758 4026.0
The result of Model Number:Dataset Number:Featuregroup Number:Rescale of 49 : 0 : 7 :
1
f1-score precision recall support
AFIB 0.711111 0.716428 0.705899 204.0
GSVT 0.729281 0.725432 0.733171 300.0
SB 0.741588 0.740596 0.742585 892.0
SR 0.735057 0.736466 0.733655 681.0
macro avg 0.729259 0.729731 0.728827 2077.0
micro avg 0.734694 0.734694 0.734694 2077.0
weighted avg 0.734675 0.734678 0.734694 2077.0
The result of Model Number:Dataset Number:Featuregroup Number:Rescale of 49 : 0 : 8 :
0

	f1-score	precision	recall	support
AFIB	0.749086	0.740250	0.758172	875.0
GSVT	0.761775	0.766595	0.757043	691.0
SB	0.812199	0.811107	0.813298	1553.0
SR	0.792246	0.799752	0.784907	907.0
macro avg	0.778827	0.779426	0.778355	4026.0
micro avg	0.785266	0.785266	0.785266	4026.0
weighted avg	0.785333	0.785509	0.785266	4026.0

The result of Model Number:Dataset Number:Featuregroup Number:Rescale of 49 : 0 : 8 : 1

	f1-score	precision	recall	support
AFIB	0.629433	0.620495	0.638701	875.0
GSVT	0.652259	0.660580	0.644244	691.0
SB	0.693172	0.692155	0.694199	1553.0
SR	0.675903	0.680973	0.670910	907.0
macro avg	0.662692	0.663551	0.662013	4026.0
micro avg	0.668316	0.668316	0.668316	4026.0
weighted avg	0.668407	0.668642	0.668316	4026.0

The result of Model Number:Dataset Number:Featuregroup Number:Rescale of 49 : 0 : 9 : 0

	f1-score	precision	recall	support
AFIB	0.894461	0.907874	0.881693	204.0
GSVT	0.926761	0.924300	0.929240	300.0
SB	0.948714	0.947450	0.949990	892.0
SR	0.939435	0.938224	0.940680	681.0
macro avg	0.927343	0.929462	0.925401	2077.0
micro avg	0.937232	0.937232	0.937232	2077.0
weighted avg	0.937172	0.937194	0.937232	2077.0

The result of Model Number:Dataset Number:Featuregroup Number:Rescale of 49 : 0 : 9 : 1

	f1-score	precision	recall	support
AFIB	0.739370	0.727774	0.751422	875.0
GSVT	0.712431	0.732855	0.693198	691.0
SB	0.748647	0.710698	0.790921	1553.0
SR	0.608271	0.676882	0.552384	907.0
macro avg	0.702180	0.712052	0.696981	4026.0
micro avg	0.711825	0.711825	0.711825	4026.0
weighted avg	0.708790	0.710594	0.711825	4026.0

The result of Model Number:Dataset Number:Featuregroup Number:Rescale of 49 : 0 : 10 : 0

	f1-score	precision	recall	support
AFIB	0.702458	0.688271	0.717297	875.0
GSVT	0.725747	0.737286	0.714720	691.0
SB	0.772206	0.772162	0.772255	1553.0

SR 0.753483 0.760349 0.746753 907.0
macro avg 0.738473 0.739517 0.737756 4026.0
micro avg 0.744690 0.744690 0.744690 4026.0
weighted avg 0.744855 0.745282 0.744690 4026.0
The result of Model Number:Dataset Number:Featuregroup Number:Rescale of 49 : 0 : 10 : 1

	f1-score	precision	recall	support
AFIB	0.822266	0.800666	0.873743	875.0
GSVT	0.791013	0.748703	0.901738	691.0
SB	0.749525	0.744789	0.754345	1553.0
SR	0.728045	0.740589	0.716122	907.0
macro avg	0.772712	0.758687	0.811487	4026.0
micro avg	0.796981	0.796981	0.796981	4026.0
weighted avg	0.767616	0.756659	0.796981	4026.0

The result of Model Number:Dataset Number:Featuregroup Number:Rescale of 49 : 1 : 0 : 0

	f1-score	precision	recall	support
AFIB	0.753732	0.831207	0.689932	204.0
GSVT	0.896920	0.884798	0.909466	300.0
SB	0.955155	0.951336	0.959010	892.0
SR	0.929231	0.916670	0.942181	681.0
macro avg	0.883760	0.896003	0.875147	2077.0
micro avg	0.919908	0.919908	0.919908	2077.0
weighted avg	0.918460	0.918560	0.919908	2077.0

The result of Model Number:Dataset Number:Featuregroup Number:Rescale of 49 : 1 : 0 : 1

	f1-score	precision	recall	support
AFIB	0.777762	0.779155	0.776398	875.0
GSVT	0.744759	0.748671	0.740908	691.0
SB	0.787774	0.744439	0.836474	1553.0
SR	0.654250	0.736406	0.588643	907.0
macro avg	0.741136	0.752168	0.735606	4026.0
micro avg	0.751182	0.751182	0.751182	4026.0
weighted avg	0.748134	0.750901	0.751182	4026.0

The result of Model Number:Dataset Number:Featuregroup Number:Rescale of 49 : 1 : 1 : 0

	f1-score	precision	recall	support
AFIB	0.797082	0.795792	0.798446	875.0
GSVT	0.804385	0.807584	0.801252	691.0
SB	0.844745	0.841674	0.847844	1553.0
SR	0.830927	0.835131	0.826812	907.0
macro avg	0.819285	0.820045	0.818589	4026.0
micro avg	0.824373	0.824373	0.824373	4026.0
weighted avg	0.824346	0.824377	0.824373	4026.0

The result of Model Number:Dataset Number:Featuregroup Number:Rescale of 49 : 1 : 1 : 1

	f1-score	precision	recall	support
AFIB	0.774505	0.773259	0.776071	875.0
GSVT	0.792563	0.788432	0.796784	691.0
SB	0.828666	0.821645	0.835814	1553.0
SR	0.797032	0.814752	0.780336	907.0
macro avg	0.798192	0.799522	0.797251	4026.0
micro avg	0.803632	0.803632	0.803632	4026.0
weighted avg	0.803572	0.803876	0.803632	4026.0

The result of Model Number:Dataset Number:Featuregroup Number:Rescale of 49 : 1 : 2 : 0

	f1-score	precision	recall	support
AFIB	0.828290	0.897435	0.770688	204.0
GSVT	0.888437	0.877266	0.900259	300.0
SB	0.911118	0.898353	0.924356	892.0
SR	0.886264	0.890416	0.882583	681.0
macro avg	0.878527	0.890868	0.869471	2077.0
micro avg	0.892086	0.892086	0.892086	2077.0
weighted avg	0.891558	0.892615	0.892086	2077.0

The result of Model Number:Dataset Number:Featuregroup Number:Rescale of 49 : 1 : 2 : 1

	f1-score	precision	recall	support
AFIB	0.866270	0.874065	0.858885	875.0
GSVT	0.883916	0.865994	0.902805	691.0
SB	0.937399	0.926971	0.948178	1553.0
SR	0.900202	0.926961	0.875156	907.0
macro avg	0.896947	0.898498	0.896256	4026.0
micro avg	0.904533	0.904533	0.904533	4026.0
weighted avg	0.904381	0.905004	0.904533	4026.0

The result of Model Number:Dataset Number:Featuregroup Number:Rescale of 49 : 1 : 3 : 0

	f1-score	precision	recall	support
AFIB	0.867471	0.899632	0.837653	204.0
GSVT	0.893459	0.882287	0.905030	300.0
SB	0.916115	0.907756	0.924637	892.0
SR	0.892030	0.898952	0.885222	681.0
macro avg	0.892268	0.897157	0.888135	2077.0
micro avg	0.900338	0.900338	0.900338	2077.0
weighted avg	0.900168	0.900393	0.900338	2077.0

The result of Model Number:Dataset Number:Featuregroup Number:Rescale of 49 : 1 : 3 : 1

	f1-score	precision	recall	support
AFIB	0.805219	0.794863	0.815906	875.0

GSVT 0.821611 0.828413 0.814953 691.0
SB 0.878467 0.874557 0.882422 1553.0
SR 0.853468 0.866091 0.841228 907.0
macro avg 0.839691 0.840981 0.838627 4026.0
micro avg 0.847105 0.847105 0.847105 4026.0
weighted avg 0.847157 0.847409 0.847105 4026.0
The result of Model Number:Dataset Number:Featuregroup Number:Rescale of 49 : 1 : 4 : 0

	f1-score	precision	recall	support
AFIB	0.731351	0.725720	0.737126	875.0
GSVT	0.740846	0.744286	0.737600	691.0
SB	0.793920	0.789696	0.798206	1553.0
SR	0.770529	0.781362	0.760076	907.0
macro avg	0.759161	0.760266	0.758252	4026.0
micro avg	0.765939	0.765939	0.765939	4026.0
weighted avg	0.765942	0.766120	0.765939	4026.0

The result of Model Number:Dataset Number:Featuregroup Number:Rescale of 49 : 1 : 4 : 1

	f1-score	precision	recall	support
AFIB	0.809379	0.824227	0.795118	204.0
GSVT	0.820054	0.811778	0.828517	300.0
SB	0.840254	0.835880	0.844679	892.0
SR	0.828814	0.834008	0.823703	681.0
macro avg	0.824625	0.826473	0.823004	2077.0
micro avg	0.830599	0.830599	0.830599	2077.0
weighted avg	0.830553	0.830640	0.830599	2077.0

The result of Model Number:Dataset Number:Featuregroup Number:Rescale of 49 : 1 : 5 : 0

	f1-score	precision	recall	support
AFIB	0.749834	0.742804	0.757093	875.0
GSVT	0.710914	0.693827	0.763174	691.0
SB	0.822039	0.819020	0.825103	1553.0
SR	0.639367	0.643851	0.634954	907.0
macro avg	0.730539	0.724876	0.745081	4026.0
micro avg	0.756855	0.756855	0.756855	4026.0
weighted avg	0.746120	0.741505	0.756855	4026.0

The result of Model Number:Dataset Number:Featuregroup Number:Rescale of 49 : 1 : 5 : 1

	f1-score	precision	recall	support
AFIB	0.666191	0.680670	0.652497	204.0
GSVT	0.693690	0.689599	0.697850	300.0
SB	0.708594	0.707022	0.710181	892.0
SR	0.701490	0.701094	0.701904	681.0
macro avg	0.692491	0.694596	0.690608	2077.0

micro avg 0.700020 0.700020 0.700020 2077.0
weighted avg 0.699947 0.699974 0.700020 2077.0
The result of Model Number:Dataset Number:Featuregroup Number:Rescale of 49 : 1 : 6 : 0

	f1-score	precision	recall	support
AFIB	0.742229	0.743664	0.740857	875.0
GSVT	0.719095	0.715936	0.722325	691.0
SB	0.751369	0.705700	0.803378	1553.0
SR	0.604747	0.700725	0.532037	907.0
macro avg	0.704360	0.716506	0.699649	4026.0
micro avg	0.714749	0.714749	0.714749	4026.0
weighted avg	0.710811	0.714587	0.714749	4026.0

The result of Model Number:Dataset Number:Featuregroup Number:Rescale of 49 : 1 : 6 : 1

	f1-score	precision	recall	support
AFIB	0.706661	0.722020	0.692135	204.0
GSVT	0.735831	0.731491	0.740244	300.0
SB	0.751640	0.749973	0.753323	892.0
SR	0.744105	0.743685	0.744544	681.0
macro avg	0.734559	0.736792	0.732562	2077.0
micro avg	0.742546	0.742546	0.742546	2077.0
weighted avg	0.742468	0.742496	0.742546	2077.0

The result of Model Number:Dataset Number:Featuregroup Number:Rescale of 49 : 1 : 7 : 0

	f1-score	precision	recall	support
AFIB	0.682422	0.696736	0.668702	204.0
GSVT	0.698253	0.694121	0.702441	300.0
SB	0.715276	0.713600	0.716959	892.0
SR	0.709234	0.709030	0.709444	681.0
macro avg	0.701296	0.703372	0.699387	2077.0
micro avg	0.707658	0.707658	0.707658	2077.0
weighted avg	0.707609	0.707632	0.707658	2077.0

The result of Model Number:Dataset Number:Featuregroup Number:Rescale of 49 : 1 : 7 : 1

	f1-score	precision	recall	support
AFIB	0.896261	0.906220	0.886978	204.0
GSVT	0.924023	0.918763	0.929612	300.0
SB	0.942405	0.941142	0.943673	892.0
SR	0.934388	0.935640	0.933152	681.0
macro avg	0.924269	0.925441	0.923354	2077.0
micro avg	0.932624	0.932624	0.932624	2077.0
weighted avg	0.932589	0.932676	0.932624	2077.0

The result of Model Number:Dataset Number:Featuregroup Number:Rescale of 49 : 1 : 8 : 0

	f1-score	precision	recall	support
AFIB	0.664392	0.666817	0.662029	875.0
GSVT	0.675876	0.667075	0.684970	691.0
SB	0.712441	0.704594	0.720469	1553.0
SR	0.675190	0.693842	0.657552	907.0
macro avg	0.681975	0.683082	0.681255	4026.0
micro avg	0.687501	0.687501	0.687501	4026.0
weighted avg	0.687330	0.687522	0.687501	4026.0

The result of Model Number:Dataset Number:Featuregroup Number:Rescale of 49 : 1 : 8 : 1

	f1-score	precision	recall	support
AFIB	0.763566	0.753746	0.773700	875.0
GSVT	0.779109	0.785560	0.772796	691.0
SB	0.833025	0.829317	0.836775	1553.0
SR	0.809319	0.821289	0.797712	907.0
macro avg	0.796255	0.797478	0.795246	4026.0
micro avg	0.803285	0.803285	0.803285	4026.0
weighted avg	0.803334	0.803574	0.803285	4026.0

The result of Model Number:Dataset Number:Featuregroup Number:Rescale of 49 : 1 : 9 : 0

	f1-score	precision	recall	support
AFIB	0.642608	0.634002	0.651492	875.0
GSVT	0.656976	0.662104	0.651938	691.0
SB	0.697525	0.695343	0.699725	1553.0
SR	0.680657	0.689681	0.671879	907.0
macro avg	0.669442	0.670282	0.668759	4026.0
micro avg	0.674767	0.674767	0.674767	4026.0
weighted avg	0.674830	0.675031	0.674767	4026.0

The result of Model Number:Dataset Number:Featuregroup Number:Rescale of 49 : 1 : 9 : 1

	f1-score	precision	recall	support
AFIB	0.615949	0.629877	0.602641	875.0
GSVT	0.653046	0.655825	0.650328	691.0
SB	0.709072	0.705730	0.712450	1553.0
SR	0.671836	0.661531	0.682496	907.0
macro avg	0.662476	0.663241	0.661979	4026.0
micro avg	0.671174	0.671174	0.671174	4026.0
weighted avg	0.670828	0.670722	0.671174	4026.0

The result of Model Number:Dataset Number:Featuregroup Number:Rescale of 49 : 1 : 10 : 0

	f1-score	precision	recall	support
AFIB	0.695673	0.709272	0.682709	204.0
GSVT	0.714789	0.709639	0.720039	300.0
SB	0.727582	0.724750	0.730437	892.0

SR 0.719629 0.721665 0.717619 681.0
macro avg 0.714418 0.716332 0.712701 2077.0
micro avg 0.720044 0.720044 0.720044 2077.0
weighted avg 0.719992 0.720036 0.720044 2077.0
The result of Model Number:Dataset Number:Featuregroup Number:Rescale of 49 : 1 : 10 : 1

	f1-score	precision	recall	support
AFIB	0.584902	0.646081	0.534479	204.0
GSVT	0.688097	0.679012	0.697502	300.0
SB	0.729724	0.726888	0.732587	892.0
SR	0.712189	0.702039	0.722645	681.0
macro avg	0.678728	0.688505	0.671803	2077.0
micro avg	0.704802	0.704802	0.704802	2077.0
weighted avg	0.703738	0.703889	0.704802	2077.0

The result of Model Number:Dataset Number:Featuregroup Number:Rescale of 50 : 0 : 0 : 0

	f1-score	precision	recall	support
AFIB	0.711274	0.694697	0.728777	875.0
GSVT	0.682798	0.690304	0.675500	691.0
SB	0.718536	0.672137	0.771832	1553.0
SR	0.575076	0.690555	0.492993	907.0
macro avg	0.671921	0.686923	0.667276	4026.0
micro avg	0.683122	0.683122	0.683122	4026.0
weighted avg	0.678504	0.684308	0.683122	4026.0

The result of Model Number:Dataset Number:Featuregroup Number:Rescale of 50 : 0 : 0 : 1

	f1-score	precision	recall	support
AFIB	0.789198	0.807044	0.772147	875.0
GSVT	0.836729	0.840290	0.833247	691.0
SB	0.908514	0.904232	0.912842	1553.0
SR	0.860805	0.847601	0.874462	907.0
macro avg	0.848811	0.849792	0.848175	4026.0
micro avg	0.859956	0.859956	0.859956	4026.0
weighted avg	0.859513	0.859377	0.859956	4026.0

The result of Model Number:Dataset Number:Featuregroup Number:Rescale of 50 : 0 : 1 : 0

	f1-score	precision	recall	support
AFIB	0.859111	0.875875	0.843125	204.0
GSVT	0.880523	0.869161	0.892244	300.0
SB	0.895510	0.886656	0.904545	892.0
SR	0.876792	0.888974	0.864945	681.0
macro avg	0.877984	0.880167	0.876215	2077.0
micro avg	0.883752	0.883752	0.883752	2077.0
weighted avg	0.883633	0.883830	0.883752	2077.0

The result of Model Number:Dataset Number:Featuregroup Number:Rescale of 50 : 0 : 1 : 1

	f1-score	precision	recall	support
AFIB	0.868050	0.849501	0.887478	875.0
GSVT	0.898921	0.912667	0.885663	691.0
SB	0.958248	0.958375	0.958125	1553.0
SR	0.933561	0.943229	0.924093	907.0
macro avg	0.914695	0.915943	0.913840	4026.0
micro avg	0.922667	0.922667	0.922667	4026.0
weighted avg	0.922900	0.923455	0.922667	4026.0

The result of Model Number:Dataset Number:Featuregroup Number:Rescale of 50 : 0 : 2 : 0

	f1-score	precision	recall	support
AFIB	0.778081	0.770254	0.786089	875.0
GSVT	0.785044	0.792033	0.778234	691.0
SB	0.830542	0.827993	0.833108	1553.0
SR	0.814458	0.821588	0.807455	907.0
macro avg	0.802031	0.802967	0.801221	4026.0
micro avg	0.807691	0.807691	0.807691	4026.0
weighted avg	0.807708	0.807829	0.807691	4026.0

The result of Model Number:Dataset Number:Featuregroup Number:Rescale of 50 : 0 : 2 : 1

	f1-score	precision	recall	support
AFIB	0.858239	0.869702	0.847315	204.0
GSVT	0.873400	0.868080	0.878920	300.0
SB	0.894454	0.893661	0.895254	892.0
SR	0.887844	0.887979	0.887715	681.0
macro avg	0.878484	0.879855	0.877301	2077.0
micro avg	0.885715	0.885715	0.885715	2077.0
weighted avg	0.885689	0.885750	0.885715	2077.0

The result of Model Number:Dataset Number:Featuregroup Number:Rescale of 50 : 0 : 3 : 0

	f1-score	precision	recall	support
AFIB	0.854985	0.845010	0.867428	875.0
GSVT	0.879728	0.869064	0.891365	691.0
SB	0.892988	0.858518	0.943548	1553.0
SR	0.709079	0.746482	0.675287	907.0
macro avg	0.834195	0.829769	0.844407	4026.0
micro avg	0.857613	0.857613	0.857613	4026.0
weighted avg	0.841020	0.832152	0.857613	4026.0

The result of Model Number:Dataset Number:Featuregroup Number:Rescale of 50 : 0 : 3 : 1

	f1-score	precision	recall	support
AFIB	0.845298	0.836795	0.853999	875.0

GSVT	0.852862	0.860456	0.845465	691.0
SB	0.902291	0.899522	0.905079	1553.0
SR	0.884818	0.892563	0.877210	907.0
macro avg	0.871317	0.872334	0.870438	4026.0
micro avg	0.877467	0.877467	0.877467	4026.0
weighted avg	0.877484	0.877616	0.877467	4026.0

The result of Model Number:Dataset Number:Featuregroup Number:Rescale of 50 : 0 : 4 : 0

	f1-score	precision	recall	support
AFIB	0.796057	0.786154	0.806301	875.0
GSVT	0.820980	0.831260	0.811032	691.0
SB	0.875871	0.874922	0.876828	1553.0
SR	0.852247	0.856618	0.847926	907.0
macro avg	0.836289	0.837238	0.835522	4026.0
micro avg	0.843696	0.843696	0.843696	4026.0
weighted avg	0.843781	0.844012	0.843696	4026.0

The result of Model Number:Dataset Number:Featuregroup Number:Rescale of 50 : 0 : 4 : 1

	f1-score	precision	recall	support
AFIB	0.718779	0.724462	0.713210	875.0
GSVT	0.736866	0.735308	0.738458	691.0
SB	0.785521	0.778578	0.792599	1553.0
SR	0.764609	0.772108	0.757273	907.0
macro avg	0.751444	0.752614	0.750385	4026.0
micro avg	0.758094	0.758094	0.758094	4026.0
weighted avg	0.757954	0.757932	0.758094	4026.0

The result of Model Number:Dataset Number:Featuregroup Number:Rescale of 50 : 0 : 5 : 0

	f1-score	precision	recall	support
AFIB	0.670588	0.683696	0.658091	204.0
GSVT	0.689014	0.684050	0.694075	300.0
SB	0.701346	0.698617	0.704098	892.0
SR	0.693680	0.695643	0.691743	681.0
macro avg	0.688657	0.690502	0.687002	2077.0
micro avg	0.694081	0.694081	0.694081	2077.0
weighted avg	0.694030	0.694072	0.694081	2077.0

The result of Model Number:Dataset Number:Featuregroup Number:Rescale of 50 : 0 : 5 : 1

	f1-score	precision	recall	support
AFIB	0.922047	0.930453	0.913913	204.0
GSVT	0.946109	0.943012	0.949257	300.0
SB	0.963405	0.962763	0.964052	892.0
SR	0.954932	0.954658	0.955220	681.0
macro avg	0.946623	0.947721	0.945610	2077.0

micro avg	0.954095	0.954095	0.954095	2077.0
weighted avg	0.954067	0.954079	0.954095	2077.0

The result of Model Number:Dataset Number:Featuregroup Number:Rescale of 50 : 0 : 6 : 0

	f1-score	precision	recall	support
AFIB	0.858632	0.879185	0.840274	204.0
GSVT	0.883916	0.871462	0.897101	300.0
SB	0.906883	0.897669	0.916329	892.0
SR	0.885207	0.897989	0.872855	681.0
macro avg	0.883659	0.886576	0.881640	2077.0
micro avg	0.891828	0.891828	0.891828	2077.0
weighted avg	0.891719	0.892173	0.891828	2077.0

The result of Model Number:Dataset Number:Featuregroup Number:Rescale of 50 : 0 : 6 : 1

	f1-score	precision	recall	support
AFIB	0.834828	0.865779	0.806132	204.0
GSVT	0.859838	0.849087	0.870974	300.0
SB	0.881642	0.873597	0.889844	892.0
SR	0.858463	0.865125	0.851911	681.0
macro avg	0.858693	0.863397	0.854715	2077.0
micro avg	0.866459	0.866459	0.866459	2077.0
weighted avg	0.866295	0.866511	0.866459	2077.0

The result of Model Number:Dataset Number:Featuregroup Number:Rescale of 50 : 0 : 7 : 0

	f1-score	precision	recall	support
AFIB	0.821480	0.811791	0.831445	875.0
GSVT	0.835396	0.840682	0.830206	691.0
SB	0.890693	0.889496	0.891898	1553.0
SR	0.868812	0.877043	0.860763	907.0
macro avg	0.854095	0.854753	0.853578	4026.0
micro avg	0.861157	0.861157	0.861157	4026.0
weighted avg	0.861230	0.861424	0.861157	4026.0

The result of Model Number:Dataset Number:Featuregroup Number:Rescale of 50 : 0 : 7 : 1

	f1-score	precision	recall	support
AFIB	0.828088	0.819969	0.885392	204.0
GSVT	0.742508	0.734253	0.751137	300.0
SB	0.954169	0.947221	0.961227	892.0
SR	0.938167	0.944665	0.931834	681.0
macro avg	0.865733	0.861527	0.882397	2077.0
micro avg	0.913796	0.913796	0.913796	2077.0
weighted avg	0.905967	0.903123	0.913796	2077.0

The result of Model Number:Dataset Number:Featuregroup Number:Rescale of 50 : 0 : 8 : 0

	f1-score	precision	recall	support
AFIB	0.686306	0.681551	0.691265	875.0
GSVT	0.696894	0.699722	0.694201	691.0
SB	0.756131	0.748269	0.764164	1553.0
SR	0.717953	0.734659	0.702056	907.0
macro avg	0.714321	0.716050	0.712922	4026.0
micro avg	0.722320	0.722320	0.722320	4026.0
weighted avg	0.722187	0.722370	0.722320	4026.0

The result of Model Number:Dataset Number:Featuregroup Number:Rescale of 50 : 0 : 8 : 1

	f1-score	precision	recall	support
AFIB	0.706486	0.698153	0.715049	875.0
GSVT	0.720369	0.726634	0.714217	691.0
SB	0.767735	0.764797	0.770699	1553.0
SR	0.749621	0.758684	0.740780	907.0
macro avg	0.736052	0.737067	0.735186	4026.0
micro avg	0.742169	0.742169	0.742169	4026.0
weighted avg	0.742213	0.742385	0.742169	4026.0

The result of Model Number:Dataset Number:Featuregroup Number:Rescale of 50 : 0 : 9 : 0

	f1-score	precision	recall	support
AFIB	0.842146	0.829234	0.855607	875.0
GSVT	0.871185	0.879724	0.862981	691.0
SB	0.927173	0.925007	0.929379	1553.0
SR	0.904012	0.915474	0.892853	907.0
macro avg	0.886129	0.887360	0.885205	4026.0
micro avg	0.893720	0.893720	0.893720	4026.0
weighted avg	0.893866	0.894272	0.893720	4026.0

The result of Model Number:Dataset Number:Featuregroup Number:Rescale of 50 : 0 : 9 : 1

	f1-score	precision	recall	support
AFIB	0.677636	0.728576	0.634060	204.0
GSVT	0.646241	0.666753	0.627051	300.0
SB	0.673291	0.639974	0.710273	892.0
SR	0.596014	0.621632	0.572485	681.0
macro avg	0.648295	0.664234	0.635967	2077.0
micro avg	0.645589	0.645589	0.645589	2077.0
weighted avg	0.644473	0.646531	0.645589	2077.0

The result of Model Number:Dataset Number:Featuregroup Number:Rescale of 50 : 0 : 10 : 0

	f1-score	precision	recall	support
AFIB	0.841098	0.867004	0.816864	204.0
GSVT	0.862556	0.851997	0.873474	300.0
SB	0.885984	0.880720	0.891341	892.0

SR 0.871050 0.875346 0.866830 681.0
macro avg 0.865172 0.868767 0.862127 2077.0
micro avg 0.873409 0.873409 0.873409 2077.0
weighted avg 0.873295 0.873462 0.873409 2077.0
The result of Model Number:Dataset Number:Featuregroup Number:Rescale of 50 : 0 : 10 : 1

	f1-score	precision	recall	support
AFIB	0.885570	0.877652	0.893671	875.0
GSVT	0.899789	0.903349	0.896289	691.0
SB	0.959367	0.957587	0.961158	1553.0
SR	0.935057	0.943700	0.926604	907.0
macro avg	0.919946	0.920572	0.919430	4026.0
micro avg	0.927572	0.927572	0.927572	4026.0
weighted avg	0.927626	0.927776	0.927572	4026.0

The result of Model Number:Dataset Number:Featuregroup Number:Rescale of 50 : 1 : 0 : 0

	f1-score	precision	recall	support
AFIB	0.868792	0.867822	0.869791	875.0
GSVT	0.884684	0.873619	0.896058	691.0
SB	0.929356	0.921884	0.936956	1553.0
SR	0.887892	0.911188	0.865772	907.0
macro avg	0.892681	0.893628	0.892144	4026.0
micro avg	0.899302	0.899302	0.899302	4026.0
weighted avg	0.899185	0.899441	0.899302	4026.0

The result of Model Number:Dataset Number:Featuregroup Number:Rescale of 50 : 1 : 0 : 1

	f1-score	precision	recall	support
AFIB	0.870249	0.864220	0.876538	875.0
GSVT	0.883675	0.887261	0.880261	691.0
SB	0.958789	0.948819	0.968975	1553.0
SR	0.910379	0.931562	0.890221	907.0
macro avg	0.905773	0.907965	0.903999	4026.0
micro avg	0.915916	0.915916	0.915916	4026.0
weighted avg	0.915748	0.915979	0.915916	4026.0

The result of Model Number:Dataset Number:Featuregroup Number:Rescale of 50 : 1 : 1 : 0

	f1-score	precision	recall	support
AFIB	0.719346	0.713844	0.725029	875.0
GSVT	0.735898	0.739922	0.731960	691.0
SB	0.779256	0.778710	0.779808	1553.0
SR	0.758558	0.762237	0.754953	907.0
macro avg	0.748264	0.748678	0.747938	4026.0
micro avg	0.754091	0.754091	0.754091	4026.0
weighted avg	0.754131	0.754244	0.754091	4026.0

The result of Model Number:Dataset Number:Featuregroup Number:Rescale of 50 : 1 : 1 : 1

	f1-score	precision	recall	support
AFIB	0.771052	0.760372	0.782067	875.0
GSVT	0.788153	0.794532	0.781881	691.0
SB	0.836615	0.834211	0.839039	1553.0
SR	0.816263	0.826887	0.805917	907.0
macro avg	0.803021	0.804001	0.802226	4026.0
micro avg	0.809384	0.809384	0.809384	4026.0
weighted avg	0.809463	0.809703	0.809384	4026.0

The result of Model Number:Dataset Number:Featuregroup Number:Rescale of 50 : 1 : 2 : 0

	f1-score	precision	recall	support
AFIB	0.752133	0.753859	0.750497	875.0
GSVT	0.754755	0.752627	0.756920	691.0
SB	0.792104	0.785544	0.798784	1553.0
SR	0.768341	0.779871	0.757178	907.0
macro avg	0.766833	0.767975	0.765845	4026.0
micro avg	0.771731	0.771731	0.771731	4026.0
weighted avg	0.771653	0.771730	0.771731	4026.0

The result of Model Number:Dataset Number:Featuregroup Number:Rescale of 50 : 1 : 2 : 1

	f1-score	precision	recall	support
AFIB	0.744663	0.755953	0.733724	204.0
GSVT	0.756637	0.750258	0.763164	300.0
SB	0.773420	0.770925	0.775933	892.0
SR	0.765118	0.767960	0.762318	681.0
macro avg	0.759959	0.761274	0.758785	2077.0
micro avg	0.765479	0.765479	0.765479	2077.0
weighted avg	0.765449	0.765497	0.765479	2077.0

The result of Model Number:Dataset Number:Featuregroup Number:Rescale of 50 : 1 : 3 : 0

	f1-score	precision	recall	support
AFIB	0.809862	0.820680	0.799554	204.0
GSVT	0.824169	0.819148	0.829378	300.0
SB	0.844036	0.843287	0.844791	892.0
SR	0.837799	0.837926	0.837677	681.0
macro avg	0.828966	0.830260	0.827850	2077.0
micro avg	0.835789	0.835789	0.835789	2077.0
weighted avg	0.835765	0.835822	0.835789	2077.0

The result of Model Number:Dataset Number:Featuregroup Number:Rescale of 50 : 1 : 3 : 1

	f1-score	precision	recall	support
AFIB	0.826459	0.828056	0.824930	875.0

GSVT	0.800699	0.797182	0.804296	691.0
SB	0.836635	0.785785	0.894546	1553.0
SR	0.673374	0.780245	0.592413	907.0
macro avg	0.784292	0.797817	0.779046	4026.0
micro avg	0.795860	0.795860	0.795860	4026.0
weighted avg	0.791475	0.795680	0.795860	4026.0

The result of Model Number:Dataset Number:Featuregroup Number:Rescale of 50 : 1 : 4 : 0

	f1-score	precision	recall	support
AFIB	0.703369	0.705431	0.701373	875.0
GSVT	0.715007	0.705824	0.724525	691.0
SB	0.756716	0.754877	0.758568	1553.0
SR	0.737555	0.746308	0.729013	907.0
macro avg	0.728162	0.728110	0.728369	4026.0
micro avg	0.733636	0.733636	0.733636	4026.0
weighted avg	0.733646	0.733781	0.733636	4026.0

The result of Model Number:Dataset Number:Featuregroup Number:Rescale of 50 : 1 : 4 : 1

	f1-score	precision	recall	support
AFIB	0.908721	0.913357	0.904162	875.0
GSVT	0.921262	0.916469	0.926186	691.0
SB	0.965626	0.964764	0.966498	1553.0
SR	0.947846	0.948619	0.947113	907.0
macro avg	0.935864	0.935802	0.935990	4026.0
micro avg	0.941664	0.941664	0.941664	4026.0
weighted avg	0.941639	0.941665	0.941664	4026.0

The result of Model Number:Dataset Number:Featuregroup Number:Rescale of 50 : 1 : 5 : 0

	f1-score	precision	recall	support
AFIB	0.790952	0.856980	0.735947	204.0
GSVT	0.848387	0.837719	0.859676	300.0
SB	0.870045	0.857856	0.882687	892.0
SR	0.846312	0.850277	0.842797	681.0
macro avg	0.838924	0.850708	0.830277	2077.0
micro avg	0.851872	0.851872	0.851872	2077.0
weighted avg	0.851367	0.852377	0.851872	2077.0

The result of Model Number:Dataset Number:Featuregroup Number:Rescale of 50 : 1 : 5 : 1

	f1-score	precision	recall	support
AFIB	0.662658	0.654842	0.670690	875.0
GSVT	0.675680	0.681556	0.669909	691.0
SB	0.720108	0.717352	0.722888	1553.0
SR	0.703117	0.711618	0.694825	907.0
macro avg	0.690391	0.691342	0.689578	4026.0

micro avg	0.696128	0.696128	0.696128	4026.0
weighted avg	0.696169	0.696331	0.696128	4026.0

The result of Model Number:Dataset Number:Featuregroup Number:Rescale of 50 : 1 : 6 : 0

	f1-score	precision	recall	support
AFIB	0.740479	0.741964	0.739033	875.0
GSVT	0.742313	0.738306	0.746384	691.0
SB	0.775967	0.771821	0.780162	1553.0
SR	0.757403	0.766385	0.748650	907.0
macro avg	0.754040	0.754619	0.753557	4026.0
micro avg	0.758327	0.758327	0.758327	4026.0
weighted avg	0.758296	0.758355	0.758327	4026.0

The result of Model Number:Dataset Number:Featuregroup Number:Rescale of 50 : 1 : 6 : 1

	f1-score	precision	recall	support
AFIB	0.913605	0.926886	0.901114	204.0
GSVT	0.933754	0.921121	0.946813	300.0
SB	0.947808	0.937930	0.957903	892.0
SR	0.929022	0.944395	0.914157	681.0
macro avg	0.931047	0.932583	0.929997	2077.0
micro avg	0.936380	0.936380	0.936380	2077.0
weighted avg	0.936259	0.936537	0.936380	2077.0

The result of Model Number:Dataset Number:Featuregroup Number:Rescale of 50 : 1 : 7 : 0

	f1-score	precision	recall	support
AFIB	0.644219	0.630380	0.658714	875.0
GSVT	0.667157	0.676832	0.657798	691.0
SB	0.710214	0.710079	0.710352	1553.0
SR	0.692414	0.700462	0.684553	907.0
macro avg	0.678501	0.679438	0.677854	4026.0
micro avg	0.684297	0.684297	0.684297	4026.0
weighted avg	0.684471	0.684885	0.684297	4026.0

The result of Model Number:Dataset Number:Featuregroup Number:Rescale of 50 : 1 : 7 : 1

	f1-score	precision	recall	support
AFIB	0.882909	0.882678	0.883225	875.0
GSVT	0.901353	0.897945	0.904884	691.0
SB	0.959626	0.955293	0.964013	1553.0
SR	0.927285	0.937879	0.916963	907.0
macro avg	0.917793	0.918449	0.917271	4026.0
micro avg	0.925706	0.925706	0.925706	4026.0
weighted avg	0.925665	0.925745	0.925706	4026.0

The result of Model Number:Dataset Number:Featuregroup Number:Rescale of 50 : 1 : 8 : 0

	f1-score	precision	recall	support
AFIB	0.657975	0.659725	0.656274	875.0
GSVT	0.670628	0.665903	0.675459	691.0
SB	0.708933	0.701704	0.716325	1553.0
SR	0.676526	0.691262	0.662430	907.0
macro avg	0.678515	0.679649	0.677622	4026.0
micro avg	0.684118	0.684118	0.684118	4026.0
weighted avg	0.683982	0.684083	0.684118	4026.0

The result of Model Number:Dataset Number:Featuregroup Number:Rescale of 50 : 1 : 8 : 1

	f1-score	precision	recall	support
AFIB	0.760922	0.765897	0.756057	204.0
GSVT	0.779986	0.778190	0.781801	300.0
SB	0.793909	0.792763	0.795064	892.0
SR	0.787652	0.788475	0.786845	681.0
macro avg	0.780617	0.781331	0.779942	2077.0
micro avg	0.786622	0.786622	0.786622	2077.0
weighted avg	0.786607	0.786614	0.786622	2077.0

The result of Model Number:Dataset Number:Featuregroup Number:Rescale of 50 : 1 : 9 : 0

	f1-score	precision	recall	support
AFIB	0.772498	0.825244	0.726420	204.0
GSVT	0.816608	0.799967	0.834027	300.0
SB	0.836862	0.829526	0.844361	892.0
SR	0.816220	0.818938	0.813592	681.0
macro avg	0.810547	0.818419	0.804600	2077.0
micro avg	0.821196	0.821196	0.821196	2077.0
weighted avg	0.820847	0.821364	0.821196	2077.0

The result of Model Number:Dataset Number:Featuregroup Number:Rescale of 50 : 1 : 9 : 1

	f1-score	precision	recall	support
AFIB	0.913407	0.954653	0.875876	204.0
GSVT	0.945176	0.934561	0.956207	300.0
SB	0.909224	0.869584	0.972883	892.0
SR	0.757509	0.763968	0.751287	681.0
macro avg	0.881329	0.880691	0.889063	2077.0
micro avg	0.888290	0.888290	0.888290	2077.0
weighted avg	0.865084	0.852695	0.888290	2077.0

The result of Model Number:Dataset Number:Featuregroup Number:Rescale of 50 : 1 : 10 : 0

	f1-score	precision	recall	support
AFIB	0.678075	0.687132	0.669444	204.0
GSVT	0.690054	0.685850	0.694415	300.0
SB	0.706688	0.706061	0.707320	892.0

SR 0.701466 0.701572 0.701364 681.0
macro avg 0.694071 0.695154 0.693136 2077.0
micro avg 0.699783 0.699783 0.699783 2077.0
weighted avg 0.699763 0.699811 0.699783 2077.0
The result of Model Number:Dataset Number:Featuregroup Number:Rescale of 50 : 1 : 10 : 1

	f1-score	precision	recall	support
AFIB	0.691454	0.710143	0.673867	204.0
GSVT	0.697153	0.686890	0.707823	300.0
SB	0.710657	0.703097	0.718384	892.0
SR	0.697477	0.707060	0.688161	681.0
macro avg	0.699185	0.701798	0.697059	2077.0
micro avg	0.702577	0.702577	0.702577	2077.0
weighted avg	0.702499	0.702748	0.702577	2077.0

The result of Model Number:Dataset Number:Featuregroup Number:Rescale of 51 : 0 : 0 : 0

	f1-score	precision	recall	support
AFIB	0.873310	0.870482	0.876183	875.0
GSVT	0.883437	0.886182	0.880796	691.0
SB	0.932169	0.930140	0.934210	1553.0
SR	0.914979	0.919295	0.910737	907.0
macro avg	0.900974	0.901525	0.900481	4026.0
micro avg	0.907143	0.907143	0.907143	4026.0
weighted avg	0.907140	0.907186	0.907143	4026.0

The result of Model Number:Dataset Number:Featuregroup Number:Rescale of 51 : 0 : 0 : 1

	f1-score	precision	recall	support
AFIB	0.851333	0.856006	0.846766	875.0
GSVT	0.852524	0.841746	0.863715	691.0
SB	0.896576	0.892979	0.900211	1553.0
SR	0.877330	0.887980	0.866986	907.0
macro avg	0.869441	0.869678	0.869420	4026.0
micro avg	0.874847	0.874847	0.874847	4026.0
weighted avg	0.874846	0.875024	0.874847	4026.0

The result of Model Number:Dataset Number:Featuregroup Number:Rescale of 51 : 0 : 1 : 0

	f1-score	precision	recall	support
AFIB	0.817303	0.834479	0.800851	875.0
GSVT	0.870332	0.873322	0.867405	691.0
SB	0.948239	0.943770	0.952755	1553.0
SR	0.897614	0.885660	0.909971	907.0
macro avg	0.883372	0.884308	0.882745	4026.0
micro avg	0.895453	0.895453	0.895453	4026.0
weighted avg	0.895005	0.894834	0.895453	4026.0

The result of Model Number:Dataset Number:Featuregroup Number:Rescale of 51 : 0 : 1 : 1

	f1-score	precision	recall	support
AFIB	0.673277	0.685629	0.661415	204.0
GSVT	0.682157	0.675273	0.689197	300.0
SB	0.698960	0.695322	0.702641	892.0
SR	0.689445	0.693765	0.685193	681.0
macro avg	0.685960	0.687497	0.684612	2077.0
micro avg	0.690929	0.690929	0.690929	2077.0
weighted avg	0.690891	0.690964	0.690929	2077.0

The result of Model Number:Dataset Number:Featuregroup Number:Rescale of 51 : 0 : 2 : 0

	f1-score	precision	recall	support
AFIB	0.899701	0.936015	0.866171	204.0
GSVT	0.933182	0.918023	0.949011	300.0
SB	0.957324	0.949627	0.965158	892.0
SR	0.931568	0.938276	0.925004	681.0
macro avg	0.930444	0.935485	0.926336	2077.0
micro avg	0.939938	0.939938	0.939938	2077.0
weighted avg	0.939732	0.940004	0.939938	2077.0

The result of Model Number:Dataset Number:Featuregroup Number:Rescale of 51 : 0 : 2 : 1

	f1-score	precision	recall	support
AFIB	0.741531	0.797275	0.693846	204.0
GSVT	0.707176	0.729623	0.686177	300.0
SB	0.736777	0.700319	0.777246	892.0
SR	0.652213	0.680247	0.626465	681.0
macro avg	0.709424	0.726866	0.695933	2077.0
micro avg	0.706463	0.706463	0.706463	2077.0
weighted avg	0.705242	0.707493	0.706463	2077.0

The result of Model Number:Dataset Number:Featuregroup Number:Rescale of 51 : 0 : 3 : 0

	f1-score	precision	recall	support
AFIB	0.654224	0.660111	0.648647	875.0
GSVT	0.667551	0.654015	0.681816	691.0
SB	0.707942	0.700066	0.716082	1553.0
SR	0.679850	0.700059	0.660935	907.0
macro avg	0.677392	0.678563	0.676870	4026.0
micro avg	0.683121	0.683121	0.683121	4026.0
weighted avg	0.683006	0.683477	0.683121	4026.0

The result of Model Number:Dataset Number:Featuregroup Number:Rescale of 51 : 0 : 3 : 1

	f1-score	precision	recall	support
AFIB	0.738342	0.742001	0.734797	875.0

GSVT 0.745693 0.746767 0.744735 691.0
SB 0.784942 0.781301 0.788627 1553.0
SR 0.770493 0.772422 0.768615 907.0
macro avg 0.759867 0.760623 0.759194 4026.0
micro avg 0.764886 0.764886 0.764886 4026.0
weighted avg 0.764822 0.764832 0.764886 4026.0
The result of Model Number:Dataset Number:Featuregroup Number:Rescale of 51 : 0 : 4 : 0
f1-score precision recall support
AFIB 0.887658 0.898038 0.877681 204.0
GSVT 0.800672 0.770103 0.913555 300.0
SB 0.742860 0.741109 0.744626 892.0
SR 0.922381 0.924154 0.920622 681.0
macro avg 0.838393 0.833351 0.864121 2077.0
micro avg 0.839799 0.839799 0.839799 2077.0
weighted avg 0.824293 0.820726 0.839799 2077.0
The result of Model Number:Dataset Number:Featuregroup Number:Rescale of 51 : 0 : 4 : 1
f1-score precision recall support
AFIB 0.760251 0.758777 0.761829 875.0
GSVT 0.774527 0.760975 0.788644 691.0
SB 0.829737 0.817965 0.841862 1553.0
SR 0.785980 0.821018 0.753844 907.0
macro avg 0.787624 0.789684 0.786545 4026.0
micro avg 0.795505 0.795505 0.795505 4026.0
weighted avg 0.795301 0.796007 0.795505 4026.0
The result of Model Number:Dataset Number:Featuregroup Number:Rescale of 51 : 0 : 5 : 0
f1-score precision recall support
AFIB 0.628649 0.640713 0.617066 875.0
GSVT 0.670120 0.671142 0.669142 691.0
SB 0.729674 0.726468 0.732915 1553.0
SR 0.689520 0.682024 0.697253 907.0
macro avg 0.679491 0.680087 0.679094 4026.0
micro avg 0.688757 0.688757 0.688757 4026.0
weighted avg 0.688450 0.688322 0.688757 4026.0
The result of Model Number:Dataset Number:Featuregroup Number:Rescale of 51 : 0 : 5 : 1
f1-score precision recall support
AFIB 0.852352 0.860218 0.844675 875.0
GSVT 0.872255 0.869900 0.874653 691.0
SB 0.928964 0.920232 0.937872 1553.0
SR 0.903297 0.912361 0.894430 907.0
macro avg 0.889217 0.890678 0.887908 4026.0

micro avg	0.896979	0.896979	0.896979	4026.0
weighted avg	0.896798	0.896777	0.896979	4026.0

The result of Model Number:Dataset Number:Featuregroup Number:Rescale of 51 : 0 : 6 : 0

	f1-score	precision	recall	support
AFIB	0.683773	0.687261	0.680342	875.0
GSVT	0.693209	0.689602	0.696914	691.0
SB	0.726591	0.725942	0.727247	1553.0
SR	0.713212	0.713794	0.712661	907.0
macro avg	0.704196	0.704150	0.704291	4026.0
micro avg	0.708560	0.708560	0.708560	4026.0
weighted avg	0.708541	0.708561	0.708560	4026.0

The result of Model Number:Dataset Number:Featuregroup Number:Rescale of 51 : 0 : 6 : 1

	f1-score	precision	recall	support
AFIB	0.795495	0.780624	0.811028	875.0
GSVT	0.823360	0.836358	0.810862	691.0
SB	0.875608	0.874043	0.877186	1553.0
SR	0.853352	0.862392	0.844504	907.0
macro avg	0.836954	0.838355	0.835895	4026.0
micro avg	0.844061	0.844061	0.844061	4026.0
weighted avg	0.844215	0.844647	0.844061	4026.0

The result of Model Number:Dataset Number:Featuregroup Number:Rescale of 51 : 0 : 7 : 0

	f1-score	precision	recall	support
AFIB	0.837239	0.826257	0.848555	875.0
GSVT	0.850057	0.859505	0.840828	691.0
SB	0.901595	0.899467	0.903738	1553.0
SR	0.879021	0.886933	0.871263	907.0
macro avg	0.866978	0.868040	0.866096	4026.0
micro avg	0.873631	0.873631	0.873631	4026.0
weighted avg	0.873677	0.873873	0.873631	4026.0

The result of Model Number:Dataset Number:Featuregroup Number:Rescale of 51 : 0 : 7 : 1

	f1-score	precision	recall	support
AFIB	0.899679	0.900896	0.898637	875.0
GSVT	0.913692	0.913222	0.914196	691.0
SB	0.954257	0.950842	0.957699	1553.0
SR	0.931405	0.936626	0.926302	907.0
macro avg	0.924758	0.925396	0.924208	4026.0
micro avg	0.930323	0.930323	0.930323	4026.0
weighted avg	0.930284	0.930327	0.930323	4026.0

The result of Model Number:Dataset Number:Featuregroup Number:Rescale of 51 : 0 : 8 : 0

	f1-score	precision	recall	support
AFIB	0.853836	0.868031	0.840294	204.0
GSVT	0.870209	0.864026	0.876545	300.0
SB	0.890393	0.885389	0.895474	892.0
SR	0.879049	0.884427	0.873764	681.0
macro avg	0.873372	0.875468	0.871519	2077.0
micro avg	0.880202	0.880202	0.880202	2077.0
weighted avg	0.880168	0.880283	0.880202	2077.0

The result of Model Number:Dataset Number:Featuregroup Number:Rescale of 51 : 0 : 8 : 1

	f1-score	precision	recall	support
AFIB	0.687837	0.691445	0.684308	875.0
GSVT	0.702220	0.700958	0.703536	691.0
SB	0.762490	0.756643	0.768431	1553.0
SR	0.735589	0.742952	0.728375	907.0
macro avg	0.722034	0.723000	0.721162	4026.0
micro avg	0.729986	0.729986	0.729986	4026.0
weighted avg	0.729860	0.729831	0.729986	4026.0

The result of Model Number:Dataset Number:Featuregroup Number:Rescale of 51 : 0 : 9 : 0

	f1-score	precision	recall	support
AFIB	0.801338	0.780287	0.851504	875.0
GSVT	0.770880	0.729647	0.878787	691.0
SB	0.730448	0.725832	0.735146	1553.0
SR	0.709515	0.721740	0.697895	907.0
macro avg	0.753045	0.739377	0.790833	4026.0
micro avg	0.776696	0.776696	0.776696	4026.0
weighted avg	0.748079	0.737400	0.776696	4026.0

The result of Model Number:Dataset Number:Featuregroup Number:Rescale of 51 : 0 : 9 : 1

	f1-score	precision	recall	support
AFIB	0.790098	0.797208	0.783363	875.0
GSVT	0.806193	0.789846	0.823421	691.0
SB	0.854973	0.845461	0.864803	1553.0
SR	0.821046	0.845452	0.798203	907.0
macro avg	0.818078	0.819492	0.817447	4026.0
micro avg	0.824997	0.824997	0.824997	4026.0
weighted avg	0.824858	0.825427	0.824997	4026.0

The result of Model Number:Dataset Number:Featuregroup Number:Rescale of 51 : 0 : 10 : 0

	f1-score	precision	recall	support
AFIB	0.706836	0.688267	0.751086	875.0
GSVT	0.679970	0.643600	0.775151	691.0
SB	0.644306	0.640235	0.648450	1553.0

SR 0.625842 0.636625 0.615592 907.0
macro avg 0.664238 0.652182 0.697570 4026.0
micro avg 0.685100 0.685100 0.685100 4026.0
weighted avg 0.659858 0.650438 0.685100 4026.0
The result of Model Number:Dataset Number:Featuregroup Number:Rescale of 51 : 0 : 10 : 1

	f1-score	precision	recall	support
AFIB	0.722472	0.726157	0.718847	875.0
GSVT	0.732442	0.728631	0.736356	691.0
SB	0.767714	0.767028	0.768406	1553.0
SR	0.753577	0.754192	0.752995	907.0
macro avg	0.744051	0.744002	0.744151	4026.0
micro avg	0.748662	0.748662	0.748662	4026.0
weighted avg	0.748642	0.748663	0.748662	4026.0

The result of Model Number:Dataset Number:Featuregroup Number:Rescale of 51 : 1 : 0 : 0

	f1-score	precision	recall	support
AFIB	0.702392	0.732495	0.674932	204.0
GSVT	0.725995	0.715026	0.737352	300.0
SB	0.740395	0.733072	0.747866	892.0
SR	0.720335	0.726498	0.714303	681.0
macro avg	0.722279	0.726773	0.718613	2077.0
micro avg	0.728180	0.728180	0.728180	2077.0
weighted avg	0.728005	0.728253	0.728180	2077.0

The result of Model Number:Dataset Number:Featuregroup Number:Rescale of 51 : 1 : 0 : 1

	f1-score	precision	recall	support
AFIB	0.785854	0.773529	0.798664	875.0
GSVT	0.757222	0.778931	0.736779	691.0
SB	0.795715	0.755380	0.840647	1553.0
SR	0.646514	0.719438	0.587113	907.0
macro avg	0.746326	0.756819	0.740801	4026.0
micro avg	0.756578	0.756578	0.756578	4026.0
weighted avg	0.753352	0.755269	0.756578	4026.0

The result of Model Number:Dataset Number:Featuregroup Number:Rescale of 51 : 1 : 1 : 0

	f1-score	precision	recall	support
AFIB	0.871619	0.890855	0.853261	204.0
GSVT	0.890668	0.875898	0.906051	300.0
SB	0.907782	0.900682	0.915005	892.0
SR	0.885752	0.896412	0.875343	681.0
macro avg	0.888955	0.890962	0.887415	2077.0
micro avg	0.894643	0.894643	0.894643	2077.0
weighted avg	0.894535	0.894737	0.894643	2077.0

The result of Model Number:Dataset Number:Featuregroup Number:Rescale of 51 : 1 : 1 : 1

	f1-score	precision	recall	support
AFIB	0.842803	0.851042	0.834919	875.0
GSVT	0.836366	0.830020	0.843084	691.0
SB	0.884970	0.875865	0.894345	1553.0
SR	0.856880	0.870660	0.843770	907.0
macro avg	0.855255	0.856897	0.854030	4026.0
micro avg	0.861238	0.861238	0.861238	4026.0
weighted avg	0.861135	0.861429	0.861238	4026.0

The result of Model Number:Dataset Number:Featuregroup Number:Rescale of 51 : 1 : 2 : 0

	f1-score	precision	recall	support
AFIB	0.740615	0.782747	0.703279	204.0
GSVT	0.782663	0.774044	0.791515	300.0
SB	0.794306	0.786402	0.802397	892.0
SR	0.785078	0.787762	0.782536	681.0
macro avg	0.775665	0.782739	0.769932	2077.0
micro avg	0.784578	0.784578	0.784578	2077.0
weighted avg	0.784325	0.784704	0.784578	2077.0

The result of Model Number:Dataset Number:Featuregroup Number:Rescale of 51 : 1 : 2 : 1

	f1-score	precision	recall	support
AFIB	0.886318	0.884079	0.888706	875.0
GSVT	0.903609	0.898641	0.908681	691.0
SB	0.946150	0.940446	0.951937	1553.0
SR	0.918254	0.934827	0.902275	907.0
macro avg	0.913583	0.914498	0.912900	4026.0
micro avg	0.919582	0.919582	0.919582	4026.0
weighted avg	0.919560	0.919754	0.919582	4026.0

The result of Model Number:Dataset Number:Featuregroup Number:Rescale of 51 : 1 : 3 : 0

	f1-score	precision	recall	support
AFIB	0.809897	0.817372	0.802603	875.0
GSVT	0.828809	0.826571	0.831087	691.0
SB	0.882693	0.874396	0.891158	1553.0
SR	0.858305	0.866917	0.849879	907.0
macro avg	0.844926	0.846314	0.843682	4026.0
micro avg	0.852302	0.852302	0.852302	4026.0
weighted avg	0.852129	0.852109	0.852302	4026.0

The result of Model Number:Dataset Number:Featuregroup Number:Rescale of 51 : 1 : 3 : 1

	f1-score	precision	recall	support
AFIB	0.701454	0.714916	0.688530	875.0

GSVT 0.747728 0.748868 0.746637 691.0
SB 0.814180 0.810601 0.817795 1553.0
SR 0.769374 0.761011 0.778003 907.0
macro avg 0.758184 0.758849 0.757741 4026.0
micro avg 0.768523 0.768523 0.768523 4026.0
weighted avg 0.768181 0.768038 0.768523 4026.0
The result of Model Number:Dataset Number:Featuregroup Number:Rescale of 51 : 1 : 4 : 0
f1-score precision recall support
AFIB 0.785494 0.795676 0.775777 204.0
GSVT 0.815887 0.815360 0.816440 300.0
SB 0.833079 0.831320 0.834851 892.0
SR 0.824700 0.824232 0.825187 681.0
macro avg 0.814790 0.816647 0.813064 2077.0
micro avg 0.823221 0.823221 0.823221 2077.0
weighted avg 0.823175 0.823190 0.823221 2077.0
The result of Model Number:Dataset Number:Featuregroup Number:Rescale of 51 : 1 : 4 : 1
f1-score precision recall support
AFIB 0.702308 0.715917 0.689522 204.0
GSVT 0.729542 0.723442 0.735880 300.0
SB 0.743154 0.741663 0.744654 892.0
SR 0.736068 0.736831 0.735311 681.0
macro avg 0.727768 0.729463 0.726342 2077.0
micro avg 0.734908 0.734908 0.734908 2077.0
weighted avg 0.734853 0.734918 0.734908 2077.0
The result of Model Number:Dataset Number:Featuregroup Number:Rescale of 51 : 1 : 5 : 0
f1-score precision recall support
AFIB 0.676605 0.742936 0.621809 204.0
GSVT 0.793837 0.783137 0.804961 300.0
SB 0.842807 0.839995 0.845641 892.0
SR 0.820903 0.809897 0.832234 681.0
macro avg 0.783538 0.793992 0.776161 2077.0
micro avg 0.813385 0.813385 0.813385 2077.0
weighted avg 0.812228 0.812381 0.813385 2077.0
The result of Model Number:Dataset Number:Featuregroup Number:Rescale of 51 : 1 : 5 : 1
f1-score precision recall support
AFIB 0.893448 0.894245 0.892744 875.0
GSVT 0.909814 0.912201 0.907592 691.0
SB 0.965780 0.959349 0.972306 1553.0
SR 0.946962 0.955608 0.938506 907.0
macro avg 0.929001 0.930351 0.927787 4026.0

micro avg 0.936293 0.936293 0.936293 4026.0
weighted avg 0.936214 0.936265 0.936293 4026.0
The result of Model Number:Dataset Number:Featuregroup Number:Rescale of 51 : 1 : 6 : 0

	f1-score	precision	recall	support
AFIB	0.823955	0.832737	0.815490	204.0
GSVT	0.839108	0.834505	0.843855	300.0
SB	0.858477	0.857046	0.859918	892.0
SR	0.851321	0.852707	0.849949	681.0
macro avg	0.843215	0.844249	0.842303	2077.0
micro avg	0.849966	0.849966	0.849966	2077.0
weighted avg	0.849942	0.849980	0.849966	2077.0

The result of Model Number:Dataset Number:Featuregroup Number:Rescale of 51 : 1 : 6 : 1

	f1-score	precision	recall	support
AFIB	0.708537	0.717722	0.699773	204.0
GSVT	0.735953	0.735477	0.736451	300.0
SB	0.751460	0.749873	0.753059	892.0
SR	0.743902	0.743480	0.744341	681.0
macro avg	0.734963	0.736638	0.733406	2077.0
micro avg	0.742568	0.742568	0.742568	2077.0
weighted avg	0.742526	0.742540	0.742568	2077.0

The result of Model Number:Dataset Number:Featuregroup Number:Rescale of 51 : 1 : 7 : 0

	f1-score	precision	recall	support
AFIB	0.840891	0.870083	0.813707	204.0
GSVT	0.857014	0.847632	0.866711	300.0
SB	0.879829	0.873511	0.886240	892.0
SR	0.864116	0.868365	0.859923	681.0
macro avg	0.860462	0.864898	0.856646	2077.0
micro avg	0.867667	0.867667	0.867667	2077.0
weighted avg	0.867557	0.867749	0.867667	2077.0

The result of Model Number:Dataset Number:Featuregroup Number:Rescale of 51 : 1 : 7 : 1

	f1-score	precision	recall	support
AFIB	0.831508	0.828950	0.834134	875.0
GSVT	0.810311	0.814285	0.806505	691.0
SB	0.851864	0.816514	0.890423	1553.0
SR	0.733100	0.799853	0.676688	907.0
macro avg	0.806696	0.814901	0.801937	4026.0
micro avg	0.815635	0.815635	0.815635	4026.0
weighted avg	0.813552	0.815081	0.815635	4026.0

The result of Model Number:Dataset Number:Featuregroup Number:Rescale of 51 : 1 : 8 : 0

	f1-score	precision	recall	support
AFIB	0.743649	0.736677	0.750849	875.0
GSVT	0.705051	0.688104	0.756880	691.0
SB	0.815260	0.812265	0.818298	1553.0
SR	0.634094	0.638541	0.629717	907.0
macro avg	0.724513	0.718897	0.738936	4026.0
micro avg	0.750613	0.750613	0.750613	4026.0
weighted avg	0.739966	0.735389	0.750613	4026.0

The result of Model Number:Dataset Number:Featuregroup Number:Rescale of 51 : 1 : 8 : 1

	f1-score	precision	recall	support
AFIB	0.743065	0.759594	0.727400	204.0
GSVT	0.774724	0.769910	0.779629	300.0
SB	0.791541	0.789874	0.793225	892.0
SR	0.783111	0.782557	0.783690	681.0
macro avg	0.773110	0.775484	0.770986	2077.0
micro avg	0.781670	0.781670	0.781670	2077.0
weighted avg	0.781587	0.781617	0.781670	2077.0

The result of Model Number:Dataset Number:Featuregroup Number:Rescale of 51 : 1 : 9 : 0

	f1-score	precision	recall	support
AFIB	0.694594	0.707359	0.682367	204.0
GSVT	0.706262	0.701636	0.710967	300.0
SB	0.718343	0.714835	0.721886	892.0
SR	0.710918	0.713874	0.707997	681.0
macro avg	0.707529	0.709426	0.705804	2077.0
micro avg	0.711873	0.711873	0.711873	2077.0
weighted avg	0.711831	0.711879	0.711873	2077.0

The result of Model Number:Dataset Number:Featuregroup Number:Rescale of 51 : 1 : 9 : 1

	f1-score	precision	recall	support
AFIB	0.901414	0.917958	0.885891	204.0
GSVT	0.935633	0.928110	0.943450	300.0
SB	0.952990	0.951288	0.954699	892.0
SR	0.943554	0.944394	0.942720	681.0
macro avg	0.933398	0.935438	0.931690	2077.0
micro avg	0.942389	0.942389	0.942389	2077.0
weighted avg	0.942323	0.942406	0.942389	2077.0

The result of Model Number:Dataset Number:Featuregroup Number:Rescale of 51 : 1 : 10 : 0

	f1-score	precision	recall	support
AFIB	0.642446	0.626006	0.659833	875.0
GSVT	0.607310	0.641731	0.576535	691.0
SB	0.643961	0.592599	0.705088	1553.0

SR 0.514416 0.624826 0.437260 907.0
macro avg 0.602033 0.621291 0.594679 4026.0
micro avg 0.612850 0.612850 0.612850 4026.0
weighted avg 0.608157 0.615553 0.612850 4026.0
The result of Model Number:Dataset Number:Featuregroup Number:Rescale of 51 : 1 : 10 : 1

	f1-score	precision	recall	support
AFIB	0.896213	0.903097	0.889636	204.0
GSVT	0.927773	0.926884	0.928694	300.0
SB	0.946754	0.944549	0.948979	892.0
SR	0.938041	0.939306	0.936801	681.0
macro avg	0.927195	0.928459	0.926028	2077.0
micro avg	0.936228	0.936228	0.936228	2077.0
weighted avg	0.936192	0.936207	0.936228	2077.0

The result of Model Number:Dataset Number:Featuregroup Number:Rescale of 52 : 0 : 0 : 0

	f1-score	precision	recall	support
AFIB	0.771169	0.800535	0.744050	204.0
GSVT	0.795739	0.787718	0.803959	300.0
SB	0.811666	0.807625	0.815759	892.0
SR	0.801862	0.802366	0.801384	681.0
macro avg	0.795109	0.799561	0.791288	2077.0
micro avg	0.802298	0.802298	0.802298	2077.0
weighted avg	0.802173	0.802329	0.802298	2077.0

The result of Model Number:Dataset Number:Featuregroup Number:Rescale of 52 : 0 : 0 : 1

	f1-score	precision	recall	support
AFIB	0.756818	0.752053	0.852504	204.0
GSVT	0.922574	0.917829	0.927524	300.0
SB	0.948843	0.944328	0.953411	892.0
SR	0.744260	0.746712	0.742273	681.0
macro avg	0.843124	0.840230	0.868928	2077.0
micro avg	0.870534	0.870534	0.870534	2077.0
weighted avg	0.859110	0.856822	0.870534	2077.0

The result of Model Number:Dataset Number:Featuregroup Number:Rescale of 52 : 0 : 1 : 0

	f1-score	precision	recall	support
AFIB	0.649307	0.634552	0.664810	875.0
GSVT	0.671559	0.682450	0.661066	691.0
SB	0.716480	0.716485	0.716481	1553.0
SR	0.698143	0.706096	0.690368	907.0
macro avg	0.683872	0.684896	0.683181	4026.0
micro avg	0.689857	0.689857	0.689857	4026.0
weighted avg	0.690040	0.690496	0.689857	4026.0

The result of Model Number:Dataset Number:Featuregroup Number:Rescale of 52 : 0 : 1 : 1

	f1-score	precision	recall	support
AFIB	0.803475	0.822709	0.786297	204.0
GSVT	0.827136	0.815481	0.839474	300.0
SB	0.848627	0.840005	0.857466	892.0
SR	0.828344	0.840304	0.816785	681.0
macro avg	0.826895	0.829625	0.825005	2077.0
micro avg	0.834539	0.834539	0.834539	2077.0
weighted avg	0.834438	0.834862	0.834539	2077.0

The result of Model Number:Dataset Number:Featuregroup Number:Rescale of 52 : 0 : 2 : 0

	f1-score	precision	recall	support
AFIB	0.785372	0.802867	0.768848	204.0
GSVT	0.795061	0.783417	0.807388	300.0
SB	0.813027	0.804948	0.821307	892.0
SR	0.798030	0.809492	0.786912	681.0
macro avg	0.797873	0.800181	0.796114	2077.0
micro avg	0.802867	0.802867	0.802867	2077.0
weighted avg	0.802799	0.803123	0.802867	2077.0

The result of Model Number:Dataset Number:Featuregroup Number:Rescale of 52 : 0 : 2 : 1

	f1-score	precision	recall	support
AFIB	0.865300	0.868457	0.862222	875.0
GSVT	0.880255	0.868793	0.892099	691.0
SB	0.927878	0.917658	0.938334	1553.0
SR	0.879362	0.903654	0.856390	907.0
macro avg	0.888199	0.889641	0.887261	4026.0
micro avg	0.895396	0.895396	0.895396	4026.0
weighted avg	0.895174	0.895423	0.895396	4026.0

The result of Model Number:Dataset Number:Featuregroup Number:Rescale of 52 : 0 : 3 : 0

	f1-score	precision	recall	support
AFIB	0.890207	0.923212	0.859608	204.0
GSVT	0.916877	0.905412	0.928752	300.0
SB	0.940127	0.931548	0.948873	892.0
SR	0.915410	0.922514	0.908424	681.0
macro avg	0.915655	0.920672	0.911414	2077.0
micro avg	0.923937	0.923937	0.923937	2077.0
weighted avg	0.923762	0.923993	0.923937	2077.0

The result of Model Number:Dataset Number:Featuregroup Number:Rescale of 52 : 0 : 3 : 1

	f1-score	precision	recall	support
AFIB	0.819184	0.831219	0.807568	204.0

GSVT 0.839940 0.832799 0.847221 300.0
SB 0.854456 0.851038 0.857904 892.0
SR 0.846006 0.850158 0.841903 681.0
macro avg 0.839897 0.841304 0.838649 2077.0
micro avg 0.846171 0.846171 0.846171 2077.0
weighted avg 0.846124 0.846168 0.846171 2077.0
The result of Model Number:Dataset Number:Featuregroup Number:Rescale of 52 : 0 : 4 : 0

	f1-score	precision	recall	support
AFIB	0.868520	0.891258	0.847310	204.0
GSVT	0.817218	0.840969	0.795216	300.0
SB	0.862339	0.831978	0.895016	892.0
SR	0.781605	0.807060	0.757865	681.0
macro avg	0.832421	0.842816	0.823852	2077.0
micro avg	0.830947	0.830947	0.830947	2077.0
weighted avg	0.829958	0.830929	0.830947	2077.0

The result of Model Number:Dataset Number:Featuregroup Number:Rescale of 52 : 0 : 4 : 1

	f1-score	precision	recall	support
AFIB	0.780631	0.786933	0.774496	204.0
GSVT	0.798543	0.794627	0.802525	300.0
SB	0.814128	0.813225	0.815040	892.0
SR	0.808049	0.809123	0.806983	681.0
macro avg	0.800338	0.800977	0.799761	2077.0
micro avg	0.806608	0.806608	0.806608	2077.0
weighted avg	0.806594	0.806612	0.806608	2077.0

The result of Model Number:Dataset Number:Featuregroup Number:Rescale of 52 : 0 : 5 : 0

	f1-score	precision	recall	support
AFIB	0.768164	0.771971	0.764476	875.0
GSVT	0.775812	0.776930	0.774815	691.0
SB	0.816647	0.812858	0.820480	1553.0
SR	0.801614	0.803621	0.799660	907.0
macro avg	0.790559	0.791345	0.789858	4026.0
micro avg	0.795780	0.795780	0.795780	4026.0
weighted avg	0.795714	0.795724	0.795780	4026.0

The result of Model Number:Dataset Number:Featuregroup Number:Rescale of 52 : 0 : 5 : 1

	f1-score	precision	recall	support
AFIB	0.758256	0.781694	0.736843	204.0
GSVT	0.707822	0.723210	0.693598	300.0
SB	0.746573	0.713439	0.782972	892.0
SR	0.672207	0.706749	0.640979	681.0
macro avg	0.721215	0.731273	0.713598	2077.0

micro avg 0.718976 0.718976 0.718976 2077.0
weighted avg 0.717741 0.719361 0.718976 2077.0
The result of Model Number:Dataset Number:Featuregroup Number:Rescale of 52 : 0 : 6 : 0

	f1-score	precision	recall	support
AFIB	0.725991	0.730131	0.722017	204.0
GSVT	0.740928	0.735584	0.746358	300.0
SB	0.755893	0.753132	0.758687	892.0
SR	0.747853	0.752764	0.743026	681.0
macro avg	0.742666	0.742903	0.742522	2077.0
micro avg	0.748170	0.748170	0.748170	2077.0
weighted avg	0.748158	0.748217	0.748170	2077.0

The result of Model Number:Dataset Number:Featuregroup Number:Rescale of 52 : 0 : 6 : 1

	f1-score	precision	recall	support
AFIB	0.803926	0.791600	0.816776	875.0
GSVT	0.831647	0.839798	0.823816	691.0
SB	0.885094	0.883026	0.887199	1553.0
SR	0.862984	0.873926	0.852331	907.0
macro avg	0.845913	0.847088	0.845031	4026.0
micro avg	0.853160	0.853160	0.853160	4026.0
weighted avg	0.853299	0.853686	0.853160	4026.0

The result of Model Number:Dataset Number:Featuregroup Number:Rescale of 52 : 0 : 7 : 0

	f1-score	precision	recall	support
AFIB	0.646699	0.655607	0.638314	875.0
GSVT	0.660817	0.658080	0.663755	691.0
SB	0.703873	0.694644	0.713380	1553.0
SR	0.681774	0.691956	0.672191	907.0
macro avg	0.673291	0.675072	0.671910	4026.0
micro avg	0.679269	0.679269	0.679269	4026.0
weighted avg	0.679078	0.679279	0.679269	4026.0

The result of Model Number:Dataset Number:Featuregroup Number:Rescale of 52 : 0 : 7 : 1

	f1-score	precision	recall	support
AFIB	0.732363	0.768955	0.699464	204.0
GSVT	0.754442	0.740460	0.769224	300.0
SB	0.771707	0.763928	0.779662	892.0
SR	0.750305	0.757007	0.743789	681.0
macro avg	0.752204	0.757588	0.748035	2077.0
micro avg	0.758516	0.758516	0.758516	2077.0
weighted avg	0.758332	0.758763	0.758516	2077.0

The result of Model Number:Dataset Number:Featuregroup Number:Rescale of 52 : 0 : 8 : 0

	f1-score	precision	recall	support
AFIB	0.736821	0.731185	0.742642	875.0
GSVT	0.753774	0.757896	0.749741	691.0
SB	0.798186	0.797627	0.798751	1553.0
SR	0.776985	0.780754	0.773292	907.0
macro avg	0.766441	0.766865	0.766107	4026.0
micro avg	0.772409	0.772409	0.772409	4026.0
weighted avg	0.772450	0.772566	0.772409	4026.0

The result of Model Number:Dataset Number:Featuregroup Number:Rescale of 52 : 0 : 8 : 1

	f1-score	precision	recall	support
AFIB	0.885461	0.954475	0.826166	204.0
GSVT	0.912464	0.927910	0.897562	300.0
SB	0.967040	0.958140	0.976136	892.0
SR	0.928550	0.915085	0.942461	681.0
macro avg	0.923379	0.938902	0.910581	2077.0
micro avg	0.939016	0.939016	0.939016	2077.0
weighted avg	0.938525	0.939297	0.939016	2077.0

The result of Model Number:Dataset Number:Featuregroup Number:Rescale of 52 : 0 : 9 : 0

	f1-score	precision	recall	support
AFIB	0.831442	0.837757	0.825293	204.0
GSVT	0.848464	0.840177	0.856935	300.0
SB	0.868255	0.866036	0.870494	892.0
SR	0.859046	0.863898	0.854257	681.0
macro avg	0.851802	0.851967	0.851745	2077.0
micro avg	0.858772	0.858772	0.858772	2077.0
weighted avg	0.858761	0.858822	0.858772	2077.0

The result of Model Number:Dataset Number:Featuregroup Number:Rescale of 52 : 0 : 9 : 1

	f1-score	precision	recall	support
AFIB	0.740448	0.746580	0.834013	204.0
GSVT	0.802966	0.768598	0.905933	300.0
SB	0.733138	0.730590	0.735705	892.0
SR	0.725848	0.725985	0.725716	681.0
macro avg	0.750600	0.742938	0.800342	2077.0
micro avg	0.766673	0.766673	0.766673	2077.0
weighted avg	0.741551	0.736140	0.766673	2077.0

The result of Model Number:Dataset Number:Featuregroup Number:Rescale of 52 : 0 : 10 : 0

	f1-score	precision	recall	support
AFIB	0.874793	0.874740	0.874900	875.0
GSVT	0.876686	0.871946	0.881493	691.0
SB	0.918569	0.912457	0.924775	1553.0

SR 0.894178 0.908848 0.880006 907.0
macro avg 0.891056 0.891998 0.890294 4026.0
micro avg 0.896421 0.896421 0.896421 4026.0
weighted avg 0.896371 0.896494 0.896421 4026.0
The result of Model Number:Dataset Number:Featuregroup Number:Rescale of 52 : 0 : 10 : 1

	f1-score	precision	recall	support
AFIB	0.829863	0.834596	0.825321	204.0
GSVT	0.846938	0.840829	0.853145	300.0
SB	0.864043	0.860887	0.867238	892.0
SR	0.854854	0.860467	0.849335	681.0
macro avg	0.848925	0.849195	0.848760	2077.0
micro avg	0.855215	0.855215	0.855215	2077.0
weighted avg	0.855202	0.855270	0.855215	2077.0

The result of Model Number:Dataset Number:Featuregroup Number:Rescale of 52 : 1 : 0 : 0

	f1-score	precision	recall	support
AFIB	0.828764	0.832148	0.825433	875.0
GSVT	0.771739	0.805799	0.740569	691.0
SB	0.828802	0.750787	0.924922	1553.0
SR	0.625194	0.778113	0.522719	907.0
macro avg	0.763625	0.791712	0.753411	4026.0
micro avg	0.781047	0.781047	0.781047	4026.0
weighted avg	0.773130	0.784068	0.781047	4026.0

The result of Model Number:Dataset Number:Featuregroup Number:Rescale of 52 : 1 : 0 : 1

	f1-score	precision	recall	support
AFIB	0.649237	0.669306	0.630903	204.0
GSVT	0.606055	0.619230	0.593876	300.0
SB	0.639235	0.610864	0.670400	892.0
SR	0.575561	0.605136	0.548823	681.0
macro avg	0.617522	0.626134	0.611000	2077.0
micro avg	0.615605	0.615605	0.615605	2077.0
weighted avg	0.614547	0.615935	0.615605	2077.0

The result of Model Number:Dataset Number:Featuregroup Number:Rescale of 52 : 1 : 1 : 0

	f1-score	precision	recall	support
AFIB	0.799804	0.810487	0.789624	204.0
GSVT	0.813933	0.808974	0.819077	300.0
SB	0.833553	0.832814	0.834299	892.0
SR	0.827393	0.827519	0.827273	681.0
macro avg	0.818671	0.819949	0.817568	2077.0
micro avg	0.825409	0.825409	0.825409	2077.0
weighted avg	0.825385	0.825442	0.825409	2077.0

The result of Model Number:Dataset Number:Featuregroup Number:Rescale of 52 : 1 : 1 : 1

	f1-score	precision	recall	support
AFIB	0.633878	0.627771	0.640202	875.0
GSVT	0.650485	0.652346	0.648703	691.0
SB	0.691578	0.689987	0.693180	1553.0
SR	0.672124	0.680032	0.664411	907.0
macro avg	0.662016	0.662534	0.661624	4026.0
micro avg	0.667551	0.667551	0.667551	4026.0
weighted avg	0.667602	0.667762	0.667551	4026.0

The result of Model Number:Dataset Number:Featuregroup Number:Rescale of 52 : 1 : 2 : 0

	f1-score	precision	recall	support
AFIB	0.711363	0.712995	0.709815	875.0
GSVT	0.713843	0.711830	0.715890	691.0
SB	0.749167	0.742963	0.755485	1553.0
SR	0.726692	0.737598	0.716134	907.0
macro avg	0.725266	0.726346	0.724331	4026.0
micro avg	0.729898	0.729898	0.729898	4026.0
weighted avg	0.729824	0.729898	0.729898	4026.0

The result of Model Number:Dataset Number:Featuregroup Number:Rescale of 52 : 1 : 2 : 1

	f1-score	precision	recall	support
AFIB	0.779534	0.808533	0.752892	204.0
GSVT	0.723378	0.756946	0.692728	300.0
SB	0.757767	0.712062	0.809756	892.0
SR	0.665466	0.711702	0.624949	681.0
macro avg	0.731536	0.747311	0.720081	2077.0
micro avg	0.726673	0.726673	0.726673	2077.0
weighted avg	0.724675	0.727903	0.726673	2077.0

The result of Model Number:Dataset Number:Featuregroup Number:Rescale of 52 : 1 : 3 : 0

	f1-score	precision	recall	support
AFIB	0.691694	0.699380	0.684530	204.0
GSVT	0.713120	0.709060	0.717433	300.0
SB	0.727306	0.726332	0.728284	892.0
SR	0.721119	0.722085	0.720165	681.0
macro avg	0.713310	0.714214	0.712603	2077.0
micro avg	0.719758	0.719758	0.719758	2077.0
weighted avg	0.719731	0.719798	0.719758	2077.0

The result of Model Number:Dataset Number:Featuregroup Number:Rescale of 52 : 1 : 3 : 1

	f1-score	precision	recall	support
AFIB	0.878676	0.885622	0.871867	875.0

GSVT 0.900786 0.898882 0.902733 691.0
SB 0.960265 0.951777 0.968917 1553.0
SR 0.934701 0.943869 0.925733 907.0
macro avg 0.918607 0.920038 0.917313 4026.0
micro avg 0.926736 0.926736 0.926736 4026.0
weighted avg 0.926565 0.926539 0.926736 4026.0
The result of Model Number:Dataset Number:Featuregroup Number:Rescale of 52 : 1 : 4 : 0

	f1-score	precision	recall	support
AFIB	0.839727	0.833262	0.846358	875.0
GSVT	0.850630	0.854579	0.846902	691.0
SB	0.911568	0.906719	0.916489	1553.0
SR	0.884711	0.897149	0.872710	907.0
macro avg	0.871659	0.872927	0.870615	4026.0
micro avg	0.879441	0.879441	0.879441	4026.0
weighted avg	0.879445	0.879649	0.879441	4026.0

The result of Model Number:Dataset Number:Featuregroup Number:Rescale of 52 : 1 : 4 : 1

	f1-score	precision	recall	support
AFIB	0.773604	0.789898	0.758092	204.0
GSVT	0.797657	0.790369	0.805098	300.0
SB	0.810684	0.808877	0.812502	892.0
SR	0.801050	0.801887	0.800223	681.0
macro avg	0.795749	0.797758	0.793978	2077.0
micro avg	0.802062	0.802062	0.802062	2077.0
weighted avg	0.802001	0.802048	0.802062	2077.0

The result of Model Number:Dataset Number:Featuregroup Number:Rescale of 52 : 1 : 5 : 0

	f1-score	precision	recall	support
AFIB	0.746414	0.828367	0.679396	204.0
GSVT	0.871539	0.860643	0.882885	300.0
SB	0.927275	0.922543	0.932056	892.0
SR	0.905552	0.892892	0.918581	681.0
macro avg	0.862695	0.876111	0.853230	2077.0
micro avg	0.895720	0.895720	0.895720	2077.0
weighted avg	0.894338	0.894631	0.895720	2077.0

The result of Model Number:Dataset Number:Featuregroup Number:Rescale of 52 : 1 : 5 : 1

	f1-score	precision	recall	support
AFIB	0.858973	0.848842	0.869384	875.0
GSVT	0.875852	0.883470	0.868373	691.0
SB	0.933442	0.929870	0.937046	1553.0
SR	0.911418	0.922437	0.900669	907.0
macro avg	0.894922	0.896155	0.893868	4026.0

micro avg 0.902359 0.902359 0.902359 4026.0
weighted avg 0.902411 0.902621 0.902359 4026.0
The result of Model Number:Dataset Number:Featuregroup Number:Rescale of 52 : 1 : 6 : 0

	f1-score	precision	recall	support
AFIB	0.675802	0.681938	0.670123	204.0
GSVT	0.698138	0.693919	0.702592	300.0
SB	0.712744	0.711788	0.713702	892.0
SR	0.706149	0.707613	0.704696	681.0
macro avg	0.698208	0.698814	0.697778	2077.0
micro avg	0.704864	0.704864	0.704864	2077.0
weighted avg	0.704843	0.704906	0.704864	2077.0

The result of Model Number:Dataset Number:Featuregroup Number:Rescale of 52 : 1 : 6 : 1

	f1-score	precision	recall	support
AFIB	0.772687	0.791487	0.754949	204.0
GSVT	0.782400	0.771629	0.793580	300.0
SB	0.798198	0.790227	0.806337	892.0
SR	0.784262	0.794554	0.774255	681.0
macro avg	0.784386	0.786974	0.782280	2077.0
micro avg	0.788928	0.788928	0.788928	2077.0
weighted avg	0.788841	0.789083	0.788928	2077.0

The result of Model Number:Dataset Number:Featuregroup Number:Rescale of 52 : 1 : 7 : 0

	f1-score	precision	recall	support
AFIB	0.793755	0.799691	0.787938	204.0
GSVT	0.814037	0.809741	0.818380	300.0
SB	0.827775	0.826668	0.828887	892.0
SR	0.820484	0.822057	0.818919	681.0
macro avg	0.814013	0.814539	0.813531	2077.0
micro avg	0.820079	0.820079	0.820079	2077.0
weighted avg	0.820059	0.820062	0.820079	2077.0

The result of Model Number:Dataset Number:Featuregroup Number:Rescale of 52 : 1 : 7 : 1

	f1-score	precision	recall	support
AFIB	0.838788	0.882122	0.799761	204.0
GSVT	0.879541	0.870944	0.888329	300.0
SB	0.895049	0.887849	0.902372	892.0
SR	0.883994	0.885070	0.882956	681.0
macro avg	0.874343	0.881496	0.868355	2077.0
micro avg	0.883899	0.883899	0.883899	2077.0
weighted avg	0.883658	0.883933	0.883899	2077.0

The result of Model Number:Dataset Number:Featuregroup Number:Rescale of 52 : 1 : 8 : 0

	f1-score	precision	recall	support
AFIB	0.659892	0.657984	0.661871	875.0
GSVT	0.672122	0.671576	0.672717	691.0
SB	0.713891	0.710935	0.716879	1553.0
SR	0.692460	0.700067	0.685053	907.0
macro avg	0.684591	0.685140	0.684130	4026.0
micro avg	0.690174	0.690174	0.690174	4026.0
weighted avg	0.690158	0.690223	0.690174	4026.0

The result of Model Number:Dataset Number:Featuregroup Number:Rescale of 52 : 1 : 8 : 1

	f1-score	precision	recall	support
AFIB	0.778604	0.771642	0.785726	875.0
GSVT	0.791105	0.794235	0.788028	691.0
SB	0.843487	0.841921	0.845061	1553.0
SR	0.822113	0.829712	0.814681	907.0
macro avg	0.808827	0.809378	0.808374	4026.0
micro avg	0.815532	0.815532	0.815532	4026.0
weighted avg	0.815579	0.815712	0.815532	4026.0

The result of Model Number:Dataset Number:Featuregroup Number:Rescale of 52 : 1 : 9 : 0

	f1-score	precision	recall	support
AFIB	0.763229	0.761022	0.765518	875.0
GSVT	0.777374	0.776742	0.778062	691.0
SB	0.825684	0.822264	0.829139	1553.0
SR	0.800896	0.809695	0.792330	907.0
macro avg	0.791796	0.792431	0.791262	4026.0
micro avg	0.798253	0.798253	0.798253	4026.0
weighted avg	0.798234	0.798309	0.798253	4026.0

The result of Model Number:Dataset Number:Featuregroup Number:Rescale of 52 : 1 : 9 : 1

	f1-score	precision	recall	support
AFIB	0.652397	0.676585	0.629972	204.0
GSVT	0.671942	0.663541	0.680645	300.0
SB	0.688981	0.682695	0.695391	892.0
SR	0.670868	0.676074	0.665747	681.0
macro avg	0.671047	0.674724	0.667939	2077.0
micro avg	0.677116	0.677116	0.677116	2077.0
weighted avg	0.676988	0.677157	0.677116	2077.0

The result of Model Number:Dataset Number:Featuregroup Number:Rescale of 52 : 1 : 10 : 0

	f1-score	precision	recall	support
AFIB	0.751801	0.779674	0.725959	204.0
GSVT	0.774324	0.764642	0.784352	300.0
SB	0.793959	0.786714	0.801345	892.0

SR 0.773085 0.779085 0.767185 681.0
macro avg 0.773292 0.777529 0.769710 2077.0
micro avg 0.780286 0.780286 0.780286 2077.0
weighted avg 0.780138 0.780333 0.780286 2077.0

The result of Model Number:Dataset Number:Featuregroup Number:Rescale of 52 : 1 : 10 : 1

	f1-score	precision	recall	support
AFIB	0.824071	0.825663	0.822547	875.0
GSVT	0.798385	0.794878	0.801971	691.0
SB	0.834218	0.783514	0.891961	1553.0
SR	0.671429	0.777990	0.590701	907.0
macro avg	0.782026	0.795511	0.776795	4026.0
micro avg	0.793560	0.793560	0.793560	4026.0
weighted avg	0.789188	0.793381	0.793560	4026.0

The result of Model Number:Dataset Number:Featuregroup Number:Rescale of 53 : 0 : 0 : 0

	f1-score	precision	recall	support
AFIB	0.859858	0.864245	0.855544	875.0
GSVT	0.871725	0.867189	0.876383	691.0
SB	0.913704	0.912887	0.914528	1553.0
SR	0.896879	0.897610	0.896186	907.0
macro avg	0.885541	0.885483	0.885660	4026.0
micro avg	0.891030	0.891030	0.891030	4026.0
weighted avg	0.891006	0.891030	0.891030	4026.0

The result of Model Number:Dataset Number:Featuregroup Number:Rescale of 53 : 0 : 0 : 1

	f1-score	precision	recall	support
AFIB	0.763453	0.782785	0.745124	204.0
GSVT	0.780033	0.771551	0.788833	300.0
SB	0.802004	0.798001	0.806049	892.0
SR	0.789573	0.793115	0.786094	681.0
macro avg	0.783766	0.786363	0.781525	2077.0
micro avg	0.791036	0.791036	0.791036	2077.0
weighted avg	0.790968	0.791084	0.791036	2077.0

The result of Model Number:Dataset Number:Featuregroup Number:Rescale of 53 : 0 : 1 : 0

	f1-score	precision	recall	support
AFIB	0.559168	0.617654	0.510963	204.0
GSVT	0.657822	0.649137	0.666813	300.0
SB	0.697618	0.694907	0.700354	892.0
SR	0.680854	0.671151	0.690850	681.0
macro avg	0.648866	0.658212	0.642245	2077.0
micro avg	0.673792	0.673792	0.673792	2077.0
weighted avg	0.672775	0.672919	0.673792	2077.0

The result of Model Number:Dataset Number:Featuregroup Number:Rescale of 53 : 0 : 1 : 1

	f1-score	precision	recall	support
AFIB	0.790838	0.800086	0.781949	204.0
GSVT	0.713340	0.686106	0.813911	300.0
SB	0.661834	0.660274	0.663407	892.0
SR	0.821774	0.823354	0.820207	681.0
macro avg	0.746946	0.742455	0.769868	2077.0
micro avg	0.748200	0.748200	0.748200	2077.0
weighted avg	0.734385	0.731207	0.748200	2077.0

The result of Model Number:Dataset Number:Featuregroup Number:Rescale of 53 : 0 : 2 : 0

	f1-score	precision	recall	support
AFIB	0.822398	0.824585	0.820272	875.0
GSVT	0.838212	0.832307	0.844251	691.0
SB	0.886089	0.877055	0.895329	1553.0
SR	0.845585	0.864003	0.827965	907.0
macro avg	0.848071	0.849487	0.846954	4026.0
micro avg	0.855074	0.855074	0.855074	4026.0
weighted avg	0.854904	0.855030	0.855074	4026.0

The result of Model Number:Dataset Number:Featuregroup Number:Rescale of 53 : 0 : 2 : 1

	f1-score	precision	recall	support
AFIB	0.904123	0.910884	0.897497	204.0
GSVT	0.927225	0.922332	0.932172	300.0
SB	0.942873	0.941612	0.944141	892.0
SR	0.934569	0.936361	0.932786	681.0
macro avg	0.927198	0.927797	0.926649	2077.0
micro avg	0.934108	0.934108	0.934108	2077.0
weighted avg	0.934084	0.934088	0.934108	2077.0

The result of Model Number:Dataset Number:Featuregroup Number:Rescale of 53 : 0 : 3 : 0

	f1-score	precision	recall	support
AFIB	0.777807	0.767033	0.788918	875.0
GSVT	0.795058	0.801492	0.788730	691.0
SB	0.843944	0.841519	0.846389	1553.0
SR	0.823414	0.834131	0.812977	907.0
macro avg	0.810056	0.811044	0.809253	4026.0
micro avg	0.816475	0.816475	0.816475	4026.0
weighted avg	0.816554	0.816796	0.816475	4026.0

The result of Model Number:Dataset Number:Featuregroup Number:Rescale of 53 : 0 : 3 : 1

	f1-score	precision	recall	support
AFIB	0.690958	0.692195	0.689746	875.0

GSVT	0.661638	0.665114	0.658217	691.0
SB	0.699853	0.661354	0.743117	1553.0
SR	0.581231	0.654218	0.522946	907.0
macro avg	0.658420	0.668220	0.653507	4026.0
micro avg	0.667344	0.667344	0.667344	4026.0
weighted avg	0.664637	0.667095	0.667344	4026.0

The result of Model Number:Dataset Number:Featuregroup Number:Rescale of 53 : 0 : 4 : 0

	f1-score	precision	recall	support
AFIB	0.776369	0.796363	0.757386	204.0
GSVT	0.712454	0.680740	0.804004	300.0
SB	0.817391	0.815479	0.819314	892.0
SR	0.648452	0.648573	0.648338	681.0
macro avg	0.738666	0.735289	0.757260	2077.0
micro avg	0.754961	0.754961	0.754961	2077.0
weighted avg	0.742814	0.739415	0.754961	2077.0

The result of Model Number:Dataset Number:Featuregroup Number:Rescale of 53 : 0 : 4 : 1

	f1-score	precision	recall	support
AFIB	0.718620	0.734628	0.703501	204.0
GSVT	0.727486	0.716831	0.738765	300.0
SB	0.743925	0.736532	0.751501	892.0
SR	0.730202	0.740690	0.720030	681.0
macro avg	0.730058	0.732170	0.728449	2077.0
micro avg	0.734628	0.734628	0.734628	2077.0
weighted avg	0.734566	0.734863	0.734628	2077.0

The result of Model Number:Dataset Number:Featuregroup Number:Rescale of 53 : 0 : 5 : 0

	f1-score	precision	recall	support
AFIB	0.681064	0.687273	0.675055	204.0
GSVT	0.698837	0.696549	0.701162	300.0
SB	0.711613	0.711138	0.712090	892.0
SR	0.705354	0.705151	0.705567	681.0
macro avg	0.699217	0.700028	0.698469	2077.0
micro avg	0.704736	0.704736	0.704736	2077.0
weighted avg	0.704715	0.704724	0.704736	2077.0

The result of Model Number:Dataset Number:Featuregroup Number:Rescale of 53 : 0 : 5 : 1

	f1-score	precision	recall	support
AFIB	0.622418	0.617727	0.706436	204.0
GSVT	0.754560	0.750092	0.759088	300.0
SB	0.617124	0.615837	0.618421	892.0
SR	0.763239	0.762017	0.764473	681.0
macro avg	0.689335	0.686418	0.712105	2077.0

micro avg 0.695271 0.695271 0.695271 2077.0
weighted avg 0.685403 0.683343 0.695271 2077.0
The result of Model Number:Dataset Number:Featuregroup Number:Rescale of 53 : 0 : 6 : 0

	f1-score	precision	recall	support
AFIB	0.892573	0.941127	0.849011	204.0
GSVT	0.937037	0.922865	0.951688	300.0
SB	0.959304	0.953064	0.965641	892.0
SR	0.947469	0.948323	0.946639	681.0
macro avg	0.934096	0.941344	0.928245	2077.0
micro avg	0.945940	0.945940	0.945940	2077.0
weighted avg	0.945653	0.945975	0.945940	2077.0

The result of Model Number:Dataset Number:Featuregroup Number:Rescale of 53 : 0 : 6 : 1

	f1-score	precision	recall	support
AFIB	0.900650	0.919036	0.883384	204.0
GSVT	0.933354	0.925245	0.941777	300.0
SB	0.951299	0.949601	0.953006	892.0
SR	0.942445	0.943007	0.941889	681.0
macro avg	0.931937	0.934222	0.930014	2077.0
micro avg	0.940901	0.940901	0.940901	2077.0
weighted avg	0.940830	0.940919	0.940901	2077.0

The result of Model Number:Dataset Number:Featuregroup Number:Rescale of 53 : 0 : 7 : 0

	f1-score	precision	recall	support
AFIB	0.847277	0.851722	0.842929	875.0
GSVT	0.864993	0.863440	0.866615	691.0
SB	0.939235	0.932032	0.946552	1553.0
SR	0.906098	0.915167	0.897212	907.0
macro avg	0.889401	0.890590	0.888327	4026.0
micro avg	0.899195	0.899195	0.899195	4026.0
weighted avg	0.899041	0.899005	0.899195	4026.0

The result of Model Number:Dataset Number:Featuregroup Number:Rescale of 53 : 0 : 7 : 1

	f1-score	precision	recall	support
AFIB	0.841267	0.834574	0.848147	875.0
GSVT	0.807910	0.792549	0.823946	691.0
SB	0.847931	0.782714	0.925018	1553.0
SR	0.649280	0.830199	0.533224	907.0
macro avg	0.786597	0.810009	0.782584	4026.0
micro avg	0.802698	0.802698	0.802698	4026.0
weighted avg	0.794861	0.806371	0.802698	4026.0

The result of Model Number:Dataset Number:Featuregroup Number:Rescale of 53 : 0 : 8 : 0

	f1-score	precision	recall	support
AFIB	0.793766	0.799864	0.787942	204.0
GSVT	0.821719	0.820931	0.822535	300.0
SB	0.838530	0.836577	0.840501	892.0
SR	0.830813	0.831933	0.829715	681.0
macro avg	0.821207	0.822326	0.820173	2077.0
micro avg	0.829207	0.829207	0.829207	2077.0
weighted avg	0.829175	0.829189	0.829207	2077.0

The result of Model Number:Dataset Number:Featuregroup Number:Rescale of 53 : 0 : 8 : 1

	f1-score	precision	recall	support
AFIB	0.775882	0.840652	0.721925	204.0
GSVT	0.832223	0.821758	0.843297	300.0
SB	0.853468	0.841512	0.865869	892.0
SR	0.830187	0.834076	0.826739	681.0
macro avg	0.822940	0.834500	0.814457	2077.0
micro avg	0.835641	0.835641	0.835641	2077.0
weighted avg	0.835146	0.836136	0.835641	2077.0

The result of Model Number:Dataset Number:Featuregroup Number:Rescale of 53 : 0 : 9 : 0

	f1-score	precision	recall	support
AFIB	0.688192	0.725913	0.654656	204.0
GSVT	0.655305	0.678281	0.633916	300.0
SB	0.674046	0.648049	0.702227	892.0
SR	0.599919	0.616195	0.584515	681.0
macro avg	0.654366	0.667109	0.643829	2077.0
micro avg	0.649093	0.649093	0.649093	2077.0
weighted avg	0.648424	0.649619	0.649093	2077.0

The result of Model Number:Dataset Number:Featuregroup Number:Rescale of 53 : 0 : 9 : 1

	f1-score	precision	recall	support
AFIB	0.854011	0.863996	0.844418	204.0
GSVT	0.877504	0.873469	0.881600	300.0
SB	0.889928	0.887161	0.892719	892.0
SR	0.882264	0.884765	0.879815	681.0
macro avg	0.875927	0.877348	0.874638	2077.0
micro avg	0.882138	0.882138	0.882138	2077.0
weighted avg	0.882093	0.882122	0.882138	2077.0

The result of Model Number:Dataset Number:Featuregroup Number:Rescale of 53 : 0 : 10 : 0

	f1-score	precision	recall	support
AFIB	0.654796	0.657186	0.652467	875.0
GSVT	0.666114	0.657440	0.675076	691.0
SB	0.702151	0.694418	0.710063	1553.0

SR 0.665438 0.683820 0.648054 907.0
macro avg 0.672125 0.673216 0.671415 4026.0
micro avg 0.677571 0.677571 0.677571 4026.0
weighted avg 0.677403 0.677592 0.677571 4026.0
The result of Model Number:Dataset Number:Featuregroup Number:Rescale of 53 : 0 : 10 : 1

	f1-score	precision	recall	support
AFIB	0.722593	0.729154	0.716521	204.0
GSVT	0.746476	0.741964	0.751238	300.0
SB	0.762093	0.761071	0.763118	892.0
SR	0.755041	0.756607	0.753487	681.0
macro avg	0.746551	0.747199	0.746091	2077.0
micro avg	0.753667	0.753667	0.753667	2077.0
weighted avg	0.753645	0.753713	0.753667	2077.0

The result of Model Number:Dataset Number:Featuregroup Number:Rescale of 53 : 1 : 0 : 0

	f1-score	precision	recall	support
AFIB	0.904569	0.921164	0.888632	204.0
GSVT	0.916500	0.907251	0.925958	300.0
SB	0.939075	0.934187	0.944021	892.0
SR	0.926291	0.932095	0.920579	681.0
macro avg	0.921609	0.923674	0.919797	2077.0
micro avg	0.928286	0.928286	0.928286	2077.0
weighted avg	0.928234	0.928331	0.928286	2077.0

The result of Model Number:Dataset Number:Featuregroup Number:Rescale of 53 : 1 : 0 : 1

	f1-score	precision	recall	support
AFIB	0.798330	0.806052	0.790898	204.0
GSVT	0.826579	0.824949	0.828228	300.0
SB	0.843856	0.842361	0.845367	892.0
SR	0.836076	0.836481	0.835708	681.0
macro avg	0.826210	0.827461	0.825050	2077.0
micro avg	0.834375	0.834375	0.834375	2077.0
weighted avg	0.834338	0.834352	0.834375	2077.0

The result of Model Number:Dataset Number:Featuregroup Number:Rescale of 53 : 1 : 1 : 0

	f1-score	precision	recall	support
AFIB	0.696349	0.766604	0.638297	204.0
GSVT	0.821199	0.809864	0.832987	300.0
SB	0.871954	0.869045	0.874886	892.0
SR	0.849624	0.837870	0.861724	681.0
macro avg	0.809781	0.820845	0.801974	2077.0
micro avg	0.841281	0.841281	0.841281	2077.0
weighted avg	0.840054	0.840213	0.841281	2077.0

The result of Model Number:Dataset Number:Featuregroup Number:Rescale of 53 : 1 : 1 : 1

	f1-score	precision	recall	support
AFIB	0.879500	0.918098	0.844237	204.0
GSVT	0.818757	0.824652	0.813282	300.0
SB	0.849116	0.806476	0.896520	892.0
SR	0.753299	0.803035	0.709523	681.0
macro avg	0.825168	0.838065	0.815891	2077.0
micro avg	0.818050	0.818050	0.818050	2077.0
weighted avg	0.816299	0.818936	0.818050	2077.0

The result of Model Number:Dataset Number:Featuregroup Number:Rescale of 53 : 1 : 2 : 0

	f1-score	precision	recall	support
AFIB	0.901802	0.914568	0.890127	204.0
GSVT	0.920525	0.903576	0.938290	300.0
SB	0.940144	0.929750	0.950790	892.0
SR	0.920386	0.939132	0.902429	681.0
macro avg	0.920714	0.921756	0.920409	2077.0
micro avg	0.927170	0.927170	0.927170	2077.0
weighted avg	0.927066	0.927554	0.927170	2077.0

The result of Model Number:Dataset Number:Featuregroup Number:Rescale of 53 : 1 : 2 : 1

	f1-score	precision	recall	support
AFIB	0.676804	0.725724	0.634529	204.0
GSVT	0.641074	0.644542	0.637906	300.0
SB	0.669614	0.629462	0.715254	892.0
SR	0.587972	0.634554	0.547836	681.0
macro avg	0.643866	0.658570	0.633881	2077.0
micro avg	0.641261	0.641261	0.641261	2077.0
weighted avg	0.639429	0.642764	0.641261	2077.0

The result of Model Number:Dataset Number:Featuregroup Number:Rescale of 53 : 1 : 3 : 0

	f1-score	precision	recall	support
AFIB	0.653439	0.652388	0.654761	875.0
GSVT	0.668675	0.665189	0.672236	691.0
SB	0.699134	0.693211	0.705165	1553.0
SR	0.672446	0.687395	0.658359	907.0
macro avg	0.673424	0.674546	0.672630	4026.0
micro avg	0.678014	0.678014	0.678014	4026.0
weighted avg	0.677963	0.678219	0.678014	4026.0

The result of Model Number:Dataset Number:Featuregroup Number:Rescale of 53 : 1 : 3 : 1

	f1-score	precision	recall	support
AFIB	0.766780	0.780577	0.753689	204.0

GSVT 0.784079 0.773054 0.795552 300.0
SB 0.796143 0.786736 0.805781 892.0
SR 0.780131 0.794020 0.766728 681.0
macro avg 0.781783 0.783597 0.780438 2077.0
micro avg 0.786383 0.786383 0.786383 2077.0
weighted avg 0.786267 0.786543 0.786383 2077.0
The result of Model Number:Dataset Number:Featuregroup Number:Rescale of 53 : 1 : 4 : 0

	f1-score	precision	recall	support
AFIB	0.715056	0.716500	0.713633	204.0
GSVT	0.734949	0.732764	0.737151	300.0
SB	0.747127	0.746461	0.747797	892.0
SR	0.740705	0.742128	0.739298	681.0
macro avg	0.734459	0.734463	0.734470	2077.0
micro avg	0.740117	0.740117	0.740117	2077.0
weighted avg	0.740113	0.740119	0.740117	2077.0

The result of Model Number:Dataset Number:Featuregroup Number:Rescale of 53 : 1 : 4 : 1

	f1-score	precision	recall	support
AFIB	0.765288	0.771466	0.759274	204.0
GSVT	0.782848	0.779010	0.786752	300.0
SB	0.798127	0.797242	0.799021	892.0
SR	0.792167	0.793221	0.791122	681.0
macro avg	0.784608	0.785235	0.784042	2077.0
micro avg	0.790755	0.790755	0.790755	2077.0
weighted avg	0.790741	0.790758	0.790755	2077.0

The result of Model Number:Dataset Number:Featuregroup Number:Rescale of 53 : 1 : 5 : 0

	f1-score	precision	recall	support
AFIB	0.654746	0.651450	0.658106	875.0
GSVT	0.667500	0.670731	0.664310	691.0
SB	0.710928	0.709292	0.712574	1553.0
SR	0.693130	0.696839	0.689468	907.0
macro avg	0.681576	0.682078	0.681114	4026.0
micro avg	0.687247	0.687247	0.687247	4026.0
weighted avg	0.687254	0.687297	0.687247	4026.0

The result of Model Number:Dataset Number:Featuregroup Number:Rescale of 53 : 1 : 5 : 1

	f1-score	precision	recall	support
AFIB	0.770425	0.775926	0.765139	204.0
GSVT	0.797364	0.796850	0.797894	300.0
SB	0.813318	0.811513	0.815139	892.0
SR	0.806048	0.807023	0.805100	681.0
macro avg	0.796789	0.797828	0.795818	2077.0

micro avg 0.804445 0.804445 0.804445 2077.0
weighted avg 0.804417 0.804428 0.804445 2077.0
The result of Model Number:Dataset Number:Featuregroup Number:Rescale of 53 : 1 : 6 : 0

	f1-score	precision	recall	support
AFIB	0.847966	0.856169	0.840073	204.0
GSVT	0.877972	0.876241	0.879724	300.0
SB	0.896323	0.894735	0.897928	892.0
SR	0.888060	0.888490	0.887668	681.0
macro avg	0.877580	0.878909	0.876348	2077.0
micro avg	0.886252	0.886252	0.886252	2077.0
weighted avg	0.886214	0.886228	0.886252	2077.0

The result of Model Number:Dataset Number:Featuregroup Number:Rescale of 53 : 1 : 6 : 1

	f1-score	precision	recall	support
AFIB	0.618188	0.689472	0.560345	204.0
GSVT	0.728907	0.716220	0.742207	300.0
SB	0.778248	0.774798	0.781736	892.0
SR	0.760187	0.749793	0.770897	681.0
macro avg	0.721382	0.732571	0.713796	2077.0
micro avg	0.750728	0.750728	0.750728	2077.0
weighted avg	0.749478	0.749758	0.750728	2077.0

The result of Model Number:Dataset Number:Featuregroup Number:Rescale of 53 : 1 : 7 : 0

	f1-score	precision	recall	support
AFIB	0.683138	0.697083	0.670041	204.0
GSVT	0.707944	0.701793	0.714332	300.0
SB	0.721555	0.720267	0.722849	892.0
SR	0.714839	0.715265	0.714417	681.0
macro avg	0.706869	0.708602	0.705410	2077.0
micro avg	0.713668	0.713668	0.713668	2077.0
weighted avg	0.713614	0.713681	0.713668	2077.0

The result of Model Number:Dataset Number:Featuregroup Number:Rescale of 53 : 1 : 7 : 1

	f1-score	precision	recall	support
AFIB	0.699883	0.707499	0.692634	204.0
GSVT	0.723568	0.720551	0.726728	300.0
SB	0.738396	0.737735	0.739058	892.0
SR	0.731417	0.731424	0.731418	681.0
macro avg	0.723316	0.724302	0.722459	2077.0
micro avg	0.730212	0.730212	0.730212	2077.0
weighted avg	0.730183	0.730214	0.730212	2077.0

The result of Model Number:Dataset Number:Featuregroup Number:Rescale of 53 : 1 : 8 : 0

	f1-score	precision	recall	support
AFIB	0.664649	0.689958	0.641275	204.0
GSVT	0.685824	0.678912	0.692909	300.0
SB	0.699552	0.696069	0.703079	892.0
SR	0.691102	0.691537	0.690690	681.0
macro avg	0.685282	0.689119	0.681988	2077.0
micro avg	0.691478	0.691478	0.691478	2077.0
weighted avg	0.691370	0.691505	0.691478	2077.0

The result of Model Number:Dataset Number:Featuregroup Number:Rescale of 53 : 1 : 8 : 1

	f1-score	precision	recall	support
AFIB	0.813383	0.818251	0.808614	204.0
GSVT	0.835050	0.832568	0.837553	300.0
SB	0.847838	0.846799	0.848882	892.0
SR	0.841657	0.842650	0.840673	681.0
macro avg	0.834482	0.835067	0.833930	2077.0
micro avg	0.840599	0.840599	0.840599	2077.0
weighted avg	0.840580	0.840579	0.840599	2077.0

The result of Model Number:Dataset Number:Featuregroup Number:Rescale of 53 : 1 : 9 : 0

	f1-score	precision	recall	support
AFIB	0.676819	0.689273	0.665150	204.0
GSVT	0.701790	0.695912	0.707889	300.0
SB	0.714967	0.713769	0.716170	892.0
SR	0.708655	0.709284	0.708030	681.0
macro avg	0.700558	0.702060	0.699310	2077.0
micro avg	0.707294	0.707294	0.707294	2077.0
weighted avg	0.707247	0.707314	0.707294	2077.0

The result of Model Number:Dataset Number:Featuregroup Number:Rescale of 53 : 1 : 9 : 1

	f1-score	precision	recall	support
AFIB	0.686119	0.758667	0.626591	204.0
GSVT	0.789469	0.780333	0.798973	300.0
SB	0.834689	0.831257	0.838151	892.0
SR	0.816764	0.804666	0.829240	681.0
macro avg	0.781760	0.793731	0.773239	2077.0
micro avg	0.808792	0.808792	0.808792	2077.0
weighted avg	0.807688	0.808053	0.808792	2077.0

The result of Model Number:Dataset Number:Featuregroup Number:Rescale of 53 : 1 : 10 : 0

	f1-score	precision	recall	support
AFIB	0.771072	0.773029	0.769152	875.0
GSVT	0.784678	0.776179	0.793399	691.0
SB	0.827008	0.817653	0.836592	1553.0

SR 0.789196 0.810802 0.768743 907.0
macro avg 0.792989 0.794416 0.791972 4026.0
micro avg 0.799236 0.799236 0.799236 4026.0
weighted avg 0.799067 0.799293 0.799236 4026.0
The result of Model Number:Dataset Number:Featuregroup Number:Rescale of 53 : 1 : 10 : 1

	f1-score	precision	recall	support
AFIB	0.740576	0.780862	0.704433	204.0
GSVT	0.777468	0.765710	0.789625	300.0
SB	0.795944	0.790766	0.801202	892.0
SR	0.786124	0.786833	0.785435	681.0
macro avg	0.775028	0.781043	0.770174	2077.0
micro avg	0.784856	0.784856	0.784856	2077.0
weighted avg	0.784618	0.784885	0.784856	2077.0

The result of Model Number:Dataset Number:Featuregroup Number:Rescale of 54 : 0 : 0 : 0

	f1-score	precision	recall	support
AFIB	0.868609	0.906229	0.834235	204.0
GSVT	0.811999	0.819576	0.804967	300.0
SB	0.841696	0.800314	0.887650	892.0
SR	0.750179	0.797521	0.708384	681.0
macro avg	0.818121	0.830910	0.808809	2077.0
micro avg	0.811684	0.811684	0.811684	2077.0
weighted avg	0.810043	0.812583	0.811684	2077.0

The result of Model Number:Dataset Number:Featuregroup Number:Rescale of 54 : 0 : 0 : 1

	f1-score	precision	recall	support
AFIB	0.879585	0.870738	0.888639	875.0
GSVT	0.887456	0.895358	0.879758	691.0
SB	0.938890	0.936008	0.941791	1553.0
SR	0.920708	0.928768	0.912791	907.0
macro avg	0.906660	0.907718	0.905745	4026.0
micro avg	0.913059	0.913059	0.913059	4026.0
weighted avg	0.913077	0.913214	0.913059	4026.0

The result of Model Number:Dataset Number:Featuregroup Number:Rescale of 54 : 0 : 1 : 0

	f1-score	precision	recall	support
AFIB	0.782559	0.752642	0.831785	875.0
GSVT	0.810864	0.782879	0.848822	691.0
SB	0.903453	0.887506	0.920976	1553.0
SR	0.765204	0.920114	0.660650	907.0
macro avg	0.815520	0.835785	0.815558	4026.0
micro avg	0.830560	0.830560	0.830560	4026.0
weighted avg	0.830141	0.847583	0.830560	4026.0

The result of Model Number:Dataset Number:Featuregroup Number:Rescale of 54 : 0 : 1 : 1

	f1-score	precision	recall	support
AFIB	0.873174	0.881679	0.865043	204.0
GSVT	0.893366	0.889264	0.897536	300.0
SB	0.905711	0.902595	0.908856	892.0
SR	0.898062	0.901547	0.894648	681.0
macro avg	0.892578	0.893771	0.891521	2077.0
micro avg	0.898259	0.898259	0.898259	2077.0
weighted avg	0.898224	0.898271	0.898259	2077.0

The result of Model Number:Dataset Number:Featuregroup Number:Rescale of 54 : 0 : 2 : 0

	f1-score	precision	recall	support
AFIB	0.657927	0.663781	0.652211	875.0
GSVT	0.671737	0.664365	0.679317	691.0
SB	0.722449	0.716824	0.728169	1553.0
SR	0.698441	0.708341	0.688817	907.0
macro avg	0.687638	0.688328	0.687128	4026.0
micro avg	0.694410	0.694410	0.694410	4026.0
weighted avg	0.694313	0.694381	0.694410	4026.0

The result of Model Number:Dataset Number:Featuregroup Number:Rescale of 54 : 0 : 2 : 1

	f1-score	precision	recall	support
AFIB	0.834132	0.850865	0.818210	204.0
GSVT	0.853463	0.844592	0.862568	300.0
SB	0.869247	0.865866	0.872658	892.0
SR	0.860113	0.863702	0.856574	681.0
macro avg	0.854239	0.856256	0.852502	2077.0
micro avg	0.860579	0.860579	0.860579	2077.0
weighted avg	0.860524	0.860610	0.860579	2077.0

The result of Model Number:Dataset Number:Featuregroup Number:Rescale of 54 : 0 : 3 : 0

	f1-score	precision	recall	support
AFIB	0.877197	0.922515	0.836383	204.0
GSVT	0.919816	0.910825	0.929006	300.0
SB	0.936034	0.928504	0.943693	892.0
SR	0.924473	0.925598	0.923388	681.0
macro avg	0.914380	0.921861	0.908117	2077.0
micro avg	0.924374	0.924374	0.924374	2077.0
weighted avg	0.924122	0.924409	0.924374	2077.0

The result of Model Number:Dataset Number:Featuregroup Number:Rescale of 54 : 0 : 3 : 1

	f1-score	precision	recall	support
AFIB	0.904581	0.914424	0.895211	204.0

GSVT 0.935193 0.931293 0.939278 300.0
SB 0.954357 0.953503 0.955213 892.0
SR 0.945338 0.945346 0.945338 681.0
macro avg 0.934867 0.936142 0.933760 2077.0
micro avg 0.943780 0.943780 0.943780 2077.0
weighted avg 0.943743 0.943782 0.943780 2077.0
The result of Model Number:Dataset Number:Featuregroup Number:Rescale of 54 : 0 : 4 : 0

	f1-score	precision	recall	support
AFIB	0.841075	0.876647	0.808557	204.0
GSVT	0.774569	0.775925	0.784836	300.0
SB	0.833895	0.794169	0.878613	892.0
SR	0.737204	0.796580	0.687399	681.0
macro avg	0.796686	0.810830	0.789851	2077.0
micro avg	0.795493	0.795493	0.795493	2077.0
weighted avg	0.794328	0.800425	0.795493	2077.0

The result of Model Number:Dataset Number:Featuregroup Number:Rescale of 54 : 0 : 4 : 1

	f1-score	precision	recall	support
AFIB	0.756983	0.764357	0.749935	204.0
GSVT	0.774488	0.770932	0.778104	300.0
SB	0.785191	0.782490	0.787917	892.0
SR	0.778560	0.781581	0.775600	681.0
macro avg	0.773806	0.774840	0.772889	2077.0
micro avg	0.778730	0.778730	0.778730	2077.0
weighted avg	0.778700	0.778741	0.778730	2077.0

The result of Model Number:Dataset Number:Featuregroup Number:Rescale of 54 : 0 : 5 : 0

	f1-score	precision	recall	support
AFIB	0.607113	0.630704	0.585266	875.0
GSVT	0.652666	0.647846	0.657644	691.0
SB	0.717230	0.713534	0.720968	1553.0
SR	0.674978	0.662107	0.688433	907.0
macro avg	0.662997	0.663548	0.663078	4026.0
micro avg	0.673277	0.673277	0.673277	4026.0
weighted avg	0.672697	0.672672	0.673277	4026.0

The result of Model Number:Dataset Number:Featuregroup Number:Rescale of 54 : 0 : 5 : 1

	f1-score	precision	recall	support
AFIB	0.747012	0.735296	0.759189	875.0
GSVT	0.719795	0.740431	0.700363	691.0
SB	0.756385	0.718044	0.799096	1553.0
SR	0.614559	0.683879	0.558094	907.0
macro avg	0.709438	0.719412	0.704186	4026.0

micro avg 0.719183 0.719183 0.719183 4026.0
weighted avg 0.716117 0.717939 0.719183 4026.0
The result of Model Number:Dataset Number:Featuregroup Number:Rescale of 54 : 0 : 6 : 0

	f1-score	precision	recall	support
AFIB	0.631393	0.634705	0.628153	875.0
GSVT	0.644595	0.643437	0.645803	691.0
SB	0.699920	0.694552	0.705373	1553.0
SR	0.675226	0.681985	0.668604	907.0
macro avg	0.662784	0.663670	0.661983	4026.0
micro avg	0.670083	0.670083	0.670083	4026.0
weighted avg	0.669968	0.669941	0.670083	4026.0

The result of Model Number:Dataset Number:Featuregroup Number:Rescale of 54 : 0 : 6 : 1

	f1-score	precision	recall	support
AFIB	0.767682	0.779453	0.756388	204.0
GSVT	0.785885	0.779217	0.792701	300.0
SB	0.799556	0.796976	0.802155	892.0
SR	0.791340	0.794277	0.788434	681.0
macro avg	0.786116	0.787481	0.784919	2077.0
micro avg	0.791795	0.791795	0.791795	2077.0
weighted avg	0.791757	0.791805	0.791795	2077.0

The result of Model Number:Dataset Number:Featuregroup Number:Rescale of 54 : 0 : 7 : 0

	f1-score	precision	recall	support
AFIB	0.669735	0.663812	0.675793	875.0
GSVT	0.608905	0.589679	0.677419	691.0
SB	0.548961	0.527079	0.583182	1553.0
SR	0.565784	0.571639	0.560085	907.0
macro avg	0.598347	0.588052	0.624120	4026.0
micro avg	0.614281	0.614281	0.614281	4026.0
weighted avg	0.589288	0.577579	0.614281	4026.0

The result of Model Number:Dataset Number:Featuregroup Number:Rescale of 54 : 0 : 7 : 1

	f1-score	precision	recall	support
AFIB	0.862414	0.868964	0.856035	204.0
GSVT	0.880070	0.871474	0.888856	300.0
SB	0.900598	0.898296	0.902920	892.0
SR	0.891045	0.896079	0.886079	681.0
macro avg	0.883532	0.883703	0.883472	2077.0
micro avg	0.890762	0.890762	0.890762	2077.0
weighted avg	0.890751	0.890814	0.890762	2077.0

The result of Model Number:Dataset Number:Featuregroup Number:Rescale of 54 : 0 : 8 : 0

	f1-score	precision	recall	support
AFIB	0.683921	0.686274	0.681670	875.0
GSVT	0.696905	0.683001	0.711513	691.0
SB	0.737730	0.733740	0.741767	1553.0
SR	0.709196	0.725685	0.693481	907.0
macro avg	0.706938	0.707175	0.707108	4026.0
micro avg	0.712635	0.712635	0.712635	4026.0
weighted avg	0.712600	0.712901	0.712635	4026.0

The result of Model Number:Dataset Number:Featuregroup Number:Rescale of 54 : 0 : 8 : 1

	f1-score	precision	recall	support
AFIB	0.746584	0.764499	0.729828	204.0
GSVT	0.773130	0.768649	0.777757	300.0
SB	0.789642	0.786745	0.792562	892.0
SR	0.782780	0.783360	0.782209	681.0
macro avg	0.773034	0.775813	0.770589	2077.0
micro avg	0.780868	0.780868	0.780868	2077.0
weighted avg	0.780778	0.780837	0.780868	2077.0

The result of Model Number:Dataset Number:Featuregroup Number:Rescale of 54 : 0 : 9 : 0

	f1-score	precision	recall	support
AFIB	0.844316	0.849369	0.839365	204.0
GSVT	0.866808	0.864231	0.869406	300.0
SB	0.880081	0.879003	0.881165	892.0
SR	0.873666	0.874696	0.872644	681.0
macro avg	0.866218	0.866825	0.865645	2077.0
micro avg	0.872567	0.872567	0.872567	2077.0
weighted avg	0.872548	0.872547	0.872567	2077.0

The result of Model Number:Dataset Number:Featuregroup Number:Rescale of 54 : 0 : 9 : 1

	f1-score	precision	recall	support
AFIB	0.587680	0.583979	0.661980	204.0
GSVT	0.716391	0.712706	0.720235	300.0
SB	0.736789	0.733283	0.740336	892.0
SR	0.577928	0.579832	0.576385	681.0
macro avg	0.654697	0.652450	0.674734	2077.0
micro avg	0.675981	0.675981	0.675981	2077.0
weighted avg	0.667111	0.665334	0.675981	2077.0

The result of Model Number:Dataset Number:Featuregroup Number:Rescale of 54 : 0 : 10 : 0

	f1-score	precision	recall	support
AFIB	0.838040	0.830184	0.846154	875.0
GSVT	0.794543	0.775445	0.852951	691.0
SB	0.918740	0.915366	0.922164	1553.0

SR	0.714579	0.719591	0.709647	907.0
macro avg	0.816476	0.810147	0.832729	4026.0
micro avg	0.845888	0.845888	0.845888	4026.0
weighted avg	0.833890	0.828732	0.845888	4026.0

The result of Model Number:Dataset Number:Featuregroup Number:Rescale of 54 : 0 : 10 : 1

	f1-score	precision	recall	support
AFIB	0.722316	0.742142	0.703576	204.0
GSVT	0.738653	0.727305	0.750431	300.0
SB	0.754882	0.747581	0.762329	892.0
SR	0.735803	0.744998	0.726838	681.0
macro avg	0.737914	0.740506	0.735794	2077.0
micro avg	0.743203	0.743203	0.743203	2077.0
weighted avg	0.743084	0.743271	0.743203	2077.0

The result of Model Number:Dataset Number:Featuregroup Number:Rescale of 54 : 1 : 0 : 0

	f1-score	precision	recall	support
AFIB	0.653037	0.642582	0.734799	204.0
GSVT	0.764534	0.755663	0.773895	300.0
SB	0.790133	0.787149	0.793145	892.0
SR	0.619953	0.623158	0.616886	681.0
macro avg	0.706914	0.702138	0.729681	2077.0
micro avg	0.726842	0.726842	0.726842	2077.0
weighted avg	0.717172	0.714633	0.726842	2077.0

The result of Model Number:Dataset Number:Featuregroup Number:Rescale of 54 : 1 : 0 : 1

	f1-score	precision	recall	support
AFIB	0.646151	0.638113	0.654466	875.0
GSVT	0.666381	0.674725	0.658306	691.0
SB	0.710935	0.710165	0.711712	1553.0
SR	0.691760	0.695307	0.688252	907.0
macro avg	0.678807	0.679578	0.678184	4026.0
micro avg	0.684819	0.684819	0.684819	4026.0
weighted avg	0.684888	0.685075	0.684819	4026.0

The result of Model Number:Dataset Number:Featuregroup Number:Rescale of 54 : 1 : 1 : 0

	f1-score	precision	recall	support
AFIB	0.182599	0.184517	0.180721	175.0
GSVT	0.182893	0.180950	0.184878	138.2
SB	0.192619	0.191880	0.193363	310.6
SR	0.188161	0.189104	0.187228	181.4
macro avg	0.186568	0.186613	0.186547	805.2
micro avg	0.187777	0.187777	0.187777	805.2
weighted avg	0.187768	0.187779	0.187777	805.2

The result of Model Number:Dataset Number:Featuregroup Number:Rescale of 54 : 1 : 1 : 1

	f1-score	precision	recall	support
AFIB	0.693247	0.679437	0.707666	875.0
GSVT	0.564242	0.524715	0.707261	691.0
SB	0.457820	0.458609	0.457034	1553.0
SR	0.742185	0.748720	0.735794	907.0
macro avg	0.614373	0.602870	0.651939	4026.0
micro avg	0.617254	0.617254	0.617254	4026.0
weighted avg	0.591316	0.583307	0.617254	4026.0

The result of Model Number:Dataset Number:Featuregroup Number:Rescale of 54 : 1 : 2 : 0

	f1-score	precision	recall	support
AFIB	0.825591	0.817939	0.833433	875.0
GSVT	0.840417	0.844981	0.835916	691.0
SB	0.890470	0.888417	0.892533	1553.0
SR	0.869723	0.877749	0.861870	907.0
macro avg	0.856550	0.857272	0.855938	4026.0
micro avg	0.863063	0.863063	0.863063	4026.0
weighted avg	0.863105	0.863241	0.863063	4026.0

The result of Model Number:Dataset Number:Featuregroup Number:Rescale of 54 : 1 : 2 : 1

	f1-score	precision	recall	support
AFIB	0.839881	0.853190	0.827092	204.0
GSVT	0.861469	0.854138	0.868932	300.0
SB	0.876343	0.874390	0.878308	892.0
SR	0.868039	0.869969	0.866135	681.0
macro avg	0.861433	0.862922	0.860117	2077.0
micro avg	0.867932	0.867932	0.867932	2077.0
weighted avg	0.867891	0.867933	0.867932	2077.0

The result of Model Number:Dataset Number:Featuregroup Number:Rescale of 54 : 1 : 3 : 0

	f1-score	precision	recall	support
AFIB	0.763037	0.762350	0.763799	875.0
GSVT	0.776256	0.769149	0.783557	691.0
SB	0.814588	0.807426	0.821879	1553.0
SR	0.776238	0.795428	0.758020	907.0
macro avg	0.782530	0.783588	0.781814	4026.0
micro avg	0.788292	0.788292	0.788292	4026.0
weighted avg	0.788166	0.788357	0.788292	4026.0

The result of Model Number:Dataset Number:Featuregroup Number:Rescale of 54 : 1 : 3 : 1

	f1-score	precision	recall	support
AFIB	0.804307	0.840054	0.771590	204.0

GSVT 0.837824 0.831247 0.844525 300.0
SB 0.850622 0.844060 0.857295 892.0
SR 0.841211 0.842600 0.839856 681.0
macro avg 0.833491 0.839490 0.828316 2077.0
micro avg 0.841315 0.841315 0.841315 2077.0
weighted avg 0.841139 0.841337 0.841315 2077.0
The result of Model Number:Dataset Number:Featuregroup Number:Rescale of 54 : 1 : 4 : 0

	f1-score	precision	recall	support
AFIB	0.767269	0.781351	0.754056	204.0
GSVT	0.796396	0.789993	0.803050	300.0
SB	0.811169	0.809721	0.812624	892.0
SR	0.803138	0.803853	0.802428	681.0
macro avg	0.794493	0.796229	0.793040	2077.0
micro avg	0.802146	0.802146	0.802146	2077.0
weighted avg	0.802090	0.802161	0.802146	2077.0

The result of Model Number:Dataset Number:Featuregroup Number:Rescale of 54 : 1 : 4 : 1

	f1-score	precision	recall	support
AFIB	0.660890	0.674381	0.648220	204.0
GSVT	0.684888	0.678937	0.691068	300.0
SB	0.698055	0.696809	0.699308	892.0
SR	0.691558	0.691970	0.691150	681.0
macro avg	0.683848	0.685524	0.682436	2077.0
micro avg	0.690425	0.690425	0.690425	2077.0
weighted avg	0.690373	0.690438	0.690425	2077.0

The result of Model Number:Dataset Number:Featuregroup Number:Rescale of 54 : 1 : 5 : 0

	f1-score	precision	recall	support
AFIB	0.842154	0.850329	0.834655	204.0
GSVT	0.867398	0.863298	0.871766	300.0
SB	0.884963	0.883975	0.885954	892.0
SR	0.877176	0.878085	0.876274	681.0
macro avg	0.867922	0.868922	0.867162	2077.0
micro avg	0.875693	0.875693	0.875693	2077.0
weighted avg	0.875668	0.875752	0.875693	2077.0

The result of Model Number:Dataset Number:Featuregroup Number:Rescale of 54 : 1 : 5 : 1

	f1-score	precision	recall	support
AFIB	0.811526	0.811743	0.811451	875.0
GSVT	0.825658	0.822609	0.828832	691.0
SB	0.883292	0.881148	0.885451	1553.0
SR	0.862227	0.868415	0.856150	907.0
macro avg	0.845676	0.845979	0.845471	4026.0

micro avg 0.853049 0.853049 0.853049 4026.0
weighted avg 0.853057 0.853148 0.853049 4026.0
The result of Model Number:Dataset Number:Featuregroup Number:Rescale of 54 : 1 : 6 : 0

	f1-score	precision	recall	support
AFIB	0.784962	0.780567	0.789444	875.0
GSVT	0.791354	0.796023	0.786788	691.0
SB	0.833626	0.831017	0.836254	1553.0
SR	0.817123	0.822529	0.811806	907.0
macro avg	0.806766	0.807534	0.806073	4026.0
micro avg	0.812083	0.812083	0.812083	4026.0
weighted avg	0.812076	0.812134	0.812083	4026.0

The result of Model Number:Dataset Number:Featuregroup Number:Rescale of 54 : 1 : 6 : 1

	f1-score	precision	recall	support
AFIB	0.668614	0.740835	0.609346	204.0
GSVT	0.775067	0.762461	0.788218	300.0
SB	0.826561	0.822798	0.830360	892.0
SR	0.806589	0.796325	0.817133	681.0
macro avg	0.769208	0.780605	0.761264	2077.0
micro avg	0.798228	0.798228	0.798228	2077.0
weighted avg	0.797061	0.797353	0.798228	2077.0

The result of Model Number:Dataset Number:Featuregroup Number:Rescale of 54 : 1 : 7 : 0

	f1-score	precision	recall	support
AFIB	0.834378	0.847600	0.821673	204.0
GSVT	0.855825	0.848542	0.863238	300.0
SB	0.870600	0.868660	0.872553	892.0
SR	0.862352	0.864269	0.860460	681.0
macro avg	0.855789	0.857268	0.854481	2077.0
micro avg	0.862245	0.862245	0.862245	2077.0
weighted avg	0.862204	0.862246	0.862245	2077.0

The result of Model Number:Dataset Number:Featuregroup Number:Rescale of 54 : 1 : 7 : 1

	f1-score	precision	recall	support
AFIB	0.849648	0.842888	0.856596	875.0
GSVT	0.815959	0.800445	0.832154	691.0
SB	0.856378	0.790512	0.934233	1553.0
SR	0.655748	0.838470	0.538536	907.0
macro avg	0.794433	0.818079	0.790380	4026.0
micro avg	0.810695	0.810695	0.810695	4026.0
weighted avg	0.802779	0.814404	0.810695	4026.0

The result of Model Number:Dataset Number:Featuregroup Number:Rescale of 54 : 1 : 8 : 0

	f1-score	precision	recall	support
AFIB	0.709830	0.740252	0.682079	204.0
GSVT	0.733682	0.722597	0.745160	300.0
SB	0.748235	0.740835	0.755786	892.0
SR	0.727963	0.734191	0.721867	681.0
macro avg	0.729927	0.734469	0.726223	2077.0
micro avg	0.735890	0.735890	0.735890	2077.0
weighted avg	0.735714	0.735965	0.735890	2077.0

The result of Model Number:Dataset Number:Featuregroup Number:Rescale of 54 : 1 : 8 : 1

	f1-score	precision	recall	support
AFIB	0.786188	0.813481	0.760772	204.0
GSVT	0.801262	0.792491	0.810329	300.0
SB	0.822593	0.816686	0.828587	892.0
SR	0.807902	0.811874	0.803982	681.0
macro avg	0.804486	0.808633	0.800917	2077.0
micro avg	0.811222	0.811222	0.811222	2077.0
weighted avg	0.811119	0.811299	0.811222	2077.0

The result of Model Number:Dataset Number:Featuregroup Number:Rescale of 54 : 1 : 9 : 0

	f1-score	precision	recall	support
AFIB	0.631104	0.683788	0.587215	204.0
GSVT	0.676932	0.668420	0.685940	300.0
SB	0.694214	0.684488	0.704300	892.0
SR	0.675277	0.678440	0.672472	681.0
macro avg	0.669382	0.678784	0.662482	2077.0
micro avg	0.679713	0.679713	0.679713	2077.0
weighted avg	0.679310	0.680115	0.679713	2077.0

The result of Model Number:Dataset Number:Featuregroup Number:Rescale of 54 : 1 : 9 : 1

	f1-score	precision	recall	support
AFIB	0.757717	0.756109	0.759366	875.0
GSVT	0.769555	0.765866	0.773344	691.0
SB	0.809953	0.800284	0.819861	1553.0
SR	0.772313	0.794193	0.751622	907.0
macro avg	0.777384	0.779113	0.776048	4026.0
micro avg	0.783356	0.783356	0.783356	4026.0
weighted avg	0.783187	0.783404	0.783356	4026.0

The result of Model Number:Dataset Number:Featuregroup Number:Rescale of 54 : 1 : 10 : 0

	f1-score	precision	recall	support
AFIB	0.707168	0.711049	0.703374	875.0
GSVT	0.708157	0.699204	0.717453	691.0
SB	0.744749	0.741761	0.747769	1553.0

	f1-score	precision	recall	support
SR	0.728762	0.737609	0.720170	907.0
macro avg	0.722209	0.722406	0.722191	4026.0
micro avg	0.726699	0.726699	0.726699	4026.0
weighted avg	0.726699	0.726846	0.726699	4026.0

The result of Model Number:Dataset Number:Featuregroup Number:Rescale of 54 : 1 : 10 : 1

	f1-score	precision	recall	support
AFIB	0.765957	0.758447	0.818961	204.0
GSVT	0.686799	0.679162	0.694780	300.0
SB	0.882579	0.876152	0.889107	892.0
SR	0.867777	0.873787	0.861919	681.0
macro avg	0.800778	0.796887	0.816192	2077.0
micro avg	0.845235	0.845235	0.845235	2077.0
weighted avg	0.837993	0.835363	0.845235	2077.0

The result of Model Number:Dataset Number:Featuregroup Number:Rescale of 55 : 0 : 0 : 0

	f1-score	precision	recall	support
AFIB	0.812753	0.829633	0.796733	875.0
GSVT	0.792596	0.765067	0.857294	691.0
SB	0.934198	0.929985	0.938464	1553.0
SR	0.710222	0.698755	0.722135	907.0
macro avg	0.812442	0.805860	0.828657	4026.0
micro avg	0.844993	0.844993	0.844993	4026.0
weighted avg	0.833041	0.827776	0.844993	4026.0

The result of Model Number:Dataset Number:Featuregroup Number:Rescale of 55 : 0 : 0 : 1

	f1-score	precision	recall	support
AFIB	0.800843	0.825509	0.777769	204.0
GSVT	0.821274	0.811220	0.831670	300.0
SB	0.843581	0.838569	0.848682	892.0
SR	0.829362	0.833452	0.825344	681.0
macro avg	0.823765	0.827188	0.820866	2077.0
micro avg	0.831608	0.831608	0.831608	2077.0
weighted avg	0.831499	0.831658	0.831608	2077.0

The result of Model Number:Dataset Number:Featuregroup Number:Rescale of 55 : 0 : 1 : 0

	f1-score	precision	recall	support
AFIB	0.877543	0.878166	0.876937	875.0
GSVT	0.889368	0.891122	0.887697	691.0
SB	0.944658	0.941519	0.947822	1553.0
SR	0.922461	0.925890	0.919088	907.0
macro avg	0.908508	0.909174	0.907886	4026.0
micro avg	0.915623	0.915623	0.915623	4026.0
weighted avg	0.915581	0.915579	0.915623	4026.0

The result of Model Number:Dataset Number:Featuregroup Number:Rescale of 55 : 0 : 1 : 1

	f1-score	precision	recall	support
AFIB	0.801669	0.807279	0.796164	204.0
GSVT	0.820320	0.816276	0.824417	300.0
SB	0.836624	0.835232	0.838032	892.0
SR	0.828298	0.830264	0.826351	681.0
macro avg	0.821728	0.822263	0.821241	2077.0
micro avg	0.828123	0.828123	0.828123	2077.0
weighted avg	0.828106	0.828120	0.828123	2077.0

The result of Model Number:Dataset Number:Featuregroup Number:Rescale of 55 : 0 : 2 : 0

	f1-score	precision	recall	support
AFIB	0.851210	0.882768	0.821951	204.0
GSVT	0.876710	0.865748	0.888065	300.0
SB	0.898942	0.890739	0.907305	892.0
SR	0.875308	0.882101	0.868628	681.0
macro avg	0.875543	0.880339	0.871487	2077.0
micro avg	0.883461	0.883461	0.883461	2077.0
weighted avg	0.883294	0.883515	0.883461	2077.0

The result of Model Number:Dataset Number:Featuregroup Number:Rescale of 55 : 0 : 2 : 1

	f1-score	precision	recall	support
AFIB	0.882768	0.904246	0.862503	204.0
GSVT	0.893864	0.881559	0.906637	300.0
SB	0.911913	0.902807	0.921212	892.0
SR	0.895992	0.907750	0.884560	681.0
macro avg	0.896134	0.899091	0.893728	2077.0
micro avg	0.901323	0.901323	0.901323	2077.0
weighted avg	0.901224	0.901500	0.901323	2077.0

The result of Model Number:Dataset Number:Featuregroup Number:Rescale of 55 : 0 : 3 : 0

	f1-score	precision	recall	support
AFIB	0.713046	0.747280	0.681954	204.0
GSVT	0.744061	0.737743	0.750509	300.0
SB	0.755754	0.750250	0.761344	892.0
SR	0.747804	0.748151	0.747480	681.0
macro avg	0.740166	0.745856	0.735322	2077.0
micro avg	0.747436	0.747436	0.747436	2077.0
weighted avg	0.747264	0.747464	0.747436	2077.0

The result of Model Number:Dataset Number:Featuregroup Number:Rescale of 55 : 0 : 3 : 1

	f1-score	precision	recall	support
AFIB	0.865682	0.887602	0.844898	204.0

GSVT 0.884482 0.874864 0.894461 300.0
SB 0.909395 0.904856 0.913982 892.0
SR 0.895299 0.899316 0.891354 681.0
macro avg 0.888714 0.891660 0.886174 2077.0
micro avg 0.896958 0.896958 0.896958 2077.0
weighted avg 0.896881 0.897013 0.896958 2077.0
The result of Model Number:Dataset Number:Featuregroup Number:Rescale of 55 : 0 : 4 : 0

	f1-score	precision	recall	support
AFIB	0.627910	0.684010	0.663086	204.0
GSVT	0.852876	0.838934	0.867583	300.0
SB	0.726061	0.720530	0.731730	892.0
SR	0.887854	0.877808	0.898159	681.0
macro avg	0.773675	0.780320	0.790140	2077.0
micro avg	0.799179	0.799179	0.799179	2077.0
weighted avg	0.787786	0.785613	0.799179	2077.0

The result of Model Number:Dataset Number:Featuregroup Number:Rescale of 55 : 0 : 4 : 1

	f1-score	precision	recall	support
AFIB	0.810483	0.800135	0.821149	875.0
GSVT	0.824709	0.829439	0.820055	691.0
SB	0.882800	0.879092	0.886548	1553.0
SR	0.856806	0.870675	0.843388	907.0
macro avg	0.843700	0.844835	0.842785	4026.0
micro avg	0.851199	0.851199	0.851199	4026.0
weighted avg	0.851256	0.851513	0.851199	4026.0

The result of Model Number:Dataset Number:Featuregroup Number:Rescale of 55 : 0 : 5 : 0

	f1-score	precision	recall	support
AFIB	0.744954	0.759229	0.731397	204.0
GSVT	0.768644	0.764685	0.772716	300.0
SB	0.787653	0.786426	0.788889	892.0
SR	0.779892	0.779117	0.780693	681.0
macro avg	0.770286	0.772364	0.768424	2077.0
micro avg	0.778219	0.778219	0.778219	2077.0
weighted avg	0.778169	0.778218	0.778219	2077.0

The result of Model Number:Dataset Number:Featuregroup Number:Rescale of 55 : 0 : 5 : 1

	f1-score	precision	recall	support
AFIB	0.769318	0.852416	0.701123	204.0
GSVT	0.891803	0.877299	0.906936	300.0
SB	0.951053	0.946723	0.955424	892.0
SR	0.928074	0.916264	0.940205	681.0
macro avg	0.885062	0.898176	0.875922	2077.0

micro avg	0.918454	0.918454	0.918454	2077.0
weighted avg	0.917111	0.917446	0.918454	2077.0

The result of Model Number:Dataset Number:Featuregroup Number:Rescale of 55 : 0 : 6 : 0

	f1-score	precision	recall	support
AFIB	0.773920	0.792317	0.756444	204.0
GSVT	0.794012	0.790948	0.797183	300.0
SB	0.813687	0.811871	0.815511	892.0
SR	0.803450	0.801834	0.805102	681.0
macro avg	0.796267	0.799242	0.793560	2077.0
micro avg	0.803649	0.803649	0.803649	2077.0
weighted avg	0.803583	0.803637	0.803649	2077.0

The result of Model Number:Dataset Number:Featuregroup Number:Rescale of 55 : 0 : 6 : 1

	f1-score	precision	recall	support
AFIB	0.878749	0.888311	0.869647	204.0
GSVT	0.908487	0.904698	0.912454	300.0
SB	0.927103	0.926274	0.927934	892.0
SR	0.918342	0.918350	0.918342	681.0
macro avg	0.908170	0.909408	0.907094	2077.0
micro avg	0.916828	0.916828	0.916828	2077.0
weighted avg	0.916792	0.916831	0.916828	2077.0

The result of Model Number:Dataset Number:Featuregroup Number:Rescale of 55 : 0 : 7 : 0

	f1-score	precision	recall	support
AFIB	0.811032	0.810301	0.811842	875.0
GSVT	0.825082	0.817528	0.832842	691.0
SB	0.865825	0.858213	0.873575	1553.0
SR	0.825063	0.845460	0.805699	907.0
macro avg	0.831751	0.832876	0.830990	4026.0
micro avg	0.837875	0.837875	0.837875	4026.0
weighted avg	0.837741	0.837944	0.837875	4026.0

The result of Model Number:Dataset Number:Featuregroup Number:Rescale of 55 : 0 : 7 : 1

	f1-score	precision	recall	support
AFIB	0.720417	0.700436	0.743839	875.0
GSVT	0.729822	0.760563	0.705960	691.0
SB	0.801735	0.801330	0.802151	1553.0
SR	0.781621	0.786513	0.776796	907.0
macro avg	0.758399	0.762210	0.757186	4026.0
micro avg	0.767256	0.767256	0.767256	4026.0
weighted avg	0.767187	0.769067	0.767256	4026.0

The result of Model Number:Dataset Number:Featuregroup Number:Rescale of 55 : 0 : 8 : 0

	f1-score	precision	recall	support
AFIB	0.847368	0.863931	0.831576	204.0
GSVT	0.870652	0.864379	0.877046	300.0
SB	0.886234	0.882785	0.889711	892.0
SR	0.876547	0.879027	0.874099	681.0
macro avg	0.870200	0.872531	0.868108	2077.0
micro avg	0.877053	0.877053	0.877053	2077.0
weighted avg	0.876990	0.877042	0.877053	2077.0

The result of Model Number:Dataset Number:Featuregroup Number:Rescale of 55 : 0 : 8 : 1

	f1-score	precision	recall	support
AFIB	0.699650	0.730356	0.671599	204.0
GSVT	0.651329	0.656018	0.646973	300.0
SB	0.675479	0.641559	0.713190	892.0
SR	0.599256	0.638822	0.564432	681.0
macro avg	0.656429	0.666689	0.649048	2077.0
micro avg	0.650766	0.650766	0.650766	2077.0
weighted avg	0.649373	0.651471	0.650766	2077.0

The result of Model Number:Dataset Number:Featuregroup Number:Rescale of 55 : 0 : 9 : 0

	f1-score	precision	recall	support
AFIB	0.803622	0.809036	0.798398	875.0
GSVT	0.817964	0.806602	0.829799	691.0
SB	0.871629	0.865661	0.877688	1553.0
SR	0.836307	0.850753	0.822389	907.0
macro avg	0.832380	0.833013	0.832069	4026.0
micro avg	0.839778	0.839778	0.839778	4026.0
weighted avg	0.839680	0.839859	0.839778	4026.0

The result of Model Number:Dataset Number:Featuregroup Number:Rescale of 55 : 0 : 9 : 1

	f1-score	precision	recall	support
AFIB	0.860727	0.888683	0.834645	204.0
GSVT	0.884490	0.873212	0.896241	300.0
SB	0.909444	0.906015	0.912909	892.0
SR	0.896724	0.898348	0.895141	681.0
macro avg	0.887846	0.891565	0.884734	2077.0
micro avg	0.896989	0.896989	0.896989	2077.0
weighted avg	0.896884	0.897061	0.896989	2077.0

The result of Model Number:Dataset Number:Featuregroup Number:Rescale of 55 : 0 : 10 : 0

	f1-score	precision	recall	support
AFIB	0.620684	0.616005	0.704467	204.0
GSVT	0.752456	0.748001	0.756973	300.0
SB	0.615404	0.614120	0.616698	892.0

SR 0.761111 0.759893 0.762343 681.0
macro avg 0.687414 0.684505 0.710120 2077.0
micro avg 0.693333 0.693333 0.693333 2077.0
weighted avg 0.683492 0.681438 0.693333 2077.0
The result of Model Number:Dataset Number:Featuregroup Number:Rescale of 55 : 0 : 10 : 1

	f1-score	precision	recall	support
AFIB	0.736281	0.815811	0.671014	204.0
GSVT	0.853506	0.839625	0.867989	300.0
SB	0.910212	0.906068	0.914395	892.0
SR	0.888219	0.876916	0.899830	681.0
macro avg	0.847054	0.859605	0.838307	2077.0
micro avg	0.879012	0.879012	0.879012	2077.0
weighted avg	0.877727	0.878048	0.879012	2077.0

The result of Model Number:Dataset Number:Featuregroup Number:Rescale of 55 : 1 : 0 : 0

	f1-score	precision	recall	support
AFIB	0.878065	0.891979	0.864694	204.0
GSVT	0.900634	0.892970	0.908436	300.0
SB	0.916184	0.914142	0.918238	892.0
SR	0.907503	0.909520	0.905512	681.0
macro avg	0.900596	0.902153	0.899220	2077.0
micro avg	0.907391	0.907391	0.907391	2077.0
weighted avg	0.907347	0.907392	0.907391	2077.0

The result of Model Number:Dataset Number:Featuregroup Number:Rescale of 55 : 1 : 0 : 1

	f1-score	precision	recall	support
AFIB	0.910732	0.921382	0.900496	204.0
GSVT	0.821485	0.790122	0.937303	300.0
SB	0.762170	0.760374	0.763982	892.0
SR	0.946358	0.948177	0.944553	681.0
macro avg	0.860186	0.855014	0.886584	2077.0
micro avg	0.861630	0.861630	0.861630	2077.0
weighted avg	0.845720	0.842061	0.861630	2077.0

The result of Model Number:Dataset Number:Featuregroup Number:Rescale of 55 : 1 : 1 : 0

	f1-score	precision	recall	support
AFIB	0.814429	0.816560	0.812470	204.0
GSVT	0.832805	0.829581	0.836124	300.0
SB	0.851482	0.850346	0.852626	892.0
SR	0.844663	0.847036	0.842306	681.0
macro avg	0.835845	0.835881	0.835881	2077.0
micro avg	0.842915	0.842915	0.842915	2077.0
weighted avg	0.842909	0.842943	0.842915	2077.0

The result of Model Number:Dataset Number:Featuregroup Number:Rescale of 55 : 1 : 1 : 1

	f1-score	precision	recall	support
AFIB	0.892569	0.912960	0.873295	204.0
GSVT	0.933117	0.927309	0.939022	300.0
SB	0.953270	0.950313	0.956259	892.0
SR	0.942353	0.942789	0.941943	681.0
macro avg	0.930327	0.933343	0.927630	2077.0
micro avg	0.940927	0.940927	0.940927	2077.0
weighted avg	0.940818	0.940855	0.940927	2077.0

The result of Model Number:Dataset Number:Featuregroup Number:Rescale of 55 : 1 : 2 : 0

	f1-score	precision	recall	support
AFIB	0.912789	0.927253	0.898890	204.0
GSVT	0.936251	0.928283	0.944361	300.0
SB	0.952415	0.950293	0.954551	892.0
SR	0.943391	0.945489	0.941322	681.0
macro avg	0.936212	0.937829	0.934781	2077.0
micro avg	0.943275	0.943275	0.943275	2077.0
weighted avg	0.943230	0.943276	0.943275	2077.0

The result of Model Number:Dataset Number:Featuregroup Number:Rescale of 55 : 1 : 2 : 1

	f1-score	precision	recall	support
AFIB	0.693278	0.714708	0.673700	204.0
GSVT	0.647166	0.661236	0.634161	300.0
SB	0.682597	0.652302	0.715877	892.0
SR	0.614604	0.646186	0.586052	681.0
macro avg	0.659411	0.668608	0.652447	2077.0
micro avg	0.657365	0.657365	0.657365	2077.0
weighted avg	0.656235	0.657716	0.657365	2077.0

The result of Model Number:Dataset Number:Featuregroup Number:Rescale of 55 : 1 : 3 : 0

	f1-score	precision	recall	support
AFIB	0.724519	0.715973	0.733300	875.0
GSVT	0.738756	0.745181	0.732447	691.0
SB	0.787331	0.784318	0.790371	1553.0
SR	0.768755	0.778049	0.759688	907.0
macro avg	0.754840	0.755880	0.753952	4026.0
micro avg	0.761113	0.761113	0.761113	4026.0
weighted avg	0.761158	0.761335	0.761113	4026.0

The result of Model Number:Dataset Number:Featuregroup Number:Rescale of 55 : 1 : 3 : 1

	f1-score	precision	recall	support
AFIB	0.927397	0.941616	0.913753	204.0

GSVT	0.949386	0.941332	0.957620	300.0
SB	0.965901	0.962784	0.969042	892.0
SR	0.955976	0.959525	0.952466	681.0
macro avg	0.949665	0.951314	0.948220	2077.0
micro avg	0.956527	0.956527	0.956527	2077.0
weighted avg	0.956480	0.956538	0.956527	2077.0

The result of Model Number:Dataset Number:Featuregroup Number:Rescale of 55 : 1 : 4 : 0

	f1-score	precision	recall	support
AFIB	0.798489	0.801061	0.796007	875.0
GSVT	0.809714	0.796859	0.823065	691.0
SB	0.853166	0.846042	0.860416	1553.0
SR	0.810280	0.830859	0.790725	907.0
macro avg	0.817912	0.818705	0.817553	4026.0
micro avg	0.824307	0.824307	0.824307	4026.0
weighted avg	0.824163	0.824404	0.824307	4026.0

The result of Model Number:Dataset Number:Featuregroup Number:Rescale of 55 : 1 : 4 : 1

	f1-score	precision	recall	support
AFIB	0.642986	0.636792	0.649402	875.0
GSVT	0.659833	0.661720	0.658025	691.0
SB	0.701516	0.699902	0.703141	1553.0
SR	0.681782	0.689804	0.673959	907.0
macro avg	0.671529	0.672055	0.671132	4026.0
micro avg	0.677144	0.677144	0.677144	4026.0
weighted avg	0.677195	0.677358	0.677144	4026.0

The result of Model Number:Dataset Number:Featuregroup Number:Rescale of 55 : 1 : 5 : 0

	f1-score	precision	recall	support
AFIB	0.724543	0.725759	0.742501	204.0
GSVT	0.717731	0.745584	0.692810	300.0
SB	0.761310	0.719559	0.808516	892.0
SR	0.659893	0.720792	0.610154	681.0
macro avg	0.715869	0.727923	0.713495	2077.0
micro avg	0.720281	0.720281	0.720281	2077.0
weighted avg	0.718152	0.724331	0.720281	2077.0

The result of Model Number:Dataset Number:Featuregroup Number:Rescale of 55 : 1 : 5 : 1

	f1-score	precision	recall	support
AFIB	0.693302	0.698334	0.688393	875.0
GSVT	0.708511	0.701925	0.715274	691.0
SB	0.761441	0.756844	0.766099	1553.0
SR	0.736486	0.744709	0.728445	907.0
macro avg	0.724935	0.725453	0.724553	4026.0

micro avg	0.732004	0.732004	0.732004	4026.0
weighted avg	0.731925	0.731968	0.732004	4026.0

The result of Model Number:Dataset Number:Featuregroup Number:Rescale of 55 : 1 : 6 : 0

	f1-score	precision	recall	support
AFIB	0.668476	0.713786	0.629019	204.0
GSVT	0.629862	0.648551	0.612458	300.0
SB	0.658363	0.626759	0.693700	892.0
SR	0.584176	0.610698	0.560499	681.0
macro avg	0.635220	0.649949	0.623919	2077.0
micro avg	0.631939	0.631939	0.631939	2077.0
weighted avg	0.630916	0.633188	0.631939	2077.0

The result of Model Number:Dataset Number:Featuregroup Number:Rescale of 55 : 1 : 6 : 1

	f1-score	precision	recall	support
AFIB	0.691215	0.673056	0.734486	875.0
GSVT	0.664942	0.629376	0.758020	691.0
SB	0.630067	0.626085	0.634119	1553.0
SR	0.612010	0.622555	0.601987	907.0
macro avg	0.649558	0.637768	0.682153	4026.0
micro avg	0.669959	0.669959	0.669959	4026.0
weighted avg	0.645274	0.636063	0.669959	4026.0

The result of Model Number:Dataset Number:Featuregroup Number:Rescale of 55 : 1 : 7 : 0

	f1-score	precision	recall	support
AFIB	0.645721	0.651532	0.640217	875.0
GSVT	0.658875	0.645515	0.672955	691.0
SB	0.698741	0.690968	0.706775	1553.0
SR	0.671014	0.690960	0.652345	907.0
macro avg	0.668588	0.669744	0.668073	4026.0
micro avg	0.674243	0.674243	0.674243	4026.0
weighted avg	0.674129	0.674594	0.674243	4026.0

The result of Model Number:Dataset Number:Featuregroup Number:Rescale of 55 : 1 : 7 : 1

	f1-score	precision	recall	support
AFIB	0.780945	0.780230	0.781755	875.0
GSVT	0.796952	0.795406	0.798588	691.0
SB	0.845670	0.843786	0.847575	1553.0
SR	0.822588	0.827954	0.817321	907.0
macro avg	0.811539	0.811844	0.811309	4026.0
micro avg	0.818046	0.818046	0.818046	4026.0
weighted avg	0.818041	0.818103	0.818046	4026.0

The result of Model Number:Dataset Number:Featuregroup Number:Rescale of 55 : 1 : 8 : 0

	f1-score	precision	recall	support
AFIB	0.600284	0.666193	0.546387	204.0
GSVT	0.700913	0.692150	0.710038	300.0
SB	0.745737	0.741932	0.749583	892.0
SR	0.728267	0.718086	0.738745	681.0
macro avg	0.693801	0.704590	0.686188	2077.0
micro avg	0.720360	0.720360	0.720360	2077.0
weighted avg	0.719249	0.719484	0.720360	2077.0

The result of Model Number:Dataset Number:Featuregroup Number:Rescale of 55 : 1 : 8 : 1

	f1-score	precision	recall	support
AFIB	0.728684	0.721853	0.735739	875.0
GSVT	0.690862	0.674257	0.741649	691.0
SB	0.798853	0.795920	0.801831	1553.0
SR	0.621333	0.625691	0.617045	907.0
macro avg	0.709933	0.704430	0.724066	4026.0
micro avg	0.735507	0.735507	0.735507	4026.0
weighted avg	0.725075	0.720590	0.735507	4026.0

The result of Model Number:Dataset Number:Featuregroup Number:Rescale of 55 : 1 : 9 : 0

	f1-score	precision	recall	support
AFIB	0.740746	0.744812	0.736772	875.0
GSVT	0.741782	0.732404	0.751520	691.0
SB	0.780112	0.776982	0.783275	1553.0
SR	0.763366	0.772633	0.754366	907.0
macro avg	0.756502	0.756708	0.756483	4026.0
micro avg	0.761205	0.761205	0.761205	4026.0
weighted avg	0.761205	0.761359	0.761205	4026.0

The result of Model Number:Dataset Number:Featuregroup Number:Rescale of 55 : 1 : 9 : 1

	f1-score	precision	recall	support
AFIB	0.674242	0.672373	0.676171	875.0
GSVT	0.679577	0.682819	0.676434	691.0
SB	0.711398	0.709125	0.713688	1553.0
SR	0.700025	0.703406	0.696708	907.0
macro avg	0.691310	0.691931	0.690751	4026.0
micro avg	0.695315	0.695315	0.695315	4026.0
weighted avg	0.695299	0.695334	0.695315	4026.0

The result of Model Number:Dataset Number:Featuregroup Number:Rescale of 55 : 1 : 10 : 0

	f1-score	precision	recall	support
AFIB	0.728997	0.724636	0.733509	875.0
GSVT	0.734967	0.738935	0.731147	691.0
SB	0.778955	0.776677	0.781257	1553.0

SR 0.761353 0.766822 0.755977 907.0
macro avg 0.751068 0.751768 0.750473 4026.0
micro avg 0.756584 0.756584 0.756584 4026.0
weighted avg 0.756582 0.756669 0.756584 4026.0
The result of Model Number:Dataset Number:Featuregroup Number:Rescale of 55 : 1 : 10 : 1

	f1-score	precision	recall	support
AFIB	0.853417	0.865955	0.841315	204.0
GSVT	0.875040	0.867601	0.882626	300.0
SB	0.890163	0.886602	0.893755	892.0
SR	0.881359	0.885685	0.877086	681.0
macro avg	0.874995	0.876461	0.873696	2077.0
micro avg	0.881532	0.881532	0.881532	2077.0
weighted avg	0.881483	0.881529	0.881532	2077.0

The result of Model Number:Dataset Number:Featuregroup Number:Rescale of 56 : 0 : 0 : 0

	f1-score	precision	recall	support
AFIB	0.791878	0.797800	0.786074	204.0
GSVT	0.812112	0.807826	0.816444	300.0
SB	0.825817	0.824713	0.826927	892.0
SR	0.818544	0.820113	0.816982	681.0
macro avg	0.812088	0.812613	0.811607	2077.0
micro avg	0.818140	0.818140	0.818140	2077.0
weighted avg	0.818119	0.818122	0.818140	2077.0

The result of Model Number:Dataset Number:Featuregroup Number:Rescale of 56 : 0 : 0 : 1

	f1-score	precision	recall	support
AFIB	0.635554	0.704039	0.579722	204.0
GSVT	0.749152	0.738651	0.760149	300.0
SB	0.795418	0.792857	0.798004	892.0
SR	0.775053	0.763265	0.787232	681.0
macro avg	0.738795	0.749703	0.731277	2077.0
micro avg	0.767565	0.767565	0.767565	2077.0
weighted avg	0.766357	0.766602	0.767565	2077.0

The result of Model Number:Dataset Number:Featuregroup Number:Rescale of 56 : 0 : 1 : 0

	f1-score	precision	recall	support
AFIB	0.655811	0.655639	0.656046	875.0
GSVT	0.669510	0.666979	0.672133	691.0
SB	0.712795	0.709577	0.716053	1553.0
SR	0.688772	0.696642	0.681105	907.0
macro avg	0.681722	0.682209	0.681334	4026.0
micro avg	0.687600	0.687600	0.687600	4026.0
weighted avg	0.687569	0.687629	0.687600	4026.0

The result of Model Number:Dataset Number:Featuregroup Number:Rescale of 56 : 0 : 1 : 1

	f1-score	precision	recall	support
AFIB	0.862883	0.871319	0.854812	875.0
GSVT	0.856293	0.849795	0.863171	691.0
SB	0.906055	0.896733	0.915653	1553.0
SR	0.877295	0.891404	0.863874	907.0
macro avg	0.875632	0.877313	0.874377	4026.0
micro avg	0.881757	0.881757	0.881757	4026.0
weighted avg	0.881652	0.881953	0.881757	4026.0

The result of Model Number:Dataset Number:Featuregroup Number:Rescale of 56 : 0 : 2 : 0

	f1-score	precision	recall	support
AFIB	0.697518	0.699373	0.695714	875.0
GSVT	0.710931	0.705923	0.716053	691.0
SB	0.751538	0.743875	0.759375	1553.0
SR	0.717184	0.732805	0.702240	907.0
macro avg	0.719292	0.720494	0.718345	4026.0
micro avg	0.725232	0.725232	0.725232	4026.0
weighted avg	0.725088	0.725195	0.725232	4026.0

The result of Model Number:Dataset Number:Featuregroup Number:Rescale of 56 : 0 : 2 : 1

	f1-score	precision	recall	support
AFIB	0.885598	0.886796	0.884572	875.0
GSVT	0.899392	0.898929	0.899888	691.0
SB	0.939321	0.935961	0.942710	1553.0
SR	0.916828	0.921967	0.911805	907.0
macro avg	0.910285	0.910913	0.909744	4026.0
micro avg	0.915762	0.915762	0.915762	4026.0
weighted avg	0.915725	0.915767	0.915762	4026.0

The result of Model Number:Dataset Number:Featuregroup Number:Rescale of 56 : 0 : 3 : 0

	f1-score	precision	recall	support
AFIB	0.857963	0.867994	0.848326	204.0
GSVT	0.881565	0.877512	0.885680	300.0
SB	0.894046	0.891266	0.896850	892.0
SR	0.886347	0.888859	0.883887	681.0
macro avg	0.879980	0.881408	0.878686	2077.0
micro avg	0.886220	0.886220	0.886220	2077.0
weighted avg	0.886175	0.886205	0.886220	2077.0

The result of Model Number:Dataset Number:Featuregroup Number:Rescale of 56 : 0 : 3 : 1

	f1-score	precision	recall	support
AFIB	0.829800	0.845879	0.814692	204.0

	f1-score	precision	recall	support
GSVT	0.861977	0.854770	0.869466	300.0
SB	0.878061	0.876299	0.879833	892.0
SR	0.869688	0.870589	0.868793	681.0
macro avg	0.859882	0.861884	0.858196	2077.0
micro avg	0.868318	0.868318	0.868318	2077.0
weighted avg	0.868253	0.868330	0.868318	2077.0

The result of Model Number:Dataset Number:Featuregroup Number:Rescale of 56 : 0 : 4 : 0

	f1-score	precision	recall	support
AFIB	0.715743	0.722345	0.709434	204.0
GSVT	0.741911	0.741937	0.741904	300.0
SB	0.757448	0.756103	0.758805	892.0
SR	0.749410	0.749209	0.749628	681.0
macro avg	0.741128	0.742398	0.739943	2077.0
micro avg	0.748506	0.748506	0.748506	2077.0
weighted avg	0.748472	0.748481	0.748506	2077.0

The result of Model Number:Dataset Number:Featuregroup Number:Rescale of 56 : 0 : 4 : 1

	f1-score	precision	recall	support
AFIB	0.884983	0.915932	0.856466	204.0
GSVT	0.922357	0.916735	0.928175	300.0
SB	0.941298	0.937430	0.945200	892.0
SR	0.932595	0.931235	0.933965	681.0
macro avg	0.920308	0.925333	0.915952	2077.0
micro avg	0.930342	0.930342	0.930342	2077.0
weighted avg	0.930177	0.930298	0.930342	2077.0

The result of Model Number:Dataset Number:Featuregroup Number:Rescale of 56 : 0 : 5 : 0

	f1-score	precision	recall	support
AFIB	0.712338	0.719622	0.705261	875.0
GSVT	0.712124	0.701123	0.723560	691.0
SB	0.751196	0.746710	0.755742	1553.0
SR	0.731313	0.741101	0.721802	907.0
macro avg	0.726743	0.727139	0.726591	4026.0
micro avg	0.731601	0.731601	0.731601	4026.0
weighted avg	0.731565	0.731735	0.731601	4026.0

The result of Model Number:Dataset Number:Featuregroup Number:Rescale of 56 : 0 : 5 : 1

	f1-score	precision	recall	support
AFIB	0.684615	0.684941	0.684388	875.0
GSVT	0.702345	0.696266	0.708556	691.0
SB	0.750107	0.744641	0.755661	1553.0
SR	0.718062	0.732336	0.704370	907.0
macro avg	0.713782	0.714546	0.713244	4026.0

micro avg 0.720531 0.720531 0.720531 4026.0
weighted avg 0.720456 0.720591 0.720531 4026.0
The result of Model Number:Dataset Number:Featuregroup Number:Rescale of 56 : 0 : 6 : 0

	f1-score	precision	recall	support
AFIB	0.744756	0.748158	0.741426	875.0
GSVT	0.757172	0.750768	0.763741	691.0
SB	0.800346	0.792173	0.808694	1553.0
SR	0.759040	0.775041	0.743727	907.0
macro avg	0.765329	0.766535	0.764397	4026.0
micro avg	0.771722	0.771722	0.771722	4026.0
weighted avg	0.771549	0.771641	0.771722	4026.0

The result of Model Number:Dataset Number:Featuregroup Number:Rescale of 56 : 0 : 6 : 1

	f1-score	precision	recall	support
AFIB	0.817754	0.812228	0.823468	875.0
GSVT	0.837705	0.838038	0.837479	691.0
SB	0.889891	0.887620	0.892183	1553.0
SR	0.865660	0.875332	0.856211	907.0
macro avg	0.852752	0.853304	0.852335	4026.0
micro avg	0.859756	0.859756	0.859756	4026.0
weighted avg	0.859797	0.859956	0.859756	4026.0

The result of Model Number:Dataset Number:Featuregroup Number:Rescale of 56 : 0 : 7 : 0

	f1-score	precision	recall	support
AFIB	0.724912	0.738251	0.712414	204.0
GSVT	0.751657	0.745362	0.758190	300.0
SB	0.765771	0.764488	0.767059	892.0
SR	0.759010	0.759684	0.758341	681.0
macro avg	0.750337	0.751946	0.749001	2077.0
micro avg	0.757552	0.757552	0.757552	2077.0
weighted avg	0.757502	0.757573	0.757552	2077.0

The result of Model Number:Dataset Number:Featuregroup Number:Rescale of 56 : 0 : 7 : 1

	f1-score	precision	recall	support
AFIB	0.658685	0.652580	0.664942	875.0
GSVT	0.670514	0.674156	0.666923	691.0
SB	0.710448	0.708810	0.712094	1553.0
SR	0.693895	0.700299	0.687630	907.0
macro avg	0.683386	0.683961	0.682897	4026.0
micro avg	0.688582	0.688582	0.688582	4026.0
weighted avg	0.688615	0.688724	0.688582	4026.0

The result of Model Number:Dataset Number:Featuregroup Number:Rescale of 56 : 0 : 8 : 0

	f1-score	precision	recall	support
AFIB	0.789224	0.779148	0.799610	875.0
GSVT	0.803077	0.807682	0.798545	691.0
SB	0.859644	0.856034	0.863293	1553.0
SR	0.834332	0.847837	0.821266	907.0
macro avg	0.821569	0.822675	0.820679	4026.0
micro avg	0.828871	0.828871	0.828871	4026.0
weighted avg	0.828928	0.829178	0.828871	4026.0

The result of Model Number:Dataset Number:Featuregroup Number:Rescale of 56 : 0 : 8 : 1

	f1-score	precision	recall	support
AFIB	0.756073	0.753887	0.758341	875.0
GSVT	0.770086	0.769460	0.770767	691.0
SB	0.817943	0.814556	0.821366	1553.0
SR	0.793388	0.802104	0.784902	907.0
macro avg	0.784373	0.785002	0.783844	4026.0
micro avg	0.790769	0.790769	0.790769	4026.0
weighted avg	0.790751	0.790825	0.790769	4026.0

The result of Model Number:Dataset Number:Featuregroup Number:Rescale of 56 : 0 : 9 : 0

	f1-score	precision	recall	support
AFIB	0.698039	0.711661	0.685051	204.0
GSVT	0.715437	0.706205	0.724961	300.0
SB	0.727614	0.720421	0.734956	892.0
SR	0.712405	0.722304	0.702780	681.0
macro avg	0.713374	0.715148	0.711937	2077.0
micro avg	0.718061	0.718061	0.718061	2077.0
weighted avg	0.717964	0.718124	0.718061	2077.0

The result of Model Number:Dataset Number:Featuregroup Number:Rescale of 56 : 0 : 9 : 1

	f1-score	precision	recall	support
AFIB	0.853909	0.856659	0.851255	875.0
GSVT	0.865914	0.852166	0.880190	691.0
SB	0.912381	0.904763	0.920135	1553.0
SR	0.866518	0.888526	0.845607	907.0
macro avg	0.874680	0.875528	0.874297	4026.0
micro avg	0.881519	0.881519	0.881519	4026.0
weighted avg	0.881365	0.881623	0.881519	4026.0

The result of Model Number:Dataset Number:Featuregroup Number:Rescale of 56 : 0 : 10 : 0

	f1-score	precision	recall	support
AFIB	0.754742	0.766998	0.742912	875.0
GSVT	0.802691	0.805206	0.800239	691.0
SB	0.873287	0.870278	0.876322	1553.0

SR 0.826748 0.817540 0.836217 907.0
macro avg 0.814367 0.815005 0.813922 4026.0
micro avg 0.825233 0.825233 0.825233 4026.0
weighted avg 0.824921 0.824782 0.825233 4026.0
The result of Model Number:Dataset Number:Featuregroup Number:Rescale of 56 : 0 : 10 : 1

	f1-score	precision	recall	support
AFIB	0.670190	0.726137	0.623583	204.0
GSVT	0.718856	0.709817	0.728421	300.0
SB	0.737207	0.726879	0.747919	892.0
SR	0.717098	0.720457	0.714119	681.0
macro avg	0.710838	0.720823	0.703511	2077.0
micro avg	0.721808	0.721808	0.721808	2077.0
weighted avg	0.721381	0.722236	0.721808	2077.0

The result of Model Number:Dataset Number:Featuregroup Number:Rescale of 56 : 1 : 0 : 0

	f1-score	precision	recall	support
AFIB	0.782660	0.783719	0.781754	875.0
GSVT	0.794850	0.794442	0.795289	691.0
SB	0.830139	0.827169	0.833133	1553.0
SR	0.810260	0.814801	0.805821	907.0
macro avg	0.804477	0.805033	0.803999	4026.0
micro avg	0.809318	0.809318	0.809318	4026.0
weighted avg	0.809285	0.809322	0.809318	4026.0

The result of Model Number:Dataset Number:Featuregroup Number:Rescale of 56 : 1 : 0 : 1

	f1-score	precision	recall	support
AFIB	0.770421	0.845949	0.708027	204.0
GSVT	0.903907	0.891724	0.916573	300.0
SB	0.959667	0.956466	0.962894	892.0
SR	0.934727	0.922194	0.947628	681.0
macro avg	0.892180	0.904083	0.883780	2077.0
micro avg	0.926165	0.926165	0.926165	2077.0
weighted avg	0.924848	0.925023	0.926165	2077.0

The result of Model Number:Dataset Number:Featuregroup Number:Rescale of 56 : 1 : 1 : 0

	f1-score	precision	recall	support
AFIB	0.840972	0.821372	0.861667	875.0
GSVT	0.807304	0.816178	0.798675	691.0
SB	0.849558	0.794699	0.912573	1553.0
SR	0.679939	0.816475	0.582888	907.0
macro avg	0.794443	0.812181	0.788951	4026.0
micro avg	0.807687	0.807687	0.807687	4026.0
weighted avg	0.802227	0.809088	0.807687	4026.0

The result of Model Number:Dataset Number:Featuregroup Number:Rescale of 56 : 1 : 1 : 1

	f1-score	precision	recall	support
AFIB	0.883051	0.899251	0.867492	204.0
GSVT	0.894697	0.885668	0.903931	300.0
SB	0.916736	0.911964	0.921564	892.0
SR	0.904256	0.909922	0.898679	681.0
macro avg	0.899685	0.901701	0.897917	2077.0
micro avg	0.906203	0.906203	0.906203	2077.0
weighted avg	0.906152	0.906248	0.906203	2077.0

The result of Model Number:Dataset Number:Featuregroup Number:Rescale of 56 : 1 : 2 : 0

	f1-score	precision	recall	support
AFIB	0.784910	0.773091	0.803630	875.0
GSVT	0.749447	0.747266	0.755840	691.0
SB	0.800313	0.745764	0.870127	1553.0
SR	0.592841	0.792453	0.491240	907.0
macro avg	0.731878	0.764643	0.730209	4026.0
micro avg	0.750701	0.750701	0.750701	4026.0
weighted avg	0.741494	0.762479	0.750701	4026.0

The result of Model Number:Dataset Number:Featuregroup Number:Rescale of 56 : 1 : 2 : 1

	f1-score	precision	recall	support
AFIB	0.701139	0.710504	0.692214	204.0
GSVT	0.713525	0.709178	0.718034	300.0
SB	0.730724	0.730076	0.731378	892.0
SR	0.725325	0.725435	0.725219	681.0
macro avg	0.717678	0.718798	0.716712	2077.0
micro avg	0.723585	0.723585	0.723585	2077.0
weighted avg	0.723564	0.723614	0.723585	2077.0

The result of Model Number:Dataset Number:Featuregroup Number:Rescale of 56 : 1 : 3 : 0

	f1-score	precision	recall	support
AFIB	0.754431	0.742598	0.766728	875.0
GSVT	0.726943	0.747783	0.707318	691.0
SB	0.763897	0.725174	0.807032	1553.0
SR	0.620662	0.690670	0.563636	907.0
macro avg	0.716483	0.726557	0.711178	4026.0
micro avg	0.726324	0.726324	0.726324	4026.0
weighted avg	0.723228	0.725068	0.726324	4026.0

The result of Model Number:Dataset Number:Featuregroup Number:Rescale of 56 : 1 : 3 : 1

	f1-score	precision	recall	support
AFIB	0.685569	0.684941	0.686280	875.0

GSVT	0.699621	0.698264	0.701057	691.0
SB	0.742389	0.740735	0.744061	1553.0
SR	0.722126	0.726837	0.717502	907.0
macro avg	0.712426	0.712694	0.712225	4026.0
micro avg	0.718139	0.718139	0.718139	4026.0
weighted avg	0.718134	0.718189	0.718139	4026.0

The result of Model Number:Dataset Number:Featuregroup Number:Rescale of 56 : 1 : 4 : 0

	f1-score	precision	recall	support
AFIB	0.795191	0.784011	0.806733	875.0
GSVT	0.812312	0.818407	0.806328	691.0
SB	0.863801	0.861262	0.866358	1553.0
SR	0.842523	0.854179	0.831195	907.0
macro avg	0.828457	0.829465	0.827654	4026.0
micro avg	0.835174	0.835174	0.835174	4026.0
weighted avg	0.835258	0.835521	0.835174	4026.0

The result of Model Number:Dataset Number:Featuregroup Number:Rescale of 56 : 1 : 4 : 1

	f1-score	precision	recall	support
AFIB	0.729877	0.724441	0.735468	875.0
GSVT	0.743489	0.746144	0.740898	691.0
SB	0.788933	0.787064	0.790812	1553.0
SR	0.770929	0.777870	0.764139	907.0
macro avg	0.758307	0.758880	0.757829	4026.0
micro avg	0.764208	0.764208	0.764208	4026.0
weighted avg	0.764242	0.764359	0.764208	4026.0

The result of Model Number:Dataset Number:Featuregroup Number:Rescale of 56 : 1 : 5 : 0

	f1-score	precision	recall	support
AFIB	0.591288	0.596185	0.666005	204.0
GSVT	0.641212	0.613767	0.723437	300.0
SB	0.585450	0.583416	0.587501	892.0
SR	0.579629	0.579739	0.579524	681.0
macro avg	0.599395	0.593277	0.639117	2077.0
micro avg	0.612230	0.612230	0.612230	2077.0
weighted avg	0.592169	0.587848	0.612230	2077.0

The result of Model Number:Dataset Number:Featuregroup Number:Rescale of 56 : 1 : 5 : 1

	f1-score	precision	recall	support
AFIB	0.813540	0.811962	0.815228	875.0
GSVT	0.828816	0.814315	0.843923	691.0
SB	0.887896	0.875298	0.900871	1553.0
SR	0.841072	0.878566	0.806683	907.0
macro avg	0.842831	0.845035	0.841677	4026.0

micro avg	0.851264	0.851264	0.851264	4026.0
weighted avg	0.851047	0.851802	0.851264	4026.0

The result of Model Number:Dataset Number:Featuregroup Number:Rescale of 56 : 1 : 6 : 0

	f1-score	precision	recall	support
AFIB	0.906010	0.907826	0.904241	875.0
GSVT	0.908254	0.903351	0.913235	691.0
SB	0.949431	0.944358	0.954564	1553.0
SR	0.926717	0.937707	0.916008	907.0
macro avg	0.922603	0.923311	0.922012	4026.0
micro avg	0.927847	0.927847	0.927847	4026.0
weighted avg	0.927809	0.927882	0.927847	4026.0

The result of Model Number:Dataset Number:Featuregroup Number:Rescale of 56 : 1 : 6 : 1

	f1-score	precision	recall	support
AFIB	0.640382	0.665267	0.617339	875.0
GSVT	0.688432	0.683348	0.693683	691.0
SB	0.756534	0.752636	0.760477	1553.0
SR	0.711967	0.698391	0.726159	907.0
macro avg	0.699329	0.699910	0.699415	4026.0
micro avg	0.710172	0.710172	0.710172	4026.0
weighted avg	0.709561	0.709535	0.710172	4026.0

The result of Model Number:Dataset Number:Featuregroup Number:Rescale of 56 : 1 : 7 : 0

	f1-score	precision	recall	support
AFIB	0.701652	0.696791	0.706722	875.0
GSVT	0.712476	0.715368	0.709724	691.0
SB	0.773038	0.765000	0.781251	1553.0
SR	0.734007	0.751086	0.717754	907.0
macro avg	0.730293	0.732061	0.728863	4026.0
micro avg	0.738472	0.738472	0.738472	4026.0
weighted avg	0.738336	0.738523	0.738472	4026.0

The result of Model Number:Dataset Number:Featuregroup Number:Rescale of 56 : 1 : 7 : 1

	f1-score	precision	recall	support
AFIB	0.818512	0.808607	0.828699	875.0
GSVT	0.829591	0.838097	0.821321	691.0
SB	0.881110	0.878243	0.884003	1553.0
SR	0.861838	0.870694	0.853191	907.0
macro avg	0.847763	0.848910	0.846803	4026.0
micro avg	0.854283	0.854283	0.854283	4026.0
weighted avg	0.854321	0.854517	0.854283	4026.0

The result of Model Number:Dataset Number:Featuregroup Number:Rescale of 56 : 1 : 8 : 0

	f1-score	precision	recall	support
AFIB	0.781083	0.812609	0.751974	204.0
GSVT	0.810150	0.796989	0.823892	300.0
SB	0.831109	0.824427	0.837910	892.0
SR	0.808749	0.814573	0.803050	681.0
macro avg	0.807772	0.812149	0.804206	2077.0
micro avg	0.816015	0.816015	0.816015	2077.0
weighted avg	0.815837	0.816072	0.816015	2077.0

The result of Model Number:Dataset Number:Featuregroup Number:Rescale of 56 : 1 : 8 : 1

	f1-score	precision	recall	support
AFIB	0.858807	0.852194	0.865588	875.0
GSVT	0.869957	0.873996	0.866145	691.0
SB	0.932280	0.927321	0.937313	1553.0
SR	0.904812	0.917533	0.892538	907.0
macro avg	0.891464	0.892761	0.890396	4026.0
micro avg	0.899423	0.899423	0.899423	4026.0
weighted avg	0.899427	0.899636	0.899423	4026.0

The result of Model Number:Dataset Number:Featuregroup Number:Rescale of 56 : 1 : 9 : 0

	f1-score	precision	recall	support
AFIB	0.797573	0.802121	0.793207	204.0
GSVT	0.813983	0.808112	0.819948	300.0
SB	0.830423	0.827390	0.833493	892.0
SR	0.821591	0.826986	0.816287	681.0
macro avg	0.815892	0.816152	0.815734	2077.0
micro avg	0.821938	0.821938	0.821938	2077.0
weighted avg	0.821926	0.821991	0.821938	2077.0

The result of Model Number:Dataset Number:Featuregroup Number:Rescale of 56 : 1 : 9 : 1

	f1-score	precision	recall	support
AFIB	0.763113	0.773306	0.753400	204.0
GSVT	0.776594	0.771863	0.781502	300.0
SB	0.795314	0.794609	0.796025	892.0
SR	0.789437	0.789557	0.789322	681.0
macro avg	0.781114	0.782334	0.780062	2077.0
micro avg	0.787543	0.787543	0.787543	2077.0
weighted avg	0.787520	0.787575	0.787543	2077.0

The result of Model Number:Dataset Number:Featuregroup Number:Rescale of 56 : 1 : 10 : 0

	f1-score	precision	recall	support
AFIB	0.735172	0.737217	0.733177	875.0
GSVT	0.752013	0.751804	0.752229	691.0
SB	0.800917	0.793389	0.808598	1553.0

SR 0.779695 0.790939 0.768776 907.0
macro avg 0.766949 0.768338 0.765695 4026.0
micro avg 0.773560 0.773560 0.773560 4026.0
weighted avg 0.773454 0.773492 0.773560 4026.0
The result of Model Number:Dataset Number:Featuregroup Number:Rescale of 56 : 1 : 10 : 1
f1-score precision recall support
AFIB 0.852737 0.851717 0.853904 875.0
GSVT 0.869360 0.872350 0.866480 691.0
SB 0.920782 0.915109 0.926532 1553.0
SR 0.902044 0.910854 0.893502 907.0
macro avg 0.886231 0.887508 0.885105 4026.0
micro avg 0.892999 0.892999 0.892999 4026.0
weighted avg 0.892946 0.893034 0.892999 4026.0
The result of Model Number:Dataset Number:Featuregroup Number:Rescale of 57 : 0 : 0 : 0
f1-score precision recall support
AFIB 0.670696 0.684150 0.657893 204.0
GSVT 0.686239 0.679106 0.693560 300.0
SB 0.698931 0.696212 0.701673 892.0
SR 0.691586 0.694472 0.688741 681.0
macro avg 0.686863 0.688485 0.685467 2077.0
micro avg 0.691961 0.691961 0.691961 2077.0
weighted avg 0.691916 0.691986 0.691961 2077.0
The result of Model Number:Dataset Number:Featuregroup Number:Rescale of 57 : 0 : 0 : 1
f1-score precision recall support
AFIB 0.843325 0.913725 0.784678 204.0
GSVT 0.904563 0.893189 0.916600 300.0
SB 0.927656 0.914660 0.941134 892.0
SR 0.902351 0.906578 0.898603 681.0
macro avg 0.894474 0.907038 0.885254 2077.0
micro avg 0.908278 0.908278 0.908278 2077.0
weighted avg 0.907741 0.908817 0.908278 2077.0
The result of Model Number:Dataset Number:Featuregroup Number:Rescale of 57 : 0 : 1 : 0
f1-score precision recall support
AFIB 0.880669 0.898412 0.864178 204.0
GSVT 0.908927 0.901627 0.916521 300.0
SB 0.928604 0.927669 0.929541 892.0
SR 0.920140 0.919612 0.920679 681.0
macro avg 0.909585 0.911830 0.907730 2077.0
micro avg 0.918335 0.918335 0.918335 2077.0
weighted avg 0.918279 0.918392 0.918335 2077.0

The result of Model Number:Dataset Number:Featuregroup Number:Rescale of 57 : 0 : 1 : 1

	f1-score	precision	recall	support
AFIB	0.728442	0.717554	0.739692	875.0
GSVT	0.742408	0.748758	0.736178	691.0
SB	0.793246	0.790515	0.796002	1553.0
SR	0.770028	0.781513	0.758902	907.0
macro avg	0.758531	0.759585	0.757693	4026.0
micro avg	0.765138	0.765138	0.765138	4026.0
weighted avg	0.765206	0.765463	0.765138	4026.0

The result of Model Number:Dataset Number:Featuregroup Number:Rescale of 57 : 0 : 2 : 0

	f1-score	precision	recall	support
AFIB	0.722537	0.727696	0.717580	204.0
GSVT	0.747802	0.747320	0.748299	300.0
SB	0.762764	0.761072	0.764472	892.0
SR	0.755946	0.756860	0.755057	681.0
macro avg	0.747262	0.748237	0.746352	2077.0
micro avg	0.754443	0.754443	0.754443	2077.0
weighted avg	0.754416	0.754427	0.754443	2077.0

The result of Model Number:Dataset Number:Featuregroup Number:Rescale of 57 : 0 : 2 : 1

	f1-score	precision	recall	support
AFIB	0.835619	0.826289	0.845171	875.0
GSVT	0.852244	0.860050	0.844592	691.0
SB	0.908957	0.905997	0.911940	1553.0
SR	0.885394	0.894263	0.876701	907.0
macro avg	0.870553	0.871650	0.869601	4026.0
micro avg	0.877931	0.877931	0.877931	4026.0
weighted avg	0.877976	0.878144	0.877931	4026.0

The result of Model Number:Dataset Number:Featuregroup Number:Rescale of 57 : 0 : 3 : 0

	f1-score	precision	recall	support
AFIB	0.652244	0.657414	0.647171	875.0
GSVT	0.660513	0.657601	0.663473	691.0
SB	0.702378	0.699601	0.705182	1553.0
SR	0.685118	0.687054	0.683226	907.0
macro avg	0.675063	0.675417	0.674763	4026.0
micro avg	0.680469	0.680469	0.680469	4026.0
weighted avg	0.680408	0.680397	0.680469	4026.0

The result of Model Number:Dataset Number:Featuregroup Number:Rescale of 57 : 0 : 3 : 1

	f1-score	precision	recall	support
AFIB	0.638481	0.628259	0.718420	204.0

GSVT 0.747492 0.738819 0.756644 300.0
SB 0.772521 0.769604 0.775465 892.0
SR 0.606134 0.609268 0.603135 681.0
macro avg 0.691157 0.686487 0.713416 2077.0
micro avg 0.710641 0.710641 0.710641 2077.0
weighted avg 0.701186 0.698704 0.710641 2077.0
The result of Model Number:Dataset Number:Featuregroup Number:Rescale of 57 : 0 : 4 : 0

	f1-score	precision	recall	support
AFIB	0.726108	0.723155	0.729103	875.0
GSVT	0.736722	0.741175	0.732350	691.0
SB	0.777012	0.776066	0.777962	1553.0
SR	0.760802	0.762007	0.759623	907.0
macro avg	0.750161	0.750601	0.749759	4026.0
micro avg	0.755383	0.755383	0.755383	4026.0
weighted avg	0.755381	0.755411	0.755383	4026.0

The result of Model Number:Dataset Number:Featuregroup Number:Rescale of 57 : 0 : 4 : 1

	f1-score	precision	recall	support
AFIB	0.678178	0.684784	0.671863	204.0
GSVT	0.693861	0.690675	0.697100	300.0
SB	0.703449	0.701029	0.705892	892.0
SR	0.697509	0.700215	0.694857	681.0
macro avg	0.693249	0.694176	0.692428	2077.0
micro avg	0.697661	0.697661	0.697661	2077.0
weighted avg	0.697635	0.697671	0.697661	2077.0

The result of Model Number:Dataset Number:Featuregroup Number:Rescale of 57 : 0 : 5 : 0

	f1-score	precision	recall	support
AFIB	0.705343	0.719992	0.691440	875.0
GSVT	0.687849	0.663959	0.743998	691.0
SB	0.810738	0.807082	0.814441	1553.0
SR	0.616361	0.606410	0.626700	907.0
macro avg	0.705073	0.699361	0.719144	4026.0
micro avg	0.733322	0.733322	0.733322	4026.0
weighted avg	0.722949	0.718381	0.733322	4026.0

The result of Model Number:Dataset Number:Featuregroup Number:Rescale of 57 : 0 : 5 : 1

	f1-score	precision	recall	support
AFIB	0.844379	0.871444	0.819196	204.0
GSVT	0.869640	0.854679	0.885263	300.0
SB	0.884684	0.875938	0.893608	892.0
SR	0.861029	0.871803	0.850536	681.0
macro avg	0.864933	0.868466	0.862151	2077.0

micro avg 0.870972 0.870972 0.870972 2077.0
weighted avg 0.870796 0.871070 0.870972 2077.0
The result of Model Number:Dataset Number:Featuregroup Number:Rescale of 57 : 0 : 6 : 0

	f1-score	precision	recall	support
AFIB	0.643601	0.625058	0.706154	875.0
GSVT	0.709335	0.707069	0.711706	691.0
SB	0.598783	0.589958	0.607924	1553.0
SR	0.716971	0.730965	0.703593	907.0
macro avg	0.667172	0.663263	0.682344	4026.0
micro avg	0.668639	0.668639	0.668639	4026.0
weighted avg	0.654124	0.649454	0.668639	4026.0

The result of Model Number:Dataset Number:Featuregroup Number:Rescale of 57 : 0 : 6 : 1

	f1-score	precision	recall	support
AFIB	0.563633	0.622586	0.515043	204.0
GSVT	0.663075	0.654320	0.672138	300.0
SB	0.703188	0.700455	0.705947	892.0
SR	0.686291	0.676510	0.696366	681.0
macro avg	0.654047	0.663468	0.647373	2077.0
micro avg	0.679172	0.679172	0.679172	2077.0
weighted avg	0.678147	0.678292	0.679172	2077.0

The result of Model Number:Dataset Number:Featuregroup Number:Rescale of 57 : 0 : 7 : 0

	f1-score	precision	recall	support
AFIB	0.702869	0.708426	0.697423	875.0
GSVT	0.720556	0.719032	0.722112	691.0
SB	0.768133	0.761344	0.775055	1553.0
SR	0.747685	0.755018	0.740511	907.0
macro avg	0.734811	0.735955	0.733775	4026.0
micro avg	0.741313	0.741313	0.741313	4026.0
weighted avg	0.741176	0.741155	0.741313	4026.0

The result of Model Number:Dataset Number:Featuregroup Number:Rescale of 57 : 0 : 7 : 1

	f1-score	precision	recall	support
AFIB	0.647154	0.637232	0.657499	875.0
GSVT	0.669469	0.676031	0.663165	691.0
SB	0.712494	0.710830	0.714189	1553.0
SR	0.694695	0.703504	0.686120	907.0
macro avg	0.680953	0.681899	0.680243	4026.0
micro avg	0.686787	0.686787	0.686787	4026.0
weighted avg	0.686899	0.687211	0.686787	4026.0

The result of Model Number:Dataset Number:Featuregroup Number:Rescale of 57 : 0 : 8 : 0

	f1-score	precision	recall	support
AFIB	0.887143	0.893352	0.881052	204.0
GSVT	0.907784	0.903308	0.912318	300.0
SB	0.925826	0.924285	0.927383	892.0
SR	0.916612	0.918788	0.914457	681.0
macro avg	0.909341	0.909934	0.908803	2077.0
micro avg	0.916419	0.916419	0.916419	2077.0
weighted avg	0.916400	0.916415	0.916419	2077.0

The result of Model Number:Dataset Number:Featuregroup Number:Rescale of 57 : 0 : 8 : 1

	f1-score	precision	recall	support
AFIB	0.627159	0.583211	0.736480	875.0
GSVT	0.537870	0.502177	0.608385	691.0
SB	0.642720	0.637297	0.648251	1553.0
SR	0.619489	0.634505	0.605383	907.0
macro avg	0.606810	0.589298	0.649625	4026.0
micro avg	0.650926	0.650926	0.650926	4026.0
weighted avg	0.616109	0.601722	0.650926	4026.0

The result of Model Number:Dataset Number:Featuregroup Number:Rescale of 57 : 0 : 9 : 0

	f1-score	precision	recall	support
AFIB	0.902200	0.899279	0.905168	875.0
GSVT	0.912662	0.915497	0.909934	691.0
SB	0.963006	0.960910	0.965115	1553.0
SR	0.945247	0.949707	0.940865	907.0
macro avg	0.930779	0.931348	0.930270	4026.0
micro avg	0.937152	0.937152	0.937152	4026.0
weighted avg	0.937149	0.937197	0.937152	4026.0

The result of Model Number:Dataset Number:Featuregroup Number:Rescale of 57 : 0 : 9 : 1

	f1-score	precision	recall	support
AFIB	0.706034	0.697797	0.716309	875.0
GSVT	0.726467	0.717661	0.736076	691.0
SB	0.737416	0.708952	0.779169	1553.0
SR	0.585547	0.616434	0.557642	907.0
macro avg	0.688866	0.685211	0.697299	4026.0
micro avg	0.708204	0.708204	0.708204	4026.0
weighted avg	0.694503	0.687179	0.708204	4026.0

The result of Model Number:Dataset Number:Featuregroup Number:Rescale of 57 : 0 : 10 : 0

	f1-score	precision	recall	support
AFIB	0.700227	0.701833	0.698703	875.0
GSVT	0.702668	0.700687	0.704683	691.0
SB	0.737439	0.731332	0.743658	1553.0

SR 0.715316 0.726051 0.704924 907.0
macro avg 0.713912 0.714976 0.712992 4026.0
micro avg 0.718472 0.718472 0.718472 4026.0
weighted avg 0.718399 0.718471 0.718472 4026.0
The result of Model Number:Dataset Number:Featuregroup Number:Rescale of 57 : 0 : 10 : 1

	f1-score	precision	recall	support
AFIB	0.898188	0.915745	0.881449	204.0
GSVT	0.922868	0.916219	0.929646	300.0
SB	0.939385	0.935729	0.943071	892.0
SR	0.929117	0.931746	0.926522	681.0
macro avg	0.922389	0.924860	0.920172	2077.0
micro avg	0.929654	0.929654	0.929654	2077.0
weighted avg	0.929586	0.929642	0.929654	2077.0

The result of Model Number:Dataset Number:Featuregroup Number:Rescale of 57 : 1 : 0 : 0

	f1-score	precision	recall	support
AFIB	0.662174	0.678064	0.647313	204.0
GSVT	0.685719	0.681745	0.689823	300.0
SB	0.700364	0.697795	0.702954	892.0
SR	0.694278	0.694793	0.693771	681.0
macro avg	0.685634	0.688099	0.683465	2077.0
micro avg	0.692582	0.692582	0.692582	2077.0
weighted avg	0.692502	0.692554	0.692582	2077.0

The result of Model Number:Dataset Number:Featuregroup Number:Rescale of 57 : 1 : 0 : 1

	f1-score	precision	recall	support
AFIB	0.765011	0.770514	0.759588	875.0
GSVT	0.763792	0.759219	0.768433	691.0
SB	0.805689	0.796762	0.814820	1553.0
SR	0.773121	0.786914	0.759828	907.0
macro avg	0.776903	0.778352	0.775667	4026.0
micro avg	0.782465	0.782465	0.782465	4026.0
weighted avg	0.782320	0.782395	0.782465	4026.0

The result of Model Number:Dataset Number:Featuregroup Number:Rescale of 57 : 1 : 1 : 0

	f1-score	precision	recall	support
AFIB	0.803520	0.786258	0.821600	875.0
GSVT	0.832129	0.844197	0.820457	691.0
SB	0.885834	0.885666	0.886007	1553.0
SR	0.863633	0.873671	0.853827	907.0
macro avg	0.846279	0.847448	0.845473	4026.0
micro avg	0.853508	0.853508	0.853508	4026.0
weighted avg	0.853725	0.854241	0.853508	4026.0

The result of Model Number:Dataset Number:Featuregroup Number:Rescale of 57 : 1 : 1 : 1

	f1-score	precision	recall	support
AFIB	0.901187	0.939811	0.865955	204.0
GSVT	0.931470	0.917397	0.946042	300.0
SB	0.949945	0.940551	0.959532	892.0
SR	0.924209	0.932116	0.916470	681.0
macro avg	0.926703	0.932468	0.922000	2077.0
micro avg	0.934273	0.934273	0.934273	2077.0
weighted avg	0.934049	0.934368	0.934273	2077.0

The result of Model Number:Dataset Number:Featuregroup Number:Rescale of 57 : 1 : 2 : 0

	f1-score	precision	recall	support
AFIB	0.886128	0.889177	0.883212	875.0
GSVT	0.902951	0.884936	0.921878	691.0
SB	0.955846	0.950677	0.961077	1553.0
SR	0.918876	0.940240	0.898515	907.0
macro avg	0.915950	0.916258	0.916170	4026.0
micro avg	0.923332	0.923332	0.923332	4026.0
weighted avg	0.923286	0.923676	0.923332	4026.0

The result of Model Number:Dataset Number:Featuregroup Number:Rescale of 57 : 1 : 2 : 1

	f1-score	precision	recall	support
AFIB	0.775078	0.809526	0.743550	204.0
GSVT	0.807377	0.801039	0.813835	300.0
SB	0.819710	0.813387	0.826140	892.0
SR	0.810641	0.811980	0.809336	681.0
macro avg	0.803202	0.808983	0.798215	2077.0
micro avg	0.810741	0.810741	0.810741	2077.0
weighted avg	0.810572	0.810763	0.810741	2077.0

The result of Model Number:Dataset Number:Featuregroup Number:Rescale of 57 : 1 : 3 : 0

	f1-score	precision	recall	support
AFIB	0.778409	0.785413	0.771773	875.0
GSVT	0.794266	0.778161	0.811239	691.0
SB	0.842324	0.832953	0.852009	1553.0
SR	0.808899	0.832944	0.786394	907.0
macro avg	0.805975	0.807368	0.805354	4026.0
micro avg	0.812791	0.812791	0.812791	4026.0
weighted avg	0.812654	0.813215	0.812791	4026.0

The result of Model Number:Dataset Number:Featuregroup Number:Rescale of 57 : 1 : 3 : 1

	f1-score	precision	recall	support
AFIB	0.704917	0.759859	0.657712	204.0

GSVT 0.726413 0.738710 0.714550 300.0
SB 0.769861 0.762776 0.777103 892.0
SR 0.739220 0.728501 0.750294 681.0
macro avg 0.735103 0.747461 0.724915 2077.0
micro avg 0.747552 0.747552 0.747552 2077.0
weighted avg 0.747161 0.747775 0.747552 2077.0
The result of Model Number:Dataset Number:Featuregroup Number:Rescale of 57 : 1 : 4 : 0

	f1-score	precision	recall	support
AFIB	0.815272	0.851506	0.782108	204.0
GSVT	0.849245	0.842579	0.856038	300.0
SB	0.862218	0.855567	0.868982	892.0
SR	0.852679	0.854087	0.851306	681.0
macro avg	0.844854	0.850935	0.839608	2077.0
micro avg	0.852784	0.852784	0.852784	2077.0
weighted avg	0.852606	0.852807	0.852784	2077.0

The result of Model Number:Dataset Number:Featuregroup Number:Rescale of 57 : 1 : 4 : 1

	f1-score	precision	recall	support
AFIB	0.743308	0.743070	0.743584	875.0
GSVT	0.754385	0.744688	0.764426	691.0
SB	0.797944	0.796208	0.799690	1553.0
SR	0.775866	0.787239	0.764829	907.0
macro avg	0.767876	0.767801	0.768133	4026.0
micro avg	0.773590	0.773590	0.773590	4026.0
weighted avg	0.773619	0.773796	0.773590	4026.0

The result of Model Number:Dataset Number:Featuregroup Number:Rescale of 57 : 1 : 5 : 0

	f1-score	precision	recall	support
AFIB	0.814678	0.834499	0.795976	204.0
GSVT	0.824918	0.813562	0.836706	300.0
SB	0.841575	0.833171	0.850157	892.0
SR	0.826882	0.837733	0.816332	681.0
macro avg	0.827013	0.829741	0.824793	2077.0
micro avg	0.831802	0.831802	0.831802	2077.0
weighted avg	0.831710	0.831965	0.831802	2077.0

The result of Model Number:Dataset Number:Featuregroup Number:Rescale of 57 : 1 : 5 : 1

	f1-score	precision	recall	support
AFIB	0.643015	0.635016	0.651290	875.0
GSVT	0.663147	0.671451	0.655112	691.0
SB	0.707485	0.706719	0.708259	1553.0
SR	0.688404	0.691934	0.684913	907.0
macro avg	0.675513	0.676280	0.674893	4026.0

	f1-score	precision	recall	support
micro avg	0.681496	0.681496	0.681496	4026.0
weighted avg	0.681565	0.681751	0.681496	4026.0

The result of Model Number:Dataset Number:Featuregroup Number:Rescale of 57 : 1 : 6 : 0

	f1-score	precision	recall	support
AFIB	0.825796	0.845244	0.807295	875.0
GSVT	0.870512	0.872710	0.868382	691.0
SB	0.946697	0.942773	0.950658	1553.0
SR	0.900789	0.886602	0.915479	907.0
macro avg	0.885949	0.886832	0.885453	4026.0
micro avg	0.897453	0.897453	0.897453	4026.0
weighted avg	0.897002	0.896897	0.897453	4026.0

The result of Model Number:Dataset Number:Featuregroup Number:Rescale of 57 : 1 : 6 : 1

	f1-score	precision	recall	support
AFIB	0.891026	0.903978	0.878843	204.0
GSVT	0.910676	0.898356	0.923413	300.0
SB	0.924383	0.914749	0.934228	892.0
SR	0.906061	0.921055	0.891564	681.0
macro avg	0.908037	0.909534	0.907012	2077.0
micro avg	0.913237	0.913237	0.913237	2077.0
weighted avg	0.913120	0.913391	0.913237	2077.0

The result of Model Number:Dataset Number:Featuregroup Number:Rescale of 57 : 1 : 7 : 0

	f1-score	precision	recall	support
AFIB	0.750042	0.804255	0.703192	204.0
GSVT	0.710445	0.714288	0.706934	300.0
SB	0.742074	0.697577	0.792652	892.0
SR	0.651597	0.703219	0.607118	681.0
macro avg	0.713539	0.729835	0.702474	2077.0
micro avg	0.710652	0.710652	0.710652	2077.0
weighted avg	0.708623	0.712318	0.710652	2077.0

The result of Model Number:Dataset Number:Featuregroup Number:Rescale of 57 : 1 : 7 : 1

	f1-score	precision	recall	support
AFIB	0.581085	0.642527	0.530669	204.0
GSVT	0.668614	0.660876	0.676663	300.0
SB	0.706911	0.704004	0.709843	892.0
SR	0.691730	0.681484	0.702296	681.0
macro avg	0.662085	0.672223	0.654868	2077.0
micro avg	0.684978	0.684978	0.684978	2077.0
weighted avg	0.684043	0.684353	0.684978	2077.0

The result of Model Number:Dataset Number:Featuregroup Number:Rescale of 57 : 1 : 8 : 0

	f1-score	precision	recall	support
AFIB	0.754592	0.779105	0.731718	204.0
GSVT	0.768897	0.762014	0.776058	300.0
SB	0.787180	0.781785	0.792652	892.0
SR	0.774342	0.777609	0.771148	681.0
macro avg	0.771253	0.775128	0.767894	2077.0
micro avg	0.777220	0.777220	0.777220	2077.0
weighted avg	0.777129	0.777297	0.777220	2077.0

The result of Model Number:Dataset Number:Featuregroup Number:Rescale of 57 : 1 : 8 : 1

	f1-score	precision	recall	support
AFIB	0.847201	0.845061	0.849484	875.0
GSVT	0.863729	0.858981	0.868577	691.0
SB	0.904393	0.898941	0.909924	1553.0
SR	0.877728	0.893569	0.862454	907.0
macro avg	0.873263	0.874138	0.872610	4026.0
micro avg	0.878998	0.878998	0.878998	4026.0
weighted avg	0.878976	0.879162	0.878998	4026.0

The result of Model Number:Dataset Number:Featuregroup Number:Rescale of 57 : 1 : 9 : 0

	f1-score	precision	recall	support
AFIB	0.619719	0.610388	0.634500	875.0
GSVT	0.591720	0.589998	0.596768	691.0
SB	0.631881	0.588812	0.687002	1553.0
SR	0.468073	0.625675	0.387855	907.0
macro avg	0.577848	0.603718	0.576531	4026.0
micro avg	0.592711	0.592711	0.592711	4026.0
weighted avg	0.585441	0.602010	0.592711	4026.0

The result of Model Number:Dataset Number:Featuregroup Number:Rescale of 57 : 1 : 9 : 1

	f1-score	precision	recall	support
AFIB	0.773988	0.768068	0.780103	875.0
GSVT	0.791797	0.796126	0.787560	691.0
SB	0.838449	0.837861	0.839043	1553.0
SR	0.816178	0.820137	0.812299	907.0
macro avg	0.805103	0.805548	0.804751	4026.0
micro avg	0.811372	0.811372	0.811372	4026.0
weighted avg	0.811415	0.811536	0.811372	4026.0

The result of Model Number:Dataset Number:Featuregroup Number:Rescale of 57 : 1 : 10 : 0

	f1-score	precision	recall	support
AFIB	0.814688	0.808206	0.821350	875.0
GSVT	0.782385	0.767509	0.797914	691.0
SB	0.821141	0.757985	0.895793	1553.0

SR 0.628766 0.803970 0.516377 907.0
macro avg 0.761745 0.784417 0.757859 4026.0
micro avg 0.777338 0.777338 0.777338 4026.0
weighted avg 0.769747 0.780894 0.777338 4026.0
The result of Model Number:Dataset Number:Featuregroup Number:Rescale of 57 : 1 : 10 : 1

	f1-score	precision	recall	support
AFIB	0.713401	0.698156	0.729368	875.0
GSVT	0.738772	0.750069	0.727876	691.0
SB	0.787529	0.787633	0.787428	1553.0
SR	0.767241	0.775186	0.759459	907.0
macro avg	0.751736	0.752761	0.751033	4026.0
micro avg	0.758287	0.758287	0.758287	4026.0
weighted avg	0.758479	0.758935	0.758287	4026.0

The result of Model Number:Dataset Number:Featuregroup Number:Rescale of 58 : 0 : 0 : 0

	f1-score	precision	recall	support
AFIB	0.703686	0.695348	0.712286	875.0
GSVT	0.713702	0.720601	0.707003	691.0
SB	0.757476	0.754578	0.760402	1553.0
SR	0.739816	0.748351	0.731494	907.0
macro avg	0.728670	0.729719	0.727796	4026.0
micro avg	0.734267	0.734267	0.734267	4026.0
weighted avg	0.734294	0.734471	0.734267	4026.0

The result of Model Number:Dataset Number:Featuregroup Number:Rescale of 58 : 0 : 0 : 1

	f1-score	precision	recall	support
AFIB	0.701832	0.720378	0.684286	204.0
GSVT	0.721080	0.715089	0.727303	300.0
SB	0.740949	0.738230	0.743690	892.0
SR	0.729477	0.730361	0.728615	681.0
macro avg	0.723335	0.726015	0.720974	2077.0
micro avg	0.730546	0.730546	0.730546	2077.0
weighted avg	0.730476	0.730554	0.730546	2077.0

The result of Model Number:Dataset Number:Featuregroup Number:Rescale of 58 : 0 : 1 : 0

	f1-score	precision	recall	support
AFIB	0.673372	0.698232	0.650284	204.0
GSVT	0.687765	0.680706	0.695078	300.0
SB	0.706602	0.703307	0.709929	892.0
SR	0.695585	0.696021	0.695172	681.0
macro avg	0.690831	0.694567	0.687616	2077.0
micro avg	0.697087	0.697087	0.697087	2077.0
weighted avg	0.697005	0.697155	0.697087	2077.0

The result of Model Number:Dataset Number:Featuregroup Number:Rescale of 58 : 0 : 1 : 1

	f1-score	precision	recall	support
AFIB	0.725756	0.721693	0.729900	875.0
GSVT	0.731666	0.735983	0.727445	691.0
SB	0.770750	0.768338	0.773180	1553.0
SR	0.755491	0.760489	0.750576	907.0
macro avg	0.745916	0.746626	0.745275	4026.0
micro avg	0.750832	0.750832	0.750832	4026.0
weighted avg	0.750825	0.750879	0.750832	4026.0

The result of Model Number:Dataset Number:Featuregroup Number:Rescale of 58 : 0 : 2 : 0

	f1-score	precision	recall	support
AFIB	0.672461	0.689206	0.656542	875.0
GSVT	0.710503	0.713520	0.707542	691.0
SB	0.770670	0.767721	0.773646	1553.0
SR	0.733076	0.719416	0.747305	907.0
macro avg	0.721677	0.722466	0.721259	4026.0
micro avg	0.730915	0.730915	0.730915	4026.0
weighted avg	0.730529	0.730471	0.730915	4026.0

The result of Model Number:Dataset Number:Featuregroup Number:Rescale of 58 : 0 : 2 : 1

	f1-score	precision	recall	support
AFIB	0.892279	0.909720	0.875650	204.0
GSVT	0.916797	0.910191	0.923530	300.0
SB	0.933205	0.929573	0.936867	892.0
SR	0.923004	0.925616	0.920427	681.0
macro avg	0.916321	0.918775	0.914118	2077.0
micro avg	0.923537	0.923537	0.923537	2077.0
weighted avg	0.923471	0.923526	0.923537	2077.0

The result of Model Number:Dataset Number:Featuregroup Number:Rescale of 58 : 0 : 3 : 0

	f1-score	precision	recall	support
AFIB	0.762462	0.780587	0.745244	204.0
GSVT	0.782256	0.779238	0.785380	300.0
SB	0.801640	0.799852	0.803438	892.0
SR	0.791555	0.789962	0.793182	681.0
macro avg	0.784478	0.787410	0.781811	2077.0
micro avg	0.791751	0.791751	0.791751	2077.0
weighted avg	0.791686	0.791739	0.791751	2077.0

The result of Model Number:Dataset Number:Featuregroup Number:Rescale of 58 : 0 : 3 : 1

	f1-score	precision	recall	support
AFIB	0.876289	0.897317	0.856623	204.0

GSVT 0.907448 0.902188 0.912879 300.0
SB 0.926828 0.923428 0.930256 892.0
SR 0.918774 0.919455 0.918104 681.0
macro avg 0.907335 0.910597 0.904465 2077.0
micro avg 0.916529 0.916529 0.916529 2077.0
weighted avg 0.916424 0.916493 0.916529 2077.0
The result of Model Number:Dataset Number:Featuregroup Number:Rescale of 58 : 0 : 4 : 0

	f1-score	precision	recall	support
AFIB	0.695801	0.687381	0.704461	875.0
GSVT	0.705220	0.712450	0.698190	691.0
SB	0.749015	0.746578	0.751474	1553.0
SR	0.732632	0.740160	0.725281	907.0
macro avg	0.720667	0.721642	0.719852	4026.0
micro avg	0.726210	0.726210	0.726210	4026.0
weighted avg	0.726242	0.726409	0.726210	4026.0

The result of Model Number:Dataset Number:Featuregroup Number:Rescale of 58 : 0 : 4 : 1

	f1-score	precision	recall	support
AFIB	0.845629	0.876849	0.816635	204.0
GSVT	0.863704	0.854839	0.872888	300.0
SB	0.887360	0.883222	0.891538	892.0
SR	0.873524	0.874071	0.873006	681.0
macro avg	0.867554	0.872245	0.863517	2077.0
micro avg	0.875411	0.875411	0.875411	2077.0
weighted avg	0.875308	0.875496	0.875411	2077.0

The result of Model Number:Dataset Number:Featuregroup Number:Rescale of 58 : 0 : 5 : 0

	f1-score	precision	recall	support
AFIB	0.870353	0.893877	0.848216	204.0
GSVT	0.877527	0.864609	0.890957	300.0
SB	0.894525	0.885009	0.904251	892.0
SR	0.877934	0.889997	0.866207	681.0
macro avg	0.880084	0.883373	0.877408	2077.0
micro avg	0.884353	0.884353	0.884353	2077.0
weighted avg	0.884256	0.884569	0.884353	2077.0

The result of Model Number:Dataset Number:Featuregroup Number:Rescale of 58 : 0 : 5 : 1

	f1-score	precision	recall	support
AFIB	0.883281	0.873419	0.893378	875.0
GSVT	0.900854	0.909106	0.892765	691.0
SB	0.960802	0.957674	0.963956	1553.0
SR	0.935895	0.945270	0.926706	907.0
macro avg	0.920208	0.921367	0.919201	4026.0

micro avg 0.928006 0.928006 0.928006 4026.0
weighted avg 0.928054 0.928232 0.928006 4026.0
The result of Model Number:Dataset Number:Featuregroup Number:Rescale of 58 : 0 : 6 : 0

	f1-score	precision	recall	support
AFIB	0.869408	0.858227	0.880946	875.0
GSVT	0.887106	0.894450	0.879917	691.0
SB	0.948495	0.944273	0.952765	1553.0
SR	0.921502	0.935132	0.908287	907.0
macro avg	0.906628	0.908020	0.905479	4026.0
micro avg	0.914633	0.914633	0.914633	4026.0
weighted avg	0.914689	0.914961	0.914633	4026.0

The result of Model Number:Dataset Number:Featuregroup Number:Rescale of 58 : 0 : 6 : 1

	f1-score	precision	recall	support
AFIB	0.765205	0.755167	0.775547	875.0
GSVT	0.776920	0.785556	0.768486	691.0
SB	0.824024	0.822079	0.825983	1553.0
SR	0.803393	0.810624	0.796301	907.0
macro avg	0.792385	0.793356	0.791579	4026.0
micro avg	0.798466	0.798466	0.798466	4026.0
weighted avg	0.798508	0.798687	0.798466	4026.0

The result of Model Number:Dataset Number:Featuregroup Number:Rescale of 58 : 0 : 7 : 0

	f1-score	precision	recall	support
AFIB	0.741345	0.747040	0.735905	204.0
GSVT	0.767452	0.766716	0.768214	300.0
SB	0.783153	0.781329	0.784993	892.0
SR	0.775946	0.776991	0.774920	681.0
macro avg	0.766974	0.768019	0.766008	2077.0
micro avg	0.774446	0.774446	0.774446	2077.0
weighted avg	0.774416	0.774428	0.774446	2077.0

The result of Model Number:Dataset Number:Featuregroup Number:Rescale of 58 : 0 : 7 : 1

	f1-score	precision	recall	support
AFIB	0.793423	0.845119	0.748038	204.0
GSVT	0.828182	0.807825	0.849703	300.0
SB	0.852470	0.841519	0.863739	892.0
SR	0.830328	0.840243	0.820700	681.0
macro avg	0.826101	0.833677	0.820545	2077.0
micro avg	0.836236	0.836236	0.836236	2077.0
weighted avg	0.835903	0.836588	0.836236	2077.0

The result of Model Number:Dataset Number:Featuregroup Number:Rescale of 58 : 0 : 8 : 0

	f1-score	precision	recall	support
AFIB	0.761518	0.776403	0.747326	204.0
GSVT	0.782443	0.776805	0.788189	300.0
SB	0.796446	0.793347	0.799571	892.0
SR	0.787741	0.789970	0.785541	681.0
macro avg	0.782037	0.784131	0.780157	2077.0
micro avg	0.788196	0.788196	0.788196	2077.0
weighted avg	0.788138	0.788186	0.788196	2077.0

The result of Model Number:Dataset Number:Featuregroup Number:Rescale of 58 : 0 : 8 : 1

	f1-score	precision	recall	support
AFIB	0.817582	0.836213	0.799893	875.0
GSVT	0.870012	0.871731	0.868590	691.0
SB	0.951851	0.946641	0.957121	1553.0
SR	0.899642	0.888729	0.910951	907.0
macro avg	0.884772	0.885829	0.884139	4026.0
micro avg	0.897353	0.897353	0.897353	4026.0
weighted avg	0.896861	0.896737	0.897353	4026.0

The result of Model Number:Dataset Number:Featuregroup Number:Rescale of 58 : 0 : 9 : 0

	f1-score	precision	recall	support
AFIB	0.687660	0.670063	0.706270	875.0
GSVT	0.650051	0.686894	0.617110	691.0
SB	0.689282	0.634305	0.754710	1553.0
SR	0.550619	0.668799	0.468033	907.0
macro avg	0.644403	0.665015	0.636531	4026.0
micro avg	0.655981	0.655981	0.655981	4026.0
weighted avg	0.650957	0.658874	0.655981	4026.0

The result of Model Number:Dataset Number:Featuregroup Number:Rescale of 58 : 0 : 9 : 1

	f1-score	precision	recall	support
AFIB	0.704530	0.686022	0.748635	875.0
GSVT	0.677751	0.641500	0.772622	691.0
SB	0.642204	0.638146	0.646334	1553.0
SR	0.623800	0.634548	0.613584	907.0
macro avg	0.662071	0.650054	0.695294	4026.0
micro avg	0.682865	0.682865	0.682865	4026.0
weighted avg	0.657705	0.648316	0.682865	4026.0

The result of Model Number:Dataset Number:Featuregroup Number:Rescale of 58 : 0 : 10 : 0

	f1-score	precision	recall	support
AFIB	0.672889	0.679099	0.666828	875.0
GSVT	0.688601	0.686743	0.690494	691.0
SB	0.733370	0.726477	0.740403	1553.0

SR 0.713108 0.720263 0.706107 907.0
macro avg 0.701992 0.703145 0.700958 4026.0
micro avg 0.708120 0.708120 0.708120 4026.0
weighted avg 0.707977 0.707960 0.708120 4026.0
The result of Model Number:Dataset Number:Featuregroup Number:Rescale of 58 : 0 : 10 : 1

	f1-score	precision	recall	support
AFIB	0.869073	0.877959	0.860438	875.0
GSVT	0.868812	0.855389	0.882764	691.0
SB	0.916480	0.911007	0.922027	1553.0
SR	0.892222	0.904164	0.880618	907.0
macro avg	0.886647	0.887130	0.886462	4026.0
micro avg	0.892574	0.892574	0.892574	4026.0
weighted avg	0.892530	0.892737	0.892574	4026.0

The result of Model Number:Dataset Number:Featuregroup Number:Rescale of 58 : 1 : 0 : 0

	f1-score	precision	recall	support
AFIB	0.680455	0.699604	0.662466	204.0
GSVT	0.698047	0.693147	0.703148	300.0
SB	0.717243	0.714373	0.720137	892.0
SR	0.707709	0.708152	0.707288	681.0
macro avg	0.700863	0.703819	0.698260	2077.0
micro avg	0.707806	0.707806	0.707806	2077.0
weighted avg	0.707731	0.707817	0.707806	2077.0

The result of Model Number:Dataset Number:Featuregroup Number:Rescale of 58 : 1 : 0 : 1

	f1-score	precision	recall	support
AFIB	0.751423	0.767928	0.735893	204.0
GSVT	0.775639	0.769370	0.782118	300.0
SB	0.791895	0.790921	0.792872	892.0
SR	0.785210	0.784527	0.785899	681.0
macro avg	0.776042	0.778187	0.774195	2077.0
micro avg	0.783436	0.783436	0.783436	2077.0
weighted avg	0.783380	0.783454	0.783436	2077.0

The result of Model Number:Dataset Number:Featuregroup Number:Rescale of 58 : 1 : 1 : 0

	f1-score	precision	recall	support
AFIB	0.903143	0.911473	0.895182	204.0
GSVT	0.936161	0.936194	0.936153	300.0
SB	0.955767	0.954069	0.957479	892.0
SR	0.945623	0.945370	0.945900	681.0
macro avg	0.935173	0.936777	0.933678	2077.0
micro avg	0.944483	0.944483	0.944483	2077.0
weighted avg	0.944441	0.944451	0.944483	2077.0

The result of Model Number:Dataset Number:Featuregroup Number:Rescale of 58 : 1 : 1 : 1

	f1-score	precision	recall	support
AFIB	0.716582	0.718049	0.715200	204.0
GSVT	0.732343	0.729497	0.735263	300.0
SB	0.748601	0.747602	0.749607	892.0
SR	0.743102	0.745299	0.740921	681.0
macro avg	0.735157	0.735112	0.735248	2077.0
micro avg	0.741308	0.741308	0.741308	2077.0
weighted avg	0.741305	0.741329	0.741308	2077.0

The result of Model Number:Dataset Number:Featuregroup Number:Rescale of 58 : 1 : 2 : 0

	f1-score	precision	recall	support
AFIB	0.902653	0.910979	0.894697	204.0
GSVT	0.935654	0.935687	0.935645	300.0
SB	0.955249	0.953552	0.956960	892.0
SR	0.945111	0.944858	0.945387	681.0
macro avg	0.934667	0.936269	0.933172	2077.0
micro avg	0.943971	0.943971	0.943971	2077.0
weighted avg	0.943929	0.943940	0.943971	2077.0

The result of Model Number:Dataset Number:Featuregroup Number:Rescale of 58 : 1 : 2 : 1

	f1-score	precision	recall	support
AFIB	0.686400	0.686584	0.686337	875.0
GSVT	0.698353	0.695775	0.701038	691.0
SB	0.747102	0.745288	0.748928	1553.0
SR	0.729284	0.734518	0.724144	907.0
macro avg	0.715285	0.715541	0.715112	4026.0
micro avg	0.721522	0.721522	0.721522	4026.0
weighted avg	0.721528	0.721605	0.721522	4026.0

The result of Model Number:Dataset Number:Featuregroup Number:Rescale of 58 : 1 : 3 : 0

	f1-score	precision	recall	support
AFIB	0.747704	0.747837	0.747603	875.0
GSVT	0.755615	0.758167	0.753127	691.0
SB	0.793734	0.790998	0.796494	1553.0
SR	0.778662	0.781288	0.776082	907.0
macro avg	0.768929	0.769573	0.768326	4026.0
micro avg	0.773826	0.773826	0.773826	4026.0
weighted avg	0.773792	0.773795	0.773826	4026.0

The result of Model Number:Dataset Number:Featuregroup Number:Rescale of 58 : 1 : 3 : 1

	f1-score	precision	recall	support
AFIB	0.761490	0.761626	0.761387	875.0

GSVT 0.769546 0.772146 0.767013 691.0
SB 0.808369 0.805582 0.811179 1553.0
SR 0.793019 0.795693 0.790391 907.0
macro avg 0.783106 0.783762 0.782493 4026.0
micro avg 0.788094 0.788094 0.788094 4026.0
weighted avg 0.788059 0.788062 0.788094 4026.0
The result of Model Number:Dataset Number:Featuregroup Number:Rescale of 58 : 1 : 4 : 0
f1-score precision recall support
AFIB 0.707315 0.700380 0.756261 204.0
GSVT 0.634217 0.627165 0.641587 300.0
SB 0.815008 0.809073 0.821036 892.0
SR 0.801339 0.806889 0.795930 681.0
macro avg 0.739470 0.735877 0.753704 2077.0
micro avg 0.780523 0.780523 0.780523 2077.0
weighted avg 0.773836 0.771407 0.780523 2077.0
The result of Model Number:Dataset Number:Featuregroup Number:Rescale of 58 : 1 : 4 : 1
f1-score precision recall support
AFIB 0.789118 0.803620 0.775227 204.0
GSVT 0.802373 0.797118 0.807719 300.0
SB 0.816098 0.812113 0.820123 892.0
SR 0.807662 0.811021 0.804344 681.0
macro avg 0.803813 0.805968 0.801853 2077.0
micro avg 0.808748 0.808748 0.808748 2077.0
weighted avg 0.808700 0.808755 0.808748 2077.0
The result of Model Number:Dataset Number:Featuregroup Number:Rescale of 58 : 1 : 5 : 0
f1-score precision recall support
AFIB 0.676398 0.672611 0.680260 875.0
GSVT 0.681906 0.685929 0.677971 691.0
SB 0.718332 0.716083 0.720596 1553.0
SR 0.704111 0.708769 0.699530 907.0
macro avg 0.695186 0.695848 0.694589 4026.0
micro avg 0.699768 0.699768 0.699768 4026.0
weighted avg 0.699762 0.699812 0.699768 4026.0
The result of Model Number:Dataset Number:Featuregroup Number:Rescale of 58 : 1 : 5 : 1
f1-score precision recall support
AFIB 0.788829 0.806238 0.772215 204.0
GSVT 0.806069 0.792702 0.819991 300.0
SB 0.821558 0.815132 0.828095 892.0
SR 0.801620 0.811268 0.792200 681.0
macro avg 0.804519 0.806335 0.803125 2077.0

micro avg   0.809667   0.809667 0.809667   2077.0
weighted avg 0.809569   0.809752 0.809667   2077.0
The result of Model Number:Dataset Number:Featuregroup Number:Rescale of 58 : 1 : 6 : 0

	f1-score	precision	recall	support
AFIB	0.742452	0.740931	0.744518	875.0
GSVT	0.752580	0.744680	0.760785	691.0
SB	0.793719	0.783002	0.804889	1553.0
SR	0.755097	0.783893	0.728513	907.0
macro avg	0.760962	0.763127	0.759676	4026.0
micro avg	0.766992	0.766992	0.766992	4026.0
weighted avg	0.766815	0.767482	0.766992	4026.0

The result of Model Number:Dataset Number:Featuregroup Number:Rescale of 58 : 1 : 6 : 1

	f1-score	precision	recall	support
AFIB	0.679646	0.681334	0.678012	875.0
GSVT	0.691350	0.693427	0.689373	691.0
SB	0.733398	0.728420	0.738450	1553.0
SR	0.719442	0.724783	0.714207	907.0
macro avg	0.705959	0.706991	0.705011	4026.0
micro avg	0.711430	0.711430	0.711430	4026.0
weighted avg	0.711355	0.711361	0.711430	4026.0

The result of Model Number:Dataset Number:Featuregroup Number:Rescale of 58 : 1 : 7 : 0

	f1-score	precision	recall	support
AFIB	0.780342	0.786510	0.774295	875.0
GSVT	0.799977	0.798286	0.801706	691.0
SB	0.852799	0.845262	0.860483	1553.0
SR	0.830097	0.838238	0.822132	907.0
macro avg	0.815804	0.817074	0.814654	4026.0
micro avg	0.823023	0.823023	0.823023	4026.0
weighted avg	0.822871	0.822848	0.823023	4026.0

The result of Model Number:Dataset Number:Featuregroup Number:Rescale of 58 : 1 : 7 : 1

	f1-score	precision	recall	support
AFIB	0.814536	0.816802	0.812325	875.0
GSVT	0.833194	0.832963	0.833434	691.0
SB	0.887378	0.879037	0.895888	1553.0
SR	0.863865	0.876323	0.851767	907.0
macro avg	0.849743	0.851281	0.848353	4026.0
micro avg	0.857067	0.857067	0.857067	4026.0
weighted avg	0.856950	0.856992	0.857067	4026.0

The result of Model Number:Dataset Number:Featuregroup Number:Rescale of 58 : 1 : 8 : 0

	f1-score	precision	recall	support
AFIB	0.634110	0.707230	0.574776	204.0
GSVT	0.747680	0.734666	0.761323	300.0
SB	0.798292	0.794753	0.801870	892.0
SR	0.779766	0.769104	0.790751	681.0
macro avg	0.739962	0.751438	0.732180	2077.0
micro avg	0.770063	0.770063	0.770063	2077.0
weighted avg	0.768782	0.769068	0.770063	2077.0

The result of Model Number:Dataset Number:Featuregroup Number:Rescale of 58 : 1 : 8 : 1

	f1-score	precision	recall	support
AFIB	0.752687	0.763628	0.742395	204.0
GSVT	0.769286	0.758879	0.780045	300.0
SB	0.780864	0.772726	0.789181	892.0
SR	0.765388	0.778053	0.753141	681.0
macro avg	0.767056	0.768322	0.766191	2077.0
micro avg	0.771450	0.771450	0.771450	2077.0
weighted avg	0.771350	0.771579	0.771450	2077.0

The result of Model Number:Dataset Number:Featuregroup Number:Rescale of 58 : 1 : 9 : 0

	f1-score	precision	recall	support
AFIB	0.626473	0.690100	0.574189	204.0
GSVT	0.578544	0.618551	0.543669	300.0
SB	0.629863	0.588528	0.677454	892.0
SR	0.545157	0.573813	0.519376	681.0
macro avg	0.595009	0.617748	0.578672	2077.0
micro avg	0.596157	0.596157	0.596157	2077.0
weighted avg	0.594345	0.598016	0.596157	2077.0

The result of Model Number:Dataset Number:Featuregroup Number:Rescale of 58 : 1 : 9 : 1

	f1-score	precision	recall	support
AFIB	0.879562	0.908134	0.852899	204.0
GSVT	0.896236	0.888213	0.904582	300.0
SB	0.917547	0.911258	0.923925	892.0
SR	0.902583	0.906391	0.898860	681.0
macro avg	0.898982	0.903499	0.895067	2077.0
micro avg	0.905937	0.905937	0.905937	2077.0
weighted avg	0.905832	0.906027	0.905937	2077.0

The result of Model Number:Dataset Number:Featuregroup Number:Rescale of 58 : 1 : 10 : 0

	f1-score	precision	recall	support
AFIB	0.824833	0.827618	0.822096	875.0
GSVT	0.822655	0.816860	0.828607	691.0
SB	0.865584	0.857550	0.873776	1553.0

SR 0.832676 0.848723 0.817266 907.0
macro avg 0.836437 0.837688 0.835436 4026.0
micro avg 0.842060 0.842060 0.842060 4026.0
weighted avg 0.841946 0.842073 0.842060 4026.0
The result of Model Number:Dataset Number:Featuregroup Number:Rescale of 58 : 1 : 10 : 1

	f1-score	precision	recall	support
AFIB	0.751688	0.750472	0.752975	875.0
GSVT	0.758576	0.761593	0.755622	691.0
SB	0.796637	0.793741	0.799560	1553.0
SR	0.783606	0.787571	0.779726	907.0
macro avg	0.772627	0.773344	0.771971	4026.0
micro avg	0.777426	0.777426	0.777426	4026.0
weighted avg	0.777400	0.777429	0.777426	4026.0

The result of Model Number:Dataset Number:Featuregroup Number:Rescale of 59 : 0 : 0 : 0

	f1-score	precision	recall	support
AFIB	0.681862	0.698452	0.666209	204.0
GSVT	0.690433	0.680928	0.700299	300.0
SB	0.704374	0.697340	0.711557	892.0
SR	0.692076	0.701158	0.683246	681.0
macro avg	0.692186	0.694470	0.690328	2077.0
micro avg	0.696194	0.696194	0.696194	2077.0
weighted avg	0.696117	0.696331	0.696194	2077.0

The result of Model Number:Dataset Number:Featuregroup Number:Rescale of 59 : 0 : 0 : 1

	f1-score	precision	recall	support
AFIB	0.731293	0.739076	0.724076	204.0
GSVT	0.752784	0.747768	0.758088	300.0
SB	0.768692	0.768005	0.769381	892.0
SR	0.762210	0.763227	0.761201	681.0
macro avg	0.753745	0.754519	0.753187	2077.0
micro avg	0.760618	0.760618	0.760618	2077.0
weighted avg	0.760596	0.760674	0.760618	2077.0

The result of Model Number:Dataset Number:Featuregroup Number:Rescale of 59 : 0 : 1 : 0

	f1-score	precision	recall	support
AFIB	0.674520	0.704979	0.646805	204.0
GSVT	0.697981	0.690142	0.706127	300.0
SB	0.671431	0.642158	0.718441	892.0
SR	0.559395	0.564164	0.554800	681.0
macro avg	0.650832	0.650361	0.656543	2077.0
micro avg	0.655972	0.655972	0.655972	2077.0
weighted avg	0.638835	0.629687	0.655972	2077.0

The result of Model Number:Dataset Number:Featuregroup Number:Rescale of 59 : 0 : 1 : 1

	f1-score	precision	recall	support
AFIB	0.784038	0.819443	0.751823	204.0
GSVT	0.811308	0.802196	0.820777	300.0
SB	0.780448	0.746422	0.835091	892.0
SR	0.650220	0.655765	0.644880	681.0
macro avg	0.756504	0.755956	0.763143	2077.0
micro avg	0.762479	0.762479	0.762479	2077.0
weighted avg	0.742560	0.731926	0.762479	2077.0

The result of Model Number:Dataset Number:Featuregroup Number:Rescale of 59 : 0 : 2 : 0

	f1-score	precision	recall	support
AFIB	0.809330	0.822473	0.796644	875.0
GSVT	0.860746	0.863444	0.858118	691.0
SB	0.936449	0.933222	0.939703	1553.0
SR	0.886544	0.876669	0.896697	907.0
macro avg	0.873267	0.873952	0.872791	4026.0
micro avg	0.884920	0.884920	0.884920	4026.0
weighted avg	0.884585	0.884435	0.884920	4026.0

The result of Model Number:Dataset Number:Featuregroup Number:Rescale of 59 : 0 : 2 : 1

	f1-score	precision	recall	support
AFIB	0.827295	0.816216	0.838733	875.0
GSVT	0.845793	0.852394	0.839307	691.0
SB	0.897995	0.895186	0.900828	1553.0
SR	0.876280	0.887897	0.864978	907.0
macro avg	0.861841	0.862923	0.860961	4026.0
micro avg	0.868697	0.868697	0.868697	4026.0
weighted avg	0.868778	0.869036	0.868697	4026.0

The result of Model Number:Dataset Number:Featuregroup Number:Rescale of 59 : 0 : 3 : 0

	f1-score	precision	recall	support
AFIB	0.854033	0.874477	0.835774	204.0
GSVT	0.879183	0.866795	0.892297	300.0
SB	0.902026	0.892862	0.911422	892.0
SR	0.880467	0.893180	0.868180	681.0
macro avg	0.878927	0.881828	0.876918	2077.0
micro avg	0.887051	0.887051	0.887051	2077.0
weighted avg	0.886944	0.887395	0.887051	2077.0

The result of Model Number:Dataset Number:Featuregroup Number:Rescale of 59 : 0 : 3 : 1

	f1-score	precision	recall	support
AFIB	0.661988	0.678497	0.646313	875.0

GSVT	0.704239	0.706939	0.701593	691.0
SB	0.762632	0.758939	0.766363	1553.0
SR	0.722636	0.710591	0.735143	907.0
macro avg	0.712874	0.713742	0.712353	4026.0
micro avg	0.722121	0.722121	0.722121	4026.0
weighted avg	0.721725	0.721639	0.722121	4026.0

The result of Model Number:Dataset Number:Featuregroup Number:Rescale of 59 : 0 : 4 : 0

	f1-score	precision	recall	support
AFIB	0.669619	0.670971	0.668287	204.0
GSVT	0.688249	0.686203	0.690311	300.0
SB	0.699653	0.699029	0.700280	892.0
SR	0.693639	0.694971	0.692321	681.0
macro avg	0.687790	0.687794	0.687800	2077.0
micro avg	0.693088	0.693088	0.693088	2077.0
weighted avg	0.693084	0.693090	0.693088	2077.0

The result of Model Number:Dataset Number:Featuregroup Number:Rescale of 59 : 0 : 4 : 1

	f1-score	precision	recall	support
AFIB	0.841811	0.857433	0.826956	204.0
GSVT	0.860321	0.855810	0.864897	300.0
SB	0.874056	0.870949	0.877190	892.0
SR	0.865057	0.866617	0.863541	681.0
macro avg	0.860311	0.862702	0.858146	2077.0
micro avg	0.866006	0.866006	0.866006	2077.0
weighted avg	0.865955	0.866014	0.866006	2077.0

The result of Model Number:Dataset Number:Featuregroup Number:Rescale of 59 : 0 : 5 : 0

	f1-score	precision	recall	support
AFIB	0.806622	0.804290	0.809041	875.0
GSVT	0.821572	0.820904	0.822298	691.0
SB	0.872629	0.869014	0.876280	1553.0
SR	0.846431	0.855730	0.837378	907.0
macro avg	0.836813	0.837485	0.836249	4026.0
micro avg	0.843637	0.843637	0.843637	4026.0
weighted avg	0.843618	0.843697	0.843637	4026.0

The result of Model Number:Dataset Number:Featuregroup Number:Rescale of 59 : 0 : 5 : 1

	f1-score	precision	recall	support
AFIB	0.681742	0.683556	0.679980	875.0
GSVT	0.694852	0.689957	0.699858	691.0
SB	0.734541	0.727051	0.742200	1553.0
SR	0.700964	0.716232	0.686358	907.0
macro avg	0.703025	0.704199	0.702099	4026.0

micro avg 0.708830 0.708830 0.708830 4026.0
weighted avg 0.708689 0.708794 0.708830 4026.0
The result of Model Number:Dataset Number:Featuregroup Number:Rescale of 59 : 0 : 6 : 0

	f1-score	precision	recall	support
AFIB	0.783354	0.787653	0.779151	875.0
GSVT	0.784449	0.774532	0.794747	691.0
SB	0.824984	0.821674	0.828329	1553.0
SR	0.807274	0.817074	0.797757	907.0
macro avg	0.800015	0.800233	0.799996	4026.0
micro avg	0.804989	0.804989	0.804989	4026.0
weighted avg	0.804989	0.805152	0.804989	4026.0

The result of Model Number:Dataset Number:Featuregroup Number:Rescale of 59 : 0 : 6 : 1

	f1-score	precision	recall	support
AFIB	0.757084	0.780402	0.735270	204.0
GSVT	0.776398	0.766894	0.786226	300.0
SB	0.797486	0.792748	0.802308	892.0
SR	0.784044	0.787911	0.780245	681.0
macro avg	0.778753	0.781988	0.776012	2077.0
micro avg	0.786167	0.786167	0.786167	2077.0
weighted avg	0.786065	0.786215	0.786167	2077.0

The result of Model Number:Dataset Number:Featuregroup Number:Rescale of 59 : 0 : 7 : 0

	f1-score	precision	recall	support
AFIB	0.928951	0.938000	0.920301	204.0
GSVT	0.950433	0.946069	0.954870	300.0
SB	0.963567	0.960252	0.966912	892.0
SR	0.955430	0.959136	0.951797	681.0
macro avg	0.949595	0.950864	0.948470	2077.0
micro avg	0.955639	0.955639	0.955639	2077.0
weighted avg	0.955602	0.955652	0.955639	2077.0

The result of Model Number:Dataset Number:Featuregroup Number:Rescale of 59 : 0 : 7 : 1

	f1-score	precision	recall	support
AFIB	0.754519	0.743158	0.772515	875.0
GSVT	0.720430	0.718333	0.726575	691.0
SB	0.769326	0.716889	0.836437	1553.0
SR	0.569888	0.761771	0.472221	907.0
macro avg	0.703541	0.735038	0.701937	4026.0
micro avg	0.721636	0.721636	0.721636	4026.0
weighted avg	0.712785	0.732958	0.721636	4026.0

The result of Model Number:Dataset Number:Featuregroup Number:Rescale of 59 : 0 : 8 : 0

	f1-score	precision	recall	support
AFIB	0.741894	0.740701	0.743395	875.0
GSVT	0.759192	0.755235	0.763235	691.0
SB	0.793775	0.787050	0.800622	1553.0
SR	0.763473	0.780446	0.747480	907.0
macro avg	0.764584	0.765858	0.763683	4026.0
micro avg	0.769795	0.769795	0.769795	4026.0
weighted avg	0.769737	0.770028	0.769795	4026.0

The result of Model Number:Dataset Number:Featuregroup Number:Rescale of 59 : 0 : 8 : 1

	f1-score	precision	recall	support
AFIB	0.769906	0.770043	0.769802	875.0
GSVT	0.778051	0.780679	0.775489	691.0
SB	0.817302	0.814485	0.820144	1553.0
SR	0.801783	0.804487	0.799126	907.0
macro avg	0.791760	0.792423	0.791140	4026.0
micro avg	0.796803	0.796803	0.796803	4026.0
weighted avg	0.796768	0.796771	0.796803	4026.0

The result of Model Number:Dataset Number:Featuregroup Number:Rescale of 59 : 0 : 9 : 0

	f1-score	precision	recall	support
AFIB	0.765064	0.786595	0.744838	204.0
GSVT	0.784844	0.779335	0.790579	300.0
SB	0.806427	0.803200	0.809681	892.0
SR	0.795708	0.796205	0.795234	681.0
macro avg	0.788011	0.791334	0.785083	2077.0
micro avg	0.795816	0.795816	0.795816	2077.0
weighted avg	0.795732	0.795828	0.795816	2077.0

The result of Model Number:Dataset Number:Featuregroup Number:Rescale of 59 : 0 : 9 : 1

	f1-score	precision	recall	support
AFIB	0.811453	0.847065	0.778919	204.0
GSVT	0.755410	0.760849	0.750358	300.0
SB	0.783420	0.744079	0.827156	892.0
SR	0.695016	0.740904	0.654627	681.0
macro avg	0.761325	0.773224	0.752765	2077.0
micro avg	0.754757	0.754757	0.754757	2077.0
weighted avg	0.753142	0.755575	0.754757	2077.0

The result of Model Number:Dataset Number:Featuregroup Number:Rescale of 59 : 0 : 10 : 0

	f1-score	precision	recall	support
AFIB	0.675165	0.668836	0.681702	875.0
GSVT	0.640121	0.624735	0.687178	691.0
SB	0.740181	0.737463	0.742939	1553.0

SR 0.575699 0.579737 0.571726 907.0
macro avg 0.657792 0.652693 0.670886 4026.0
micro avg 0.681488 0.681488 0.681488 4026.0
weighted avg 0.671821 0.667666 0.681488 4026.0
The result of Model Number:Dataset Number:Featuregroup Number:Rescale of 59 : 0 : 10 : 1

	f1-score	precision	recall	support
AFIB	0.786149	0.845246	0.735594	204.0
GSVT	0.749726	0.773524	0.727463	300.0
SB	0.781108	0.742456	0.824012	892.0
SR	0.691456	0.721177	0.664159	681.0
macro avg	0.752110	0.770601	0.737807	2077.0
micro avg	0.748970	0.748970	0.748970	2077.0
weighted avg	0.747676	0.750062	0.748970	2077.0

The result of Model Number:Dataset Number:Featuregroup Number:Rescale of 59 : 1 : 0 : 0

	f1-score	precision	recall	support
AFIB	0.775391	0.814132	0.740559	204.0
GSVT	0.798767	0.783964	0.814418	300.0
SB	0.817047	0.808811	0.825469	892.0
SR	0.794387	0.801483	0.787488	681.0
macro avg	0.796398	0.802097	0.791983	2077.0
micro avg	0.803080	0.803080	0.803080	2077.0
weighted avg	0.802885	0.803342	0.803080	2077.0

The result of Model Number:Dataset Number:Featuregroup Number:Rescale of 59 : 1 : 0 : 1

	f1-score	precision	recall	support
AFIB	0.701112	0.711862	0.690797	204.0
GSVT	0.717736	0.711647	0.723961	300.0
SB	0.730222	0.727865	0.732596	892.0
SR	0.722718	0.725401	0.720064	681.0
macro avg	0.717947	0.719194	0.716855	2077.0
micro avg	0.723134	0.723134	0.723134	2077.0
weighted avg	0.723099	0.723143	0.723134	2077.0

The result of Model Number:Dataset Number:Featuregroup Number:Rescale of 59 : 1 : 1 : 0

	f1-score	precision	recall	support
AFIB	0.745967	0.760996	0.731998	204.0
GSVT	0.769903	0.763719	0.776335	300.0
SB	0.786570	0.785778	0.787364	892.0
SR	0.779400	0.778953	0.779857	681.0
macro avg	0.770460	0.772362	0.768889	2077.0
micro avg	0.777872	0.777872	0.777872	2077.0
weighted avg	0.777824	0.777920	0.777872	2077.0

The result of Model Number:Dataset Number:Featuregroup Number:Rescale of 59 : 1 : 1 : 1

	f1-score	precision	recall	support
AFIB	0.740834	0.740470	0.741256	875.0
GSVT	0.756916	0.753192	0.760725	691.0
SB	0.791748	0.786572	0.796998	1553.0
SR	0.769209	0.781739	0.757096	907.0
macro avg	0.764677	0.765493	0.764019	4026.0
micro avg	0.769668	0.769668	0.769668	4026.0
weighted avg	0.769627	0.769734	0.769668	4026.0

The result of Model Number:Dataset Number:Featuregroup Number:Rescale of 59 : 1 : 2 : 0

	f1-score	precision	recall	support
AFIB	0.639919	0.666636	0.615377	875.0
GSVT	0.691336	0.687836	0.694955	691.0
SB	0.758070	0.753804	0.762406	1553.0
SR	0.709819	0.694057	0.726325	907.0
macro avg	0.699786	0.700583	0.699766	4026.0
micro avg	0.710745	0.710745	0.710745	4026.0
weighted avg	0.710067	0.710077	0.710745	4026.0

The result of Model Number:Dataset Number:Featuregroup Number:Rescale of 59 : 1 : 2 : 1

	f1-score	precision	recall	support
AFIB	0.668002	0.661283	0.674877	875.0
GSVT	0.673980	0.679980	0.668133	691.0
SB	0.713041	0.710852	0.715244	1553.0
SR	0.699233	0.705353	0.693220	907.0
macro avg	0.688564	0.689367	0.687869	4026.0
micro avg	0.693423	0.693423	0.693423	4026.0
weighted avg	0.693437	0.693541	0.693423	4026.0

The result of Model Number:Dataset Number:Featuregroup Number:Rescale of 59 : 1 : 3 : 0

	f1-score	precision	recall	support
AFIB	0.884035	0.875841	0.892433	875.0
GSVT	0.899911	0.904798	0.895091	691.0
SB	0.953507	0.951309	0.955716	1553.0
SR	0.931291	0.939885	0.922882	907.0
macro avg	0.917186	0.917958	0.916530	4026.0
micro avg	0.924160	0.924160	0.924160	4026.0
weighted avg	0.924204	0.924350	0.924160	4026.0

The result of Model Number:Dataset Number:Featuregroup Number:Rescale of 59 : 1 : 3 : 1

	f1-score	precision	recall	support
AFIB	0.755388	0.760674	0.750201	204.0

GSVT	0.772963	0.769152	0.776823	300.0
SB	0.788326	0.787014	0.789651	892.0
SR	0.780480	0.782333	0.778645	681.0
macro avg	0.774289	0.774793	0.773830	2077.0
micro avg	0.780315	0.780315	0.780315	2077.0
weighted avg	0.780299	0.780312	0.780315	2077.0

The result of Model Number:Dataset Number:Featuregroup Number:Rescale of 59 : 1 : 4 : 0

	f1-score	precision	recall	support
AFIB	0.697686	0.727903	0.670076	204.0
GSVT	0.652215	0.658302	0.646567	300.0
SB	0.676068	0.642830	0.712980	892.0
SR	0.602560	0.640586	0.568990	681.0
macro avg	0.657132	0.667405	0.649653	2077.0
micro avg	0.651962	0.651962	0.651962	2077.0
weighted avg	0.650645	0.652685	0.651962	2077.0

The result of Model Number:Dataset Number:Featuregroup Number:Rescale of 59 : 1 : 4 : 1

	f1-score	precision	recall	support
AFIB	0.875088	0.894400	0.856657	204.0
GSVT	0.894213	0.879384	0.909657	300.0
SB	0.911395	0.904267	0.918647	892.0
SR	0.889277	0.899980	0.878827	681.0
macro avg	0.892493	0.894508	0.890947	2077.0
micro avg	0.898204	0.898204	0.898204	2077.0
weighted avg	0.898095	0.898298	0.898204	2077.0

The result of Model Number:Dataset Number:Featuregroup Number:Rescale of 59 : 1 : 5 : 0

	f1-score	precision	recall	support
AFIB	0.799027	0.807906	0.790752	204.0
GSVT	0.823778	0.819088	0.828761	300.0
SB	0.840166	0.839040	0.841296	892.0
SR	0.833019	0.834134	0.831916	681.0
macro avg	0.823997	0.825042	0.823181	2077.0
micro avg	0.831446	0.831446	0.831446	2077.0
weighted avg	0.831415	0.831492	0.831446	2077.0

The result of Model Number:Dataset Number:Featuregroup Number:Rescale of 59 : 1 : 5 : 1

	f1-score	precision	recall	support
AFIB	0.750318	0.743682	0.757104	875.0
GSVT	0.682169	0.660629	0.758927	691.0
SB	0.615012	0.590497	0.653350	1553.0
SR	0.633859	0.640418	0.627475	907.0
macro avg	0.670340	0.658806	0.699214	4026.0

micro avg	0.688191	0.688191	0.688191	4026.0
weighted avg	0.660192	0.647073	0.688191	4026.0

The result of Model Number:Dataset Number:Featuregroup Number:Rescale of 59 : 1 : 6 : 0

	f1-score	precision	recall	support
AFIB	0.748001	0.746469	0.750082	875.0
GSVT	0.758205	0.750246	0.766471	691.0
SB	0.799651	0.788854	0.810904	1553.0
SR	0.760740	0.789751	0.733958	907.0
macro avg	0.766649	0.768830	0.765354	4026.0
micro avg	0.772724	0.772724	0.772724	4026.0
weighted avg	0.772546	0.773218	0.772724	4026.0

The result of Model Number:Dataset Number:Featuregroup Number:Rescale of 59 : 1 : 6 : 1

	f1-score	precision	recall	support
AFIB	0.880116	0.879324	0.880995	875.0
GSVT	0.895364	0.887165	0.903785	691.0
SB	0.939577	0.931317	0.947987	1553.0
SR	0.895343	0.917477	0.874330	907.0
macro avg	0.902600	0.903821	0.901774	4026.0
micro avg	0.909246	0.909246	0.909246	4026.0
weighted avg	0.909100	0.909321	0.909246	4026.0

The result of Model Number:Dataset Number:Featuregroup Number:Rescale of 59 : 1 : 7 : 0

	f1-score	precision	recall	support
AFIB	0.722622	0.760670	0.688393	204.0
GSVT	0.758909	0.752261	0.765708	300.0
SB	0.778601	0.773794	0.783482	892.0
SR	0.767978	0.766421	0.769561	681.0
macro avg	0.757027	0.763286	0.751786	2077.0
micro avg	0.767010	0.767010	0.767010	2077.0
weighted avg	0.766775	0.766977	0.767010	2077.0

The result of Model Number:Dataset Number:Featuregroup Number:Rescale of 59 : 1 : 7 : 1

	f1-score	precision	recall	support
AFIB	0.781555	0.773112	0.889670	204.0
GSVT	0.935080	0.927795	0.942562	300.0
SB	0.763115	0.761519	0.764719	892.0
SR	0.946279	0.946014	0.946557	681.0
macro avg	0.856507	0.852110	0.885877	2077.0
micro avg	0.862299	0.862299	0.862299	2077.0
weighted avg	0.849820	0.847166	0.862299	2077.0

The result of Model Number:Dataset Number:Featuregroup Number:Rescale of 59 : 1 : 8 : 0

	f1-score	precision	recall	support
AFIB	0.752250	0.758213	0.746400	875.0
GSVT	0.761787	0.758429	0.765202	691.0
SB	0.810071	0.806869	0.813305	1553.0
SR	0.790165	0.792398	0.787983	907.0
macro avg	0.778568	0.778977	0.778222	4026.0
micro avg	0.784803	0.784803	0.784803	4026.0
weighted avg	0.784733	0.784720	0.784803	4026.0

The result of Model Number:Dataset Number:Featuregroup Number:Rescale of 59 : 1 : 8 : 1

	f1-score	precision	recall	support
AFIB	0.885495	0.895623	0.875936	204.0
GSVT	0.906222	0.900859	0.911663	300.0
SB	0.869956	0.837821	0.924199	892.0
SR	0.729756	0.733288	0.726277	681.0
macro avg	0.847857	0.841898	0.859519	2077.0
micro avg	0.852754	0.852754	0.852754	2077.0
weighted avg	0.830752	0.818330	0.852754	2077.0

The result of Model Number:Dataset Number:Featuregroup Number:Rescale of 59 : 1 : 9 : 0

	f1-score	precision	recall	support
AFIB	0.852590	0.852171	0.853076	875.0
GSVT	0.871098	0.866812	0.875481	691.0
SB	0.911184	0.905227	0.917226	1553.0
SR	0.885245	0.899665	0.871305	907.0
macro avg	0.880029	0.880969	0.879272	4026.0
micro avg	0.885773	0.885773	0.885773	4026.0
weighted avg	0.885726	0.885850	0.885773	4026.0

The result of Model Number:Dataset Number:Featuregroup Number:Rescale of 59 : 1 : 9 : 1

	f1-score	precision	recall	support
AFIB	0.852582	0.857685	0.847584	204.0
GSVT	0.875294	0.872693	0.877918	300.0
SB	0.888698	0.887609	0.889793	892.0
SR	0.882219	0.883260	0.881188	681.0
macro avg	0.874699	0.875312	0.874121	2077.0
micro avg	0.881111	0.881111	0.881111	2077.0
weighted avg	0.881091	0.881090	0.881111	2077.0

The result of Model Number:Dataset Number:Featuregroup Number:Rescale of 59 : 1 : 10 : 0

	f1-score	precision	recall	support
AFIB	0.723424	0.730496	0.716657	875.0
GSVT	0.717899	0.712452	0.723665	691.0
SB	0.759618	0.751803	0.767665	1553.0

SR 0.735507 0.747335 0.724254 907.0
macro avg 0.734112 0.735522 0.733061 4026.0
micro avg 0.739248 0.739248 0.739248 4026.0
weighted avg 0.739159 0.739412 0.739248 4026.0
The result of Model Number:Dataset Number:Featuregroup Number:Rescale of 59 : 1 : 10 : 1

	f1-score	precision	recall	support
AFIB	0.910894	0.932752	0.890451	204.0
GSVT	0.943283	0.937816	0.948928	300.0
SB	0.963429	0.959894	0.966992	892.0
SR	0.955056	0.955765	0.954360	681.0
macro avg	0.943165	0.946557	0.940183	2077.0
micro avg	0.952723	0.952723	0.952723	2077.0
weighted avg	0.952614	0.952686	0.952723	2077.0

The result of Model Number:Dataset Number:Featuregroup Number:Rescale of 60 : 0 : 0 : 0

	f1-score	precision	recall	support
AFIB	0.790408	0.796673	0.784260	875.0
GSVT	0.800428	0.796900	0.804016	691.0
SB	0.851161	0.847796	0.854559	1553.0
SR	0.830245	0.832591	0.827953	907.0
macro avg	0.818061	0.818490	0.817697	4026.0
micro avg	0.824612	0.824612	0.824612	4026.0
weighted avg	0.824538	0.824524	0.824612	4026.0

The result of Model Number:Dataset Number:Featuregroup Number:Rescale of 60 : 0 : 0 : 1

	f1-score	precision	recall	support
AFIB	0.842639	0.832060	0.853736	875.0
GSVT	0.781802	0.805472	0.762097	691.0
SB	0.841734	0.779632	0.916812	1553.0
SR	0.654475	0.801028	0.558085	907.0
macro avg	0.780163	0.804548	0.772682	4026.0
micro avg	0.795733	0.795733	0.795733	4026.0
weighted avg	0.789458	0.800282	0.795733	4026.0

The result of Model Number:Dataset Number:Featuregroup Number:Rescale of 60 : 0 : 1 : 0

	f1-score	precision	recall	support
AFIB	0.659897	0.656592	0.663452	875.0
GSVT	0.674807	0.675719	0.674032	691.0
SB	0.718577	0.716210	0.720978	1553.0
SR	0.697474	0.704713	0.690464	907.0
macro avg	0.687688	0.688308	0.687232	4026.0
micro avg	0.693544	0.693544	0.693544	4026.0
weighted avg	0.693557	0.693713	0.693544	4026.0

The result of Model Number:Dataset Number:Featuregroup Number:Rescale of 60 : 0 : 1 : 1

	f1-score	precision	recall	support
AFIB	0.780150	0.797928	0.763271	875.0
GSVT	0.830179	0.831820	0.828822	691.0
SB	0.908271	0.903300	0.913300	1553.0
SR	0.858452	0.848039	0.869244	907.0
macro avg	0.844263	0.845272	0.843659	4026.0
micro avg	0.856269	0.856269	0.856269	4026.0
weighted avg	0.855799	0.855681	0.856269	4026.0

The result of Model Number:Dataset Number:Featuregroup Number:Rescale of 60 : 0 : 2 : 0

	f1-score	precision	recall	support
AFIB	0.713295	0.708475	0.718279	875.0
GSVT	0.730697	0.730988	0.730501	691.0
SB	0.776217	0.774236	0.778216	1553.0
SR	0.755082	0.763518	0.746840	907.0
macro avg	0.743823	0.744304	0.743459	4026.0
micro avg	0.749931	0.749931	0.749931	4026.0
weighted avg	0.749968	0.750106	0.749931	4026.0

The result of Model Number:Dataset Number:Featuregroup Number:Rescale of 60 : 0 : 2 : 1

	f1-score	precision	recall	support
AFIB	0.716142	0.737860	0.695696	875.0
GSVT	0.764492	0.759190	0.769909	691.0
SB	0.836447	0.833247	0.839677	1553.0
SR	0.791077	0.779204	0.803364	907.0
macro avg	0.777039	0.777375	0.777161	4026.0
micro avg	0.788229	0.788229	0.788229	4026.0
weighted avg	0.787729	0.787630	0.788229	4026.0

The result of Model Number:Dataset Number:Featuregroup Number:Rescale of 60 : 0 : 3 : 0

	f1-score	precision	recall	support
AFIB	0.746569	0.839451	0.672852	204.0
GSVT	0.885749	0.866835	0.905546	300.0
SB	0.945554	0.940734	0.950428	892.0
SR	0.924341	0.912636	0.936410	681.0
macro avg	0.875553	0.889914	0.866309	2077.0
micro avg	0.912086	0.912086	0.912086	2077.0
weighted avg	0.910417	0.910899	0.912086	2077.0

The result of Model Number:Dataset Number:Featuregroup Number:Rescale of 60 : 0 : 3 : 1

	f1-score	precision	recall	support
AFIB	0.776893	0.786964	0.767283	204.0

GSVT 0.806954 0.806432 0.807501 300.0
SB 0.823957 0.822217 0.825710 892.0
SR 0.815670 0.815207 0.816152 681.0
macro avg 0.805869 0.807705 0.804161 2077.0
micro avg 0.814207 0.814207 0.814207 2077.0
weighted avg 0.814162 0.814176 0.814207 2077.0
The result of Model Number:Dataset Number:Featuregroup Number:Rescale of 60 : 0 : 4 : 0
f1-score precision recall support
AFIB 0.735621 0.733582 0.737726 875.0
GSVT 0.741442 0.744978 0.738013 691.0
SB 0.776160 0.773679 0.778658 1553.0
SR 0.763751 0.767440 0.760133 907.0
macro avg 0.754243 0.754920 0.753633 4026.0
micro avg 0.758613 0.758613 0.758613 4026.0
weighted avg 0.758595 0.758633 0.758613 4026.0
The result of Model Number:Dataset Number:Featuregroup Number:Rescale of 60 : 0 : 4 : 1
f1-score precision recall support
AFIB 0.684734 0.686555 0.682964 875.0
GSVT 0.697901 0.692985 0.702929 691.0
SB 0.737764 0.730241 0.745457 1553.0
SR 0.704039 0.719374 0.689369 907.0
macro avg 0.706109 0.707289 0.705180 4026.0
micro avg 0.711940 0.711940 0.711940 4026.0
weighted avg 0.711799 0.711904 0.711940 4026.0
The result of Model Number:Dataset Number:Featuregroup Number:Rescale of 60 : 0 : 5 : 0
f1-score precision recall support
AFIB 0.838641 0.904005 0.782481 204.0
GSVT 0.864215 0.878844 0.850102 300.0
SB 0.915905 0.907476 0.924521 892.0
SR 0.879451 0.866698 0.892626 681.0
macro avg 0.874553 0.889256 0.862432 2077.0
micro avg 0.889363 0.889363 0.889363 2077.0
weighted avg 0.888898 0.889629 0.889363 2077.0
The result of Model Number:Dataset Number:Featuregroup Number:Rescale of 60 : 0 : 5 : 1
f1-score precision recall support
AFIB 0.865850 0.849665 0.882757 875.0
GSVT 0.896180 0.910328 0.882577 691.0
SB 0.953049 0.951346 0.954767 1553.0
SR 0.928824 0.938665 0.919194 907.0
macro avg 0.910976 0.912501 0.909824 4026.0

micro avg 0.918712 0.918712 0.918712 4026.0
weighted avg 0.918880 0.919350 0.918712 4026.0
The result of Model Number:Dataset Number:Featuregroup Number:Rescale of 60 : 0 : 6 : 0

	f1-score	precision	recall	support
AFIB	0.702436	0.718320	0.687260	875.0
GSVT	0.744742	0.747911	0.741643	691.0
SB	0.808635	0.804824	0.812487	1553.0
SR	0.766171	0.754418	0.778327	907.0
macro avg	0.755496	0.756368	0.754929	4026.0
micro avg	0.765415	0.765415	0.765415	4026.0
weighted avg	0.765021	0.764900	0.765415	4026.0

The result of Model Number:Dataset Number:Featuregroup Number:Rescale of 60 : 0 : 6 : 1

	f1-score	precision	recall	support
AFIB	0.684169	0.708095	0.662122	204.0
GSVT	0.713062	0.708715	0.717560	300.0
SB	0.727704	0.724715	0.730722	892.0
SR	0.720977	0.719926	0.722036	681.0
macro avg	0.711478	0.715363	0.708110	2077.0
micro avg	0.719235	0.719235	0.719235	2077.0
weighted avg	0.719108	0.719201	0.719235	2077.0

The result of Model Number:Dataset Number:Featuregroup Number:Rescale of 60 : 0 : 7 : 0

	f1-score	precision	recall	support
AFIB	0.838958	0.838539	0.839414	875.0
GSVT	0.849393	0.855076	0.843853	691.0
SB	0.900473	0.898404	0.902557	1553.0
SR	0.874392	0.874185	0.874751	907.0
macro avg	0.865804	0.866551	0.865144	4026.0
micro avg	0.872494	0.872494	0.872494	4026.0
weighted avg	0.872461	0.872501	0.872494	4026.0

The result of Model Number:Dataset Number:Featuregroup Number:Rescale of 60 : 0 : 7 : 1

	f1-score	precision	recall	support
AFIB	0.863587	0.863157	0.864057	875.0
GSVT	0.874329	0.880179	0.868627	691.0
SB	0.926909	0.924779	0.929054	1553.0
SR	0.900062	0.899849	0.900431	907.0
macro avg	0.891222	0.891991	0.890542	4026.0
micro avg	0.898108	0.898108	0.898108	4026.0
weighted avg	0.898074	0.898115	0.898108	4026.0

The result of Model Number:Dataset Number:Featuregroup Number:Rescale of 60 : 0 : 8 : 0

	f1-score	precision	recall	support
AFIB	0.828310	0.816549	0.840507	875.0
GSVT	0.858349	0.869299	0.847801	691.0
SB	0.912188	0.910850	0.913539	1553.0
SR	0.889463	0.896135	0.882892	907.0
macro avg	0.872078	0.873208	0.871185	4026.0
micro avg	0.879479	0.879479	0.879479	4026.0
weighted avg	0.879598	0.879908	0.879479	4026.0

The result of Model Number:Dataset Number:Featuregroup Number:Rescale of 60 : 0 : 8 : 1

	f1-score	precision	recall	support
AFIB	0.755965	0.762768	0.749521	875.0
GSVT	0.771365	0.755724	0.787848	691.0
SB	0.818037	0.808937	0.827443	1553.0
SR	0.785576	0.808928	0.763720	907.0
macro avg	0.782736	0.784089	0.782133	4026.0
micro avg	0.789356	0.789356	0.789356	4026.0
weighted avg	0.789223	0.789767	0.789356	4026.0

The result of Model Number:Dataset Number:Featuregroup Number:Rescale of 60 : 0 : 9 : 0

	f1-score	precision	recall	support
AFIB	0.682698	0.696043	0.669975	204.0
GSVT	0.701457	0.696403	0.706609	300.0
SB	0.714011	0.711233	0.716813	892.0
SR	0.706207	0.708205	0.704235	681.0
macro avg	0.701093	0.702971	0.699408	2077.0
micro avg	0.706615	0.706615	0.706615	2077.0
weighted avg	0.706564	0.706606	0.706615	2077.0

The result of Model Number:Dataset Number:Featuregroup Number:Rescale of 60 : 0 : 9 : 1

	f1-score	precision	recall	support
AFIB	0.661398	0.652892	0.670176	875.0
GSVT	0.674862	0.680449	0.669393	691.0
SB	0.721563	0.718351	0.724812	1553.0
SR	0.701029	0.711397	0.690975	907.0
macro avg	0.689713	0.690772	0.688839	4026.0
micro avg	0.695803	0.695803	0.695803	4026.0
weighted avg	0.695845	0.696053	0.695803	4026.0

The result of Model Number:Dataset Number:Featuregroup Number:Rescale of 60 : 0 : 10 : 0

	f1-score	precision	recall	support
AFIB	0.746256	0.826864	0.680106	204.0
GSVT	0.865071	0.851001	0.879749	300.0
SB	0.922545	0.918344	0.926784	892.0

SR 0.900254 0.888798 0.912022 681.0
macro avg 0.858531 0.871252 0.849665 2077.0
micro avg 0.890922 0.890922 0.890922 2077.0
weighted avg 0.889620 0.889945 0.890922 2077.0
The result of Model Number:Dataset Number:Featuregroup Number:Rescale of 60 : 0 : 10 : 1

	f1-score	precision	recall	support
AFIB	0.661171	0.651289	0.671382	875.0
GSVT	0.673848	0.679611	0.668192	691.0
SB	0.719991	0.717512	0.722492	1553.0
SR	0.698917	0.709341	0.688818	907.0
macro avg	0.688482	0.689438	0.687721	4026.0
micro avg	0.694478	0.694478	0.694478	4026.0
weighted avg	0.694540	0.694773	0.694478	4026.0

The result of Model Number:Dataset Number:Featuregroup Number:Rescale of 60 : 1 : 0 : 0

	f1-score	precision	recall	support
AFIB	0.596977	0.651044	0.552197	204.0
GSVT	0.692901	0.684984	0.701218	300.0
SB	0.734781	0.732900	0.736675	892.0
SR	0.716791	0.707084	0.726788	681.0
macro avg	0.685363	0.694003	0.679219	2077.0
micro avg	0.710193	0.710193	0.710193	2077.0
weighted avg	0.709298	0.709475	0.710193	2077.0

The result of Model Number:Dataset Number:Featuregroup Number:Rescale of 60 : 1 : 0 : 1

	f1-score	precision	recall	support
AFIB	0.767427	0.775778	0.759478	204.0
GSVT	0.793398	0.790089	0.796863	300.0
SB	0.809656	0.808932	0.810382	892.0
SR	0.802005	0.802011	0.802005	681.0
macro avg	0.793121	0.794203	0.792182	2077.0
micro avg	0.800683	0.800683	0.800683	2077.0
weighted avg	0.800651	0.800685	0.800683	2077.0

The result of Model Number:Dataset Number:Featuregroup Number:Rescale of 60 : 1 : 1 : 0

	f1-score	precision	recall	support
AFIB	0.873850	0.912687	0.838304	204.0
GSVT	0.910265	0.903119	0.917545	300.0
SB	0.924169	0.917040	0.931419	892.0
SR	0.913945	0.915454	0.912473	681.0
macro avg	0.905557	0.912075	0.899935	2077.0
micro avg	0.914057	0.914057	0.914057	2077.0
weighted avg	0.913866	0.914082	0.914057	2077.0

The result of Model Number:Dataset Number:Featuregroup Number:Rescale of 60 : 1 : 1 : 1

	f1-score	precision	recall	support
AFIB	0.814993	0.821572	0.808587	204.0
GSVT	0.833693	0.829605	0.837850	300.0
SB	0.849964	0.849021	0.850916	892.0
SR	0.843617	0.844739	0.842504	681.0
macro avg	0.835567	0.836234	0.834965	2077.0
micro avg	0.842114	0.842114	0.842114	2077.0
weighted avg	0.842098	0.842117	0.842114	2077.0

The result of Model Number:Dataset Number:Featuregroup Number:Rescale of 60 : 1 : 2 : 0

	f1-score	precision	recall	support
AFIB	0.653539	0.649630	0.657585	875.0
GSVT	0.658891	0.662449	0.655467	691.0
SB	0.698326	0.696285	0.700390	1553.0
SR	0.682546	0.687449	0.677726	907.0
macro avg	0.673326	0.673953	0.672792	4026.0
micro avg	0.678271	0.678271	0.678271	4026.0
weighted avg	0.678269	0.678347	0.678271	4026.0

The result of Model Number:Dataset Number:Featuregroup Number:Rescale of 60 : 1 : 2 : 1

	f1-score	precision	recall	support
AFIB	0.701577	0.713241	0.690449	204.0
GSVT	0.715030	0.709950	0.720236	300.0
SB	0.731615	0.727503	0.735790	892.0
SR	0.722294	0.726713	0.717951	681.0
macro avg	0.717629	0.719352	0.716107	2077.0
micro avg	0.723241	0.723241	0.723241	2077.0
weighted avg	0.723213	0.723308	0.723241	2077.0

The result of Model Number:Dataset Number:Featuregroup Number:Rescale of 60 : 1 : 3 : 0

	f1-score	precision	recall	support
AFIB	0.750470	0.757807	0.743450	875.0
GSVT	0.744738	0.739087	0.750720	691.0
SB	0.788017	0.779910	0.796365	1553.0
SR	0.763005	0.775275	0.751332	907.0
macro avg	0.761558	0.763020	0.760467	4026.0
micro avg	0.766885	0.766885	0.766885	4026.0
weighted avg	0.766794	0.767055	0.766885	4026.0

The result of Model Number:Dataset Number:Featuregroup Number:Rescale of 60 : 1 : 3 : 1

	f1-score	precision	recall	support
AFIB	0.670069	0.734563	0.616214	204.0

GSVT 0.779292 0.771285 0.787613 300.0
SB 0.826109 0.822896 0.829350 892.0
SR 0.806969 0.795810 0.818459 681.0
macro avg 0.770610 0.781139 0.762909 2077.0
micro avg 0.798817 0.798817 0.798817 2077.0
weighted avg 0.797745 0.797885 0.798817 2077.0
The result of Model Number:Dataset Number:Featuregroup Number:Rescale of 60 : 1 : 4 : 0
f1-score precision recall support
AFIB 0.730609 0.737352 0.724029 875.0
GSVT 0.747669 0.745651 0.749725 691.0
SB 0.796278 0.788794 0.803914 1553.0
SR 0.774278 0.782047 0.766677 907.0
macro avg 0.762209 0.763461 0.761086 4026.0
micro avg 0.768862 0.768862 0.768862 4026.0
weighted avg 0.768707 0.768689 0.768862 4026.0
The result of Model Number:Dataset Number:Featuregroup Number:Rescale of 60 : 1 : 4 : 1
f1-score precision recall support
AFIB 0.760768 0.777691 0.744729 204.0
GSVT 0.793181 0.788252 0.798202 300.0
SB 0.810399 0.808692 0.812123 892.0
SR 0.801767 0.801200 0.802360 681.0
macro avg 0.791529 0.793959 0.789354 2077.0
micro avg 0.800292 0.800292 0.800292 2077.0
weighted avg 0.800207 0.800238 0.800292 2077.0
The result of Model Number:Dataset Number:Featuregroup Number:Rescale of 60 : 1 : 5 : 0
f1-score precision recall support
AFIB 0.787563 0.794569 0.780720 875.0
GSVT 0.804093 0.795269 0.813167 691.0
SB 0.864797 0.858064 0.871644 1553.0
SR 0.836059 0.847909 0.824538 907.0
macro avg 0.823128 0.823953 0.822517 4026.0
micro avg 0.831234 0.831234 0.831234 4026.0
weighted avg 0.831118 0.831199 0.831234 4026.0
The result of Model Number:Dataset Number:Featuregroup Number:Rescale of 60 : 1 : 5 : 1
f1-score precision recall support
AFIB 0.798632 0.805776 0.791713 875.0
GSVT 0.813885 0.798409 0.830129 691.0
SB 0.863871 0.856387 0.871490 1553.0
SR 0.828389 0.847436 0.810217 907.0
macro avg 0.826194 0.827002 0.825887 4026.0

micro avg 0.833248 0.833248 0.833248 4026.0
weighted avg 0.833119 0.833420 0.833248 4026.0
The result of Model Number:Dataset Number:Featuregroup Number:Rescale of 60 : 1 : 6 : 0

	f1-score	precision	recall	support
AFIB	0.699328	0.728525	0.672507	875.0
GSVT	0.755518	0.751693	0.759474	691.0
SB	0.828448	0.823786	0.833186	1553.0
SR	0.775717	0.758492	0.793756	907.0
macro avg	0.764753	0.765624	0.764731	4026.0
micro avg	0.776730	0.776730	0.776730	4026.0
weighted avg	0.775989	0.775999	0.776730	4026.0

The result of Model Number:Dataset Number:Featuregroup Number:Rescale of 60 : 1 : 6 : 1

	f1-score	precision	recall	support
AFIB	0.886152	0.884337	0.888618	875.0
GSVT	0.898240	0.888811	0.908033	691.0
SB	0.947342	0.934550	0.960674	1553.0
SR	0.901245	0.935614	0.869516	907.0
macro avg	0.908245	0.910828	0.906710	4026.0
micro avg	0.915442	0.915442	0.915442	4026.0
weighted avg	0.915231	0.916026	0.915442	4026.0

The result of Model Number:Dataset Number:Featuregroup Number:Rescale of 60 : 1 : 7 : 0

	f1-score	precision	recall	support
AFIB	0.830766	0.833571	0.828009	875.0
GSVT	0.828572	0.822736	0.834566	691.0
SB	0.871810	0.863718	0.880060	1553.0
SR	0.838666	0.854828	0.823144	907.0
macro avg	0.842453	0.843713	0.841445	4026.0
micro avg	0.848117	0.848117	0.848117	4026.0
weighted avg	0.848001	0.848129	0.848117	4026.0

The result of Model Number:Dataset Number:Featuregroup Number:Rescale of 60 : 1 : 7 : 1

	f1-score	precision	recall	support
AFIB	0.775266	0.763679	0.787239	875.0
GSVT	0.790130	0.796888	0.783499	691.0
SB	0.844236	0.841329	0.847169	1553.0
SR	0.819526	0.831749	0.807685	907.0
macro avg	0.807290	0.808411	0.806398	4026.0
micro avg	0.814321	0.814321	0.814321	4026.0
weighted avg	0.814393	0.814667	0.814321	4026.0

The result of Model Number:Dataset Number:Featuregroup Number:Rescale of 60 : 1 : 8 : 0

	f1-score	precision	recall	support
AFIB	0.809736	0.821199	0.799254	204.0
GSVT	0.826548	0.811330	0.842500	300.0
SB	0.844165	0.834831	0.853723	892.0
SR	0.826424	0.843256	0.810300	681.0
macro avg	0.826718	0.827654	0.826444	2077.0
micro avg	0.832515	0.832515	0.832515	2077.0
weighted avg	0.832422	0.832860	0.832515	2077.0

The result of Model Number:Dataset Number:Featuregroup Number:Rescale of 60 : 1 : 8 : 1

	f1-score	precision	recall	support
AFIB	0.860934	0.902153	0.823520	204.0
GSVT	0.906795	0.897088	0.916749	300.0
SB	0.926057	0.920941	0.931241	892.0
SR	0.915595	0.914801	0.916406	681.0
macro avg	0.902345	0.908746	0.896979	2077.0
micro avg	0.913704	0.913704	0.913704	2077.0
weighted avg	0.913448	0.913637	0.913704	2077.0

The result of Model Number:Dataset Number:Featuregroup Number:Rescale of 60 : 1 : 9 : 0

	f1-score	precision	recall	support
AFIB	0.642630	0.628825	0.657089	875.0
GSVT	0.665511	0.675162	0.656175	691.0
SB	0.708462	0.708327	0.708600	1553.0
SR	0.690706	0.698734	0.682864	907.0
macro avg	0.676827	0.677762	0.676182	4026.0
micro avg	0.682609	0.682609	0.682609	4026.0
weighted avg	0.682782	0.683195	0.682609	4026.0

The result of Model Number:Dataset Number:Featuregroup Number:Rescale of 60 : 1 : 9 : 1

	f1-score	precision	recall	support
AFIB	0.714932	0.699655	0.730933	875.0
GSVT	0.740358	0.751679	0.729439	691.0
SB	0.789220	0.789324	0.789119	1553.0
SR	0.768888	0.776850	0.761090	907.0
macro avg	0.753350	0.754377	0.752645	4026.0
micro avg	0.759915	0.759915	0.759915	4026.0
weighted avg	0.760108	0.760564	0.759915	4026.0

The result of Model Number:Dataset Number:Featuregroup Number:Rescale of 60 : 1 : 10 : 0

	f1-score	precision	recall	support
AFIB	0.783697	0.790187	0.882727	204.0
GSVT	0.849866	0.813491	0.958848	300.0
SB	0.775959	0.773263	0.778677	892.0

SR 0.768244 0.768389 0.768104 681.0
macro avg 0.794442 0.786333 0.847089 2077.0
micro avg 0.811454 0.811454 0.811454 2077.0
weighted avg 0.784865 0.779138 0.811454 2077.0
The result of Model Number:Dataset Number:Featuregroup Number:Rescale of 60 : 1 : 10 : 1

	f1-score	precision	recall	support
AFIB	0.690603	0.679546	0.777068	204.0
GSVT	0.808514	0.799132	0.818413	300.0
SB	0.835586	0.832430	0.838770	892.0
SR	0.655616	0.659005	0.652372	681.0
macro avg	0.747579	0.742529	0.771656	2077.0
micro avg	0.768654	0.768654	0.768654	2077.0
weighted avg	0.758427	0.755743	0.768654	2077.0

The result of Model Number:Dataset Number:Featuregroup Number:Rescale of 61 : 0 : 0 : 0

	f1-score	precision	recall	support
AFIB	0.758149	0.767886	0.748693	875.0
GSVT	0.766536	0.760337	0.772960	691.0
SB	0.819974	0.817509	0.822457	1553.0
SR	0.799670	0.799334	0.800017	907.0
macro avg	0.786082	0.786266	0.786031	4026.0
micro avg	0.792874	0.792874	0.792874	4026.0
weighted avg	0.792791	0.792817	0.792874	4026.0

The result of Model Number:Dataset Number:Featuregroup Number:Rescale of 61 : 0 : 0 : 1

	f1-score	precision	recall	support
AFIB	0.837762	0.830353	0.845339	875.0
GSVT	0.761671	0.737620	0.847374	691.0
SB	0.686688	0.659315	0.729494	1553.0
SR	0.707731	0.715054	0.700602	907.0
macro avg	0.748463	0.735586	0.780702	4026.0
micro avg	0.768395	0.768395	0.768395	4026.0
weighted avg	0.737132	0.722485	0.768395	4026.0

The result of Model Number:Dataset Number:Featuregroup Number:Rescale of 61 : 0 : 1 : 0

	f1-score	precision	recall	support
AFIB	0.702790	0.774169	0.644137	204.0
GSVT	0.649023	0.693903	0.609899	300.0
SB	0.706593	0.660222	0.759981	892.0
SR	0.611568	0.643716	0.582647	681.0
macro avg	0.667494	0.693002	0.649166	2077.0
micro avg	0.668782	0.668782	0.668782	2077.0
weighted avg	0.666748	0.670867	0.668782	2077.0

The result of Model Number:Dataset Number:Featuregroup Number:Rescale of 61 : 0 : 1 : 1

	f1-score	precision	recall	support
AFIB	0.704459	0.697858	0.711213	875.0
GSVT	0.716299	0.722043	0.710716	691.0
SB	0.759314	0.756407	0.762248	1553.0
SR	0.742325	0.749860	0.734960	907.0
macro avg	0.730599	0.731542	0.729784	4026.0
micro avg	0.736164	0.736164	0.736164	4026.0
weighted avg	0.736182	0.736309	0.736164	4026.0

The result of Model Number:Dataset Number:Featuregroup Number:Rescale of 61 : 0 : 2 : 0

	f1-score	precision	recall	support
AFIB	0.675488	0.678705	0.672332	875.0
GSVT	0.683833	0.671405	0.696799	691.0
SB	0.723547	0.720776	0.726341	1553.0
SR	0.702161	0.714179	0.690551	907.0
macro avg	0.696257	0.696266	0.696506	4026.0
micro avg	0.701469	0.701469	0.701469	4026.0
weighted avg	0.701468	0.701673	0.701469	4026.0

The result of Model Number:Dataset Number:Featuregroup Number:Rescale of 61 : 0 : 2 : 1

	f1-score	precision	recall	support
AFIB	0.703144	0.776690	0.642527	204.0
GSVT	0.827200	0.816278	0.838507	300.0
SB	0.877243	0.873833	0.880684	892.0
SR	0.856163	0.843961	0.868732	681.0
macro avg	0.815937	0.827691	0.807612	2077.0
micro avg	0.847282	0.847282	0.847282	2077.0
weighted avg	0.846003	0.846184	0.847282	2077.0

The result of Model Number:Dataset Number:Featuregroup Number:Rescale of 61 : 0 : 3 : 0

	f1-score	precision	recall	support
AFIB	0.580522	0.652746	0.523201	204.0
GSVT	0.688747	0.674040	0.704141	300.0
SB	0.735250	0.731502	0.739041	892.0
SR	0.718756	0.709654	0.728140	681.0
macro avg	0.680819	0.691985	0.673631	2077.0
micro avg	0.709226	0.709226	0.709226	2077.0
weighted avg	0.707928	0.708303	0.709226	2077.0

The result of Model Number:Dataset Number:Featuregroup Number:Rescale of 61 : 0 : 3 : 1

	f1-score	precision	recall	support
AFIB	0.745045	0.744678	0.745469	875.0

GSVT	0.761218	0.757472	0.765048	691.0
SB	0.796247	0.791042	0.801527	1553.0
SR	0.773581	0.786181	0.761398	907.0
macro avg	0.769023	0.769843	0.768361	4026.0
micro avg	0.774042	0.774042	0.774042	4026.0
weighted avg	0.774000	0.774109	0.774042	4026.0

The result of Model Number:Dataset Number:Featuregroup Number:Rescale of 61 : 0 : 4 : 0

	f1-score	precision	recall	support
AFIB	0.729861	0.724929	0.734961	875.0
GSVT	0.747667	0.747965	0.747466	691.0
SB	0.794244	0.792217	0.796290	1553.0
SR	0.772618	0.781250	0.764185	907.0
macro avg	0.761098	0.761590	0.760725	4026.0
micro avg	0.767348	0.767348	0.767348	4026.0
weighted avg	0.767385	0.767527	0.767348	4026.0

The result of Model Number:Dataset Number:Featuregroup Number:Rescale of 61 : 0 : 4 : 1

	f1-score	precision	recall	support
AFIB	0.815830	0.820483	0.811365	204.0
GSVT	0.832616	0.826611	0.838718	300.0
SB	0.849432	0.846329	0.852572	892.0
SR	0.840398	0.845916	0.834973	681.0
macro avg	0.834569	0.834835	0.834407	2077.0
micro avg	0.840753	0.840753	0.840753	2077.0
weighted avg	0.840741	0.840807	0.840753	2077.0

The result of Model Number:Dataset Number:Featuregroup Number:Rescale of 61 : 0 : 5 : 0

	f1-score	precision	recall	support
AFIB	0.734327	0.748398	0.720963	204.0
GSVT	0.757679	0.753776	0.761692	300.0
SB	0.776417	0.775207	0.777635	892.0
SR	0.768766	0.768002	0.769555	681.0
macro avg	0.759297	0.761346	0.757461	2077.0
micro avg	0.767117	0.767117	0.767117	2077.0
weighted avg	0.767068	0.767116	0.767117	2077.0

The result of Model Number:Dataset Number:Featuregroup Number:Rescale of 61 : 0 : 5 : 1

	f1-score	precision	recall	support
AFIB	0.860929	0.928030	0.803276	204.0
GSVT	0.887183	0.902201	0.872694	300.0
SB	0.940247	0.931593	0.949091	892.0
SR	0.902824	0.889732	0.916349	681.0
macro avg	0.897795	0.912889	0.885353	2077.0

micro avg 0.912999 0.912999 0.912999 2077.0
weighted avg 0.912522 0.913272 0.912999 2077.0
The result of Model Number:Dataset Number:Featuregroup Number:Rescale of 61 : 0 : 6 : 0

	f1-score	precision	recall	support
AFIB	0.638523	0.706039	0.583124	204.0
GSVT	0.734704	0.726201	0.743549	300.0
SB	0.776787	0.773593	0.780009	892.0
SR	0.760105	0.748846	0.771716	681.0
macro avg	0.727530	0.738670	0.719599	2077.0
micro avg	0.752686	0.752686	0.752686	2077.0
weighted avg	0.751659	0.751999	0.752686	2077.0

The result of Model Number:Dataset Number:Featuregroup Number:Rescale of 61 : 0 : 6 : 1

	f1-score	precision	recall	support
AFIB	0.793691	0.784330	0.803319	875.0
GSVT	0.807136	0.812243	0.802122	691.0
SB	0.860563	0.859406	0.861727	1553.0
SR	0.839422	0.847374	0.831645	907.0
macro avg	0.825203	0.825838	0.824703	4026.0
micro avg	0.832025	0.832025	0.832025	4026.0
weighted avg	0.832097	0.832284	0.832025	4026.0

The result of Model Number:Dataset Number:Featuregroup Number:Rescale of 61 : 0 : 7 : 0

	f1-score	precision	recall	support
AFIB	0.774323	0.791849	0.757655	204.0
GSVT	0.794929	0.791037	0.798894	300.0
SB	0.811191	0.808848	0.813553	892.0
SR	0.805979	0.805631	0.806340	681.0
macro avg	0.796606	0.799341	0.794110	2077.0
micro avg	0.803580	0.803580	0.803580	2077.0
weighted avg	0.803512	0.803551	0.803580	2077.0

The result of Model Number:Dataset Number:Featuregroup Number:Rescale of 61 : 0 : 7 : 1

	f1-score	precision	recall	support
AFIB	0.817246	0.818662	0.815848	875.0
GSVT	0.827155	0.817651	0.836952	691.0
SB	0.871023	0.860520	0.881789	1553.0
SR	0.829621	0.854758	0.805939	907.0
macro avg	0.836262	0.837898	0.835132	4026.0
micro avg	0.842674	0.842674	0.842674	4026.0
weighted avg	0.842479	0.842767	0.842674	4026.0

The result of Model Number:Dataset Number:Featuregroup Number:Rescale of 61 : 0 : 8 : 0

	f1-score	precision	recall	support
AFIB	0.912616	0.942258	0.884962	204.0
GSVT	0.937813	0.925855	0.950272	300.0
SB	0.964271	0.960635	0.967945	892.0
SR	0.950784	0.952506	0.949105	681.0
macro avg	0.941371	0.945313	0.938071	2077.0
micro avg	0.951065	0.951065	0.951065	2077.0
weighted avg	0.950954	0.951141	0.951065	2077.0

The result of Model Number:Dataset Number:Featuregroup Number:Rescale of 61 : 0 : 8 : 1

	f1-score	precision	recall	support
AFIB	0.725246	0.723015	0.727536	875.0
GSVT	0.706758	0.710224	0.703438	691.0
SB	0.743001	0.712168	0.776632	1553.0
SR	0.639414	0.697637	0.590211	907.0
macro avg	0.703605	0.710761	0.699454	4026.0
micro avg	0.711401	0.711401	0.711401	4026.0
weighted avg	0.709585	0.710918	0.711401	4026.0

The result of Model Number:Dataset Number:Featuregroup Number:Rescale of 61 : 0 : 9 : 0

	f1-score	precision	recall	support
AFIB	0.770329	0.780530	0.760507	204.0
GSVT	0.793575	0.790711	0.796478	300.0
SB	0.803460	0.800959	0.805979	892.0
SR	0.794705	0.796236	0.793202	681.0
macro avg	0.790517	0.792109	0.789042	2077.0
micro avg	0.795951	0.795951	0.795951	2077.0
weighted avg	0.795907	0.795924	0.795951	2077.0

The result of Model Number:Dataset Number:Featuregroup Number:Rescale of 61 : 0 : 9 : 1

	f1-score	precision	recall	support
AFIB	0.696834	0.712357	0.682173	204.0
GSVT	0.705431	0.695099	0.716368	300.0
SB	0.721372	0.714203	0.728717	892.0
SR	0.708065	0.718235	0.698201	681.0
macro avg	0.707925	0.709973	0.706365	2077.0
micro avg	0.712356	0.712356	0.712356	2077.0
weighted avg	0.712296	0.712584	0.712356	2077.0

The result of Model Number:Dataset Number:Featuregroup Number:Rescale of 61 : 0 : 10 : 0

	f1-score	precision	recall	support
AFIB	0.717120	0.708117	0.726564	875.0
GSVT	0.665346	0.685490	0.648576	691.0
SB	0.716350	0.663499	0.780245	1553.0

SR 0.556985 0.681707 0.474953 907.0
macro avg 0.663950 0.684703 0.657585 4026.0
micro avg 0.677201 0.677201 0.677201 4026.0
weighted avg 0.671861 0.681073 0.677201 4026.0
The result of Model Number:Dataset Number:Featuregroup Number:Rescale of 61 : 0 : 10 : 1

	f1-score	precision	recall	support
AFIB	0.737274	0.746743	0.728079	875.0
GSVT	0.745430	0.739402	0.751677	691.0
SB	0.797397	0.795000	0.799811	1553.0
SR	0.777652	0.777325	0.777990	907.0
macro avg	0.764438	0.764618	0.764389	4026.0
micro avg	0.771044	0.771044	0.771044	4026.0
weighted avg	0.770963	0.770988	0.771044	4026.0

The result of Model Number:Dataset Number:Featuregroup Number:Rescale of 61 : 1 : 0 : 0

	f1-score	precision	recall	support
AFIB	0.744219	0.836808	0.670734	204.0
GSVT	0.882961	0.864107	0.902696	300.0
SB	0.942578	0.937773	0.947437	892.0
SR	0.921432	0.909763	0.933463	681.0
macro avg	0.872797	0.887113	0.863582	2077.0
micro avg	0.909215	0.909215	0.909215	2077.0
weighted avg	0.907551	0.908032	0.909215	2077.0

The result of Model Number:Dataset Number:Featuregroup Number:Rescale of 61 : 1 : 0 : 1

	f1-score	precision	recall	support
AFIB	0.634374	0.628262	0.640703	875.0
GSVT	0.650994	0.652856	0.649210	691.0
SB	0.692119	0.690527	0.693723	1553.0
SR	0.672649	0.680564	0.664931	907.0
macro avg	0.662534	0.663052	0.662142	4026.0
micro avg	0.668073	0.668073	0.668073	4026.0
weighted avg	0.668124	0.668285	0.668073	4026.0

The result of Model Number:Dataset Number:Featuregroup Number:Rescale of 61 : 1 : 1 : 0

	f1-score	precision	recall	support
AFIB	0.755667	0.746180	0.765618	875.0
GSVT	0.701109	0.722337	0.683438	691.0
SB	0.754856	0.699163	0.822185	1553.0
SR	0.586924	0.718351	0.500483	907.0
macro avg	0.699639	0.721507	0.692931	4026.0
micro avg	0.713602	0.713602	0.713602	4026.0
weighted avg	0.707975	0.717682	0.713602	4026.0

The result of Model Number:Dataset Number:Featuregroup Number:Rescale of 61 : 1 : 1 : 1

	f1-score	precision	recall	support
AFIB	0.716385	0.748734	0.686949	204.0
GSVT	0.741301	0.732976	0.749953	300.0
SB	0.713104	0.682014	0.763032	892.0
SR	0.594114	0.599179	0.589234	681.0
macro avg	0.691226	0.690726	0.697292	2077.0
micro avg	0.696686	0.696686	0.696686	2077.0
weighted avg	0.678485	0.668768	0.696686	2077.0

The result of Model Number:Dataset Number:Featuregroup Number:Rescale of 61 : 1 : 2 : 0

	f1-score	precision	recall	support
AFIB	0.746065	0.747560	0.744608	875.0
GSVT	0.747913	0.743875	0.752014	691.0
SB	0.781820	0.777643	0.786047	1553.0
SR	0.763116	0.772166	0.754297	907.0
macro avg	0.759728	0.760311	0.759242	4026.0
micro avg	0.764047	0.764047	0.764047	4026.0
weighted avg	0.764016	0.764075	0.764047	4026.0

The result of Model Number:Dataset Number:Featuregroup Number:Rescale of 61 : 1 : 2 : 1

	f1-score	precision	recall	support
AFIB	0.886639	0.895307	0.878345	875.0
GSVT	0.879867	0.873191	0.886935	691.0
SB	0.930999	0.921420	0.940861	1553.0
SR	0.901448	0.915945	0.887657	907.0
macro avg	0.899738	0.901466	0.898449	4026.0
micro avg	0.906032	0.906032	0.906032	4026.0
weighted avg	0.905924	0.906233	0.906032	4026.0

The result of Model Number:Dataset Number:Featuregroup Number:Rescale of 61 : 1 : 3 : 0

	f1-score	precision	recall	support
AFIB	0.864583	0.923186	0.813551	204.0
GSVT	0.814642	0.838812	0.792131	300.0
SB	0.851504	0.810628	0.897207	892.0
SR	0.755553	0.789856	0.724929	681.0
macro avg	0.821570	0.840620	0.806954	2077.0
micro avg	0.817327	0.817327	0.817327	2077.0
weighted avg	0.816004	0.818943	0.817327	2077.0

The result of Model Number:Dataset Number:Featuregroup Number:Rescale of 61 : 1 : 3 : 1

	f1-score	precision	recall	support
AFIB	0.841135	0.852370	0.830429	204.0

GSVT 0.855994 0.850780 0.861404 300.0
SB 0.876628 0.875851 0.877413 892.0
SR 0.870151 0.870283 0.870024 681.0
macro avg 0.860977 0.862321 0.859818 2077.0
micro avg 0.868063 0.868063 0.868063 2077.0
weighted avg 0.868038 0.868098 0.868063 2077.0
The result of Model Number:Dataset Number:Featuregroup Number:Rescale of 61 : 1 : 4 : 0

	f1-score	precision	recall	support
AFIB	0.723746	0.730796	0.717007	204.0
GSVT	0.740483	0.737083	0.743939	300.0
SB	0.750716	0.748133	0.753322	892.0
SR	0.744376	0.747264	0.741545	681.0
macro avg	0.739830	0.740819	0.738953	2077.0
micro avg	0.744539	0.744539	0.744539	2077.0
weighted avg	0.744510	0.744549	0.744539	2077.0

The result of Model Number:Dataset Number:Featuregroup Number:Rescale of 61 : 1 : 4 : 1

	f1-score	precision	recall	support
AFIB	0.812736	0.837566	0.789426	875.0
GSVT	0.862487	0.859566	0.865504	691.0
SB	0.942478	0.938692	0.946300	1553.0
SR	0.892632	0.877149	0.908759	907.0
macro avg	0.877583	0.878243	0.877497	4026.0
micro avg	0.889880	0.889880	0.889880	4026.0
weighted avg	0.889321	0.889268	0.889880	4026.0

The result of Model Number:Dataset Number:Featuregroup Number:Rescale of 61 : 1 : 5 : 0

	f1-score	precision	recall	support
AFIB	0.629133	0.610329	0.670820	875.0
GSVT	0.538759	0.526961	0.551297	691.0
SB	0.714600	0.708778	0.720709	1553.0
SR	0.670398	0.698544	0.645690	907.0
macro avg	0.638223	0.636153	0.647129	4026.0
micro avg	0.663889	0.663889	0.663889	4026.0
weighted avg	0.655887	0.653870	0.663889	4026.0

The result of Model Number:Dataset Number:Featuregroup Number:Rescale of 61 : 1 : 5 : 1

	f1-score	precision	recall	support
AFIB	0.633688	0.638287	0.629200	875.0
GSVT	0.647588	0.641569	0.653770	691.0
SB	0.695967	0.691766	0.700225	1553.0
SR	0.673158	0.680674	0.665808	907.0
macro avg	0.662600	0.663074	0.662251	4026.0

micro avg 0.669061 0.669061 0.669061 4026.0
weighted avg 0.668989 0.669028 0.669061 4026.0
The result of Model Number:Dataset Number:Featuregroup Number:Rescale of 61 : 1 : 6 : 0

	f1-score	precision	recall	support
AFIB	0.901895	0.926757	0.878512	204.0
GSVT	0.925897	0.908757	0.943784	300.0
SB	0.940913	0.932117	0.949879	892.0
SR	0.917263	0.929724	0.905146	681.0
macro avg	0.921492	0.924339	0.919330	2077.0
micro avg	0.927322	0.927322	0.927322	2077.0
weighted avg	0.927157	0.927432	0.927322	2077.0

The result of Model Number:Dataset Number:Featuregroup Number:Rescale of 61 : 1 : 6 : 1

	f1-score	precision	recall	support
AFIB	0.677496	0.675538	0.679528	875.0
GSVT	0.690053	0.689492	0.690663	691.0
SB	0.732936	0.729901	0.736003	1553.0
SR	0.710933	0.718743	0.703329	907.0
macro avg	0.702855	0.703418	0.702381	4026.0
micro avg	0.708586	0.708586	0.708586	4026.0
weighted avg	0.708570	0.708636	0.708586	4026.0

The result of Model Number:Dataset Number:Featuregroup Number:Rescale of 61 : 1 : 7 : 0

	f1-score	precision	recall	support
AFIB	0.777345	0.802262	0.754162	204.0
GSVT	0.800601	0.786827	0.814984	300.0
SB	0.814450	0.806399	0.822666	892.0
SR	0.792673	0.802593	0.783014	681.0
macro avg	0.796267	0.799520	0.793706	2077.0
micro avg	0.801827	0.801827	0.801827	2077.0
weighted avg	0.801665	0.801918	0.801827	2077.0

The result of Model Number:Dataset Number:Featuregroup Number:Rescale of 61 : 1 : 7 : 1

	f1-score	precision	recall	support
AFIB	0.763023	0.777045	0.749591	204.0
GSVT	0.775839	0.770758	0.781008	300.0
SB	0.789110	0.785258	0.793003	892.0
SR	0.780954	0.784201	0.777745	681.0
macro avg	0.777232	0.779315	0.775337	2077.0
micro avg	0.782004	0.782004	0.782004	2077.0
weighted avg	0.781957	0.782010	0.782004	2077.0

The result of Model Number:Dataset Number:Featuregroup Number:Rescale of 61 : 1 : 8 : 0

	f1-score	precision	recall	support
AFIB	0.699128	0.690202	0.708329	875.0
GSVT	0.711399	0.715479	0.707385	691.0
SB	0.761509	0.758311	0.764742	1553.0
SR	0.739087	0.751050	0.727512	907.0
macro avg	0.727781	0.728761	0.726992	4026.0
micro avg	0.734250	0.734250	0.734250	4026.0
weighted avg	0.734299	0.734521	0.734250	4026.0

The result of Model Number:Dataset Number:Featuregroup Number:Rescale of 61 : 1 : 8 : 1

	f1-score	precision	recall	support
AFIB	0.922799	0.941392	0.905520	204.0
GSVT	0.952409	0.944760	0.960367	300.0
SB	0.973028	0.972048	0.974010	892.0
SR	0.964158	0.963605	0.964724	681.0
macro avg	0.953099	0.955451	0.951155	2077.0
micro avg	0.962268	0.962268	0.962268	2077.0
weighted avg	0.962208	0.962328	0.962268	2077.0

The result of Model Number:Dataset Number:Featuregroup Number:Rescale of 61 : 1 : 9 : 0

	f1-score	precision	recall	support
AFIB	0.747003	0.766559	0.728761	204.0
GSVT	0.702878	0.723306	0.683955	300.0
SB	0.741687	0.715574	0.769792	892.0
SR	0.672249	0.694142	0.651830	681.0
macro avg	0.715954	0.724895	0.708584	2077.0
micro avg	0.714687	0.714687	0.714687	2077.0
weighted avg	0.713836	0.714671	0.714687	2077.0

The result of Model Number:Dataset Number:Featuregroup Number:Rescale of 61 : 1 : 9 : 1

	f1-score	precision	recall	support
AFIB	0.624824	0.622902	0.626797	875.0
GSVT	0.608896	0.611882	0.606036	691.0
SB	0.640120	0.613556	0.669094	1553.0
SR	0.550877	0.601037	0.508487	907.0
macro avg	0.606179	0.612344	0.602603	4026.0
micro avg	0.612896	0.612896	0.612896	4026.0
weighted avg	0.611331	0.612480	0.612896	4026.0

The result of Model Number:Dataset Number:Featuregroup Number:Rescale of 61 : 1 : 10 : 0

	f1-score	precision	recall	support
AFIB	0.791633	0.803635	0.780004	204.0
GSVT	0.804362	0.797581	0.811301	300.0
SB	0.822204	0.819551	0.824875	892.0

SR	0.813378	0.816399	0.810401	681.0
macro avg	0.807894	0.809292	0.806645	2077.0
micro avg	0.813762	0.813762	0.813762	2077.0
weighted avg	0.813730	0.813781	0.813762	2077.0

The result of Model Number:Dataset Number:Featuregroup Number:Rescale of 61 : 1 : 10 : 1

	f1-score	precision	recall	support
AFIB	0.780288	0.780039	0.780579	875.0
GSVT	0.791917	0.781738	0.802458	691.0
SB	0.837642	0.835820	0.839476	1553.0
SR	0.814466	0.826405	0.802881	907.0
macro avg	0.806078	0.806000	0.806348	4026.0
micro avg	0.812077	0.812077	0.812077	4026.0
weighted avg	0.812108	0.812293	0.812077	4026.0

The result of Model Number:Dataset Number:Featuregroup Number:Rescale of 62 : 0 : 0 : 0

	f1-score	precision	recall	support
AFIB	0.697427	0.697551	0.697333	875.0
GSVT	0.704805	0.707186	0.702485	691.0
SB	0.740362	0.737809	0.742936	1553.0
SR	0.726303	0.728753	0.723896	907.0
macro avg	0.717224	0.717825	0.716662	4026.0
micro avg	0.721792	0.721792	0.721792	4026.0
weighted avg	0.721760	0.721763	0.721792	4026.0

The result of Model Number:Dataset Number:Featuregroup Number:Rescale of 62 : 0 : 0 : 1

	f1-score	precision	recall	support
AFIB	0.818187	0.836689	0.800510	875.0
GSVT	0.867464	0.871156	0.863854	691.0
SB	0.941886	0.937447	0.946373	1553.0
SR	0.892424	0.878735	0.906583	907.0
macro avg	0.879990	0.881007	0.879330	4026.0
micro avg	0.891544	0.891544	0.891544	4026.0
weighted avg	0.891085	0.890944	0.891544	4026.0

The result of Model Number:Dataset Number:Featuregroup Number:Rescale of 62 : 0 : 1 : 0

	f1-score	precision	recall	support
AFIB	0.908576	0.928816	0.889460	204.0
GSVT	0.919785	0.906314	0.934046	300.0
SB	0.940570	0.931223	0.950148	892.0
SR	0.923220	0.936480	0.910358	681.0
macro avg	0.923038	0.925708	0.921003	2077.0
micro avg	0.928815	0.928815	0.928815	2077.0
weighted avg	0.928737	0.929112	0.928815	2077.0

The result of Model Number:Dataset Number:Featuregroup Number:Rescale of 62 : 0 : 1 : 1

	f1-score	precision	recall	support
AFIB	0.763098	0.845326	0.696061	204.0
GSVT	0.899493	0.886885	0.912697	300.0
SB	0.955044	0.951969	0.958148	892.0
SR	0.930592	0.916437	0.945215	681.0
macro avg	0.887057	0.900154	0.878030	2077.0
micro avg	0.921601	0.921601	0.921601	2077.0
weighted avg	0.920150	0.920444	0.921601	2077.0

The result of Model Number:Dataset Number:Featuregroup Number:Rescale of 62 : 0 : 2 : 0

	f1-score	precision	recall	support
AFIB	0.726868	0.723208	0.730597	875.0
GSVT	0.741025	0.744613	0.737484	691.0
SB	0.789237	0.787421	0.791065	1553.0
SR	0.769479	0.773596	0.765414	907.0
macro avg	0.756652	0.757209	0.756140	4026.0
micro avg	0.762948	0.762948	0.762948	4026.0
weighted avg	0.762956	0.763003	0.762948	4026.0

The result of Model Number:Dataset Number:Featuregroup Number:Rescale of 62 : 0 : 2 : 1

	f1-score	precision	recall	support
AFIB	0.642062	0.652488	0.631998	875.0
GSVT	0.682852	0.684991	0.680766	691.0
SB	0.742908	0.740348	0.745490	1553.0
SR	0.703317	0.695484	0.711372	907.0
macro avg	0.692785	0.693328	0.692407	4026.0
micro avg	0.702029	0.702029	0.702029	4026.0
weighted avg	0.701763	0.701644	0.702029	4026.0

The result of Model Number:Dataset Number:Featuregroup Number:Rescale of 62 : 0 : 3 : 0

	f1-score	precision	recall	support
AFIB	0.660524	0.660618	0.660516	875.0
GSVT	0.672060	0.666489	0.677775	691.0
SB	0.703638	0.697551	0.709843	1553.0
SR	0.680451	0.695823	0.665768	907.0
macro avg	0.679168	0.680120	0.678475	4026.0
micro avg	0.683689	0.683689	0.683689	4026.0
weighted avg	0.683624	0.683803	0.683689	4026.0

The result of Model Number:Dataset Number:Featuregroup Number:Rescale of 62 : 0 : 3 : 1

	f1-score	precision	recall	support
AFIB	0.864543	0.845971	0.883996	875.0

GSVT	0.895325	0.908309	0.882766	691.0
SB	0.953109	0.952928	0.953294	1553.0
SR	0.929221	0.940021	0.918671	907.0
macro avg	0.910550	0.911807	0.909682	4026.0
micro avg	0.918328	0.918328	0.918328	4026.0
weighted avg	0.918561	0.919116	0.918328	4026.0

The result of Model Number:Dataset Number:Featuregroup Number:Rescale of 62 : 0 : 4 : 0

	f1-score	precision	recall	support
AFIB	0.675208	0.744611	0.618055	204.0
GSVT	0.803478	0.792620	0.814718	300.0
SB	0.855647	0.852225	0.859100	892.0
SR	0.832423	0.821171	0.844025	681.0
macro avg	0.791689	0.802657	0.783974	2077.0
micro avg	0.824071	0.824071	0.824071	2077.0
weighted avg	0.822775	0.822864	0.824071	2077.0

The result of Model Number:Dataset Number:Featuregroup Number:Rescale of 62 : 0 : 4 : 1

	f1-score	precision	recall	support
AFIB	0.775284	0.782323	0.768769	204.0
GSVT	0.800908	0.796068	0.806017	300.0
SB	0.817664	0.816568	0.818764	892.0
SR	0.810098	0.811778	0.808431	681.0
macro avg	0.800989	0.801684	0.800495	2077.0
micro avg	0.808624	0.808624	0.808624	2077.0
weighted avg	0.808601	0.808673	0.808624	2077.0

The result of Model Number:Dataset Number:Featuregroup Number:Rescale of 62 : 0 : 5 : 0

	f1-score	precision	recall	support
AFIB	0.774119	0.789120	0.760026	204.0
GSVT	0.804138	0.797414	0.811124	300.0
SB	0.819142	0.817498	0.820795	892.0
SR	0.811331	0.812172	0.810496	681.0
macro avg	0.802182	0.804051	0.800610	2077.0
micro avg	0.810052	0.810052	0.810052	2077.0
weighted avg	0.809992	0.810063	0.810052	2077.0

The result of Model Number:Dataset Number:Featuregroup Number:Rescale of 62 : 0 : 5 : 1

	f1-score	precision	recall	support
AFIB	0.648854	0.676956	0.623177	204.0
GSVT	0.606566	0.612226	0.601313	300.0
SB	0.628749	0.597837	0.663077	892.0
SR	0.560386	0.595751	0.529166	681.0
macro avg	0.611139	0.620693	0.604183	2077.0

micro avg 0.606331 0.606331 0.606331 2077.0

weighted avg 0.605105 0.607002 0.606331 2077.0

The result of Model Number:Dataset Number:Featuregroup Number:Rescale of 62 : 0 : 6 : 0

	f1-score	precision	recall	support
AFIB	0.681127	0.697472	0.665841	204.0
GSVT	0.705346	0.701258	0.709568	300.0
SB	0.720410	0.717767	0.723074	892.0
SR	0.714150	0.714679	0.713629	681.0
macro avg	0.705258	0.707794	0.703028	2077.0
micro avg	0.712405	0.712405	0.712405	2077.0
weighted avg	0.712323	0.712377	0.712405	2077.0

The result of Model Number:Dataset Number:Featuregroup Number:Rescale of 62 : 0 : 6 : 1

	f1-score	precision	recall	support
AFIB	0.819276	0.827201	0.811650	204.0
GSVT	0.848267	0.846594	0.849960	300.0
SB	0.865997	0.864463	0.867548	892.0
SR	0.858014	0.858429	0.857635	681.0
macro avg	0.847888	0.849172	0.846698	2077.0
micro avg	0.856267	0.856267	0.856267	2077.0
weighted avg	0.856230	0.856244	0.856267	2077.0

The result of Model Number:Dataset Number:Featuregroup Number:Rescale of 62 : 0 : 7 : 0

	f1-score	precision	recall	support
AFIB	0.648567	0.623773	0.689364	875.0
GSVT	0.672026	0.648832	0.703485	691.0
SB	0.748761	0.735545	0.763284	1553.0
SR	0.634184	0.762569	0.547532	907.0
macro avg	0.675885	0.692680	0.675916	4026.0
micro avg	0.688349	0.688349	0.688349	4026.0
weighted avg	0.688002	0.702458	0.688349	4026.0

The result of Model Number:Dataset Number:Featuregroup Number:Rescale of 62 : 0 : 7 : 1

	f1-score	precision	recall	support
AFIB	0.790215	0.861783	0.730939	204.0
GSVT	0.917188	0.906708	0.928197	300.0
SB	0.972624	0.970134	0.975132	892.0
SR	0.948810	0.935962	0.962043	681.0
macro avg	0.907209	0.918647	0.899078	2077.0
micro avg	0.940077	0.940077	0.940077	2077.0
weighted avg	0.938893	0.939127	0.940077	2077.0

The result of Model Number:Dataset Number:Featuregroup Number:Rescale of 62 : 0 : 8 : 0

	f1-score	precision	recall	support
AFIB	0.712204	0.760479	0.670166	204.0
GSVT	0.671065	0.690976	0.652522	300.0
SB	0.701430	0.667759	0.739078	892.0
SR	0.622390	0.650647	0.597164	681.0
macro avg	0.676773	0.692465	0.664733	2077.0
micro avg	0.673277	0.673277	0.673277	2077.0
weighted avg	0.672187	0.674609	0.673277	2077.0

The result of Model Number:Dataset Number:Featuregroup Number:Rescale of 62 : 0 : 8 : 1

	f1-score	precision	recall	support
AFIB	0.739336	0.737287	0.741452	875.0
GSVT	0.745187	0.748741	0.741740	691.0
SB	0.780080	0.777587	0.782591	1553.0
SR	0.767608	0.771316	0.763972	907.0
macro avg	0.758052	0.758732	0.757439	4026.0
micro avg	0.762444	0.762444	0.762444	4026.0
weighted avg	0.762426	0.762464	0.762444	4026.0

The result of Model Number:Dataset Number:Featuregroup Number:Rescale of 62 : 0 : 9 : 0

	f1-score	precision	recall	support
AFIB	0.688005	0.689196	0.686827	875.0
GSVT	0.696347	0.688345	0.704594	691.0
SB	0.733277	0.724435	0.742340	1553.0
SR	0.698422	0.719584	0.678485	907.0
macro avg	0.704013	0.705390	0.703061	4026.0
micro avg	0.709411	0.709411	0.709411	4026.0
weighted avg	0.709247	0.709489	0.709411	4026.0

The result of Model Number:Dataset Number:Featuregroup Number:Rescale of 62 : 0 : 9 : 1

	f1-score	precision	recall	support
AFIB	0.857913	0.863918	0.852023	204.0
GSVT	0.877874	0.873546	0.882258	300.0
SB	0.895322	0.893832	0.896828	892.0
SR	0.886411	0.888515	0.884327	681.0
macro avg	0.879380	0.879953	0.878859	2077.0
micro avg	0.886224	0.886224	0.886224	2077.0
weighted avg	0.886206	0.886220	0.886224	2077.0

The result of Model Number:Dataset Number:Featuregroup Number:Rescale of 62 : 0 : 10 : 0

	f1-score	precision	recall	support
AFIB	0.849157	0.851266	0.847116	875.0
GSVT	0.863781	0.866376	0.861310	691.0
SB	0.916316	0.910096	0.922627	1553.0

SR 0.898879 0.905552 0.892338 907.0
macro avg 0.882033 0.883322 0.880848 4026.0
micro avg 0.888868 0.888868 0.888868 4026.0
weighted avg 0.888775 0.888782 0.888868 4026.0
The result of Model Number:Dataset Number:Featuregroup Number:Rescale of 62 : 0 : 10 : 1

	f1-score	precision	recall	support
AFIB	0.815905	0.806032	0.826060	875.0
GSVT	0.826950	0.835428	0.818706	691.0
SB	0.878304	0.875446	0.881188	1553.0
SR	0.859093	0.867921	0.850474	907.0
macro avg	0.845063	0.846207	0.844107	4026.0
micro avg	0.851563	0.851563	0.851563	4026.0
weighted avg	0.851601	0.851796	0.851563	4026.0

The result of Model Number:Dataset Number:Featuregroup Number:Rescale of 62 : 1 : 0 : 0

	f1-score	precision	recall	support
AFIB	0.634978	0.643724	0.626745	875.0
GSVT	0.648840	0.646153	0.651725	691.0
SB	0.691116	0.682055	0.700450	1553.0
SR	0.669418	0.679415	0.660008	907.0
macro avg	0.661088	0.662837	0.659732	4026.0
micro avg	0.666957	0.666957	0.666957	4026.0
weighted avg	0.666771	0.666967	0.666957	4026.0

The result of Model Number:Dataset Number:Featuregroup Number:Rescale of 62 : 1 : 0 : 1

	f1-score	precision	recall	support
AFIB	0.685002	0.703885	0.667242	204.0
GSVT	0.703232	0.690214	0.716817	300.0
SB	0.714637	0.707956	0.721447	892.0
SR	0.696674	0.706139	0.687471	681.0
macro avg	0.699886	0.702048	0.698244	2077.0
micro avg	0.704314	0.704314	0.704314	2077.0
weighted avg	0.704189	0.704397	0.704314	2077.0

The result of Model Number:Dataset Number:Featuregroup Number:Rescale of 62 : 1 : 1 : 0

	f1-score	precision	recall	support
AFIB	0.714886	0.721246	0.708675	875.0
GSVT	0.729891	0.721881	0.738128	691.0
SB	0.784994	0.778882	0.791209	1553.0
SR	0.758907	0.769664	0.748450	907.0
macro avg	0.747170	0.747919	0.746615	4026.0
micro avg	0.754528	0.754528	0.754528	4026.0
weighted avg	0.754422	0.754496	0.754528	4026.0

The result of Model Number:Dataset Number:Featuregroup Number:Rescale of 62 : 1 : 1 : 1

	f1-score	precision	recall	support
AFIB	0.737036	0.814968	0.673090	204.0
GSVT	0.848056	0.838241	0.858266	300.0
SB	0.896632	0.892945	0.900351	892.0
SR	0.877376	0.864381	0.890778	681.0
macro avg	0.839775	0.852634	0.830621	2077.0
micro avg	0.868812	0.868812	0.868812	2077.0
weighted avg	0.867627	0.868019	0.868812	2077.0

The result of Model Number:Dataset Number:Featuregroup Number:Rescale of 62 : 1 : 2 : 0

	f1-score	precision	recall	support
AFIB	0.875299	0.898215	0.853924	204.0
GSVT	0.823596	0.847533	0.801423	300.0
SB	0.869070	0.838472	0.902002	892.0
SR	0.787706	0.813359	0.763780	681.0
macro avg	0.838918	0.849395	0.830282	2077.0
micro avg	0.837433	0.837433	0.837433	2077.0
weighted avg	0.836437	0.837415	0.837433	2077.0

The result of Model Number:Dataset Number:Featuregroup Number:Rescale of 62 : 1 : 2 : 1

	f1-score	precision	recall	support
AFIB	0.679650	0.693964	0.666021	204.0
GSVT	0.700781	0.694379	0.707319	300.0
SB	0.712226	0.710638	0.713823	892.0
SR	0.703762	0.704498	0.703036	681.0
macro avg	0.699105	0.700870	0.697550	2077.0
micro avg	0.704652	0.704652	0.704652	2077.0
weighted avg	0.704598	0.704639	0.704652	2077.0

The result of Model Number:Dataset Number:Featuregroup Number:Rescale of 62 : 1 : 3 : 0

	f1-score	precision	recall	support
AFIB	0.549768	0.598886	0.580567	204.0
GSVT	0.746737	0.734530	0.759614	300.0
SB	0.635704	0.630862	0.640668	892.0
SR	0.777362	0.768567	0.786385	681.0
macro avg	0.677393	0.683211	0.691808	2077.0
micro avg	0.699723	0.699723	0.699723	2077.0
weighted avg	0.689747	0.687845	0.699723	2077.0

The result of Model Number:Dataset Number:Featuregroup Number:Rescale of 62 : 1 : 3 : 1

	f1-score	precision	recall	support
AFIB	0.821817	0.841489	0.804246	204.0

GSVT 0.846017 0.834097 0.858637 300.0
SB 0.867999 0.859181 0.877040 892.0
SR 0.847253 0.859486 0.835430 681.0
macro avg 0.845771 0.848563 0.843838 2077.0
micro avg 0.853589 0.853589 0.853589 2077.0
weighted avg 0.853486 0.853920 0.853589 2077.0
The result of Model Number:Dataset Number:Featuregroup Number:Rescale of 62 : 1 : 4 : 0
f1-score precision recall support
AFIB 0.688829 0.691405 0.686564 875.0
GSVT 0.704334 0.711129 0.697809 691.0
SB 0.748927 0.741072 0.757008 1553.0
SR 0.722993 0.729356 0.716988 907.0
macro avg 0.716271 0.718240 0.714592 4026.0
micro avg 0.722522 0.722522 0.722522 4026.0
weighted avg 0.722369 0.722499 0.722522 4026.0
The result of Model Number:Dataset Number:Featuregroup Number:Rescale of 62 : 1 : 4 : 1
f1-score precision recall support
AFIB 0.821368 0.825120 0.817696 875.0
GSVT 0.835061 0.827999 0.842306 691.0
SB 0.882677 0.873663 0.891883 1553.0
SR 0.837121 0.854769 0.820233 907.0
macro avg 0.844057 0.845388 0.843029 4026.0
micro avg 0.851108 0.851108 0.851108 4026.0
weighted avg 0.850917 0.851019 0.851108 4026.0
The result of Model Number:Dataset Number:Featuregroup Number:Rescale of 62 : 1 : 5 : 0
f1-score precision recall support
AFIB 0.695820 0.697165 0.694534 875.0
GSVT 0.674133 0.671171 0.677161 691.0
SB 0.704388 0.661575 0.753145 1553.0
SR 0.566934 0.656911 0.498770 907.0
macro avg 0.660319 0.671706 0.655902 4026.0
micro avg 0.670058 0.670058 0.670058 4026.0
weighted avg 0.666367 0.669907 0.670058 4026.0
The result of Model Number:Dataset Number:Featuregroup Number:Rescale of 62 : 1 : 5 : 1
f1-score precision recall support
AFIB 0.725716 0.708802 0.743575 875.0
GSVT 0.696662 0.704320 0.689216 691.0
SB 0.733125 0.685785 0.787504 1553.0
SR 0.586753 0.704576 0.503003 907.0
macro avg 0.685564 0.700871 0.680824 4026.0

micro avg 0.696993 0.696993 0.696993 4026.0
weighted avg 0.692281 0.698202 0.696993 4026.0
The result of Model Number:Dataset Number:Featuregroup Number:Rescale of 62 : 1 : 6 : 0

	f1-score	precision	recall	support
AFIB	0.740048	0.745733	0.734618	204.0
GSVT	0.766109	0.765375	0.766870	300.0
SB	0.781783	0.779962	0.783620	892.0
SR	0.774588	0.775632	0.773564	681.0
macro avg	0.765632	0.766676	0.764668	2077.0
micro avg	0.773091	0.773091	0.773091	2077.0
weighted avg	0.773061	0.773074	0.773091	2077.0

The result of Model Number:Dataset Number:Featuregroup Number:Rescale of 62 : 1 : 6 : 1

	f1-score	precision	recall	support
AFIB	0.701421	0.756090	0.654450	204.0
GSVT	0.722811	0.735046	0.711007	300.0
SB	0.766043	0.758993	0.773250	892.0
SR	0.735554	0.724888	0.746573	681.0
macro avg	0.731457	0.743754	0.721320	2077.0
micro avg	0.743844	0.743844	0.743844	2077.0
weighted avg	0.743455	0.744067	0.743844	2077.0

The result of Model Number:Dataset Number:Featuregroup Number:Rescale of 62 : 1 : 7 : 0

	f1-score	precision	recall	support
AFIB	0.756625	0.735640	0.781225	875.0
GSVT	0.766503	0.798789	0.741441	691.0
SB	0.842030	0.841604	0.842467	1553.0
SR	0.820906	0.826043	0.815838	907.0
macro avg	0.796516	0.800519	0.795243	4026.0
micro avg	0.805818	0.805818	0.805818	4026.0
weighted avg	0.805746	0.807720	0.805818	4026.0

The result of Model Number:Dataset Number:Featuregroup Number:Rescale of 62 : 1 : 7 : 1

	f1-score	precision	recall	support
AFIB	0.671149	0.692948	0.650812	204.0
GSVT	0.689679	0.680885	0.698842	300.0
SB	0.709136	0.706462	0.711839	892.0
SR	0.699218	0.700484	0.697984	681.0
macro avg	0.692296	0.695195	0.689869	2077.0
micro avg	0.699425	0.699425	0.699425	2077.0
weighted avg	0.699343	0.699481	0.699425	2077.0

The result of Model Number:Dataset Number:Featuregroup Number:Rescale of 62 : 1 : 8 : 0

f1-score precision recall support
AFIB 0.736534 0.747827 0.725698 204.0
GSVT 0.753998 0.747601 0.760537 300.0
SB 0.767114 0.764639 0.769608 892.0
SR 0.759231 0.762050 0.756443 681.0
macro avg 0.754219 0.755529 0.753072 2077.0
micro avg 0.759669 0.759669 0.759669 2077.0
weighted avg 0.759631 0.759678 0.759669 2077.0
The result of Model Number:Dataset Number:Featuregroup Number:Rescale of 62 : 1 : 8 : 1
f1-score precision recall support
AFIB 0.768807 0.800905 0.739322 875.0
GSVT 0.830580 0.826375 0.834929 691.0
SB 0.910756 0.905631 0.915964 1553.0
SR 0.852786 0.833849 0.872617 907.0
macro avg 0.840732 0.841690 0.840708 4026.0
micro avg 0.853899 0.853899 0.853899 4026.0
weighted avg 0.853084 0.853096 0.853899 4026.0
The result of Model Number:Dataset Number:Featuregroup Number:Rescale of 62 : 1 : 9 : 0
f1-score precision recall support
AFIB 0.641335 0.639363 0.643361 875.0
GSVT 0.624987 0.628052 0.622051 691.0
SB 0.657036 0.629770 0.686776 1553.0
SR 0.565434 0.616921 0.521924 907.0
macro avg 0.622198 0.628526 0.618528 4026.0
micro avg 0.629092 0.629092 0.629092 4026.0
weighted avg 0.627486 0.628665 0.629092 4026.0
The result of Model Number:Dataset Number:Featuregroup Number:Rescale of 62 : 1 : 9 : 1
f1-score precision recall support
AFIB 0.750123 0.757045 0.743366 875.0
GSVT 0.767638 0.765566 0.769749 691.0
SB 0.817546 0.809861 0.825386 1553.0
SR 0.794958 0.802934 0.787153 907.0
macro avg 0.782566 0.783852 0.781413 4026.0
micro avg 0.789397 0.789397 0.789397 4026.0
weighted avg 0.789238 0.789219 0.789397 4026.0
The result of Model Number:Dataset Number:Featuregroup Number:Rescale of 62 : 1 : 10 : 0
f1-score precision recall support
AFIB 0.710404 0.707515 0.713335 875.0
GSVT 0.720788 0.725146 0.716511 691.0
SB 0.760207 0.759281 0.761137 1553.0

SR 0.744348 0.745527 0.743194 907.0
macro avg 0.733937 0.734367 0.733544 4026.0
micro avg 0.739046 0.739046 0.739046 4026.0
weighted avg 0.739044 0.739073 0.739046 4026.0
The result of Model Number:Dataset Number:Featuregroup Number:Rescale of 62 : 1 : 10 : 1

	f1-score	precision	recall	support
AFIB	0.754025	0.755568	0.752570	204.0
GSVT	0.770609	0.767614	0.773682	300.0
SB	0.787716	0.786665	0.788775	892.0
SR	0.781931	0.784242	0.779635	681.0
macro avg	0.773570	0.773522	0.773665	2077.0
micro avg	0.780042	0.780042	0.780042	2077.0
weighted avg	0.780039	0.780065	0.780042	2077.0

The result of Model Number:Dataset Number:Featuregroup Number:Rescale of 63 : 0 : 0 : 0

	f1-score	precision	recall	support
AFIB	0.783256	0.777265	0.789444	875.0
GSVT	0.801278	0.805659	0.796991	691.0
SB	0.848488	0.847894	0.849090	1553.0
SR	0.825951	0.829958	0.822026	907.0
macro avg	0.814743	0.815194	0.814388	4026.0
micro avg	0.821087	0.821087	0.821087	4026.0
weighted avg	0.821131	0.821254	0.821087	4026.0

The result of Model Number:Dataset Number:Featuregroup Number:Rescale of 63 : 0 : 0 : 1

	f1-score	precision	recall	support
AFIB	0.871892	0.899342	0.846280	204.0
GSVT	0.828344	0.846037	0.811513	300.0
SB	0.855283	0.825355	0.887495	892.0
SR	0.781090	0.807488	0.756419	681.0
macro avg	0.834152	0.844556	0.825427	2077.0
micro avg	0.829496	0.829496	0.829496	2077.0
weighted avg	0.828697	0.829751	0.829496	2077.0

The result of Model Number:Dataset Number:Featuregroup Number:Rescale of 63 : 0 : 1 : 0

	f1-score	precision	recall	support
AFIB	0.838508	0.847446	0.829893	204.0
GSVT	0.853928	0.849244	0.858759	300.0
SB	0.873640	0.872183	0.875106	892.0
SR	0.866357	0.867768	0.864962	681.0
macro avg	0.858108	0.859160	0.857180	2077.0
micro avg	0.864978	0.864978	0.864978	2077.0
weighted avg	0.864954	0.864993	0.864978	2077.0

The result of Model Number:Dataset Number:Featuregroup Number:Rescale of 63 : 0 : 1 : 1

	f1-score	precision	recall	support
AFIB	0.593035	0.666815	0.534478	204.0
GSVT	0.703592	0.688568	0.719318	300.0
SB	0.751098	0.747269	0.754970	892.0
SR	0.734248	0.724950	0.743835	681.0
macro avg	0.695493	0.706901	0.688150	2077.0
micro avg	0.724513	0.724513	0.724513	2077.0
weighted avg	0.723187	0.723570	0.724513	2077.0

The result of Model Number:Dataset Number:Featuregroup Number:Rescale of 63 : 0 : 2 : 0

	f1-score	precision	recall	support
AFIB	0.908335	0.929927	0.887823	204.0
GSVT	0.931916	0.928320	0.935638	300.0
SB	0.955009	0.952878	0.957150	892.0
SR	0.942994	0.941097	0.944932	681.0
macro avg	0.934563	0.938055	0.931386	2077.0
micro avg	0.943228	0.943228	0.943228	2077.0
weighted avg	0.943150	0.943214	0.943228	2077.0

The result of Model Number:Dataset Number:Featuregroup Number:Rescale of 63 : 0 : 2 : 1

	f1-score	precision	recall	support
AFIB	0.910712	0.932361	0.890147	204.0
GSVT	0.934355	0.930749	0.938087	300.0
SB	0.957508	0.955372	0.959655	892.0
SR	0.945462	0.943560	0.947405	681.0
macro avg	0.937009	0.940510	0.933823	2077.0
micro avg	0.945696	0.945696	0.945696	2077.0
weighted avg	0.945618	0.945682	0.945696	2077.0

The result of Model Number:Dataset Number:Featuregroup Number:Rescale of 63 : 0 : 3 : 0

	f1-score	precision	recall	support
AFIB	0.754887	0.761603	0.748328	875.0
GSVT	0.770732	0.762274	0.779429	691.0
SB	0.828918	0.822464	0.835481	1553.0
SR	0.801371	0.812730	0.790329	907.0
macro avg	0.788977	0.789768	0.788392	4026.0
micro avg	0.796747	0.796747	0.796747	4026.0
weighted avg	0.796636	0.796713	0.796747	4026.0

The result of Model Number:Dataset Number:Featuregroup Number:Rescale of 63 : 0 : 3 : 1

	f1-score	precision	recall	support
AFIB	0.895966	0.921181	0.872280	204.0

GSVT 0.919130 0.912679 0.925847 300.0
SB 0.944406 0.940627 0.948217 892.0
SR 0.931853 0.932436 0.931299 681.0
macro avg 0.922839 0.926731 0.919411 2077.0
micro avg 0.931980 0.931980 0.931980 2077.0
weighted avg 0.931882 0.931994 0.931980 2077.0
The result of Model Number:Dataset Number:Featuregroup Number:Rescale of 63 : 0 : 4 : 0

	f1-score	precision	recall	support
AFIB	0.833649	0.841799	0.825851	875.0
GSVT	0.827282	0.821005	0.833927	691.0
SB	0.875358	0.866352	0.884631	1553.0
SR	0.847573	0.861204	0.834606	907.0
macro avg	0.845965	0.847590	0.844754	4026.0
micro avg	0.851883	0.851883	0.851883	4026.0
weighted avg	0.851782	0.852073	0.851883	4026.0

The result of Model Number:Dataset Number:Featuregroup Number:Rescale of 63 : 0 : 4 : 1

	f1-score	precision	recall	support
AFIB	0.737910	0.815093	0.674297	204.0
GSVT	0.868101	0.856639	0.879966	300.0
SB	0.920617	0.917039	0.924229	892.0
SR	0.898495	0.885690	0.911686	681.0
macro avg	0.856281	0.868615	0.847544	2077.0
micro avg	0.889175	0.889175	0.889175	2077.0
weighted avg	0.887833	0.888023	0.889175	2077.0

The result of Model Number:Dataset Number:Featuregroup Number:Rescale of 63 : 0 : 5 : 0

	f1-score	precision	recall	support
AFIB	0.669431	0.738240	0.612766	204.0
GSVT	0.796603	0.785837	0.807746	300.0
SB	0.848325	0.844933	0.851748	892.0
SR	0.825300	0.814144	0.836802	681.0
macro avg	0.784915	0.795789	0.777266	2077.0
micro avg	0.817020	0.817020	0.817020	2077.0
weighted avg	0.815734	0.815823	0.817020	2077.0

The result of Model Number:Dataset Number:Featuregroup Number:Rescale of 63 : 0 : 5 : 1

	f1-score	precision	recall	support
AFIB	0.645784	0.647580	0.644031	875.0
GSVT	0.660577	0.660393	0.660766	691.0
SB	0.703535	0.696922	0.710282	1553.0
SR	0.684893	0.694770	0.675301	907.0
macro avg	0.673697	0.674916	0.672595	4026.0

micro avg 0.679504 0.679504 0.679504 4026.0
weighted avg 0.679411 0.679444 0.679504 4026.0
The result of Model Number:Dataset Number:Featuregroup Number:Rescale of 63 : 0 : 6 : 0

	f1-score	precision	recall	support
AFIB	0.644081	0.702414	0.595767	204.0
GSVT	0.747574	0.739032	0.756546	300.0
SB	0.792758	0.790728	0.794801	892.0
SR	0.773348	0.762875	0.784134	681.0
macro avg	0.739440	0.748762	0.732812	2077.0
micro avg	0.766229	0.766229	0.766229	2077.0
weighted avg	0.765264	0.765455	0.766229	2077.0

The result of Model Number:Dataset Number:Featuregroup Number:Rescale of 63 : 0 : 6 : 1

	f1-score	precision	recall	support
AFIB	0.783901	0.785136	0.782773	204.0
GSVT	0.802860	0.801607	0.804184	300.0
SB	0.819422	0.818422	0.820430	892.0
SR	0.813684	0.815245	0.812131	681.0
macro avg	0.804967	0.805103	0.804880	2077.0
micro avg	0.811664	0.811664	0.811664	2077.0
weighted avg	0.811660	0.811683	0.811664	2077.0

The result of Model Number:Dataset Number:Featuregroup Number:Rescale of 63 : 0 : 7 : 0

	f1-score	precision	recall	support
AFIB	0.903211	0.916904	0.889943	204.0
GSVT	0.917734	0.909998	0.925651	300.0
SB	0.938091	0.935065	0.941139	892.0
SR	0.928021	0.931468	0.924624	681.0
macro avg	0.921764	0.923359	0.920339	2077.0
micro avg	0.928459	0.928459	0.928459	2077.0
weighted avg	0.928423	0.928481	0.928459	2077.0

The result of Model Number:Dataset Number:Featuregroup Number:Rescale of 63 : 0 : 7 : 1

	f1-score	precision	recall	support
AFIB	0.717773	0.727078	0.708895	204.0
GSVT	0.745547	0.745065	0.746052	300.0
SB	0.761256	0.759649	0.762876	892.0
SR	0.753600	0.753171	0.754044	681.0
macro avg	0.744544	0.746241	0.742967	2077.0
micro avg	0.752248	0.752248	0.752248	2077.0
weighted avg	0.752206	0.752219	0.752248	2077.0

The result of Model Number:Dataset Number:Featuregroup Number:Rescale of 63 : 0 : 8 : 0

	f1-score	precision	recall	support
AFIB	0.834850	0.815879	0.854784	875.0
GSVT	0.863462	0.877464	0.849970	691.0
SB	0.921219	0.921225	0.921220	1553.0
SR	0.897641	0.907868	0.887645	907.0
macro avg	0.879293	0.880609	0.878405	4026.0
micro avg	0.886988	0.886988	0.886988	4026.0
weighted avg	0.887223	0.887809	0.886988	4026.0

The result of Model Number:Dataset Number:Featuregroup Number:Rescale of 63 : 0 : 8 : 1

	f1-score	precision	recall	support
AFIB	0.831406	0.823612	0.839455	875.0
GSVT	0.788253	0.769306	0.846198	691.0
SB	0.911467	0.908120	0.914864	1553.0
SR	0.708922	0.713894	0.704029	907.0
macro avg	0.810012	0.803733	0.826137	4026.0
micro avg	0.839191	0.839191	0.839191	4026.0
weighted avg	0.827288	0.822171	0.839191	4026.0

The result of Model Number:Dataset Number:Featuregroup Number:Rescale of 63 : 0 : 9 : 0

	f1-score	precision	recall	support
AFIB	0.735687	0.739883	0.731661	204.0
GSVT	0.750825	0.745409	0.756327	300.0
SB	0.765988	0.763191	0.768820	892.0
SR	0.757842	0.762818	0.752950	681.0
macro avg	0.752585	0.752825	0.752439	2077.0
micro avg	0.758162	0.758162	0.758162	2077.0
weighted avg	0.758151	0.758211	0.758162	2077.0

The result of Model Number:Dataset Number:Featuregroup Number:Rescale of 63 : 0 : 9 : 1

	f1-score	precision	recall	support
AFIB	0.892329	0.894546	0.890184	875.0
GSVT	0.907696	0.910423	0.905100	691.0
SB	0.962902	0.956366	0.969534	1553.0
SR	0.944579	0.951591	0.937706	907.0
macro avg	0.926877	0.928231	0.925631	4026.0
micro avg	0.934059	0.934059	0.934059	4026.0
weighted avg	0.933961	0.933969	0.934059	4026.0

The result of Model Number:Dataset Number:Featuregroup Number:Rescale of 63 : 0 : 10 : 0

	f1-score	precision	recall	support
AFIB	0.854645	0.842773	0.866937	875.0
GSVT	0.885186	0.896995	0.873799	691.0
SB	0.940355	0.938428	0.942293	1553.0

SR 0.915880 0.922957 0.908912 907.0
macro avg 0.899016 0.900288 0.897985 4026.0
micro avg 0.906639 0.906639 0.906639 4026.0
weighted avg 0.906744 0.907042 0.906639 4026.0
The result of Model Number:Dataset Number:Featuregroup Number:Rescale of 63 : 0 : 10 : 1

	f1-score	precision	recall	support
AFIB	0.783168	0.785863	0.780591	875.0
GSVT	0.798037	0.782115	0.814764	691.0
SB	0.844786	0.840217	0.849409	1553.0
SR	0.812111	0.830994	0.794116	907.0
macro avg	0.809526	0.809797	0.809720	4026.0
micro avg	0.816049	0.816049	0.816049	4026.0
weighted avg	0.816009	0.816354	0.816049	4026.0

The result of Model Number:Dataset Number:Featuregroup Number:Rescale of 63 : 1 : 0 : 0

	f1-score	precision	recall	support
AFIB	0.845760	0.861282	0.831195	204.0
GSVT	0.877866	0.870808	0.885201	300.0
SB	0.894151	0.892555	0.895755	892.0
SR	0.885297	0.886085	0.884515	681.0
macro avg	0.875768	0.877682	0.874166	2077.0
micro avg	0.884204	0.884204	0.884204	2077.0
weighted avg	0.884143	0.884221	0.884204	2077.0

The result of Model Number:Dataset Number:Featuregroup Number:Rescale of 63 : 1 : 0 : 1

	f1-score	precision	recall	support
AFIB	0.878711	0.881542	0.875981	875.0
GSVT	0.891065	0.876918	0.905756	691.0
SB	0.938882	0.931042	0.946861	1553.0
SR	0.891687	0.914334	0.870168	907.0
macro avg	0.900086	0.900959	0.899691	4026.0
micro avg	0.907123	0.907123	0.907123	4026.0
weighted avg	0.906965	0.907230	0.907123	4026.0

The result of Model Number:Dataset Number:Featuregroup Number:Rescale of 63 : 1 : 1 : 0

	f1-score	precision	recall	support
AFIB	0.828902	0.845455	0.813111	204.0
GSVT	0.838211	0.827737	0.849079	300.0
SB	0.854213	0.846046	0.862542	892.0
SR	0.838701	0.849703	0.828002	681.0
macro avg	0.840007	0.842235	0.838184	2077.0
micro avg	0.844418	0.844418	0.844418	2077.0
weighted avg	0.844330	0.844542	0.844418	2077.0

The result of Model Number:Dataset Number:Featuregroup Number:Rescale of 63 : 1 : 1 : 1

	f1-score	precision	recall	support
AFIB	0.771367	0.767505	0.775523	875.0
GSVT	0.788796	0.789863	0.787891	691.0
SB	0.839960	0.837194	0.842767	1553.0
SR	0.815292	0.823754	0.807099	907.0
macro avg	0.803854	0.804579	0.803320	4026.0
micro avg	0.810698	0.810698	0.810698	4026.0
weighted avg	0.810713	0.810896	0.810698	4026.0

The result of Model Number:Dataset Number:Featuregroup Number:Rescale of 63 : 1 : 2 : 0

	f1-score	precision	recall	support
AFIB	0.697758	0.691036	0.704720	875.0
GSVT	0.716039	0.718087	0.714077	691.0
SB	0.761273	0.759522	0.763037	1553.0
SR	0.739858	0.748563	0.731368	907.0
macro avg	0.728732	0.729302	0.728300	4026.0
micro avg	0.734825	0.734825	0.734825	4026.0
weighted avg	0.734880	0.735057	0.734825	4026.0

The result of Model Number:Dataset Number:Featuregroup Number:Rescale of 63 : 1 : 2 : 1

	f1-score	precision	recall	support
AFIB	0.879375	0.900771	0.859188	204.0
GSVT	0.890429	0.878171	0.903153	300.0
SB	0.908408	0.899337	0.917671	892.0
SR	0.892548	0.904261	0.881160	681.0
macro avg	0.892690	0.895635	0.890293	2077.0
micro avg	0.897859	0.897859	0.897859	2077.0
weighted avg	0.897760	0.898035	0.897859	2077.0

The result of Model Number:Dataset Number:Featuregroup Number:Rescale of 63 : 1 : 3 : 0

	f1-score	precision	recall	support
AFIB	0.652366	0.680996	0.626210	204.0
GSVT	0.607310	0.611683	0.603249	300.0
SB	0.629828	0.598200	0.664990	892.0
SR	0.558756	0.595648	0.526286	681.0
macro avg	0.612065	0.621632	0.605184	2077.0
micro avg	0.606785	0.606785	0.606785	2077.0
weighted avg	0.605487	0.607443	0.606785	2077.0

The result of Model Number:Dataset Number:Featuregroup Number:Rescale of 63 : 1 : 3 : 1

	f1-score	precision	recall	support
AFIB	0.742611	0.766735	0.720100	204.0

GSVT 0.756689 0.749916 0.763736 300.0
SB 0.774682 0.769372 0.780067 892.0
SR 0.762048 0.765263 0.758905 681.0
macro avg 0.759008 0.762821 0.755702 2077.0
micro avg 0.764880 0.764880 0.764880 2077.0
weighted avg 0.764791 0.764956 0.764880 2077.0
The result of Model Number:Dataset Number:Featuregroup Number:Rescale of 63 : 1 : 4 : 0
f1-score precision recall support
AFIB 0.670994 0.687056 0.656648 204.0
GSVT 0.690753 0.681020 0.701057 300.0
SB 0.708701 0.701501 0.716083 892.0
SR 0.691762 0.701750 0.682109 681.0
macro avg 0.690552 0.692832 0.688974 2077.0
micro avg 0.696936 0.696936 0.696936 2077.0
weighted avg 0.696851 0.697206 0.696936 2077.0
The result of Model Number:Dataset Number:Featuregroup Number:Rescale of 63 : 1 : 4 : 1
f1-score precision recall support
AFIB 0.701085 0.705992 0.696272 204.0
GSVT 0.717397 0.713860 0.720980 300.0
SB 0.731655 0.730438 0.732886 892.0
SR 0.724374 0.726093 0.722671 681.0
macro avg 0.718628 0.719096 0.718202 2077.0
micro avg 0.724221 0.724221 0.724221 2077.0
weighted avg 0.724206 0.724218 0.724221 2077.0
The result of Model Number:Dataset Number:Featuregroup Number:Rescale of 63 : 1 : 5 : 0
f1-score precision recall support
AFIB 0.738803 0.730048 0.747832 875.0
GSVT 0.749318 0.756562 0.742285 691.0
SB 0.795276 0.792233 0.798348 1553.0
SR 0.776735 0.785696 0.767998 907.0
macro avg 0.765033 0.766135 0.764116 4026.0
micro avg 0.770909 0.770909 0.770909 4026.0
weighted avg 0.770937 0.771123 0.770909 4026.0
The result of Model Number:Dataset Number:Featuregroup Number:Rescale of 63 : 1 : 5 : 1
f1-score precision recall support
AFIB 0.662741 0.666379 0.659186 875.0
GSVT 0.663668 0.655278 0.672381 691.0
SB 0.697962 0.695161 0.700792 1553.0
SR 0.682979 0.691270 0.674927 907.0
macro avg 0.676838 0.677022 0.676821 4026.0

micro avg 0.681046 0.681046 0.681046 4026.0
weighted avg 0.681046 0.681184 0.681046 4026.0
The result of Model Number:Dataset Number:Featuregroup Number:Rescale of 63 : 1 : 6 : 0

	f1-score	precision	recall	support
AFIB	0.657087	0.689917	0.627569	204.0
GSVT	0.676897	0.664352	0.690159	300.0
SB	0.692387	0.685408	0.699525	892.0
SR	0.673185	0.679198	0.667338	681.0
macro avg	0.674889	0.679719	0.671148	2077.0
micro avg	0.680551	0.680551	0.680551	2077.0
weighted avg	0.680386	0.680773	0.680551	2077.0

The result of Model Number:Dataset Number:Featuregroup Number:Rescale of 63 : 1 : 6 : 1

	f1-score	precision	recall	support
AFIB	0.603031	0.587600	0.619351	875.0
GSVT	0.570051	0.602359	0.541164	691.0
SB	0.604453	0.556242	0.661829	1553.0
SR	0.482856	0.586492	0.410433	907.0
macro avg	0.565098	0.583173	0.558194	4026.0
micro avg	0.575251	0.575251	0.575251	4026.0
weighted avg	0.570845	0.577787	0.575251	4026.0

The result of Model Number:Dataset Number:Featuregroup Number:Rescale of 63 : 1 : 7 : 0

	f1-score	precision	recall	support
AFIB	0.794419	0.782545	0.806688	875.0
GSVT	0.809650	0.816574	0.802855	691.0
SB	0.865093	0.862114	0.868098	1553.0
SR	0.839772	0.852297	0.827638	907.0
macro avg	0.827233	0.828383	0.826320	4026.0
micro avg	0.834438	0.834438	0.834438	4026.0
weighted avg	0.834512	0.834793	0.834438	4026.0

The result of Model Number:Dataset Number:Featuregroup Number:Rescale of 63 : 1 : 7 : 1

	f1-score	precision	recall	support
AFIB	0.704999	0.712833	0.697698	204.0
GSVT	0.726837	0.722700	0.731234	300.0
SB	0.741297	0.740303	0.742294	892.0
SR	0.734991	0.735975	0.734018	681.0
macro avg	0.727031	0.727953	0.726311	2077.0
micro avg	0.733603	0.733603	0.733603	2077.0
weighted avg	0.733575	0.733644	0.733603	2077.0

The result of Model Number:Dataset Number:Featuregroup Number:Rescale of 63 : 1 : 8 : 0

	f1-score	precision	recall	support
AFIB	0.680418	0.701053	0.660992	875.0
GSVT	0.726356	0.721319	0.731503	691.0
SB	0.794722	0.791682	0.797791	1553.0
SR	0.751616	0.740335	0.763290	907.0
macro avg	0.738278	0.738597	0.738394	4026.0
micro avg	0.748910	0.748910	0.748910	4026.0
weighted avg	0.748434	0.748340	0.748910	4026.0

The result of Model Number:Dataset Number:Featuregroup Number:Rescale of 63 : 1 : 8 : 1

	f1-score	precision	recall	support
AFIB	0.811632	0.813890	0.809429	875.0
GSVT	0.830224	0.829994	0.830463	691.0
SB	0.884215	0.875904	0.892695	1553.0
SR	0.860786	0.873199	0.848731	907.0
macro avg	0.846714	0.848247	0.845329	4026.0
micro avg	0.854013	0.854013	0.854013	4026.0
weighted avg	0.853895	0.853937	0.854013	4026.0

The result of Model Number:Dataset Number:Featuregroup Number:Rescale of 63 : 1 : 9 : 0

	f1-score	precision	recall	support
AFIB	0.856725	0.848693	0.865019	875.0
GSVT	0.812257	0.792734	0.871968	691.0
SB	0.939224	0.935775	0.942725	1553.0
SR	0.730511	0.735634	0.725469	907.0
macro avg	0.834679	0.828209	0.851295	4026.0
micro avg	0.864747	0.864747	0.864747	4026.0
weighted avg	0.852482	0.847209	0.864747	4026.0

The result of Model Number:Dataset Number:Featuregroup Number:Rescale of 63 : 1 : 9 : 1

	f1-score	precision	recall	support
AFIB	0.773954	0.784910	0.763935	204.0
GSVT	0.790023	0.775477	0.805270	300.0
SB	0.806861	0.797940	0.815997	892.0
SR	0.789904	0.805992	0.774493	681.0
macro avg	0.790185	0.791080	0.789924	2077.0
micro avg	0.795726	0.795726	0.795726	2077.0
weighted avg	0.795637	0.796056	0.795726	2077.0

The result of Model Number:Dataset Number:Featuregroup Number:Rescale of 63 : 1 : 10 : 0

	f1-score	precision	recall	support
AFIB	0.797797	0.806288	0.789924	204.0
GSVT	0.821243	0.815771	0.827029	300.0
SB	0.838597	0.837848	0.839349	892.0

SR 0.831526 0.832636 0.830425 681.0
macro avg 0.822291 0.823136 0.821682 2077.0
micro avg 0.829789 0.829789 0.829789 2077.0
weighted avg 0.829765 0.829850 0.829789 2077.0
The result of Model Number:Dataset Number:Featuregroup Number:Rescale of 63 : 1 : 10 : 1

	f1-score	precision	recall	support
AFIB	0.726914	0.734663	0.719446	204.0
GSVT	0.740283	0.736222	0.744471	300.0
SB	0.757371	0.756108	0.758642	892.0
SR	0.751057	0.752281	0.749847	681.0
macro avg	0.743906	0.744818	0.743102	2077.0
micro avg	0.749862	0.749862	0.749862	2077.0
weighted avg	0.749841	0.749875	0.749862	2077.0

The result of Model Number:Dataset Number:Featuregroup Number:Rescale of 64 : 0 : 0 : 0

	f1-score	precision	recall	support
AFIB	0.767340	0.774336	0.760571	204.0
GSVT	0.787365	0.784787	0.789985	300.0
SB	0.801759	0.801224	0.802297	892.0
SR	0.794708	0.794479	0.794948	681.0
macro avg	0.787793	0.788707	0.786950	2077.0
micro avg	0.794011	0.794011	0.794011	2077.0
weighted avg	0.793988	0.793998	0.794011	2077.0

The result of Model Number:Dataset Number:Featuregroup Number:Rescale of 64 : 0 : 0 : 1

	f1-score	precision	recall	support
AFIB	0.828941	0.812428	0.846182	875.0
GSVT	0.674685	0.627421	0.845698	691.0
SB	0.547432	0.548375	0.546492	1553.0
SR	0.887457	0.895272	0.879815	907.0
macro avg	0.734629	0.720874	0.779547	4026.0
micro avg	0.738073	0.738073	0.738073	4026.0
weighted avg	0.707058	0.697481	0.738073	4026.0

The result of Model Number:Dataset Number:Featuregroup Number:Rescale of 64 : 0 : 1 : 0

	f1-score	precision	recall	support
AFIB	0.707247	0.706610	0.707954	875.0
GSVT	0.719500	0.712912	0.726267	691.0
SB	0.755029	0.748391	0.761787	1553.0
SR	0.719483	0.737270	0.702597	907.0
macro avg	0.725315	0.726296	0.724651	4026.0
micro avg	0.730656	0.730656	0.730656	4026.0
weighted avg	0.730538	0.730716	0.730656	4026.0

The result of Model Number:Dataset Number:Featuregroup Number:Rescale of 64 : 0 : 1 : 1

	f1-score	precision	recall	support
AFIB	0.724870	0.722640	0.727159	875.0
GSVT	0.706392	0.709856	0.703074	691.0
SB	0.742616	0.711799	0.776229	1553.0
SR	0.639083	0.697275	0.589905	907.0
macro avg	0.703240	0.710393	0.699092	4026.0
micro avg	0.711032	0.711032	0.711032	4026.0
weighted avg	0.709217	0.710550	0.711032	4026.0

The result of Model Number:Dataset Number:Featuregroup Number:Rescale of 64 : 0 : 2 : 0

	f1-score	precision	recall	support
AFIB	0.708577	0.717277	0.700195	204.0
GSVT	0.722849	0.719761	0.725980	300.0
SB	0.731599	0.729403	0.733811	892.0
SR	0.725441	0.727167	0.723746	681.0
macro avg	0.722116	0.723402	0.720933	2077.0
micro avg	0.726078	0.726078	0.726078	2077.0
weighted avg	0.726055	0.726086	0.726078	2077.0

The result of Model Number:Dataset Number:Featuregroup Number:Rescale of 64 : 0 : 2 : 1

	f1-score	precision	recall	support
AFIB	0.876484	0.897304	0.856688	204.0
GSVT	0.905274	0.896786	0.914019	300.0
SB	0.924715	0.923681	0.925752	892.0
SR	0.915790	0.914870	0.916727	681.0
macro avg	0.905566	0.908160	0.903297	2077.0
micro avg	0.914315	0.914315	0.914315	2077.0
weighted avg	0.914244	0.914317	0.914315	2077.0

The result of Model Number:Dataset Number:Featuregroup Number:Rescale of 64 : 0 : 3 : 0

	f1-score	precision	recall	support
AFIB	0.812230	0.818788	0.805847	204.0
GSVT	0.830868	0.826794	0.835011	300.0
SB	0.847084	0.846144	0.848033	892.0
SR	0.840758	0.841876	0.839649	681.0
macro avg	0.832735	0.833400	0.832135	2077.0
micro avg	0.839260	0.839260	0.839260	2077.0
weighted avg	0.839244	0.839263	0.839260	2077.0

The result of Model Number:Dataset Number:Featuregroup Number:Rescale of 64 : 0 : 3 : 1

	f1-score	precision	recall	support
AFIB	0.849804	0.841617	0.858283	875.0

GSVT 0.872069 0.874563 0.869679 691.0
SB 0.927159 0.925027 0.929308 1553.0
SR 0.901078 0.911680 0.890738 907.0
macro avg 0.887527 0.888222 0.887002 4026.0
micro avg 0.894948 0.894948 0.894948 4026.0
weighted avg 0.895016 0.895231 0.894948 4026.0
The result of Model Number:Dataset Number:Featuregroup Number:Rescale of 64 : 0 : 4 : 0
f1-score precision recall support
AFIB 0.674356 0.673739 0.675056 875.0
GSVT 0.688179 0.686844 0.689591 691.0
SB 0.730247 0.728621 0.731892 1553.0
SR 0.710316 0.714950 0.705767 907.0
macro avg 0.700774 0.701038 0.700576 4026.0
micro avg 0.706394 0.706394 0.706394 4026.0
weighted avg 0.706389 0.706443 0.706394 4026.0
The result of Model Number:Dataset Number:Featuregroup Number:Rescale of 64 : 0 : 4 : 1
f1-score precision recall support
AFIB 0.807291 0.805725 0.808966 875.0
GSVT 0.822450 0.808060 0.837441 691.0
SB 0.881076 0.868575 0.893952 1553.0
SR 0.834611 0.871817 0.800487 907.0
macro avg 0.836357 0.838544 0.835211 4026.0
micro avg 0.844726 0.844726 0.844726 4026.0
weighted avg 0.844510 0.845259 0.844726 4026.0
The result of Model Number:Dataset Number:Featuregroup Number:Rescale of 64 : 0 : 5 : 0
f1-score precision recall support
AFIB 0.854941 0.862831 0.847241 875.0
GSVT 0.874905 0.872543 0.877310 691.0
SB 0.931786 0.923027 0.940721 1553.0
SR 0.906041 0.915132 0.897147 907.0
macro avg 0.891918 0.893383 0.890605 4026.0
micro avg 0.899704 0.899704 0.899704 4026.0
weighted avg 0.899522 0.899501 0.899704 4026.0
The result of Model Number:Dataset Number:Featuregroup Number:Rescale of 64 : 0 : 5 : 1
f1-score precision recall support
AFIB 0.768283 0.811988 0.729552 204.0
GSVT 0.811902 0.802961 0.821085 300.0
SB 0.823979 0.815780 0.832372 892.0
SR 0.814407 0.817191 0.811770 681.0
macro avg 0.804643 0.811980 0.798695 2077.0

	f1-score	precision	recall	support
micro avg	0.813888	0.813888	0.813888	2077.0
weighted avg	0.813626	0.814019	0.813888	2077.0

The result of Model Number:Dataset Number:Featuregroup Number:Rescale of 64 : 0 : 6 : 0

	f1-score	precision	recall	support
AFIB	0.650129	0.651858	0.648448	875.0
GSVT	0.662631	0.657963	0.667405	691.0
SB	0.700479	0.693337	0.707784	1553.0
SR	0.668459	0.683019	0.654531	907.0
macro avg	0.670424	0.671544	0.669542	4026.0
micro avg	0.675960	0.675960	0.675960	4026.0
weighted avg	0.675826	0.675926	0.675960	4026.0

The result of Model Number:Dataset Number:Featuregroup Number:Rescale of 64 : 0 : 6 : 1

	f1-score	precision	recall	support
AFIB	0.714348	0.754985	0.678335	204.0
GSVT	0.754904	0.746591	0.763443	300.0
SB	0.766134	0.758511	0.773938	892.0
SR	0.757234	0.759822	0.754782	681.0
macro avg	0.748155	0.754977	0.742625	2077.0
micro avg	0.756751	0.756751	0.756751	2077.0
weighted avg	0.756507	0.756873	0.756751	2077.0

The result of Model Number:Dataset Number:Featuregroup Number:Rescale of 64 : 0 : 7 : 0

	f1-score	precision	recall	support
AFIB	0.675149	0.685068	0.665576	204.0
GSVT	0.692256	0.686370	0.698257	300.0
SB	0.704220	0.701402	0.707062	892.0
SR	0.697255	0.700677	0.693874	681.0
macro avg	0.692220	0.693380	0.691192	2077.0
micro avg	0.697391	0.697391	0.697391	2077.0
weighted avg	0.697353	0.697389	0.697391	2077.0

The result of Model Number:Dataset Number:Featuregroup Number:Rescale of 64 : 0 : 7 : 1

	f1-score	precision	recall	support
AFIB	0.709180	0.699110	0.719623	875.0
GSVT	0.734899	0.744274	0.725868	691.0
SB	0.780995	0.779849	0.782152	1553.0
SR	0.761538	0.767250	0.755912	907.0
macro avg	0.746653	0.747621	0.745889	4026.0
micro avg	0.752990	0.752990	0.752990	4026.0
weighted avg	0.753092	0.753357	0.752990	4026.0

The result of Model Number:Dataset Number:Featuregroup Number:Rescale of 64 : 0 : 8 : 0

	f1-score	precision	recall	support
AFIB	0.867524	0.855327	0.880116	875.0
GSVT	0.886202	0.892851	0.879674	691.0
SB	0.942374	0.939604	0.945163	1553.0
SR	0.919161	0.931877	0.906802	907.0
macro avg	0.903815	0.904915	0.902939	4026.0
micro avg	0.911144	0.911144	0.911144	4026.0
weighted avg	0.911235	0.911522	0.911144	4026.0

The result of Model Number:Dataset Number:Featuregroup Number:Rescale of 64 : 0 : 8 : 1

	f1-score	precision	recall	support
AFIB	0.821242	0.822621	0.841597	204.0
GSVT	0.813522	0.845091	0.785275	300.0
SB	0.862916	0.815593	0.916423	892.0
SR	0.747964	0.816991	0.691587	681.0
macro avg	0.811411	0.825074	0.808720	2077.0
micro avg	0.816412	0.816412	0.816412	2077.0
weighted avg	0.813999	0.821002	0.816412	2077.0

The result of Model Number:Dataset Number:Featuregroup Number:Rescale of 64 : 0 : 9 : 0

	f1-score	precision	recall	support
AFIB	0.719696	0.716769	0.722665	875.0
GSVT	0.730216	0.734630	0.725882	691.0
SB	0.770150	0.769212	0.771092	1553.0
SR	0.754083	0.755278	0.752914	907.0
macro avg	0.743536	0.743972	0.743138	4026.0
micro avg	0.748712	0.748712	0.748712	4026.0
weighted avg	0.748711	0.748740	0.748712	4026.0

The result of Model Number:Dataset Number:Featuregroup Number:Rescale of 64 : 0 : 9 : 1

	f1-score	precision	recall	support
AFIB	0.896737	0.941542	0.856454	204.0
GSVT	0.923772	0.906652	0.941872	300.0
SB	0.944912	0.935387	0.954653	892.0
SR	0.918706	0.926912	0.910727	681.0
macro avg	0.921032	0.927623	0.915926	2077.0
micro avg	0.928760	0.928760	0.928760	2077.0
weighted avg	0.928535	0.929063	0.928760	2077.0

The result of Model Number:Dataset Number:Featuregroup Number:Rescale of 64 : 0 : 10 : 0

	f1-score	precision	recall	support
AFIB	0.698515	0.702499	0.694692	204.0
GSVT	0.712888	0.707746	0.718112	300.0
SB	0.727285	0.724629	0.729974	892.0

SR 0.719550 0.724275 0.714905 681.0
macro avg 0.714559 0.714787 0.714421 2077.0
micro avg 0.719855 0.719855 0.719855 2077.0
weighted avg 0.719844 0.719901 0.719855 2077.0
The result of Model Number:Dataset Number:Featuregroup Number:Rescale of 64 : 0 : 10 : 1

	f1-score	precision	recall	support
AFIB	0.841504	0.842450	0.840712	204.0
GSVT	0.860824	0.858902	0.862820	300.0
SB	0.879070	0.877896	0.880251	892.0
SR	0.872486	0.874683	0.870311	681.0
macro avg	0.863471	0.863483	0.863524	2077.0
micro avg	0.870591	0.870591	0.870591	2077.0
weighted avg	0.870586	0.870618	0.870591	2077.0

The result of Model Number:Dataset Number:Featuregroup Number:Rescale of 64 : 1 : 0 : 0

	f1-score	precision	recall	support
AFIB	0.768186	0.802328	0.736938	204.0
GSVT	0.800198	0.793916	0.806598	300.0
SB	0.812421	0.806154	0.818794	892.0
SR	0.803433	0.804760	0.802139	681.0
macro avg	0.796060	0.801789	0.791117	2077.0
micro avg	0.803532	0.803532	0.803532	2077.0
weighted avg	0.803364	0.803554	0.803532	2077.0

The result of Model Number:Dataset Number:Featuregroup Number:Rescale of 64 : 1 : 0 : 1

	f1-score	precision	recall	support
AFIB	0.739292	0.824541	0.670117	204.0
GSVT	0.871701	0.856529	0.887606	300.0
SB	0.930707	0.926582	0.934879	892.0
SR	0.909109	0.896679	0.921917	681.0
macro avg	0.862702	0.876083	0.853630	2077.0
micro avg	0.897796	0.897796	0.897796	2077.0
weighted avg	0.896303	0.896637	0.897796	2077.0

The result of Model Number:Dataset Number:Featuregroup Number:Rescale of 64 : 1 : 1 : 0

	f1-score	precision	recall	support
AFIB	0.703350	0.790855	0.633901	204.0
GSVT	0.834474	0.816655	0.853125	300.0
SB	0.890816	0.886275	0.895409	892.0
SR	0.870832	0.859804	0.882202	681.0
macro avg	0.824868	0.838397	0.816159	2077.0
micro avg	0.859286	0.859286	0.859286	2077.0
weighted avg	0.857713	0.858168	0.859286	2077.0

The result of Model Number:Dataset Number:Featuregroup Number:Rescale of 64 : 1 : 1 : 1

	f1-score	precision	recall	support
AFIB	0.832804	0.846327	0.819750	875.0
GSVT	0.885711	0.888487	0.883006	691.0
SB	0.963609	0.960288	0.966958	1553.0
SR	0.912257	0.902096	0.922705	907.0
macro avg	0.898595	0.899300	0.898105	4026.0
micro avg	0.910585	0.910585	0.910585	4026.0
weighted avg	0.910241	0.910087	0.910585	4026.0

The result of Model Number:Dataset Number:Featuregroup Number:Rescale of 64 : 1 : 2 : 0

	f1-score	precision	recall	support
AFIB	0.887501	0.896575	0.878683	875.0
GSVT	0.887234	0.873527	0.901482	691.0
SB	0.935913	0.930325	0.941577	1553.0
SR	0.911141	0.923336	0.899291	907.0
macro avg	0.905447	0.905941	0.905258	4026.0
micro avg	0.911500	0.911500	0.911500	4026.0
weighted avg	0.911456	0.911667	0.911500	4026.0

The result of Model Number:Dataset Number:Featuregroup Number:Rescale of 64 : 1 : 2 : 1

	f1-score	precision	recall	support
AFIB	0.690641	0.692760	0.688643	204.0
GSVT	0.708783	0.706982	0.710657	300.0
SB	0.724617	0.723731	0.725508	892.0
SR	0.718686	0.720068	0.717315	681.0
macro avg	0.710682	0.710885	0.710531	2077.0
micro avg	0.717056	0.717056	0.717056	2077.0
weighted avg	0.717048	0.717069	0.717056	2077.0

The result of Model Number:Dataset Number:Featuregroup Number:Rescale of 64 : 1 : 3 : 0

	f1-score	precision	recall	support
AFIB	0.826154	0.820571	0.831926	875.0
GSVT	0.846310	0.846647	0.846082	691.0
SB	0.899032	0.896737	0.901347	1553.0
SR	0.874552	0.884323	0.865006	907.0
macro avg	0.861512	0.862069	0.861090	4026.0
micro avg	0.868587	0.868587	0.868587	4026.0
weighted avg	0.868629	0.868789	0.868587	4026.0

The result of Model Number:Dataset Number:Featuregroup Number:Rescale of 64 : 1 : 3 : 1

	f1-score	precision	recall	support
AFIB	0.779461	0.789565	0.769819	204.0

GSVT 0.809621 0.809098 0.810169 300.0
SB 0.826680 0.824935 0.828439 892.0
SR 0.818366 0.817901 0.818849 681.0
macro avg 0.808532 0.810375 0.806819 2077.0
micro avg 0.816898 0.816898 0.816898 2077.0
weighted avg 0.816852 0.816867 0.816898 2077.0
The result of Model Number:Dataset Number:Featuregroup Number:Rescale of 64 : 1 : 4 : 0
f1-score precision recall support
AFIB 0.735312 0.753623 0.717906 875.0
GSVT 0.776910 0.780209 0.773672 691.0
SB 0.842700 0.839476 0.845955 1553.0
SR 0.801593 0.786656 0.817151 907.0
macro avg 0.789129 0.789991 0.788671 4026.0
micro avg 0.799230 0.799230 0.799230 4026.0
weighted avg 0.798808 0.798745 0.799230 4026.0
The result of Model Number:Dataset Number:Featuregroup Number:Rescale of 64 : 1 : 4 : 1
f1-score precision recall support
AFIB 0.822257 0.848613 0.797734 204.0
GSVT 0.846856 0.832287 0.862070 300.0
SB 0.861506 0.852989 0.870196 892.0
SR 0.838471 0.848963 0.828253 681.0
macro avg 0.842272 0.845713 0.839563 2077.0
micro avg 0.848153 0.848153 0.848153 2077.0
weighted avg 0.847982 0.848249 0.848153 2077.0
The result of Model Number:Dataset Number:Featuregroup Number:Rescale of 64 : 1 : 5 : 0
f1-score precision recall support
AFIB 0.794458 0.805070 0.784346 204.0
GSVT 0.808493 0.803568 0.813602 300.0
SB 0.827982 0.827248 0.828723 892.0
SR 0.821864 0.821989 0.821744 681.0
macro avg 0.813199 0.814468 0.812104 2077.0
micro avg 0.819892 0.819892 0.819892 2077.0
weighted avg 0.819868 0.819925 0.819892 2077.0
The result of Model Number:Dataset Number:Featuregroup Number:Rescale of 64 : 1 : 5 : 1
f1-score precision recall support
AFIB 0.726637 0.743069 0.710938 875.0
GSVT 0.770401 0.773679 0.767195 691.0
SB 0.836495 0.832553 0.840480 1553.0
SR 0.792568 0.780411 0.805143 907.0
macro avg 0.781525 0.782428 0.780939 4026.0

micro avg 0.791787 0.791787 0.791787 4026.0
weighted avg 0.791379 0.791253 0.791787 4026.0
The result of Model Number:Dataset Number:Featuregroup Number:Rescale of 64 : 1 : 6 : 0

	f1-score	precision	recall	support
AFIB	0.705530	0.728449	0.684143	204.0
GSVT	0.718905	0.712470	0.725600	300.0
SB	0.736000	0.730955	0.741115	892.0
SR	0.723996	0.727051	0.721010	681.0
macro avg	0.721108	0.724731	0.717967	2077.0
micro avg	0.726687	0.726687	0.726687	2077.0
weighted avg	0.726602	0.726759	0.726687	2077.0

The result of Model Number:Dataset Number:Featuregroup Number:Rescale of 64 : 1 : 6 : 1

	f1-score	precision	recall	support
AFIB	0.818500	0.801009	0.836819	875.0
GSVT	0.847609	0.860570	0.835108	691.0
SB	0.903549	0.903668	0.903433	1553.0
SR	0.880271	0.889387	0.871343	907.0
macro avg	0.862482	0.863659	0.861676	4026.0
micro avg	0.869999	0.869999	0.869999	4026.0
weighted avg	0.870219	0.870742	0.869999	4026.0

The result of Model Number:Dataset Number:Featuregroup Number:Rescale of 64 : 1 : 7 : 0

	f1-score	precision	recall	support
AFIB	0.630141	0.633038	0.627302	875.0
GSVT	0.603949	0.607588	0.600374	691.0
SB	0.647834	0.618957	0.679569	1553.0
SR	0.554040	0.603145	0.512440	907.0
macro avg	0.608991	0.615682	0.604921	4026.0
micro avg	0.616965	0.616965	0.616965	4026.0
weighted avg	0.615326	0.616504	0.616965	4026.0

The result of Model Number:Dataset Number:Featuregroup Number:Rescale of 64 : 1 : 7 : 1

	f1-score	precision	recall	support
AFIB	0.761791	0.757955	0.765699	875.0
GSVT	0.776629	0.780389	0.772918	691.0
SB	0.827157	0.825254	0.829073	1553.0
SR	0.806450	0.810765	0.802190	907.0
macro avg	0.793007	0.793591	0.792470	4026.0
micro avg	0.799605	0.799605	0.799605	4026.0
weighted avg	0.799613	0.799663	0.799605	4026.0

The result of Model Number:Dataset Number:Featuregroup Number:Rescale of 64 : 1 : 8 : 0

	f1-score	precision	recall	support
AFIB	0.907353	0.912784	0.902033	204.0
GSVT	0.931524	0.928755	0.934316	300.0
SB	0.945789	0.944630	0.946954	892.0
SR	0.938894	0.940002	0.937796	681.0
macro avg	0.930890	0.931543	0.930275	2077.0
micro avg	0.937714	0.937714	0.937714	2077.0
weighted avg	0.937693	0.937692	0.937714	2077.0

The result of Model Number:Dataset Number:Featuregroup Number:Rescale of 64 : 1 : 8 : 1

	f1-score	precision	recall	support
AFIB	0.875564	0.891318	0.860616	204.0
GSVT	0.895317	0.882728	0.908418	300.0
SB	0.909093	0.898352	0.920098	892.0
SR	0.890809	0.906669	0.875505	681.0
macro avg	0.892696	0.894767	0.891159	2077.0
micro avg	0.897948	0.897948	0.897948	2077.0
weighted avg	0.897815	0.898131	0.897948	2077.0

The result of Model Number:Dataset Number:Featuregroup Number:Rescale of 64 : 1 : 9 : 0

	f1-score	precision	recall	support
AFIB	0.738215	0.734082	0.742430	875.0
GSVT	0.744226	0.748617	0.739932	691.0
SB	0.783981	0.781527	0.786453	1553.0
SR	0.768460	0.773544	0.763461	907.0
macro avg	0.758721	0.759443	0.758069	4026.0
micro avg	0.763721	0.763721	0.763721	4026.0
weighted avg	0.763714	0.763769	0.763721	4026.0

The result of Model Number:Dataset Number:Featuregroup Number:Rescale of 64 : 1 : 9 : 1

	f1-score	precision	recall	support
AFIB	0.673819	0.626601	0.791273	875.0
GSVT	0.577887	0.539538	0.653648	691.0
SB	0.690538	0.684712	0.696480	1553.0
SR	0.665578	0.681711	0.650423	907.0
macro avg	0.651956	0.633141	0.697956	4026.0
micro avg	0.699355	0.699355	0.699355	4026.0
weighted avg	0.661947	0.646489	0.699355	4026.0

The result of Model Number:Dataset Number:Featuregroup Number:Rescale of 64 : 1 : 10 : 0

	f1-score	precision	recall	support
AFIB	0.859847	0.873031	0.847196	204.0
GSVT	0.880235	0.872767	0.887869	300.0
SB	0.895547	0.892657	0.898458	892.0

SR 0.886344 0.889634 0.883089 681.0
macro avg 0.880493 0.882022 0.879153 2077.0
micro avg 0.886855 0.886855 0.886855 2077.0
weighted avg 0.886811 0.886865 0.886855 2077.0
The result of Model Number:Dataset Number:Featuregroup Number:Rescale of 64 : 1 : 10 : 1

	f1-score	precision	recall	support
AFIB	0.643862	0.635416	0.652564	875.0
GSVT	0.653719	0.660985	0.646622	691.0
SB	0.693353	0.691717	0.695002	1553.0
SR	0.675994	0.682078	0.670027	907.0
macro avg	0.666732	0.667549	0.666054	4026.0
micro avg	0.671848	0.671848	0.671848	4026.0
weighted avg	0.671883	0.672034	0.671848	4026.0

The result of Model Number:Dataset Number:Featuregroup Number:Rescale of 65 : 0 : 0 : 0

	f1-score	precision	recall	support
AFIB	0.707858	0.696526	0.796484	204.0
GSVT	0.828715	0.819099	0.838862	300.0
SB	0.856463	0.853229	0.859728	892.0
SR	0.671997	0.675471	0.668672	681.0
macro avg	0.766258	0.761081	0.790936	2077.0
micro avg	0.787859	0.787859	0.787859	2077.0
weighted avg	0.777377	0.774626	0.787859	2077.0

The result of Model Number:Dataset Number:Featuregroup Number:Rescale of 65 : 0 : 0 : 1

	f1-score	precision	recall	support
AFIB	0.727852	0.731670	0.724117	875.0
GSVT	0.743071	0.741736	0.744463	691.0
SB	0.806848	0.800660	0.813133	1553.0
SR	0.778381	0.786172	0.770748	907.0
macro avg	0.764038	0.765059	0.763115	4026.0
micro avg	0.772452	0.772452	0.772452	4026.0
weighted avg	0.772319	0.772289	0.772452	4026.0

The result of Model Number:Dataset Number:Featuregroup Number:Rescale of 65 : 0 : 1 : 0

	f1-score	precision	recall	support
AFIB	0.655688	0.691355	0.623687	204.0
GSVT	0.688351	0.677940	0.699114	300.0
SB	0.704708	0.700124	0.709364	892.0
SR	0.696014	0.696642	0.695404	681.0
macro avg	0.686190	0.691515	0.681892	2077.0
micro avg	0.694891	0.694891	0.694891	2077.0
weighted avg	0.694680	0.694917	0.694891	2077.0

The result of Model Number:Dataset Number:Featuregroup Number:Rescale of 65 : 0 : 1 : 1

	f1-score	precision	recall	support
AFIB	0.883052	0.907395	0.860158	204.0
GSVT	0.906553	0.889771	0.924066	300.0
SB	0.921255	0.912642	0.930034	892.0
SR	0.898099	0.910300	0.886236	681.0
macro avg	0.902240	0.905027	0.900123	2077.0
micro avg	0.907948	0.907948	0.907948	2077.0
weighted avg	0.907787	0.908055	0.907948	2077.0

The result of Model Number:Dataset Number:Featuregroup Number:Rescale of 65 : 0 : 2 : 0

	f1-score	precision	recall	support
AFIB	0.824431	0.824578	0.824320	875.0
GSVT	0.833154	0.835968	0.830411	691.0
SB	0.875185	0.872168	0.878228	1553.0
SR	0.858566	0.861462	0.855721	907.0
macro avg	0.847834	0.848544	0.847170	4026.0
micro avg	0.853234	0.853234	0.853234	4026.0
weighted avg	0.853196	0.853200	0.853234	4026.0

The result of Model Number:Dataset Number:Featuregroup Number:Rescale of 65 : 0 : 2 : 1

	f1-score	precision	recall	support
AFIB	0.800516	0.862909	0.746910	204.0
GSVT	0.824928	0.838893	0.811456	300.0
SB	0.874269	0.866222	0.882493	892.0
SR	0.839472	0.827299	0.852048	681.0
macro avg	0.834796	0.848831	0.823227	2077.0
micro avg	0.848933	0.848933	0.848933	2077.0
weighted avg	0.848489	0.849187	0.848933	2077.0

The result of Model Number:Dataset Number:Featuregroup Number:Rescale of 65 : 0 : 3 : 0

	f1-score	precision	recall	support
AFIB	0.820073	0.822895	0.817374	875.0
GSVT	0.835642	0.818970	0.853158	691.0
SB	0.884594	0.879810	0.889435	1553.0
SR	0.850380	0.870152	0.831537	907.0
macro avg	0.847672	0.847957	0.847876	4026.0
micro avg	0.854503	0.854503	0.854503	4026.0
weighted avg	0.854461	0.854822	0.854503	4026.0

The result of Model Number:Dataset Number:Featuregroup Number:Rescale of 65 : 0 : 3 : 1

	f1-score	precision	recall	support
AFIB	0.798619	0.799960	0.818413	204.0

GSVT 0.791111 0.821811 0.763642 300.0
SB 0.839145 0.793126 0.891178 892.0
SR 0.727360 0.794485 0.672535 681.0
macro avg 0.789059 0.802345 0.786442 2077.0
micro avg 0.793922 0.793922 0.793922 2077.0
weighted avg 0.791575 0.798386 0.793922 2077.0
The result of Model Number:Dataset Number:Featuregroup Number:Rescale of 65 : 0 : 4 : 0

	f1-score	precision	recall	support
AFIB	0.707402	0.722301	0.693217	204.0
GSVT	0.729396	0.722732	0.736200	300.0
SB	0.741308	0.739656	0.742971	892.0
SR	0.732499	0.733264	0.731743	681.0
macro avg	0.727651	0.729488	0.726033	2077.0
micro avg	0.733425	0.733425	0.733425	2077.0
weighted avg	0.733369	0.733411	0.733425	2077.0

The result of Model Number:Dataset Number:Featuregroup Number:Rescale of 65 : 0 : 4 : 1

	f1-score	precision	recall	support
AFIB	0.848916	0.864671	0.833936	204.0
GSVT	0.867583	0.863034	0.872198	300.0
SB	0.881434	0.878301	0.884595	892.0
SR	0.872360	0.873932	0.870831	681.0
macro avg	0.867573	0.869984	0.865390	2077.0
micro avg	0.873316	0.873316	0.873316	2077.0
weighted avg	0.873264	0.873325	0.873316	2077.0

The result of Model Number:Dataset Number:Featuregroup Number:Rescale of 65 : 0 : 5 : 0

	f1-score	precision	recall	support
AFIB	0.764077	0.748647	0.780219	875.0
GSVT	0.789410	0.801962	0.777415	691.0
SB	0.839944	0.839897	0.839998	1553.0
SR	0.819579	0.827047	0.812258	907.0
macro avg	0.803253	0.804388	0.802473	4026.0
micro avg	0.810015	0.810015	0.810015	4026.0
weighted avg	0.810194	0.810659	0.810015	4026.0

The result of Model Number:Dataset Number:Featuregroup Number:Rescale of 65 : 0 : 5 : 1

	f1-score	precision	recall	support
AFIB	0.766480	0.785596	0.748331	875.0
GSVT	0.815401	0.818527	0.812337	691.0
SB	0.883011	0.878736	0.887331	1553.0
SR	0.836702	0.822756	0.851183	907.0
macro avg	0.825399	0.826404	0.824795	4026.0

micro avg 0.836106 0.836106 0.836106 4026.0
weighted avg 0.835648 0.835548 0.836106 4026.0
The result of Model Number:Dataset Number:Featuregroup Number:Rescale of 65 : 0 : 6 : 0

	f1-score	precision	recall	support
AFIB	0.782293	0.800889	0.764628	204.0
GSVT	0.802602	0.799505	0.805808	300.0
SB	0.822491	0.820655	0.824335	892.0
SR	0.812143	0.810509	0.813813	681.0
macro avg	0.804882	0.807890	0.802146	2077.0
micro avg	0.812344	0.812344	0.812344	2077.0
weighted avg	0.812277	0.812332	0.812344	2077.0

The result of Model Number:Dataset Number:Featuregroup Number:Rescale of 65 : 0 : 6 : 1

	f1-score	precision	recall	support
AFIB	0.731798	0.726200	0.737579	875.0
GSVT	0.748636	0.752729	0.744630	691.0
SB	0.792745	0.792189	0.793306	1553.0
SR	0.771688	0.775431	0.768021	907.0
macro avg	0.761216	0.761637	0.760884	4026.0
micro avg	0.767144	0.767144	0.767144	4026.0
weighted avg	0.767184	0.767299	0.767144	4026.0

The result of Model Number:Dataset Number:Featuregroup Number:Rescale of 65 : 0 : 7 : 0

	f1-score	precision	recall	support
AFIB	0.615717	0.606446	0.630402	875.0
GSVT	0.587898	0.586187	0.592914	691.0
SB	0.627800	0.585009	0.682565	1553.0
SR	0.465050	0.621635	0.385350	907.0
macro avg	0.574116	0.599819	0.572808	4026.0
micro avg	0.588883	0.588883	0.588883	4026.0
weighted avg	0.581660	0.598122	0.588883	4026.0

The result of Model Number:Dataset Number:Featuregroup Number:Rescale of 65 : 0 : 7 : 1

	f1-score	precision	recall	support
AFIB	0.756138	0.780375	0.733588	204.0
GSVT	0.778760	0.765362	0.792750	300.0
SB	0.792231	0.784400	0.800223	892.0
SR	0.771049	0.780697	0.761652	681.0
macro avg	0.774545	0.777709	0.772053	2077.0
micro avg	0.779952	0.779952	0.779952	2077.0
weighted avg	0.779795	0.780041	0.779952	2077.0

The result of Model Number:Dataset Number:Featuregroup Number:Rescale of 65 : 0 : 8 : 0

	f1-score	precision	recall	support
AFIB	0.854009	0.869682	0.839302	204.0
GSVT	0.886428	0.879301	0.893834	300.0
SB	0.902872	0.901260	0.904491	892.0
SR	0.893932	0.894728	0.893143	681.0
macro avg	0.884310	0.886243	0.882693	2077.0
micro avg	0.892828	0.892828	0.892828	2077.0
weighted avg	0.892766	0.892845	0.892828	2077.0

The result of Model Number:Dataset Number:Featuregroup Number:Rescale of 65 : 0 : 8 : 1

	f1-score	precision	recall	support
AFIB	0.836930	0.815513	0.859580	875.0
GSVT	0.791158	0.835998	0.751066	691.0
SB	0.838904	0.771993	0.918535	1553.0
SR	0.670143	0.813976	0.569629	907.0
macro avg	0.784284	0.809370	0.774703	4026.0
micro avg	0.798375	0.798375	0.798375	4026.0
weighted avg	0.792261	0.801895	0.798375	4026.0

The result of Model Number:Dataset Number:Featuregroup Number:Rescale of 65 : 0 : 9 : 0

	f1-score	precision	recall	support
AFIB	0.730924	0.733067	0.728850	875.0
GSVT	0.743018	0.733475	0.752909	691.0
SB	0.786361	0.784450	0.788285	1553.0
SR	0.766449	0.775545	0.757572	907.0
macro avg	0.756688	0.756634	0.756904	4026.0
micro avg	0.762377	0.762377	0.762377	4026.0
weighted avg	0.762388	0.762527	0.762377	4026.0

The result of Model Number:Dataset Number:Featuregroup Number:Rescale of 65 : 0 : 9 : 1

	f1-score	precision	recall	support
AFIB	0.868708	0.887004	0.851288	204.0
GSVT	0.895717	0.887534	0.904073	300.0
SB	0.910346	0.908316	0.912387	892.0
SR	0.899527	0.900467	0.898599	681.0
macro avg	0.893574	0.895830	0.891587	2077.0
micro avg	0.900664	0.900664	0.900664	2077.0
weighted avg	0.900596	0.900648	0.900664	2077.0

The result of Model Number:Dataset Number:Featuregroup Number:Rescale of 65 : 0 : 10 : 0

	f1-score	precision	recall	support
AFIB	0.734249	0.739999	0.728626	875.0
GSVT	0.736186	0.726790	0.745873	691.0
SB	0.772176	0.769079	0.775306	1553.0

SR 0.755862 0.763281 0.748613 907.0
macro avg 0.749618 0.749787 0.749604 4026.0
micro avg 0.754095 0.754095 0.754095 4026.0
weighted avg 0.754080 0.754194 0.754095 4026.0
The result of Model Number:Dataset Number:Featuregroup Number:Rescale of 65 : 0 : 10 : 1

	f1-score	precision	recall	support
AFIB	0.825930	0.830023	0.821965	875.0
GSVT	0.834154	0.835355	0.833082	691.0
SB	0.878059	0.873985	0.882181	1553.0
SR	0.861895	0.864054	0.859795	907.0
macro avg	0.850009	0.850854	0.849256	4026.0
micro avg	0.855623	0.855623	0.855623	4026.0
weighted avg	0.855552	0.855563	0.855623	4026.0

The result of Model Number:Dataset Number:Featuregroup Number:Rescale of 65 : 1 : 0 : 0

	f1-score	precision	recall	support
AFIB	0.872336	0.878611	0.866152	875.0
GSVT	0.870946	0.865731	0.876238	691.0
SB	0.918720	0.908541	0.929132	1553.0
SR	0.881583	0.897312	0.866426	907.0
macro avg	0.885896	0.887549	0.884487	4026.0
micro avg	0.892239	0.892239	0.892239	4026.0
weighted avg	0.892073	0.892159	0.892239	4026.0

The result of Model Number:Dataset Number:Featuregroup Number:Rescale of 65 : 1 : 0 : 1

	f1-score	precision	recall	support
AFIB	0.859327	0.849144	0.869830	875.0
GSVT	0.871558	0.879984	0.863378	691.0
SB	0.925014	0.921475	0.928587	1553.0
SR	0.903448	0.913871	0.893286	907.0
macro avg	0.889837	0.891119	0.888770	4026.0
micro avg	0.896672	0.896672	0.896672	4026.0
weighted avg	0.896704	0.896920	0.896672	4026.0

The result of Model Number:Dataset Number:Featuregroup Number:Rescale of 65 : 1 : 1 : 0

	f1-score	precision	recall	support
AFIB	0.674948	0.665573	0.684656	875.0
GSVT	0.699068	0.708394	0.690075	691.0
SB	0.742637	0.741115	0.744167	1553.0
SR	0.723308	0.728897	0.717805	907.0
macro avg	0.709990	0.710995	0.709176	4026.0
micro avg	0.716010	0.716010	0.716010	4026.0
weighted avg	0.716093	0.716328	0.716010	4026.0

The result of Model Number:Dataset Number:Featuregroup Number:Rescale of 65 : 1 : 1 : 1

	f1-score	precision	recall	support
AFIB	0.769052	0.797565	0.742617	204.0
GSVT	0.792092	0.782188	0.802351	300.0
SB	0.812178	0.804767	0.819733	892.0
SR	0.790825	0.796962	0.784789	681.0
macro avg	0.791037	0.795370	0.787372	2077.0
micro avg	0.798191	0.798191	0.798191	2077.0
weighted avg	0.798040	0.798239	0.798191	2077.0

The result of Model Number:Dataset Number:Featuregroup Number:Rescale of 65 : 1 : 2 : 0

	f1-score	precision	recall	support
AFIB	0.858309	0.858538	0.858230	875.0
GSVT	0.873255	0.870031	0.876613	691.0
SB	0.934213	0.931945	0.936496	1553.0
SR	0.911933	0.918477	0.905506	907.0
macro avg	0.894427	0.894748	0.894211	4026.0
micro avg	0.902226	0.902226	0.902226	4026.0
weighted avg	0.902234	0.902330	0.902226	4026.0

The result of Model Number:Dataset Number:Featuregroup Number:Rescale of 65 : 1 : 2 : 1

	f1-score	precision	recall	support
AFIB	0.729797	0.745903	0.714426	204.0
GSVT	0.745747	0.733380	0.758627	300.0
SB	0.760076	0.754131	0.766124	892.0
SR	0.741630	0.750557	0.732916	681.0
macro avg	0.744313	0.745993	0.743023	2077.0
micro avg	0.749075	0.749075	0.749075	2077.0
weighted avg	0.748985	0.749154	0.749075	2077.0

The result of Model Number:Dataset Number:Featuregroup Number:Rescale of 65 : 1 : 3 : 0

	f1-score	precision	recall	support
AFIB	0.827052	0.845476	0.809652	204.0
GSVT	0.837256	0.824993	0.850237	300.0
SB	0.856176	0.847667	0.864894	892.0
SR	0.840382	0.852453	0.828675	681.0
macro avg	0.840216	0.842647	0.838364	2077.0
micro avg	0.845476	0.845476	0.845476	2077.0
weighted avg	0.845404	0.845746	0.845476	2077.0

The result of Model Number:Dataset Number:Featuregroup Number:Rescale of 65 : 1 : 3 : 1

	f1-score	precision	recall	support
AFIB	0.676489	0.678206	0.674805	875.0

GSVT	0.688427	0.680969	0.696078	691.0
SB	0.725564	0.717356	0.733973	1553.0
SR	0.692390	0.711346	0.674446	907.0
macro avg	0.695717	0.696969	0.694825	4026.0
micro avg	0.701199	0.701199	0.701199	4026.0
weighted avg	0.701051	0.701248	0.701199	4026.0

The result of Model Number:Dataset Number:Featuregroup Number:Rescale of 65 : 1 : 4 : 0

	f1-score	precision	recall	support
AFIB	0.662254	0.666983	0.657711	204.0
GSVT	0.685412	0.684970	0.685867	300.0
SB	0.699125	0.697574	0.700690	892.0
SR	0.692876	0.693714	0.692061	681.0
macro avg	0.684917	0.685810	0.684082	2077.0
micro avg	0.691499	0.691499	0.691499	2077.0
weighted avg	0.691474	0.691483	0.691499	2077.0

The result of Model Number:Dataset Number:Featuregroup Number:Rescale of 65 : 1 : 4 : 1

	f1-score	precision	recall	support
AFIB	0.690988	0.699473	0.682815	204.0
GSVT	0.704906	0.701895	0.707959	300.0
SB	0.713439	0.711297	0.715597	892.0
SR	0.707434	0.709118	0.705781	681.0
macro avg	0.704192	0.705446	0.703038	2077.0
micro avg	0.708055	0.708055	0.708055	2077.0
weighted avg	0.708033	0.708063	0.708055	2077.0

The result of Model Number:Dataset Number:Featuregroup Number:Rescale of 65 : 1 : 5 : 0

	f1-score	precision	recall	support
AFIB	0.852616	0.857279	0.848033	875.0
GSVT	0.848553	0.841398	0.855916	691.0
SB	0.892466	0.884014	0.901084	1553.0
SR	0.859251	0.875279	0.843811	907.0
macro avg	0.863222	0.864493	0.862211	4026.0
micro avg	0.868899	0.868899	0.868899	4026.0
weighted avg	0.868785	0.868921	0.868899	4026.0

The result of Model Number:Dataset Number:Featuregroup Number:Rescale of 65 : 1 : 5 : 1

	f1-score	precision	recall	support
AFIB	0.891307	0.884490	0.898349	875.0
GSVT	0.911815	0.916801	0.906937	691.0
SB	0.965539	0.964862	0.966223	1553.0
SR	0.939892	0.944451	0.935426	907.0
macro avg	0.927138	0.927651	0.926734	4026.0

micro avg 0.934358 0.934358 0.934358 4026.0
weighted avg 0.934407 0.934547 0.934358 4026.0
The result of Model Number:Dataset Number:Featuregroup Number:Rescale of 65 : 1 : 6 : 0

	f1-score	precision	recall	support
AFIB	0.733927	0.739729	0.728240	875.0
GSVT	0.752395	0.750804	0.754021	691.0
SB	0.802075	0.794986	0.809302	1553.0
SR	0.780723	0.788380	0.773232	907.0
macro avg	0.767280	0.768475	0.766199	4026.0
micro avg	0.774070	0.774070	0.774070	4026.0
weighted avg	0.773927	0.773905	0.774070	4026.0

The result of Model Number:Dataset Number:Featuregroup Number:Rescale of 65 : 1 : 6 : 1

	f1-score	precision	recall	support
AFIB	0.738889	0.771728	0.708832	204.0
GSVT	0.769679	0.763638	0.775835	300.0
SB	0.781437	0.775408	0.787566	892.0
SR	0.772791	0.774067	0.771547	681.0
macro avg	0.765699	0.771210	0.760945	2077.0
micro avg	0.772886	0.772886	0.772886	2077.0
weighted avg	0.772725	0.772907	0.772886	2077.0

The result of Model Number:Dataset Number:Featuregroup Number:Rescale of 65 : 1 : 7 : 0

	f1-score	precision	recall	support
AFIB	0.705040	0.703544	0.706574	875.0
GSVT	0.716054	0.712622	0.719580	691.0
SB	0.753644	0.744647	0.762863	1553.0
SR	0.718621	0.738980	0.699369	907.0
macro avg	0.723340	0.724948	0.722097	4026.0
micro avg	0.728896	0.728896	0.728896	4026.0
weighted avg	0.728739	0.728941	0.728896	4026.0

The result of Model Number:Dataset Number:Featuregroup Number:Rescale of 65 : 1 : 7 : 1

	f1-score	precision	recall	support
AFIB	0.651583	0.641363	0.662204	875.0
GSVT	0.627843	0.645842	0.610893	691.0
SB	0.659758	0.626315	0.697013	1553.0
SR	0.536050	0.596515	0.486799	907.0
macro avg	0.618808	0.627509	0.614227	4026.0
micro avg	0.627308	0.627308	0.627308	4026.0
weighted avg	0.624634	0.626223	0.627308	4026.0

The result of Model Number:Dataset Number:Featuregroup Number:Rescale of 65 : 1 : 8 : 0

	f1-score	precision	recall	support
AFIB	0.827882	0.829553	0.826234	204.0
GSVT	0.850914	0.848384	0.853463	300.0
SB	0.865013	0.864242	0.865788	892.0
SR	0.857578	0.859225	0.855949	681.0
macro avg	0.850347	0.850351	0.850359	2077.0
micro avg	0.856897	0.856897	0.856897	2077.0
weighted avg	0.856892	0.856900	0.856897	2077.0

The result of Model Number:Dataset Number:Featuregroup Number:Rescale of 65 : 1 : 8 : 1

	f1-score	precision	recall	support
AFIB	0.871968	0.885188	0.859159	204.0
GSVT	0.885989	0.878520	0.893632	300.0
SB	0.905641	0.902720	0.908584	892.0
SR	0.895919	0.899247	0.892641	681.0
macro avg	0.889879	0.891419	0.888504	2077.0
micro avg	0.896342	0.896342	0.896342	2077.0
weighted avg	0.896308	0.896364	0.896342	2077.0

The result of Model Number:Dataset Number:Featuregroup Number:Rescale of 65 : 1 : 9 : 0

	f1-score	precision	recall	support
AFIB	0.822325	0.811749	0.833239	875.0
GSVT	0.839065	0.846011	0.832266	691.0
SB	0.897129	0.893136	0.901168	1553.0
SR	0.871598	0.884490	0.859099	907.0
macro avg	0.857529	0.858846	0.856443	4026.0
micro avg	0.865101	0.865101	0.865101	4026.0
weighted avg	0.865154	0.865411	0.865101	4026.0

The result of Model Number:Dataset Number:Featuregroup Number:Rescale of 65 : 1 : 9 : 1

	f1-score	precision	recall	support
AFIB	0.795250	0.798883	0.791695	875.0
GSVT	0.808508	0.801670	0.815522	691.0
SB	0.854609	0.845882	0.863523	1553.0
SR	0.810503	0.827589	0.794151	907.0
macro avg	0.817218	0.818506	0.816223	4026.0
micro avg	0.824045	0.824045	0.824045	4026.0
weighted avg	0.823859	0.823958	0.824045	4026.0

The result of Model Number:Dataset Number:Featuregroup Number:Rescale of 65 : 1 : 10 : 0

	f1-score	precision	recall	support
AFIB	0.775262	0.765092	0.785740	875.0
GSVT	0.787131	0.795880	0.778586	691.0
SB	0.834854	0.832883	0.836839	1553.0

SR	0.813951	0.821277	0.806767	907.0
macro avg	0.802799	0.803783	0.801983	4026.0
micro avg	0.808960	0.808960	0.808960	4026.0
weighted avg	0.809002	0.809184	0.808960	4026.0

The result of Model Number:Dataset Number:Featuregroup Number:Rescale of 65 : 1 : 10 : 1

	f1-score	precision	recall	support
AFIB	0.733829	0.743753	0.724475	204.0
GSVT	0.761703	0.760467	0.762970	300.0
SB	0.778244	0.777208	0.779291	892.0
SR	0.770290	0.769303	0.771306	681.0
macro avg	0.761017	0.762682	0.759510	2077.0
micro avg	0.768932	0.768932	0.768932	2077.0
weighted avg	0.768884	0.768912	0.768932	2077.0

The result of Model Number:Dataset Number:Featuregroup Number:Rescale of 66 : 0 : 0 : 0

	f1-score	precision	recall	support
AFIB	0.821445	0.818918	0.824039	875.0
GSVT	0.800505	0.804431	0.796745	691.0
SB	0.841555	0.806632	0.879647	1553.0
SR	0.724228	0.790174	0.668499	907.0
macro avg	0.796933	0.805039	0.792232	4026.0
micro avg	0.805764	0.805764	0.805764	4026.0
weighted avg	0.803707	0.805217	0.805764	4026.0

The result of Model Number:Dataset Number:Featuregroup Number:Rescale of 66 : 0 : 0 : 1

	f1-score	precision	recall	support
AFIB	0.703949	0.713646	0.694822	875.0
GSVT	0.719317	0.716338	0.722516	691.0
SB	0.766185	0.756139	0.776533	1553.0
SR	0.742130	0.753213	0.731698	907.0
macro avg	0.732895	0.734834	0.731392	4026.0
micro avg	0.739402	0.739402	0.739402	4026.0
weighted avg	0.739195	0.739413	0.739402	4026.0

The result of Model Number:Dataset Number:Featuregroup Number:Rescale of 66 : 0 : 1 : 0

	f1-score	precision	recall	support
AFIB	0.911028	0.927420	0.895474	204.0
GSVT	0.931581	0.918482	0.945212	300.0
SB	0.945915	0.934738	0.957366	892.0
SR	0.926891	0.943393	0.910966	681.0
macro avg	0.928853	0.931008	0.927255	2077.0
micro avg	0.934318	0.934318	0.934318	2077.0
weighted avg	0.934180	0.934509	0.934318	2077.0

The result of Model Number:Dataset Number:Featuregroup Number:Rescale of 66 : 0 : 1 : 1

	f1-score	precision	recall	support
AFIB	0.887137	0.903419	0.871860	204.0
GSVT	0.920815	0.913411	0.928508	300.0
SB	0.937896	0.936222	0.939578	892.0
SR	0.928609	0.929436	0.927789	681.0
macro avg	0.918614	0.920622	0.916934	2077.0
micro avg	0.927463	0.927463	0.927463	2077.0
weighted avg	0.927398	0.927480	0.927463	2077.0

The result of Model Number:Dataset Number:Featuregroup Number:Rescale of 66 : 0 : 2 : 0

	f1-score	precision	recall	support
AFIB	0.789844	0.793452	0.786313	875.0
GSVT	0.803012	0.796220	0.809978	691.0
SB	0.848799	0.840132	0.857652	1553.0
SR	0.804993	0.821963	0.788752	907.0
macro avg	0.811662	0.812942	0.810674	4026.0
micro avg	0.818443	0.818443	0.818443	4026.0
weighted avg	0.818258	0.818357	0.818443	4026.0

The result of Model Number:Dataset Number:Featuregroup Number:Rescale of 66 : 0 : 2 : 1

	f1-score	precision	recall	support
AFIB	0.660413	0.680441	0.641558	875.0
GSVT	0.705000	0.700111	0.709996	691.0
SB	0.771356	0.768405	0.774335	1553.0
SR	0.729517	0.718568	0.740848	907.0
macro avg	0.716571	0.716881	0.716684	4026.0
micro avg	0.726890	0.726890	0.726890	4026.0
weighted avg	0.726429	0.726338	0.726890	4026.0

The result of Model Number:Dataset Number:Featuregroup Number:Rescale of 66 : 0 : 3 : 0

	f1-score	precision	recall	support
AFIB	0.796787	0.856684	0.745549	204.0
GSVT	0.759872	0.783991	0.737308	300.0
SB	0.791679	0.752503	0.835163	892.0
SR	0.700813	0.730936	0.673147	681.0
macro avg	0.762288	0.781029	0.747792	2077.0
micro avg	0.759106	0.759106	0.759106	2077.0
weighted avg	0.757794	0.760213	0.759106	2077.0

The result of Model Number:Dataset Number:Featuregroup Number:Rescale of 66 : 0 : 3 : 1

	f1-score	precision	recall	support
AFIB	0.674324	0.680391	0.668575	875.0

GSVT 0.688060 0.674108 0.702763 691.0
SB 0.729692 0.721574 0.738082 1553.0
SR 0.700736 0.721566 0.681241 907.0
macro avg 0.698203 0.699410 0.697665 4026.0
micro avg 0.704108 0.704108 0.704108 4026.0
weighted avg 0.703990 0.704475 0.704108 4026.0
The result of Model Number:Dataset Number:Featuregroup Number:Rescale of 66 : 0 : 4 : 0

	f1-score	precision	recall	support
AFIB	0.694178	0.699783	0.688723	204.0
GSVT	0.710107	0.706625	0.713648	300.0
SB	0.723966	0.723163	0.724777	892.0
SR	0.718560	0.719515	0.717612	681.0
macro avg	0.711703	0.712272	0.711190	2077.0
micro avg	0.717279	0.717279	0.717279	2077.0
weighted avg	0.717266	0.717282	0.717279	2077.0

The result of Model Number:Dataset Number:Featuregroup Number:Rescale of 66 : 0 : 4 : 1

	f1-score	precision	recall	support
AFIB	0.697503	0.712133	0.683480	204.0
GSVT	0.713684	0.709461	0.717964	300.0
SB	0.731083	0.729370	0.732803	892.0
SR	0.724907	0.724699	0.725122	681.0
macro avg	0.716794	0.718916	0.714843	2077.0
micro avg	0.723297	0.723297	0.723297	2077.0
weighted avg	0.723247	0.723270	0.723297	2077.0

The result of Model Number:Dataset Number:Featuregroup Number:Rescale of 66 : 0 : 5 : 0

	f1-score	precision	recall	support
AFIB	0.766302	0.773260	0.759862	204.0
GSVT	0.791629	0.786845	0.796679	300.0
SB	0.808191	0.807108	0.809278	892.0
SR	0.800713	0.802373	0.799065	681.0
macro avg	0.791709	0.792396	0.791221	2077.0
micro avg	0.799256	0.799256	0.799256	2077.0
weighted avg	0.799233	0.799304	0.799256	2077.0

The result of Model Number:Dataset Number:Featuregroup Number:Rescale of 66 : 0 : 5 : 1

	f1-score	precision	recall	support
AFIB	0.699921	0.745525	0.659884	204.0
GSVT	0.730583	0.712625	0.749569	300.0
SB	0.752009	0.742349	0.761951	892.0
SR	0.732477	0.741223	0.723983	681.0
macro avg	0.728748	0.735431	0.723847	2077.0

micro avg 0.737689 0.737689 0.737689 2077.0
weighted avg 0.737394 0.737998 0.737689 2077.0
The result of Model Number:Dataset Number:Featuregroup Number:Rescale of 66 : 0 : 6 : 0
f1-score precision recall support
AFIB 0.883077 0.872661 0.893780 875.0
GSVT 0.900429 0.908260 0.892740 691.0
SB 0.959635 0.955962 0.963340 1553.0
SR 0.936993 0.948322 0.925942 907.0
macro avg 0.920034 0.921301 0.918951 4026.0
micro avg 0.927679 0.927679 0.927679 4026.0
weighted avg 0.927733 0.927949 0.927679 4026.0
The result of Model Number:Dataset Number:Featuregroup Number:Rescale of 66 : 0 : 6 : 1
f1-score precision recall support
AFIB 0.643510 0.629686 0.657989 875.0
GSVT 0.666422 0.676086 0.657074 691.0
SB 0.709432 0.709297 0.709570 1553.0
SR 0.691652 0.699691 0.683799 907.0
macro avg 0.677754 0.678690 0.677108 4026.0
micro avg 0.683544 0.683544 0.683544 4026.0
weighted avg 0.683717 0.684130 0.683544 4026.0
The result of Model Number:Dataset Number:Featuregroup Number:Rescale of 66 : 0 : 7 : 0
f1-score precision recall support
AFIB 0.662770 0.668886 0.656800 875.0
GSVT 0.678246 0.676415 0.680110 691.0
SB 0.722341 0.715551 0.729268 1553.0
SR 0.702383 0.709431 0.695488 907.0
macro avg 0.691435 0.692571 0.690417 4026.0
micro avg 0.697471 0.697471 0.697471 4026.0
weighted avg 0.697330 0.697313 0.697471 4026.0
The result of Model Number:Dataset Number:Featuregroup Number:Rescale of 66 : 0 : 7 : 1
f1-score precision recall support
AFIB 0.920296 0.921331 0.919431 204.0
GSVT 0.941425 0.939323 0.943608 300.0
SB 0.961379 0.960096 0.962671 892.0
SR 0.954179 0.956582 0.951800 681.0
macro avg 0.944320 0.944333 0.944378 2077.0
micro avg 0.952106 0.952106 0.952106 2077.0
weighted avg 0.952101 0.952136 0.952106 2077.0
The result of Model Number:Dataset Number:Featuregroup Number:Rescale of 66 : 0 : 8 : 0

	f1-score	precision	recall	support
AFIB	0.747707	0.730715	0.765559	875.0
GSVT	0.773332	0.785872	0.761248	691.0
SB	0.825060	0.825065	0.825061	1553.0
SR	0.803944	0.813102	0.794991	907.0
macro avg	0.787511	0.788689	0.786715	4026.0
micro avg	0.794402	0.794402	0.794402	4026.0
weighted avg	0.794613	0.795138	0.794402	4026.0

The result of Model Number:Dataset Number:Featuregroup Number:Rescale of 66 : 0 : 8 : 1

	f1-score	precision	recall	support
AFIB	0.716865	0.731929	0.702435	875.0
GSVT	0.763377	0.765999	0.760810	691.0
SB	0.831710	0.827790	0.835670	1553.0
SR	0.787306	0.776822	0.798144	907.0
macro avg	0.774814	0.775635	0.774265	4026.0
micro avg	0.785410	0.785410	0.785410	4026.0
weighted avg	0.785018	0.784868	0.785410	4026.0

The result of Model Number:Dataset Number:Featuregroup Number:Rescale of 66 : 0 : 9 : 0

	f1-score	precision	recall	support
AFIB	0.697951	0.715331	0.681429	875.0
GSVT	0.737435	0.740566	0.734361	691.0
SB	0.799882	0.796822	0.802971	1553.0
SR	0.760864	0.746685	0.775631	907.0
macro avg	0.749033	0.749851	0.748598	4026.0
micro avg	0.758620	0.758620	0.758620	4026.0
weighted avg	0.758220	0.758160	0.758620	4026.0

The result of Model Number:Dataset Number:Featuregroup Number:Rescale of 66 : 0 : 9 : 1

	f1-score	precision	recall	support
AFIB	0.824037	0.840639	0.808606	204.0
GSVT	0.850478	0.843647	0.857584	300.0
SB	0.868890	0.868015	0.869767	892.0
SR	0.860969	0.860475	0.861474	681.0
macro avg	0.851093	0.853194	0.849358	2077.0
micro avg	0.859281	0.859281	0.859281	2077.0
weighted avg	0.859228	0.859334	0.859281	2077.0

The result of Model Number:Dataset Number:Featuregroup Number:Rescale of 66 : 0 : 10 : 0

	f1-score	precision	recall	support
AFIB	0.893119	0.894909	0.891374	875.0
GSVT	0.895331	0.890497	0.900241	691.0
SB	0.935921	0.930921	0.940982	1553.0

SR 0.913531 0.924364 0.902974 907.0
macro avg 0.909475 0.910173 0.908893 4026.0
micro avg 0.914645 0.914645 0.914645 4026.0
weighted avg 0.914608 0.914679 0.914645 4026.0
The result of Model Number:Dataset Number:Featuregroup Number:Rescale of 66 : 0 : 10 : 1

	f1-score	precision	recall	support
AFIB	0.916754	0.931281	0.902795	204.0
GSVT	0.940318	0.932316	0.948463	300.0
SB	0.956553	0.954421	0.958698	892.0
SR	0.947490	0.949596	0.945411	681.0
macro avg	0.940279	0.941903	0.938842	2077.0
micro avg	0.947372	0.947372	0.947372	2077.0
weighted avg	0.947327	0.947373	0.947372	2077.0

The result of Model Number:Dataset Number:Featuregroup Number:Rescale of 66 : 1 : 0 : 0

	f1-score	precision	recall	support
AFIB	0.870989	0.875137	0.866919	875.0
GSVT	0.881749	0.865725	0.898468	691.0
SB	0.932957	0.929385	0.936560	1553.0
SR	0.905381	0.920878	0.890411	907.0
macro avg	0.897769	0.897781	0.898090	4026.0
micro avg	0.904490	0.904490	0.904490	4026.0
weighted avg	0.904488	0.904752	0.904490	4026.0

The result of Model Number:Dataset Number:Featuregroup Number:Rescale of 66 : 1 : 0 : 1

	f1-score	precision	recall	support
AFIB	0.686270	0.761619	0.624652	204.0
GSVT	0.801313	0.791295	0.811745	300.0
SB	0.852558	0.848207	0.856954	892.0
SR	0.832585	0.820945	0.844564	681.0
macro avg	0.793181	0.805517	0.784479	2077.0
micro avg	0.823545	0.823545	0.823545	2077.0
weighted avg	0.822275	0.822544	0.823545	2077.0

The result of Model Number:Dataset Number:Featuregroup Number:Rescale of 66 : 1 : 1 : 0

	f1-score	precision	recall	support
AFIB	0.651766	0.650120	0.653523	875.0
GSVT	0.664482	0.660829	0.668212	691.0
SB	0.695765	0.691570	0.700020	1553.0
SR	0.675251	0.687438	0.663501	907.0
macro avg	0.671816	0.672489	0.671314	4026.0
micro avg	0.676228	0.676228	0.676228	4026.0
weighted avg	0.676212	0.676354	0.676228	4026.0

The result of Model Number:Dataset Number:Featuregroup Number:Rescale of 66 : 1 : 1 : 1

	f1-score	precision	recall	support
AFIB	0.875189	0.902744	0.849481	204.0
GSVT	0.831477	0.849237	0.814582	300.0
SB	0.858518	0.828476	0.890852	892.0
SR	0.784044	0.810542	0.759279	681.0
macro avg	0.837307	0.847750	0.828549	2077.0
micro avg	0.832633	0.832633	0.832633	2077.0
weighted avg	0.831831	0.832889	0.832633	2077.0

The result of Model Number:Dataset Number:Featuregroup Number:Rescale of 66 : 1 : 2 : 0

	f1-score	precision	recall	support
AFIB	0.672914	0.745424	0.613799	204.0
GSVT	0.793190	0.782071	0.804833	300.0
SB	0.842175	0.839464	0.844913	892.0
SR	0.820613	0.808131	0.833508	681.0
macro avg	0.782223	0.793773	0.774263	2077.0
micro avg	0.812685	0.812685	0.812685	2077.0
weighted avg	0.811406	0.811665	0.812685	2077.0

The result of Model Number:Dataset Number:Featuregroup Number:Rescale of 66 : 1 : 2 : 1

	f1-score	precision	recall	support
AFIB	0.556566	0.620744	0.504489	204.0
GSVT	0.656248	0.644826	0.668222	300.0
SB	0.700671	0.697565	0.703811	892.0
SR	0.684411	0.675052	0.694052	681.0
macro avg	0.649474	0.659547	0.642644	2077.0
micro avg	0.675894	0.675894	0.675894	2077.0
weighted avg	0.674769	0.675021	0.675894	2077.0

The result of Model Number:Dataset Number:Featuregroup Number:Rescale of 66 : 1 : 3 : 0

	f1-score	precision	recall	support
AFIB	0.696906	0.709691	0.684628	204.0
GSVT	0.706098	0.698972	0.713385	300.0
SB	0.723491	0.719725	0.727301	892.0
SR	0.713641	0.718113	0.709240	681.0
macro avg	0.710034	0.711625	0.708638	2077.0
micro avg	0.715178	0.715178	0.715178	2077.0
weighted avg	0.715138	0.715213	0.715178	2077.0

The result of Model Number:Dataset Number:Featuregroup Number:Rescale of 66 : 1 : 3 : 1

	f1-score	precision	recall	support
AFIB	0.752518	0.759797	0.745513	204.0

	f1-score	precision	recall	support
GSVT	0.779146	0.777610	0.780701	300.0
SB	0.795431	0.794023	0.796856	892.0
SR	0.788099	0.788480	0.787751	681.0
macro avg	0.778798	0.779977	0.777705	2077.0
micro avg	0.786494	0.786494	0.786494	2077.0
weighted avg	0.786460	0.786473	0.786494	2077.0

The result of Model Number:Dataset Number:Featuregroup Number:Rescale of 66 : 1 : 4 : 0

	f1-score	precision	recall	support
AFIB	0.839116	0.836691	0.841633	875.0
GSVT	0.854669	0.853973	0.855424	691.0
SB	0.907782	0.904022	0.911581	1553.0
SR	0.880529	0.890203	0.871112	907.0
macro avg	0.870524	0.871222	0.869937	4026.0
micro avg	0.877623	0.877623	0.877623	4026.0
weighted avg	0.877603	0.877685	0.877623	4026.0

The result of Model Number:Dataset Number:Featuregroup Number:Rescale of 66 : 1 : 4 : 1

	f1-score	precision	recall	support
AFIB	0.746339	0.738092	0.754786	875.0
GSVT	0.759786	0.764464	0.755177	691.0
SB	0.808646	0.807301	0.810000	1553.0
SR	0.788956	0.796333	0.781735	907.0
macro avg	0.775932	0.776547	0.775425	4026.0
micro avg	0.782223	0.782223	0.782223	4026.0
weighted avg	0.782283	0.782436	0.782223	4026.0

The result of Model Number:Dataset Number:Featuregroup Number:Rescale of 66 : 1 : 5 : 0

	f1-score	precision	recall	support
AFIB	0.809960	0.819430	0.800862	204.0
GSVT	0.832241	0.828415	0.836126	300.0
SB	0.844025	0.841400	0.846672	892.0
SR	0.836756	0.839127	0.834433	681.0
macro avg	0.830745	0.832093	0.829523	2077.0
micro avg	0.836636	0.836636	0.836636	2077.0
weighted avg	0.836593	0.836621	0.836636	2077.0

The result of Model Number:Dataset Number:Featuregroup Number:Rescale of 66 : 1 : 5 : 1

	f1-score	precision	recall	support
AFIB	0.859906	0.847995	0.872190	875.0
GSVT	0.878978	0.886091	0.871982	691.0
SB	0.933024	0.930343	0.935727	1553.0
SR	0.910327	0.922175	0.898788	907.0
macro avg	0.895559	0.896651	0.894672	4026.0

micro avg	0.902655	0.902655	0.902655	4026.0
weighted avg	0.902743	0.903011	0.902655	4026.0

The result of Model Number:Dataset Number:Featuregroup Number:Rescale of 66 : 1 : 6 : 0

	f1-score	precision	recall	support
AFIB	0.889900	0.918804	0.862934	204.0
GSVT	0.914469	0.902809	0.926618	300.0
SB	0.940268	0.936723	0.943851	892.0
SR	0.927117	0.928796	0.925480	681.0
macro avg	0.917939	0.921783	0.914721	2077.0
micro avg	0.927391	0.927391	0.927391	2077.0
weighted avg	0.927283	0.927465	0.927391	2077.0

The result of Model Number:Dataset Number:Featuregroup Number:Rescale of 66 : 1 : 6 : 1

	f1-score	precision	recall	support
AFIB	0.916113	0.926082	0.906624	204.0
GSVT	0.947116	0.943166	0.951252	300.0
SB	0.966524	0.965659	0.967391	892.0
SR	0.957390	0.957398	0.957390	681.0
macro avg	0.946786	0.948076	0.945664	2077.0
micro avg	0.955812	0.955812	0.955812	2077.0
weighted avg	0.955774	0.955814	0.955812	2077.0

The result of Model Number:Dataset Number:Featuregroup Number:Rescale of 66 : 1 : 7 : 0

	f1-score	precision	recall	support
AFIB	0.914730	0.926949	0.903087	204.0
GSVT	0.930889	0.925219	0.936773	300.0
SB	0.953329	0.952484	0.954182	892.0
SR	0.946284	0.946428	0.946147	681.0
macro avg	0.936308	0.937770	0.935047	2077.0
micro avg	0.944014	0.944014	0.944014	2077.0
weighted avg	0.943987	0.944052	0.944014	2077.0

The result of Model Number:Dataset Number:Featuregroup Number:Rescale of 66 : 1 : 7 : 1

	f1-score	precision	recall	support
AFIB	0.747756	0.761516	0.734865	204.0
GSVT	0.775344	0.768850	0.782083	300.0
SB	0.789903	0.788579	0.791231	892.0
SR	0.782928	0.783624	0.782239	681.0
macro avg	0.773983	0.775642	0.772604	2077.0
micro avg	0.781425	0.781425	0.781425	2077.0
weighted avg	0.781374	0.781447	0.781425	2077.0

The result of Model Number:Dataset Number:Featuregroup Number:Rescale of 66 : 1 : 8 : 0

	f1-score	precision	recall	support
AFIB	0.826903	0.839101	0.815145	204.0
GSVT	0.847202	0.843877	0.850581	300.0
SB	0.866018	0.864281	0.867765	892.0
SR	0.859875	0.860005	0.859753	681.0
macro avg	0.850000	0.851816	0.848311	2077.0
micro avg	0.857488	0.857488	0.857488	2077.0
weighted avg	0.857444	0.857459	0.857488	2077.0

The result of Model Number:Dataset Number:Featuregroup Number:Rescale of 66 : 1 : 8 : 1

	f1-score	precision	recall	support
AFIB	0.788162	0.812064	0.765659	875.0
GSVT	0.841374	0.835539	0.847336	691.0
SB	0.920565	0.917044	0.924120	1553.0
SR	0.870633	0.857566	0.884156	907.0
macro avg	0.855183	0.855553	0.855318	4026.0
micro avg	0.867498	0.867498	0.867498	4026.0
weighted avg	0.866948	0.866839	0.867498	4026.0

The result of Model Number:Dataset Number:Featuregroup Number:Rescale of 66 : 1 : 9 : 0

	f1-score	precision	recall	support
AFIB	0.693779	0.727412	0.663195	204.0
GSVT	0.722673	0.713230	0.732516	300.0
SB	0.742137	0.736493	0.747878	892.0
SR	0.721473	0.723629	0.719362	681.0
macro avg	0.720015	0.725191	0.715738	2077.0
micro avg	0.727992	0.727992	0.727992	2077.0
weighted avg	0.727801	0.728023	0.727992	2077.0

The result of Model Number:Dataset Number:Featuregroup Number:Rescale of 66 : 1 : 9 : 1

	f1-score	precision	recall	support
AFIB	0.680695	0.765381	0.613482	204.0
GSVT	0.807594	0.790349	0.825645	300.0
SB	0.862122	0.857727	0.866566	892.0
SR	0.842781	0.832108	0.853785	681.0
macro avg	0.798298	0.811391	0.789869	2077.0
micro avg	0.831607	0.831607	0.831607	2077.0
weighted avg	0.830085	0.830525	0.831607	2077.0

The result of Model Number:Dataset Number:Featuregroup Number:Rescale of 66 : 1 : 10 : 0

	f1-score	precision	recall	support
AFIB	0.788187	0.790726	0.785738	875.0
GSVT	0.799268	0.786579	0.812446	691.0
SB	0.842159	0.835128	0.849316	1553.0

SR 0.799826 0.820140 0.780524 907.0
macro avg 0.807360 0.808143 0.807006 4026.0
micro avg 0.813672 0.813672 0.813672 4026.0
weighted avg 0.813531 0.813768 0.813672 4026.0
The result of Model Number:Dataset Number:Featuregroup Number:Rescale of 66 : 1 : 10 : 1

	f1-score	precision	recall	support
AFIB	0.726119	0.733217	0.719326	875.0
GSVT	0.720573	0.715105	0.726361	691.0
SB	0.762448	0.754603	0.770525	1553.0
SR	0.738246	0.750119	0.726952	907.0
macro avg	0.736846	0.738261	0.735791	4026.0
micro avg	0.742001	0.742001	0.742001	4026.0
weighted avg	0.741913	0.742166	0.742001	4026.0

The result of Model Number:Dataset Number:Featuregroup Number:Rescale of 67 : 0 : 0 : 0

	f1-score	precision	recall	support
AFIB	0.751800	0.776766	0.728674	204.0
GSVT	0.780331	0.774071	0.786811	300.0
SB	0.797571	0.794294	0.800878	892.0
SR	0.790122	0.790007	0.790239	681.0
macro avg	0.779956	0.783784	0.776650	2077.0
micro avg	0.788266	0.788266	0.788266	2077.0
weighted avg	0.788143	0.788246	0.788266	2077.0

The result of Model Number:Dataset Number:Featuregroup Number:Rescale of 67 : 0 : 0 : 1

	f1-score	precision	recall	support
AFIB	0.904967	0.941494	0.871241	204.0
GSVT	0.938644	0.923396	0.954566	300.0
SB	0.962927	0.955186	0.970807	892.0
SR	0.937021	0.943769	0.930419	681.0
macro avg	0.935890	0.940961	0.931758	2077.0
micro avg	0.945440	0.945440	0.945440	2077.0
weighted avg	0.945233	0.945506	0.945440	2077.0

The result of Model Number:Dataset Number:Featuregroup Number:Rescale of 67 : 0 : 1 : 0

	f1-score	precision	recall	support
AFIB	0.751817	0.751140	0.752568	875.0
GSVT	0.764842	0.757839	0.772035	691.0
SB	0.802610	0.795554	0.809793	1553.0
SR	0.764824	0.783732	0.746874	907.0
macro avg	0.771023	0.772066	0.770318	4026.0
micro avg	0.776701	0.776701	0.776701	4026.0
weighted avg	0.776576	0.776764	0.776701	4026.0

The result of Model Number:Dataset Number:Featuregroup Number:Rescale of 67 : 0 : 1 : 1

	f1-score	precision	recall	support
AFIB	0.649913	0.640200	0.659951	875.0
GSVT	0.662374	0.668039	0.656815	691.0
SB	0.707732	0.705295	0.710190	1553.0
SR	0.687017	0.697264	0.677090	907.0
macro avg	0.676759	0.677699	0.676012	4026.0
micro avg	0.682653	0.682653	0.682653	4026.0
weighted avg	0.682714	0.682944	0.682653	4026.0

The result of Model Number:Dataset Number:Featuregroup Number:Rescale of 67 : 0 : 2 : 0

	f1-score	precision	recall	support
AFIB	0.885377	0.895011	0.876207	204.0
GSVT	0.915339	0.911522	0.919337	300.0
SB	0.934097	0.933261	0.934934	892.0
SR	0.925269	0.925277	0.925269	681.0
macro avg	0.915021	0.916268	0.913937	2077.0
micro avg	0.923744	0.923744	0.923744	2077.0
weighted avg	0.923708	0.923747	0.923744	2077.0

The result of Model Number:Dataset Number:Featuregroup Number:Rescale of 67 : 0 : 2 : 1

	f1-score	precision	recall	support
AFIB	0.777287	0.766877	0.788033	875.0
GSVT	0.794666	0.800869	0.788572	691.0
SB	0.843714	0.841074	0.846375	1553.0
SR	0.823311	0.834225	0.812692	907.0
macro avg	0.809744	0.810761	0.808918	4026.0
micro avg	0.816186	0.816186	0.816186	4026.0
weighted avg	0.816262	0.816505	0.816186	4026.0

The result of Model Number:Dataset Number:Featuregroup Number:Rescale of 67 : 0 : 3 : 0

	f1-score	precision	recall	support
AFIB	0.790103	0.797212	0.783367	875.0
GSVT	0.806197	0.789851	0.823425	691.0
SB	0.854978	0.845466	0.864808	1553.0
SR	0.821051	0.845457	0.798207	907.0
macro avg	0.818082	0.819496	0.817452	4026.0
micro avg	0.825001	0.825001	0.825001	4026.0
weighted avg	0.824862	0.825431	0.825001	4026.0

The result of Model Number:Dataset Number:Featuregroup Number:Rescale of 67 : 0 : 3 : 1

	f1-score	precision	recall	support
AFIB	0.723838	0.727811	0.719955	875.0

GSVT 0.724850 0.715686 0.734366 691.0
SB 0.762306 0.759247 0.765396 1553.0
SR 0.745941 0.754997 0.737147 907.0
macro avg 0.739234 0.739435 0.739216 4026.0
micro avg 0.743830 0.743830 0.743830 4026.0
weighted avg 0.743830 0.743981 0.743830 4026.0
The result of Model Number:Dataset Number:Featuregroup Number:Rescale of 67 : 0 : 4 : 0

	f1-score	precision	recall	support
AFIB	0.733412	0.735562	0.731331	875.0
GSVT	0.745547	0.735972	0.755472	691.0
SB	0.789038	0.787120	0.790968	1553.0
SR	0.769058	0.778185	0.760151	907.0
macro avg	0.759264	0.759210	0.759480	4026.0
micro avg	0.764972	0.764972	0.764972	4026.0
weighted avg	0.764983	0.765123	0.764972	4026.0

The result of Model Number:Dataset Number:Featuregroup Number:Rescale of 67 : 0 : 4 : 1

	f1-score	precision	recall	support
AFIB	0.846867	0.858318	0.836071	204.0
GSVT	0.879034	0.877607	0.880496	300.0
SB	0.898123	0.896927	0.899331	892.0
SR	0.888943	0.887804	0.890116	681.0
macro avg	0.878242	0.880164	0.876504	2077.0
micro avg	0.887376	0.887376	0.887376	2077.0
weighted avg	0.887322	0.887353	0.887376	2077.0

The result of Model Number:Dataset Number:Featuregroup Number:Rescale of 67 : 0 : 5 : 0

	f1-score	precision	recall	support
AFIB	0.662443	0.679948	0.645882	204.0
GSVT	0.680610	0.674956	0.686484	300.0
SB	0.699365	0.696798	0.701952	892.0
SR	0.688536	0.689370	0.687722	681.0
macro avg	0.682738	0.685268	0.680510	2077.0
micro avg	0.689545	0.689545	0.689545	2077.0
weighted avg	0.689479	0.689553	0.689545	2077.0

The result of Model Number:Dataset Number:Featuregroup Number:Rescale of 67 : 0 : 5 : 1

	f1-score	precision	recall	support
AFIB	0.740200	0.719670	0.764265	875.0
GSVT	0.749863	0.781448	0.725346	691.0
SB	0.823750	0.823334	0.824178	1553.0
SR	0.803085	0.808110	0.798127	907.0
macro avg	0.779224	0.783141	0.777979	4026.0

micro avg 0.788325 0.788325 0.788325 4026.0
weighted avg 0.788254 0.790185 0.788325 4026.0
The result of Model Number:Dataset Number:Featuregroup Number:Rescale of 67 : 0 : 6 : 0

	f1-score	precision	recall	support
AFIB	0.682362	0.706051	0.660303	204.0
GSVT	0.695446	0.687833	0.703315	300.0
SB	0.713960	0.708833	0.719162	892.0
SR	0.701209	0.704657	0.697807	681.0
macro avg	0.698244	0.701843	0.695147	2077.0
micro avg	0.704090	0.704090	0.704090	2077.0
weighted avg	0.704001	0.704157	0.704090	2077.0

The result of Model Number:Dataset Number:Featuregroup Number:Rescale of 67 : 0 : 6 : 1

	f1-score	precision	recall	support
AFIB	0.811421	0.815442	0.807526	875.0
GSVT	0.819500	0.820681	0.818447	691.0
SB	0.862634	0.858632	0.866683	1553.0
SR	0.846754	0.848875	0.844691	907.0
macro avg	0.835077	0.835907	0.834337	4026.0
micro avg	0.840593	0.840593	0.840593	4026.0
weighted avg	0.840523	0.840533	0.840593	4026.0

The result of Model Number:Dataset Number:Featuregroup Number:Rescale of 67 : 0 : 7 : 0

	f1-score	precision	recall	support
AFIB	0.874232	0.881078	0.867537	875.0
GSVT	0.876538	0.865351	0.888071	691.0
SB	0.919389	0.915701	0.923116	1553.0
SR	0.899965	0.908799	0.891334	907.0
macro avg	0.892531	0.892732	0.892514	4026.0
micro avg	0.897862	0.897862	0.897862	4026.0
weighted avg	0.897844	0.897980	0.897862	4026.0

The result of Model Number:Dataset Number:Featuregroup Number:Rescale of 67 : 0 : 7 : 1

	f1-score	precision	recall	support
AFIB	0.822531	0.829932	0.815519	875.0
GSVT	0.839286	0.822268	0.857221	691.0
SB	0.890068	0.880167	0.900303	1553.0
SR	0.854749	0.880157	0.830968	907.0
macro avg	0.851659	0.853131	0.851003	4026.0
micro avg	0.858862	0.858862	0.858862	4026.0
weighted avg	0.858717	0.859309	0.858862	4026.0

The result of Model Number:Dataset Number:Featuregroup Number:Rescale of 67 : 0 : 8 : 0

	f1-score	precision	recall	support
AFIB	0.704671	0.708012	0.701367	875.0
GSVT	0.713413	0.710047	0.716827	691.0
SB	0.752484	0.750895	0.754083	1553.0
SR	0.738224	0.740291	0.736186	907.0
macro avg	0.727198	0.727311	0.727116	4026.0
micro avg	0.732199	0.732199	0.732199	4026.0
weighted avg	0.732174	0.732175	0.732199	4026.0

The result of Model Number:Dataset Number:Featuregroup Number:Rescale of 67 : 0 : 8 : 1

	f1-score	precision	recall	support
AFIB	0.889886	0.911240	0.869914	204.0
GSVT	0.921528	0.916187	0.927043	300.0
SB	0.941209	0.937756	0.944690	892.0
SR	0.933030	0.933722	0.932349	681.0
macro avg	0.921413	0.924726	0.918499	2077.0
micro avg	0.930750	0.930750	0.930750	2077.0
weighted avg	0.930644	0.930714	0.930750	2077.0

The result of Model Number:Dataset Number:Featuregroup Number:Rescale of 67 : 0 : 9 : 0

	f1-score	precision	recall	support
AFIB	0.883192	0.890174	0.876348	875.0
GSVT	0.905416	0.903501	0.907372	691.0
SB	0.965200	0.956668	0.973896	1553.0
SR	0.939505	0.948719	0.930490	907.0
macro avg	0.923328	0.924766	0.922027	4026.0
micro avg	0.931499	0.931499	0.931499	4026.0
weighted avg	0.931326	0.931300	0.931499	4026.0

The result of Model Number:Dataset Number:Featuregroup Number:Rescale of 67 : 0 : 9 : 1

	f1-score	precision	recall	support
AFIB	0.855108	0.843230	0.867407	875.0
GSVT	0.885665	0.897480	0.874272	691.0
SB	0.940864	0.938936	0.942803	1553.0
SR	0.916376	0.923457	0.909404	907.0
macro avg	0.899503	0.900776	0.898471	4026.0
micro avg	0.907130	0.907130	0.907130	4026.0
weighted avg	0.907235	0.907533	0.907130	4026.0

The result of Model Number:Dataset Number:Featuregroup Number:Rescale of 67 : 0 : 10 : 0

	f1-score	precision	recall	support
AFIB	0.872804	0.880761	0.865104	204.0
GSVT	0.895582	0.892649	0.898561	300.0
SB	0.911954	0.911346	0.912566	892.0

SR 0.903933 0.903673 0.904206 681.0
macro avg 0.896068 0.897107 0.895109 2077.0
micro avg 0.903140 0.903140 0.903140 2077.0
weighted avg 0.903114 0.903126 0.903140 2077.0
The result of Model Number:Dataset Number:Featuregroup Number:Rescale of 67 : 0 : 10 : 1

	f1-score	precision	recall	support
AFIB	0.701320	0.715637	0.687875	204.0
GSVT	0.726786	0.720472	0.733345	300.0
SB	0.740760	0.739437	0.742088	892.0
SR	0.733865	0.734302	0.733432	681.0
macro avg	0.725683	0.727462	0.724185	2077.0
micro avg	0.732663	0.732663	0.732663	2077.0
weighted avg	0.732607	0.732677	0.732663	2077.0

The result of Model Number:Dataset Number:Featuregroup Number:Rescale of 67 : 1 : 0 : 0

	f1-score	precision	recall	support
AFIB	0.643616	0.634676	0.652873	875.0
GSVT	0.666616	0.675509	0.658041	691.0
SB	0.708162	0.706712	0.709622	1553.0
SR	0.689731	0.695061	0.684484	907.0
macro avg	0.677031	0.677989	0.676255	4026.0
micro avg	0.682772	0.682772	0.682772	4026.0
weighted avg	0.682851	0.683075	0.682772	4026.0

The result of Model Number:Dataset Number:Featuregroup Number:Rescale of 67 : 1 : 0 : 1

	f1-score	precision	recall	support
AFIB	0.825979	0.814917	0.837398	875.0
GSVT	0.844446	0.851037	0.837971	691.0
SB	0.896566	0.893761	0.899394	1553.0
SR	0.874885	0.886483	0.863602	907.0
macro avg	0.860469	0.861550	0.859591	4026.0
micro avg	0.867314	0.867314	0.867314	4026.0
weighted avg	0.867395	0.867653	0.867314	4026.0

The result of Model Number:Dataset Number:Featuregroup Number:Rescale of 67 : 1 : 1 : 0

	f1-score	precision	recall	support
AFIB	0.844877	0.858265	0.832012	204.0
GSVT	0.866594	0.859219	0.874100	300.0
SB	0.881555	0.879591	0.883532	892.0
SR	0.873203	0.875144	0.871287	681.0
macro avg	0.866557	0.868055	0.865233	2077.0
micro avg	0.873095	0.873095	0.873095	2077.0
weighted avg	0.873053	0.873096	0.873095	2077.0

The result of Model Number:Dataset Number:Featuregroup Number:Rescale of 67 : 1 : 1 : 1

	f1-score	precision	recall	support
AFIB	0.695490	0.698802	0.692240	875.0
GSVT	0.704081	0.691286	0.717431	691.0
SB	0.744971	0.742119	0.747848	1553.0
SR	0.722952	0.735326	0.710998	907.0
macro avg	0.716873	0.716883	0.717129	4026.0
micro avg	0.722240	0.722240	0.722240	4026.0
weighted avg	0.722238	0.722449	0.722240	4026.0

The result of Model Number:Dataset Number:Featuregroup Number:Rescale of 67 : 1 : 2 : 0

	f1-score	precision	recall	support
AFIB	0.700170	0.692606	0.797027	204.0
GSVT	0.837708	0.831182	0.844411	300.0
SB	0.683650	0.682220	0.685087	892.0
SR	0.847741	0.847504	0.847990	681.0
macro avg	0.767318	0.763378	0.793629	2077.0
micro avg	0.772506	0.772506	0.772506	2077.0
weighted avg	0.761326	0.758949	0.772506	2077.0

The result of Model Number:Dataset Number:Featuregroup Number:Rescale of 67 : 1 : 2 : 1

	f1-score	precision	recall	support
AFIB	0.741581	0.759210	0.724835	204.0
GSVT	0.760833	0.757897	0.763872	300.0
SB	0.779687	0.777947	0.781435	892.0
SR	0.769877	0.768329	0.771460	681.0
macro avg	0.762995	0.765846	0.760401	2077.0
micro avg	0.770068	0.770068	0.770068	2077.0
weighted avg	0.770005	0.770057	0.770068	2077.0

The result of Model Number:Dataset Number:Featuregroup Number:Rescale of 67 : 1 : 3 : 0

	f1-score	precision	recall	support
AFIB	0.833668	0.844803	0.823057	204.0
GSVT	0.848395	0.843227	0.853757	300.0
SB	0.868846	0.868076	0.869624	892.0
SR	0.862426	0.862557	0.862301	681.0
macro avg	0.853334	0.854666	0.852185	2077.0
micro avg	0.860357	0.860357	0.860357	2077.0
weighted avg	0.860332	0.860391	0.860357	2077.0

The result of Model Number:Dataset Number:Featuregroup Number:Rescale of 67 : 1 : 3 : 1

	f1-score	precision	recall	support
AFIB	0.725102	0.725749	0.724531	875.0

GSVT	0.738384	0.740321	0.736581	691.0
SB	0.783804	0.778586	0.789101	1553.0
SR	0.768533	0.775549	0.761669	907.0
macro avg	0.753956	0.755051	0.752971	4026.0
micro avg	0.759873	0.759873	0.759873	4026.0
weighted avg	0.759810	0.759851	0.759873	4026.0

The result of Model Number:Dataset Number:Featuregroup Number:Rescale of 67 : 1 : 4 : 0

	f1-score	precision	recall	support
AFIB	0.855765	0.860006	0.851657	875.0
GSVT	0.864285	0.865531	0.863175	691.0
SB	0.909777	0.905556	0.914047	1553.0
SR	0.893029	0.895266	0.890853	907.0
macro avg	0.880714	0.881589	0.879933	4026.0
micro avg	0.886531	0.886531	0.886531	4026.0
weighted avg	0.886457	0.886468	0.886531	4026.0

The result of Model Number:Dataset Number:Featuregroup Number:Rescale of 67 : 1 : 4 : 1

	f1-score	precision	recall	support
AFIB	0.698267	0.712913	0.684229	204.0
GSVT	0.714465	0.710238	0.718750	300.0
SB	0.731883	0.730169	0.733606	892.0
SR	0.725701	0.725493	0.725917	681.0
macro avg	0.717579	0.719703	0.715626	2077.0
micro avg	0.724089	0.724089	0.724089	2077.0
weighted avg	0.724039	0.724062	0.724089	2077.0

The result of Model Number:Dataset Number:Featuregroup Number:Rescale of 67 : 1 : 5 : 0

	f1-score	precision	recall	support
AFIB	0.676034	0.679384	0.672789	875.0
GSVT	0.682765	0.683748	0.681887	691.0
SB	0.718702	0.715367	0.722076	1553.0
SR	0.705472	0.707238	0.703753	907.0
macro avg	0.695743	0.696435	0.695126	4026.0
micro avg	0.700338	0.700338	0.700338	4026.0
weighted avg	0.700280	0.700289	0.700338	4026.0

The result of Model Number:Dataset Number:Featuregroup Number:Rescale of 67 : 1 : 5 : 1

	f1-score	precision	recall	support
AFIB	0.742284	0.770550	0.716181	204.0
GSVT	0.765933	0.758214	0.773846	300.0
SB	0.781264	0.777375	0.785204	892.0
SR	0.771827	0.772313	0.771367	681.0
macro avg	0.765327	0.769613	0.761649	2077.0

micro avg   0.772247   0.772247 0.772247   2077.0
weighted avg 0.772127   0.772277 0.772247   2077.0
The result of Model Number:Dataset Number:Featuregroup Number:Rescale of 67 : 1 : 6 : 0

	f1-score	precision	recall	support
AFIB	0.583707	0.635858	0.616408	204.0
GSVT	0.792837	0.779876	0.806509	300.0
SB	0.674949	0.669808	0.680220	892.0
SR	0.825352	0.816014	0.834932	681.0
macro avg	0.719211	0.725389	0.734517	2077.0
micro avg	0.742920	0.742920	0.742920	2077.0
weighted avg	0.732329	0.730309	0.742920	2077.0

The result of Model Number:Dataset Number:Featuregroup Number:Rescale of 67 : 1 : 6 : 1

	f1-score	precision	recall	support
AFIB	0.788545	0.784130	0.793048	875.0
GSVT	0.794966	0.799657	0.790380	691.0
SB	0.837432	0.834811	0.840072	1553.0
SR	0.820853	0.826283	0.815512	907.0
macro avg	0.810449	0.811220	0.809753	4026.0
micro avg	0.815790	0.815790	0.815790	4026.0
weighted avg	0.815783	0.815841	0.815790	4026.0

The result of Model Number:Dataset Number:Featuregroup Number:Rescale of 67 : 1 : 7 : 0

	f1-score	precision	recall	support
AFIB	0.656471	0.732169	0.595045	204.0
GSVT	0.774046	0.760574	0.788170	300.0
SB	0.826443	0.822779	0.830147	892.0
SR	0.807264	0.796226	0.818636	681.0
macro avg	0.766056	0.777937	0.758000	2077.0
micro avg	0.797218	0.797218	0.797218	2077.0
weighted avg	0.795892	0.796189	0.797218	2077.0

The result of Model Number:Dataset Number:Featuregroup Number:Rescale of 67 : 1 : 7 : 1

	f1-score	precision	recall	support
AFIB	0.746043	0.740487	0.751758	875.0
GSVT	0.759957	0.762671	0.757308	691.0
SB	0.806408	0.804497	0.808329	1553.0
SR	0.788005	0.795099	0.781064	907.0
macro avg	0.775103	0.775689	0.774615	4026.0
micro avg	0.781135	0.781135	0.781135	4026.0
weighted avg	0.781170	0.781289	0.781135	4026.0

The result of Model Number:Dataset Number:Featuregroup Number:Rescale of 67 : 1 : 8 : 0

	f1-score	precision	recall	support
AFIB	0.817006	0.833974	0.800902	875.0
GSVT	0.796743	0.769071	0.861780	691.0
SB	0.939086	0.934852	0.943375	1553.0
SR	0.713938	0.702411	0.725913	907.0
macro avg	0.816693	0.810077	0.832993	4026.0
micro avg	0.849415	0.849415	0.849415	4026.0
weighted avg	0.837400	0.832108	0.849415	4026.0

The result of Model Number:Dataset Number:Featuregroup Number:Rescale of 67 : 1 : 8 : 1

	f1-score	precision	recall	support
AFIB	0.653490	0.640471	0.667081	875.0
GSVT	0.531883	0.494623	0.666700	691.0
SB	0.431564	0.432308	0.430823	1553.0
SR	0.699621	0.705781	0.693596	907.0
macro avg	0.579139	0.568296	0.614550	4026.0
micro avg	0.581854	0.581854	0.581854	4026.0
weighted avg	0.557404	0.549855	0.581854	4026.0

The result of Model Number:Dataset Number:Featuregroup Number:Rescale of 67 : 1 : 9 : 0

	f1-score	precision	recall	support
AFIB	0.748687	0.767968	0.730381	204.0
GSVT	0.687051	0.656468	0.775337	300.0
SB	0.788246	0.786402	0.790101	892.0
SR	0.625331	0.625448	0.625221	681.0
macro avg	0.712329	0.709072	0.730260	2077.0
micro avg	0.728042	0.728042	0.728042	2077.0
weighted avg	0.716328	0.713051	0.728042	2077.0

The result of Model Number:Dataset Number:Featuregroup Number:Rescale of 67 : 1 : 9 : 1

	f1-score	precision	recall	support
AFIB	0.856614	0.832855	0.884464	875.0
GSVT	0.867796	0.904349	0.839423	691.0
SB	0.953304	0.952823	0.953799	1553.0
SR	0.929389	0.935205	0.923651	907.0
macro avg	0.901776	0.906308	0.900334	4026.0
micro avg	0.912307	0.912307	0.912307	4026.0
weighted avg	0.912226	0.914460	0.912307	4026.0

The result of Model Number:Dataset Number:Featuregroup Number:Rescale of 67 : 1 : 10 : 0

	f1-score	precision	recall	support
AFIB	0.669116	0.670169	0.668152	204.0
GSVT	0.685298	0.684229	0.686428	300.0
SB	0.699435	0.698582	0.700296	892.0

SR 0.694537 0.695870 0.693212 681.0
macro avg 0.687096 0.687212 0.687022 2077.0
micro avg 0.692813 0.692813 0.692813 2077.0
weighted avg 0.692809 0.692829 0.692813 2077.0
The result of Model Number:Dataset Number:Featuregroup Number:Rescale of 67 : 1 : 10 : 1

	f1-score	precision	recall	support
AFIB	0.726406	0.744495	0.709211	875.0
GSVT	0.767501	0.770759	0.764302	691.0
SB	0.832494	0.829308	0.835709	1553.0
SR	0.791885	0.777128	0.807254	907.0
macro avg	0.779571	0.780423	0.779119	4026.0
micro avg	0.789550	0.789550	0.789550	4026.0
weighted avg	0.789133	0.789071	0.789550	4026.0

The result of Model Number:Dataset Number:Featuregroup Number:Rescale of 68 : 0 : 0 : 0

	f1-score	precision	recall	support
AFIB	0.890515	0.903598	0.877887	204.0
GSVT	0.913078	0.905315	0.920993	300.0
SB	0.928858	0.925142	0.932607	892.0
SR	0.919672	0.924186	0.915212	681.0
macro avg	0.913031	0.914560	0.911675	2077.0
micro avg	0.919851	0.919851	0.919851	2077.0
weighted avg	0.919801	0.919849	0.919851	2077.0

The result of Model Number:Dataset Number:Featuregroup Number:Rescale of 68 : 0 : 0 : 1

	f1-score	precision	recall	support
AFIB	0.737558	0.772969	0.705397	204.0
GSVT	0.769639	0.763104	0.776308	300.0
SB	0.781734	0.776041	0.787516	892.0
SR	0.773511	0.773869	0.773176	681.0
macro avg	0.765610	0.771496	0.760599	2077.0
micro avg	0.773130	0.773130	0.773130	2077.0
weighted avg	0.772952	0.773159	0.773130	2077.0

The result of Model Number:Dataset Number:Featuregroup Number:Rescale of 68 : 0 : 1 : 0

	f1-score	precision	recall	support
AFIB	0.775086	0.860187	0.705494	204.0
GSVT	0.905018	0.893704	0.916800	300.0
SB	0.962895	0.957981	0.967860	892.0
SR	0.940338	0.927191	0.953867	681.0
macro avg	0.895834	0.909766	0.886005	2077.0
micro avg	0.930128	0.930128	0.930128	2077.0
weighted avg	0.928693	0.928997	0.930128	2077.0

The result of Model Number:Dataset Number:Featuregroup Number:Rescale of 68 : 0 : 1 : 1

	f1-score	precision	recall	support
AFIB	0.653156	0.646863	0.659673	875.0
GSVT	0.670268	0.672186	0.668432	691.0
SB	0.712611	0.710972	0.714262	1553.0
SR	0.692565	0.700714	0.684618	907.0
macro avg	0.682150	0.682684	0.681746	4026.0
micro avg	0.687853	0.687853	0.687853	4026.0
weighted avg	0.687905	0.688071	0.687853	4026.0

The result of Model Number:Dataset Number:Featuregroup Number:Rescale of 68 : 0 : 2 : 0

	f1-score	precision	recall	support
AFIB	0.832807	0.810929	0.884943	875.0
GSVT	0.801152	0.758300	0.913297	691.0
SB	0.759133	0.754336	0.764015	1553.0
SR	0.737378	0.750082	0.725302	907.0
macro avg	0.782617	0.768412	0.821889	4026.0
micro avg	0.807197	0.807197	0.807197	4026.0
weighted avg	0.777456	0.766358	0.807197	4026.0

The result of Model Number:Dataset Number:Featuregroup Number:Rescale of 68 : 0 : 2 : 1

	f1-score	precision	recall	support
AFIB	0.681815	0.688005	0.676085	204.0
GSVT	0.704349	0.700092	0.708842	300.0
SB	0.719085	0.718121	0.720052	892.0
SR	0.712431	0.713909	0.710965	681.0
macro avg	0.704420	0.705032	0.703986	2077.0
micro avg	0.711135	0.711135	0.711135	2077.0
weighted avg	0.711114	0.711178	0.711135	2077.0

The result of Model Number:Dataset Number:Featuregroup Number:Rescale of 68 : 0 : 3 : 0

	f1-score	precision	recall	support
AFIB	0.725621	0.724453	0.727088	875.0
GSVT	0.742539	0.738668	0.746493	691.0
SB	0.776363	0.769786	0.783060	1553.0
SR	0.746726	0.763327	0.731084	907.0
macro avg	0.747812	0.749059	0.746931	4026.0
micro avg	0.752910	0.752910	0.752910	4026.0
weighted avg	0.752853	0.753137	0.752910	4026.0

The result of Model Number:Dataset Number:Featuregroup Number:Rescale of 68 : 0 : 3 : 1

	f1-score	precision	recall	support
AFIB	0.724409	0.724047	0.724802	875.0

GSVT 0.733420 0.738327 0.728636 691.0
SB 0.777525 0.775739 0.779325 1553.0
SR 0.755005 0.754826 0.755315 907.0
macro avg 0.747590 0.748235 0.747020 4026.0
micro avg 0.753366 0.753366 0.753366 4026.0
weighted avg 0.753338 0.753372 0.753366 4026.0
The result of Model Number:Dataset Number:Featuregroup Number:Rescale of 68 : 0 : 4 :
0
f1-score precision recall support
AFIB 0.856359 0.872070 0.841271 204.0
GSVT 0.867654 0.858898 0.876608 300.0
SB 0.889026 0.884399 0.893709 892.0
SR 0.876923 0.882418 0.871515 681.0
macro avg 0.872491 0.874446 0.870776 2077.0
micro avg 0.878812 0.878812 0.878812 2077.0
weighted avg 0.878763 0.878855 0.878812 2077.0
The result of Model Number:Dataset Number:Featuregroup Number:Rescale of 68 : 0 : 4 :
1
f1-score precision recall support
AFIB 0.826181 0.823792 0.828659 875.0
GSVT 0.841493 0.840809 0.842237 691.0
SB 0.893788 0.890086 0.897528 1553.0
SR 0.866955 0.876479 0.857682 907.0
macro avg 0.857104 0.857791 0.856526 4026.0
micro avg 0.864094 0.864094 0.864094 4026.0
weighted avg 0.864074 0.864155 0.864094 4026.0
The result of Model Number:Dataset Number:Featuregroup Number:Rescale of 68 : 0 : 5 :
0
f1-score precision recall support
AFIB 0.781251 0.792311 0.771137 204.0
GSVT 0.797471 0.782788 0.812862 300.0
SB 0.814468 0.805463 0.823691 892.0
SR 0.797352 0.813591 0.781795 681.0
macro avg 0.797636 0.798538 0.797371 2077.0
micro avg 0.803228 0.803228 0.803228 2077.0
weighted avg 0.803139 0.803561 0.803228 2077.0
The result of Model Number:Dataset Number:Featuregroup Number:Rescale of 68 : 0 : 5 :
1
f1-score precision recall support
AFIB 0.784852 0.809026 0.762239 204.0
GSVT 0.804875 0.795022 0.815063 300.0
SB 0.826737 0.821824 0.831736 892.0
SR 0.812801 0.816810 0.808863 681.0
macro avg 0.807316 0.810671 0.804475 2077.0

micro avg	0.815002	0.815002	0.815002	2077.0
weighted avg	0.814896	0.815052	0.815002	2077.0

The result of Model Number:Dataset Number:Featuregroup Number:Rescale of 68 : 0 : 6 : 0

	f1-score	precision	recall	support
AFIB	0.827039	0.809272	0.845648	875.0
GSVT	0.856486	0.868907	0.844472	691.0
SB	0.911763	0.911590	0.911940	1553.0
SR	0.888912	0.899243	0.878819	907.0
macro avg	0.871050	0.872253	0.870220	4026.0
micro avg	0.878491	0.878491	0.878491	4026.0
weighted avg	0.878714	0.879245	0.878491	4026.0

The result of Model Number:Dataset Number:Featuregroup Number:Rescale of 68 : 0 : 6 : 1

	f1-score	precision	recall	support
AFIB	0.920335	0.927217	0.913589	204.0
GSVT	0.943851	0.938870	0.948886	300.0
SB	0.959779	0.958496	0.961069	892.0
SR	0.951326	0.953150	0.949511	681.0
macro avg	0.943823	0.944433	0.943264	2077.0
micro avg	0.950857	0.950857	0.950857	2077.0
weighted avg	0.950833	0.950836	0.950857	2077.0

The result of Model Number:Dataset Number:Featuregroup Number:Rescale of 68 : 0 : 7 : 0

	f1-score	precision	recall	support
AFIB	0.631807	0.696749	0.578327	204.0
GSVT	0.751832	0.741671	0.762349	300.0
SB	0.800647	0.797445	0.803878	892.0
SR	0.778916	0.768387	0.789772	681.0
macro avg	0.740801	0.751063	0.733582	2077.0
micro avg	0.771101	0.771101	0.771101	2077.0
weighted avg	0.769888	0.769972	0.771101	2077.0

The result of Model Number:Dataset Number:Featuregroup Number:Rescale of 68 : 0 : 7 : 1

	f1-score	precision	recall	support
AFIB	0.760969	0.762900	0.759074	875.0
GSVT	0.774397	0.766009	0.783004	691.0
SB	0.816172	0.806940	0.825631	1553.0
SR	0.778855	0.800178	0.758671	907.0
macro avg	0.782598	0.784007	0.781595	4026.0
micro avg	0.788764	0.788764	0.788764	4026.0
weighted avg	0.788598	0.788820	0.788764	4026.0

The result of Model Number:Dataset Number:Featuregroup Number:Rescale of 68 : 0 : 8 : 0

	f1-score	precision	recall	support
AFIB	0.794726	0.815532	0.775318	204.0
GSVT	0.747782	0.769515	0.727650	300.0
SB	0.789070	0.761289	0.818971	892.0
SR	0.715196	0.738487	0.693472	681.0
macro avg	0.761693	0.771206	0.753853	2077.0
micro avg	0.760345	0.760345	0.760345	2077.0
weighted avg	0.759440	0.760329	0.760345	2077.0

The result of Model Number:Dataset Number:Featuregroup Number:Rescale of 68 : 0 : 8 : 1

	f1-score	precision	recall	support
AFIB	0.760543	0.777486	0.744542	204.0
GSVT	0.769926	0.758650	0.781864	300.0
SB	0.787325	0.779500	0.795342	892.0
SR	0.772802	0.783901	0.762035	681.0
macro avg	0.772649	0.774884	0.770946	2077.0
micro avg	0.777485	0.777485	0.777485	2077.0
weighted avg	0.777420	0.777734	0.777485	2077.0

The result of Model Number:Dataset Number:Featuregroup Number:Rescale of 68 : 0 : 9 : 0

	f1-score	precision	recall	support
AFIB	0.740308	0.718182	0.789361	875.0
GSVT	0.633965	0.620082	0.648718	691.0
SB	0.840879	0.834027	0.848067	1553.0
SR	0.788865	0.821986	0.759791	907.0
macro avg	0.751004	0.748569	0.761484	4026.0
micro avg	0.781206	0.781206	0.781206	4026.0
weighted avg	0.771790	0.769416	0.781206	4026.0

The result of Model Number:Dataset Number:Featuregroup Number:Rescale of 68 : 0 : 9 : 1

	f1-score	precision	recall	support
AFIB	0.707026	0.708648	0.705488	875.0
GSVT	0.709490	0.707490	0.711526	691.0
SB	0.744599	0.738433	0.750879	1553.0
SR	0.722261	0.733101	0.711768	907.0
macro avg	0.720844	0.721918	0.719915	4026.0
micro avg	0.725448	0.725448	0.725448	4026.0
weighted avg	0.725375	0.725447	0.725448	4026.0

The result of Model Number:Dataset Number:Featuregroup Number:Rescale of 68 : 0 : 10 : 0

	f1-score	precision	recall	support
AFIB	0.844298	0.870302	0.819972	204.0
GSVT	0.865837	0.855238	0.876798	300.0
SB	0.889355	0.884071	0.894733	892.0

SR 0.874364 0.878677 0.870128 681.0
macro avg 0.868464 0.872072 0.865408 2077.0
micro avg 0.876732 0.876732 0.876732 2077.0
weighted avg 0.876618 0.876785 0.876732 2077.0
The result of Model Number:Dataset Number:Featuregroup Number:Rescale of 68 : 0 : 10 : 1

	f1-score	precision	recall	support
AFIB	0.806042	0.813481	0.798782	875.0
GSVT	0.824864	0.822637	0.827131	691.0
SB	0.878491	0.870234	0.886916	1553.0
SR	0.854219	0.862790	0.845833	907.0
macro avg	0.840904	0.842285	0.839666	4026.0
micro avg	0.848245	0.848245	0.848245	4026.0
weighted avg	0.848073	0.848053	0.848245	4026.0

The result of Model Number:Dataset Number:Featuregroup Number:Rescale of 68 : 1 : 0 : 0

	f1-score	precision	recall	support
AFIB	0.683153	0.695707	0.671127	204.0
GSVT	0.694628	0.690079	0.699256	300.0
SB	0.706510	0.703060	0.709995	892.0
SR	0.699207	0.702114	0.696334	681.0
macro avg	0.695874	0.697740	0.694178	2077.0
micro avg	0.700147	0.700147	0.700147	2077.0
weighted avg	0.700105	0.700153	0.700147	2077.0

The result of Model Number:Dataset Number:Featuregroup Number:Rescale of 68 : 1 : 0 : 1

	f1-score	precision	recall	support
AFIB	0.906057	0.922708	0.890108	204.0
GSVT	0.921276	0.915243	0.927414	300.0
SB	0.937035	0.932460	0.941657	892.0
SR	0.927350	0.931206	0.923540	681.0
macro avg	0.922930	0.925404	0.920680	2077.0
micro avg	0.928596	0.928596	0.928596	2077.0
weighted avg	0.928541	0.928604	0.928596	2077.0

The result of Model Number:Dataset Number:Featuregroup Number:Rescale of 68 : 1 : 1 : 0

	f1-score	precision	recall	support
AFIB	0.667590	0.690937	0.646078	204.0
GSVT	0.695784	0.691542	0.700172	300.0
SB	0.710071	0.707154	0.713016	892.0
SR	0.703507	0.702481	0.704540	681.0
macro avg	0.694238	0.698029	0.690952	2077.0
micro avg	0.701807	0.701807	0.701807	2077.0
weighted avg	0.701683	0.701774	0.701807	2077.0

The result of Model Number:Dataset Number:Featuregroup Number:Rescale of 68 : 1 : 1 : 1

	f1-score	precision	recall	support
AFIB	0.692114	0.731487	0.657223	204.0
GSVT	0.731409	0.723354	0.739681	300.0
SB	0.742289	0.734903	0.749850	892.0
SR	0.733665	0.736174	0.731290	681.0
macro avg	0.724869	0.731479	0.719511	2077.0
micro avg	0.733198	0.733198	0.733198	2077.0
weighted avg	0.732962	0.733316	0.733198	2077.0

The result of Model Number:Dataset Number:Featuregroup Number:Rescale of 68 : 1 : 2 : 0

	f1-score	precision	recall	support
AFIB	0.637104	0.710570	0.577491	204.0
GSVT	0.751211	0.738136	0.764918	300.0
SB	0.802062	0.798506	0.805657	892.0
SR	0.783449	0.772736	0.794486	681.0
macro avg	0.743456	0.754987	0.735638	2077.0
micro avg	0.773700	0.773700	0.773700	2077.0
weighted avg	0.772412	0.772700	0.773700	2077.0

The result of Model Number:Dataset Number:Featuregroup Number:Rescale of 68 : 1 : 2 : 1

	f1-score	precision	recall	support
AFIB	0.903565	0.904988	0.902264	204.0
GSVT	0.925418	0.923974	0.926944	300.0
SB	0.944508	0.943356	0.945670	892.0
SR	0.937894	0.939694	0.936105	681.0
macro avg	0.927846	0.928003	0.927746	2077.0
micro avg	0.935566	0.935566	0.935566	2077.0
weighted avg	0.935561	0.935587	0.935566	2077.0

The result of Model Number:Dataset Number:Featuregroup Number:Rescale of 68 : 1 : 3 : 0

	f1-score	precision	recall	support
AFIB	0.881985	0.873113	0.891063	875.0
GSVT	0.889878	0.897801	0.882159	691.0
SB	0.941451	0.938562	0.944360	1553.0
SR	0.923220	0.931301	0.915281	907.0
macro avg	0.909133	0.910194	0.908216	4026.0
micro avg	0.915550	0.915550	0.915550	4026.0
weighted avg	0.915568	0.915706	0.915550	4026.0

The result of Model Number:Dataset Number:Featuregroup Number:Rescale of 68 : 1 : 3 : 1

	f1-score	precision	recall	support
AFIB	0.711176	0.718757	0.703870	204.0

GSVT	0.724255	0.720282	0.728352	300.0
SB	0.740973	0.739738	0.742217	892.0
SR	0.734796	0.735993	0.733613	681.0
macro avg	0.727800	0.728693	0.727013	2077.0
micro avg	0.733627	0.733627	0.733627	2077.0
weighted avg	0.733607	0.733639	0.733627	2077.0

The result of Model Number:Dataset Number:Featuregroup Number:Rescale of 68 : 1 : 4 : 0

	f1-score	precision	recall	support
AFIB	0.893681	0.910267	0.877911	204.0
GSVT	0.913332	0.908543	0.918191	300.0
SB	0.927914	0.924615	0.931241	892.0
SR	0.918360	0.920016	0.916751	681.0
macro avg	0.913322	0.915860	0.911023	2077.0
micro avg	0.919367	0.919367	0.919367	2077.0
weighted avg	0.919313	0.919376	0.919367	2077.0

The result of Model Number:Dataset Number:Featuregroup Number:Rescale of 68 : 1 : 4 : 1

	f1-score	precision	recall	support
AFIB	0.808590	0.798805	0.818654	875.0
GSVT	0.819536	0.827938	0.811366	691.0
SB	0.870430	0.867597	0.873288	1553.0
SR	0.851391	0.860139	0.842849	907.0
macro avg	0.837487	0.838620	0.836539	4026.0
micro avg	0.843928	0.843928	0.843928	4026.0
weighted avg	0.843966	0.844159	0.843928	4026.0

The result of Model Number:Dataset Number:Featuregroup Number:Rescale of 68 : 1 : 5 : 0

	f1-score	precision	recall	support
AFIB	0.763563	0.752721	0.774807	875.0
GSVT	0.791254	0.801348	0.781530	691.0
SB	0.840885	0.839651	0.842130	1553.0
SR	0.819936	0.826086	0.813879	907.0
macro avg	0.803909	0.804952	0.803087	4026.0
micro avg	0.810733	0.810733	0.810733	4026.0
weighted avg	0.810842	0.811128	0.810733	4026.0

The result of Model Number:Dataset Number:Featuregroup Number:Rescale of 68 : 1 : 5 : 1

	f1-score	precision	recall	support
AFIB	0.844611	0.865161	0.825222	204.0
GSVT	0.855228	0.843455	0.867449	300.0
SB	0.872497	0.863784	0.881394	892.0
SR	0.857263	0.868513	0.846326	681.0
macro avg	0.857400	0.860228	0.855098	2077.0

micro avg 0.862364 0.862364 0.862364 2077.0
weighted avg 0.862269 0.862533 0.862364 2077.0
The result of Model Number:Dataset Number:Featuregroup Number:Rescale of 68 : 1 : 6 : 0

	f1-score	precision	recall	support
AFIB	0.810035	0.803839	0.816435	875.0
GSVT	0.828673	0.833205	0.824240	691.0
SB	0.877498	0.876884	0.878120	1553.0
SR	0.854190	0.858334	0.850131	907.0
macro avg	0.842599	0.843065	0.842231	4026.0
micro avg	0.849160	0.849160	0.849160	4026.0
weighted avg	0.849205	0.849333	0.849160	4026.0

The result of Model Number:Dataset Number:Featuregroup Number:Rescale of 68 : 1 : 6 : 1

	f1-score	precision	recall	support
AFIB	0.680793	0.683339	0.678555	875.0
GSVT	0.696117	0.702833	0.689668	691.0
SB	0.740190	0.732427	0.748177	1553.0
SR	0.714558	0.720847	0.708624	907.0
macro avg	0.707914	0.709861	0.706256	4026.0
micro avg	0.714093	0.714093	0.714093	4026.0
weighted avg	0.713942	0.714070	0.714093	4026.0

The result of Model Number:Dataset Number:Featuregroup Number:Rescale of 68 : 1 : 7 : 0

	f1-score	precision	recall	support
AFIB	0.700898	0.732548	0.672099	204.0
GSVT	0.725276	0.717131	0.733741	300.0
SB	0.697689	0.667271	0.746537	892.0
SR	0.581271	0.586227	0.576496	681.0
macro avg	0.676283	0.675794	0.682218	2077.0
micro avg	0.681625	0.681625	0.681625	2077.0
weighted avg	0.663818	0.654312	0.681625	2077.0

The result of Model Number:Dataset Number:Featuregroup Number:Rescale of 68 : 1 : 7 : 1

	f1-score	precision	recall	support
AFIB	0.720696	0.729953	0.711708	875.0
GSVT	0.728669	0.722777	0.734776	691.0
SB	0.779468	0.777124	0.781828	1553.0
SR	0.760167	0.759847	0.760496	907.0
macro avg	0.747250	0.747425	0.747202	4026.0
micro avg	0.753707	0.753707	0.753707	4026.0
weighted avg	0.753628	0.753652	0.753707	4026.0

The result of Model Number:Dataset Number:Featuregroup Number:Rescale of 68 : 1 : 8 : 0

	f1-score	precision	recall	support
AFIB	0.866716	0.916021	0.823023	204.0
GSVT	0.915924	0.905837	0.926283	300.0
SB	0.929548	0.920299	0.939017	892.0
SR	0.918750	0.921891	0.915775	681.0
macro avg	0.907735	0.916012	0.901025	2077.0
micro avg	0.918165	0.918165	0.918165	2077.0
weighted avg	0.917869	0.918312	0.918165	2077.0

The result of Model Number:Dataset Number:Featuregroup Number:Rescale of 68 : 1 : 8 : 1

	f1-score	precision	recall	support
AFIB	0.661291	0.660009	0.662664	875.0
GSVT	0.673708	0.661921	0.685988	691.0
SB	0.721732	0.711492	0.732279	1553.0
SR	0.683671	0.714148	0.655718	907.0
macro avg	0.685101	0.686893	0.684162	4026.0
micro avg	0.691956	0.691956	0.691956	4026.0
weighted avg	0.691779	0.692393	0.691956	4026.0

The result of Model Number:Dataset Number:Featuregroup Number:Rescale of 68 : 1 : 9 : 0

	f1-score	precision	recall	support
AFIB	0.829320	0.869136	0.793158	204.0
GSVT	0.865393	0.858044	0.872892	300.0
SB	0.878992	0.872591	0.885494	892.0
SR	0.869746	0.870149	0.869369	681.0
macro avg	0.860863	0.867480	0.855228	2077.0
micro avg	0.869318	0.869318	0.869318	2077.0
weighted avg	0.869118	0.869350	0.869318	2077.0

The result of Model Number:Dataset Number:Featuregroup Number:Rescale of 68 : 1 : 9 : 1

	f1-score	precision	recall	support
AFIB	0.629175	0.686158	0.581980	204.0
GSVT	0.730273	0.721928	0.739038	300.0
SB	0.774411	0.772429	0.776408	892.0
SR	0.755451	0.745221	0.765987	681.0
macro avg	0.722328	0.731434	0.715853	2077.0
micro avg	0.748497	0.748497	0.748497	2077.0
weighted avg	0.747554	0.747740	0.748497	2077.0

The result of Model Number:Dataset Number:Featuregroup Number:Rescale of 68 : 1 : 10 : 0

	f1-score	precision	recall	support
AFIB	0.628021	0.624067	0.707423	204.0
GSVT	0.765569	0.761631	0.769676	300.0
SB	0.787366	0.783620	0.791158	892.0

SR 0.617600 0.619635 0.615951 681.0
macro avg 0.699639 0.697238 0.721052 2077.0
micro avg 0.722384 0.722384 0.722384 2077.0
weighted avg 0.712905 0.711006 0.722384 2077.0
The result of Model Number:Dataset Number:Featuregroup Number:Rescale of 68 : 1 : 10 : 1

	f1-score	precision	recall	support
AFIB	0.898648	0.918480	0.879721	204.0
GSVT	0.918288	0.903060	0.934148	300.0
SB	0.935933	0.928612	0.943380	892.0
SR	0.913219	0.924211	0.902488	681.0
macro avg	0.916522	0.918591	0.914934	2077.0
micro avg	0.922387	0.922387	0.922387	2077.0
weighted avg	0.922275	0.922483	0.922387	2077.0

The result of Model Number:Dataset Number:Featuregroup Number:Rescale of 69 : 0 : 0 : 0

	f1-score	precision	recall	support
AFIB	0.685397	0.699206	0.672563	204.0
GSVT	0.707390	0.701708	0.713300	300.0
SB	0.722704	0.721976	0.723433	892.0
SR	0.716116	0.715705	0.716536	681.0
macro avg	0.707902	0.709649	0.706458	2077.0
micro avg	0.714712	0.714712	0.714712	2077.0
weighted avg	0.714668	0.714756	0.714712	2077.0

The result of Model Number:Dataset Number:Featuregroup Number:Rescale of 69 : 0 : 0 : 1

	f1-score	precision	recall	support
AFIB	0.662609	0.663201	0.662088	875.0
GSVT	0.674747	0.676517	0.673099	691.0
SB	0.716253	0.711484	0.721093	1553.0
SR	0.702297	0.708709	0.696026	907.0
macro avg	0.688977	0.689978	0.688076	4026.0
micro avg	0.694384	0.694384	0.694384	4026.0
weighted avg	0.694326	0.694363	0.694384	4026.0

The result of Model Number:Dataset Number:Featuregroup Number:Rescale of 69 : 0 : 1 : 0

	f1-score	precision	recall	support
AFIB	0.687453	0.693638	0.681592	875.0
GSVT	0.701456	0.687233	0.716446	691.0
SB	0.743899	0.735623	0.752453	1553.0
SR	0.714380	0.735616	0.694505	907.0
macro avg	0.711797	0.713028	0.711249	4026.0
micro avg	0.717817	0.717817	0.717817	4026.0
weighted avg	0.717696	0.718191	0.717817	4026.0

The result of Model Number:Dataset Number:Featuregroup Number:Rescale of 69 : 0 : 1 : 1

	f1-score	precision	recall	support
AFIB	0.721085	0.670555	0.846778	875.0
GSVT	0.618424	0.577385	0.699499	691.0
SB	0.738976	0.732742	0.745335	1553.0
SR	0.712266	0.729531	0.696048	907.0
macro avg	0.697688	0.677553	0.746915	4026.0
micro avg	0.748412	0.748412	0.748412	4026.0
weighted avg	0.708380	0.691838	0.748412	4026.0

The result of Model Number:Dataset Number:Featuregroup Number:Rescale of 69 : 0 : 2 : 0

	f1-score	precision	recall	support
AFIB	0.826963	0.829808	0.824242	875.0
GSVT	0.842663	0.825851	0.860326	691.0
SB	0.892026	0.887202	0.896908	1553.0
SR	0.857524	0.877462	0.838523	907.0
macro avg	0.854794	0.855081	0.854999	4026.0
micro avg	0.861683	0.861683	0.861683	4026.0
weighted avg	0.861640	0.862004	0.861683	4026.0

The result of Model Number:Dataset Number:Featuregroup Number:Rescale of 69 : 0 : 2 : 1

	f1-score	precision	recall	support
AFIB	0.707414	0.729281	0.687437	204.0
GSVT	0.660362	0.674718	0.647091	300.0
SB	0.696515	0.665602	0.730473	892.0
SR	0.627135	0.659361	0.598001	681.0
macro avg	0.672857	0.682241	0.665751	2077.0
micro avg	0.670768	0.670768	0.670768	2077.0
weighted avg	0.669615	0.671127	0.670768	2077.0

The result of Model Number:Dataset Number:Featuregroup Number:Rescale of 69 : 0 : 3 : 0

	f1-score	precision	recall	support
AFIB	0.761500	0.772279	0.751641	204.0
GSVT	0.777309	0.762998	0.792311	300.0
SB	0.793877	0.785099	0.802866	892.0
SR	0.777193	0.793022	0.762030	681.0
macro avg	0.777470	0.778350	0.777212	2077.0
micro avg	0.782921	0.782921	0.782921	2077.0
weighted avg	0.782834	0.783246	0.782921	2077.0

The result of Model Number:Dataset Number:Featuregroup Number:Rescale of 69 : 0 : 3 : 1

	f1-score	precision	recall	support
AFIB	0.834332	0.844684	0.824510	204.0

GSVT 0.849292 0.843489 0.855264 300.0
SB 0.869404 0.868046 0.870767 892.0
SR 0.863159 0.864559 0.861771 681.0
macro avg 0.854047 0.855195 0.853078 2077.0
micro avg 0.861035 0.861035 0.861035 2077.0
weighted avg 0.861007 0.861061 0.861035 2077.0
The result of Model Number:Dataset Number:Featuregroup Number:Rescale of 69 : 0 : 4 : 0

	f1-score	precision	recall	support
AFIB	0.845848	0.846992	0.844869	875.0
GSVT	0.859022	0.858581	0.859497	691.0
SB	0.897160	0.893950	0.900396	1553.0
SR	0.875676	0.880584	0.870878	907.0
macro avg	0.869427	0.870027	0.868910	4026.0
micro avg	0.874658	0.874658	0.874658	4026.0
weighted avg	0.874622	0.874663	0.874658	4026.0

The result of Model Number:Dataset Number:Featuregroup Number:Rescale of 69 : 0 : 4 : 1

	f1-score	precision	recall	support
AFIB	0.681408	0.696936	0.666665	875.0
GSVT	0.725105	0.726538	0.723920	691.0
SB	0.793313	0.788971	0.797706	1553.0
SR	0.749800	0.740705	0.759226	907.0
macro avg	0.737406	0.738288	0.736879	4026.0
micro avg	0.747893	0.747893	0.747893	4026.0
weighted avg	0.747482	0.747379	0.747893	4026.0

The result of Model Number:Dataset Number:Featuregroup Number:Rescale of 69 : 0 : 5 : 0

	f1-score	precision	recall	support
AFIB	0.752451	0.839217	0.682045	204.0
GSVT	0.887217	0.871774	0.903405	300.0
SB	0.947273	0.943075	0.951520	892.0
SR	0.925291	0.912639	0.938326	681.0
macro avg	0.878058	0.891676	0.868824	2077.0
micro avg	0.913777	0.913777	0.913777	2077.0
weighted avg	0.912256	0.912596	0.913777	2077.0

The result of Model Number:Dataset Number:Featuregroup Number:Rescale of 69 : 0 : 5 : 1

	f1-score	precision	recall	support
AFIB	0.804091	0.801618	0.806630	875.0
GSVT	0.783593	0.787436	0.779913	691.0
SB	0.823776	0.789591	0.861064	1553.0
SR	0.708928	0.773480	0.654376	907.0
macro avg	0.780097	0.788031	0.775496	4026.0

micro avg 0.788741 0.788741 0.788741 4026.0
weighted avg 0.786728 0.788206 0.788741 4026.0
The result of Model Number:Dataset Number:Featuregroup Number:Rescale of 69 : 0 : 6 : 0

	f1-score	precision	recall	support
AFIB	0.808591	0.810705	0.806645	204.0
GSVT	0.826834	0.823633	0.830129	300.0
SB	0.845378	0.844250	0.846513	892.0
SR	0.838607	0.840963	0.836267	681.0
macro avg	0.829852	0.829888	0.829889	2077.0
micro avg	0.836872	0.836872	0.836872	2077.0
weighted avg	0.836866	0.836900	0.836872	2077.0

The result of Model Number:Dataset Number:Featuregroup Number:Rescale of 69 : 0 : 6 : 1

	f1-score	precision	recall	support
AFIB	0.907540	0.942099	0.875625	204.0
GSVT	0.936454	0.927016	0.946128	300.0
SB	0.955198	0.950443	0.960014	892.0
SR	0.943660	0.944254	0.943098	681.0
macro avg	0.935713	0.940953	0.931216	2077.0
micro avg	0.944173	0.944173	0.944173	2077.0
weighted avg	0.944027	0.944210	0.944173	2077.0

The result of Model Number:Dataset Number:Featuregroup Number:Rescale of 69 : 0 : 7 : 0

	f1-score	precision	recall	support
AFIB	0.878476	0.883734	0.873325	204.0
GSVT	0.901877	0.899197	0.904581	300.0
SB	0.915688	0.914566	0.916816	892.0
SR	0.909013	0.910085	0.907950	681.0
macro avg	0.901263	0.901895	0.900668	2077.0
micro avg	0.907870	0.907870	0.907870	2077.0
weighted avg	0.907850	0.907849	0.907870	2077.0

The result of Model Number:Dataset Number:Featuregroup Number:Rescale of 69 : 0 : 7 : 1

	f1-score	precision	recall	support
AFIB	0.696724	0.687074	0.706678	875.0
GSVT	0.712177	0.717941	0.706509	691.0
SB	0.755968	0.753795	0.758157	1553.0
SR	0.737577	0.747177	0.728228	907.0
macro avg	0.725612	0.726497	0.724893	4026.0
micro avg	0.731362	0.731362	0.731362	4026.0
weighted avg	0.731433	0.731650	0.731362	4026.0

The result of Model Number:Dataset Number:Featuregroup Number:Rescale of 69 : 0 : 8 : 0

	f1-score	precision	recall	support
AFIB	0.686094	0.716204	0.658586	204.0
GSVT	0.638709	0.643307	0.634438	300.0
SB	0.662391	0.629128	0.699371	892.0
SR	0.587645	0.626444	0.553496	681.0
macro avg	0.643710	0.653771	0.636473	2077.0
micro avg	0.638157	0.638157	0.638157	2077.0
weighted avg	0.636791	0.638849	0.638157	2077.0

The result of Model Number:Dataset Number:Featuregroup Number:Rescale of 69 : 0 : 8 : 1

	f1-score	precision	recall	support
AFIB	0.876703	0.869953	0.883626	875.0
GSVT	0.888085	0.892209	0.884194	691.0
SB	0.951707	0.946644	0.956845	1553.0
SR	0.923667	0.936653	0.911138	907.0
macro avg	0.910041	0.911365	0.908951	4026.0
micro avg	0.918165	0.918165	0.918165	4026.0
weighted avg	0.918169	0.918383	0.918165	4026.0

The result of Model Number:Dataset Number:Featuregroup Number:Rescale of 69 : 0 : 9 : 0

	f1-score	precision	recall	support
AFIB	0.812931	0.814387	0.811506	875.0
GSVT	0.778436	0.782525	0.774411	691.0
SB	0.823397	0.778102	0.874298	1553.0
SR	0.683835	0.769706	0.615261	907.0
macro avg	0.774650	0.786180	0.768869	4026.0
micro avg	0.785150	0.785150	0.785150	4026.0
weighted avg	0.781964	0.784856	0.785150	4026.0

The result of Model Number:Dataset Number:Featuregroup Number:Rescale of 69 : 0 : 9 : 1

	f1-score	precision	recall	support
AFIB	0.577604	0.639995	0.526403	204.0
GSVT	0.669567	0.658677	0.680928	300.0
SB	0.714052	0.710801	0.717333	892.0
SR	0.696798	0.687931	0.705907	681.0
macro avg	0.664505	0.674351	0.657643	2077.0
micro avg	0.689576	0.689576	0.689576	2077.0
weighted avg	0.688568	0.688819	0.689576	2077.0

The result of Model Number:Dataset Number:Featuregroup Number:Rescale of 69 : 0 : 10 : 0

	f1-score	precision	recall	support
AFIB	0.783772	0.797874	0.770391	204.0
GSVT	0.801454	0.790185	0.813182	300.0
SB	0.813786	0.804171	0.823637	892.0

SR 0.797419 0.811616 0.783719 681.0
macro avg 0.799108 0.800961 0.797732 2077.0
micro avg 0.803809 0.803809 0.803809 2077.0
weighted avg 0.803690 0.803973 0.803809 2077.0
The result of Model Number:Dataset Number:Featuregroup Number:Rescale of 69 : 0 : 10 : 1

	f1-score	precision	recall	support
AFIB	0.778079	0.793069	0.764013	204.0
GSVT	0.792690	0.779810	0.806146	300.0
SB	0.805924	0.796312	0.815772	892.0
SR	0.790931	0.805760	0.776652	681.0
macro avg	0.791906	0.793738	0.790646	2077.0
micro avg	0.796472	0.796472	0.796472	2077.0
weighted avg	0.796362	0.796708	0.796472	2077.0

The result of Model Number:Dataset Number:Featuregroup Number:Rescale of 69 : 1 : 0 : 0

	f1-score	precision	recall	support
AFIB	0.837601	0.815597	0.890037	875.0
GSVT	0.805764	0.762666	0.918554	691.0
SB	0.763503	0.758678	0.768413	1553.0
SR	0.741623	0.754400	0.729477	907.0
macro avg	0.787123	0.772835	0.826620	4026.0
micro avg	0.811844	0.811844	0.811844	4026.0
weighted avg	0.781931	0.770769	0.811844	4026.0

The result of Model Number:Dataset Number:Featuregroup Number:Rescale of 69 : 1 : 0 : 1

	f1-score	precision	recall	support
AFIB	0.671323	0.664855	0.678021	875.0
GSVT	0.688911	0.690882	0.687024	691.0
SB	0.732431	0.730747	0.734129	1553.0
SR	0.711828	0.720203	0.703660	907.0
macro avg	0.701123	0.701672	0.700708	4026.0
micro avg	0.706985	0.706985	0.706985	4026.0
weighted avg	0.707039	0.707209	0.706985	4026.0

The result of Model Number:Dataset Number:Featuregroup Number:Rescale of 69 : 1 : 1 : 0

	f1-score	precision	recall	support
AFIB	0.780988	0.777077	0.785196	875.0
GSVT	0.798634	0.799714	0.797717	691.0
SB	0.850436	0.847635	0.853278	1553.0
SR	0.825460	0.834028	0.817165	907.0
macro avg	0.813880	0.814614	0.813339	4026.0
micro avg	0.820809	0.820809	0.820809	4026.0
weighted avg	0.820825	0.821010	0.820809	4026.0

The result of Model Number:Dataset Number:Featuregroup Number:Rescale of 69 : 1 : 1 : 1

	f1-score	precision	recall	support
AFIB	0.839920	0.847545	0.832861	204.0
GSVT	0.867679	0.862436	0.873215	300.0
SB	0.885832	0.884645	0.887024	892.0
SR	0.877636	0.879456	0.875830	681.0
macro avg	0.867767	0.868520	0.867232	2077.0
micro avg	0.876039	0.876039	0.876039	2077.0
weighted avg	0.876013	0.876092	0.876039	2077.0

The result of Model Number:Dataset Number:Featuregroup Number:Rescale of 69 : 1 : 2 : 0

	f1-score	precision	recall	support
AFIB	0.832999	0.823281	0.845122	875.0
GSVT	0.857106	0.846717	0.868444	691.0
SB	0.870025	0.836442	0.919285	1553.0
SR	0.690845	0.727286	0.657923	907.0
macro avg	0.812744	0.808431	0.822693	4026.0
micro avg	0.835559	0.835559	0.835559	4026.0
weighted avg	0.819394	0.810754	0.835559	4026.0

The result of Model Number:Dataset Number:Featuregroup Number:Rescale of 69 : 1 : 2 : 1

	f1-score	precision	recall	support
AFIB	0.687096	0.685638	0.688592	875.0
GSVT	0.697830	0.694485	0.701266	691.0
SB	0.734464	0.725696	0.743448	1553.0
SR	0.700332	0.720173	0.681569	907.0
macro avg	0.704930	0.706498	0.703719	4026.0
micro avg	0.710346	0.710346	0.710346	4026.0
weighted avg	0.710192	0.710389	0.710346	4026.0

The result of Model Number:Dataset Number:Featuregroup Number:Rescale of 69 : 1 : 3 : 0

	f1-score	precision	recall	support
AFIB	0.663998	0.648909	0.679852	875.0
GSVT	0.686754	0.697891	0.676023	691.0
SB	0.732691	0.732696	0.732692	1553.0
SR	0.713939	0.722072	0.705988	907.0
macro avg	0.699345	0.700392	0.698639	4026.0
micro avg	0.705465	0.705465	0.705465	4026.0
weighted avg	0.705653	0.706119	0.705465	4026.0

The result of Model Number:Dataset Number:Featuregroup Number:Rescale of 69 : 1 : 3 : 1

	f1-score	precision	recall	support
AFIB	0.737269	0.730749	0.743938	875.0

GSVT 0.670306 0.649140 0.745729 691.0
SB 0.604317 0.580228 0.641988 1553.0
SR 0.622836 0.629281 0.616563 907.0
macro avg 0.658682 0.647350 0.687054 4026.0
micro avg 0.676223 0.676223 0.676223 4026.0
weighted avg 0.648711 0.635820 0.676223 4026.0
The result of Model Number:Dataset Number:Featuregroup Number:Rescale of 69 : 1 : 4 : 0

	f1-score	precision	recall	support
AFIB	0.888564	0.877812	0.899624	875.0
GSVT	0.900592	0.909826	0.891614	691.0
SB	0.956520	0.953408	0.959661	1553.0
SR	0.935599	0.945212	0.926211	907.0
macro avg	0.920319	0.921564	0.919277	4026.0
micro avg	0.927398	0.927398	0.927398	4026.0
weighted avg	0.927438	0.927651	0.927398	4026.0

The result of Model Number:Dataset Number:Featuregroup Number:Rescale of 69 : 1 : 4 : 1

	f1-score	precision	recall	support
AFIB	0.766000	0.854329	0.694326	204.0
GSVT	0.903193	0.887472	0.919672	300.0
SB	0.964331	0.960056	0.968653	892.0
SR	0.941952	0.929073	0.955222	681.0
macro avg	0.893869	0.907732	0.884468	2077.0
micro avg	0.930231	0.930231	0.930231	2077.0
weighted avg	0.928683	0.929029	0.930231	2077.0

The result of Model Number:Dataset Number:Featuregroup Number:Rescale of 69 : 1 : 5 : 0

	f1-score	precision	recall	support
AFIB	0.677481	0.672299	0.682833	875.0
GSVT	0.693069	0.696859	0.689361	691.0
SB	0.733904	0.733390	0.734424	1553.0
SR	0.714410	0.717875	0.711015	907.0
macro avg	0.704716	0.705106	0.704408	4026.0
micro avg	0.710203	0.710203	0.710203	4026.0
weighted avg	0.710241	0.710347	0.710203	4026.0

The result of Model Number:Dataset Number:Featuregroup Number:Rescale of 69 : 1 : 5 : 1

	f1-score	precision	recall	support
AFIB	0.877191	0.899403	0.856131	204.0
GSVT	0.896242	0.886495	0.906353	300.0
SB	0.921485	0.916886	0.926133	892.0
SR	0.907202	0.911273	0.903205	681.0
macro avg	0.900530	0.903514	0.897955	2077.0

micro avg 0.908883 0.908883 0.908883 2077.0
weighted avg 0.908805 0.908939 0.908883 2077.0
The result of Model Number:Dataset Number:Featuregroup Number:Rescale of 69 : 1 : 6 : 0

	f1-score	precision	recall	support
AFIB	0.709597	0.726308	0.693698	875.0
GSVT	0.748020	0.749909	0.746190	691.0
SB	0.813485	0.810113	0.816889	1553.0
SR	0.774036	0.761846	0.786660	907.0
macro avg	0.761285	0.762044	0.760859	4026.0
micro avg	0.771170	0.771170	0.771170	4026.0
weighted avg	0.770783	0.770692	0.771170	4026.0

The result of Model Number:Dataset Number:Featuregroup Number:Rescale of 69 : 1 : 6 : 1

	f1-score	precision	recall	support
AFIB	0.884673	0.873967	0.895684	875.0
GSVT	0.896648	0.905841	0.887709	691.0
SB	0.952331	0.949232	0.955458	1553.0
SR	0.931501	0.941073	0.922155	907.0
macro avg	0.916288	0.917528	0.915251	4026.0
micro avg	0.923336	0.923336	0.923336	4026.0
weighted avg	0.923377	0.923589	0.923336	4026.0

The result of Model Number:Dataset Number:Featuregroup Number:Rescale of 69 : 1 : 7 : 0

	f1-score	precision	recall	support
AFIB	0.651543	0.643362	0.660123	875.0
GSVT	0.604502	0.622805	0.589266	691.0
SB	0.650843	0.602824	0.708895	1553.0
SR	0.506051	0.619368	0.431520	907.0
macro avg	0.603235	0.622090	0.597451	4026.0
micro avg	0.615274	0.615274	0.615274	4026.0
weighted avg	0.610422	0.618791	0.615274	4026.0

The result of Model Number:Dataset Number:Featuregroup Number:Rescale of 69 : 1 : 7 : 1

	f1-score	precision	recall	support
AFIB	0.723160	0.728221	0.718194	204.0
GSVT	0.739985	0.736337	0.743681	300.0
SB	0.754692	0.753436	0.755961	892.0
SR	0.747181	0.748955	0.745424	681.0
macro avg	0.741254	0.741737	0.740815	2077.0
micro avg	0.747023	0.747023	0.747023	2077.0
weighted avg	0.747008	0.747020	0.747023	2077.0

The result of Model Number:Dataset Number:Featuregroup Number:Rescale of 69 : 1 : 8 : 0

	f1-score	precision	recall	support
AFIB	0.845555	0.856515	0.835095	204.0
GSVT	0.878272	0.877704	0.878867	300.0
SB	0.896778	0.894885	0.898686	892.0
SR	0.887759	0.887254	0.888283	681.0
macro avg	0.877091	0.879090	0.875233	2077.0
micro avg	0.886167	0.886167	0.886167	2077.0
weighted avg	0.886117	0.886133	0.886167	2077.0

The result of Model Number:Dataset Number:Featuregroup Number:Rescale of 69 : 1 : 8 : 1

	f1-score	precision	recall	support
AFIB	0.738520	0.813030	0.676952	204.0
GSVT	0.870930	0.858909	0.883432	300.0
SB	0.924759	0.921674	0.927869	892.0
SR	0.901077	0.888611	0.913910	681.0
macro avg	0.858822	0.870556	0.850541	2077.0
micro avg	0.892229	0.892229	0.892229	2077.0
weighted avg	0.890927	0.891097	0.892229	2077.0

The result of Model Number:Dataset Number:Featuregroup Number:Rescale of 69 : 1 : 9 : 0

	f1-score	precision	recall	support
AFIB	0.764270	0.752035	0.859959	204.0
GSVT	0.894759	0.884376	0.905714	300.0
SB	0.924719	0.921227	0.928243	892.0
SR	0.725551	0.729302	0.721961	681.0
macro avg	0.827325	0.821735	0.853969	2077.0
micro avg	0.850647	0.850647	0.850647	2077.0
weighted avg	0.839330	0.836359	0.850647	2077.0

The result of Model Number:Dataset Number:Featuregroup Number:Rescale of 69 : 1 : 9 : 1

	f1-score	precision	recall	support
AFIB	0.872782	0.879681	0.866018	875.0
GSVT	0.894743	0.892852	0.896676	691.0
SB	0.953823	0.945392	0.962417	1553.0
SR	0.928431	0.937537	0.919523	907.0
macro avg	0.912445	0.913865	0.911159	4026.0
micro avg	0.920519	0.920519	0.920519	4026.0
weighted avg	0.920349	0.920323	0.920519	4026.0

The result of Model Number:Dataset Number:Featuregroup Number:Rescale of 69 : 1 : 10 : 0

	f1-score	precision	recall	support
AFIB	0.731072	0.769564	0.696442	204.0
GSVT	0.767782	0.761058	0.774662	300.0
SB	0.787705	0.782842	0.792643	892.0

SR 0.776958 0.775382 0.778559 681.0
macro avg 0.765879 0.772212 0.760576 2077.0
micro avg 0.775979 0.775979 0.775979 2077.0
weighted avg 0.775741 0.775946 0.775979 2077.0
The result of Model Number:Dataset Number:Featuregroup Number:Rescale of 69 : 1 : 10 : 1

	f1-score	precision	recall	support
AFIB	0.800653	0.852821	0.754855	204.0
GSVT	0.835728	0.815186	0.857446	300.0
SB	0.860238	0.849188	0.871610	892.0
SR	0.837894	0.847899	0.828178	681.0
macro avg	0.833628	0.841273	0.828022	2077.0
micro avg	0.843856	0.843856	0.843856	2077.0
weighted avg	0.843520	0.844211	0.843856	2077.0

The result of Model Number:Dataset Number:Featuregroup Number:Rescale of 70 : 0 : 0 : 0

	f1-score	precision	recall	support
AFIB	0.758929	0.765758	0.752459	875.0
GSVT	0.774389	0.758687	0.790937	691.0
SB	0.821244	0.812108	0.830687	1553.0
SR	0.788656	0.812099	0.766714	907.0
macro avg	0.785804	0.787163	0.785199	4026.0
micro avg	0.792450	0.792450	0.792450	4026.0
weighted avg	0.792317	0.792863	0.792450	4026.0

The result of Model Number:Dataset Number:Featuregroup Number:Rescale of 70 : 0 : 0 : 1

	f1-score	precision	recall	support
AFIB	0.751721	0.774688	0.730162	875.0
GSVT	0.797738	0.795036	0.800528	691.0
SB	0.871724	0.868222	0.875258	1553.0
SR	0.825620	0.811299	0.840536	907.0
macro avg	0.811701	0.812311	0.811621	4026.0
micro avg	0.823075	0.823075	0.823075	4026.0
weighted avg	0.822558	0.822508	0.823075	4026.0

The result of Model Number:Dataset Number:Featuregroup Number:Rescale of 70 : 0 : 1 : 0

	f1-score	precision	recall	support
AFIB	0.798281	0.812971	0.784519	204.0
GSVT	0.827733	0.820801	0.834927	300.0
SB	0.843276	0.841863	0.844694	892.0
SR	0.835830	0.836573	0.835094	681.0
macro avg	0.826280	0.828052	0.824808	2077.0
micro avg	0.834225	0.834225	0.834225	2077.0
weighted avg	0.834170	0.834248	0.834225	2077.0

The result of Model Number:Dataset Number:Featuregroup Number:Rescale of 70 : 0 : 1 : 1

	f1-score	precision	recall	support
AFIB	0.875414	0.892895	0.858737	204.0
GSVT	0.885245	0.874184	0.896723	300.0
SB	0.902146	0.893520	0.910941	892.0
SR	0.885763	0.897382	0.874464	681.0
macro avg	0.887142	0.889495	0.885216	2077.0
micro avg	0.891800	0.891800	0.891800	2077.0
weighted avg	0.891707	0.891932	0.891800	2077.0

The result of Model Number:Dataset Number:Featuregroup Number:Rescale of 70 : 0 : 2 : 0

	f1-score	precision	recall	support
AFIB	0.621063	0.636551	0.606356	875.0
GSVT	0.660702	0.663235	0.658219	691.0
SB	0.715485	0.712021	0.718985	1553.0
SR	0.677961	0.666661	0.689695	907.0
macro avg	0.668803	0.669617	0.668314	4026.0
micro avg	0.677478	0.677478	0.677478	4026.0
weighted avg	0.677107	0.677026	0.677478	4026.0

The result of Model Number:Dataset Number:Featuregroup Number:Rescale of 70 : 0 : 2 : 1

	f1-score	precision	recall	support
AFIB	0.654270	0.653002	0.655628	875.0
GSVT	0.666556	0.654894	0.678705	691.0
SB	0.714070	0.703938	0.724505	1553.0
SR	0.676413	0.706566	0.648756	907.0
macro avg	0.677827	0.679600	0.676899	4026.0
micro avg	0.684610	0.684610	0.684610	4026.0
weighted avg	0.684435	0.685042	0.684610	4026.0

The result of Model Number:Dataset Number:Featuregroup Number:Rescale of 70 : 0 : 3 : 0

	f1-score	precision	recall	support
AFIB	0.666139	0.672133	0.660460	875.0
GSVT	0.679708	0.665926	0.694233	691.0
SB	0.720835	0.712816	0.729123	1553.0
SR	0.692231	0.712808	0.672972	907.0
macro avg	0.689728	0.690921	0.689197	4026.0
micro avg	0.695562	0.695562	0.695562	4026.0
weighted avg	0.695445	0.695924	0.695562	4026.0

The result of Model Number:Dataset Number:Featuregroup Number:Rescale of 70 : 0 : 3 : 1

	f1-score	precision	recall	support
AFIB	0.705263	0.753418	0.663195	204.0

GSVT 0.745533 0.730341 0.761436 300.0
SB 0.764025 0.757327 0.770871 892.0
SR 0.745179 0.747661 0.742780 681.0
macro avg 0.740000 0.747187 0.734571 2077.0
micro avg 0.749722 0.749722 0.749722 2077.0
weighted avg 0.749403 0.749876 0.749722 2077.0

The result of Model Number:Dataset Number:Featuregroup Number:Rescale of 70 : 0 : 4 : 0

	f1-score	precision	recall	support
AFIB	0.728992	0.719701	0.738550	875.0
GSVT	0.696054	0.700844	0.691348	691.0
SB	0.736997	0.694408	0.785167	1553.0
SR	0.601046	0.695394	0.529275	907.0
macro avg	0.690772	0.702587	0.686085	4026.0
micro avg	0.701284	0.701284	0.701284	4026.0
weighted avg	0.697602	0.701232	0.701284	4026.0

The result of Model Number:Dataset Number:Featuregroup Number:Rescale of 70 : 0 : 4 : 1

	f1-score	precision	recall	support
AFIB	0.713558	0.722320	0.705118	204.0
GSVT	0.727931	0.724821	0.731083	300.0
SB	0.736742	0.734531	0.738970	892.0
SR	0.730541	0.732280	0.728834	681.0
macro avg	0.727193	0.728488	0.726001	2077.0
micro avg	0.731183	0.731183	0.731183	2077.0
weighted avg	0.731159	0.731191	0.731183	2077.0

The result of Model Number:Dataset Number:Featuregroup Number:Rescale of 70 : 0 : 5 : 0

	f1-score	precision	recall	support
AFIB	0.878077	0.882690	0.873564	875.0
GSVT	0.892895	0.894552	0.891357	691.0
SB	0.945698	0.938982	0.952516	1553.0
SR	0.927566	0.933281	0.921942	907.0
macro avg	0.911059	0.912376	0.909845	4026.0
micro avg	0.917972	0.917972	0.917972	4026.0
weighted avg	0.917854	0.917838	0.917972	4026.0

The result of Model Number:Dataset Number:Featuregroup Number:Rescale of 70 : 0 : 5 : 1

	f1-score	precision	recall	support
AFIB	0.138349	0.139802	0.136926	175.0
GSVT	0.138572	0.137100	0.140076	138.2
SB	0.145941	0.145381	0.146505	310.6
SR	0.142563	0.143278	0.141856	181.4
macro avg	0.141356	0.141390	0.141341	805.2

micro avg 0.142272 0.142272 0.142272 805.2
weighted avg 0.142265 0.142273 0.142272 805.2
The result of Model Number:Dataset Number:Featuregroup Number:Rescale of 70 : 0 : 6 : 0

	f1-score	precision	recall	support
AFIB	0.886528	0.895163	0.878273	204.0
GSVT	0.907029	0.902864	0.911263	300.0
SB	0.919563	0.916399	0.922756	892.0
SR	0.911797	0.915335	0.908330	681.0
macro avg	0.906229	0.907440	0.905156	2077.0
micro avg	0.911997	0.911997	0.911997	2077.0
weighted avg	0.911962	0.912010	0.911997	2077.0

The result of Model Number:Dataset Number:Featuregroup Number:Rescale of 70 : 0 : 6 : 1

	f1-score	precision	recall	support
AFIB	0.770456	0.778657	0.762853	204.0
GSVT	0.793099	0.787814	0.798686	300.0
SB	0.809858	0.809134	0.810584	892.0
SR	0.803030	0.804101	0.801966	681.0
macro avg	0.794111	0.794927	0.793523	2077.0
micro avg	0.801352	0.801352	0.801352	2077.0
weighted avg	0.801329	0.801411	0.801352	2077.0

The result of Model Number:Dataset Number:Featuregroup Number:Rescale of 70 : 0 : 7 : 0

	f1-score	precision	recall	support
AFIB	0.675259	0.675356	0.675251	875.0
GSVT	0.687052	0.681357	0.692895	691.0
SB	0.719335	0.713112	0.725678	1553.0
SR	0.695631	0.711345	0.680620	907.0
macro avg	0.694319	0.695293	0.693611	4026.0
micro avg	0.698941	0.698941	0.698941	4026.0
weighted avg	0.698875	0.699058	0.698941	4026.0

The result of Model Number:Dataset Number:Featuregroup Number:Rescale of 70 : 0 : 7 : 1

	f1-score	precision	recall	support
AFIB	0.741685	0.748358	0.735362	875.0
GSVT	0.756793	0.741448	0.772965	691.0
SB	0.802584	0.793655	0.811812	1553.0
SR	0.770736	0.793647	0.749293	907.0
macro avg	0.767949	0.769277	0.767358	4026.0
micro avg	0.774444	0.774444	0.774444	4026.0
weighted avg	0.774314	0.774848	0.774444	4026.0

The result of Model Number:Dataset Number:Featuregroup Number:Rescale of 70 : 0 : 8 : 0

	f1-score	precision	recall	support
AFIB	0.864623	0.881190	0.848887	204.0
GSVT	0.892117	0.887522	0.896843	300.0
SB	0.914180	0.912757	0.915615	892.0
SR	0.905172	0.904273	0.906102	681.0
macro avg	0.894023	0.896435	0.891862	2077.0
micro avg	0.903231	0.903231	0.903231	2077.0
weighted avg	0.903172	0.903230	0.903231	2077.0

The result of Model Number:Dataset Number:Featuregroup Number:Rescale of 70 : 0 : 8 : 1

	f1-score	precision	recall	support
AFIB	0.751892	0.792793	0.715196	204.0
GSVT	0.789348	0.777410	0.801691	300.0
SB	0.808106	0.802849	0.813444	892.0
SR	0.798136	0.798855	0.797437	681.0
macro avg	0.786871	0.792977	0.781942	2077.0
micro avg	0.796848	0.796848	0.796848	2077.0
weighted avg	0.796606	0.796878	0.796848	2077.0

The result of Model Number:Dataset Number:Featuregroup Number:Rescale of 70 : 0 : 9 : 0

	f1-score	precision	recall	support
AFIB	0.751715	0.747506	0.756007	875.0
GSVT	0.757836	0.762307	0.753464	691.0
SB	0.798318	0.795819	0.800835	1553.0
SR	0.782514	0.787690	0.777422	907.0
macro avg	0.772595	0.773331	0.771932	4026.0
micro avg	0.777687	0.777687	0.777687	4026.0
weighted avg	0.777681	0.777736	0.777687	4026.0

The result of Model Number:Dataset Number:Featuregroup Number:Rescale of 70 : 0 : 9 : 1

	f1-score	precision	recall	support
AFIB	0.844998	0.862795	0.828054	204.0
GSVT	0.871270	0.863310	0.879398	300.0
SB	0.885500	0.883526	0.887485	892.0
SR	0.874976	0.875891	0.874073	681.0
macro avg	0.869186	0.871381	0.867253	2077.0
micro avg	0.876083	0.876083	0.876083	2077.0
weighted avg	0.876016	0.876067	0.876083	2077.0

The result of Model Number:Dataset Number:Featuregroup Number:Rescale of 70 : 0 : 10 : 0

	f1-score	precision	recall	support
AFIB	0.665801	0.695392	0.638718	204.0
GSVT	0.693546	0.688102	0.699093	300.0
SB	0.704140	0.698708	0.709664	892.0

SR	0.696350	0.697500	0.695229	681.0
macro avg	0.689959	0.694926	0.685676	2077.0
micro avg	0.696436	0.696436	0.696436	2077.0
weighted avg	0.696290	0.696454	0.696436	2077.0

The result of Model Number:Dataset Number:Featuregroup Number:Rescale of 70 : 0 : 10 : 1

	f1-score	precision	recall	support
AFIB	0.924092	0.925131	0.923223	204.0
GSVT	0.945308	0.943197	0.947500	300.0
SB	0.965344	0.964056	0.966642	892.0
SR	0.958115	0.960528	0.955726	681.0
macro avg	0.948215	0.948228	0.948272	2077.0
micro avg	0.956033	0.956033	0.956033	2077.0
weighted avg	0.956028	0.956063	0.956033	2077.0

The result of Model Number:Dataset Number:Featuregroup Number:Rescale of 70 : 1 : 0 : 0

	f1-score	precision	recall	support
AFIB	0.741366	0.783540	0.703991	204.0
GSVT	0.783456	0.774829	0.792317	300.0
SB	0.795110	0.787199	0.803210	892.0
SR	0.785874	0.788560	0.783329	681.0
macro avg	0.776451	0.783532	0.770712	2077.0
micro avg	0.785373	0.785373	0.785373	2077.0
weighted avg	0.785120	0.785499	0.785373	2077.0

The result of Model Number:Dataset Number:Featuregroup Number:Rescale of 70 : 1 : 0 : 1

	f1-score	precision	recall	support
AFIB	0.820214	0.809742	0.831008	875.0
GSVT	0.834611	0.839397	0.829901	691.0
SB	0.893399	0.889647	0.897192	1553.0
SR	0.867094	0.881128	0.853514	907.0
macro avg	0.853829	0.854979	0.852904	4026.0
micro avg	0.861418	0.861418	0.861418	4026.0
weighted avg	0.861477	0.861737	0.861418	4026.0

The result of Model Number:Dataset Number:Featuregroup Number:Rescale of 70 : 1 : 1 : 0

	f1-score	precision	recall	support
AFIB	0.663626	0.664740	0.680074	204.0
GSVT	0.657387	0.682898	0.634561	300.0
SB	0.697302	0.659061	0.740539	892.0
SR	0.604412	0.660191	0.558854	681.0
macro avg	0.655682	0.666722	0.653507	2077.0
micro avg	0.659723	0.659723	0.659723	2077.0
weighted avg	0.657773	0.663432	0.659723	2077.0

The result of Model Number:Dataset Number:Featuregroup Number:Rescale of 70 : 1 : 1 : 1

	f1-score	precision	recall	support
AFIB	0.597148	0.620352	0.575660	875.0
GSVT	0.641953	0.637212	0.646850	691.0
SB	0.705457	0.701823	0.709135	1553.0
SR	0.663899	0.651239	0.677133	907.0
macro avg	0.652114	0.652657	0.652194	4026.0
micro avg	0.662226	0.662226	0.662226	4026.0
weighted avg	0.661656	0.661631	0.662226	4026.0

The result of Model Number:Dataset Number:Featuregroup Number:Rescale of 70 : 1 : 2 : 0

	f1-score	precision	recall	support
AFIB	0.683738	0.676183	0.691477	875.0
GSVT	0.696057	0.700342	0.691835	691.0
SB	0.740819	0.739586	0.742059	1553.0
SR	0.722780	0.729539	0.716164	907.0
macro avg	0.710848	0.711413	0.710384	4026.0
micro avg	0.716612	0.716612	0.716612	4026.0
weighted avg	0.716667	0.716807	0.716612	4026.0

The result of Model Number:Dataset Number:Featuregroup Number:Rescale of 70 : 1 : 2 : 1

	f1-score	precision	recall	support
AFIB	0.739567	0.746721	0.732682	204.0
GSVT	0.765737	0.764227	0.767265	300.0
SB	0.781742	0.780357	0.783142	892.0
SR	0.774535	0.774910	0.774194	681.0
macro avg	0.765395	0.766554	0.764321	2077.0
micro avg	0.772959	0.772959	0.772959	2077.0
weighted avg	0.772925	0.772938	0.772959	2077.0

The result of Model Number:Dataset Number:Featuregroup Number:Rescale of 70 : 1 : 3 : 0

	f1-score	precision	recall	support
AFIB	0.791105	0.794335	0.787925	875.0
GSVT	0.736671	0.769183	0.706917	691.0
SB	0.791141	0.716671	0.882893	1553.0
SR	0.596785	0.742755	0.498966	907.0
macro avg	0.728925	0.755736	0.719175	4026.0
micro avg	0.745556	0.745556	0.745556	4026.0
weighted avg	0.737998	0.748439	0.745556	4026.0

The result of Model Number:Dataset Number:Featuregroup Number:Rescale of 70 : 1 : 3 : 1

	f1-score	precision	recall	support
AFIB	0.654514	0.698878	0.615881	204.0

GSVT 0.616707 0.635005 0.599666 300.0
SB 0.644613 0.613668 0.679211 892.0
SR 0.571975 0.597943 0.548792 681.0
macro avg 0.621952 0.636374 0.610888 2077.0
micro avg 0.618740 0.618740 0.618740 2077.0
weighted avg 0.617738 0.619964 0.618740 2077.0
The result of Model Number:Dataset Number:Featuregroup Number:Rescale of 70 : 1 : 4 : 0

	f1-score	precision	recall	support
AFIB	0.638593	0.634572	0.719331	204.0
GSVT	0.778456	0.774452	0.782633	300.0
SB	0.800621	0.796812	0.804476	892.0
SR	0.627997	0.630066	0.626320	681.0
macro avg	0.711417	0.708975	0.733190	2077.0
micro avg	0.734545	0.734545	0.734545	2077.0
weighted avg	0.724906	0.722975	0.734545	2077.0

The result of Model Number:Dataset Number:Featuregroup Number:Rescale of 70 : 1 : 4 : 1

	f1-score	precision	recall	support
AFIB	0.872424	0.880229	0.864865	875.0
GSVT	0.889087	0.872181	0.906831	691.0
SB	0.943691	0.935516	0.952014	1553.0
SR	0.904931	0.925738	0.885080	907.0
macro avg	0.902533	0.903416	0.902197	4026.0
micro avg	0.910239	0.910239	0.910239	4026.0
weighted avg	0.910098	0.910427	0.910239	4026.0

The result of Model Number:Dataset Number:Featuregroup Number:Rescale of 70 : 1 : 5 : 0

	f1-score	precision	recall	support
AFIB	0.756879	0.760483	0.753342	875.0
GSVT	0.766229	0.752304	0.780757	691.0
SB	0.810728	0.807623	0.813859	1553.0
SR	0.786765	0.800232	0.773756	907.0
macro avg	0.780150	0.780160	0.780429	4026.0
micro avg	0.785990	0.785990	0.785990	4026.0
weighted avg	0.785988	0.786218	0.785990	4026.0

The result of Model Number:Dataset Number:Featuregroup Number:Rescale of 70 : 1 : 5 : 1

	f1-score	precision	recall	support
AFIB	0.712445	0.713679	0.711225	875.0
GSVT	0.721083	0.712797	0.729623	691.0
SB	0.759325	0.750169	0.768711	1553.0
SR	0.723232	0.745146	0.702587	907.0
macro avg	0.729021	0.730448	0.728037	4026.0

micro avg 0.734611 0.734611 0.734611 4026.0
weighted avg 0.734442 0.734693 0.734611 4026.0
The result of Model Number:Dataset Number:Featuregroup Number:Rescale of 70 : 1 : 6 : 0

	f1-score	precision	recall	support
AFIB	0.935659	0.947148	0.924592	204.0
GSVT	0.954505	0.950428	0.958640	300.0
SB	0.966059	0.963160	0.968981	892.0
SR	0.957928	0.960208	0.955690	681.0
macro avg	0.953538	0.955236	0.951976	2077.0
micro avg	0.958770	0.958770	0.958770	2077.0
weighted avg	0.958739	0.958780	0.958770	2077.0

The result of Model Number:Dataset Number:Featuregroup Number:Rescale of 70 : 1 : 6 : 1

	f1-score	precision	recall	support
AFIB	0.752912	0.777915	0.729752	204.0
GSVT	0.781485	0.775216	0.787974	300.0
SB	0.798751	0.795469	0.802063	892.0
SR	0.791291	0.791175	0.791408	681.0
macro avg	0.781110	0.784944	0.777799	2077.0
micro avg	0.789432	0.789432	0.789432	2077.0
weighted avg	0.789309	0.789412	0.789432	2077.0

The result of Model Number:Dataset Number:Featuregroup Number:Rescale of 70 : 1 : 7 : 0

	f1-score	precision	recall	support
AFIB	0.869289	0.878555	0.860359	204.0
GSVT	0.885276	0.880419	0.890284	300.0
SB	0.905711	0.904201	0.907231	892.0
SR	0.898161	0.899624	0.896714	681.0
macro avg	0.889609	0.890700	0.888647	2077.0
micro avg	0.896731	0.896731	0.896731	2077.0
weighted avg	0.896706	0.896746	0.896731	2077.0

The result of Model Number:Dataset Number:Featuregroup Number:Rescale of 70 : 1 : 7 : 1

	f1-score	precision	recall	support
AFIB	0.728504	0.739094	0.718543	204.0
GSVT	0.744570	0.734497	0.754984	300.0
SB	0.755776	0.747900	0.763826	892.0
SR	0.740797	0.753055	0.728944	681.0
macro avg	0.742412	0.743637	0.741574	2077.0
micro avg	0.746664	0.746664	0.746664	2077.0
weighted avg	0.746568	0.746789	0.746664	2077.0

The result of Model Number:Dataset Number:Featuregroup Number:Rescale of 70 : 1 : 8 : 0

	f1-score	precision	recall	support
AFIB	0.663868	0.655054	0.672973	875.0
GSVT	0.677342	0.683763	0.671070	691.0
SB	0.720789	0.719084	0.722506	1553.0
SR	0.701235	0.708608	0.694043	907.0
macro avg	0.690808	0.691628	0.690148	4026.0
micro avg	0.696500	0.696500	0.696500	4026.0
weighted avg	0.696556	0.696746	0.696500	4026.0

The result of Model Number:Dataset Number:Featuregroup Number:Rescale of 70 : 1 : 8 : 1

	f1-score	precision	recall	support
AFIB	0.733641	0.773549	0.697836	204.0
GSVT	0.770187	0.758539	0.782230	300.0
SB	0.788490	0.783361	0.793699	892.0
SR	0.778762	0.779464	0.778080	681.0
macro avg	0.767770	0.773728	0.762961	2077.0
micro avg	0.777506	0.777506	0.777506	2077.0
weighted avg	0.777270	0.777534	0.777506	2077.0

The result of Model Number:Dataset Number:Featuregroup Number:Rescale of 70 : 1 : 9 : 0

	f1-score	precision	recall	support
AFIB	0.765102	0.860289	0.689555	204.0
GSVT	0.907737	0.888354	0.928026	300.0
SB	0.969027	0.964087	0.974022	892.0
SR	0.947287	0.935291	0.959656	681.0
macro avg	0.897288	0.912005	0.887815	2077.0
micro avg	0.934728	0.934728	0.934728	2077.0
weighted avg	0.933017	0.933512	0.934728	2077.0

The result of Model Number:Dataset Number:Featuregroup Number:Rescale of 70 : 1 : 9 : 1

	f1-score	precision	recall	support
AFIB	0.856827	0.877675	0.837159	204.0
GSVT	0.867598	0.855654	0.879996	300.0
SB	0.885117	0.876278	0.894142	892.0
SR	0.869663	0.881076	0.858567	681.0
macro avg	0.869801	0.872671	0.867466	2077.0
micro avg	0.874838	0.874838	0.874838	2077.0
weighted avg	0.874741	0.875009	0.874838	2077.0

The result of Model Number:Dataset Number:Featuregroup Number:Rescale of 70 : 1 : 10 : 0

	f1-score	precision	recall	support
AFIB	0.792109	0.794540	0.789818	204.0
GSVT	0.812917	0.810852	0.815066	300.0
SB	0.831077	0.830062	0.832100	892.0

SR 0.824275 0.825860 0.822703 681.0
macro avg 0.815095 0.815328 0.814922 2077.0
micro avg 0.822406 0.822406 0.822406 2077.0
weighted avg 0.822397 0.822421 0.822406 2077.0
The result of Model Number:Dataset Number:Featuregroup Number:Rescale of 70 : 1 : 10 : 1

	f1-score	precision	recall	support
AFIB	0.748816	0.750263	0.747431	875.0
GSVT	0.725476	0.722289	0.728735	691.0
SB	0.758036	0.711963	0.810507	1553.0
SR	0.610113	0.706943	0.536758	907.0
macro avg	0.710610	0.722865	0.705858	4026.0
micro avg	0.721091	0.721091	0.721091	4026.0
weighted avg	0.717119	0.720928	0.721091	4026.0

The result of Model Number:Dataset Number:Featuregroup Number:Rescale of 71 : 0 : 0 : 0

	f1-score	precision	recall	support
AFIB	0.861183	0.852520	0.870047	875.0
GSVT	0.868889	0.876626	0.861352	691.0
SB	0.919247	0.916425	0.922087	1553.0
SR	0.901446	0.909336	0.893694	907.0
macro avg	0.887691	0.888727	0.886795	4026.0
micro avg	0.893956	0.893956	0.893956	4026.0
weighted avg	0.893974	0.894108	0.893956	4026.0

The result of Model Number:Dataset Number:Featuregroup Number:Rescale of 71 : 0 : 0 : 1

	f1-score	precision	recall	support
AFIB	0.655026	0.641978	0.668650	875.0
GSVT	0.533134	0.495786	0.668268	691.0
SB	0.432579	0.433324	0.431837	1553.0
SR	0.701266	0.707441	0.695227	907.0
macro avg	0.580501	0.569632	0.615995	4026.0
micro avg	0.583223	0.583223	0.583223	4026.0
weighted avg	0.558715	0.551148	0.583223	4026.0

The result of Model Number:Dataset Number:Featuregroup Number:Rescale of 71 : 0 : 1 : 0

	f1-score	precision	recall	support
AFIB	0.650417	0.677573	0.625472	875.0
GSVT	0.702678	0.699120	0.706357	691.0
SB	0.770507	0.766171	0.774914	1553.0
SR	0.721464	0.705444	0.738241	907.0
macro avg	0.711267	0.712077	0.711246	4026.0
micro avg	0.722406	0.722406	0.722406	4026.0
weighted avg	0.721717	0.721726	0.722406	4026.0

The result of Model Number:Dataset Number:Featuregroup Number:Rescale of 71 : 0 : 1 : 1

	f1-score	precision	recall	support
AFIB	0.662682	0.667147	0.658375	875.0
GSVT	0.674509	0.665140	0.684269	691.0
SB	0.718763	0.713841	0.723759	1553.0
SR	0.689635	0.701548	0.678158	907.0
macro avg	0.686397	0.686919	0.686140	4026.0
micro avg	0.692498	0.692498	0.692498	4026.0
weighted avg	0.692417	0.692565	0.692498	4026.0

The result of Model Number:Dataset Number:Featuregroup Number:Rescale of 71 : 0 : 2 : 0

	f1-score	precision	recall	support
AFIB	0.914202	0.919220	0.909298	875.0
GSVT	0.915481	0.903907	0.927499	691.0
SB	0.962787	0.958923	0.966690	1553.0
SR	0.942119	0.953556	0.931011	907.0
macro avg	0.933647	0.933901	0.933624	4026.0
micro avg	0.939452	0.939452	0.939452	4026.0
weighted avg	0.939452	0.939642	0.939452	4026.0

The result of Model Number:Dataset Number:Featuregroup Number:Rescale of 71 : 0 : 2 : 1

	f1-score	precision	recall	support
AFIB	0.874715	0.883236	0.866570	204.0
GSVT	0.894943	0.890834	0.899121	300.0
SB	0.907310	0.904189	0.910460	892.0
SR	0.899648	0.903138	0.896227	681.0
macro avg	0.894154	0.895349	0.893095	2077.0
micro avg	0.899845	0.899845	0.899845	2077.0
weighted avg	0.899810	0.899857	0.899845	2077.0

The result of Model Number:Dataset Number:Featuregroup Number:Rescale of 71 : 0 : 3 : 0

	f1-score	precision	recall	support
AFIB	0.826337	0.842196	0.811112	875.0
GSVT	0.880849	0.882193	0.879565	691.0
SB	0.959132	0.954917	0.963391	1553.0
SR	0.906350	0.896497	0.916515	907.0
macro avg	0.893167	0.893951	0.892646	4026.0
micro avg	0.905347	0.905347	0.905347	4026.0
weighted avg	0.904944	0.904775	0.905347	4026.0

The result of Model Number:Dataset Number:Featuregroup Number:Rescale of 71 : 0 : 3 : 1

	f1-score	precision	recall	support
AFIB	0.711319	0.725102	0.698369	204.0

GSVT 0.738902 0.732724 0.745321 300.0
SB 0.752689 0.751179 0.754208 892.0
SR 0.745512 0.746284 0.744745 681.0
macro avg 0.737105 0.738822 0.735661 2077.0
micro avg 0.744337 0.744337 0.744337 2077.0
weighted avg 0.744281 0.744347 0.744337 2077.0
The result of Model Number:Dataset Number:Featuregroup Number:Rescale of 71 : 0 : 4 : 0

	f1-score	precision	recall	support
AFIB	0.724238	0.729118	0.719531	875.0
GSVT	0.737163	0.726924	0.747830	691.0
SB	0.785528	0.780149	0.790988	1553.0
SR	0.753695	0.766714	0.741152	907.0
macro avg	0.750156	0.750726	0.749875	4026.0
micro avg	0.756823	0.756823	0.756823	4026.0
weighted avg	0.756735	0.756896	0.756823	4026.0

The result of Model Number:Dataset Number:Featuregroup Number:Rescale of 71 : 0 : 4 : 1

	f1-score	precision	recall	support
AFIB	0.647888	0.648061	0.647828	875.0
GSVT	0.659170	0.656737	0.661705	691.0
SB	0.705184	0.703472	0.706907	1553.0
SR	0.688366	0.693306	0.683514	907.0
macro avg	0.675152	0.675394	0.674989	4026.0
micro avg	0.681039	0.681039	0.681039	4026.0
weighted avg	0.681045	0.681117	0.681039	4026.0

The result of Model Number:Dataset Number:Featuregroup Number:Rescale of 71 : 0 : 5 : 0

	f1-score	precision	recall	support
AFIB	0.824170	0.832198	0.816496	204.0
GSVT	0.843229	0.839357	0.847165	300.0
SB	0.854882	0.851941	0.857850	892.0
SR	0.847662	0.850951	0.844439	681.0
macro avg	0.842486	0.843612	0.841488	2077.0
micro avg	0.847848	0.847848	0.847848	2077.0
weighted avg	0.847815	0.847859	0.847848	2077.0

The result of Model Number:Dataset Number:Featuregroup Number:Rescale of 71 : 0 : 5 : 1

	f1-score	precision	recall	support
AFIB	0.745905	0.746104	0.745836	875.0
GSVT	0.758894	0.756092	0.761812	691.0
SB	0.811868	0.809897	0.813852	1553.0
SR	0.792506	0.798194	0.786921	907.0
macro avg	0.777293	0.777572	0.777105	4026.0

micro avg 0.784071 0.784071 0.784071 4026.0
weighted avg 0.784078 0.784161 0.784071 4026.0
The result of Model Number:Dataset Number:Featuregroup Number:Rescale of 71 : 0 : 6 : 0

	f1-score	precision	recall	support
AFIB	0.650003	0.640216	0.665506	875.0
GSVT	0.620635	0.618829	0.625930	691.0
SB	0.662759	0.617585	0.720574	1553.0
SR	0.490947	0.656250	0.406808	907.0
macro avg	0.606086	0.633220	0.604704	4026.0
micro avg	0.621674	0.621674	0.621674	4026.0
weighted avg	0.614050	0.631428	0.621674	4026.0

The result of Model Number:Dataset Number:Featuregroup Number:Rescale of 71 : 0 : 6 : 1

	f1-score	precision	recall	support
AFIB	0.770248	0.770935	0.769641	875.0
GSVT	0.784357	0.786415	0.782442	691.0
SB	0.832605	0.827062	0.838232	1553.0
SR	0.816383	0.823836	0.809092	907.0
macro avg	0.800898	0.802062	0.799852	4026.0
micro avg	0.807184	0.807184	0.807184	4026.0
weighted avg	0.807117	0.807160	0.807184	4026.0

The result of Model Number:Dataset Number:Featuregroup Number:Rescale of 71 : 0 : 7 : 0

	f1-score	precision	recall	support
AFIB	0.923183	0.930883	0.915927	204.0
GSVT	0.947571	0.943207	0.951995	300.0
SB	0.961861	0.958444	0.965308	892.0
SR	0.952977	0.957245	0.948796	681.0
macro avg	0.946398	0.947445	0.945507	2077.0
micro avg	0.953121	0.953121	0.953121	2077.0
weighted avg	0.953085	0.953143	0.953121	2077.0

The result of Model Number:Dataset Number:Featuregroup Number:Rescale of 71 : 0 : 7 : 1

	f1-score	precision	recall	support
AFIB	0.831519	0.834380	0.828783	875.0
GSVT	0.847305	0.830401	0.865066	691.0
SB	0.896941	0.892090	0.901849	1553.0
SR	0.862249	0.882297	0.843143	907.0
macro avg	0.859503	0.859792	0.859710	4026.0
micro avg	0.866430	0.866430	0.866430	4026.0
weighted avg	0.866387	0.866753	0.866430	4026.0

The result of Model Number:Dataset Number:Featuregroup Number:Rescale of 71 : 0 : 8 : 0

	f1-score	precision	recall	support
AFIB	0.746769	0.753282	0.740555	204.0
GSVT	0.772595	0.771334	0.773879	300.0
SB	0.789017	0.787093	0.790961	892.0
SR	0.781784	0.782962	0.780635	681.0
macro avg	0.772541	0.773668	0.771507	2077.0
micro avg	0.780157	0.780157	0.780157	2077.0
weighted avg	0.780124	0.780141	0.780157	2077.0

The result of Model Number:Dataset Number:Featuregroup Number:Rescale of 71 : 0 : 8 : 1

	f1-score	precision	recall	support
AFIB	0.896591	0.902994	0.890441	204.0
GSVT	0.927943	0.927344	0.928560	300.0
SB	0.946509	0.944409	0.948628	892.0
SR	0.938048	0.939183	0.936945	681.0
macro avg	0.927273	0.928482	0.926143	2077.0
micro avg	0.936184	0.936184	0.936184	2077.0
weighted avg	0.936150	0.936163	0.936184	2077.0

The result of Model Number:Dataset Number:Featuregroup Number:Rescale of 71 : 0 : 9 : 0

	f1-score	precision	recall	support
AFIB	0.812146	0.815184	0.809476	875.0
GSVT	0.830427	0.838439	0.822734	691.0
SB	0.883004	0.873742	0.892532	1553.0
SR	0.852427	0.859929	0.845347	907.0
macro avg	0.844501	0.846823	0.842522	4026.0
micro avg	0.851871	0.851871	0.851871	4026.0
weighted avg	0.851691	0.851844	0.851871	4026.0

The result of Model Number:Dataset Number:Featuregroup Number:Rescale of 71 : 0 : 9 : 1

	f1-score	precision	recall	support
AFIB	0.662778	0.712601	0.620157	204.0
GSVT	0.632071	0.652134	0.613302	300.0
SB	0.658528	0.625942	0.694700	892.0
SR	0.582946	0.608002	0.559932	681.0
macro avg	0.634081	0.649670	0.622023	2077.0
micro avg	0.631434	0.631434	0.631434	2077.0
weighted avg	0.630342	0.632355	0.631434	2077.0

The result of Model Number:Dataset Number:Featuregroup Number:Rescale of 71 : 0 : 10 : 0

	f1-score	precision	recall	support
AFIB	0.710363	0.719486	0.701503	875.0
GSVT	0.718221	0.712413	0.724240	691.0
SB	0.768291	0.765982	0.770617	1553.0

SR 0.749267 0.748952 0.749592 907.0
macro avg 0.736536 0.736708 0.736488 4026.0
micro avg 0.742900 0.742900 0.742900 4026.0
weighted avg 0.742822 0.742846 0.742900 4026.0
The result of Model Number:Dataset Number:Featuregroup Number:Rescale of 71 : 0 : 10 : 1
f1-score precision recall support
AFIB 0.749861 0.739223 0.760828 875.0
GSVT 0.765288 0.772747 0.757978 691.0
SB 0.814416 0.811762 0.817089 1553.0
SR 0.795397 0.805563 0.785493 907.0
macro avg 0.781241 0.782324 0.780347 4026.0
micro avg 0.787598 0.787598 0.787598 4026.0
weighted avg 0.787669 0.787904 0.787598 4026.0
The result of Model Number:Dataset Number:Featuregroup Number:Rescale of 71 : 1 : 0 : 0
f1-score precision recall support
AFIB 0.741493 0.730328 0.759178 875.0
GSVT 0.707992 0.705931 0.714032 691.0
SB 0.756044 0.704513 0.821997 1553.0
SR 0.560049 0.748620 0.464068 907.0
macro avg 0.691395 0.722348 0.689819 4026.0
micro avg 0.709177 0.709177 0.709177 4026.0
weighted avg 0.700480 0.720304 0.709177 4026.0
The result of Model Number:Dataset Number:Featuregroup Number:Rescale of 71 : 1 : 0 : 1
f1-score precision recall support
AFIB 0.758672 0.759032 0.758420 875.0
GSVT 0.778320 0.771583 0.785202 691.0
SB 0.831248 0.825191 0.837403 1553.0
SR 0.795736 0.811555 0.780564 907.0
macro avg 0.790994 0.791840 0.790397 4026.0
micro avg 0.798472 0.798472 0.798472 4026.0
weighted avg 0.798390 0.798539 0.798472 4026.0
The result of Model Number:Dataset Number:Featuregroup Number:Rescale of 71 : 1 : 1 : 0
f1-score precision recall support
AFIB 0.827188 0.849046 0.806509 204.0
GSVT 0.849873 0.842813 0.857208 300.0
SB 0.873292 0.870088 0.876523 892.0
SR 0.859771 0.860812 0.858754 681.0
macro avg 0.852531 0.855690 0.849749 2077.0
micro avg 0.861031 0.861031 0.861031 2077.0
weighted avg 0.860948 0.861040 0.861031 2077.0

The result of Model Number:Dataset Number:Featuregroup Number:Rescale of 71 : 1 : 1 : 1

	f1-score	precision	recall	support
AFIB	0.857344	0.847870	0.867048	875.0
GSVT	0.872790	0.878164	0.867497	691.0
SB	0.928918	0.927372	0.930474	1553.0
SR	0.906300	0.914774	0.898004	907.0
macro avg	0.891338	0.892045	0.890755	4026.0
micro avg	0.898565	0.898565	0.898565	4026.0
weighted avg	0.898633	0.898810	0.898565	4026.0

The result of Model Number:Dataset Number:Featuregroup Number:Rescale of 71 : 1 : 2 : 0

	f1-score	precision	recall	support
AFIB	0.913416	0.918429	0.908516	875.0
GSVT	0.914694	0.903129	0.926701	691.0
SB	0.961958	0.958098	0.965858	1553.0
SR	0.941308	0.952735	0.930211	907.0
macro avg	0.932844	0.933098	0.932821	4026.0
micro avg	0.938644	0.938644	0.938644	4026.0
weighted avg	0.938644	0.938834	0.938644	4026.0

The result of Model Number:Dataset Number:Featuregroup Number:Rescale of 71 : 1 : 2 : 1

	f1-score	precision	recall	support
AFIB	0.921597	0.929999	0.913467	204.0
GSVT	0.945648	0.942551	0.948794	300.0
SB	0.962935	0.962293	0.963581	892.0
SR	0.954466	0.954192	0.954754	681.0
macro avg	0.946161	0.947259	0.945149	2077.0
micro avg	0.953629	0.953629	0.953629	2077.0
weighted avg	0.953601	0.953613	0.953629	2077.0

The result of Model Number:Dataset Number:Featuregroup Number:Rescale of 71 : 1 : 3 : 0

	f1-score	precision	recall	support
AFIB	0.677533	0.683630	0.671757	875.0
GSVT	0.691335	0.677317	0.706108	691.0
SB	0.733165	0.725009	0.741595	1553.0
SR	0.704072	0.725001	0.684483	907.0
macro avg	0.701526	0.702739	0.700986	4026.0
micro avg	0.707460	0.707460	0.707460	4026.0
weighted avg	0.707340	0.707828	0.707460	4026.0

The result of Model Number:Dataset Number:Featuregroup Number:Rescale of 71 : 1 : 3 : 1

	f1-score	precision	recall	support
AFIB	0.671155	0.676475	0.665935	875.0

GSVT 0.679664 0.676667 0.682710 691.0
SB 0.722742 0.719885 0.725627 1553.0
SR 0.704982 0.706974 0.703035 907.0
macro avg 0.694636 0.695000 0.694327 4026.0
micro avg 0.700198 0.700198 0.700198 4026.0
weighted avg 0.700135 0.700124 0.700198 4026.0
The result of Model Number:Dataset Number:Featuregroup Number:Rescale of 71 : 1 : 4 : 0

	f1-score	precision	recall	support
AFIB	0.826682	0.851753	0.803080	875.0
GSVT	0.882495	0.876375	0.888748	691.0
SB	0.965557	0.961864	0.969286	1553.0
SR	0.913184	0.899478	0.927368	907.0
macro avg	0.896980	0.897367	0.897121	4026.0
micro avg	0.909897	0.909897	0.909897	4026.0
weighted avg	0.909319	0.909205	0.909897	4026.0

The result of Model Number:Dataset Number:Featuregroup Number:Rescale of 71 : 1 : 4 : 1

	f1-score	precision	recall	support
AFIB	0.695376	0.696622	0.694157	875.0
GSVT	0.665870	0.669367	0.662427	691.0
SB	0.704328	0.665584	0.747869	1553.0
SR	0.584948	0.658402	0.526290	907.0
macro avg	0.662630	0.672494	0.657686	4026.0
micro avg	0.671612	0.671612	0.671612	4026.0
weighted avg	0.668887	0.671361	0.671612	4026.0

The result of Model Number:Dataset Number:Featuregroup Number:Rescale of 71 : 1 : 5 : 0

	f1-score	precision	recall	support
AFIB	0.814394	0.815496	0.813451	875.0
GSVT	0.827079	0.826653	0.827535	691.0
SB	0.863798	0.860707	0.866914	1553.0
SR	0.843113	0.847839	0.838494	907.0
macro avg	0.837096	0.837674	0.836598	4026.0
micro avg	0.842133	0.842133	0.842133	4026.0
weighted avg	0.842098	0.842137	0.842133	4026.0

The result of Model Number:Dataset Number:Featuregroup Number:Rescale of 71 : 1 : 5 : 1

	f1-score	precision	recall	support
AFIB	0.659610	0.660718	0.675959	204.0
GSVT	0.653409	0.678766	0.630722	300.0
SB	0.693083	0.655073	0.736058	892.0
SR	0.600755	0.656196	0.555473	681.0
macro avg	0.651714	0.662688	0.649553	2077.0

micro avg	0.655731	0.655731	0.655731	2077.0
weighted avg	0.653792	0.659418	0.655731	2077.0

The result of Model Number:Dataset Number:Featuregroup Number:Rescale of 71 : 1 : 6 : 0

	f1-score	precision	recall	support
AFIB	0.892312	0.894113	0.890536	204.0
GSVT	0.917137	0.914410	0.919885	300.0
SB	0.932333	0.931502	0.933169	892.0
SR	0.924319	0.926095	0.922563	681.0
macro avg	0.916525	0.916530	0.916538	2077.0
micro avg	0.923585	0.923585	0.923585	2077.0
weighted avg	0.923580	0.923588	0.923585	2077.0

The result of Model Number:Dataset Number:Featuregroup Number:Rescale of 71 : 1 : 6 : 1

	f1-score	precision	recall	support
AFIB	0.667105	0.670753	0.663518	875.0
GSVT	0.663925	0.658327	0.669686	691.0
SB	0.698284	0.691671	0.705027	1553.0
SR	0.672296	0.684836	0.660215	907.0
macro avg	0.675402	0.676397	0.674612	4026.0
micro avg	0.679844	0.679844	0.679844	4026.0
weighted avg	0.679755	0.679862	0.679844	4026.0

The result of Model Number:Dataset Number:Featuregroup Number:Rescale of 71 : 1 : 7 : 0

	f1-score	precision	recall	support
AFIB	0.737907	0.739386	0.736465	875.0
GSVT	0.739734	0.735741	0.743791	691.0
SB	0.773271	0.769140	0.777452	1553.0
SR	0.754771	0.763722	0.746049	907.0
macro avg	0.751421	0.751997	0.750939	4026.0
micro avg	0.755692	0.755692	0.755692	4026.0
weighted avg	0.755661	0.755720	0.755692	4026.0

The result of Model Number:Dataset Number:Featuregroup Number:Rescale of 71 : 1 : 7 : 1

	f1-score	precision	recall	support
AFIB	0.871013	0.875582	0.866544	875.0
GSVT	0.889226	0.887629	0.890892	691.0
SB	0.965547	0.958142	0.973069	1553.0
SR	0.931482	0.940805	0.922347	907.0
macro avg	0.914317	0.915540	0.913213	4026.0
micro avg	0.924386	0.924386	0.924386	4026.0
weighted avg	0.924228	0.924191	0.924386	4026.0

The result of Model Number:Dataset Number:Featuregroup Number:Rescale of 71 : 1 : 8 : 0

	f1-score	precision	recall	support
AFIB	0.788955	0.802071	0.776441	204.0
GSVT	0.804083	0.798371	0.809938	300.0
SB	0.822734	0.818110	0.827429	892.0
SR	0.812252	0.817221	0.807369	681.0
macro avg	0.807006	0.808943	0.805294	2077.0
micro avg	0.813317	0.813317	0.813317	2077.0
weighted avg	0.813285	0.813392	0.813317	2077.0

The result of Model Number:Dataset Number:Featuregroup Number:Rescale of 71 : 1 : 8 : 1

	f1-score	precision	recall	support
AFIB	0.823600	0.836651	0.811060	204.0
GSVT	0.844770	0.837581	0.852088	300.0
SB	0.859355	0.857440	0.861282	892.0
SR	0.851213	0.853105	0.849345	681.0
macro avg	0.844734	0.846194	0.843444	2077.0
micro avg	0.851107	0.851107	0.851107	2077.0
weighted avg	0.851067	0.851108	0.851107	2077.0

The result of Model Number:Dataset Number:Featuregroup Number:Rescale of 71 : 1 : 9 : 0

	f1-score	precision	recall	support
AFIB	0.921172	0.923999	0.918508	204.0
GSVT	0.945371	0.942968	0.947870	300.0
SB	0.966489	0.965309	0.967679	892.0
SR	0.958579	0.960422	0.956751	681.0
macro avg	0.947903	0.948175	0.947702	2077.0
micro avg	0.956405	0.956405	0.956405	2077.0
weighted avg	0.956394	0.956422	0.956405	2077.0

The result of Model Number:Dataset Number:Featuregroup Number:Rescale of 71 : 1 : 9 : 1

	f1-score	precision	recall	support
AFIB	0.661808	0.666266	0.657506	875.0
GSVT	0.673619	0.664262	0.683366	691.0
SB	0.717814	0.712899	0.722804	1553.0
SR	0.688725	0.700622	0.677263	907.0
macro avg	0.685491	0.686012	0.685235	4026.0
micro avg	0.691584	0.691584	0.691584	4026.0
weighted avg	0.691503	0.691650	0.691584	4026.0

The result of Model Number:Dataset Number:Featuregroup Number:Rescale of 71 : 1 : 10 : 0

	f1-score	precision	recall	support
AFIB	0.712471	0.704516	0.720615	875.0
GSVT	0.726646	0.733302	0.720121	691.0
SB	0.775001	0.772478	0.777545	1553.0

SR 0.754910 0.762473 0.747499 907.0
macro avg 0.742257 0.743192 0.741445 4026.0
micro avg 0.748547 0.748547 0.748547 4026.0
weighted avg 0.748586 0.748729 0.748547 4026.0
The result of Model Number:Dataset Number:Featuregroup Number:Rescale of 71 : 1 : 10 : 1
f1-score precision recall support
AFIB 0.852474 0.900969 0.809499 204.0
GSVT 0.900873 0.890953 0.911062 300.0
SB 0.914274 0.905176 0.923587 892.0
SR 0.903653 0.906742 0.900727 681.0
macro avg 0.892818 0.900960 0.886219 2077.0
micro avg 0.903077 0.903077 0.903077 2077.0
weighted avg 0.902786 0.903222 0.903077 2077.0
The result of Model Number:Dataset Number:Featuregroup Number:Rescale of 72 : 0 : 0 : 0
f1-score precision recall support
AFIB 0.913331 0.923778 0.903472 204.0
GSVT 0.934710 0.929179 0.940322 300.0
SB 0.897304 0.864159 0.953252 892.0
SR 0.752696 0.756339 0.749108 681.0
macro avg 0.874510 0.868364 0.886539 2077.0
micro avg 0.879561 0.879561 0.879561 2077.0
weighted avg 0.856868 0.844054 0.879561 2077.0
The result of Model Number:Dataset Number:Featuregroup Number:Rescale of 72 : 0 : 0 : 1
f1-score precision recall support
AFIB 0.904588 0.915695 0.893888 204.0
GSVT 0.922808 0.918866 0.926805 300.0
SB 0.933978 0.931175 0.936803 892.0
SR 0.926117 0.928321 0.923953 681.0
macro avg 0.921873 0.923514 0.920362 2077.0
micro avg 0.926931 0.926931 0.926931 2077.0
weighted avg 0.926901 0.926941 0.926931 2077.0
The result of Model Number:Dataset Number:Featuregroup Number:Rescale of 72 : 0 : 1 : 0
f1-score precision recall support
AFIB 0.683277 0.689932 0.676914 204.0
GSVT 0.699077 0.695867 0.702341 300.0
SB 0.708738 0.706299 0.711198 892.0
SR 0.702752 0.705479 0.700080 681.0
macro avg 0.698461 0.699394 0.697633 2077.0
micro avg 0.702906 0.702906 0.702906 2077.0
weighted avg 0.702879 0.702916 0.702906 2077.0

The result of Model Number:Dataset Number:Featuregroup Number:Rescale of 72 : 0 : 1 : 1

	f1-score	precision	recall	support
AFIB	0.890089	0.891293	0.889058	875.0
GSVT	0.903952	0.903487	0.904451	691.0
SB	0.944085	0.940706	0.947490	1553.0
SR	0.921477	0.926642	0.916428	907.0
macro avg	0.914901	0.915532	0.914357	4026.0
micro avg	0.920406	0.920406	0.920406	4026.0
weighted avg	0.920368	0.920410	0.920406	4026.0

The result of Model Number:Dataset Number:Featuregroup Number:Rescale of 72 : 0 : 2 : 0

	f1-score	precision	recall	support
AFIB	0.693731	0.688924	0.698744	875.0
GSVT	0.704433	0.707292	0.701711	691.0
SB	0.764311	0.756364	0.772431	1553.0
SR	0.725720	0.742607	0.709651	907.0
macro avg	0.722049	0.723797	0.720634	4026.0
micro avg	0.730135	0.730135	0.730135	4026.0
weighted avg	0.730000	0.730185	0.730135	4026.0

The result of Model Number:Dataset Number:Featuregroup Number:Rescale of 72 : 0 : 2 : 1

	f1-score	precision	recall	support
AFIB	0.645160	0.628212	0.685549	875.0
GSVT	0.620638	0.587441	0.707514	691.0
SB	0.588087	0.584370	0.591868	1553.0
SR	0.571233	0.581075	0.561878	907.0
macro avg	0.606279	0.595275	0.636702	4026.0
micro avg	0.625321	0.625321	0.625321	4026.0
weighted avg	0.602281	0.593683	0.625321	4026.0

The result of Model Number:Dataset Number:Featuregroup Number:Rescale of 72 : 0 : 3 : 0

	f1-score	precision	recall	support
AFIB	0.842349	0.846972	0.837830	875.0
GSVT	0.843527	0.832863	0.854600	691.0
SB	0.887115	0.883555	0.890711	1553.0
SR	0.868071	0.878609	0.857837	907.0
macro avg	0.860266	0.860500	0.860245	4026.0
micro avg	0.865614	0.865614	0.865614	4026.0
weighted avg	0.865614	0.865789	0.865614	4026.0

The result of Model Number:Dataset Number:Featuregroup Number:Rescale of 72 : 0 : 3 : 1

	f1-score	precision	recall	support
AFIB	0.675945	0.669608	0.682489	875.0

GSVT 0.640860 0.625457 0.687971 691.0
SB 0.741036 0.738314 0.743797 1553.0
SR 0.576364 0.580406 0.572386 907.0
macro avg 0.658551 0.653446 0.671661 4026.0
micro avg 0.682274 0.682274 0.682274 4026.0
weighted avg 0.672597 0.668437 0.682274 4026.0
The result of Model Number:Dataset Number:Featuregroup Number:Rescale of 72 : 0 : 4 : 0

	f1-score	precision	recall	support
AFIB	0.859187	0.854861	0.863595	875.0
GSVT	0.875922	0.880163	0.871736	691.0
SB	0.932910	0.930764	0.935071	1553.0
SR	0.909556	0.914422	0.904751	907.0
macro avg	0.894394	0.895052	0.893788	4026.0
micro avg	0.901836	0.901836	0.901836	4026.0
weighted avg	0.901845	0.901901	0.901836	4026.0

The result of Model Number:Dataset Number:Featuregroup Number:Rescale of 72 : 0 : 4 : 1

	f1-score	precision	recall	support
AFIB	0.882433	0.900447	0.865516	204.0
GSVT	0.914476	0.906531	0.922728	300.0
SB	0.932058	0.930394	0.933730	892.0
SR	0.923382	0.923933	0.922838	681.0
macro avg	0.913087	0.915326	0.911203	2077.0
micro avg	0.921870	0.921870	0.921870	2077.0
weighted avg	0.921800	0.921887	0.921870	2077.0

The result of Model Number:Dataset Number:Featuregroup Number:Rescale of 72 : 0 : 5 : 0

	f1-score	precision	recall	support
AFIB	0.804550	0.795016	0.814383	875.0
GSVT	0.816001	0.823889	0.808342	691.0
SB	0.866049	0.862736	0.869395	1553.0
SR	0.845858	0.855617	0.836344	907.0
macro avg	0.833115	0.834315	0.832116	4026.0
micro avg	0.839514	0.839514	0.839514	4026.0
weighted avg	0.839544	0.839747	0.839514	4026.0

The result of Model Number:Dataset Number:Featuregroup Number:Rescale of 72 : 0 : 5 : 1

	f1-score	precision	recall	support
AFIB	0.782848	0.792562	0.773632	204.0
GSVT	0.796885	0.791440	0.802489	300.0
SB	0.815756	0.814482	0.817035	892.0
SR	0.809896	0.811210	0.808594	681.0
macro avg	0.801346	0.802423	0.800437	2077.0

micro avg 0.807903 0.807903 0.807903 2077.0
weighted avg 0.807877 0.807928 0.807903 2077.0
The result of Model Number:Dataset Number:Featuregroup Number:Rescale of 72 : 0 : 6 : 0

	f1-score	precision	recall	support
AFIB	0.705505	0.711030	0.700102	875.0
GSVT	0.707366	0.698338	0.716674	691.0
SB	0.741947	0.738971	0.744954	1553.0
SR	0.726272	0.733401	0.719306	907.0
macro avg	0.720272	0.720435	0.720259	4026.0
micro avg	0.724574	0.724574	0.724574	4026.0
weighted avg	0.724560	0.724669	0.724574	4026.0

The result of Model Number:Dataset Number:Featuregroup Number:Rescale of 72 : 0 : 6 : 1

	f1-score	precision	recall	support
AFIB	0.700475	0.707323	0.693923	875.0
GSVT	0.695125	0.689851	0.700709	691.0
SB	0.735521	0.727953	0.743313	1553.0
SR	0.712174	0.723628	0.701279	907.0
macro avg	0.710824	0.712189	0.709806	4026.0
micro avg	0.715797	0.715797	0.715797	4026.0
weighted avg	0.715711	0.715955	0.715797	4026.0

The result of Model Number:Dataset Number:Featuregroup Number:Rescale of 72 : 0 : 7 : 0

	f1-score	precision	recall	support
AFIB	0.658734	0.661424	0.656086	875.0
GSVT	0.613408	0.640481	0.588633	691.0
SB	0.658764	0.596755	0.735164	1553.0
SR	0.496929	0.618475	0.415477	907.0
macro avg	0.606959	0.629284	0.598840	4026.0
micro avg	0.620807	0.620807	0.620807	4026.0
weighted avg	0.614514	0.623208	0.620807	4026.0

The result of Model Number:Dataset Number:Featuregroup Number:Rescale of 72 : 0 : 7 : 1

	f1-score	precision	recall	support
AFIB	0.854040	0.874534	0.834873	204.0
GSVT	0.884407	0.879281	0.889700	300.0
SB	0.903296	0.899982	0.906636	892.0
SR	0.895446	0.896110	0.894793	681.0
macro avg	0.884297	0.887477	0.881501	2077.0
micro avg	0.893258	0.893258	0.893258	2077.0
weighted avg	0.893156	0.893223	0.893258	2077.0

The result of Model Number:Dataset Number:Featuregroup Number:Rescale of 72 : 0 : 8 : 0

	f1-score	precision	recall	support
AFIB	0.764778	0.754536	0.775351	875.0
GSVT	0.781877	0.787980	0.775882	691.0
SB	0.830136	0.827539	0.832754	1553.0
SR	0.810061	0.820800	0.799614	907.0
macro avg	0.796713	0.797714	0.795900	4026.0
micro avg	0.803051	0.803051	0.803051	4026.0
weighted avg	0.803126	0.803365	0.803051	4026.0

The result of Model Number:Dataset Number:Featuregroup Number:Rescale of 72 : 0 : 8 : 1

	f1-score	precision	recall	support
AFIB	0.827483	0.854962	0.802028	204.0
GSVT	0.858886	0.851995	0.866018	300.0
SB	0.877862	0.874255	0.881502	892.0
SR	0.869663	0.869536	0.869791	681.0
macro avg	0.858473	0.862687	0.854835	2077.0
micro avg	0.867620	0.867620	0.867620	2077.0
weighted avg	0.867485	0.867597	0.867620	2077.0

The result of Model Number:Dataset Number:Featuregroup Number:Rescale of 72 : 0 : 9 : 0

	f1-score	precision	recall	support
AFIB	0.899958	0.916151	0.884593	204.0
GSVT	0.920261	0.907322	0.933727	300.0
SB	0.934420	0.923380	0.945733	892.0
SR	0.915628	0.931929	0.899897	681.0
macro avg	0.917567	0.919695	0.915987	2077.0
micro avg	0.922965	0.922965	0.922965	2077.0
weighted avg	0.922829	0.923154	0.922965	2077.0

The result of Model Number:Dataset Number:Featuregroup Number:Rescale of 72 : 0 : 9 : 1

	f1-score	precision	recall	support
AFIB	0.808022	0.806455	0.809699	875.0
GSVT	0.823194	0.808791	0.838199	691.0
SB	0.881874	0.869361	0.894761	1553.0
SR	0.835367	0.872607	0.801212	907.0
macro avg	0.837114	0.839304	0.835967	4026.0
micro avg	0.845490	0.845490	0.845490	4026.0
weighted avg	0.845274	0.846025	0.845490	4026.0

The result of Model Number:Dataset Number:Featuregroup Number:Rescale of 72 : 0 : 10 : 0

	f1-score	precision	recall	support
AFIB	0.792213	0.782869	0.801815	875.0
GSVT	0.807780	0.814805	0.800882	691.0
SB	0.860894	0.857599	0.864218	1553.0

SR	0.840582	0.850745	0.830668	907.0
macro avg	0.825367	0.826504	0.824396	4026.0
micro avg	0.832226	0.832226	0.832226	4026.0
weighted avg	0.832275	0.832468	0.832226	4026.0

The result of Model Number:Dataset Number:Featuregroup Number:Rescale of 72 : 0 : 10 : 1

	f1-score	precision	recall	support
AFIB	0.843791	0.833197	0.854903	875.0
GSVT	0.782871	0.806574	0.763139	691.0
SB	0.842885	0.780698	0.918066	1553.0
SR	0.655370	0.802123	0.558848	907.0
macro avg	0.781229	0.805648	0.773739	4026.0
micro avg	0.796821	0.796821	0.796821	4026.0
weighted avg	0.790537	0.801376	0.796821	4026.0

The result of Model Number:Dataset Number:Featuregroup Number:Rescale of 72 : 1 : 0 : 0

	f1-score	precision	recall	support
AFIB	0.822180	0.830217	0.814489	875.0
GSVT	0.815900	0.809709	0.822454	691.0
SB	0.863314	0.854432	0.872460	1553.0
SR	0.835912	0.849355	0.823123	907.0
macro avg	0.834326	0.835928	0.833131	4026.0
micro avg	0.840163	0.840163	0.840163	4026.0
weighted avg	0.840063	0.840349	0.840163	4026.0

The result of Model Number:Dataset Number:Featuregroup Number:Rescale of 72 : 1 : 0 : 1

	f1-score	precision	recall	support
AFIB	0.800752	0.793670	0.807995	875.0
GSVT	0.728023	0.705035	0.809940	691.0
SB	0.656352	0.630188	0.697267	1553.0
SR	0.676466	0.683465	0.669652	907.0
macro avg	0.715398	0.703090	0.746213	4026.0
micro avg	0.734449	0.734449	0.734449	4026.0
weighted avg	0.704568	0.690568	0.734449	4026.0

The result of Model Number:Dataset Number:Featuregroup Number:Rescale of 72 : 1 : 1 : 0

	f1-score	precision	recall	support
AFIB	0.774919	0.756859	0.793988	875.0
GSVT	0.743895	0.752072	0.735944	691.0
SB	0.782831	0.732280	0.840896	1553.0
SR	0.626534	0.752346	0.537106	907.0
macro avg	0.732045	0.748389	0.726984	4026.0
micro avg	0.744248	0.744248	0.744248	4026.0
weighted avg	0.739217	0.745540	0.744248	4026.0

The result of Model Number:Dataset Number:Featuregroup Number:Rescale of 72 : 1 : 1 : 1

	f1-score	precision	recall	support
AFIB	0.810537	0.812792	0.808337	875.0
GSVT	0.829104	0.828874	0.829343	691.0
SB	0.883022	0.874723	0.891491	1553.0
SR	0.859625	0.872021	0.847586	907.0
macro avg	0.845572	0.847103	0.844189	4026.0
micro avg	0.852860	0.852860	0.852860	4026.0
weighted avg	0.852743	0.852785	0.852860	4026.0

The result of Model Number:Dataset Number:Featuregroup Number:Rescale of 72 : 1 : 2 : 0

	f1-score	precision	recall	support
AFIB	0.746238	0.762630	0.730815	204.0
GSVT	0.770288	0.764062	0.776721	300.0
SB	0.786431	0.785464	0.787402	892.0
SR	0.779792	0.779114	0.780477	681.0
macro avg	0.770687	0.772818	0.768854	2077.0
micro avg	0.778031	0.778031	0.778031	2077.0
weighted avg	0.777975	0.778048	0.778031	2077.0

The result of Model Number:Dataset Number:Featuregroup Number:Rescale of 72 : 1 : 2 : 1

	f1-score	precision	recall	support
AFIB	0.734191	0.731814	0.736606	875.0
GSVT	0.742704	0.745012	0.740485	691.0
SB	0.783674	0.781968	0.785390	1553.0
SR	0.769222	0.772851	0.765656	907.0
macro avg	0.757448	0.757911	0.757034	4026.0
micro avg	0.762634	0.762634	0.762634	4026.0
weighted avg	0.762632	0.762671	0.762634	4026.0

The result of Model Number:Dataset Number:Featuregroup Number:Rescale of 72 : 1 : 3 : 0

	f1-score	precision	recall	support
AFIB	0.685762	0.680307	0.691371	875.0
GSVT	0.658571	0.646050	0.671643	691.0
SB	0.691195	0.638033	0.754032	1553.0
SR	0.529263	0.676740	0.434660	907.0
macro avg	0.641198	0.660282	0.637927	4026.0
micro avg	0.654323	0.654323	0.654323	4026.0
weighted avg	0.647934	0.657317	0.654323	4026.0

The result of Model Number:Dataset Number:Featuregroup Number:Rescale of 72 : 1 : 3 : 1

	f1-score	precision	recall	support
AFIB	0.798115	0.804365	0.792003	875.0

GSVT	0.800220	0.790007	0.810749	691.0
SB	0.839340	0.835974	0.842742	1553.0
SR	0.821607	0.829672	0.813728	907.0
macro avg	0.814820	0.815004	0.814806	4026.0
micro avg	0.819687	0.819687	0.819687	4026.0
weighted avg	0.819671	0.819795	0.819687	4026.0

The result of Model Number:Dataset Number:Featuregroup Number:Rescale of 72 : 1 : 4 : 0

	f1-score	precision	recall	support
AFIB	0.807393	0.805680	0.809150	875.0
GSVT	0.820007	0.816076	0.824044	691.0
SB	0.863054	0.852751	0.873611	1553.0
SR	0.822946	0.846261	0.800898	907.0
macro avg	0.828350	0.830192	0.826926	4026.0
micro avg	0.834713	0.834713	0.834713	4026.0
weighted avg	0.834532	0.834764	0.834713	4026.0

The result of Model Number:Dataset Number:Featuregroup Number:Rescale of 72 : 1 : 4 : 1

	f1-score	precision	recall	support
AFIB	0.734516	0.725155	0.744147	875.0
GSVT	0.701329	0.706155	0.696587	691.0
SB	0.742582	0.699671	0.791117	1553.0
SR	0.605601	0.700664	0.533286	907.0
macro avg	0.696007	0.707911	0.691284	4026.0
micro avg	0.706598	0.706598	0.706598	4026.0
weighted avg	0.702889	0.706546	0.706598	4026.0

The result of Model Number:Dataset Number:Featuregroup Number:Rescale of 72 : 1 : 5 : 0

	f1-score	precision	recall	support
AFIB	0.819098	0.795499	0.898708	875.0
GSVT	0.902756	0.899873	0.905774	691.0
SB	0.762059	0.750828	0.773693	1553.0
SR	0.912475	0.930285	0.895448	907.0
macro avg	0.849097	0.844121	0.868406	4026.0
micro avg	0.850963	0.850963	0.850963	4026.0
weighted avg	0.832491	0.826547	0.850963	4026.0

The result of Model Number:Dataset Number:Featuregroup Number:Rescale of 72 : 1 : 5 : 1

	f1-score	precision	recall	support
AFIB	0.726564	0.810345	0.658580	204.0
GSVT	0.856693	0.841782	0.872325	300.0
SB	0.914684	0.910629	0.918784	892.0
SR	0.893457	0.881241	0.906044	681.0
macro avg	0.847850	0.860999	0.838933	2077.0

micro avg 0.882339 0.882339 0.882339 2077.0
weighted avg 0.880871 0.881199 0.882339 2077.0
The result of Model Number:Dataset Number:Featuregroup Number:Rescale of 72 : 1 : 6 : 0

	f1-score	precision	recall	support
AFIB	0.699633	0.733129	0.669228	204.0
GSVT	0.736902	0.729013	0.744990	300.0
SB	0.752554	0.748397	0.756768	892.0
SR	0.744053	0.743408	0.744712	681.0
macro avg	0.733285	0.738487	0.728925	2077.0
micro avg	0.742516	0.742516	0.742516	2077.0
weighted avg	0.742308	0.742462	0.742516	2077.0

The result of Model Number:Dataset Number:Featuregroup Number:Rescale of 72 : 1 : 6 : 1

	f1-score	precision	recall	support
AFIB	0.646140	0.641165	0.651243	875.0
GSVT	0.654529	0.657568	0.651661	691.0
SB	0.701419	0.697688	0.705206	1553.0
SR	0.680753	0.690324	0.671519	907.0
macro avg	0.670711	0.671686	0.669907	4026.0
micro avg	0.676698	0.676698	0.676698	4026.0
weighted avg	0.676701	0.676859	0.676698	4026.0

The result of Model Number:Dataset Number:Featuregroup Number:Rescale of 72 : 1 : 7 : 0

	f1-score	precision	recall	support
AFIB	0.775811	0.767429	0.883131	204.0
GSVT	0.928208	0.920976	0.935634	300.0
SB	0.757506	0.755922	0.759098	892.0
SR	0.939324	0.939061	0.939600	681.0
macro avg	0.850212	0.845847	0.879366	2077.0
micro avg	0.855961	0.855961	0.855961	2077.0
weighted avg	0.843574	0.840939	0.855961	2077.0

The result of Model Number:Dataset Number:Featuregroup Number:Rescale of 72 : 1 : 7 : 1

	f1-score	precision	recall	support
AFIB	0.711911	0.713349	0.710495	204.0
GSVT	0.731717	0.729542	0.733910	300.0
SB	0.743842	0.743178	0.744508	892.0
SR	0.737448	0.738864	0.736047	681.0
macro avg	0.731229	0.731233	0.731240	2077.0
micro avg	0.736862	0.736862	0.736862	2077.0
weighted avg	0.736858	0.736864	0.736862	2077.0

The result of Model Number:Dataset Number:Featuregroup Number:Rescale of 72 : 1 : 8 : 0

	f1-score	precision	recall	support
AFIB	0.761740	0.768517	0.755121	875.0
GSVT	0.777728	0.769194	0.786504	691.0
SB	0.836442	0.829930	0.843065	1553.0
SR	0.808646	0.820108	0.797503	907.0
macro avg	0.796139	0.796937	0.795548	4026.0
micro avg	0.803979	0.803979	0.803979	4026.0
weighted avg	0.803867	0.803945	0.803979	4026.0

The result of Model Number:Dataset Number:Featuregroup Number:Rescale of 72 : 1 : 8 : 1

	f1-score	precision	recall	support
AFIB	0.694700	0.705981	0.683811	875.0
GSVT	0.738834	0.741149	0.736577	691.0
SB	0.803814	0.801044	0.806607	1553.0
SR	0.760977	0.752502	0.769693	907.0
macro avg	0.749581	0.750169	0.749172	4026.0
micro avg	0.759583	0.759583	0.759583	4026.0
weighted avg	0.759296	0.759167	0.759583	4026.0

The result of Model Number:Dataset Number:Featuregroup Number:Rescale of 72 : 1 : 9 : 0

	f1-score	precision	recall	support
AFIB	0.814307	0.817632	0.811034	875.0
GSVT	0.758276	0.791742	0.727650	691.0
SB	0.814344	0.737690	0.908787	1553.0
SR	0.614288	0.764539	0.513600	907.0
macro avg	0.750304	0.777901	0.740268	4026.0
micro avg	0.767422	0.767422	0.767422	4026.0
weighted avg	0.759643	0.770390	0.767422	4026.0

The result of Model Number:Dataset Number:Featuregroup Number:Rescale of 72 : 1 : 9 : 1

	f1-score	precision	recall	support
AFIB	0.786719	0.784445	0.789078	875.0
GSVT	0.801300	0.800648	0.802008	691.0
SB	0.851097	0.847572	0.854658	1553.0
SR	0.825546	0.834615	0.816716	907.0
macro avg	0.816165	0.816820	0.815615	4026.0
micro avg	0.822821	0.822821	0.822821	4026.0
weighted avg	0.822802	0.822879	0.822821	4026.0

The result of Model Number:Dataset Number:Featuregroup Number:Rescale of 72 : 1 : 10 : 0

	f1-score	precision	recall	support
AFIB	0.634776	0.639053	0.630650	875.0
GSVT	0.646105	0.637131	0.655454	691.0
SB	0.688495	0.683781	0.693281	1553.0

SR 0.660594 0.672005 0.649601 907.0
macro avg 0.657493 0.657992 0.657247 4026.0
micro avg 0.663336 0.663336 0.663336 4026.0
weighted avg 0.663259 0.663400 0.663336 4026.0
The result of Model Number:Dataset Number:Featuregroup Number:Rescale of 72 : 1 : 10 : 1

	f1-score	precision	recall	support
AFIB	0.698861	0.718043	0.680729	204.0
GSVT	0.714668	0.703688	0.726063	300.0
SB	0.730370	0.723305	0.737575	892.0
SR	0.711910	0.720806	0.703236	681.0
macro avg	0.713952	0.716461	0.711901	2077.0
micro avg	0.719070	0.719070	0.719070	2077.0
weighted avg	0.718955	0.719135	0.719070	2077.0

The result of Model Number:Dataset Number:Featuregroup Number:Rescale of 73 : 0 : 0 : 0

	f1-score	precision	recall	support
AFIB	0.831257	0.843341	0.819891	204.0
GSVT	0.849590	0.838096	0.861472	300.0
SB	0.862376	0.853389	0.871561	892.0
SR	0.845284	0.859272	0.831759	681.0
macro avg	0.847127	0.848524	0.846171	2077.0
micro avg	0.851979	0.851979	0.851979	2077.0
weighted avg	0.851869	0.852122	0.851979	2077.0

The result of Model Number:Dataset Number:Featuregroup Number:Rescale of 73 : 0 : 0 : 1

	f1-score	precision	recall	support
AFIB	0.895032	0.920220	0.871370	204.0
GSVT	0.918171	0.911726	0.924881	300.0
SB	0.943421	0.939646	0.947228	892.0
SR	0.930881	0.931463	0.930327	681.0
macro avg	0.921876	0.925764	0.918451	2077.0
micro avg	0.931008	0.931008	0.931008	2077.0
weighted avg	0.930909	0.931022	0.931008	2077.0

The result of Model Number:Dataset Number:Featuregroup Number:Rescale of 73 : 0 : 1 : 0

	f1-score	precision	recall	support
AFIB	0.631933	0.646816	0.617775	875.0
GSVT	0.666152	0.667834	0.664521	691.0
SB	0.724452	0.721449	0.727483	1553.0
SR	0.689320	0.678464	0.700562	907.0
macro avg	0.677964	0.678641	0.677585	4026.0
micro avg	0.686768	0.686768	0.686768	4026.0
weighted avg	0.686423	0.686342	0.686768	4026.0

The result of Model Number:Dataset Number:Featuregroup Number:Rescale of 73 : 0 : 1 : 1

	f1-score	precision	recall	support
AFIB	0.887279	0.879161	0.895587	875.0
GSVT	0.898648	0.906577	0.890991	691.0
SB	0.955234	0.950198	0.960337	1553.0
SR	0.933640	0.944891	0.922738	907.0
macro avg	0.918700	0.920207	0.917413	4026.0
micro avg	0.925892	0.925892	0.925892	4026.0
weighted avg	0.925888	0.926077	0.925892	4026.0

The result of Model Number:Dataset Number:Featuregroup Number:Rescale of 73 : 0 : 2 : 0

	f1-score	precision	recall	support
AFIB	0.706704	0.725805	0.688730	204.0
GSVT	0.712529	0.702040	0.723434	300.0
SB	0.726331	0.718604	0.734229	892.0
SR	0.712860	0.722655	0.703338	681.0
macro avg	0.714606	0.717276	0.712433	2077.0
micro avg	0.718072	0.718072	0.718072	2077.0
weighted avg	0.717993	0.718247	0.718072	2077.0

The result of Model Number:Dataset Number:Featuregroup Number:Rescale of 73 : 0 : 2 : 1

	f1-score	precision	recall	support
AFIB	0.663451	0.666738	0.660266	875.0
GSVT	0.670056	0.671022	0.669195	691.0
SB	0.705325	0.702052	0.708636	1553.0
SR	0.692341	0.694074	0.690654	907.0
macro avg	0.682793	0.683472	0.682188	4026.0
micro avg	0.687303	0.687303	0.687303	4026.0
weighted avg	0.687246	0.687254	0.687303	4026.0

The result of Model Number:Dataset Number:Featuregroup Number:Rescale of 73 : 0 : 3 : 0

	f1-score	precision	recall	support
AFIB	0.881221	0.897575	0.865671	204.0
GSVT	0.900598	0.895876	0.905389	300.0
SB	0.914976	0.911724	0.918257	892.0
SR	0.905556	0.907188	0.903969	681.0
macro avg	0.900588	0.903091	0.898322	2077.0
micro avg	0.906549	0.906549	0.906549	2077.0
weighted avg	0.906496	0.906558	0.906549	2077.0

The result of Model Number:Dataset Number:Featuregroup Number:Rescale of 73 : 0 : 3 : 1

	f1-score	precision	recall	support
AFIB	0.719721	0.711769	0.727867	875.0

	f1-score	precision	recall	support
GSVT	0.732689	0.737200	0.728245	691.0
SB	0.779807	0.778509	0.781112	1553.0
SR	0.760819	0.767933	0.753855	907.0
macro avg	0.748259	0.748853	0.747770	4026.0
micro avg	0.754326	0.754326	0.754326	4026.0
weighted avg	0.754383	0.754531	0.754326	4026.0

The result of Model Number:Dataset Number:Featuregroup Number:Rescale of 73 : 0 : 4 : 0

	f1-score	precision	recall	support
AFIB	0.799726	0.859844	0.748299	204.0
GSVT	0.762675	0.786883	0.740027	300.0
SB	0.794599	0.755279	0.838244	892.0
SR	0.703399	0.733632	0.675630	681.0
macro avg	0.765100	0.783910	0.750550	2077.0
micro avg	0.761906	0.761906	0.761906	2077.0
weighted avg	0.760589	0.763017	0.761906	2077.0

The result of Model Number:Dataset Number:Featuregroup Number:Rescale of 73 : 0 : 4 : 1

	f1-score	precision	recall	support
AFIB	0.708819	0.724388	0.694169	204.0
GSVT	0.731662	0.725748	0.737773	300.0
SB	0.746996	0.746077	0.747918	892.0
SR	0.740690	0.740046	0.741340	681.0
macro avg	0.732042	0.734065	0.730300	2077.0
micro avg	0.739017	0.739017	0.739017	2077.0
weighted avg	0.738964	0.739033	0.739017	2077.0

The result of Model Number:Dataset Number:Featuregroup Number:Rescale of 73 : 0 : 5 : 0

	f1-score	precision	recall	support
AFIB	0.879263	0.892180	0.866794	204.0
GSVT	0.901540	0.893876	0.909356	300.0
SB	0.917121	0.913452	0.920822	892.0
SR	0.908051	0.912508	0.903648	681.0
macro avg	0.901494	0.903004	0.900155	2077.0
micro avg	0.908229	0.908229	0.908229	2077.0
weighted avg	0.908179	0.908226	0.908229	2077.0

The result of Model Number:Dataset Number:Featuregroup Number:Rescale of 73 : 0 : 5 : 1

	f1-score	precision	recall	support
AFIB	0.644781	0.653062	0.636739	875.0
GSVT	0.651914	0.646642	0.657377	691.0
SB	0.697361	0.695265	0.699472	1553.0
SR	0.680093	0.679807	0.680388	907.0
macro avg	0.668537	0.668694	0.668494	4026.0

micro avg 0.674314 0.674314 0.674314 4026.0
weighted avg 0.674243 0.674265 0.674314 4026.0
The result of Model Number:Dataset Number:Featuregroup Number:Rescale of 73 : 0 : 6 : 0

	f1-score	precision	recall	support
AFIB	0.809740	0.809341	0.810201	875.0
GSVT	0.827317	0.823247	0.831480	691.0
SB	0.865389	0.859731	0.871127	1553.0
SR	0.840754	0.854449	0.827513	907.0
macro avg	0.835800	0.836692	0.835080	4026.0
micro avg	0.841255	0.841255	0.841255	4026.0
weighted avg	0.841210	0.841328	0.841255	4026.0

The result of Model Number:Dataset Number:Featuregroup Number:Rescale of 73 : 0 : 6 : 1

	f1-score	precision	recall	support
AFIB	0.641501	0.704390	0.589548	204.0
GSVT	0.752650	0.742505	0.763196	300.0
SB	0.799079	0.796413	0.801765	892.0
SR	0.778312	0.767877	0.789054	681.0
macro avg	0.742885	0.752796	0.735891	2077.0
micro avg	0.771183	0.771183	0.771183	2077.0
weighted avg	0.770086	0.770232	0.771183	2077.0

The result of Model Number:Dataset Number:Featuregroup Number:Rescale of 73 : 0 : 7 : 0

	f1-score	precision	recall	support
AFIB	0.905300	0.941840	0.871561	204.0
GSVT	0.938989	0.923735	0.954916	300.0
SB	0.963281	0.955536	0.971164	892.0
SR	0.937365	0.944115	0.930760	681.0
macro avg	0.936234	0.941307	0.932100	2077.0
micro avg	0.945787	0.945787	0.945787	2077.0
weighted avg	0.945580	0.945853	0.945787	2077.0

The result of Model Number:Dataset Number:Featuregroup Number:Rescale of 73 : 0 : 7 : 1

	f1-score	precision	recall	support
AFIB	0.688320	0.682232	0.694546	875.0
GSVT	0.625802	0.606042	0.696217	691.0
SB	0.564195	0.541705	0.599365	1553.0
SR	0.581484	0.587501	0.575627	907.0
macro avg	0.614950	0.604370	0.641439	4026.0
micro avg	0.631327	0.631327	0.631327	4026.0
weighted avg	0.605641	0.593606	0.631327	4026.0

The result of Model Number:Dataset Number:Featuregroup Number:Rescale of 73 : 0 : 8 : 0

	f1-score	precision	recall	support
AFIB	0.750834	0.789625	0.715900	204.0
GSVT	0.787314	0.779618	0.795180	300.0
SB	0.801196	0.794750	0.807751	892.0
SR	0.791300	0.792263	0.790371	681.0
macro avg	0.782661	0.789064	0.777301	2077.0
micro avg	0.791215	0.791215	0.791215	2077.0
weighted avg	0.791000	0.791246	0.791215	2077.0

The result of Model Number:Dataset Number:Featuregroup Number:Rescale of 73 : 0 : 8 : 1

	f1-score	precision	recall	support
AFIB	0.738087	0.799702	0.686758	204.0
GSVT	0.791684	0.781729	0.802218	300.0
SB	0.811894	0.800520	0.823691	892.0
SR	0.789747	0.793447	0.786467	681.0
macro avg	0.782853	0.793850	0.774784	2077.0
micro avg	0.794935	0.794935	0.794935	2077.0
weighted avg	0.794464	0.795407	0.794935	2077.0

The result of Model Number:Dataset Number:Featuregroup Number:Rescale of 73 : 0 : 9 : 0

	f1-score	precision	recall	support
AFIB	0.828515	0.884674	0.779612	204.0
GSVT	0.780657	0.803820	0.759086	300.0
SB	0.815982	0.776811	0.859778	892.0
SR	0.724034	0.756905	0.694687	681.0
macro avg	0.787297	0.805552	0.773291	2077.0
micro avg	0.783231	0.783231	0.783231	2077.0
weighted avg	0.781963	0.784780	0.783231	2077.0

The result of Model Number:Dataset Number:Featuregroup Number:Rescale of 73 : 0 : 9 : 1

	f1-score	precision	recall	support
AFIB	0.647636	0.639997	0.655485	875.0
GSVT	0.660362	0.666105	0.654723	691.0
SB	0.703783	0.701089	0.706500	1553.0
SR	0.687177	0.695486	0.679073	907.0
macro avg	0.674739	0.675669	0.673945	4026.0
micro avg	0.680347	0.680347	0.680347	4026.0
weighted avg	0.680386	0.680545	0.680347	4026.0

The result of Model Number:Dataset Number:Featuregroup Number:Rescale of 73 : 0 : 10 : 0

	f1-score	precision	recall	support
AFIB	0.661427	0.657102	0.665867	875.0
GSVT	0.681086	0.686881	0.675479	691.0
SB	0.725108	0.723529	0.726696	1553.0

SR 0.707437 0.710277 0.704629 907.0
macro avg 0.693764 0.694447 0.693168 4026.0
micro avg 0.699713 0.699713 0.699713 4026.0
weighted avg 0.699731 0.699816 0.699713 4026.0
The result of Model Number:Dataset Number:Featuregroup Number:Rescale of 73 : 0 : 10 : 1

	f1-score	precision	recall	support
AFIB	0.715577	0.729363	0.702641	204.0
GSVT	0.729015	0.717169	0.741390	300.0
SB	0.741186	0.732346	0.750243	892.0
SR	0.727398	0.741035	0.714265	681.0
macro avg	0.728294	0.729979	0.727135	2077.0
micro avg	0.732493	0.732493	0.732493	2077.0
weighted avg	0.732392	0.732710	0.732493	2077.0

The result of Model Number:Dataset Number:Featuregroup Number:Rescale of 73 : 1 : 0 : 0

	f1-score	precision	recall	support
AFIB	0.697989	0.699760	0.696251	875.0
GSVT	0.710306	0.702612	0.718200	691.0
SB	0.748624	0.740155	0.757299	1553.0
SR	0.714395	0.733953	0.695881	907.0
macro avg	0.717828	0.719120	0.716908	4026.0
micro avg	0.723484	0.723484	0.723484	4026.0
weighted avg	0.723331	0.723535	0.723484	4026.0

The result of Model Number:Dataset Number:Featuregroup Number:Rescale of 73 : 1 : 0 : 1

	f1-score	precision	recall	support
AFIB	0.914063	0.937603	0.891712	204.0
GSVT	0.838812	0.801473	0.946599	300.0
SB	0.962360	0.960108	0.964624	892.0
SR	0.763458	0.763601	0.763324	681.0
macro avg	0.869673	0.865696	0.891565	2077.0
micro avg	0.888858	0.888858	0.888858	2077.0
weighted avg	0.874556	0.870555	0.888858	2077.0

The result of Model Number:Dataset Number:Featuregroup Number:Rescale of 73 : 1 : 1 : 0

	f1-score	precision	recall	support
AFIB	0.705533	0.699225	0.711987	875.0
GSVT	0.716861	0.719697	0.714073	691.0
SB	0.764327	0.762908	0.765754	1553.0
SR	0.744959	0.751845	0.738225	907.0
macro avg	0.732920	0.733419	0.732510	4026.0
micro avg	0.738996	0.738996	0.738996	4026.0
weighted avg	0.739039	0.739159	0.738996	4026.0

The result of Model Number:Dataset Number:Featuregroup Number:Rescale of 73 : 1 : 1 : 1

	f1-score	precision	recall	support
AFIB	0.801185	0.811126	0.791753	204.0
GSVT	0.815551	0.809978	0.821286	300.0
SB	0.834864	0.833560	0.836173	892.0
SR	0.828867	0.830211	0.827534	681.0
macro avg	0.820116	0.821219	0.819187	2077.0
micro avg	0.826827	0.826827	0.826827	2077.0
weighted avg	0.826800	0.826852	0.826827	2077.0

The result of Model Number:Dataset Number:Featuregroup Number:Rescale of 73 : 1 : 2 : 0

	f1-score	precision	recall	support
AFIB	0.689693	0.703058	0.677137	204.0
GSVT	0.716438	0.710448	0.722662	300.0
SB	0.729806	0.728342	0.731279	892.0
SR	0.722847	0.723596	0.722103	681.0
macro avg	0.714696	0.716361	0.713295	2077.0
micro avg	0.721708	0.721708	0.721708	2077.0
weighted avg	0.721654	0.721718	0.721708	2077.0

The result of Model Number:Dataset Number:Featuregroup Number:Rescale of 73 : 1 : 2 : 1

	f1-score	precision	recall	support
AFIB	0.900075	0.929965	0.872388	204.0
GSVT	0.934233	0.926738	0.941991	300.0
SB	0.954874	0.950951	0.958833	892.0
SR	0.945956	0.945817	0.946095	681.0
macro avg	0.933785	0.938368	0.929827	2077.0
micro avg	0.943733	0.943733	0.943733	2077.0
weighted avg	0.943586	0.943709	0.943733	2077.0

The result of Model Number:Dataset Number:Featuregroup Number:Rescale of 73 : 1 : 3 : 0

	f1-score	precision	recall	support
AFIB	0.839529	0.873414	0.808242	204.0
GSVT	0.870771	0.856625	0.885541	300.0
SB	0.893298	0.886116	0.900608	892.0
SR	0.869265	0.875524	0.863140	681.0
macro avg	0.868215	0.872920	0.864383	2077.0
micro avg	0.877075	0.877075	0.877075	2077.0
weighted avg	0.876883	0.877136	0.877075	2077.0

The result of Model Number:Dataset Number:Featuregroup Number:Rescale of 73 : 1 : 3 : 1

	f1-score	precision	recall	support
AFIB	0.830411	0.833268	0.827678	875.0

GSVT 0.846176 0.829294 0.863913 691.0
SB 0.895745 0.890901 0.900647 1553.0
SR 0.861100 0.881121 0.842019 907.0
macro avg 0.858358 0.858646 0.858564 4026.0
micro avg 0.865275 0.865275 0.865275 4026.0
weighted avg 0.865233 0.865598 0.865275 4026.0
The result of Model Number:Dataset Number:Featuregroup Number:Rescale of 73 : 1 : 4 : 0
f1-score precision recall support
AFIB 0.714950 0.728089 0.702365 204.0
GSVT 0.726959 0.722199 0.731803 300.0
SB 0.739394 0.735784 0.743041 892.0
SR 0.731752 0.734794 0.728745 681.0
macro avg 0.728264 0.730216 0.726488 2077.0
micro avg 0.732735 0.732735 0.732735 2077.0
weighted avg 0.732692 0.732741 0.732735 2077.0
The result of Model Number:Dataset Number:Featuregroup Number:Rescale of 73 : 1 : 4 : 1
f1-score precision recall support
AFIB 0.665043 0.649544 0.681408 875.0
GSVT 0.638418 0.645436 0.631594 691.0
SB 0.671833 0.628450 0.721665 1553.0
SR 0.537697 0.645671 0.460950 907.0
macro avg 0.628248 0.642275 0.623904 4026.0
micro avg 0.638721 0.638721 0.638721 4026.0
weighted avg 0.634403 0.639829 0.638721 4026.0
The result of Model Number:Dataset Number:Featuregroup Number:Rescale of 73 : 1 : 5 : 0
f1-score precision recall support
AFIB 0.686979 0.709793 0.665847 204.0
GSVT 0.713050 0.707329 0.718971 300.0
SB 0.728804 0.725810 0.731826 892.0
SR 0.721997 0.721891 0.722104 681.0
macro avg 0.712708 0.716206 0.709687 2077.0
micro avg 0.720301 0.720301 0.720301 2077.0
weighted avg 0.720189 0.720282 0.720301 2077.0
The result of Model Number:Dataset Number:Featuregroup Number:Rescale of 73 : 1 : 5 : 1
f1-score precision recall support
AFIB 0.851453 0.874823 0.829362 204.0
GSVT 0.870711 0.857334 0.884594 300.0
SB 0.889841 0.881234 0.898620 892.0
SR 0.867351 0.878189 0.856783 681.0
macro avg 0.869839 0.872895 0.867340 2077.0

micro avg 0.876074 0.876074 0.876074 2077.0
weighted avg 0.875934 0.876154 0.876074 2077.0
The result of Model Number:Dataset Number:Featuregroup Number:Rescale of 73 : 1 : 6 : 0

	f1-score	precision	recall	support
AFIB	0.748458	0.745539	0.751453	875.0
GSVT	0.762465	0.764073	0.760909	691.0
SB	0.809383	0.807365	0.811415	1553.0
SR	0.790712	0.796023	0.785488	907.0
macro avg	0.777754	0.778250	0.777316	4026.0
micro avg	0.783873	0.783873	0.783873	4026.0
weighted avg	0.783883	0.783942	0.783873	4026.0

The result of Model Number:Dataset Number:Featuregroup Number:Rescale of 73 : 1 : 6 : 1

	f1-score	precision	recall	support
AFIB	0.819886	0.826724	0.813442	204.0
GSVT	0.841545	0.837669	0.845473	300.0
SB	0.854236	0.851201	0.857297	892.0
SR	0.846346	0.850136	0.842633	681.0
macro avg	0.840503	0.841433	0.839711	2077.0
micro avg	0.846474	0.846474	0.846474	2077.0
weighted avg	0.846442	0.846493	0.846474	2077.0

The result of Model Number:Dataset Number:Featuregroup Number:Rescale of 73 : 1 : 7 : 0

	f1-score	precision	recall	support
AFIB	0.770272	0.768779	0.771871	875.0
GSVT	0.784736	0.771006	0.799039	691.0
SB	0.840674	0.828746	0.852959	1553.0
SR	0.796340	0.831840	0.763780	907.0
macro avg	0.798005	0.800093	0.796912	4026.0
micro avg	0.805990	0.805990	0.805990	4026.0
weighted avg	0.805784	0.806500	0.805990	4026.0

The result of Model Number:Dataset Number:Featuregroup Number:Rescale of 73 : 1 : 7 : 1

	f1-score	precision	recall	support
AFIB	0.852728	0.854255	0.851233	875.0
GSVT	0.816544	0.820833	0.812322	691.0
SB	0.863706	0.816194	0.917099	1553.0
SR	0.717311	0.807387	0.645381	907.0
macro avg	0.812572	0.824667	0.806509	4026.0
micro avg	0.823586	0.823586	0.823586	4026.0
weighted avg	0.820245	0.823278	0.823586	4026.0

The result of Model Number:Dataset Number:Featuregroup Number:Rescale of 73 : 1 : 8 : 0

	f1-score	precision	recall	support
AFIB	0.656682	0.651732	0.745325	204.0
GSVT	0.796097	0.791384	0.800875	300.0
SB	0.651097	0.649738	0.652465	892.0
SR	0.805254	0.803965	0.806557	681.0
macro avg	0.727283	0.724205	0.751306	2077.0
micro avg	0.733545	0.733545	0.733545	2077.0
weighted avg	0.723134	0.720960	0.733545	2077.0

The result of Model Number:Dataset Number:Featuregroup Number:Rescale of 73 : 1 : 8 : 1

	f1-score	precision	recall	support
AFIB	0.844021	0.846739	0.841398	875.0
GSVT	0.855887	0.842298	0.869998	691.0
SB	0.901816	0.894286	0.909480	1553.0
SR	0.856484	0.878237	0.835814	907.0
macro avg	0.864552	0.865390	0.864172	4026.0
micro avg	0.871311	0.871311	0.871311	4026.0
weighted avg	0.871159	0.871414	0.871311	4026.0

The result of Model Number:Dataset Number:Featuregroup Number:Rescale of 73 : 1 : 9 : 0

	f1-score	precision	recall	support
AFIB	0.771009	0.768872	0.773215	875.0
GSVT	0.777110	0.780817	0.773516	691.0
SB	0.813498	0.810898	0.816117	1553.0
SR	0.800492	0.804359	0.796700	907.0
macro avg	0.790527	0.791236	0.789887	4026.0
micro avg	0.795107	0.795107	0.795107	4026.0
weighted avg	0.795088	0.795128	0.795107	4026.0

The result of Model Number:Dataset Number:Featuregroup Number:Rescale of 73 : 1 : 9 : 1

	f1-score	precision	recall	support
AFIB	0.793247	0.801690	0.785419	204.0
GSVT	0.816559	0.811118	0.822312	300.0
SB	0.833815	0.833069	0.834562	892.0
SR	0.826784	0.827887	0.825689	681.0
macro avg	0.817601	0.818441	0.816996	2077.0
micro avg	0.825057	0.825057	0.825057	2077.0
weighted avg	0.825032	0.825118	0.825057	2077.0

The result of Model Number:Dataset Number:Featuregroup Number:Rescale of 73 : 1 : 10 : 0

	f1-score	precision	recall	support
AFIB	0.674610	0.678154	0.671143	875.0
GSVT	0.685995	0.687268	0.684813	691.0
SB	0.726562	0.721403	0.731800	1553.0

SR 0.712632 0.717023 0.708311 907.0
macro avg 0.699950 0.700962 0.699017 4026.0
micro avg 0.705261 0.705261 0.705261 4026.0
weighted avg 0.705170 0.705158 0.705261 4026.0
The result of Model Number:Dataset Number:Featuregroup Number:Rescale of 73 : 1 : 10 : 1

	f1-score	precision	recall	support
AFIB	0.821162	0.822086	0.820390	204.0
GSVT	0.840015	0.838140	0.841963	300.0
SB	0.857820	0.856675	0.858973	892.0
SR	0.851395	0.853540	0.849273	681.0
macro avg	0.842598	0.842610	0.842650	2077.0
micro avg	0.849546	0.849546	0.849546	2077.0
weighted avg	0.849541	0.849572	0.849546	2077.0

The result of Model Number:Dataset Number:Featuregroup Number:Rescale of 74 : 0 : 0 : 0

	f1-score	precision	recall	support
AFIB	0.634351	0.634653	0.634140	875.0
GSVT	0.650779	0.645146	0.656534	691.0
SB	0.695035	0.689970	0.700181	1553.0
SR	0.665342	0.678568	0.652656	907.0
macro avg	0.661377	0.662084	0.660878	4026.0
micro avg	0.667630	0.667630	0.667630	4026.0
weighted avg	0.667561	0.667686	0.667630	4026.0

The result of Model Number:Dataset Number:Featuregroup Number:Rescale of 74 : 0 : 0 : 1

	f1-score	precision	recall	support
AFIB	0.628321	0.623968	0.632861	875.0
GSVT	0.638014	0.640603	0.635549	691.0
SB	0.692247	0.685048	0.699601	1553.0
SR	0.657294	0.672589	0.642740	907.0
macro avg	0.653969	0.655552	0.652688	4026.0
micro avg	0.661293	0.661293	0.661293	4026.0
weighted avg	0.661171	0.661338	0.661293	4026.0

The result of Model Number:Dataset Number:Featuregroup Number:Rescale of 74 : 0 : 1 : 0

	f1-score	precision	recall	support
AFIB	0.680854	0.689303	0.672839	204.0
GSVT	0.693063	0.688327	0.697936	300.0
SB	0.709475	0.708367	0.710587	892.0
SR	0.704378	0.705521	0.703246	681.0
macro avg	0.696943	0.697879	0.696152	2077.0
micro avg	0.702646	0.702646	0.702646	2077.0
weighted avg	0.702622	0.702667	0.702646	2077.0

The result of Model Number:Dataset Number:Featuregroup Number:Rescale of 74 : 0 : 1 : 1

	f1-score	precision	recall	support
AFIB	0.743308	0.767450	0.720784	204.0
GSVT	0.763829	0.754090	0.773977	300.0
SB	0.785379	0.782417	0.788372	892.0
SR	0.774394	0.775797	0.773027	681.0
macro avg	0.766728	0.769939	0.764040	2077.0
micro avg	0.774623	0.774623	0.774623	2077.0
weighted avg	0.774533	0.774685	0.774623	2077.0

The result of Model Number:Dataset Number:Featuregroup Number:Rescale of 74 : 0 : 2 : 0

	f1-score	precision	recall	support
AFIB	0.806722	0.815321	0.798434	204.0
GSVT	0.821558	0.817051	0.826206	300.0
SB	0.840522	0.839121	0.841933	892.0
SR	0.833516	0.834873	0.832173	681.0
macro avg	0.825579	0.826592	0.824687	2077.0
micro avg	0.832189	0.832189	0.832189	2077.0
weighted avg	0.832166	0.832203	0.832189	2077.0

The result of Model Number:Dataset Number:Featuregroup Number:Rescale of 74 : 0 : 2 : 1

	f1-score	precision	recall	support
AFIB	0.730810	0.742391	0.719682	204.0
GSVT	0.749594	0.743215	0.756088	300.0
SB	0.762536	0.760837	0.764246	892.0
SR	0.755311	0.756990	0.753654	681.0
macro avg	0.749563	0.750858	0.748418	2077.0
micro avg	0.755218	0.755218	0.755218	2077.0
weighted avg	0.755182	0.755219	0.755218	2077.0

The result of Model Number:Dataset Number:Featuregroup Number:Rescale of 74 : 0 : 3 : 0

	f1-score	precision	recall	support
AFIB	0.725531	0.735342	0.716282	204.0
GSVT	0.753089	0.751867	0.754342	300.0
SB	0.769444	0.768419	0.770478	892.0
SR	0.761579	0.760603	0.762584	681.0
macro avg	0.752411	0.754058	0.750922	2077.0
micro avg	0.760236	0.760236	0.760236	2077.0
weighted avg	0.760190	0.760217	0.760236	2077.0

The result of Model Number:Dataset Number:Featuregroup Number:Rescale of 74 : 0 : 3 : 1

	f1-score	precision	recall	support
AFIB	0.688432	0.690142	0.686777	875.0

GSVT 0.700288 0.702392 0.698285 691.0
SB 0.742879 0.737837 0.747996 1553.0
SR 0.728743 0.734153 0.723440 907.0
macro avg 0.715086 0.716131 0.714125 4026.0
micro avg 0.720627 0.720627 0.720627 4026.0
weighted avg 0.720551 0.720557 0.720627 4026.0
The result of Model Number:Dataset Number:Featuregroup Number:Rescale of 74 : 0 : 4 : 0

	f1-score	precision	recall	support
AFIB	0.666763	0.710206	0.628623	204.0
GSVT	0.695972	0.678865	0.714059	300.0
SB	0.716384	0.707181	0.725854	892.0
SR	0.697776	0.706108	0.689685	681.0
macro avg	0.694224	0.700590	0.689555	2077.0
micro avg	0.702741	0.702741	0.702741	2077.0
weighted avg	0.702461	0.703037	0.702741	2077.0

The result of Model Number:Dataset Number:Featuregroup Number:Rescale of 74 : 0 : 4 : 1

	f1-score	precision	recall	support
AFIB	0.901865	0.910589	0.893469	204.0
GSVT	0.933778	0.931937	0.935641	300.0
SB	0.953295	0.951607	0.955002	892.0
SR	0.944507	0.944964	0.944090	681.0
macro avg	0.933361	0.934774	0.932051	2077.0
micro avg	0.942584	0.942584	0.942584	2077.0
weighted avg	0.942543	0.942559	0.942584	2077.0

The result of Model Number:Dataset Number:Featuregroup Number:Rescale of 74 : 0 : 5 : 0

	f1-score	precision	recall	support
AFIB	0.731726	0.746338	0.717786	204.0
GSVT	0.739943	0.730698	0.749538	300.0
SB	0.754070	0.746860	0.761422	892.0
SR	0.740376	0.750088	0.730932	681.0
macro avg	0.741529	0.743496	0.739919	2077.0
micro avg	0.745423	0.745423	0.745423	2077.0
weighted avg	0.745345	0.745533	0.745423	2077.0

The result of Model Number:Dataset Number:Featuregroup Number:Rescale of 74 : 0 : 5 : 1

	f1-score	precision	recall	support
AFIB	0.664564	0.663203	0.666413	875.0
GSVT	0.673629	0.666558	0.680973	691.0
SB	0.710453	0.700860	0.720451	1553.0
SR	0.675882	0.701657	0.652087	907.0
macro avg	0.681132	0.683069	0.679981	4026.0

micro avg 0.686529 0.686529 0.686529 4026.0
weighted avg 0.686371 0.686968 0.686529 4026.0
The result of Model Number:Dataset Number:Featuregroup Number:Rescale of 74 : 0 : 6 : 0

	f1-score	precision	recall	support
AFIB	0.664075	0.665406	0.662777	875.0
GSVT	0.665719	0.662125	0.669370	691.0
SB	0.695900	0.692182	0.699663	1553.0
SR	0.679252	0.687307	0.671402	907.0
macro avg	0.676236	0.676755	0.675803	4026.0
micro avg	0.680080	0.680080	0.680080	4026.0
weighted avg	0.680053	0.680106	0.680080	4026.0

The result of Model Number:Dataset Number:Featuregroup Number:Rescale of 74 : 0 : 6 : 1

	f1-score	precision	recall	support
AFIB	0.632005	0.700272	0.575982	204.0
GSVT	0.732629	0.720714	0.745060	300.0
SB	0.781304	0.777747	0.784895	892.0
SR	0.762426	0.752724	0.772392	681.0
macro avg	0.727091	0.737864	0.719582	2077.0
micro avg	0.754523	0.754523	0.754523	2077.0
weighted avg	0.753420	0.753695	0.754523	2077.0

The result of Model Number:Dataset Number:Featuregroup Number:Rescale of 74 : 0 : 7 : 0

	f1-score	precision	recall	support
AFIB	0.703216	0.758025	0.656125	204.0
GSVT	0.724660	0.736927	0.712826	300.0
SB	0.768004	0.760935	0.775228	892.0
SR	0.737436	0.726742	0.748483	681.0
macro avg	0.733329	0.745657	0.723165	2077.0
micro avg	0.745748	0.745748	0.745748	2077.0
weighted avg	0.745357	0.745971	0.745748	2077.0

The result of Model Number:Dataset Number:Featuregroup Number:Rescale of 74 : 0 : 7 : 1

	f1-score	precision	recall	support
AFIB	0.801353	0.820389	0.783255	204.0
GSVT	0.827676	0.819915	0.835671	300.0
SB	0.845451	0.844505	0.846399	892.0
SR	0.837290	0.836449	0.838147	681.0
macro avg	0.827943	0.830315	0.825868	2077.0
micro avg	0.835942	0.835942	0.835942	2077.0
weighted avg	0.835877	0.835943	0.835942	2077.0

The result of Model Number:Dataset Number:Featuregroup Number:Rescale of 74 : 0 : 8 : 0

	f1-score	precision	recall	support
AFIB	0.808998	0.861709	0.762722	204.0
GSVT	0.844439	0.823683	0.866383	300.0
SB	0.869204	0.858039	0.880695	892.0
SR	0.846628	0.856737	0.836811	681.0
macro avg	0.842317	0.850042	0.836653	2077.0
micro avg	0.852652	0.852652	0.852652	2077.0
weighted avg	0.852312	0.853010	0.852652	2077.0

The result of Model Number:Dataset Number:Featuregroup Number:Rescale of 74 : 0 : 8 : 1

	f1-score	precision	recall	support
AFIB	0.669667	0.671367	0.668000	875.0
GSVT	0.681484	0.674102	0.689058	691.0
SB	0.718247	0.710122	0.726571	1553.0
SR	0.685408	0.704172	0.667645	907.0
macro avg	0.688702	0.689941	0.687819	4026.0
micro avg	0.694128	0.694128	0.694128	4026.0
weighted avg	0.693981	0.694177	0.694128	4026.0

The result of Model Number:Dataset Number:Featuregroup Number:Rescale of 74 : 0 : 9 : 0

	f1-score	precision	recall	support
AFIB	0.809921	0.812295	0.807622	875.0
GSVT	0.823322	0.812748	0.834282	691.0
SB	0.871350	0.869232	0.873482	1553.0
SR	0.849286	0.859365	0.839450	907.0
macro avg	0.838470	0.838410	0.838709	4026.0
micro avg	0.844773	0.844773	0.844773	4026.0
weighted avg	0.844785	0.844940	0.844773	4026.0

The result of Model Number:Dataset Number:Featuregroup Number:Rescale of 74 : 0 : 9 : 1

	f1-score	precision	recall	support
AFIB	0.796725	0.808431	0.785428	204.0
GSVT	0.816912	0.809967	0.823994	300.0
SB	0.831031	0.827706	0.834384	892.0
SR	0.822812	0.826850	0.818822	681.0
macro avg	0.816870	0.818238	0.815657	2077.0
micro avg	0.822972	0.822972	0.822972	2077.0
weighted avg	0.822927	0.822970	0.822972	2077.0

The result of Model Number:Dataset Number:Featuregroup Number:Rescale of 74 : 0 : 10 : 0

	f1-score	precision	recall	support
AFIB	0.689219	0.689342	0.689126	875.0
GSVT	0.696511	0.698864	0.694218	691.0
SB	0.731649	0.729126	0.734193	1553.0

SR 0.717755 0.720176 0.715377 907.0
macro avg 0.708783 0.709377 0.708228 4026.0
micro avg 0.713298 0.713298 0.713298 4026.0
weighted avg 0.713266 0.713269 0.713298 4026.0
The result of Model Number:Dataset Number:Featuregroup Number:Rescale of 74 : 0 : 10 : 1

	f1-score	precision	recall	support
AFIB	0.680414	0.681593	0.679249	875.0
GSVT	0.688664	0.680750	0.696820	691.0
SB	0.725187	0.716442	0.734150	1553.0
SR	0.690716	0.711645	0.670999	907.0
macro avg	0.696245	0.697608	0.695305	4026.0
micro avg	0.701584	0.701584	0.701584	4026.0
weighted avg	0.701422	0.701661	0.701584	4026.0

The result of Model Number:Dataset Number:Featuregroup Number:Rescale of 74 : 1 : 0 : 0

	f1-score	precision	recall	support
AFIB	0.788243	0.766380	0.813870	875.0
GSVT	0.798533	0.832168	0.772424	691.0
SB	0.877216	0.876773	0.877671	1553.0
SR	0.855209	0.860561	0.849929	907.0
macro avg	0.829800	0.833971	0.828474	4026.0
micro avg	0.839491	0.839491	0.839491	4026.0
weighted avg	0.839416	0.841473	0.839491	4026.0

The result of Model Number:Dataset Number:Featuregroup Number:Rescale of 74 : 1 : 0 : 1

	f1-score	precision	recall	support
AFIB	0.695443	0.708790	0.682630	875.0
GSVT	0.741320	0.742451	0.740239	691.0
SB	0.807203	0.803655	0.810787	1553.0
SR	0.762782	0.754490	0.771336	907.0
macro avg	0.751687	0.752347	0.751248	4026.0
micro avg	0.761938	0.761938	0.761938	4026.0
weighted avg	0.761598	0.761456	0.761938	4026.0

The result of Model Number:Dataset Number:Featuregroup Number:Rescale of 74 : 1 : 1 : 0

	f1-score	precision	recall	support
AFIB	0.858116	0.860749	0.855634	204.0
GSVT	0.880658	0.878420	0.882986	300.0
SB	0.900331	0.899231	0.901439	892.0
SR	0.892962	0.894679	0.891259	681.0
macro avg	0.883017	0.883270	0.882830	2077.0
micro avg	0.890937	0.890937	0.890937	2077.0
weighted avg	0.890927	0.890953	0.890937	2077.0

The result of Model Number:Dataset Number:Featuregroup Number:Rescale of 74 : 1 : 1 : 1

	f1-score	precision	recall	support
AFIB	0.798306	0.812957	0.784558	204.0
GSVT	0.828611	0.821948	0.835534	300.0
SB	0.843982	0.842475	0.845496	892.0
SR	0.835625	0.836369	0.834887	681.0
macro avg	0.826631	0.828437	0.825119	2077.0
micro avg	0.834593	0.834593	0.834593	2077.0
weighted avg	0.834535	0.834609	0.834593	2077.0

The result of Model Number:Dataset Number:Featuregroup Number:Rescale of 74 : 1 : 2 : 0

	f1-score	precision	recall	support
AFIB	0.640337	0.710643	0.582844	204.0
GSVT	0.747680	0.738333	0.757414	300.0
SB	0.795495	0.791436	0.799597	892.0
SR	0.776860	0.765998	0.788037	681.0
macro avg	0.740093	0.751603	0.731973	2077.0
micro avg	0.768424	0.768424	0.768424	2077.0
weighted avg	0.767239	0.767490	0.768424	2077.0

The result of Model Number:Dataset Number:Featuregroup Number:Rescale of 74 : 1 : 2 : 1

	f1-score	precision	recall	support
AFIB	0.776623	0.800176	0.754450	875.0
GSVT	0.829056	0.823307	0.834931	691.0
SB	0.907089	0.903619	0.910591	1553.0
SR	0.857887	0.845011	0.871212	907.0
macro avg	0.842664	0.843028	0.842796	4026.0
micro avg	0.854799	0.854799	0.854799	4026.0
weighted avg	0.854256	0.854149	0.854799	4026.0

The result of Model Number:Dataset Number:Featuregroup Number:Rescale of 74 : 1 : 3 : 0

	f1-score	precision	recall	support
AFIB	0.690583	0.700770	0.680764	204.0
GSVT	0.707536	0.704759	0.710358	300.0
SB	0.723250	0.721800	0.724709	892.0
SR	0.718120	0.718228	0.718018	681.0
macro avg	0.709873	0.711389	0.708462	2077.0
micro avg	0.716126	0.716126	0.716126	2077.0
weighted avg	0.716090	0.716102	0.716126	2077.0

The result of Model Number:Dataset Number:Featuregroup Number:Rescale of 74 : 1 : 3 : 1

	f1-score	precision	recall	support
AFIB	0.759708	0.777767	0.742553	204.0

GSVT 0.779431 0.776423 0.782543 300.0
SB 0.798745 0.796962 0.800535 892.0
SR 0.788696 0.787109 0.790317 681.0
macro avg 0.781645 0.784565 0.778987 2077.0
micro avg 0.788891 0.788891 0.788891 2077.0
weighted avg 0.788826 0.788880 0.788891 2077.0
The result of Model Number:Dataset Number:Featuregroup Number:Rescale of 74 : 1 : 4 : 0

	f1-score	precision	recall	support
AFIB	0.922107	0.931059	0.913897	204.0
GSVT	0.949748	0.945259	0.954532	300.0
SB	0.968981	0.967900	0.970067	892.0
SR	0.960455	0.961450	0.959467	681.0
macro avg	0.950323	0.951417	0.949491	2077.0
micro avg	0.958831	0.958831	0.958831	2077.0
weighted avg	0.958804	0.958896	0.958831	2077.0

The result of Model Number:Dataset Number:Featuregroup Number:Rescale of 74 : 1 : 4 : 1

	f1-score	precision	recall	support
AFIB	0.736795	0.743922	0.729936	204.0
GSVT	0.762867	0.761363	0.764389	300.0
SB	0.778812	0.777433	0.780207	892.0
SR	0.771632	0.772006	0.771292	681.0
macro avg	0.762527	0.763681	0.761456	2077.0
micro avg	0.770062	0.770062	0.770062	2077.0
weighted avg	0.770028	0.770041	0.770062	2077.0

The result of Model Number:Dataset Number:Featuregroup Number:Rescale of 74 : 1 : 5 : 0

	f1-score	precision	recall	support
AFIB	0.770686	0.797441	0.745772	204.0
GSVT	0.785463	0.776865	0.794351	300.0
SB	0.806373	0.800583	0.812249	892.0
SR	0.791972	0.795866	0.788130	681.0
macro avg	0.788624	0.792689	0.785125	2077.0
micro avg	0.795226	0.795226	0.795226	2077.0
weighted avg	0.795126	0.795302	0.795226	2077.0

The result of Model Number:Dataset Number:Featuregroup Number:Rescale of 74 : 1 : 5 : 1

	f1-score	precision	recall	support
AFIB	0.740490	0.741733	0.758843	204.0
GSVT	0.733528	0.761994	0.708059	300.0
SB	0.778066	0.735396	0.826312	892.0
SR	0.674417	0.736657	0.623583	681.0
macro avg	0.731625	0.743945	0.729199	2077.0

micro avg	0.736135	0.736135	0.736135	2077.0
weighted avg	0.733958	0.740274	0.736135	2077.0

The result of Model Number:Dataset Number:Featuregroup Number:Rescale of 74 : 1 : 6 : 0

	f1-score	precision	recall	support
AFIB	0.658840	0.674356	0.644079	875.0
GSVT	0.694515	0.696269	0.692816	691.0
SB	0.755298	0.752167	0.758458	1553.0
SR	0.718671	0.707352	0.730391	907.0
macro avg	0.706831	0.707536	0.706436	4026.0
micro avg	0.716010	0.716010	0.716010	4026.0
weighted avg	0.715650	0.715566	0.716010	4026.0

The result of Model Number:Dataset Number:Featuregroup Number:Rescale of 74 : 1 : 6 : 1

	f1-score	precision	recall	support
AFIB	0.746414	0.736045	0.757149	875.0
GSVT	0.773087	0.783400	0.763142	691.0
SB	0.821269	0.819586	0.822962	1553.0
SR	0.799894	0.806074	0.793808	907.0
macro avg	0.785166	0.786277	0.784265	4026.0
micro avg	0.791823	0.791823	0.791823	4026.0
weighted avg	0.791915	0.792175	0.791823	4026.0

The result of Model Number:Dataset Number:Featuregroup Number:Rescale of 74 : 1 : 7 : 0

	f1-score	precision	recall	support
AFIB	0.564705	0.625702	0.514647	204.0
GSVT	0.654613	0.643967	0.665721	300.0
SB	0.698105	0.694926	0.701313	892.0
SR	0.681237	0.672568	0.690142	681.0
macro avg	0.649665	0.659291	0.642956	2077.0
micro avg	0.674175	0.674175	0.674175	2077.0
weighted avg	0.673190	0.673436	0.674175	2077.0

The result of Model Number:Dataset Number:Featuregroup Number:Rescale of 74 : 1 : 7 : 1

	f1-score	precision	recall	support
AFIB	0.849094	0.832179	0.866754	875.0
GSVT	0.691087	0.642674	0.866258	691.0
SB	0.560741	0.561707	0.559779	1553.0
SR	0.909033	0.917037	0.901205	907.0
macro avg	0.752489	0.738400	0.798499	4026.0
micro avg	0.756017	0.756017	0.756017	4026.0
weighted avg	0.724248	0.714438	0.756017	4026.0

The result of Model Number:Dataset Number:Featuregroup Number:Rescale of 74 : 1 : 8 : 0

	f1-score	precision	recall	support
AFIB	0.683389	0.693429	0.673698	204.0
GSVT	0.700704	0.694746	0.706778	300.0
SB	0.712814	0.709962	0.715690	892.0
SR	0.705764	0.709228	0.702342	681.0
macro avg	0.700667	0.701841	0.699627	2077.0
micro avg	0.705902	0.705902	0.705902	2077.0
weighted avg	0.705863	0.705900	0.705902	2077.0

The result of Model Number:Dataset Number:Featuregroup Number:Rescale of 74 : 1 : 8 : 1

	f1-score	precision	recall	support
AFIB	0.831263	0.829163	0.833503	875.0
GSVT	0.847481	0.842821	0.852238	691.0
SB	0.887379	0.882029	0.892807	1553.0
SR	0.861215	0.876759	0.846229	907.0
macro avg	0.856835	0.857693	0.856194	4026.0
micro avg	0.862461	0.862461	0.862461	4026.0
weighted avg	0.862441	0.862623	0.862461	4026.0

The result of Model Number:Dataset Number:Featuregroup Number:Rescale of 74 : 1 : 9 : 0

	f1-score	precision	recall	support
AFIB	0.700213	0.713782	0.687465	204.0
GSVT	0.727366	0.721284	0.733685	300.0
SB	0.740938	0.739451	0.742433	892.0
SR	0.733873	0.734633	0.733117	681.0
macro avg	0.725597	0.727287	0.724175	2077.0
micro avg	0.732716	0.732716	0.732716	2077.0
weighted avg	0.732661	0.732726	0.732716	2077.0

The result of Model Number:Dataset Number:Featuregroup Number:Rescale of 74 : 1 : 9 : 1

	f1-score	precision	recall	support
AFIB	0.923607	0.925061	0.922277	204.0
GSVT	0.945944	0.944468	0.947504	300.0
SB	0.965458	0.964280	0.966646	892.0
SR	0.958696	0.960536	0.956868	681.0
macro avg	0.948426	0.948586	0.948323	2077.0
micro avg	0.956317	0.956317	0.956317	2077.0
weighted avg	0.956312	0.956339	0.956317	2077.0

The result of Model Number:Dataset Number:Featuregroup Number:Rescale of 74 : 1 : 10 : 0

	f1-score	precision	recall	support
AFIB	0.836906	0.831434	0.842525	875.0
GSVT	0.861781	0.869113	0.854686	691.0
SB	0.917482	0.915484	0.919491	1553.0

SR 0.895123 0.898716 0.891570 907.0
macro avg 0.877823 0.878687 0.877068 4026.0
micro avg 0.885350 0.885350 0.885350 4026.0
weighted avg 0.885373 0.885480 0.885350 4026.0
The result of Model Number:Dataset Number:Featuregroup Number:Rescale of 74 : 1 : 10 : 1

	f1-score	precision	recall	support
AFIB	0.820463	0.865433	0.780480	204.0
GSVT	0.781255	0.808646	0.755755	300.0
SB	0.803597	0.772603	0.837195	892.0
SR	0.715223	0.734627	0.696858	681.0
macro avg	0.780134	0.795327	0.767572	2077.0
micro avg	0.773848	0.773848	0.773848	2077.0
weighted avg	0.773051	0.774475	0.773848	2077.0

The result of Model Number:Dataset Number:Featuregroup Number:Rescale of 75 : 0 : 0 : 0

	f1-score	precision	recall	support
AFIB	0.694514	0.700020	0.689113	875.0
GSVT	0.703319	0.700219	0.706472	691.0
SB	0.747897	0.744941	0.750883	1553.0
SR	0.729519	0.731580	0.727505	907.0
macro avg	0.718813	0.719190	0.718493	4026.0
micro avg	0.724569	0.724569	0.724569	4026.0
weighted avg	0.724504	0.724492	0.724569	4026.0

The result of Model Number:Dataset Number:Featuregroup Number:Rescale of 75 : 0 : 0 : 1

	f1-score	precision	recall	support
AFIB	0.762776	0.748517	0.777670	875.0
GSVT	0.789495	0.801959	0.777511	691.0
SB	0.839594	0.838094	0.841107	1553.0
SR	0.818253	0.826922	0.809769	907.0
macro avg	0.802529	0.803873	0.801514	4026.0
micro avg	0.809345	0.809345	0.809345	4026.0
weighted avg	0.809492	0.809907	0.809345	4026.0

The result of Model Number:Dataset Number:Featuregroup Number:Rescale of 75 : 0 : 1 : 0

	f1-score	precision	recall	support
AFIB	0.724837	0.718356	0.731467	875.0
GSVT	0.736474	0.739388	0.733610	691.0
SB	0.785239	0.783782	0.786705	1553.0
SR	0.765341	0.772416	0.758423	907.0
macro avg	0.752973	0.753485	0.752551	4026.0
micro avg	0.759215	0.759215	0.759215	4026.0
weighted avg	0.759259	0.759382	0.759215	4026.0

The result of Model Number:Dataset Number:Featuregroup Number:Rescale of 75 : 0 : 1 : 1

	f1-score	precision	recall	support
AFIB	0.776239	0.764638	0.788228	875.0
GSVT	0.791122	0.797888	0.784483	691.0
SB	0.845296	0.842385	0.848233	1553.0
SR	0.820555	0.832793	0.808699	907.0
macro avg	0.808303	0.809426	0.807410	4026.0
micro avg	0.815343	0.815343	0.815343	4026.0
weighted avg	0.815416	0.815690	0.815343	4026.0

The result of Model Number:Dataset Number:Featuregroup Number:Rescale of 75 : 0 : 2 : 0

	f1-score	precision	recall	support
AFIB	0.657649	0.712549	0.611914	204.0
GSVT	0.705405	0.696535	0.714791	300.0
SB	0.723413	0.713278	0.733924	892.0
SR	0.703679	0.706976	0.700756	681.0
macro avg	0.697536	0.707334	0.690346	2077.0
micro avg	0.708302	0.708302	0.708302	2077.0
weighted avg	0.707882	0.708722	0.708302	2077.0

The result of Model Number:Dataset Number:Featuregroup Number:Rescale of 75 : 0 : 2 : 1

	f1-score	precision	recall	support
AFIB	0.757946	0.769956	0.746405	204.0
GSVT	0.777428	0.770812	0.784162	300.0
SB	0.790850	0.789088	0.792623	892.0
SR	0.783357	0.785098	0.781639	681.0
macro avg	0.777395	0.778739	0.776207	2077.0
micro avg	0.783260	0.783260	0.783260	2077.0
weighted avg	0.783223	0.783261	0.783260	2077.0

The result of Model Number:Dataset Number:Featuregroup Number:Rescale of 75 : 0 : 3 : 0

	f1-score	precision	recall	support
AFIB	0.750104	0.758684	0.742007	204.0
GSVT	0.767662	0.763119	0.772271	300.0
SB	0.736941	0.709720	0.782891	892.0
SR	0.618177	0.621169	0.615230	681.0
macro avg	0.718221	0.713173	0.728100	2077.0
micro avg	0.722369	0.722369	0.722369	2077.0
weighted avg	0.703731	0.693208	0.722369	2077.0

The result of Model Number:Dataset Number:Featuregroup Number:Rescale of 75 : 0 : 3 : 1

	f1-score	precision	recall	support
AFIB	0.848957	0.839478	0.858662	875.0

GSVT	0.865848	0.873778	0.858073	691.0
SB	0.923466	0.920459	0.926497	1553.0
SR	0.899526	0.908538	0.890695	907.0
macro avg	0.884449	0.885563	0.883482	4026.0
micro avg	0.891944	0.891944	0.891944	4026.0
weighted avg	0.891990	0.892161	0.891944	4026.0

The result of Model Number:Dataset Number:Featuregroup Number:Rescale of 75 : 0 : 4 : 0

	f1-score	precision	recall	support
AFIB	0.830638	0.838729	0.822903	204.0
GSVT	0.849846	0.845944	0.853813	300.0
SB	0.861590	0.858626	0.864581	892.0
SR	0.854314	0.857628	0.851065	681.0
macro avg	0.849097	0.850232	0.848091	2077.0
micro avg	0.854501	0.854501	0.854501	2077.0
weighted avg	0.854468	0.854513	0.854501	2077.0

The result of Model Number:Dataset Number:Featuregroup Number:Rescale of 75 : 0 : 4 : 1

	f1-score	precision	recall	support
AFIB	0.730226	0.821074	0.658122	204.0
GSVT	0.866359	0.847859	0.885723	300.0
SB	0.924855	0.920140	0.929622	892.0
SR	0.904106	0.892657	0.915911	681.0
macro avg	0.856386	0.870432	0.847345	2077.0
micro avg	0.892120	0.892120	0.892120	2077.0
weighted avg	0.890486	0.890959	0.892120	2077.0

The result of Model Number:Dataset Number:Featuregroup Number:Rescale of 75 : 0 : 5 : 0

	f1-score	precision	recall	support
AFIB	0.673511	0.674678	0.672358	875.0
GSVT	0.681677	0.673844	0.689751	691.0
SB	0.717830	0.709174	0.726702	1553.0
SR	0.683709	0.704426	0.664192	907.0
macro avg	0.689182	0.690531	0.688251	4026.0
micro avg	0.694466	0.694466	0.694466	4026.0
weighted avg	0.694306	0.694543	0.694466	4026.0

The result of Model Number:Dataset Number:Featuregroup Number:Rescale of 75 : 0 : 5 : 1

	f1-score	precision	recall	support
AFIB	0.757774	0.771408	0.744836	204.0
GSVT	0.774869	0.763974	0.786208	300.0
SB	0.786791	0.777496	0.796317	892.0
SR	0.770968	0.784694	0.757722	681.0
macro avg	0.772600	0.774393	0.771271	2077.0

micro avg 0.777146 0.777146 0.777146 2077.0
weighted avg 0.777031 0.777305 0.777146 2077.0
The result of Model Number:Dataset Number:Featuregroup Number:Rescale of 75 : 0 : 6 : 0

	f1-score	precision	recall	support
AFIB	0.679719	0.683776	0.675728	204.0
GSVT	0.696420	0.693430	0.699437	300.0
SB	0.708347	0.707321	0.709378	892.0
SR	0.701870	0.703320	0.700430	681.0
macro avg	0.696589	0.696962	0.696243	2077.0
micro avg	0.701703	0.701703	0.701703	2077.0
weighted avg	0.701689	0.701690	0.701703	2077.0

The result of Model Number:Dataset Number:Featuregroup Number:Rescale of 75 : 0 : 6 : 1

	f1-score	precision	recall	support
AFIB	0.865785	0.857864	0.873892	875.0
GSVT	0.876879	0.884615	0.869407	691.0
SB	0.932094	0.927180	0.937074	1553.0
SR	0.911023	0.922002	0.900386	907.0
macro avg	0.896445	0.897915	0.895190	4026.0
micro avg	0.903463	0.903463	0.903463	4026.0
weighted avg	0.903459	0.903643	0.903463	4026.0

The result of Model Number:Dataset Number:Featuregroup Number:Rescale of 75 : 0 : 7 : 0

	f1-score	precision	recall	support
AFIB	0.749682	0.748092	0.751314	875.0
GSVT	0.761395	0.757745	0.765143	691.0
SB	0.801365	0.791798	0.811167	1553.0
SR	0.764124	0.785772	0.743652	907.0
macro avg	0.769141	0.770852	0.767819	4026.0
micro avg	0.775050	0.775050	0.775050	4026.0
weighted avg	0.774882	0.775097	0.775050	4026.0

The result of Model Number:Dataset Number:Featuregroup Number:Rescale of 75 : 0 : 7 : 1

	f1-score	precision	recall	support
AFIB	0.692668	0.683074	0.702563	875.0
GSVT	0.708031	0.713761	0.702396	691.0
SB	0.751566	0.749407	0.753743	1553.0
SR	0.733283	0.742827	0.723988	907.0
macro avg	0.721387	0.722267	0.720673	4026.0
micro avg	0.727103	0.727103	0.727103	4026.0
weighted avg	0.727174	0.727390	0.727103	4026.0

The result of Model Number:Dataset Number:Featuregroup Number:Rescale of 75 : 0 : 8 : 0

	f1-score	precision	recall	support
AFIB	0.678905	0.652951	0.721611	875.0
GSVT	0.703461	0.679183	0.736391	691.0
SB	0.783786	0.769951	0.798988	1553.0
SR	0.663849	0.798240	0.573144	907.0
macro avg	0.707500	0.725081	0.707534	4026.0
micro avg	0.720548	0.720548	0.720548	4026.0
weighted avg	0.720185	0.735317	0.720548	4026.0

The result of Model Number:Dataset Number:Featuregroup Number:Rescale of 75 : 0 : 8 : 1

	f1-score	precision	recall	support
AFIB	0.706103	0.779959	0.645231	204.0
GSVT	0.830682	0.819714	0.842036	300.0
SB	0.880934	0.877511	0.884390	892.0
SR	0.859766	0.847513	0.872388	681.0
macro avg	0.819371	0.831174	0.811011	2077.0
micro avg	0.850848	0.850848	0.850848	2077.0
weighted avg	0.849564	0.849746	0.850848	2077.0

The result of Model Number:Dataset Number:Featuregroup Number:Rescale of 75 : 0 : 9 : 0

	f1-score	precision	recall	support
AFIB	0.860122	0.875598	0.845438	204.0
GSVT	0.879527	0.867160	0.892397	300.0
SB	0.893060	0.882508	0.903871	892.0
SR	0.875099	0.890679	0.860064	681.0
macro avg	0.876952	0.878986	0.875442	2077.0
micro avg	0.882111	0.882111	0.882111	2077.0
weighted avg	0.881981	0.882291	0.882111	2077.0

The result of Model Number:Dataset Number:Featuregroup Number:Rescale of 75 : 0 : 9 : 1

	f1-score	precision	recall	support
AFIB	0.669894	0.663969	0.675953	875.0
GSVT	0.609050	0.589818	0.677580	691.0
SB	0.549091	0.527203	0.583320	1553.0
SR	0.565918	0.571774	0.560218	907.0
macro avg	0.598488	0.588191	0.624268	4026.0
micro avg	0.614426	0.614426	0.614426	4026.0
weighted avg	0.589428	0.577716	0.614426	4026.0

The result of Model Number:Dataset Number:Featuregroup Number:Rescale of 75 : 0 : 10 : 0

	f1-score	precision	recall	support
AFIB	0.859021	0.867804	0.850486	875.0
GSVT	0.858763	0.845496	0.872554	691.0
SB	0.905880	0.900471	0.911363	1553.0

SR 0.881903 0.893707 0.870433 907.0
macro avg 0.876392 0.876870 0.876209 4026.0
micro avg 0.882250 0.882250 0.882250 4026.0
weighted avg 0.882207 0.882412 0.882250 4026.0
The result of Model Number:Dataset Number:Featuregroup Number:Rescale of 75 : 0 : 10 : 1
f1-score precision recall support
AFIB 0.856588 0.850799 0.862573 875.0
GSVT 0.877486 0.877836 0.877250 691.0
SB 0.932150 0.929772 0.934551 1553.0
SR 0.906769 0.916900 0.896872 907.0
macro avg 0.893249 0.893827 0.892812 4026.0
micro avg 0.900584 0.900584 0.900584 4026.0
weighted avg 0.900628 0.900794 0.900584 4026.0
The result of Model Number:Dataset Number:Featuregroup Number:Rescale of 75 : 1 : 0 : 0
f1-score precision recall support
AFIB 0.788276 0.792030 0.784593 875.0
GSVT 0.798014 0.783512 0.813146 691.0
SB 0.844359 0.841126 0.847620 1553.0
SR 0.819402 0.833428 0.805854 907.0
macro avg 0.812513 0.812524 0.812803 4026.0
micro avg 0.818596 0.818596 0.818596 4026.0
weighted avg 0.818594 0.818833 0.818596 4026.0
The result of Model Number:Dataset Number:Featuregroup Number:Rescale of 75 : 1 : 0 : 1
f1-score precision recall support
AFIB 0.744235 0.786572 0.706716 204.0
GSVT 0.786489 0.777828 0.795384 300.0
SB 0.798188 0.790246 0.806318 892.0
SR 0.788915 0.791612 0.786361 681.0
macro avg 0.779457 0.786565 0.773695 2077.0
micro avg 0.788413 0.788413 0.788413 2077.0
weighted avg 0.788159 0.788539 0.788413 2077.0
The result of Model Number:Dataset Number:Featuregroup Number:Rescale of 75 : 1 : 1 : 0
f1-score precision recall support
AFIB 0.678806 0.682039 0.675634 875.0
GSVT 0.687192 0.674703 0.700222 691.0
SB 0.727101 0.724317 0.729909 1553.0
SR 0.705609 0.717687 0.693943 907.0
macro avg 0.699677 0.699686 0.699927 4026.0
micro avg 0.704915 0.704915 0.704915 4026.0
weighted avg 0.704913 0.705119 0.704915 4026.0

The result of Model Number:Dataset Number:Featuregroup Number:Rescale of 75 : 1 : 1 : 1

	f1-score	precision	recall	support
AFIB	0.773698	0.775248	0.772186	875.0
GSVT	0.775614	0.771426	0.779867	691.0
SB	0.810777	0.806445	0.815161	1553.0
SR	0.791380	0.800765	0.782235	907.0
macro avg	0.787867	0.788471	0.787362	4026.0
micro avg	0.792345	0.792345	0.792345	4026.0
weighted avg	0.792313	0.792375	0.792345	4026.0

The result of Model Number:Dataset Number:Featuregroup Number:Rescale of 75 : 1 : 2 : 0

	f1-score	precision	recall	support
AFIB	0.679496	0.680673	0.678333	875.0
GSVT	0.687735	0.679832	0.695880	691.0
SB	0.724209	0.715476	0.733160	1553.0
SR	0.689785	0.710685	0.670094	907.0
macro avg	0.695306	0.696667	0.694367	4026.0
micro avg	0.700638	0.700638	0.700638	4026.0
weighted avg	0.700476	0.700715	0.700638	4026.0

The result of Model Number:Dataset Number:Featuregroup Number:Rescale of 75 : 1 : 2 : 1

	f1-score	precision	recall	support
AFIB	0.895454	0.906955	0.884286	875.0
GSVT	0.905360	0.898039	0.912947	691.0
SB	0.968476	0.965565	0.971408	1553.0
SR	0.944495	0.944098	0.944905	907.0
macro avg	0.928446	0.928664	0.928387	4026.0
micro avg	0.936469	0.936469	0.936469	4026.0
weighted avg	0.936370	0.936401	0.936469	4026.0

The result of Model Number:Dataset Number:Featuregroup Number:Rescale of 75 : 1 : 3 : 0

	f1-score	precision	recall	support
AFIB	0.890001	0.927450	0.855538	204.0
GSVT	0.919861	0.905781	0.934525	300.0
SB	0.942048	0.935291	0.948914	892.0
SR	0.917598	0.922264	0.913014	681.0
macro avg	0.917377	0.922697	0.912998	2077.0
micro avg	0.925894	0.925894	0.925894	2077.0
weighted avg	0.925715	0.925987	0.925894	2077.0

The result of Model Number:Dataset Number:Featuregroup Number:Rescale of 75 : 1 : 3 : 1

	f1-score	precision	recall	support
AFIB	0.689108	0.692890	0.685411	875.0

GSVT 0.690072 0.681347 0.699131 691.0
SB 0.725730 0.722818 0.728672 1553.0
SR 0.710151 0.718772 0.701778 907.0
macro avg 0.703765 0.703957 0.703748 4026.0
micro avg 0.708141 0.708141 0.708141 4026.0
weighted avg 0.708141 0.708284 0.708141 4026.0
The result of Model Number:Dataset Number:Featuregroup Number:Rescale of 75 : 1 : 4 : 0

	f1-score	precision	recall	support
AFIB	0.688378	0.685579	0.691218	875.0
GSVT	0.698440	0.702663	0.694295	691.0
SB	0.736637	0.735740	0.737538	1553.0
SR	0.721269	0.722412	0.720151	907.0
macro avg	0.711181	0.711598	0.710801	4026.0
micro avg	0.716132	0.716132	0.716132	4026.0
weighted avg	0.716130	0.716158	0.716132	4026.0

The result of Model Number:Dataset Number:Featuregroup Number:Rescale of 75 : 1 : 4 : 1

	f1-score	precision	recall	support
AFIB	0.833623	0.822902	0.844687	875.0
GSVT	0.850593	0.857635	0.843700	691.0
SB	0.909455	0.905407	0.913550	1553.0
SR	0.883574	0.896642	0.870902	907.0
macro avg	0.869311	0.870646	0.868210	4026.0
micro avg	0.876987	0.876987	0.876987	4026.0
weighted avg	0.877040	0.877302	0.876987	4026.0

The result of Model Number:Dataset Number:Featuregroup Number:Rescale of 75 : 1 : 5 : 0

	f1-score	precision	recall	support
AFIB	0.795751	0.793546	0.798029	875.0
GSVT	0.802049	0.805874	0.798339	691.0
SB	0.839604	0.836921	0.842307	1553.0
SR	0.826181	0.830172	0.822267	907.0
macro avg	0.815896	0.816628	0.815236	4026.0
micro avg	0.820623	0.820623	0.820623	4026.0
weighted avg	0.820603	0.820645	0.820623	4026.0

The result of Model Number:Dataset Number:Featuregroup Number:Rescale of 75 : 1 : 5 : 1

	f1-score	precision	recall	support
AFIB	0.722798	0.704301	0.742359	875.0
GSVT	0.683267	0.721993	0.648643	691.0
SB	0.724502	0.666716	0.793274	1553.0
SR	0.578755	0.702974	0.491948	907.0
macro avg	0.677330	0.698996	0.669056	4026.0

micro avg 0.689500 0.689500 0.689500 4026.0
weighted avg 0.684220 0.692541 0.689500 4026.0
The result of Model Number:Dataset Number:Featuregroup Number:Rescale of 75 : 1 : 6 : 0

	f1-score	precision	recall	support
AFIB	0.821913	0.841911	0.803045	204.0
GSVT	0.832245	0.820788	0.844137	300.0
SB	0.849049	0.840571	0.857707	892.0
SR	0.834225	0.845173	0.823582	681.0
macro avg	0.834358	0.837111	0.832118	2077.0
micro avg	0.839189	0.839189	0.839189	2077.0
weighted avg	0.839096	0.839354	0.839189	2077.0

The result of Model Number:Dataset Number:Featuregroup Number:Rescale of 75 : 1 : 6 : 1

	f1-score	precision	recall	support
AFIB	0.738831	0.748321	0.729616	875.0
GSVT	0.747005	0.740964	0.753265	691.0
SB	0.799082	0.796679	0.801501	1553.0
SR	0.779295	0.778967	0.779633	907.0
macro avg	0.766053	0.766233	0.766004	4026.0
micro avg	0.772672	0.772672	0.772672	4026.0
weighted avg	0.772591	0.772616	0.772672	4026.0

The result of Model Number:Dataset Number:Featuregroup Number:Rescale of 75 : 1 : 7 : 0

	f1-score	precision	recall	support
AFIB	0.796665	0.790571	0.802958	875.0
GSVT	0.814995	0.819452	0.810634	691.0
SB	0.863014	0.862409	0.863625	1553.0
SR	0.840091	0.844166	0.836098	907.0
macro avg	0.828691	0.829149	0.828329	4026.0
micro avg	0.835144	0.835144	0.835144	4026.0
weighted avg	0.835188	0.835313	0.835144	4026.0

The result of Model Number:Dataset Number:Featuregroup Number:Rescale of 75 : 1 : 7 : 1

	f1-score	precision	recall	support
AFIB	0.808182	0.813839	0.802633	204.0
GSVT	0.826986	0.822909	0.831116	300.0
SB	0.843422	0.842019	0.844841	892.0
SR	0.835028	0.837010	0.833065	681.0
macro avg	0.828405	0.828944	0.827914	2077.0
micro avg	0.834852	0.834852	0.834852	2077.0
weighted avg	0.834835	0.834848	0.834852	2077.0

The result of Model Number:Dataset Number:Featuregroup Number:Rescale of 75 : 1 : 8 : 0

	f1-score	precision	recall	support
AFIB	0.834462	0.836170	0.832852	204.0
GSVT	0.852816	0.849501	0.856216	300.0
SB	0.871748	0.870585	0.872919	892.0
SR	0.865345	0.867903	0.862804	681.0
macro avg	0.856093	0.856040	0.856198	2077.0
micro avg	0.863255	0.863255	0.863255	2077.0
weighted avg	0.863252	0.863280	0.863255	2077.0

The result of Model Number:Dataset Number:Featuregroup Number:Rescale of 75 : 1 : 8 : 1

	f1-score	precision	recall	support
AFIB	0.923250	0.936814	0.910159	204.0
GSVT	0.946643	0.938595	0.954849	300.0
SB	0.963003	0.959151	0.966890	892.0
SR	0.953479	0.958159	0.948856	681.0
macro avg	0.946594	0.948180	0.945188	2077.0
micro avg	0.953666	0.953666	0.953666	2077.0
weighted avg	0.953613	0.953663	0.953666	2077.0

The result of Model Number:Dataset Number:Featuregroup Number:Rescale of 75 : 1 : 9 : 0

	f1-score	precision	recall	support
AFIB	0.671985	0.674499	0.669776	875.0
GSVT	0.687112	0.693740	0.680746	691.0
SB	0.730615	0.722951	0.738498	1553.0
SR	0.705314	0.711522	0.699457	907.0
macro avg	0.698756	0.700678	0.697119	4026.0
micro avg	0.704855	0.704855	0.704855	4026.0
weighted avg	0.704706	0.704832	0.704855	4026.0

The result of Model Number:Dataset Number:Featuregroup Number:Rescale of 75 : 1 : 9 : 1

	f1-score	precision	recall	support
AFIB	0.692789	0.719709	0.667859	875.0
GSVT	0.744770	0.739270	0.750451	691.0
SB	0.818445	0.814228	0.822712	1553.0
SR	0.770232	0.755544	0.785584	907.0
macro avg	0.756559	0.757188	0.756652	4026.0
micro avg	0.768290	0.768290	0.768290	4026.0
weighted avg	0.767628	0.767600	0.768290	4026.0

The result of Model Number:Dataset Number:Featuregroup Number:Rescale of 75 : 1 : 10 : 0

	f1-score	precision	recall	support
AFIB	0.822273	0.817355	0.827363	875.0
GSVT	0.829007	0.833483	0.824699	691.0
SB	0.878624	0.876055	0.881220	1553.0

SR	0.858770	0.864938	0.852705	907.0
macro avg	0.847169	0.847958	0.846497	4026.0
micro avg	0.853390	0.853390	0.853390	4026.0
weighted avg	0.853388	0.853486	0.853390	4026.0

The result of Model Number:Dataset Number:Featuregroup Number:Rescale of 75 : 1 : 10 : 1

	f1-score	precision	recall	support
AFIB	0.891102	0.903152	0.879742	204.0
GSVT	0.924949	0.923448	0.926488	300.0
SB	0.945036	0.943777	0.946306	892.0
SR	0.935376	0.934178	0.936611	681.0
macro avg	0.924116	0.926139	0.922287	2077.0
micro avg	0.933727	0.933727	0.933727	2077.0
weighted avg	0.933670	0.933703	0.933727	2077.0

The result of Model Number:Dataset Number:Featuregroup Number:Rescale of 76 : 0 : 0 : 0

	f1-score	precision	recall	support
AFIB	0.836868	0.878681	0.799274	204.0
GSVT	0.862098	0.846121	0.878990	300.0
SB	0.881827	0.872938	0.890917	892.0
SR	0.857370	0.865029	0.849924	681.0
macro avg	0.859541	0.865692	0.854776	2077.0
micro avg	0.866753	0.866753	0.866753	2077.0
weighted avg	0.866543	0.867035	0.866753	2077.0

The result of Model Number:Dataset Number:Featuregroup Number:Rescale of 76 : 0 : 0 : 1

	f1-score	precision	recall	support
AFIB	0.677273	0.682109	0.672627	204.0
GSVT	0.700955	0.700503	0.701421	300.0
SB	0.714980	0.713394	0.716581	892.0
SR	0.708589	0.709446	0.707755	681.0
macro avg	0.700449	0.701363	0.699596	2077.0
micro avg	0.707180	0.707180	0.707180	2077.0
weighted avg	0.707155	0.707165	0.707180	2077.0

The result of Model Number:Dataset Number:Featuregroup Number:Rescale of 76 : 0 : 1 : 0

	f1-score	precision	recall	support
AFIB	0.740801	0.757150	0.725198	204.0
GSVT	0.756991	0.744438	0.770065	300.0
SB	0.771537	0.765502	0.777676	892.0
SR	0.752813	0.761873	0.743966	681.0
macro avg	0.755535	0.757241	0.754226	2077.0
micro avg	0.760370	0.760370	0.760370	2077.0
weighted avg	0.760278	0.760449	0.760370	2077.0

The result of Model Number:Dataset Number:Featuregroup Number:Rescale of 76 : 0 : 1 : 1

	f1-score	precision	recall	support
AFIB	0.688060	0.693054	0.683188	875.0
GSVT	0.703153	0.696618	0.709866	691.0
SB	0.755684	0.751122	0.760306	1553.0
SR	0.730917	0.739079	0.722937	907.0
macro avg	0.719454	0.719968	0.719074	4026.0
micro avg	0.726469	0.726469	0.726469	4026.0
weighted avg	0.726391	0.726434	0.726469	4026.0

The result of Model Number:Dataset Number:Featuregroup Number:Rescale of 76 : 0 : 2 : 0

	f1-score	precision	recall	support
AFIB	0.686577	0.696296	0.677689	204.0
GSVT	0.700831	0.687928	0.714357	300.0
SB	0.715769	0.707855	0.723873	892.0
SR	0.700726	0.714998	0.687055	681.0
macro avg	0.700976	0.701769	0.700744	2077.0
micro avg	0.705891	0.705891	0.705891	2077.0
weighted avg	0.705812	0.706183	0.705891	2077.0

The result of Model Number:Dataset Number:Featuregroup Number:Rescale of 76 : 0 : 2 : 1

	f1-score	precision	recall	support
AFIB	0.741892	0.748037	0.835640	204.0
GSVT	0.804532	0.770097	0.907701	300.0
SB	0.734568	0.732015	0.737141	892.0
SR	0.727264	0.727401	0.727132	681.0
macro avg	0.752064	0.744388	0.801903	2077.0
micro avg	0.768169	0.768169	0.768169	2077.0
weighted avg	0.742998	0.737577	0.768169	2077.0

The result of Model Number:Dataset Number:Featuregroup Number:Rescale of 76 : 0 : 3 : 0

	f1-score	precision	recall	support
AFIB	0.891695	0.893520	0.889974	204.0
GSVT	0.911308	0.907765	0.914941	300.0
SB	0.931538	0.930295	0.932789	892.0
SR	0.924696	0.927429	0.921981	681.0
macro avg	0.914809	0.914752	0.914922	2077.0
micro avg	0.922462	0.922462	0.922462	2077.0
weighted avg	0.922459	0.922489	0.922462	2077.0

The result of Model Number:Dataset Number:Featuregroup Number:Rescale of 76 : 0 : 3 : 1

	f1-score	precision	recall	support
AFIB	0.776985	0.781234	0.772808	875.0

GSVT 0.773282 0.766762 0.779992 691.0
SB 0.813300 0.805598 0.821154 1553.0
SR 0.783031 0.797638 0.768961 907.0
macro avg 0.786650 0.787808 0.785729 4026.0
micro avg 0.791823 0.791823 0.791823 4026.0
weighted avg 0.791720 0.791844 0.791823 4026.0
The result of Model Number:Dataset Number:Featuregroup Number:Rescale of 76 : 0 : 4 : 0
f1-score precision recall support
AFIB 0.777546 0.768332 0.787048 875.0
GSVT 0.788612 0.796236 0.781211 691.0
SB 0.836981 0.833779 0.840214 1553.0
SR 0.817468 0.826899 0.808272 907.0
macro avg 0.805152 0.806311 0.804186 4026.0
micro avg 0.811336 0.811336 0.811336 4026.0
weighted avg 0.811366 0.811561 0.811336 4026.0
The result of Model Number:Dataset Number:Featuregroup Number:Rescale of 76 : 0 : 4 : 1
f1-score precision recall support
AFIB 0.887408 0.878482 0.896542 875.0
GSVT 0.895349 0.903321 0.887583 691.0
SB 0.947240 0.944333 0.950167 1553.0
SR 0.928897 0.937028 0.920909 907.0
macro avg 0.914723 0.915791 0.913800 4026.0
micro avg 0.921179 0.921179 0.921179 4026.0
weighted avg 0.921198 0.921336 0.921179 4026.0
The result of Model Number:Dataset Number:Featuregroup Number:Rescale of 76 : 0 : 5 : 0
f1-score precision recall support
AFIB 0.697022 0.692633 0.785147 204.0
GSVT 0.849681 0.845311 0.854240 300.0
SB 0.873874 0.869716 0.878082 892.0
SR 0.685456 0.687714 0.683626 681.0
macro avg 0.776508 0.773843 0.800274 2077.0
micro avg 0.801752 0.801752 0.801752 2077.0
weighted avg 0.791232 0.789124 0.801752 2077.0
The result of Model Number:Dataset Number:Featuregroup Number:Rescale of 76 : 0 : 5 : 1
f1-score precision recall support
AFIB 0.727020 0.754066 0.702173 204.0
GSVT 0.674647 0.705954 0.646062 300.0
SB 0.706720 0.664094 0.755206 892.0
SR 0.620636 0.663758 0.582848 681.0
macro avg 0.682256 0.696968 0.671572 2077.0

micro avg 0.677720 0.677720 0.677720 2077.0
weighted avg 0.675856 0.678867 0.677720 2077.0
The result of Model Number:Dataset Number:Featuregroup Number:Rescale of 76 : 0 : 6 : 0

	f1-score	precision	recall	support
AFIB	0.770874	0.783090	0.759136	204.0
GSVT	0.790689	0.783960	0.797538	300.0
SB	0.804340	0.802547	0.806144	892.0
SR	0.796719	0.798490	0.794971	681.0
macro avg	0.790656	0.792022	0.789447	2077.0
micro avg	0.796620	0.796620	0.796620	2077.0
weighted avg	0.796583	0.796621	0.796620	2077.0

The result of Model Number:Dataset Number:Featuregroup Number:Rescale of 76 : 0 : 6 : 1

	f1-score	precision	recall	support
AFIB	0.872507	0.894976	0.851173	204.0
GSVT	0.800677	0.765036	0.903564	300.0
SB	0.918608	0.916459	0.920769	892.0
SR	0.728749	0.728886	0.728621	681.0
macro avg	0.830135	0.826339	0.851032	2077.0
micro avg	0.848448	0.848448	0.848448	2077.0
weighted avg	0.834796	0.830977	0.848448	2077.0

The result of Model Number:Dataset Number:Featuregroup Number:Rescale of 76 : 0 : 7 : 0

	f1-score	precision	recall	support
AFIB	0.744838	0.825292	0.678813	204.0
GSVT	0.863426	0.849384	0.878077	300.0
SB	0.920791	0.916599	0.925023	892.0
SR	0.898542	0.887108	0.910288	681.0
macro avg	0.856899	0.869596	0.848050	2077.0
micro avg	0.889228	0.889228	0.889228	2077.0
weighted avg	0.887929	0.888253	0.889228	2077.0

The result of Model Number:Dataset Number:Featuregroup Number:Rescale of 76 : 0 : 7 : 1

	f1-score	precision	recall	support
AFIB	0.765329	0.774083	0.757067	204.0
GSVT	0.783243	0.778608	0.787946	300.0
SB	0.751899	0.724125	0.798781	892.0
SR	0.630725	0.633777	0.627717	681.0
macro avg	0.732799	0.727648	0.742878	2077.0
micro avg	0.737031	0.737031	0.737031	2077.0
weighted avg	0.718015	0.707278	0.737031	2077.0

The result of Model Number:Dataset Number:Featuregroup Number:Rescale of 76 : 0 : 8 : 0

	f1-score	precision	recall	support
AFIB	0.784972	0.792646	0.777629	875.0
GSVT	0.778976	0.773066	0.785234	691.0
SB	0.824246	0.815765	0.832977	1553.0
SR	0.798083	0.810918	0.785873	907.0
macro avg	0.796569	0.798099	0.795428	4026.0
micro avg	0.802142	0.802142	0.802142	4026.0
weighted avg	0.802046	0.802320	0.802142	4026.0

The result of Model Number:Dataset Number:Featuregroup Number:Rescale of 76 : 0 : 8 : 1

	f1-score	precision	recall	support
AFIB	0.871248	0.868833	0.873741	875.0
GSVT	0.878142	0.882330	0.874081	691.0
SB	0.919261	0.916323	0.922220	1553.0
SR	0.904564	0.908933	0.900279	907.0
macro avg	0.893304	0.894105	0.892580	4026.0
micro avg	0.898478	0.898478	0.898478	4026.0
weighted avg	0.898457	0.898503	0.898478	4026.0

The result of Model Number:Dataset Number:Featuregroup Number:Rescale of 76 : 0 : 9 : 0

	f1-score	precision	recall	support
AFIB	0.629988	0.613438	0.669427	875.0
GSVT	0.606042	0.573627	0.690876	691.0
SB	0.574256	0.570627	0.577949	1553.0
SR	0.557799	0.567410	0.548664	907.0
macro avg	0.592021	0.581275	0.621729	4026.0
micro avg	0.610615	0.610615	0.610615	4026.0
weighted avg	0.588117	0.579722	0.610615	4026.0

The result of Model Number:Dataset Number:Featuregroup Number:Rescale of 76 : 0 : 9 : 1

	f1-score	precision	recall	support
AFIB	0.798605	0.832208	0.767681	204.0
GSVT	0.825399	0.812765	0.838557	300.0
SB	0.845307	0.839244	0.851468	892.0
SR	0.823368	0.827555	0.819254	681.0
macro avg	0.823170	0.827943	0.819240	2077.0
micro avg	0.830812	0.830812	0.830812	2077.0
weighted avg	0.830651	0.830896	0.830812	2077.0

The result of Model Number:Dataset Number:Featuregroup Number:Rescale of 76 : 0 : 10 : 0

	f1-score	precision	recall	support
AFIB	0.663345	0.733485	0.605793	204.0
GSVT	0.763264	0.754431	0.772453	300.0
SB	0.806983	0.803665	0.810331	892.0

SR 0.789653 0.777957 0.801715 681.0
macro avg 0.755811 0.767384 0.747573 2077.0
micro avg 0.781945 0.781945 0.781945 2077.0
weighted avg 0.780878 0.781232 0.781945 2077.0
The result of Model Number:Dataset Number:Featuregroup Number:Rescale of 76 : 0 : 10 : 1

	f1-score	precision	recall	support
AFIB	0.804775	0.793596	0.816350	875.0
GSVT	0.833533	0.844653	0.822811	691.0
SB	0.885483	0.883669	0.887308	1553.0
SR	0.862437	0.869101	0.855875	907.0
macro avg	0.846557	0.847755	0.845586	4026.0
micro avg	0.853735	0.853735	0.853735	4026.0
weighted avg	0.853834	0.854114	0.853735	4026.0

The result of Model Number:Dataset Number:Featuregroup Number:Rescale of 76 : 1 : 0 : 0

	f1-score	precision	recall	support
AFIB	0.895497	0.896709	0.894460	875.0
GSVT	0.909445	0.908978	0.909947	691.0
SB	0.949821	0.946423	0.953247	1553.0
SR	0.927076	0.932272	0.921997	907.0
macro avg	0.920460	0.921095	0.919913	4026.0
micro avg	0.925999	0.925999	0.925999	4026.0
weighted avg	0.925961	0.926003	0.925999	4026.0

The result of Model Number:Dataset Number:Featuregroup Number:Rescale of 76 : 1 : 0 : 1

	f1-score	precision	recall	support
AFIB	0.735627	0.750007	0.721918	204.0
GSVT	0.755841	0.750395	0.761392	300.0
SB	0.769368	0.766374	0.772387	892.0
SR	0.760959	0.763112	0.758834	681.0
macro avg	0.755449	0.757472	0.753633	2077.0
micro avg	0.761398	0.761398	0.761398	2077.0
weighted avg	0.761343	0.761389	0.761398	2077.0

The result of Model Number:Dataset Number:Featuregroup Number:Rescale of 76 : 1 : 1 : 0

	f1-score	precision	recall	support
AFIB	0.650226	0.642966	0.657658	875.0
GSVT	0.663162	0.669236	0.657208	691.0
SB	0.707293	0.704990	0.709614	1553.0
SR	0.688957	0.695859	0.682193	907.0
macro avg	0.677410	0.678263	0.676668	4026.0
micro avg	0.683150	0.683150	0.683150	4026.0
weighted avg	0.683185	0.683316	0.683150	4026.0

The result of Model Number:Dataset Number:Featuregroup Number:Rescale of 76 : 1 : 1 : 1

	f1-score	precision	recall	support
AFIB	0.899050	0.930262	0.869986	204.0
GSVT	0.916289	0.906258	0.926657	300.0
SB	0.940681	0.933927	0.947536	892.0
SR	0.923881	0.928424	0.919399	681.0
macro avg	0.919975	0.924718	0.915895	2077.0
micro avg	0.927678	0.927678	0.927678	2077.0
weighted avg	0.927561	0.927766	0.927678	2077.0

The result of Model Number:Dataset Number:Featuregroup Number:Rescale of 76 : 1 : 2 : 0

	f1-score	precision	recall	support
AFIB	0.842666	0.859494	0.826613	204.0
GSVT	0.852129	0.841482	0.863178	300.0
SB	0.868398	0.860095	0.876865	892.0
SR	0.852628	0.863813	0.841752	681.0
macro avg	0.853955	0.856221	0.852102	2077.0
micro avg	0.858439	0.858439	0.858439	2077.0
weighted avg	0.858350	0.858566	0.858439	2077.0

The result of Model Number:Dataset Number:Featuregroup Number:Rescale of 76 : 1 : 2 : 1

	f1-score	precision	recall	support
AFIB	0.858405	0.874943	0.842887	204.0
GSVT	0.874525	0.860315	0.889370	300.0
SB	0.889125	0.878521	0.899990	892.0
SR	0.872585	0.888945	0.856831	681.0
macro avg	0.873660	0.875681	0.872270	2077.0
micro avg	0.878697	0.878697	0.878697	2077.0
weighted avg	0.878576	0.878958	0.878697	2077.0

The result of Model Number:Dataset Number:Featuregroup Number:Rescale of 76 : 1 : 3 : 0

	f1-score	precision	recall	support
AFIB	0.701934	0.693744	0.712149	875.0
GSVT	0.722247	0.713493	0.731801	691.0
SB	0.733134	0.704834	0.774643	1553.0
SR	0.582146	0.612854	0.554404	907.0
macro avg	0.684865	0.681231	0.693249	4026.0
micro avg	0.704091	0.704091	0.704091	4026.0
weighted avg	0.690469	0.683188	0.704091	4026.0

The result of Model Number:Dataset Number:Featuregroup Number:Rescale of 76 : 1 : 3 : 1

	f1-score	precision	recall	support
AFIB	0.689803	0.689182	0.690492	875.0

GSVT 0.701754 0.695328 0.708354 691.0
SB 0.736407 0.729932 0.742998 1553.0
SR 0.701737 0.719086 0.685268 907.0
macro avg 0.707425 0.708382 0.706778 4026.0
micro avg 0.712634 0.712634 0.712634 4026.0
weighted avg 0.712520 0.712693 0.712634 4026.0
The result of Model Number:Dataset Number:Featuregroup Number:Rescale of 76 : 1 : 4 : 0

	f1-score	precision	recall	support
AFIB	0.727582	0.727224	0.727996	875.0
GSVT	0.743376	0.739718	0.747117	691.0
SB	0.777584	0.772501	0.782741	1553.0
SR	0.755449	0.767754	0.743552	907.0
macro avg	0.750998	0.751799	0.750351	4026.0
micro avg	0.755900	0.755900	0.755900	4026.0
weighted avg	0.755859	0.755965	0.755900	4026.0

The result of Model Number:Dataset Number:Featuregroup Number:Rescale of 76 : 1 : 4 : 1

	f1-score	precision	recall	support
AFIB	0.686713	0.765898	0.622457	204.0
GSVT	0.809704	0.795611	0.824478	300.0
SB	0.864514	0.860682	0.868389	892.0
SR	0.844452	0.832906	0.856348	681.0
macro avg	0.801346	0.813774	0.792918	2077.0
micro avg	0.833944	0.833944	0.833944	2077.0
weighted avg	0.832556	0.832866	0.833944	2077.0

The result of Model Number:Dataset Number:Featuregroup Number:Rescale of 76 : 1 : 5 : 0

	f1-score	precision	recall	support
AFIB	0.863207	0.883727	0.843715	204.0
GSVT	0.885617	0.882199	0.889154	300.0
SB	0.907562	0.905537	0.909597	892.0
SR	0.896144	0.894341	0.897986	681.0
macro avg	0.888133	0.891451	0.885113	2077.0
micro avg	0.896367	0.896367	0.896367	2077.0
weighted avg	0.896292	0.896353	0.896367	2077.0

The result of Model Number:Dataset Number:Featuregroup Number:Rescale of 76 : 1 : 5 : 1

	f1-score	precision	recall	support
AFIB	0.705184	0.710219	0.700346	204.0
GSVT	0.729842	0.729371	0.730327	300.0
SB	0.744445	0.742793	0.746111	892.0
SR	0.737790	0.738683	0.736922	681.0
macro avg	0.729315	0.730266	0.728427	2077.0

micro avg 0.736323 0.736323 0.736323 2077.0
weighted avg 0.736297 0.736307 0.736323 2077.0
The result of Model Number:Dataset Number:Featuregroup Number:Rescale of 76 : 1 : 6 : 0

	f1-score	precision	recall	support
AFIB	0.744924	0.724262	0.769143	875.0
GSVT	0.754648	0.786435	0.729974	691.0
SB	0.829007	0.828588	0.829437	1553.0
SR	0.808210	0.813267	0.803220	907.0
macro avg	0.784197	0.788138	0.782944	4026.0
micro avg	0.793355	0.793355	0.793355	4026.0
weighted avg	0.793285	0.795228	0.793355	4026.0

The result of Model Number:Dataset Number:Featuregroup Number:Rescale of 76 : 1 : 6 : 1

	f1-score	precision	recall	support
AFIB	0.856694	0.854320	0.859146	875.0
GSVT	0.863474	0.867592	0.859480	691.0
SB	0.903905	0.901017	0.906815	1553.0
SR	0.889454	0.893750	0.885240	907.0
macro avg	0.878382	0.879170	0.877670	4026.0
micro avg	0.883470	0.883470	0.883470	4026.0
weighted avg	0.883449	0.883494	0.883470	4026.0

The result of Model Number:Dataset Number:Featuregroup Number:Rescale of 76 : 1 : 7 : 0

	f1-score	precision	recall	support
AFIB	0.664870	0.649232	0.699962	875.0
GSVT	0.638880	0.632989	0.653438	691.0
SB	0.692908	0.644119	0.750492	1553.0
SR	0.432037	0.541844	0.359792	907.0
macro avg	0.607174	0.617046	0.615921	4026.0
micro avg	0.634833	0.634833	0.634833	4026.0
weighted avg	0.618771	0.620279	0.634833	4026.0

The result of Model Number:Dataset Number:Featuregroup Number:Rescale of 76 : 1 : 7 : 1

	f1-score	precision	recall	support
AFIB	0.770671	0.781873	0.760133	204.0
GSVT	0.787667	0.777011	0.798683	300.0
SB	0.799522	0.791189	0.808037	892.0
SR	0.783675	0.796643	0.771136	681.0
macro avg	0.785384	0.786679	0.784497	2077.0
micro avg	0.789882	0.789882	0.789882	2077.0
weighted avg	0.789780	0.790014	0.789882	2077.0

The result of Model Number:Dataset Number:Featuregroup Number:Rescale of 76 : 1 : 8 : 0

	f1-score	precision	recall	support
AFIB	0.903287	0.910226	0.896659	204.0
GSVT	0.935097	0.934200	0.936025	300.0
SB	0.954228	0.952005	0.956470	892.0
SR	0.945446	0.946720	0.944196	681.0
macro avg	0.934514	0.935788	0.933338	2077.0
micro avg	0.943618	0.943618	0.943618	2077.0
weighted avg	0.943582	0.943597	0.943618	2077.0

The result of Model Number:Dataset Number:Featuregroup Number:Rescale of 76 : 1 : 8 : 1

	f1-score	precision	recall	support
AFIB	0.677730	0.668115	0.687642	875.0
GSVT	0.691673	0.698414	0.685066	691.0
SB	0.736075	0.733676	0.738491	1553.0
SR	0.718885	0.728073	0.709934	907.0
macro avg	0.706091	0.707070	0.705283	4026.0
micro avg	0.711837	0.711837	0.711837	4026.0
weighted avg	0.711901	0.712113	0.711837	4026.0

The result of Model Number:Dataset Number:Featuregroup Number:Rescale of 76 : 1 : 9 : 0

	f1-score	precision	recall	support
AFIB	0.705785	0.716968	0.695038	204.0
GSVT	0.723926	0.717765	0.730197	300.0
SB	0.736424	0.734783	0.738076	892.0
SR	0.729447	0.731068	0.727847	681.0
macro avg	0.723895	0.725146	0.722789	2077.0
micro avg	0.729357	0.729357	0.729357	2077.0
weighted avg	0.729322	0.729357	0.729357	2077.0

The result of Model Number:Dataset Number:Featuregroup Number:Rescale of 76 : 1 : 9 : 1

	f1-score	precision	recall	support
AFIB	0.888759	0.885293	0.892316	875.0
GSVT	0.905393	0.907302	0.903545	691.0
SB	0.961105	0.958709	0.963517	1553.0
SR	0.938934	0.945240	0.932731	907.0
macro avg	0.923548	0.924136	0.923027	4026.0
micro avg	0.930813	0.930813	0.930813	4026.0
weighted avg	0.930825	0.930895	0.930813	4026.0

The result of Model Number:Dataset Number:Featuregroup Number:Rescale of 76 : 1 : 10 : 0

	f1-score	precision	recall	support
AFIB	0.836100	0.822987	0.849728	875.0
GSVT	0.805637	0.828733	0.783887	691.0
SB	0.846591	0.803676	0.894395	1553.0

SR 0.687850 0.765437 0.624652 907.0
macro avg 0.794045 0.805208 0.788166 4026.0
micro avg 0.804951 0.804951 0.804951 4026.0
weighted avg 0.801520 0.803559 0.804951 4026.0
The result of Model Number:Dataset Number:Featuregroup Number:Rescale of 76 : 1 : 10 : 1

	f1-score	precision	recall	support
AFIB	0.894337	0.901426	0.887381	875.0
GSVT	0.905675	0.901682	0.909734	691.0
SB	0.963079	0.959271	0.966923	1553.0
SR	0.939413	0.942067	0.936819	907.0
macro avg	0.925626	0.926112	0.925214	4026.0
micro avg	0.933038	0.933038	0.933038	4026.0
weighted avg	0.932955	0.932939	0.933038	4026.0

The result of Model Number:Dataset Number:Featuregroup Number:Rescale of 77 : 0 : 0 : 0

	f1-score	precision	recall	support
AFIB	0.800967	0.831468	0.772800	204.0
GSVT	0.826486	0.818156	0.835024	300.0
SB	0.843028	0.838832	0.847279	892.0
SR	0.832845	0.833369	0.832349	681.0
macro avg	0.825832	0.830456	0.821863	2077.0
micro avg	0.833299	0.833299	0.833299	2077.0
weighted avg	0.833169	0.833331	0.833299	2077.0

The result of Model Number:Dataset Number:Featuregroup Number:Rescale of 77 : 0 : 0 : 1

	f1-score	precision	recall	support
AFIB	0.911588	0.913680	0.909605	875.0
GSVT	0.914766	0.912187	0.917390	691.0
SB	0.960032	0.952082	0.968129	1553.0
SR	0.931232	0.945207	0.917703	907.0
macro avg	0.929404	0.930789	0.928207	4026.0
micro avg	0.935341	0.935341	0.935341	4026.0
weighted avg	0.935246	0.935340	0.935341	4026.0

The result of Model Number:Dataset Number:Featuregroup Number:Rescale of 77 : 0 : 1 : 0

	f1-score	precision	recall	support
AFIB	0.895017	0.896426	0.893728	204.0
GSVT	0.916663	0.915232	0.918175	300.0
SB	0.935573	0.934431	0.936724	892.0
SR	0.929021	0.930804	0.927249	681.0
macro avg	0.919068	0.919223	0.918969	2077.0
micro avg	0.926715	0.926715	0.926715	2077.0
weighted avg	0.926710	0.926736	0.926715	2077.0

The result of Model Number:Dataset Number:Featuregroup Number:Rescale of 77 : 0 : 1 : 1

	f1-score	precision	recall	support
AFIB	0.843580	0.821419	0.896390	875.0
GSVT	0.811516	0.768110	0.925111	691.0
SB	0.768953	0.764094	0.773898	1553.0
SR	0.746916	0.759786	0.734684	907.0
macro avg	0.792741	0.778352	0.832521	4026.0
micro avg	0.817639	0.817639	0.817639	4026.0
weighted avg	0.787513	0.776271	0.817639	4026.0

The result of Model Number:Dataset Number:Featuregroup Number:Rescale of 77 : 0 : 2 : 0

	f1-score	precision	recall	support
AFIB	0.811316	0.820144	0.802912	204.0
GSVT	0.838772	0.835274	0.842435	300.0
SB	0.855960	0.855194	0.856727	892.0
SR	0.847871	0.847878	0.847871	681.0
macro avg	0.838480	0.839623	0.837486	2077.0
micro avg	0.846473	0.846473	0.846473	2077.0
weighted avg	0.846440	0.846476	0.846473	2077.0

The result of Model Number:Dataset Number:Featuregroup Number:Rescale of 77 : 0 : 2 : 1

	f1-score	precision	recall	support
AFIB	0.756293	0.780861	0.733368	204.0
GSVT	0.770631	0.763732	0.777808	300.0
SB	0.788955	0.783548	0.794439	892.0
SR	0.776088	0.779363	0.772887	681.0
macro avg	0.772992	0.776876	0.769625	2077.0
micro avg	0.778972	0.778972	0.778972	2077.0
weighted avg	0.778882	0.779049	0.778972	2077.0

The result of Model Number:Dataset Number:Featuregroup Number:Rescale of 77 : 0 : 3 : 0

	f1-score	precision	recall	support
AFIB	0.812819	0.820050	0.805756	875.0
GSVT	0.829879	0.820772	0.839244	691.0
SB	0.892530	0.885581	0.899597	1553.0
SR	0.862870	0.875101	0.850980	907.0
macro avg	0.849525	0.850376	0.848894	4026.0
micro avg	0.857891	0.857891	0.857891	4026.0
weighted avg	0.857771	0.857854	0.857891	4026.0

The result of Model Number:Dataset Number:Featuregroup Number:Rescale of 77 : 0 : 3 : 1

	f1-score	precision	recall	support
AFIB	0.764175	0.787797	0.742595	204.0

GSVT 0.713348 0.728856 0.699012 300.0
SB 0.752401 0.719009 0.789085 892.0
SR 0.677455 0.712267 0.645983 681.0
macro avg 0.726845 0.736982 0.719169 2077.0
micro avg 0.724589 0.724589 0.724589 2077.0
weighted avg 0.723344 0.724977 0.724589 2077.0
The result of Model Number:Dataset Number:Featuregroup Number:Rescale of 77 : 0 : 4 : 0

	f1-score	precision	recall	support
AFIB	0.764900	0.814738	0.721147	204.0
GSVT	0.798409	0.778784	0.819157	300.0
SB	0.821824	0.811267	0.832688	892.0
SR	0.800478	0.810036	0.791196	681.0
macro avg	0.796403	0.803706	0.791047	2077.0
micro avg	0.806174	0.806174	0.806174	2077.0
weighted avg	0.805852	0.806513	0.806174	2077.0

The result of Model Number:Dataset Number:Featuregroup Number:Rescale of 77 : 0 : 4 : 1

	f1-score	precision	recall	support
AFIB	0.693402	0.692287	0.694805	875.0
GSVT	0.709570	0.705871	0.713348	691.0
SB	0.741892	0.735606	0.748291	1553.0
SR	0.713571	0.729435	0.698623	907.0
macro avg	0.714609	0.715800	0.713767	4026.0
micro avg	0.719480	0.719480	0.719480	4026.0
weighted avg	0.719425	0.719697	0.719480	4026.0

The result of Model Number:Dataset Number:Featuregroup Number:Rescale of 77 : 0 : 5 : 0

	f1-score	precision	recall	support
AFIB	0.705221	0.678260	0.749581	875.0
GSVT	0.730728	0.705509	0.764935	691.0
SB	0.814167	0.799795	0.829958	1553.0
SR	0.689581	0.829181	0.595360	907.0
macro avg	0.734924	0.753186	0.734959	4026.0
micro avg	0.748477	0.748477	0.748477	4026.0
weighted avg	0.748100	0.763819	0.748477	4026.0

The result of Model Number:Dataset Number:Featuregroup Number:Rescale of 77 : 0 : 5 : 1

	f1-score	precision	recall	support
AFIB	0.864703	0.882049	0.848198	204.0
GSVT	0.884743	0.875547	0.894182	300.0
SB	0.901106	0.897601	0.904641	892.0
SR	0.891637	0.895357	0.887968	681.0
macro avg	0.885547	0.887638	0.883747	2077.0

micro avg 0.892120 0.892120 0.892120 2077.0
weighted avg 0.892062 0.892152 0.892120 2077.0
The result of Model Number:Dataset Number:Featuregroup Number:Rescale of 77 : 0 : 6 : 0

	f1-score	precision	recall	support
AFIB	0.771072	0.765260	0.875157	204.0
GSVT	0.934773	0.929238	0.940384	300.0
SB	0.764514	0.762919	0.766121	892.0
SR	0.945525	0.944012	0.947055	681.0
macro avg	0.853971	0.850357	0.882179	2077.0
micro avg	0.861324	0.861324	0.861324	2077.0
weighted avg	0.849100	0.846548	0.861324	2077.0

The result of Model Number:Dataset Number:Featuregroup Number:Rescale of 77 : 0 : 6 : 1

	f1-score	precision	recall	support
AFIB	0.734249	0.809720	0.672098	204.0
GSVT	0.873735	0.861927	0.885957	300.0
SB	0.930465	0.926744	0.934220	892.0
SR	0.905211	0.892974	0.917826	681.0
macro avg	0.860915	0.872841	0.852525	2077.0
micro avg	0.896128	0.896128	0.896128	2077.0
weighted avg	0.894719	0.894816	0.896128	2077.0

The result of Model Number:Dataset Number:Featuregroup Number:Rescale of 77 : 0 : 7 : 0

	f1-score	precision	recall	support
AFIB	0.824931	0.844359	0.806449	875.0
GSVT	0.869600	0.871796	0.867472	691.0
SB	0.945706	0.941785	0.949662	1553.0
SR	0.899845	0.885673	0.914520	907.0
macro avg	0.885020	0.885903	0.884526	4026.0
micro avg	0.896513	0.896513	0.896513	4026.0
weighted avg	0.896063	0.895957	0.896513	4026.0

The result of Model Number:Dataset Number:Featuregroup Number:Rescale of 77 : 0 : 7 : 1

	f1-score	precision	recall	support
AFIB	0.686195	0.692425	0.680428	204.0
GSVT	0.708874	0.704590	0.713396	300.0
SB	0.723704	0.722735	0.724678	892.0
SR	0.717008	0.718495	0.715533	681.0
macro avg	0.708945	0.709561	0.708509	2077.0
micro avg	0.715704	0.715704	0.715704	2077.0
weighted avg	0.715683	0.715747	0.715704	2077.0

The result of Model Number:Dataset Number:Featuregroup Number:Rescale of 77 : 0 : 8 : 0

	f1-score	precision	recall	support
AFIB	0.672879	0.668217	0.677741	875.0
GSVT	0.683260	0.686032	0.680620	691.0
SB	0.741338	0.733629	0.749214	1553.0
SR	0.703907	0.720286	0.688321	907.0
macro avg	0.700346	0.702041	0.698974	4026.0
micro avg	0.708189	0.708189	0.708189	4026.0
weighted avg	0.708058	0.708238	0.708189	4026.0

The result of Model Number:Dataset Number:Featuregroup Number:Rescale of 77 : 0 : 8 : 1

	f1-score	precision	recall	support
AFIB	0.681772	0.680676	0.683151	875.0
GSVT	0.697669	0.694032	0.701384	691.0
SB	0.729449	0.723269	0.735741	1553.0
SR	0.701603	0.717200	0.686905	907.0
macro avg	0.702623	0.703794	0.701795	4026.0
micro avg	0.707412	0.707412	0.707412	4026.0
weighted avg	0.707359	0.707626	0.707412	4026.0

The result of Model Number:Dataset Number:Featuregroup Number:Rescale of 77 : 0 : 9 : 0

	f1-score	precision	recall	support
AFIB	0.730601	0.715847	0.746035	875.0
GSVT	0.754824	0.766825	0.743355	691.0
SB	0.803144	0.803099	0.803195	1553.0
SR	0.783671	0.790812	0.776671	907.0
macro avg	0.768060	0.769146	0.767314	4026.0
micro avg	0.774526	0.774526	0.774526	4026.0
weighted avg	0.774697	0.775142	0.774526	4026.0

The result of Model Number:Dataset Number:Featuregroup Number:Rescale of 77 : 0 : 9 : 1

	f1-score	precision	recall	support
AFIB	0.730595	0.730404	0.730857	875.0
GSVT	0.745857	0.743037	0.748779	691.0
SB	0.794078	0.790492	0.797707	1553.0
SR	0.767316	0.776082	0.758774	907.0
macro avg	0.759462	0.760004	0.759029	4026.0
micro avg	0.766010	0.766010	0.766010	4026.0
weighted avg	0.765975	0.766042	0.766010	4026.0

The result of Model Number:Dataset Number:Featuregroup Number:Rescale of 77 : 0 : 10 : 0

	f1-score	precision	recall	support
AFIB	0.801022	0.784845	0.817944	875.0
GSVT	0.827580	0.840738	0.815005	691.0
SB	0.880557	0.880508	0.880613	1553.0

SR	0.859208	0.867037	0.851533	907.0
macro avg	0.842092	0.843282	0.841274	4026.0
micro avg	0.849181	0.849181	0.849181	4026.0
weighted avg	0.849369	0.849856	0.849181	4026.0

The result of Model Number:Dataset Number:Featuregroup Number:Rescale of 77 : 0 : 10 : 1

	f1-score	precision	recall	support
AFIB	0.862888	0.864868	0.861011	875.0
GSVT	0.865896	0.863455	0.868380	691.0
SB	0.908744	0.901219	0.916408	1553.0
SR	0.881482	0.894711	0.868676	907.0
macro avg	0.879753	0.881063	0.878619	4026.0
micro avg	0.885372	0.885372	0.885372	4026.0
weighted avg	0.885282	0.885371	0.885372	4026.0

The result of Model Number:Dataset Number:Featuregroup Number:Rescale of 77 : 1 : 0 : 0

	f1-score	precision	recall	support
AFIB	0.686223	0.671559	0.701581	875.0
GSVT	0.710627	0.721494	0.700146	691.0
SB	0.757527	0.757627	0.757430	1553.0
SR	0.738011	0.745654	0.730526	907.0
macro avg	0.723097	0.724083	0.722421	4026.0
micro avg	0.729399	0.729399	0.729399	4026.0
weighted avg	0.729584	0.730022	0.729399	4026.0

The result of Model Number:Dataset Number:Featuregroup Number:Rescale of 77 : 1 : 0 : 1

	f1-score	precision	recall	support
AFIB	0.614192	0.619279	0.691803	204.0
GSVT	0.666050	0.637542	0.751460	300.0
SB	0.608128	0.606015	0.610258	892.0
SR	0.602082	0.602195	0.601972	681.0
macro avg	0.622613	0.616258	0.663873	2077.0
micro avg	0.635946	0.635946	0.635946	2077.0
weighted avg	0.615107	0.610619	0.635946	2077.0

The result of Model Number:Dataset Number:Featuregroup Number:Rescale of 77 : 1 : 1 : 0

	f1-score	precision	recall	support
AFIB	0.855371	0.857542	0.853242	875.0
GSVT	0.870466	0.861037	0.880140	691.0
SB	0.917423	0.907045	0.928055	1553.0
SR	0.875477	0.899445	0.852788	907.0
macro avg	0.879684	0.881267	0.878556	4026.0
micro avg	0.886615	0.886615	0.886615	4026.0
weighted avg	0.886428	0.886678	0.886615	4026.0

The result of Model Number:Dataset Number:Featuregroup Number:Rescale of 77 : 1 : 1 : 1

	f1-score	precision	recall	support
AFIB	0.793727	0.805388	0.782471	204.0
GSVT	0.813837	0.806918	0.820893	300.0
SB	0.827903	0.824590	0.831244	892.0
SR	0.819715	0.823738	0.815740	681.0
macro avg	0.813795	0.815159	0.812587	2077.0
micro avg	0.819875	0.819875	0.819875	2077.0
weighted avg	0.819830	0.819872	0.819875	2077.0

The result of Model Number:Dataset Number:Featuregroup Number:Rescale of 77 : 1 : 2 : 0

	f1-score	precision	recall	support
AFIB	0.786758	0.774162	0.885262	204.0
GSVT	0.921086	0.910398	0.932364	300.0
SB	0.951927	0.948333	0.955556	892.0
SR	0.746899	0.750761	0.743204	681.0
macro avg	0.851668	0.845914	0.879096	2077.0
micro avg	0.875677	0.875677	0.875677	2077.0
weighted avg	0.864026	0.860968	0.875677	2077.0

The result of Model Number:Dataset Number:Featuregroup Number:Rescale of 77 : 1 : 2 : 1

	f1-score	precision	recall	support
AFIB	0.848508	0.853165	0.843956	875.0
GSVT	0.849695	0.838952	0.860849	691.0
SB	0.893601	0.890015	0.897223	1553.0
SR	0.874418	0.885033	0.864109	907.0
macro avg	0.866555	0.866791	0.866534	4026.0
micro avg	0.871943	0.871943	0.871943	4026.0
weighted avg	0.871943	0.872120	0.871943	4026.0

The result of Model Number:Dataset Number:Featuregroup Number:Rescale of 77 : 1 : 3 : 0

	f1-score	precision	recall	support
AFIB	0.903374	0.911706	0.895411	204.0
GSVT	0.936401	0.936434	0.936392	300.0
SB	0.956011	0.954313	0.957724	892.0
SR	0.945865	0.945612	0.946142	681.0
macro avg	0.935413	0.937016	0.933917	2077.0
micro avg	0.944725	0.944725	0.944725	2077.0
weighted avg	0.944682	0.944693	0.944725	2077.0

The result of Model Number:Dataset Number:Featuregroup Number:Rescale of 77 : 1 : 3 : 1

	f1-score	precision	recall	support
AFIB	0.634933	0.637525	0.632381	875.0

GSVT 0.591245 0.617339 0.567365 691.0
SB 0.634962 0.575193 0.708601 1553.0
SR 0.478974 0.596128 0.400465 907.0
macro avg 0.585028 0.606546 0.577203 4026.0
micro avg 0.598376 0.598376 0.598376 4026.0
weighted avg 0.592310 0.600690 0.598376 4026.0
The result of Model Number:Dataset Number:Featuregroup Number:Rescale of 77 : 1 : 4 : 0

	f1-score	precision	recall	support
AFIB	0.724756	0.728719	0.720860	875.0
GSVT	0.721302	0.715220	0.727561	691.0
SB	0.758630	0.751445	0.765956	1553.0
SR	0.730396	0.744020	0.717271	907.0
macro avg	0.733771	0.734851	0.732912	4026.0
micro avg	0.738597	0.738597	0.738597	4026.0
weighted avg	0.738500	0.738616	0.738597	4026.0

The result of Model Number:Dataset Number:Featuregroup Number:Rescale of 77 : 1 : 4 : 1

	f1-score	precision	recall	support
AFIB	0.772480	0.779493	0.765988	204.0
GSVT	0.798011	0.793188	0.803101	300.0
SB	0.814706	0.813614	0.815801	892.0
SR	0.807168	0.808841	0.805506	681.0
macro avg	0.798091	0.798784	0.797599	2077.0
micro avg	0.805699	0.805699	0.805699	2077.0
weighted avg	0.805675	0.805747	0.805699	2077.0

The result of Model Number:Dataset Number:Featuregroup Number:Rescale of 77 : 1 : 5 : 0

	f1-score	precision	recall	support
AFIB	0.891992	0.894325	0.889846	204.0
GSVT	0.912117	0.908586	0.915752	300.0
SB	0.932573	0.931329	0.933826	892.0
SR	0.925104	0.927703	0.922523	681.0
macro avg	0.915446	0.915486	0.915487	2077.0
micro avg	0.923190	0.923190	0.923190	2077.0
weighted avg	0.923184	0.923221	0.923190	2077.0

The result of Model Number:Dataset Number:Featuregroup Number:Rescale of 77 : 1 : 5 : 1

	f1-score	precision	recall	support
AFIB	0.898860	0.923531	0.875539	204.0
GSVT	0.919190	0.905068	0.933846	300.0
SB	0.939385	0.930299	0.948653	892.0
SR	0.915643	0.927085	0.904487	681.0
macro avg	0.918269	0.921496	0.915631	2077.0

micro avg 0.924852 0.924852 0.924852 2077.0
weighted avg 0.924703 0.924936 0.924852 2077.0
The result of Model Number:Dataset Number:Featuregroup Number:Rescale of 77 : 1 : 6 : 0

	f1-score	precision	recall	support
AFIB	0.807527	0.813928	0.801246	875.0
GSVT	0.817764	0.814159	0.821429	691.0
SB	0.869596	0.866158	0.873068	1553.0
SR	0.848227	0.850624	0.845885	907.0
macro avg	0.835779	0.836217	0.835407	4026.0
micro avg	0.842471	0.842471	0.842471	4026.0
weighted avg	0.842396	0.842382	0.842471	4026.0

The result of Model Number:Dataset Number:Featuregroup Number:Rescale of 77 : 1 : 6 : 1

	f1-score	precision	recall	support
AFIB	0.691004	0.706023	0.676800	204.0
GSVT	0.719528	0.715284	0.723843	300.0
SB	0.734987	0.733357	0.736632	892.0
SR	0.727619	0.727208	0.728048	681.0
macro avg	0.718284	0.720468	0.716331	2077.0
micro avg	0.726094	0.726094	0.726094	2077.0
weighted avg	0.726018	0.726046	0.726094	2077.0

The result of Model Number:Dataset Number:Featuregroup Number:Rescale of 77 : 1 : 7 : 0

	f1-score	precision	recall	support
AFIB	0.780572	0.784440	0.776825	875.0
GSVT	0.788344	0.789480	0.787331	691.0
SB	0.829838	0.825988	0.833734	1553.0
SR	0.814562	0.816602	0.812577	907.0
macro avg	0.803329	0.804128	0.802617	4026.0
micro avg	0.808635	0.808635	0.808635	4026.0
weighted avg	0.808568	0.808578	0.808635	4026.0

The result of Model Number:Dataset Number:Featuregroup Number:Rescale of 77 : 1 : 7 : 1

	f1-score	precision	recall	support
AFIB	0.777534	0.779507	0.775598	875.0
GSVT	0.791254	0.782683	0.800048	691.0
SB	0.833939	0.824505	0.843603	1553.0
SR	0.795809	0.817597	0.775186	907.0
macro avg	0.799634	0.801073	0.798609	4026.0
micro avg	0.805934	0.805934	0.805934	4026.0
weighted avg	0.805764	0.805991	0.805934	4026.0

The result of Model Number:Dataset Number:Featuregroup Number:Rescale of 77 : 1 : 8 : 0

	f1-score	precision	recall	support
AFIB	0.771312	0.808705	0.737310	204.0
GSVT	0.803435	0.792937	0.814378	300.0
SB	0.825075	0.818801	0.831457	892.0
SR	0.802102	0.804499	0.799755	681.0
macro avg	0.800481	0.806235	0.795725	2077.0
micro avg	0.809349	0.809349	0.809349	2077.0
weighted avg	0.809136	0.809384	0.809349	2077.0

The result of Model Number:Dataset Number:Featuregroup Number:Rescale of 77 : 1 : 8 : 1

	f1-score	precision	recall	support
AFIB	0.816180	0.792666	0.895507	875.0
GSVT	0.899540	0.896667	0.902548	691.0
SB	0.759345	0.748154	0.770937	1553.0
SR	0.909224	0.926971	0.892259	907.0
macro avg	0.846072	0.841114	0.865313	4026.0
micro avg	0.847932	0.847932	0.847932	4026.0
weighted avg	0.829525	0.823603	0.847932	4026.0

The result of Model Number:Dataset Number:Featuregroup Number:Rescale of 77 : 1 : 9 : 0

	f1-score	precision	recall	support
AFIB	0.908147	0.918238	0.898741	204.0
GSVT	0.936278	0.930948	0.941941	300.0
SB	0.954904	0.953624	0.956188	892.0
SR	0.946780	0.948049	0.945528	681.0
macro avg	0.936527	0.937715	0.935600	2077.0
micro avg	0.944993	0.944993	0.944993	2077.0
weighted avg	0.944958	0.945045	0.944993	2077.0

The result of Model Number:Dataset Number:Featuregroup Number:Rescale of 77 : 1 : 9 : 1

	f1-score	precision	recall	support
AFIB	0.800485	0.791547	0.809635	875.0
GSVT	0.816411	0.823889	0.809080	691.0
SB	0.870740	0.867904	0.873598	1553.0
SR	0.848167	0.856664	0.839840	907.0
macro avg	0.833951	0.835001	0.833038	4026.0
micro avg	0.841018	0.841018	0.841018	4026.0
weighted avg	0.841061	0.841222	0.841018	4026.0

The result of Model Number:Dataset Number:Featuregroup Number:Rescale of 77 : 1 : 10 : 0

	f1-score	precision	recall	support
AFIB	0.792084	0.791136	0.793168	875.0
GSVT	0.807524	0.810302	0.804848	691.0
SB	0.855288	0.850019	0.860629	1553.0

SR 0.837883 0.846066 0.829949 907.0
macro avg 0.823195 0.824381 0.822149 4026.0
micro avg 0.829482 0.829482 0.829482 4026.0
weighted avg 0.829432 0.829514 0.829482 4026.0
The result of Model Number:Dataset Number:Featuregroup Number:Rescale of 77 : 1 : 10 : 1

	f1-score	precision	recall	support
AFIB	0.838291	0.857248	0.820180	875.0
GSVT	0.888779	0.892562	0.885081	691.0
SB	0.965030	0.960482	0.969627	1553.0
SR	0.914353	0.900328	0.928860	907.0
macro avg	0.901614	0.902655	0.900937	4026.0
micro avg	0.913452	0.913452	0.913452	4026.0
weighted avg	0.912981	0.912836	0.913452	4026.0

The result of Model Number:Dataset Number:Featuregroup Number:Rescale of 78 : 0 : 0 : 0

	f1-score	precision	recall	support
AFIB	0.713348	0.729091	0.698324	204.0
GSVT	0.728938	0.716850	0.741528	300.0
SB	0.742945	0.737134	0.748857	892.0
SR	0.724915	0.733640	0.716396	681.0
macro avg	0.727536	0.729179	0.726276	2077.0
micro avg	0.732192	0.732192	0.732192	2077.0
weighted avg	0.732103	0.732269	0.732192	2077.0

The result of Model Number:Dataset Number:Featuregroup Number:Rescale of 78 : 0 : 0 : 1

	f1-score	precision	recall	support
AFIB	0.793569	0.795583	0.791593	875.0
GSVT	0.807572	0.798825	0.816548	691.0
SB	0.851137	0.841509	0.861001	1553.0
SR	0.812221	0.834458	0.791172	907.0
macro avg	0.816125	0.817594	0.815079	4026.0
micro avg	0.822555	0.822555	0.822555	4026.0
weighted avg	0.822381	0.822613	0.822555	4026.0

The result of Model Number:Dataset Number:Featuregroup Number:Rescale of 78 : 0 : 1 : 0

	f1-score	precision	recall	support
AFIB	0.805438	0.825525	0.786366	875.0
GSVT	0.856846	0.860130	0.853626	691.0
SB	0.927892	0.923399	0.932431	1553.0
SR	0.879229	0.864574	0.894446	907.0
macro avg	0.867351	0.868407	0.866717	4026.0
micro avg	0.878602	0.878602	0.878602	4026.0
weighted avg	0.878121	0.878016	0.878602	4026.0

The result of Model Number:Dataset Number:Featuregroup Number:Rescale of 78 : 0 : 1 : 1

	f1-score	precision	recall	support
AFIB	0.704987	0.763839	0.655960	204.0
GSVT	0.756180	0.746671	0.766242	300.0
SB	0.775484	0.764620	0.786752	892.0
SR	0.754330	0.757864	0.751197	681.0
macro avg	0.747745	0.758249	0.740038	2077.0
micro avg	0.759286	0.759286	0.759286	2077.0
weighted avg	0.758836	0.759736	0.759286	2077.0

The result of Model Number:Dataset Number:Featuregroup Number:Rescale of 78 : 0 : 2 : 0

	f1-score	precision	recall	support
AFIB	0.788833	0.789720	0.788091	204.0
GSVT	0.806944	0.805142	0.808815	300.0
SB	0.824048	0.822948	0.825155	892.0
SR	0.817876	0.819936	0.815837	681.0
macro avg	0.809425	0.809436	0.809474	2077.0
micro avg	0.816099	0.816099	0.816099	2077.0
weighted avg	0.816095	0.816125	0.816099	2077.0

The result of Model Number:Dataset Number:Featuregroup Number:Rescale of 78 : 0 : 2 : 1

	f1-score	precision	recall	support
AFIB	0.859881	0.852993	0.866981	875.0
GSVT	0.883628	0.885136	0.882229	691.0
SB	0.936786	0.934275	0.939318	1553.0
SR	0.911190	0.921797	0.900844	907.0
macro avg	0.897871	0.898550	0.897343	4026.0
micro avg	0.905130	0.905130	0.905130	4026.0
weighted avg	0.905181	0.905364	0.905130	4026.0

The result of Model Number:Dataset Number:Featuregroup Number:Rescale of 78 : 0 : 3 : 0

	f1-score	precision	recall	support
AFIB	0.862788	0.852722	0.875345	875.0
GSVT	0.887757	0.876996	0.899500	691.0
SB	0.901138	0.866354	0.952160	1553.0
SR	0.715550	0.753295	0.681451	907.0
macro avg	0.841808	0.837342	0.852114	4026.0
micro avg	0.865440	0.865440	0.865440	4026.0
weighted avg	0.848696	0.839747	0.865440	4026.0

The result of Model Number:Dataset Number:Featuregroup Number:Rescale of 78 : 0 : 3 : 1

	f1-score	precision	recall	support
AFIB	0.662955	0.663546	0.662432	875.0

GSVT 0.675098 0.676869 0.673450 691.0
SB 0.716626 0.711854 0.721469 1553.0
SR 0.702663 0.709078 0.696388 907.0
macro avg 0.689335 0.690337 0.688435 4026.0
micro avg 0.694746 0.694746 0.694746 4026.0
weighted avg 0.694688 0.694725 0.694746 4026.0
The result of Model Number:Dataset Number:Featuregroup Number:Rescale of 78 : 0 : 4 : 0

	f1-score	precision	recall	support
AFIB	0.781303	0.790439	0.772521	204.0
GSVT	0.704739	0.677833	0.804097	300.0
SB	0.653854	0.652313	0.655408	892.0
SR	0.811866	0.813426	0.810317	681.0
macro avg	0.737940	0.733503	0.760586	2077.0
micro avg	0.739178	0.739178	0.739178	2077.0
weighted avg	0.725530	0.722391	0.739178	2077.0

The result of Model Number:Dataset Number:Featuregroup Number:Rescale of 78 : 0 : 4 : 1

	f1-score	precision	recall	support
AFIB	0.751764	0.753110	0.750446	875.0
GSVT	0.719864	0.723646	0.716142	691.0
SB	0.761442	0.719555	0.808513	1553.0
SR	0.632381	0.711791	0.568967	907.0
macro avg	0.716363	0.727026	0.711017	4026.0
micro avg	0.726073	0.726073	0.726073	4026.0
weighted avg	0.723127	0.725801	0.726073	4026.0

The result of Model Number:Dataset Number:Featuregroup Number:Rescale of 78 : 0 : 5 : 0

	f1-score	precision	recall	support
AFIB	0.725690	0.739752	0.712478	204.0
GSVT	0.753830	0.747527	0.760379	300.0
SB	0.767896	0.766355	0.769445	892.0
SR	0.760574	0.761362	0.759791	681.0
macro avg	0.751997	0.753749	0.750523	2077.0
micro avg	0.759375	0.759375	0.759375	2077.0
weighted avg	0.759318	0.759385	0.759375	2077.0

The result of Model Number:Dataset Number:Featuregroup Number:Rescale of 78 : 0 : 5 : 1

	f1-score	precision	recall	support
AFIB	0.741986	0.735109	0.749034	875.0
GSVT	0.755311	0.759412	0.751266	691.0
SB	0.800295	0.798450	0.802149	1553.0
SR	0.781649	0.788862	0.774591	907.0
macro avg	0.769810	0.770458	0.769260	4026.0

micro avg 0.775663 0.775663 0.775663 4026.0
weighted avg 0.775700 0.775823 0.775663 4026.0
The result of Model Number:Dataset Number:Featuregroup Number:Rescale of 78 : 0 : 6 : 0

	f1-score	precision	recall	support
AFIB	0.855400	0.842284	0.869073	875.0
GSVT	0.884895	0.893569	0.876563	691.0
SB	0.941765	0.939565	0.944005	1553.0
SR	0.918239	0.929882	0.906904	907.0
macro avg	0.900075	0.901325	0.899136	4026.0
micro avg	0.907786	0.907786	0.907786	4026.0
weighted avg	0.907934	0.908346	0.907786	4026.0

The result of Model Number:Dataset Number:Featuregroup Number:Rescale of 78 : 0 : 6 : 1

	f1-score	precision	recall	support
AFIB	0.871928	0.879200	0.865075	204.0
GSVT	0.894961	0.890840	0.899140	300.0
SB	0.908458	0.905231	0.911714	892.0
SR	0.900068	0.904099	0.896119	681.0
macro avg	0.893854	0.894842	0.893012	2077.0
micro avg	0.900204	0.900204	0.900204	2077.0
weighted avg	0.900170	0.900224	0.900204	2077.0

The result of Model Number:Dataset Number:Featuregroup Number:Rescale of 78 : 0 : 7 : 0

	f1-score	precision	recall	support
AFIB	0.677712	0.675069	0.680424	875.0
GSVT	0.690395	0.691851	0.688986	691.0
SB	0.732878	0.731051	0.734718	1553.0
SR	0.715972	0.720781	0.711242	907.0
macro avg	0.704239	0.704688	0.703842	4026.0
micro avg	0.709780	0.709780	0.709780	4026.0
weighted avg	0.709788	0.709842	0.709780	4026.0

The result of Model Number:Dataset Number:Featuregroup Number:Rescale of 78 : 0 : 7 : 1

	f1-score	precision	recall	support
AFIB	0.744142	0.749008	0.739385	204.0
GSVT	0.762786	0.761029	0.764561	300.0
SB	0.776402	0.775282	0.777532	892.0
SR	0.770283	0.771088	0.769494	681.0
macro avg	0.763404	0.764102	0.762743	2077.0
micro avg	0.769276	0.769276	0.769276	2077.0
weighted avg	0.769261	0.769268	0.769276	2077.0

The result of Model Number:Dataset Number:Featuregroup Number:Rescale of 78 : 0 : 8 : 0

	f1-score	precision	recall	support
AFIB	0.705259	0.707897	0.702941	875.0
GSVT	0.721134	0.728091	0.714454	691.0
SB	0.766791	0.758749	0.775065	1553.0
SR	0.740238	0.746753	0.734091	907.0
macro avg	0.733356	0.735373	0.731638	4026.0
micro avg	0.739756	0.739756	0.739756	4026.0
weighted avg	0.739600	0.739733	0.739756	4026.0

The result of Model Number:Dataset Number:Featuregroup Number:Rescale of 78 : 0 : 8 : 1

	f1-score	precision	recall	support
AFIB	0.773864	0.759398	0.788975	875.0
GSVT	0.800971	0.813616	0.788813	691.0
SB	0.851799	0.850277	0.853334	1553.0
SR	0.830148	0.838943	0.821540	907.0
macro avg	0.814195	0.815558	0.813166	4026.0
micro avg	0.821110	0.821110	0.821110	4026.0
weighted avg	0.821259	0.821680	0.821110	4026.0

The result of Model Number:Dataset Number:Featuregroup Number:Rescale of 78 : 0 : 9 : 0

	f1-score	precision	recall	support
AFIB	0.597132	0.609681	0.585113	875.0
GSVT	0.635876	0.638060	0.633738	691.0
SB	0.692796	0.689531	0.696095	1553.0
SR	0.655809	0.647075	0.664836	907.0
macro avg	0.645403	0.646087	0.644945	4026.0
micro avg	0.654230	0.654230	0.654230	4026.0
weighted avg	0.653902	0.653778	0.654230	4026.0

The result of Model Number:Dataset Number:Featuregroup Number:Rescale of 78 : 0 : 9 : 1

	f1-score	precision	recall	support
AFIB	0.737757	0.746384	0.729465	204.0
GSVT	0.665461	0.640054	0.759281	300.0
SB	0.617411	0.615956	0.618879	892.0
SR	0.766616	0.768090	0.765154	681.0
macro avg	0.696811	0.692621	0.718195	2077.0
micro avg	0.697980	0.697980	0.697980	2077.0
weighted avg	0.685093	0.682129	0.697980	2077.0

The result of Model Number:Dataset Number:Featuregroup Number:Rescale of 78 : 0 : 10 : 0

	f1-score	precision	recall	support
AFIB	0.878680	0.885057	0.872457	875.0
GSVT	0.897954	0.889608	0.906526	691.0
SB	0.965037	0.959211	0.970941	1553.0

SR 0.933409 0.943832 0.923218 907.0
macro avg 0.918770 0.919427 0.918285 4026.0
micro avg 0.927729 0.927729 0.927729 4026.0
weighted avg 0.927630 0.927684 0.927729 4026.0
The result of Model Number:Dataset Number:Featuregroup Number:Rescale of 78 : 0 : 10 : 1

	f1-score	precision	recall	support
AFIB	0.759674	0.748994	0.770701	875.0
GSVT	0.776030	0.781853	0.770314	691.0
SB	0.825219	0.822794	0.827662	1553.0
SR	0.804892	0.816027	0.794070	907.0
macro avg	0.791454	0.792417	0.790687	4026.0
micro avg	0.797871	0.797871	0.797871	4026.0
weighted avg	0.797952	0.798203	0.797871	4026.0

The result of Model Number:Dataset Number:Featuregroup Number:Rescale of 78 : 1 : 0 : 0

	f1-score	precision	recall	support
AFIB	0.798002	0.816187	0.780736	875.0
GSVT	0.849176	0.850854	0.847788	691.0
SB	0.929055	0.923970	0.934199	1553.0
SR	0.878096	0.867445	0.889135	907.0
macro avg	0.863582	0.864614	0.862965	4026.0
micro avg	0.875862	0.875862	0.875862	4026.0
weighted avg	0.875382	0.875261	0.875862	4026.0

The result of Model Number:Dataset Number:Featuregroup Number:Rescale of 78 : 1 : 0 : 1

	f1-score	precision	recall	support
AFIB	0.919865	0.936747	0.904024	204.0
GSVT	0.954784	0.947107	0.962762	300.0
SB	0.972496	0.970760	0.974240	892.0
SR	0.962867	0.963724	0.962016	681.0
macro avg	0.952503	0.954585	0.950761	2077.0
micro avg	0.961678	0.961678	0.961678	2077.0
weighted avg	0.961611	0.961696	0.961678	2077.0

The result of Model Number:Dataset Number:Featuregroup Number:Rescale of 78 : 1 : 1 : 0

	f1-score	precision	recall	support
AFIB	0.851352	0.864259	0.838846	204.0
GSVT	0.865041	0.857749	0.872504	300.0
SB	0.884229	0.881377	0.887103	892.0
SR	0.874737	0.877987	0.871536	681.0
macro avg	0.868840	0.870343	0.867497	2077.0
micro avg	0.875150	0.875150	0.875150	2077.0
weighted avg	0.875116	0.875171	0.875150	2077.0

The result of Model Number:Dataset Number:Featuregroup Number:Rescale of 78 : 1 : 1 : 1

	f1-score	precision	recall	support
AFIB	0.772672	0.780147	0.765480	204.0
GSVT	0.800014	0.798437	0.801610	300.0
SB	0.816736	0.815289	0.818198	892.0
SR	0.809206	0.809598	0.808849	681.0
macro avg	0.799657	0.800867	0.798534	2077.0
micro avg	0.807559	0.807559	0.807559	2077.0
weighted avg	0.807524	0.807537	0.807559	2077.0

The result of Model Number:Dataset Number:Featuregroup Number:Rescale of 78 : 1 : 2 : 0

	f1-score	precision	recall	support
AFIB	0.760388	0.754572	0.766395	875.0
GSVT	0.777884	0.782137	0.773722	691.0
SB	0.823716	0.823139	0.824300	1553.0
SR	0.801837	0.805726	0.798026	907.0
macro avg	0.790956	0.791393	0.790611	4026.0
micro avg	0.797115	0.797115	0.797115	4026.0
weighted avg	0.797157	0.797277	0.797115	4026.0

The result of Model Number:Dataset Number:Featuregroup Number:Rescale of 78 : 1 : 2 : 1

	f1-score	precision	recall	support
AFIB	0.819802	0.825325	0.814473	875.0
GSVT	0.834432	0.822842	0.846507	691.0
SB	0.889178	0.883090	0.895359	1553.0
SR	0.853145	0.867882	0.838947	907.0
macro avg	0.849139	0.849785	0.848821	4026.0
micro avg	0.856686	0.856686	0.856686	4026.0
weighted avg	0.856586	0.856769	0.856686	4026.0

The result of Model Number:Dataset Number:Featuregroup Number:Rescale of 78 : 1 : 3 : 0

	f1-score	precision	recall	support
AFIB	0.761532	0.762211	0.760932	875.0
GSVT	0.775481	0.777515	0.773587	691.0
SB	0.823184	0.817703	0.828747	1553.0
SR	0.807144	0.814514	0.799937	907.0
macro avg	0.791835	0.792986	0.790801	4026.0
micro avg	0.798050	0.798050	0.798050	4026.0
weighted avg	0.797983	0.798026	0.798050	4026.0

The result of Model Number:Dataset Number:Featuregroup Number:Rescale of 78 : 1 : 3 : 1

	f1-score	precision	recall	support
AFIB	0.766179	0.776019	0.756623	875.0

GSVT 0.774655 0.768390 0.781146 691.0
SB 0.828659 0.826168 0.831168 1553.0
SR 0.808140 0.807800 0.808490 907.0
macro avg 0.794408 0.794594 0.794357 4026.0
micro avg 0.801272 0.801272 0.801272 4026.0
weighted avg 0.801188 0.801214 0.801272 4026.0
The result of Model Number:Dataset Number:Featuregroup Number:Rescale of 78 : 1 : 4 : 0

	f1-score	precision	recall	support
AFIB	0.793837	0.797600	0.790114	875.0
GSVT	0.803684	0.799892	0.807530	691.0
SB	0.847699	0.845910	0.849501	1553.0
SR	0.831635	0.833964	0.829339	907.0
macro avg	0.819214	0.819341	0.819121	4026.0
micro avg	0.824848	0.824848	0.824848	4026.0
weighted avg	0.824819	0.824821	0.824848	4026.0

The result of Model Number:Dataset Number:Featuregroup Number:Rescale of 78 : 1 : 4 : 1

	f1-score	precision	recall	support
AFIB	0.747353	0.754247	0.740766	204.0
GSVT	0.774676	0.774704	0.774669	300.0
SB	0.790900	0.789495	0.792317	892.0
SR	0.782506	0.782297	0.782735	681.0
macro avg	0.773859	0.775186	0.772622	2077.0
micro avg	0.781563	0.781563	0.781563	2077.0
weighted avg	0.781528	0.781537	0.781563	2077.0

The result of Model Number:Dataset Number:Featuregroup Number:Rescale of 78 : 1 : 5 : 0

	f1-score	precision	recall	support
AFIB	0.853611	0.853387	0.853916	875.0
GSVT	0.871442	0.868147	0.874856	691.0
SB	0.927782	0.923593	0.932023	1553.0
SR	0.896514	0.906757	0.886534	907.0
macro avg	0.887337	0.887971	0.886832	4026.0
micro avg	0.894988	0.894988	0.894988	4026.0
weighted avg	0.894948	0.895025	0.894988	4026.0

The result of Model Number:Dataset Number:Featuregroup Number:Rescale of 78 : 1 : 5 : 1

	f1-score	precision	recall	support
AFIB	0.730844	0.744848	0.717544	204.0
GSVT	0.754085	0.750201	0.758080	300.0
SB	0.772734	0.771531	0.773947	892.0
SR	0.765120	0.764360	0.765906	681.0
macro avg	0.755696	0.757735	0.753869	2077.0

micro avg	0.763479	0.763479	0.763479	2077.0
weighted avg	0.763430	0.763478	0.763479	2077.0

The result of Model Number:Dataset Number:Featuregroup Number:Rescale of 78 : 1 : 6 : 0

	f1-score	precision	recall	support
AFIB	0.883757	0.881525	0.886139	875.0
GSVT	0.900999	0.896045	0.906056	691.0
SB	0.943417	0.937729	0.949187	1553.0
SR	0.915601	0.932126	0.899668	907.0
macro avg	0.910943	0.911856	0.910263	4026.0
micro avg	0.916926	0.916926	0.916926	4026.0
weighted avg	0.916904	0.917097	0.916926	4026.0

The result of Model Number:Dataset Number:Featuregroup Number:Rescale of 78 : 1 : 6 : 1

	f1-score	precision	recall	support
AFIB	0.705951	0.716213	0.696299	204.0
GSVT	0.721520	0.711759	0.731611	300.0
SB	0.732379	0.724747	0.740180	892.0
SR	0.717864	0.729743	0.706377	681.0
macro avg	0.719429	0.720615	0.718617	2077.0
micro avg	0.723549	0.723549	0.723549	2077.0
weighted avg	0.723456	0.723671	0.723549	2077.0

The result of Model Number:Dataset Number:Featuregroup Number:Rescale of 78 : 1 : 7 : 0

	f1-score	precision	recall	support
AFIB	0.773877	0.779494	0.768396	875.0
GSVT	0.790852	0.783502	0.798402	691.0
SB	0.849934	0.844803	0.855133	1553.0
SR	0.822079	0.831258	0.813103	907.0
macro avg	0.809186	0.809764	0.808759	4026.0
micro avg	0.817076	0.817076	0.817076	4026.0
weighted avg	0.816988	0.817036	0.817076	4026.0

The result of Model Number:Dataset Number:Featuregroup Number:Rescale of 78 : 1 : 7 : 1

	f1-score	precision	recall	support
AFIB	0.719258	0.732476	0.706597	204.0
GSVT	0.731340	0.726550	0.736212	300.0
SB	0.743850	0.740218	0.747519	892.0
SR	0.736161	0.739222	0.733136	681.0
macro avg	0.732652	0.734617	0.730866	2077.0
micro avg	0.737151	0.737151	0.737151	2077.0
weighted avg	0.737107	0.737157	0.737151	2077.0

The result of Model Number:Dataset Number:Featuregroup Number:Rescale of 78 : 1 : 8 : 0

	f1-score	precision	recall	support
AFIB	0.560530	0.619159	0.512208	204.0
GSVT	0.659425	0.650718	0.668438	300.0
SB	0.699317	0.696600	0.702061	892.0
SR	0.682513	0.672786	0.692533	681.0
macro avg	0.650446	0.659816	0.643810	2077.0
micro avg	0.675433	0.675433	0.675433	2077.0
weighted avg	0.674414	0.674558	0.675433	2077.0

The result of Model Number:Dataset Number:Featuregroup Number:Rescale of 78 : 1 : 8 : 1

	f1-score	precision	recall	support
AFIB	0.860480	0.857993	0.863061	875.0
GSVT	0.876428	0.875715	0.877203	691.0
SB	0.930894	0.927038	0.934789	1553.0
SR	0.902947	0.912867	0.893290	907.0
macro avg	0.892687	0.893403	0.892086	4026.0
micro avg	0.899967	0.899967	0.899967	4026.0
weighted avg	0.899946	0.900031	0.899967	4026.0

The result of Model Number:Dataset Number:Featuregroup Number:Rescale of 78 : 1 : 9 : 0

	f1-score	precision	recall	support
AFIB	0.527557	0.574691	0.557112	204.0
GSVT	0.716569	0.704855	0.728925	300.0
SB	0.610021	0.605375	0.614785	892.0
SR	0.745956	0.737516	0.754614	681.0
macro avg	0.650026	0.655609	0.663859	2077.0
micro avg	0.671453	0.671453	0.671453	2077.0
weighted avg	0.661881	0.660056	0.671453	2077.0

The result of Model Number:Dataset Number:Featuregroup Number:Rescale of 78 : 1 : 9 : 1

	f1-score	precision	recall	support
AFIB	0.864732	0.887582	0.843113	204.0
GSVT	0.888446	0.881066	0.896114	300.0
SB	0.912928	0.909578	0.916305	892.0
SR	0.898793	0.899882	0.897730	681.0
macro avg	0.891225	0.894527	0.888316	2077.0
micro avg	0.900110	0.900110	0.900110	2077.0
weighted avg	0.900024	0.900120	0.900110	2077.0

The result of Model Number:Dataset Number:Featuregroup Number:Rescale of 78 : 1 : 10 : 0

	f1-score	precision	recall	support
AFIB	0.880240	0.880010	0.880556	875.0
GSVT	0.898628	0.895231	0.902148	691.0
SB	0.956725	0.952406	0.961099	1553.0

SR 0.924482 0.935044 0.914191 907.0
macro avg 0.915019 0.915673 0.914499 4026.0
micro avg 0.922908 0.922908 0.922908 4026.0
weighted avg 0.922867 0.922947 0.922908 4026.0
The result of Model Number:Dataset Number:Featuregroup Number:Rescale of 78 : 1 : 10 : 1

	f1-score	precision	recall	support
AFIB	0.763102	0.778410	0.748536	204.0
GSVT	0.780787	0.772671	0.789117	300.0
SB	0.795227	0.792134	0.798347	892.0
SR	0.786871	0.790154	0.783633	681.0
macro avg	0.781497	0.783342	0.779908	2077.0
micro avg	0.787297	0.787297	0.787297	2077.0
weighted avg	0.787246	0.787326	0.787297	2077.0

The result of Model Number:Dataset Number:Featuregroup Number:Rescale of 79 : 0 : 0 : 0

	f1-score	precision	recall	support
AFIB	0.798385	0.800411	0.796397	875.0
GSVT	0.812473	0.803672	0.821503	691.0
SB	0.856302	0.846616	0.866226	1553.0
SR	0.817150	0.839522	0.795974	907.0
macro avg	0.821078	0.822555	0.820025	4026.0
micro avg	0.827547	0.827547	0.827547	4026.0
weighted avg	0.827372	0.827605	0.827547	4026.0

The result of Model Number:Dataset Number:Featuregroup Number:Rescale of 79 : 0 : 0 : 1

	f1-score	precision	recall	support
AFIB	0.651577	0.710589	0.602700	204.0
GSVT	0.756274	0.747632	0.765350	300.0
SB	0.801983	0.799930	0.804051	892.0
SR	0.782348	0.771753	0.793259	681.0
macro avg	0.748045	0.757476	0.741340	2077.0
micro avg	0.775146	0.775146	0.775146	2077.0
weighted avg	0.774170	0.774363	0.775146	2077.0

The result of Model Number:Dataset Number:Featuregroup Number:Rescale of 79 : 0 : 1 : 0

	f1-score	precision	recall	support
AFIB	0.775024	0.775575	0.774489	875.0
GSVT	0.785468	0.787017	0.783992	691.0
SB	0.834299	0.831526	0.837092	1553.0
SR	0.814695	0.817723	0.811716	907.0
macro avg	0.802371	0.802960	0.801822	4026.0
micro avg	0.808656	0.808656	0.808656	4026.0
weighted avg	0.808619	0.808617	0.808656	4026.0

The result of Model Number:Dataset Number:Featuregroup Number:Rescale of 79 : 0 : 1 : 1

	f1-score	precision	recall	support
AFIB	0.712198	0.724564	0.700338	204.0
GSVT	0.726232	0.724083	0.728410	300.0
SB	0.734796	0.732182	0.737432	892.0
SR	0.728822	0.729583	0.728079	681.0
macro avg	0.725512	0.727603	0.723565	2077.0
micro avg	0.729419	0.729419	0.729419	2077.0
weighted avg	0.729381	0.729412	0.729419	2077.0

The result of Model Number:Dataset Number:Featuregroup Number:Rescale of 79 : 0 : 2 : 0

	f1-score	precision	recall	support
AFIB	0.897317	0.916820	0.878873	204.0
GSVT	0.934358	0.928846	0.939961	300.0
SB	0.954432	0.952315	0.956569	892.0
SR	0.944864	0.944331	0.945421	681.0
macro avg	0.932743	0.935578	0.930206	2077.0
micro avg	0.942884	0.942884	0.942884	2077.0
weighted avg	0.942786	0.942821	0.942884	2077.0

The result of Model Number:Dataset Number:Featuregroup Number:Rescale of 79 : 0 : 2 : 1

	f1-score	precision	recall	support
AFIB	0.801596	0.829424	0.775682	204.0
GSVT	0.816966	0.808022	0.826210	300.0
SB	0.838714	0.832692	0.844826	892.0
SR	0.823735	0.827786	0.819739	681.0
macro avg	0.820253	0.824481	0.816614	2077.0
micro avg	0.827120	0.827120	0.827120	2077.0
weighted avg	0.827016	0.827199	0.827120	2077.0

The result of Model Number:Dataset Number:Featuregroup Number:Rescale of 79 : 0 : 3 : 0

	f1-score	precision	recall	support
AFIB	0.889349	0.912632	0.867630	204.0
GSVT	0.836816	0.861137	0.814287	300.0
SB	0.883020	0.851931	0.916480	892.0
SR	0.800350	0.826414	0.776040	681.0
macro avg	0.852384	0.863028	0.843609	2077.0
micro avg	0.850874	0.850874	0.850874	2077.0
weighted avg	0.849862	0.850856	0.850874	2077.0

The result of Model Number:Dataset Number:Featuregroup Number:Rescale of 79 : 0 : 3 : 1

	f1-score	precision	recall	support
AFIB	0.873633	0.883341	0.864585	204.0

GSVT 0.900695 0.895567 0.906143 300.0
SB 0.918613 0.917382 0.919848 892.0
SR 0.910798 0.912018 0.909593 681.0
macro avg 0.900935 0.902077 0.900042 2077.0
micro avg 0.909078 0.909078 0.909078 2077.0
weighted avg 0.909045 0.909129 0.909078 2077.0
The result of Model Number:Dataset Number:Featuregroup Number:Rescale of 79 : 0 : 4 : 0

	f1-score	precision	recall	support
AFIB	0.624817	0.640400	0.610022	875.0
GSVT	0.664696	0.667244	0.662198	691.0
SB	0.719810	0.716325	0.723331	1553.0
SR	0.682060	0.670691	0.693864	907.0
macro avg	0.672846	0.673665	0.672354	4026.0
micro avg	0.681574	0.681574	0.681574	4026.0
weighted avg	0.681200	0.681119	0.681574	4026.0

The result of Model Number:Dataset Number:Featuregroup Number:Rescale of 79 : 0 : 4 : 1

	f1-score	precision	recall	support
AFIB	0.795242	0.792943	0.797627	875.0
GSVT	0.809981	0.809323	0.810698	691.0
SB	0.860318	0.856754	0.863918	1553.0
SR	0.834490	0.843658	0.825565	907.0
macro avg	0.825008	0.825669	0.824452	4026.0
micro avg	0.831736	0.831736	0.831736	4026.0
weighted avg	0.831716	0.831794	0.831736	4026.0

The result of Model Number:Dataset Number:Featuregroup Number:Rescale of 79 : 0 : 5 : 0

	f1-score	precision	recall	support
AFIB	0.728006	0.719512	0.738601	875.0
GSVT	0.749074	0.739994	0.758983	691.0
SB	0.760365	0.731015	0.803416	1553.0
SR	0.603769	0.635617	0.574996	907.0
macro avg	0.710304	0.706535	0.718999	4026.0
micro avg	0.730243	0.730243	0.730243	4026.0
weighted avg	0.716115	0.708564	0.730243	4026.0

The result of Model Number:Dataset Number:Featuregroup Number:Rescale of 79 : 0 : 5 : 1

	f1-score	precision	recall	support
AFIB	0.744337	0.732663	0.756469	875.0
GSVT	0.717217	0.737779	0.697854	691.0
SB	0.753676	0.715472	0.796234	1553.0
SR	0.612358	0.681430	0.556095	907.0
macro avg	0.706897	0.716836	0.701663	4026.0

	f1-score	precision	recall	support
micro avg	0.716607	0.716607	0.716607	4026.0
weighted avg	0.713552	0.715367	0.716607	4026.0

The result of Model Number:Dataset Number:Featuregroup Number:Rescale of 79 : 0 : 6 : 0

	f1-score	precision	recall	support
AFIB	0.837965	0.858863	0.818123	875.0
GSVT	0.891448	0.894866	0.888098	691.0
SB	0.965364	0.960690	0.970086	1553.0
SR	0.914735	0.899489	0.930567	907.0
macro avg	0.902378	0.903477	0.901719	4026.0
micro avg	0.914084	0.914084	0.914084	4026.0
weighted avg	0.913583	0.913474	0.914084	4026.0

The result of Model Number:Dataset Number:Featuregroup Number:Rescale of 79 : 0 : 6 : 1

	f1-score	precision	recall	support
AFIB	0.690088	0.721250	0.661733	204.0
GSVT	0.714090	0.706071	0.722425	300.0
SB	0.686928	0.656980	0.735023	892.0
SR	0.572306	0.577185	0.567605	681.0
macro avg	0.665853	0.665371	0.671697	2077.0
micro avg	0.671113	0.671113	0.671113	2077.0
weighted avg	0.653580	0.644220	0.671113	2077.0

The result of Model Number:Dataset Number:Featuregroup Number:Rescale of 79 : 0 : 7 : 0

	f1-score	precision	recall	support
AFIB	0.853665	0.844134	0.863424	875.0
GSVT	0.870649	0.878624	0.862832	691.0
SB	0.928588	0.925564	0.931635	1553.0
SR	0.904515	0.913576	0.895635	907.0
macro avg	0.889354	0.890474	0.888381	4026.0
micro avg	0.896891	0.896891	0.896891	4026.0
weighted avg	0.896937	0.897109	0.896891	4026.0

The result of Model Number:Dataset Number:Featuregroup Number:Rescale of 79 : 0 : 7 : 1

	f1-score	precision	recall	support
AFIB	0.802082	0.805884	0.798320	875.0
GSVT	0.812032	0.808201	0.815918	691.0
SB	0.856504	0.854696	0.858324	1553.0
SR	0.840273	0.842626	0.837953	907.0
macro avg	0.827723	0.827852	0.827629	4026.0
micro avg	0.833415	0.833415	0.833415	4026.0
weighted avg	0.833386	0.833388	0.833415	4026.0

The result of Model Number:Dataset Number:Featuregroup Number:Rescale of 79 : 0 : 8 : 0

	f1-score	precision	recall	support
AFIB	0.778284	0.795884	0.761469	875.0
GSVT	0.825158	0.828670	0.821725	691.0
SB	0.895950	0.891728	0.900218	1553.0
SR	0.848901	0.835879	0.862370	907.0
macro avg	0.837073	0.838040	0.836445	4026.0
micro avg	0.848064	0.848064	0.848064	4026.0
weighted avg	0.847627	0.847493	0.848064	4026.0

The result of Model Number:Dataset Number:Featuregroup Number:Rescale of 79 : 0 : 8 : 1

	f1-score	precision	recall	support
AFIB	0.772261	0.783652	0.761279	204.0
GSVT	0.791219	0.788113	0.794374	300.0
SB	0.808791	0.807169	0.810423	892.0
SR	0.803054	0.803175	0.802941	681.0
macro avg	0.793831	0.795527	0.792254	2077.0
micro avg	0.800825	0.800825	0.800825	2077.0
weighted avg	0.800784	0.800797	0.800825	2077.0

The result of Model Number:Dataset Number:Featuregroup Number:Rescale of 79 : 0 : 9 : 0

	f1-score	precision	recall	support
AFIB	0.668371	0.675643	0.661448	204.0
GSVT	0.690989	0.688108	0.694007	300.0
SB	0.705149	0.704518	0.705781	892.0
SR	0.698485	0.698491	0.698485	681.0
macro avg	0.690748	0.691690	0.689930	2077.0
micro avg	0.697334	0.697334	0.697334	2077.0
weighted avg	0.697306	0.697336	0.697334	2077.0

The result of Model Number:Dataset Number:Featuregroup Number:Rescale of 79 : 0 : 9 : 1

	f1-score	precision	recall	support
AFIB	0.735960	0.737279	0.734670	875.0
GSVT	0.704731	0.708433	0.701088	691.0
SB	0.745435	0.704429	0.791517	1553.0
SR	0.619087	0.696828	0.557006	907.0
macro avg	0.701303	0.711742	0.696070	4026.0
micro avg	0.710809	0.710809	0.710809	4026.0
weighted avg	0.707925	0.710543	0.710809	4026.0

The result of Model Number:Dataset Number:Featuregroup Number:Rescale of 79 : 0 : 10 : 0

	f1-score	precision	recall	support
AFIB	0.843845	0.852065	0.835988	204.0
GSVT	0.863359	0.859395	0.867389	300.0
SB	0.875290	0.872279	0.878329	892.0

SR 0.867898 0.871265 0.864598 681.0
macro avg 0.862598 0.863751 0.861576 2077.0
micro avg 0.868088 0.868088 0.868088 2077.0
weighted avg 0.868055 0.868100 0.868088 2077.0
The result of Model Number:Dataset Number:Featuregroup Number:Rescale of 79 : 0 : 10 : 1

	f1-score	precision	recall	support
AFIB	0.671552	0.700638	0.644977	204.0
GSVT	0.627785	0.633644	0.622349	300.0
SB	0.650745	0.618751	0.686273	892.0
SR	0.579990	0.616592	0.547677	681.0
macro avg	0.632518	0.642406	0.625319	2077.0
micro avg	0.627542	0.627542	0.627542	2077.0
weighted avg	0.626273	0.628237	0.627542	2077.0

The result of Model Number:Dataset Number:Featuregroup Number:Rescale of 79 : 1 : 0 : 0

	f1-score	precision	recall	support
AFIB	0.740187	0.725442	0.755582	875.0
GSVT	0.602447	0.560243	0.755149	691.0
SB	0.488819	0.489661	0.487980	1553.0
SR	0.792438	0.799416	0.785614	907.0
macro avg	0.655973	0.643690	0.696081	4026.0
micro avg	0.659048	0.659048	0.659048	4026.0
weighted avg	0.631354	0.622803	0.659048	4026.0

The result of Model Number:Dataset Number:Featuregroup Number:Rescale of 79 : 1 : 0 : 1

	f1-score	precision	recall	support
AFIB	0.808187	0.816033	0.800991	204.0
GSVT	0.832413	0.828478	0.836605	300.0
SB	0.849269	0.848322	0.850221	892.0
SR	0.841797	0.842669	0.840931	681.0
macro avg	0.832916	0.833875	0.832187	2077.0
micro avg	0.840373	0.840373	0.840373	2077.0
weighted avg	0.840349	0.840431	0.840373	2077.0

The result of Model Number:Dataset Number:Featuregroup Number:Rescale of 79 : 1 : 1 : 0

	f1-score	precision	recall	support
AFIB	0.816149	0.784948	0.867487	875.0
GSVT	0.845668	0.816482	0.885256	691.0
SB	0.942232	0.925600	0.960507	1553.0
SR	0.798049	0.959608	0.689007	907.0
macro avg	0.850524	0.871659	0.850564	4026.0
micro avg	0.866210	0.866210	0.866210	4026.0
weighted avg	0.865773	0.883964	0.866210	4026.0

The result of Model Number:Dataset Number:Featuregroup Number:Rescale of 79 : 1 : 1 : 1

	f1-score	precision	recall	support
AFIB	0.765324	0.776927	0.754082	204.0
GSVT	0.777630	0.771075	0.784339	300.0
SB	0.794879	0.792315	0.797462	892.0
SR	0.786346	0.789267	0.783469	681.0
macro avg	0.781045	0.782396	0.779838	2077.0
micro avg	0.786718	0.786718	0.786718	2077.0
weighted avg	0.786687	0.786736	0.786718	2077.0

The result of Model Number:Dataset Number:Featuregroup Number:Rescale of 79 : 1 : 2 : 0

	f1-score	precision	recall	support
AFIB	0.797942	0.807068	0.789328	204.0
GSVT	0.816619	0.811787	0.821523	300.0
SB	0.783939	0.754982	0.832819	892.0
SR	0.657601	0.660784	0.654466	681.0
macro avg	0.764025	0.758655	0.774534	2077.0
micro avg	0.768438	0.768438	0.768438	2077.0
weighted avg	0.748612	0.737417	0.768438	2077.0

The result of Model Number:Dataset Number:Featuregroup Number:Rescale of 79 : 1 : 2 : 1

	f1-score	precision	recall	support
AFIB	0.694961	0.701296	0.688830	204.0
GSVT	0.713097	0.710762	0.715469	300.0
SB	0.726133	0.725649	0.726620	892.0
SR	0.719746	0.719540	0.719964	681.0
macro avg	0.713484	0.714311	0.712721	2077.0
micro avg	0.719115	0.719115	0.719115	2077.0
weighted avg	0.719094	0.719103	0.719115	2077.0

The result of Model Number:Dataset Number:Featuregroup Number:Rescale of 79 : 1 : 3 : 0

	f1-score	precision	recall	support
AFIB	0.900456	0.946976	0.858560	204.0
GSVT	0.944205	0.934976	0.953639	300.0
SB	0.960853	0.953124	0.968715	892.0
SR	0.948985	0.950140	0.947871	681.0
macro avg	0.938625	0.946304	0.932196	2077.0
micro avg	0.948884	0.948884	0.948884	2077.0
weighted avg	0.948625	0.948920	0.948884	2077.0

The result of Model Number:Dataset Number:Featuregroup Number:Rescale of 79 : 1 : 3 : 1

	f1-score	precision	recall	support
AFIB	0.694195	0.715576	0.674193	204.0

GSVT 0.711904 0.703190 0.720916 300.0
SB 0.731241 0.726896 0.735662 892.0
SR 0.718915 0.722461 0.715432 681.0
macro avg 0.714064 0.717031 0.711551 2077.0
micro avg 0.720862 0.720862 0.720862 2077.0
weighted avg 0.720768 0.720906 0.720862 2077.0
The result of Model Number:Dataset Number:Featuregroup Number:Rescale of 79 : 1 : 4 : 0

	f1-score	precision	recall	support
AFIB	0.662411	0.665456	0.659426	875.0
GSVT	0.634877	0.638702	0.631119	691.0
SB	0.681009	0.650654	0.714370	1553.0
SR	0.582412	0.634032	0.538682	907.0
macro avg	0.640177	0.647211	0.635899	4026.0
micro avg	0.648560	0.648560	0.648560	4026.0
weighted avg	0.646837	0.648075	0.648560	4026.0

The result of Model Number:Dataset Number:Featuregroup Number:Rescale of 79 : 1 : 4 : 1

	f1-score	precision	recall	support
AFIB	0.917550	0.922783	0.912528	204.0
GSVT	0.936429	0.929675	0.943292	300.0
SB	0.955342	0.951852	0.958874	892.0
SR	0.945181	0.951387	0.939080	681.0
macro avg	0.938626	0.938924	0.938443	2077.0
micro avg	0.945581	0.945581	0.945581	2077.0
weighted avg	0.945567	0.945641	0.945581	2077.0

The result of Model Number:Dataset Number:Featuregroup Number:Rescale of 79 : 1 : 5 : 0

	f1-score	precision	recall	support
AFIB	0.832634	0.830227	0.835132	875.0
GSVT	0.848066	0.847377	0.848816	691.0
SB	0.900770	0.897039	0.904539	1553.0
SR	0.873727	0.883326	0.864382	907.0
macro avg	0.863799	0.864492	0.863217	4026.0
micro avg	0.870843	0.870843	0.870843	4026.0
weighted avg	0.870823	0.870905	0.870843	4026.0

The result of Model Number:Dataset Number:Featuregroup Number:Rescale of 79 : 1 : 5 : 1

	f1-score	precision	recall	support
AFIB	0.722890	0.745236	0.702476	204.0
GSVT	0.674809	0.689479	0.661248	300.0
SB	0.711753	0.680164	0.746454	892.0
SR	0.640855	0.673786	0.611084	681.0
macro avg	0.687577	0.697166	0.680316	2077.0

micro avg 0.685443 0.685443 0.685443 2077.0
weighted avg 0.684265 0.685810 0.685443 2077.0
The result of Model Number:Dataset Number:Featuregroup Number:Rescale of 79 : 1 : 6 : 0

	f1-score	precision	recall	support
AFIB	0.806309	0.830762	0.783289	875.0
GSVT	0.860747	0.854777	0.866846	691.0
SB	0.941762	0.938159	0.945398	1553.0
SR	0.890679	0.877311	0.904513	907.0
macro avg	0.874874	0.875252	0.875012	4026.0
micro avg	0.887473	0.887473	0.887473	4026.0
weighted avg	0.886910	0.886798	0.887473	4026.0

The result of Model Number:Dataset Number:Featuregroup Number:Rescale of 79 : 1 : 6 : 1

	f1-score	precision	recall	support
AFIB	0.782224	0.822636	0.745829	204.0
GSVT	0.820229	0.812211	0.828424	300.0
SB	0.834691	0.827976	0.841521	892.0
SR	0.824381	0.825385	0.823414	681.0
macro avg	0.815381	0.822052	0.809797	2077.0
micro avg	0.824293	0.824293	0.824293	2077.0
weighted avg	0.824069	0.824325	0.824293	2077.0

The result of Model Number:Dataset Number:Featuregroup Number:Rescale of 79 : 1 : 7 : 0

	f1-score	precision	recall	support
AFIB	0.825032	0.821814	0.828333	875.0
GSVT	0.840473	0.842245	0.838757	691.0
SB	0.892190	0.889966	0.894430	1553.0
SR	0.871609	0.877463	0.865850	907.0
macro avg	0.857326	0.857872	0.856843	4026.0
micro avg	0.864071	0.864071	0.864071	4026.0
weighted avg	0.864081	0.864147	0.864071	4026.0

The result of Model Number:Dataset Number:Featuregroup Number:Rescale of 79 : 1 : 7 : 1

	f1-score	precision	recall	support
AFIB	0.676755	0.657985	0.698758	875.0
GSVT	0.685590	0.714468	0.663174	691.0
SB	0.753144	0.752764	0.753535	1553.0
SR	0.734250	0.738845	0.729717	907.0
macro avg	0.712435	0.716015	0.711296	4026.0
micro avg	0.720755	0.720755	0.720755	4026.0
weighted avg	0.720691	0.722456	0.720755	4026.0

The result of Model Number:Dataset Number:Featuregroup Number:Rescale of 79 : 1 : 8 : 0

	f1-score	precision	recall	support
AFIB	0.789883	0.806520	0.774044	204.0
GSVT	0.814442	0.807001	0.822040	300.0
SB	0.827743	0.825898	0.829599	892.0
SR	0.817906	0.818761	0.817062	681.0
macro avg	0.812494	0.814545	0.810686	2077.0
micro avg	0.818940	0.818940	0.818940	2077.0
weighted avg	0.818878	0.818925	0.818940	2077.0

The result of Model Number:Dataset Number:Featuregroup Number:Rescale of 79 : 1 : 8 : 1

	f1-score	precision	recall	support
AFIB	0.849654	0.877870	0.823518	204.0
GSVT	0.881899	0.874823	0.889222	300.0
SB	0.901383	0.897680	0.905120	892.0
SR	0.892965	0.892834	0.893096	681.0
macro avg	0.881475	0.885802	0.877739	2077.0
micro avg	0.890867	0.890867	0.890867	2077.0
weighted avg	0.890728	0.890844	0.890867	2077.0

The result of Model Number:Dataset Number:Featuregroup Number:Rescale of 79 : 1 : 9 : 0

	f1-score	precision	recall	support
AFIB	0.802923	0.837697	0.771148	204.0
GSVT	0.750593	0.757598	0.744093	300.0
SB	0.778044	0.739792	0.820523	892.0
SR	0.693448	0.737211	0.654815	681.0
macro avg	0.756252	0.768075	0.747645	2077.0
micro avg	0.750302	0.750302	0.750302	2077.0
weighted avg	0.748786	0.751134	0.750302	2077.0

The result of Model Number:Dataset Number:Featuregroup Number:Rescale of 79 : 1 : 9 : 1

	f1-score	precision	recall	support
AFIB	0.835233	0.824570	0.846225	875.0
GSVT	0.849894	0.854768	0.845098	691.0
SB	0.909759	0.905938	0.913621	1553.0
SR	0.882971	0.897263	0.869143	907.0
macro avg	0.869464	0.870635	0.868522	4026.0
micro avg	0.877192	0.877192	0.877192	4026.0
weighted avg	0.877252	0.877517	0.877192	4026.0

The result of Model Number:Dataset Number:Featuregroup Number:Rescale of 79 : 1 : 10 : 0

	f1-score	precision	recall	support
AFIB	0.768444	0.768397	0.768538	875.0
GSVT	0.770107	0.765943	0.774329	691.0
SB	0.806898	0.801529	0.812349	1553.0

SR 0.785472 0.798359 0.773023 907.0
macro avg 0.782730 0.783557 0.782060 4026.0
micro avg 0.787442 0.787442 0.787442 4026.0
weighted avg 0.787399 0.787506 0.787442 4026.0
The result of Model Number:Dataset Number:Featuregroup Number:Rescale of 79 : 1 : 10 : 1

	f1-score	precision	recall	support
AFIB	0.815171	0.807713	0.822804	875.0
GSVT	0.825616	0.832900	0.818581	691.0
SB	0.877603	0.872976	0.882292	1553.0
SR	0.857764	0.868101	0.847748	907.0
macro avg	0.844038	0.845423	0.842856	4026.0
micro avg	0.850646	0.850646	0.850646	4026.0
weighted avg	0.850642	0.850815	0.850646	4026.0

The result of Model Number:Dataset Number:Featuregroup Number:Rescale of 80 : 0 : 0 : 0

	f1-score	precision	recall	support
AFIB	0.863580	0.926000	0.809639	204.0
GSVT	0.817990	0.822415	0.813947	300.0
SB	0.854407	0.803174	0.912641	892.0
SR	0.750233	0.809670	0.699022	681.0
macro avg	0.821553	0.840315	0.808812	2077.0
micro avg	0.818228	0.818228	0.818228	2077.0
weighted avg	0.815892	0.820147	0.818228	2077.0

The result of Model Number:Dataset Number:Featuregroup Number:Rescale of 80 : 0 : 0 : 1

	f1-score	precision	recall	support
AFIB	0.662245	0.663356	0.678659	204.0
GSVT	0.656019	0.681476	0.633241	300.0
SB	0.695851	0.657690	0.738998	892.0
SR	0.603154	0.658817	0.557691	681.0
macro avg	0.654317	0.665335	0.652147	2077.0
micro avg	0.658350	0.658350	0.658350	2077.0
weighted avg	0.656404	0.662051	0.658350	2077.0

The result of Model Number:Dataset Number:Featuregroup Number:Rescale of 80 : 0 : 1 : 0

	f1-score	precision	recall	support
AFIB	0.824454	0.867863	0.785401	204.0
GSVT	0.865854	0.858270	0.873612	300.0
SB	0.888321	0.882838	0.893890	892.0
SR	0.876202	0.874425	0.878007	681.0
macro avg	0.863708	0.870849	0.857728	2077.0
micro avg	0.875098	0.875098	0.875098	2077.0
weighted avg	0.874830	0.875060	0.875098	2077.0

The result of Model Number:Dataset Number:Featuregroup Number:Rescale of 80 : 0 : 1 : 1

	f1-score	precision	recall	support
AFIB	0.854266	0.857018	0.851612	875.0
GSVT	0.866276	0.852523	0.880559	691.0
SB	0.912763	0.905142	0.920520	1553.0
SR	0.866881	0.888897	0.845960	907.0
macro avg	0.875046	0.875895	0.874663	4026.0
micro avg	0.881888	0.881888	0.881888	4026.0
weighted avg	0.881734	0.881992	0.881888	4026.0

The result of Model Number:Dataset Number:Featuregroup Number:Rescale of 80 : 0 : 2 : 0

	f1-score	precision	recall	support
AFIB	0.743664	0.757553	0.823349	204.0
GSVT	0.893063	0.874971	0.912370	300.0
SB	0.734675	0.727812	0.741679	892.0
SR	0.894202	0.901672	0.886979	681.0
macro avg	0.816401	0.815502	0.841094	2077.0
micro avg	0.821995	0.821995	0.821995	2077.0
weighted avg	0.810741	0.808993	0.821995	2077.0

The result of Model Number:Dataset Number:Featuregroup Number:Rescale of 80 : 0 : 2 : 1

	f1-score	precision	recall	support
AFIB	0.874511	0.874081	0.875009	875.0
GSVT	0.893495	0.889098	0.897991	691.0
SB	0.934611	0.928502	0.940809	1553.0
SR	0.908006	0.922796	0.893707	907.0
macro avg	0.902656	0.903619	0.901879	4026.0
micro avg	0.908547	0.908547	0.908547	4026.0
weighted avg	0.908498	0.908626	0.908547	4026.0

The result of Model Number:Dataset Number:Featuregroup Number:Rescale of 80 : 0 : 3 : 0

	f1-score	precision	recall	support
AFIB	0.739391	0.746006	0.732985	875.0
GSVT	0.753513	0.739185	0.768552	691.0
SB	0.799791	0.792862	0.806845	1553.0
SR	0.766941	0.784575	0.750117	907.0
macro avg	0.764909	0.765657	0.764624	4026.0
micro avg	0.771440	0.771440	0.771440	4026.0
weighted avg	0.771320	0.771599	0.771440	4026.0

The result of Model Number:Dataset Number:Featuregroup Number:Rescale of 80 : 0 : 3 : 1

	f1-score	precision	recall	support
AFIB	0.722533	0.726846	0.718291	204.0

GSVT	0.740287	0.737108	0.743494	300.0
SB	0.752965	0.751874	0.754061	892.0
SR	0.746080	0.747621	0.744549	681.0
macro avg	0.740466	0.740862	0.740099	2077.0
micro avg	0.745903	0.745903	0.745903	2077.0
weighted avg	0.745887	0.745889	0.745903	2077.0

The result of Model Number:Dataset Number:Featuregroup Number:Rescale of 80 : 0 : 4 : 0

	f1-score	precision	recall	support
AFIB	0.871232	0.869830	0.872994	875.0
GSVT	0.891545	0.886898	0.896293	691.0
SB	0.932157	0.924260	0.940198	1553.0
SR	0.896573	0.916505	0.877791	907.0
macro avg	0.897877	0.899373	0.896819	4026.0
micro avg	0.903997	0.903997	0.903997	4026.0
weighted avg	0.903929	0.904270	0.903997	4026.0

The result of Model Number:Dataset Number:Featuregroup Number:Rescale of 80 : 0 : 4 : 1

	f1-score	precision	recall	support
AFIB	0.882310	0.922152	0.846056	204.0
GSVT	0.912998	0.902744	0.923653	300.0
SB	0.878270	0.839979	0.939761	892.0
SR	0.731719	0.737958	0.725709	681.0
macro avg	0.851324	0.850708	0.858795	2077.0
micro avg	0.858048	0.858048	0.858048	2077.0
weighted avg	0.835632	0.823665	0.858048	2077.0

The result of Model Number:Dataset Number:Featuregroup Number:Rescale of 80 : 0 : 5 : 0

	f1-score	precision	recall	support
AFIB	0.838686	0.846189	0.831420	875.0
GSVT	0.854704	0.838452	0.871763	691.0
SB	0.907197	0.899338	0.915198	1553.0
SR	0.869936	0.889938	0.850852	907.0
macro avg	0.867631	0.868479	0.867308	4026.0
micro avg	0.875039	0.875039	0.875039	4026.0
weighted avg	0.874903	0.875219	0.875039	4026.0

The result of Model Number:Dataset Number:Featuregroup Number:Rescale of 80 : 0 : 5 : 1

	f1-score	precision	recall	support
AFIB	0.759339	0.763871	0.754880	204.0
GSVT	0.777997	0.774656	0.781367	300.0
SB	0.791321	0.790175	0.792473	892.0
SR	0.784085	0.785705	0.782476	681.0
macro avg	0.778185	0.778602	0.777799	2077.0

micro avg 0.783899 0.783899 0.783899 2077.0
weighted avg 0.783883 0.783884 0.783899 2077.0
The result of Model Number:Dataset Number:Featuregroup Number:Rescale of 80 : 0 : 6 : 0

	f1-score	precision	recall	support
AFIB	0.893093	0.886262	0.900149	875.0
GSVT	0.913643	0.918639	0.908754	691.0
SB	0.967474	0.966796	0.968159	1553.0
SR	0.941776	0.946344	0.937300	907.0
macro avg	0.928996	0.929510	0.928591	4026.0
micro avg	0.936230	0.936230	0.936230	4026.0
weighted avg	0.936280	0.936420	0.936230	4026.0

The result of Model Number:Dataset Number:Featuregroup Number:Rescale of 80 : 0 : 6 : 1

	f1-score	precision	recall	support
AFIB	0.786113	0.802160	0.771042	204.0
GSVT	0.814658	0.807580	0.822009	300.0
SB	0.830321	0.828838	0.831810	892.0
SR	0.822592	0.823083	0.822107	681.0
macro avg	0.813421	0.815415	0.811742	2077.0
micro avg	0.821245	0.821245	0.821245	2077.0
weighted avg	0.821182	0.821260	0.821245	2077.0

The result of Model Number:Dataset Number:Featuregroup Number:Rescale of 80 : 0 : 7 : 0

	f1-score	precision	recall	support
AFIB	0.765046	0.754193	0.776236	875.0
GSVT	0.780786	0.788396	0.773328	691.0
SB	0.830909	0.828201	0.833636	1553.0
SR	0.811504	0.821876	0.801400	907.0
macro avg	0.797062	0.798167	0.796150	4026.0
micro avg	0.803548	0.803548	0.803548	4026.0
weighted avg	0.803620	0.803860	0.803548	4026.0

The result of Model Number:Dataset Number:Featuregroup Number:Rescale of 80 : 0 : 7 : 1

	f1-score	precision	recall	support
AFIB	0.640085	0.709058	0.583854	204.0
GSVT	0.754493	0.743917	0.765568	300.0
SB	0.801089	0.798509	0.803693	892.0
SR	0.780579	0.768706	0.792844	681.0
macro avg	0.744061	0.755047	0.736490	2077.0
micro avg	0.773037	0.773037	0.773037	2077.0
weighted avg	0.771820	0.772066	0.773037	2077.0

The result of Model Number:Dataset Number:Featuregroup Number:Rescale of 80 : 0 : 8 : 0

f1-score precision recall support
AFIB 0.714205 0.729038 0.700127 875.0
GSVT 0.696492 0.672301 0.753346 691.0
SB 0.820925 0.817223 0.824674 1553.0
SR 0.624106 0.614030 0.634574 907.0
macro avg 0.713932 0.708148 0.728180 4026.0
micro avg 0.742536 0.742536 0.742536 4026.0
weighted avg 0.732033 0.727407 0.742536 4026.0
The result of Model Number:Dataset Number:Featuregroup Number:Rescale of 80 : 0 : 8 : 1

f1-score precision recall support
AFIB 0.680355 0.671243 0.689761 875.0
GSVT 0.695567 0.700996 0.690233 691.0
SB 0.738498 0.736187 0.740827 1553.0
SR 0.720639 0.730193 0.711345 907.0
macro avg 0.708765 0.709655 0.708041 4026.0
micro avg 0.714403 0.714403 0.714403 4026.0
weighted avg 0.714469 0.714682 0.714403 4026.0
The result of Model Number:Dataset Number:Featuregroup Number:Rescale of 80 : 0 : 9 : 0

f1-score precision recall support
AFIB 0.883960 0.926282 0.845545 204.0
GSVT 0.931048 0.921081 0.941267 300.0
SB 0.950824 0.945572 0.956148 892.0
SR 0.940083 0.939268 0.940916 681.0
macro avg 0.926479 0.933051 0.920969 2077.0
micro avg 0.938141 0.938141 0.938141 2077.0
weighted avg 0.937879 0.938073 0.938141 2077.0
The result of Model Number:Dataset Number:Featuregroup Number:Rescale of 80 : 0 : 9 : 1

f1-score precision recall support
AFIB 0.871271 0.877368 0.865289 204.0
GSVT 0.891542 0.887146 0.895995 300.0
SB 0.909262 0.907748 0.910791 892.0
SR 0.900212 0.902349 0.898096 681.0
macro avg 0.893072 0.893653 0.892542 2077.0
micro avg 0.900022 0.900022 0.900022 2077.0
weighted avg 0.900004 0.900018 0.900022 2077.0
The result of Model Number:Dataset Number:Featuregroup Number:Rescale of 80 : 0 : 10 : 0

f1-score precision recall support
AFIB 0.847182 0.850043 0.844371 875.0
GSVT 0.844945 0.838993 0.851058 691.0
SB 0.889037 0.880786 0.897451 1553.0

SR 0.855238 0.871720 0.839410 907.0
macro avg 0.859101 0.860386 0.858073 4026.0
micro avg 0.864876 0.864876 0.864876 4026.0
weighted avg 0.864758 0.864889 0.864876 4026.0
The result of Model Number:Dataset Number:Featuregroup Number:Rescale of 80 : 0 : 10 : 1

	f1-score	precision	recall	support
AFIB	0.672592	0.677859	0.667441	875.0
GSVT	0.674366	0.665759	0.683240	691.0
SB	0.707334	0.704497	0.710201	1553.0
SR	0.692390	0.699186	0.685750	907.0
macro avg	0.686670	0.686825	0.686658	4026.0
micro avg	0.690772	0.690772	0.690772	4026.0
weighted avg	0.690758	0.690862	0.690772	4026.0

The result of Model Number:Dataset Number:Featuregroup Number:Rescale of 80 : 1 : 0 : 0

	f1-score	precision	recall	support
AFIB	0.732484	0.739162	0.726022	204.0
GSVT	0.751599	0.749138	0.754100	300.0
SB	0.765339	0.764829	0.765853	892.0
SR	0.758608	0.758390	0.758837	681.0
macro avg	0.752008	0.752880	0.751203	2077.0
micro avg	0.757943	0.757943	0.757943	2077.0
weighted avg	0.757921	0.757931	0.757943	2077.0

The result of Model Number:Dataset Number:Featuregroup Number:Rescale of 80 : 1 : 0 : 1

	f1-score	precision	recall	support
AFIB	0.649421	0.693440	0.611089	204.0
GSVT	0.611908	0.630063	0.595000	300.0
SB	0.639597	0.608893	0.673926	892.0
SR	0.567524	0.593290	0.544522	681.0
macro avg	0.617112	0.631422	0.606134	2077.0
micro avg	0.613925	0.613925	0.613925	2077.0
weighted avg	0.612931	0.615139	0.613925	2077.0

The result of Model Number:Dataset Number:Featuregroup Number:Rescale of 80 : 1 : 1 : 0

	f1-score	precision	recall	support
AFIB	0.698114	0.678751	0.720811	875.0
GSVT	0.707228	0.737017	0.684104	691.0
SB	0.776914	0.776521	0.777317	1553.0
SR	0.757423	0.762163	0.752747	907.0
macro avg	0.734920	0.738613	0.733745	4026.0
micro avg	0.743502	0.743502	0.743502	4026.0
weighted avg	0.743436	0.745257	0.743502	4026.0

The result of Model Number:Dataset Number:Featuregroup Number:Rescale of 80 : 1 : 1 : 1

	f1-score	precision	recall	support
AFIB	0.692889	0.707033	0.679606	204.0
GSVT	0.718049	0.711810	0.724529	300.0
SB	0.731854	0.730548	0.733167	892.0
SR	0.725042	0.725475	0.724615	681.0
macro avg	0.716959	0.718716	0.715479	2077.0
micro avg	0.723854	0.723854	0.723854	2077.0
weighted avg	0.723800	0.723868	0.723854	2077.0

The result of Model Number:Dataset Number:Featuregroup Number:Rescale of 80 : 1 : 2 : 0

	f1-score	precision	recall	support
AFIB	0.666496	0.660075	0.673146	875.0
GSVT	0.683958	0.685915	0.682084	691.0
SB	0.727165	0.725493	0.728850	1553.0
SR	0.706710	0.715025	0.698601	907.0
macro avg	0.696082	0.696627	0.695670	4026.0
micro avg	0.701902	0.701902	0.701902	4026.0
weighted avg	0.701955	0.702124	0.701902	4026.0

The result of Model Number:Dataset Number:Featuregroup Number:Rescale of 80 : 1 : 2 : 1

	f1-score	precision	recall	support
AFIB	0.661190	0.661307	0.661100	875.0
GSVT	0.668185	0.670442	0.665985	691.0
SB	0.701894	0.699474	0.704334	1553.0
SR	0.688565	0.690888	0.686284	907.0
macro avg	0.679958	0.680528	0.679426	4026.0
micro avg	0.684289	0.684289	0.684289	4026.0
weighted avg	0.684259	0.684262	0.684289	4026.0

The result of Model Number:Dataset Number:Featuregroup Number:Rescale of 80 : 1 : 3 : 0

	f1-score	precision	recall	support
AFIB	0.701708	0.714813	0.776898	204.0
GSVT	0.842678	0.825607	0.860896	300.0
SB	0.693226	0.686750	0.699834	892.0
SR	0.843753	0.850801	0.836937	681.0
macro avg	0.770341	0.769493	0.793641	2077.0
micro avg	0.775620	0.775620	0.775620	2077.0
weighted avg	0.765000	0.763351	0.775620	2077.0

The result of Model Number:Dataset Number:Featuregroup Number:Rescale of 80 : 1 : 3 : 1

	f1-score	precision	recall	support
AFIB	0.498490	0.543027	0.526416	204.0

GSVT	0.677087	0.666019	0.688763	300.0
SB	0.576410	0.572020	0.580912	892.0
SR	0.704856	0.696881	0.713037	681.0
macro avg	0.614211	0.619487	0.627282	2077.0
micro avg	0.634458	0.634458	0.634458	2077.0
weighted avg	0.625413	0.623688	0.634458	2077.0

The result of Model Number:Dataset Number:Featuregroup Number:Rescale of 80 : 1 : 4 : 0

	f1-score	precision	recall	support
AFIB	0.806800	0.812928	0.800833	204.0
GSVT	0.823317	0.815276	0.831537	300.0
SB	0.842522	0.840368	0.844694	892.0
SR	0.833585	0.838294	0.828939	681.0
macro avg	0.826556	0.826716	0.826501	2077.0
micro avg	0.833320	0.833320	0.833320	2077.0
weighted avg	0.833309	0.833369	0.833320	2077.0

The result of Model Number:Dataset Number:Featuregroup Number:Rescale of 80 : 1 : 4 : 1

	f1-score	precision	recall	support
AFIB	0.932793	0.933842	0.931916	204.0
GSVT	0.954209	0.952079	0.956422	300.0
SB	0.974434	0.973133	0.975744	892.0
SR	0.967136	0.969572	0.964725	681.0
macro avg	0.957143	0.957156	0.957201	2077.0
micro avg	0.965035	0.965035	0.965035	2077.0
weighted avg	0.965030	0.965065	0.965035	2077.0

The result of Model Number:Dataset Number:Featuregroup Number:Rescale of 80 : 1 : 5 : 0

	f1-score	precision	recall	support
AFIB	0.762377	0.763905	0.760888	875.0
GSVT	0.764265	0.760139	0.768457	691.0
SB	0.798914	0.794646	0.803233	1553.0
SR	0.779801	0.789048	0.770790	907.0
macro avg	0.776339	0.776935	0.775842	4026.0
micro avg	0.780752	0.780752	0.780752	4026.0
weighted avg	0.780720	0.780781	0.780752	4026.0

The result of Model Number:Dataset Number:Featuregroup Number:Rescale of 80 : 1 : 5 : 1

	f1-score	precision	recall	support
AFIB	0.690553	0.708801	0.673289	204.0
GSVT	0.709491	0.703597	0.715615	300.0
SB	0.729042	0.726366	0.731739	892.0
SR	0.717754	0.718623	0.716905	681.0
macro avg	0.711710	0.714347	0.709387	2077.0

micro avg 0.718805 0.718805 0.718805 2077.0
weighted avg 0.718737 0.718814 0.718805 2077.0
The result of Model Number:Dataset Number:Featuregroup Number:Rescale of 80 : 1 : 6 : 0

	f1-score	precision	recall	support
AFIB	0.786567	0.792225	0.780991	875.0
GSVT	0.785314	0.780612	0.790086	691.0
SB	0.828391	0.819213	0.837779	1553.0
SR	0.794905	0.809087	0.781238	907.0
macro avg	0.798794	0.800284	0.797524	4026.0
micro avg	0.804513	0.804513	0.804513	4026.0
weighted avg	0.804364	0.804441	0.804513	4026.0

The result of Model Number:Dataset Number:Featuregroup Number:Rescale of 80 : 1 : 6 : 1

	f1-score	precision	recall	support
AFIB	0.842243	0.852090	0.832782	204.0
GSVT	0.865413	0.861433	0.869452	300.0
SB	0.877665	0.874936	0.880418	892.0
SR	0.870107	0.872573	0.867692	681.0
macro avg	0.863857	0.865258	0.862586	2077.0
micro avg	0.869983	0.869983	0.869983	2077.0
weighted avg	0.869938	0.869967	0.869983	2077.0

The result of Model Number:Dataset Number:Featuregroup Number:Rescale of 80 : 1 : 7 : 0

	f1-score	precision	recall	support
AFIB	0.764208	0.766788	0.761672	875.0
GSVT	0.762189	0.756821	0.767704	691.0
SB	0.801963	0.794520	0.809553	1553.0
SR	0.771475	0.786342	0.757197	907.0
macro avg	0.774959	0.776118	0.774031	4026.0
micro avg	0.780169	0.780169	0.780169	4026.0
weighted avg	0.780062	0.780180	0.780169	4026.0

The result of Model Number:Dataset Number:Featuregroup Number:Rescale of 80 : 1 : 7 : 1

	f1-score	precision	recall	support
AFIB	0.751519	0.766082	0.737837	204.0
GSVT	0.780661	0.774134	0.787444	300.0
SB	0.795228	0.793632	0.796832	892.0
SR	0.787645	0.788461	0.786834	681.0
macro avg	0.778763	0.780577	0.777237	2077.0
micro avg	0.786404	0.786404	0.786404	2077.0
weighted avg	0.786345	0.786414	0.786404	2077.0

The result of Model Number:Dataset Number:Featuregroup Number:Rescale of 80 : 1 : 8 : 0

	f1-score	precision	recall	support
AFIB	0.852646	0.844432	0.861154	875.0
GSVT	0.874986	0.877489	0.872588	691.0
SB	0.930260	0.928121	0.932416	1553.0
SR	0.904092	0.914730	0.893718	907.0
macro avg	0.890496	0.891193	0.889969	4026.0
micro avg	0.897941	0.897941	0.897941	4026.0
weighted avg	0.898009	0.898225	0.897941	4026.0

The result of Model Number:Dataset Number:Featuregroup Number:Rescale of 80 : 1 : 8 : 1

	f1-score	precision	recall	support
AFIB	0.860577	0.889155	0.834105	204.0
GSVT	0.893236	0.886070	0.900653	300.0
SB	0.912971	0.909220	0.916756	892.0
SR	0.904444	0.904312	0.904578	681.0
macro avg	0.892807	0.897189	0.889023	2077.0
micro avg	0.902319	0.902319	0.902319	2077.0
weighted avg	0.902179	0.902296	0.902319	2077.0

The result of Model Number:Dataset Number:Featuregroup Number:Rescale of 80 : 1 : 9 : 0

	f1-score	precision	recall	support
AFIB	0.727659	0.729466	0.725910	875.0
GSVT	0.740190	0.742414	0.738073	691.0
SB	0.785208	0.779879	0.790617	1553.0
SR	0.770267	0.775985	0.764662	907.0
macro avg	0.755831	0.756936	0.754815	4026.0
micro avg	0.761688	0.761688	0.761688	4026.0
weighted avg	0.761608	0.761615	0.761688	4026.0

The result of Model Number:Dataset Number:Featuregroup Number:Rescale of 80 : 1 : 9 : 1

	f1-score	precision	recall	support
AFIB	0.669872	0.666499	0.673309	875.0
GSVT	0.682920	0.686226	0.679656	691.0
SB	0.727351	0.725677	0.729036	1553.0
SR	0.709143	0.712937	0.705396	907.0
macro avg	0.697322	0.697835	0.696849	4026.0
micro avg	0.703123	0.703123	0.703123	4026.0
weighted avg	0.703131	0.703174	0.703123	4026.0

The result of Model Number:Dataset Number:Featuregroup Number:Rescale of 80 : 1 : 10 : 0

	f1-score	precision	recall	support
AFIB	0.761854	0.790196	0.735816	204.0
GSVT	0.706972	0.739778	0.677017	300.0
SB	0.740581	0.695913	0.791390	892.0

SR 0.650373 0.695560 0.610775 681.0
macro avg 0.714945 0.730362 0.703749 2077.0
micro avg 0.710192 0.710192 0.710192 2077.0
weighted avg 0.708239 0.711393 0.710192 2077.0
The result of Model Number:Dataset Number:Featuregroup Number:Rescale of 80 : 1 : 10 : 1

	f1-score	precision	recall	support
AFIB	0.839326	0.845979	0.832798	875.0
GSVT	0.849967	0.846219	0.853776	691.0
SB	0.903839	0.900266	0.907448	1553.0
SR	0.881629	0.884120	0.879195	907.0
macro avg	0.868690	0.869146	0.868304	4026.0
micro avg	0.875646	0.875646	0.875646	4026.0
weighted avg	0.875568	0.875554	0.875646	4026.0

The result of Model Number:Dataset Number:Featuregroup Number:Rescale of 81 : 0 : 0 : 0

	f1-score	precision	recall	support
AFIB	0.838438	0.850756	0.826549	204.0
GSVT	0.859682	0.852373	0.867135	300.0
SB	0.874540	0.871041	0.878069	892.0
SR	0.865890	0.870140	0.861692	681.0
macro avg	0.859638	0.861078	0.858361	2077.0
micro avg	0.866060	0.866060	0.866060	2077.0
weighted avg	0.866012	0.866057	0.866060	2077.0

The result of Model Number:Dataset Number:Featuregroup Number:Rescale of 81 : 0 : 0 : 1

	f1-score	precision	recall	support
AFIB	0.826267	0.805123	0.848628	875.0
GSVT	0.781078	0.825347	0.741497	691.0
SB	0.828216	0.762158	0.906832	1553.0
SR	0.661605	0.803606	0.562372	907.0
macro avg	0.774291	0.799058	0.764832	4026.0
micro avg	0.788203	0.788203	0.788203	4026.0
weighted avg	0.782167	0.791679	0.788203	4026.0

The result of Model Number:Dataset Number:Featuregroup Number:Rescale of 81 : 0 : 1 : 0

	f1-score	precision	recall	support
AFIB	0.854368	0.847833	0.861118	875.0
GSVT	0.874026	0.878806	0.869350	691.0
SB	0.925523	0.924875	0.926179	1553.0
SR	0.900940	0.905310	0.896658	907.0
macro avg	0.888714	0.889206	0.888326	4026.0
micro avg	0.895634	0.895634	0.895634	4026.0
weighted avg	0.895682	0.895816	0.895634	4026.0

The result of Model Number:Dataset Number:Featuregroup Number:Rescale of 81 : 0 : 1 : 1

	f1-score	precision	recall	support
AFIB	0.799160	0.788091	0.810577	875.0
GSVT	0.816885	0.823496	0.810384	691.0
SB	0.867114	0.864622	0.869625	1553.0
SR	0.846020	0.857031	0.835296	907.0
macro avg	0.832295	0.833310	0.831470	4026.0
micro avg	0.838890	0.838890	0.838890	4026.0
weighted avg	0.838972	0.839220	0.838890	4026.0

The result of Model Number:Dataset Number:Featuregroup Number:Rescale of 81 : 0 : 2 : 0

	f1-score	precision	recall	support
AFIB	0.820657	0.833662	0.808161	204.0
GSVT	0.841751	0.834588	0.849043	300.0
SB	0.856284	0.854376	0.858204	892.0
SR	0.848171	0.850056	0.846310	681.0
macro avg	0.841716	0.843170	0.840430	2077.0
micro avg	0.848066	0.848066	0.848066	2077.0
weighted avg	0.848026	0.848067	0.848066	2077.0

The result of Model Number:Dataset Number:Featuregroup Number:Rescale of 81 : 0 : 2 : 1

	f1-score	precision	recall	support
AFIB	0.668813	0.684054	0.654343	875.0
GSVT	0.711702	0.713109	0.710539	691.0
SB	0.778650	0.774388	0.782961	1553.0
SR	0.735941	0.727014	0.745192	907.0
macro avg	0.723776	0.724641	0.723259	4026.0
micro avg	0.734069	0.734069	0.734069	4026.0
weighted avg	0.733666	0.733565	0.734069	4026.0

The result of Model Number:Dataset Number:Featuregroup Number:Rescale of 81 : 0 : 3 : 0

	f1-score	precision	recall	support
AFIB	0.867418	0.873714	0.861275	875.0
GSVT	0.886446	0.878207	0.894908	691.0
SB	0.952670	0.946918	0.958497	1553.0
SR	0.921447	0.931736	0.911386	907.0
macro avg	0.906995	0.907644	0.906517	4026.0
micro avg	0.915840	0.915840	0.915840	4026.0
weighted avg	0.915741	0.915795	0.915840	4026.0

The result of Model Number:Dataset Number:Featuregroup Number:Rescale of 81 : 0 : 3 : 1

	f1-score	precision	recall	support
AFIB	0.842481	0.869845	0.816952	204.0

GSVT	0.865741	0.854702	0.877243	300.0
SB	0.890166	0.886809	0.893558	892.0
SR	0.877716	0.879305	0.876166	681.0
macro avg	0.869026	0.872665	0.865980	2077.0
micro avg	0.877975	0.877975	0.877975	2077.0
weighted avg	0.877872	0.878045	0.877975	2077.0

The result of Model Number:Dataset Number:Featuregroup Number:Rescale of 81 : 0 : 4 : 0

	f1-score	precision	recall	support
AFIB	0.838255	0.923392	0.768297	204.0
GSVT	0.774124	0.827655	0.727459	300.0
SB	0.842791	0.787482	0.906470	892.0
SR	0.729450	0.767794	0.694953	681.0
macro avg	0.796155	0.826581	0.774295	2077.0
micro avg	0.797691	0.797691	0.797691	2077.0
weighted avg	0.795266	0.800178	0.797691	2077.0

The result of Model Number:Dataset Number:Featuregroup Number:Rescale of 81 : 0 : 4 : 1

	f1-score	precision	recall	support
AFIB	0.923662	0.932083	0.915514	204.0
GSVT	0.947767	0.944663	0.950920	300.0
SB	0.965093	0.964449	0.965740	892.0
SR	0.956605	0.956330	0.956894	681.0
macro avg	0.948282	0.949381	0.947267	2077.0
micro avg	0.955766	0.955766	0.955766	2077.0
weighted avg	0.955738	0.955750	0.955766	2077.0

The result of Model Number:Dataset Number:Featuregroup Number:Rescale of 81 : 0 : 5 : 0

	f1-score	precision	recall	support
AFIB	0.817356	0.809342	0.873917	204.0
GSVT	0.732885	0.724737	0.741402	300.0
SB	0.941803	0.934945	0.948769	892.0
SR	0.926008	0.932422	0.919758	681.0
macro avg	0.854513	0.850361	0.870962	2077.0
micro avg	0.901953	0.901953	0.901953	2077.0
weighted avg	0.894225	0.891419	0.901953	2077.0

The result of Model Number:Dataset Number:Featuregroup Number:Rescale of 81 : 0 : 5 : 1

	f1-score	precision	recall	support
AFIB	0.846024	0.840492	0.851704	875.0
GSVT	0.871170	0.878582	0.863998	691.0
SB	0.927478	0.925458	0.929509	1553.0
SR	0.904875	0.908508	0.901284	907.0
macro avg	0.887387	0.888260	0.886623	4026.0

micro avg 0.894996 0.894996 0.894996 4026.0
weighted avg 0.895018 0.895127 0.894996 4026.0
The result of Model Number:Dataset Number:Featuregroup Number:Rescale of 81 : 0 : 6 : 0

	f1-score	precision	recall	support
AFIB	0.784631	0.783109	0.786259	875.0
GSVT	0.799364	0.785378	0.813934	691.0
SB	0.856345	0.844195	0.868859	1553.0
SR	0.811185	0.847346	0.778018	907.0
macro avg	0.812881	0.815007	0.811768	4026.0
micro avg	0.821015	0.821015	0.821015	4026.0
weighted avg	0.820805	0.821534	0.821015	4026.0

The result of Model Number:Dataset Number:Featuregroup Number:Rescale of 81 : 0 : 6 : 1

	f1-score	precision	recall	support
AFIB	0.704693	0.695449	0.714218	875.0
GSVT	0.715482	0.723435	0.707714	691.0
SB	0.758860	0.757070	0.760665	1553.0
SR	0.739861	0.746520	0.733330	907.0
macro avg	0.729724	0.730618	0.728982	4026.0
micro avg	0.735324	0.735324	0.735324	4026.0
weighted avg	0.735362	0.735528	0.735324	4026.0

The result of Model Number:Dataset Number:Featuregroup Number:Rescale of 81 : 0 : 7 : 0

	f1-score	precision	recall	support
AFIB	0.737963	0.747529	0.728835	204.0
GSVT	0.766518	0.766022	0.767037	300.0
SB	0.782669	0.781016	0.784334	892.0
SR	0.774797	0.774357	0.775254	681.0
macro avg	0.765487	0.767231	0.763865	2077.0
micro avg	0.773407	0.773407	0.773407	2077.0
weighted avg	0.773364	0.773378	0.773407	2077.0

The result of Model Number:Dataset Number:Featuregroup Number:Rescale of 81 : 0 : 7 : 1

	f1-score	precision	recall	support
AFIB	0.702429	0.724142	0.682593	204.0
GSVT	0.655709	0.669964	0.642531	300.0
SB	0.691607	0.660912	0.725326	892.0
SR	0.622716	0.654715	0.593787	681.0
macro avg	0.668115	0.677433	0.661059	2077.0
micro avg	0.666042	0.666042	0.666042	2077.0
weighted avg	0.664897	0.666398	0.666042	2077.0

The result of Model Number:Dataset Number:Featuregroup Number:Rescale of 81 : 0 : 8 : 0

	f1-score	precision	recall	support
AFIB	0.773442	0.743873	0.822094	875.0
GSVT	0.801417	0.773758	0.838933	691.0
SB	0.892927	0.877166	0.910246	1553.0
SR	0.756289	0.909394	0.652953	907.0
macro avg	0.806019	0.826048	0.806056	4026.0
micro avg	0.820883	0.820883	0.820883	4026.0
weighted avg	0.820469	0.837708	0.820883	4026.0

The result of Model Number:Dataset Number:Featuregroup Number:Rescale of 81 : 0 : 8 : 1

	f1-score	precision	recall	support
AFIB	0.742597	0.759519	0.726530	875.0
GSVT	0.790218	0.791780	0.788926	691.0
SB	0.864551	0.859819	0.869338	1553.0
SR	0.817131	0.807219	0.827403	907.0
macro avg	0.803624	0.804584	0.803049	4026.0
micro avg	0.815052	0.815052	0.815052	4026.0
weighted avg	0.814605	0.814492	0.815052	4026.0

The result of Model Number:Dataset Number:Featuregroup Number:Rescale of 81 : 0 : 9 : 0

	f1-score	precision	recall	support
AFIB	0.823517	0.852106	0.796895	204.0
GSVT	0.839307	0.830119	0.848804	300.0
SB	0.861650	0.855463	0.867929	892.0
SR	0.846262	0.850423	0.842156	681.0
macro avg	0.842684	0.847028	0.838946	2077.0
micro avg	0.849739	0.849739	0.849739	2077.0
weighted avg	0.849632	0.849820	0.849739	2077.0

The result of Model Number:Dataset Number:Featuregroup Number:Rescale of 81 : 0 : 9 : 1

	f1-score	precision	recall	support
AFIB	0.606777	0.611802	0.683451	204.0
GSVT	0.658008	0.629845	0.742387	300.0
SB	0.600786	0.598698	0.602890	892.0
SR	0.594812	0.594925	0.594704	681.0
macro avg	0.615096	0.608817	0.655858	2077.0
micro avg	0.628268	0.628268	0.628268	2077.0
weighted avg	0.607681	0.603247	0.628268	2077.0

The result of Model Number:Dataset Number:Featuregroup Number:Rescale of 81 : 0 : 10 : 0

	f1-score	precision	recall	support
AFIB	0.736627	0.716196	0.760576	875.0
GSVT	0.746243	0.777676	0.721844	691.0
SB	0.819774	0.819360	0.820199	1553.0

SR 0.799208 0.804209 0.794274 907.0
macro avg 0.775463 0.779360 0.774223 4026.0
micro avg 0.784519 0.784519 0.784519 4026.0
weighted avg 0.784449 0.786371 0.784519 4026.0
The result of Model Number:Dataset Number:Featuregroup Number:Rescale of 81 : 0 : 10 : 1

	f1-score	precision	recall	support
AFIB	0.858017	0.867150	0.849550	204.0
GSVT	0.883233	0.877348	0.889456	300.0
SB	0.901897	0.901091	0.902706	892.0
SR	0.894293	0.895486	0.893108	681.0
macro avg	0.884360	0.885269	0.883705	2077.0
micro avg	0.892424	0.892424	0.892424	2077.0
weighted avg	0.892398	0.892490	0.892424	2077.0

The result of Model Number:Dataset Number:Featuregroup Number:Rescale of 81 : 1 : 0 : 0

	f1-score	precision	recall	support
AFIB	0.597243	0.622178	0.574337	875.0
GSVT	0.645231	0.641964	0.648609	691.0
SB	0.707515	0.703533	0.711561	1553.0
SR	0.662481	0.647771	0.677887	907.0
macro avg	0.653117	0.653862	0.653099	4026.0
micro avg	0.663346	0.663346	0.663346	4026.0
weighted avg	0.662713	0.662722	0.663346	4026.0

The result of Model Number:Dataset Number:Featuregroup Number:Rescale of 81 : 1 : 0 : 1

	f1-score	precision	recall	support
AFIB	0.781577	0.780062	0.783200	875.0
GSVT	0.796253	0.782322	0.810767	691.0
SB	0.853013	0.840910	0.865478	1553.0
SR	0.808028	0.844049	0.774990	907.0
macro avg	0.809718	0.811836	0.808609	4026.0
micro avg	0.817820	0.817820	0.817820	4026.0
weighted avg	0.817611	0.818337	0.817820	4026.0

The result of Model Number:Dataset Number:Featuregroup Number:Rescale of 81 : 1 : 1 : 0

	f1-score	precision	recall	support
AFIB	0.886531	0.906251	0.867840	204.0
GSVT	0.924302	0.918558	0.930154	300.0
SB	0.944366	0.942377	0.946375	892.0
SR	0.934308	0.933647	0.934999	681.0
macro avg	0.922377	0.925208	0.919842	2077.0
micro avg	0.932589	0.932589	0.932589	2077.0
weighted avg	0.932490	0.932526	0.932589	2077.0

The result of Model Number:Dataset Number:Featuregroup Number:Rescale of 81 : 1 : 1 : 1

	f1-score	precision	recall	support
AFIB	0.599574	0.613237	0.586602	875.0
GSVT	0.638023	0.639284	0.636980	691.0
SB	0.698040	0.694219	0.701905	1553.0
SR	0.659753	0.651750	0.668046	907.0
macro avg	0.648847	0.649623	0.648383	4026.0
micro avg	0.658074	0.658074	0.658074	4026.0
weighted avg	0.657713	0.657622	0.658074	4026.0

The result of Model Number:Dataset Number:Featuregroup Number:Rescale of 81 : 1 : 2 : 0

	f1-score	precision	recall	support
AFIB	0.871900	0.886395	0.858071	204.0
GSVT	0.888619	0.882306	0.895089	300.0
SB	0.909230	0.904120	0.914419	892.0
SR	0.897646	0.903138	0.892250	681.0
macro avg	0.891849	0.893990	0.889957	2077.0
micro avg	0.898824	0.898824	0.898824	2077.0
weighted avg	0.898788	0.898906	0.898824	2077.0

The result of Model Number:Dataset Number:Featuregroup Number:Rescale of 81 : 1 : 2 : 1

	f1-score	precision	recall	support
AFIB	0.848505	0.854563	0.842684	204.0
GSVT	0.878174	0.877608	0.878758	300.0
SB	0.895745	0.893757	0.897750	892.0
SR	0.887738	0.888812	0.886694	681.0
macro avg	0.877541	0.878685	0.876472	2077.0
micro avg	0.885973	0.885973	0.885973	2077.0
weighted avg	0.885942	0.885954	0.885973	2077.0

The result of Model Number:Dataset Number:Featuregroup Number:Rescale of 81 : 1 : 3 : 0

	f1-score	precision	recall	support
AFIB	0.742939	0.744784	0.741153	875.0
GSVT	0.755733	0.758003	0.753572	691.0
SB	0.801697	0.796255	0.807219	1553.0
SR	0.786441	0.792279	0.780719	907.0
macro avg	0.771702	0.772830	0.770665	4026.0
micro avg	0.777682	0.777682	0.777682	4026.0
weighted avg	0.777600	0.777607	0.777682	4026.0

The result of Model Number:Dataset Number:Featuregroup Number:Rescale of 81 : 1 : 3 : 1

	f1-score	precision	recall	support
AFIB	0.698943	0.697812	0.700140	875.0

GSVT	0.705347	0.708152	0.702600	691.0
SB	0.740737	0.738045	0.743455	1553.0
SR	0.728621	0.732307	0.725013	907.0
macro avg	0.718412	0.719079	0.717802	4026.0
micro avg	0.722874	0.722874	0.722874	4026.0
weighted avg	0.722850	0.722877	0.722874	4026.0

The result of Model Number:Dataset Number:Featuregroup Number:Rescale of 81 : 1 : 4 : 0

	f1-score	precision	recall	support
AFIB	0.658900	0.717769	0.695813	204.0
GSVT	0.894969	0.880339	0.910402	300.0
SB	0.761895	0.756091	0.767844	892.0
SR	0.931673	0.921131	0.942486	681.0
macro avg	0.811859	0.818832	0.829136	2077.0
micro avg	0.838621	0.838621	0.838621	2077.0
weighted avg	0.826666	0.824386	0.838621	2077.0

The result of Model Number:Dataset Number:Featuregroup Number:Rescale of 81 : 1 : 4 : 1

	f1-score	precision	recall	support
AFIB	0.817321	0.853646	0.784075	204.0
GSVT	0.851381	0.844697	0.858190	300.0
SB	0.864386	0.857718	0.871166	892.0
SR	0.854823	0.856234	0.853446	681.0
macro avg	0.846978	0.853074	0.841719	2077.0
micro avg	0.854928	0.854928	0.854928	2077.0
weighted avg	0.854749	0.854951	0.854928	2077.0

The result of Model Number:Dataset Number:Featuregroup Number:Rescale of 81 : 1 : 5 : 0

	f1-score	precision	recall	support
AFIB	0.911455	0.931265	0.892720	204.0
GSVT	0.949079	0.943480	0.954770	300.0
SB	0.969469	0.967319	0.971640	892.0
SR	0.959750	0.959209	0.960316	681.0
macro avg	0.947438	0.950318	0.944862	2077.0
micro avg	0.957739	0.957739	0.957739	2077.0
weighted avg	0.957639	0.957675	0.957739	2077.0

The result of Model Number:Dataset Number:Featuregroup Number:Rescale of 81 : 1 : 5 : 1

	f1-score	precision	recall	support
AFIB	0.794638	0.822427	0.769032	204.0
GSVT	0.828197	0.823148	0.833421	300.0
SB	0.845203	0.841731	0.848708	892.0
SR	0.837389	0.836168	0.838619	681.0
macro avg	0.826357	0.830869	0.822445	2077.0

micro avg 0.835366 0.835366 0.835366 2077.0
weighted avg 0.835218 0.835327 0.835366 2077.0
The result of Model Number:Dataset Number:Featuregroup Number:Rescale of 81 : 1 : 6 : 0

	f1-score	precision	recall	support
AFIB	0.898089	0.898035	0.898199	875.0
GSVT	0.900033	0.895167	0.904967	691.0
SB	0.943031	0.936756	0.949402	1553.0
SR	0.917990	0.933052	0.903441	907.0
macro avg	0.914786	0.915752	0.914003	4026.0
micro avg	0.920293	0.920293	0.920293	4026.0
weighted avg	0.920242	0.920368	0.920293	4026.0

The result of Model Number:Dataset Number:Featuregroup Number:Rescale of 81 : 1 : 6 : 1

	f1-score	precision	recall	support
AFIB	0.779686	0.778984	0.780465	875.0
GSVT	0.793194	0.785931	0.800654	691.0
SB	0.832362	0.825044	0.839812	1553.0
SR	0.793175	0.812784	0.774560	907.0
macro avg	0.799604	0.800686	0.798873	4026.0
micro avg	0.805492	0.805492	0.805492	4026.0
weighted avg	0.805363	0.805558	0.805492	4026.0

The result of Model Number:Dataset Number:Featuregroup Number:Rescale of 81 : 1 : 7 : 0

	f1-score	precision	recall	support
AFIB	0.809690	0.805157	0.814314	875.0
GSVT	0.816283	0.821100	0.811574	691.0
SB	0.859888	0.857196	0.862599	1553.0
SR	0.842864	0.848440	0.837381	907.0
macro avg	0.832181	0.832973	0.831467	4026.0
micro avg	0.837666	0.837666	0.837666	4026.0
weighted avg	0.837659	0.837718	0.837666	4026.0

The result of Model Number:Dataset Number:Featuregroup Number:Rescale of 81 : 1 : 7 : 1

	f1-score	precision	recall	support
AFIB	0.766693	0.770327	0.763097	875.0
GSVT	0.776204	0.772542	0.779918	691.0
SB	0.818714	0.816985	0.820453	1553.0
SR	0.803199	0.805448	0.800981	907.0
macro avg	0.791202	0.791326	0.791113	4026.0
micro avg	0.796644	0.796644	0.796644	4026.0
weighted avg	0.796616	0.796618	0.796644	4026.0

The result of Model Number:Dataset Number:Featuregroup Number:Rescale of 81 : 1 : 8 : 0

f1-score precision recall support
AFIB 0.758341 0.740505 0.798367 875.0
GSVT 0.728697 0.721979 0.745302 691.0
SB 0.790321 0.734673 0.856000 1553.0
SR 0.492775 0.618019 0.410373 907.0
macro avg 0.692533 0.703794 0.702511 4026.0
micro avg 0.724081 0.724081 0.724081 4026.0
weighted avg 0.705761 0.707481 0.724081 4026.0
The result of Model Number:Dataset Number:Featuregroup Number:Rescale of 81 : 1 : 8 : 1
f1-score precision recall support
AFIB 0.638554 0.634533 0.719287 204.0
GSVT 0.778408 0.774404 0.782584 300.0
SB 0.800571 0.796762 0.804426 892.0
SR 0.627958 0.630027 0.626281 681.0
macro avg 0.711373 0.708931 0.733144 2077.0
micro avg 0.734499 0.734499 0.734499 2077.0
weighted avg 0.724861 0.722930 0.734499 2077.0
The result of Model Number:Dataset Number:Featuregroup Number:Rescale of 81 : 1 : 9 : 0
f1-score precision recall support
AFIB 0.705103 0.708802 0.701485 875.0
GSVT 0.719847 0.718554 0.721196 691.0
SB 0.781631 0.775636 0.787720 1553.0
SR 0.754054 0.761601 0.746659 907.0
macro avg 0.740159 0.741148 0.739265 4026.0
micro avg 0.748310 0.748310 0.748310 4026.0
weighted avg 0.748182 0.748152 0.748310 4026.0
The result of Model Number:Dataset Number:Featuregroup Number:Rescale of 81 : 1 : 9 : 1
f1-score precision recall support
AFIB 0.679032 0.683096 0.675051 204.0
GSVT 0.697121 0.695049 0.699210 300.0
SB 0.707796 0.706929 0.708668 892.0
SR 0.702636 0.703465 0.701815 681.0
macro avg 0.696646 0.697135 0.696186 2077.0
micro avg 0.701753 0.701753 0.701753 2077.0
weighted avg 0.701737 0.701736 0.701753 2077.0
The result of Model Number:Dataset Number:Featuregroup Number:Rescale of 81 : 1 : 10 : 0
f1-score precision recall support
AFIB 0.655458 0.635867 0.698889 875.0
GSVT 0.561303 0.549011 0.574365 691.0
SB 0.744501 0.738435 0.750865 1553.0

SR 0.698449 0.727774 0.672708 907.0
macro avg 0.664928 0.662772 0.674207 4026.0
micro avg 0.691668 0.691668 0.691668 4026.0
weighted avg 0.683331 0.681230 0.691668 4026.0
The result of Model Number:Dataset Number:Featuregroup Number:Rescale of 81 : 1 : 10 : 1

	f1-score	precision	recall	support
AFIB	0.709986	0.724290	0.696691	204.0
GSVT	0.732767	0.726882	0.738890	300.0
SB	0.748631	0.747877	0.749386	892.0
SR	0.741807	0.741381	0.742242	681.0
macro avg	0.733298	0.735107	0.731802	2077.0
micro avg	0.740352	0.740352	0.740352	2077.0
weighted avg	0.740306	0.740398	0.740352	2077.0

The result of Model Number:Dataset Number:Featuregroup Number:Rescale of 82 : 0 : 0 : 0

	f1-score	precision	recall	support
AFIB	0.639876	0.652156	0.628086	875.0
GSVT	0.682087	0.683128	0.681093	691.0
SB	0.742706	0.739442	0.746004	1553.0
SR	0.701834	0.694204	0.709705	907.0
macro avg	0.691626	0.692233	0.691222	4026.0
micro avg	0.701057	0.701057	0.701057	4026.0
weighted avg	0.700745	0.700615	0.701057	4026.0

The result of Model Number:Dataset Number:Featuregroup Number:Rescale of 82 : 0 : 0 : 1

	f1-score	precision	recall	support
AFIB	0.699826	0.692093	0.707747	875.0
GSVT	0.712435	0.716822	0.708114	691.0
SB	0.758251	0.756989	0.759520	1553.0
SR	0.739788	0.746705	0.733016	907.0
macro avg	0.727575	0.728152	0.727099	4026.0
micro avg	0.733474	0.733474	0.733474	4026.0
weighted avg	0.733530	0.733674	0.733474	4026.0

The result of Model Number:Dataset Number:Featuregroup Number:Rescale of 82 : 0 : 1 : 0

	f1-score	precision	recall	support
AFIB	0.755237	0.749422	0.761201	875.0
GSVT	0.765042	0.768594	0.761690	691.0
SB	0.819850	0.815488	0.824275	1553.0
SR	0.795694	0.806881	0.784901	907.0
macro avg	0.783956	0.785096	0.783017	4026.0
micro avg	0.790955	0.790955	0.790955	4026.0
weighted avg	0.790958	0.791142	0.790955	4026.0

The result of Model Number:Dataset Number:Featuregroup Number:Rescale of 82 : 0 : 1 : 1

	f1-score	precision	recall	support
AFIB	0.805771	0.800325	0.811401	875.0
GSVT	0.825429	0.825758	0.825207	691.0
SB	0.876850	0.874613	0.879109	1553.0
SR	0.852975	0.862505	0.843665	907.0
macro avg	0.840256	0.840800	0.839845	4026.0
micro avg	0.847157	0.847157	0.847157	4026.0
weighted avg	0.847198	0.847355	0.847157	4026.0

The result of Model Number:Dataset Number:Featuregroup Number:Rescale of 82 : 0 : 2 : 0

	f1-score	precision	recall	support
AFIB	0.811418	0.790654	0.833377	875.0
GSVT	0.767040	0.810514	0.728171	691.0
SB	0.813331	0.748460	0.890535	1553.0
SR	0.649714	0.789163	0.552265	907.0
macro avg	0.760376	0.784698	0.751087	4026.0
micro avg	0.774038	0.774038	0.774038	4026.0
weighted avg	0.768110	0.777451	0.774038	4026.0

The result of Model Number:Dataset Number:Featuregroup Number:Rescale of 82 : 0 : 2 : 1

	f1-score	precision	recall	support
AFIB	0.681618	0.706782	0.658247	204.0
GSVT	0.696187	0.689041	0.703589	300.0
SB	0.715254	0.711919	0.718622	892.0
SR	0.704102	0.704543	0.703685	681.0
macro avg	0.699290	0.703071	0.696036	2077.0
micro avg	0.705623	0.705623	0.705623	2077.0
weighted avg	0.705540	0.705692	0.705623	2077.0

The result of Model Number:Dataset Number:Featuregroup Number:Rescale of 82 : 0 : 3 : 0

	f1-score	precision	recall	support
AFIB	0.811655	0.799433	0.831013	875.0
GSVT	0.774984	0.772728	0.781595	691.0
SB	0.827583	0.771175	0.899776	1553.0
SR	0.613042	0.819455	0.507979	907.0
macro avg	0.756816	0.790698	0.755091	4026.0
micro avg	0.776281	0.776281	0.776281	4026.0
weighted avg	0.766760	0.788460	0.776281	4026.0

The result of Model Number:Dataset Number:Featuregroup Number:Rescale of 82 : 0 : 3 : 1

	f1-score	precision	recall	support
AFIB	0.761403	0.758307	0.764544	875.0

	f1-score	precision	recall	support
GSVT	0.772533	0.777203	0.767948	691.0
SB	0.814781	0.813789	0.815778	1553.0
SR	0.797783	0.799047	0.796547	907.0
macro avg	0.786625	0.787086	0.786204	4026.0
micro avg	0.792101	0.792101	0.792101	4026.0
weighted avg	0.792099	0.792130	0.792101	4026.0

The result of Model Number:Dataset Number:Featuregroup Number:Rescale of 82 : 0 : 4 : 0

	f1-score	precision	recall	support
AFIB	0.724245	0.727574	0.720982	875.0
GSVT	0.694141	0.698323	0.690032	691.0
SB	0.744580	0.711391	0.781055	1553.0
SR	0.636779	0.693217	0.588967	907.0
macro avg	0.699936	0.707626	0.695259	4026.0
micro avg	0.709101	0.709101	0.709101	4026.0
weighted avg	0.707217	0.708571	0.709101	4026.0

The result of Model Number:Dataset Number:Featuregroup Number:Rescale of 82 : 0 : 4 : 1

	f1-score	precision	recall	support
AFIB	0.855184	0.863928	0.846687	875.0
GSVT	0.854927	0.841719	0.868656	691.0
SB	0.901833	0.896448	0.907292	1553.0
SR	0.877964	0.889715	0.866545	907.0
macro avg	0.872477	0.872953	0.872295	4026.0
micro avg	0.878309	0.878309	0.878309	4026.0
weighted avg	0.878266	0.878470	0.878309	4026.0

The result of Model Number:Dataset Number:Featuregroup Number:Rescale of 82 : 0 : 5 : 0

	f1-score	precision	recall	support
AFIB	0.742720	0.740573	0.744948	875.0
GSVT	0.756485	0.755870	0.757154	691.0
SB	0.803497	0.800169	0.806860	1553.0
SR	0.779375	0.787938	0.771039	907.0
macro avg	0.770520	0.771137	0.770000	4026.0
micro avg	0.776803	0.776803	0.776803	4026.0
weighted avg	0.776785	0.776858	0.776803	4026.0

The result of Model Number:Dataset Number:Featuregroup Number:Rescale of 82 : 0 : 5 : 1

	f1-score	precision	recall	support
AFIB	0.770975	0.782347	0.760012	204.0
GSVT	0.789901	0.786801	0.793051	300.0
SB	0.807444	0.805825	0.809073	892.0
SR	0.801717	0.801838	0.801603	681.0
macro avg	0.792509	0.794203	0.790935	2077.0

micro avg 0.799491 0.799491 0.799491 2077.0
weighted avg 0.799450 0.799464 0.799491 2077.0
The result of Model Number:Dataset Number:Featuregroup Number:Rescale of 82 : 0 : 6 : 0

	f1-score	precision	recall	support
AFIB	0.754402	0.837232	0.686667	204.0
GSVT	0.880867	0.869854	0.892334	300.0
SB	0.937199	0.932416	0.942031	892.0
SR	0.915244	0.902448	0.928412	681.0
macro avg	0.871928	0.885488	0.862361	2077.0
micro avg	0.905306	0.905306	0.905306	2077.0
weighted avg	0.903909	0.904205	0.905306	2077.0

The result of Model Number:Dataset Number:Featuregroup Number:Rescale of 82 : 0 : 6 : 1

	f1-score	precision	recall	support
AFIB	0.676613	0.679692	0.673603	875.0
GSVT	0.679901	0.673124	0.686873	691.0
SB	0.716616	0.708808	0.724600	1553.0
SR	0.685366	0.701592	0.669877	907.0
macro avg	0.689624	0.690804	0.688738	4026.0
micro avg	0.694713	0.694713	0.694713	4026.0
weighted avg	0.694580	0.694729	0.694713	4026.0

The result of Model Number:Dataset Number:Featuregroup Number:Rescale of 82 : 0 : 7 : 0

	f1-score	precision	recall	support
AFIB	0.880998	0.881784	0.880304	875.0
GSVT	0.897135	0.899489	0.894945	691.0
SB	0.952321	0.945980	0.958757	1553.0
SR	0.933766	0.942291	0.925427	907.0
macro avg	0.916055	0.917386	0.914858	4026.0
micro avg	0.923245	0.923245	0.923245	4026.0
weighted avg	0.923168	0.923217	0.923245	4026.0

The result of Model Number:Dataset Number:Featuregroup Number:Rescale of 82 : 0 : 7 : 1

	f1-score	precision	recall	support
AFIB	0.679683	0.697644	0.662691	204.0
GSVT	0.698323	0.692522	0.704350	300.0
SB	0.717566	0.714933	0.720220	892.0
SR	0.706455	0.707311	0.705620	681.0
macro avg	0.700507	0.703102	0.698220	2077.0
micro avg	0.707491	0.707491	0.707491	2077.0
weighted avg	0.707423	0.707499	0.707491	2077.0

The result of Model Number:Dataset Number:Featuregroup Number:Rescale of 82 : 0 : 8 : 0

	f1-score	precision	recall	support
AFIB	0.832991	0.831376	0.834720	875.0
GSVT	0.848632	0.833784	0.864100	691.0
SB	0.909125	0.896226	0.922410	1553.0
SR	0.861181	0.899572	0.825970	907.0
macro avg	0.862982	0.865239	0.861800	4026.0
micro avg	0.871617	0.871617	0.871617	4026.0
weighted avg	0.871394	0.872168	0.871617	4026.0

The result of Model Number:Dataset Number:Featuregroup Number:Rescale of 82 : 0 : 8 : 1

	f1-score	precision	recall	support
AFIB	0.671589	0.663157	0.680433	875.0
GSVT	0.623101	0.641967	0.607396	691.0
SB	0.670867	0.621372	0.730705	1553.0
SR	0.521621	0.638424	0.444797	907.0
macro avg	0.621795	0.641230	0.615833	4026.0
micro avg	0.634204	0.634204	0.634204	4026.0
weighted avg	0.629203	0.637830	0.634204	4026.0

The result of Model Number:Dataset Number:Featuregroup Number:Rescale of 82 : 0 : 9 : 0

	f1-score	precision	recall	support
AFIB	0.820997	0.826859	0.815364	204.0
GSVT	0.849705	0.849157	0.850270	300.0
SB	0.866706	0.864782	0.868646	892.0
SR	0.858958	0.859997	0.857948	681.0
macro avg	0.849091	0.850199	0.848057	2077.0
micro avg	0.857251	0.857251	0.857251	2077.0
weighted avg	0.857220	0.857232	0.857251	2077.0

The result of Model Number:Dataset Number:Featuregroup Number:Rescale of 82 : 0 : 9 : 1

	f1-score	precision	recall	support
AFIB	0.860723	0.854096	0.867520	875.0
GSVT	0.871898	0.875946	0.868078	691.0
SB	0.934361	0.929390	0.939405	1553.0
SR	0.906831	0.919581	0.894530	907.0
macro avg	0.893453	0.894753	0.892383	4026.0
micro avg	0.901430	0.901430	0.901430	4026.0
weighted avg	0.901434	0.901643	0.901430	4026.0

The result of Model Number:Dataset Number:Featuregroup Number:Rescale of 82 : 0 : 10 : 0

	f1-score	precision	recall	support
AFIB	0.798536	0.798487	0.798634	875.0
GSVT	0.800264	0.795937	0.804652	691.0
SB	0.838496	0.832916	0.844161	1553.0

SR 0.816231 0.829623 0.803294 907.0
macro avg 0.813382 0.814241 0.812685 4026.0
micro avg 0.818278 0.818278 0.818278 4026.0
weighted avg 0.818233 0.818345 0.818278 4026.0
The result of Model Number:Dataset Number:Featuregroup Number:Rescale of 82 : 0 : 10 : 1

	f1-score	precision	recall	support
AFIB	0.858490	0.875711	0.842103	204.0
GSVT	0.878385	0.869255	0.887756	300.0
SB	0.894631	0.891150	0.898141	892.0
SR	0.885230	0.888923	0.881587	681.0
macro avg	0.879184	0.881260	0.877397	2077.0
micro avg	0.885709	0.885709	0.885709	2077.0
weighted avg	0.885652	0.885741	0.885709	2077.0

The result of Model Number:Dataset Number:Featuregroup Number:Rescale of 82 : 1 : 0 : 0

	f1-score	precision	recall	support
AFIB	0.854421	0.854827	0.854137	875.0
GSVT	0.876549	0.868962	0.884300	691.0
SB	0.936157	0.929335	0.943089	1553.0
SR	0.896163	0.913978	0.879076	907.0
macro avg	0.890823	0.891776	0.890150	4026.0
micro avg	0.899245	0.899245	0.899245	4026.0
weighted avg	0.899152	0.899320	0.899245	4026.0

The result of Model Number:Dataset Number:Featuregroup Number:Rescale of 82 : 1 : 0 : 1

	f1-score	precision	recall	support
AFIB	0.793507	0.805537	0.781850	204.0
GSVT	0.806266	0.799469	0.813222	300.0
SB	0.824150	0.821492	0.826828	892.0
SR	0.815303	0.818332	0.812319	681.0
macro avg	0.809807	0.811207	0.808555	2077.0
micro avg	0.815688	0.815688	0.815688	2077.0
weighted avg	0.815657	0.815708	0.815688	2077.0

The result of Model Number:Dataset Number:Featuregroup Number:Rescale of 82 : 1 : 1 : 0

	f1-score	precision	recall	support
AFIB	0.664181	0.688806	0.641351	204.0
GSVT	0.684079	0.675526	0.692939	300.0
SB	0.701426	0.695026	0.707951	892.0
SR	0.682985	0.688285	0.677772	681.0
macro avg	0.683168	0.686911	0.680003	2077.0
micro avg	0.689346	0.689346	0.689346	2077.0
weighted avg	0.689216	0.689388	0.689346	2077.0

The result of Model Number:Dataset Number:Featuregroup Number:Rescale of 82 : 1 : 1 : 1

	f1-score	precision	recall	support
AFIB	0.868784	0.858648	0.881428	875.0
GSVT	0.893926	0.883091	0.905751	691.0
SB	0.907400	0.872374	0.958777	1553.0
SR	0.720523	0.758530	0.686186	907.0
macro avg	0.847658	0.843161	0.858035	4026.0
micro avg	0.871454	0.871454	0.871454	4026.0
weighted avg	0.854594	0.845583	0.871454	4026.0

The result of Model Number:Dataset Number:Featuregroup Number:Rescale of 82 : 1 : 2 : 0

	f1-score	precision	recall	support
AFIB	0.695424	0.701489	0.689636	204.0
GSVT	0.719474	0.718299	0.720670	300.0
SB	0.734767	0.732974	0.736576	892.0
SR	0.728030	0.729128	0.726961	681.0
macro avg	0.719424	0.720472	0.718461	2077.0
micro avg	0.726516	0.726516	0.726516	2077.0
weighted avg	0.726485	0.726501	0.726516	2077.0

The result of Model Number:Dataset Number:Featuregroup Number:Rescale of 82 : 1 : 2 : 1

	f1-score	precision	recall	support
AFIB	0.675703	0.677009	0.674453	875.0
GSVT	0.654642	0.651767	0.657583	691.0
SB	0.684023	0.642448	0.731370	1553.0
SR	0.550543	0.637919	0.484350	907.0
macro avg	0.641228	0.652286	0.636939	4026.0
micro avg	0.650685	0.650685	0.650685	4026.0
weighted avg	0.647101	0.650538	0.650685	4026.0

The result of Model Number:Dataset Number:Featuregroup Number:Rescale of 82 : 1 : 3 : 0

	f1-score	precision	recall	support
AFIB	0.694870	0.696803	0.692984	875.0
GSVT	0.710787	0.710590	0.710992	691.0
SB	0.757011	0.749896	0.764271	1553.0
SR	0.736952	0.747580	0.726631	907.0
macro avg	0.724905	0.726217	0.723719	4026.0
micro avg	0.731153	0.731153	0.731153	4026.0
weighted avg	0.731053	0.731089	0.731153	4026.0

The result of Model Number:Dataset Number:Featuregroup Number:Rescale of 82 : 1 : 3 : 1

	f1-score	precision	recall	support
AFIB	0.679003	0.682716	0.675353	875.0

GSVT 0.675767 0.670069 0.681631 691.0
SB 0.710738 0.704007 0.717602 1553.0
SR 0.684287 0.697051 0.671991 907.0
macro avg 0.687449 0.688461 0.686644 4026.0
micro avg 0.691970 0.691970 0.691970 4026.0
weighted avg 0.691880 0.691988 0.691970 4026.0
The result of Model Number:Dataset Number:Featuregroup Number:Rescale of 82 : 1 : 4 : 0

	f1-score	precision	recall	support
AFIB	0.789550	0.809241	0.770855	875.0
GSVT	0.839943	0.843163	0.836787	691.0
SB	0.909588	0.905184	0.914038	1553.0
SR	0.861885	0.847519	0.876802	907.0
macro avg	0.850242	0.851277	0.849620	4026.0
micro avg	0.861271	0.861271	0.861271	4026.0
weighted avg	0.860799	0.860696	0.861271	4026.0

The result of Model Number:Dataset Number:Featuregroup Number:Rescale of 82 : 1 : 4 : 1

	f1-score	precision	recall	support
AFIB	0.862693	0.877671	0.848327	204.0
GSVT	0.879693	0.877090	0.882330	300.0
SB	0.890066	0.886900	0.893259	892.0
SR	0.882830	0.883751	0.881930	681.0
macro avg	0.878820	0.881353	0.876461	2077.0
micro avg	0.883553	0.883553	0.883553	2077.0
weighted avg	0.883507	0.883544	0.883553	2077.0

The result of Model Number:Dataset Number:Featuregroup Number:Rescale of 82 : 1 : 5 : 0

	f1-score	precision	recall	support
AFIB	0.810675	0.805374	0.816117	875.0
GSVT	0.834770	0.841873	0.827898	691.0
SB	0.888725	0.886790	0.890671	1553.0
SR	0.867067	0.870548	0.863626	907.0
macro avg	0.850309	0.851146	0.849578	4026.0
micro avg	0.857601	0.857601	0.857601	4026.0
weighted avg	0.857622	0.857727	0.857601	4026.0

The result of Model Number:Dataset Number:Featuregroup Number:Rescale of 82 : 1 : 5 : 1

	f1-score	precision	recall	support
AFIB	0.564179	0.614585	0.595785	204.0
GSVT	0.766311	0.753785	0.779526	300.0
SB	0.652368	0.647399	0.657462	892.0
SR	0.797739	0.788713	0.806998	681.0
macro avg	0.695149	0.701120	0.709943	2077.0

micro avg 0.718065 0.718065 0.718065 2077.0
weighted avg 0.707828 0.705876 0.718065 2077.0
The result of Model Number:Dataset Number:Featuregroup Number:Rescale of 82 : 1 : 6 : 0

	f1-score	precision	recall	support
AFIB	0.858499	0.868536	0.848856	204.0
GSVT	0.882116	0.878060	0.886233	300.0
SB	0.894605	0.891823	0.897411	892.0
SR	0.886900	0.889414	0.884439	681.0
macro avg	0.880530	0.881958	0.879235	2077.0
micro avg	0.886774	0.886774	0.886774	2077.0
weighted avg	0.886729	0.886758	0.886774	2077.0

The result of Model Number:Dataset Number:Featuregroup Number:Rescale of 82 : 1 : 6 : 1

	f1-score	precision	recall	support
AFIB	0.585041	0.645176	0.535519	204.0
GSVT	0.696181	0.686773	0.705920	300.0
SB	0.741384	0.738419	0.744375	892.0
SR	0.721261	0.711511	0.731313	681.0
macro avg	0.685967	0.695470	0.679282	2077.0
micro avg	0.714025	0.714025	0.714025	2077.0
weighted avg	0.712901	0.712979	0.714025	2077.0

The result of Model Number:Dataset Number:Featuregroup Number:Rescale of 82 : 1 : 7 : 0

	f1-score	precision	recall	support
AFIB	0.826773	0.866466	0.790722	204.0
GSVT	0.862734	0.855408	0.870210	300.0
SB	0.876292	0.869910	0.882774	892.0
SR	0.867075	0.867476	0.866699	681.0
macro avg	0.858218	0.864815	0.852601	2077.0
micro avg	0.866647	0.866647	0.866647	2077.0
weighted avg	0.866448	0.866679	0.866647	2077.0

The result of Model Number:Dataset Number:Featuregroup Number:Rescale of 82 : 1 : 7 : 1

	f1-score	precision	recall	support
AFIB	0.822404	0.815131	0.829843	875.0
GSVT	0.747708	0.724098	0.831840	691.0
SB	0.674099	0.647228	0.716121	1553.0
SR	0.694757	0.701946	0.687759	907.0
macro avg	0.734742	0.722101	0.766391	4026.0
micro avg	0.754309	0.754309	0.754309	4026.0
weighted avg	0.723619	0.709240	0.754309	4026.0

The result of Model Number:Dataset Number:Featuregroup Number:Rescale of 82 : 1 : 8 : 0

	f1-score	precision	recall	support
AFIB	0.852612	0.873072	0.833477	204.0
GSVT	0.882929	0.877811	0.888213	300.0
SB	0.901786	0.898478	0.905121	892.0
SR	0.893949	0.894612	0.893297	681.0
macro avg	0.882819	0.885993	0.880027	2077.0
micro avg	0.891765	0.891765	0.891765	2077.0
weighted avg	0.891663	0.891730	0.891765	2077.0

The result of Model Number:Dataset Number:Featuregroup Number:Rescale of 82 : 1 : 8 : 1

	f1-score	precision	recall	support
AFIB	0.643984	0.701520	0.680061	204.0
GSVT	0.874709	0.860410	0.889793	300.0
SB	0.744647	0.738975	0.750462	892.0
SR	0.910582	0.900279	0.921151	681.0
macro avg	0.793480	0.800296	0.810367	2077.0
micro avg	0.819637	0.819637	0.819637	2077.0
weighted avg	0.807952	0.805724	0.819637	2077.0

The result of Model Number:Dataset Number:Featuregroup Number:Rescale of 82 : 1 : 9 : 0

	f1-score	precision	recall	support
AFIB	0.759402	0.833850	0.697900	204.0
GSVT	0.890979	0.878970	0.903464	300.0
SB	0.945941	0.942786	0.949122	892.0
SR	0.921358	0.909005	0.934074	681.0
macro avg	0.879420	0.891153	0.871140	2077.0
micro avg	0.912919	0.912919	0.912919	2077.0
weighted avg	0.911621	0.911793	0.912919	2077.0

The result of Model Number:Dataset Number:Featuregroup Number:Rescale of 82 : 1 : 9 : 1

	f1-score	precision	recall	support
AFIB	0.709564	0.738204	0.683120	204.0
GSVT	0.735969	0.724014	0.748453	300.0
SB	0.755009	0.748939	0.761188	892.0
SR	0.734696	0.739987	0.729520	681.0
macro avg	0.733810	0.737786	0.730570	2077.0
micro avg	0.741297	0.741297	0.741297	2077.0
weighted avg	0.741135	0.741349	0.741297	2077.0

The result of Model Number:Dataset Number:Featuregroup Number:Rescale of 82 : 1 : 10 : 0

	f1-score	precision	recall	support
AFIB	0.765251	0.764550	0.766045	875.0
GSVT	0.780936	0.779422	0.782539	691.0
SB	0.828675	0.826830	0.830542	1553.0

SR	0.806057	0.811316	0.800896	907.0
macro avg	0.795230	0.795529	0.795005	4026.0
micro avg	0.801606	0.801606	0.801606	4026.0
weighted avg	0.801602	0.801662	0.801606	4026.0

The result of Model Number:Dataset Number:Featuregroup Number:Rescale of 82 : 1 : 10 : 1

	f1-score	precision	recall	support
AFIB	0.626580	0.690218	0.574288	204.0
GSVT	0.578643	0.618657	0.543762	300.0
SB	0.629971	0.588628	0.677570	892.0
SR	0.545250	0.573912	0.519465	681.0
macro avg	0.595111	0.617854	0.578771	2077.0
micro avg	0.596259	0.596259	0.596259	2077.0
weighted avg	0.594446	0.598118	0.596259	2077.0

The result of Model Number:Dataset Number:Featuregroup Number:Rescale of 83 : 0 : 0 : 0

	f1-score	precision	recall	support
AFIB	0.861904	0.866300	0.857579	875.0
GSVT	0.873798	0.869252	0.878468	691.0
SB	0.915877	0.915059	0.916704	1553.0
SR	0.899012	0.899746	0.898318	907.0
macro avg	0.887648	0.887589	0.887767	4026.0
micro avg	0.893149	0.893149	0.893149	4026.0
weighted avg	0.893125	0.893150	0.893149	4026.0

The result of Model Number:Dataset Number:Featuregroup Number:Rescale of 83 : 0 : 0 : 1

	f1-score	precision	recall	support
AFIB	0.869831	0.904939	0.837414	204.0
GSVT	0.902200	0.887544	0.917503	300.0
SB	0.925540	0.918099	0.933114	892.0
SR	0.900640	0.907125	0.894294	681.0
macro avg	0.899553	0.904427	0.895581	2077.0
micro avg	0.908732	0.908732	0.908732	2077.0
weighted avg	0.908533	0.908795	0.908732	2077.0

The result of Model Number:Dataset Number:Featuregroup Number:Rescale of 83 : 0 : 1 : 0

	f1-score	precision	recall	support
AFIB	0.727072	0.787767	0.676509	204.0
GSVT	0.779869	0.770062	0.790246	300.0
SB	0.799778	0.788573	0.811398	892.0
SR	0.777961	0.781606	0.774730	681.0
macro avg	0.771170	0.782002	0.763221	2077.0
micro avg	0.783072	0.783072	0.783072	2077.0
weighted avg	0.782608	0.783536	0.783072	2077.0

The result of Model Number:Dataset Number:Featuregroup Number:Rescale of 83 : 0 : 1 : 1

	f1-score	precision	recall	support
AFIB	0.672580	0.675783	0.669437	875.0
GSVT	0.680889	0.668515	0.693799	691.0
SB	0.720432	0.717673	0.723214	1553.0
SR	0.699138	0.711104	0.687578	907.0
macro avg	0.693259	0.693269	0.693507	4026.0
micro avg	0.698449	0.698449	0.698449	4026.0
weighted avg	0.698447	0.698652	0.698449	4026.0

The result of Model Number:Dataset Number:Featuregroup Number:Rescale of 83 : 0 : 2 : 0

	f1-score	precision	recall	support
AFIB	0.775864	0.792138	0.760266	204.0
GSVT	0.793862	0.789165	0.798624	300.0
SB	0.813216	0.811312	0.815130	892.0
SR	0.806347	0.806115	0.806586	681.0
macro avg	0.797322	0.799683	0.795152	2077.0
micro avg	0.804556	0.804556	0.804556	2077.0
weighted avg	0.804500	0.804526	0.804556	2077.0

The result of Model Number:Dataset Number:Featuregroup Number:Rescale of 83 : 0 : 2 : 1

	f1-score	precision	recall	support
AFIB	0.821042	0.808771	0.833722	875.0
GSVT	0.836784	0.843941	0.829761	691.0
SB	0.894085	0.891006	0.897190	1553.0
SR	0.867915	0.880860	0.855375	907.0
macro avg	0.854957	0.856144	0.854012	4026.0
micro avg	0.862403	0.862403	0.862403	4026.0
weighted avg	0.862479	0.862769	0.862403	4026.0

The result of Model Number:Dataset Number:Featuregroup Number:Rescale of 83 : 0 : 3 : 0

	f1-score	precision	recall	support
AFIB	0.720960	0.781146	0.670823	204.0
GSVT	0.773314	0.763590	0.783604	300.0
SB	0.793055	0.781945	0.804578	892.0
SR	0.771422	0.775036	0.768218	681.0
macro avg	0.764688	0.775429	0.756806	2077.0
micro avg	0.776490	0.776490	0.776490	2077.0
weighted avg	0.776030	0.776950	0.776490	2077.0

The result of Model Number:Dataset Number:Featuregroup Number:Rescale of 83 : 0 : 3 : 1

	f1-score	precision	recall	support
AFIB	0.857322	0.876242	0.839265	204.0

GSVT 0.876058 0.861530 0.891189 300.0
SB 0.892892 0.885908 0.899997 892.0
SR 0.871222 0.881708 0.860985 681.0
macro avg 0.874373 0.876347 0.872859 2077.0
micro avg 0.879968 0.879968 0.879968 2077.0
weighted avg 0.879862 0.880061 0.879968 2077.0
The result of Model Number:Dataset Number:Featuregroup Number:Rescale of 83 : 0 : 4 : 0

	f1-score	precision	recall	support
AFIB	0.884972	0.922900	0.850374	204.0
GSVT	0.914710	0.900890	0.929019	300.0
SB	0.932853	0.923627	0.942267	892.0
SR	0.907579	0.915344	0.899979	681.0
macro avg	0.910028	0.915690	0.905410	2077.0
micro avg	0.917463	0.917463	0.917463	2077.0
weighted avg	0.917243	0.917556	0.917463	2077.0

The result of Model Number:Dataset Number:Featuregroup Number:Rescale of 83 : 0 : 4 : 1

	f1-score	precision	recall	support
AFIB	0.775822	0.813070	0.741993	204.0
GSVT	0.809568	0.802693	0.816583	300.0
SB	0.822290	0.816301	0.828372	892.0
SR	0.813640	0.814017	0.813288	681.0
macro avg	0.805330	0.811520	0.800059	2077.0
micro avg	0.813239	0.813239	0.813239	2077.0
weighted avg	0.813052	0.813270	0.813239	2077.0

The result of Model Number:Dataset Number:Featuregroup Number:Rescale of 83 : 0 : 5 : 0

	f1-score	precision	recall	support
AFIB	0.806399	0.787604	0.826242	875.0
GSVT	0.774114	0.782624	0.765840	691.0
SB	0.814632	0.762028	0.875055	1553.0
SR	0.651985	0.782909	0.558925	907.0
macro avg	0.761783	0.778791	0.756516	4026.0
micro avg	0.774482	0.774482	0.774482	4026.0
weighted avg	0.769246	0.775826	0.774482	4026.0

The result of Model Number:Dataset Number:Featuregroup Number:Rescale of 83 : 0 : 5 : 1

	f1-score	precision	recall	support
AFIB	0.671284	0.674025	0.668586	875.0
GSVT	0.625095	0.652682	0.599847	691.0
SB	0.671315	0.608124	0.749170	1553.0
SR	0.506396	0.630257	0.423392	907.0
macro avg	0.618522	0.641272	0.610249	4026.0

micro avg 0.632634 0.632634 0.632634 4026.0
weighted avg 0.626221 0.635081 0.632634 4026.0
The result of Model Number:Dataset Number:Featuregroup Number:Rescale of 83 : 0 : 6 : 0

	f1-score	precision	recall	support
AFIB	0.675453	0.691510	0.660201	204.0
GSVT	0.692989	0.690314	0.695756	300.0
SB	0.710161	0.708576	0.711753	892.0
SR	0.701226	0.699815	0.702668	681.0
macro avg	0.694957	0.697554	0.692594	2077.0
micro avg	0.701400	0.701400	0.701400	2077.0
weighted avg	0.701342	0.701390	0.701400	2077.0

The result of Model Number:Dataset Number:Featuregroup Number:Rescale of 83 : 0 : 6 : 1

	f1-score	precision	recall	support
AFIB	0.899596	0.907797	0.891659	204.0
GSVT	0.923072	0.920050	0.926143	300.0
SB	0.939947	0.939320	0.940577	892.0
SR	0.931680	0.931412	0.931961	681.0
macro avg	0.923574	0.924645	0.922585	2077.0
micro avg	0.930863	0.930863	0.930863	2077.0
weighted avg	0.930836	0.930848	0.930863	2077.0

The result of Model Number:Dataset Number:Featuregroup Number:Rescale of 83 : 0 : 7 : 0

	f1-score	precision	recall	support
AFIB	0.865668	0.868906	0.862822	875.0
GSVT	0.885153	0.893693	0.876953	691.0
SB	0.941195	0.931323	0.951351	1553.0
SR	0.908603	0.916599	0.901057	907.0
macro avg	0.900155	0.902630	0.898046	4026.0
micro avg	0.908010	0.908010	0.908010	4026.0
weighted avg	0.907819	0.907982	0.908010	4026.0

The result of Model Number:Dataset Number:Featuregroup Number:Rescale of 83 : 0 : 7 : 1

	f1-score	precision	recall	support
AFIB	0.778541	0.779594	0.777639	875.0
GSVT	0.790666	0.790260	0.791103	691.0
SB	0.825769	0.822815	0.828748	1553.0
SR	0.805995	0.810513	0.801579	907.0
macro avg	0.800243	0.800795	0.799767	4026.0
micro avg	0.805058	0.805058	0.805058	4026.0
weighted avg	0.805025	0.805062	0.805058	4026.0

The result of Model Number:Dataset Number:Featuregroup Number:Rescale of 83 : 0 : 8 : 0

	f1-score	precision	recall	support
AFIB	0.780313	0.785976	0.774786	875.0
GSVT	0.797429	0.790017	0.805041	691.0
SB	0.857003	0.851829	0.862245	1553.0
SR	0.828915	0.838171	0.819865	907.0
macro avg	0.815915	0.816498	0.815485	4026.0
micro avg	0.823871	0.823871	0.823871	4026.0
weighted avg	0.823783	0.823831	0.823871	4026.0

The result of Model Number:Dataset Number:Featuregroup Number:Rescale of 83 : 0 : 8 : 1

	f1-score	precision	recall	support
AFIB	0.576471	0.635724	0.527675	204.0
GSVT	0.685983	0.676713	0.695579	300.0
SB	0.730523	0.727602	0.733471	892.0
SR	0.710696	0.701089	0.720600	681.0
macro avg	0.675918	0.685282	0.669331	2077.0
micro avg	0.703565	0.703565	0.703565	2077.0
weighted avg	0.702458	0.702534	0.703565	2077.0

The result of Model Number:Dataset Number:Featuregroup Number:Rescale of 83 : 0 : 9 : 0

	f1-score	precision	recall	support
AFIB	0.888832	0.888032	0.889720	875.0
GSVT	0.904230	0.895951	0.912735	691.0
SB	0.948882	0.940539	0.957375	1553.0
SR	0.904210	0.926563	0.882988	907.0
macro avg	0.911539	0.912771	0.910704	4026.0
micro avg	0.918251	0.918251	0.918251	4026.0
weighted avg	0.918103	0.918326	0.918251	4026.0

The result of Model Number:Dataset Number:Featuregroup Number:Rescale of 83 : 0 : 9 : 1

	f1-score	precision	recall	support
AFIB	0.653952	0.646203	0.661944	875.0
GSVT	0.663260	0.669672	0.657034	691.0
SB	0.703940	0.701246	0.706659	1553.0
SR	0.687528	0.695460	0.679795	907.0
macro avg	0.677170	0.678145	0.676358	4026.0
micro avg	0.682371	0.682371	0.682371	4026.0
weighted avg	0.682396	0.682561	0.682371	4026.0

The result of Model Number:Dataset Number:Featuregroup Number:Rescale of 83 : 0 : 10 : 0

	f1-score	precision	recall	support
AFIB	0.755166	0.832788	0.691244	204.0
GSVT	0.898626	0.886481	0.911196	300.0
SB	0.956972	0.953145	0.960834	892.0

SR 0.930998 0.918413 0.943973 681.0
macro avg 0.885440 0.897707 0.876812 2077.0
micro avg 0.921657 0.921657 0.921657 2077.0
weighted avg 0.920207 0.920307 0.921657 2077.0
The result of Model Number:Dataset Number:Featuregroup Number:Rescale of 83 : 0 : 10 : 1

	f1-score	precision	recall	support
AFIB	0.911765	0.930890	0.893435	204.0
GSVT	0.932916	0.927396	0.938511	300.0
SB	0.955660	0.953422	0.957909	892.0
SR	0.947588	0.947315	0.947869	681.0
macro avg	0.936982	0.939756	0.934431	2077.0
micro avg	0.945483	0.945483	0.945483	2077.0
weighted avg	0.945417	0.945447	0.945483	2077.0

The result of Model Number:Dataset Number:Featuregroup Number:Rescale of 83 : 1 : 0 : 0

	f1-score	precision	recall	support
AFIB	0.871550	0.884354	0.859191	204.0
GSVT	0.893632	0.886035	0.901379	300.0
SB	0.909077	0.905440	0.912745	892.0
SR	0.900086	0.904504	0.895721	681.0
macro avg	0.893586	0.895083	0.892259	2077.0
micro avg	0.900262	0.900262	0.900262	2077.0
weighted avg	0.900212	0.900259	0.900262	2077.0

The result of Model Number:Dataset Number:Featuregroup Number:Rescale of 83 : 1 : 0 : 1

	f1-score	precision	recall	support
AFIB	0.687352	0.689673	0.685071	875.0
GSVT	0.685537	0.680708	0.690497	691.0
SB	0.721311	0.714617	0.728137	1553.0
SR	0.693888	0.707261	0.681047	907.0
macro avg	0.697022	0.698065	0.696188	4026.0
micro avg	0.701708	0.701708	0.701708	4026.0
weighted avg	0.701613	0.701718	0.701708	4026.0

The result of Model Number:Dataset Number:Featuregroup Number:Rescale of 83 : 1 : 1 : 0

	f1-score	precision	recall	support
AFIB	0.792384	0.817331	0.769108	204.0
GSVT	0.752807	0.768887	0.737511	300.0
SB	0.777289	0.750090	0.806564	892.0
SR	0.709862	0.733853	0.687440	681.0
macro avg	0.758085	0.767540	0.750156	2077.0
micro avg	0.753853	0.753853	0.753853	2077.0
weighted avg	0.753128	0.754086	0.753853	2077.0

The result of Model Number:Dataset Number:Featuregroup Number:Rescale of 83 : 1 : 1 : 1

	f1-score	precision	recall	support
AFIB	0.651283	0.650556	0.652032	875.0
GSVT	0.663197	0.654902	0.671724	691.0
SB	0.696684	0.691084	0.702382	1553.0
SR	0.665602	0.683066	0.649019	907.0
macro avg	0.669192	0.669902	0.668789	4026.0
micro avg	0.674155	0.674155	0.674155	4026.0
weighted avg	0.674067	0.674259	0.674155	4026.0

The result of Model Number:Dataset Number:Featuregroup Number:Rescale of 83 : 1 : 2 : 0

	f1-score	precision	recall	support
AFIB	0.792113	0.799856	0.784703	875.0
GSVT	0.786062	0.780098	0.792377	691.0
SB	0.831743	0.823186	0.840554	1553.0
SR	0.805342	0.818294	0.793022	907.0
macro avg	0.803815	0.805359	0.802664	4026.0
micro avg	0.809438	0.809438	0.809438	4026.0
weighted avg	0.809342	0.809618	0.809438	4026.0

The result of Model Number:Dataset Number:Featuregroup Number:Rescale of 83 : 1 : 2 : 1

	f1-score	precision	recall	support
AFIB	0.857686	0.861590	0.853871	875.0
GSVT	0.861854	0.853264	0.870693	691.0
SB	0.908396	0.898497	0.918515	1553.0
SR	0.868782	0.889350	0.849148	907.0
macro avg	0.874180	0.875675	0.873057	4026.0
micro avg	0.880630	0.880630	0.880630	4026.0
weighted avg	0.880462	0.880651	0.880630	4026.0

The result of Model Number:Dataset Number:Featuregroup Number:Rescale of 83 : 1 : 3 : 0

	f1-score	precision	recall	support
AFIB	0.660468	0.732985	0.601167	204.0
GSVT	0.771186	0.761545	0.781226	300.0
SB	0.820504	0.816317	0.824735	892.0
SR	0.801283	0.790080	0.812811	681.0
macro avg	0.763360	0.775232	0.754985	2077.0
micro avg	0.792583	0.792583	0.792583	2077.0
weighted avg	0.791360	0.791619	0.792583	2077.0

The result of Model Number:Dataset Number:Featuregroup Number:Rescale of 83 : 1 : 3 : 1

	f1-score	precision	recall	support
AFIB	0.794167	0.781711	0.807112	875.0

GSVT 0.765232 0.787170 0.744573 691.0
SB 0.804132 0.763370 0.849539 1553.0
SR 0.653352 0.727048 0.593323 907.0
macro avg 0.754221 0.764825 0.748637 4026.0
micro avg 0.764580 0.764580 0.764580 4026.0
weighted avg 0.761321 0.763258 0.764580 4026.0
The result of Model Number:Dataset Number:Featuregroup Number:Rescale of 83 : 1 : 4 : 0

	f1-score	precision	recall	support
AFIB	0.671839	0.677442	0.666558	204.0
GSVT	0.689587	0.686411	0.692806	300.0
SB	0.699986	0.697500	0.702495	892.0
SR	0.693521	0.696627	0.690478	681.0
macro avg	0.688733	0.689495	0.688085	2077.0
micro avg	0.693626	0.693626	0.693626	2077.0
weighted avg	0.693600	0.693642	0.693626	2077.0

The result of Model Number:Dataset Number:Featuregroup Number:Rescale of 83 : 1 : 4 : 1

	f1-score	precision	recall	support
AFIB	0.694023	0.698699	0.689512	875.0
GSVT	0.706409	0.696597	0.716631	691.0
SB	0.752756	0.747601	0.757989	1553.0
SR	0.722251	0.734727	0.710231	907.0
macro avg	0.718860	0.719406	0.718591	4026.0
micro avg	0.725249	0.725249	0.725249	4026.0
weighted avg	0.725164	0.725319	0.725249	4026.0

The result of Model Number:Dataset Number:Featuregroup Number:Rescale of 83 : 1 : 5 : 0

	f1-score	precision	recall	support
AFIB	0.706782	0.720574	0.693631	204.0
GSVT	0.724398	0.715050	0.734041	300.0
SB	0.736727	0.729444	0.744161	892.0
SR	0.721328	0.731350	0.711582	681.0
macro avg	0.722309	0.724105	0.720854	2077.0
micro avg	0.727054	0.727054	0.727054	2077.0
weighted avg	0.726956	0.727119	0.727054	2077.0

The result of Model Number:Dataset Number:Featuregroup Number:Rescale of 83 : 1 : 5 : 1

	f1-score	precision	recall	support
AFIB	0.913776	0.923719	0.904311	204.0
GSVT	0.944699	0.940759	0.948825	300.0
SB	0.964058	0.963195	0.964922	892.0
SR	0.954947	0.954955	0.954947	681.0
macro avg	0.944370	0.945657	0.943251	2077.0

micro avg 0.953373 0.953373 0.953373 2077.0
weighted avg 0.953336 0.953376 0.953373 2077.0
The result of Model Number:Dataset Number:Featuregroup Number:Rescale of 83 : 1 : 6 : 0

	f1-score	precision	recall	support
AFIB	0.684548	0.686860	0.682276	875.0
GSVT	0.682740	0.677931	0.687680	691.0
SB	0.718368	0.711701	0.725166	1553.0
SR	0.691057	0.704375	0.678268	907.0
macro avg	0.694178	0.695217	0.693348	4026.0
micro avg	0.698845	0.698845	0.698845	4026.0
weighted avg	0.698750	0.698855	0.698845	4026.0

The result of Model Number:Dataset Number:Featuregroup Number:Rescale of 83 : 1 : 6 : 1

	f1-score	precision	recall	support
AFIB	0.667579	0.684203	0.651776	875.0
GSVT	0.705345	0.708340	0.702405	691.0
SB	0.765075	0.762147	0.768029	1553.0
SR	0.727754	0.714193	0.741879	907.0
macro avg	0.716438	0.717221	0.716022	4026.0
micro avg	0.725609	0.725609	0.725609	4026.0
weighted avg	0.725226	0.725168	0.725609	4026.0

The result of Model Number:Dataset Number:Featuregroup Number:Rescale of 83 : 1 : 7 : 0

	f1-score	precision	recall	support
AFIB	0.674148	0.730426	0.627266	204.0
GSVT	0.723102	0.714009	0.732724	300.0
SB	0.741562	0.731173	0.752337	892.0
SR	0.721333	0.724712	0.718337	681.0
macro avg	0.715036	0.725080	0.707666	2077.0
micro avg	0.726072	0.726072	0.726072	2077.0
weighted avg	0.725642	0.726502	0.726072	2077.0

The result of Model Number:Dataset Number:Featuregroup Number:Rescale of 83 : 1 : 7 : 1

	f1-score	precision	recall	support
AFIB	0.803752	0.839026	0.771526	204.0
GSVT	0.748240	0.753628	0.743237	300.0
SB	0.775984	0.737017	0.819305	892.0
SR	0.688420	0.733872	0.648414	681.0
macro avg	0.754099	0.765886	0.745621	2077.0
micro avg	0.747594	0.747594	0.747594	2077.0
weighted avg	0.745994	0.748404	0.747594	2077.0

The result of Model Number:Dataset Number:Featuregroup Number:Rescale of 83 : 1 : 8 : 0

	f1-score	precision	recall	support
AFIB	0.671752	0.680836	0.663189	204.0
GSVT	0.697268	0.696136	0.698428	300.0
SB	0.712410	0.711461	0.713368	892.0
SR	0.705128	0.704225	0.706059	681.0
macro avg	0.696640	0.698165	0.695261	2077.0
micro avg	0.703885	0.703885	0.703885	2077.0
weighted avg	0.703842	0.703867	0.703885	2077.0

The result of Model Number:Dataset Number:Featuregroup Number:Rescale of 83 : 1 : 8 : 1

	f1-score	precision	recall	support
AFIB	0.877610	0.883523	0.871906	875.0
GSVT	0.893273	0.880865	0.906198	691.0
SB	0.951879	0.945361	0.958496	1553.0
SR	0.913305	0.929081	0.898105	907.0
macro avg	0.909017	0.909707	0.908676	4026.0
micro avg	0.917095	0.917095	0.917095	4026.0
weighted avg	0.916989	0.917184	0.917095	4026.0

The result of Model Number:Dataset Number:Featuregroup Number:Rescale of 83 : 1 : 9 : 0

	f1-score	precision	recall	support
AFIB	0.725274	0.724407	0.726267	875.0
GSVT	0.739412	0.741956	0.736962	691.0
SB	0.783148	0.778323	0.788039	1553.0
SR	0.767211	0.774704	0.759946	907.0
macro avg	0.753761	0.754847	0.752803	4026.0
micro avg	0.759518	0.759518	0.759518	4026.0
weighted avg	0.759473	0.759548	0.759518	4026.0

The result of Model Number:Dataset Number:Featuregroup Number:Rescale of 83 : 1 : 9 : 1

	f1-score	precision	recall	support
AFIB	0.671670	0.693975	0.651009	204.0
GSVT	0.697160	0.691567	0.702949	300.0
SB	0.712563	0.709635	0.715517	892.0
SR	0.705908	0.705805	0.706012	681.0
macro avg	0.696825	0.700246	0.693872	2077.0
micro avg	0.704250	0.704250	0.704250	2077.0
weighted avg	0.704140	0.704232	0.704250	2077.0

The result of Model Number:Dataset Number:Featuregroup Number:Rescale of 83 : 1 : 10 : 0

	f1-score	precision	recall	support
AFIB	0.900199	0.911252	0.889551	204.0
GSVT	0.918330	0.914408	0.922308	300.0
SB	0.929447	0.926657	0.932258	892.0

SR 0.921624 0.923817 0.919470 681.0
macro avg 0.917400 0.919034 0.915897 2077.0
micro avg 0.922433 0.922433 0.922433 2077.0
weighted avg 0.922403 0.922444 0.922433 2077.0
The result of Model Number:Dataset Number:Featuregroup Number:Rescale of 83 : 1 : 10 : 1

	f1-score	precision	recall	support
AFIB	0.538868	0.587013	0.569057	204.0
GSVT	0.731933	0.719968	0.744554	300.0
SB	0.623101	0.618355	0.627966	892.0
SR	0.761950	0.753329	0.770794	681.0
macro avg	0.663963	0.669666	0.678093	2077.0
micro avg	0.685850	0.685850	0.685850	2077.0
weighted avg	0.676073	0.674208	0.685850	2077.0

The result of Model Number:Dataset Number:Featuregroup Number:Rescale of 84 : 0 : 0 : 0

	f1-score	precision	recall	support
AFIB	0.773949	0.789077	0.759525	204.0
GSVT	0.795215	0.789486	0.801056	300.0
SB	0.809447	0.806298	0.812624	892.0
SR	0.800600	0.802865	0.798364	681.0
macro avg	0.794803	0.796932	0.792892	2077.0
micro avg	0.801062	0.801062	0.801062	2077.0
weighted avg	0.801004	0.801053	0.801062	2077.0

The result of Model Number:Dataset Number:Featuregroup Number:Rescale of 84 : 0 : 0 : 1

	f1-score	precision	recall	support
AFIB	0.622514	0.688337	0.568504	204.0
GSVT	0.716283	0.707994	0.724906	300.0
SB	0.757311	0.754197	0.760452	892.0
SR	0.741047	0.730071	0.752367	681.0
macro avg	0.709289	0.720150	0.701557	2077.0
micro avg	0.733814	0.733814	0.733814	2077.0
weighted avg	0.732813	0.733144	0.733814	2077.0

The result of Model Number:Dataset Number:Featuregroup Number:Rescale of 84 : 0 : 1 : 0

	f1-score	precision	recall	support
AFIB	0.818880	0.843898	0.795394	875.0
GSVT	0.869007	0.866064	0.872047	691.0
SB	0.949602	0.945788	0.953453	1553.0
SR	0.899380	0.883780	0.915628	907.0
macro avg	0.884217	0.884882	0.884130	4026.0
micro avg	0.896607	0.896607	0.896607	4026.0
weighted avg	0.896044	0.895991	0.896607	4026.0

The result of Model Number:Dataset Number:Featuregroup Number:Rescale of 84 : 0 : 1 : 1

	f1-score	precision	recall	support
AFIB	0.811104	0.827950	0.795116	875.0
GSVT	0.790987	0.763515	0.855555	691.0
SB	0.932302	0.928098	0.936560	1553.0
SR	0.708780	0.697337	0.720669	907.0
macro avg	0.810793	0.804225	0.826975	4026.0
micro avg	0.843279	0.843279	0.843279	4026.0
weighted avg	0.831350	0.826097	0.843279	4026.0

The result of Model Number:Dataset Number:Featuregroup Number:Rescale of 84 : 0 : 2 : 0

	f1-score	precision	recall	support
AFIB	0.760174	0.750082	0.770600	875.0
GSVT	0.775603	0.782956	0.768421	691.0
SB	0.825353	0.823401	0.827318	1553.0
SR	0.802962	0.811405	0.794726	907.0
macro avg	0.791023	0.791961	0.790266	4026.0
micro avg	0.797540	0.797540	0.797540	4026.0
weighted avg	0.797604	0.797822	0.797540	4026.0

The result of Model Number:Dataset Number:Featuregroup Number:Rescale of 84 : 0 : 2 : 1

	f1-score	precision	recall	support
AFIB	0.717001	0.717129	0.716904	875.0
GSVT	0.724587	0.727034	0.722201	691.0
SB	0.761141	0.758517	0.763787	1553.0
SR	0.746687	0.749206	0.744213	907.0
macro avg	0.737354	0.737971	0.736776	4026.0
micro avg	0.742050	0.742050	0.742050	4026.0
weighted avg	0.742017	0.742020	0.742050	4026.0

The result of Model Number:Dataset Number:Featuregroup Number:Rescale of 84 : 0 : 3 : 0

	f1-score	precision	recall	support
AFIB	0.744662	0.737761	0.751736	875.0
GSVT	0.758036	0.762152	0.753976	691.0
SB	0.803182	0.801330	0.805043	1553.0
SR	0.784468	0.791708	0.777385	907.0
macro avg	0.772587	0.773238	0.772035	4026.0
micro avg	0.778461	0.778461	0.778461	4026.0
weighted avg	0.778499	0.778622	0.778461	4026.0

The result of Model Number:Dataset Number:Featuregroup Number:Rescale of 84 : 0 : 3 : 1

	f1-score	precision	recall	support
AFIB	0.711827	0.735466	0.689931	204.0

GSVT 0.738841 0.732914 0.744976 300.0
SB 0.755165 0.752062 0.758296 892.0
SR 0.748112 0.748002 0.748223 681.0
macro avg 0.738486 0.742111 0.735356 2077.0
micro avg 0.746355 0.746355 0.746355 2077.0
weighted avg 0.746238 0.746335 0.746355 2077.0
The result of Model Number:Dataset Number:Featuregroup Number:Rescale of 84 : 0 : 4 : 0
f1-score precision recall support
AFIB 0.848835 0.837700 0.860308 875.0
GSVT 0.861830 0.871410 0.852474 691.0
SB 0.914082 0.911925 0.916255 1553.0
SR 0.891196 0.899217 0.883330 907.0
macro avg 0.878986 0.880063 0.878092 4026.0
micro avg 0.885731 0.885731 0.885731 4026.0
weighted avg 0.885777 0.885976 0.885731 4026.0
The result of Model Number:Dataset Number:Featuregroup Number:Rescale of 84 : 0 : 4 : 1
f1-score precision recall support
AFIB 0.845089 0.881690 0.811646 204.0
GSVT 0.790012 0.797384 0.783171 300.0
SB 0.818904 0.778643 0.863614 892.0
SR 0.729866 0.775926 0.689203 681.0
macro avg 0.795968 0.808411 0.786909 2077.0
micro avg 0.789705 0.789705 0.789705 2077.0
weighted avg 0.788109 0.790580 0.789705 2077.0
The result of Model Number:Dataset Number:Featuregroup Number:Rescale of 84 : 0 : 5 : 0
f1-score precision recall support
AFIB 0.819774 0.821954 0.817655 875.0
GSVT 0.835538 0.829652 0.841557 691.0
SB 0.883262 0.874256 0.892473 1553.0
SR 0.842887 0.861246 0.825324 907.0
macro avg 0.845365 0.846777 0.844252 4026.0
micro avg 0.852345 0.852345 0.852345 4026.0
weighted avg 0.852176 0.852302 0.852345 4026.0
The result of Model Number:Dataset Number:Featuregroup Number:Rescale of 84 : 0 : 5 : 1
f1-score precision recall support
AFIB 0.721934 0.733003 0.711312 204.0
GSVT 0.739052 0.732781 0.745461 300.0
SB 0.751908 0.749481 0.754352 892.0
SR 0.744181 0.746944 0.741448 681.0
macro avg 0.739269 0.740552 0.738144 2077.0

micro avg 0.744610 0.744610 0.744610 2077.0
weighted avg 0.744573 0.744619 0.744610 2077.0
The result of Model Number:Dataset Number:Featuregroup Number:Rescale of 84 : 0 : 6 : 0

	f1-score	precision	recall	support
AFIB	0.906105	0.940609	0.874240	204.0
GSVT	0.934974	0.925550	0.944632	300.0
SB	0.953688	0.948940	0.958497	892.0
SR	0.942168	0.942761	0.941607	681.0
macro avg	0.934233	0.939465	0.929744	2077.0
micro avg	0.942681	0.942681	0.942681	2077.0
weighted avg	0.942534	0.942717	0.942681	2077.0

The result of Model Number:Dataset Number:Featuregroup Number:Rescale of 84 : 0 : 6 : 1

	f1-score	precision	recall	support
AFIB	0.669017	0.684146	0.654563	875.0
GSVT	0.709310	0.712329	0.706358	691.0
SB	0.770163	0.766534	0.773832	1553.0
SR	0.729719	0.718526	0.741297	907.0
macro avg	0.719552	0.720383	0.719013	4026.0
micro avg	0.729000	0.729000	0.729000	4026.0
weighted avg	0.728624	0.728509	0.729000	4026.0

The result of Model Number:Dataset Number:Featuregroup Number:Rescale of 84 : 0 : 7 : 0

	f1-score	precision	recall	support
AFIB	0.735872	0.741635	0.730236	875.0
GSVT	0.737812	0.728396	0.747521	691.0
SB	0.773882	0.770778	0.777019	1553.0
SR	0.757532	0.764968	0.750267	907.0
macro avg	0.751274	0.751444	0.751261	4026.0
micro avg	0.755762	0.755762	0.755762	4026.0
weighted avg	0.755747	0.755861	0.755762	4026.0

The result of Model Number:Dataset Number:Featuregroup Number:Rescale of 84 : 0 : 7 : 1

	f1-score	precision	recall	support
AFIB	0.777343	0.789262	0.765907	204.0
GSVT	0.795775	0.789023	0.802677	300.0
SB	0.809618	0.807005	0.812250	892.0
SR	0.801299	0.804273	0.798356	681.0
macro avg	0.796009	0.797391	0.794797	2077.0
micro avg	0.801760	0.801760	0.801760	2077.0
weighted avg	0.801721	0.801769	0.801760	2077.0

The result of Model Number:Dataset Number:Featuregroup Number:Rescale of 84 : 0 : 8 : 0

	f1-score	precision	recall	support
AFIB	0.716359	0.772192	0.668388	204.0
GSVT	0.738204	0.750701	0.726149	300.0
SB	0.782358	0.775157	0.789717	892.0
SR	0.751219	0.740325	0.762473	681.0
macro avg	0.747035	0.759594	0.736682	2077.0
micro avg	0.759686	0.759686	0.759686	2077.0
weighted avg	0.759288	0.759913	0.759686	2077.0

The result of Model Number:Dataset Number:Featuregroup Number:Rescale of 84 : 0 : 8 :
1

	f1-score	precision	recall	support
AFIB	0.910547	0.910492	0.910659	875.0
GSVT	0.912518	0.907585	0.917521	691.0
SB	0.956113	0.949751	0.962573	1553.0
SR	0.930724	0.945995	0.915974	907.0
macro avg	0.927476	0.928456	0.926682	4026.0
micro avg	0.933059	0.933059	0.933059	4026.0
weighted avg	0.933008	0.933135	0.933059	4026.0

The result of Model Number:Dataset Number:Featuregroup Number:Rescale of 84 : 0 : 9 :
0

	f1-score	precision	recall	support
AFIB	0.783864	0.793932	0.774087	875.0
GSVT	0.792536	0.786127	0.799177	691.0
SB	0.847787	0.845238	0.850353	1553.0
SR	0.826794	0.826446	0.827152	907.0
macro avg	0.812745	0.812936	0.812693	4026.0
micro avg	0.819767	0.819767	0.819767	4026.0
weighted avg	0.819682	0.819708	0.819767	4026.0

The result of Model Number:Dataset Number:Featuregroup Number:Rescale of 84 : 0 : 9 :
1

	f1-score	precision	recall	support
AFIB	0.682197	0.714950	0.652450	204.0
GSVT	0.711870	0.705825	0.718039	300.0
SB	0.723057	0.717791	0.728405	892.0
SR	0.715451	0.715783	0.715141	681.0
macro avg	0.708144	0.713587	0.703509	2077.0
micro avg	0.715099	0.715099	0.715099	2077.0
weighted avg	0.714934	0.715125	0.715099	2077.0

The result of Model Number:Dataset Number:Featuregroup Number:Rescale of 84 : 0 :
10 : 0

	f1-score	precision	recall	support
AFIB	0.846699	0.863758	0.830844	204.0
GSVT	0.873867	0.866849	0.881169	300.0
SB	0.892786	0.891887	0.893687	892.0

SR 0.884648 0.884140 0.885166 681.0
macro avg 0.874500 0.876659 0.872717 2077.0
micro avg 0.882913 0.882913 0.882913 2077.0
weighted avg 0.882858 0.882968 0.882913 2077.0
The result of Model Number:Dataset Number:Featuregroup Number:Rescale of 84 : 0 : 10 : 1

	f1-score	precision	recall	support
AFIB	0.763124	0.751718	0.774910	875.0
GSVT	0.777756	0.784407	0.771228	691.0
SB	0.831014	0.828153	0.833901	1553.0
SR	0.806691	0.818722	0.795035	907.0
macro avg	0.794646	0.795750	0.793769	4026.0
micro avg	0.801567	0.801567	0.801567	4026.0
weighted avg	0.801638	0.801908	0.801567	4026.0

The result of Model Number:Dataset Number:Featuregroup Number:Rescale of 84 : 1 : 0 : 0

	f1-score	precision	recall	support
AFIB	0.769646	0.793160	0.747572	875.0
GSVT	0.816760	0.813993	0.819617	691.0
SB	0.892509	0.888924	0.896128	1553.0
SR	0.845306	0.830644	0.860578	907.0
macro avg	0.831055	0.831680	0.830974	4026.0
micro avg	0.842701	0.842701	0.842701	4026.0
weighted avg	0.842171	0.842121	0.842701	4026.0

The result of Model Number:Dataset Number:Featuregroup Number:Rescale of 84 : 1 : 0 : 1

	f1-score	precision	recall	support
AFIB	0.770040	0.781399	0.759090	204.0
GSVT	0.788943	0.785846	0.792090	300.0
SB	0.806465	0.804848	0.808092	892.0
SR	0.800745	0.800865	0.800631	681.0
macro avg	0.791548	0.793240	0.789976	2077.0
micro avg	0.798521	0.798521	0.798521	2077.0
weighted avg	0.798481	0.798494	0.798521	2077.0

The result of Model Number:Dataset Number:Featuregroup Number:Rescale of 84 : 1 : 1 : 0

	f1-score	precision	recall	support
AFIB	0.680702	0.685873	0.675668	204.0
GSVT	0.694638	0.687854	0.701573	300.0
SB	0.710842	0.709024	0.712674	892.0
SR	0.703301	0.707274	0.699381	681.0
macro avg	0.697371	0.697506	0.697324	2077.0
micro avg	0.703078	0.703078	0.703078	2077.0
weighted avg	0.703069	0.703119	0.703078	2077.0

The result of Model Number:Dataset Number:Featuregroup Number:Rescale of 84 : 1 : 1 : 1

	f1-score	precision	recall	support
AFIB	0.710812	0.711317	0.710321	875.0
GSVT	0.720390	0.721811	0.719037	691.0
SB	0.765175	0.762632	0.767738	1553.0
SR	0.747196	0.749973	0.744463	907.0
macro avg	0.735893	0.736433	0.735390	4026.0
micro avg	0.741657	0.741657	0.741657	4026.0
weighted avg	0.741623	0.741621	0.741657	4026.0

The result of Model Number:Dataset Number:Featuregroup Number:Rescale of 84 : 1 : 2 : 0

	f1-score	precision	recall	support
AFIB	0.757408	0.745529	0.769754	875.0
GSVT	0.729812	0.750735	0.710109	691.0
SB	0.766912	0.728036	0.810217	1553.0
SR	0.623111	0.693396	0.565861	907.0
macro avg	0.719311	0.729424	0.713985	4026.0
micro avg	0.729191	0.729191	0.729191	4026.0
weighted avg	0.726082	0.727930	0.729191	4026.0

The result of Model Number:Dataset Number:Featuregroup Number:Rescale of 84 : 1 : 2 : 1

	f1-score	precision	recall	support
AFIB	0.795497	0.812971	0.779056	204.0
GSVT	0.821134	0.814498	0.827993	300.0
SB	0.838344	0.837313	0.839378	892.0
SR	0.831266	0.830543	0.831996	681.0
macro avg	0.821560	0.823831	0.819606	2077.0
micro avg	0.829388	0.829388	0.829388	2077.0
weighted avg	0.829329	0.829407	0.829388	2077.0

The result of Model Number:Dataset Number:Featuregroup Number:Rescale of 84 : 1 : 3 : 0

	f1-score	precision	recall	support
AFIB	0.625075	0.608654	0.664206	875.0
GSVT	0.601316	0.569153	0.685488	691.0
SB	0.569778	0.566177	0.573442	1553.0
SR	0.553449	0.562985	0.544386	907.0
macro avg	0.587405	0.576742	0.616880	4026.0
micro avg	0.605853	0.605853	0.605853	4026.0
weighted avg	0.583531	0.575201	0.605853	4026.0

The result of Model Number:Dataset Number:Featuregroup Number:Rescale of 84 : 1 : 3 : 1

	f1-score	precision	recall	support
AFIB	0.595054	0.599982	0.670247	204.0

GSVT 0.645296 0.617676 0.728045 300.0
SB 0.589179 0.587132 0.591243 892.0
SR 0.583321 0.583431 0.583215 681.0
macro avg 0.603212 0.597055 0.643187 2077.0
micro avg 0.616130 0.616130 0.616130 2077.0
weighted avg 0.595941 0.591592 0.616130 2077.0
The result of Model Number:Dataset Number:Featuregroup Number:Rescale of 84 : 1 : 4 : 0

	f1-score	precision	recall	support
AFIB	0.689613	0.690995	0.688266	875.0
GSVT	0.691321	0.687589	0.695112	691.0
SB	0.722663	0.718802	0.726570	1553.0
SR	0.705374	0.713739	0.697223	907.0
macro avg	0.702243	0.702781	0.701793	4026.0
micro avg	0.706234	0.706234	0.706234	4026.0
weighted avg	0.706206	0.706261	0.706234	4026.0

The result of Model Number:Dataset Number:Featuregroup Number:Rescale of 84 : 1 : 4 : 1

	f1-score	precision	recall	support
AFIB	0.880169	0.918797	0.844880	204.0
GSVT	0.819380	0.825279	0.813901	300.0
SB	0.849762	0.807089	0.897202	892.0
SR	0.753872	0.803646	0.710063	681.0
macro avg	0.825796	0.838703	0.816511	2077.0
micro avg	0.818672	0.818672	0.818672	2077.0
weighted avg	0.816920	0.819560	0.818672	2077.0

The result of Model Number:Dataset Number:Featuregroup Number:Rescale of 84 : 1 : 5 : 0

	f1-score	precision	recall	support
AFIB	0.744804	0.738144	0.751617	875.0
GSVT	0.756762	0.759756	0.753819	691.0
SB	0.806870	0.805373	0.808376	1553.0
SR	0.786424	0.793694	0.779315	907.0
macro avg	0.773715	0.774242	0.773282	4026.0
micro avg	0.780129	0.780129	0.780129	4026.0
weighted avg	0.780174	0.780301	0.780129	4026.0

The result of Model Number:Dataset Number:Featuregroup Number:Rescale of 84 : 1 : 5 : 1

	f1-score	precision	recall	support
AFIB	0.780501	0.793477	0.768122	204.0
GSVT	0.795468	0.789816	0.801260	300.0
SB	0.813918	0.809344	0.818563	892.0
SR	0.803549	0.808465	0.798718	681.0
macro avg	0.798359	0.800276	0.796666	2077.0

micro avg 0.804603 0.804603 0.804603 2077.0
weighted avg 0.804571 0.804677 0.804603 2077.0
The result of Model Number:Dataset Number:Featuregroup Number:Rescale of 84 : 1 : 6 : 0

	f1-score	precision	recall	support
AFIB	0.702540	0.778243	0.640823	204.0
GSVT	0.828111	0.816503	0.840267	300.0
SB	0.879253	0.876422	0.882112	892.0
SR	0.856742	0.843711	0.870204	681.0
macro avg	0.816661	0.828720	0.808351	2077.0
micro avg	0.848464	0.848464	0.848464	2077.0
weighted avg	0.847129	0.847399	0.848464	2077.0

The result of Model Number:Dataset Number:Featuregroup Number:Rescale of 84 : 1 : 6 : 1

	f1-score	precision	recall	support
AFIB	0.686380	0.707350	0.666694	875.0
GSVT	0.728397	0.725930	0.730944	691.0
SB	0.795951	0.792754	0.799179	1553.0
SR	0.753855	0.740779	0.767474	907.0
macro avg	0.741146	0.741703	0.741073	4026.0
micro avg	0.751531	0.751531	0.751531	4026.0
weighted avg	0.751059	0.751014	0.751531	4026.0

The result of Model Number:Dataset Number:Featuregroup Number:Rescale of 84 : 1 : 7 : 0

	f1-score	precision	recall	support
AFIB	0.803330	0.772618	0.853862	875.0
GSVT	0.832385	0.803658	0.871351	691.0
SB	0.927432	0.911061	0.945420	1553.0
SR	0.785514	0.944535	0.678185	907.0
macro avg	0.837165	0.857968	0.837205	4026.0
micro avg	0.852604	0.852604	0.852604	4026.0
weighted avg	0.852174	0.870080	0.852604	4026.0

The result of Model Number:Dataset Number:Featuregroup Number:Rescale of 84 : 1 : 7 : 1

	f1-score	precision	recall	support
AFIB	0.815129	0.804213	0.826398	875.0
GSVT	0.833354	0.839859	0.826964	691.0
SB	0.884790	0.882022	0.887580	1553.0
SR	0.863393	0.874839	0.852258	907.0
macro avg	0.849167	0.850233	0.848300	4026.0
micro avg	0.855922	0.855922	0.855922	4026.0
weighted avg	0.856001	0.856256	0.855922	4026.0

The result of Model Number:Dataset Number:Featuregroup Number:Rescale of 84 : 1 : 8 : 0

	f1-score	precision	recall	support
AFIB	0.816129	0.818634	0.813768	204.0
GSVT	0.837568	0.835440	0.839782	300.0
SB	0.856278	0.855232	0.857332	892.0
SR	0.849270	0.850903	0.847650	681.0
macro avg	0.839811	0.840052	0.839633	2077.0
micro avg	0.847344	0.847344	0.847344	2077.0
weighted avg	0.847334	0.847359	0.847344	2077.0

The result of Model Number:Dataset Number:Featuregroup Number:Rescale of 84 : 1 : 8 : 1

	f1-score	precision	recall	support
AFIB	0.736009	0.744187	0.728386	204.0
GSVT	0.758807	0.754488	0.763397	300.0
SB	0.773903	0.772866	0.774944	892.0
SR	0.767319	0.768347	0.766304	681.0
macro avg	0.759010	0.759972	0.758258	2077.0
micro avg	0.765870	0.765870	0.765870	2077.0
weighted avg	0.765842	0.765913	0.765870	2077.0

The result of Model Number:Dataset Number:Featuregroup Number:Rescale of 84 : 1 : 9 : 0

	f1-score	precision	recall	support
AFIB	0.911996	0.920880	0.903505	204.0
GSVT	0.933086	0.928802	0.937442	300.0
SB	0.945981	0.942726	0.949265	892.0
SR	0.937992	0.941631	0.934425	681.0
macro avg	0.932264	0.933510	0.931159	2077.0
micro avg	0.938197	0.938197	0.938197	2077.0
weighted avg	0.938161	0.938210	0.938197	2077.0

The result of Model Number:Dataset Number:Featuregroup Number:Rescale of 84 : 1 : 9 : 1

	f1-score	precision	recall	support
AFIB	0.796211	0.801784	0.790745	204.0
GSVT	0.814736	0.810719	0.818805	300.0
SB	0.830929	0.829546	0.832327	892.0
SR	0.822659	0.824612	0.820725	681.0
macro avg	0.816134	0.816665	0.815651	2077.0
micro avg	0.822486	0.822486	0.822486	2077.0
weighted avg	0.822469	0.822482	0.822486	2077.0

The result of Model Number:Dataset Number:Featuregroup Number:Rescale of 84 : 1 : 10 : 0

	f1-score	precision	recall	support
AFIB	0.717934	0.734184	0.702479	204.0
GSVT	0.737039	0.733430	0.740716	300.0
SB	0.752117	0.749945	0.754307	892.0

SR 0.747285 0.746962 0.747619 681.0
macro avg 0.738594 0.741130 0.736280 2077.0
micro avg 0.745061 0.745061 0.745061 2077.0
weighted avg 0.744997 0.745033 0.745061 2077.0
The result of Model Number:Dataset Number:Featuregroup Number:Rescale of 84 : 1 : 10 : 1

	f1-score	precision	recall	support
AFIB	0.872329	0.914210	0.834292	204.0
GSVT	0.910272	0.902542	0.918160	300.0
SB	0.924577	0.917844	0.931415	892.0
SR	0.914851	0.915275	0.914455	681.0
macro avg	0.905507	0.912468	0.899580	2077.0
micro avg	0.914400	0.914400	0.914400	2077.0
weighted avg	0.914190	0.914435	0.914400	2077.0

The result of Model Number:Dataset Number:Featuregroup Number:Rescale of 85 : 0 : 0 : 0

	f1-score	precision	recall	support
AFIB	0.777125	0.796033	0.759286	204.0
GSVT	0.786894	0.776061	0.798138	300.0
SB	0.802783	0.794766	0.810969	892.0
SR	0.788767	0.799118	0.778703	681.0
macro avg	0.788892	0.791495	0.786774	2077.0
micro avg	0.793460	0.793460	0.793460	2077.0
weighted avg	0.793372	0.793616	0.793460	2077.0

The result of Model Number:Dataset Number:Featuregroup Number:Rescale of 85 : 0 : 0 : 1

	f1-score	precision	recall	support
AFIB	0.713421	0.750984	0.679628	204.0
GSVT	0.749246	0.742683	0.755959	300.0
SB	0.768687	0.763942	0.773506	892.0
SR	0.758200	0.756662	0.759762	681.0
macro avg	0.747388	0.753568	0.742213	2077.0
micro avg	0.757244	0.757244	0.757244	2077.0
weighted avg	0.757012	0.757212	0.757244	2077.0

The result of Model Number:Dataset Number:Featuregroup Number:Rescale of 85 : 0 : 1 : 0

	f1-score	precision	recall	support
AFIB	0.744277	0.755255	0.733693	204.0
GSVT	0.762548	0.759555	0.765589	300.0
SB	0.779483	0.777920	0.781056	892.0
SR	0.773954	0.774071	0.773845	681.0
macro avg	0.765065	0.766700	0.763546	2077.0
micro avg	0.771806	0.771806	0.771806	2077.0
weighted avg	0.771766	0.771779	0.771806	2077.0

The result of Model Number:Dataset Number:Featuregroup Number:Rescale of 85 : 0 : 1 : 1

	f1-score	precision	recall	support
AFIB	0.723198	0.728691	0.717849	204.0
GSVT	0.738004	0.730796	0.745372	300.0
SB	0.755219	0.753288	0.757166	892.0
SR	0.747208	0.751429	0.743043	681.0
macro avg	0.740907	0.741051	0.740858	2077.0
micro avg	0.746970	0.746970	0.746970	2077.0
weighted avg	0.746961	0.747014	0.746970	2077.0

The result of Model Number:Dataset Number:Featuregroup Number:Rescale of 85 : 0 : 2 : 0

	f1-score	precision	recall	support
AFIB	0.704214	0.702178	0.706326	875.0
GSVT	0.717266	0.716682	0.717900	691.0
SB	0.761840	0.758685	0.765028	1553.0
SR	0.738969	0.747087	0.731065	907.0
macro avg	0.730572	0.731158	0.730080	4026.0
micro avg	0.736530	0.736530	0.736530	4026.0
weighted avg	0.736513	0.736582	0.736530	4026.0

The result of Model Number:Dataset Number:Featuregroup Number:Rescale of 85 : 0 : 2 : 1

	f1-score	precision	recall	support
AFIB	0.784607	0.822172	0.750510	204.0
GSVT	0.826403	0.817556	0.835473	300.0
SB	0.843956	0.839294	0.848681	892.0
SR	0.834422	0.833699	0.835161	681.0
macro avg	0.822347	0.828180	0.817456	2077.0
micro avg	0.832698	0.832698	0.832698	2077.0
weighted avg	0.832466	0.832638	0.832698	2077.0

The result of Model Number:Dataset Number:Featuregroup Number:Rescale of 85 : 0 : 3 : 0

	f1-score	precision	recall	support
AFIB	0.791486	0.799142	0.784118	204.0
GSVT	0.819493	0.817877	0.821128	300.0
SB	0.836622	0.835140	0.838120	892.0
SR	0.828909	0.829310	0.828543	681.0
macro avg	0.819127	0.820367	0.817977	2077.0
micro avg	0.827222	0.827222	0.827222	2077.0
weighted avg	0.827186	0.827199	0.827222	2077.0

The result of Model Number:Dataset Number:Featuregroup Number:Rescale of 85 : 0 : 3 : 1

	f1-score	precision	recall	support
AFIB	0.747203	0.779565	0.717634	204.0

GSVT	0.698506	0.705024	0.692457	300.0
SB	0.724052	0.688454	0.763583	892.0
SR	0.645326	0.686051	0.609373	681.0
macro avg	0.703772	0.714774	0.695762	2077.0
micro avg	0.698235	0.698235	0.698235	2077.0
weighted avg	0.696823	0.699008	0.698235	2077.0

The result of Model Number:Dataset Number:Featuregroup Number:Rescale of 85 : 0 : 4 : 0

	f1-score	precision	recall	support
AFIB	0.820861	0.847526	0.795978	204.0
GSVT	0.836422	0.828935	0.844212	300.0
SB	0.856311	0.850442	0.862263	892.0
SR	0.842346	0.845900	0.838871	681.0
macro avg	0.838985	0.843201	0.835331	2077.0
micro avg	0.845476	0.845476	0.845476	2077.0
weighted avg	0.845378	0.845560	0.845476	2077.0

The result of Model Number:Dataset Number:Featuregroup Number:Rescale of 85 : 0 : 4 : 1

	f1-score	precision	recall	support
AFIB	0.741904	0.761956	0.723034	204.0
GSVT	0.748019	0.737007	0.759467	300.0
SB	0.762508	0.754396	0.770799	892.0
SR	0.748366	0.758648	0.738370	681.0
macro avg	0.750199	0.753002	0.747917	2077.0
micro avg	0.753838	0.753838	0.753838	2077.0
weighted avg	0.753755	0.754021	0.753838	2077.0

The result of Model Number:Dataset Number:Featuregroup Number:Rescale of 85 : 0 : 5 : 0

	f1-score	precision	recall	support
AFIB	0.832209	0.901681	0.774335	204.0
GSVT	0.892640	0.881416	0.904518	300.0
SB	0.915428	0.902604	0.928729	892.0
SR	0.890457	0.894629	0.886758	681.0
macro avg	0.882684	0.895082	0.873585	2077.0
micro avg	0.896307	0.896307	0.896307	2077.0
weighted avg	0.895776	0.896838	0.896307	2077.0

The result of Model Number:Dataset Number:Featuregroup Number:Rescale of 85 : 0 : 5 : 1

	f1-score	precision	recall	support
AFIB	0.886126	0.896262	0.876561	204.0
GSVT	0.906868	0.901501	0.912313	300.0
SB	0.870576	0.838419	0.924858	892.0
SR	0.730276	0.733810	0.726794	681.0
macro avg	0.848462	0.842498	0.860132	2077.0

micro avg 0.853362 0.853362 0.853362 2077.0
weighted avg 0.831344 0.818913 0.853362 2077.0
The result of Model Number:Dataset Number:Featuregroup Number:Rescale of 85 : 0 : 6 : 0

	f1-score	precision	recall	support
AFIB	0.832081	0.879416	0.790134	204.0
GSVT	0.879322	0.869639	0.889267	300.0
SB	0.892402	0.883523	0.901492	892.0
SR	0.882035	0.885051	0.879180	681.0
macro avg	0.871460	0.879407	0.865018	2077.0
micro avg	0.881473	0.881473	0.881473	2077.0
weighted avg	0.881189	0.881615	0.881473	2077.0

The result of Model Number:Dataset Number:Featuregroup Number:Rescale of 85 : 0 : 6 : 1

	f1-score	precision	recall	support
AFIB	0.770097	0.802667	0.740323	204.0
GSVT	0.709204	0.710445	0.718605	300.0
SB	0.763523	0.727150	0.804468	892.0
SR	0.674992	0.729358	0.629390	681.0
macro avg	0.729454	0.742405	0.723197	2077.0
micro avg	0.728362	0.728362	0.728362	2077.0
weighted avg	0.727296	0.732878	0.728362	2077.0

The result of Model Number:Dataset Number:Featuregroup Number:Rescale of 85 : 0 : 7 : 0

	f1-score	precision	recall	support
AFIB	0.848083	0.862808	0.833960	204.0
GSVT	0.864795	0.862236	0.867388	300.0
SB	0.874993	0.871880	0.878131	892.0
SR	0.867879	0.868785	0.866994	681.0
macro avg	0.863937	0.866427	0.861618	2077.0
micro avg	0.868589	0.868589	0.868589	2077.0
weighted avg	0.868544	0.868581	0.868589	2077.0

The result of Model Number:Dataset Number:Featuregroup Number:Rescale of 85 : 0 : 7 : 1

	f1-score	precision	recall	support
AFIB	0.708998	0.725155	0.693659	875.0
GSVT	0.754464	0.755956	0.753231	691.0
SB	0.825434	0.820917	0.830005	1553.0
SR	0.780160	0.770696	0.789967	907.0
macro avg	0.767264	0.768181	0.766715	4026.0
micro avg	0.778175	0.778175	0.778175	4026.0
weighted avg	0.777748	0.777641	0.778175	4026.0

The result of Model Number:Dataset Number:Featuregroup Number:Rescale of 85 : 0 : 8 : 0

	f1-score	precision	recall	support
AFIB	0.833314	0.848607	0.818963	204.0
GSVT	0.864948	0.857993	0.872174	300.0
SB	0.880993	0.879420	0.882573	892.0
SR	0.872270	0.873046	0.871499	681.0
macro avg	0.862881	0.864767	0.861302	2077.0
micro avg	0.871192	0.871192	0.871192	2077.0
weighted avg	0.871132	0.871209	0.871192	2077.0

The result of Model Number:Dataset Number:Featuregroup Number:Rescale of 85 : 0 : 8 : 1

	f1-score	precision	recall	support
AFIB	0.751143	0.756760	0.745638	204.0
GSVT	0.770336	0.766271	0.774446	300.0
SB	0.783337	0.782289	0.784389	892.0
SR	0.776437	0.777926	0.774956	681.0
macro avg	0.770313	0.770812	0.769857	2077.0
micro avg	0.776054	0.776054	0.776054	2077.0
weighted avg	0.776035	0.776038	0.776054	2077.0

The result of Model Number:Dataset Number:Featuregroup Number:Rescale of 85 : 0 : 9 : 0

	f1-score	precision	recall	support
AFIB	0.726197	0.719704	0.732840	875.0
GSVT	0.737857	0.740776	0.734987	691.0
SB	0.786713	0.785253	0.788182	1553.0
SR	0.766778	0.773866	0.759847	907.0
macro avg	0.754386	0.754900	0.753964	4026.0
micro avg	0.760641	0.760641	0.760641	4026.0
weighted avg	0.760684	0.760808	0.760641	4026.0

The result of Model Number:Dataset Number:Featuregroup Number:Rescale of 85 : 0 : 9 : 1

	f1-score	precision	recall	support
AFIB	0.658287	0.648948	0.667915	875.0
GSVT	0.671830	0.678378	0.665412	691.0
SB	0.714958	0.712628	0.717305	1553.0
SR	0.698262	0.707186	0.689567	907.0
macro avg	0.685834	0.686785	0.685050	4026.0
micro avg	0.691415	0.691415	0.691415	4026.0
weighted avg	0.691478	0.691684	0.691415	4026.0

The result of Model Number:Dataset Number:Featuregroup Number:Rescale of 85 : 0 : 10 : 0

	f1-score	precision	recall	support
AFIB	0.919147	0.937666	0.901936	204.0
GSVT	0.948640	0.941021	0.956566	300.0
SB	0.969177	0.968201	0.970155	892.0

SR 0.960343 0.959791 0.960906 681.0
macro avg 0.949327 0.951670 0.947391 2077.0
micro avg 0.958459 0.958459 0.958459 2077.0
weighted avg 0.958400 0.958519 0.958459 2077.0
The result of Model Number:Dataset Number:Featuregroup Number:Rescale of 85 : 0 : 10 : 1

	f1-score	precision	recall	support
AFIB	0.647766	0.648888	0.646657	875.0
GSVT	0.655620	0.648086	0.663384	691.0
SB	0.690390	0.682065	0.698923	1553.0
SR	0.657574	0.677498	0.638803	907.0
macro avg	0.662837	0.664134	0.661942	4026.0
micro avg	0.667920	0.667920	0.667920	4026.0
weighted avg	0.667765	0.667994	0.667920	4026.0

The result of Model Number:Dataset Number:Featuregroup Number:Rescale of 85 : 1 : 0 : 0

	f1-score	precision	recall	support
AFIB	0.668031	0.660572	0.675667	875.0
GSVT	0.681322	0.687562	0.675204	691.0
SB	0.726661	0.724294	0.729046	1553.0
SR	0.707823	0.714914	0.700874	907.0
macro avg	0.695959	0.696836	0.695198	4026.0
micro avg	0.701857	0.701857	0.701857	4026.0
weighted avg	0.701893	0.702027	0.701857	4026.0

The result of Model Number:Dataset Number:Featuregroup Number:Rescale of 85 : 1 : 0 : 1

	f1-score	precision	recall	support
AFIB	0.668452	0.667377	0.669804	875.0
GSVT	0.684038	0.680472	0.687680	691.0
SB	0.715197	0.709138	0.721366	1553.0
SR	0.687895	0.703188	0.673485	907.0
macro avg	0.688895	0.690044	0.688084	4026.0
micro avg	0.693591	0.693591	0.693591	4026.0
weighted avg	0.693539	0.693801	0.693591	4026.0

The result of Model Number:Dataset Number:Featuregroup Number:Rescale of 85 : 1 : 1 : 0

	f1-score	precision	recall	support
AFIB	0.646855	0.704648	0.683093	204.0
GSVT	0.878608	0.864246	0.893760	300.0
SB	0.747967	0.742270	0.753808	892.0
SR	0.914642	0.904293	0.925258	681.0
macro avg	0.797018	0.803864	0.813980	2077.0
micro avg	0.823291	0.823291	0.823291	2077.0
weighted avg	0.811555	0.809317	0.823291	2077.0

The result of Model Number:Dataset Number:Featuregroup Number:Rescale of 85 : 1 : 1 : 1

	f1-score	precision	recall	support
AFIB	0.556842	0.517822	0.653906	875.0
GSVT	0.477564	0.445873	0.540173	691.0
SB	0.570658	0.565844	0.575569	1553.0
SR	0.550032	0.563364	0.537508	907.0
macro avg	0.538774	0.523226	0.576789	4026.0
micro avg	0.577945	0.577945	0.577945	4026.0
weighted avg	0.547031	0.534257	0.577945	4026.0

The result of Model Number:Dataset Number:Featuregroup Number:Rescale of 85 : 1 : 2 : 0

	f1-score	precision	recall	support
AFIB	0.721060	0.727349	0.715060	204.0
GSVT	0.745997	0.744779	0.747237	300.0
SB	0.761854	0.759996	0.763730	892.0
SR	0.754869	0.756007	0.753760	681.0
macro avg	0.745945	0.747033	0.744947	2077.0
micro avg	0.753299	0.753299	0.753299	2077.0
weighted avg	0.753267	0.753283	0.753299	2077.0

The result of Model Number:Dataset Number:Featuregroup Number:Rescale of 85 : 1 : 2 : 1

	f1-score	precision	recall	support
AFIB	0.731044	0.715339	0.747493	875.0
GSVT	0.757073	0.768052	0.746453	691.0
SB	0.805934	0.805780	0.806090	1553.0
SR	0.785734	0.794867	0.776814	907.0
macro avg	0.769946	0.771009	0.769212	4026.0
micro avg	0.776523	0.776523	0.776523	4026.0
weighted avg	0.776720	0.777190	0.776523	4026.0

The result of Model Number:Dataset Number:Featuregroup Number:Rescale of 85 : 1 : 3 : 0

	f1-score	precision	recall	support
AFIB	0.854519	0.844549	0.866955	875.0
GSVT	0.879248	0.868590	0.890878	691.0
SB	0.892501	0.858050	0.943034	1553.0
SR	0.708692	0.746075	0.674919	907.0
macro avg	0.833740	0.829316	0.843946	4026.0
micro avg	0.857145	0.857145	0.857145	4026.0
weighted avg	0.840562	0.831698	0.857145	4026.0

The result of Model Number:Dataset Number:Featuregroup Number:Rescale of 85 : 1 : 3 : 1

	f1-score	precision	recall	support
AFIB	0.715549	0.713480	0.717695	875.0

GSVT 0.728811 0.728218 0.729455 691.0
SB 0.774103 0.770896 0.777342 1553.0
SR 0.750863 0.759112 0.742832 907.0
macro avg 0.742331 0.742927 0.741831 4026.0
micro avg 0.748385 0.748385 0.748385 4026.0
weighted avg 0.748367 0.748438 0.748385 4026.0

The result of Model Number:Dataset Number:Featuregroup Number:Rescale of 85 : 1 : 4 : 0

	f1-score	precision	recall	support
AFIB	0.677421	0.678975	0.675947	875.0
GSVT	0.679782	0.677866	0.681733	691.0
SB	0.713421	0.707513	0.719438	1553.0
SR	0.692019	0.702404	0.681965	907.0
macro avg	0.690661	0.691690	0.689771	4026.0
micro avg	0.695072	0.695072	0.695072	4026.0
weighted avg	0.695002	0.695071	0.695072	4026.0

The result of Model Number:Dataset Number:Featuregroup Number:Rescale of 85 : 1 : 4 : 1

	f1-score	precision	recall	support
AFIB	0.842282	0.844419	0.840185	875.0
GSVT	0.857145	0.847860	0.866671	691.0
SB	0.903384	0.893165	0.913853	1553.0
SR	0.862079	0.885681	0.839738	907.0
macro avg	0.866223	0.867781	0.865112	4026.0
micro avg	0.873047	0.873047	0.873047	4026.0
weighted avg	0.872863	0.873109	0.873047	4026.0

The result of Model Number:Dataset Number:Featuregroup Number:Rescale of 85 : 1 : 5 : 0

	f1-score	precision	recall	support
AFIB	0.709797	0.725224	0.695207	204.0
GSVT	0.739097	0.734737	0.743529	300.0
SB	0.754976	0.753301	0.756666	892.0
SR	0.747407	0.746985	0.747848	681.0
macro avg	0.737819	0.740062	0.735813	2077.0
micro avg	0.745841	0.745841	0.745841	2077.0
weighted avg	0.745763	0.745791	0.745841	2077.0

The result of Model Number:Dataset Number:Featuregroup Number:Rescale of 85 : 1 : 5 : 1

	f1-score	precision	recall	support
AFIB	0.730496	0.737562	0.723696	204.0
GSVT	0.756345	0.754854	0.757855	300.0
SB	0.772154	0.770786	0.773537	892.0
SR	0.765036	0.765406	0.764698	681.0
macro avg	0.756008	0.757152	0.754947	2077.0

micro avg 0.763479 0.763479 0.763479 2077.0
weighted avg 0.763445 0.763458 0.763479 2077.0
The result of Model Number:Dataset Number:Featuregroup Number:Rescale of 85 : 1 : 6 : 0
f1-score precision recall support
AFIB 0.813217 0.847169 0.782028 875.0
GSVT 0.878558 0.874110 0.883158 691.0
SB 0.963365 0.957944 0.968875 1553.0
SR 0.902047 0.882016 0.923023 907.0
macro avg 0.889297 0.890310 0.889271 4026.0
micro avg 0.903224 0.903224 0.903224 4026.0
weighted avg 0.902362 0.902374 0.903224 4026.0
The result of Model Number:Dataset Number:Featuregroup Number:Rescale of 85 : 1 : 6 : 1
f1-score precision recall support
AFIB 0.782184 0.789803 0.774901 204.0
GSVT 0.800272 0.796597 0.804007 300.0
SB 0.811331 0.808539 0.814148 892.0
SR 0.804479 0.807600 0.801420 681.0
macro avg 0.799566 0.800635 0.798619 2077.0
micro avg 0.804655 0.804655 0.804655 2077.0
weighted avg 0.804624 0.804666 0.804655 2077.0
The result of Model Number:Dataset Number:Featuregroup Number:Rescale of 85 : 1 : 7 : 0
f1-score precision recall support
AFIB 0.807886 0.815074 0.800867 875.0
GSVT 0.824843 0.815792 0.834151 691.0
SB 0.887114 0.880208 0.894138 1553.0
SR 0.857634 0.869791 0.845816 907.0
macro avg 0.844370 0.845216 0.843743 4026.0
micro avg 0.852685 0.852685 0.852685 4026.0
weighted avg 0.852566 0.852649 0.852685 4026.0
The result of Model Number:Dataset Number:Featuregroup Number:Rescale of 85 : 1 : 7 : 1
f1-score precision recall support
AFIB 0.688899 0.700860 0.677427 204.0
GSVT 0.702474 0.700396 0.704580 300.0
SB 0.710758 0.708229 0.713307 892.0
SR 0.704979 0.705715 0.704261 681.0
macro avg 0.701778 0.703800 0.699894 2077.0
micro avg 0.705557 0.705557 0.705557 2077.0
weighted avg 0.705520 0.705550 0.705557 2077.0
The result of Model Number:Dataset Number:Featuregroup Number:Rescale of 85 : 1 : 8 : 0

	f1-score	precision	recall	support
AFIB	0.855233	0.866217	0.844566	875.0
GSVT	0.864694	0.857701	0.871940	691.0
SB	0.924975	0.922194	0.927775	1553.0
SR	0.902071	0.901691	0.902462	907.0
macro avg	0.886743	0.886951	0.886686	4026.0
micro avg	0.894405	0.894405	0.894405	4026.0
weighted avg	0.894311	0.894340	0.894405	4026.0

The result of Model Number:Dataset Number:Featuregroup Number:Rescale of 85 : 1 : 8 : 1

	f1-score	precision	recall	support
AFIB	0.746764	0.736420	0.757432	875.0
GSVT	0.763326	0.769504	0.757251	691.0
SB	0.810262	0.807933	0.812609	1553.0
SR	0.790551	0.800840	0.780530	907.0
macro avg	0.777725	0.778674	0.776955	4026.0
micro avg	0.783888	0.783888	0.783888	4026.0
weighted avg	0.783965	0.784197	0.783888	4026.0

The result of Model Number:Dataset Number:Featuregroup Number:Rescale of 85 : 1 : 9 : 0

	f1-score	precision	recall	support
AFIB	0.738675	0.811090	0.678852	204.0
GSVT	0.866660	0.854979	0.878804	300.0
SB	0.920122	0.917053	0.923216	892.0
SR	0.896210	0.884194	0.908579	681.0
macro avg	0.855417	0.866829	0.847363	2077.0
micro avg	0.888001	0.888001	0.888001	2077.0
weighted avg	0.886738	0.886906	0.888001	2077.0

The result of Model Number:Dataset Number:Featuregroup Number:Rescale of 85 : 1 : 9 : 1

	f1-score	precision	recall	support
AFIB	0.826717	0.832621	0.821046	204.0
GSVT	0.855625	0.855074	0.856194	300.0
SB	0.872745	0.870808	0.874699	892.0
SR	0.864943	0.865990	0.863926	681.0
macro avg	0.855008	0.856123	0.853966	2077.0
micro avg	0.863224	0.863224	0.863224	2077.0
weighted avg	0.863193	0.863205	0.863224	2077.0

The result of Model Number:Dataset Number:Featuregroup Number:Rescale of 85 : 1 : 10 : 0

	f1-score	precision	recall	support
AFIB	0.729104	0.733456	0.724823	204.0
GSVT	0.747019	0.743811	0.750255	300.0
SB	0.759812	0.758712	0.760919	892.0

SR 0.752865 0.754420 0.751320 681.0
macro avg 0.747200 0.747600 0.746829 2077.0
micro avg 0.752686 0.752686 0.752686 2077.0
weighted avg 0.752670 0.752672 0.752686 2077.0
The result of Model Number:Dataset Number:Featuregroup Number:Rescale of 85 : 1 : 10 : 1

	f1-score	precision	recall	support
AFIB	0.865272	0.885039	0.846587	204.0
GSVT	0.904580	0.898949	0.910304	300.0
SB	0.924116	0.921250	0.927013	892.0
SR	0.913533	0.913955	0.913135	681.0
macro avg	0.901875	0.904798	0.899260	2077.0
micro avg	0.912150	0.912150	0.912150	2077.0
weighted avg	0.912045	0.912080	0.912150	2077.0

The result of Model Number:Dataset Number:Featuregroup Number:Rescale of 86 : 0 : 0 : 0

	f1-score	precision	recall	support
AFIB	0.777013	0.774497	0.779569	875.0
GSVT	0.786023	0.788465	0.783674	691.0
SB	0.829382	0.827577	0.831198	1553.0
SR	0.814087	0.817928	0.810313	907.0
macro avg	0.801626	0.802117	0.801189	4026.0
micro avg	0.807115	0.807115	0.807115	4026.0
weighted avg	0.807113	0.807154	0.807115	4026.0

The result of Model Number:Dataset Number:Featuregroup Number:Rescale of 86 : 0 : 0 : 1

	f1-score	precision	recall	support
AFIB	0.683633	0.673151	0.694560	875.0
GSVT	0.707206	0.714137	0.700546	691.0
SB	0.752656	0.750897	0.754446	1553.0
SR	0.733854	0.743159	0.724795	907.0
macro avg	0.719337	0.720336	0.718587	4026.0
micro avg	0.725500	0.725500	0.725500	4026.0
weighted avg	0.725618	0.725947	0.725500	4026.0

The result of Model Number:Dataset Number:Featuregroup Number:Rescale of 86 : 0 : 1 : 0

	f1-score	precision	recall	support
AFIB	0.765497	0.769519	0.761564	875.0
GSVT	0.778416	0.779861	0.777074	691.0
SB	0.824449	0.818594	0.830393	1553.0
SR	0.808641	0.813624	0.803739	907.0
macro avg	0.794251	0.795399	0.793192	4026.0
micro avg	0.800278	0.800278	0.800278	4026.0
weighted avg	0.800174	0.800160	0.800278	4026.0

The result of Model Number:Dataset Number:Featuregroup Number:Rescale of 86 : 0 : 1 : 1

	f1-score	precision	recall	support
AFIB	0.793645	0.813408	0.774858	875.0
GSVT	0.838543	0.842103	0.835048	691.0
SB	0.909552	0.906072	0.913065	1553.0
SR	0.865184	0.849062	0.881976	907.0
macro avg	0.851731	0.852661	0.851237	4026.0
micro avg	0.862633	0.862633	0.862633	4026.0
weighted avg	0.862178	0.862110	0.862633	4026.0

The result of Model Number:Dataset Number:Featuregroup Number:Rescale of 86 : 0 : 2 : 0

	f1-score	precision	recall	support
AFIB	0.671934	0.702276	0.644324	204.0
GSVT	0.695304	0.687496	0.703419	300.0
SB	0.668857	0.639696	0.715687	892.0
SR	0.557250	0.562001	0.552673	681.0
macro avg	0.648336	0.647867	0.654026	2077.0
micro avg	0.653457	0.653457	0.653457	2077.0
weighted avg	0.636386	0.627272	0.653457	2077.0

The result of Model Number:Dataset Number:Featuregroup Number:Rescale of 86 : 0 : 2 : 1

	f1-score	precision	recall	support
AFIB	0.747975	0.748174	0.747906	875.0
GSVT	0.761000	0.758190	0.763926	691.0
SB	0.814121	0.812145	0.816111	1553.0
SR	0.794705	0.800409	0.789104	907.0
macro avg	0.779450	0.779730	0.779262	4026.0
micro avg	0.786246	0.786246	0.786246	4026.0
weighted avg	0.786253	0.786337	0.786246	4026.0

The result of Model Number:Dataset Number:Featuregroup Number:Rescale of 86 : 0 : 3 : 0

	f1-score	precision	recall	support
AFIB	0.897145	0.915963	0.879108	204.0
GSVT	0.917956	0.912525	0.923462	300.0
SB	0.940335	0.938133	0.942549	892.0
SR	0.932393	0.932125	0.932669	681.0
macro avg	0.921957	0.924686	0.919447	2077.0
micro avg	0.930322	0.930322	0.930322	2077.0
weighted avg	0.930257	0.930287	0.930322	2077.0

The result of Model Number:Dataset Number:Featuregroup Number:Rescale of 86 : 0 : 3 : 1

	f1-score	precision	recall	support
AFIB	0.801683	0.835589	0.770688	204.0

GSVT	0.738292	0.739584	0.748079	300.0
SB	0.794839	0.756974	0.837463	892.0
SR	0.702677	0.759272	0.655205	681.0
macro avg	0.759373	0.772855	0.752859	2077.0
micro avg	0.758236	0.758236	0.758236	2077.0
weighted avg	0.757126	0.762937	0.758236	2077.0

The result of Model Number:Dataset Number:Featuregroup Number:Rescale of 86 : 0 : 4 : 0

	f1-score	precision	recall	support
AFIB	0.722004	0.745458	0.700118	204.0
GSVT	0.735691	0.729106	0.742543	300.0
SB	0.753185	0.748023	0.758421	892.0
SR	0.740902	0.744028	0.737846	681.0
macro avg	0.737946	0.741654	0.734732	2077.0
micro avg	0.743655	0.743655	0.743655	2077.0
weighted avg	0.743569	0.743729	0.743655	2077.0

The result of Model Number:Dataset Number:Featuregroup Number:Rescale of 86 : 0 : 4 : 1

	f1-score	precision	recall	support
AFIB	0.736617	0.742440	0.730909	875.0
GSVT	0.755152	0.753556	0.756784	691.0
SB	0.805014	0.797899	0.812268	1553.0
SR	0.783584	0.791269	0.776066	907.0
macro avg	0.770092	0.771291	0.769006	4026.0
micro avg	0.776907	0.776907	0.776907	4026.0
weighted avg	0.776763	0.776741	0.776907	4026.0

The result of Model Number:Dataset Number:Featuregroup Number:Rescale of 86 : 0 : 5 : 0

	f1-score	precision	recall	support
AFIB	0.671397	0.680101	0.663092	204.0
GSVT	0.697376	0.696925	0.697849	300.0
SB	0.712070	0.710567	0.713585	892.0
SR	0.704909	0.704508	0.705325	681.0
macro avg	0.696438	0.698025	0.694963	2077.0
micro avg	0.703645	0.703645	0.703645	2077.0
weighted avg	0.703605	0.703618	0.703645	2077.0

The result of Model Number:Dataset Number:Featuregroup Number:Rescale of 86 : 0 : 5 : 1

	f1-score	precision	recall	support
AFIB	0.783510	0.785308	0.781806	875.0
GSVT	0.786241	0.784025	0.788497	691.0
SB	0.825148	0.818315	0.832107	1553.0
SR	0.800394	0.812406	0.788766	907.0
macro avg	0.798823	0.800013	0.797794	4026.0

micro avg 0.803926 0.803926 0.803926 4026.0
weighted avg 0.803844 0.803925 0.803926 4026.0
The result of Model Number:Dataset Number:Featuregroup Number:Rescale of 86 : 0 : 6 : 0

	f1-score	precision	recall	support
AFIB	0.690087	0.685574	0.694719	875.0
GSVT	0.710597	0.716643	0.704747	691.0
SB	0.756527	0.754879	0.758183	1553.0
SR	0.738090	0.741053	0.735161	907.0
macro avg	0.723825	0.724537	0.723202	4026.0
micro avg	0.730032	0.730032	0.730032	4026.0
weighted avg	0.730050	0.730139	0.730032	4026.0

The result of Model Number:Dataset Number:Featuregroup Number:Rescale of 86 : 0 : 6 : 1

	f1-score	precision	recall	support
AFIB	0.727667	0.733435	0.722008	875.0
GSVT	0.736893	0.733644	0.740195	691.0
SB	0.783598	0.780501	0.786727	1553.0
SR	0.764343	0.766502	0.762232	907.0
macro avg	0.753125	0.753521	0.752790	4026.0
micro avg	0.759156	0.759156	0.759156	4026.0
weighted avg	0.759088	0.759076	0.759156	4026.0

The result of Model Number:Dataset Number:Featuregroup Number:Rescale of 86 : 0 : 7 : 0

	f1-score	precision	recall	support
AFIB	0.740265	0.832363	0.667170	204.0
GSVT	0.878270	0.859516	0.897900	300.0
SB	0.937570	0.932790	0.942403	892.0
SR	0.916536	0.904930	0.928503	681.0
macro avg	0.868160	0.882400	0.858994	2077.0
micro avg	0.904385	0.904385	0.904385	2077.0
weighted avg	0.902729	0.903208	0.904385	2077.0

The result of Model Number:Dataset Number:Featuregroup Number:Rescale of 86 : 0 : 7 : 1

	f1-score	precision	recall	support
AFIB	0.740993	0.815753	0.679220	204.0
GSVT	0.873847	0.861785	0.886391	300.0
SB	0.927856	0.924761	0.930977	892.0
SR	0.904095	0.891587	0.916971	681.0
macro avg	0.861698	0.873471	0.853390	2077.0
micro avg	0.895217	0.895217	0.895217	2077.0
weighted avg	0.893911	0.894081	0.895217	2077.0

The result of Model Number:Dataset Number:Featuregroup Number:Rescale of 86 : 0 : 8 : 0

	f1-score	precision	recall	support
AFIB	0.754888	0.756196	0.753596	875.0
GSVT	0.764041	0.755262	0.773090	691.0
SB	0.804561	0.794860	0.814506	1553.0
SR	0.766318	0.789538	0.744443	907.0
macro avg	0.772452	0.773964	0.771409	4026.0
micro avg	0.778375	0.778375	0.778375	4026.0
weighted avg	0.778195	0.778461	0.778375	4026.0

The result of Model Number:Dataset Number:Featuregroup Number:Rescale of 86 : 0 : 8 : 1

	f1-score	precision	recall	support
AFIB	0.833152	0.828487	0.837909	875.0
GSVT	0.839936	0.844892	0.835090	691.0
SB	0.884804	0.882034	0.887593	1553.0
SR	0.867287	0.873025	0.861644	907.0
macro avg	0.856295	0.857110	0.855559	4026.0
micro avg	0.861938	0.861938	0.861938	4026.0
weighted avg	0.861931	0.861992	0.861938	4026.0

The result of Model Number:Dataset Number:Featuregroup Number:Rescale of 86 : 0 : 9 : 0

	f1-score	precision	recall	support
AFIB	0.699814	0.747597	0.658071	204.0
GSVT	0.739773	0.724698	0.755553	300.0
SB	0.758122	0.751476	0.764916	892.0
SR	0.739422	0.741884	0.737042	681.0
macro avg	0.734283	0.741414	0.728896	2077.0
micro avg	0.743930	0.743930	0.743930	2077.0
weighted avg	0.743614	0.744082	0.743930	2077.0

The result of Model Number:Dataset Number:Featuregroup Number:Rescale of 86 : 0 : 9 : 1

	f1-score	precision	recall	support
AFIB	0.754900	0.769656	0.740832	204.0
GSVT	0.775643	0.770055	0.781340	300.0
SB	0.789525	0.786453	0.792623	892.0
SR	0.780895	0.783105	0.778715	681.0
macro avg	0.775241	0.777317	0.773377	2077.0
micro avg	0.781346	0.781346	0.781346	2077.0
weighted avg	0.781290	0.781337	0.781346	2077.0

The result of Model Number:Dataset Number:Featuregroup Number:Rescale of 86 : 0 : 10 : 0

	f1-score	precision	recall	support
AFIB	0.920611	0.937697	0.904366	204.0
GSVT	0.940855	0.935921	0.945860	300.0
SB	0.955875	0.952477	0.959303	892.0

SR 0.946034 0.947739 0.944376 681.0
macro avg 0.940844 0.943459 0.938476 2077.0
micro avg 0.947071 0.947071 0.947071 2077.0
weighted avg 0.947016 0.947081 0.947071 2077.0
The result of Model Number:Dataset Number:Featuregroup Number:Rescale of 86 : 0 : 10 : 1

	f1-score	precision	recall	support
AFIB	0.867280	0.909325	0.829048	204.0
GSVT	0.903400	0.891596	0.915705	300.0
SB	0.927732	0.920677	0.934909	892.0
SR	0.901901	0.904596	0.899262	681.0
macro avg	0.900078	0.906549	0.894731	2077.0
micro avg	0.910050	0.910050	0.910050	2077.0
weighted avg	0.909811	0.910089	0.910050	2077.0

The result of Model Number:Dataset Number:Featuregroup Number:Rescale of 86 : 1 : 0 : 0

	f1-score	precision	recall	support
AFIB	0.898819	0.912446	0.885615	204.0
GSVT	0.913271	0.905572	0.921150	300.0
SB	0.933529	0.930518	0.936562	892.0
SR	0.923508	0.926938	0.920128	681.0
macro avg	0.917282	0.918868	0.915864	2077.0
micro avg	0.923944	0.923944	0.923944	2077.0
weighted avg	0.923908	0.923966	0.923944	2077.0

The result of Model Number:Dataset Number:Featuregroup Number:Rescale of 86 : 1 : 0 : 1

	f1-score	precision	recall	support
AFIB	0.760644	0.778557	0.743602	875.0
GSVT	0.801831	0.803856	0.799869	691.0
SB	0.872006	0.868391	0.875655	1553.0
SR	0.829719	0.816652	0.843251	907.0
macro avg	0.816050	0.816864	0.815594	4026.0
micro avg	0.826647	0.826647	0.826647	4026.0
weighted avg	0.826232	0.826134	0.826647	4026.0

The result of Model Number:Dataset Number:Featuregroup Number:Rescale of 86 : 1 : 1 : 0

	f1-score	precision	recall	support
AFIB	0.839506	0.843504	0.835583	875.0
GSVT	0.849877	0.834432	0.865991	691.0
SB	0.899234	0.895791	0.902707	1553.0
SR	0.872655	0.887592	0.858226	907.0
macro avg	0.865318	0.865329	0.865627	4026.0
micro avg	0.871796	0.871796	0.871796	4026.0
weighted avg	0.871794	0.872048	0.871796	4026.0

The result of Model Number:Dataset Number:Featuregroup Number:Rescale of 86 : 1 : 1 : 1

	f1-score	precision	recall	support
AFIB	0.817505	0.835992	0.799843	875.0
GSVT	0.866741	0.870430	0.863135	691.0
SB	0.941101	0.936666	0.945584	1553.0
SR	0.891681	0.878003	0.905828	907.0
macro avg	0.879257	0.880273	0.878597	4026.0
micro avg	0.890801	0.890801	0.890801	4026.0
weighted avg	0.890343	0.890201	0.890801	4026.0

The result of Model Number:Dataset Number:Featuregroup Number:Rescale of 86 : 1 : 2 : 0

	f1-score	precision	recall	support
AFIB	0.693867	0.701979	0.686073	204.0
GSVT	0.712955	0.709676	0.716283	300.0
SB	0.723049	0.720800	0.725316	892.0
SR	0.716822	0.718854	0.714832	681.0
macro avg	0.711673	0.712827	0.710626	2077.0
micro avg	0.716720	0.716720	0.716720	2077.0
weighted avg	0.716683	0.716707	0.716720	2077.0

The result of Model Number:Dataset Number:Featuregroup Number:Rescale of 86 : 1 : 2 : 1

	f1-score	precision	recall	support
AFIB	0.871272	0.899199	0.845287	204.0
GSVT	0.897337	0.881900	0.913458	300.0
SB	0.912860	0.903836	0.922069	892.0
SR	0.888452	0.899570	0.877625	681.0
macro avg	0.892480	0.896126	0.889610	2077.0
micro avg	0.898712	0.898712	0.898712	2077.0
weighted avg	0.898531	0.898813	0.898712	2077.0

The result of Model Number:Dataset Number:Featuregroup Number:Rescale of 86 : 1 : 3 : 0

	f1-score	precision	recall	support
AFIB	0.658421	0.644277	0.673236	875.0
GSVT	0.681865	0.691753	0.672300	691.0
SB	0.725872	0.725733	0.726013	1553.0
SR	0.707679	0.715904	0.699644	907.0
macro avg	0.693459	0.694417	0.692798	4026.0
micro avg	0.699383	0.699383	0.699383	4026.0
weighted avg	0.699560	0.699983	0.699383	4026.0

The result of Model Number:Dataset Number:Featuregroup Number:Rescale of 86 : 1 : 3 : 1

	f1-score	precision	recall	support
AFIB	0.643269	0.653715	0.633186	875.0

GSVT	0.684135	0.686279	0.682046	691.0
SB	0.744305	0.741740	0.746891	1553.0
SR	0.704640	0.696791	0.712710	907.0
macro avg	0.694087	0.694631	0.693708	4026.0
micro avg	0.703349	0.703349	0.703349	4026.0
weighted avg	0.703083	0.702964	0.703349	4026.0

The result of Model Number:Dataset Number:Featuregroup Number:Rescale of 86 : 1 : 4 : 0

	f1-score	precision	recall	support
AFIB	0.826662	0.820297	0.833189	875.0
GSVT	0.837394	0.841282	0.833725	691.0
SB	0.897385	0.892611	0.902229	1553.0
SR	0.870945	0.883190	0.859131	907.0
macro avg	0.858096	0.859345	0.857069	4026.0
micro avg	0.865757	0.865757	0.865757	4026.0
weighted avg	0.865761	0.865962	0.865757	4026.0

The result of Model Number:Dataset Number:Featuregroup Number:Rescale of 86 : 1 : 4 : 1

	f1-score	precision	recall	support
AFIB	0.828600	0.832371	0.824914	875.0
GSVT	0.832626	0.824327	0.841165	691.0
SB	0.877590	0.868027	0.887366	1553.0
SR	0.839320	0.859190	0.820351	907.0
macro avg	0.844534	0.845979	0.843449	4026.0
micro avg	0.850766	0.850766	0.850766	4026.0
weighted avg	0.850603	0.850786	0.850766	4026.0

The result of Model Number:Dataset Number:Featuregroup Number:Rescale of 86 : 1 : 5 : 0

	f1-score	precision	recall	support
AFIB	0.801399	0.835293	0.770415	204.0
GSVT	0.738030	0.739322	0.747813	300.0
SB	0.794558	0.756706	0.837167	892.0
SR	0.702428	0.759003	0.654972	681.0
macro avg	0.759104	0.772581	0.752592	2077.0
micro avg	0.757967	0.757967	0.757967	2077.0
weighted avg	0.756858	0.762667	0.757967	2077.0

The result of Model Number:Dataset Number:Featuregroup Number:Rescale of 86 : 1 : 5 : 1

	f1-score	precision	recall	support
AFIB	0.767844	0.757650	0.778375	875.0
GSVT	0.783429	0.790856	0.776174	691.0
SB	0.833680	0.831708	0.835666	1553.0
SR	0.811064	0.819592	0.802745	907.0
macro avg	0.799004	0.799951	0.798240	4026.0

micro avg 0.805587 0.805587 0.805587 4026.0
weighted avg 0.805651 0.805871 0.805587 4026.0
The result of Model Number:Dataset Number:Featuregroup Number:Rescale of 86 : 1 : 6 : 0

	f1-score	precision	recall	support
AFIB	0.686558	0.695649	0.677803	204.0
GSVT	0.707276	0.704723	0.709863	300.0
SB	0.716085	0.713857	0.718331	892.0
SR	0.708282	0.709647	0.706943	681.0
macro avg	0.704550	0.705969	0.703235	2077.0
micro avg	0.709393	0.709393	0.709393	2077.0
weighted avg	0.709354	0.709369	0.709393	2077.0

The result of Model Number:Dataset Number:Featuregroup Number:Rescale of 86 : 1 : 6 : 1

	f1-score	precision	recall	support
AFIB	0.746119	0.748307	0.744002	875.0
GSVT	0.758465	0.748723	0.768562	691.0
SB	0.802709	0.800758	0.804673	1553.0
SR	0.782383	0.791668	0.773322	907.0
macro avg	0.772419	0.772364	0.772640	4026.0
micro avg	0.778226	0.778226	0.778226	4026.0
weighted avg	0.778237	0.778380	0.778226	4026.0

The result of Model Number:Dataset Number:Featuregroup Number:Rescale of 86 : 1 : 7 : 0

	f1-score	precision	recall	support
AFIB	0.837794	0.830029	0.845753	875.0
GSVT	0.852840	0.857471	0.848272	691.0
SB	0.903632	0.901549	0.905726	1553.0
SR	0.882579	0.890723	0.874609	907.0
macro avg	0.869211	0.869943	0.868590	4026.0
micro avg	0.875820	0.875820	0.875820	4026.0
weighted avg	0.875862	0.876001	0.875820	4026.0

The result of Model Number:Dataset Number:Featuregroup Number:Rescale of 86 : 1 : 7 : 1

	f1-score	precision	recall	support
AFIB	0.883624	0.876820	0.890601	875.0
GSVT	0.895096	0.899252	0.891174	691.0
SB	0.959220	0.954118	0.964399	1553.0
SR	0.930959	0.944047	0.918330	907.0
macro avg	0.917225	0.918559	0.916126	4026.0
micro avg	0.925413	0.925413	0.925413	4026.0
weighted avg	0.925418	0.925633	0.925413	4026.0

The result of Model Number:Dataset Number:Featuregroup Number:Rescale of 86 : 1 : 8 : 0

	f1-score	precision	recall	support
AFIB	0.618542	0.628587	0.608847	875.0
GSVT	0.657838	0.659899	0.655829	691.0
SB	0.715694	0.713228	0.718182	1553.0
SR	0.677554	0.670007	0.685314	907.0
macro avg	0.667407	0.667930	0.667043	4026.0
micro avg	0.676313	0.676313	0.676313	4026.0
weighted avg	0.676057	0.675942	0.676313	4026.0

The result of Model Number:Dataset Number:Featuregroup Number:Rescale of 86 : 1 : 8 : 1

	f1-score	precision	recall	support
AFIB	0.703493	0.704445	0.702679	875.0
GSVT	0.714450	0.714083	0.714845	691.0
SB	0.746170	0.743500	0.748861	1553.0
SR	0.728301	0.732383	0.724311	907.0
macro avg	0.723104	0.723603	0.722674	4026.0
micro avg	0.727455	0.727455	0.727455	4026.0
weighted avg	0.727425	0.727458	0.727455	4026.0

The result of Model Number:Dataset Number:Featuregroup Number:Rescale of 86 : 1 : 9 : 0

	f1-score	precision	recall	support
AFIB	0.831981	0.820458	0.843867	875.0
GSVT	0.850434	0.857317	0.843666	691.0
SB	0.902726	0.900132	0.905340	1553.0
SR	0.880765	0.892229	0.869601	907.0
macro avg	0.866477	0.867534	0.865619	4026.0
micro avg	0.873343	0.873343	0.873343	4026.0
weighted avg	0.873428	0.873687	0.873343	4026.0

The result of Model Number:Dataset Number:Featuregroup Number:Rescale of 86 : 1 : 9 : 1

	f1-score	precision	recall	support
AFIB	0.671377	0.677418	0.665653	875.0
GSVT	0.685053	0.671162	0.699692	691.0
SB	0.726503	0.718421	0.734856	1553.0
SR	0.697674	0.718413	0.678263	907.0
macro avg	0.695152	0.696353	0.694616	4026.0
micro avg	0.701031	0.701031	0.701031	4026.0
weighted avg	0.700913	0.701396	0.701031	4026.0

The result of Model Number:Dataset Number:Featuregroup Number:Rescale of 86 : 1 : 10 : 0

	f1-score	precision	recall	support
AFIB	0.709746	0.694579	0.725631	875.0
GSVT	0.734987	0.746226	0.724147	691.0
SB	0.783495	0.783598	0.783394	1553.0

SR 0.763310 0.771215 0.755568 907.0
macro avg 0.747884 0.748905 0.747185 4026.0
micro avg 0.754403 0.754403 0.754403 4026.0
weighted avg 0.754593 0.755047 0.754403 4026.0
The result of Model Number:Dataset Number:Featuregroup Number:Rescale of 86 : 1 : 10 : 1

	f1-score	precision	recall	support
AFIB	0.824655	0.828446	0.820940	875.0
GSVT	0.790378	0.795140	0.785699	691.0
SB	0.847809	0.810018	0.889341	1553.0
SR	0.725063	0.789325	0.670622	907.0
macro avg	0.796976	0.805732	0.791650	4026.0
micro avg	0.807412	0.807412	0.807412	4026.0
weighted avg	0.805267	0.806808	0.807412	4026.0

The result of Model Number:Dataset Number:Featuregroup Number:Rescale of 87 : 0 : 0 : 0

	f1-score	precision	recall	support
AFIB	0.679195	0.716141	0.646047	204.0
GSVT	0.713029	0.702245	0.724178	300.0
SB	0.729973	0.725225	0.734796	892.0
SR	0.720967	0.721617	0.720336	681.0
macro avg	0.710791	0.716307	0.706339	2077.0
micro avg	0.719804	0.719804	0.719804	2077.0
weighted avg	0.719586	0.719830	0.719804	2077.0

The result of Model Number:Dataset Number:Featuregroup Number:Rescale of 87 : 0 : 0 : 1

	f1-score	precision	recall	support
AFIB	0.845108	0.883270	0.810383	204.0
GSVT	0.874502	0.864681	0.884708	300.0
SB	0.841238	0.804562	0.900137	892.0
SR	0.700867	0.706843	0.695110	681.0
macro avg	0.815429	0.814839	0.822585	2077.0
micro avg	0.821870	0.821870	0.821870	2077.0
weighted avg	0.800399	0.788936	0.821870	2077.0

The result of Model Number:Dataset Number:Featuregroup Number:Rescale of 87 : 0 : 1 : 0

	f1-score	precision	recall	support
AFIB	0.637830	0.622965	0.653526	875.0
GSVT	0.612294	0.619025	0.605750	691.0
SB	0.644342	0.602734	0.692135	1553.0
SR	0.515695	0.619250	0.442088	907.0
macro avg	0.602541	0.615994	0.598375	4026.0
micro avg	0.612585	0.612585	0.612585	4026.0
weighted avg	0.608444	0.613648	0.612585	4026.0

The result of Model Number:Dataset Number:Featuregroup Number:Rescale of 87 : 0 : 1 : 1

	f1-score	precision	recall	support
AFIB	0.691599	0.709410	0.674688	204.0
GSVT	0.634663	0.606411	0.716216	300.0
SB	0.728141	0.726438	0.729855	892.0
SR	0.577648	0.577757	0.577547	681.0
macro avg	0.658013	0.655004	0.674577	2077.0
micro avg	0.672528	0.672528	0.672528	2077.0
weighted avg	0.661707	0.658680	0.672528	2077.0

The result of Model Number:Dataset Number:Featuregroup Number:Rescale of 87 : 0 : 2 : 0

	f1-score	precision	recall	support
AFIB	0.808753	0.812604	0.804974	875.0
GSVT	0.818744	0.803864	0.834268	691.0
SB	0.866293	0.862975	0.869638	1553.0
SR	0.840687	0.855077	0.826787	907.0
macro avg	0.833619	0.833630	0.833917	4026.0
micro avg	0.839860	0.839860	0.839860	4026.0
weighted avg	0.839857	0.840103	0.839860	4026.0

The result of Model Number:Dataset Number:Featuregroup Number:Rescale of 87 : 0 : 2 : 1

	f1-score	precision	recall	support
AFIB	0.721715	0.701697	0.745179	875.0
GSVT	0.731136	0.761933	0.707231	691.0
SB	0.803179	0.802773	0.803595	1553.0
SR	0.783029	0.787929	0.778195	907.0
macro avg	0.759765	0.763583	0.758550	4026.0
micro avg	0.768638	0.768638	0.768638	4026.0
weighted avg	0.768569	0.770452	0.768638	4026.0

The result of Model Number:Dataset Number:Featuregroup Number:Rescale of 87 : 0 : 3 : 0

	f1-score	precision	recall	support
AFIB	0.582930	0.579260	0.656631	204.0
GSVT	0.710602	0.706947	0.714414	300.0
SB	0.730835	0.727358	0.734354	892.0
SR	0.573258	0.575146	0.571727	681.0
macro avg	0.649406	0.647178	0.669282	2077.0
micro avg	0.670518	0.670518	0.670518	2077.0
weighted avg	0.661720	0.659957	0.670518	2077.0

The result of Model Number:Dataset Number:Featuregroup Number:Rescale of 87 : 0 : 3 : 1

	f1-score	precision	recall	support
AFIB	0.812077	0.803104	0.821269	875.0

GSVT   0.826708   0.831799 0.821694   691.0
SB   0.879873   0.878408 0.881346   1553.0
SR   0.858448   0.866475 0.850590   907.0
macro avg   0.844277   0.844947 0.843725   4026.0
micro avg   0.851122   0.851122 0.851122   4026.0
weighted avg 0.851187   0.851354 0.851122   4026.0
The result of Model Number:Dataset Number:Featuregroup Number:Rescale of 87 : 0 : 4 : 0

	f1-score	precision	recall	support
AFIB	0.664383	0.659301	0.669631	875.0
GSVT	0.679669	0.683386	0.676033	691.0
SB	0.719715	0.719211	0.720225	1553.0
SR	0.700598	0.703997	0.697269	907.0
macro avg	0.691091	0.691474	0.690790	4026.0
micro avg	0.696473	0.696473	0.696473	4026.0
weighted avg	0.696509	0.696614	0.696473	4026.0

The result of Model Number:Dataset Number:Featuregroup Number:Rescale of 87 : 0 : 4 : 1

	f1-score	precision	recall	support
AFIB	0.660666	0.655612	0.665885	875.0
GSVT	0.675867	0.679563	0.672251	691.0
SB	0.715688	0.715187	0.716195	1553.0
SR	0.696678	0.700058	0.693368	907.0
macro avg	0.687225	0.687605	0.686925	4026.0
micro avg	0.692576	0.692576	0.692576	4026.0
weighted avg	0.692612	0.692716	0.692576	4026.0

The result of Model Number:Dataset Number:Featuregroup Number:Rescale of 87 : 0 : 5 : 0

	f1-score	precision	recall	support
AFIB	0.867675	0.857175	0.878475	875.0
GSVT	0.879420	0.888437	0.870653	691.0
SB	0.934034	0.930994	0.937100	1553.0
SR	0.913604	0.922991	0.904437	907.0
macro avg	0.898683	0.899899	0.897666	4026.0
micro avg	0.905596	0.905596	0.905596	4026.0
weighted avg	0.905636	0.905843	0.905596	4026.0

The result of Model Number:Dataset Number:Featuregroup Number:Rescale of 87 : 0 : 5 : 1

	f1-score	precision	recall	support
AFIB	0.775605	0.769435	0.781948	875.0
GSVT	0.744852	0.730690	0.759636	691.0
SB	0.781749	0.721623	0.852820	1553.0
SR	0.598603	0.765401	0.491606	907.0
macro avg	0.725202	0.746787	0.721502	4026.0

micro avg 0.740047 0.740047 0.740047 4026.0
weighted avg 0.732821 0.743433 0.740047 4026.0
The result of Model Number:Dataset Number:Featuregroup Number:Rescale of 87 : 0 : 6 : 0

	f1-score	precision	recall	support
AFIB	0.768682	0.759447	0.778172	875.0
GSVT	0.783466	0.789809	0.777229	691.0
SB	0.835114	0.831918	0.838339	1553.0
SR	0.815533	0.825961	0.805376	907.0
macro avg	0.800699	0.801784	0.799779	4026.0
micro avg	0.807348	0.807348	0.807348	4026.0
weighted avg	0.807400	0.807598	0.807348	4026.0

The result of Model Number:Dataset Number:Featuregroup Number:Rescale of 87 : 0 : 6 : 1

	f1-score	precision	recall	support
AFIB	0.644637	0.636422	0.653089	875.0
GSVT	0.615511	0.619747	0.611349	691.0
SB	0.651716	0.614055	0.694312	1553.0
SR	0.531497	0.614927	0.468031	907.0
macro avg	0.610840	0.621288	0.606695	4026.0
micro avg	0.620135	0.620135	0.620135	4026.0
weighted avg	0.616880	0.620090	0.620135	4026.0

The result of Model Number:Dataset Number:Featuregroup Number:Rescale of 87 : 0 : 7 : 0

	f1-score	precision	recall	support
AFIB	0.794997	0.811014	0.780110	204.0
GSVT	0.820506	0.813917	0.827362	300.0
SB	0.838269	0.837425	0.839115	892.0
SR	0.830628	0.830151	0.831115	681.0
macro avg	0.821100	0.823127	0.819426	2077.0
micro avg	0.828999	0.828999	0.828999	2077.0
weighted avg	0.828948	0.829051	0.828999	2077.0

The result of Model Number:Dataset Number:Featuregroup Number:Rescale of 87 : 0 : 7 : 1

	f1-score	precision	recall	support
AFIB	0.714937	0.724486	0.705837	204.0
GSVT	0.727567	0.723134	0.732165	300.0
SB	0.745105	0.744444	0.745772	892.0
SR	0.739599	0.739711	0.739492	681.0
macro avg	0.731802	0.732944	0.730816	2077.0
micro avg	0.737825	0.737825	0.737825	2077.0
weighted avg	0.737803	0.737854	0.737825	2077.0

The result of Model Number:Dataset Number:Featuregroup Number:Rescale of 87 : 0 : 8 : 0

	f1-score	precision	recall	support
AFIB	0.700037	0.758476	0.651354	204.0
GSVT	0.750871	0.741429	0.760862	300.0
SB	0.770039	0.759251	0.781228	892.0
SR	0.749034	0.752543	0.745923	681.0
macro avg	0.742495	0.752925	0.734842	2077.0
micro avg	0.753955	0.753955	0.753955	2077.0
weighted avg	0.753508	0.754401	0.753955	2077.0

The result of Model Number:Dataset Number:Featuregroup Number:Rescale of 87 : 0 : 8 : 1

	f1-score	precision	recall	support
AFIB	0.784303	0.775153	0.795718	875.0
GSVT	0.807001	0.797219	0.817675	691.0
SB	0.819165	0.787544	0.865545	1553.0
SR	0.650459	0.684770	0.619461	907.0
macro avg	0.765232	0.761172	0.774600	4026.0
micro avg	0.786714	0.786714	0.786714	4026.0
weighted avg	0.771493	0.763358	0.786714	4026.0

The result of Model Number:Dataset Number:Featuregroup Number:Rescale of 87 : 0 : 9 : 0

	f1-score	precision	recall	support
AFIB	0.775987	0.785515	0.766808	204.0
GSVT	0.791617	0.788236	0.795046	300.0
SB	0.801199	0.798794	0.803622	892.0
SR	0.794456	0.796346	0.792599	681.0
macro avg	0.790815	0.792223	0.789519	2077.0
micro avg	0.795154	0.795154	0.795154	2077.0
weighted avg	0.795128	0.795162	0.795154	2077.0

The result of Model Number:Dataset Number:Featuregroup Number:Rescale of 87 : 0 : 9 : 1

	f1-score	precision	recall	support
AFIB	0.650337	0.653327	0.647407	875.0
GSVT	0.623306	0.627061	0.619616	691.0
SB	0.668597	0.638795	0.701350	1553.0
SR	0.571797	0.622475	0.528864	907.0
macro avg	0.628509	0.635414	0.624309	4026.0
micro avg	0.636739	0.636739	0.636739	4026.0
weighted avg	0.635047	0.636263	0.636739	4026.0

The result of Model Number:Dataset Number:Featuregroup Number:Rescale of 87 : 0 : 10 : 0

	f1-score	precision	recall	support
AFIB	0.807637	0.795566	0.820111	875.0
GSVT	0.823122	0.830162	0.816214	691.0
SB	0.879487	0.876459	0.882542	1553.0

SR 0.853745 0.866479 0.841410 907.0
macro avg 0.840998 0.842166 0.840069 4026.0
micro avg 0.848323 0.848323 0.848323 4026.0
weighted avg 0.848398 0.848683 0.848323 4026.0
The result of Model Number:Dataset Number:Featuregroup Number:Rescale of 87 : 0 : 10 : 1

	f1-score	precision	recall	support
AFIB	0.740093	0.728485	0.752156	875.0
GSVT	0.713128	0.733572	0.693875	691.0
SB	0.749379	0.711392	0.791694	1553.0
SR	0.608866	0.677544	0.552925	907.0
macro avg	0.702866	0.712749	0.697663	4026.0
micro avg	0.712521	0.712521	0.712521	4026.0
weighted avg	0.709483	0.711289	0.712521	4026.0

The result of Model Number:Dataset Number:Featuregroup Number:Rescale of 87 : 1 : 0 : 0

	f1-score	precision	recall	support
AFIB	0.766251	0.845012	0.701391	204.0
GSVT	0.911817	0.899494	0.924572	300.0
SB	0.971020	0.967137	0.974938	892.0
SR	0.944665	0.931895	0.957830	681.0
macro avg	0.898438	0.910885	0.889683	2077.0
micro avg	0.935187	0.935187	0.935187	2077.0
weighted avg	0.933715	0.933817	0.935187	2077.0

The result of Model Number:Dataset Number:Featuregroup Number:Rescale of 87 : 1 : 0 : 1

	f1-score	precision	recall	support
AFIB	0.891630	0.909832	0.874537	204.0
GSVT	0.924007	0.915979	0.932345	300.0
SB	0.941772	0.940091	0.943461	892.0
SR	0.933006	0.933562	0.932456	681.0
macro avg	0.922604	0.924866	0.920700	2077.0
micro avg	0.931478	0.931478	0.931478	2077.0
weighted avg	0.931407	0.931495	0.931478	2077.0

The result of Model Number:Dataset Number:Featuregroup Number:Rescale of 87 : 1 : 1 : 0

	f1-score	precision	recall	support
AFIB	0.880421	0.879005	0.882202	875.0
GSVT	0.900949	0.896252	0.905747	691.0
SB	0.941989	0.934008	0.950114	1553.0
SR	0.906029	0.926172	0.887050	907.0
macro avg	0.907347	0.908859	0.906278	4026.0
micro avg	0.913532	0.913532	0.913532	4026.0
weighted avg	0.913463	0.913808	0.913532	4026.0

The result of Model Number:Dataset Number:Featuregroup Number:Rescale of 87 : 1 : 1 : 1

	f1-score	precision	recall	support
AFIB	0.731145	0.729727	0.732663	875.0
GSVT	0.744874	0.731841	0.758451	691.0
SB	0.797971	0.786649	0.809632	1553.0
SR	0.755889	0.789585	0.724983	907.0
macro avg	0.757470	0.759451	0.756432	4026.0
micro avg	0.765049	0.765049	0.765049	4026.0
weighted avg	0.764853	0.765532	0.765049	4026.0

The result of Model Number:Dataset Number:Featuregroup Number:Rescale of 87 : 1 : 2 : 0

	f1-score	precision	recall	support
AFIB	0.884938	0.886135	0.883913	875.0
GSVT	0.898721	0.898259	0.899217	691.0
SB	0.938621	0.935262	0.942007	1553.0
SR	0.916144	0.921279	0.911125	907.0
macro avg	0.909606	0.910234	0.909065	4026.0
micro avg	0.915079	0.915079	0.915079	4026.0
weighted avg	0.915042	0.915084	0.915079	4026.0

The result of Model Number:Dataset Number:Featuregroup Number:Rescale of 87 : 1 : 2 : 1

	f1-score	precision	recall	support
AFIB	0.835259	0.836755	0.833795	875.0
GSVT	0.799816	0.804018	0.795681	691.0
SB	0.846012	0.799473	0.898311	1553.0
SR	0.702616	0.790846	0.632159	907.0
macro avg	0.795926	0.807773	0.789986	4026.0
micro avg	0.806714	0.806714	0.806714	4026.0
weighted avg	0.803441	0.806412	0.806714	4026.0

The result of Model Number:Dataset Number:Featuregroup Number:Rescale of 87 : 1 : 3 : 0

	f1-score	precision	recall	support
AFIB	0.825951	0.863248	0.792013	204.0
GSVT	0.854679	0.845080	0.864653	300.0
SB	0.822169	0.786324	0.879733	892.0
SR	0.684980	0.690820	0.679353	681.0
macro avg	0.796945	0.796368	0.803938	2077.0
micro avg	0.803239	0.803239	0.803239	2077.0
weighted avg	0.782255	0.771052	0.803239	2077.0

The result of Model Number:Dataset Number:Featuregroup Number:Rescale of 87 : 1 : 3 : 1

	f1-score	precision	recall	support
AFIB	0.716614	0.708005	0.725462	875.0

GSVT 0.730397 0.736311 0.724582 691.0
SB 0.778547 0.775567 0.781553 1553.0
SR 0.760292 0.770014 0.750823 907.0
macro avg 0.746463 0.747474 0.745605 4026.0
micro avg 0.752661 0.752661 0.752661 4026.0
weighted avg 0.752710 0.752894 0.752661 4026.0
The result of Model Number:Dataset Number:Featuregroup Number:Rescale of 87 : 1 : 4 : 0

	f1-score	precision	recall	support
AFIB	0.562803	0.624596	0.512271	204.0
GSVT	0.657148	0.648933	0.665703	300.0
SB	0.699173	0.695606	0.702779	892.0
SR	0.682795	0.673249	0.692618	681.0
macro avg	0.650480	0.660596	0.643343	2077.0
micro avg	0.675381	0.675381	0.675381	2077.0
weighted avg	0.674339	0.674559	0.675381	2077.0

The result of Model Number:Dataset Number:Featuregroup Number:Rescale of 87 : 1 : 4 : 1

	f1-score	precision	recall	support
AFIB	0.647708	0.656630	0.639310	875.0
GSVT	0.661848	0.659107	0.664791	691.0
SB	0.704971	0.695728	0.714493	1553.0
SR	0.682838	0.693036	0.673240	907.0
macro avg	0.674341	0.676125	0.672958	4026.0
micro avg	0.680329	0.680329	0.680329	4026.0
weighted avg	0.680138	0.680339	0.680329	4026.0

The result of Model Number:Dataset Number:Featuregroup Number:Rescale of 87 : 1 : 5 : 0

	f1-score	precision	recall	support
AFIB	0.819283	0.806947	0.838823	875.0
GSVT	0.782267	0.779990	0.788940	691.0
SB	0.835361	0.778423	0.908232	1553.0
SR	0.618804	0.827157	0.512753	907.0
macro avg	0.763929	0.798129	0.762187	4026.0
micro avg	0.783577	0.783577	0.783577	4026.0
weighted avg	0.773967	0.795870	0.783577	4026.0

The result of Model Number:Dataset Number:Featuregroup Number:Rescale of 87 : 1 : 5 : 1

	f1-score	precision	recall	support
AFIB	0.888066	0.894454	0.881770	875.0
GSVT	0.886651	0.881342	0.892038	691.0
SB	0.935287	0.924924	0.945887	1553.0
SR	0.897480	0.913492	0.882049	907.0
macro avg	0.901871	0.903553	0.900436	4026.0

micro avg   0.908328   0.908328 0.908328   4026.0
weighted avg 0.908159   0.908246 0.908328   4026.0
The result of Model Number:Dataset Number:Featuregroup Number:Rescale of 87 : 1 : 6 : 0

	f1-score	precision	recall	support
AFIB	0.740790	0.756176	0.726189	875.0
GSVT	0.722418	0.697327	0.781388	691.0
SB	0.851482	0.847643	0.855371	1553.0
SR	0.647337	0.636886	0.658196	907.0
macro avg	0.740507	0.734508	0.755286	4026.0
micro avg	0.770176	0.770176	0.770176	4026.0
weighted avg	0.759282	0.754484	0.770176	4026.0

The result of Model Number:Dataset Number:Featuregroup Number:Rescale of 87 : 1 : 6 : 1

	f1-score	precision	recall	support
AFIB	0.654255	0.657687	0.650897	875.0
GSVT	0.667935	0.666735	0.669187	691.0
SB	0.725263	0.719701	0.730913	1553.0
SR	0.699675	0.706678	0.692814	907.0
macro avg	0.686782	0.687700	0.685953	4026.0
micro avg	0.694345	0.694345	0.694345	4026.0
weighted avg	0.694226	0.694198	0.694345	4026.0

The result of Model Number:Dataset Number:Featuregroup Number:Rescale of 87 : 1 : 7 : 0

	f1-score	precision	recall	support
AFIB	0.787779	0.790088	0.785543	875.0
GSVT	0.800814	0.790528	0.811474	691.0
SB	0.847529	0.845469	0.849602	1553.0
SR	0.826068	0.835871	0.816500	907.0
macro avg	0.815547	0.815489	0.815780	4026.0
micro avg	0.821678	0.821678	0.821678	4026.0
weighted avg	0.821690	0.821841	0.821678	4026.0

The result of Model Number:Dataset Number:Featuregroup Number:Rescale of 87 : 1 : 7 : 1

	f1-score	precision	recall	support
AFIB	0.824300	0.816359	0.832525	875.0
GSVT	0.845897	0.848317	0.843579	691.0
SB	0.899334	0.897266	0.901418	1553.0
SR	0.874036	0.884320	0.864007	907.0
macro avg	0.860892	0.861566	0.860382	4026.0
micro avg	0.868090	0.868090	0.868090	4026.0
weighted avg	0.868155	0.868364	0.868090	4026.0

The result of Model Number:Dataset Number:Featuregroup Number:Rescale of 87 : 1 : 8 : 0

	f1-score	precision	recall	support
AFIB	0.809178	0.824195	0.794899	204.0
GSVT	0.826970	0.822634	0.831369	300.0
SB	0.840173	0.837186	0.843186	892.0
SR	0.831523	0.833022	0.830066	681.0
macro avg	0.826961	0.829259	0.824880	2077.0
micro avg	0.832435	0.832435	0.832435	2077.0
weighted avg	0.832386	0.832443	0.832435	2077.0

The result of Model Number:Dataset Number:Featuregroup Number:Rescale of 87 : 1 : 8 : 1

	f1-score	precision	recall	support
AFIB	0.794213	0.798149	0.790401	875.0
GSVT	0.802121	0.803276	0.801090	691.0
SB	0.844340	0.840423	0.848304	1553.0
SR	0.828797	0.830873	0.826778	907.0
macro avg	0.817368	0.818180	0.816643	4026.0
micro avg	0.822766	0.822766	0.822766	4026.0
weighted avg	0.822698	0.822708	0.822766	4026.0

The result of Model Number:Dataset Number:Featuregroup Number:Rescale of 87 : 1 : 9 : 0

	f1-score	precision	recall	support
AFIB	0.646922	0.642045	0.734248	204.0
GSVT	0.784265	0.779622	0.788972	300.0
SB	0.641420	0.640081	0.642767	892.0
SR	0.793286	0.792016	0.794569	681.0
macro avg	0.716473	0.713441	0.740139	2077.0
micro avg	0.722643	0.722643	0.722643	2077.0
weighted avg	0.712386	0.710245	0.722643	2077.0

The result of Model Number:Dataset Number:Featuregroup Number:Rescale of 87 : 1 : 9 : 1

	f1-score	precision	recall	support
AFIB	0.780583	0.779320	0.781919	875.0
GSVT	0.787735	0.790868	0.784667	691.0
SB	0.827259	0.824252	0.830294	1553.0
SR	0.813728	0.817844	0.809698	907.0
macro avg	0.802326	0.803071	0.801644	4026.0
micro avg	0.807309	0.807309	0.807309	4026.0
weighted avg	0.807282	0.807313	0.807309	4026.0

The result of Model Number:Dataset Number:Featuregroup Number:Rescale of 87 : 1 : 10 : 0

	f1-score	precision	recall	support
AFIB	0.664446	0.668923	0.660128	875.0
GSVT	0.676304	0.666911	0.686091	691.0
SB	0.720676	0.715741	0.725686	1553.0

SR 0.691471 0.703415 0.679963 907.0
macro avg 0.688224 0.688747 0.687967 4026.0
micro avg 0.694341 0.694341 0.694341 4026.0
weighted avg 0.694260 0.694408 0.694341 4026.0
The result of Model Number:Dataset Number:Featuregroup Number:Rescale of 87 : 1 : 10 : 1

	f1-score	precision	recall	support
AFIB	0.676350	0.694057	0.659833	204.0
GSVT	0.636399	0.654895	0.619265	300.0
SB	0.671537	0.647893	0.696983	892.0
SR	0.608666	0.628488	0.590178	681.0
macro avg	0.648238	0.656333	0.641565	2077.0
micro avg	0.647090	0.647090	0.647090	2077.0
weighted avg	0.646320	0.647076	0.647090	2077.0

The result of Model Number:Dataset Number:Featuregroup Number:Rescale of 88 : 0 : 0 : 0

	f1-score	precision	recall	support
AFIB	0.897958	0.937867	0.861432	204.0
GSVT	0.935378	0.928035	0.942859	300.0
SB	0.949666	0.942340	0.957116	892.0
SR	0.939160	0.940711	0.937647	681.0
macro avg	0.930541	0.937238	0.924763	2077.0
micro avg	0.939275	0.939275	0.939275	2077.0
weighted avg	0.939079	0.939301	0.939275	2077.0

The result of Model Number:Dataset Number:Featuregroup Number:Rescale of 88 : 0 : 0 : 1

	f1-score	precision	recall	support
AFIB	0.866773	0.881822	0.852339	204.0
GSVT	0.883853	0.881238	0.886503	300.0
SB	0.894276	0.891094	0.897483	892.0
SR	0.887005	0.887931	0.886101	681.0
macro avg	0.882977	0.885521	0.880606	2077.0
micro avg	0.887731	0.887731	0.887731	2077.0
weighted avg	0.887685	0.887723	0.887731	2077.0

The result of Model Number:Dataset Number:Featuregroup Number:Rescale of 88 : 0 : 1 : 0

	f1-score	precision	recall	support
AFIB	0.795113	0.789739	0.800668	875.0
GSVT	0.814511	0.814835	0.814292	691.0
SB	0.865252	0.863044	0.867481	1553.0
SR	0.841692	0.851096	0.832505	907.0
macro avg	0.829142	0.829679	0.828736	4026.0
micro avg	0.835951	0.835951	0.835951	4026.0
weighted avg	0.835992	0.836146	0.835951	4026.0

The result of Model Number:Dataset Number:Featuregroup Number:Rescale of 88 : 0 : 1 : 1
[truncated: 222,248 more chars]
